# Analysis of Lymphoma-Related Genes with Gene Ontology and Kyoto Encyclopedia of Genes and Genomes Enrichment

**DOI:** 10.1155/2022/8503511

**Published:** 2022-06-26

**Authors:** Qiao Sun, Lin Bai, Shaopin Zhu, Lu Cheng, Yang Xu, Yu-Dong Cai, Hui Chen, Jian Zhang

**Affiliations:** ^1^Department of Ophthalmology, Shanghai General Hospital, Shanghai Jiao Tong University School of Medicine, Shanghai 200080, China; ^2^National Clinical Research Center for Eye Diseases, Shanghai 20080, China; ^3^Shanghai Key Laboratory of Ocular Fundus Diseases, Shanghai 200080, China; ^4^Shanghai Engineering Center for Visual Science and Photomedicine, Shanghai 200080, China; ^5^Shanghai Engineering Center for Precise Diagnosis and Treatment of Eye Diseases, Shanghai 20080, China; ^6^Eye School of Chengdu University of TCM, Chengdu 611137, China; ^7^Key Laboratory of Sichuan Province Ophthalmopathy Prevention & Cure and Visual Function Protection, Chengdu 611137, China; ^8^School of Life Sciences, Shanghai University, Shanghai 200444, China

## Abstract

Lymphoma is a serious malignant tumor that contains more than 70 different types and seriously endangers the body's lymphatic system. The lymphatic system is the regulatory center of the immune system and is important in the immune response to foreign antigens and tumors. Studies showed that multiple genetic variants are associated with lymphoma but determining the pathogenic mechanisms remains a challenge. In the present study, we first applied the Gene Ontology (GO) and KEGG pathway enrichment analyses of lymphoma-associated and lymphoma-nonassociated genes. Next, the Boruta and max-relevance and min-redundancy feature selection methods were performed to filter and rank features. Then, features preselected and ranked using the incremental feature selection method were applied for the decision tree model to identify the best GO terms and KEGG pathways and extract classification rules. Results indicate that our predicted features, such as B-cell activation, negative regulation of protein processing, negative regulation of mast cell cytokine production, and natural killer cell-mediated cytotoxicity, are associated with the biological process of lymphoma, consistent with those of recent publications. This study provides a new perspective for future research on the molecular mechanisms of lymphoma.

## 1. Introduction

Lymphoma, as one of the major cancer subtypes involving the lymphatic system, is a severe subgroup of malignancies in human beings [1, 2]. The lymphatic system is the center of the circulation immune system, thereby regulating the immune response against external antigens, germs, virus, and even cancers [3]. As a system throughout the body, the lymphatic system includes multiple levels of organs, including lymph nodes, spleen, thymus, and bone marrow [1, 3, 4]. Considering that the lymphatic system can affect the whole body, the malignant transformation of the lymphatic system, also known as lymphoma, is also a severe kind of malignancy affecting the whole body.

Multiple subtypes of lymphoma, including chronic lymphocytic leukemia [5], cutaneous B-cell lymphoma [6], Hodgkin's lymphoma [7], and non-Hodgkin's lymphoma [7, 8], are identified in clinics. Despite the diversity of lymphoma, most patients with lymphoma share similar symptoms, including painless swelling of lymph nodes, persistent fatigue, fever, and itchy skin [7]. Although the detailed pathogenesis of lymphoma remains unclear, external environmental effects, like Epstein–Barr virus [9] and *Helicobacter pylori* [10] infection, and genetic variations are shown to be associated with the disease. In recent years, with the development of sequencing techniques, genetic variations from multiple functional genes including *CASP10* [11], *ATM* [12], *RAD54L* [13], *BRAF* [14], and *CARD11* [15] have been shown to be associated with lymphoma. However, revealing the pathogenic mechanisms based on only a group of genes remains challenging. Further functional exploration, like gene ontology (GO) and pathway enrichment analyses, may help explore the biological foundation for the initiation and progression of lymphoma.

In this study, we summarized and compared the functional enrichment patterns of lymphoma-associated and lymphoma-nonassociated genes for the first time. By using Boruta, max-relevance and min-redundancy (mRMR), and incremental feature selection (IFS) methods and decision tree (DT) algorithms, we attempt to identify key functional enrichment terms (GO terms [16] or KEGG pathways [17]) contributing to the identification of lymphoma-associated genes. The identified functional enrichment terms are associated with the pathogenesis of lymphoma. Overall, our study has identified lymphoma-associated GO terms and KEGG pathways for the first time, thereby helping validate previous reports on the key biological effects of identified lymphoma biomarkers and establishing a novel approach to explore disease-associated pathogenesis at the functional level.

## 2. Materials and Methods

**Figure 1:**
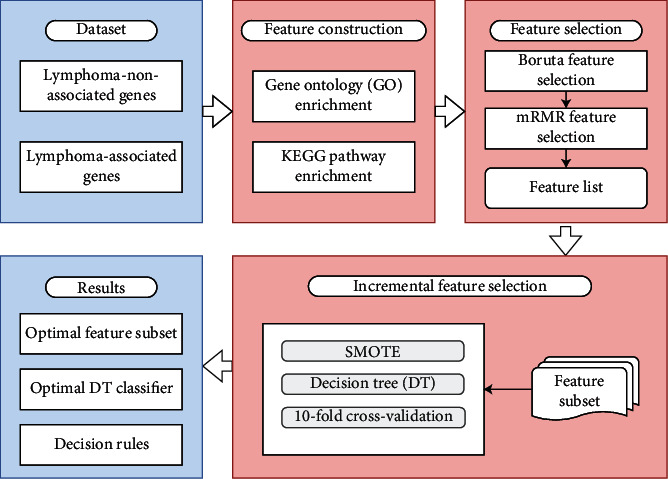
Flow chart for classifying samples for two types of genes in lymphoma. The gene ontology (GO) and KEGG pathway enrichment are used to construct the features of the dataset, and the Boruta and mRMR feature selection methods are used to filter and rank features. The optimal number of features and optimal classifiers are obtained by the incremental feature selection method with DT.

In this study, we investigated the functional enrichment patterns of lymphoma genes by using machine learning methods. The procedures are shown in Figure 1.

### 2.1. Dataset

In this study, we summarized 1548 lymphoma-associated genes from the DisGeNET database (https://www.disgenet.org/, v7.0) [18]. These genes were termed as positive samples, whereas the rest of the human genes were termed as negative samples. Given that the purpose of this study was to analyze the functional terms of lymphoma-associated genes, positive and negative samples without GO or KEGG pathway information were discarded. A total of 1330 positive and 16338 negative samples remained.

### 2.2. Feature Construction

Certain informative features should be used to express genes and identify distinctions between positive and negative samples. In this study, GO and KEGG enrichment scores were used as features of each gene.

GO enrichment denotes the association between a gene and a GO term. The ES_GO_(*g*, GO*j*) score, which is commonly called the GO enrichment score, is produced between each gene *g* and each GO term GO_*j*_. This score is defined by −log10 of the hypergeometric test *P* value [19] of the set *G* composed of the direct neighbors of *g* in STRING and another set consisting of genes annotated by GO term GO_*j*_ and calculated as follows:

(1) $$\begin{array}{c} {\text{ES}}_{\text{GO}}\left(g,{\text{GO}}_{j}\right)=-\log_{10}\left(\sum_{k=m}^{n}\frac{\left(\frac{M}{m}\right)\left(\frac{N-M}{n-m}\right)}{\left(\frac{N}{n}\right)}\right), \end{array}$$

where *N* indicates the overall number of human genes, *M* indicates the number of genes annotated by the GO term GO_*j*_, *n* indicates the number of genes in *G*, and *m* indicates the number of genes in *G* that are also annotated by GO_*j*_.

Similarly, the KEGG enrichment score ES_GO_(*g*, GO*j*) for each gene *g* and each KEGG pathway *P*_*j*_ can be computed as follows:

(2) $$\begin{array}{c} {\text{ES}}_{\text{KEGG}}\left(g,P_{j}\right)=-\log_{10}\left(\sum_{k=m}^{n}\frac{\left(\frac{M}{m}\right)\left(\frac{N-M}{n-m}\right)}{\left(\frac{N}{n}\right)}\right), \end{array}$$

where *N* and *M* indicate the number of human genes and number of genes annotated by the KEGG pathway *P*_*j*_, respectively, whereas *n* and *m* indicate the number of proteins in *G* and number of proteins in *G* that are also annotated by *P*_*j*_, respectively.

Certainly, a high enrichment score of a gene with one GO term or KEGG pathway indicated a strong relationship. In this study, 20681 GO and 297 KEGG enrichment scores were obtained for each gene. Thus, these 20978 features might be used to represent gene *g*, which can be expressed using the following formula:

(3) $$\begin{array}{c} v\left(g\right)={\left({\text{ES}}_{\text{GO}}\left(g,{\text{GO}}_{1}\right),\cdots ,{\text{ES}}_{\text{GO}}\left(g,{\text{GO}}_{20681}\right),{\text{ES}}_{\text{KEGG}}\left(g,P_{1}\right),\cdots ,{\text{ES}}_{\text{KEGG}}\left(g,P_{297}\right)\right)}^{T}. \end{array}$$

### 2.3. Feature Selection

As shown in Figure 1, we used the Boruta [20], mRMR [21], and incremental feature selection (IFS) [22] algorithms to perform feature selection. The Boruta method eliminated nonrelevant features, the mRMR method sorted the features into a feature list, and the IFS combined specific classifiers to determine the optimal number of features.

#### 2.3.1. Boruta Feature Selection

The presence of a large number of features in the dataset could cause some technical problems. Thus, we applied the Boruta algorithm to assess the importance of features and eventually selected significant features. As a wrapper feature selection method, the Boruta algorithm was designed on the basis of the random forest classification algorithm. The algorithm randomly created shadow features from original features and operated a random forest classifier on the collection of original and shadow features to filter important and unimportant features. In accordance with the outcomes of statistical tests (e.g., *z*-scores), the algorithm iteratively eliminated features that had lower *z*-scores compared with shadow features. The algorithm was implemented using the “boruta” package in https://github.com/scikit-learn-contrib/boruta_py.

#### 2.3.2. mRMR Feature Selection

To evaluate the degree of importance for each feature, we used the mRMR algorithm to sort features in terms of their importance. The informative features selected by this method had the maximum relevance to class labels and the minimum redundancy with each other. The method calculated the relationship between features or classified labels by using mutual information (MI). The MI values of variables *x* and *y* could be expressed as follows:

(4) $$\begin{array}{c} I\left(x,y\right)=\iint p\left(x,y\right)\log\frac{p\left(x,y\right)}{p\left(x\right)p\left(y\right)}dxdy, \end{array}$$

where *p*(*x*) and *p*(*y*) indicate the marginal probability densities of variables and *p*(*x*, *y*) refers to the joint probability density of two variables. The features that had the highest relevance to class labels and least redundancy with those already in the list were chosen from the remaining features one by one. If all features were in the list, the program was stopped. The mRMR program was retrieved from http://home.penglab.com/proj/mRMR/and executed using default parameters.

#### 2.3.3. IFS

Although the mRMR method ranked the features by importance, which features were essential in the feature list remained a problem. The IFS method was used to determine the optimum features in the feature list. In the first step, IFS had output a set of feature subsets from the list. For example, when the step size was set to 5, the 1st and 2nd feature subsets were composed of the top 5 and top 10 features, respectively, in the list. The training samples represented by features in each subset were next trained with the desired classifier. The classifier was assessed by 10-fold crossvalidation and synthetic minority oversampling technique (SMOTE) to obtain the performance metrics of the classification model, and the best classification model could be determined by performance metrics.

### 2.4. DT

Different from other algorithms, such as the support vector machine (SVM) [23] and random forest (RF) [24], DT [25] is a white box model that constructs classification or regression models that are easy to interpret. DT creates a tree structure in the IF–THEN format and generates rules that can be understood, thereby further enhancing the knowledge of the model prediction mechanism. This study adopted the DT program implemented by python in Scikit-learn (https://scikit-learn.org/stable/) [26]. Such program implements the CART tree with the Gini index to expand the tree.

### 2.5. SMOTE

An imbalance problem is present between the sizes of positive and negative samples in the abovementioned constructed dataset, where the positive sample size is much smaller than the negative sample size. To address this issue, we used the SMOTE [27] algorithm in this research. The SMOTE algorithm analyzes and simulates a minority class of samples by using the kNN technique and adds the newly synthesized samples to the dataset to produce a new training set. The SMOTE program that was run in this work was sourced from https://github.com/scikit-learn-contrib/imbalanced-learn, and parameters were set to default.

### 2.6. Performance Measurements

Evaluation metrics, such as accuracy (ACC), sensitivity (SN) (same as recall), specificity (SP), precision, F1-measure, and MCC [28–31], were used in the 10-fold crossvalidation [32–38] process to assess the performance of prediction models. The formulas of these evaluation metrics are shown as follows:

(5) $$\begin{array}{c} \text{ACC}=\frac{\text{tp}+\text{tn}}{\text{tp}+\text{fp}+\text{tn}+\text{fn}},\\ \text{SN}=\frac{\text{tp}}{\text{tp}+\text{fn}},\\ \text{SP}=\frac{\text{tn}}{\text{tn}+\text{fp}}, \end{array}$$

Precision = tp/tp + fp,

(6) $$\begin{array}{c} F1-\text{measure}=\frac{2\times \text{precision}\times \text{recall}}{\text{precision}+\text{recall}},\\ \text{MCC}=\frac{\text{tp}\times \text{tn}-\text{fp}\times \text{fn}}{\sqrt{\left(\text{tp}+\text{fp}\right)\left(\text{tp}+\text{fn}\right)\left(\text{tn}+\text{fp}\right)\left(\text{tn}+\text{fn}\right)}}, \end{array}$$

where tp, tn, fp, and fn represent the true-positive, true-negative, false-positive, and false-negative samples, respectively. Among the abovementioned measurements, *F*1 − measure was selected as the key measurement to evaluate the performance of different DT classifiers.

## 3. Results

### 3.1. Results of Boruta and mRMR Methods on the Dataset

The Boruta and mRMR feature selection methods were adopted to analyze the dataset and select key features. A total of 1075 features were retained after processing the original dataset by using the Boruta method. These preserved features are listed in Table S1. These features are composed of 1034 GO terms and 41 KEGG pathways. Further, these features were sorted by the mRMR method to evaluate their importance. Results are also listed in Table S1.

### 3.2. Results of the IFS Method with DT

**Figure 2:**
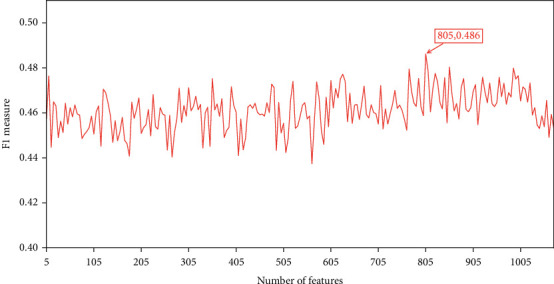
Incremental feature selection (IFS) curves of the DT classifier on the different number of features. DT provides the highest *F*1 − measure of 0.486 when the top 805 features are used.

A series of feature subsets was generated when the step size was set to 5 from the mRMR feature list and subjected to the IFS method to acquire the best features for classifying lymphoma-related genes and other genes and obtain the best number of features. The classification results using the different number of features are provided in Table S2. IFS curves were plotted by setting the number of features as the *x*-axis and the *F*1 − measure as the *y*-axis. As shown in Figure 2, the DT reached the highest *F*1 − measure of 0.486 when the top 805 features were used. Therefore, we considered these top 805 features as the best feature set and constructed the best DT classifier. The ACC and MCC of such classifier were 0.891 and 0.455, respectively. Furthermore, the SN, SP, and precision were 0.683, 0.908, and 0.378, respectively. As the positive samples were much less than the negative samples, SN was much lower than SP and precision was also not very high. Although the performance of the best DT classifier was not very high, it can still provide new clues, which can help us uncover the differences between lymphoma-associated genes and other ones.

**Figure 3:**
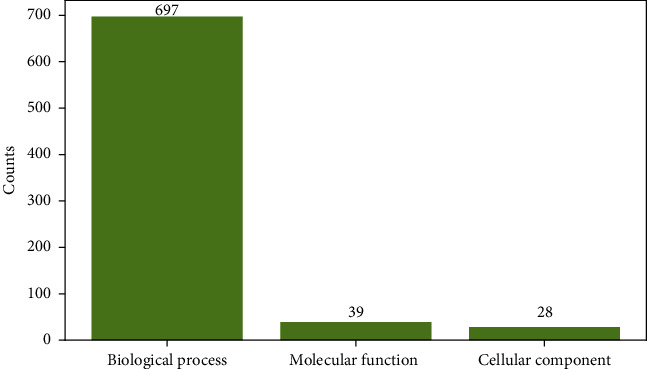
Distribution of GO features used in the best DT classifier on three GO groups. The BP GP terms are the most, followed by MF and CC GO terms.

805 features were used in the best DT classifier, which are the top 805 features in Table S1. Among them, 41 features were related to KEGG pathways, whereas the remaining 764 features were about GO terms. It is known that all GO terms can be divided into three groups: biological process (BP), cellular component (CC), and molecular function (MF). The distribution of 764 GO features on three groups is illustrated in Figure 3. It can be observed that BP GO terms were the most, followed by MF and CC GO terms.

### 3.3. Results of Classification Rules by Using the Optimal DT Classifier

The DT is a white-box model that provides clear decision rules and is beneficial for further analysis. Thus, we used these 805 features to construct a DT by using all samples. From such DT, 799 decision rules were extracted (Table S3). A detailed description of these rules is provided in “Discussion.”

## 4. Discussion

The functional enrichment annotations of lymphoma-associated genes were used to identify a group of functional enrichment terms, i.e., GO and KEGG pathway terms, and reveal the key biological effects distinguishing lymphoma-associated genes and other genes. On the basis of machine learning models, we identified a group of terms associated with lymphoma. The detailed discussion on these terms are shown as follows.

### 4.1. Functional Enrichment Terms Associated with Lymphoma-Associated Genes

The first identified functional enrichment term is GO:0042113, describing B-cell activation. Early in 2002, researchers from the University of California, Los Angeles, confirmed that the activation of B cells participates in the initiation and progression of lymphoma, such as in patients with HIV [39]. Further, similar results are validated in South Africa by researchers from the University of the Western Cape in 2018, indicating that B-cell activation is associated with the pathogenesis and progression of lymphoma [40]. Therefore, B-cell activation is an effective biological process associated with lymphoma.

The next identified functional enrichment term is GO:0044424, which describes a cellular component as the obsolete intracellular part and is now named as the intracellular anatomical structure. Although no direct report confirmed that any intracellular structure is specifically associated with the pathogenesis of lymphoma, structural variants associated with programmed cell death-associated proteins are specifically associated with Epstein–Barr virus-associated lymphomas [41]. This finding is consistent with our prediction.

The next identified GO term is the general GO term GO:0010955 that describes the negative regulation of protein processing. Such GO term summarizes any process associated with peptide bond cleavage frequency and protein maturation efficacy. According to recent publications, BAFF has been regarded as an important driver for B-cell non-Hodgkin lymphoma. BAFF and its pathway BAFF/BAFF-R pathway are processed by cleavage from the plasma membrane and transformation into a soluble form [42]. Therefore, functional protein processing, like bond cleavage, may also be essential to trigger lymphoma.

The next two identified GO terms are GO:0032764 (negative regulation of mast cell cytokine production) and GO:0002643 (regulation of tolerance induction). Early in 1982, a long-term in vitro culture of mast cells in mouse models confirmed that mast cell cytokines are associated with the growth and maturation of mast cells and that such cytokines are validated in T-cell lymphoma [43], confirming the correlations between mast cell cytokines and T-cell lymphoma. As for the regulation of tolerance induction, tolerance induction describes a physiological status in which immune cells do not react against antigens or external stimulations. During the pathogenesis of lymphoma, tolerance is quite common in multiple lymphoma subtypes especially for B-cell lymphoma [44–46].

Similarly, although not in the top, we identified KEGG pathways, like hsa04650 (natural killer cell-mediated cytotoxicity) and hsa05202 (transcriptional misregulation in cancer). Early in 1995, T lymphomas are reported to induce optimal natural killer cell-mediated cytotoxicity [47], revealing the correlations between such pathway and lymphoma. Recent reports [48–50] also validated that natural killer cells play an irreplaceable role during lymphoma pathogenesis. As for another KEGG pathway, i.e., transcriptional misregulation in cancer, researchers from the Massachusetts Institute of Technology summarized disease-associated transcriptional regulation and validated that T-cell lymphoma is associated with transcriptional regulations in 2013 [51], thereby validating our prediction.

### 4.2. Quantitative Rules for Functional Enrichment Terms Associated with Lymphoma-Associated Genes

Apart from the identification of lymphoma-associated biological processes, we established quantitative rules by using enriched functional terms. The detailed analyses on top features from three optimal rules are shown as follows.

The first rule involves 59 features. Here, we selected two features for discussion. The first selected feature is the GO term GO:0042113, describing B-cell activation. As we have discussed, such GO term is associated with lymphoma pathogenesis, thereby validating our prediction. The next feature is GO:0007568 (aging). Aging has been shown to be associated with the initiation and progression of lymphoma [52]. Therefore, predicting that aging is a determinative biological process associated with lymphoma is reasonable.

The next rule involves 44 features. Apart from B-cell activation (GO:0042113) and aging (GO:0007568), the GO term GO:0002705, the positive regulation of leukocyte-mediated immunity as a candidate to be associated with lymphoma, is identified. In 2019, researchers from Shanghai Rui Jin Hospital reported that leukocyte-mediated immune responses are associated with the pathogenesis of lymphoma, thereby validating our prediction. In addition, the negative regulation of mitophagy (GO:1901525) has been reported to be associated with lymphoma and this finding has also been supported by recent publications [53, 54]. Overall, such quantitative rule can help the identification of lymphoma-associated genes.

The third rule also includes multiple lymphoma-associated functional terms (GO terms and KEGG pathways). Except for shared GO terms with the abovementioned two rules, like GO:0042113 and GO:0007568, GO:0048539, as another predicted GO term, describes the bone marrow development and has been shown to be associated with lymphoma. According to recent publications, bone marrow biopsy has been regarded as one of the major methods for clinical diagnosis on lymphoma [55]. The development of bone marrow is tightly associated with the initiation and progression of lymphoma, thereby validating our prediction.

Overall, by using machine learning models, we identified a group of functional enrichment terms associated with lymphoma and established quantitative rules for lymphoma prediction. The prediction results that we presented can help promote the exploration on the fundamental pathological mechanisms for lymphoma and provide us a new tool to analyze the functional characteristics of complex diseases.

## 5. Conclusion

This study is aimed at identifying key GO terms and KEGG pathways for lymphoma-associated genes. A total of 805 key features and 799 quantitative rules were identified using a machine learning approach, which has been validated by research results in recent years. This study contributes to a deep understanding of the underlying pathological mechanisms of lymphoma and provides us with new tools to analyze the functional characteristics of the disease.

## Data Availability

The original data used to support the findings of this study are available at DisGeNET (https://www.disgenet.org/).

## Supplementary Materials

Supplementary Materials Table S1: feature list obtained using the Boruta and max-relevance and min-redundancy (mRMR) feature selection methods. Table S2: performance of the decision tree model on the different number of features. Table S3: classification rules obtained by the optimal decision tree model.
