# Supplementary material for: Analysis of Lymphoma-Related Genes with Gene Ontology and Kyoto Encyclopedia of Genes and Genomes Enrichment
Source: Biomed Res Int. 2022 Jun 26;2022:8503511. doi: 10.1155/2022/8503511 (PMC9251090; doi:10.1155/2022/8503511)
Supplement: Supplementary Materials — Table S1: feature list obtained using the Boruta and max-relevance and min-redundancy (mRMR) feature selection methods. Table S2: performance of the decision tree model on the different number of features. Table S3: classification rules obtained by the optimal decision tree model. [file 8503511.f1.zip › Table S1 (1).pdf]

Rank	Features	Name	Class
1	GO:0042113	B cell activation	biological process
2	GO:0044424	intracellular part	cellular component
3	GO:0010955	negative regulation of protein processing	biological process
4	GO:0032764	negative regulation of mast cell cytokine production	biological process
5	GO:0002643	regulation of tolerance induction	biological process
6	GO:0031049	programmed DNA elimination	biological process
7	GO:0045060	negative thymic T cell selection	biological process
8	GO:0033151	V(D)J recombination	biological process
9	GO:0070265	necrotic cell death	biological process
10	GO:0060313	negative regulation of blood vessel remodeling	biological process
11	GO:0002903	negative regulation of B cell apoptotic process	biological process
12	GO:0003908	methylated-DNA-[protein]-cysteine S-methyltransferase activity	molecular function
13	GO:0035259	nuclear glucocorticoid receptor binding	molecular function
14	hsa04650	Natural killer cell mediated cytotoxicity	KEGG
15	GO:0000018	regulation of DNA recombination	biological process
16	GO:0044026	DNA hypermethylation	biological process
17	GO:0042130	negative regulation of T cell proliferation	biological process
18	GO:0005575	cellular component	cellular component
19	GO:2000516	positive regulation of CD4-positive, alpha-beta T cell activation	biological process
20	GO:0048597	post-embryonic camera-type eye morphogenesis	biological process
21	GO:0042287	MHC protein binding	molecular function
22	GO:0001776	leukocyte homeostasis	biological process
23	GO:0030852	regulation of granulocyte differentiation	biological process
24	GO:0002713	negative regulation of B cell mediated immunity	biological process
25	GO:2001242	regulation of intrinsic apoptotic signaling pathway	biological process
26	GO:0002562	somatic diversification of immune receptors via germline recombination within a	biological process
27	GO:0008588	positive regulation of NIK/NF-kappaB signaling	biological process
28	GO:2001238	positive regulation of extrinsic apoptotic signaling pathway	biological process
29	GO:0038001	paracrine signaling	biological process
30	GO:0050851	antigen receptor-mediated signaling pathway	biological process
31	GO:1901989	positive regulation of cell cycle phase transition	biological process
32	GO:0043368	positive T cell selection	biological process
33	GO:0004126	cytidine deaminase activity	molecular function
34	GO:0001783	B cell apoptotic process	biological process

35	GO:0002507	tolerance induction	biological process
36	GO:2001269	positive regulation of cysteine-type endopeptidase activity involved in apoptotic	biological process
37	GO:0032389	MutLalpha complex	cellular component
38	GO:0070141	response to UV-A	biological process
39	GO:0009897	external side of plasma membrane	cellular component
40	GO:0002467	germinal center formation	biological process
41	GO:0071850	germinal center formation	biological process
42	GO:0045579	positive regulation of B cell differentiation	biological process
43	GO:0002664	regulation of T cell tolerance induction	biological process
44	GO:0010605	negative regulation of macromolecule metabolic process	biological process
45	GO:0006979	response to oxidative stress	biological process
46	GO:0023030	MHC class Ib protein binding, via antigen binding groove	molecular function
47	GO:0046634	regulation of alpha-beta T cell activation	biological process
48	GO:0003968	RNA-directed 5'-3' RNA polymerase activity	molecular function
49	GO:0031265	CD95 death-inducing signaling complex	cellular component
50	GO:0070102	interleukin-6-mediated signaling pathway	biological process
51	hsa05202	Transcriptional misregulation in cancer	KEGG
52	GO:0097506	deaminated base DNA N-glycosylase activity	molecular function
53	hsa04940	Type I diabetes mellitus	KEGG
54	GO:0016444	somatic cell DNA recombination	biological process
55	GO:0009008	DNA-methyltransferase activity	molecular function
56	GO:0032845	homeostatic process	biological process
57	GO:0044464	cell part	cellular component
58	GO:0070227	lymphocyte apoptotic process	biological process
59	GO:0051311	meiotic metaphase plate congression	biological process
60	GO:0002902	regulation of B cell apoptotic process	biological process
61	GO:0002644	negative regulation of tolerance induction	biological process
62	GO:0045736	negative regulation of cyclin-dependent protein serine/threonine kinase activity	biological process
63	GO:0045064	T-helper 2 cell differentiation	biological process
64	GO:2000811	negative regulation of anoikis	biological process
65	GO:0070230	positive regulation of lymphocyte apoptotic process	biological process
66	GO:0005031	tumor necrosis factor receptor activity	molecular function
67	GO:0048537	mucosa-associated lymphoid tissue development	biological process
68	GO:0009411	response to UV	biological process
69	GO:0072539	T-helper 17 cell differentiation	biological process

70	GO:0023026	MHC class II protein complex binding	molecular function
71	GO:0019660	glycolytic fermentation	biological process
72	GO:0033256	I-kappaB/NF-kappaB complex	cellular component
73	GO:0002524	hypersensitivity	biological process
74	GO:0031100	animal organ regeneration	biological process
75	GO:0016447	somatic recombination of immunoglobulin gene segments	biological process
76	GO:0031264	death-inducing signaling complex	cellular component
77	GO:0060374	mast cell differentiation	biological process
78	GO:0008327	methyl-CpG binding	molecular function
79	GO:0046635	positive regulation of alpha-beta T cell activation	biological process
80	hsa04640	Hematopoietic cell lineage	KEGG
81	GO:0006275	regulation of DNA replication	biological process
82	GO:0070301	cellular response to hydrogen peroxide	biological process
83	GO:0002200	somatic diversification of immune receptors	biological process
84	GO:0032464	positive regulation of protein homooligomerization	biological process
85	GO:0070266	necroptotic process	biological process
86	GO:1902564	negative regulation of neutrophil activation	biological process
87	GO:0046631	alpha-beta T cell activation	biological process
88	GO:0006359	regulation of transcription by RNA polymerase III	biological process
89	GO:0002517	T cell tolerance induction	biological process
90	GO:1903317	regulation of protein maturation	biological process
91	GO:0016363	nuclear matrix	cellular component
92	GO:0001772	immunological synapse	cellular component
93	GO:2001243	negative regulation of intrinsic apoptotic signaling pathway	biological process
94	GO:0002911	regulation of lymphocyte anergy	biological process
95	GO:0045638	negative regulation of myeloid cell differentiation	biological process
96	GO:2000772	regulation of cellular senescence	biological process
97	GO:0002381	immunoglobulin production involved in immunoglobulin-mediated immune	biological process
98	GO:0090116	C-5 methylation of cytosine	biological process
99	GO:0002890	negative regulation of immunoglobulin mediated immune response	biological process
100	GO:0008625	extrinsic apoptotic signaling pathway via death domain receptors	biological process
101	GO:0042288	MHC class I protein binding	molecular function
102	GO:0032753	positive regulation of interleukin-4 production	biological process
103	hsa05206	MicroRNAs in cancer	KEGG
104	GO:0015671	oxygen transport	biological process

105	GO:0002204	somatic recombination of immunoglobulin genes involved in immune response	biological process
106	GO:1901992	positive regulation of mitotic cell cycle phase transition	biological process
107	GO:0042100	B cell proliferation	biological process
108	GO:0002339	B cell selection	biological process
109	GO:0050794	regulation of cellular process	biological process
110	GO:0045577	regulation of B cell differentiation	biological process
111	GO:0044764	multi-organism cellular process	biological process
112	GO:0031104	dendrite regeneration	biological process
113	GO:0032496	response to lipopolysaccharide	biological process
114	GO:0048294	negative regulation of isotype switching to IgE isotypes	biological process
115	GO:2000773	negative regulation of cellular senescence	biological process
116	GO:0046633	alpha-beta T cell proliferation	biological process
117	GO:0008626	granzyme-mediated apoptotic signaling pathway	biological process
118	GO:0097190	apoptotic signaling pathway	biological process
119	GO:0002283	neutrophil activation involved in immune response	biological process
120	GO:0023023	MHC protein complex binding	molecular function
121	GO:0030888	regulation of B cell proliferation	biological process
122	GO:0047485	protein N-terminus binding	molecular function
123	GO:0007600	sensory perception	biological process
124	GO:0042093	T-helper cell differentiation	biological process
125	GO:0019104	DNA N-glycosylase activity	molecular function
126	GO:0046668	regulation of retinal cell programmed cell death	biological process
127	GO:0051348	negative regulation of transferase activity	biological process
128	GO:0070231	T cell apoptotic process	biological process
129	GO:0002707	negative regulation of lymphocyte mediated immunity	biological process
130	GO:0019222	regulation of metabolic process	biological process
131	GO:0002274	myeloid leukocyte activation	biological process
132	GO:0008152	metabolic process	biological process
133	GO:0010224	response to UV-B	biological process
134	GO:0005035	death receptor activity	molecular function
135	GO:0033077	T cell differentiation in thymus	biological process
136	GO:1903896	positive regulation of IRE1-mediated unfolded protein response	biological process
137	GO:0045829	negative regulation of isotype switching	biological process
138	GO:2000378	negative regulation of reactive oxygen species metabolic process	biological process
139	GO:0043226	organelle	cellular component

140	GO:0000307	cyclin-dependent protein kinase holoenzyme complex	cellular component
141	GO:0002697	regulation of immune effector process	biological process
142	GO:0002666	positive regulation of T cell tolerance induction	biological process
143	GO:0010939	regulation of necrotic cell death	biological process
144	GO:0045621	positive regulation of lymphocyte differentiation	biological process
145	GO:0072341	modified amino acid binding	molecular function
146	GO:0010212	response to ionizing radiation	biological process
147	GO:0045554	regulation of TRAIL production	biological process
148	GO:0030291	protein serine/threonine kinase inhibitor activity	molecular function
149	GO:0042267	natural killer cell mediated cytotoxicity	biological process
150	GO:0042493	response to xenobiotic stimulus	biological process
151	GO:0043011	myeloid dendritic cell differentiation	biological process
152	GO:1900119	positive regulation of execution phase of apoptosis	biological process
153	GO:0046007	negative regulation of activated T cell proliferation	biological process
154	GO:0043154	negative regulation of cysteine-type endopeptidase activity involved in apoptotic	biological process
155	GO:0050852	T cell receptor signaling pathway	biological process
156	GO:0031343	positive regulation of cell killing	biological process
157	GO:0044260	cellular macromolecule metabolic process	biological process
158	GO:0045581	negative regulation of T cell differentiation	biological process
159	GO:0005515	protein binding	molecular function
160	GO:0002208	somatic diversification of immunoglobulins involved in immune response	biological process
161	GO:0031060	regulation of histone methylation	biological process
162	GO:0046898	response to cycloheximide	biological process
163	hsa05219	Bladder cancer	KEGG
164	GO:0005164	tumor necrosis factor receptor binding	molecular function
165	GO:0050897	cobalt ion binding	molecular function
166	GO:1903037	regulation of leukocyte cell-cell adhesion	biological process
167	GO:0031052	programmed DNA elimination by chromosome breakage	biological process
168	GO:0097193	intrinsic apoptotic signaling pathway	biological process
169	GO:0002885	positive regulation of hypersensitivity	biological process
170	GO:0002684	positive regulation of immune system process	biological process
171	GO:0050798	activated T cell proliferation	biological process
172	GO:0046500	S-adenosylmethionine metabolic process	biological process
173	GO:0046982	protein heterodimerization activity	molecular function
174	GO:0043369	CD4-positive or CD8-positive, alpha-beta T cell lineage commitment	biological process

175	GO:0043280	positive regulation of cysteine-type endopeptidase activity involved in apoptotic	biological process
176	GO:0002366	leukocyte activation involved in immune response	biological process
177	GO:0097694	establishment of RNA localization to telomere	biological process
178	GO:0042981	regulation of apoptotic process	biological process
179	GO:0080090	regulation of primary metabolic process	biological process
180	GO:0071478	cellular response to radiation	biological process
181	GO:0045087	innate immune response	biological process
182	hsa00983	Drug metabolism - other enzymes	KEGG
183	GO:0016445	somatic diversification of immunoglobulins	biological process
184	GO:0010039	response to iron ion	biological process
185	GO:0044446	intracellular organelle part	cellular component
186	GO:0070513	death domain binding	molecular function
187	GO:0045076	regulation of interleukin-2 production	biological process
188	GO:0005488	binding	molecular function
189	GO:0001913	T cell mediated cytotoxicity	biological process
190	GO:0019814	immunoglobulin complex	cellular component
191	GO:0045619	regulation of lymphocyte differentiation	biological process
192	GO:0000783	nuclear telomere cap complex	cellular component
193	GO:0048144	fibroblast proliferation	biological process
194	GO:0042110	T cell activation	biological process
195	GO:0010033	response to organic substance	biological process
196	GO:2000757	negative regulation of peptidyl-lysine acetylation	biological process
197	GO:1903318	negative regulation of protein maturation	biological process
198	GO:0032943	mononuclear cell proliferation	biological process
199	GO:0042994	cytoplasmic sequestering of transcription factor	biological process
200	GO:0051054	positive regulation of DNA metabolic process	biological process
201	GO:1903147	negative regulation of autophagy of mitochondrion	biological process
202	GO:0043029	T cell homeostasis	biological process
203	GO:0098552	side of membrane	cellular component
204	GO:0046498	S-adenosylhomocysteine metabolic process	biological process
205	GO:0060576	intestinal epithelial cell development	biological process
206	GO:0052548	regulation of endopeptidase activity	biological process
207	GO:0002293	alpha-beta T cell differentiation involved in immune response	biological process
208	GO:0032300	mismatch repair complex	cellular component
209	GO:1904029	regulation of cyclin-dependent protein kinase activity	biological process

210	GO:0035710	CD4-positive, alpha-beta T cell activation	biological process
211	GO:0008630	intrinsic apoptotic signaling pathway in response to DNA damage	biological process
212	GO:0038117	C-C motif chemokine 19 receptor activity	molecular function
213	GO:0032623	interleukin-2 production	biological process
214	GO:0019661	glucose catabolic process to lactate via pyruvate	biological process
215	GO:0072577	endothelial cell apoptotic process	biological process
216	GO:1902583	multi-organism intracellular transport	biological process
217	GO:0060255	regulation of macromolecule metabolic process	biological process
218	hsa05215	Prostate cancer	KEGG
219	GO:0045830	positive regulation of isotype switching	biological process
220	GO:0070887	cellular response to chemical stimulus	biological process
221	GO:2001251	negative regulation of chromosome organization	biological process
222	GO:0034111	negative regulation of homotypic cell-cell adhesion	biological process
223	GO:0043229	intracellular organelle	cellular component
224	GO:0000060	protein import into nucleus, translocation	biological process
225	GO:0042092	type 2 immune response	biological process
226	GO:0006927	transformed cell apoptotic process	biological process
227	GO:0097153	cysteine-type endopeptidase activity involved in apoptotic process	molecular function
228	GO:0046983	protein dimerization activity	molecular function
229	GO:0003720	telomerase activity	molecular function
230	GO:0034121	regulation of toll-like receptor signaling pathway	biological process
231	hsa05320	Autoimmune thyroid disease	KEGG
232	GO:0014042	positive regulation of neuron maturation	biological process
233	GO:0043227	membrane-bounded organelle	cellular component
234	GO:0005622	intracellular anatomical structure	cellular component
235	GO:0050865	regulation of cell activation	biological process
236	GO:0016265	death	biological process
237	GO:0006346	DNA methylation-dependent heterochromatin assembly	biological process
238	GO:0031324	negative regulation of cellular metabolic process	biological process
239	GO:0048305	immunoglobulin production	biological process
240	GO:0008156	negative regulation of DNA replication	biological process
241	GO:0001782	B cell homeostasis	biological process
242	GO:0045637	regulation of myeloid cell differentiation	biological process
243	GO:0043372	positive regulation of CD4-positive, alpha-beta T cell differentiation	biological process
244	GO:1990572	TERT-RMRP complex	cellular component

245	GO:0032504	multicellular organism reproduction	biological process
246	GO:0019058	viral life cycle	biological process
247	GO:0006919	activation of cysteine-type endopeptidase activity involved in apoptotic process	biological process
248	GO:0005634	nucleus	cellular component
249	GO:0046632	alpha-beta T cell differentiation	biological process
250	GO:0032268	regulation of cellular protein metabolic process	biological process
251	GO:0042269	regulation of natural killer cell mediated cytotoxicity	biological process
252	GO:0072710	response to hydroxyurea	biological process
253	GO:0036296	response to increased oxygen levels	biological process
254	GO:0043367	CD4-positive, alpha-beta T cell differentiation	biological process
255	GO:0070424	regulation of nucleotide-binding oligomerization domain containing signaling	biological process
256	GO:0006955	immune response	biological process
257	GO:0048519	negative regulation of biological process	biological process
258	GO:0090342	regulation of cell aging	biological process
259	GO:0032743	positive regulation of interleukin-2 production	biological process
260	GO:0007049	cell cycle	biological process
261	GO:0045620	negative regulation of lymphocyte differentiation	biological process
262	GO:0008635	activation of cysteine-type endopeptidase activity involved in apoptotic process by	biological process
263	GO:0072610	interleukin-12 production	biological process
264	GO:0032848	negative regulation of cellular pH reduction	biological process
265	GO:0043231	intracellular membrane-bounded organelle	cellular component
266	GO:0009615	response to virus	biological process
267	GO:0045931	positive regulation of mitotic cell cycle	biological process
268	GO:0045945	positive regulation of transcription by RNA polymerase III	biological process
269	GO:1903428	positive regulation of reactive oxygen species biosynthetic process	biological process
270	GO:0032759	positive regulation of TRAIL production	biological process
271	GO:0035726	common myeloid progenitor cell proliferation	biological process
272	GO:0002763	positive regulation of myeloid leukocyte differentiation	biological process
273	GO:0060011	Sertoli cell proliferation	biological process
274	GO:0030098	lymphocyte differentiation	biological process
275	GO:0030887	positive regulation of myeloid dendritic cell activation	biological process
276	GO:2001020	regulation of response to DNA damage stimulus	biological process
277	hsa04672	Intestinal immune network for IgA production	KEGG
278	GO:0002376	immune system process	biological process
279	GO:0050730	regulation of peptidyl-tyrosine phosphorylation	biological process

280	GO:0022408	negative regulation of cell-cell adhesion	biological process
281	GO:0051400	BH domain binding	molecular function
282	GO:0033209	tumor necrosis factor-mediated signaling pathway	biological process
283	GO:0010216	maintenance of DNA methylation	biological process
284	GO:0071887	leukocyte apoptotic process	biological process
285	GO:0006285	base-excision repair, AP site formation	biological process
286	GO:0045061	thymic T cell selection	biological process
287	GO:0045402	regulation of interleukin-4 production	biological process
288	GO:0000079	regulation of cyclin-dependent protein serine/threonine kinase activity	biological process
289	GO:0050864	regulation of B cell activation	biological process
290	GO:0003953	NAD+ nucleosidase activity	molecular function
291	GO:0071704	organic substance metabolic process	biological process
292	GO:0030983	mismatched DNA binding	molecular function
293	GO:0097202	activation of cysteine-type endopeptidase activity	biological process
294	GO:0048523	negative regulation of cellular process	biological process
295	GO:0045066	regulatory T cell differentiation	biological process
296	GO:0044238	primary metabolic process	biological process
297	GO:0009314	response to radiation	biological process
298	GO:0006266	DNA ligation	biological process
299	GO:0002891	positive regulation of immunoglobulin mediated immune response	biological process
300	GO:0055064	chloride ion homeostasis	biological process
301	GO:0030097	hemopoiesis	biological process
302	GO:1902166	negative regulation of intrinsic apoptotic signaling pathway in response to DNA	biological process
303	GO:1902532	negative regulation of intracellular signal transduction	biological process
304	GO:0046636	negative regulation of alpha-beta T cell activation	biological process
305	GO:0030851	granulocyte differentiation	biological process
306	GO:1903039	positive regulation of leukocyte cell-cell adhesion	biological process
307	GO:1904868	telomerase catalytic core complex assembly	biological process
308	GO:0051454	intracellular pH elevation	biological process
309	GO:0001773	myeloid dendritic cell activation	biological process
310	GO:0002287	alpha-beta T cell activation involved in immune response	biological process
311	GO:0051052	regulation of DNA metabolic process	biological process
312	hsa04215	Apoptosis - multiple species	KEGG
313	hsa04612	Antigen processing and presentation	KEGG
314	GO:0031341	regulation of cell killing	biological process

315	GO:0031323	regulation of cellular metabolic process	biological process
316	hsa05161	Hepatitis B	KEGG
317	GO:0009264	deoxyribonucleotide catabolic process	biological process
318	GO:0003964	RNA-directed DNA polymerase activity	molecular function
319	GO:0034103	regulation of tissue remodeling	biological process
320	GO:0048539	bone marrow development	biological process
321	GO:0009892	negative regulation of metabolic process	biological process
322	GO:0070233	negative regulation of T cell apoptotic process	biological process
323	GO:0002377	immunoglobulin production	biological process
324	hsa04210	Apoptosis	KEGG
325	hsa04115	p53 signaling pathway	KEGG
326	GO:2001233	regulation of apoptotic signaling pathway	biological process
327	GO:0070245	positive regulation of thymocyte apoptotic process	biological process
328	GO:0045580	regulation of T cell differentiation	biological process
329	hsa04110	Cell cycle	KEGG
330	hsa04064	NF-kappa B signaling pathway	KEGG
331	GO:0010165	response to X-ray	biological process
332	hsa04060	Cytokine-cytokine receptor interaction	KEGG
333	GO:0050671	positive regulation of lymphocyte proliferation	biological process
334	hsa04668	TNF signaling pathway	KEGG
335	hsa04662	B cell receptor signaling pathway	KEGG
336	GO:0045639	positive regulation of myeloid cell differentiation	biological process
337	hsa05169	Epstein-Barr virus infection	KEGG
338	hsa05166	Human T-cell leukemia virus 1 infection	KEGG
339	GO:0050866	negative regulation of cell activation	biological process
340	GO:0051053	negative regulation of DNA metabolic process	biological process
341	hsa05164	Influenza A	KEGG
342	GO:1901030	positive regulation of mitochondrial outer membrane permeabilization involved in	biological process
343	GO:0097191	extrinsic apoptotic signaling pathway	biological process
344	hsa05162	Measles	KEGG
345	GO:0001912	positive regulation of leukocyte mediated cytotoxicity	biological process
346	GO:0006306	DNA methylation	biological process
347	hsa05222	Small cell lung cancer	KEGG
348	GO:0045589	regulation of regulatory T cell differentiation	biological process
349	GO:0048145	regulation of fibroblast proliferation	biological process

350	hsa05221	Acute myeloid leukemia	KEGG
351	hsa05220	Chronic myeloid leukemia	KEGG
352	hsa05340	Primary immunodeficiency	KEGG
353	GO:0002292	T cell differentiation involved in immune response	biological process
354	hsa05332	Graft-versus-host disease	KEGG
355	GO:0045471	response to ethanol	biological process
356	GO:0042162	telomeric DNA binding	molecular function
357	hsa05330	Allograft rejection	KEGG
358	GO:0002460	adaptive immune response based on somatic recombination of immune receptors	biological process
359	hsa05210	Colorectal cancer	KEGG
360	GO:0036498	IRE1-mediated unfolded protein response	biological process
361	GO:0048545	response to steroid hormone	biological process
362	GO:2001236	regulation of extrinsic apoptotic signaling pathway	biological process
363	GO:0045930	negative regulation of mitotic cell cycle	biological process
364	GO:2000106	regulation of leukocyte apoptotic process	biological process
365	GO:0046006	regulation of activated T cell proliferation	biological process
366	hsa05321	Inflammatory bowel disease	KEGG
367	GO:0001552	ovarian follicle atresia	biological process
368	GO:0002285	lymphocyte activation involved in immune response	biological process
369	GO:0001172	transcription, RNA-templated	biological process
370	GO:0060574	intestinal epithelial cell maturation	biological process
371	GO:0002761	regulation of myeloid leukocyte differentiation	biological process
372	GO:0000737	DNA catabolic process, endonucleolytic	biological process
373	GO:0000723	telomere maintenance	biological process
374	GO:0048583	regulation of response to stimulus	biological process
375	GO:0043122	regulation of I-kappaB kinase/NF-kappaB signaling	biological process
376	GO:0000302	response to reactive oxygen species	biological process
377	GO:0000097	sulfur amino acid biosynthetic process	biological process
378	GO:0050868	negative regulation of T cell activation	biological process
379	GO:0001779	natural killer cell differentiation	biological process
380	GO:0033158	regulation of protein import into nucleus, translocation	biological process
381	GO:0001777	T cell homeostatic proliferation	biological process
382	GO:0072593	reactive oxygen species metabolic process	biological process
383	GO:0045954	positive regulation of natural killer cell mediated cytotoxicity	biological process
384	GO:0033044	regulation of chromosome organization	biological process

385	GO:0034112	positive regulation of homotypic cell-cell adhesion	biological process
386	GO:0001910	regulation of leukocyte mediated cytotoxicity	biological process
387	GO:0001909	leukocyte mediated cytotoxicity	biological process
388	GO:0001889	liver development	biological process
389	GO:0045582	positive regulation of T cell differentiation	biological process
390	GO:0001844	protein insertion into mitochondrial membrane involved in apoptotic signaling	biological process
391	GO:0060249	anatomical structure homeostasis	biological process
392	GO:0046483	heterocycle metabolic process	biological process
393	GO:0002263	cell activation involved in immune response	biological process
394	GO:2001234	negative regulation of apoptotic signaling pathway	biological process
395	GO:0002262	myeloid cell homeostasis	biological process
396	GO:0002698	negative regulation of immune effector process	biological process
397	GO:0002260	lymphocyte homeostasis	biological process
398	GO:0002253	activation of immune response	biological process
399	GO:0007050	regulation of cell cycle	biological process
400	GO:0002246	wound healing involved in inflammatory response	biological process
401	GO:0098609	cell-cell adhesion	biological process
402	GO:0002237	response to molecule of bacterial origin	biological process
403	GO:0002228	natural killer cell mediated immunity	biological process
404	GO:0048660	regulation of smooth muscle cell proliferation	biological process
405	GO:0002331	pre-B cell allelic exclusion	biological process
406	GO:0002822	regulation of adaptive immune response based on somatic recombination of immune	biological process
407	GO:0002329	pre-B cell differentiation	biological process
408	GO:0071901	negative regulation of protein serine/threonine kinase activity	biological process
409	GO:0002327	immature B cell differentiation	biological process
410	GO:0033598	mammary gland epithelial cell proliferation	biological process
411	GO:0002326	B cell lineage commitment	biological process
412	GO:0045591	positive regulation of regulatory T cell differentiation	biological process
413	GO:0009262	deoxyribonucleotide metabolic process	biological process
414	GO:0002318	myeloid progenitor cell differentiation	biological process
415	GO:0002312	B cell activation involved in immune response	biological process
416	GO:0008283	cell population proliferation	biological process
417	GO:0002309	T cell proliferation involved in immune response	biological process
418	GO:0002250	adaptive immune response	biological process
419	GO:0033554	cellular response to stress	biological process

420	GO:0002294	CD4-positive, alpha-beta T cell differentiation involved in immune response	biological process
421	GO:0002363	alpha-beta T cell lineage commitment	biological process
422	GO:0033993	response to lipid	biological process
423	GO:0002360	T cell lineage commitment	biological process
424	GO:0043281	regulation of cysteine-type endopeptidase activity involved in apoptotic process	biological process
425	GO:0002358	B cell homeostatic proliferation	biological process
426	GO:0002352	B cell negative selection	biological process
427	GO:0045346	regulation of MHC class II biosynthetic process	biological process
428	GO:0002449	lymphocyte mediated immunity	biological process
429	GO:0002443	leukocyte mediated immunity	biological process
430	GO:0002440	production of molecular mediator of immune response	biological process
431	GO:0050867	positive regulation of cell activation	biological process
432	GO:0002439	chronic inflammatory response to antigenic stimulus	biological process
433	GO:0044092	negative regulation of molecular function	biological process
434	GO:0097159	organic cyclic compound binding	molecular function
435	GO:0002438	acute inflammatory response to antigenic stimulus	biological process
436	GO:1902275	regulation of chromatin organization	biological process
437	GO:0002889	regulation of immunoglobulin mediated immune response	biological process
438	GO:0002521	leukocyte differentiation	biological process
439	GO:0002520	immune system development	biological process
440	GO:0010941	regulation of cell death	biological process
441	GO:0002638	negative regulation of immunoglobulin production	biological process
442	GO:0032479	regulation of type I interferon production	biological process
443	GO:0071301	cellular response to vitamin B1	biological process
444	GO:0097028	dendritic cell differentiation	biological process
445	GO:0002637	regulation of immunoglobulin production	biological process
446	GO:0036037	CD8-positive, alpha-beta T cell activation	biological process
447	GO:0045787	positive regulation of cell cycle	biological process
448	GO:0002636	positive regulation of germinal center formation	biological process
449	GO:0045321	leukocyte activation	biological process
450	GO:0002634	regulation of germinal center formation	biological process
451	GO:0001836	release of cytochrome c from mitochondria	biological process
452	GO:0002573	myeloid leukocyte differentiation	biological process
453	GO:0007253	cytoplasmic sequestering of NF-kappaB	biological process
454	GO:0002665	negative regulation of T cell tolerance induction	biological process

455	GO:0002286	T cell activation involved in immune response	biological process
456	hsa03410	Base excision repair	KEGG
457	GO:0002683	negative regulation of immune system process	biological process
458	GO:0010038	response to metal ion	biological process
459	GO:0002682	regulation of immune system process	biological process
460	GO:0051984	positive regulation of chromosome segregation	biological process
461	GO:0002252	immune effector process	biological process
462	GO:0002668	negative regulation of T cell anergy	biological process
463	GO:0002696	positive regulation of leukocyte activation	biological process
464	GO:0002695	negative regulation of leukocyte activation	biological process
465	GO:0071260	cellular response to mechanical stimulus	biological process
466	GO:0034397	telomere localization	biological process
467	GO:0002639	positive regulation of immunoglobulin production	biological process
468	GO:0043170	macromolecule metabolic process	biological process
469	GO:0008219	cell death	biological process
470	GO:0002694	regulation of leukocyte activation	biological process
471	GO:0002704	negative regulation of leukocyte mediated immunity	biological process
472	GO:0048304	positive regulation of isotype switching to IgG isotypes	biological process
473	hsa04660	T cell receptor signaling pathway	KEGG
474	GO:0038052	nuclear receptor activity	molecular function
475	GO:0006139	nucleobase-containing compound metabolic process	biological process
476	GO:0070661	leukocyte proliferation	biological process
477	GO:0022409	positive regulation of cell-cell adhesion	biological process
478	GO:0002703	regulation of leukocyte mediated immunity	biological process
479	GO:0000082	G1/S transition of mitotic cell cycle	biological process
480	GO:0002719	negative regulation of cytokine production involved in immune response	biological process
481	GO:0042127	regulation of cell population proliferation	biological process
482	GO:0002718	regulation of cytokine production involved in immune response	biological process
483	GO:0002717	positive regulation of natural killer cell mediated immunity	biological process
484	GO:0010952	positive regulation of peptidase activity	biological process
485	GO:0002715	regulation of natural killer cell mediated immunity	biological process
486	GO:0002714	positive regulation of B cell mediated immunity	biological process
487	GO:0046651	lymphocyte proliferation	biological process
488	GO:0002883	regulation of hypersensitivity	biological process
489	GO:0002864	regulation of acute inflammatory response to antigenic stimulus	biological process

490	GO:1904019	epithelial cell apoptotic process	biological process
491	GO:0002863	positive regulation of inflammatory response to antigenic stimulus	biological process
492	GO:0002832	negative regulation of response to biotic stimulus	biological process
493	GO:1903038	negative regulation of leukocyte cell-cell adhesion	biological process
494	GO:0002829	negative regulation of type 2 immune response	biological process
495	GO:0002820	negative regulation of adaptive immune response	biological process
496	GO:2001235	positive regulation of apoptotic signaling pathway	biological process
497	GO:0002819	regulation of adaptive immune response	biological process
498	GO:0006113	fermentation	biological process
499	GO:0048872	homeostasis of number of cells	biological process
500	GO:0006260	DNA replication	biological process
501	GO:0071456	cellular response to hypoxia	biological process
502	GO:0006259	DNA metabolic process	biological process
503	GO:0002712	regulation of B cell mediated immunity	biological process
504	GO:0006244	pyrimidine nucleotide catabolic process	biological process
505	GO:0006216	cytidine catabolic process	biological process
506	GO:0001775	cell activation	biological process
507	GO:0032606	type I interferon production	biological process
508	GO:0006213	pyrimidine nucleoside metabolic process	biological process
509	GO:0006305	DNA alkylation	biological process
510	GO:0009991	response to extracellular stimulus	biological process
511	GO:0006304	DNA modification	biological process
512	GO:0006809	nitric oxide biosynthetic process	biological process
513	GO:0050670	regulation of lymphocyte proliferation	biological process
514	GO:0006808	regulation of nitrogen utilization	biological process
515	GO:0006555	methionine metabolic process	biological process
516	GO:0097192	extrinsic apoptotic signaling pathway in absence of ligand	biological process
517	GO:0006974	cellular response to DNA damage stimulus	biological process
518	GO:0006968	cellular defense response	biological process
519	GO:0071391	cellular response to estrogen stimulus	biological process
520	GO:0007127	meiosis I	biological process
521	GO:0007126	meiotic cell cycle	biological process
522	GO:0050863	regulation of T cell activation	biological process
523	GO:0007089	traversing start control point of mitotic cell cycle	biological process
524	GO:0006725	cellular aromatic compound metabolic process	biological process

525	GO:0043525	positive regulation of neuron apoptotic process	biological process
526	GO:0032480	negative regulation of type I interferon production	biological process
527	GO:0002708	positive regulation of lymphocyte mediated immunity	biological process
528	GO:0007064	mitotic sister chromatid cohesion	biological process
529	GO:0008150	biological process	biological process
530	GO:0007584	response to nutrient	biological process
531	GO:0012501	programmed cell death	biological process
532	GO:0007569	cell aging	biological process
533	GO:0016337	cell-cell adhesion	biological process
534	GO:0071216	cellular response to biotic stimulus	biological process
535	GO:0007568	aging	biological process
536	GO:0007565	female pregnancy	biological process
537	GO:0045003	double-strand break repair via synthesis-dependent strand annealing	biological process
538	GO:0007406	negative regulation of neuroblast proliferation	biological process
539	GO:0070741	response to interleukin-6	biological process
540	GO:0008340	determination of adult lifespan	biological process
541	hsa05223	Non-small cell lung cancer	KEGG
542	GO:0002705	positive regulation of leukocyte mediated immunity	biological process
543	GO:0008285	negative regulation of cell population proliferation	biological process
544	GO:0009223	pyrimidine deoxyribonucleotide catabolic process	biological process
545	GO:0009219	pyrimidine deoxyribonucleotide metabolic process	biological process
546	GO:1903426	regulation of reactive oxygen species biosynthetic process	biological process
547	GO:0009164	nucleoside catabolic process	biological process
548	GO:0009086	methionine biosynthetic process	biological process
549	hsa04630	JAK-STAT signaling pathway	KEGG
550	GO:0031099	regeneration	biological process
551	GO:1990391	DNA repair complex	cellular component
552	GO:0009636	response to toxic substance	biological process
553	GO:0071706	tumor necrosis factor superfamily cytokine production	biological process
554	GO:0009628	response to abiotic stimulus	biological process
555	GO:0009987	cellular process	biological process
556	GO:0009972	cytidine deamination	biological process
557	GO:0045785	positive regulation of cell adhesion	biological process
558	GO:0010332	response to gamma radiation	biological process
559	GO:0010225	response to UV-C	biological process

560	GO:0032615	interleukin-12 production	biological process
561	GO:0010835	regulation of protein ADP-ribosylation	biological process
562	GO:0010663	positive regulation of striated muscle cell apoptotic process	biological process
563	GO:0043627	response to estrogen	biological process
564	GO:0034502	protein localization to chromosome	biological process
565	GO:0010639	negative regulation of organelle organization	biological process
566	GO:0002706	regulation of lymphocyte mediated immunity	biological process
567	GO:0010638	positive regulation of organelle organization	biological process
568	GO:0010950	positive regulation of endopeptidase activity	biological process
569	GO:0051402	neuron apoptotic process	biological process
570	GO:0010948	negative regulation of cell cycle process	biological process
571	GO:0016064	immunoglobulin mediated immune response	biological process
572	GO:0016032	viral process	biological process
573	GO:1901099	negative regulation of signal transduction in absence of ligand	biological process
574	GO:0015672	inorganic cation transmembrane transport	biological process
575	GO:0019659	glucose catabolic process to lactate	biological process
576	GO:0001819	positive regulation of cytokine production	biological process
577	GO:1901525	negative regulation of mitophagy	biological process
578	GO:0022407	regulation of cell-cell adhesion	biological process
579	hsa05203	Viral carcinogenesis	KEGG
580	GO:0007159	leukocyte cell-cell adhesion	biological process
581	GO:0022402	cell cycle process	biological process
582	GO:0019740	nitrogen utilization	biological process
583	GO:0031638	zymogen activation	biological process
584	GO:0019724	B cell mediated immunity	biological process
585	GO:0019692	deoxyribose phosphate metabolic process	biological process
586	GO:0030644	cellular chloride ion homeostasis	biological process
587	GO:0006915	apoptotic process	biological process
588	GO:0030225	macrophage differentiation	biological process
589	GO:0002821	positive regulation of adaptive immune response	biological process
590	GO:0030217	T cell differentiation	biological process
591	GO:0030183	B cell differentiation	biological process
592	GO:0046677	response to antibiotic	biological process
593	GO:0044454	nuclear chromosome part	cellular component
594	GO:0042129	regulation of T cell proliferation	biological process

595	GO:0030155	regulation of cell adhesion	biological process
596	GO:0008637	apoptotic mitochondrial changes	biological process
597	GO:0030101	natural killer cell activation	biological process
598	GO:0008284	positive regulation of cell population proliferation	biological process
599	GO:0030099	myeloid cell differentiation	biological process
600	GO:0030890	positive regulation of B cell proliferation	biological process
601	GO:0002484	antigen processing and presentation of endogenous peptide antigen via MHC class I	biological process
602	GO:0034109	homotypic cell-cell adhesion	biological process
603	GO:0030889	negative regulation of B cell proliferation	biological process
604	GO:0031297	replication fork processing	biological process
605	GO:0032770	positive regulation of monooxygenase activity	biological process
606	GO:0031295	T cell costimulation	biological process
607	GO:0002699	positive regulation of immune effector process	biological process
608	GO:0031294	lymphocyte costimulation	biological process
609	GO:0032461	positive regulation of protein oligomerization	biological process
610	GO:0032355	response to estradiol	biological process
611	GO:0007260	tyrosine phosphorylation of STAT protein	biological process
612	GO:0032269	negative regulation of cellular protein metabolic process	biological process
613	GO:0036294	cellular response to decreased oxygen levels	biological process
614	GO:0032200	telomere organization	biological process
615	GO:0032069	regulation of nuclease activity	biological process
616	GO:0005694	chromosome	cellular component
617	GO:0009607	response to biotic stimulus	biological process
618	GO:0032703	negative regulation of interleukin-2 production	biological process
619	GO:0032680	regulation of tumor necrosis factor production	biological process
620	GO:0072131	kidney mesenchyme morphogenesis	biological process
621	GO:0070486	leukocyte aggregation	biological process
622	GO:0006298	mismatch repair	biological process
623	GO:0032679	regulation of TRAIL production	biological process
624	GO:0032673	regulation of interleukin-4 production	biological process
625	GO:2000379	positive regulation of reactive oxygen species metabolic process	biological process
626	GO:0032663	regulation of interleukin-2 production	biological process
627	GO:0032649	regulation of interferon-gamma production	biological process
628	GO:0032633	interleukin-4 production	biological process
629	GO:0031347	regulation of defense response	biological process

630	GO:0032763	regulation of mast cell cytokine production	biological process
631	GO:0032762	mast cell cytokine production	biological process
632	GO:0006284	base-excision repair	biological process
633	GO:0023056	positive regulation of signaling	biological process
634	GO:0032761	positive regulation of lymphotoxin A production	biological process
635	GO:0033033	negative regulation of myeloid cell apoptotic process	biological process
636	GO:0045073	regulation of chemokine production	biological process
637	GO:0032946	positive regulation of mononuclear cell proliferation	biological process
638	GO:0032945	negative regulation of mononuclear cell proliferation	biological process
639	GO:0032944	regulation of mononuclear cell proliferation	biological process
640	GO:0033343	positive regulation of collagen binding	biological process
641	GO:0048146	positive regulation of fibroblast proliferation	biological process
642	GO:0033273	response to vitamin	biological process
643	GO:0051246	regulation of protein metabolic process	biological process
644	GO:0033262	regulation of nuclear cell cycle DNA replication	biological process
645	GO:0033689	negative regulation of osteoblast proliferation	biological process
646	GO:0034110	regulation of homotypic cell-cell adhesion	biological process
647	GO:0034101	erythrocyte homeostasis	biological process
648	GO:0034097	response to cytokine	biological process
649	GO:0031667	response to nutrient levels	biological process
650	GO:0035067	negative regulation of histone acetylation	biological process
651	GO:0080134	regulation of response to stress	biological process
652	GO:0042035	regulation of cytokine production	biological process
653	GO:0034644	cellular response to UV	biological process
654	GO:0003684	damaged DNA binding	molecular function
655	GO:1904035	regulation of epithelial cell apoptotic process	biological process
656	GO:0034614	cellular response to reactive oxygen species	biological process
657	GO:0034612	response to tumor necrosis factor	biological process
658	GO:0034599	cellular response to oxidative stress	biological process
659	GO:0010942	positive regulation of cell death	biological process
660	GO:0035872	nucleotide-binding domain, leucine rich repeat containing receptor signaling	biological process
661	GO:1902554	serine/threonine protein kinase complex	cellular component
662	GO:0070489	T cell aggregation	biological process
663	GO:0035825	homologous recombination	biological process
664	GO:1901360	organic cyclic compound metabolic process	biological process

665	GO:0035732	nitric oxide storage	biological process
666	GO:0070198	protein localization to chromosome, telomeric region	biological process
667	GO:0043374	CD8-positive, alpha-beta T cell differentiation	biological process
668	GO:0043066	negative regulation of apoptotic process	biological process
669	GO:0038116	chemokine (C-C motif) ligand 21 signaling pathway	biological process
670	GO:0038115	chemokine (C-C motif) ligand 19 signaling pathway	biological process
671	GO:0038065	collagen-activated signaling pathway	biological process
672	GO:0038061	NIK/NF-kappaB signaling	biological process
673	GO:0009967	positive regulation of signal transduction	biological process
674	GO:0038034	signal transduction in absence of ligand	biological process
675	GO:0042098	T cell proliferation	biological process
676	GO:0042097	interleukin-4 production	biological process
677	GO:0042368	vitamin D biosynthetic process	biological process
678	GO:0042094	interleukin-2 production	biological process
679	GO:0042108	positive regulation of cytokine production	biological process
680	hsa05145	Toxoplasmosis	KEGG
681	GO:0042107	cytokine production	biological process
682	GO:0042104	positive regulation of activated T cell proliferation	biological process
683	GO:0001666	response to hypoxia	biological process
684	GO:0042102	positive regulation of T cell proliferation	biological process
685	GO:0042991	transcription factor import into nucleus	biological process
686	GO:0001816	cytokine production	biological process
687	GO:0042771	intrinsic apoptotic signaling pathway in response to DNA damage by p53 class	biological process
688	GO:1901363	heterocyclic compound binding	molecular function
689	GO:0042770	signal transduction in response to DNA damage	biological process
690	GO:0042542	response to hydrogen peroxide	biological process
691	GO:0006952	defense response	biological process
692	GO:0042531	positive regulation of tyrosine phosphorylation of STAT protein	biological process
693	GO:0042522	regulation of tyrosine phosphorylation of STAT protein	biological process
694	GO:0043067	regulation of programmed cell death	biological process
695	GO:0042509	regulation of tyrosine phosphorylation of STAT protein	biological process
696	GO:0097022	lymphocyte migration into lymph node	biological process
697	GO:0043065	positive regulation of apoptotic process	biological process
698	GO:0045840	positive regulation of mitotic nuclear division	biological process
699	GO:0071222	cellular response to lipopolysaccharide	biological process

700	GO:0010833	telomere maintenance via telomere lengthening	biological process
701	GO:0043060	meiotic metaphase I plate congression	biological process
702	GO:0043276	anoikis	biological process
703	GO:0098602	cell adhesion	biological process
704	GO:0043207	response to external biotic stimulus	biological process
705	GO:0090329	regulation of DNA-templated DNA replication	biological process
706	GO:0042306	regulation of protein import into nucleus	biological process
707	GO:0043200	response to amino acid	biological process
708	GO:0043371	negative regulation of CD4-positive, alpha-beta T cell differentiation	biological process
709	GO:0043370	regulation of CD4-positive, alpha-beta T cell differentiation	biological process
710	GO:0002700	regulation of production of molecular mediator of immune response	biological process
711	GO:0043465	regulation of fermentation	biological process
712	GO:0043383	negative T cell selection	biological process
713	GO:0014070	response to organic cyclic compound	biological process
714	GO:0043380	regulation of memory T cell differentiation	biological process
715	GO:0043379	memory T cell differentiation	biological process
716	GO:0001817	regulation of cytokine production	biological process
717	GO:0090304	nucleic acid metabolic process	biological process
718	GO:0043375	CD8-positive, alpha-beta T cell lineage commitment	biological process
719	GO:0044728	DNA methylation or demethylation	biological process
720	GO:0044710	metabolic process	biological process
721	GO:0043068	positive regulation of programmed cell death	biological process
722	GO:0044419	biological process involved in interspecies interaction between organisms	biological process
723	GO:0044409	entry into host	biological process
724	GO:0044403	biological process involved in symbiotic interaction	biological process
725	GO:0044346	fibroblast apoptotic process	biological process
726	GO:0016571	histone methylation	biological process
727	GO:1904894	positive regulation of receptor signaling pathway via STAT	biological process
728	GO:0045058	T cell selection	biological process
729	GO:0045008	depyrimidination	biological process
730	GO:0071219	cellular response to molecule of bacterial origin	biological process
731	GO:0045191	regulation of isotype switching	biological process
732	GO:0045190	isotype switching	biological process
733	GO:0045143	homologous chromosome segregation	biological process
734	GO:0048661	positive regulation of smooth muscle cell proliferation	biological process

735	GO:0045141	meiotic telomere clustering	biological process
736	GO:0045366	regulation of interleukin-13 production	biological process
737	GO:0045553	TRAIL production	biological process
738	GO:0045429	positive regulation of nitric oxide biosynthetic process	biological process
739	GO:0090594	inflammatory response to wounding	biological process
740	GO:0045428	regulation of nitric oxide biosynthetic process	biological process
741	GO:0071453	cellular response to oxygen levels	biological process
742	GO:0045404	positive regulation of interleukin-4 production	biological process
743	GO:0045556	positive regulation of TRAIL production	biological process
744	GO:0045636	positive regulation of melanocyte differentiation	biological process
745	hsa04933	AGE-RAGE signaling pathway in diabetic complications	KEGG
746	GO:0045630	positive regulation of T-helper 2 cell differentiation	biological process
747	GO:0045629	negative regulation of T-helper 2 cell differentiation	biological process
748	GO:0032204	regulation of telomere maintenance	biological process
749	GO:0002429	immune response-activating cell surface receptor signaling pathway	biological process
750	GO:0045628	regulation of T-helper 2 cell differentiation	biological process
751	GO:0045624	positive regulation of T-helper cell differentiation	biological process
752	hsa05144	Malaria	KEGG
753	GO:0045623	negative regulation of T-helper cell differentiation	biological process
754	GO:0045622	regulation of T-helper cell differentiation	biological process
755	GO:0045670	regulation of osteoclast differentiation	biological process
756	GO:0043069	negative regulation of programmed cell death	biological process
757	GO:0045656	negative regulation of monocyte differentiation	biological process
758	GO:0045651	positive regulation of macrophage differentiation	biological process
759	GO:0045911	positive regulation of DNA recombination	biological process
760	GO:0045910	negative regulation of DNA recombination	biological process
761	GO:0071310	cellular response to organic substance	biological process
762	GO:0045861	negative regulation of proteolysis	biological process
763	GO:0045852	pH elevation	biological process
764	GO:0046449	creatinine metabolic process	biological process
765	GO:0042089	cytokine production	biological process
766	GO:0000217	DNA secondary structure binding	molecular function
767	GO:0046427	positive regulation of receptor signaling pathway via JAK-STAT	biological process
768	GO:0046386	deoxyribose phosphate catabolic process	biological process
769	GO:0046133	pyrimidine ribonucleoside catabolic process	biological process

770	hsa05142	Chagas disease	KEGG
771	GO:0046087	cytidine metabolic process	biological process
772	GO:0046649	lymphocyte activation	biological process
773	GO:0036293	response to decreased oxygen levels	biological process
774	GO:0046641	positive regulation of alpha-beta T cell proliferation	biological process
775	GO:0046640	regulation of alpha-beta T cell proliferation	biological process
776	GO:0046639	negative regulation of alpha-beta T cell differentiation	biological process
777	GO:0046638	positive regulation of alpha-beta T cell differentiation	biological process
778	GO:0046637	regulation of alpha-beta T cell differentiation	biological process
779	GO:1901700	response to oxygen-containing compound	biological process
780	GO:0046666	retinal cell programmed cell death	biological process
781	GO:0046671	negative regulation of retinal cell programmed cell death	biological process
782	GO:0046718	viral entry into host cell	biological process
783	GO:0046685	response to arsenic-containing substance	biological process
784	GO:0048293	regulation of isotype switching to IgE isotypes	biological process
785	GO:0010556	regulation of macromolecule biosynthetic process	biological process
786	GO:0048291	isotype switching to IgG isotypes	biological process
787	GO:0046209	nitric oxide metabolic process	biological process
788	GO:0048289	isotype switching to IgE isotypes	biological process
789	GO:0048147	negative regulation of fibroblast proliferation	biological process
790	GO:0048388	endosomal lumen acidification	biological process
791	GO:0048534	hematopoietic or lymphoid organ development	biological process
792	GO:0048541	Peyer's patch development	biological process
793	GO:1901698	response to nitrogen compound	biological process
794	GO:0048569	post-embryonic animal organ development	biological process
795	GO:0046794	transport of virus	biological process
796	GO:0042802	identical protein binding	molecular function
797	GO:2001237	negative regulation of extrinsic apoptotic signaling pathway	biological process
798	GO:0050707	regulation of cytokine production	biological process
799	GO:0050678	regulation of epithelial cell proliferation	biological process
800	GO:0000775	chromosome, centromeric region	cellular component
801	GO:0050672	negative regulation of lymphocyte proliferation	biological process
802	GO:1902533	positive regulation of intracellular signal transduction	biological process
803	GO:0050778	positive regulation of immune response	biological process
804	GO:0050776	regulation of immune response	biological process

805	GO:0050731	positive regulation of peptidyl-tyrosine phosphorylation	biological process
806	GO:0050854	regulation of antigen receptor-mediated signaling pathway	biological process
807	GO:0050853	B cell receptor signaling pathway	biological process
808	GO:0007276	gamete generation	biological process
809	GO:0051023	regulation of immunoglobulin production	biological process
810	GO:2000377	regulation of reactive oxygen species metabolic process	biological process
811	GO:0050942	positive regulation of pigment cell differentiation	biological process
812	GO:0050871	positive regulation of B cell activation	biological process
813	GO:0050870	positive regulation of T cell activation	biological process
814	GO:0050869	negative regulation of B cell activation	biological process
815	GO:0051220	cytoplasmic sequestering of protein	biological process
816	GO:0051204	protein insertion into mitochondrial membrane	biological process
817	GO:0051251	positive regulation of lymphocyte activation	biological process
818	GO:0048584	positive regulation of response to stimulus	biological process
819	GO:0051250	negative regulation of lymphocyte activation	biological process
820	GO:0051249	regulation of lymphocyte activation	biological process
821	GO:0051248	negative regulation of protein metabolic process	biological process
822	GO:0051247	positive regulation of protein metabolic process	biological process
823	GO:0051974	negative regulation of telomerase activity	biological process
824	GO:0060548	negative regulation of cell death	biological process
825	GO:0051707	response to other organism	biological process
826	GO:0051704	multi-organism process	biological process
827	GO:0006310	DNA recombination	biological process
828	GO:0051701	biological process involved in interaction with host	biological process
829	GO:0070201	regulation of establishment of protein localization	biological process
830	GO:0051593	response to folic acid	biological process
831	GO:0051573	negative regulation of histone H3-K9 methylation	biological process
832	GO:0061419	positive regulation of transcription from RNA polymerase II promoter in response to	biological process
833	GO:0071496	cellular response to external stimulus	biological process
834	GO:0061377	mammary gland lobule development	biological process
835	GO:0061077	chaperone-mediated protein folding	biological process
836	GO:0061008	hepaticobiliary system development	biological process
837	GO:0060749	mammary gland alveolus development	biological process
838	GO:0070229	negative regulation of lymphocyte apoptotic process	biological process
839	GO:0070228	regulation of lymphocyte apoptotic process	biological process

840	GO:0070242	thymocyte apoptotic process	biological process
841	GO:0070234	positive regulation of T cell apoptotic process	biological process
842	GO:0070232	regulation of T cell apoptotic process	biological process
843	GO:0070555	response to interleukin-1	biological process
844	GO:0048732	gland development	biological process
845	GO:0070541	response to platinum ion	biological process
846	GO:0070512	positive regulation of histone H4-K20 methylation	biological process
847	GO:0070510	regulation of histone H4-K20 methylation	biological process
848	GO:0070669	response to interleukin-2	biological process
849	GO:1903409	reactive oxygen species biosynthetic process	biological process
850	GO:0070666	regulation of mast cell proliferation	biological process
851	GO:0070665	positive regulation of leukocyte proliferation	biological process
852	GO:0032259	methylation	biological process
853	GO:1901214	regulation of neuron death	biological process
854	GO:0070664	negative regulation of leukocyte proliferation	biological process
855	GO:0070663	regulation of leukocyte proliferation	biological process
856	GO:0002367	cytokine production involved in immune response	biological process
857	GO:0071214	cellular response to abiotic stimulus	biological process
858	GO:0070997	neuron death	biological process
859	GO:0004520	endodeoxyribonuclease activity	molecular function
860	GO:0071354	cellular response to interleukin-6	biological process
861	GO:0071353	cellular response to interleukin-4	biological process
862	GO:0071352	cellular response to interleukin-2	biological process
863	GO:0071347	cellular response to interleukin-1	biological process
864	GO:0071234	cellular response to phenylalanine	biological process
865	GO:0071464	cellular response to hydrostatic pressure	biological process
866	GO:0071492	cellular response to UV-A	biological process
867	GO:0071480	cellular response to gamma radiation	biological process
868	GO:0071479	cellular response to ionizing radiation	biological process
869	GO:0071594	thymocyte aggregation	biological process
870	GO:0071593	lymphocyte aggregation	biological process
871	GO:0070482	response to oxygen levels	biological process
872	GO:0071514	genomic imprinting	biological process
873	GO:0071500	cellular response to nitrosative stress	biological process
874	GO:0072529	pyrimidine-containing compound catabolic process	biological process

875	GO:0072527	pyrimidine-containing compound metabolic process	biological process
876	GO:0072434	mitotic G2 DNA damage checkpoint signaling	biological process
877	GO:0072361	regulation of glycolytic process by regulation of transcription from RNA polymerase	biological process
878	GO:1900740	positive regulation of protein insertion into mitochondrial membrane involved in	biological process
879	GO:0072338	cellular lactam metabolic process	biological process
880	GO:0072332	intrinsic apoptotic signaling pathway by p53 class mediator	biological process
881	GO:0072298	regulation of metanephric glomerulus development	biological process
882	GO:0002658	regulation of peripheral tolerance induction	biological process
883	GO:0072223	metanephric glomerular mesangium development	biological process
884	GO:0032847	regulation of cellular pH reduction	biological process
885	GO:0072216	positive regulation of metanephros development	biological process
886	GO:0072186	metanephric cap morphogenesis	biological process
887	GO:0072185	metanephric cap development	biological process
888	GO:0002764	immune response-regulating signaling pathway	biological process
889	GO:0072133	metanephric mesenchyme morphogenesis	biological process
890	GO:0080053	response to phenylalanine	biological process
891	GO:0090200	positive regulation of release of cytochrome c from mitochondria	biological process
892	GO:0090096	positive regulation of metanephric cap mesenchymal cell proliferation	biological process
893	GO:0090095	regulation of metanephric cap mesenchymal cell proliferation	biological process
894	GO:2000104	negative regulation of DNA-templated DNA replication	biological process
895	GO:0090065	regulation of production of siRNA involved in post-transcriptional gene silencing by	biological process
896	GO:0080135	regulation of cellular response to stress	biological process
897	GO:0090094	metanephric cap mesenchymal cell proliferation involved in metanephros	biological process
898	GO:0090403	oxidative stress-induced premature senescence	biological process
899	GO:0090400	stress-induced premature senescence	biological process
900	GO:0090399	replicative senescence	biological process
901	GO:0090398	cellular senescence	biological process
902	GO:0090344	negative regulation of cell aging	biological process
903	GO:0097527	necroptotic signaling pathway	biological process
904	GO:0097341	zymogen inhibition	biological process
905	GO:0097340	inhibition of cysteine-type endopeptidase activity	biological process
906	GO:0035510	DNA dealkylation	biological process
907	GO:0097305	response to alcohol	biological process
908	GO:0097300	programmed necrotic cell death	biological process
909	GO:0097296	activation of cysteine-type endopeptidase activity involved in apoptotic signaling	biological process

910	GO:0097285	cell-type specific apoptotic process	biological process
911	GO:0097284	hepatocyte apoptotic process	biological process
912	GO:0051222	positive regulation of protein transport	biological process
913	GO:1900103	positive regulation of endoplasmic reticulum unfolded protein response	biological process
914	GO:1900101	regulation of endoplasmic reticulum unfolded protein response	biological process
915	GO:1901003	negative regulation of fermentation	biological process
916	GO:1901223	negative regulation of NIK/NF-kappaB signaling	biological process
917	GO:0002768	immune response-regulating cell surface receptor signaling pathway	biological process
918	GO:1901216	positive regulation of neuron death	biological process
919	GO:1901524	regulation of mitophagy	biological process
920	GO:0034641	cellular nitrogen compound metabolic process	biological process
921	GO:1901984	negative regulation of protein acetylation	biological process
922	GO:1901796	regulation of signal transduction by p53 class mediator	biological process
923	GO:0072363	regulation of glycolytic process by positive regulation of transcription from RNA	biological process
924	GO:1902165	regulation of intrinsic apoptotic signaling pathway in response to DNA damage by	biological process
925	GO:1902107	positive regulation of leukocyte differentiation	biological process
926	GO:1902106	negative regulation of leukocyte differentiation	biological process
927	GO:1902105	regulation of leukocyte differentiation	biological process
928	GO:1902041	regulation of extrinsic apoptotic signaling pathway via death domain receptors	biological process
929	GO:1902570	protein localization to nucleolus	biological process
930	GO:0000781	chromosome, telomeric region	cellular component
931	GO:1902913	positive regulation of neuroepithelial cell differentiation	biological process
932	GO:1902751	positive regulation of cell cycle G2/M phase transition	biological process
933	GO:1902749	regulation of cell cycle G2/M phase transition	biological process
934	GO:1902728	positive regulation of growth factor dependent skeletal muscle satellite cell	biological process
935	GO:1902724	positive regulation of skeletal muscle satellite cell proliferation	biological process
936	GO:1902715	positive regulation of interferon-gamma production	biological process
937	GO:0002757	immune response-activating signal transduction	biological process
938	GO:1902689	negative regulation of NAD metabolic process	biological process
939	GO:1902688	regulation of NAD metabolic process	biological process
940	GO:1903071	positive regulation of ER-associated ubiquitin-dependent protein catabolic process	biological process
941	GO:1903069	regulation of ER-associated ubiquitin-dependent protein catabolic process	biological process
942	GO:1903894	regulation of IRE1-mediated unfolded protein response	biological process
943	GO:1903708	positive regulation of hemopoiesis	biological process
944	GO:1903707	negative regulation of hemopoiesis	biological process

945	GO:1903706	regulation of hemopoiesis	biological process
946	GO:1903651	positive regulation of cytoplasmic transport	biological process
947	GO:1904024	negative regulation of glucose catabolic process to lactate via pyruvate	biological process
948	GO:1901701	cellular response to oxygen-containing compound	biological process
949	GO:1904023	regulation of glucose catabolic process to lactate via pyruvate	biological process
950	GO:1904589	regulation of protein import	biological process
951	GO:1904407	positive regulation of nitric oxide metabolic process	biological process
952	GO:2000353	positive regulation of endothelial cell apoptotic process	biological process
953	GO:2000279	negative regulation of DNA biosynthetic process	biological process
954	GO:0044422	organelle part	cellular component
955	GO:2000278	regulation of DNA biosynthetic process	biological process
956	GO:2000209	regulation of anoikis	biological process
957	GO:2000117	negative regulation of cysteine-type endopeptidase activity	biological process
958	GO:2000116	regulation of cysteine-type endopeptidase activity	biological process
959	GO:2000107	negative regulation of leukocyte apoptotic process	biological process
960	GO:0051091	positive regulation of DNA-binding transcription factor activity	biological process
961	GO:1990375	baculum development	biological process
962	GO:1990267	response to transition metal nanoparticle	biological process
963	GO:1990144	intrinsic apoptotic signaling pathway in response to hypoxia	biological process
964	GO:1990001	inhibition of cysteine-type endopeptidase activity involved in apoptotic process	biological process
965	GO:1904951	positive regulation of establishment of protein localization	biological process
966	GO:2000515	negative regulation of CD4-positive, alpha-beta T cell activation	biological process
967	GO:2000514	regulation of CD4-positive, alpha-beta T cell activation	biological process
968	GO:2001057	reactive nitrogen species metabolic process	biological process
969	GO:2001056	positive regulation of cysteine-type endopeptidase activity	biological process
970	GO:2001022	positive regulation of response to DNA damage stimulus	biological process
971	GO:2001021	negative regulation of response to DNA damage stimulus	biological process
972	GO:2001241	positive regulation of extrinsic apoptotic signaling pathway in absence of ligand	biological process
973	GO:2001239	regulation of extrinsic apoptotic signaling pathway in absence of ligand	biological process
974	GO:2001244	positive regulation of intrinsic apoptotic signaling pathway	biological process
975	GO:2001267	regulation of cysteine-type endopeptidase activity involved in apoptotic signaling	biological process
976	GO:2001252	positive regulation of chromosome organization	biological process
977	GO:0005623	cell	cellular component
978	GO:0005721	pericentric heterochromatin	cellular component
979	GO:0005712	chiasma	cellular component

980	GO:0031021	interphase microtubule organizing center	cellular component
981	GO:0019815	B cell receptor complex	cellular component
982	GO:0042101	T cell receptor complex	cellular component
983	GO:0034666	integrin alpha2-beta1 complex	cellular component
984	GO:0034399	nuclear periphery	cellular component
985	GO:0033257	Bcl3/NF-kappaB2 complex	cellular component
986	GO:0006479	protein methylation	biological process
987	GO:0044194	cytolytic granule	cellular component
988	GO:0044427	chromosomal part	cellular component
989	GO:0097136	Bcl-2 family protein complex	cellular component
990	GO:0097057	TRAF2-GSTP1 complex	cellular component
991	GO:0070557	PCNA-p21 complex	cellular component
992	GO:0046930	pore complex	cellular component
993	GO:0045171	intercellular bridge	cellular component
994	GO:0001223	transcription coactivator binding	molecular function
995	GO:1903046	meiotic cell cycle process	biological process
996	GO:0001161	intronic transcription regulatory region sequence-specific DNA binding	molecular function
997	GO:0000400	four-way junction DNA binding	molecular function
998	GO:0006950	response to stress	biological process
999	GO:0003823	antigen binding	molecular function
1000	GO:0003725	double-stranded RNA binding	molecular function
1001	GO:0003721	telomerase RNA reverse transcriptase activity	molecular function
1002	GO:0004861	cyclin-dependent protein serine/threonine kinase inhibitor activity	molecular function
1003	GO:0004844	uracil DNA N-glycosylase activity	molecular function
1004	GO:0004677	DNA-dependent protein kinase activity	molecular function
1005	GO:0004536	deoxyribonuclease activity	molecular function
1006	GO:0005129	granulocyte macrophage colony-stimulating factor receptor binding	molecular function
1007	GO:0008094	ATP-dependent activity, acting on DNA	molecular function
1008	GO:0016799	hydrolase activity, hydrolyzing N-glycosyl compounds	molecular function
1009	GO:0016769	transferase activity, transferring nitrogenous groups	molecular function
1010	GO:0016538	cyclin-dependent protein serine/threonine kinase regulator activity	molecular function
1011	GO:0015616	DNA translocase activity	molecular function
1012	GO:0035033	histone deacetylase regulator activity	molecular function
1013	GO:0019912	cyclin-dependent protein kinase activating kinase activity	molecular function
1014	GO:0034618	arginine binding	molecular function

1015	GO:0032813	tumor necrosis factor receptor superfamily binding	molecular function
1016	GO:0032407	MutSalpha complex binding	molecular function
1017	GO:0032405	MutLalpha complex binding	molecular function
1018	GO:0032404	mismatch repair complex binding	molecular function
1019	GO:0032135	DNA insertion or deletion binding	molecular function
1020	GO:0031851	kappa-type opioid receptor binding	molecular function
1021	GO:0003697	single-stranded DNA binding	molecular function
1022	GO:0031798	type 1 metabotropic glutamate receptor binding	molecular function
1023	GO:0035758	chemokine (C-C motif) ligand 21 binding	molecular function
1024	GO:0035757	chemokine (C-C motif) ligand 19 binding	molecular function
1025	GO:0035731	dinitrosyl-iron complex binding	molecular function
1026	GO:0035730	S-nitrosoglutathione binding	molecular function
1027	GO:0038121	C-C motif chemokine 21 receptor activity	molecular function
1028	GO:0043028	cysteine-type endopeptidase regulator activity involved in apoptotic process	molecular function
1029	GO:0043027	cysteine-type endopeptidase inhibitor activity involved in apoptotic process	molecular function
1030	GO:0042826	histone deacetylase binding	molecular function
1031	GO:0042803	protein homodimerization activity	molecular function
1032	GO:0050135	NAD(P)+ nucleosidase activity	molecular function
1033	GO:0070087	chromo shadow domain binding	molecular function
1034	GO:0051434	BH3 domain binding	molecular function
1035	GO:0044237	cellular metabolic process	biological process
1036	GO:0051569	regulation of histone H3-K4 methylation	biological process
1037	GO:0046135	pyrimidine nucleoside catabolic process	biological process
1038	GO:0005657	replication fork	cellular component
1039	GO:0051276	chromosome organization	biological process
1040	GO:0043314	negative regulation of neutrophil degranulation	biological process
1041	GO:0048087	positive regulation of developmental pigmentation	biological process
1042	GO:1901658	glycosyl compound catabolic process	biological process
1043	GO:0006312	mitotic recombination	biological process
1044	GO:1903431	positive regulation of cell maturation	biological process
1045	GO:0006807	nitrogen compound metabolic process	biological process
1046	GO:1904882	regulation of telomerase catalytic core complex assembly	biological process
1047	GO:0018022	peptidyl-lysine methylation	biological process
1048	GO:1901724	positive regulation of cell proliferation involved in kidney development	biological process
1049	GO:0060700	regulation of ribonuclease activity	biological process

1050	GO:0043504	mitochondrial DNA repair	biological process
1051	GO:0044766	multi-organism transport	biological process
1052	GO:0003674	molecular function	molecular function
1053	GO:0097252	oligodendrocyte apoptotic process	biological process
1054	GO:0002266	follicular dendritic cell activation	biological process
1055	GO:0098687	chromosomal region	cellular component
1056	GO:0000083	regulation of transcription involved in G1/S transition of mitotic cell cycle	biological process
1057	GO:0010035	response to inorganic substance	biological process
1058	GO:0032133	chromosome passenger complex	cellular component
1059	GO:0003676	nucleic acid binding	molecular function
1060	GO:0000784	chromosome, telomeric region	cellular component
1061	GO:0075733	intracellular transport of virus	biological process
1062	GO:0015291	secondary active transmembrane transporter activity	molecular function
1063	GO:0051095	regulation of helicase activity	biological process
1064	GO:0071494	cellular response to UV-C	biological process
1065	GO:0016903	oxidoreductase activity, acting on the aldehyde or oxo group of donors	molecular function
1066	GO:1902579	multi-organism localization	biological process
1067	GO:0003677	DNA binding	molecular function
1068	GO:0002268	follicular dendritic cell differentiation	biological process
1069	GO:0048478	replication fork protection	biological process
1070	GO:0003678	DNA helicase activity	molecular function
1071	GO:0005730	nucleolus	cellular component
1072	GO:0051321	meiotic cell cycle	biological process
1073	GO:0000794	condensed nuclear chromosome	cellular component
1074	GO:0045005	DNA-templated DNA replication maintenance of fidelity	biological process
1075	GO:0007131	reciprocal meiotic recombination	biological process
