# Supplementary material for: Analysis of Lymphoma-Related Genes with Gene Ontology and Kyoto Encyclopedia of Genes and Genomes Enrichment
Source: Biomed Res Int. 2022 Jun 26;2022:8503511. doi: 10.1155/2022/8503511 (PMC9251090; doi:10.1155/2022/8503511)
Supplement: Supplementary Materials — Table S1: feature list obtained using the Boruta and max-relevance and min-redundancy (mRMR) feature selection methods. Table S2: performance of the decision tree model on the different number of features. Table S3: classification rules obtained by the optimal decision tree model. [file 8503511.f1.zip › Table S2 (1).pdf]

Number of features	SN	SP	ACC	MCC	Precision	F1-measure
5	0.738	0.879	0.868	0.436	0.332	0.458
10	0.705	0.898	0.883	0.448	0.360	0.476
15	0.664	0.892	0.875	0.412	0.334	0.445
20	0.680	0.899	0.882	0.434	0.353	0.465
25	0.679	0.898	0.881	0.432	0.351	0.463
30	0.659	0.896	0.878	0.415	0.341	0.449
35	0.669	0.897	0.880	0.424	0.346	0.456
40	0.663	0.896	0.879	0.418	0.342	0.451
45	0.677	0.899	0.882	0.433	0.353	0.464
50	0.656	0.900	0.882	0.421	0.348	0.455
55	0.671	0.900	0.883	0.430	0.353	0.462
60	0.656	0.902	0.883	0.424	0.352	0.458
65	0.683	0.897	0.881	0.433	0.351	0.464
70	0.653	0.903	0.884	0.425	0.354	0.460
75	0.667	0.899	0.882	0.426	0.350	0.459
80	0.657	0.896	0.878	0.414	0.340	0.449
85	0.658	0.897	0.879	0.416	0.342	0.450
90	0.665	0.896	0.879	0.419	0.342	0.452
95	0.662	0.897	0.880	0.420	0.345	0.453
100	0.674	0.897	0.880	0.427	0.347	0.459
105	0.658	0.897	0.879	0.417	0.343	0.451
110	0.653	0.904	0.885	0.426	0.356	0.460
115	0.665	0.902	0.884	0.430	0.355	0.463
120	0.652	0.896	0.878	0.410	0.338	0.445
125	0.681	0.901	0.885	0.439	0.359	0.471
130	0.678	0.901	0.884	0.437	0.358	0.469
135	0.680	0.898	0.882	0.433	0.352	0.464
140	0.662	0.900	0.882	0.425	0.351	0.459
145	0.650	0.898	0.879	0.412	0.341	0.447

150	0.669	0.897	0.880	0.424	0.346	0.457
155	0.663	0.894	0.877	0.414	0.338	0.448
160	0.655	0.899	0.880	0.417	0.345	0.452
165	0.666	0.899	0.881	0.425	0.349	0.458
170	0.654	0.897	0.879	0.413	0.340	0.448
175	0.648	0.898	0.879	0.411	0.340	0.446
180	0.631	0.900	0.880	0.403	0.339	0.441
185	0.662	0.903	0.885	0.431	0.358	0.465
190	0.661	0.900	0.882	0.424	0.350	0.458
195	0.667	0.900	0.883	0.428	0.353	0.461
200	0.673	0.901	0.884	0.434	0.357	0.467
205	0.653	0.899	0.880	0.416	0.344	0.451
210	0.635	0.905	0.885	0.416	0.353	0.453
215	0.652	0.901	0.882	0.420	0.349	0.455
220	0.677	0.898	0.881	0.430	0.350	0.461
225	0.646	0.900	0.881	0.414	0.345	0.450
230	0.674	0.902	0.885	0.436	0.359	0.468
235	0.650	0.901	0.882	0.419	0.349	0.454
240	0.653	0.900	0.881	0.418	0.347	0.453
245	0.670	0.900	0.883	0.430	0.353	0.462
250	0.644	0.906	0.886	0.423	0.357	0.460
255	0.653	0.903	0.884	0.424	0.354	0.459
260	0.625	0.903	0.882	0.405	0.344	0.443
265	0.652	0.903	0.884	0.424	0.354	0.459
270	0.635	0.898	0.878	0.403	0.337	0.440
275	0.654	0.899	0.880	0.417	0.344	0.451
280	0.654	0.902	0.883	0.423	0.352	0.457
285	0.685	0.900	0.884	0.440	0.359	0.471
290	0.654	0.901	0.882	0.421	0.350	0.456
295	0.664	0.902	0.884	0.430	0.356	0.463

300	0.653	0.903	0.884	0.424	0.353	0.459
305	0.674	0.903	0.886	0.439	0.362	0.471
310	0.662	0.901	0.883	0.427	0.353	0.461
315	0.656	0.904	0.885	0.428	0.358	0.463
320	0.668	0.903	0.885	0.434	0.359	0.467
325	0.653	0.904	0.885	0.426	0.357	0.461
330	0.676	0.899	0.882	0.432	0.353	0.464
335	0.640	0.899	0.880	0.408	0.340	0.444
340	0.665	0.900	0.883	0.427	0.352	0.460
345	0.663	0.902	0.884	0.429	0.355	0.463
350	0.637	0.900	0.880	0.408	0.342	0.445
355	0.685	0.902	0.886	0.444	0.364	0.475
360	0.666	0.901	0.883	0.428	0.353	0.461
365	0.665	0.902	0.884	0.431	0.356	0.464
370	0.664	0.900	0.882	0.425	0.350	0.458
375	0.684	0.898	0.882	0.436	0.354	0.466
380	0.647	0.899	0.880	0.414	0.344	0.449
385	0.652	0.900	0.881	0.417	0.346	0.452
390	0.651	0.901	0.882	0.418	0.348	0.454
395	0.677	0.903	0.886	0.440	0.362	0.472
400	0.651	0.906	0.886	0.428	0.360	0.463
405	0.668	0.900	0.882	0.427	0.351	0.460
410	0.653	0.893	0.875	0.406	0.333	0.441
415	0.652	0.902	0.884	0.422	0.352	0.457
420	0.646	0.897	0.878	0.408	0.338	0.444
425	0.651	0.898	0.880	0.414	0.342	0.449
430	0.668	0.901	0.883	0.430	0.354	0.463
435	0.677	0.899	0.882	0.432	0.353	0.464
440	0.658	0.903	0.885	0.428	0.356	0.462
445	0.674	0.900	0.883	0.432	0.354	0.464

450	0.672	0.898	0.881	0.428	0.350	0.460
455	0.658	0.902	0.883	0.425	0.353	0.459
460	0.663	0.900	0.883	0.426	0.352	0.459
465	0.663	0.900	0.882	0.425	0.350	0.459
470	0.669	0.901	0.884	0.432	0.356	0.465
475	0.662	0.901	0.883	0.426	0.353	0.460
480	0.676	0.904	0.887	0.441	0.364	0.473
485	0.679	0.902	0.885	0.440	0.361	0.471
490	0.639	0.899	0.879	0.407	0.339	0.443
495	0.674	0.900	0.883	0.433	0.354	0.465
500	0.655	0.898	0.880	0.417	0.344	0.451
505	0.663	0.898	0.881	0.422	0.347	0.455
510	0.638	0.899	0.879	0.406	0.339	0.442
515	0.640	0.901	0.882	0.412	0.346	0.449
520	0.661	0.904	0.886	0.432	0.360	0.466
525	0.682	0.903	0.886	0.443	0.363	0.474
530	0.650	0.901	0.882	0.418	0.348	0.453
535	0.656	0.900	0.881	0.420	0.347	0.454
540	0.656	0.902	0.883	0.424	0.352	0.458
545	0.666	0.902	0.884	0.430	0.355	0.463
550	0.672	0.901	0.883	0.432	0.355	0.465
555	0.659	0.901	0.882	0.423	0.350	0.457
560	0.658	0.901	0.883	0.424	0.352	0.459
565	0.637	0.896	0.877	0.401	0.333	0.437
570	0.665	0.898	0.881	0.423	0.347	0.456
575	0.665	0.907	0.889	0.440	0.368	0.474
580	0.671	0.902	0.885	0.434	0.358	0.467
585	0.662	0.897	0.879	0.418	0.343	0.452
590	0.638	0.900	0.881	0.409	0.343	0.446
595	0.678	0.900	0.883	0.435	0.356	0.467

600	0.653	0.900	0.882	0.419	0.348	0.454
605	0.665	0.907	0.889	0.441	0.369	0.474
610	0.662	0.902	0.884	0.428	0.355	0.462
615	0.676	0.903	0.886	0.439	0.361	0.471
620	0.668	0.903	0.885	0.434	0.358	0.467
625	0.683	0.903	0.886	0.444	0.364	0.475
630	0.680	0.905	0.888	0.446	0.367	0.477
635	0.676	0.904	0.887	0.442	0.365	0.474
640	0.669	0.897	0.880	0.423	0.346	0.456
645	0.674	0.902	0.885	0.436	0.359	0.469
650	0.653	0.901	0.882	0.421	0.349	0.455
655	0.667	0.901	0.884	0.430	0.355	0.463
660	0.671	0.900	0.883	0.431	0.354	0.464
665	0.665	0.899	0.881	0.424	0.348	0.457
670	0.669	0.901	0.884	0.431	0.355	0.464
675	0.669	0.905	0.887	0.439	0.364	0.472
680	0.650	0.904	0.885	0.424	0.355	0.459
685	0.656	0.902	0.883	0.423	0.352	0.458
690	0.663	0.902	0.884	0.430	0.356	0.464
695	0.662	0.901	0.883	0.427	0.353	0.460
700	0.653	0.903	0.884	0.425	0.355	0.460
705	0.653	0.901	0.882	0.420	0.349	0.455
710	0.669	0.905	0.887	0.439	0.365	0.472
715	0.654	0.899	0.881	0.418	0.346	0.453
720	0.660	0.902	0.884	0.428	0.355	0.462
725	0.665	0.897	0.880	0.422	0.346	0.455
730	0.643	0.906	0.886	0.423	0.358	0.460
735	0.670	0.901	0.883	0.431	0.355	0.464
740	0.668	0.904	0.887	0.437	0.362	0.470
745	0.662	0.902	0.884	0.428	0.355	0.462

750	0.675	0.899	0.882	0.432	0.353	0.463
755	0.663	0.901	0.883	0.428	0.354	0.461
760	0.647	0.904	0.884	0.421	0.354	0.457
765	0.662	0.897	0.879	0.419	0.343	0.452
770	0.690	0.903	0.887	0.449	0.367	0.479
775	0.676	0.902	0.885	0.438	0.360	0.470
780	0.687	0.896	0.881	0.434	0.351	0.464
785	0.674	0.899	0.882	0.431	0.352	0.463
790	0.680	0.904	0.887	0.444	0.365	0.475
795	0.666	0.901	0.884	0.430	0.354	0.463
800	0.659	0.901	0.883	0.425	0.352	0.459
805	0.683	0.908	0.891	0.455	0.378	0.486
810	0.676	0.906	0.889	0.446	0.370	0.479
815	0.668	0.900	0.882	0.428	0.351	0.460
820	0.682	0.901	0.885	0.440	0.359	0.471
825	0.679	0.905	0.888	0.446	0.368	0.478
830	0.683	0.902	0.886	0.443	0.363	0.474
835	0.664	0.903	0.885	0.431	0.357	0.465
840	0.665	0.901	0.883	0.429	0.354	0.462
845	0.683	0.903	0.887	0.445	0.365	0.476
850	0.650	0.902	0.883	0.420	0.350	0.456
855	0.680	0.906	0.889	0.449	0.371	0.480
860	0.678	0.901	0.884	0.437	0.359	0.469
865	0.664	0.901	0.883	0.427	0.353	0.461
870	0.654	0.905	0.886	0.429	0.360	0.464
875	0.658	0.901	0.882	0.423	0.350	0.457
880	0.672	0.904	0.887	0.439	0.363	0.471
885	0.683	0.903	0.887	0.444	0.365	0.475
890	0.670	0.900	0.882	0.429	0.352	0.462
895	0.659	0.902	0.884	0.426	0.354	0.460

900	0.668	0.900	0.883	0.429	0.353	0.462
905	0.671	0.903	0.886	0.437	0.361	0.470
910	0.671	0.905	0.887	0.440	0.365	0.473
915	0.651	0.901	0.882	0.420	0.349	0.455
920	0.673	0.901	0.884	0.434	0.357	0.466
925	0.678	0.905	0.888	0.444	0.367	0.476
930	0.678	0.901	0.885	0.438	0.359	0.469
935	0.662	0.903	0.885	0.431	0.358	0.465
940	0.669	0.906	0.888	0.440	0.366	0.473
945	0.660	0.904	0.885	0.430	0.358	0.464
950	0.656	0.904	0.885	0.428	0.357	0.463
955	0.665	0.903	0.885	0.431	0.357	0.465
960	0.683	0.903	0.887	0.445	0.365	0.476
965	0.670	0.902	0.885	0.434	0.359	0.467
970	0.677	0.903	0.886	0.441	0.364	0.473
975	0.672	0.900	0.883	0.431	0.354	0.464
980	0.665	0.905	0.887	0.435	0.362	0.469
985	0.669	0.903	0.885	0.434	0.359	0.467
990	0.691	0.903	0.887	0.450	0.368	0.480
995	0.668	0.907	0.889	0.442	0.368	0.475
1000	0.683	0.904	0.887	0.445	0.366	0.477
1005	0.665	0.903	0.885	0.432	0.358	0.465
1010	0.682	0.901	0.885	0.440	0.360	0.472
1015	0.668	0.905	0.887	0.437	0.363	0.470
1020	0.660	0.904	0.886	0.431	0.359	0.465
1025	0.670	0.905	0.888	0.440	0.366	0.473
1030	0.641	0.906	0.886	0.423	0.358	0.459
1035	0.669	0.900	0.883	0.430	0.353	0.462
1040	0.650	0.902	0.883	0.419	0.350	0.455
1045	0.638	0.904	0.884	0.416	0.351	0.453

1050	0.653	0.903	0.884	0.424	0.353	0.459
1055	0.641	0.904	0.884	0.417	0.351	0.454
1060	0.650	0.907	0.888	0.430	0.363	0.466
1065	0.634	0.903	0.883	0.412	0.348	0.449
1070	0.651	0.904	0.885	0.424	0.355	0.459
1075	0.652	0.900	0.882	0.418	0.347	0.453
