# Supplementary material for: Analysis of Lymphoma-Related Genes with Gene Ontology and Kyoto Encyclopedia of Genes and Genomes Enrichment
Source: Biomed Res Int. 2022 Jun 26;2022:8503511. doi: 10.1155/2022/8503511 (PMC9251090; doi:10.1155/2022/8503511)
Supplement: Supplementary Materials — Table S1: feature list obtained using the Boruta and max-relevance and min-redundancy (mRMR) feature selection methods. Table S2: performance of the decision tree model on the different number of features. Table S3: classification rules obtained by the optimal decision tree model. [file 8503511.f1.zip › Table S3 (1).pdf]

Rules_0	passed counts:4555
node_0: feature_name=GO:0042113	feature_id[0].value <= threshold=13.408552169799805
node_1: feature_name=GO:0007568	feature_id[534].value <= threshold=5.0313897132873535
node_2: feature_name=GO:0002705	feature_id[541].value <= threshold=3.200145721435547
node_3: feature_name=GO:1901525	feature_id[576].value <= threshold=0.4466460347175598
node_4: feature_name=GO:0048539	feature_id[319].value <= threshold=3.0399646759033203
node_5: feature_name=GO:0001910	feature_id[385].value <= threshold=3.7437864542007446
node_6: feature_name=GO:0043200	feature_id[706].value <= threshold=9.307284355163574
node_7: feature_name=GO:0001773	feature_id[308].value <= threshold=4.016931533813477
node_8: feature_name=GO:0090116	feature_id[97].value <= threshold=7.99645471572876
node_9: feature_name=GO:0019814	feature_id[189].value <= threshold=4.525782108306885
node_10: feature_name=GO:1902583	feature_id[215].value <= threshold=17.60310649871826
node_11: feature_name=GO:0045429	feature_id[737].value <= threshold=1.6133361458778381
node_12: feature_name=GO:0003720	feature_id[228].value <= threshold=5.519326210021973
node_13: feature_name=GO:0046006	feature_id[364].value <= threshold=4.9985432624816895
node_14: feature_name=GO:0070424	feature_id[254].value <= threshold=6.233297109603882
node_15: feature_name=GO:0009892	feature_id[320].value <= threshold=59.99180793762207
node_16: feature_name=GO:0007064	feature_id[527].value <= threshold=11.57377815246582
node_17: feature_name=GO:0005575	feature_id[17].value <= threshold=15.417460918426514
node_18: feature_name=GO:0043368	feature_id[31].value <= threshold=4.059496641159058
node_19: feature_name=GO:0005164	feature_id[163].value <= threshold=5.793607711791992
node_20: feature_name=GO:0042130	feature_id[16].value <= threshold=5.073179721832275
node_21: feature_name=GO:0010216	feature_id[282].value <= threshold=4.834584474563599
node_22: feature_name=GO:0009628	feature_id[553].value <= threshold=29.314892768859863
node_23: feature_name=GO:0045628	feature_id[749].value <= threshold=3.1001367568969727
node_24: feature_name=GO:0042288	feature_id[100].value <= threshold=5.174932956695557
node_25: feature_name=GO:0002329	feature_id[406].value <= threshold=2.348930835723877
node_26: feature_name=GO:0051246	feature_id[642].value <= threshold=0.6498909294605255
node_27: feature_name=GO:0042162	feature_id[355].value <= threshold=8.05103588104248
node_28: feature_name=hsa04668	feature_id[333].value <= threshold=5.614926338195801
node_29: feature_name=GO:0032200	feature_id[613].value <= threshold=28.735913276672363
node_30: feature_name=hsa04662	feature_id[334].value <= threshold=9.588344097137451
node_31: feature_name=GO:1901992	feature_id[105].value <= threshold=5.291427850723267
node_32: feature_name=GO:0072341	feature_id[144].value <= threshold=6.01465106010437
node_33: feature_name=GO:0047485	feature_id[121].value <= threshold=6.657836437225342

node_34: feature_name=GO:0090594 feature_id[738].value <= threshold=1.8673912286758423
node_35: feature_name=GO:0043525 feature_id[524].value <= threshold=2.881397008895874
node_36: feature_name=GO:0032504 feature_id[244].value <= threshold=17.22207546234131
node_37: feature_name=GO:0051348 feature_id[126].value <= threshold=5.933559417724609
node_38: feature_name=GO:0042522 feature_id[692].value <= threshold=1.78694087266922
node_39: feature_name=GO:0031100 feature_id[73].value <= threshold=3.2312101125717163
node_40: feature_name=GO:0042493 feature_id[149].value <= threshold=19.771170616149902
node_41: feature_name=GO:0001552 feature_id[366].value <= threshold=2.1711617708206177
node_42: feature_name=GO:0046483 feature_id[391].value <= threshold=320.9667053222656
node_43: feature_name=GO:0002439 feature_id[431].value <= threshold=1.832722783088684
node_44: feature_name=GO:0006555 feature_id[514].value <= threshold=5.321640968322754
node_45: feature_name=GO:0070424 feature_id[254].value <= threshold=3.321003556251526
node_46: feature_name=hsa05144 feature_id[751].value <= threshold=2.635499954223633
node_47: feature_name=GO:0032633 feature_id[627].value <= threshold=1.2592533230781555
node_48: feature_name=GO:0019814 feature_id[189].value <= threshold=2.1583125591278076
node_49: feature_name=GO:1901989 feature_id[30].value <= threshold=3.3150794506073
node_50: feature_name=GO:1903318 feature_id[196].value <= threshold=1.62252539396286
node_51: feature_name=GO:0060576 feature_id[204].value <= threshold=2.1097792387008667
node_52: feature_name=hsa05221 feature_id[349].value <= threshold=0.8723124265670776
node_53: feature_name=GO:1903896 feature_id[135].value <= threshold=1.397229254245758
node_54: feature_name=GO:0097028 feature_id[443].value <= threshold=1.2365349531173706
node_55: feature_name=GO:0048660 feature_id[403].value <= threshold=1.9425984621047974
node_56: feature_name=GO:0043011 feature_id[150].value <= threshold=1.3008450269699097
node_57: feature_name=GO:0023056 feature_id[632].value <= threshold=2.5618948936462402
node_58: feature_name=GO:0005164 feature_id[163].value <= threshold=0.9787287414073944
node_59: feature_name=GO:0050707 feature_id[797].value <= threshold=0.9616408348083496
node_60: feature_name=hsa05202 feature_id[50].value <= threshold=1.5331347584724426
node_61: feature_name=GO:0002718 feature_id[481].value <= threshold=0.6544264256954193
Class: negative genes

Rules_1 passed counts:1665
node_0: feature_name=GO:0042113 feature_id[0].value <= threshold=13.408552169799805
node_1: feature_name=GO:0007568 feature_id[534].value <= threshold=5.0313897132873535
node_2: feature_name=GO:0002705 feature_id[541].value <= threshold=3.200145721435547
node_3: feature_name=GO:1901525 feature_id[576].value <= threshold=0.4466460347175598

node_4: feature_name=GO:0048539	feature_id[319].value <= threshold=3.0399646759033203
node_5: feature_name=GO:0001910	feature_id[385].value <= threshold=3.7437864542007446
node_6: feature_name=GO:0043200	feature_id[706].value <= threshold=9.307284355163574
node_7: feature_name=GO:0001773	feature_id[308].value <= threshold=4.016931533813477
node_8: feature_name=GO:0090116	feature_id[97].value <= threshold=7.99645471572876
node_9: feature_name=GO:0019814	feature_id[189].value <= threshold=4.525782108306885
node_10: feature_name=GO:1902583	feature_id[215].value <= threshold=17.60310649871826
node_11: feature_name=GO:0045429	feature_id[737].value <= threshold=1.6133361458778381
node_12: feature_name=GO:0003720	feature_id[228].value <= threshold=5.519326210021973
node_13: feature_name=GO:0046006	feature_id[364].value <= threshold=4.9985432624816895
node_14: feature_name=GO:0070424	feature_id[254].value <= threshold=6.233297109603882
node_15: feature_name=GO:0009892	feature_id[320].value <= threshold=59.99180793762207
node_16: feature_name=GO:0007064	feature_id[527].value <= threshold=11.57377815246582
node_17: feature_name=GO:0005575	feature_id[17].value <= threshold=15.417460918426514
node_18: feature_name=GO:0043368	feature_id[31].value <= threshold=4.059496641159058
node_19: feature_name=GO:0005164	feature_id[163].value <= threshold=5.793607711791992
node_20: feature_name=GO:0042130	feature_id[16].value <= threshold=5.073179721832275
node_21: feature_name=GO:0010216	feature_id[282].value <= threshold=4.834584474563599
node_22: feature_name=GO:0009628	feature_id[553].value <= threshold=29.314892768859863
node_23: feature_name=GO:0045628	feature_id[749].value <= threshold=3.1001367568969727
node_24: feature_name=GO:0042288	feature_id[100].value <= threshold=5.174932956695557
node_25: feature_name=GO:0002329	feature_id[406].value <= threshold=2.348930835723877
node_26: feature_name=GO:0051246	feature_id[642].value > threshold=0.6498909294605255
node_194: feature_name=GO:0009164	feature_id[546].value <= threshold=17.11970329284668
node_195: feature_name=GO:0001909	feature_id[386].value <= threshold=7.292566299438477
node_196: feature_name=GO:0003908	feature_id[11].value <= threshold=1.887226164340973
node_197: feature_name=GO:0009164	feature_id[546].value <= threshold=0.26642198860645294
node_198: feature_name=GO:2001242	feature_id[24].value <= threshold=9.207026481628418
node_199: feature_name=GO:0051246	feature_id[642].value > threshold=0.6504445374011993
node_203: feature_name=GO:0032461	feature_id[608].value <= threshold=2.607542634010315
node_204: feature_name=GO:0007584	feature_id[529].value <= threshold=16.099515914916992
node_205: feature_name=hsa05221	feature_id[349].value <= threshold=9.359850406646729
node_206: feature_name=GO:0002636	feature_id[447].value <= threshold=1.615447759628296
node_207: feature_name=GO:0033993	feature_id[421].value > threshold=2.96540611088858e-05
node_217: feature_name=GO:0002331	feature_id[404].value <= threshold=2.1883513927459717

node_218: feature_name=GO:0046498 feature_id[203].value <= threshold=4.085246205329895
node_219: feature_name=GO:0002832 feature_id[491].value <= threshold=3.0228612422943115
node_220: feature_name=GO:1904868 feature_id[306].value <= threshold=3.8674756288528442
node_221: feature_name=GO:0048545 feature_id[360].value <= threshold=27.38136100769043
node_222: feature_name=GO:0071310 feature_id[760].value > threshold=2.3522024154663086
node_306: feature_name=GO:0070233 feature_id[321].value <= threshold=1.293377935886383
node_307: feature_name=hsa05221 feature_id[349].value <= threshold=9.005411148071289
node_308: feature_name=GO:0032770 feature_id[604].value <= threshold=3.2187339067459106
Class: negative genes

Rules_2 passed counts:1501
node_0: feature_name=GO:0042113 feature_id[0].value <= threshold=13.408552169799805
node_1: feature_name=GO:0007568 feature_id[534].value <= threshold=5.0313897132873535
node_2: feature_name=GO:0002705 feature_id[541].value <= threshold=3.200145721435547
node_3: feature_name=GO:1901525 feature_id[576].value <= threshold=0.4466460347175598
node_4: feature_name=GO:0048539 feature_id[319].value <= threshold=3.0399646759033203
node_5: feature_name=GO:0001910 feature_id[385].value <= threshold=3.7437864542007446
node_6: feature_name=GO:0043200 feature_id[706].value <= threshold=9.307284355163574
node_7: feature_name=GO:0001773 feature_id[308].value <= threshold=4.016931533813477
node_8: feature_name=GO:0090116 feature_id[97].value <= threshold=7.99645471572876
node_9: feature_name=GO:0019814 feature_id[189].value <= threshold=4.525782108306885
node_10: feature_name=GO:1902583 feature_id[215].value <= threshold=17.60310649871826
node_11: feature_name=GO:0045429 feature_id[737].value <= threshold=1.6133361458778381
node_12: feature_name=GO:0003720 feature_id[228].value <= threshold=5.519326210021973
node_13: feature_name=GO:0046006 feature_id[364].value <= threshold=4.9985432624816895
node_14: feature_name=GO:0070424 feature_id[254].value <= threshold=6.233297109603882
node_15: feature_name=GO:0009892 feature_id[320].value <= threshold=59.99180793762207
node_16: feature_name=GO:0007064 feature_id[527].value <= threshold=11.57377815246582
node_17: feature_name=GO:0005575 feature_id[17].value <= threshold=15.417460918426514
node_18: feature_name=GO:0043368 feature_id[31].value <= threshold=4.059496641159058
node_19: feature_name=GO:0005164 feature_id[163].value <= threshold=5.793607711791992
node_20: feature_name=GO:0042130 feature_id[16].value <= threshold=5.073179721832275
node_21: feature_name=GO:0010216 feature_id[282].value <= threshold=4.834584474563599
node_22: feature_name=GO:0009628 feature_id[553].value <= threshold=29.314892768859863
node_23: feature_name=GO:0045628 feature_id[749].value <= threshold=3.1001367568969727

node_24: feature_name=GO:0042288	feature_id[100].value <= threshold=5.174932956695557
node_25: feature_name=GO:0002329	feature_id[406].value <= threshold=2.348930835723877
node_26: feature_name=GO:0051246	feature_id[642].value > threshold=0.6498909294605255
node_194: feature_name=GO:0009164	feature_id[546].value <= threshold=17.11970329284668
node_195: feature_name=GO:0001909	feature_id[386].value <= threshold=7.292566299438477
node_196: feature_name=GO:0003908	feature_id[11].value <= threshold=1.887226164340973
node_197: feature_name=GO:0009164	feature_id[546].value <= threshold=0.26642198860645294
node_198: feature_name=GO:2001242	feature_id[24].value <= threshold=9.207026481628418
node_199: feature_name=GO:0051246	feature_id[642].value > threshold=0.6504445374011993
node_203: feature_name=GO:0032461	feature_id[608].value <= threshold=2.607542634010315
node_204: feature_name=GO:0007584	feature_id[529].value <= threshold=16.099515914916992
node_205: feature_name=hsa05221	feature_id[349].value <= threshold=9.359850406646729
node_206: feature_name=GO:0002636	feature_id[447].value <= threshold=1.615447759628296
node_207: feature_name=GO:0033993	feature_id[421].value > threshold=2.96540611088858e-05
node_217: feature_name=GO:0002331	feature_id[404].value <= threshold=2.1883513927459717
node_218: feature_name=GO:0046498	feature_id[203].value <= threshold=4.085246205329895
node_219: feature_name=GO:0002832	feature_id[491].value <= threshold=3.0228612422943115
node_220: feature_name=GO:1904868	feature_id[306].value <= threshold=3.8674756288528442
node_221: feature_name=GO:0048545	feature_id[360].value <= threshold=27.38136100769043
node_222: feature_name=GO:0071310	feature_id[760].value <= threshold=2.3522024154663086
node_223: feature_name=GO:0001775	feature_id[505].value <= threshold=9.71301555633545
node_224: feature_name=GO:0071310	feature_id[760].value <= threshold=2.346743583679199
node_225: feature_name=GO:0001772	feature_id[91].value <= threshold=3.252573609352112
node_226: feature_name=GO:0036498	feature_id[359].value <= threshold=12.092026710510254
node_227: feature_name=hsa05340	feature_id[351].value <= threshold=0.7677814364433289
node_228: feature_name=GO:0032703	feature_id[617].value <= threshold=1.7450646758079529
node_229: feature_name=hsa04640	feature_id[79].value <= threshold=2.118402123451233
node_230: feature_name=GO:0042097	feature_id[675].value <= threshold=1.8110727667808533
node_231: feature_name=GO:0071391	feature_id[518].value <= threshold=2.691588521003723
node_232: feature_name=GO:0001666	feature_id[682].value <= threshold=5.225740432739258
node_233: feature_name=GO:0048539	feature_id[319].value <= threshold=1.8523842096328735
node_234: feature_name=GO:0038061	feature_id[671].value <= threshold=8.564527988433838
node_235: feature_name=GO:0002484	feature_id[600].value <= threshold=3.1121134757995605
node_236: feature_name=GO:0023023	feature_id[119].value <= threshold=2.868617057800293
node_237: feature_name=GO:0010639	feature_id[564].value <= threshold=13.410505771636963

node_238: feature_name=GO:0080134 feature_id[650].value > threshold=0.013442101422697306
node_244: feature_name=hsa04060 feature_id[331].value <= threshold=0.7175639569759369
Class: negative genes

Rules_3 passed counts:1145
node_0: feature_name=GO:0042113 feature_id[0].value <= threshold=13.408552169799805
node_1: feature_name=GO:0007568 feature_id[534].value <= threshold=5.0313897132873535
node_2: feature_name=GO:0002705 feature_id[541].value <= threshold=3.200145721435547
node_3: feature_name=GO:1901525 feature_id[576].value > threshold=0.4466460347175598
node_617: feature_name=GO:0005622 feature_id[233].value <= threshold=90.85730743408203
node_618: feature_name=GO:1903147 feature_id[200].value <= threshold=7.326428413391113
node_619: feature_name=GO:0003964 feature_id[317].value <= threshold=5.575642108917236
node_620: feature_name=GO:0046498 feature_id[203].value <= threshold=6.620223522186279
node_621: feature_name=GO:0002562 feature_id[25].value <= threshold=10.287704467773438
node_622: feature_name=GO:0006919 feature_id[246].value <= threshold=5.801334381103516
node_623: feature_name=GO:0001889 feature_id[387].value <= threshold=2.679414987564087
node_624: feature_name=GO:0006555 feature_id[514].value <= threshold=7.819535493850708
node_625: feature_name=GO:0080134 feature_id[650].value <= threshold=38.91967582702637
node_626: feature_name=GO:0042127 feature_id[480].value <= threshold=37.72536659240723
node_627: feature_name=GO:2001251 feature_id[220].value <= threshold=32.564422607421875
node_628: feature_name=GO:0002829 feature_id[493].value <= threshold=2.444612741470337
node_629: feature_name=GO:0008588 feature_id[26].value <= threshold=2.3601274490356445
node_630: feature_name=GO:0033151 feature_id[7].value <= threshold=5.134376287460327
node_631: feature_name=GO:0048145 feature_id[348].value <= threshold=8.836549282073975
node_632: feature_name=GO:0030887 feature_id[274].value <= threshold=1.7000296115875244
node_633: feature_name=GO:0097190 feature_id[117].value <= threshold=33.02078819274902
node_634: feature_name=GO:0046685 feature_id[782].value <= threshold=8.040813446044922
node_635: feature_name=GO:0006346 feature_id[236].value <= threshold=6.384642839431763
node_636: feature_name=GO:0048537 feature_id[66].value <= threshold=3.1739262342453003
node_637: feature_name=GO:0070245 feature_id[326].value <= threshold=3.0491198301315308
node_638: feature_name=GO:0038065 feature_id[670].value <= threshold=2.0590850114822388
node_639: feature_name=GO:0005488 feature_id[187].value <= threshold=84.80076217651367
node_640: feature_name=GO:0060249 feature_id[390].value > threshold=0.07226398587226868
node_652: feature_name=GO:0006974 feature_id[516].value > threshold=5.17782768838515e-06
node_654: feature_name=hsa05210 feature_id[358].value <= threshold=18.730005264282227

node_655: feature_name=GO:0071850	feature_id[40].value <= threshold=5.475317001342773
node_656: feature_name=GO:1904029	feature_id[208].value <= threshold=26.575013160705566
node_657: feature_name=GO:0090594	feature_id[738].value <= threshold=3.000791072845459
node_658: feature_name=GO:0010948	feature_id[569].value > threshold=0.04386143572628498
node_666: feature_name=GO:0032764	feature_id[3].value <= threshold=2.5201677083969116
node_667: feature_name=GO:0023026	feature_id[69].value <= threshold=3.2760006189346313
node_668: feature_name=GO:0032464	feature_id[83].value <= threshold=2.5927021503448486
node_669: feature_name=GO:0071901	feature_id[407].value <= threshold=18.238012313842773
node_670: feature_name=GO:0015671	feature_id[103].value <= threshold=1.3113192915916443
node_671: feature_name=GO:0008340	feature_id[539].value <= threshold=4.860779762268066
node_672: feature_name=GO:0046006	feature_id[364].value <= threshold=2.4317692518234253
node_673: feature_name=GO:0042991	feature_id[684].value <= threshold=5.553565502166748
node_674: feature_name=GO:0002327	feature_id[408].value <= threshold=2.404940366744995
node_675: feature_name=GO:0001836	feature_id[450].value <= threshold=8.647934436798096
node_676: feature_name=GO:0048539	feature_id[319].value <= threshold=1.6308802962303162
node_677: feature_name=GO:0022408	feature_id[279].value <= threshold=4.064727783203125
node_678: feature_name=GO:0030099	feature_id[598].value > threshold=0.0001809061213862151
node_684: feature_name=GO:0001816	feature_id[685].value > threshold=1.3516043054551119e-05
node_688: feature_name=GO:0043200	feature_id[706].value <= threshold=4.01608681678772
node_689: feature_name=GO:1904029	feature_id[208].value <= threshold=16.503823280334473
node_690: feature_name=GO:0045840	feature_id[697].value <= threshold=6.23440408706665

Class: negative genes

Rules_4	passed counts:982
node_0: feature_name=GO:0042113	feature_id[0].value <= threshold=13.408552169799805
node_1: feature_name=GO:0007568	feature_id[534].value <= threshold=5.0313897132873535
node_2: feature_name=GO:0002705	feature_id[541].value <= threshold=3.200145721435547
node_3: feature_name=GO:1901525	feature_id[576].value <= threshold=0.4466460347175598
node_4: feature_name=GO:0048539	feature_id[319].value <= threshold=3.0399646759033203
node_5: feature_name=GO:0001910	feature_id[385].value <= threshold=3.7437864542007446
node_6: feature_name=GO:0043200	feature_id[706].value <= threshold=9.307284355163574
node_7: feature_name=GO:0001773	feature_id[308].value <= threshold=4.016931533813477
node_8: feature_name=GO:0090116	feature_id[97].value <= threshold=7.99645471572876
node_9: feature_name=GO:0019814	feature_id[189].value <= threshold=4.525782108306885
node_10: feature_name=GO:1902583	feature_id[215].value <= threshold=17.60310649871826

node_11: feature_name=GO:0045429 feature_id[737].value > threshold=1.6133361458778381
node_477: feature_name=GO:0036037 feature_id[445].value <= threshold=1.7181594371795654
node_478: feature_name=GO:0006244 feature_id[503].value <= threshold=0.951388418674469
node_479: feature_name=GO:0032461 feature_id[608].value <= threshold=2.6103241443634033
node_480: feature_name=GO:0044710 feature_id[719].value > threshold=2.228096718681627e-06
node_494: feature_name=GO:0009314 feature_id[296].value <= threshold=34.93696117401123
node_495: feature_name=GO:0050778 feature_id[802].value <= threshold=39.29364013671875
node_496: feature_name=GO:1903038 feature_id[492].value <= threshold=5.849650859832764
node_497: feature_name=GO:0002699 feature_id[606].value <= threshold=5.5405943393707275
node_498: feature_name=hsa00983 feature_id[181].value <= threshold=25.851045608520508
node_499: feature_name=GO:0002309 feature_id[416].value <= threshold=1.6235689520835876
node_500: feature_name=GO:1901698 feature_id[792].value <= threshold=47.70275688171387
node_501: feature_name=GO:0010216 feature_id[282].value <= threshold=1.6849713921546936
node_502: feature_name=GO:0006266 feature_id[297].value <= threshold=2.7199249267578125
node_503: feature_name=GO:0005488 feature_id[187].value > threshold=3.0373169010999845e-06
node_507: feature_name=GO:0045429 feature_id[737].value > threshold=1.614579975605011
node_511: feature_name=GO:0034103 feature_id[318].value <= threshold=5.832815647125244
node_512: feature_name=GO:0010663 feature_id[561].value <= threshold=4.157005429267883
node_513: feature_name=GO:0030887 feature_id[274].value <= threshold=1.8964150547981262
node_514: feature_name=GO:0048294 feature_id[113].value <= threshold=1.866134524345398
node_515: feature_name=GO:0072539 feature_id[68].value <= threshold=0.8600535988807678
node_516: feature_name=GO:0045840 feature_id[697].value <= threshold=4.963376045227051
node_517: feature_name=GO:0019222 feature_id[129].value > threshold=4.0122095015249215e-06
node_523: feature_name=GO:0008150 feature_id[528].value <= threshold=40.503862380981445
node_524: feature_name=GO:0006359 feature_id[87].value <= threshold=2.954534411430359
node_525: feature_name=GO:0010225 feature_id[558].value <= threshold=1.8247058987617493
node_526: feature_name=GO:0032461 feature_id[608].value <= threshold=1.6246166825294495
node_527: feature_name=GO:0007406 feature_id[537].value <= threshold=2.375624656677246
Class: negative genes

Rules_5 passed counts:763
node_0: feature_name=GO:0042113 feature_id[0].value <= threshold=13.408552169799805
node_1: feature_name=GO:0007568 feature_id[534].value > threshold=5.0313897132873535
node_913: feature_name=GO:0032763 feature_id[629].value <= threshold=0.31753237545490265
node_914: feature_name=GO:0097193 feature_id[167].value <= threshold=28.171168327331543

node_915: feature_name=GO:0002903 feature_id[10].value <= threshold=1.0534588098526
node_916: feature_name=GO:1904029 feature_id[208].value <= threshold=23.852136611938477
node_917: feature_name=GO:0050897 feature_id[164].value <= threshold=0.6870408356189728
node_918: feature_name=GO:0006139 feature_id[474].value > threshold=1.3052097624921544e-07
node_920: feature_name=GO:0002821 feature_id[588].value <= threshold=13.81072187423706
node_921: feature_name=GO:0006298 feature_id[621].value <= threshold=24.078600883483887
node_922: feature_name=GO:0003908 feature_id[11].value <= threshold=1.7776933312416077
node_923: feature_name=GO:0030098 feature_id[273].value > threshold=0.0079949083738029
node_927: feature_name=GO:0006808 feature_id[513].value <= threshold=4.34592080116272
node_928: feature_name=GO:0071887 feature_id[283].value <= threshold=10.353787899017334
node_929: feature_name=GO:0038001 feature_id[28].value <= threshold=3.9469382762908936
node_930: feature_name=GO:0042287 feature_id[20].value <= threshold=3.6209195852279663
node_931: feature_name=GO:0003968 feature_id[47].value <= threshold=2.004227638244629
node_932: feature_name=GO:0002698 feature_id[395].value <= threshold=18.51447582244873
node_933: feature_name=GO:0044710 feature_id[719].value <= threshold=179.7340316772461
node_934: feature_name=GO:0007568 feature_id[534].value > threshold=5.035318374633789
node_936: feature_name=GO:0006216 feature_id[504].value <= threshold=2.2605666518211365
node_937: feature_name=GO:0048569 feature_id[793].value <= threshold=5.340231895446777
node_938: feature_name=GO:0001777 feature_id[380].value <= threshold=4.100832939147949
node_939: feature_name=GO:0007600 feature_id[122].value <= threshold=171.13383102416992
node_940: feature_name=GO:0001779 feature_id[378].value <= threshold=6.234851121902466
node_941: feature_name=GO:0030291 feature_id[147].value <= threshold=10.8051118850708
node_942: feature_name=GO:0048534 feature_id[790].value > threshold=0.017984486185014248
node_944: feature_name=GO:0070245 feature_id[326].value <= threshold=3.1282339096069336
node_945: feature_name=GO:0009086 feature_id[547].value <= threshold=0.2896959036588669
node_946: feature_name=GO:0001889 feature_id[387].value <= threshold=17.643128395080566
node_947: feature_name=GO:0048144 feature_id[192].value > threshold=0.20887330174446106
node_951: feature_name=GO:0023026 feature_id[69].value <= threshold=3.403424024581909
node_952: feature_name=GO:0048147 feature_id[788].value <= threshold=4.188283443450928
node_953: feature_name=GO:0090116 feature_id[97].value <= threshold=4.617154359817505
node_954: feature_name=GO:0002863 feature_id[490].value <= threshold=4.8968470096588135
node_955: feature_name=GO:0002524 feature_id[72].value <= threshold=3.035340189933777
node_956: feature_name=GO:0042130 feature_id[16].value <= threshold=7.285413980484009
node_957: feature_name=GO:0071456 feature_id[500].value <= threshold=21.212870597839355
node_958: feature_name=GO:2001238 feature_id[27].value <= threshold=7.849715709686279

node_959: feature_name=GO:0023030 feature_id[45].value <= threshold=1.7420591711997986
node_960: feature_name=GO:0072593 feature_id[381].value <= threshold=22.993029594421387
node_961: feature_name=GO:0034101 feature_id[646].value <= threshold=13.430044651031494
node_962: feature_name=GO:0097153 feature_id[226].value <= threshold=10.147814273834229
node_963: feature_name=GO:0001836 feature_id[450].value <= threshold=7.252067804336548
node_964: feature_name=GO:0032504 feature_id[244].value > threshold=0.15777301788330078
node_968: feature_name=GO:0043379 feature_id[714].value <= threshold=1.5959861278533936
node_969: feature_name=GO:0001889 feature_id[387].value > threshold=0.3281313180923462
Class: negative genes

Rules_6 passed counts:654
node_0: feature_name=GO:0042113 feature_id[0].value <= threshold=13.408552169799805
node_1: feature_name=GO:0007568 feature_id[534].value <= threshold=5.0313897132873535
node_2: feature_name=GO:0002705 feature_id[541].value <= threshold=3.200145721435547
node_3: feature_name=GO:1901525 feature_id[576].value <= threshold=0.4466460347175598
node_4: feature_name=GO:0048539 feature_id[319].value <= threshold=3.0399646759033203
node_5: feature_name=GO:0001910 feature_id[385].value <= threshold=3.7437864542007446
node_6: feature_name=GO:0043200 feature_id[706].value <= threshold=9.307284355163574
node_7: feature_name=GO:0001773 feature_id[308].value <= threshold=4.016931533813477
node_8: feature_name=GO:0090116 feature_id[97].value <= threshold=7.99645471572876
node_9: feature_name=GO:0019814 feature_id[189].value <= threshold=4.525782108306885
node_10: feature_name=GO:1902583 feature_id[215].value <= threshold=17.60310649871826
node_11: feature_name=GO:0045429 feature_id[737].value <= threshold=1.6133361458778381
node_12: feature_name=GO:0003720 feature_id[228].value <= threshold=5.519326210021973
node_13: feature_name=GO:0046006 feature_id[364].value <= threshold=4.9985432624816895
node_14: feature_name=GO:0070424 feature_id[254].value <= threshold=6.233297109603882
node_15: feature_name=GO:0009892 feature_id[320].value <= threshold=59.99180793762207
node_16: feature_name=GO:0007064 feature_id[527].value <= threshold=11.57377815246582
node_17: feature_name=GO:0005575 feature_id[17].value <= threshold=15.417460918426514
node_18: feature_name=GO:0043368 feature_id[31].value <= threshold=4.059496641159058
node_19: feature_name=GO:0005164 feature_id[163].value <= threshold=5.793607711791992
node_20: feature_name=GO:0042130 feature_id[16].value <= threshold=5.073179721832275
node_21: feature_name=GO:0010216 feature_id[282].value <= threshold=4.834584474563599
node_22: feature_name=GO:0009628 feature_id[553].value <= threshold=29.314892768859863
node_23: feature_name=GO:0045628 feature_id[749].value <= threshold=3.1001367568969727

node_24: feature_name=GO:0042288 feature_id[100].value <= threshold=5.174932956695557
node_25: feature_name=GO:0002329 feature_id[406].value <= threshold=2.348930835723877
node_26: feature_name=GO:0051246 feature_id[642].value > threshold=0.6498909294605255
node_194: feature_name=GO:0009164 feature_id[546].value <= threshold=17.11970329284668
node_195: feature_name=GO:0001909 feature_id[386].value <= threshold=7.292566299438477
node_196: feature_name=GO:0003908 feature_id[11].value <= threshold=1.887226164340973
node_197: feature_name=GO:0009164 feature_id[546].value > threshold=0.26642198860645294
node_359: feature_name=GO:0009164 feature_id[546].value > threshold=0.2696942090988159
node_361: feature_name=hsa05202 feature_id[50].value <= threshold=14.989679336547852
node_362: feature_name=GO:0001775 feature_id[505].value <= threshold=15.726949691772461
node_363: feature_name=GO:0002634 feature_id[449].value <= threshold=2.825531005859375
node_364: feature_name=GO:0010038 feature_id[457].value <= threshold=9.901018619537354
node_365: feature_name=GO:0044238 feature_id[295].value <= threshold=139.80535888671875
node_366: feature_name=GO:0051246 feature_id[642].value > threshold=0.6527281999588013
node_368: feature_name=GO:0044092 feature_id[432].value <= threshold=11.084641933441162
node_369: feature_name=GO:0042162 feature_id[355].value <= threshold=9.683155536651611
node_370: feature_name=GO:0045945 feature_id[267].value <= threshold=3.6181851625442505
node_371: feature_name=GO:0036296 feature_id[252].value <= threshold=3.214281916618347
node_372: feature_name=GO:0097506 feature_id[51].value <= threshold=2.9644681215286255
node_373: feature_name=GO:0019740 feature_id[581].value <= threshold=2.2853575944900513
node_374: feature_name=GO:0019660 feature_id[70].value <= threshold=1.2831979990005493
node_375: feature_name=GO:0071901 feature_id[407].value <= threshold=14.498775482177734
node_376: feature_name=GO:0043170 feature_id[467].value > threshold=4.473330818655086e-06
node_380: feature_name=GO:0046718 feature_id[781].value <= threshold=3.494875192642212
node_381: feature_name=GO:0030983 feature_id[291].value <= threshold=10.994179248809814
node_382: feature_name=GO:0010663 feature_id[561].value <= threshold=2.7056833505630493
node_383: feature_name=GO:0031667 feature_id[648].value > threshold=0.0020462179090827703
node_387: feature_name=GO:1990572 feature_id[243].value <= threshold=1.9359037280082703
node_388: feature_name=GO:0032753 feature_id[101].value <= threshold=1.182796835899353
node_389: feature_name=GO:0097153 feature_id[226].value <= threshold=0.34601132571697235
node_390: feature_name=GO:0002829 feature_id[493].value <= threshold=1.246164083480835
node_391: feature_name=GO:0006346 feature_id[236].value <= threshold=2.040414571762085
Class: negative genes

Rules_7 passed counts:386

node_0: feature_name=GO:0042113	feature_id[0].value <= threshold=13.408552169799805
node_1: feature_name=GO:0007568	feature_id[534].value > threshold=5.0313897132873535
node_913: feature_name=GO:0032763	feature_id[629].value > threshold=0.31753237545490265
node_1195: feature_name=GO:0071301	feature_id[442].value <= threshold=0.11534593254327774
node_1196: feature_name=GO:0046500	feature_id[171].value <= threshold=0.6314916908740997
node_1197: feature_name=GO:0030852	feature_id[22].value <= threshold=2.9768868684768677
node_1198: feature_name=hsa04672	feature_id[276].value <= threshold=8.06681227684021
node_1199: feature_name=GO:0001836	feature_id[450].value > threshold=0.09961023926734924
node_1201: feature_name=GO:0070230	feature_id[64].value <= threshold=3.3956379890441895
node_1202: feature_name=GO:0070102	feature_id[49].value <= threshold=4.805386543273926
node_1203: feature_name=GO:0045553	feature_id[736].value <= threshold=1.4970332980155945
node_1204: feature_name=GO:0051311	feature_id[58].value <= threshold=3.301889181137085
node_1205: feature_name=GO:0030291	feature_id[147].value <= threshold=8.156315326690674
node_1206: feature_name=GO:0045637	feature_id[241].value > threshold=0.01834342861548066
node_1208: feature_name=GO:0002890	feature_id[98].value <= threshold=2.767483353614807
node_1209: feature_name=GO:0046632	feature_id[248].value <= threshold=11.663254261016846
node_1210: feature_name=GO:0008588	feature_id[26].value <= threshold=1.391375720500946
node_1211: feature_name=GO:0010639	feature_id[564].value <= threshold=18.975126266479492
node_1212: feature_name=GO:0046685	feature_id[782].value <= threshold=5.153738260269165
node_1213: feature_name=GO:0038116	feature_id[668].value <= threshold=1.7859655618667603
node_1214: feature_name=GO:0046449	feature_id[763].value <= threshold=5.603115558624268
node_1215: feature_name=GO:0048539	feature_id[319].value <= threshold=0.5478232800960541
node_1216: feature_name=GO:0007089	feature_id[522].value <= threshold=0.9940084517002106
node_1217: feature_name=GO:0042287	feature_id[20].value <= threshold=2.693045496940613
node_1218: feature_name=GO:0044446	feature_id[184].value <= threshold=113.0758171081543
node_1219: feature_name=GO:0098602	feature_id[702].value <= threshold=68.95328903198242
node_1220: feature_name=GO:0035726	feature_id[270].value <= threshold=1.7167096138000488
node_1221: feature_name=GO:1902564	feature_id[85].value <= threshold=2.8364468812942505
node_1222: feature_name=GO:0006979	feature_id[44].value > threshold=0.6869173645973206
node_1224: feature_name=GO:0044464	feature_id[56].value <= threshold=67.13260269165039
node_1225: feature_name=GO:0045629	feature_id[746].value <= threshold=2.3414241075515747
node_1226: feature_name=GO:0002903	feature_id[10].value <= threshold=2.715258240699768
node_1227: feature_name=GO:0043375	feature_id[717].value <= threshold=1.9525017738342285
node_1228: feature_name=GO:0032762	feature_id[630].value > threshold=0.29527929425239563
node_1232: feature_name=GO:0048569	feature_id[793].value <= threshold=3.419509768486023

node_1233: feature_name=GO:0045577 feature_id[109].value <= threshold=4.633661270141602
node_1234: feature_name=GO:0070245 feature_id[326].value <= threshold=2.441925883293152
node_1235: feature_name=GO:0002864 feature_id[488].value <= threshold=3.03904390335083
Class: negative genes

Rules_8 passed counts:355
node_0: feature_name=GO:0042113 feature_id[0].value <= threshold=13.408552169799805
node_1: feature_name=GO:0007568 feature_id[534].value <= threshold=5.0313897132873535
node_2: feature_name=GO:0002705 feature_id[541].value <= threshold=3.200145721435547
node_3: feature_name=GO:1901525 feature_id[576].value <= threshold=0.4466460347175598
node_4: feature_name=GO:0048539 feature_id[319].value <= threshold=3.0399646759033203
node_5: feature_name=GO:0001910 feature_id[385].value <= threshold=3.7437864542007446
node_6: feature_name=GO:0043200 feature_id[706].value <= threshold=9.307284355163574
node_7: feature_name=GO:0001773 feature_id[308].value <= threshold=4.016931533813477
node_8: feature_name=GO:0090116 feature_id[97].value <= threshold=7.99645471572876
node_9: feature_name=GO:0019814 feature_id[189].value <= threshold=4.525782108306885
node_10: feature_name=GO:1902583 feature_id[215].value <= threshold=17.60310649871826
node_11: feature_name=GO:0045429 feature_id[737].value <= threshold=1.6133361458778381
node_12: feature_name=GO:0003720 feature_id[228].value <= threshold=5.519326210021973
node_13: feature_name=GO:0046006 feature_id[364].value <= threshold=4.9985432624816895
node_14: feature_name=GO:0070424 feature_id[254].value <= threshold=6.233297109603882
node_15: feature_name=GO:0009892 feature_id[320].value <= threshold=59.99180793762207
node_16: feature_name=GO:0007064 feature_id[527].value <= threshold=11.57377815246582
node_17: feature_name=GO:0005575 feature_id[17].value <= threshold=15.417460918426514
node_18: feature_name=GO:0043368 feature_id[31].value <= threshold=4.059496641159058
node_19: feature_name=GO:0005164 feature_id[163].value <= threshold=5.793607711791992
node_20: feature_name=GO:0042130 feature_id[16].value <= threshold=5.073179721832275
node_21: feature_name=GO:0010216 feature_id[282].value <= threshold=4.834584474563599
node_22: feature_name=GO:0009628 feature_id[553].value <= threshold=29.314892768859863
node_23: feature_name=GO:0045628 feature_id[749].value <= threshold=3.1001367568969727
node_24: feature_name=GO:0042288 feature_id[100].value <= threshold=5.174932956695557
node_25: feature_name=GO:0002329 feature_id[406].value <= threshold=2.348930835723877
node_26: feature_name=GO:0051246 feature_id[642].value <= threshold=0.6498909294605255
node_27: feature_name=GO:0042162 feature_id[355].value <= threshold=8.05103588104248
node_28: feature_name=hsa04668 feature_id[333].value <= threshold=5.614926338195801

node_29: feature_name=GO:0032200 feature_id[613].value <= threshold=28.735913276672363
node_30: feature_name=hsa04662 feature_id[334].value <= threshold=9.588344097137451
node_31: feature_name=GO:1901992 feature_id[105].value <= threshold=5.291427850723267
node_32: feature_name=GO:0072341 feature_id[144].value <= threshold=6.01465106010437
node_33: feature_name=GO:0047485 feature_id[121].value <= threshold=6.657836437225342
node_34: feature_name=GO:0090594 feature_id[738].value <= threshold=1.8673912286758423
node_35: feature_name=GO:0043525 feature_id[524].value <= threshold=2.881397008895874
node_36: feature_name=GO:0032504 feature_id[244].value <= threshold=17.22207546234131
node_37: feature_name=GO:0051348 feature_id[126].value <= threshold=5.933559417724609
node_38: feature_name=GO:0042522 feature_id[692].value <= threshold=1.78694087266922
node_39: feature_name=GO:0031100 feature_id[73].value <= threshold=3.2312101125717163
node_40: feature_name=GO:0042493 feature_id[149].value <= threshold=19.771170616149902
node_41: feature_name=GO:0001552 feature_id[366].value <= threshold=2.1711617708206177
node_42: feature_name=GO:0046483 feature_id[391].value <= threshold=320.9667053222656
node_43: feature_name=GO:0002439 feature_id[431].value <= threshold=1.832722783088684
node_44: feature_name=GO:0006555 feature_id[514].value <= threshold=5.321640968322754
node_45: feature_name=GO:0070424 feature_id[254].value <= threshold=3.321003556251526
node_46: feature_name=hsa05144 feature_id[751].value <= threshold=2.635499954223633
node_47: feature_name=GO:0032633 feature_id[627].value <= threshold=1.2592533230781555
node_48: feature_name=GO:0019814 feature_id[189].value <= threshold=2.1583125591278076
node_49: feature_name=GO:1901989 feature_id[30].value <= threshold=3.3150794506073
node_50: feature_name=GO:1903318 feature_id[196].value <= threshold=1.62252539396286
node_51: feature_name=GO:0060576 feature_id[204].value <= threshold=2.1097792387008667
node_52: feature_name=hsa05221 feature_id[349].value > threshold=0.8723124265670776
node_96: feature_name=hsa05221 feature_id[349].value > threshold=0.8756309151649475
node_98: feature_name=GO:0046794 feature_id[794].value <= threshold=6.585465431213379
node_99: feature_name=GO:0005035 feature_id[133].value <= threshold=0.6986835598945618
node_100: feature_name=hsa04640 feature_id[79].value <= threshold=1.458605408668518
node_101: feature_name=GO:0045945 feature_id[267].value <= threshold=1.6378425359725952
Class: negative genes

Rules_9 passed counts:241
node_0: feature_name=GO:0042113 feature_id[0].value <= threshold=13.408552169799805
node_1: feature_name=GO:0007568 feature_id[534].value > threshold=5.0313897132873535
node_913: feature_name=GO:0032763 feature_id[629].value <= threshold=0.31753237545490265

node_914: feature_name=GO:0097193	feature_id[167].value <= threshold=28.171168327331543
node_915: feature_name=GO:0002903	feature_id[10].value <= threshold=1.0534588098526
node_916: feature_name=GO:1904029	feature_id[208].value <= threshold=23.852136611938477
node_917: feature_name=GO:0050897	feature_id[164].value <= threshold=0.6870408356189728
node_918: feature_name=GO:0006139	feature_id[474].value > threshold=1.3052097624921544e-07
node_920: feature_name=GO:0002821	feature_id[588].value <= threshold=13.81072187423706
node_921: feature_name=GO:0006298	feature_id[621].value <= threshold=24.078600883483887
node_922: feature_name=GO:0003908	feature_id[11].value <= threshold=1.7776933312416077
node_923: feature_name=GO:0030098	feature_id[273].value > threshold=0.0079949083738029
node_927: feature_name=GO:0006808	feature_id[513].value <= threshold=4.34592080116272
node_928: feature_name=GO:0071887	feature_id[283].value <= threshold=10.353787899017334
node_929: feature_name=GO:0038001	feature_id[28].value <= threshold=3.9469382762908936
node_930: feature_name=GO:0042287	feature_id[20].value <= threshold=3.6209195852279663
node_931: feature_name=GO:0003968	feature_id[47].value <= threshold=2.004227638244629
node_932: feature_name=GO:0002698	feature_id[395].value <= threshold=18.51447582244873
node_933: feature_name=GO:0044710	feature_id[719].value <= threshold=179.7340316772461
node_934: feature_name=GO:0007568	feature_id[534].value > threshold=5.035318374633789
node_936: feature_name=GO:0006216	feature_id[504].value <= threshold=2.2605666518211365
node_937: feature_name=GO:0048569	feature_id[793].value <= threshold=5.340231895446777
node_938: feature_name=GO:0001777	feature_id[380].value <= threshold=4.100832939147949
node_939: feature_name=GO:0007600	feature_id[122].value <= threshold=171.13383102416992
node_940: feature_name=GO:0001779	feature_id[378].value <= threshold=6.234851121902466
node_941: feature_name=GO:0030291	feature_id[147].value <= threshold=10.8051118850708
node_942: feature_name=GO:0048534	feature_id[790].value > threshold=0.017984486185014248
node_944: feature_name=GO:0070245	feature_id[326].value <= threshold=3.1282339096069336
node_945: feature_name=GO:0009086	feature_id[547].value > threshold=0.2896959036588669
node_1017: feature_name=GO:0000302	feature_id[375].value > threshold=0.305715873837471
node_1019: feature_name=GO:0070141	feature_id[37].value <= threshold=4.857294321060181
node_1020: feature_name=GO:0009086	feature_id[547].value > threshold=0.2945319563150406
node_1024: feature_name=GO:0010332	feature_id[557].value <= threshold=15.092710971832275
node_1025: feature_name=GO:0002524	feature_id[72].value <= threshold=2.531021237373352
node_1026: feature_name=GO:0016032	feature_id[571].value <= threshold=60.173330307006836
node_1027: feature_name=GO:0002832	feature_id[491].value <= threshold=2.0699684023857117
node_1028: feature_name=GO:0008588	feature_id[26].value <= threshold=2.150238513946533
node_1029: feature_name=GO:0009615	feature_id[265].value > threshold=0.02372477948665619

node_1031: feature_name=GO:0002763 feature_id[271].value <= threshold=6.462319850921631
node_1032: feature_name=GO:0045840 feature_id[697].value <= threshold=10.30175256729126
node_1033: feature_name=GO:0051402 feature_id[568].value > threshold=1.2523645758628845
node_1041: feature_name=GO:0050852 feature_id[154].value > threshold=0.0008433434413745999
node_1043: feature_name=GO:0031667 feature_id[648].value > threshold=1.5418170094490051
node_1047: feature_name=GO:0009987 feature_id[554].value > threshold=1.8002276420593262
node_1051: feature_name=GO:0008588 feature_id[26].value <= threshold=0.9623689949512482
Class: negative genes

Rules_10 passed counts:240
node_0: feature_name=GO:0042113 feature_id[0].value <= threshold=13.408552169799805
node_1: feature_name=GO:0007568 feature_id[534].value > threshold=5.0313897132873535
node_913: feature_name=GO:0032763 feature_id[629].value <= threshold=0.31753237545490265
node_914: feature_name=GO:0097193 feature_id[167].value <= threshold=28.171168327331543
node_915: feature_name=GO:0002903 feature_id[10].value <= threshold=1.0534588098526
node_916: feature_name=GO:1904029 feature_id[208].value <= threshold=23.852136611938477
node_917: feature_name=GO:0050897 feature_id[164].value > threshold=0.6870408356189728
node_1095: feature_name=GO:0072539 feature_id[68].value <= threshold=2.5667877197265625
node_1096: feature_name=GO:0046500 feature_id[171].value <= threshold=3.195197820663452
node_1097: feature_name=GO:0045861 feature_id[761].value > threshold=0.22614753991365433
node_1099: feature_name=GO:0043372 feature_id[242].value <= threshold=4.892506122589111
node_1100: feature_name=GO:0042162 feature_id[355].value <= threshold=11.339290618896484
node_1101: feature_name=hsa04660 feature_id[472].value <= threshold=24.27848720550537
node_1102: feature_name=GO:0007049 feature_id[259].value <= threshold=209.23831176757812
node_1103: feature_name=GO:0050897 feature_id[164].value > threshold=0.6919849812984467
node_1105: feature_name=GO:0045945 feature_id[267].value <= threshold=3.307852625846863
node_1106: feature_name=GO:0046641 feature_id[773].value <= threshold=3.527579426765442
node_1107: feature_name=GO:0002262 feature_id[394].value > threshold=0.08302562311291695
node_1111: feature_name=GO:0043226 feature_id[138].value > threshold=2.4930146992119262e-05
node_1113: feature_name=GO:0045787 feature_id[446].value > threshold=0.07611752673983574
node_1115: feature_name=GO:0045840 feature_id[697].value > threshold=0.10580676794052124
node_1117: feature_name=GO:0006927 feature_id[225].value <= threshold=4.950021743774414
node_1118: feature_name=GO:0002643 feature_id[4].value <= threshold=3.364396333694458
node_1119: feature_name=GO:0016571 feature_id[725].value <= threshold=38.268903732299805
node_1120: feature_name=GO:0072593 feature_id[381].value <= threshold=19.997477531433105

node_1121: feature_name=GO:0070245 feature_id[326].value <= threshold=3.2926437854766846
node_1122: feature_name=GO:0008327 feature_id[77].value <= threshold=1.4845957159996033
Class: negative genes

Rules_11 passed counts:211
node_0: feature_name=GO:0042113 feature_id[0].value <= threshold=13.408552169799805
node_1: feature_name=GO:0007568 feature_id[534].value <= threshold=5.0313897132873535
node_2: feature_name=GO:0002705 feature_id[541].value <= threshold=3.200145721435547
node_3: feature_name=GO:1901525 feature_id[576].value > threshold=0.4466460347175598
node_617: feature_name=GO:0005622 feature_id[233].value <= threshold=90.85730743408203
node_618: feature_name=GO:1903147 feature_id[200].value <= threshold=7.326428413391113
node_619: feature_name=GO:0003964 feature_id[317].value <= threshold=5.575642108917236
node_620: feature_name=GO:0046498 feature_id[203].value <= threshold=6.620223522186279
node_621: feature_name=GO:0002562 feature_id[25].value <= threshold=10.287704467773438
node_622: feature_name=GO:0006919 feature_id[246].value <= threshold=5.801334381103516
node_623: feature_name=GO:0001889 feature_id[387].value > threshold=2.679414987564087
node_785: feature_name=GO:0030887 feature_id[274].value <= threshold=1.1379476189613342
node_786: feature_name=GO:1902564 feature_id[85].value <= threshold=1.4637635350227356
node_787: feature_name=GO:0045402 feature_id[286].value <= threshold=1.5249269604682922
node_788: feature_name=GO:0034614 feature_id[655].value <= threshold=9.142115116119385
node_789: feature_name=GO:0000783 feature_id[191].value <= threshold=1.5124736428260803
node_790: feature_name=GO:0048145 feature_id[348].value > threshold=0.026725558564066887
node_794: feature_name=GO:0044092 feature_id[432].value <= threshold=31.540730476379395
node_795: feature_name=GO:0042771 feature_id[686].value <= threshold=6.047890663146973
node_796: feature_name=GO:0070301 feature_id[81].value <= threshold=5.959718465805054
node_797: feature_name=GO:0006809 feature_id[511].value <= threshold=9.000734329223633
node_798: feature_name=GO:0008150 feature_id[528].value > threshold=0.40075723826885223
node_800: feature_name=GO:0010663 feature_id[561].value > threshold=0.20622049272060394
node_802: feature_name=GO:0009636 feature_id[551].value > threshold=0.12915228679776192
node_804: feature_name=GO:0002524 feature_id[72].value <= threshold=1.0410467386245728
node_805: feature_name=GO:0035872 feature_id[659].value <= threshold=6.628145217895508
node_806: feature_name=GO:0010225 feature_id[558].value <= threshold=4.188441753387451
node_807: feature_name=GO:0002262 feature_id[394].value <= threshold=8.843595027923584
node_808: feature_name=GO:0048569 feature_id[793].value <= threshold=4.370251417160034
node_809: feature_name=GO:0046982 feature_id[172].value > threshold=0.010676630306988955

Class: negative genes

Rules_12 passed counts:165
node_0: feature_name=GO:0042113 feature_id[0].value <= threshold=13.408552169799805
node_1: feature_name=GO:0007568 feature_id[534].value <= threshold=5.0313897132873535
node_2: feature_name=GO:0002705 feature_id[541].value > threshold=3.200145721435547
node_870: feature_name=GO:0046634 feature_id[46].value <= threshold=8.9394211769104
node_871: feature_name=GO:0048523 feature_id[293].value <= threshold=19.963229179382324
node_872: feature_name=GO:2001237 feature_id[796].value > threshold=0.09761488810181618
node_880: feature_name=GO:0006808 feature_id[513].value <= threshold=1.7969900965690613
node_881: feature_name=GO:0032479 feature_id[441].value > threshold=0.13597124069929123
node_887: feature_name=GO:0043226 feature_id[138].value <= threshold=12.855401992797852
node_888: feature_name=GO:0060576 feature_id[204].value <= threshold=2.0569241046905518
node_889: feature_name=GO:0002719 feature_id[479].value <= threshold=5.209295988082886
node_890: feature_name=GO:0031104 feature_id[111].value <= threshold=1.9710296988487244
node_891: feature_name=GO:2001251 feature_id[220].value <= threshold=2.3109558820724487
node_892: feature_name=GO:0033077 feature_id[134].value <= threshold=8.212203025817871
node_893: feature_name=GO:0044026 feature_id[15].value <= threshold=1.613231599330902
Class: negative genes

Rules_13 passed counts:144
node_0: feature_name=GO:0042113 feature_id[0].value > threshold=13.408552169799805
node_1454: feature_name=GO:0050851 feature_id[29].value > threshold=9.650307655334473
node_1534: feature_name=GO:1902166 feature_id[301].value > threshold=0.303210511803627
node_1548: feature_name=GO:0035872 feature_id[659].value <= threshold=35.09038162231445
node_1549: feature_name=GO:0032069 feature_id[614].value > threshold=3.441983938217163
node_1571: feature_name=GO:0050864 feature_id[288].value > threshold=7.516624450683594
node_1575: feature_name=GO:2000772 feature_id[95].value > threshold=0.9979664981365204
node_1579: feature_name=GO:0043525 feature_id[524].value <= threshold=22.19614315032959
node_1580: feature_name=GO:0032479 feature_id[441].value > threshold=4.490016937255859
node_1584: feature_name=GO:0042130 feature_id[16].value <= threshold=14.421725749969482
node_1585: feature_name=GO:0001782 feature_id[240].value <= threshold=14.421442985534668
node_1586: feature_name=GO:0038061 feature_id[671].value <= threshold=36.05139923095703
Class: positive genes

Rules_14	passed counts:135
node_0: feature_name=GO:0042113	feature_id[0].value <= threshold=13.408552169799805
node_1: feature_name=GO:0007568	feature_id[534].value <= threshold=5.0313897132873535
node_2: feature_name=GO:0002705	feature_id[541].value <= threshold=3.200145721435547
node_3: feature_name=GO:1901525	feature_id[576].value <= threshold=0.4466460347175598
node_4: feature_name=GO:0048539	feature_id[319].value <= threshold=3.0399646759033203
node_5: feature_name=GO:0001910	feature_id[385].value <= threshold=3.7437864542007446
node_6: feature_name=GO:0043200	feature_id[706].value <= threshold=9.307284355163574
node_7: feature_name=GO:0001773	feature_id[308].value <= threshold=4.016931533813477
node_8: feature_name=GO:0090116	feature_id[97].value <= threshold=7.99645471572876
node_9: feature_name=GO:0019814	feature_id[189].value <= threshold=4.525782108306885
node_10: feature_name=GO:1902583	feature_id[215].value <= threshold=17.60310649871826
node_11: feature_name=GO:0045429	feature_id[737].value <= threshold=1.6133361458778381
node_12: feature_name=GO:0003720	feature_id[228].value <= threshold=5.519326210021973
node_13: feature_name=GO:0046006	feature_id[364].value <= threshold=4.9985432624816895
node_14: feature_name=GO:0070424	feature_id[254].value <= threshold=6.233297109603882
node_15: feature_name=GO:0009892	feature_id[320].value <= threshold=59.99180793762207
node_16: feature_name=GO:0007064	feature_id[527].value <= threshold=11.57377815246582
node_17: feature_name=GO:0005575	feature_id[17].value <= threshold=15.417460918426514
node_18: feature_name=GO:0043368	feature_id[31].value <= threshold=4.059496641159058
node_19: feature_name=GO:0005164	feature_id[163].value <= threshold=5.793607711791992
node_20: feature_name=GO:0042130	feature_id[16].value <= threshold=5.073179721832275
node_21: feature_name=GO:0010216	feature_id[282].value <= threshold=4.834584474563599
node_22: feature_name=GO:0009628	feature_id[553].value <= threshold=29.314892768859863
node_23: feature_name=GO:0045628	feature_id[749].value <= threshold=3.1001367568969727
node_24: feature_name=GO:0042288	feature_id[100].value <= threshold=5.174932956695557
node_25: feature_name=GO:0002329	feature_id[406].value <= threshold=2.348930835723877
node_26: feature_name=GO:0051246	feature_id[642].value <= threshold=0.6498909294605255
node_27: feature_name=GO:0042162	feature_id[355].value <= threshold=8.05103588104248
node_28: feature_name=hsa04668	feature_id[333].value <= threshold=5.614926338195801
node_29: feature_name=GO:0032200	feature_id[613].value <= threshold=28.735913276672363
node_30: feature_name=hsa04662	feature_id[334].value <= threshold=9.588344097137451
node_31: feature_name=GO:1901992	feature_id[105].value <= threshold=5.291427850723267
node_32: feature_name=GO:0072341	feature_id[144].value <= threshold=6.01465106010437
node_33: feature_name=GO:0047485	feature_id[121].value <= threshold=6.657836437225342

node_34: feature_name=GO:0090594	feature_id[738].value <= threshold=1.8673912286758423
node_35: feature_name=GO:0043525	feature_id[524].value <= threshold=2.881397008895874
node_36: feature_name=GO:0032504	feature_id[244].value <= threshold=17.22207546234131
node_37: feature_name=GO:0051348	feature_id[126].value <= threshold=5.933559417724609
node_38: feature_name=GO:0042522	feature_id[692].value <= threshold=1.78694087266922
node_39: feature_name=GO:0031100	feature_id[73].value <= threshold=3.2312101125717163
node_40: feature_name=GO:0042493	feature_id[149].value <= threshold=19.771170616149902
node_41: feature_name=GO:0001552	feature_id[366].value <= threshold=2.1711617708206177
node_42: feature_name=GO:0046483	feature_id[391].value <= threshold=320.9667053222656
node_43: feature_name=GO:0002439	feature_id[431].value <= threshold=1.832722783088684
node_44: feature_name=GO:0006555	feature_id[514].value <= threshold=5.321640968322754
node_45: feature_name=GO:0070424	feature_id[254].value <= threshold=3.321003556251526
node_46: feature_name=hsa05144	feature_id[751].value <= threshold=2.635499954223633
node_47: feature_name=GO:0032633	feature_id[627].value <= threshold=1.2592533230781555
node_48: feature_name=GO:0019814	feature_id[189].value <= threshold=2.1583125591278076
node_49: feature_name=GO:1901989	feature_id[30].value <= threshold=3.3150794506073
node_50: feature_name=GO:1903318	feature_id[196].value <= threshold=1.62252539396286
node_51: feature_name=GO:0060576	feature_id[204].value <= threshold=2.1097792387008667
node_52: feature_name=hsa05221	feature_id[349].value <= threshold=0.8723124265670776
node_53: feature_name=GO:1903896	feature_id[135].value <= threshold=1.397229254245758
node_54: feature_name=GO:0097028	feature_id[443].value <= threshold=1.2365349531173706
node_55: feature_name=GO:0048660	feature_id[403].value <= threshold=1.9425984621047974
node_56: feature_name=GO:0043011	feature_id[150].value <= threshold=1.3008450269699097
node_57: feature_name=GO:0023056	feature_id[632].value <= threshold=2.5618948936462402
node_58: feature_name=GO:0005164	feature_id[163].value <= threshold=0.9787287414073944
node_59: feature_name=GO:0050707	feature_id[797].value <= threshold=0.9616408348083496
node_60: feature_name=hsa05202	feature_id[50].value <= threshold=1.5331347584724426
node_61: feature_name=GO:0002718	feature_id[481].value > threshold=0.6544264256954193
node_63: feature_name=GO:0043200	feature_id[706].value <= threshold=0.9459066987037659

Class: negative genes

Rules_15	passed counts:126
node_0: feature_name=GO:0042113	feature_id[0].value <= threshold=13.408552169799805
node_1: feature_name=GO:0007568	feature_id[534].value <= threshold=5.0313897132873535
node_2: feature_name=GO:0002705	feature_id[541].value <= threshold=3.200145721435547

node_3: feature_name=GO:1901525	feature_id[576].value <= threshold=0.4466460347175598
node_4: feature_name=GO:0048539	feature_id[319].value <= threshold=3.0399646759033203
node_5: feature_name=GO:0001910	feature_id[385].value <= threshold=3.7437864542007446
node_6: feature_name=GO:0043200	feature_id[706].value <= threshold=9.307284355163574
node_7: feature_name=GO:0001773	feature_id[308].value <= threshold=4.016931533813477
node_8: feature_name=GO:0090116	feature_id[97].value <= threshold=7.99645471572876
node_9: feature_name=GO:0019814	feature_id[189].value <= threshold=4.525782108306885
node_10: feature_name=GO:1902583	feature_id[215].value <= threshold=17.60310649871826
node_11: feature_name=GO:0045429	feature_id[737].value <= threshold=1.6133361458778381
node_12: feature_name=GO:0003720	feature_id[228].value <= threshold=5.519326210021973
node_13: feature_name=GO:0046006	feature_id[364].value <= threshold=4.9985432624816895
node_14: feature_name=GO:0070424	feature_id[254].value <= threshold=6.233297109603882
node_15: feature_name=GO:0009892	feature_id[320].value <= threshold=59.99180793762207
node_16: feature_name=GO:0007064	feature_id[527].value <= threshold=11.57377815246582
node_17: feature_name=GO:0005575	feature_id[17].value <= threshold=15.417460918426514
node_18: feature_name=GO:0043368	feature_id[31].value <= threshold=4.059496641159058
node_19: feature_name=GO:0005164	feature_id[163].value <= threshold=5.793607711791992
node_20: feature_name=GO:0042130	feature_id[16].value <= threshold=5.073179721832275
node_21: feature_name=GO:0010216	feature_id[282].value <= threshold=4.834584474563599
node_22: feature_name=GO:0009628	feature_id[553].value <= threshold=29.314892768859863
node_23: feature_name=GO:0045628	feature_id[749].value <= threshold=3.1001367568969727
node_24: feature_name=GO:0042288	feature_id[100].value <= threshold=5.174932956695557
node_25: feature_name=GO:0002329	feature_id[406].value <= threshold=2.348930835723877
node_26: feature_name=GO:0051246	feature_id[642].value <= threshold=0.6498909294605255
node_27: feature_name=GO:0042162	feature_id[355].value <= threshold=8.05103588104248
node_28: feature_name=hsa04668	feature_id[333].value <= threshold=5.614926338195801
node_29: feature_name=GO:0032200	feature_id[613].value <= threshold=28.735913276672363
node_30: feature_name=hsa04662	feature_id[334].value <= threshold=9.588344097137451
node_31: feature_name=GO:1901992	feature_id[105].value <= threshold=5.291427850723267
node_32: feature_name=GO:0072341	feature_id[144].value <= threshold=6.01465106010437
node_33: feature_name=GO:0047485	feature_id[121].value <= threshold=6.657836437225342
node_34: feature_name=GO:0090594	feature_id[738].value <= threshold=1.8673912286758423
node_35: feature_name=GO:0043525	feature_id[524].value <= threshold=2.881397008895874
node_36: feature_name=GO:0032504	feature_id[244].value <= threshold=17.22207546234131
node_37: feature_name=GO:0051348	feature_id[126].value <= threshold=5.933559417724609

node_38: feature_name=GO:0042522 feature_id[692].value <= threshold=1.78694087266922
node_39: feature_name=GO:0031100 feature_id[73].value <= threshold=3.2312101125717163
node_40: feature_name=GO:0042493 feature_id[149].value <= threshold=19.771170616149902
node_41: feature_name=GO:0001552 feature_id[366].value <= threshold=2.1711617708206177
node_42: feature_name=GO:0046483 feature_id[391].value <= threshold=320.9667053222656
node_43: feature_name=GO:0002439 feature_id[431].value <= threshold=1.832722783088684
node_44: feature_name=GO:0006555 feature_id[514].value <= threshold=5.321640968322754
node_45: feature_name=GO:0070424 feature_id[254].value <= threshold=3.321003556251526
node_46: feature_name=hsa05144 feature_id[751].value <= threshold=2.635499954223633
node_47: feature_name=GO:0032633 feature_id[627].value <= threshold=1.2592533230781555
node_48: feature_name=GO:0019814 feature_id[189].value <= threshold=2.1583125591278076
node_49: feature_name=GO:1901989 feature_id[30].value <= threshold=3.3150794506073
node_50: feature_name=GO:1903318 feature_id[196].value <= threshold=1.62252539396286
node_51: feature_name=GO:0060576 feature_id[204].value <= threshold=2.1097792387008667
node_52: feature_name=hsa05221 feature_id[349].value <= threshold=0.8723124265670776
node_53: feature_name=GO:1903896 feature_id[135].value <= threshold=1.397229254245758
node_54: feature_name=GO:0097028 feature_id[443].value <= threshold=1.2365349531173706
node_55: feature_name=GO:0048660 feature_id[403].value <= threshold=1.9425984621047974
node_56: feature_name=GO:0043011 feature_id[150].value <= threshold=1.3008450269699097
node_57: feature_name=GO:0023056 feature_id[632].value <= threshold=2.5618948936462402
node_58: feature_name=GO:0005164 feature_id[163].value <= threshold=0.9787287414073944
node_59: feature_name=GO:0050707 feature_id[797].value <= threshold=0.9616408348083496
node_60: feature_name=hsa05202 feature_id[50].value > threshold=1.5331347584724426
node_68: feature_name=GO:0007049 feature_id[259].value > threshold=4.808156063518254e-06
Class: negative genes

Rules_16 passed counts:107
node_0: feature_name=GO:0042113 feature_id[0].value <= threshold=13.408552169799805
node_1: feature_name=GO:0007568 feature_id[534].value <= threshold=5.0313897132873535
node_2: feature_name=GO:0002705 feature_id[541].value <= threshold=3.200145721435547
node_3: feature_name=GO:1901525 feature_id[576].value <= threshold=0.4466460347175598
node_4: feature_name=GO:0048539 feature_id[319].value <= threshold=3.0399646759033203
node_5: feature_name=GO:0001910 feature_id[385].value <= threshold=3.7437864542007446
node_6: feature_name=GO:0043200 feature_id[706].value <= threshold=9.307284355163574
node_7: feature_name=GO:0001773 feature_id[308].value <= threshold=4.016931533813477

node_8: feature_name=GO:0090116	feature_id[97].value <= threshold=7.99645471572876
node_9: feature_name=GO:0019814	feature_id[189].value <= threshold=4.525782108306885
node_10: feature_name=GO:1902583	feature_id[215].value <= threshold=17.60310649871826
node_11: feature_name=GO:0045429	feature_id[737].value <= threshold=1.6133361458778381
node_12: feature_name=GO:0003720	feature_id[228].value <= threshold=5.519326210021973
node_13: feature_name=GO:0046006	feature_id[364].value <= threshold=4.9985432624816895
node_14: feature_name=GO:0070424	feature_id[254].value <= threshold=6.233297109603882
node_15: feature_name=GO:0009892	feature_id[320].value <= threshold=59.99180793762207
node_16: feature_name=GO:0007064	feature_id[527].value <= threshold=11.57377815246582
node_17: feature_name=GO:0005575	feature_id[17].value <= threshold=15.417460918426514
node_18: feature_name=GO:0043368	feature_id[31].value <= threshold=4.059496641159058
node_19: feature_name=GO:0005164	feature_id[163].value <= threshold=5.793607711791992
node_20: feature_name=GO:0042130	feature_id[16].value <= threshold=5.073179721832275
node_21: feature_name=GO:0010216	feature_id[282].value <= threshold=4.834584474563599
node_22: feature_name=GO:0009628	feature_id[553].value <= threshold=29.314892768859863
node_23: feature_name=GO:0045628	feature_id[749].value <= threshold=3.1001367568969727
node_24: feature_name=GO:0042288	feature_id[100].value <= threshold=5.174932956695557
node_25: feature_name=GO:0002329	feature_id[406].value <= threshold=2.348930835723877
node_26: feature_name=GO:0051246	feature_id[642].value > threshold=0.6498909294605255
node_194: feature_name=GO:0009164	feature_id[546].value <= threshold=17.11970329284668
node_195: feature_name=GO:0001909	feature_id[386].value <= threshold=7.292566299438477
node_196: feature_name=GO:0003908	feature_id[11].value <= threshold=1.887226164340973
node_197: feature_name=GO:0009164	feature_id[546].value <= threshold=0.26642198860645294
node_198: feature_name=GO:2001242	feature_id[24].value <= threshold=9.207026481628418
node_199: feature_name=GO:0051246	feature_id[642].value > threshold=0.6504445374011993
node_203: feature_name=GO:0032461	feature_id[608].value <= threshold=2.607542634010315
node_204: feature_name=GO:0007584	feature_id[529].value <= threshold=16.099515914916992
node_205: feature_name=hsa05221	feature_id[349].value <= threshold=9.359850406646729
node_206: feature_name=GO:0002636	feature_id[447].value <= threshold=1.615447759628296
node_207: feature_name=GO:0033993	feature_id[421].value <= threshold=2.96540611088858e-05
node_208: feature_name=GO:0006968	feature_id[517].value <= threshold=1.0216356217861176
node_209: feature_name=GO:0048545	feature_id[360].value <= threshold=0.007158883148804307
node_210: feature_name=GO:0002250	feature_id[417].value <= threshold=0.3659738004207611

Class: negative genes

Rules_17	passed counts:104
node_0: feature_name=GO:0042113	feature_id[0].value <= threshold=13.408552169799805
node_1: feature_name=GO:0007568	feature_id[534].value <= threshold=5.0313897132873535
node_2: feature_name=GO:0002705	feature_id[541].value <= threshold=3.200145721435547
node_3: feature_name=GO:1901525	feature_id[576].value <= threshold=0.4466460347175598
node_4: feature_name=GO:0048539	feature_id[319].value <= threshold=3.0399646759033203
node_5: feature_name=GO:0001910	feature_id[385].value <= threshold=3.7437864542007446
node_6: feature_name=GO:0043200	feature_id[706].value <= threshold=9.307284355163574
node_7: feature_name=GO:0001773	feature_id[308].value <= threshold=4.016931533813477
node_8: feature_name=GO:0090116	feature_id[97].value <= threshold=7.99645471572876
node_9: feature_name=GO:0019814	feature_id[189].value <= threshold=4.525782108306885
node_10: feature_name=GO:1902583	feature_id[215].value <= threshold=17.60310649871826
node_11: feature_name=GO:0045429	feature_id[737].value <= threshold=1.6133361458778381
node_12: feature_name=GO:0003720	feature_id[228].value <= threshold=5.519326210021973
node_13: feature_name=GO:0046006	feature_id[364].value <= threshold=4.9985432624816895
node_14: feature_name=GO:0070424	feature_id[254].value <= threshold=6.233297109603882
node_15: feature_name=GO:0009892	feature_id[320].value <= threshold=59.99180793762207
node_16: feature_name=GO:0007064	feature_id[527].value <= threshold=11.57377815246582
node_17: feature_name=GO:0005575	feature_id[17].value <= threshold=15.417460918426514
node_18: feature_name=GO:0043368	feature_id[31].value <= threshold=4.059496641159058
node_19: feature_name=GO:0005164	feature_id[163].value <= threshold=5.793607711791992
node_20: feature_name=GO:0042130	feature_id[16].value <= threshold=5.073179721832275
node_21: feature_name=GO:0010216	feature_id[282].value <= threshold=4.834584474563599
node_22: feature_name=GO:0009628	feature_id[553].value <= threshold=29.314892768859863
node_23: feature_name=GO:0045628	feature_id[749].value <= threshold=3.1001367568969727
node_24: feature_name=GO:0042288	feature_id[100].value <= threshold=5.174932956695557
node_25: feature_name=GO:0002329	feature_id[406].value <= threshold=2.348930835723877
node_26: feature_name=GO:0051246	feature_id[642].value > threshold=0.6498909294605255
node_194: feature_name=GO:0009164	feature_id[546].value <= threshold=17.11970329284668
node_195: feature_name=GO:0001909	feature_id[386].value <= threshold=7.292566299438477
node_196: feature_name=GO:0003908	feature_id[11].value <= threshold=1.887226164340973
node_197: feature_name=GO:0009164	feature_id[546].value <= threshold=0.26642198860645294
node_198: feature_name=GO:2001242	feature_id[24].value <= threshold=9.207026481628418
node_199: feature_name=GO:0051246	feature_id[642].value > threshold=0.6504445374011993
node_203: feature_name=GO:0032461	feature_id[608].value <= threshold=2.607542634010315

node_204: feature_name=GO:0007584 feature_id[529].value <= threshold=16.099515914916992
node_205: feature_name=hsa05221 feature_id[349].value <= threshold=9.359850406646729
node_206: feature_name=GO:0002636 feature_id[447].value <= threshold=1.615447759628296
node_207: feature_name=GO:0033993 feature_id[421].value > threshold=2.96540611088858e-05
node_217: feature_name=GO:0002331 feature_id[404].value <= threshold=2.1883513927459717
node_218: feature_name=GO:0046498 feature_id[203].value <= threshold=4.085246205329895
node_219: feature_name=GO:0002832 feature_id[491].value <= threshold=3.0228612422943115
node_220: feature_name=GO:1904868 feature_id[306].value <= threshold=3.8674756288528442
node_221: feature_name=GO:0048545 feature_id[360].value <= threshold=27.38136100769043
node_222: feature_name=GO:0071310 feature_id[760].value <= threshold=2.3522024154663086
node_223: feature_name=GO:0001775 feature_id[505].value <= threshold=9.71301555633545
node_224: feature_name=GO:0071310 feature_id[760].value <= threshold=2.346743583679199
node_225: feature_name=GO:0001772 feature_id[91].value <= threshold=3.252573609352112
node_226: feature_name=GO:0036498 feature_id[359].value <= threshold=12.092026710510254
node_227: feature_name=hsa05340 feature_id[351].value > threshold=0.7677814364433289
node_281: feature_name=GO:0006359 feature_id[87].value <= threshold=0.846381276845932
node_282: feature_name=GO:0002326 feature_id[410].value <= threshold=1.929194152355194
node_283: feature_name=GO:0072610 feature_id[262].value <= threshold=1.4640317559242249
node_284: feature_name=GO:0005575 feature_id[17].value > threshold=0.0011180754081578925
node_286: feature_name=GO:0070741 feature_id[538].value <= threshold=1.5534479022026062
node_287: feature_name=GO:0048583 feature_id[373].value > threshold=0.018909001722931862
Class: negative genes

Rules_18 passed counts:92
node_0: feature_name=GO:0042113 feature_id[0].value <= threshold=13.408552169799805
node_1: feature_name=GO:0007568 feature_id[534].value <= threshold=5.0313897132873535
node_2: feature_name=GO:0002705 feature_id[541].value <= threshold=3.200145721435547
node_3: feature_name=GO:1901525 feature_id[576].value <= threshold=0.4466460347175598
node_4: feature_name=GO:0048539 feature_id[319].value <= threshold=3.0399646759033203
node_5: feature_name=GO:0001910 feature_id[385].value <= threshold=3.7437864542007446
node_6: feature_name=GO:0043200 feature_id[706].value <= threshold=9.307284355163574
node_7: feature_name=GO:0001773 feature_id[308].value <= threshold=4.016931533813477
node_8: feature_name=GO:0090116 feature_id[97].value <= threshold=7.99645471572876
node_9: feature_name=GO:0019814 feature_id[189].value <= threshold=4.525782108306885
node_10: feature_name=GO:1902583 feature_id[215].value <= threshold=17.60310649871826

node_11: feature_name=GO:0045429	feature_id[737].value <= threshold=1.6133361458778381
node_12: feature_name=GO:0003720	feature_id[228].value <= threshold=5.519326210021973
node_13: feature_name=GO:0046006	feature_id[364].value <= threshold=4.9985432624816895
node_14: feature_name=GO:0070424	feature_id[254].value <= threshold=6.233297109603882
node_15: feature_name=GO:0009892	feature_id[320].value <= threshold=59.99180793762207
node_16: feature_name=GO:0007064	feature_id[527].value <= threshold=11.57377815246582
node_17: feature_name=GO:0005575	feature_id[17].value <= threshold=15.417460918426514
node_18: feature_name=GO:0043368	feature_id[31].value <= threshold=4.059496641159058
node_19: feature_name=GO:0005164	feature_id[163].value <= threshold=5.793607711791992
node_20: feature_name=GO:0042130	feature_id[16].value <= threshold=5.073179721832275
node_21: feature_name=GO:0010216	feature_id[282].value <= threshold=4.834584474563599
node_22: feature_name=GO:0009628	feature_id[553].value <= threshold=29.314892768859863
node_23: feature_name=GO:0045628	feature_id[749].value <= threshold=3.1001367568969727
node_24: feature_name=GO:0042288	feature_id[100].value <= threshold=5.174932956695557
node_25: feature_name=GO:0002329	feature_id[406].value <= threshold=2.348930835723877
node_26: feature_name=GO:0051246	feature_id[642].value <= threshold=0.6498909294605255
node_27: feature_name=GO:0042162	feature_id[355].value <= threshold=8.05103588104248
node_28: feature_name=hsa04668	feature_id[333].value <= threshold=5.614926338195801
node_29: feature_name=GO:0032200	feature_id[613].value <= threshold=28.735913276672363
node_30: feature_name=hsa04662	feature_id[334].value <= threshold=9.588344097137451
node_31: feature_name=GO:1901992	feature_id[105].value <= threshold=5.291427850723267
node_32: feature_name=GO:0072341	feature_id[144].value <= threshold=6.01465106010437
node_33: feature_name=GO:0047485	feature_id[121].value <= threshold=6.657836437225342
node_34: feature_name=GO:0090594	feature_id[738].value <= threshold=1.8673912286758423
node_35: feature_name=GO:0043525	feature_id[524].value <= threshold=2.881397008895874
node_36: feature_name=GO:0032504	feature_id[244].value <= threshold=17.22207546234131
node_37: feature_name=GO:0051348	feature_id[126].value <= threshold=5.933559417724609
node_38: feature_name=GO:0042522	feature_id[692].value <= threshold=1.78694087266922
node_39: feature_name=GO:0031100	feature_id[73].value <= threshold=3.2312101125717163
node_40: feature_name=GO:0042493	feature_id[149].value <= threshold=19.771170616149902
node_41: feature_name=GO:0001552	feature_id[366].value <= threshold=2.1711617708206177
node_42: feature_name=GO:0046483	feature_id[391].value <= threshold=320.9667053222656
node_43: feature_name=GO:0002439	feature_id[431].value <= threshold=1.832722783088684
node_44: feature_name=GO:0006555	feature_id[514].value <= threshold=5.321640968322754
node_45: feature_name=GO:0070424	feature_id[254].value <= threshold=3.321003556251526

node_46: feature_name=hsa05144 feature_id[751].value <= threshold=2.635499954223633
node_47: feature_name=GO:0032633 feature_id[627].value <= threshold=1.2592533230781555
node_48: feature_name=GO:0019814 feature_id[189].value <= threshold=2.1583125591278076
node_49: feature_name=GO:1901989 feature_id[30].value <= threshold=3.3150794506073
node_50: feature_name=GO:1903318 feature_id[196].value <= threshold=1.62252539396286
node_51: feature_name=GO:0060576 feature_id[204].value <= threshold=2.1097792387008667
node_52: feature_name=hsa05221 feature_id[349].value <= threshold=0.8723124265670776
node_53: feature_name=GO:1903896 feature_id[135].value <= threshold=1.397229254245758
node_54: feature_name=GO:0097028 feature_id[443].value <= threshold=1.2365349531173706
node_55: feature_name=GO:0048660 feature_id[403].value <= threshold=1.9425984621047974
node_56: feature_name=GO:0043011 feature_id[150].value <= threshold=1.3008450269699097
node_57: feature_name=GO:0023056 feature_id[632].value <= threshold=2.5618948936462402
node_58: feature_name=GO:0005164 feature_id[163].value <= threshold=0.9787287414073944
node_59: feature_name=GO:0050707 feature_id[797].value > threshold=0.9616408348083496
node_71: feature_name=GO:0009967 feature_id[672].value > threshold=0.0008242506301030517
Class: negative genes

Rules_19 passed counts:85
node_0: feature_name=GO:0042113 feature_id[0].value <= threshold=13.408552169799805
node_1: feature_name=GO:0007568 feature_id[534].value > threshold=5.0313897132873535
node_913: feature_name=GO:0032763 feature_id[629].value > threshold=0.31753237545490265
node_1195: feature_name=GO:0071301 feature_id[442].value <= threshold=0.11534593254327774
node_1196: feature_name=GO:0046500 feature_id[171].value > threshold=0.6314916908740997
node_1340: feature_name=GO:0043627 feature_id[562].value <= threshold=25.17277240753174
node_1341: feature_name=GO:0010835 feature_id[560].value <= threshold=1.0601619482040405
node_1342: feature_name=GO:0016363 feature_id[90].value <= threshold=6.534365892410278
node_1343: feature_name=GO:0030983 feature_id[291].value <= threshold=3.5806901454925537
node_1344: feature_name=GO:0023030 feature_id[45].value <= threshold=0.6541633009910583
node_1345: feature_name=GO:0070245 feature_id[326].value <= threshold=1.9657342433929443
node_1346: feature_name=GO:0050897 feature_id[164].value <= threshold=1.6622408628463745
node_1347: feature_name=GO:0052548 feature_id[205].value > threshold=0.1624109297990799
node_1349: feature_name=GO:0000082 feature_id[478].value > threshold=0.03540035802870989
node_1351: feature_name=GO:0045556 feature_id[742].value <= threshold=1.6088207960128784
Class: negative genes

Rules_20	passed counts:77
node_0: feature_name=GO:0042113	feature_id[0].value <= threshold=13.408552169799805
node_1: feature_name=GO:0007568	feature_id[534].value > threshold=5.0313897132873535
node_913: feature_name=GO:0032763	feature_id[629].value > threshold=0.31753237545490265
node_1195: feature_name=GO:0071301	feature_id[442].value > threshold=0.11534593254327774
node_1375: feature_name=GO:0046500	feature_id[171].value <= threshold=1.130434513092041
node_1376: feature_name=GO:0001782	feature_id[240].value <= threshold=6.639636993408203
node_1377: feature_name=GO:0043371	feature_id[707].value <= threshold=2.562113642692566
node_1378: feature_name=GO:0030291	feature_id[147].value <= threshold=6.834916830062866
node_1379: feature_name=GO:0045656	feature_id[756].value <= threshold=0.6281269192695618
node_1380: feature_name=GO:0034103	feature_id[318].value > threshold=0.0158024481497705
node_1382: feature_name=GO:0048872	feature_id[498].value <= threshold=19.454792976379395
node_1383: feature_name=GO:0002665	feature_id[453].value <= threshold=1.584219217300415
node_1384: feature_name=GO:0045628	feature_id[749].value <= threshold=3.1363080739974976
node_1385: feature_name=GO:0010639	feature_id[564].value <= threshold=26.597777366638184
Class: negative genes	

Rules_21	passed counts:75
node_0: feature_name=GO:0042113	feature_id[0].value <= threshold=13.408552169799805
node_1: feature_name=GO:0007568	feature_id[534].value <= threshold=5.0313897132873535
node_2: feature_name=GO:0002705	feature_id[541].value <= threshold=3.200145721435547
node_3: feature_name=GO:1901525	feature_id[576].value > threshold=0.4466460347175598
node_617: feature_name=GO:0005622	feature_id[233].value <= threshold=90.85730743408203
node_618: feature_name=GO:1903147	feature_id[200].value <= threshold=7.326428413391113
node_619: feature_name=GO:0003964	feature_id[317].value <= threshold=5.575642108917236
node_620: feature_name=GO:0046498	feature_id[203].value <= threshold=6.620223522186279
node_621: feature_name=GO:0002562	feature_id[25].value <= threshold=10.287704467773438
node_622: feature_name=GO:0006919	feature_id[246].value <= threshold=5.801334381103516
node_623: feature_name=GO:0001889	feature_id[387].value <= threshold=2.679414987564087
node_624: feature_name=GO:0006555	feature_id[514].value <= threshold=7.819535493850708
node_625: feature_name=GO:0080134	feature_id[650].value <= threshold=38.91967582702637
node_626: feature_name=GO:0042127	feature_id[480].value <= threshold=37.72536659240723
node_627: feature_name=GO:2001251	feature_id[220].value <= threshold=32.564422607421875
node_628: feature_name=GO:0002829	feature_id[493].value <= threshold=2.444612741470337
node_629: feature_name=GO:0008588	feature_id[26].value <= threshold=2.3601274490356445

node_630: feature_name=GO:0033151 feature_id[7].value <= threshold=5.134376287460327
node_631: feature_name=GO:0048145 feature_id[348].value <= threshold=8.836549282073975
node_632: feature_name=GO:0030887 feature_id[274].value <= threshold=1.7000296115875244
node_633: feature_name=GO:0097190 feature_id[117].value <= threshold=33.02078819274902
node_634: feature_name=GO:0046685 feature_id[782].value <= threshold=8.040813446044922
node_635: feature_name=GO:0006346 feature_id[236].value <= threshold=6.384642839431763
node_636: feature_name=GO:0048537 feature_id[66].value <= threshold=3.1739262342453003
node_637: feature_name=GO:0070245 feature_id[326].value <= threshold=3.0491198301315308
node_638: feature_name=GO:0038065 feature_id[670].value <= threshold=2.0590850114822388
node_639: feature_name=GO:0005488 feature_id[187].value <= threshold=84.80076217651367
node_640: feature_name=GO:0060249 feature_id[390].value <= threshold=0.07226398587226868
node_641: feature_name=GO:0002683 feature_id[456].value <= threshold=0.7958214282989502
node_642: feature_name=GO:0006346 feature_id[236].value <= threshold=0.4950565919280052
Class: negative genes

Rules_22 passed counts:74
node_0: feature_name=GO:0042113 feature_id[0].value <= threshold=13.408552169799805
node_1: feature_name=GO:0007568 feature_id[534].value > threshold=5.0313897132873535
node_913: feature_name=GO:0032763 feature_id[629].value > threshold=0.31753237545490265
node_1195: feature_name=GO:0071301 feature_id[442].value <= threshold=0.11534593254327774
node_1196: feature_name=GO:0046500 feature_id[171].value <= threshold=0.6314916908740997
node_1197: feature_name=GO:0030852 feature_id[22].value <= threshold=2.9768868684768677
node_1198: feature_name=hsa04672 feature_id[276].value <= threshold=8.06681227684021
node_1199: feature_name=GO:0001836 feature_id[450].value > threshold=0.09961023926734924
node_1201: feature_name=GO:0070230 feature_id[64].value <= threshold=3.3956379890441895
node_1202: feature_name=GO:0070102 feature_id[49].value <= threshold=4.805386543273926
node_1203: feature_name=GO:0045553 feature_id[736].value <= threshold=1.4970332980155945
node_1204: feature_name=GO:0051311 feature_id[58].value <= threshold=3.301889181137085
node_1205: feature_name=GO:0030291 feature_id[147].value <= threshold=8.156315326690674
node_1206: feature_name=GO:0045637 feature_id[241].value > threshold=0.01834342861548066
node_1208: feature_name=GO:0002890 feature_id[98].value <= threshold=2.767483353614807
node_1209: feature_name=GO:0046632 feature_id[248].value <= threshold=11.663254261016846
node_1210: feature_name=GO:0008588 feature_id[26].value <= threshold=1.391375720500946
node_1211: feature_name=GO:0010639 feature_id[564].value <= threshold=18.975126266479492
node_1212: feature_name=GO:0046685 feature_id[782].value <= threshold=5.153738260269165

node_1213: feature_name=GO:0038116 feature_id[668].value <= threshold=1.7859655618667603
node_1214: feature_name=GO:0046449 feature_id[763].value <= threshold=5.603115558624268
node_1215: feature_name=GO:0048539 feature_id[319].value > threshold=0.5478232800960541
node_1269: feature_name=GO:0072341 feature_id[144].value <= threshold=1.3205850720405579
node_1270: feature_name=GO:0010835 feature_id[560].value <= threshold=0.7877185940742493
node_1271: feature_name=GO:0000097 feature_id[376].value <= threshold=0.9960366785526276
node_1272: feature_name=GO:0042509 feature_id[694].value <= threshold=11.929262161254883
node_1273: feature_name=GO:0010216 feature_id[282].value <= threshold=2.920253038406372
node_1274: feature_name=hsa04650 feature_id[13].value > threshold=0.013154025655523982
Class: negative genes

Rules_23 passed counts:73
node_0: feature_name=GO:0042113 feature_id[0].value <= threshold=13.408552169799805
node_1: feature_name=GO:0007568 feature_id[534].value <= threshold=5.0313897132873535
node_2: feature_name=GO:0002705 feature_id[541].value <= threshold=3.200145721435547
node_3: feature_name=GO:1901525 feature_id[576].value > threshold=0.4466460347175598
node_617: feature_name=GO:0005622 feature_id[233].value > threshold=90.85730743408203
node_847: feature_name=GO:0002639 feature_id[466].value <= threshold=0.32853202521800995
node_848: feature_name=GO:0002636 feature_id[447].value <= threshold=0.3303467631340027
node_849: feature_name=GO:0001783 feature_id[33].value > threshold=0.07426292821764946
node_851: feature_name=GO:0032763 feature_id[629].value > threshold=0.21307963132858276
node_853: feature_name=GO:0045404 feature_id[741].value <= threshold=0.4299612492322922
node_854: feature_name=GO:0033158 feature_id[379].value > threshold=0.09315907582640648
node_856: feature_name=GO:0008156 feature_id[239].value > threshold=0.07084834203124046
node_858: feature_name=GO:0038065 feature_id[670].value <= threshold=0.5210016071796417
Class: negative genes

Rules_24 passed counts:73
node_0: feature_name=GO:0042113 feature_id[0].value <= threshold=13.408552169799805
node_1: feature_name=GO:0007568 feature_id[534].value <= threshold=5.0313897132873535
node_2: feature_name=GO:0002705 feature_id[541].value <= threshold=3.200145721435547
node_3: feature_name=GO:1901525 feature_id[576].value <= threshold=0.4466460347175598
node_4: feature_name=GO:0048539 feature_id[319].value <= threshold=3.0399646759033203
node_5: feature_name=GO:0001910 feature_id[385].value <= threshold=3.7437864542007446
node_6: feature_name=GO:0043200 feature_id[706].value <= threshold=9.307284355163574

node_7: feature_name=GO:0001773 feature_id[308].value <= threshold=4.016931533813477
node_8: feature_name=GO:0090116 feature_id[97].value <= threshold=7.99645471572876
node_9: feature_name=GO:0019814 feature_id[189].value <= threshold=4.525782108306885
node_10: feature_name=GO:1902583 feature_id[215].value <= threshold=17.60310649871826
node_11: feature_name=GO:0045429 feature_id[737].value <= threshold=1.6133361458778381
node_12: feature_name=GO:0003720 feature_id[228].value <= threshold=5.519326210021973
node_13: feature_name=GO:0046006 feature_id[364].value <= threshold=4.9985432624816895
node_14: feature_name=GO:0070424 feature_id[254].value <= threshold=6.233297109603882
node_15: feature_name=GO:0009892 feature_id[320].value <= threshold=59.99180793762207
node_16: feature_name=GO:0007064 feature_id[527].value <= threshold=11.57377815246582
node_17: feature_name=GO:0005575 feature_id[17].value <= threshold=15.417460918426514
node_18: feature_name=GO:0043368 feature_id[31].value <= threshold=4.059496641159058
node_19: feature_name=GO:0005164 feature_id[163].value <= threshold=5.793607711791992
node_20: feature_name=GO:0042130 feature_id[16].value <= threshold=5.073179721832275
node_21: feature_name=GO:0010216 feature_id[282].value <= threshold=4.834584474563599
node_22: feature_name=GO:0009628 feature_id[553].value <= threshold=29.314892768859863
node_23: feature_name=GO:0045628 feature_id[749].value <= threshold=3.1001367568969727
node_24: feature_name=GO:0042288 feature_id[100].value <= threshold=5.174932956695557
node_25: feature_name=GO:0002329 feature_id[406].value <= threshold=2.348930835723877
node_26: feature_name=GO:0051246 feature_id[642].value <= threshold=0.6498909294605255
node_27: feature_name=GO:0042162 feature_id[355].value <= threshold=8.05103588104248
node_28: feature_name=hsa04668 feature_id[333].value <= threshold=5.614926338195801
node_29: feature_name=GO:0032200 feature_id[613].value <= threshold=28.735913276672363
node_30: feature_name=hsa04662 feature_id[334].value <= threshold=9.588344097137451
node_31: feature_name=GO:1901992 feature_id[105].value <= threshold=5.291427850723267
node_32: feature_name=GO:0072341 feature_id[144].value <= threshold=6.01465106010437
node_33: feature_name=GO:0047485 feature_id[121].value <= threshold=6.657836437225342
node_34: feature_name=GO:0090594 feature_id[738].value <= threshold=1.8673912286758423
node_35: feature_name=GO:0043525 feature_id[524].value <= threshold=2.881397008895874
node_36: feature_name=GO:0032504 feature_id[244].value <= threshold=17.22207546234131
node_37: feature_name=GO:0051348 feature_id[126].value <= threshold=5.933559417724609
node_38: feature_name=GO:0042522 feature_id[692].value <= threshold=1.78694087266922
node_39: feature_name=GO:0031100 feature_id[73].value <= threshold=3.2312101125717163
node_40: feature_name=GO:0042493 feature_id[149].value <= threshold=19.771170616149902
node_41: feature_name=GO:0001552 feature_id[366].value <= threshold=2.1711617708206177

node_42: feature_name=GO:0046483 feature_id[391].value <= threshold=320.9667053222656
node_43: feature_name=GO:0002439 feature_id[431].value <= threshold=1.832722783088684
node_44: feature_name=GO:0006555 feature_id[514].value <= threshold=5.321640968322754
node_45: feature_name=GO:0070424 feature_id[254].value <= threshold=3.321003556251526
node_46: feature_name=hsa05144 feature_id[751].value <= threshold=2.635499954223633
node_47: feature_name=GO:0032633 feature_id[627].value > threshold=1.2592533230781555
node_125: feature_name=GO:0033158 feature_id[379].value <= threshold=0.9298970401287079
node_126: feature_name=GO:0051246 feature_id[642].value > threshold=5.402702754508937e-06
Class: negative genes

Rules_25 passed counts:69
node_0: feature_name=GO:0042113 feature_id[0].value <= threshold=13.408552169799805
node_1: feature_name=GO:0007568 feature_id[534].value > threshold=5.0313897132873535
node_913: feature_name=GO:0032763 feature_id[629].value > threshold=0.31753237545490265
node_1195: feature_name=GO:0071301 feature_id[442].value > threshold=0.11534593254327774
node_1375: feature_name=GO:0046500 feature_id[171].value <= threshold=1.130434513092041
node_1376: feature_name=GO:0001782 feature_id[240].value <= threshold=6.639636993408203
node_1377: feature_name=GO:0043371 feature_id[707].value <= threshold=2.562113642692566
node_1378: feature_name=GO:0030291 feature_id[147].value <= threshold=6.834916830062866
node_1379: feature_name=GO:0045656 feature_id[756].value > threshold=0.6281269192695618
node_1393: feature_name=GO:0046007 feature_id[152].value <= threshold=1.020785927772522
node_1394: feature_name=GO:0000302 feature_id[375].value <= threshold=20.847331047058105
node_1395: feature_name=hsa04110 feature_id[328].value <= threshold=43.297359466552734
node_1396: feature_name=GO:0050731 feature_id[804].value <= threshold=15.756641864776611
node_1397: feature_name=GO:0002440 feature_id[429].value > threshold=0.4019751101732254
node_1403: feature_name=GO:0051348 feature_id[126].value > threshold=0.8975991010665894
node_1405: feature_name=GO:0009636 feature_id[551].value > threshold=1.019210398197174
node_1407: feature_name=GO:0045636 feature_id[743].value <= threshold=4.099599123001099
Class: negative genes

Rules_26 passed counts:66
node_0: feature_name=GO:0042113 feature_id[0].value <= threshold=13.408552169799805
node_1: feature_name=GO:0007568 feature_id[534].value <= threshold=5.0313897132873535
node_2: feature_name=GO:0002705 feature_id[541].value <= threshold=3.200145721435547
node_3: feature_name=GO:1901525 feature_id[576].value <= threshold=0.4466460347175598

node_4: feature_name=GO:0048539	feature_id[319].value <= threshold=3.0399646759033203
node_5: feature_name=GO:0001910	feature_id[385].value <= threshold=3.7437864542007446
node_6: feature_name=GO:0043200	feature_id[706].value <= threshold=9.307284355163574
node_7: feature_name=GO:0001773	feature_id[308].value <= threshold=4.016931533813477
node_8: feature_name=GO:0090116	feature_id[97].value <= threshold=7.99645471572876
node_9: feature_name=GO:0019814	feature_id[189].value <= threshold=4.525782108306885
node_10: feature_name=GO:1902583	feature_id[215].value <= threshold=17.60310649871826
node_11: feature_name=GO:0045429	feature_id[737].value > threshold=1.6133361458778381
node_477: feature_name=GO:0036037	feature_id[445].value <= threshold=1.7181594371795654
node_478: feature_name=GO:0006244	feature_id[503].value <= threshold=0.951388418674469
node_479: feature_name=GO:0032461	feature_id[608].value <= threshold=2.6103241443634033
node_480: feature_name=GO:0044710	feature_id[719].value > threshold=2.228096718681627e-06
node_494: feature_name=GO:0009314	feature_id[296].value <= threshold=34.93696117401123
node_495: feature_name=GO:0050778	feature_id[802].value <= threshold=39.29364013671875
node_496: feature_name=GO:1903038	feature_id[492].value <= threshold=5.849650859832764
node_497: feature_name=GO:0002699	feature_id[606].value <= threshold=5.5405943393707275
node_498: feature_name=hsa00983	feature_id[181].value <= threshold=25.851045608520508
node_499: feature_name=GO:0002309	feature_id[416].value <= threshold=1.6235689520835876
node_500: feature_name=GO:1901698	feature_id[792].value <= threshold=47.70275688171387
node_501: feature_name=GO:0010216	feature_id[282].value <= threshold=1.6849713921546936
node_502: feature_name=GO:0006266	feature_id[297].value <= threshold=2.7199249267578125
node_503: feature_name=GO:0005488	feature_id[187].value > threshold=3.0373169010999845e-06
node_507: feature_name=GO:0045429	feature_id[737].value > threshold=1.614579975605011
node_511: feature_name=GO:0034103	feature_id[318].value <= threshold=5.832815647125244
node_512: feature_name=GO:0010663	feature_id[561].value <= threshold=4.157005429267883
node_513: feature_name=GO:0030887	feature_id[274].value <= threshold=1.8964150547981262
node_514: feature_name=GO:0048294	feature_id[113].value <= threshold=1.866134524345398
node_515: feature_name=GO:0072539	feature_id[68].value > threshold=0.8600535988807678
node_547: feature_name=GO:0002718	feature_id[481].value <= threshold=4.013465642929077
node_548: feature_name=GO:0072539	feature_id[68].value > threshold=0.8829970061779022
node_550: feature_name=GO:0055064	feature_id[299].value <= threshold=1.2614808082580566
node_551: feature_name=GO:0000307	feature_id[139].value <= threshold=2.679197907447815
node_552: feature_name=GO:0046631	feature_id[86].value > threshold=0.2878068685531616

Class: negative genes

Rules_27	passed counts:62
node_0: feature_name=GO:0042113	feature_id[0].value <= threshold=13.408552169799805
node_1: feature_name=GO:0007568	feature_id[534].value <= threshold=5.0313897132873535
node_2: feature_name=GO:0002705	feature_id[541].value <= threshold=3.200145721435547
node_3: feature_name=GO:1901525	feature_id[576].value <= threshold=0.4466460347175598
node_4: feature_name=GO:0048539	feature_id[319].value <= threshold=3.0399646759033203
node_5: feature_name=GO:0001910	feature_id[385].value <= threshold=3.7437864542007446
node_6: feature_name=GO:0043200	feature_id[706].value <= threshold=9.307284355163574
node_7: feature_name=GO:0001773	feature_id[308].value <= threshold=4.016931533813477
node_8: feature_name=GO:0090116	feature_id[97].value <= threshold=7.99645471572876
node_9: feature_name=GO:0019814	feature_id[189].value <= threshold=4.525782108306885
node_10: feature_name=GO:1902583	feature_id[215].value <= threshold=17.60310649871826
node_11: feature_name=GO:0045429	feature_id[737].value <= threshold=1.6133361458778381
node_12: feature_name=GO:0003720	feature_id[228].value <= threshold=5.519326210021973
node_13: feature_name=GO:0046006	feature_id[364].value <= threshold=4.9985432624816895
node_14: feature_name=GO:0070424	feature_id[254].value <= threshold=6.233297109603882
node_15: feature_name=GO:0009892	feature_id[320].value <= threshold=59.99180793762207
node_16: feature_name=GO:0007064	feature_id[527].value <= threshold=11.57377815246582
node_17: feature_name=GO:0005575	feature_id[17].value <= threshold=15.417460918426514
node_18: feature_name=GO:0043368	feature_id[31].value <= threshold=4.059496641159058
node_19: feature_name=GO:0005164	feature_id[163].value <= threshold=5.793607711791992
node_20: feature_name=GO:0042130	feature_id[16].value <= threshold=5.073179721832275
node_21: feature_name=GO:0010216	feature_id[282].value <= threshold=4.834584474563599
node_22: feature_name=GO:0009628	feature_id[553].value <= threshold=29.314892768859863
node_23: feature_name=GO:0045628	feature_id[749].value <= threshold=3.1001367568969727
node_24: feature_name=GO:0042288	feature_id[100].value <= threshold=5.174932956695557
node_25: feature_name=GO:0002329	feature_id[406].value <= threshold=2.348930835723877
node_26: feature_name=GO:0051246	feature_id[642].value > threshold=0.6498909294605255
node_194: feature_name=GO:0009164	feature_id[546].value <= threshold=17.11970329284668
node_195: feature_name=GO:0001909	feature_id[386].value <= threshold=7.292566299438477
node_196: feature_name=GO:0003908	feature_id[11].value <= threshold=1.887226164340973
node_197: feature_name=GO:0009164	feature_id[546].value <= threshold=0.26642198860645294
node_198: feature_name=GO:2001242	feature_id[24].value <= threshold=9.207026481628418
node_199: feature_name=GO:0051246	feature_id[642].value > threshold=0.6504445374011993
node_203: feature_name=GO:0032461	feature_id[608].value <= threshold=2.607542634010315

node_204: feature_name=GO:0007584 feature_id[529].value <= threshold=16.099515914916992
node_205: feature_name=hsa05221 feature_id[349].value <= threshold=9.359850406646729
node_206: feature_name=GO:0002636 feature_id[447].value <= threshold=1.615447759628296
node_207: feature_name=GO:0033993 feature_id[421].value > threshold=2.96540611088858e-05
node_217: feature_name=GO:0002331 feature_id[404].value <= threshold=2.1883513927459717
node_218: feature_name=GO:0046498 feature_id[203].value <= threshold=4.085246205329895
node_219: feature_name=GO:0002832 feature_id[491].value <= threshold=3.0228612422943115
node_220: feature_name=GO:1904868 feature_id[306].value <= threshold=3.8674756288528442
node_221: feature_name=GO:0048545 feature_id[360].value <= threshold=27.38136100769043
node_222: feature_name=GO:0071310 feature_id[760].value <= threshold=2.3522024154663086
node_223: feature_name=GO:0001775 feature_id[505].value <= threshold=9.71301555633545
node_224: feature_name=GO:0071310 feature_id[760].value <= threshold=2.346743583679199
node_225: feature_name=GO:0001772 feature_id[91].value <= threshold=3.252573609352112
node_226: feature_name=GO:0036498 feature_id[359].value <= threshold=12.092026710510254
node_227: feature_name=hsa05340 feature_id[351].value <= threshold=0.7677814364433289
node_228: feature_name=GO:0032703 feature_id[617].value <= threshold=1.7450646758079529
node_229: feature_name=hsa04640 feature_id[79].value <= threshold=2.118402123451233
node_230: feature_name=GO:0042097 feature_id[675].value <= threshold=1.8110727667808533
node_231: feature_name=GO:0071391 feature_id[518].value <= threshold=2.691588521003723
node_232: feature_name=GO:0001666 feature_id[682].value <= threshold=5.225740432739258
node_233: feature_name=GO:0048539 feature_id[319].value <= threshold=1.8523842096328735
node_234: feature_name=GO:0038061 feature_id[671].value <= threshold=8.564527988433838
node_235: feature_name=GO:0002484 feature_id[600].value <= threshold=3.1121134757995605
node_236: feature_name=GO:0023023 feature_id[119].value <= threshold=2.868617057800293
node_237: feature_name=GO:0010639 feature_id[564].value <= threshold=13.410505771636963
node_238: feature_name=GO:0080134 feature_id[650].value > threshold=0.013442101422697306
node_244: feature_name=hsa04060 feature_id[331].value > threshold=0.7175639569759369
node_246: feature_name=hsa04060 feature_id[331].value > threshold=0.7203521430492401
node_248: feature_name=hsa04110 feature_id[328].value <= threshold=2.0177602767944336
Class: negative genes

Rules_28 passed counts:61
node_0: feature_name=GO:0042113 feature_id[0].value <= threshold=13.408552169799805
node_1: feature_name=GO:0007568 feature_id[534].value <= threshold=5.0313897132873535
node_2: feature_name=GO:0002705 feature_id[541].value <= threshold=3.200145721435547

node_3: feature_name=GO:1901525	feature_id[576].value <= threshold=0.4466460347175598
node_4: feature_name=GO:0048539	feature_id[319].value <= threshold=3.0399646759033203
node_5: feature_name=GO:0001910	feature_id[385].value <= threshold=3.7437864542007446
node_6: feature_name=GO:0043200	feature_id[706].value <= threshold=9.307284355163574
node_7: feature_name=GO:0001773	feature_id[308].value <= threshold=4.016931533813477
node_8: feature_name=GO:0090116	feature_id[97].value <= threshold=7.99645471572876
node_9: feature_name=GO:0019814	feature_id[189].value <= threshold=4.525782108306885
node_10: feature_name=GO:1902583	feature_id[215].value <= threshold=17.60310649871826
node_11: feature_name=GO:0045429	feature_id[737].value <= threshold=1.6133361458778381
node_12: feature_name=GO:0003720	feature_id[228].value <= threshold=5.519326210021973
node_13: feature_name=GO:0046006	feature_id[364].value <= threshold=4.9985432624816895
node_14: feature_name=GO:0070424	feature_id[254].value <= threshold=6.233297109603882
node_15: feature_name=GO:0009892	feature_id[320].value <= threshold=59.99180793762207
node_16: feature_name=GO:0007064	feature_id[527].value <= threshold=11.57377815246582
node_17: feature_name=GO:0005575	feature_id[17].value <= threshold=15.417460918426514
node_18: feature_name=GO:0043368	feature_id[31].value <= threshold=4.059496641159058
node_19: feature_name=GO:0005164	feature_id[163].value <= threshold=5.793607711791992
node_20: feature_name=GO:0042130	feature_id[16].value <= threshold=5.073179721832275
node_21: feature_name=GO:0010216	feature_id[282].value <= threshold=4.834584474563599
node_22: feature_name=GO:0009628	feature_id[553].value <= threshold=29.314892768859863
node_23: feature_name=GO:0045628	feature_id[749].value <= threshold=3.1001367568969727
node_24: feature_name=GO:0042288	feature_id[100].value <= threshold=5.174932956695557
node_25: feature_name=GO:0002329	feature_id[406].value <= threshold=2.348930835723877
node_26: feature_name=GO:0051246	feature_id[642].value <= threshold=0.6498909294605255
node_27: feature_name=GO:0042162	feature_id[355].value <= threshold=8.05103588104248
node_28: feature_name=hsa04668	feature_id[333].value <= threshold=5.614926338195801
node_29: feature_name=GO:0032200	feature_id[613].value <= threshold=28.735913276672363
node_30: feature_name=hsa04662	feature_id[334].value <= threshold=9.588344097137451
node_31: feature_name=GO:1901992	feature_id[105].value <= threshold=5.291427850723267
node_32: feature_name=GO:0072341	feature_id[144].value <= threshold=6.01465106010437
node_33: feature_name=GO:0047485	feature_id[121].value <= threshold=6.657836437225342
node_34: feature_name=GO:0090594	feature_id[738].value <= threshold=1.8673912286758423
node_35: feature_name=GO:0043525	feature_id[524].value <= threshold=2.881397008895874
node_36: feature_name=GO:0032504	feature_id[244].value <= threshold=17.22207546234131
node_37: feature_name=GO:0051348	feature_id[126].value <= threshold=5.933559417724609

node_38: feature_name=GO:0042522	feature_id[692].value <= threshold=1.78694087266922
node_39: feature_name=GO:0031100	feature_id[73].value <= threshold=3.2312101125717163
node_40: feature_name=GO:0042493	feature_id[149].value <= threshold=19.771170616149902
node_41: feature_name=GO:0001552	feature_id[366].value <= threshold=2.1711617708206177
node_42: feature_name=GO:0046483	feature_id[391].value <= threshold=320.9667053222656
node_43: feature_name=GO:0002439	feature_id[431].value <= threshold=1.832722783088684
node_44: feature_name=GO:0006555	feature_id[514].value <= threshold=5.321640968322754
node_45: feature_name=GO:0070424	feature_id[254].value <= threshold=3.321003556251526
node_46: feature_name=hsa05144	feature_id[751].value <= threshold=2.635499954223633
node_47: feature_name=GO:0032633	feature_id[627].value <= threshold=1.2592533230781555
node_48: feature_name=GO:0019814	feature_id[189].value <= threshold=2.1583125591278076
node_49: feature_name=GO:1901989	feature_id[30].value <= threshold=3.3150794506073
node_50: feature_name=GO:1903318	feature_id[196].value <= threshold=1.62252539396286
node_51: feature_name=GO:0060576	feature_id[204].value <= threshold=2.1097792387008667
node_52: feature_name=hsa05221	feature_id[349].value <= threshold=0.8723124265670776
node_53: feature_name=GO:1903896	feature_id[135].value <= threshold=1.397229254245758
node_54: feature_name=GO:0097028	feature_id[443].value <= threshold=1.2365349531173706
node_55: feature_name=GO:0048660	feature_id[403].value <= threshold=1.9425984621047974
node_56: feature_name=GO:0043011	feature_id[150].value <= threshold=1.3008450269699097
node_57: feature_name=GO:0023056	feature_id[632].value > threshold=2.5618948936462402
node_79: feature_name=GO:0048583	feature_id[373].value > threshold=0.21299096941947937

Class: negative genes

Rules_29	passed counts:59
node_0: feature_name=GO:0042113	feature_id[0].value <= threshold=13.408552169799805
node_1: feature_name=GO:0007568	feature_id[534].value <= threshold=5.0313897132873535
node_2: feature_name=GO:0002705	feature_id[541].value <= threshold=3.200145721435547
node_3: feature_name=GO:1901525	feature_id[576].value <= threshold=0.4466460347175598
node_4: feature_name=GO:0048539	feature_id[319].value <= threshold=3.0399646759033203
node_5: feature_name=GO:0001910	feature_id[385].value <= threshold=3.7437864542007446
node_6: feature_name=GO:0043200	feature_id[706].value <= threshold=9.307284355163574
node_7: feature_name=GO:0001773	feature_id[308].value <= threshold=4.016931533813477
node_8: feature_name=GO:0090116	feature_id[97].value <= threshold=7.99645471572876
node_9: feature_name=GO:0019814	feature_id[189].value <= threshold=4.525782108306885
node_10: feature_name=GO:1902583	feature_id[215].value <= threshold=17.60310649871826

node_11: feature_name=GO:0045429 feature_id[737].value <= threshold=1.6133361458778381
node_12: feature_name=GO:0003720 feature_id[228].value <= threshold=5.519326210021973
node_13: feature_name=GO:0046006 feature_id[364].value <= threshold=4.9985432624816895
node_14: feature_name=GO:0070424 feature_id[254].value <= threshold=6.233297109603882
node_15: feature_name=GO:0009892 feature_id[320].value <= threshold=59.99180793762207
node_16: feature_name=GO:0007064 feature_id[527].value <= threshold=11.57377815246582
node_17: feature_name=GO:0005575 feature_id[17].value <= threshold=15.417460918426514
node_18: feature_name=GO:0043368 feature_id[31].value <= threshold=4.059496641159058
node_19: feature_name=GO:0005164 feature_id[163].value <= threshold=5.793607711791992
node_20: feature_name=GO:0042130 feature_id[16].value <= threshold=5.073179721832275
node_21: feature_name=GO:0010216 feature_id[282].value <= threshold=4.834584474563599
node_22: feature_name=GO:0009628 feature_id[553].value <= threshold=29.314892768859863
node_23: feature_name=GO:0045628 feature_id[749].value <= threshold=3.1001367568969727
node_24: feature_name=GO:0042288 feature_id[100].value <= threshold=5.174932956695557
node_25: feature_name=GO:0002329 feature_id[406].value <= threshold=2.348930835723877
node_26: feature_name=GO:0051246 feature_id[642].value > threshold=0.6498909294605255
node_194: feature_name=GO:0009164 feature_id[546].value <= threshold=17.11970329284668
node_195: feature_name=GO:0001909 feature_id[386].value <= threshold=7.292566299438477
node_196: feature_name=GO:0003908 feature_id[11].value <= threshold=1.887226164340973
node_197: feature_name=GO:0009164 feature_id[546].value <= threshold=0.26642198860645294
node_198: feature_name=GO:2001242 feature_id[24].value <= threshold=9.207026481628418
node_199: feature_name=GO:0051246 feature_id[642].value > threshold=0.6504445374011993
node_203: feature_name=GO:0032461 feature_id[608].value <= threshold=2.607542634010315
node_204: feature_name=GO:0007584 feature_id[529].value <= threshold=16.099515914916992
node_205: feature_name=hsa05221 feature_id[349].value > threshold=9.359850406646729
node_337: feature_name=GO:0032743 feature_id[258].value <= threshold=2.931445837020874
node_338: feature_name=GO:0002460 feature_id[357].value > threshold=0.0003823822917183861
node_340: feature_name=GO:0019740 feature_id[581].value <= threshold=1.0357294380664825
node_341: feature_name=hsa05221 feature_id[349].value > threshold=9.390207767486572
node_343: feature_name=GO:0045629 feature_id[746].value <= threshold=1.07101109623909
Class: negative genes

Rules_30 passed counts:59
node_0: feature_name=GO:0042113 feature_id[0].value <= threshold=13.408552169799805
node_1: feature_name=GO:0007568 feature_id[534].value <= threshold=5.0313897132873535

node_2: feature_name=GO:0002705 feature_id[541].value <= threshold=3.200145721435547
node_3: feature_name=GO:1901525 feature_id[576].value <= threshold=0.4466460347175598
node_4: feature_name=GO:0048539 feature_id[319].value <= threshold=3.0399646759033203
node_5: feature_name=GO:0001910 feature_id[385].value <= threshold=3.7437864542007446
node_6: feature_name=GO:0043200 feature_id[706].value <= threshold=9.307284355163574
node_7: feature_name=GO:0001773 feature_id[308].value <= threshold=4.016931533813477
node_8: feature_name=GO:0090116 feature_id[97].value <= threshold=7.99645471572876
node_9: feature_name=GO:0019814 feature_id[189].value <= threshold=4.525782108306885
node_10: feature_name=GO:1902583 feature_id[215].value <= threshold=17.60310649871826
node_11: feature_name=GO:0045429 feature_id[737].value <= threshold=1.6133361458778381
node_12: feature_name=GO:0003720 feature_id[228].value <= threshold=5.519326210021973
node_13: feature_name=GO:0046006 feature_id[364].value <= threshold=4.9985432624816895
node_14: feature_name=GO:0070424 feature_id[254].value <= threshold=6.233297109603882
node_15: feature_name=GO:0009892 feature_id[320].value <= threshold=59.99180793762207
node_16: feature_name=GO:0007064 feature_id[527].value <= threshold=11.57377815246582
node_17: feature_name=GO:0005575 feature_id[17].value <= threshold=15.417460918426514
node_18: feature_name=GO:0043368 feature_id[31].value <= threshold=4.059496641159058
node_19: feature_name=GO:0005164 feature_id[163].value <= threshold=5.793607711791992
node_20: feature_name=GO:0042130 feature_id[16].value <= threshold=5.073179721832275
node_21: feature_name=GO:0010216 feature_id[282].value <= threshold=4.834584474563599
node_22: feature_name=GO:0009628 feature_id[553].value <= threshold=29.314892768859863
node_23: feature_name=GO:0045628 feature_id[749].value <= threshold=3.1001367568969727
node_24: feature_name=GO:0042288 feature_id[100].value <= threshold=5.174932956695557
node_25: feature_name=GO:0002329 feature_id[406].value <= threshold=2.348930835723877
node_26: feature_name=GO:0051246 feature_id[642].value <= threshold=0.6498909294605255
node_27: feature_name=GO:0042162 feature_id[355].value <= threshold=8.05103588104248
node_28: feature_name=hsa04668 feature_id[333].value <= threshold=5.614926338195801
node_29: feature_name=GO:0032200 feature_id[613].value <= threshold=28.735913276672363
node_30: feature_name=hsa04662 feature_id[334].value <= threshold=9.588344097137451
node_31: feature_name=GO:1901992 feature_id[105].value <= threshold=5.291427850723267
node_32: feature_name=GO:0072341 feature_id[144].value <= threshold=6.01465106010437
node_33: feature_name=GO:0047485 feature_id[121].value <= threshold=6.657836437225342
node_34: feature_name=GO:0090594 feature_id[738].value <= threshold=1.8673912286758423
node_35: feature_name=GO:0043525 feature_id[524].value <= threshold=2.881397008895874
node_36: feature_name=GO:0032504 feature_id[244].value <= threshold=17.22207546234131

node_37: feature_name=GO:0051348 feature_id[126].value <= threshold=5.933559417724609
node_38: feature_name=GO:0042522 feature_id[692].value <= threshold=1.78694087266922
node_39: feature_name=GO:0031100 feature_id[73].value <= threshold=3.2312101125717163
node_40: feature_name=GO:0042493 feature_id[149].value <= threshold=19.771170616149902
node_41: feature_name=GO:0001552 feature_id[366].value <= threshold=2.1711617708206177
node_42: feature_name=GO:0046483 feature_id[391].value <= threshold=320.9667053222656
node_43: feature_name=GO:0002439 feature_id[431].value <= threshold=1.832722783088684
node_44: feature_name=GO:0006555 feature_id[514].value <= threshold=5.321640968322754
node_45: feature_name=GO:0070424 feature_id[254].value <= threshold=3.321003556251526
node_46: feature_name=hsa05144 feature_id[751].value <= threshold=2.635499954223633
node_47: feature_name=GO:0032633 feature_id[627].value <= threshold=1.2592533230781555
node_48: feature_name=GO:0019814 feature_id[189].value <= threshold=2.1583125591278076
node_49: feature_name=GO:1901989 feature_id[30].value <= threshold=3.3150794506073
node_50: feature_name=GO:1903318 feature_id[196].value <= threshold=1.62252539396286
node_51: feature_name=GO:0060576 feature_id[204].value <= threshold=2.1097792387008667
node_52: feature_name=hsa05221 feature_id[349].value <= threshold=0.8723124265670776
node_53: feature_name=GO:1903896 feature_id[135].value <= threshold=1.397229254245758
node_54: feature_name=GO:0097028 feature_id[443].value <= threshold=1.2365349531173706
node_55: feature_name=GO:0048660 feature_id[403].value <= threshold=1.9425984621047974
node_56: feature_name=GO:0043011 feature_id[150].value <= threshold=1.3008450269699097
node_57: feature_name=GO:0023056 feature_id[632].value <= threshold=2.5618948936462402
node_58: feature_name=GO:0005164 feature_id[163].value > threshold=0.9787287414073944
node_74: feature_name=GO:0015671 feature_id[103].value <= threshold=1.0425233840942383
Class: negative genes

Rules_31 passed counts:58
node_0: feature_name=GO:0042113 feature_id[0].value <= threshold=13.408552169799805
node_1: feature_name=GO:0007568 feature_id[534].value > threshold=5.0313897132873535
node_913: feature_name=GO:0032763 feature_id[629].value <= threshold=0.31753237545490265
node_914: feature_name=GO:0097193 feature_id[167].value <= threshold=28.171168327331543
node_915: feature_name=GO:0002903 feature_id[10].value > threshold=1.0534588098526
node_1159: feature_name=GO:0070198 feature_id[665].value <= threshold=0.16219981759786606
node_1160: feature_name=GO:0019692 feature_id[584].value <= threshold=1.4449326992034912
node_1161: feature_name=GO:0019814 feature_id[189].value <= threshold=3.0963553190231323
Class: negative genes

Rules_32 passed counts:58
node_0: feature_name=GO:0042113 feature_id[0].value <= threshold=13.408552169799805
node_1: feature_name=GO:0007568 feature_id[534].value <= threshold=5.0313897132873535
node_2: feature_name=GO:0002705 feature_id[541].value <= threshold=3.200145721435547
node_3: feature_name=GO:1901525 feature_id[576].value <= threshold=0.4466460347175598
node_4: feature_name=GO:0048539 feature_id[319].value <= threshold=3.0399646759033203
node_5: feature_name=GO:0001910 feature_id[385].value <= threshold=3.7437864542007446
node_6: feature_name=GO:0043200 feature_id[706].value <= threshold=9.307284355163574
node_7: feature_name=GO:0001773 feature_id[308].value <= threshold=4.016931533813477
node_8: feature_name=GO:0090116 feature_id[97].value <= threshold=7.99645471572876
node_9: feature_name=GO:0019814 feature_id[189].value <= threshold=4.525782108306885
node_10: feature_name=GO:1902583 feature_id[215].value <= threshold=17.60310649871826
node_11: feature_name=GO:0045429 feature_id[737].value > threshold=1.6133361458778381
node_477: feature_name=GO:0036037 feature_id[445].value <= threshold=1.7181594371795654
node_478: feature_name=GO:0006244 feature_id[503].value <= threshold=0.951388418674469
node_479: feature_name=GO:0032461 feature_id[608].value <= threshold=2.6103241443634033
node_480: feature_name=GO:0044710 feature_id[719].value <= threshold=2.228096718681627e-06
node_481: feature_name=GO:1904035 feature_id[654].value > threshold=0.05152595415711403
node_487: feature_name=GO:0046633 feature_id[115].value <= threshold=0.7000146508216858
node_488: feature_name=GO:0060576 feature_id[204].value <= threshold=0.8788235783576965
node_489: feature_name=GO:0002694 feature_id[469].value <= threshold=3.035288095474243
Class: negative genes

Rules_33 passed counts:56
node_0: feature_name=GO:0042113 feature_id[0].value > threshold=13.408552169799805
node_1454: feature_name=GO:0050851 feature_id[29].value <= threshold=9.650307655334473
node_1455: feature_name=GO:0006304 feature_id[510].value <= threshold=5.960662126541138
node_1456: feature_name=GO:0032673 feature_id[623].value <= threshold=4.092082738876343
node_1457: feature_name=GO:0002429 feature_id[748].value > threshold=4.626799821853638
node_1463: feature_name=GO:0015672 feature_id[573].value > threshold=0.08226438239216805
node_1475: feature_name=GO:0030217 feature_id[589].value > threshold=19.77315902709961
node_1497: feature_name=GO:0030291 feature_id[147].value <= threshold=4.140083312988281
node_1498: feature_name=GO:0050798 feature_id[170].value <= threshold=12.055219173431396
node_1499: feature_name=GO:2000773 feature_id[114].value <= threshold=7.385613679885864

node_1500: feature_name=GO:0007050 feature_id[398].value <= threshold=23.01229476928711
Class: negative genes

Rules_34 passed counts:56
node_0: feature_name=GO:0042113 feature_id[0].value <= threshold=13.408552169799805
node_1: feature_name=GO:0007568 feature_id[534].value <= threshold=5.0313897132873535
node_2: feature_name=GO:0002705 feature_id[541].value <= threshold=3.200145721435547
node_3: feature_name=GO:1901525 feature_id[576].value > threshold=0.4466460347175598
node_617: feature_name=GO:0005622 feature_id[233].value <= threshold=90.85730743408203
node_618: feature_name=GO:1903147 feature_id[200].value <= threshold=7.326428413391113
node_619: feature_name=GO:0003964 feature_id[317].value <= threshold=5.575642108917236
node_620: feature_name=GO:0046498 feature_id[203].value <= threshold=6.620223522186279
node_621: feature_name=GO:0002562 feature_id[25].value <= threshold=10.287704467773438
node_622: feature_name=GO:0006919 feature_id[246].value <= threshold=5.801334381103516
node_623: feature_name=GO:0001889 feature_id[387].value <= threshold=2.679414987564087
node_624: feature_name=GO:0006555 feature_id[514].value <= threshold=7.819535493850708
node_625: feature_name=GO:0080134 feature_id[650].value <= threshold=38.91967582702637
node_626: feature_name=GO:0042127 feature_id[480].value <= threshold=37.72536659240723
node_627: feature_name=GO:2001251 feature_id[220].value <= threshold=32.564422607421875
node_628: feature_name=GO:0002829 feature_id[493].value <= threshold=2.444612741470337
node_629: feature_name=GO:0008588 feature_id[26].value <= threshold=2.3601274490356445
node_630: feature_name=GO:0033151 feature_id[7].value <= threshold=5.134376287460327
node_631: feature_name=GO:0048145 feature_id[348].value <= threshold=8.836549282073975
node_632: feature_name=GO:0030887 feature_id[274].value <= threshold=1.7000296115875244
node_633: feature_name=GO:0097190 feature_id[117].value <= threshold=33.02078819274902
node_634: feature_name=GO:0046685 feature_id[782].value <= threshold=8.040813446044922
node_635: feature_name=GO:0006346 feature_id[236].value <= threshold=6.384642839431763
node_636: feature_name=GO:0048537 feature_id[66].value <= threshold=3.1739262342453003
node_637: feature_name=GO:0070245 feature_id[326].value <= threshold=3.0491198301315308
node_638: feature_name=GO:0038065 feature_id[670].value <= threshold=2.0590850114822388
node_639: feature_name=GO:0005488 feature_id[187].value <= threshold=84.80076217651367
node_640: feature_name=GO:0060249 feature_id[390].value > threshold=0.07226398587226868
node_652: feature_name=GO:0006974 feature_id[516].value > threshold=5.17782768838515e-06
node_654: feature_name=hsa05210 feature_id[358].value <= threshold=18.730005264282227
node_655: feature_name=GO:0071850 feature_id[40].value <= threshold=5.475317001342773

node_656: feature_name=GO:1904029 feature_id[208].value <= threshold=26.575013160705566
node_657: feature_name=GO:0090594 feature_id[738].value <= threshold=3.000791072845459
node_658: feature_name=GO:0010948 feature_id[569].value > threshold=0.04386143572628498
node_666: feature_name=GO:0032764 feature_id[3].value <= threshold=2.5201677083969116
node_667: feature_name=GO:0023026 feature_id[69].value <= threshold=3.2760006189346313
node_668: feature_name=GO:0032464 feature_id[83].value <= threshold=2.5927021503448486
node_669: feature_name=GO:0071901 feature_id[407].value <= threshold=18.238012313842773
node_670: feature_name=GO:0015671 feature_id[103].value > threshold=1.3113192915916443
node_722: feature_name=GO:0035067 feature_id[649].value <= threshold=2.5819170475006104
node_723: feature_name=GO:0046677 feature_id[591].value <= threshold=2.915908694267273
node_724: feature_name=GO:0045579 feature_id[41].value <= threshold=1.265580177307129
Class: negative genes

Rules_35 passed counts:56
node_0: feature_name=GO:0042113 feature_id[0].value <= threshold=13.408552169799805
node_1: feature_name=GO:0007568 feature_id[534].value <= threshold=5.0313897132873535
node_2: feature_name=GO:0002705 feature_id[541].value <= threshold=3.200145721435547
node_3: feature_name=GO:1901525 feature_id[576].value <= threshold=0.4466460347175598
node_4: feature_name=GO:0048539 feature_id[319].value <= threshold=3.0399646759033203
node_5: feature_name=GO:0001910 feature_id[385].value <= threshold=3.7437864542007446
node_6: feature_name=GO:0043200 feature_id[706].value <= threshold=9.307284355163574
node_7: feature_name=GO:0001773 feature_id[308].value <= threshold=4.016931533813477
node_8: feature_name=GO:0090116 feature_id[97].value <= threshold=7.99645471572876
node_9: feature_name=GO:0019814 feature_id[189].value <= threshold=4.525782108306885
node_10: feature_name=GO:1902583 feature_id[215].value <= threshold=17.60310649871826
node_11: feature_name=GO:0045429 feature_id[737].value <= threshold=1.6133361458778381
node_12: feature_name=GO:0003720 feature_id[228].value <= threshold=5.519326210021973
node_13: feature_name=GO:0046006 feature_id[364].value <= threshold=4.9985432624816895
node_14: feature_name=GO:0070424 feature_id[254].value <= threshold=6.233297109603882
node_15: feature_name=GO:0009892 feature_id[320].value <= threshold=59.99180793762207
node_16: feature_name=GO:0007064 feature_id[527].value <= threshold=11.57377815246582
node_17: feature_name=GO:0005575 feature_id[17].value <= threshold=15.417460918426514
node_18: feature_name=GO:0043368 feature_id[31].value <= threshold=4.059496641159058
node_19: feature_name=GO:0005164 feature_id[163].value <= threshold=5.793607711791992
node_20: feature_name=GO:0042130 feature_id[16].value <= threshold=5.073179721832275

node_21: feature_name=GO:0010216	feature_id[282].value <= threshold=4.834584474563599
node_22: feature_name=GO:0009628	feature_id[553].value <= threshold=29.314892768859863
node_23: feature_name=GO:0045628	feature_id[749].value <= threshold=3.1001367568969727
node_24: feature_name=GO:0042288	feature_id[100].value <= threshold=5.174932956695557
node_25: feature_name=GO:0002329	feature_id[406].value <= threshold=2.348930835723877
node_26: feature_name=GO:0051246	feature_id[642].value <= threshold=0.6498909294605255
node_27: feature_name=GO:0042162	feature_id[355].value <= threshold=8.05103588104248
node_28: feature_name=hsa04668	feature_id[333].value <= threshold=5.614926338195801
node_29: feature_name=GO:0032200	feature_id[613].value <= threshold=28.735913276672363
node_30: feature_name=hsa04662	feature_id[334].value <= threshold=9.588344097137451
node_31: feature_name=GO:1901992	feature_id[105].value <= threshold=5.291427850723267
node_32: feature_name=GO:0072341	feature_id[144].value <= threshold=6.01465106010437
node_33: feature_name=GO:0047485	feature_id[121].value <= threshold=6.657836437225342
node_34: feature_name=GO:0090594	feature_id[738].value <= threshold=1.8673912286758423
node_35: feature_name=GO:0043525	feature_id[524].value <= threshold=2.881397008895874
node_36: feature_name=GO:0032504	feature_id[244].value <= threshold=17.22207546234131
node_37: feature_name=GO:0051348	feature_id[126].value <= threshold=5.933559417724609
node_38: feature_name=GO:0042522	feature_id[692].value <= threshold=1.78694087266922
node_39: feature_name=GO:0031100	feature_id[73].value <= threshold=3.2312101125717163
node_40: feature_name=GO:0042493	feature_id[149].value <= threshold=19.771170616149902
node_41: feature_name=GO:0001552	feature_id[366].value <= threshold=2.1711617708206177
node_42: feature_name=GO:0046483	feature_id[391].value <= threshold=320.9667053222656
node_43: feature_name=GO:0002439	feature_id[431].value <= threshold=1.832722783088684
node_44: feature_name=GO:0006555	feature_id[514].value <= threshold=5.321640968322754
node_45: feature_name=GO:0070424	feature_id[254].value <= threshold=3.321003556251526
node_46: feature_name=hsa05144	feature_id[751].value <= threshold=2.635499954223633
node_47: feature_name=GO:0032633	feature_id[627].value <= threshold=1.2592533230781555
node_48: feature_name=GO:0019814	feature_id[189].value <= threshold=2.1583125591278076
node_49: feature_name=GO:1901989	feature_id[30].value <= threshold=3.3150794506073
node_50: feature_name=GO:1903318	feature_id[196].value <= threshold=1.62252539396286
node_51: feature_name=GO:0060576	feature_id[204].value <= threshold=2.1097792387008667
node_52: feature_name=hsa05221	feature_id[349].value <= threshold=0.8723124265670776
node_53: feature_name=GO:1903896	feature_id[135].value <= threshold=1.397229254245758
node_54: feature_name=GO:0097028	feature_id[443].value > threshold=1.2365349531173706
node_88: feature_name=GO:0005488	feature_id[187].value > threshold=0.0009100764873437583

node_90: feature_name=GO:0042306 feature_id[705].value <= threshold=1.845421314239502
Class: negative genes

Rules_36 passed counts:50
node_0: feature_name=GO:0042113 feature_id[0].value <= threshold=13.408552169799805
node_1: feature_name=GO:0007568 feature_id[534].value <= threshold=5.0313897132873535
node_2: feature_name=GO:0002705 feature_id[541].value <= threshold=3.200145721435547
node_3: feature_name=GO:1901525 feature_id[576].value <= threshold=0.4466460347175598
node_4: feature_name=GO:0048539 feature_id[319].value <= threshold=3.0399646759033203
node_5: feature_name=GO:0001910 feature_id[385].value <= threshold=3.7437864542007446
node_6: feature_name=GO:0043200 feature_id[706].value <= threshold=9.307284355163574
node_7: feature_name=GO:0001773 feature_id[308].value <= threshold=4.016931533813477
node_8: feature_name=GO:0090116 feature_id[97].value <= threshold=7.99645471572876
node_9: feature_name=GO:0019814 feature_id[189].value <= threshold=4.525782108306885
node_10: feature_name=GO:1902583 feature_id[215].value <= threshold=17.60310649871826
node_11: feature_name=GO:0045429 feature_id[737].value <= threshold=1.6133361458778381
node_12: feature_name=GO:0003720 feature_id[228].value <= threshold=5.519326210021973
node_13: feature_name=GO:0046006 feature_id[364].value <= threshold=4.9985432624816895
node_14: feature_name=GO:0070424 feature_id[254].value <= threshold=6.233297109603882
node_15: feature_name=GO:0009892 feature_id[320].value <= threshold=59.99180793762207
node_16: feature_name=GO:0007064 feature_id[527].value <= threshold=11.57377815246582
node_17: feature_name=GO:0005575 feature_id[17].value <= threshold=15.417460918426514
node_18: feature_name=GO:0043368 feature_id[31].value <= threshold=4.059496641159058
node_19: feature_name=GO:0005164 feature_id[163].value <= threshold=5.793607711791992
node_20: feature_name=GO:0042130 feature_id[16].value <= threshold=5.073179721832275
node_21: feature_name=GO:0010216 feature_id[282].value <= threshold=4.834584474563599
node_22: feature_name=GO:0009628 feature_id[553].value <= threshold=29.314892768859863
node_23: feature_name=GO:0045628 feature_id[749].value <= threshold=3.1001367568969727
node_24: feature_name=GO:0042288 feature_id[100].value <= threshold=5.174932956695557
node_25: feature_name=GO:0002329 feature_id[406].value <= threshold=2.348930835723877
node_26: feature_name=GO:0051246 feature_id[642].value > threshold=0.6498909294605255
node_194: feature_name=GO:0009164 feature_id[546].value <= threshold=17.11970329284668
node_195: feature_name=GO:0001909 feature_id[386].value <= threshold=7.292566299438477
node_196: feature_name=GO:0003908 feature_id[11].value <= threshold=1.887226164340973
node_197: feature_name=GO:0009164 feature_id[546].value > threshold=0.26642198860645294

node_359: feature_name=GO:0009164 feature_id[546].value > threshold=0.2696942090988159
node_361: feature_name=hsa05202 feature_id[50].value <= threshold=14.989679336547852
node_362: feature_name=GO:0001775 feature_id[505].value <= threshold=15.726949691772461
node_363: feature_name=GO:0002634 feature_id[449].value <= threshold=2.825531005859375
node_364: feature_name=GO:0010038 feature_id[457].value <= threshold=9.901018619537354
node_365: feature_name=GO:0044238 feature_id[295].value <= threshold=139.80535888671875
node_366: feature_name=GO:0051246 feature_id[642].value > threshold=0.6527281999588013
node_368: feature_name=GO:0044092 feature_id[432].value <= threshold=11.084641933441162
node_369: feature_name=GO:0042162 feature_id[355].value <= threshold=9.683155536651611
node_370: feature_name=GO:0045945 feature_id[267].value <= threshold=3.6181851625442505
node_371: feature_name=GO:0036296 feature_id[252].value <= threshold=3.214281916618347
node_372: feature_name=GO:0097506 feature_id[51].value <= threshold=2.9644681215286255
node_373: feature_name=GO:0019740 feature_id[581].value <= threshold=2.2853575944900513
node_374: feature_name=GO:0019660 feature_id[70].value <= threshold=1.2831979990005493
node_375: feature_name=GO:0071901 feature_id[407].value <= threshold=14.498775482177734
node_376: feature_name=GO:0043170 feature_id[467].value > threshold=4.473330818655086e-06
node_380: feature_name=GO:0046718 feature_id[781].value <= threshold=3.494875192642212
node_381: feature_name=GO:0030983 feature_id[291].value <= threshold=10.994179248809814
node_382: feature_name=GO:0010663 feature_id[561].value <= threshold=2.7056833505630493
node_383: feature_name=GO:0031667 feature_id[648].value > threshold=0.0020462179090827703
node_387: feature_name=GO:1990572 feature_id[243].value <= threshold=1.9359037280082703
node_388: feature_name=GO:0032753 feature_id[101].value <= threshold=1.182796835899353
node_389: feature_name=GO:0097153 feature_id[226].value > threshold=0.34601132571697235
node_399: feature_name=GO:0036294 feature_id[612].value > threshold=0.022128281882032752
node_401: feature_name=GO:0097153 feature_id[226].value > threshold=0.40342725813388824
Class: negative genes

Rules_37 passed counts:48
node_0: feature_name=GO:0042113 feature_id[0].value <= threshold=13.408552169799805
node_1: feature_name=GO:0007568 feature_id[534].value <= threshold=5.0313897132873535
node_2: feature_name=GO:0002705 feature_id[541].value <= threshold=3.200145721435547
node_3: feature_name=GO:1901525 feature_id[576].value <= threshold=0.4466460347175598
node_4: feature_name=GO:0048539 feature_id[319].value <= threshold=3.0399646759033203
node_5: feature_name=GO:0001910 feature_id[385].value <= threshold=3.7437864542007446
node_6: feature_name=GO:0043200 feature_id[706].value <= threshold=9.307284355163574

node_7: feature_name=GO:0001773	feature_id[308].value <= threshold=4.016931533813477
node_8: feature_name=GO:0090116	feature_id[97].value <= threshold=7.99645471572876
node_9: feature_name=GO:0019814	feature_id[189].value <= threshold=4.525782108306885
node_10: feature_name=GO:1902583	feature_id[215].value <= threshold=17.60310649871826
node_11: feature_name=GO:0045429	feature_id[737].value <= threshold=1.6133361458778381
node_12: feature_name=GO:0003720	feature_id[228].value <= threshold=5.519326210021973
node_13: feature_name=GO:0046006	feature_id[364].value <= threshold=4.9985432624816895
node_14: feature_name=GO:0070424	feature_id[254].value <= threshold=6.233297109603882
node_15: feature_name=GO:0009892	feature_id[320].value <= threshold=59.99180793762207
node_16: feature_name=GO:0007064	feature_id[527].value <= threshold=11.57377815246582
node_17: feature_name=GO:0005575	feature_id[17].value <= threshold=15.417460918426514
node_18: feature_name=GO:0043368	feature_id[31].value <= threshold=4.059496641159058
node_19: feature_name=GO:0005164	feature_id[163].value <= threshold=5.793607711791992
node_20: feature_name=GO:0042130	feature_id[16].value <= threshold=5.073179721832275
node_21: feature_name=GO:0010216	feature_id[282].value <= threshold=4.834584474563599
node_22: feature_name=GO:0009628	feature_id[553].value <= threshold=29.314892768859863
node_23: feature_name=GO:0045628	feature_id[749].value <= threshold=3.1001367568969727
node_24: feature_name=GO:0042288	feature_id[100].value <= threshold=5.174932956695557
node_25: feature_name=GO:0002329	feature_id[406].value <= threshold=2.348930835723877
node_26: feature_name=GO:0051246	feature_id[642].value <= threshold=0.6498909294605255
node_27: feature_name=GO:0042162	feature_id[355].value <= threshold=8.05103588104248
node_28: feature_name=hsa04668	feature_id[333].value <= threshold=5.614926338195801
node_29: feature_name=GO:0032200	feature_id[613].value <= threshold=28.735913276672363
node_30: feature_name=hsa04662	feature_id[334].value <= threshold=9.588344097137451
node_31: feature_name=GO:1901992	feature_id[105].value <= threshold=5.291427850723267
node_32: feature_name=GO:0072341	feature_id[144].value <= threshold=6.01465106010437
node_33: feature_name=GO:0047485	feature_id[121].value <= threshold=6.657836437225342
node_34: feature_name=GO:0090594	feature_id[738].value <= threshold=1.8673912286758423
node_35: feature_name=GO:0043525	feature_id[524].value <= threshold=2.881397008895874
node_36: feature_name=GO:0032504	feature_id[244].value <= threshold=17.22207546234131
node_37: feature_name=GO:0051348	feature_id[126].value <= threshold=5.933559417724609
node_38: feature_name=GO:0042522	feature_id[692].value <= threshold=1.78694087266922
node_39: feature_name=GO:0031100	feature_id[73].value <= threshold=3.2312101125717163
node_40: feature_name=GO:0042493	feature_id[149].value <= threshold=19.771170616149902
node_41: feature_name=GO:0001552	feature_id[366].value <= threshold=2.1711617708206177

node_42: feature_name=GO:0046483	feature_id[391].value <= threshold=320.9667053222656
node_43: feature_name=GO:0002439	feature_id[431].value <= threshold=1.832722783088684
node_44: feature_name=GO:0006555	feature_id[514].value <= threshold=5.321640968322754
node_45: feature_name=GO:0070424	feature_id[254].value <= threshold=3.321003556251526
node_46: feature_name=hsa05144	feature_id[751].value <= threshold=2.635499954223633
node_47: feature_name=GO:0032633	feature_id[627].value <= threshold=1.2592533230781555
node_48: feature_name=GO:0019814	feature_id[189].value <= threshold=2.1583125591278076
node_49: feature_name=GO:1901989	feature_id[30].value <= threshold=3.3150794506073
node_50: feature_name=GO:1903318	feature_id[196].value <= threshold=1.62252539396286
node_51: feature_name=GO:0060576	feature_id[204].value <= threshold=2.1097792387008667
node_52: feature_name=hsa05221	feature_id[349].value <= threshold=0.8723124265670776
node_53: feature_name=GO:1903896	feature_id[135].value > threshold=1.397229254245758
node_93: feature_name=GO:0033554	feature_id[418].value > threshold=0.0016432370175607502
Class: negative genes	
Rules_38	passed counts:45
node_0: feature_name=GO:0042113	feature_id[0].value > threshold=13.408552169799805
node_1454: feature_name=GO:0050851	feature_id[29].value <= threshold=9.650307655334473
node_1455: feature_name=GO:0006304	feature_id[510].value <= threshold=5.960662126541138
node_1456: feature_name=GO:0032673	feature_id[623].value <= threshold=4.092082738876343
node_1457: feature_name=GO:0002429	feature_id[748].value <= threshold=4.626799821853638
node_1458: feature_name=GO:0005575	feature_id[17].value > threshold=0.7239105105400085
node_1460: feature_name=GO:0090116	feature_id[97].value <= threshold=2.8600313663482666
Class: negative genes	
Rules_39	passed counts:44
node_0: feature_name=GO:0042113	feature_id[0].value <= threshold=13.408552169799805
node_1: feature_name=GO:0007568	feature_id[534].value > threshold=5.0313897132873535
node_913: feature_name=GO:0032763	feature_id[629].value <= threshold=0.31753237545490265
node_914: feature_name=GO:0097193	feature_id[167].value <= threshold=28.171168327331543
node_915: feature_name=GO:0002903	feature_id[10].value <= threshold=1.0534588098526
node_916: feature_name=GO:1904029	feature_id[208].value <= threshold=23.852136611938477
node_917: feature_name=GO:0050897	feature_id[164].value <= threshold=0.6870408356189728
node_918: feature_name=GO:0006139	feature_id[474].value > threshold=1.3052097624921544e-07
node_920: feature_name=GO:0002821	feature_id[588].value <= threshold=13.81072187423706

node_921: feature_name=GO:0006298	feature_id[621].value <= threshold=24.078600883483887
node_922: feature_name=GO:0003908	feature_id[11].value <= threshold=1.7776933312416077
node_923: feature_name=GO:0030098	feature_id[273].value > threshold=0.0079949083738029
node_927: feature_name=GO:0006808	feature_id[513].value <= threshold=4.34592080116272
node_928: feature_name=GO:0071887	feature_id[283].value <= threshold=10.353787899017334
node_929: feature_name=GO:0038001	feature_id[28].value <= threshold=3.9469382762908936
node_930: feature_name=GO:0042287	feature_id[20].value <= threshold=3.6209195852279663
node_931: feature_name=GO:0003968	feature_id[47].value <= threshold=2.004227638244629
node_932: feature_name=GO:0002698	feature_id[395].value <= threshold=18.51447582244873
node_933: feature_name=GO:0044710	feature_id[719].value <= threshold=179.7340316772461
node_934: feature_name=GO:0007568	feature_id[534].value > threshold=5.035318374633789
node_936: feature_name=GO:0006216	feature_id[504].value <= threshold=2.2605666518211365
node_937: feature_name=GO:0048569	feature_id[793].value <= threshold=5.340231895446777
node_938: feature_name=GO:0001777	feature_id[380].value <= threshold=4.100832939147949
node_939: feature_name=GO:0007600	feature_id[122].value <= threshold=171.13383102416992
node_940: feature_name=GO:0001779	feature_id[378].value <= threshold=6.234851121902466
node_941: feature_name=GO:0030291	feature_id[147].value <= threshold=10.8051118850708
node_942: feature_name=GO:0048534	feature_id[790].value > threshold=0.017984486185014248
node_944: feature_name=GO:0070245	feature_id[326].value <= threshold=3.1282339096069336
node_945: feature_name=GO:0009086	feature_id[547].value <= threshold=0.2896959036588669
node_946: feature_name=GO:0001889	feature_id[387].value <= threshold=17.643128395080566
node_947: feature_name=GO:0048144	feature_id[192].value > threshold=0.20887330174446106
node_951: feature_name=GO:0023026	feature_id[69].value <= threshold=3.403424024581909
node_952: feature_name=GO:0048147	feature_id[788].value <= threshold=4.188283443450928
node_953: feature_name=GO:0090116	feature_id[97].value <= threshold=4.617154359817505
node_954: feature_name=GO:0002863	feature_id[490].value <= threshold=4.8968470096588135
node_955: feature_name=GO:0002524	feature_id[72].value <= threshold=3.035340189933777
node_956: feature_name=GO:0042130	feature_id[16].value <= threshold=7.285413980484009
node_957: feature_name=GO:0071456	feature_id[500].value <= threshold=21.212870597839355
node_958: feature_name=GO:2001238	feature_id[27].value <= threshold=7.849715709686279
node_959: feature_name=GO:0023030	feature_id[45].value <= threshold=1.7420591711997986
node_960: feature_name=GO:0072593	feature_id[381].value <= threshold=22.993029594421387
node_961: feature_name=GO:0034101	feature_id[646].value <= threshold=13.430044651031494
node_962: feature_name=GO:0097153	feature_id[226].value <= threshold=10.147814273834229
node_963: feature_name=GO:0001836	feature_id[450].value <= threshold=7.252067804336548

node_964: feature_name=GO:0032504 feature_id[244].value > threshold=0.15777301788330078
node_968: feature_name=GO:0043379 feature_id[714].value <= threshold=1.5959861278533936
node_969: feature_name=GO:0001889 feature_id[387].value <= threshold=0.3281313180923462
node_970: feature_name=GO:0002467 feature_id[39].value <= threshold=0.9327549636363983
Class: negative genes

Rules_40 passed counts:43
node_0: feature_name=GO:0042113 feature_id[0].value <= threshold=13.408552169799805
node_1: feature_name=GO:0007568 feature_id[534].value <= threshold=5.0313897132873535
node_2: feature_name=GO:0002705 feature_id[541].value <= threshold=3.200145721435547
node_3: feature_name=GO:1901525 feature_id[576].value <= threshold=0.4466460347175598
node_4: feature_name=GO:0048539 feature_id[319].value <= threshold=3.0399646759033203
node_5: feature_name=GO:0001910 feature_id[385].value <= threshold=3.7437864542007446
node_6: feature_name=GO:0043200 feature_id[706].value <= threshold=9.307284355163574
node_7: feature_name=GO:0001773 feature_id[308].value <= threshold=4.016931533813477
node_8: feature_name=GO:0090116 feature_id[97].value <= threshold=7.99645471572876
node_9: feature_name=GO:0019814 feature_id[189].value <= threshold=4.525782108306885
node_10: feature_name=GO:1902583 feature_id[215].value <= threshold=17.60310649871826
node_11: feature_name=GO:0045429 feature_id[737].value <= threshold=1.6133361458778381
node_12: feature_name=GO:0003720 feature_id[228].value <= threshold=5.519326210021973
node_13: feature_name=GO:0046006 feature_id[364].value <= threshold=4.9985432624816895
node_14: feature_name=GO:0070424 feature_id[254].value <= threshold=6.233297109603882
node_15: feature_name=GO:0009892 feature_id[320].value <= threshold=59.99180793762207
node_16: feature_name=GO:0007064 feature_id[527].value <= threshold=11.57377815246582
node_17: feature_name=GO:0005575 feature_id[17].value <= threshold=15.417460918426514
node_18: feature_name=GO:0043368 feature_id[31].value <= threshold=4.059496641159058
node_19: feature_name=GO:0005164 feature_id[163].value <= threshold=5.793607711791992
node_20: feature_name=GO:0042130 feature_id[16].value <= threshold=5.073179721832275
node_21: feature_name=GO:0010216 feature_id[282].value <= threshold=4.834584474563599
node_22: feature_name=GO:0009628 feature_id[553].value <= threshold=29.314892768859863
node_23: feature_name=GO:0045628 feature_id[749].value <= threshold=3.1001367568969727
node_24: feature_name=GO:0042288 feature_id[100].value <= threshold=5.174932956695557
node_25: feature_name=GO:0002329 feature_id[406].value <= threshold=2.348930835723877
node_26: feature_name=GO:0051246 feature_id[642].value > threshold=0.6498909294605255
node_194: feature_name=GO:0009164 feature_id[546].value <= threshold=17.11970329284668

node_195: feature_name=GO:0001909	feature_id[386].value <= threshold=7.292566299438477
node_196: feature_name=GO:0003908	feature_id[11].value <= threshold=1.887226164340973
node_197: feature_name=GO:0009164	feature_id[546].value <= threshold=0.26642198860645294
node_198: feature_name=GO:2001242	feature_id[24].value <= threshold=9.207026481628418
node_199: feature_name=GO:0051246	feature_id[642].value > threshold=0.6504445374011993
node_203: feature_name=GO:0032461	feature_id[608].value <= threshold=2.607542634010315
node_204: feature_name=GO:0007584	feature_id[529].value <= threshold=16.099515914916992
node_205: feature_name=hsa05221	feature_id[349].value <= threshold=9.359850406646729
node_206: feature_name=GO:0002636	feature_id[447].value <= threshold=1.615447759628296
node_207: feature_name=GO:0033993	feature_id[421].value > threshold=2.96540611088858e-05
node_217: feature_name=GO:0002331	feature_id[404].value <= threshold=2.1883513927459717
node_218: feature_name=GO:0046498	feature_id[203].value <= threshold=4.085246205329895
node_219: feature_name=GO:0002832	feature_id[491].value <= threshold=3.0228612422943115
node_220: feature_name=GO:1904868	feature_id[306].value <= threshold=3.8674756288528442
node_221: feature_name=GO:0048545	feature_id[360].value <= threshold=27.38136100769043
node_222: feature_name=GO:0071310	feature_id[760].value <= threshold=2.3522024154663086
node_223: feature_name=GO:0001775	feature_id[505].value <= threshold=9.71301555633545
node_224: feature_name=GO:0071310	feature_id[760].value <= threshold=2.346743583679199
node_225: feature_name=GO:0001772	feature_id[91].value <= threshold=3.252573609352112
node_226: feature_name=GO:0036498	feature_id[359].value <= threshold=12.092026710510254
node_227: feature_name=hsa05340	feature_id[351].value <= threshold=0.7677814364433289
node_228: feature_name=GO:0032703	feature_id[617].value <= threshold=1.7450646758079529
node_229: feature_name=hsa04640	feature_id[79].value <= threshold=2.118402123451233
node_230: feature_name=GO:0042097	feature_id[675].value <= threshold=1.8110727667808533
node_231: feature_name=GO:0071391	feature_id[518].value <= threshold=2.691588521003723
node_232: feature_name=GO:0001666	feature_id[682].value <= threshold=5.225740432739258
node_233: feature_name=GO:0048539	feature_id[319].value <= threshold=1.8523842096328735
node_234: feature_name=GO:0038061	feature_id[671].value <= threshold=8.564527988433838
node_235: feature_name=GO:0002484	feature_id[600].value <= threshold=3.1121134757995605
node_236: feature_name=GO:0023023	feature_id[119].value <= threshold=2.868617057800293
node_237: feature_name=GO:0010639	feature_id[564].value <= threshold=13.410505771636963
node_238: feature_name=GO:0080134	feature_id[650].value <= threshold=0.013442101422697306
node_239: feature_name=GO:0042991	feature_id[684].value <= threshold=0.9682981073856354
node_240: feature_name=GO:0050707	feature_id[797].value <= threshold=1.0614736676216125

Class: negative genes

Rules_41	passed counts:39
node_0: feature_name=GO:0042113	feature_id[0].value <= threshold=13.408552169799805
node_1: feature_name=GO:0007568	feature_id[534].value > threshold=5.0313897132873535
node_913: feature_name=GO:0032763	feature_id[629].value <= threshold=0.31753237545490265
node_914: feature_name=GO:0097193	feature_id[167].value <= threshold=28.171168327331543
node_915: feature_name=GO:0002903	feature_id[10].value > threshold=1.0534588098526
node_1159: feature_name=GO:0070198	feature_id[665].value > threshold=0.16219981759786606
node_1165: feature_name=GO:0042287	feature_id[20].value <= threshold=0.915093183517456
node_1166: feature_name=GO:0046006	feature_id[364].value > threshold=0.12353959679603577
node_1168: feature_name=GO:0046634	feature_id[46].value <= threshold=4.166545033454895
node_1169: feature_name=GO:0044346	feature_id[724].value > threshold=0.9571778774261475
node_1173: feature_name=GO:0060374	feature_id[76].value <= threshold=2.239573121070862
Class: negative genes	

Rules_42	passed counts:38
node_0: feature_name=GO:0042113	feature_id[0].value > threshold=13.408552169799805
node_1454: feature_name=GO:0050851	feature_id[29].value > threshold=9.650307655334473
node_1534: feature_name=GO:1902166	feature_id[301].value > threshold=0.303210511803627
node_1548: feature_name=GO:0035872	feature_id[659].value <= threshold=35.09038162231445
node_1549: feature_name=GO:0032069	feature_id[614].value <= threshold=3.441983938217163
node_1550: feature_name=GO:0050897	feature_id[164].value <= threshold=1.3939869403839111
node_1551: feature_name=GO:0051454	feature_id[307].value > threshold=0.9144491851329803
node_1565: feature_name=GO:0071453	feature_id[740].value > threshold=1.420312225818634
Class: positive genes	

Rules_43	passed counts:34
node_0: feature_name=GO:0042113	feature_id[0].value <= threshold=13.408552169799805
node_1: feature_name=GO:0007568	feature_id[534].value <= threshold=5.0313897132873535
node_2: feature_name=GO:0002705	feature_id[541].value <= threshold=3.200145721435547
node_3: feature_name=GO:1901525	feature_id[576].value <= threshold=0.4466460347175598
node_4: feature_name=GO:0048539	feature_id[319].value <= threshold=3.0399646759033203
node_5: feature_name=GO:0001910	feature_id[385].value <= threshold=3.7437864542007446
node_6: feature_name=GO:0043200	feature_id[706].value <= threshold=9.307284355163574
node_7: feature_name=GO:0001773	feature_id[308].value <= threshold=4.016931533813477

node_8: feature_name=GO:0090116 feature_id[97].value <= threshold=7.99645471572876
node_9: feature_name=GO:0019814 feature_id[189].value <= threshold=4.525782108306885
node_10: feature_name=GO:1902583 feature_id[215].value <= threshold=17.60310649871826
node_11: feature_name=GO:0045429 feature_id[737].value > threshold=1.6133361458778381
node_477: feature_name=GO:0036037 feature_id[445].value <= threshold=1.7181594371795654
node_478: feature_name=GO:0006244 feature_id[503].value > threshold=0.951388418674469
node_588: feature_name=GO:0010556 feature_id[784].value > threshold=0.0021156859584152
node_590: feature_name=GO:0016363 feature_id[90].value <= threshold=1.3301835656166077
Class: negative genes

Rules_44 passed counts:32
node_0: feature_name=GO:0042113 feature_id[0].value <= threshold=13.408552169799805
node_1: feature_name=GO:0007568 feature_id[534].value <= threshold=5.0313897132873535
node_2: feature_name=GO:0002705 feature_id[541].value <= threshold=3.200145721435547
node_3: feature_name=GO:1901525 feature_id[576].value <= threshold=0.4466460347175598
node_4: feature_name=GO:0048539 feature_id[319].value <= threshold=3.0399646759033203
node_5: feature_name=GO:0001910 feature_id[385].value <= threshold=3.7437864542007446
node_6: feature_name=GO:0043200 feature_id[706].value <= threshold=9.307284355163574
node_7: feature_name=GO:0001773 feature_id[308].value <= threshold=4.016931533813477
node_8: feature_name=GO:0090116 feature_id[97].value <= threshold=7.99645471572876
node_9: feature_name=GO:0019814 feature_id[189].value <= threshold=4.525782108306885
node_10: feature_name=GO:1902583 feature_id[215].value <= threshold=17.60310649871826
node_11: feature_name=GO:0045429 feature_id[737].value <= threshold=1.6133361458778381
node_12: feature_name=GO:0003720 feature_id[228].value <= threshold=5.519326210021973
node_13: feature_name=GO:0046006 feature_id[364].value <= threshold=4.9985432624816895
node_14: feature_name=GO:0070424 feature_id[254].value <= threshold=6.233297109603882
node_15: feature_name=GO:0009892 feature_id[320].value <= threshold=59.99180793762207
node_16: feature_name=GO:0007064 feature_id[527].value <= threshold=11.57377815246582
node_17: feature_name=GO:0005575 feature_id[17].value <= threshold=15.417460918426514
node_18: feature_name=GO:0043368 feature_id[31].value <= threshold=4.059496641159058
node_19: feature_name=GO:0005164 feature_id[163].value <= threshold=5.793607711791992
node_20: feature_name=GO:0042130 feature_id[16].value <= threshold=5.073179721832275
node_21: feature_name=GO:0010216 feature_id[282].value <= threshold=4.834584474563599
node_22: feature_name=GO:0009628 feature_id[553].value <= threshold=29.314892768859863
node_23: feature_name=GO:0045628 feature_id[749].value <= threshold=3.1001367568969727

node_24: feature_name=GO:0042288 feature_id[100].value <= threshold=5.174932956695557
node_25: feature_name=GO:0002329 feature_id[406].value <= threshold=2.348930835723877
node_26: feature_name=GO:0051246 feature_id[642].value > threshold=0.6498909294605255
node_194: feature_name=GO:0009164 feature_id[546].value <= threshold=17.11970329284668
node_195: feature_name=GO:0001909 feature_id[386].value <= threshold=7.292566299438477
node_196: feature_name=GO:0003908 feature_id[11].value <= threshold=1.887226164340973
node_197: feature_name=GO:0009164 feature_id[546].value > threshold=0.26642198860645294
node_359: feature_name=GO:0009164 feature_id[546].value > threshold=0.2696942090988159
node_361: feature_name=hsa05202 feature_id[50].value <= threshold=14.989679336547852
node_362: feature_name=GO:0001775 feature_id[505].value <= threshold=15.726949691772461
node_363: feature_name=GO:0002634 feature_id[449].value <= threshold=2.825531005859375
node_364: feature_name=GO:0010038 feature_id[457].value <= threshold=9.901018619537354
node_365: feature_name=GO:0044238 feature_id[295].value <= threshold=139.80535888671875
node_366: feature_name=GO:0051246 feature_id[642].value > threshold=0.6527281999588013
node_368: feature_name=GO:0044092 feature_id[432].value <= threshold=11.084641933441162
node_369: feature_name=GO:0042162 feature_id[355].value <= threshold=9.683155536651611
node_370: feature_name=GO:0045945 feature_id[267].value <= threshold=3.6181851625442505
node_371: feature_name=GO:0036296 feature_id[252].value <= threshold=3.214281916618347
node_372: feature_name=GO:0097506 feature_id[51].value <= threshold=2.9644681215286255
node_373: feature_name=GO:0019740 feature_id[581].value <= threshold=2.2853575944900513
node_374: feature_name=GO:0019660 feature_id[70].value <= threshold=1.2831979990005493
node_375: feature_name=GO:0071901 feature_id[407].value <= threshold=14.498775482177734
node_376: feature_name=GO:0043170 feature_id[467].value > threshold=4.473330818655086e-06
node_380: feature_name=GO:0046718 feature_id[781].value <= threshold=3.494875192642212
node_381: feature_name=GO:0030983 feature_id[291].value <= threshold=10.994179248809814
node_382: feature_name=GO:0010663 feature_id[561].value <= threshold=2.7056833505630493
node_383: feature_name=GO:0031667 feature_id[648].value > threshold=0.0020462179090827703
node_387: feature_name=GO:1990572 feature_id[243].value <= threshold=1.9359037280082703
node_388: feature_name=GO:0032753 feature_id[101].value > threshold=1.182796835899353
node_404: feature_name=GO:0002449 feature_id[427].value > threshold=0.09284071624279022
node_406: feature_name=GO:0097506 feature_id[51].value <= threshold=0.9462986290454865
Class: negative genes

Rules_45 passed counts:32
node_0: feature_name=GO:0042113 feature_id[0].value <= threshold=13.408552169799805

node_1: feature_name=GO:0007568	feature_id[534].value <= threshold=5.0313897132873535
node_2: feature_name=GO:0002705	feature_id[541].value <= threshold=3.200145721435547
node_3: feature_name=GO:1901525	feature_id[576].value <= threshold=0.4466460347175598
node_4: feature_name=GO:0048539	feature_id[319].value <= threshold=3.0399646759033203
node_5: feature_name=GO:0001910	feature_id[385].value <= threshold=3.7437864542007446
node_6: feature_name=GO:0043200	feature_id[706].value <= threshold=9.307284355163574
node_7: feature_name=GO:0001773	feature_id[308].value <= threshold=4.016931533813477
node_8: feature_name=GO:0090116	feature_id[97].value <= threshold=7.99645471572876
node_9: feature_name=GO:0019814	feature_id[189].value <= threshold=4.525782108306885
node_10: feature_name=GO:1902583	feature_id[215].value <= threshold=17.60310649871826
node_11: feature_name=GO:0045429	feature_id[737].value <= threshold=1.6133361458778381
node_12: feature_name=GO:0003720	feature_id[228].value <= threshold=5.519326210021973
node_13: feature_name=GO:0046006	feature_id[364].value <= threshold=4.9985432624816895
node_14: feature_name=GO:0070424	feature_id[254].value <= threshold=6.233297109603882
node_15: feature_name=GO:0009892	feature_id[320].value <= threshold=59.99180793762207
node_16: feature_name=GO:0007064	feature_id[527].value <= threshold=11.57377815246582
node_17: feature_name=GO:0005575	feature_id[17].value <= threshold=15.417460918426514
node_18: feature_name=GO:0043368	feature_id[31].value <= threshold=4.059496641159058
node_19: feature_name=GO:0005164	feature_id[163].value <= threshold=5.793607711791992
node_20: feature_name=GO:0042130	feature_id[16].value <= threshold=5.073179721832275
node_21: feature_name=GO:0010216	feature_id[282].value <= threshold=4.834584474563599
node_22: feature_name=GO:0009628	feature_id[553].value <= threshold=29.314892768859863
node_23: feature_name=GO:0045628	feature_id[749].value <= threshold=3.1001367568969727
node_24: feature_name=GO:0042288	feature_id[100].value <= threshold=5.174932956695557
node_25: feature_name=GO:0002329	feature_id[406].value <= threshold=2.348930835723877
node_26: feature_name=GO:0051246	feature_id[642].value > threshold=0.6498909294605255
node_194: feature_name=GO:0009164	feature_id[546].value <= threshold=17.11970329284668
node_195: feature_name=GO:0001909	feature_id[386].value <= threshold=7.292566299438477
node_196: feature_name=GO:0003908	feature_id[11].value <= threshold=1.887226164340973
node_197: feature_name=GO:0009164	feature_id[546].value <= threshold=0.26642198860645294
node_198: feature_name=GO:2001242	feature_id[24].value <= threshold=9.207026481628418
node_199: feature_name=GO:0051246	feature_id[642].value > threshold=0.6504445374011993
node_203: feature_name=GO:0032461	feature_id[608].value <= threshold=2.607542634010315
node_204: feature_name=GO:0007584	feature_id[529].value <= threshold=16.099515914916992
node_205: feature_name=hsa05221	feature_id[349].value <= threshold=9.359850406646729

node_206: feature_name=GO:0002636 feature_id[447].value <= threshold=1.615447759628296
node_207: feature_name=GO:0033993 feature_id[421].value > threshold=2.96540611088858e-05
node_217: feature_name=GO:0002331 feature_id[404].value <= threshold=2.1883513927459717
node_218: feature_name=GO:0046498 feature_id[203].value <= threshold=4.085246205329895
node_219: feature_name=GO:0002832 feature_id[491].value <= threshold=3.0228612422943115
node_220: feature_name=GO:1904868 feature_id[306].value <= threshold=3.8674756288528442
node_221: feature_name=GO:0048545 feature_id[360].value <= threshold=27.38136100769043
node_222: feature_name=GO:0071310 feature_id[760].value > threshold=2.3522024154663086
node_306: feature_name=GO:0070233 feature_id[321].value <= threshold=1.293377935886383
node_307: feature_name=hsa05221 feature_id[349].value <= threshold=9.005411148071289
node_308: feature_name=GO:0032770 feature_id[604].value > threshold=3.2187339067459106
node_310: feature_name=GO:0002902 feature_id[59].value <= threshold=1.6938291788101196
Class: negative genes

Rules_46 passed counts:31
node_0: feature_name=GO:0042113 feature_id[0].value > threshold=13.408552169799805
node_1454: feature_name=GO:0050851 feature_id[29].value <= threshold=9.650307655334473
node_1455: feature_name=GO:0006304 feature_id[510].value <= threshold=5.960662126541138
node_1456: feature_name=GO:0032673 feature_id[623].value <= threshold=4.092082738876343
node_1457: feature_name=GO:0002429 feature_id[748].value > threshold=4.626799821853638
node_1463: feature_name=GO:0015672 feature_id[573].value > threshold=0.08226438239216805
node_1475: feature_name=GO:0030217 feature_id[589].value <= threshold=19.77315902709961
node_1476: feature_name=GO:0002381 feature_id[96].value <= threshold=4.645626783370972
node_1477: feature_name=GO:0045058 feature_id[727].value <= threshold=5.092289447784424
node_1478: feature_name=GO:0032703 feature_id[617].value <= threshold=2.6942304372787476
node_1479: feature_name=GO:0001909 feature_id[386].value > threshold=0.47530554234981537
Class: negative genes

Rules_47 passed counts:30
node_0: feature_name=GO:0042113 feature_id[0].value <= threshold=13.408552169799805
node_1: feature_name=GO:0007568 feature_id[534].value > threshold=5.0313897132873535
node_913: feature_name=GO:0032763 feature_id[629].value <= threshold=0.31753237545490265
node_914: feature_name=GO:0097193 feature_id[167].value <= threshold=28.171168327331543
node_915: feature_name=GO:0002903 feature_id[10].value <= threshold=1.0534588098526
node_916: feature_name=GO:1904029 feature_id[208].value <= threshold=23.852136611938477

node_917: feature_name=GO:0050897	feature_id[164].value <= threshold=0.6870408356189728
node_918: feature_name=GO:0006139	feature_id[474].value > threshold=1.3052097624921544e-07
node_920: feature_name=GO:0002821	feature_id[588].value <= threshold=13.81072187423706
node_921: feature_name=GO:0006298	feature_id[621].value <= threshold=24.078600883483887
node_922: feature_name=GO:0003908	feature_id[11].value <= threshold=1.7776933312416077
node_923: feature_name=GO:0030098	feature_id[273].value > threshold=0.0079949083738029
node_927: feature_name=GO:0006808	feature_id[513].value <= threshold=4.34592080116272
node_928: feature_name=GO:0071887	feature_id[283].value <= threshold=10.353787899017334
node_929: feature_name=GO:0038001	feature_id[28].value <= threshold=3.9469382762908936
node_930: feature_name=GO:0042287	feature_id[20].value <= threshold=3.6209195852279663
node_931: feature_name=GO:0003968	feature_id[47].value <= threshold=2.004227638244629
node_932: feature_name=GO:0002698	feature_id[395].value <= threshold=18.51447582244873
node_933: feature_name=GO:0044710	feature_id[719].value <= threshold=179.7340316772461
node_934: feature_name=GO:0007568	feature_id[534].value > threshold=5.035318374633789
node_936: feature_name=GO:0006216	feature_id[504].value <= threshold=2.2605666518211365
node_937: feature_name=GO:0048569	feature_id[793].value <= threshold=5.340231895446777
node_938: feature_name=GO:0001777	feature_id[380].value <= threshold=4.100832939147949
node_939: feature_name=GO:0007600	feature_id[122].value <= threshold=171.13383102416992
node_940: feature_name=GO:0001779	feature_id[378].value <= threshold=6.234851121902466
node_941: feature_name=GO:0030291	feature_id[147].value <= threshold=10.8051118850708
node_942: feature_name=GO:0048534	feature_id[790].value > threshold=0.017984486185014248
node_944: feature_name=GO:0070245	feature_id[326].value <= threshold=3.1282339096069336
node_945: feature_name=GO:0009086	feature_id[547].value > threshold=0.2896959036588669
node_1017: feature_name=GO:0000302	feature_id[375].value > threshold=0.305715873837471
node_1019: feature_name=GO:0070141	feature_id[37].value <= threshold=4.857294321060181
node_1020: feature_name=GO:0009086	feature_id[547].value > threshold=0.2945319563150406
node_1024: feature_name=GO:0010332	feature_id[557].value <= threshold=15.092710971832275
node_1025: feature_name=GO:0002524	feature_id[72].value <= threshold=2.531021237373352
node_1026: feature_name=GO:0016032	feature_id[571].value <= threshold=60.173330307006836
node_1027: feature_name=GO:0002832	feature_id[491].value <= threshold=2.0699684023857117
node_1028: feature_name=GO:0008588	feature_id[26].value <= threshold=2.150238513946533
node_1029: feature_name=GO:0009615	feature_id[265].value > threshold=0.02372477948665619
node_1031: feature_name=GO:0002763	feature_id[271].value <= threshold=6.462319850921631
node_1032: feature_name=GO:0045840	feature_id[697].value <= threshold=10.30175256729126
node_1033: feature_name=GO:0051402	feature_id[568].value <= threshold=1.2523645758628845

node_1034: feature_name=GO:0008625 feature_id[99].value <= threshold=1.086933195590973
node_1035: feature_name=GO:0009636 feature_id[551].value <= threshold=11.690044641494751
Class: negative genes

Rules_48 passed counts:29
node_0: feature_name=GO:0042113 feature_id[0].value > threshold=13.408552169799805
node_1454: feature_name=GO:0050851 feature_id[29].value <= threshold=9.650307655334473
node_1455: feature_name=GO:0006304 feature_id[510].value > threshold=5.960662126541138
node_1531: feature_name=GO:2001251 feature_id[220].value <= threshold=24.98499584197998
Class: positive genes

Rules_49 passed counts:29
node_0: feature_name=GO:0042113 feature_id[0].value <= threshold=13.408552169799805
node_1: feature_name=GO:0007568 feature_id[534].value <= threshold=5.0313897132873535
node_2: feature_name=GO:0002705 feature_id[541].value <= threshold=3.200145721435547
node_3: feature_name=GO:1901525 feature_id[576].value > threshold=0.4466460347175598
node_617: feature_name=GO:0005622 feature_id[233].value <= threshold=90.85730743408203
node_618: feature_name=GO:1903147 feature_id[200].value <= threshold=7.326428413391113
node_619: feature_name=GO:0003964 feature_id[317].value <= threshold=5.575642108917236
node_620: feature_name=GO:0046498 feature_id[203].value <= threshold=6.620223522186279
node_621: feature_name=GO:0002562 feature_id[25].value <= threshold=10.287704467773438
node_622: feature_name=GO:0006919 feature_id[246].value <= threshold=5.801334381103516
node_623: feature_name=GO:0001889 feature_id[387].value <= threshold=2.679414987564087
node_624: feature_name=GO:0006555 feature_id[514].value <= threshold=7.819535493850708
node_625: feature_name=GO:0080134 feature_id[650].value <= threshold=38.91967582702637
node_626: feature_name=GO:0042127 feature_id[480].value <= threshold=37.72536659240723
node_627: feature_name=GO:2001251 feature_id[220].value <= threshold=32.564422607421875
node_628: feature_name=GO:0002829 feature_id[493].value <= threshold=2.444612741470337
node_629: feature_name=GO:0008588 feature_id[26].value <= threshold=2.3601274490356445
node_630: feature_name=GO:0033151 feature_id[7].value <= threshold=5.134376287460327
node_631: feature_name=GO:0048145 feature_id[348].value <= threshold=8.836549282073975
node_632: feature_name=GO:0030887 feature_id[274].value <= threshold=1.7000296115875244
node_633: feature_name=GO:0097190 feature_id[117].value <= threshold=33.02078819274902
node_634: feature_name=GO:0046685 feature_id[782].value <= threshold=8.040813446044922
node_635: feature_name=GO:0006346 feature_id[236].value <= threshold=6.384642839431763

node_636: feature_name=GO:0048537 feature_id[66].value <= threshold=3.1739262342453003
node_637: feature_name=GO:0070245 feature_id[326].value <= threshold=3.0491198301315308
node_638: feature_name=GO:0038065 feature_id[670].value <= threshold=2.0590850114822388
node_639: feature_name=GO:0005488 feature_id[187].value <= threshold=84.80076217651367
node_640: feature_name=GO:0060249 feature_id[390].value > threshold=0.07226398587226868
node_652: feature_name=GO:0006974 feature_id[516].value > threshold=5.17782768838515e-06
node_654: feature_name=hsa05210 feature_id[358].value <= threshold=18.730005264282227
node_655: feature_name=GO:0071850 feature_id[40].value <= threshold=5.475317001342773
node_656: feature_name=GO:1904029 feature_id[208].value <= threshold=26.575013160705566
node_657: feature_name=GO:0090594 feature_id[738].value <= threshold=3.000791072845459
node_658: feature_name=GO:0010948 feature_id[569].value <= threshold=0.04386143572628498
node_659: feature_name=GO:0002704 feature_id[470].value <= threshold=0.7501857876777649
node_660: feature_name=GO:0006275 feature_id[80].value <= threshold=3.2739195823669434
Class: negative genes

Rules_50 passed counts:28
node_0: feature_name=GO:0042113 feature_id[0].value <= threshold=13.408552169799805
node_1: feature_name=GO:0007568 feature_id[534].value <= threshold=5.0313897132873535
node_2: feature_name=GO:0002705 feature_id[541].value <= threshold=3.200145721435547
node_3: feature_name=GO:1901525 feature_id[576].value <= threshold=0.4466460347175598
node_4: feature_name=GO:0048539 feature_id[319].value <= threshold=3.0399646759033203
node_5: feature_name=GO:0001910 feature_id[385].value <= threshold=3.7437864542007446
node_6: feature_name=GO:0043200 feature_id[706].value <= threshold=9.307284355163574
node_7: feature_name=GO:0001773 feature_id[308].value <= threshold=4.016931533813477
node_8: feature_name=GO:0090116 feature_id[97].value <= threshold=7.99645471572876
node_9: feature_name=GO:0019814 feature_id[189].value <= threshold=4.525782108306885
node_10: feature_name=GO:1902583 feature_id[215].value <= threshold=17.60310649871826
node_11: feature_name=GO:0045429 feature_id[737].value <= threshold=1.6133361458778381
node_12: feature_name=GO:0003720 feature_id[228].value <= threshold=5.519326210021973
node_13: feature_name=GO:0046006 feature_id[364].value <= threshold=4.9985432624816895
node_14: feature_name=GO:0070424 feature_id[254].value <= threshold=6.233297109603882
node_15: feature_name=GO:0009892 feature_id[320].value <= threshold=59.99180793762207
node_16: feature_name=GO:0007064 feature_id[527].value <= threshold=11.57377815246582
node_17: feature_name=GO:0005575 feature_id[17].value <= threshold=15.417460918426514
node_18: feature_name=GO:0043368 feature_id[31].value <= threshold=4.059496641159058

node_19: feature_name=GO:0005164	feature_id[163].value <= threshold=5.793607711791992
node_20: feature_name=GO:0042130	feature_id[16].value <= threshold=5.073179721832275
node_21: feature_name=GO:0010216	feature_id[282].value <= threshold=4.834584474563599
node_22: feature_name=GO:0009628	feature_id[553].value <= threshold=29.314892768859863
node_23: feature_name=GO:0045628	feature_id[749].value <= threshold=3.1001367568969727
node_24: feature_name=GO:0042288	feature_id[100].value <= threshold=5.174932956695557
node_25: feature_name=GO:0002329	feature_id[406].value <= threshold=2.348930835723877
node_26: feature_name=GO:0051246	feature_id[642].value > threshold=0.6498909294605255
node_194: feature_name=GO:0009164	feature_id[546].value <= threshold=17.11970329284668
node_195: feature_name=GO:0001909	feature_id[386].value <= threshold=7.292566299438477
node_196: feature_name=GO:0003908	feature_id[11].value <= threshold=1.887226164340973
node_197: feature_name=GO:0009164	feature_id[546].value <= threshold=0.26642198860645294
node_198: feature_name=GO:2001242	feature_id[24].value <= threshold=9.207026481628418
node_199: feature_name=GO:0051246	feature_id[642].value > threshold=0.6504445374011993
node_203: feature_name=GO:0032461	feature_id[608].value <= threshold=2.607542634010315
node_204: feature_name=GO:0007584	feature_id[529].value <= threshold=16.099515914916992
node_205: feature_name=hsa05221	feature_id[349].value <= threshold=9.359850406646729
node_206: feature_name=GO:0002636	feature_id[447].value <= threshold=1.615447759628296
node_207: feature_name=GO:0033993	feature_id[421].value > threshold=2.96540611088858e-05
node_217: feature_name=GO:0002331	feature_id[404].value <= threshold=2.1883513927459717
node_218: feature_name=GO:0046498	feature_id[203].value <= threshold=4.085246205329895
node_219: feature_name=GO:0002832	feature_id[491].value <= threshold=3.0228612422943115
node_220: feature_name=GO:1904868	feature_id[306].value <= threshold=3.8674756288528442
node_221: feature_name=GO:0048545	feature_id[360].value <= threshold=27.38136100769043
node_222: feature_name=GO:0071310	feature_id[760].value > threshold=2.3522024154663086
node_306: feature_name=GO:0070233	feature_id[321].value <= threshold=1.293377935886383
node_307: feature_name=hsa05221	feature_id[349].value > threshold=9.005411148071289
node_313: feature_name=GO:0008340	feature_id[539].value <= threshold=1.3541167378425598

Class: negative genes

Rules_51	passed counts:28
node_0: feature_name=GO:0042113	feature_id[0].value <= threshold=13.408552169799805
node_1: feature_name=GO:0007568	feature_id[534].value <= threshold=5.0313897132873535
node_2: feature_name=GO:0002705	feature_id[541].value <= threshold=3.200145721435547
node_3: feature_name=GO:1901525	feature_id[576].value <= threshold=0.4466460347175598

node_4: feature_name=GO:0048539	feature_id[319].value <= threshold=3.0399646759033203
node_5: feature_name=GO:0001910	feature_id[385].value <= threshold=3.7437864542007446
node_6: feature_name=GO:0043200	feature_id[706].value <= threshold=9.307284355163574
node_7: feature_name=GO:0001773	feature_id[308].value <= threshold=4.016931533813477
node_8: feature_name=GO:0090116	feature_id[97].value <= threshold=7.99645471572876
node_9: feature_name=GO:0019814	feature_id[189].value <= threshold=4.525782108306885
node_10: feature_name=GO:1902583	feature_id[215].value <= threshold=17.60310649871826
node_11: feature_name=GO:0045429	feature_id[737].value <= threshold=1.6133361458778381
node_12: feature_name=GO:0003720	feature_id[228].value <= threshold=5.519326210021973
node_13: feature_name=GO:0046006	feature_id[364].value <= threshold=4.9985432624816895
node_14: feature_name=GO:0070424	feature_id[254].value <= threshold=6.233297109603882
node_15: feature_name=GO:0009892	feature_id[320].value <= threshold=59.99180793762207
node_16: feature_name=GO:0007064	feature_id[527].value <= threshold=11.57377815246582
node_17: feature_name=GO:0005575	feature_id[17].value <= threshold=15.417460918426514
node_18: feature_name=GO:0043368	feature_id[31].value <= threshold=4.059496641159058
node_19: feature_name=GO:0005164	feature_id[163].value <= threshold=5.793607711791992
node_20: feature_name=GO:0042130	feature_id[16].value <= threshold=5.073179721832275
node_21: feature_name=GO:0010216	feature_id[282].value <= threshold=4.834584474563599
node_22: feature_name=GO:0009628	feature_id[553].value <= threshold=29.314892768859863
node_23: feature_name=GO:0045628	feature_id[749].value <= threshold=3.1001367568969727
node_24: feature_name=GO:0042288	feature_id[100].value <= threshold=5.174932956695557
node_25: feature_name=GO:0002329	feature_id[406].value <= threshold=2.348930835723877
node_26: feature_name=GO:0051246	feature_id[642].value <= threshold=0.6498909294605255
node_27: feature_name=GO:0042162	feature_id[355].value <= threshold=8.05103588104248
node_28: feature_name=hsa04668	feature_id[333].value <= threshold=5.614926338195801
node_29: feature_name=GO:0032200	feature_id[613].value <= threshold=28.735913276672363
node_30: feature_name=hsa04662	feature_id[334].value <= threshold=9.588344097137451
node_31: feature_name=GO:1901992	feature_id[105].value <= threshold=5.291427850723267
node_32: feature_name=GO:0072341	feature_id[144].value <= threshold=6.01465106010437
node_33: feature_name=GO:0047485	feature_id[121].value <= threshold=6.657836437225342
node_34: feature_name=GO:0090594	feature_id[738].value <= threshold=1.8673912286758423
node_35: feature_name=GO:0043525	feature_id[524].value <= threshold=2.881397008895874
node_36: feature_name=GO:0032504	feature_id[244].value <= threshold=17.22207546234131
node_37: feature_name=GO:0051348	feature_id[126].value <= threshold=5.933559417724609
node_38: feature_name=GO:0042522	feature_id[692].value <= threshold=1.78694087266922

node_39: feature_name=GO:0031100 feature_id[73].value <= threshold=3.2312101125717163
node_40: feature_name=GO:0042493 feature_id[149].value <= threshold=19.771170616149902
node_41: feature_name=GO:0001552 feature_id[366].value <= threshold=2.1711617708206177
node_42: feature_name=GO:0046483 feature_id[391].value <= threshold=320.9667053222656
node_43: feature_name=GO:0002439 feature_id[431].value <= threshold=1.832722783088684
node_44: feature_name=GO:0006555 feature_id[514].value <= threshold=5.321640968322754
node_45: feature_name=GO:0070424 feature_id[254].value <= threshold=3.321003556251526
node_46: feature_name=hsa05144 feature_id[751].value <= threshold=2.635499954223633
node_47: feature_name=GO:0032633 feature_id[627].value <= threshold=1.2592533230781555
node_48: feature_name=GO:0019814 feature_id[189].value <= threshold=2.1583125591278076
node_49: feature_name=GO:1901989 feature_id[30].value <= threshold=3.3150794506073
node_50: feature_name=GO:1903318 feature_id[196].value <= threshold=1.62252539396286
node_51: feature_name=GO:0060576 feature_id[204].value <= threshold=2.1097792387008667
node_52: feature_name=hsa05221 feature_id[349].value <= threshold=0.8723124265670776
node_53: feature_name=GO:1903896 feature_id[135].value <= threshold=1.397229254245758
node_54: feature_name=GO:0097028 feature_id[443].value <= threshold=1.2365349531173706
node_55: feature_name=GO:0048660 feature_id[403].value <= threshold=1.9425984621047974
node_56: feature_name=GO:0043011 feature_id[150].value > threshold=1.3008450269699097
node_82: feature_name=GO:0007049 feature_id[259].value > threshold=4.681470727518899e-06
Class: negative genes

Rules_52 passed counts:27
node_0: feature_name=GO:0042113 feature_id[0].value <= threshold=13.408552169799805
node_1: feature_name=GO:0007568 feature_id[534].value > threshold=5.0313897132873535
node_913: feature_name=GO:0032763 feature_id[629].value > threshold=0.31753237545490265
node_1195: feature_name=GO:0071301 feature_id[442].value > threshold=0.11534593254327774
node_1375: feature_name=GO:0046500 feature_id[171].value > threshold=1.130434513092041
node_1437: feature_name=GO:0033343 feature_id[639].value > threshold=0.98012974858284
node_1443: feature_name=GO:0007126 feature_id[520].value > threshold=0.40390433371067047
node_1447: feature_name=GO:0019058 feature_id[245].value <= threshold=9.540197849273682
node_1448: feature_name=GO:0002718 feature_id[481].value > threshold=0.052317868918180466
Class: positive genes

Rules_53 passed counts:26
node_0: feature_name=GO:0042113 feature_id[0].value <= threshold=13.408552169799805

node_1: feature_name=GO:0007568 feature_id[534].value <= threshold=5.0313897132873535
node_2: feature_name=GO:0002705 feature_id[541].value <= threshold=3.200145721435547
node_3: feature_name=GO:1901525 feature_id[576].value > threshold=0.4466460347175598
node_617: feature_name=GO:0005622 feature_id[233].value <= threshold=90.85730743408203
node_618: feature_name=GO:1903147 feature_id[200].value <= threshold=7.326428413391113
node_619: feature_name=GO:0003964 feature_id[317].value <= threshold=5.575642108917236
node_620: feature_name=GO:0046498 feature_id[203].value <= threshold=6.620223522186279
node_621: feature_name=GO:0002562 feature_id[25].value <= threshold=10.287704467773438
node_622: feature_name=GO:0006919 feature_id[246].value <= threshold=5.801334381103516
node_623: feature_name=GO:0001889 feature_id[387].value <= threshold=2.679414987564087
node_624: feature_name=GO:0006555 feature_id[514].value <= threshold=7.819535493850708
node_625: feature_name=GO:0080134 feature_id[650].value <= threshold=38.91967582702637
node_626: feature_name=GO:0042127 feature_id[480].value <= threshold=37.72536659240723
node_627: feature_name=GO:2001251 feature_id[220].value <= threshold=32.564422607421875
node_628: feature_name=GO:0002829 feature_id[493].value <= threshold=2.444612741470337
node_629: feature_name=GO:0008588 feature_id[26].value <= threshold=2.3601274490356445
node_630: feature_name=GO:0033151 feature_id[7].value <= threshold=5.134376287460327
node_631: feature_name=GO:0048145 feature_id[348].value <= threshold=8.836549282073975
node_632: feature_name=GO:0030887 feature_id[274].value <= threshold=1.7000296115875244
node_633: feature_name=GO:0097190 feature_id[117].value <= threshold=33.02078819274902
node_634: feature_name=GO:0046685 feature_id[782].value <= threshold=8.040813446044922
node_635: feature_name=GO:0006346 feature_id[236].value <= threshold=6.384642839431763
node_636: feature_name=GO:0048537 feature_id[66].value <= threshold=3.1739262342453003
node_637: feature_name=GO:0070245 feature_id[326].value <= threshold=3.0491198301315308
node_638: feature_name=GO:0038065 feature_id[670].value <= threshold=2.0590850114822388
node_639: feature_name=GO:0005488 feature_id[187].value <= threshold=84.80076217651367
node_640: feature_name=GO:0060249 feature_id[390].value > threshold=0.07226398587226868
node_652: feature_name=GO:0006974 feature_id[516].value > threshold=5.17782768838515e-06
node_654: feature_name=hsa05210 feature_id[358].value <= threshold=18.730005264282227
node_655: feature_name=GO:0071850 feature_id[40].value <= threshold=5.475317001342773
node_656: feature_name=GO:1904029 feature_id[208].value <= threshold=26.575013160705566
node_657: feature_name=GO:0090594 feature_id[738].value <= threshold=3.000791072845459
node_658: feature_name=GO:0010948 feature_id[569].value > threshold=0.04386143572628498
node_666: feature_name=GO:0032764 feature_id[3].value <= threshold=2.5201677083969116
node_667: feature_name=GO:0023026 feature_id[69].value <= threshold=3.2760006189346313

node_668: feature_name=GO:0032464 feature_id[83].value <= threshold=2.5927021503448486
node_669: feature_name=GO:0071901 feature_id[407].value <= threshold=18.238012313842773
node_670: feature_name=GO:0015671 feature_id[103].value <= threshold=1.3113192915916443
node_671: feature_name=GO:0008340 feature_id[539].value <= threshold=4.860779762268066
node_672: feature_name=GO:0046006 feature_id[364].value <= threshold=2.4317692518234253
node_673: feature_name=GO:0042991 feature_id[684].value <= threshold=5.553565502166748
node_674: feature_name=GO:0002327 feature_id[408].value <= threshold=2.404940366744995
node_675: feature_name=GO:0001836 feature_id[450].value <= threshold=8.647934436798096
node_676: feature_name=GO:0048539 feature_id[319].value <= threshold=1.6308802962303162
node_677: feature_name=GO:0022408 feature_id[279].value <= threshold=4.064727783203125
node_678: feature_name=GO:0030099 feature_id[598].value <= threshold=0.0001809061213862151
node_679: feature_name=hsa05219 feature_id[162].value <= threshold=3.047200083732605
Class: negative genes

Rules_54 passed counts:26
node_0: feature_name=GO:0042113 feature_id[0].value <= threshold=13.408552169799805
node_1: feature_name=GO:0007568 feature_id[534].value <= threshold=5.0313897132873535
node_2: feature_name=GO:0002705 feature_id[541].value <= threshold=3.200145721435547
node_3: feature_name=GO:1901525 feature_id[576].value <= threshold=0.4466460347175598
node_4: feature_name=GO:0048539 feature_id[319].value <= threshold=3.0399646759033203
node_5: feature_name=GO:0001910 feature_id[385].value <= threshold=3.7437864542007446
node_6: feature_name=GO:0043200 feature_id[706].value <= threshold=9.307284355163574
node_7: feature_name=GO:0001773 feature_id[308].value <= threshold=4.016931533813477
node_8: feature_name=GO:0090116 feature_id[97].value <= threshold=7.99645471572876
node_9: feature_name=GO:0019814 feature_id[189].value <= threshold=4.525782108306885
node_10: feature_name=GO:1902583 feature_id[215].value <= threshold=17.60310649871826
node_11: feature_name=GO:0045429 feature_id[737].value <= threshold=1.6133361458778381
node_12: feature_name=GO:0003720 feature_id[228].value <= threshold=5.519326210021973
node_13: feature_name=GO:0046006 feature_id[364].value <= threshold=4.9985432624816895
node_14: feature_name=GO:0070424 feature_id[254].value <= threshold=6.233297109603882
node_15: feature_name=GO:0009892 feature_id[320].value <= threshold=59.99180793762207
node_16: feature_name=GO:0007064 feature_id[527].value <= threshold=11.57377815246582
node_17: feature_name=GO:0005575 feature_id[17].value <= threshold=15.417460918426514
node_18: feature_name=GO:0043368 feature_id[31].value <= threshold=4.059496641159058
node_19: feature_name=GO:0005164 feature_id[163].value <= threshold=5.793607711791992

node_20: feature_name=GO:0042130 feature_id[16].value <= threshold=5.073179721832275
node_21: feature_name=GO:0010216 feature_id[282].value <= threshold=4.834584474563599
node_22: feature_name=GO:0009628 feature_id[553].value <= threshold=29.314892768859863
node_23: feature_name=GO:0045628 feature_id[749].value <= threshold=3.1001367568969727
node_24: feature_name=GO:0042288 feature_id[100].value <= threshold=5.174932956695557
node_25: feature_name=GO:0002329 feature_id[406].value <= threshold=2.348930835723877
node_26: feature_name=GO:0051246 feature_id[642].value > threshold=0.6498909294605255
node_194: feature_name=GO:0009164 feature_id[546].value <= threshold=17.11970329284668
node_195: feature_name=GO:0001909 feature_id[386].value <= threshold=7.292566299438477
node_196: feature_name=GO:0003908 feature_id[11].value <= threshold=1.887226164340973
node_197: feature_name=GO:0009164 feature_id[546].value <= threshold=0.26642198860645294
node_198: feature_name=GO:2001242 feature_id[24].value <= threshold=9.207026481628418
node_199: feature_name=GO:0051246 feature_id[642].value > threshold=0.6504445374011993
node_203: feature_name=GO:0032461 feature_id[608].value <= threshold=2.607542634010315
node_204: feature_name=GO:0007584 feature_id[529].value <= threshold=16.099515914916992
node_205: feature_name=hsa05221 feature_id[349].value <= threshold=9.359850406646729
node_206: feature_name=GO:0002636 feature_id[447].value <= threshold=1.615447759628296
node_207: feature_name=GO:0033993 feature_id[421].value > threshold=2.96540611088858e-05
node_217: feature_name=GO:0002331 feature_id[404].value <= threshold=2.1883513927459717
node_218: feature_name=GO:0046498 feature_id[203].value <= threshold=4.085246205329895
node_219: feature_name=GO:0002832 feature_id[491].value <= threshold=3.0228612422943115
node_220: feature_name=GO:1904868 feature_id[306].value <= threshold=3.8674756288528442
node_221: feature_name=GO:0048545 feature_id[360].value <= threshold=27.38136100769043
node_222: feature_name=GO:0071310 feature_id[760].value > threshold=2.3522024154663086
node_306: feature_name=GO:0070233 feature_id[321].value > threshold=1.293377935886383
node_316: feature_name=GO:0009897 feature_id[38].value > threshold=0.002804968156851828
Class: negative genes

Rules_55 passed counts:25
node_0: feature_name=GO:0042113 feature_id[0].value > threshold=13.408552169799805
node_1454: feature_name=GO:0050851 feature_id[29].value > threshold=9.650307655334473
node_1534: feature_name=GO:1902166 feature_id[301].value <= threshold=0.303210511803627
node_1535: feature_name=GO:0042287 feature_id[20].value <= threshold=1.4291933178901672
node_1536: feature_name=GO:0023026 feature_id[69].value <= threshold=1.7696257829666138
node_1537: feature_name=GO:0048661 feature_id[733].value > threshold=3.2364230155944824

node_1539: feature_name=GO:0002263 feature_id[392].value > threshold=5.861323833465576
Class: negative genes

Rules_56 passed counts:24
node_0: feature_name=GO:0042113 feature_id[0].value > threshold=13.408552169799805
node_1454: feature_name=GO:0050851 feature_id[29].value <= threshold=9.650307655334473
node_1455: feature_name=GO:0006304 feature_id[510].value <= threshold=5.960662126541138
node_1456: feature_name=GO:0032673 feature_id[623].value <= threshold=4.092082738876343
node_1457: feature_name=GO:0002429 feature_id[748].value > threshold=4.626799821853638
node_1463: feature_name=GO:0015672 feature_id[573].value <= threshold=0.08226438239216805
node_1464: feature_name=GO:0038065 feature_id[670].value > threshold=0.3765842020511627
node_1468: feature_name=GO:0046685 feature_id[782].value <= threshold=2.9275591373443604
node_1469: feature_name=GO:0050897 feature_id[164].value <= threshold=2.1307075023651123
Class: positive genes

Rules_57 passed counts:24
node_0: feature_name=GO:0042113 feature_id[0].value <= threshold=13.408552169799805
node_1: feature_name=GO:0007568 feature_id[534].value <= threshold=5.0313897132873535
node_2: feature_name=GO:0002705 feature_id[541].value <= threshold=3.200145721435547
node_3: feature_name=GO:1901525 feature_id[576].value <= threshold=0.4466460347175598
node_4: feature_name=GO:0048539 feature_id[319].value <= threshold=3.0399646759033203
node_5: feature_name=GO:0001910 feature_id[385].value <= threshold=3.7437864542007446
node_6: feature_name=GO:0043200 feature_id[706].value <= threshold=9.307284355163574
node_7: feature_name=GO:0001773 feature_id[308].value <= threshold=4.016931533813477
node_8: feature_name=GO:0090116 feature_id[97].value <= threshold=7.99645471572876
node_9: feature_name=GO:0019814 feature_id[189].value <= threshold=4.525782108306885
node_10: feature_name=GO:1902583 feature_id[215].value <= threshold=17.60310649871826
node_11: feature_name=GO:0045429 feature_id[737].value <= threshold=1.6133361458778381
node_12: feature_name=GO:0003720 feature_id[228].value <= threshold=5.519326210021973
node_13: feature_name=GO:0046006 feature_id[364].value <= threshold=4.9985432624816895
node_14: feature_name=GO:0070424 feature_id[254].value <= threshold=6.233297109603882
node_15: feature_name=GO:0009892 feature_id[320].value <= threshold=59.99180793762207
node_16: feature_name=GO:0007064 feature_id[527].value <= threshold=11.57377815246582
node_17: feature_name=GO:0005575 feature_id[17].value <= threshold=15.417460918426514
node_18: feature_name=GO:0043368 feature_id[31].value <= threshold=4.059496641159058

node_19: feature_name=GO:0005164 feature_id[163].value <= threshold=5.793607711791992
node_20: feature_name=GO:0042130 feature_id[16].value <= threshold=5.073179721832275
node_21: feature_name=GO:0010216 feature_id[282].value <= threshold=4.834584474563599
node_22: feature_name=GO:0009628 feature_id[553].value <= threshold=29.314892768859863
node_23: feature_name=GO:0045628 feature_id[749].value <= threshold=3.1001367568969727
node_24: feature_name=GO:0042288 feature_id[100].value <= threshold=5.174932956695557
node_25: feature_name=GO:0002329 feature_id[406].value <= threshold=2.348930835723877
node_26: feature_name=GO:0051246 feature_id[642].value <= threshold=0.6498909294605255
node_27: feature_name=GO:0042162 feature_id[355].value <= threshold=8.05103588104248
node_28: feature_name=hsa04668 feature_id[333].value <= threshold=5.614926338195801
node_29: feature_name=GO:0032200 feature_id[613].value <= threshold=28.735913276672363
node_30: feature_name=hsa04662 feature_id[334].value <= threshold=9.588344097137451
node_31: feature_name=GO:1901992 feature_id[105].value <= threshold=5.291427850723267
node_32: feature_name=GO:0072341 feature_id[144].value <= threshold=6.01465106010437
node_33: feature_name=GO:0047485 feature_id[121].value <= threshold=6.657836437225342
node_34: feature_name=GO:0090594 feature_id[738].value <= threshold=1.8673912286758423
node_35: feature_name=GO:0043525 feature_id[524].value <= threshold=2.881397008895874
node_36: feature_name=GO:0032504 feature_id[244].value <= threshold=17.22207546234131
node_37: feature_name=GO:0051348 feature_id[126].value <= threshold=5.933559417724609
node_38: feature_name=GO:0042522 feature_id[692].value <= threshold=1.78694087266922
node_39: feature_name=GO:0031100 feature_id[73].value <= threshold=3.2312101125717163
node_40: feature_name=GO:0042493 feature_id[149].value <= threshold=19.771170616149902
node_41: feature_name=GO:0001552 feature_id[366].value <= threshold=2.1711617708206177
node_42: feature_name=GO:0046483 feature_id[391].value <= threshold=320.9667053222656
node_43: feature_name=GO:0002439 feature_id[431].value <= threshold=1.832722783088684
node_44: feature_name=GO:0006555 feature_id[514].value <= threshold=5.321640968322754
node_45: feature_name=GO:0070424 feature_id[254].value <= threshold=3.321003556251526
node_46: feature_name=hsa05144 feature_id[751].value > threshold=2.635499954223633
node_134: feature_name=GO:0010165 feature_id[330].value <= threshold=0.49422845244407654
Class: negative genes

Rules_58 passed counts:23
node_0: feature_name=GO:0042113 feature_id[0].value > threshold=13.408552169799805
node_1454: feature_name=GO:0050851 feature_id[29].value <= threshold=9.650307655334473
node_1455: feature_name=GO:0006304 feature_id[510].value <= threshold=5.960662126541138

node_1456: feature_name=GO:0032673 feature_id[623].value > threshold=4.092082738876343
node_1510: feature_name=GO:0046898 feature_id[161].value > threshold=2.2367727756500244
node_1528: feature_name=GO:0038001 feature_id[28].value <= threshold=4.990844249725342
Class: positive genes

Rules_59 passed counts:21
node_0: feature_name=GO:0042113 feature_id[0].value > threshold=13.408552169799805
node_1454: feature_name=GO:0050851 feature_id[29].value <= threshold=9.650307655334473
node_1455: feature_name=GO:0006304 feature_id[510].value <= threshold=5.960662126541138
node_1456: feature_name=GO:0032673 feature_id[623].value > threshold=4.092082738876343
node_1510: feature_name=GO:0046898 feature_id[161].value <= threshold=2.2367727756500244
node_1511: feature_name=GO:0030183 feature_id[590].value > threshold=12.193635940551758
node_1521: feature_name=GO:0032504 feature_id[244].value > threshold=0.8662047982215881
node_1523: feature_name=GO:0045589 feature_id[347].value > threshold=3.9446849822998047
node_1525: feature_name=GO:2001235 feature_id[495].value <= threshold=15.975362300872803
Class: negative genes

Rules_60 passed counts:21
node_0: feature_name=GO:0042113 feature_id[0].value > threshold=13.408552169799805
node_1454: feature_name=GO:0050851 feature_id[29].value <= threshold=9.650307655334473
node_1455: feature_name=GO:0006304 feature_id[510].value <= threshold=5.960662126541138
node_1456: feature_name=GO:0032673 feature_id[623].value > threshold=4.092082738876343
node_1510: feature_name=GO:0046898 feature_id[161].value <= threshold=2.2367727756500244
node_1511: feature_name=GO:0030183 feature_id[590].value <= threshold=12.193635940551758
node_1512: feature_name=GO:0032480 feature_id[525].value > threshold=0.5560729652643204
node_1514: feature_name=GO:1902554 feature_id[660].value <= threshold=1.3245088458061218
node_1515: feature_name=GO:0042130 feature_id[16].value > threshold=2.3747211694717407
Class: positive genes

Rules_61 passed counts:21
node_0: feature_name=GO:0042113 feature_id[0].value <= threshold=13.408552169799805
node_1: feature_name=GO:0007568 feature_id[534].value <= threshold=5.0313897132873535
node_2: feature_name=GO:0002705 feature_id[541].value <= threshold=3.200145721435547
node_3: feature_name=GO:1901525 feature_id[576].value <= threshold=0.4466460347175598
node_4: feature_name=GO:0048539 feature_id[319].value <= threshold=3.0399646759033203

node_5: feature_name=GO:0001910 feature_id[385].value <= threshold=3.7437864542007446
node_6: feature_name=GO:0043200 feature_id[706].value <= threshold=9.307284355163574
node_7: feature_name=GO:0001773 feature_id[308].value <= threshold=4.016931533813477
node_8: feature_name=GO:0090116 feature_id[97].value <= threshold=7.99645471572876
node_9: feature_name=GO:0019814 feature_id[189].value <= threshold=4.525782108306885
node_10: feature_name=GO:1902583 feature_id[215].value <= threshold=17.60310649871826
node_11: feature_name=GO:0045429 feature_id[737].value <= threshold=1.6133361458778381
node_12: feature_name=GO:0003720 feature_id[228].value <= threshold=5.519326210021973
node_13: feature_name=GO:0046006 feature_id[364].value <= threshold=4.9985432624816895
node_14: feature_name=GO:0070424 feature_id[254].value <= threshold=6.233297109603882
node_15: feature_name=GO:0009892 feature_id[320].value <= threshold=59.99180793762207
node_16: feature_name=GO:0007064 feature_id[527].value <= threshold=11.57377815246582
node_17: feature_name=GO:0005575 feature_id[17].value <= threshold=15.417460918426514
node_18: feature_name=GO:0043368 feature_id[31].value <= threshold=4.059496641159058
node_19: feature_name=GO:0005164 feature_id[163].value <= threshold=5.793607711791992
node_20: feature_name=GO:0042130 feature_id[16].value <= threshold=5.073179721832275
node_21: feature_name=GO:0010216 feature_id[282].value <= threshold=4.834584474563599
node_22: feature_name=GO:0009628 feature_id[553].value <= threshold=29.314892768859863
node_23: feature_name=GO:0045628 feature_id[749].value <= threshold=3.1001367568969727
node_24: feature_name=GO:0042288 feature_id[100].value <= threshold=5.174932956695557
node_25: feature_name=GO:0002329 feature_id[406].value <= threshold=2.348930835723877
node_26: feature_name=GO:0051246 feature_id[642].value <= threshold=0.6498909294605255
node_27: feature_name=GO:0042162 feature_id[355].value <= threshold=8.05103588104248
node_28: feature_name=hsa04668 feature_id[333].value <= threshold=5.614926338195801
node_29: feature_name=GO:0032200 feature_id[613].value <= threshold=28.735913276672363
node_30: feature_name=hsa04662 feature_id[334].value <= threshold=9.588344097137451
node_31: feature_name=GO:1901992 feature_id[105].value <= threshold=5.291427850723267
node_32: feature_name=GO:0072341 feature_id[144].value <= threshold=6.01465106010437
node_33: feature_name=GO:0047485 feature_id[121].value <= threshold=6.657836437225342
node_34: feature_name=GO:0090594 feature_id[738].value <= threshold=1.8673912286758423
node_35: feature_name=GO:0043525 feature_id[524].value <= threshold=2.881397008895874
node_36: feature_name=GO:0032504 feature_id[244].value <= threshold=17.22207546234131
node_37: feature_name=GO:0051348 feature_id[126].value <= threshold=5.933559417724609
node_38: feature_name=GO:0042522 feature_id[692].value <= threshold=1.78694087266922
node_39: feature_name=GO:0031100 feature_id[73].value <= threshold=3.2312101125717163

node_40: feature_name=GO:0042493 feature_id[149].value <= threshold=19.771170616149902
node_41: feature_name=GO:0001552 feature_id[366].value <= threshold=2.1711617708206177
node_42: feature_name=GO:0046483 feature_id[391].value <= threshold=320.9667053222656
node_43: feature_name=GO:0002439 feature_id[431].value <= threshold=1.832722783088684
node_44: feature_name=GO:0006555 feature_id[514].value > threshold=5.321640968322754
node_142: feature_name=GO:0006555 feature_id[514].value > threshold=6.031885147094727
Class: negative genes

Rules_62 passed counts:21
node_0: feature_name=GO:0042113 feature_id[0].value <= threshold=13.408552169799805
node_1: feature_name=GO:0007568 feature_id[534].value <= threshold=5.0313897132873535
node_2: feature_name=GO:0002705 feature_id[541].value <= threshold=3.200145721435547
node_3: feature_name=GO:1901525 feature_id[576].value <= threshold=0.4466460347175598
node_4: feature_name=GO:0048539 feature_id[319].value <= threshold=3.0399646759033203
node_5: feature_name=GO:0001910 feature_id[385].value <= threshold=3.7437864542007446
node_6: feature_name=GO:0043200 feature_id[706].value <= threshold=9.307284355163574
node_7: feature_name=GO:0001773 feature_id[308].value <= threshold=4.016931533813477
node_8: feature_name=GO:0090116 feature_id[97].value <= threshold=7.99645471572876
node_9: feature_name=GO:0019814 feature_id[189].value <= threshold=4.525782108306885
node_10: feature_name=GO:1902583 feature_id[215].value <= threshold=17.60310649871826
node_11: feature_name=GO:0045429 feature_id[737].value <= threshold=1.6133361458778381
node_12: feature_name=GO:0003720 feature_id[228].value <= threshold=5.519326210021973
node_13: feature_name=GO:0046006 feature_id[364].value <= threshold=4.9985432624816895
node_14: feature_name=GO:0070424 feature_id[254].value <= threshold=6.233297109603882
node_15: feature_name=GO:0009892 feature_id[320].value <= threshold=59.99180793762207
node_16: feature_name=GO:0007064 feature_id[527].value <= threshold=11.57377815246582
node_17: feature_name=GO:0005575 feature_id[17].value <= threshold=15.417460918426514
node_18: feature_name=GO:0043368 feature_id[31].value <= threshold=4.059496641159058
node_19: feature_name=GO:0005164 feature_id[163].value <= threshold=5.793607711791992
node_20: feature_name=GO:0042130 feature_id[16].value <= threshold=5.073179721832275
node_21: feature_name=GO:0010216 feature_id[282].value <= threshold=4.834584474563599
node_22: feature_name=GO:0009628 feature_id[553].value <= threshold=29.314892768859863
node_23: feature_name=GO:0045628 feature_id[749].value <= threshold=3.1001367568969727
node_24: feature_name=GO:0042288 feature_id[100].value <= threshold=5.174932956695557
node_25: feature_name=GO:0002329 feature_id[406].value <= threshold=2.348930835723877

node_26: feature_name=GO:0051246 feature_id[642].value <= threshold=0.6498909294605255
node_27: feature_name=GO:0042162 feature_id[355].value <= threshold=8.05103588104248
node_28: feature_name=hsa04668 feature_id[333].value <= threshold=5.614926338195801
node_29: feature_name=GO:0032200 feature_id[613].value <= threshold=28.735913276672363
node_30: feature_name=hsa04662 feature_id[334].value <= threshold=9.588344097137451
node_31: feature_name=GO:1901992 feature_id[105].value <= threshold=5.291427850723267
node_32: feature_name=GO:0072341 feature_id[144].value <= threshold=6.01465106010437
node_33: feature_name=GO:0047485 feature_id[121].value <= threshold=6.657836437225342
node_34: feature_name=GO:0090594 feature_id[738].value <= threshold=1.8673912286758423
node_35: feature_name=GO:0043525 feature_id[524].value <= threshold=2.881397008895874
node_36: feature_name=GO:0032504 feature_id[244].value <= threshold=17.22207546234131
node_37: feature_name=GO:0051348 feature_id[126].value <= threshold=5.933559417724609
node_38: feature_name=GO:0042522 feature_id[692].value <= threshold=1.78694087266922
node_39: feature_name=GO:0031100 feature_id[73].value <= threshold=3.2312101125717163
node_40: feature_name=GO:0042493 feature_id[149].value <= threshold=19.771170616149902
node_41: feature_name=GO:0001552 feature_id[366].value <= threshold=2.1711617708206177
node_42: feature_name=GO:0046483 feature_id[391].value <= threshold=320.9667053222656
node_43: feature_name=GO:0002439 feature_id[431].value <= threshold=1.832722783088684
node_44: feature_name=GO:0006555 feature_id[514].value <= threshold=5.321640968322754
node_45: feature_name=GO:0070424 feature_id[254].value <= threshold=3.321003556251526
node_46: feature_name=hsa05144 feature_id[751].value <= threshold=2.635499954223633
node_47: feature_name=GO:0032633 feature_id[627].value <= threshold=1.2592533230781555
node_48: feature_name=GO:0019814 feature_id[189].value <= threshold=2.1583125591278076
node_49: feature_name=GO:1901989 feature_id[30].value <= threshold=3.3150794506073
node_50: feature_name=GO:1903318 feature_id[196].value <= threshold=1.62252539396286
node_51: feature_name=GO:0060576 feature_id[204].value <= threshold=2.1097792387008667
node_52: feature_name=hsa05221 feature_id[349].value <= threshold=0.8723124265670776
node_53: feature_name=GO:1903896 feature_id[135].value <= threshold=1.397229254245758
node_54: feature_name=GO:0097028 feature_id[443].value <= threshold=1.2365349531173706
node_55: feature_name=GO:0048660 feature_id[403].value > threshold=1.9425984621047974
node_85: feature_name=GO:0002682 feature_id[458].value > threshold=0.005244476022198796
Class: negative genes

Rules_63 passed counts:20
node_0: feature_name=GO:0042113 feature_id[0].value > threshold=13.408552169799805

node_1454: feature_name=GO:0050851	feature_id[29].value > threshold=9.650307655334473
node_1534: feature_name=GO:1902166	feature_id[301].value > threshold=0.303210511803627
node_1548: feature_name=GO:0035872	feature_id[659].value <= threshold=35.09038162231445
node_1549: feature_name=GO:0032069	feature_id[614].value <= threshold=3.441983938217163
node_1550: feature_name=GO:0050897	feature_id[164].value <= threshold=1.3939869403839111
node_1551: feature_name=GO:0051454	feature_id[307].value <= threshold=0.9144491851329803
node_1552: feature_name=GO:0035825	feature_id[662].value > threshold=0.0855268123745918
node_1556: feature_name=GO:0031265	feature_id[48].value > threshold=0.7173363864421844
node_1560: feature_name=GO:0002562	feature_id[25].value > threshold=5.642775535583496
node_1562: feature_name=hsa04660	feature_id[472].value > threshold=17.786678314208984
Class: positive genes	

Rules_64	passed counts:20
node_0: feature_name=GO:0042113	feature_id[0].value <= threshold=13.408552169799805
node_1: feature_name=GO:0007568	feature_id[534].value > threshold=5.0313897132873535
node_913: feature_name=GO:0032763	feature_id[629].value > threshold=0.31753237545490265
node_1195: feature_name=GO:0071301	feature_id[442].value <= threshold=0.11534593254327774
node_1196: feature_name=GO:0046500	feature_id[171].value <= threshold=0.6314916908740997
node_1197: feature_name=GO:0030852	feature_id[22].value <= threshold=2.9768868684768677
node_1198: feature_name=hsa04672	feature_id[276].value <= threshold=8.06681227684021
node_1199: feature_name=GO:0001836	feature_id[450].value > threshold=0.09961023926734924
node_1201: feature_name=GO:0070230	feature_id[64].value <= threshold=3.3956379890441895
node_1202: feature_name=GO:0070102	feature_id[49].value <= threshold=4.805386543273926
node_1203: feature_name=GO:0045553	feature_id[736].value <= threshold=1.4970332980155945
node_1204: feature_name=GO:0051311	feature_id[58].value <= threshold=3.301889181137085
node_1205: feature_name=GO:0030291	feature_id[147].value <= threshold=8.156315326690674
node_1206: feature_name=GO:0045637	feature_id[241].value > threshold=0.01834342861548066
node_1208: feature_name=GO:0002890	feature_id[98].value <= threshold=2.767483353614807
node_1209: feature_name=GO:0046632	feature_id[248].value <= threshold=11.663254261016846
node_1210: feature_name=GO:0008588	feature_id[26].value <= threshold=1.391375720500946
node_1211: feature_name=GO:0010639	feature_id[564].value <= threshold=18.975126266479492
node_1212: feature_name=GO:0046685	feature_id[782].value > threshold=5.153738260269165
node_1296: feature_name=GO:0019692	feature_id[584].value > threshold=0.0685717724263668
node_1300: feature_name=GO:0032649	feature_id[626].value > threshold=0.2627730891108513
Class: negative genes	

Rules_65	passed counts:19
node_0: feature_name=GO:0042113	feature_id[0].value <= threshold=13.408552169799805
node_1: feature_name=GO:0007568	feature_id[534].value <= threshold=5.0313897132873535
node_2: feature_name=GO:0002705	feature_id[541].value <= threshold=3.200145721435547
node_3: feature_name=GO:1901525	feature_id[576].value > threshold=0.4466460347175598
node_617: feature_name=GO:0005622	feature_id[233].value <= threshold=90.85730743408203
node_618: feature_name=GO:1903147	feature_id[200].value <= threshold=7.326428413391113
node_619: feature_name=GO:0003964	feature_id[317].value <= threshold=5.575642108917236
node_620: feature_name=GO:0046498	feature_id[203].value <= threshold=6.620223522186279
node_621: feature_name=GO:0002562	feature_id[25].value <= threshold=10.287704467773438
node_622: feature_name=GO:0006919	feature_id[246].value <= threshold=5.801334381103516
node_623: feature_name=GO:0001889	feature_id[387].value <= threshold=2.679414987564087
node_624: feature_name=GO:0006555	feature_id[514].value <= threshold=7.819535493850708
node_625: feature_name=GO:0080134	feature_id[650].value <= threshold=38.91967582702637
node_626: feature_name=GO:0042127	feature_id[480].value <= threshold=37.72536659240723
node_627: feature_name=GO:2001251	feature_id[220].value <= threshold=32.564422607421875
node_628: feature_name=GO:0002829	feature_id[493].value <= threshold=2.444612741470337
node_629: feature_name=GO:0008588	feature_id[26].value <= threshold=2.3601274490356445
node_630: feature_name=GO:0033151	feature_id[7].value <= threshold=5.134376287460327
node_631: feature_name=GO:0048145	feature_id[348].value <= threshold=8.836549282073975
node_632: feature_name=GO:0030887	feature_id[274].value <= threshold=1.7000296115875244
node_633: feature_name=GO:0097190	feature_id[117].value <= threshold=33.02078819274902
node_634: feature_name=GO:0046685	feature_id[782].value <= threshold=8.040813446044922
node_635: feature_name=GO:0006346	feature_id[236].value <= threshold=6.384642839431763
node_636: feature_name=GO:0048537	feature_id[66].value <= threshold=3.1739262342453003
node_637: feature_name=GO:0070245	feature_id[326].value <= threshold=3.0491198301315308
node_638: feature_name=GO:0038065	feature_id[670].value <= threshold=2.0590850114822388
node_639: feature_name=GO:0005488	feature_id[187].value <= threshold=84.80076217651367
node_640: feature_name=GO:0060249	feature_id[390].value > threshold=0.07226398587226868
node_652: feature_name=GO:0006974	feature_id[516].value > threshold=5.17782768838515e-06
node_654: feature_name=hsa05210	feature_id[358].value <= threshold=18.730005264282227
node_655: feature_name=GO:0071850	feature_id[40].value <= threshold=5.475317001342773
node_656: feature_name=GO:1904029	feature_id[208].value <= threshold=26.575013160705566
node_657: feature_name=GO:0090594	feature_id[738].value <= threshold=3.000791072845459

node_658: feature_name=GO:0010948 feature_id[569].value > threshold=0.04386143572628498
node_666: feature_name=GO:0032764 feature_id[3].value <= threshold=2.5201677083969116
node_667: feature_name=GO:0023026 feature_id[69].value <= threshold=3.2760006189346313
node_668: feature_name=GO:0032464 feature_id[83].value <= threshold=2.5927021503448486
node_669: feature_name=GO:0071901 feature_id[407].value <= threshold=18.238012313842773
node_670: feature_name=GO:0015671 feature_id[103].value <= threshold=1.3113192915916443
node_671: feature_name=GO:0008340 feature_id[539].value <= threshold=4.860779762268066
node_672: feature_name=GO:0046006 feature_id[364].value <= threshold=2.4317692518234253
node_673: feature_name=GO:0042991 feature_id[684].value <= threshold=5.553565502166748
node_674: feature_name=GO:0002327 feature_id[408].value <= threshold=2.404940366744995
node_675: feature_name=GO:0001836 feature_id[450].value <= threshold=8.647934436798096
node_676: feature_name=GO:0048539 feature_id[319].value <= threshold=1.6308802962303162
node_677: feature_name=GO:0022408 feature_id[279].value <= threshold=4.064727783203125
node_678: feature_name=GO:0030099 feature_id[598].value > threshold=0.0001809061213862151
node_684: feature_name=GO:0001816 feature_id[685].value > threshold=1.3516043054551119e-05
node_688: feature_name=GO:0043200 feature_id[706].value <= threshold=4.01608681678772
node_689: feature_name=GO:1904029 feature_id[208].value <= threshold=16.503823280334473
node_690: feature_name=GO:0045840 feature_id[697].value > threshold=6.23440408706665
node_692: feature_name=GO:0009615 feature_id[265].value <= threshold=1.909807026386261
Class: negative genes

Rules_66 passed counts:18
node_0: feature_name=GO:0042113 feature_id[0].value <= threshold=13.408552169799805
node_1: feature_name=GO:0007568 feature_id[534].value > threshold=5.0313897132873535
node_913: feature_name=GO:0032763 feature_id[629].value > threshold=0.31753237545490265
node_1195: feature_name=GO:0071301 feature_id[442].value > threshold=0.11534593254327774
node_1375: feature_name=GO:0046500 feature_id[171].value <= threshold=1.130434513092041
node_1376: feature_name=GO:0001782 feature_id[240].value <= threshold=6.639636993408203
node_1377: feature_name=GO:0043371 feature_id[707].value <= threshold=2.562113642692566
node_1378: feature_name=GO:0030291 feature_id[147].value <= threshold=6.834916830062866
node_1379: feature_name=GO:0045656 feature_id[756].value > threshold=0.6281269192695618
node_1393: feature_name=GO:0046007 feature_id[152].value > threshold=1.020785927772522
node_1419: feature_name=GO:0009636 feature_id[551].value <= threshold=15.796520233154297
node_1420: feature_name=GO:0008588 feature_id[26].value <= threshold=0.5794805586338043
Class: positive genes

Rules_67 passed counts:18
node_0: feature_name=GO:0042113 feature_id[0].value <= threshold=13.408552169799805
node_1: feature_name=GO:0007568 feature_id[534].value <= threshold=5.0313897132873535
node_2: feature_name=GO:0002705 feature_id[541].value <= threshold=3.200145721435547
node_3: feature_name=GO:1901525 feature_id[576].value > threshold=0.4466460347175598
node_617: feature_name=GO:0005622 feature_id[233].value <= threshold=90.85730743408203
node_618: feature_name=GO:1903147 feature_id[200].value <= threshold=7.326428413391113
node_619: feature_name=GO:0003964 feature_id[317].value <= threshold=5.575642108917236
node_620: feature_name=GO:0046498 feature_id[203].value <= threshold=6.620223522186279
node_621: feature_name=GO:0002562 feature_id[25].value <= threshold=10.287704467773438
node_622: feature_name=GO:0006919 feature_id[246].value <= threshold=5.801334381103516
node_623: feature_name=GO:0001889 feature_id[387].value <= threshold=2.679414987564087
node_624: feature_name=GO:0006555 feature_id[514].value <= threshold=7.819535493850708
node_625: feature_name=GO:0080134 feature_id[650].value <= threshold=38.91967582702637
node_626: feature_name=GO:0042127 feature_id[480].value <= threshold=37.72536659240723
node_627: feature_name=GO:2001251 feature_id[220].value <= threshold=32.564422607421875
node_628: feature_name=GO:0002829 feature_id[493].value <= threshold=2.444612741470337
node_629: feature_name=GO:0008588 feature_id[26].value <= threshold=2.3601274490356445
node_630: feature_name=GO:0033151 feature_id[7].value <= threshold=5.134376287460327
node_631: feature_name=GO:0048145 feature_id[348].value <= threshold=8.836549282073975
node_632: feature_name=GO:0030887 feature_id[274].value <= threshold=1.7000296115875244
node_633: feature_name=GO:0097190 feature_id[117].value <= threshold=33.02078819274902
node_634: feature_name=GO:0046685 feature_id[782].value <= threshold=8.040813446044922
node_635: feature_name=GO:0006346 feature_id[236].value <= threshold=6.384642839431763
node_636: feature_name=GO:0048537 feature_id[66].value <= threshold=3.1739262342453003
node_637: feature_name=GO:0070245 feature_id[326].value <= threshold=3.0491198301315308
node_638: feature_name=GO:0038065 feature_id[670].value <= threshold=2.0590850114822388
node_639: feature_name=GO:0005488 feature_id[187].value > threshold=84.80076217651367
node_755: feature_name=GO:0031049 feature_id[5].value <= threshold=0.707693487405777
node_756: feature_name=GO:0006306 feature_id[345].value <= threshold=15.554558753967285
Class: negative genes

Rules_68 passed counts:18
node_0: feature_name=GO:0042113 feature_id[0].value <= threshold=13.408552169799805

node_1: feature_name=GO:0007568 feature_id[534].value <= threshold=5.0313897132873535
node_2: feature_name=GO:0002705 feature_id[541].value <= threshold=3.200145721435547
node_3: feature_name=GO:1901525 feature_id[576].value <= threshold=0.4466460347175598
node_4: feature_name=GO:0048539 feature_id[319].value <= threshold=3.0399646759033203
node_5: feature_name=GO:0001910 feature_id[385].value <= threshold=3.7437864542007446
node_6: feature_name=GO:0043200 feature_id[706].value <= threshold=9.307284355163574
node_7: feature_name=GO:0001773 feature_id[308].value <= threshold=4.016931533813477
node_8: feature_name=GO:0090116 feature_id[97].value <= threshold=7.99645471572876
node_9: feature_name=GO:0019814 feature_id[189].value <= threshold=4.525782108306885
node_10: feature_name=GO:1902583 feature_id[215].value <= threshold=17.60310649871826
node_11: feature_name=GO:0045429 feature_id[737].value > threshold=1.6133361458778381
node_477: feature_name=GO:0036037 feature_id[445].value <= threshold=1.7181594371795654
node_478: feature_name=GO:0006244 feature_id[503].value <= threshold=0.951388418674469
node_479: feature_name=GO:0032461 feature_id[608].value <= threshold=2.6103241443634033
node_480: feature_name=GO:0044710 feature_id[719].value > threshold=2.228096718681627e-06
node_494: feature_name=GO:0009314 feature_id[296].value <= threshold=34.93696117401123
node_495: feature_name=GO:0050778 feature_id[802].value <= threshold=39.29364013671875
node_496: feature_name=GO:1903038 feature_id[492].value <= threshold=5.849650859832764
node_497: feature_name=GO:0002699 feature_id[606].value <= threshold=5.5405943393707275
node_498: feature_name=hsa00983 feature_id[181].value <= threshold=25.851045608520508
node_499: feature_name=GO:0002309 feature_id[416].value <= threshold=1.6235689520835876
node_500: feature_name=GO:1901698 feature_id[792].value <= threshold=47.70275688171387
node_501: feature_name=GO:0010216 feature_id[282].value <= threshold=1.6849713921546936
node_502: feature_name=GO:0006266 feature_id[297].value <= threshold=2.7199249267578125
node_503: feature_name=GO:0005488 feature_id[187].value > threshold=3.0373169010999845e-06
node_507: feature_name=GO:0045429 feature_id[737].value > threshold=1.614579975605011
node_511: feature_name=GO:0034103 feature_id[318].value <= threshold=5.832815647125244
node_512: feature_name=GO:0010663 feature_id[561].value <= threshold=4.157005429267883
node_513: feature_name=GO:0030887 feature_id[274].value <= threshold=1.8964150547981262
node_514: feature_name=GO:0048294 feature_id[113].value <= threshold=1.866134524345398
node_515: feature_name=GO:0072539 feature_id[68].value <= threshold=0.8600535988807678
node_516: feature_name=GO:0045840 feature_id[697].value <= threshold=4.963376045227051
node_517: feature_name=GO:0019222 feature_id[129].value <= threshold=4.0122095015249215e-06
node_518: feature_name=GO:0007568 feature_id[534].value <= threshold=3.2433676719665527
Class: negative genes

Rules_69	passed counts:17
node_0: feature_name=GO:0042113	feature_id[0].value <= threshold=13.408552169799805
node_1: feature_name=GO:0007568	feature_id[534].value > threshold=5.0313897132873535
node_913: feature_name=GO:0032763	feature_id[629].value > threshold=0.31753237545490265
node_1195: feature_name=GO:0071301	feature_id[442].value > threshold=0.11534593254327774
node_1375: feature_name=GO:0046500	feature_id[171].value <= threshold=1.130434513092041
node_1376: feature_name=GO:0001782	feature_id[240].value <= threshold=6.639636993408203
node_1377: feature_name=GO:0043371	feature_id[707].value <= threshold=2.562113642692566
node_1378: feature_name=GO:0030291	feature_id[147].value > threshold=6.834916830062866
node_1424: feature_name=GO:0002439	feature_id[431].value <= threshold=0.6129993498325348
node_1425: feature_name=GO:0042162	feature_id[355].value > threshold=0.09355577081441879
Class: positive genes	

Rules_70	passed counts:17
node_0: feature_name=GO:0042113	feature_id[0].value <= threshold=13.408552169799805
node_1: feature_name=GO:0007568	feature_id[534].value > threshold=5.0313897132873535
node_913: feature_name=GO:0032763	feature_id[629].value <= threshold=0.31753237545490265
node_914: feature_name=GO:0097193	feature_id[167].value <= threshold=28.171168327331543
node_915: feature_name=GO:0002903	feature_id[10].value <= threshold=1.0534588098526
node_916: feature_name=GO:1904029	feature_id[208].value <= threshold=23.852136611938477
node_917: feature_name=GO:0050897	feature_id[164].value <= threshold=0.6870408356189728
node_918: feature_name=GO:0006139	feature_id[474].value > threshold=1.3052097624921544e-07
node_920: feature_name=GO:0002821	feature_id[588].value <= threshold=13.81072187423706
node_921: feature_name=GO:0006298	feature_id[621].value <= threshold=24.078600883483887
node_922: feature_name=GO:0003908	feature_id[11].value <= threshold=1.7776933312416077
node_923: feature_name=GO:0030098	feature_id[273].value > threshold=0.0079949083738029
node_927: feature_name=GO:0006808	feature_id[513].value <= threshold=4.34592080116272
node_928: feature_name=GO:0071887	feature_id[283].value <= threshold=10.353787899017334
node_929: feature_name=GO:0038001	feature_id[28].value <= threshold=3.9469382762908936
node_930: feature_name=GO:0042287	feature_id[20].value <= threshold=3.6209195852279663
node_931: feature_name=GO:0003968	feature_id[47].value <= threshold=2.004227638244629
node_932: feature_name=GO:0002698	feature_id[395].value <= threshold=18.51447582244873
node_933: feature_name=GO:0044710	feature_id[719].value <= threshold=179.7340316772461
node_934: feature_name=GO:0007568	feature_id[534].value > threshold=5.035318374633789

node_936: feature_name=GO:0006216 feature_id[504].value <= threshold=2.2605666518211365
node_937: feature_name=GO:0048569 feature_id[793].value <= threshold=5.340231895446777
node_938: feature_name=GO:0001777 feature_id[380].value <= threshold=4.100832939147949
node_939: feature_name=GO:0007600 feature_id[122].value <= threshold=171.13383102416992
node_940: feature_name=GO:0001779 feature_id[378].value <= threshold=6.234851121902466
node_941: feature_name=GO:0030291 feature_id[147].value <= threshold=10.8051118850708
node_942: feature_name=GO:0048534 feature_id[790].value > threshold=0.017984486185014248
node_944: feature_name=GO:0070245 feature_id[326].value <= threshold=3.1282339096069336
node_945: feature_name=GO:0009086 feature_id[547].value <= threshold=0.2896959036588669
node_946: feature_name=GO:0001889 feature_id[387].value <= threshold=17.643128395080566
node_947: feature_name=GO:0048144 feature_id[192].value > threshold=0.20887330174446106
node_951: feature_name=GO:0023026 feature_id[69].value <= threshold=3.403424024581909
node_952: feature_name=GO:0048147 feature_id[788].value <= threshold=4.188283443450928
node_953: feature_name=GO:0090116 feature_id[97].value <= threshold=4.617154359817505
node_954: feature_name=GO:0002863 feature_id[490].value <= threshold=4.8968470096588135
node_955: feature_name=GO:0002524 feature_id[72].value <= threshold=3.035340189933777
node_956: feature_name=GO:0042130 feature_id[16].value <= threshold=7.285413980484009
node_957: feature_name=GO:0071456 feature_id[500].value <= threshold=21.212870597839355
node_958: feature_name=GO:2001238 feature_id[27].value <= threshold=7.849715709686279
node_959: feature_name=GO:0023030 feature_id[45].value <= threshold=1.7420591711997986
node_960: feature_name=GO:0072593 feature_id[381].value <= threshold=22.993029594421387
node_961: feature_name=GO:0034101 feature_id[646].value <= threshold=13.430044651031494
node_962: feature_name=GO:0097153 feature_id[226].value <= threshold=10.147814273834229
node_963: feature_name=GO:0001836 feature_id[450].value > threshold=7.252067804336548
node_977: feature_name=GO:1901030 feature_id[341].value > threshold=2.0671581029891968
Class: negative genes

Rules_71 passed counts:17
node_0: feature_name=GO:0042113 feature_id[0].value <= threshold=13.408552169799805
node_1: feature_name=GO:0007568 feature_id[534].value <= threshold=5.0313897132873535
node_2: feature_name=GO:0002705 feature_id[541].value <= threshold=3.200145721435547
node_3: feature_name=GO:1901525 feature_id[576].value > threshold=0.4466460347175598
node_617: feature_name=GO:0005622 feature_id[233].value <= threshold=90.85730743408203
node_618: feature_name=GO:1903147 feature_id[200].value <= threshold=7.326428413391113
node_619: feature_name=GO:0003964 feature_id[317].value <= threshold=5.575642108917236

node_620: feature_name=GO:0046498 feature_id[203].value <= threshold=6.620223522186279
node_621: feature_name=GO:0002562 feature_id[25].value <= threshold=10.287704467773438
node_622: feature_name=GO:0006919 feature_id[246].value <= threshold=5.801334381103516
node_623: feature_name=GO:0001889 feature_id[387].value <= threshold=2.679414987564087
node_624: feature_name=GO:0006555 feature_id[514].value <= threshold=7.819535493850708
node_625: feature_name=GO:0080134 feature_id[650].value <= threshold=38.91967582702637
node_626: feature_name=GO:0042127 feature_id[480].value <= threshold=37.72536659240723
node_627: feature_name=GO:2001251 feature_id[220].value <= threshold=32.564422607421875
node_628: feature_name=GO:0002829 feature_id[493].value <= threshold=2.444612741470337
node_629: feature_name=GO:0008588 feature_id[26].value <= threshold=2.3601274490356445
node_630: feature_name=GO:0033151 feature_id[7].value <= threshold=5.134376287460327
node_631: feature_name=GO:0048145 feature_id[348].value <= threshold=8.836549282073975
node_632: feature_name=GO:0030887 feature_id[274].value <= threshold=1.7000296115875244
node_633: feature_name=GO:0097190 feature_id[117].value <= threshold=33.02078819274902
node_634: feature_name=GO:0046685 feature_id[782].value <= threshold=8.040813446044922
node_635: feature_name=GO:0006346 feature_id[236].value <= threshold=6.384642839431763
node_636: feature_name=GO:0048537 feature_id[66].value <= threshold=3.1739262342453003
node_637: feature_name=GO:0070245 feature_id[326].value <= threshold=3.0491198301315308
node_638: feature_name=GO:0038065 feature_id[670].value <= threshold=2.0590850114822388
node_639: feature_name=GO:0005488 feature_id[187].value <= threshold=84.80076217651367
node_640: feature_name=GO:0060249 feature_id[390].value > threshold=0.07226398587226868
node_652: feature_name=GO:0006974 feature_id[516].value > threshold=5.17782768838515e-06
node_654: feature_name=hsa05210 feature_id[358].value <= threshold=18.730005264282227
node_655: feature_name=GO:0071850 feature_id[40].value <= threshold=5.475317001342773
node_656: feature_name=GO:1904029 feature_id[208].value <= threshold=26.575013160705566
node_657: feature_name=GO:0090594 feature_id[738].value <= threshold=3.000791072845459
node_658: feature_name=GO:0010948 feature_id[569].value > threshold=0.04386143572628498
node_666: feature_name=GO:0032764 feature_id[3].value <= threshold=2.5201677083969116
node_667: feature_name=GO:0023026 feature_id[69].value <= threshold=3.2760006189346313
node_668: feature_name=GO:0032464 feature_id[83].value <= threshold=2.5927021503448486
node_669: feature_name=GO:0071901 feature_id[407].value <= threshold=18.238012313842773
node_670: feature_name=GO:0015671 feature_id[103].value <= threshold=1.3113192915916443
node_671: feature_name=GO:0008340 feature_id[539].value <= threshold=4.860779762268066
node_672: feature_name=GO:0046006 feature_id[364].value <= threshold=2.4317692518234253
node_673: feature_name=GO:0042991 feature_id[684].value <= threshold=5.553565502166748

node_674: feature_name=GO:0002327	feature_id[408].value <= threshold=2.404940366744995
node_675: feature_name=GO:0001836	feature_id[450].value <= threshold=8.647934436798096
node_676: feature_name=GO:0048539	feature_id[319].value <= threshold=1.6308802962303162
node_677: feature_name=GO:0022408	feature_id[279].value <= threshold=4.064727783203125
node_678: feature_name=GO:0030099	feature_id[598].value > threshold=0.0001809061213862151
node_684: feature_name=GO:0001816	feature_id[685].value > threshold=1.3516043054551119e-05
node_688: feature_name=GO:0043200	feature_id[706].value <= threshold=4.01608681678772
node_689: feature_name=GO:1904029	feature_id[208].value > threshold=16.503823280334473
node_695: feature_name=GO:0007049	feature_id[259].value > threshold=10.547314643859863
Class: negative genes	
Rules_72	passed counts:16
node_0: feature_name=GO:0042113	feature_id[0].value > threshold=13.408552169799805
node_1454: feature_name=GO:0050851	feature_id[29].value <= threshold=9.650307655334473
node_1455: feature_name=GO:0006304	feature_id[510].value <= threshold=5.960662126541138
node_1456: feature_name=GO:0032673	feature_id[623].value <= threshold=4.092082738876343
node_1457: feature_name=GO:0002429	feature_id[748].value > threshold=4.626799821853638
node_1463: feature_name=GO:0015672	feature_id[573].value > threshold=0.08226438239216805
node_1475: feature_name=GO:0030217	feature_id[589].value <= threshold=19.77315902709961
node_1476: feature_name=GO:0002381	feature_id[96].value > threshold=4.645626783370972
node_1490: feature_name=GO:0042113	feature_id[0].value > threshold=17.225549697875977
Class: positive genes	
Rules_73	passed counts:16
node_0: feature_name=GO:0042113	feature_id[0].value <= threshold=13.408552169799805
node_1: feature_name=GO:0007568	feature_id[534].value > threshold=5.0313897132873535
node_913: feature_name=GO:0032763	feature_id[629].value > threshold=0.31753237545490265
node_1195: feature_name=GO:0071301	feature_id[442].value <= threshold=0.11534593254327774
node_1196: feature_name=GO:0046500	feature_id[171].value > threshold=0.6314916908740997
node_1340: feature_name=GO:0043627	feature_id[562].value > threshold=25.17277240753174
node_1368: feature_name=GO:0071310	feature_id[760].value <= threshold=80.00252914428711
node_1369: feature_name=GO:1901989	feature_id[30].value > threshold=1.159576177597046
Class: positive genes	
Rules_74	passed counts:16

node_0: feature_name=GO:0042113	feature_id[0].value <= threshold=13.408552169799805
node_1: feature_name=GO:0007568	feature_id[534].value > threshold=5.0313897132873535
node_913: feature_name=GO:0032763	feature_id[629].value > threshold=0.31753237545490265
node_1195: feature_name=GO:0071301	feature_id[442].value <= threshold=0.11534593254327774
node_1196: feature_name=GO:0046500	feature_id[171].value <= threshold=0.6314916908740997
node_1197: feature_name=GO:0030852	feature_id[22].value > threshold=2.9768868684768677
node_1329: feature_name=GO:0046668	feature_id[125].value <= threshold=0.9442969560623169
node_1330: feature_name=GO:0002467	feature_id[39].value <= threshold=2.990131974220276
Class: negative genes	

Rules_75	passed counts:16
node_0: feature_name=GO:0042113	feature_id[0].value <= threshold=13.408552169799805
node_1: feature_name=GO:0007568	feature_id[534].value <= threshold=5.0313897132873535
node_2: feature_name=GO:0002705	feature_id[541].value <= threshold=3.200145721435547
node_3: feature_name=GO:1901525	feature_id[576].value > threshold=0.4466460347175598
node_617: feature_name=GO:0005622	feature_id[233].value <= threshold=90.85730743408203
node_618: feature_name=GO:1903147	feature_id[200].value <= threshold=7.326428413391113
node_619: feature_name=GO:0003964	feature_id[317].value <= threshold=5.575642108917236
node_620: feature_name=GO:0046498	feature_id[203].value <= threshold=6.620223522186279
node_621: feature_name=GO:0002562	feature_id[25].value <= threshold=10.287704467773438
node_622: feature_name=GO:0006919	feature_id[246].value <= threshold=5.801334381103516
node_623: feature_name=GO:0001889	feature_id[387].value <= threshold=2.679414987564087
node_624: feature_name=GO:0006555	feature_id[514].value <= threshold=7.819535493850708
node_625: feature_name=GO:0080134	feature_id[650].value <= threshold=38.91967582702637
node_626: feature_name=GO:0042127	feature_id[480].value <= threshold=37.72536659240723
node_627: feature_name=GO:2001251	feature_id[220].value <= threshold=32.564422607421875
node_628: feature_name=GO:0002829	feature_id[493].value <= threshold=2.444612741470337
node_629: feature_name=GO:0008588	feature_id[26].value <= threshold=2.3601274490356445
node_630: feature_name=GO:0033151	feature_id[7].value <= threshold=5.134376287460327
node_631: feature_name=GO:0048145	feature_id[348].value <= threshold=8.836549282073975
node_632: feature_name=GO:0030887	feature_id[274].value <= threshold=1.7000296115875244
node_633: feature_name=GO:0097190	feature_id[117].value <= threshold=33.02078819274902
node_634: feature_name=GO:0046685	feature_id[782].value <= threshold=8.040813446044922
node_635: feature_name=GO:0006346	feature_id[236].value <= threshold=6.384642839431763
node_636: feature_name=GO:0048537	feature_id[66].value <= threshold=3.1739262342453003

node_637: feature_name=GO:0070245 feature_id[326].value <= threshold=3.0491198301315308
node_638: feature_name=GO:0038065 feature_id[670].value <= threshold=2.0590850114822388
node_639: feature_name=GO:0005488 feature_id[187].value <= threshold=84.80076217651367
node_640: feature_name=GO:0060249 feature_id[390].value > threshold=0.07226398587226868
node_652: feature_name=GO:0006974 feature_id[516].value > threshold=5.17782768838515e-06
node_654: feature_name=hsa05210 feature_id[358].value <= threshold=18.730005264282227
node_655: feature_name=GO:0071850 feature_id[40].value <= threshold=5.475317001342773
node_656: feature_name=GO:1904029 feature_id[208].value <= threshold=26.575013160705566
node_657: feature_name=GO:0090594 feature_id[738].value <= threshold=3.000791072845459
node_658: feature_name=GO:0010948 feature_id[569].value > threshold=0.04386143572628498
node_666: feature_name=GO:0032764 feature_id[3].value <= threshold=2.5201677083969116
node_667: feature_name=GO:0023026 feature_id[69].value <= threshold=3.2760006189346313
node_668: feature_name=GO:0032464 feature_id[83].value <= threshold=2.5927021503448486
node_669: feature_name=GO:0071901 feature_id[407].value <= threshold=18.238012313842773
node_670: feature_name=GO:0015671 feature_id[103].value <= threshold=1.3113192915916443
node_671: feature_name=GO:0008340 feature_id[539].value <= threshold=4.860779762268066
node_672: feature_name=GO:0046006 feature_id[364].value <= threshold=2.4317692518234253
node_673: feature_name=GO:0042991 feature_id[684].value <= threshold=5.553565502166748
node_674: feature_name=GO:0002327 feature_id[408].value > threshold=2.404940366744995
node_710: feature_name=GO:0010939 feature_id[142].value <= threshold=3.3515632152557373
Class: negative genes

Rules_76 passed counts:16
node_0: feature_name=GO:0042113 feature_id[0].value <= threshold=13.408552169799805
node_1: feature_name=GO:0007568 feature_id[534].value <= threshold=5.0313897132873535
node_2: feature_name=GO:0002705 feature_id[541].value <= threshold=3.200145721435547
node_3: feature_name=GO:1901525 feature_id[576].value <= threshold=0.4466460347175598
node_4: feature_name=GO:0048539 feature_id[319].value <= threshold=3.0399646759033203
node_5: feature_name=GO:0001910 feature_id[385].value <= threshold=3.7437864542007446
node_6: feature_name=GO:0043200 feature_id[706].value <= threshold=9.307284355163574
node_7: feature_name=GO:0001773 feature_id[308].value <= threshold=4.016931533813477
node_8: feature_name=GO:0090116 feature_id[97].value <= threshold=7.99645471572876
node_9: feature_name=GO:0019814 feature_id[189].value <= threshold=4.525782108306885
node_10: feature_name=GO:1902583 feature_id[215].value <= threshold=17.60310649871826
node_11: feature_name=GO:0045429 feature_id[737].value <= threshold=1.6133361458778381

node_12: feature_name=GO:0003720 feature_id[228].value <= threshold=5.519326210021973
node_13: feature_name=GO:0046006 feature_id[364].value <= threshold=4.9985432624816895
node_14: feature_name=GO:0070424 feature_id[254].value <= threshold=6.233297109603882
node_15: feature_name=GO:0009892 feature_id[320].value <= threshold=59.99180793762207
node_16: feature_name=GO:0007064 feature_id[527].value <= threshold=11.57377815246582
node_17: feature_name=GO:0005575 feature_id[17].value <= threshold=15.417460918426514
node_18: feature_name=GO:0043368 feature_id[31].value <= threshold=4.059496641159058
node_19: feature_name=GO:0005164 feature_id[163].value <= threshold=5.793607711791992
node_20: feature_name=GO:0042130 feature_id[16].value <= threshold=5.073179721832275
node_21: feature_name=GO:0010216 feature_id[282].value <= threshold=4.834584474563599
node_22: feature_name=GO:0009628 feature_id[553].value <= threshold=29.314892768859863
node_23: feature_name=GO:0045628 feature_id[749].value <= threshold=3.1001367568969727
node_24: feature_name=GO:0042288 feature_id[100].value <= threshold=5.174932956695557
node_25: feature_name=GO:0002329 feature_id[406].value <= threshold=2.348930835723877
node_26: feature_name=GO:0051246 feature_id[642].value <= threshold=0.6498909294605255
node_27: feature_name=GO:0042162 feature_id[355].value <= threshold=8.05103588104248
node_28: feature_name=hsa04668 feature_id[333].value <= threshold=5.614926338195801
node_29: feature_name=GO:0032200 feature_id[613].value <= threshold=28.735913276672363
node_30: feature_name=hsa04662 feature_id[334].value <= threshold=9.588344097137451
node_31: feature_name=GO:1901992 feature_id[105].value <= threshold=5.291427850723267
node_32: feature_name=GO:0072341 feature_id[144].value > threshold=6.01465106010437
node_180: feature_name=GO:0045621 feature_id[143].value <= threshold=0.2295876294374466
node_181: feature_name=GO:0002294 feature_id[419].value <= threshold=0.146267332136631
Class: negative genes

Rules_77 passed counts:15
node_0: feature_name=GO:0042113 feature_id[0].value <= threshold=13.408552169799805
node_1: feature_name=GO:0007568 feature_id[534].value <= threshold=5.0313897132873535
node_2: feature_name=GO:0002705 feature_id[541].value <= threshold=3.200145721435547
node_3: feature_name=GO:1901525 feature_id[576].value <= threshold=0.4466460347175598
node_4: feature_name=GO:0048539 feature_id[319].value <= threshold=3.0399646759033203
node_5: feature_name=GO:0001910 feature_id[385].value <= threshold=3.7437864542007446
node_6: feature_name=GO:0043200 feature_id[706].value <= threshold=9.307284355163574
node_7: feature_name=GO:0001773 feature_id[308].value <= threshold=4.016931533813477
node_8: feature_name=GO:0090116 feature_id[97].value <= threshold=7.99645471572876

node_9: feature_name=GO:0019814 feature_id[189].value <= threshold=4.525782108306885
node_10: feature_name=GO:1902583 feature_id[215].value <= threshold=17.60310649871826
node_11: feature_name=GO:0045429 feature_id[737].value <= threshold=1.6133361458778381
node_12: feature_name=GO:0003720 feature_id[228].value <= threshold=5.519326210021973
node_13: feature_name=GO:0046006 feature_id[364].value <= threshold=4.9985432624816895
node_14: feature_name=GO:0070424 feature_id[254].value <= threshold=6.233297109603882
node_15: feature_name=GO:0009892 feature_id[320].value <= threshold=59.99180793762207
node_16: feature_name=GO:0007064 feature_id[527].value <= threshold=11.57377815246582
node_17: feature_name=GO:0005575 feature_id[17].value <= threshold=15.417460918426514
node_18: feature_name=GO:0043368 feature_id[31].value <= threshold=4.059496641159058
node_19: feature_name=GO:0005164 feature_id[163].value <= threshold=5.793607711791992
node_20: feature_name=GO:0042130 feature_id[16].value <= threshold=5.073179721832275
node_21: feature_name=GO:0010216 feature_id[282].value <= threshold=4.834584474563599
node_22: feature_name=GO:0009628 feature_id[553].value <= threshold=29.314892768859863
node_23: feature_name=GO:0045628 feature_id[749].value <= threshold=3.1001367568969727
node_24: feature_name=GO:0042288 feature_id[100].value <= threshold=5.174932956695557
node_25: feature_name=GO:0002329 feature_id[406].value > threshold=2.348930835723877
node_449: feature_name=GO:0006725 feature_id[523].value > threshold=2.542858638321377e-06
Class: negative genes

Rules_78 passed counts:14
node_0: feature_name=GO:0042113 feature_id[0].value > threshold=13.408552169799805
node_1454: feature_name=GO:0050851 feature_id[29].value > threshold=9.650307655334473
node_1534: feature_name=GO:1902166 feature_id[301].value <= threshold=0.303210511803627
node_1535: feature_name=GO:0042287 feature_id[20].value > threshold=1.4291933178901672
node_1545: feature_name=GO:0006968 feature_id[517].value > threshold=0.4102974385023117
Class: positive genes

Rules_79 passed counts:14
node_0: feature_name=GO:0042113 feature_id[0].value <= threshold=13.408552169799805
node_1: feature_name=GO:0007568 feature_id[534].value <= threshold=5.0313897132873535
node_2: feature_name=GO:0002705 feature_id[541].value <= threshold=3.200145721435547
node_3: feature_name=GO:1901525 feature_id[576].value <= threshold=0.4466460347175598
node_4: feature_name=GO:0048539 feature_id[319].value <= threshold=3.0399646759033203
node_5: feature_name=GO:0001910 feature_id[385].value <= threshold=3.7437864542007446

node_6: feature_name=GO:0043200	feature_id[706].value <= threshold=9.307284355163574
node_7: feature_name=GO:0001773	feature_id[308].value <= threshold=4.016931533813477
node_8: feature_name=GO:0090116	feature_id[97].value <= threshold=7.99645471572876
node_9: feature_name=GO:0019814	feature_id[189].value <= threshold=4.525782108306885
node_10: feature_name=GO:1902583	feature_id[215].value <= threshold=17.60310649871826
node_11: feature_name=GO:0045429	feature_id[737].value <= threshold=1.6133361458778381
node_12: feature_name=GO:0003720	feature_id[228].value <= threshold=5.519326210021973
node_13: feature_name=GO:0046006	feature_id[364].value <= threshold=4.9985432624816895
node_14: feature_name=GO:0070424	feature_id[254].value <= threshold=6.233297109603882
node_15: feature_name=GO:0009892	feature_id[320].value <= threshold=59.99180793762207
node_16: feature_name=GO:0007064	feature_id[527].value <= threshold=11.57377815246582
node_17: feature_name=GO:0005575	feature_id[17].value <= threshold=15.417460918426514
node_18: feature_name=GO:0043368	feature_id[31].value <= threshold=4.059496641159058
node_19: feature_name=GO:0005164	feature_id[163].value <= threshold=5.793607711791992
node_20: feature_name=GO:0042130	feature_id[16].value <= threshold=5.073179721832275
node_21: feature_name=GO:0010216	feature_id[282].value <= threshold=4.834584474563599
node_22: feature_name=GO:0009628	feature_id[553].value <= threshold=29.314892768859863
node_23: feature_name=GO:0045628	feature_id[749].value <= threshold=3.1001367568969727
node_24: feature_name=GO:0042288	feature_id[100].value <= threshold=5.174932956695557
node_25: feature_name=GO:0002329	feature_id[406].value <= threshold=2.348930835723877
node_26: feature_name=GO:0051246	feature_id[642].value <= threshold=0.6498909294605255
node_27: feature_name=GO:0042162	feature_id[355].value <= threshold=8.05103588104248
node_28: feature_name=hsa04668	feature_id[333].value <= threshold=5.614926338195801
node_29: feature_name=GO:0032200	feature_id[613].value <= threshold=28.735913276672363
node_30: feature_name=hsa04662	feature_id[334].value <= threshold=9.588344097137451
node_31: feature_name=GO:1901992	feature_id[105].value <= threshold=5.291427850723267
node_32: feature_name=GO:0072341	feature_id[144].value <= threshold=6.01465106010437
node_33: feature_name=GO:0047485	feature_id[121].value <= threshold=6.657836437225342
node_34: feature_name=GO:0090594	feature_id[738].value <= threshold=1.8673912286758423
node_35: feature_name=GO:0043525	feature_id[524].value <= threshold=2.881397008895874
node_36: feature_name=GO:0032504	feature_id[244].value <= threshold=17.22207546234131
node_37: feature_name=GO:0051348	feature_id[126].value <= threshold=5.933559417724609
node_38: feature_name=GO:0042522	feature_id[692].value > threshold=1.78694087266922
node_162: feature_name=GO:0006113	feature_id[497].value <= threshold=1.119098722934723

Class: negative genes

Rules_80	passed counts:14
node_0: feature_name=GO:0042113	feature_id[0].value <= threshold=13.408552169799805
node_1: feature_name=GO:0007568	feature_id[534].value <= threshold=5.0313897132873535
node_2: feature_name=GO:0002705	feature_id[541].value <= threshold=3.200145721435547
node_3: feature_name=GO:1901525	feature_id[576].value <= threshold=0.4466460347175598
node_4: feature_name=GO:0048539	feature_id[319].value <= threshold=3.0399646759033203
node_5: feature_name=GO:0001910	feature_id[385].value <= threshold=3.7437864542007446
node_6: feature_name=GO:0043200	feature_id[706].value <= threshold=9.307284355163574
node_7: feature_name=GO:0001773	feature_id[308].value <= threshold=4.016931533813477
node_8: feature_name=GO:0090116	feature_id[97].value <= threshold=7.99645471572876
node_9: feature_name=GO:0019814	feature_id[189].value <= threshold=4.525782108306885
node_10: feature_name=GO:1902583	feature_id[215].value <= threshold=17.60310649871826
node_11: feature_name=GO:0045429	feature_id[737].value <= threshold=1.6133361458778381
node_12: feature_name=GO:0003720	feature_id[228].value <= threshold=5.519326210021973
node_13: feature_name=GO:0046006	feature_id[364].value <= threshold=4.9985432624816895
node_14: feature_name=GO:0070424	feature_id[254].value <= threshold=6.233297109603882
node_15: feature_name=GO:0009892	feature_id[320].value <= threshold=59.99180793762207
node_16: feature_name=GO:0007064	feature_id[527].value <= threshold=11.57377815246582
node_17: feature_name=GO:0005575	feature_id[17].value <= threshold=15.417460918426514
node_18: feature_name=GO:0043368	feature_id[31].value <= threshold=4.059496641159058
node_19: feature_name=GO:0005164	feature_id[163].value <= threshold=5.793607711791992
node_20: feature_name=GO:0042130	feature_id[16].value <= threshold=5.073179721832275
node_21: feature_name=GO:0010216	feature_id[282].value <= threshold=4.834584474563599
node_22: feature_name=GO:0009628	feature_id[553].value <= threshold=29.314892768859863
node_23: feature_name=GO:0045628	feature_id[749].value <= threshold=3.1001367568969727
node_24: feature_name=GO:0042288	feature_id[100].value <= threshold=5.174932956695557
node_25: feature_name=GO:0002329	feature_id[406].value <= threshold=2.348930835723877
node_26: feature_name=GO:0051246	feature_id[642].value <= threshold=0.6498909294605255
node_27: feature_name=GO:0042162	feature_id[355].value <= threshold=8.05103588104248
node_28: feature_name=hsa04668	feature_id[333].value <= threshold=5.614926338195801
node_29: feature_name=GO:0032200	feature_id[613].value <= threshold=28.735913276672363
node_30: feature_name=hsa04662	feature_id[334].value <= threshold=9.588344097137451
node_31: feature_name=GO:1901992	feature_id[105].value <= threshold=5.291427850723267
node_32: feature_name=GO:0072341	feature_id[144].value <= threshold=6.01465106010437

node_33: feature_name=GO:0047485 feature_id[121].value <= threshold=6.657836437225342
node_34: feature_name=GO:0090594 feature_id[738].value <= threshold=1.8673912286758423
node_35: feature_name=GO:0043525 feature_id[524].value <= threshold=2.881397008895874
node_36: feature_name=GO:0032504 feature_id[244].value <= threshold=17.22207546234131
node_37: feature_name=GO:0051348 feature_id[126].value <= threshold=5.933559417724609
node_38: feature_name=GO:0042522 feature_id[692].value <= threshold=1.78694087266922
node_39: feature_name=GO:0031100 feature_id[73].value > threshold=3.2312101125717163
node_157: feature_name=GO:0002376 feature_id[277].value <= threshold=0.046988584101200104
Class: negative genes

Rules_81 passed counts:13
node_0: feature_name=GO:0042113 feature_id[0].value <= threshold=13.408552169799805
node_1: feature_name=GO:0007568 feature_id[534].value > threshold=5.0313897132873535
node_913: feature_name=GO:0032763 feature_id[629].value > threshold=0.31753237545490265
node_1195: feature_name=GO:0071301 feature_id[442].value <= threshold=0.11534593254327774
node_1196: feature_name=GO:0046500 feature_id[171].value <= threshold=0.6314916908740997
node_1197: feature_name=GO:0030852 feature_id[22].value > threshold=2.9768868684768677
node_1329: feature_name=GO:0046668 feature_id[125].value > threshold=0.9442969560623169
node_1333: feature_name=GO:0006298 feature_id[621].value > threshold=0.45032089948654175
node_1337: feature_name=GO:0050868 feature_id[377].value > threshold=0.2427361086010933
Class: positive genes

Rules_82 passed counts:13
node_0: feature_name=GO:0042113 feature_id[0].value <= threshold=13.408552169799805
node_1: feature_name=GO:0007568 feature_id[534].value > threshold=5.0313897132873535
node_913: feature_name=GO:0032763 feature_id[629].value > threshold=0.31753237545490265
node_1195: feature_name=GO:0071301 feature_id[442].value <= threshold=0.11534593254327774
node_1196: feature_name=GO:0046500 feature_id[171].value <= threshold=0.6314916908740997
node_1197: feature_name=GO:0030852 feature_id[22].value <= threshold=2.9768868684768677
node_1198: feature_name=hsa04672 feature_id[276].value <= threshold=8.06681227684021
node_1199: feature_name=GO:0001836 feature_id[450].value > threshold=0.09961023926734924
node_1201: feature_name=GO:0070230 feature_id[64].value <= threshold=3.3956379890441895
node_1202: feature_name=GO:0070102 feature_id[49].value <= threshold=4.805386543273926
node_1203: feature_name=GO:0045553 feature_id[736].value <= threshold=1.4970332980155945
node_1204: feature_name=GO:0051311 feature_id[58].value <= threshold=3.301889181137085

node_1205: feature_name=GO:0030291 feature_id[147].value <= threshold=8.156315326690674
node_1206: feature_name=GO:0045637 feature_id[241].value > threshold=0.01834342861548066
node_1208: feature_name=GO:0002890 feature_id[98].value <= threshold=2.767483353614807
node_1209: feature_name=GO:0046632 feature_id[248].value <= threshold=11.663254261016846
node_1210: feature_name=GO:0008588 feature_id[26].value > threshold=1.391375720500946
node_1308: feature_name=GO:0002200 feature_id[82].value <= threshold=1.0774684846401215
Class: negative genes

Rules_83 passed counts:13
node_0: feature_name=GO:0042113 feature_id[0].value <= threshold=13.408552169799805
node_1: feature_name=GO:0007568 feature_id[534].value <= threshold=5.0313897132873535
node_2: feature_name=GO:0002705 feature_id[541].value <= threshold=3.200145721435547
node_3: feature_name=GO:1901525 feature_id[576].value <= threshold=0.4466460347175598
node_4: feature_name=GO:0048539 feature_id[319].value <= threshold=3.0399646759033203
node_5: feature_name=GO:0001910 feature_id[385].value <= threshold=3.7437864542007446
node_6: feature_name=GO:0043200 feature_id[706].value <= threshold=9.307284355163574
node_7: feature_name=GO:0001773 feature_id[308].value <= threshold=4.016931533813477
node_8: feature_name=GO:0090116 feature_id[97].value <= threshold=7.99645471572876
node_9: feature_name=GO:0019814 feature_id[189].value <= threshold=4.525782108306885
node_10: feature_name=GO:1902583 feature_id[215].value <= threshold=17.60310649871826
node_11: feature_name=GO:0045429 feature_id[737].value > threshold=1.6133361458778381
node_477: feature_name=GO:0036037 feature_id[445].value > threshold=1.7181594371795654
node_595: feature_name=GO:0015671 feature_id[103].value <= threshold=0.0885394886136055
node_596: feature_name=GO:0097193 feature_id[167].value <= threshold=2.440826654434204
Class: negative genes

Rules_84 passed counts:12
node_0: feature_name=GO:0042113 feature_id[0].value <= threshold=13.408552169799805
node_1: feature_name=GO:0007568 feature_id[534].value > threshold=5.0313897132873535
node_913: feature_name=GO:0032763 feature_id[629].value > threshold=0.31753237545490265
node_1195: feature_name=GO:0071301 feature_id[442].value <= threshold=0.11534593254327774
node_1196: feature_name=GO:0046500 feature_id[171].value > threshold=0.6314916908740997
node_1340: feature_name=GO:0043627 feature_id[562].value <= threshold=25.17277240753174
node_1341: feature_name=GO:0010835 feature_id[560].value <= threshold=1.0601619482040405
node_1342: feature_name=GO:0016363 feature_id[90].value <= threshold=6.534365892410278

node_1343: feature_name=GO:0030983 feature_id[291].value > threshold=3.5806901454925537
node_1359: feature_name=GO:0046632 feature_id[248].value > threshold=0.5181337743997574
node_1361: feature_name=GO:0033554 feature_id[418].value <= threshold=43.66302299499512
node_1362: feature_name=GO:2000773 feature_id[114].value > threshold=0.22911779582500458
Class: positive genes

Rules_85 passed counts:12
node_0: feature_name=GO:0042113 feature_id[0].value <= threshold=13.408552169799805
node_1: feature_name=GO:0007568 feature_id[534].value > threshold=5.0313897132873535
node_913: feature_name=GO:0032763 feature_id[629].value > threshold=0.31753237545490265
node_1195: feature_name=GO:0071301 feature_id[442].value <= threshold=0.11534593254327774
node_1196: feature_name=GO:0046500 feature_id[171].value <= threshold=0.6314916908740997
node_1197: feature_name=GO:0030852 feature_id[22].value <= threshold=2.9768868684768677
node_1198: feature_name=hsa04672 feature_id[276].value <= threshold=8.06681227684021
node_1199: feature_name=GO:0001836 feature_id[450].value > threshold=0.09961023926734924
node_1201: feature_name=GO:0070230 feature_id[64].value <= threshold=3.3956379890441895
node_1202: feature_name=GO:0070102 feature_id[49].value <= threshold=4.805386543273926
node_1203: feature_name=GO:0045553 feature_id[736].value <= threshold=1.4970332980155945
node_1204: feature_name=GO:0051311 feature_id[58].value <= threshold=3.301889181137085
node_1205: feature_name=GO:0030291 feature_id[147].value <= threshold=8.156315326690674
node_1206: feature_name=GO:0045637 feature_id[241].value > threshold=0.01834342861548066
node_1208: feature_name=GO:0002890 feature_id[98].value <= threshold=2.767483353614807
node_1209: feature_name=GO:0046632 feature_id[248].value <= threshold=11.663254261016846
node_1210: feature_name=GO:0008588 feature_id[26].value <= threshold=1.391375720500946
node_1211: feature_name=GO:0010639 feature_id[564].value <= threshold=18.975126266479492
node_1212: feature_name=GO:0046685 feature_id[782].value <= threshold=5.153738260269165
node_1213: feature_name=GO:0038116 feature_id[668].value <= threshold=1.7859655618667603
node_1214: feature_name=GO:0046449 feature_id[763].value <= threshold=5.603115558624268
node_1215: feature_name=GO:0048539 feature_id[319].value <= threshold=0.5478232800960541
node_1216: feature_name=GO:0007089 feature_id[522].value <= threshold=0.9940084517002106
node_1217: feature_name=GO:0042287 feature_id[20].value <= threshold=2.693045496940613
node_1218: feature_name=GO:0044446 feature_id[184].value <= threshold=113.0758171081543
node_1219: feature_name=GO:0098602 feature_id[702].value <= threshold=68.95328903198242
node_1220: feature_name=GO:0035726 feature_id[270].value <= threshold=1.7167096138000488
node_1221: feature_name=GO:1902564 feature_id[85].value <= threshold=2.8364468812942505

node_1222: feature_name=GO:0006979	feature_id[44].value > threshold=0.6869173645973206
node_1224: feature_name=GO:0044464	feature_id[56].value <= threshold=67.13260269165039
node_1225: feature_name=GO:0045629	feature_id[746].value <= threshold=2.3414241075515747
node_1226: feature_name=GO:0002903	feature_id[10].value <= threshold=2.715258240699768
node_1227: feature_name=GO:0043375	feature_id[717].value <= threshold=1.9525017738342285
node_1228: feature_name=GO:0032762	feature_id[630].value > threshold=0.29527929425239563
node_1232: feature_name=GO:0048569	feature_id[793].value > threshold=3.419509768486023
node_1246: feature_name=GO:0032649	feature_id[626].value > threshold=0.7534940838813782
Class: negative genes	
Rules_86	passed counts:12
node_0: feature_name=GO:0042113	feature_id[0].value <= threshold=13.408552169799805
node_1: feature_name=GO:0007568	feature_id[534].value > threshold=5.0313897132873535
node_913: feature_name=GO:0032763	feature_id[629].value <= threshold=0.31753237545490265
node_914: feature_name=GO:0097193	feature_id[167].value > threshold=28.171168327331543
node_1188: feature_name=GO:0002704	feature_id[470].value <= threshold=0.8429980874061584
node_1189: feature_name=GO:0009411	feature_id[67].value > threshold=6.957151174545288
Class: positive genes	
Rules_87	passed counts:12
node_0: feature_name=GO:0042113	feature_id[0].value <= threshold=13.408552169799805
node_1: feature_name=GO:0007568	feature_id[534].value > threshold=5.0313897132873535
node_913: feature_name=GO:0032763	feature_id[629].value <= threshold=0.31753237545490265
node_914: feature_name=GO:0097193	feature_id[167].value <= threshold=28.171168327331543
node_915: feature_name=GO:0002903	feature_id[10].value > threshold=1.0534588098526
node_1159: feature_name=GO:0070198	feature_id[665].value > threshold=0.16219981759786606
node_1165: feature_name=GO:0042287	feature_id[20].value > threshold=0.915093183517456
node_1181: feature_name=GO:0060576	feature_id[204].value > threshold=0.3862239122390747
Class: positive genes	
Rules_88	passed counts:12
node_0: feature_name=GO:0042113	feature_id[0].value <= threshold=13.408552169799805
node_1: feature_name=GO:0007568	feature_id[534].value > threshold=5.0313897132873535
node_913: feature_name=GO:0032763	feature_id[629].value <= threshold=0.31753237545490265
node_914: feature_name=GO:0097193	feature_id[167].value <= threshold=28.171168327331543

node_915: feature_name=GO:0002903 feature_id[10].value <= threshold=1.0534588098526
node_916: feature_name=GO:1904029 feature_id[208].value <= threshold=23.852136611938477
node_917: feature_name=GO:0050897 feature_id[164].value > threshold=0.6870408356189728
node_1095: feature_name=GO:0072539 feature_id[68].value <= threshold=2.5667877197265625
node_1096: feature_name=GO:0046500 feature_id[171].value <= threshold=3.195197820663452
node_1097: feature_name=GO:0045861 feature_id[761].value > threshold=0.22614753991365433
node_1099: feature_name=GO:0043372 feature_id[242].value <= threshold=4.892506122589111
node_1100: feature_name=GO:0042162 feature_id[355].value <= threshold=11.339290618896484
node_1101: feature_name=hsa04660 feature_id[472].value <= threshold=24.27848720550537
node_1102: feature_name=GO:0007049 feature_id[259].value <= threshold=209.23831176757812
node_1103: feature_name=GO:0050897 feature_id[164].value > threshold=0.6919849812984467
node_1105: feature_name=GO:0045945 feature_id[267].value <= threshold=3.307852625846863
node_1106: feature_name=GO:0046641 feature_id[773].value <= threshold=3.527579426765442
node_1107: feature_name=GO:0002262 feature_id[394].value > threshold=0.08302562311291695
node_1111: feature_name=GO:0043226 feature_id[138].value > threshold=2.4930146992119262e-05
node_1113: feature_name=GO:0045787 feature_id[446].value > threshold=0.07611752673983574
node_1115: feature_name=GO:0045840 feature_id[697].value > threshold=0.10580676794052124
node_1117: feature_name=GO:0006927 feature_id[225].value <= threshold=4.950021743774414
node_1118: feature_name=GO:0002643 feature_id[4].value <= threshold=3.364396333694458
node_1119: feature_name=GO:0016571 feature_id[725].value <= threshold=38.268903732299805
node_1120: feature_name=GO:0072593 feature_id[381].value <= threshold=19.997477531433105
node_1121: feature_name=GO:0070245 feature_id[326].value <= threshold=3.2926437854766846
node_1122: feature_name=GO:0008327 feature_id[77].value > threshold=1.4845957159996033
node_1124: feature_name=GO:0000783 feature_id[191].value <= threshold=2.9206448197364807
Class: negative genes

Rules_89 passed counts:12
node_0: feature_name=GO:0042113 feature_id[0].value <= threshold=13.408552169799805
node_1: feature_name=GO:0007568 feature_id[534].value > threshold=5.0313897132873535
node_913: feature_name=GO:0032763 feature_id[629].value <= threshold=0.31753237545490265
node_914: feature_name=GO:0097193 feature_id[167].value <= threshold=28.171168327331543
node_915: feature_name=GO:0002903 feature_id[10].value <= threshold=1.0534588098526
node_916: feature_name=GO:1904029 feature_id[208].value <= threshold=23.852136611938477
node_917: feature_name=GO:0050897 feature_id[164].value <= threshold=0.6870408356189728
node_918: feature_name=GO:0006139 feature_id[474].value > threshold=1.3052097624921544e-07

node_920: feature_name=GO:0002821 feature_id[588].value <= threshold=13.81072187423706
node_921: feature_name=GO:0006298 feature_id[621].value <= threshold=24.078600883483887
node_922: feature_name=GO:0003908 feature_id[11].value <= threshold=1.7776933312416077
node_923: feature_name=GO:0030098 feature_id[273].value > threshold=0.0079949083738029
node_927: feature_name=GO:0006808 feature_id[513].value <= threshold=4.34592080116272
node_928: feature_name=GO:0071887 feature_id[283].value <= threshold=10.353787899017334
node_929: feature_name=GO:0038001 feature_id[28].value <= threshold=3.9469382762908936
node_930: feature_name=GO:0042287 feature_id[20].value <= threshold=3.6209195852279663
node_931: feature_name=GO:0003968 feature_id[47].value <= threshold=2.004227638244629
node_932: feature_name=GO:0002698 feature_id[395].value <= threshold=18.51447582244873
node_933: feature_name=GO:0044710 feature_id[719].value <= threshold=179.7340316772461
node_934: feature_name=GO:0007568 feature_id[534].value > threshold=5.035318374633789
node_936: feature_name=GO:0006216 feature_id[504].value <= threshold=2.2605666518211365
node_937: feature_name=GO:0048569 feature_id[793].value <= threshold=5.340231895446777
node_938: feature_name=GO:0001777 feature_id[380].value <= threshold=4.100832939147949
node_939: feature_name=GO:0007600 feature_id[122].value <= threshold=171.13383102416992
node_940: feature_name=GO:0001779 feature_id[378].value <= threshold=6.234851121902466
node_941: feature_name=GO:0030291 feature_id[147].value <= threshold=10.8051118850708
node_942: feature_name=GO:0048534 feature_id[790].value > threshold=0.017984486185014248
node_944: feature_name=GO:0070245 feature_id[326].value <= threshold=3.1282339096069336
node_945: feature_name=GO:0009086 feature_id[547].value <= threshold=0.2896959036588669
node_946: feature_name=GO:0001889 feature_id[387].value <= threshold=17.643128395080566
node_947: feature_name=GO:0048144 feature_id[192].value > threshold=0.20887330174446106
node_951: feature_name=GO:0023026 feature_id[69].value <= threshold=3.403424024581909
node_952: feature_name=GO:0048147 feature_id[788].value > threshold=4.188283443450928
node_1010: feature_name=GO:0016363 feature_id[90].value > threshold=1.0286503434181213
Class: negative genes

Rules_90 passed counts:12
node_0: feature_name=GO:0042113 feature_id[0].value <= threshold=13.408552169799805
node_1: feature_name=GO:0007568 feature_id[534].value <= threshold=5.0313897132873535
node_2: feature_name=GO:0002705 feature_id[541].value <= threshold=3.200145721435547
node_3: feature_name=GO:1901525 feature_id[576].value <= threshold=0.4466460347175598
node_4: feature_name=GO:0048539 feature_id[319].value <= threshold=3.0399646759033203
node_5: feature_name=GO:0001910 feature_id[385].value <= threshold=3.7437864542007446

node_6: feature_name=GO:0043200 feature_id[706].value <= threshold=9.307284355163574
node_7: feature_name=GO:0001773 feature_id[308].value <= threshold=4.016931533813477
node_8: feature_name=GO:0090116 feature_id[97].value <= threshold=7.99645471572876
node_9: feature_name=GO:0019814 feature_id[189].value <= threshold=4.525782108306885
node_10: feature_name=GO:1902583 feature_id[215].value <= threshold=17.60310649871826
node_11: feature_name=GO:0045429 feature_id[737].value <= threshold=1.6133361458778381
node_12: feature_name=GO:0003720 feature_id[228].value <= threshold=5.519326210021973
node_13: feature_name=GO:0046006 feature_id[364].value <= threshold=4.9985432624816895
node_14: feature_name=GO:0070424 feature_id[254].value <= threshold=6.233297109603882
node_15: feature_name=GO:0009892 feature_id[320].value <= threshold=59.99180793762207
node_16: feature_name=GO:0007064 feature_id[527].value <= threshold=11.57377815246582
node_17: feature_name=GO:0005575 feature_id[17].value <= threshold=15.417460918426514
node_18: feature_name=GO:0043368 feature_id[31].value <= threshold=4.059496641159058
node_19: feature_name=GO:0005164 feature_id[163].value <= threshold=5.793607711791992
node_20: feature_name=GO:0042130 feature_id[16].value <= threshold=5.073179721832275
node_21: feature_name=GO:0010216 feature_id[282].value <= threshold=4.834584474563599
node_22: feature_name=GO:0009628 feature_id[553].value <= threshold=29.314892768859863
node_23: feature_name=GO:0045628 feature_id[749].value <= threshold=3.1001367568969727
node_24: feature_name=GO:0042288 feature_id[100].value <= threshold=5.174932956695557
node_25: feature_name=GO:0002329 feature_id[406].value <= threshold=2.348930835723877
node_26: feature_name=GO:0051246 feature_id[642].value <= threshold=0.6498909294605255
node_27: feature_name=GO:0042162 feature_id[355].value <= threshold=8.05103588104248
node_28: feature_name=hsa04668 feature_id[333].value <= threshold=5.614926338195801
node_29: feature_name=GO:0032200 feature_id[613].value <= threshold=28.735913276672363
node_30: feature_name=hsa04662 feature_id[334].value <= threshold=9.588344097137451
node_31: feature_name=GO:1901992 feature_id[105].value <= threshold=5.291427850723267
node_32: feature_name=GO:0072341 feature_id[144].value <= threshold=6.01465106010437
node_33: feature_name=GO:0047485 feature_id[121].value <= threshold=6.657836437225342
node_34: feature_name=GO:0090594 feature_id[738].value <= threshold=1.8673912286758423
node_35: feature_name=GO:0043525 feature_id[524].value <= threshold=2.881397008895874
node_36: feature_name=GO:0032504 feature_id[244].value <= threshold=17.22207546234131
node_37: feature_name=GO:0051348 feature_id[126].value <= threshold=5.933559417724609
node_38: feature_name=GO:0042522 feature_id[692].value <= threshold=1.78694087266922
node_39: feature_name=GO:0031100 feature_id[73].value <= threshold=3.2312101125717163
node_40: feature_name=GO:0042493 feature_id[149].value <= threshold=19.771170616149902

node_41: feature_name=GO:0001552 feature_id[366].value <= threshold=2.1711617708206177
node_42: feature_name=GO:0046483 feature_id[391].value <= threshold=320.9667053222656
node_43: feature_name=GO:0002439 feature_id[431].value <= threshold=1.832722783088684
node_44: feature_name=GO:0006555 feature_id[514].value <= threshold=5.321640968322754
node_45: feature_name=GO:0070424 feature_id[254].value <= threshold=3.321003556251526
node_46: feature_name=hsa05144 feature_id[751].value <= threshold=2.635499954223633
node_47: feature_name=GO:0032633 feature_id[627].value <= threshold=1.2592533230781555
node_48: feature_name=GO:0019814 feature_id[189].value <= threshold=2.1583125591278076
node_49: feature_name=GO:1901989 feature_id[30].value <= threshold=3.3150794506073
node_50: feature_name=GO:1903318 feature_id[196].value > threshold=1.62252539396286
node_116: feature_name=GO:0005634 feature_id[247].value > threshold=4.554542215373658e-06
Class: negative genes

Rules_91 passed counts:12
node_0: feature_name=GO:0042113 feature_id[0].value <= threshold=13.408552169799805
node_1: feature_name=GO:0007568 feature_id[534].value <= threshold=5.0313897132873535
node_2: feature_name=GO:0002705 feature_id[541].value <= threshold=3.200145721435547
node_3: feature_name=GO:1901525 feature_id[576].value <= threshold=0.4466460347175598
node_4: feature_name=GO:0048539 feature_id[319].value <= threshold=3.0399646759033203
node_5: feature_name=GO:0001910 feature_id[385].value <= threshold=3.7437864542007446
node_6: feature_name=GO:0043200 feature_id[706].value <= threshold=9.307284355163574
node_7: feature_name=GO:0001773 feature_id[308].value <= threshold=4.016931533813477
node_8: feature_name=GO:0090116 feature_id[97].value <= threshold=7.99645471572876
node_9: feature_name=GO:0019814 feature_id[189].value <= threshold=4.525782108306885
node_10: feature_name=GO:1902583 feature_id[215].value <= threshold=17.60310649871826
node_11: feature_name=GO:0045429 feature_id[737].value <= threshold=1.6133361458778381
node_12: feature_name=GO:0003720 feature_id[228].value <= threshold=5.519326210021973
node_13: feature_name=GO:0046006 feature_id[364].value <= threshold=4.9985432624816895
node_14: feature_name=GO:0070424 feature_id[254].value <= threshold=6.233297109603882
node_15: feature_name=GO:0009892 feature_id[320].value <= threshold=59.99180793762207
node_16: feature_name=GO:0007064 feature_id[527].value <= threshold=11.57377815246582
node_17: feature_name=GO:0005575 feature_id[17].value <= threshold=15.417460918426514
node_18: feature_name=GO:0043368 feature_id[31].value <= threshold=4.059496641159058
node_19: feature_name=GO:0005164 feature_id[163].value <= threshold=5.793607711791992
node_20: feature_name=GO:0042130 feature_id[16].value <= threshold=5.073179721832275

node_21: feature_name=GO:0010216 feature_id[282].value <= threshold=4.834584474563599
node_22: feature_name=GO:0009628 feature_id[553].value <= threshold=29.314892768859863
node_23: feature_name=GO:0045628 feature_id[749].value <= threshold=3.1001367568969727
node_24: feature_name=GO:0042288 feature_id[100].value <= threshold=5.174932956695557
node_25: feature_name=GO:0002329 feature_id[406].value <= threshold=2.348930835723877
node_26: feature_name=GO:0051246 feature_id[642].value <= threshold=0.6498909294605255
node_27: feature_name=GO:0042162 feature_id[355].value <= threshold=8.05103588104248
node_28: feature_name=hsa04668 feature_id[333].value <= threshold=5.614926338195801
node_29: feature_name=GO:0032200 feature_id[613].value <= threshold=28.735913276672363
node_30: feature_name=hsa04662 feature_id[334].value <= threshold=9.588344097137451
node_31: feature_name=GO:1901992 feature_id[105].value <= threshold=5.291427850723267
node_32: feature_name=GO:0072341 feature_id[144].value <= threshold=6.01465106010437
node_33: feature_name=GO:0047485 feature_id[121].value <= threshold=6.657836437225342
node_34: feature_name=GO:0090594 feature_id[738].value <= threshold=1.8673912286758423
node_35: feature_name=GO:0043525 feature_id[524].value <= threshold=2.881397008895874
node_36: feature_name=GO:0032504 feature_id[244].value <= threshold=17.22207546234131
node_37: feature_name=GO:0051348 feature_id[126].value <= threshold=5.933559417724609
node_38: feature_name=GO:0042522 feature_id[692].value <= threshold=1.78694087266922
node_39: feature_name=GO:0031100 feature_id[73].value <= threshold=3.2312101125717163
node_40: feature_name=GO:0042493 feature_id[149].value <= threshold=19.771170616149902
node_41: feature_name=GO:0001552 feature_id[366].value <= threshold=2.1711617708206177
node_42: feature_name=GO:0046483 feature_id[391].value <= threshold=320.9667053222656
node_43: feature_name=GO:0002439 feature_id[431].value <= threshold=1.832722783088684
node_44: feature_name=GO:0006555 feature_id[514].value <= threshold=5.321640968322754
node_45: feature_name=GO:0070424 feature_id[254].value <= threshold=3.321003556251526
node_46: feature_name=hsa05144 feature_id[751].value <= threshold=2.635499954223633
node_47: feature_name=GO:0032633 feature_id[627].value <= threshold=1.2592533230781555
node_48: feature_name=GO:0019814 feature_id[189].value <= threshold=2.1583125591278076
node_49: feature_name=GO:1901989 feature_id[30].value <= threshold=3.3150794506073
node_50: feature_name=GO:1903318 feature_id[196].value <= threshold=1.62252539396286
node_51: feature_name=GO:0060576 feature_id[204].value > threshold=2.1097792387008667
node_113: feature_name=GO:0060374 feature_id[76].value <= threshold=1.0223779380321503
Class: negative genes

Rules_92 passed counts:11

node_0: feature_name=GO:0042113 feature_id[0].value > threshold=13.408552169799805
node_1454: feature_name=GO:0050851 feature_id[29].value > threshold=9.650307655334473
node_1534: feature_name=GO:1902166 feature_id[301].value > threshold=0.303210511803627
node_1548: feature_name=GO:0035872 feature_id[659].value <= threshold=35.09038162231445
node_1549: feature_name=GO:0032069 feature_id[614].value <= threshold=3.441983938217163
node_1550: feature_name=GO:0050897 feature_id[164].value <= threshold=1.3939869403839111
node_1551: feature_name=GO:0051454 feature_id[307].value <= threshold=0.9144491851329803
node_1552: feature_name=GO:0035825 feature_id[662].value <= threshold=0.08555268123745918
node_1553: feature_name=GO:0032761 feature_id[633].value <= threshold=1.6124996542930603
Class: negative genes

Rules_93 passed counts:11
node_0: feature_name=GO:0042113 feature_id[0].value > threshold=13.408552169799805
node_1454: feature_name=GO:0050851 feature_id[29].value <= threshold=9.650307655334473
node_1455: feature_name=GO:0006304 feature_id[510].value <= threshold=5.960662126541138
node_1456: feature_name=GO:0032673 feature_id[623].value <= threshold=4.092082738876343
node_1457: feature_name=GO:0002429 feature_id[748].value > threshold=4.626799821853638
node_1463: feature_name=GO:0015672 feature_id[573].value <= threshold=0.08226438239216805
node_1464: feature_name=GO:0038065 feature_id[670].value <= threshold=0.3765842020511627
node_1465: feature_name=GO:0002253 feature_id[397].value > threshold=11.476495742797852
Class: negative genes

Rules_94 passed counts:11
node_0: feature_name=GO:0042113 feature_id[0].value <= threshold=13.408552169799805
node_1: feature_name=GO:0007568 feature_id[534].value > threshold=5.0313897132873535
node_913: feature_name=GO:0032763 feature_id[629].value > threshold=0.31753237545490265
node_1195: feature_name=GO:0071301 feature_id[442].value > threshold=0.11534593254327774
node_1375: feature_name=GO:0046500 feature_id[171].value > threshold=1.130434513092041
node_1437: feature_name=GO:0033343 feature_id[639].value <= threshold=0.98012974858284
node_1438: feature_name=GO:0019058 feature_id[245].value <= threshold=11.779548168182373
Class: negative genes

Rules_95 passed counts:11
node_0: feature_name=GO:0042113 feature_id[0].value <= threshold=13.408552169799805
node_1: feature_name=GO:0007568 feature_id[534].value > threshold=5.0313897132873535

node_913: feature_name=GO:0032763 feature_id[629].value > threshold=0.31753237545490265
node_1195: feature_name=GO:0071301 feature_id[442].value > threshold=0.11534593254327774
node_1375: feature_name=GO:0046500 feature_id[171].value <= threshold=1.130434513092041
node_1376: feature_name=GO:0001782 feature_id[240].value <= threshold=6.639636993408203
node_1377: feature_name=GO:0043371 feature_id[707].value <= threshold=2.562113642692566
node_1378: feature_name=GO:0030291 feature_id[147].value > threshold=6.834916830062866
node_1424: feature_name=GO:0002439 feature_id[431].value > threshold=0.6129993498325348
node_1428: feature_name=GO:0009314 feature_id[296].value > threshold=13.65259075164795
Class: negative genes

Rules_96 passed counts:11
node_0: feature_name=GO:0042113 feature_id[0].value <= threshold=13.408552169799805
node_1: feature_name=GO:0007568 feature_id[534].value > threshold=5.0313897132873535
node_913: feature_name=GO:0032763 feature_id[629].value > threshold=0.31753237545490265
node_1195: feature_name=GO:0071301 feature_id[442].value <= threshold=0.11534593254327774
node_1196: feature_name=GO:0046500 feature_id[171].value > threshold=0.6314916908740997
node_1340: feature_name=GO:0043627 feature_id[562].value <= threshold=25.17277240753174
node_1341: feature_name=GO:0010835 feature_id[560].value <= threshold=1.0601619482040405
node_1342: feature_name=GO:0016363 feature_id[90].value <= threshold=6.534365892410278
node_1343: feature_name=GO:0030983 feature_id[291].value > threshold=3.5806901454925537
node_1359: feature_name=GO:0046632 feature_id[248].value <= threshold=0.5181337743997574
Class: negative genes

Rules_97 passed counts:11
node_0: feature_name=GO:0042113 feature_id[0].value <= threshold=13.408552169799805
node_1: feature_name=GO:0007568 feature_id[534].value > threshold=5.0313897132873535
node_913: feature_name=GO:0032763 feature_id[629].value <= threshold=0.31753237545490265
node_914: feature_name=GO:0097193 feature_id[167].value <= threshold=28.171168327331543
node_915: feature_name=GO:0002903 feature_id[10].value <= threshold=1.0534588098526
node_916: feature_name=GO:1904029 feature_id[208].value <= threshold=23.852136611938477
node_917: feature_name=GO:0050897 feature_id[164].value <= threshold=0.6870408356189728
node_918: feature_name=GO:0006139 feature_id[474].value > threshold=1.3052097624921544e-07
node_920: feature_name=GO:0002821 feature_id[588].value <= threshold=13.81072187423706
node_921: feature_name=GO:0006298 feature_id[621].value <= threshold=24.078600883483887
node_922: feature_name=GO:0003908 feature_id[11].value <= threshold=1.7776933312416077

node_923: feature_name=GO:0030098 feature_id[273].value > threshold=0.0079949083738029
node_927: feature_name=GO:0006808 feature_id[513].value <= threshold=4.34592080116272
node_928: feature_name=GO:0071887 feature_id[283].value <= threshold=10.353787899017334
node_929: feature_name=GO:0038001 feature_id[28].value <= threshold=3.9469382762908936
node_930: feature_name=GO:0042287 feature_id[20].value <= threshold=3.6209195852279663
node_931: feature_name=GO:0003968 feature_id[47].value <= threshold=2.004227638244629
node_932: feature_name=GO:0002698 feature_id[395].value <= threshold=18.51447582244873
node_933: feature_name=GO:0044710 feature_id[719].value <= threshold=179.7340316772461
node_934: feature_name=GO:0007568 feature_id[534].value > threshold=5.035318374633789
node_936: feature_name=GO:0006216 feature_id[504].value <= threshold=2.2605666518211365
node_937: feature_name=GO:0048569 feature_id[793].value <= threshold=5.340231895446777
node_938: feature_name=GO:0001777 feature_id[380].value <= threshold=4.100832939147949
node_939: feature_name=GO:0007600 feature_id[122].value <= threshold=171.13383102416992
node_940: feature_name=GO:0001779 feature_id[378].value <= threshold=6.234851121902466
node_941: feature_name=GO:0030291 feature_id[147].value <= threshold=10.8051118850708
node_942: feature_name=GO:0048534 feature_id[790].value > threshold=0.017984486185014248
node_944: feature_name=GO:0070245 feature_id[326].value <= threshold=3.1282339096069336
node_945: feature_name=GO:0009086 feature_id[547].value > threshold=0.2896959036588669
node_1017: feature_name=GO:0000302 feature_id[375].value > threshold=0.305715873837471
node_1019: feature_name=GO:0070141 feature_id[37].value <= threshold=4.857294321060181
node_1020: feature_name=GO:0009086 feature_id[547].value > threshold=0.2945319563150406
node_1024: feature_name=GO:0010332 feature_id[557].value <= threshold=15.092710971832275
node_1025: feature_name=GO:0002524 feature_id[72].value <= threshold=2.531021237373352
node_1026: feature_name=GO:0016032 feature_id[571].value <= threshold=60.173330307006836
node_1027: feature_name=GO:0002832 feature_id[491].value <= threshold=2.0699684023857117
node_1028: feature_name=GO:0008588 feature_id[26].value <= threshold=2.150238513946533
node_1029: feature_name=GO:0009615 feature_id[265].value > threshold=0.02372477948665619
node_1031: feature_name=GO:0002763 feature_id[271].value <= threshold=6.462319850921631
node_1032: feature_name=GO:0045840 feature_id[697].value <= threshold=10.30175256729126
node_1033: feature_name=GO:0051402 feature_id[568].value > threshold=1.2523645758628845
node_1041: feature_name=GO:0050852 feature_id[154].value > threshold=0.0008433434413745999
node_1043: feature_name=GO:0031667 feature_id[648].value > threshold=1.5418170094490051
node_1047: feature_name=GO:0009987 feature_id[554].value > threshold=1.8002276420593262
node_1051: feature_name=GO:0008588 feature_id[26].value > threshold=0.9623689949512482
node_1053: feature_name=GO:0005035 feature_id[133].value > threshold=0.4232639968395233

Class: negative genes

Rules_98	passed counts:11
node_0: feature_name=GO:0042113	feature_id[0].value <= threshold=13.408552169799805
node_1: feature_name=GO:0007568	feature_id[534].value <= threshold=5.0313897132873535
node_2: feature_name=GO:0002705	feature_id[541].value > threshold=3.200145721435547
node_870: feature_name=GO:0046634	feature_id[46].value <= threshold=8.9394211769104
node_871: feature_name=GO:0048523	feature_id[293].value <= threshold=19.963229179382324
node_872: feature_name=GO:2001237	feature_id[796].value <= threshold=0.09761488810181618
node_873: feature_name=GO:0097028	feature_id[443].value > threshold=1.0470510721206665
node_877: feature_name=GO:0002718	feature_id[481].value <= threshold=3.8375377655029297

Class: positive genes

Rules_99	passed counts:11
node_0: feature_name=GO:0042113	feature_id[0].value <= threshold=13.408552169799805
node_1: feature_name=GO:0007568	feature_id[534].value <= threshold=5.0313897132873535
node_2: feature_name=GO:0002705	feature_id[541].value > threshold=3.200145721435547
node_870: feature_name=GO:0046634	feature_id[46].value <= threshold=8.9394211769104
node_871: feature_name=GO:0048523	feature_id[293].value <= threshold=19.963229179382324
node_872: feature_name=GO:2001237	feature_id[796].value <= threshold=0.09761488810181618
node_873: feature_name=GO:0097028	feature_id[443].value <= threshold=1.0470510721206665
node_874: feature_name=GO:0043066	feature_id[667].value <= threshold=2.3243058919906616

Class: negative genes

Rules_100	passed counts:11
node_0: feature_name=GO:0042113	feature_id[0].value <= threshold=13.408552169799805
node_1: feature_name=GO:0007568	feature_id[534].value <= threshold=5.0313897132873535
node_2: feature_name=GO:0002705	feature_id[541].value <= threshold=3.200145721435547
node_3: feature_name=GO:1901525	feature_id[576].value <= threshold=0.4466460347175598
node_4: feature_name=GO:0048539	feature_id[319].value <= threshold=3.0399646759033203
node_5: feature_name=GO:0001910	feature_id[385].value <= threshold=3.7437864542007446
node_6: feature_name=GO:0043200	feature_id[706].value <= threshold=9.307284355163574
node_7: feature_name=GO:0001773	feature_id[308].value <= threshold=4.016931533813477
node_8: feature_name=GO:0090116	feature_id[97].value <= threshold=7.99645471572876
node_9: feature_name=GO:0019814	feature_id[189].value <= threshold=4.525782108306885

node_10: feature_name=GO:1902583	feature_id[215].value <= threshold=17.60310649871826
node_11: feature_name=GO:0045429	feature_id[737].value <= threshold=1.6133361458778381
node_12: feature_name=GO:0003720	feature_id[228].value <= threshold=5.519326210021973
node_13: feature_name=GO:0046006	feature_id[364].value <= threshold=4.9985432624816895
node_14: feature_name=GO:0070424	feature_id[254].value <= threshold=6.233297109603882
node_15: feature_name=GO:0009892	feature_id[320].value <= threshold=59.99180793762207
node_16: feature_name=GO:0007064	feature_id[527].value <= threshold=11.57377815246582
node_17: feature_name=GO:0005575	feature_id[17].value <= threshold=15.417460918426514
node_18: feature_name=GO:0043368	feature_id[31].value <= threshold=4.059496641159058
node_19: feature_name=GO:0005164	feature_id[163].value <= threshold=5.793607711791992
node_20: feature_name=GO:0042130	feature_id[16].value <= threshold=5.073179721832275
node_21: feature_name=GO:0010216	feature_id[282].value <= threshold=4.834584474563599
node_22: feature_name=GO:0009628	feature_id[553].value <= threshold=29.314892768859863
node_23: feature_name=GO:0045628	feature_id[749].value <= threshold=3.1001367568969727
node_24: feature_name=GO:0042288	feature_id[100].value <= threshold=5.174932956695557
node_25: feature_name=GO:0002329	feature_id[406].value <= threshold=2.348930835723877
node_26: feature_name=GO:0051246	feature_id[642].value > threshold=0.6498909294605255
node_194: feature_name=GO:0009164	feature_id[546].value <= threshold=17.11970329284668
node_195: feature_name=GO:0001909	feature_id[386].value <= threshold=7.292566299438477
node_196: feature_name=GO:0003908	feature_id[11].value <= threshold=1.887226164340973
node_197: feature_name=GO:0009164	feature_id[546].value > threshold=0.26642198860645294
node_359: feature_name=GO:0009164	feature_id[546].value > threshold=0.2696942090988159
node_361: feature_name=hsa05202	feature_id[50].value <= threshold=14.989679336547852
node_362: feature_name=GO:0001775	feature_id[505].value <= threshold=15.726949691772461
node_363: feature_name=GO:0002634	feature_id[449].value <= threshold=2.825531005859375
node_364: feature_name=GO:0010038	feature_id[457].value <= threshold=9.901018619537354
node_365: feature_name=GO:0044238	feature_id[295].value <= threshold=139.80535888671875
node_366: feature_name=GO:0051246	feature_id[642].value > threshold=0.6527281999588013
node_368: feature_name=GO:0044092	feature_id[432].value <= threshold=11.084641933441162
node_369: feature_name=GO:0042162	feature_id[355].value <= threshold=9.683155536651611
node_370: feature_name=GO:0045945	feature_id[267].value <= threshold=3.6181851625442505
node_371: feature_name=GO:0036296	feature_id[252].value <= threshold=3.214281916618347
node_372: feature_name=GO:0097506	feature_id[51].value <= threshold=2.9644681215286255
node_373: feature_name=GO:0019740	feature_id[581].value <= threshold=2.2853575944900513
node_374: feature_name=GO:0019660	feature_id[70].value <= threshold=1.2831979990005493

node_375: feature_name=GO:0071901 feature_id[407].value <= threshold=14.498775482177734
node_376: feature_name=GO:0043170 feature_id[467].value > threshold=4.473330818655086e-06
node_380: feature_name=GO:0046718 feature_id[781].value <= threshold=3.494875192642212
node_381: feature_name=GO:0030983 feature_id[291].value <= threshold=10.994179248809814
node_382: feature_name=GO:0010663 feature_id[561].value <= threshold=2.7056833505630493
node_383: feature_name=GO:0031667 feature_id[648].value > threshold=0.0020462179090827703
node_387: feature_name=GO:1990572 feature_id[243].value <= threshold=1.9359037280082703
node_388: feature_name=GO:0032753 feature_id[101].value <= threshold=1.182796835899353
node_389: feature_name=GO:0097153 feature_id[226].value <= threshold=0.34601132571697235
node_390: feature_name=GO:0002829 feature_id[493].value <= threshold=1.246164083480835
node_391: feature_name=GO:0006346 feature_id[236].value > threshold=2.040414571762085
node_393: feature_name=GO:0002821 feature_id[588].value <= threshold=0.7741671800613403
Class: negative genes

Rules_101 passed counts:11
node_0: feature_name=GO:0042113 feature_id[0].value <= threshold=13.408552169799805
node_1: feature_name=GO:0007568 feature_id[534].value <= threshold=5.0313897132873535
node_2: feature_name=GO:0002705 feature_id[541].value <= threshold=3.200145721435547
node_3: feature_name=GO:1901525 feature_id[576].value <= threshold=0.4466460347175598
node_4: feature_name=GO:0048539 feature_id[319].value <= threshold=3.0399646759033203
node_5: feature_name=GO:0001910 feature_id[385].value <= threshold=3.7437864542007446
node_6: feature_name=GO:0043200 feature_id[706].value <= threshold=9.307284355163574
node_7: feature_name=GO:0001773 feature_id[308].value <= threshold=4.016931533813477
node_8: feature_name=GO:0090116 feature_id[97].value <= threshold=7.99645471572876
node_9: feature_name=GO:0019814 feature_id[189].value <= threshold=4.525782108306885
node_10: feature_name=GO:1902583 feature_id[215].value <= threshold=17.60310649871826
node_11: feature_name=GO:0045429 feature_id[737].value <= threshold=1.6133361458778381
node_12: feature_name=GO:0003720 feature_id[228].value <= threshold=5.519326210021973
node_13: feature_name=GO:0046006 feature_id[364].value <= threshold=4.9985432624816895
node_14: feature_name=GO:0070424 feature_id[254].value <= threshold=6.233297109603882
node_15: feature_name=GO:0009892 feature_id[320].value <= threshold=59.99180793762207
node_16: feature_name=GO:0007064 feature_id[527].value <= threshold=11.57377815246582
node_17: feature_name=GO:0005575 feature_id[17].value <= threshold=15.417460918426514
node_18: feature_name=GO:0043368 feature_id[31].value <= threshold=4.059496641159058
node_19: feature_name=GO:0005164 feature_id[163].value <= threshold=5.793607711791992

node_20: feature_name=GO:0042130 feature_id[16].value <= threshold=5.073179721832275
node_21: feature_name=GO:0010216 feature_id[282].value <= threshold=4.834584474563599
node_22: feature_name=GO:0009628 feature_id[553].value <= threshold=29.314892768859863
node_23: feature_name=GO:0045628 feature_id[749].value <= threshold=3.1001367568969727
node_24: feature_name=GO:0042288 feature_id[100].value <= threshold=5.174932956695557
node_25: feature_name=GO:0002329 feature_id[406].value <= threshold=2.348930835723877
node_26: feature_name=GO:0051246 feature_id[642].value > threshold=0.6498909294605255
node_194: feature_name=GO:0009164 feature_id[546].value <= threshold=17.11970329284668
node_195: feature_name=GO:0001909 feature_id[386].value <= threshold=7.292566299438477
node_196: feature_name=GO:0003908 feature_id[11].value <= threshold=1.887226164340973
node_197: feature_name=GO:0009164 feature_id[546].value > threshold=0.26642198860645294
node_359: feature_name=GO:0009164 feature_id[546].value > threshold=0.2696942090988159
node_361: feature_name=hsa05202 feature_id[50].value <= threshold=14.989679336547852
node_362: feature_name=GO:0001775 feature_id[505].value <= threshold=15.726949691772461
node_363: feature_name=GO:0002634 feature_id[449].value <= threshold=2.825531005859375
node_364: feature_name=GO:0010038 feature_id[457].value <= threshold=9.901018619537354
node_365: feature_name=GO:0044238 feature_id[295].value <= threshold=139.80535888671875
node_366: feature_name=GO:0051246 feature_id[642].value > threshold=0.6527281999588013
node_368: feature_name=GO:0044092 feature_id[432].value <= threshold=11.084641933441162
node_369: feature_name=GO:0042162 feature_id[355].value <= threshold=9.683155536651611
node_370: feature_name=GO:0045945 feature_id[267].value <= threshold=3.6181851625442505
node_371: feature_name=GO:0036296 feature_id[252].value <= threshold=3.214281916618347
node_372: feature_name=GO:0097506 feature_id[51].value <= threshold=2.9644681215286255
node_373: feature_name=GO:0019740 feature_id[581].value <= threshold=2.2853575944900513
node_374: feature_name=GO:0019660 feature_id[70].value <= threshold=1.2831979990005493
node_375: feature_name=GO:0071901 feature_id[407].value <= threshold=14.498775482177734
node_376: feature_name=GO:0043170 feature_id[467].value > threshold=4.473330818655086e-06
node_380: feature_name=GO:0046718 feature_id[781].value <= threshold=3.494875192642212
node_381: feature_name=GO:0030983 feature_id[291].value <= threshold=10.994179248809814
node_382: feature_name=GO:0010663 feature_id[561].value <= threshold=2.7056833505630493
node_383: feature_name=GO:0031667 feature_id[648].value <= threshold=0.0020462179090827703
node_384: feature_name=GO:0070102 feature_id[49].value <= threshold=0.34830209612846375
Class: negative genes

Rules_102 passed counts:10

node_0: feature_name=GO:0042113	feature_id[0].value <= threshold=13.408552169799805
node_1: feature_name=GO:0007568	feature_id[534].value <= threshold=5.0313897132873535
node_2: feature_name=GO:0002705	feature_id[541].value <= threshold=3.200145721435547
node_3: feature_name=GO:1901525	feature_id[576].value > threshold=0.4466460347175598
node_617: feature_name=GO:0005622	feature_id[233].value <= threshold=90.85730743408203
node_618: feature_name=GO:1903147	feature_id[200].value <= threshold=7.326428413391113
node_619: feature_name=GO:0003964	feature_id[317].value <= threshold=5.575642108917236
node_620: feature_name=GO:0046498	feature_id[203].value <= threshold=6.620223522186279
node_621: feature_name=GO:0002562	feature_id[25].value <= threshold=10.287704467773438
node_622: feature_name=GO:0006919	feature_id[246].value <= threshold=5.801334381103516
node_623: feature_name=GO:0001889	feature_id[387].value <= threshold=2.679414987564087
node_624: feature_name=GO:0006555	feature_id[514].value <= threshold=7.819535493850708
node_625: feature_name=GO:0080134	feature_id[650].value <= threshold=38.91967582702637
node_626: feature_name=GO:0042127	feature_id[480].value <= threshold=37.72536659240723
node_627: feature_name=GO:2001251	feature_id[220].value <= threshold=32.564422607421875
node_628: feature_name=GO:0002829	feature_id[493].value <= threshold=2.444612741470337
node_629: feature_name=GO:0008588	feature_id[26].value <= threshold=2.3601274490356445
node_630: feature_name=GO:0033151	feature_id[7].value <= threshold=5.134376287460327
node_631: feature_name=GO:0048145	feature_id[348].value <= threshold=8.836549282073975
node_632: feature_name=GO:0030887	feature_id[274].value <= threshold=1.7000296115875244
node_633: feature_name=GO:0097190	feature_id[117].value <= threshold=33.02078819274902
node_634: feature_name=GO:0046685	feature_id[782].value <= threshold=8.040813446044922
node_635: feature_name=GO:0006346	feature_id[236].value <= threshold=6.384642839431763
node_636: feature_name=GO:0048537	feature_id[66].value <= threshold=3.1739262342453003
node_637: feature_name=GO:0070245	feature_id[326].value <= threshold=3.0491198301315308
node_638: feature_name=GO:0038065	feature_id[670].value <= threshold=2.0590850114822388
node_639: feature_name=GO:0005488	feature_id[187].value <= threshold=84.80076217651367
node_640: feature_name=GO:0060249	feature_id[390].value > threshold=0.07226398587226868
node_652: feature_name=GO:0006974	feature_id[516].value > threshold=5.17782768838515e-06
node_654: feature_name=hsa05210	feature_id[358].value <= threshold=18.730005264282227
node_655: feature_name=GO:0071850	feature_id[40].value <= threshold=5.475317001342773
node_656: feature_name=GO:1904029	feature_id[208].value <= threshold=26.575013160705566
node_657: feature_name=GO:0090594	feature_id[738].value <= threshold=3.000791072845459
node_658: feature_name=GO:0010948	feature_id[569].value > threshold=0.04386143572628498
node_666: feature_name=GO:0032764	feature_id[3].value <= threshold=2.5201677083969116

node_667: feature_name=GO:0023026 feature_id[69].value <= threshold=3.2760006189346313
node_668: feature_name=GO:0032464 feature_id[83].value <= threshold=2.5927021503448486
node_669: feature_name=GO:0071901 feature_id[407].value <= threshold=18.238012313842773
node_670: feature_name=GO:0015671 feature_id[103].value <= threshold=1.3113192915916443
node_671: feature_name=GO:0008340 feature_id[539].value <= threshold=4.860779762268066
node_672: feature_name=GO:0046006 feature_id[364].value <= threshold=2.4317692518234253
node_673: feature_name=GO:0042991 feature_id[684].value <= threshold=5.553565502166748
node_674: feature_name=GO:0002327 feature_id[408].value <= threshold=2.404940366744995
node_675: feature_name=GO:0001836 feature_id[450].value <= threshold=8.647934436798096
node_676: feature_name=GO:0048539 feature_id[319].value <= threshold=1.6308802962303162
node_677: feature_name=GO:0022408 feature_id[279].value <= threshold=4.064727783203125
node_678: feature_name=GO:0030099 feature_id[598].value > threshold=0.0001809061213862151
node_684: feature_name=GO:0001816 feature_id[685].value > threshold=1.3516043054551119e-05
node_688: feature_name=GO:0043200 feature_id[706].value > threshold=4.01608681678772
node_698: feature_name=GO:1901099 feature_id[572].value > threshold=0.4925118386745453
Class: negative genes

Rules_103 passed counts:10
node_0: feature_name=GO:0042113 feature_id[0].value <= threshold=13.408552169799805
node_1: feature_name=GO:0007568 feature_id[534].value <= threshold=5.0313897132873535
node_2: feature_name=GO:0002705 feature_id[541].value <= threshold=3.200145721435547
node_3: feature_name=GO:1901525 feature_id[576].value <= threshold=0.4466460347175598
node_4: feature_name=GO:0048539 feature_id[319].value <= threshold=3.0399646759033203
node_5: feature_name=GO:0001910 feature_id[385].value <= threshold=3.7437864542007446
node_6: feature_name=GO:0043200 feature_id[706].value <= threshold=9.307284355163574
node_7: feature_name=GO:0001773 feature_id[308].value <= threshold=4.016931533813477
node_8: feature_name=GO:0090116 feature_id[97].value <= threshold=7.99645471572876
node_9: feature_name=GO:0019814 feature_id[189].value <= threshold=4.525782108306885
node_10: feature_name=GO:1902583 feature_id[215].value <= threshold=17.60310649871826
node_11: feature_name=GO:0045429 feature_id[737].value <= threshold=1.6133361458778381
node_12: feature_name=GO:0003720 feature_id[228].value <= threshold=5.519326210021973
node_13: feature_name=GO:0046006 feature_id[364].value <= threshold=4.9985432624816895
node_14: feature_name=GO:0070424 feature_id[254].value <= threshold=6.233297109603882
node_15: feature_name=GO:0009892 feature_id[320].value <= threshold=59.99180793762207
node_16: feature_name=GO:0007064 feature_id[527].value <= threshold=11.57377815246582

node_17: feature_name=GO:0005575 feature_id[17].value <= threshold=15.417460918426514
node_18: feature_name=GO:0043368 feature_id[31].value <= threshold=4.059496641159058
node_19: feature_name=GO:0005164 feature_id[163].value <= threshold=5.793607711791992
node_20: feature_name=GO:0042130 feature_id[16].value <= threshold=5.073179721832275
node_21: feature_name=GO:0010216 feature_id[282].value <= threshold=4.834584474563599
node_22: feature_name=GO:0009628 feature_id[553].value <= threshold=29.314892768859863
node_23: feature_name=GO:0045628 feature_id[749].value <= threshold=3.1001367568969727
node_24: feature_name=GO:0042288 feature_id[100].value <= threshold=5.174932956695557
node_25: feature_name=GO:0002329 feature_id[406].value <= threshold=2.348930835723877
node_26: feature_name=GO:0051246 feature_id[642].value <= threshold=0.6498909294605255
node_27: feature_name=GO:0042162 feature_id[355].value <= threshold=8.05103588104248
node_28: feature_name=hsa04668 feature_id[333].value <= threshold=5.614926338195801
node_29: feature_name=GO:0032200 feature_id[613].value <= threshold=28.735913276672363
node_30: feature_name=hsa04662 feature_id[334].value <= threshold=9.588344097137451
node_31: feature_name=GO:1901992 feature_id[105].value <= threshold=5.291427850723267
node_32: feature_name=GO:0072341 feature_id[144].value <= threshold=6.01465106010437
node_33: feature_name=GO:0047485 feature_id[121].value <= threshold=6.657836437225342
node_34: feature_name=GO:0090594 feature_id[738].value <= threshold=1.8673912286758423
node_35: feature_name=GO:0043525 feature_id[524].value <= threshold=2.881397008895874
node_36: feature_name=GO:0032504 feature_id[244].value <= threshold=17.22207546234131
node_37: feature_name=GO:0051348 feature_id[126].value <= threshold=5.933559417724609
node_38: feature_name=GO:0042522 feature_id[692].value <= threshold=1.78694087266922
node_39: feature_name=GO:0031100 feature_id[73].value <= threshold=3.2312101125717163
node_40: feature_name=GO:0042493 feature_id[149].value <= threshold=19.771170616149902
node_41: feature_name=GO:0001552 feature_id[366].value <= threshold=2.1711617708206177
node_42: feature_name=GO:0046483 feature_id[391].value <= threshold=320.9667053222656
node_43: feature_name=GO:0002439 feature_id[431].value <= threshold=1.832722783088684
node_44: feature_name=GO:0006555 feature_id[514].value <= threshold=5.321640968322754
node_45: feature_name=GO:0070424 feature_id[254].value <= threshold=3.321003556251526
node_46: feature_name=hsa05144 feature_id[751].value <= threshold=2.635499954223633
node_47: feature_name=GO:0032633 feature_id[627].value <= threshold=1.2592533230781555
node_48: feature_name=GO:0019814 feature_id[189].value <= threshold=2.1583125591278076
node_49: feature_name=GO:1901989 feature_id[30].value > threshold=3.3150794506073
node_119: feature_name=GO:0032268 feature_id[249].value > threshold=0.04715394601225853
Class: negative genes

Rules_104 passed counts:9
node_0: feature_name=GO:0042113 feature_id[0].value > threshold=13.408552169799805
node_1454: feature_name=GO:0050851 feature_id[29].value <= threshold=9.650307655334473
node_1455: feature_name=GO:0006304 feature_id[510].value <= threshold=5.960662126541138
node_1456: feature_name=GO:0032673 feature_id[623].value <= threshold=4.092082738876343
node_1457: feature_name=GO:0002429 feature_id[748].value > threshold=4.626799821853638
node_1463: feature_name=GO:0015672 feature_id[573].value > threshold=0.08226438239216805
node_1475: feature_name=GO:0030217 feature_id[589].value <= threshold=19.77315902709961
node_1476: feature_name=GO:0002381 feature_id[96].value > threshold=4.645626783370972
node_1490: feature_name=GO:0042113 feature_id[0].value <= threshold=17.225549697875977
node_1491: feature_name=GO:0002902 feature_id[59].value > threshold=3.440378785133362
node_1493: feature_name=GO:0006979 feature_id[44].value <= threshold=24.42934513092041
Class: positive genes

Rules_105 passed counts:9
node_0: feature_name=GO:0042113 feature_id[0].value > threshold=13.408552169799805
node_1454: feature_name=GO:0050851 feature_id[29].value <= threshold=9.650307655334473
node_1455: feature_name=GO:0006304 feature_id[510].value <= threshold=5.960662126541138
node_1456: feature_name=GO:0032673 feature_id[623].value <= threshold=4.092082738876343
node_1457: feature_name=GO:0002429 feature_id[748].value > threshold=4.626799821853638
node_1463: feature_name=GO:0015672 feature_id[573].value > threshold=0.08226438239216805
node_1475: feature_name=GO:0030217 feature_id[589].value <= threshold=19.77315902709961
node_1476: feature_name=GO:0002381 feature_id[96].value > threshold=4.645626783370972
node_1490: feature_name=GO:0042113 feature_id[0].value <= threshold=17.225549697875977
node_1491: feature_name=GO:0002902 feature_id[59].value <= threshold=3.440378785133362
Class: negative genes

Rules_106 passed counts:9
node_0: feature_name=GO:0042113 feature_id[0].value > threshold=13.408552169799805
node_1454: feature_name=GO:0050851 feature_id[29].value <= threshold=9.650307655334473
node_1455: feature_name=GO:0006304 feature_id[510].value <= threshold=5.960662126541138
node_1456: feature_name=GO:0032673 feature_id[623].value <= threshold=4.092082738876343
node_1457: feature_name=GO:0002429 feature_id[748].value > threshold=4.626799821853638
node_1463: feature_name=GO:0015672 feature_id[573].value > threshold=0.08226438239216805

node_1475: feature_name=GO:0030217 feature_id[589].value <= threshold=19.77315902709961
node_1476: feature_name=GO:0002381 feature_id[96].value <= threshold=4.645626783370972
node_1477: feature_name=GO:0045058 feature_id[727].value > threshold=5.092289447784424
node_1485: feature_name=GO:0016447 feature_id[74].value <= threshold=4.237667560577393
Class: positive genes

Rules_107 passed counts:9
node_0: feature_name=GO:0042113 feature_id[0].value <= threshold=13.408552169799805
node_1: feature_name=GO:0007568 feature_id[534].value > threshold=5.0313897132873535
node_913: feature_name=GO:0032763 feature_id[629].value > threshold=0.31753237545490265
node_1195: feature_name=GO:0071301 feature_id[442].value > threshold=0.11534593254327774
node_1375: feature_name=GO:0046500 feature_id[171].value <= threshold=1.130434513092041
node_1376: feature_name=GO:0001782 feature_id[240].value > threshold=6.639636993408203
Class: positive genes

Rules_108 passed counts:9
node_0: feature_name=GO:0042113 feature_id[0].value <= threshold=13.408552169799805
node_1: feature_name=GO:0007568 feature_id[534].value > threshold=5.0313897132873535
node_913: feature_name=GO:0032763 feature_id[629].value > threshold=0.31753237545490265
node_1195: feature_name=GO:0071301 feature_id[442].value > threshold=0.11534593254327774
node_1375: feature_name=GO:0046500 feature_id[171].value <= threshold=1.130434513092041
node_1376: feature_name=GO:0001782 feature_id[240].value <= threshold=6.639636993408203
node_1377: feature_name=GO:0043371 feature_id[707].value > threshold=2.562113642692566
node_1433: feature_name=GO:0044446 feature_id[184].value > threshold=5.497895836830139
Class: positive genes

Rules_109 passed counts:9
node_0: feature_name=GO:0042113 feature_id[0].value <= threshold=13.408552169799805
node_1: feature_name=GO:0007568 feature_id[534].value > threshold=5.0313897132873535
node_913: feature_name=GO:0032763 feature_id[629].value > threshold=0.31753237545490265
node_1195: feature_name=GO:0071301 feature_id[442].value <= threshold=0.11534593254327774
node_1196: feature_name=GO:0046500 feature_id[171].value <= threshold=0.6314916908740997
node_1197: feature_name=GO:0030852 feature_id[22].value <= threshold=2.9768868684768677
node_1198: feature_name=hsa04672 feature_id[276].value <= threshold=8.06681227684021
node_1199: feature_name=GO:0001836 feature_id[450].value > threshold=0.09961023926734924

node_1201: feature_name=GO:0070230 feature_id[64].value <= threshold=3.3956379890441895
node_1202: feature_name=GO:0070102 feature_id[49].value <= threshold=4.805386543273926
node_1203: feature_name=GO:0045553 feature_id[736].value <= threshold=1.4970332980155945
node_1204: feature_name=GO:0051311 feature_id[58].value <= threshold=3.301889181137085
node_1205: feature_name=GO:0030291 feature_id[147].value <= threshold=8.156315326690674
node_1206: feature_name=GO:0045637 feature_id[241].value > threshold=0.01834342861548066
node_1208: feature_name=GO:0002890 feature_id[98].value <= threshold=2.767483353614807
node_1209: feature_name=GO:0046632 feature_id[248].value <= threshold=11.663254261016846
node_1210: feature_name=GO:0008588 feature_id[26].value > threshold=1.391375720500946
node_1308: feature_name=GO:0002200 feature_id[82].value > threshold=1.0774684846401215
node_1310: feature_name=GO:0019814 feature_id[189].value <= threshold=0.7463377118110657
node_1311: feature_name=GO:0002517 feature_id[88].value <= threshold=1.2976589798927307
Class: positive genes

Rules_110 passed counts:9
node_0: feature_name=GO:0042113 feature_id[0].value <= threshold=13.408552169799805
node_1: feature_name=GO:0007568 feature_id[534].value > threshold=5.0313897132873535
node_913: feature_name=GO:0032763 feature_id[629].value <= threshold=0.31753237545490265
node_914: feature_name=GO:0097193 feature_id[167].value <= threshold=28.171168327331543
node_915: feature_name=GO:0002903 feature_id[10].value <= threshold=1.0534588098526
node_916: feature_name=GO:1904029 feature_id[208].value <= threshold=23.852136611938477
node_917: feature_name=GO:0050897 feature_id[164].value > threshold=0.6870408356189728
node_1095: feature_name=GO:0072539 feature_id[68].value <= threshold=2.5667877197265625
node_1096: feature_name=GO:0046500 feature_id[171].value <= threshold=3.195197820663452
node_1097: feature_name=GO:0045861 feature_id[761].value > threshold=0.22614753991365433
node_1099: feature_name=GO:0043372 feature_id[242].value <= threshold=4.892506122589111
node_1100: feature_name=GO:0042162 feature_id[355].value <= threshold=11.339290618896484
node_1101: feature_name=hsa04660 feature_id[472].value <= threshold=24.27848720550537
node_1102: feature_name=GO:0007049 feature_id[259].value <= threshold=209.23831176757812
node_1103: feature_name=GO:0050897 feature_id[164].value > threshold=0.6919849812984467
node_1105: feature_name=GO:0045945 feature_id[267].value > threshold=3.307852625846863
node_1137: feature_name=GO:0072710 feature_id[251].value <= threshold=0.4050363004207611
Class: negative genes

Rules_111 passed counts:9

node_0: feature_name=GO:0042113	feature_id[0].value <= threshold=13.408552169799805
node_1: feature_name=GO:0007568	feature_id[534].value > threshold=5.0313897132873535
node_913: feature_name=GO:0032763	feature_id[629].value <= threshold=0.31753237545490265
node_914: feature_name=GO:0097193	feature_id[167].value <= threshold=28.171168327331543
node_915: feature_name=GO:0002903	feature_id[10].value <= threshold=1.0534588098526
node_916: feature_name=GO:1904029	feature_id[208].value <= threshold=23.852136611938477
node_917: feature_name=GO:0050897	feature_id[164].value <= threshold=0.6870408356189728
node_918: feature_name=GO:0006139	feature_id[474].value > threshold=1.3052097624921544e-07
node_920: feature_name=GO:0002821	feature_id[588].value <= threshold=13.81072187423706
node_921: feature_name=GO:0006298	feature_id[621].value <= threshold=24.078600883483887
node_922: feature_name=GO:0003908	feature_id[11].value <= threshold=1.7776933312416077
node_923: feature_name=GO:0030098	feature_id[273].value > threshold=0.0079949083738029
node_927: feature_name=GO:0006808	feature_id[513].value <= threshold=4.34592080116272
node_928: feature_name=GO:0071887	feature_id[283].value <= threshold=10.353787899017334
node_929: feature_name=GO:0038001	feature_id[28].value <= threshold=3.9469382762908936
node_930: feature_name=GO:0042287	feature_id[20].value <= threshold=3.6209195852279663
node_931: feature_name=GO:0003968	feature_id[47].value <= threshold=2.004227638244629
node_932: feature_name=GO:0002698	feature_id[395].value <= threshold=18.51447582244873
node_933: feature_name=GO:0044710	feature_id[719].value <= threshold=179.7340316772461
node_934: feature_name=GO:0007568	feature_id[534].value > threshold=5.035318374633789
node_936: feature_name=GO:0006216	feature_id[504].value <= threshold=2.2605666518211365
node_937: feature_name=GO:0048569	feature_id[793].value <= threshold=5.340231895446777
node_938: feature_name=GO:0001777	feature_id[380].value <= threshold=4.100832939147949
node_939: feature_name=GO:0007600	feature_id[122].value <= threshold=171.13383102416992
node_940: feature_name=GO:0001779	feature_id[378].value <= threshold=6.234851121902466
node_941: feature_name=GO:0030291	feature_id[147].value <= threshold=10.8051118850708
node_942: feature_name=GO:0048534	feature_id[790].value > threshold=0.017984486185014248
node_944: feature_name=GO:0070245	feature_id[326].value <= threshold=3.1282339096069336
node_945: feature_name=GO:0009086	feature_id[547].value <= threshold=0.2896959036588669
node_946: feature_name=GO:0001889	feature_id[387].value <= threshold=17.643128395080566
node_947: feature_name=GO:0048144	feature_id[192].value > threshold=0.20887330174446106
node_951: feature_name=GO:0023026	feature_id[69].value <= threshold=3.403424024581909
node_952: feature_name=GO:0048147	feature_id[788].value <= threshold=4.188283443450928
node_953: feature_name=GO:0090116	feature_id[97].value <= threshold=4.617154359817505
node_954: feature_name=GO:0002863	feature_id[490].value <= threshold=4.8968470096588135

node_955: feature_name=GO:0002524 feature_id[72].value <= threshold=3.035340189933777
node_956: feature_name=GO:0042130 feature_id[16].value <= threshold=7.285413980484009
node_957: feature_name=GO:0071456 feature_id[500].value <= threshold=21.212870597839355
node_958: feature_name=GO:2001238 feature_id[27].value <= threshold=7.849715709686279
node_959: feature_name=GO:0023030 feature_id[45].value <= threshold=1.7420591711997986
node_960: feature_name=GO:0072593 feature_id[381].value > threshold=22.993029594421387
node_986: feature_name=hsa04115 feature_id[324].value > threshold=2.2171006202697754
Class: negative genes

Rules_112 passed counts:9
node_0: feature_name=GO:0042113 feature_id[0].value <= threshold=13.408552169799805
node_1: feature_name=GO:0007568 feature_id[534].value <= threshold=5.0313897132873535
node_2: feature_name=GO:0002705 feature_id[541].value <= threshold=3.200145721435547
node_3: feature_name=GO:1901525 feature_id[576].value > threshold=0.4466460347175598
node_617: feature_name=GO:0005622 feature_id[233].value <= threshold=90.85730743408203
node_618: feature_name=GO:1903147 feature_id[200].value <= threshold=7.326428413391113
node_619: feature_name=GO:0003964 feature_id[317].value <= threshold=5.575642108917236
node_620: feature_name=GO:0046498 feature_id[203].value <= threshold=6.620223522186279
node_621: feature_name=GO:0002562 feature_id[25].value <= threshold=10.287704467773438
node_622: feature_name=GO:0006919 feature_id[246].value <= threshold=5.801334381103516
node_623: feature_name=GO:0001889 feature_id[387].value <= threshold=2.679414987564087
node_624: feature_name=GO:0006555 feature_id[514].value <= threshold=7.819535493850708
node_625: feature_name=GO:0080134 feature_id[650].value <= threshold=38.91967582702637
node_626: feature_name=GO:0042127 feature_id[480].value <= threshold=37.72536659240723
node_627: feature_name=GO:2001251 feature_id[220].value <= threshold=32.564422607421875
node_628: feature_name=GO:0002829 feature_id[493].value <= threshold=2.444612741470337
node_629: feature_name=GO:0008588 feature_id[26].value <= threshold=2.3601274490356445
node_630: feature_name=GO:0033151 feature_id[7].value <= threshold=5.134376287460327
node_631: feature_name=GO:0048145 feature_id[348].value <= threshold=8.836549282073975
node_632: feature_name=GO:0030887 feature_id[274].value <= threshold=1.7000296115875244
node_633: feature_name=GO:0097190 feature_id[117].value <= threshold=33.02078819274902
node_634: feature_name=GO:0046685 feature_id[782].value <= threshold=8.040813446044922
node_635: feature_name=GO:0006346 feature_id[236].value <= threshold=6.384642839431763
node_636: feature_name=GO:0048537 feature_id[66].value <= threshold=3.1739262342453003
node_637: feature_name=GO:0070245 feature_id[326].value <= threshold=3.0491198301315308

node_638: feature_name=GO:0038065 feature_id[670].value <= threshold=2.0590850114822388
node_639: feature_name=GO:0005488 feature_id[187].value <= threshold=84.80076217651367
node_640: feature_name=GO:0060249 feature_id[390].value > threshold=0.07226398587226868
node_652: feature_name=GO:0006974 feature_id[516].value > threshold=5.17782768838515e-06
node_654: feature_name=hsa05210 feature_id[358].value <= threshold=18.730005264282227
node_655: feature_name=GO:0071850 feature_id[40].value <= threshold=5.475317001342773
node_656: feature_name=GO:1904029 feature_id[208].value <= threshold=26.575013160705566
node_657: feature_name=GO:0090594 feature_id[738].value <= threshold=3.000791072845459
node_658: feature_name=GO:0010948 feature_id[569].value > threshold=0.04386143572628498
node_666: feature_name=GO:0032764 feature_id[3].value <= threshold=2.5201677083969116
node_667: feature_name=GO:0023026 feature_id[69].value > threshold=3.2760006189346313
node_737: feature_name=GO:0033158 feature_id[379].value > threshold=0.46020837128162384
Class: negative genes

Rules_113 passed counts:9
node_0: feature_name=GO:0042113 feature_id[0].value <= threshold=13.408552169799805
node_1: feature_name=GO:0007568 feature_id[534].value <= threshold=5.0313897132873535
node_2: feature_name=GO:0002705 feature_id[541].value <= threshold=3.200145721435547
node_3: feature_name=GO:1901525 feature_id[576].value <= threshold=0.4466460347175598
node_4: feature_name=GO:0048539 feature_id[319].value <= threshold=3.0399646759033203
node_5: feature_name=GO:0001910 feature_id[385].value <= threshold=3.7437864542007446
node_6: feature_name=GO:0043200 feature_id[706].value <= threshold=9.307284355163574
node_7: feature_name=GO:0001773 feature_id[308].value <= threshold=4.016931533813477
node_8: feature_name=GO:0090116 feature_id[97].value <= threshold=7.99645471572876
node_9: feature_name=GO:0019814 feature_id[189].value <= threshold=4.525782108306885
node_10: feature_name=GO:1902583 feature_id[215].value <= threshold=17.60310649871826
node_11: feature_name=GO:0045429 feature_id[737].value <= threshold=1.6133361458778381
node_12: feature_name=GO:0003720 feature_id[228].value <= threshold=5.519326210021973
node_13: feature_name=GO:0046006 feature_id[364].value <= threshold=4.9985432624816895
node_14: feature_name=GO:0070424 feature_id[254].value <= threshold=6.233297109603882
node_15: feature_name=GO:0009892 feature_id[320].value <= threshold=59.99180793762207
node_16: feature_name=GO:0007064 feature_id[527].value <= threshold=11.57377815246582
node_17: feature_name=GO:0005575 feature_id[17].value <= threshold=15.417460918426514
node_18: feature_name=GO:0043368 feature_id[31].value <= threshold=4.059496641159058
node_19: feature_name=GO:0005164 feature_id[163].value <= threshold=5.793607711791992

node_20: feature_name=GO:0042130	feature_id[16].value <= threshold=5.073179721832275
node_21: feature_name=GO:0010216	feature_id[282].value <= threshold=4.834584474563599
node_22: feature_name=GO:0009628	feature_id[553].value <= threshold=29.314892768859863
node_23: feature_name=GO:0045628	feature_id[749].value <= threshold=3.1001367568969727
node_24: feature_name=GO:0042288	feature_id[100].value <= threshold=5.174932956695557
node_25: feature_name=GO:0002329	feature_id[406].value <= threshold=2.348930835723877
node_26: feature_name=GO:0051246	feature_id[642].value > threshold=0.6498909294605255
node_194: feature_name=GO:0009164	feature_id[546].value <= threshold=17.11970329284668
node_195: feature_name=GO:0001909	feature_id[386].value <= threshold=7.292566299438477
node_196: feature_name=GO:0003908	feature_id[11].value <= threshold=1.887226164340973
node_197: feature_name=GO:0009164	feature_id[546].value > threshold=0.26642198860645294
node_359: feature_name=GO:0009164	feature_id[546].value > threshold=0.2696942090988159
node_361: feature_name=hsa05202	feature_id[50].value <= threshold=14.989679336547852
node_362: feature_name=GO:0001775	feature_id[505].value <= threshold=15.726949691772461
node_363: feature_name=GO:0002634	feature_id[449].value <= threshold=2.825531005859375
node_364: feature_name=GO:0010038	feature_id[457].value <= threshold=9.901018619537354
node_365: feature_name=GO:0044238	feature_id[295].value <= threshold=139.80535888671875
node_366: feature_name=GO:0051246	feature_id[642].value > threshold=0.6527281999588013
node_368: feature_name=GO:0044092	feature_id[432].value <= threshold=11.084641933441162
node_369: feature_name=GO:0042162	feature_id[355].value <= threshold=9.683155536651611
node_370: feature_name=GO:0045945	feature_id[267].value <= threshold=3.6181851625442505
node_371: feature_name=GO:0036296	feature_id[252].value <= threshold=3.214281916618347
node_372: feature_name=GO:0097506	feature_id[51].value <= threshold=2.9644681215286255
node_373: feature_name=GO:0019740	feature_id[581].value <= threshold=2.2853575944900513
node_374: feature_name=GO:0019660	feature_id[70].value <= threshold=1.2831979990005493
node_375: feature_name=GO:0071901	feature_id[407].value <= threshold=14.498775482177734
node_376: feature_name=GO:0043170	feature_id[467].value > threshold=4.473330818655086e-06
node_380: feature_name=GO:0046718	feature_id[781].value <= threshold=3.494875192642212
node_381: feature_name=GO:0030983	feature_id[291].value > threshold=10.994179248809814
node_415: feature_name=GO:0009615	feature_id[265].value <= threshold=2.0143595933914185
Class: negative genes	

Rules_114	passed counts:9
node_0: feature_name=GO:0042113	feature_id[0].value <= threshold=13.408552169799805
node_1: feature_name=GO:0007568	feature_id[534].value <= threshold=5.0313897132873535

node_2: feature_name=GO:0002705	feature_id[541].value <= threshold=3.200145721435547
node_3: feature_name=GO:1901525	feature_id[576].value <= threshold=0.4466460347175598
node_4: feature_name=GO:0048539	feature_id[319].value <= threshold=3.0399646759033203
node_5: feature_name=GO:0001910	feature_id[385].value <= threshold=3.7437864542007446
node_6: feature_name=GO:0043200	feature_id[706].value <= threshold=9.307284355163574
node_7: feature_name=GO:0001773	feature_id[308].value <= threshold=4.016931533813477
node_8: feature_name=GO:0090116	feature_id[97].value <= threshold=7.99645471572876
node_9: feature_name=GO:0019814	feature_id[189].value <= threshold=4.525782108306885
node_10: feature_name=GO:1902583	feature_id[215].value <= threshold=17.60310649871826
node_11: feature_name=GO:0045429	feature_id[737].value <= threshold=1.6133361458778381
node_12: feature_name=GO:0003720	feature_id[228].value <= threshold=5.519326210021973
node_13: feature_name=GO:0046006	feature_id[364].value <= threshold=4.9985432624816895
node_14: feature_name=GO:0070424	feature_id[254].value <= threshold=6.233297109603882
node_15: feature_name=GO:0009892	feature_id[320].value <= threshold=59.99180793762207
node_16: feature_name=GO:0007064	feature_id[527].value <= threshold=11.57377815246582
node_17: feature_name=GO:0005575	feature_id[17].value <= threshold=15.417460918426514
node_18: feature_name=GO:0043368	feature_id[31].value <= threshold=4.059496641159058
node_19: feature_name=GO:0005164	feature_id[163].value <= threshold=5.793607711791992
node_20: feature_name=GO:0042130	feature_id[16].value <= threshold=5.073179721832275
node_21: feature_name=GO:0010216	feature_id[282].value <= threshold=4.834584474563599
node_22: feature_name=GO:0009628	feature_id[553].value <= threshold=29.314892768859863
node_23: feature_name=GO:0045628	feature_id[749].value <= threshold=3.1001367568969727
node_24: feature_name=GO:0042288	feature_id[100].value <= threshold=5.174932956695557
node_25: feature_name=GO:0002329	feature_id[406].value <= threshold=2.348930835723877
node_26: feature_name=GO:0051246	feature_id[642].value > threshold=0.6498909294605255
node_194: feature_name=GO:0009164	feature_id[546].value <= threshold=17.11970329284668
node_195: feature_name=GO:0001909	feature_id[386].value <= threshold=7.292566299438477
node_196: feature_name=GO:0003908	feature_id[11].value <= threshold=1.887226164340973
node_197: feature_name=GO:0009164	feature_id[546].value <= threshold=0.26642198860645294
node_198: feature_name=GO:2001242	feature_id[24].value <= threshold=9.207026481628418
node_199: feature_name=GO:0051246	feature_id[642].value > threshold=0.6504445374011993
node_203: feature_name=GO:0032461	feature_id[608].value <= threshold=2.607542634010315
node_204: feature_name=GO:0007584	feature_id[529].value <= threshold=16.099515914916992
node_205: feature_name=hsa05221	feature_id[349].value <= threshold=9.359850406646729
node_206: feature_name=GO:0002636	feature_id[447].value <= threshold=1.615447759628296

node_207: feature_name=GO:0033993 feature_id[421].value > threshold=2.96540611088858e-05
node_217: feature_name=GO:0002331 feature_id[404].value <= threshold=2.1883513927459717
node_218: feature_name=GO:0046498 feature_id[203].value <= threshold=4.085246205329895
node_219: feature_name=GO:0002832 feature_id[491].value <= threshold=3.0228612422943115
node_220: feature_name=GO:1904868 feature_id[306].value <= threshold=3.8674756288528442
node_221: feature_name=GO:0048545 feature_id[360].value <= threshold=27.38136100769043
node_222: feature_name=GO:0071310 feature_id[760].value <= threshold=2.3522024154663086
node_223: feature_name=GO:0001775 feature_id[505].value <= threshold=9.71301555633545
node_224: feature_name=GO:0071310 feature_id[760].value <= threshold=2.346743583679199
node_225: feature_name=GO:0001772 feature_id[91].value <= threshold=3.252573609352112
node_226: feature_name=GO:0036498 feature_id[359].value <= threshold=12.092026710510254
node_227: feature_name=hsa05340 feature_id[351].value <= threshold=0.7677814364433289
node_228: feature_name=GO:0032703 feature_id[617].value > threshold=1.7450646758079529
node_278: feature_name=GO:0002683 feature_id[456].value > threshold=0.05331694148480892
Class: negative genes

Rules_115 passed counts:9
node_0: feature_name=GO:0042113 feature_id[0].value <= threshold=13.408552169799805
node_1: feature_name=GO:0007568 feature_id[534].value <= threshold=5.0313897132873535
node_2: feature_name=GO:0002705 feature_id[541].value <= threshold=3.200145721435547
node_3: feature_name=GO:1901525 feature_id[576].value <= threshold=0.4466460347175598
node_4: feature_name=GO:0048539 feature_id[319].value <= threshold=3.0399646759033203
node_5: feature_name=GO:0001910 feature_id[385].value <= threshold=3.7437864542007446
node_6: feature_name=GO:0043200 feature_id[706].value <= threshold=9.307284355163574
node_7: feature_name=GO:0001773 feature_id[308].value <= threshold=4.016931533813477
node_8: feature_name=GO:0090116 feature_id[97].value <= threshold=7.99645471572876
node_9: feature_name=GO:0019814 feature_id[189].value <= threshold=4.525782108306885
node_10: feature_name=GO:1902583 feature_id[215].value <= threshold=17.60310649871826
node_11: feature_name=GO:0045429 feature_id[737].value <= threshold=1.6133361458778381
node_12: feature_name=GO:0003720 feature_id[228].value <= threshold=5.519326210021973
node_13: feature_name=GO:0046006 feature_id[364].value <= threshold=4.9985432624816895
node_14: feature_name=GO:0070424 feature_id[254].value <= threshold=6.233297109603882
node_15: feature_name=GO:0009892 feature_id[320].value <= threshold=59.99180793762207
node_16: feature_name=GO:0007064 feature_id[527].value <= threshold=11.57377815246582
node_17: feature_name=GO:0005575 feature_id[17].value <= threshold=15.417460918426514

node_18: feature_name=GO:0043368 feature_id[31].value <= threshold=4.059496641159058
node_19: feature_name=GO:0005164 feature_id[163].value <= threshold=5.793607711791992
node_20: feature_name=GO:0042130 feature_id[16].value <= threshold=5.073179721832275
node_21: feature_name=GO:0010216 feature_id[282].value <= threshold=4.834584474563599
node_22: feature_name=GO:0009628 feature_id[553].value <= threshold=29.314892768859863
node_23: feature_name=GO:0045628 feature_id[749].value <= threshold=3.1001367568969727
node_24: feature_name=GO:0042288 feature_id[100].value <= threshold=5.174932956695557
node_25: feature_name=GO:0002329 feature_id[406].value <= threshold=2.348930835723877
node_26: feature_name=GO:0051246 feature_id[642].value > threshold=0.6498909294605255
node_194: feature_name=GO:0009164 feature_id[546].value <= threshold=17.11970329284668
node_195: feature_name=GO:0001909 feature_id[386].value <= threshold=7.292566299438477
node_196: feature_name=GO:0003908 feature_id[11].value <= threshold=1.887226164340973
node_197: feature_name=GO:0009164 feature_id[546].value <= threshold=0.26642198860645294
node_198: feature_name=GO:2001242 feature_id[24].value <= threshold=9.207026481628418
node_199: feature_name=GO:0051246 feature_id[642].value > threshold=0.6504445374011993
node_203: feature_name=GO:0032461 feature_id[608].value <= threshold=2.607542634010315
node_204: feature_name=GO:0007584 feature_id[529].value <= threshold=16.099515914916992
node_205: feature_name=hsa05221 feature_id[349].value <= threshold=9.359850406646729
node_206: feature_name=GO:0002636 feature_id[447].value <= threshold=1.615447759628296
node_207: feature_name=GO:0033993 feature_id[421].value > threshold=2.96540611088858e-05
node_217: feature_name=GO:0002331 feature_id[404].value <= threshold=2.1883513927459717
node_218: feature_name=GO:0046498 feature_id[203].value <= threshold=4.085246205329895
node_219: feature_name=GO:0002832 feature_id[491].value <= threshold=3.0228612422943115
node_220: feature_name=GO:1904868 feature_id[306].value <= threshold=3.8674756288528442
node_221: feature_name=GO:0048545 feature_id[360].value <= threshold=27.38136100769043
node_222: feature_name=GO:0071310 feature_id[760].value <= threshold=2.3522024154663086
node_223: feature_name=GO:0001775 feature_id[505].value <= threshold=9.71301555633545
node_224: feature_name=GO:0071310 feature_id[760].value <= threshold=2.346743583679199
node_225: feature_name=GO:0001772 feature_id[91].value <= threshold=3.252573609352112
node_226: feature_name=GO:0036498 feature_id[359].value <= threshold=12.092026710510254
node_227: feature_name=hsa05340 feature_id[351].value <= threshold=0.7677814364433289
node_228: feature_name=GO:0032703 feature_id[617].value <= threshold=1.7450646758079529
node_229: feature_name=hsa04640 feature_id[79].value > threshold=2.118402123451233
node_275: feature_name=GO:0002250 feature_id[417].value > threshold=0.040423303842544556
Class: negative genes

Rules_116	passed counts:9
node_0: feature_name=GO:0042113	feature_id[0].value <= threshold=13.408552169799805
node_1: feature_name=GO:0007568	feature_id[534].value <= threshold=5.0313897132873535
node_2: feature_name=GO:0002705	feature_id[541].value <= threshold=3.200145721435547
node_3: feature_name=GO:1901525	feature_id[576].value <= threshold=0.4466460347175598
node_4: feature_name=GO:0048539	feature_id[319].value <= threshold=3.0399646759033203
node_5: feature_name=GO:0001910	feature_id[385].value <= threshold=3.7437864542007446
node_6: feature_name=GO:0043200	feature_id[706].value <= threshold=9.307284355163574
node_7: feature_name=GO:0001773	feature_id[308].value <= threshold=4.016931533813477
node_8: feature_name=GO:0090116	feature_id[97].value <= threshold=7.99645471572876
node_9: feature_name=GO:0019814	feature_id[189].value <= threshold=4.525782108306885
node_10: feature_name=GO:1902583	feature_id[215].value <= threshold=17.60310649871826
node_11: feature_name=GO:0045429	feature_id[737].value <= threshold=1.6133361458778381
node_12: feature_name=GO:0003720	feature_id[228].value <= threshold=5.519326210021973
node_13: feature_name=GO:0046006	feature_id[364].value <= threshold=4.9985432624816895
node_14: feature_name=GO:0070424	feature_id[254].value <= threshold=6.233297109603882
node_15: feature_name=GO:0009892	feature_id[320].value <= threshold=59.99180793762207
node_16: feature_name=GO:0007064	feature_id[527].value <= threshold=11.57377815246582
node_17: feature_name=GO:0005575	feature_id[17].value <= threshold=15.417460918426514
node_18: feature_name=GO:0043368	feature_id[31].value <= threshold=4.059496641159058
node_19: feature_name=GO:0005164	feature_id[163].value <= threshold=5.793607711791992
node_20: feature_name=GO:0042130	feature_id[16].value <= threshold=5.073179721832275
node_21: feature_name=GO:0010216	feature_id[282].value <= threshold=4.834584474563599
node_22: feature_name=GO:0009628	feature_id[553].value <= threshold=29.314892768859863
node_23: feature_name=GO:0045628	feature_id[749].value <= threshold=3.1001367568969727
node_24: feature_name=GO:0042288	feature_id[100].value <= threshold=5.174932956695557
node_25: feature_name=GO:0002329	feature_id[406].value <= threshold=2.348930835723877
node_26: feature_name=GO:0051246	feature_id[642].value > threshold=0.6498909294605255
node_194: feature_name=GO:0009164	feature_id[546].value <= threshold=17.11970329284668
node_195: feature_name=GO:0001909	feature_id[386].value <= threshold=7.292566299438477
node_196: feature_name=GO:0003908	feature_id[11].value <= threshold=1.887226164340973
node_197: feature_name=GO:0009164	feature_id[546].value <= threshold=0.26642198860645294
node_198: feature_name=GO:2001242	feature_id[24].value <= threshold=9.207026481628418
node_199: feature_name=GO:0051246	feature_id[642].value > threshold=0.6504445374011993

node_203: feature_name=GO:0032461 feature_id[608].value <= threshold=2.607542634010315
node_204: feature_name=GO:0007584 feature_id[529].value <= threshold=16.099515914916992
node_205: feature_name=hsa05221 feature_id[349].value <= threshold=9.359850406646729
node_206: feature_name=GO:0002636 feature_id[447].value <= threshold=1.615447759628296
node_207: feature_name=GO:0033993 feature_id[421].value > threshold=2.96540611088858e-05
node_217: feature_name=GO:0002331 feature_id[404].value <= threshold=2.1883513927459717
node_218: feature_name=GO:0046498 feature_id[203].value <= threshold=4.085246205329895
node_219: feature_name=GO:0002832 feature_id[491].value <= threshold=3.0228612422943115
node_220: feature_name=GO:1904868 feature_id[306].value <= threshold=3.8674756288528442
node_221: feature_name=GO:0048545 feature_id[360].value <= threshold=27.38136100769043
node_222: feature_name=GO:0071310 feature_id[760].value <= threshold=2.3522024154663086
node_223: feature_name=GO:0001775 feature_id[505].value <= threshold=9.71301555633545
node_224: feature_name=GO:0071310 feature_id[760].value <= threshold=2.346743583679199
node_225: feature_name=GO:0001772 feature_id[91].value <= threshold=3.252573609352112
node_226: feature_name=GO:0036498 feature_id[359].value <= threshold=12.092026710510254
node_227: feature_name=hsa05340 feature_id[351].value <= threshold=0.7677814364433289
node_228: feature_name=GO:0032703 feature_id[617].value <= threshold=1.7450646758079529
node_229: feature_name=hsa04640 feature_id[79].value <= threshold=2.118402123451233
node_230: feature_name=GO:0042097 feature_id[675].value <= threshold=1.8110727667808533
node_231: feature_name=GO:0071391 feature_id[518].value <= threshold=2.691588521003723
node_232: feature_name=GO:0001666 feature_id[682].value <= threshold=5.225740432739258
node_233: feature_name=GO:0048539 feature_id[319].value <= threshold=1.8523842096328735
node_234: feature_name=GO:0038061 feature_id[671].value <= threshold=8.564527988433838
node_235: feature_name=GO:0002484 feature_id[600].value <= threshold=3.1121134757995605
node_236: feature_name=GO:0023023 feature_id[119].value <= threshold=2.868617057800293
node_237: feature_name=GO:0010639 feature_id[564].value > threshold=13.410505771636963
node_251: feature_name=GO:0002696 feature_id[462].value <= threshold=0.6140799224376678
Class: negative genes

Rules_117 passed counts:9
node_0: feature_name=GO:0042113 feature_id[0].value <= threshold=13.408552169799805
node_1: feature_name=GO:0007568 feature_id[534].value <= threshold=5.0313897132873535
node_2: feature_name=GO:0002705 feature_id[541].value <= threshold=3.200145721435547
node_3: feature_name=GO:1901525 feature_id[576].value <= threshold=0.4466460347175598
node_4: feature_name=GO:0048539 feature_id[319].value <= threshold=3.0399646759033203

node_5: feature_name=GO:0001910 feature_id[385].value <= threshold=3.7437864542007446
node_6: feature_name=GO:0043200 feature_id[706].value <= threshold=9.307284355163574
node_7: feature_name=GO:0001773 feature_id[308].value <= threshold=4.016931533813477
node_8: feature_name=GO:0090116 feature_id[97].value <= threshold=7.99645471572876
node_9: feature_name=GO:0019814 feature_id[189].value <= threshold=4.525782108306885
node_10: feature_name=GO:1902583 feature_id[215].value <= threshold=17.60310649871826
node_11: feature_name=GO:0045429 feature_id[737].value <= threshold=1.6133361458778381
node_12: feature_name=GO:0003720 feature_id[228].value <= threshold=5.519326210021973
node_13: feature_name=GO:0046006 feature_id[364].value <= threshold=4.9985432624816895
node_14: feature_name=GO:0070424 feature_id[254].value <= threshold=6.233297109603882
node_15: feature_name=GO:0009892 feature_id[320].value <= threshold=59.99180793762207
node_16: feature_name=GO:0007064 feature_id[527].value <= threshold=11.57377815246582
node_17: feature_name=GO:0005575 feature_id[17].value <= threshold=15.417460918426514
node_18: feature_name=GO:0043368 feature_id[31].value <= threshold=4.059496641159058
node_19: feature_name=GO:0005164 feature_id[163].value <= threshold=5.793607711791992
node_20: feature_name=GO:0042130 feature_id[16].value <= threshold=5.073179721832275
node_21: feature_name=GO:0010216 feature_id[282].value <= threshold=4.834584474563599
node_22: feature_name=GO:0009628 feature_id[553].value <= threshold=29.314892768859863
node_23: feature_name=GO:0045628 feature_id[749].value <= threshold=3.1001367568969727
node_24: feature_name=GO:0042288 feature_id[100].value <= threshold=5.174932956695557
node_25: feature_name=GO:0002329 feature_id[406].value <= threshold=2.348930835723877
node_26: feature_name=GO:0051246 feature_id[642].value <= threshold=0.6498909294605255
node_27: feature_name=GO:0042162 feature_id[355].value <= threshold=8.05103588104248
node_28: feature_name=hsa04668 feature_id[333].value <= threshold=5.614926338195801
node_29: feature_name=GO:0032200 feature_id[613].value <= threshold=28.735913276672363
node_30: feature_name=hsa04662 feature_id[334].value <= threshold=9.588344097137451
node_31: feature_name=GO:1901992 feature_id[105].value <= threshold=5.291427850723267
node_32: feature_name=GO:0072341 feature_id[144].value <= threshold=6.01465106010437
node_33: feature_name=GO:0047485 feature_id[121].value <= threshold=6.657836437225342
node_34: feature_name=GO:0090594 feature_id[738].value <= threshold=1.8673912286758423
node_35: feature_name=GO:0043525 feature_id[524].value <= threshold=2.881397008895874
node_36: feature_name=GO:0032504 feature_id[244].value <= threshold=17.22207546234131
node_37: feature_name=GO:0051348 feature_id[126].value <= threshold=5.933559417724609
node_38: feature_name=GO:0042522 feature_id[692].value <= threshold=1.78694087266922
node_39: feature_name=GO:0031100 feature_id[73].value <= threshold=3.2312101125717163

node_40: feature_name=GO:0042493 feature_id[149].value <= threshold=19.771170616149902
node_41: feature_name=GO:0001552 feature_id[366].value <= threshold=2.1711617708206177
node_42: feature_name=GO:0046483 feature_id[391].value <= threshold=320.9667053222656
node_43: feature_name=GO:0002439 feature_id[431].value <= threshold=1.832722783088684
node_44: feature_name=GO:0006555 feature_id[514].value <= threshold=5.321640968322754
node_45: feature_name=GO:0070424 feature_id[254].value <= threshold=3.321003556251526
node_46: feature_name=hsa05144 feature_id[751].value <= threshold=2.635499954223633
node_47: feature_name=GO:0032633 feature_id[627].value <= threshold=1.2592533230781555
node_48: feature_name=GO:0019814 feature_id[189].value > threshold=2.1583125591278076
node_122: feature_name=GO:0008156 feature_id[239].value <= threshold=1.1327534914016724
Class: negative genes

Rules_118 passed counts:8
node_0: feature_name=GO:0042113 feature_id[0].value > threshold=13.408552169799805
node_1454: feature_name=GO:0050851 feature_id[29].value > threshold=9.650307655334473
node_1534: feature_name=GO:1902166 feature_id[301].value > threshold=0.303210511803627
node_1548: feature_name=GO:0035872 feature_id[659].value > threshold=35.09038162231445
node_1594: feature_name=hsa05210 feature_id[358].value <= threshold=27.461133003234863
Class: negative genes

Rules_119 passed counts:8
node_0: feature_name=GO:0042113 feature_id[0].value > threshold=13.408552169799805
node_1454: feature_name=GO:0050851 feature_id[29].value > threshold=9.650307655334473
node_1534: feature_name=GO:1902166 feature_id[301].value <= threshold=0.303210511803627
node_1535: feature_name=GO:0042287 feature_id[20].value <= threshold=1.4291933178901672
node_1536: feature_name=GO:0023026 feature_id[69].value > threshold=1.7696257829666138
node_1542: feature_name=GO:0042288 feature_id[100].value > threshold=0.17694193124771118
Class: positive genes

Rules_120 passed counts:8
node_0: feature_name=GO:0042113 feature_id[0].value <= threshold=13.408552169799805
node_1: feature_name=GO:0007568 feature_id[534].value > threshold=5.0313897132873535
node_913: feature_name=GO:0032763 feature_id[629].value > threshold=0.31753237545490265
node_1195: feature_name=GO:0071301 feature_id[442].value <= threshold=0.11534593254327774
node_1196: feature_name=GO:0046500 feature_id[171].value > threshold=0.6314916908740997

node_1340: feature_name=GO:0043627 feature_id[562].value > threshold=25.17277240753174
node_1368: feature_name=GO:0071310 feature_id[760].value > threshold=80.00252914428711
node_1372: feature_name=GO:0044403 feature_id[723].value > threshold=6.509235143661499
Class: negative genes

Rules_121 passed counts:8
node_0: feature_name=GO:0042113 feature_id[0].value <= threshold=13.408552169799805
node_1: feature_name=GO:0007568 feature_id[534].value <= threshold=5.0313897132873535
node_2: feature_name=GO:0002705 feature_id[541].value <= threshold=3.200145721435547
node_3: feature_name=GO:1901525 feature_id[576].value > threshold=0.4466460347175598
node_617: feature_name=GO:0005622 feature_id[233].value > threshold=90.85730743408203
node_847: feature_name=GO:0002639 feature_id[466].value > threshold=0.32853202521800995
node_867: feature_name=GO:0046498 feature_id[203].value <= threshold=0.5923898071050644
Class: positive genes

Rules_122 passed counts:8
node_0: feature_name=GO:0042113 feature_id[0].value <= threshold=13.408552169799805
node_1: feature_name=GO:0007568 feature_id[534].value <= threshold=5.0313897132873535
node_2: feature_name=GO:0002705 feature_id[541].value <= threshold=3.200145721435547
node_3: feature_name=GO:1901525 feature_id[576].value > threshold=0.4466460347175598
node_617: feature_name=GO:0005622 feature_id[233].value <= threshold=90.85730743408203
node_618: feature_name=GO:1903147 feature_id[200].value <= threshold=7.326428413391113
node_619: feature_name=GO:0003964 feature_id[317].value <= threshold=5.575642108917236
node_620: feature_name=GO:0046498 feature_id[203].value <= threshold=6.620223522186279
node_621: feature_name=GO:0002562 feature_id[25].value <= threshold=10.287704467773438
node_622: feature_name=GO:0006919 feature_id[246].value > threshold=5.801334381103516
node_834: feature_name=GO:0045931 feature_id[266].value <= threshold=0.973282665014267
Class: negative genes

Rules_123 passed counts:8
node_0: feature_name=GO:0042113 feature_id[0].value <= threshold=13.408552169799805
node_1: feature_name=GO:0007568 feature_id[534].value <= threshold=5.0313897132873535
node_2: feature_name=GO:0002705 feature_id[541].value <= threshold=3.200145721435547
node_3: feature_name=GO:1901525 feature_id[576].value <= threshold=0.4466460347175598
node_4: feature_name=GO:0048539 feature_id[319].value <= threshold=3.0399646759033203

node_5: feature_name=GO:0001910 feature_id[385].value <= threshold=3.7437864542007446
node_6: feature_name=GO:0043200 feature_id[706].value <= threshold=9.307284355163574
node_7: feature_name=GO:0001773 feature_id[308].value <= threshold=4.016931533813477
node_8: feature_name=GO:0090116 feature_id[97].value <= threshold=7.99645471572876
node_9: feature_name=GO:0019814 feature_id[189].value <= threshold=4.525782108306885
node_10: feature_name=GO:1902583 feature_id[215].value <= threshold=17.60310649871826
node_11: feature_name=GO:0045429 feature_id[737].value > threshold=1.6133361458778381
node_477: feature_name=GO:0036037 feature_id[445].value <= threshold=1.7181594371795654
node_478: feature_name=GO:0006244 feature_id[503].value <= threshold=0.951388418674469
node_479: feature_name=GO:0032461 feature_id[608].value <= threshold=2.6103241443634033
node_480: feature_name=GO:0044710 feature_id[719].value > threshold=2.228096718681627e-06
node_494: feature_name=GO:0009314 feature_id[296].value <= threshold=34.93696117401123
node_495: feature_name=GO:0050778 feature_id[802].value <= threshold=39.29364013671875
node_496: feature_name=GO:1903038 feature_id[492].value <= threshold=5.849650859832764
node_497: feature_name=GO:0002699 feature_id[606].value <= threshold=5.5405943393707275
node_498: feature_name=hsa00983 feature_id[181].value <= threshold=25.851045608520508
node_499: feature_name=GO:0002309 feature_id[416].value <= threshold=1.6235689520835876
node_500: feature_name=GO:1901698 feature_id[792].value <= threshold=47.70275688171387
node_501: feature_name=GO:0010216 feature_id[282].value <= threshold=1.6849713921546936
node_502: feature_name=GO:0006266 feature_id[297].value <= threshold=2.7199249267578125
node_503: feature_name=GO:0005488 feature_id[187].value > threshold=3.0373169010999845e-06
node_507: feature_name=GO:0045429 feature_id[737].value > threshold=1.614579975605011
node_511: feature_name=GO:0034103 feature_id[318].value <= threshold=5.832815647125244
node_512: feature_name=GO:0010663 feature_id[561].value <= threshold=4.157005429267883
node_513: feature_name=GO:0030887 feature_id[274].value <= threshold=1.8964150547981262
node_514: feature_name=GO:0048294 feature_id[113].value <= threshold=1.866134524345398
node_515: feature_name=GO:0072539 feature_id[68].value <= threshold=0.8600535988807678
node_516: feature_name=GO:0045840 feature_id[697].value > threshold=4.963376045227051
node_544: feature_name=GO:0030888 feature_id[120].value <= threshold=0.6045695245265961
Class: negative genes

Rules_124 passed counts:8
node_0: feature_name=GO:0042113 feature_id[0].value <= threshold=13.408552169799805
node_1: feature_name=GO:0007568 feature_id[534].value <= threshold=5.0313897132873535
node_2: feature_name=GO:0002705 feature_id[541].value <= threshold=3.200145721435547

node_3: feature_name=GO:1901525 feature_id[576].value <= threshold=0.4466460347175598
node_4: feature_name=GO:0048539 feature_id[319].value <= threshold=3.0399646759033203
node_5: feature_name=GO:0001910 feature_id[385].value <= threshold=3.7437864542007446
node_6: feature_name=GO:0043200 feature_id[706].value <= threshold=9.307284355163574
node_7: feature_name=GO:0001773 feature_id[308].value <= threshold=4.016931533813477
node_8: feature_name=GO:0090116 feature_id[97].value <= threshold=7.99645471572876
node_9: feature_name=GO:0019814 feature_id[189].value <= threshold=4.525782108306885
node_10: feature_name=GO:1902583 feature_id[215].value <= threshold=17.60310649871826
node_11: feature_name=GO:0045429 feature_id[737].value > threshold=1.6133361458778381
node_477: feature_name=GO:0036037 feature_id[445].value <= threshold=1.7181594371795654
node_478: feature_name=GO:0006244 feature_id[503].value <= threshold=0.951388418674469
node_479: feature_name=GO:0032461 feature_id[608].value <= threshold=2.6103241443634033
node_480: feature_name=GO:0044710 feature_id[719].value > threshold=2.228096718681627e-06
node_494: feature_name=GO:0009314 feature_id[296].value <= threshold=34.93696117401123
node_495: feature_name=GO:0050778 feature_id[802].value <= threshold=39.29364013671875
node_496: feature_name=GO:1903038 feature_id[492].value <= threshold=5.849650859832764
node_497: feature_name=GO:0002699 feature_id[606].value <= threshold=5.5405943393707275
node_498: feature_name=hsa00983 feature_id[181].value <= threshold=25.851045608520508
node_499: feature_name=GO:0002309 feature_id[416].value <= threshold=1.6235689520835876
node_500: feature_name=GO:1901698 feature_id[792].value <= threshold=47.70275688171387
node_501: feature_name=GO:0010216 feature_id[282].value <= threshold=1.6849713921546936
node_502: feature_name=GO:0006266 feature_id[297].value <= threshold=2.7199249267578125
node_503: feature_name=GO:0005488 feature_id[187].value > threshold=3.0373169010999845e-06
node_507: feature_name=GO:0045429 feature_id[737].value > threshold=1.614579975605011
node_511: feature_name=GO:0034103 feature_id[318].value <= threshold=5.832815647125244
node_512: feature_name=GO:0010663 feature_id[561].value <= threshold=4.157005429267883
node_513: feature_name=GO:0030887 feature_id[274].value <= threshold=1.8964150547981262
node_514: feature_name=GO:0048294 feature_id[113].value <= threshold=1.866134524345398
node_515: feature_name=GO:0072539 feature_id[68].value <= threshold=0.8600535988807678
node_516: feature_name=GO:0045840 feature_id[697].value <= threshold=4.963376045227051
node_517: feature_name=GO:0019222 feature_id[129].value > threshold=4.0122095015249215e-06
node_523: feature_name=GO:0008150 feature_id[528].value <= threshold=40.503862380981445
node_524: feature_name=GO:0006359 feature_id[87].value <= threshold=2.954534411430359
node_525: feature_name=GO:0010225 feature_id[558].value <= threshold=1.8247058987617493
node_526: feature_name=GO:0032461 feature_id[608].value <= threshold=1.6246166825294495

node_527: feature_name=GO:0007406 feature_id[537].value > threshold=2.375624656677246
node_529: feature_name=GO:0042368 feature_id[676].value <= threshold=0.4141124486923218
Class: negative genes

Rules_125 passed counts:7
node_0: feature_name=GO:0042113 feature_id[0].value > threshold=13.408552169799805
node_1454: feature_name=GO:0050851 feature_id[29].value > threshold=9.650307655334473
node_1534: feature_name=GO:1902166 feature_id[301].value > threshold=0.303210511803627
node_1548: feature_name=GO:0035872 feature_id[659].value <= threshold=35.09038162231445
node_1549: feature_name=GO:0032069 feature_id[614].value <= threshold=3.441983938217163
node_1550: feature_name=GO:0050897 feature_id[164].value > threshold=1.3939869403839111
node_1568: feature_name=GO:0007406 feature_id[537].value <= threshold=4.206701993942261
Class: negative genes

Rules_126 passed counts:7
node_0: feature_name=GO:0042113 feature_id[0].value > threshold=13.408552169799805
node_1454: feature_name=GO:0050851 feature_id[29].value > threshold=9.650307655334473
node_1534: feature_name=GO:1902166 feature_id[301].value > threshold=0.303210511803627
node_1548: feature_name=GO:0035872 feature_id[659].value <= threshold=35.09038162231445
node_1549: feature_name=GO:0032069 feature_id[614].value <= threshold=3.441983938217163
node_1550: feature_name=GO:0050897 feature_id[164].value <= threshold=1.3939869403839111
node_1551: feature_name=GO:0051454 feature_id[307].value <= threshold=0.9144491851329803
node_1552: feature_name=GO:0035825 feature_id[662].value > threshold=0.08555268123745918
node_1556: feature_name=GO:0031265 feature_id[48].value <= threshold=0.7173363864421844
node_1557: feature_name=GO:0002200 feature_id[82].value > threshold=5.254605531692505
Class: negative genes

Rules_127 passed counts:7
node_0: feature_name=GO:0042113 feature_id[0].value > threshold=13.408552169799805
node_1454: feature_name=GO:0050851 feature_id[29].value <= threshold=9.650307655334473
node_1455: feature_name=GO:0006304 feature_id[510].value <= threshold=5.960662126541138
node_1456: feature_name=GO:0032673 feature_id[623].value <= threshold=4.092082738876343
node_1457: feature_name=GO:0002429 feature_id[748].value > threshold=4.626799821853638
node_1463: feature_name=GO:0015672 feature_id[573].value > threshold=0.08226438239216805
node_1475: feature_name=GO:0030217 feature_id[589].value > threshold=19.77315902709961

node_1497: feature_name=GO:0030291 feature_id[147].value > threshold=4.140083312988281
node_1507: feature_name=GO:0006213 feature_id[507].value <= threshold=0.31206823140382767
Class: positive genes

Rules_128 passed counts:7
node_0: feature_name=GO:0042113 feature_id[0].value <= threshold=13.408552169799805
node_1: feature_name=GO:0007568 feature_id[534].value > threshold=5.0313897132873535
node_913: feature_name=GO:0032763 feature_id[629].value > threshold=0.31753237545490265
node_1195: feature_name=GO:0071301 feature_id[442].value > threshold=0.11534593254327774
node_1375: feature_name=GO:0046500 feature_id[171].value <= threshold=1.130434513092041
node_1376: feature_name=GO:0001782 feature_id[240].value <= threshold=6.639636993408203
node_1377: feature_name=GO:0043371 feature_id[707].value <= threshold=2.562113642692566
node_1378: feature_name=GO:0030291 feature_id[147].value <= threshold=6.834916830062866
node_1379: feature_name=GO:0045656 feature_id[756].value > threshold=0.6281269192695618
node_1393: feature_name=GO:0046007 feature_id[152].value > threshold=1.020785927772522
node_1419: feature_name=GO:0009636 feature_id[551].value > threshold=15.796520233154297
Class: negative genes

Rules_129 passed counts:7
node_0: feature_name=GO:0042113 feature_id[0].value <= threshold=13.408552169799805
node_1: feature_name=GO:0007568 feature_id[534].value > threshold=5.0313897132873535
node_913: feature_name=GO:0032763 feature_id[629].value > threshold=0.31753237545490265
node_1195: feature_name=GO:0071301 feature_id[442].value > threshold=0.11534593254327774
node_1375: feature_name=GO:0046500 feature_id[171].value <= threshold=1.130434513092041
node_1376: feature_name=GO:0001782 feature_id[240].value <= threshold=6.639636993408203
node_1377: feature_name=GO:0043371 feature_id[707].value <= threshold=2.562113642692566
node_1378: feature_name=GO:0030291 feature_id[147].value <= threshold=6.834916830062866
node_1379: feature_name=GO:0045656 feature_id[756].value > threshold=0.6281269192695618
node_1393: feature_name=GO:0046007 feature_id[152].value <= threshold=1.020785927772522
node_1394: feature_name=GO:0000302 feature_id[375].value <= threshold=20.847331047058105
node_1395: feature_name=hsa04110 feature_id[328].value > threshold=43.297359466552734
node_1411: feature_name=GO:0002318 feature_id[413].value > threshold=0.8484916985034943
Class: positive genes

Rules_130 passed counts:7

node_0: feature_name=GO:0042113 feature_id[0].value <= threshold=13.408552169799805
node_1: feature_name=GO:0007568 feature_id[534].value > threshold=5.0313897132873535
node_913: feature_name=GO:0032763 feature_id[629].value > threshold=0.31753237545490265
node_1195: feature_name=GO:0071301 feature_id[442].value > threshold=0.11534593254327774
node_1375: feature_name=GO:0046500 feature_id[171].value <= threshold=1.130434513092041
node_1376: feature_name=GO:0001782 feature_id[240].value <= threshold=6.639636993408203
node_1377: feature_name=GO:0043371 feature_id[707].value <= threshold=2.562113642692566
node_1378: feature_name=GO:0030291 feature_id[147].value <= threshold=6.834916830062866
node_1379: feature_name=GO:0045656 feature_id[756].value > threshold=0.6281269192695618
node_1393: feature_name=GO:0046007 feature_id[152].value <= threshold=1.020785927772522
node_1394: feature_name=GO:0000302 feature_id[375].value <= threshold=20.847331047058105
node_1395: feature_name=hsa04110 feature_id[328].value > threshold=43.297359466552734
node_1411: feature_name=GO:0002318 feature_id[413].value <= threshold=0.8484916985034943
node_1412: feature_name=GO:0045191 feature_id[730].value > threshold=0.5140747725963593
Class: negative genes

Rules_131 passed counts:7
node_0: feature_name=GO:0042113 feature_id[0].value <= threshold=13.408552169799805
node_1: feature_name=GO:0007568 feature_id[534].value > threshold=5.0313897132873535
node_913: feature_name=GO:0032763 feature_id[629].value > threshold=0.31753237545490265
node_1195: feature_name=GO:0071301 feature_id[442].value <= threshold=0.11534593254327774
node_1196: feature_name=GO:0046500 feature_id[171].value > threshold=0.6314916908740997
node_1340: feature_name=GO:0043627 feature_id[562].value <= threshold=25.17277240753174
node_1341: feature_name=GO:0010835 feature_id[560].value > threshold=1.0601619482040405
Class: positive genes

Rules_132 passed counts:7
node_0: feature_name=GO:0042113 feature_id[0].value <= threshold=13.408552169799805
node_1: feature_name=GO:0007568 feature_id[534].value > threshold=5.0313897132873535
node_913: feature_name=GO:0032763 feature_id[629].value > threshold=0.31753237545490265
node_1195: feature_name=GO:0071301 feature_id[442].value <= threshold=0.11534593254327774
node_1196: feature_name=GO:0046500 feature_id[171].value <= threshold=0.6314916908740997
node_1197: feature_name=GO:0030852 feature_id[22].value <= threshold=2.9768868684768677
node_1198: feature_name=hsa04672 feature_id[276].value > threshold=8.06681227684021
node_1326: feature_name=hsa04640 feature_id[79].value <= threshold=26.757009506225586

Class: positive genes

Rules_133 passed counts:7
node_0: feature_name=GO:0042113 feature_id[0].value <= threshold=13.408552169799805
node_1: feature_name=GO:0007568 feature_id[534].value > threshold=5.0313897132873535
node_913: feature_name=GO:0032763 feature_id[629].value <= threshold=0.31753237545490265
node_914: feature_name=GO:0097193 feature_id[167].value > threshold=28.171168327331543
node_1188: feature_name=GO:0002704 feature_id[470].value > threshold=0.8429980874061584
node_1192: feature_name=GO:0019660 feature_id[70].value > threshold=0.9662298560142517
Class: negative genes

Rules_134 passed counts:7
node_0: feature_name=GO:0042113 feature_id[0].value <= threshold=13.408552169799805
node_1: feature_name=GO:0007568 feature_id[534].value > threshold=5.0313897132873535
node_913: feature_name=GO:0032763 feature_id[629].value <= threshold=0.31753237545490265
node_914: feature_name=GO:0097193 feature_id[167].value <= threshold=28.171168327331543
node_915: feature_name=GO:0002903 feature_id[10].value > threshold=1.0534588098526
node_1159: feature_name=GO:0070198 feature_id[665].value > threshold=0.16219981759786606
node_1165: feature_name=GO:0042287 feature_id[20].value > threshold=0.915093183517456
node_1181: feature_name=GO:0060576 feature_id[204].value <= threshold=0.3862239122390747
node_1182: feature_name=GO:0042509 feature_id[694].value <= threshold=5.3463897705078125
Class: negative genes

Rules_135 passed counts:7
node_0: feature_name=GO:0042113 feature_id[0].value <= threshold=13.408552169799805
node_1: feature_name=GO:0007568 feature_id[534].value > threshold=5.0313897132873535
node_913: feature_name=GO:0032763 feature_id[629].value <= threshold=0.31753237545490265
node_914: feature_name=GO:0097193 feature_id[167].value <= threshold=28.171168327331543
node_915: feature_name=GO:0002903 feature_id[10].value <= threshold=1.0534588098526
node_916: feature_name=GO:1904029 feature_id[208].value <= threshold=23.852136611938477
node_917: feature_name=GO:0050897 feature_id[164].value > threshold=0.6870408356189728
node_1095: feature_name=GO:0072539 feature_id[68].value <= threshold=2.5667877197265625
node_1096: feature_name=GO:0046500 feature_id[171].value <= threshold=3.195197820663452
node_1097: feature_name=GO:0045861 feature_id[761].value > threshold=0.22614753991365433
node_1099: feature_name=GO:0043372 feature_id[242].value <= threshold=4.892506122589111

node_1100: feature_name=GO:0042162 feature_id[355].value <= threshold=11.339290618896484
node_1101: feature_name=hsa04660 feature_id[472].value > threshold=24.27848720550537
node_1143: feature_name=GO:0019814 feature_id[189].value > threshold=0.5058850646018982
node_1145: feature_name=GO:0032204 feature_id[747].value <= threshold=3.3982995748519897
Class: negative genes

Rules_136 passed counts:7
node_0: feature_name=GO:0042113 feature_id[0].value <= threshold=13.408552169799805
node_1: feature_name=GO:0007568 feature_id[534].value > threshold=5.0313897132873535
node_913: feature_name=GO:0032763 feature_id[629].value <= threshold=0.31753237545490265
node_914: feature_name=GO:0097193 feature_id[167].value <= threshold=28.171168327331543
node_915: feature_name=GO:0002903 feature_id[10].value <= threshold=1.0534588098526
node_916: feature_name=GO:1904029 feature_id[208].value <= threshold=23.852136611938477
node_917: feature_name=GO:0050897 feature_id[164].value > threshold=0.6870408356189728
node_1095: feature_name=GO:0072539 feature_id[68].value <= threshold=2.5667877197265625
node_1096: feature_name=GO:0046500 feature_id[171].value <= threshold=3.195197820663452
node_1097: feature_name=GO:0045861 feature_id[761].value > threshold=0.22614753991365433
node_1099: feature_name=GO:0043372 feature_id[242].value <= threshold=4.892506122589111
node_1100: feature_name=GO:0042162 feature_id[355].value <= threshold=11.339290618896484
node_1101: feature_name=hsa04660 feature_id[472].value <= threshold=24.27848720550537
node_1102: feature_name=GO:0007049 feature_id[259].value <= threshold=209.23831176757812
node_1103: feature_name=GO:0050897 feature_id[164].value > threshold=0.6919849812984467
node_1105: feature_name=GO:0045945 feature_id[267].value <= threshold=3.307852625846863
node_1106: feature_name=GO:0046641 feature_id[773].value <= threshold=3.527579426765442
node_1107: feature_name=GO:0002262 feature_id[394].value > threshold=0.08302562311291695
node_1111: feature_name=GO:0043226 feature_id[138].value > threshold=2.4930146992119262e-05
node_1113: feature_name=GO:0045787 feature_id[446].value > threshold=0.07611752673983574
node_1115: feature_name=GO:0045840 feature_id[697].value > threshold=0.10580676794052124
node_1117: feature_name=GO:0006927 feature_id[225].value <= threshold=4.950021743774414
node_1118: feature_name=GO:0002643 feature_id[4].value <= threshold=3.364396333694458
node_1119: feature_name=GO:0016571 feature_id[725].value <= threshold=38.268903732299805
node_1120: feature_name=GO:0072593 feature_id[381].value > threshold=19.997477531433105
node_1128: feature_name=GO:0006304 feature_id[510].value <= threshold=0.7586463987827301
Class: negative genes

Rules_137 passed counts:7
node_0: feature_name=GO:0042113 feature_id[0].value <= threshold=13.408552169799805
node_1: feature_name=GO:0007568 feature_id[534].value <= threshold=5.0313897132873535
node_2: feature_name=GO:0002705 feature_id[541].value > threshold=3.200145721435547
node_870: feature_name=GO:0046634 feature_id[46].value <= threshold=8.9394211769104
node_871: feature_name=GO:0048523 feature_id[293].value > threshold=19.963229179382324
node_909: feature_name=GO:0050731 feature_id[804].value > threshold=1.0901886820793152
Class: positive genes

Rules_138 passed counts:7
node_0: feature_name=GO:0042113 feature_id[0].value <= threshold=13.408552169799805
node_1: feature_name=GO:0007568 feature_id[534].value <= threshold=5.0313897132873535
node_2: feature_name=GO:0002705 feature_id[541].value > threshold=3.200145721435547
node_870: feature_name=GO:0046634 feature_id[46].value <= threshold=8.9394211769104
node_871: feature_name=GO:0048523 feature_id[293].value <= threshold=19.963229179382324
node_872: feature_name=GO:2001237 feature_id[796].value > threshold=0.09761488810181618
node_880: feature_name=GO:0006808 feature_id[513].value <= threshold=1.7969900965690613
node_881: feature_name=GO:0032479 feature_id[441].value <= threshold=0.13597124069929123
node_882: feature_name=GO:0000060 feature_id[223].value <= threshold=2.2508251667022705
node_883: feature_name=GO:0048144 feature_id[192].value > threshold=0.0181948971003294
Class: negative genes

Rules_139 passed counts:7
node_0: feature_name=GO:0042113 feature_id[0].value <= threshold=13.408552169799805
node_1: feature_name=GO:0007568 feature_id[534].value <= threshold=5.0313897132873535
node_2: feature_name=GO:0002705 feature_id[541].value <= threshold=3.200145721435547
node_3: feature_name=GO:1901525 feature_id[576].value <= threshold=0.4466460347175598
node_4: feature_name=GO:0048539 feature_id[319].value <= threshold=3.0399646759033203
node_5: feature_name=GO:0001910 feature_id[385].value <= threshold=3.7437864542007446
node_6: feature_name=GO:0043200 feature_id[706].value <= threshold=9.307284355163574
node_7: feature_name=GO:0001773 feature_id[308].value <= threshold=4.016931533813477
node_8: feature_name=GO:0090116 feature_id[97].value <= threshold=7.99645471572876
node_9: feature_name=GO:0019814 feature_id[189].value <= threshold=4.525782108306885
node_10: feature_name=GO:1902583 feature_id[215].value <= threshold=17.60310649871826
node_11: feature_name=GO:0045429 feature_id[737].value > threshold=1.6133361458778381

node_477: feature_name=GO:0036037 feature_id[445].value <= threshold=1.7181594371795654
node_478: feature_name=GO:0006244 feature_id[503].value <= threshold=0.951388418674469
node_479: feature_name=GO:0032461 feature_id[608].value <= threshold=2.6103241443634033
node_480: feature_name=GO:0044710 feature_id[719].value <= threshold=2.228096718681627e-06
node_481: feature_name=GO:1904035 feature_id[654].value <= threshold=0.05152595415711403
node_482: feature_name=GO:2001235 feature_id[495].value > threshold=1.1330987215042114
Class: positive genes

Rules_140 passed counts:7
node_0: feature_name=GO:0042113 feature_id[0].value <= threshold=13.408552169799805
node_1: feature_name=GO:0007568 feature_id[534].value <= threshold=5.0313897132873535
node_2: feature_name=GO:0002705 feature_id[541].value <= threshold=3.200145721435547
node_3: feature_name=GO:1901525 feature_id[576].value <= threshold=0.4466460347175598
node_4: feature_name=GO:0048539 feature_id[319].value <= threshold=3.0399646759033203
node_5: feature_name=GO:0001910 feature_id[385].value <= threshold=3.7437864542007446
node_6: feature_name=GO:0043200 feature_id[706].value <= threshold=9.307284355163574
node_7: feature_name=GO:0001773 feature_id[308].value <= threshold=4.016931533813477
node_8: feature_name=GO:0090116 feature_id[97].value <= threshold=7.99645471572876
node_9: feature_name=GO:0019814 feature_id[189].value <= threshold=4.525782108306885
node_10: feature_name=GO:1902583 feature_id[215].value <= threshold=17.60310649871826
node_11: feature_name=GO:0045429 feature_id[737].value > threshold=1.6133361458778381
node_477: feature_name=GO:0036037 feature_id[445].value <= threshold=1.7181594371795654
node_478: feature_name=GO:0006244 feature_id[503].value <= threshold=0.951388418674469
node_479: feature_name=GO:0032461 feature_id[608].value <= threshold=2.6103241443634033
node_480: feature_name=GO:0044710 feature_id[719].value <= threshold=2.228096718681627e-06
node_481: feature_name=GO:1904035 feature_id[654].value <= threshold=0.05152595415711403
node_482: feature_name=GO:2001235 feature_id[495].value <= threshold=1.1330987215042114
node_483: feature_name=hsa05169 feature_id[336].value <= threshold=8.282931327819824
Class: negative genes

Rules_141 passed counts:7
node_0: feature_name=GO:0042113 feature_id[0].value <= threshold=13.408552169799805
node_1: feature_name=GO:0007568 feature_id[534].value <= threshold=5.0313897132873535
node_2: feature_name=GO:0002705 feature_id[541].value <= threshold=3.200145721435547
node_3: feature_name=GO:1901525 feature_id[576].value <= threshold=0.4466460347175598

node_4: feature_name=GO:0048539 feature_id[319].value <= threshold=3.0399646759033203
node_5: feature_name=GO:0001910 feature_id[385].value <= threshold=3.7437864542007446
node_6: feature_name=GO:0043200 feature_id[706].value <= threshold=9.307284355163574
node_7: feature_name=GO:0001773 feature_id[308].value <= threshold=4.016931533813477
node_8: feature_name=GO:0090116 feature_id[97].value <= threshold=7.99645471572876
node_9: feature_name=GO:0019814 feature_id[189].value <= threshold=4.525782108306885
node_10: feature_name=GO:1902583 feature_id[215].value <= threshold=17.60310649871826
node_11: feature_name=GO:0045429 feature_id[737].value <= threshold=1.6133361458778381
node_12: feature_name=GO:0003720 feature_id[228].value <= threshold=5.519326210021973
node_13: feature_name=GO:0046006 feature_id[364].value <= threshold=4.9985432624816895
node_14: feature_name=GO:0070424 feature_id[254].value <= threshold=6.233297109603882
node_15: feature_name=GO:0009892 feature_id[320].value <= threshold=59.99180793762207
node_16: feature_name=GO:0007064 feature_id[527].value <= threshold=11.57377815246582
node_17: feature_name=GO:0005575 feature_id[17].value <= threshold=15.417460918426514
node_18: feature_name=GO:0043368 feature_id[31].value <= threshold=4.059496641159058
node_19: feature_name=GO:0005164 feature_id[163].value <= threshold=5.793607711791992
node_20: feature_name=GO:0042130 feature_id[16].value <= threshold=5.073179721832275
node_21: feature_name=GO:0010216 feature_id[282].value <= threshold=4.834584474563599
node_22: feature_name=GO:0009628 feature_id[553].value <= threshold=29.314892768859863
node_23: feature_name=GO:0045628 feature_id[749].value <= threshold=3.1001367568969727
node_24: feature_name=GO:0042288 feature_id[100].value <= threshold=5.174932956695557
node_25: feature_name=GO:0002329 feature_id[406].value <= threshold=2.348930835723877
node_26: feature_name=GO:0051246 feature_id[642].value > threshold=0.6498909294605255
node_194: feature_name=GO:0009164 feature_id[546].value <= threshold=17.11970329284668
node_195: feature_name=GO:0001909 feature_id[386].value <= threshold=7.292566299438477
node_196: feature_name=GO:0003908 feature_id[11].value > threshold=1.887226164340973
node_444: feature_name=GO:0034103 feature_id[318].value <= threshold=0.34810671210289
Class: negative genes

Rules_142 passed counts:7
node_0: feature_name=GO:0042113 feature_id[0].value <= threshold=13.408552169799805
node_1: feature_name=GO:0007568 feature_id[534].value <= threshold=5.0313897132873535
node_2: feature_name=GO:0002705 feature_id[541].value <= threshold=3.200145721435547
node_3: feature_name=GO:1901525 feature_id[576].value <= threshold=0.4466460347175598
node_4: feature_name=GO:0048539 feature_id[319].value <= threshold=3.0399646759033203

node_5: feature_name=GO:0001910	feature_id[385].value <= threshold=3.7437864542007446
node_6: feature_name=GO:0043200	feature_id[706].value <= threshold=9.307284355163574
node_7: feature_name=GO:0001773	feature_id[308].value <= threshold=4.016931533813477
node_8: feature_name=GO:0090116	feature_id[97].value <= threshold=7.99645471572876
node_9: feature_name=GO:0019814	feature_id[189].value <= threshold=4.525782108306885
node_10: feature_name=GO:1902583	feature_id[215].value <= threshold=17.60310649871826
node_11: feature_name=GO:0045429	feature_id[737].value <= threshold=1.6133361458778381
node_12: feature_name=GO:0003720	feature_id[228].value <= threshold=5.519326210021973
node_13: feature_name=GO:0046006	feature_id[364].value <= threshold=4.9985432624816895
node_14: feature_name=GO:0070424	feature_id[254].value <= threshold=6.233297109603882
node_15: feature_name=GO:0009892	feature_id[320].value <= threshold=59.99180793762207
node_16: feature_name=GO:0007064	feature_id[527].value <= threshold=11.57377815246582
node_17: feature_name=GO:0005575	feature_id[17].value <= threshold=15.417460918426514
node_18: feature_name=GO:0043368	feature_id[31].value <= threshold=4.059496641159058
node_19: feature_name=GO:0005164	feature_id[163].value <= threshold=5.793607711791992
node_20: feature_name=GO:0042130	feature_id[16].value <= threshold=5.073179721832275
node_21: feature_name=GO:0010216	feature_id[282].value <= threshold=4.834584474563599
node_22: feature_name=GO:0009628	feature_id[553].value <= threshold=29.314892768859863
node_23: feature_name=GO:0045628	feature_id[749].value <= threshold=3.1001367568969727
node_24: feature_name=GO:0042288	feature_id[100].value <= threshold=5.174932956695557
node_25: feature_name=GO:0002329	feature_id[406].value <= threshold=2.348930835723877
node_26: feature_name=GO:0051246	feature_id[642].value > threshold=0.6498909294605255
node_194: feature_name=GO:0009164	feature_id[546].value <= threshold=17.11970329284668
node_195: feature_name=GO:0001909	feature_id[386].value <= threshold=7.292566299438477
node_196: feature_name=GO:0003908	feature_id[11].value <= threshold=1.887226164340973
node_197: feature_name=GO:0009164	feature_id[546].value > threshold=0.26642198860645294
node_359: feature_name=GO:0009164	feature_id[546].value > threshold=0.2696942090988159
node_361: feature_name=hsa05202	feature_id[50].value <= threshold=14.989679336547852
node_362: feature_name=GO:0001775	feature_id[505].value <= threshold=15.726949691772461
node_363: feature_name=GO:0002634	feature_id[449].value <= threshold=2.825531005859375
node_364: feature_name=GO:0010038	feature_id[457].value <= threshold=9.901018619537354
node_365: feature_name=GO:0044238	feature_id[295].value <= threshold=139.80535888671875
node_366: feature_name=GO:0051246	feature_id[642].value > threshold=0.6527281999588013
node_368: feature_name=GO:0044092	feature_id[432].value <= threshold=11.084641933441162
node_369: feature_name=GO:0042162	feature_id[355].value <= threshold=9.683155536651611

node_370: feature_name=GO:0045945 feature_id[267].value <= threshold=3.6181851625442505
node_371: feature_name=GO:0036296 feature_id[252].value <= threshold=3.214281916618347
node_372: feature_name=GO:0097506 feature_id[51].value <= threshold=2.9644681215286255
node_373: feature_name=GO:0019740 feature_id[581].value <= threshold=2.2853575944900513
node_374: feature_name=GO:0019660 feature_id[70].value <= threshold=1.2831979990005493
node_375: feature_name=GO:0071901 feature_id[407].value <= threshold=14.498775482177734
node_376: feature_name=GO:0043170 feature_id[467].value > threshold=4.473330818655086e-06
node_380: feature_name=GO:0046718 feature_id[781].value > threshold=3.494875192642212
node_418: feature_name=GO:0002708 feature_id[526].value > threshold=0.14747196435928345
Class: negative genes

Rules_143 passed counts:7
node_0: feature_name=GO:0042113 feature_id[0].value <= threshold=13.408552169799805
node_1: feature_name=GO:0007568 feature_id[534].value <= threshold=5.0313897132873535
node_2: feature_name=GO:0002705 feature_id[541].value <= threshold=3.200145721435547
node_3: feature_name=GO:1901525 feature_id[576].value <= threshold=0.4466460347175598
node_4: feature_name=GO:0048539 feature_id[319].value <= threshold=3.0399646759033203
node_5: feature_name=GO:0001910 feature_id[385].value <= threshold=3.7437864542007446
node_6: feature_name=GO:0043200 feature_id[706].value <= threshold=9.307284355163574
node_7: feature_name=GO:0001773 feature_id[308].value <= threshold=4.016931533813477
node_8: feature_name=GO:0090116 feature_id[97].value <= threshold=7.99645471572876
node_9: feature_name=GO:0019814 feature_id[189].value <= threshold=4.525782108306885
node_10: feature_name=GO:1902583 feature_id[215].value <= threshold=17.60310649871826
node_11: feature_name=GO:0045429 feature_id[737].value <= threshold=1.6133361458778381
node_12: feature_name=GO:0003720 feature_id[228].value <= threshold=5.519326210021973
node_13: feature_name=GO:0046006 feature_id[364].value <= threshold=4.9985432624816895
node_14: feature_name=GO:0070424 feature_id[254].value <= threshold=6.233297109603882
node_15: feature_name=GO:0009892 feature_id[320].value <= threshold=59.99180793762207
node_16: feature_name=GO:0007064 feature_id[527].value <= threshold=11.57377815246582
node_17: feature_name=GO:0005575 feature_id[17].value <= threshold=15.417460918426514
node_18: feature_name=GO:0043368 feature_id[31].value <= threshold=4.059496641159058
node_19: feature_name=GO:0005164 feature_id[163].value <= threshold=5.793607711791992
node_20: feature_name=GO:0042130 feature_id[16].value <= threshold=5.073179721832275
node_21: feature_name=GO:0010216 feature_id[282].value <= threshold=4.834584474563599
node_22: feature_name=GO:0009628 feature_id[553].value <= threshold=29.314892768859863

node_23: feature_name=GO:0045628 feature_id[749].value <= threshold=3.1001367568969727
node_24: feature_name=GO:0042288 feature_id[100].value <= threshold=5.174932956695557
node_25: feature_name=GO:0002329 feature_id[406].value <= threshold=2.348930835723877
node_26: feature_name=GO:0051246 feature_id[642].value > threshold=0.6498909294605255
node_194: feature_name=GO:0009164 feature_id[546].value <= threshold=17.11970329284668
node_195: feature_name=GO:0001909 feature_id[386].value <= threshold=7.292566299438477
node_196: feature_name=GO:0003908 feature_id[11].value <= threshold=1.887226164340973
node_197: feature_name=GO:0009164 feature_id[546].value > threshold=0.26642198860645294
node_359: feature_name=GO:0009164 feature_id[546].value > threshold=0.2696942090988159
node_361: feature_name=hsa05202 feature_id[50].value <= threshold=14.989679336547852
node_362: feature_name=GO:0001775 feature_id[505].value <= threshold=15.726949691772461
node_363: feature_name=GO:0002634 feature_id[449].value <= threshold=2.825531005859375
node_364: feature_name=GO:0010038 feature_id[457].value <= threshold=9.901018619537354
node_365: feature_name=GO:0044238 feature_id[295].value <= threshold=139.80535888671875
node_366: feature_name=GO:0051246 feature_id[642].value > threshold=0.6527281999588013
node_368: feature_name=GO:0044092 feature_id[432].value <= threshold=11.084641933441162
node_369: feature_name=GO:0042162 feature_id[355].value <= threshold=9.683155536651611
node_370: feature_name=GO:0045945 feature_id[267].value <= threshold=3.6181851625442505
node_371: feature_name=GO:0036296 feature_id[252].value <= threshold=3.214281916618347
node_372: feature_name=GO:0097506 feature_id[51].value <= threshold=2.9644681215286255
node_373: feature_name=GO:0019740 feature_id[581].value <= threshold=2.2853575944900513
node_374: feature_name=GO:0019660 feature_id[70].value <= threshold=1.2831979990005493
node_375: feature_name=GO:0071901 feature_id[407].value <= threshold=14.498775482177734
node_376: feature_name=GO:0043170 feature_id[467].value > threshold=4.473330818655086e-06
node_380: feature_name=GO:0046718 feature_id[781].value <= threshold=3.494875192642212
node_381: feature_name=GO:0030983 feature_id[291].value <= threshold=10.994179248809814
node_382: feature_name=GO:0010663 feature_id[561].value <= threshold=2.7056833505630493
node_383: feature_name=GO:0031667 feature_id[648].value > threshold=0.0020462179090827703
node_387: feature_name=GO:1990572 feature_id[243].value <= threshold=1.9359037280082703
node_388: feature_name=GO:0032753 feature_id[101].value <= threshold=1.182796835899353
node_389: feature_name=GO:0097153 feature_id[226].value <= threshold=0.34601132571697235
node_390: feature_name=GO:0002829 feature_id[493].value > threshold=1.246164083480835
node_396: feature_name=GO:0032649 feature_id[626].value > threshold=0.03106011636555195
Class: negative genes

Rules_144	passed counts:7
node_0: feature_name=GO:0042113	feature_id[0].value <= threshold=13.408552169799805
node_1: feature_name=GO:0007568	feature_id[534].value <= threshold=5.0313897132873535
node_2: feature_name=GO:0002705	feature_id[541].value <= threshold=3.200145721435547
node_3: feature_name=GO:1901525	feature_id[576].value <= threshold=0.4466460347175598
node_4: feature_name=GO:0048539	feature_id[319].value <= threshold=3.0399646759033203
node_5: feature_name=GO:0001910	feature_id[385].value <= threshold=3.7437864542007446
node_6: feature_name=GO:0043200	feature_id[706].value <= threshold=9.307284355163574
node_7: feature_name=GO:0001773	feature_id[308].value <= threshold=4.016931533813477
node_8: feature_name=GO:0090116	feature_id[97].value <= threshold=7.99645471572876
node_9: feature_name=GO:0019814	feature_id[189].value <= threshold=4.525782108306885
node_10: feature_name=GO:1902583	feature_id[215].value <= threshold=17.60310649871826
node_11: feature_name=GO:0045429	feature_id[737].value <= threshold=1.6133361458778381
node_12: feature_name=GO:0003720	feature_id[228].value <= threshold=5.519326210021973
node_13: feature_name=GO:0046006	feature_id[364].value <= threshold=4.9985432624816895
node_14: feature_name=GO:0070424	feature_id[254].value <= threshold=6.233297109603882
node_15: feature_name=GO:0009892	feature_id[320].value <= threshold=59.99180793762207
node_16: feature_name=GO:0007064	feature_id[527].value <= threshold=11.57377815246582
node_17: feature_name=GO:0005575	feature_id[17].value <= threshold=15.417460918426514
node_18: feature_name=GO:0043368	feature_id[31].value <= threshold=4.059496641159058
node_19: feature_name=GO:0005164	feature_id[163].value <= threshold=5.793607711791992
node_20: feature_name=GO:0042130	feature_id[16].value <= threshold=5.073179721832275
node_21: feature_name=GO:0010216	feature_id[282].value <= threshold=4.834584474563599
node_22: feature_name=GO:0009628	feature_id[553].value <= threshold=29.314892768859863
node_23: feature_name=GO:0045628	feature_id[749].value <= threshold=3.1001367568969727
node_24: feature_name=GO:0042288	feature_id[100].value <= threshold=5.174932956695557
node_25: feature_name=GO:0002329	feature_id[406].value <= threshold=2.348930835723877
node_26: feature_name=GO:0051246	feature_id[642].value > threshold=0.6498909294605255
node_194: feature_name=GO:0009164	feature_id[546].value <= threshold=17.11970329284668
node_195: feature_name=GO:0001909	feature_id[386].value <= threshold=7.292566299438477
node_196: feature_name=GO:0003908	feature_id[11].value <= threshold=1.887226164340973
node_197: feature_name=GO:0009164	feature_id[546].value <= threshold=0.26642198860645294
node_198: feature_name=GO:2001242	feature_id[24].value <= threshold=9.207026481628418
node_199: feature_name=GO:0051246	feature_id[642].value > threshold=0.6504445374011993
node_203: feature_name=GO:0032461	feature_id[608].value <= threshold=2.607542634010315

node_204: feature_name=GO:0007584 feature_id[529].value <= threshold=16.099515914916992
node_205: feature_name=hsa05221 feature_id[349].value <= threshold=9.359850406646729
node_206: feature_name=GO:0002636 feature_id[447].value <= threshold=1.615447759628296
node_207: feature_name=GO:0033993 feature_id[421].value > threshold=2.96540611088858e-05
node_217: feature_name=GO:0002331 feature_id[404].value <= threshold=2.1883513927459717
node_218: feature_name=GO:0046498 feature_id[203].value <= threshold=4.085246205329895
node_219: feature_name=GO:0002832 feature_id[491].value <= threshold=3.0228612422943115
node_220: feature_name=GO:1904868 feature_id[306].value <= threshold=3.8674756288528442
node_221: feature_name=GO:0048545 feature_id[360].value <= threshold=27.38136100769043
node_222: feature_name=GO:0071310 feature_id[760].value <= threshold=2.3522024154663086
node_223: feature_name=GO:0001775 feature_id[505].value <= threshold=9.71301555633545
node_224: feature_name=GO:0071310 feature_id[760].value <= threshold=2.346743583679199
node_225: feature_name=GO:0001772 feature_id[91].value <= threshold=3.252573609352112
node_226: feature_name=GO:0036498 feature_id[359].value <= threshold=12.092026710510254
node_227: feature_name=hsa05340 feature_id[351].value <= threshold=0.7677814364433289
node_228: feature_name=GO:0032703 feature_id[617].value <= threshold=1.7450646758079529
node_229: feature_name=hsa04640 feature_id[79].value <= threshold=2.118402123451233
node_230: feature_name=GO:0042097 feature_id[675].value <= threshold=1.8110727667808533
node_231: feature_name=GO:0071391 feature_id[518].value <= threshold=2.691588521003723
node_232: feature_name=GO:0001666 feature_id[682].value <= threshold=5.225740432739258
node_233: feature_name=GO:0048539 feature_id[319].value <= threshold=1.8523842096328735
node_234: feature_name=GO:0038061 feature_id[671].value <= threshold=8.564527988433838
node_235: feature_name=GO:0002484 feature_id[600].value <= threshold=3.1121134757995605
node_236: feature_name=GO:0023023 feature_id[119].value > threshold=2.868617057800293
node_254: feature_name=GO:0019222 feature_id[129].value > threshold=0.11867357417941093
Class: negative genes

Rules_145 passed counts:6
node_0: feature_name=GO:0042113 feature_id[0].value > threshold=13.408552169799805
node_1454: feature_name=GO:0050851 feature_id[29].value <= threshold=9.650307655334473
node_1455: feature_name=GO:0006304 feature_id[510].value <= threshold=5.960662126541138
node_1456: feature_name=GO:0032673 feature_id[623].value <= threshold=4.092082738876343
node_1457: feature_name=GO:0002429 feature_id[748].value > threshold=4.626799821853638
node_1463: feature_name=GO:0015672 feature_id[573].value > threshold=0.08226438239216805
node_1475: feature_name=GO:0030217 feature_id[589].value <= threshold=19.77315902709961

node_1476: feature_name=GO:0002381 feature_id[96].value <= threshold=4.645626783370972
node_1477: feature_name=GO:0045058 feature_id[727].value > threshold=5.092289447784424
node_1485: feature_name=GO:0016447 feature_id[74].value > threshold=4.237667560577393
node_1487: feature_name=GO:0045628 feature_id[749].value <= threshold=3.333345651626587
Class: negative genes

Rules_146 passed counts:6
node_0: feature_name=GO:0042113 feature_id[0].value <= threshold=13.408552169799805
node_1: feature_name=GO:0007568 feature_id[534].value > threshold=5.0313897132873535
node_913: feature_name=GO:0032763 feature_id[629].value > threshold=0.31753237545490265
node_1195: feature_name=GO:0071301 feature_id[442].value > threshold=0.11534593254327774
node_1375: feature_name=GO:0046500 feature_id[171].value > threshold=1.130434513092041
node_1437: feature_name=GO:0033343 feature_id[639].value <= threshold=0.98012974858284
node_1438: feature_name=GO:0019058 feature_id[245].value > threshold=11.779548168182373
node_1440: feature_name=GO:0032480 feature_id[525].value <= threshold=6.815735578536987
Class: positive genes

Rules_147 passed counts:6
node_0: feature_name=GO:0042113 feature_id[0].value <= threshold=13.408552169799805
node_1: feature_name=GO:0007568 feature_id[534].value > threshold=5.0313897132873535
node_913: feature_name=GO:0032763 feature_id[629].value > threshold=0.31753237545490265
node_1195: feature_name=GO:0071301 feature_id[442].value > threshold=0.11534593254327774
node_1375: feature_name=GO:0046500 feature_id[171].value <= threshold=1.130434513092041
node_1376: feature_name=GO:0001782 feature_id[240].value <= threshold=6.639636993408203
node_1377: feature_name=GO:0043371 feature_id[707].value <= threshold=2.562113642692566
node_1378: feature_name=GO:0030291 feature_id[147].value <= threshold=6.834916830062866
node_1379: feature_name=GO:0045656 feature_id[756].value > threshold=0.6281269192695618
node_1393: feature_name=GO:0046007 feature_id[152].value <= threshold=1.020785927772522
node_1394: feature_name=GO:0000302 feature_id[375].value <= threshold=20.847331047058105
node_1395: feature_name=hsa04110 feature_id[328].value <= threshold=43.297359466552734
node_1396: feature_name=GO:0050731 feature_id[804].value <= threshold=15.756641864776611
node_1397: feature_name=GO:0002440 feature_id[429].value <= threshold=0.4019751101732254
node_1398: feature_name=GO:0072610 feature_id[262].value > threshold=0.5024027079343796
node_1400: feature_name=GO:0048523 feature_id[293].value > threshold=20.737138748168945
Class: positive genes

Rules_148	passed counts:6
node_0: feature_name=GO:0042113	feature_id[0].value <= threshold=13.408552169799805
node_1: feature_name=GO:0007568	feature_id[534].value > threshold=5.0313897132873535
node_913: feature_name=GO:0032763	feature_id[629].value > threshold=0.31753237545490265
node_1195: feature_name=GO:0071301	feature_id[442].value <= threshold=0.11534593254327774
node_1196: feature_name=GO:0046500	feature_id[171].value <= threshold=0.6314916908740997
node_1197: feature_name=GO:0030852	feature_id[22].value <= threshold=2.9768868684768677
node_1198: feature_name=hsa04672	feature_id[276].value <= threshold=8.06681227684021
node_1199: feature_name=GO:0001836	feature_id[450].value > threshold=0.09961023926734924
node_1201: feature_name=GO:0070230	feature_id[64].value <= threshold=3.3956379890441895
node_1202: feature_name=GO:0070102	feature_id[49].value <= threshold=4.805386543273926
node_1203: feature_name=GO:0045553	feature_id[736].value <= threshold=1.4970332980155945
node_1204: feature_name=GO:0051311	feature_id[58].value <= threshold=3.301889181137085
node_1205: feature_name=GO:0030291	feature_id[147].value <= threshold=8.156315326690674
node_1206: feature_name=GO:0045637	feature_id[241].value > threshold=0.01834342861548066
node_1208: feature_name=GO:0002890	feature_id[98].value <= threshold=2.767483353614807
node_1209: feature_name=GO:0046632	feature_id[248].value <= threshold=11.663254261016846
node_1210: feature_name=GO:0008588	feature_id[26].value > threshold=1.391375720500946
node_1308: feature_name=GO:0002200	feature_id[82].value > threshold=1.0774684846401215
node_1310: feature_name=GO:0019814	feature_id[189].value > threshold=0.7463377118110657
Class: negative genes	

Rules_149	passed counts:6
node_0: feature_name=GO:0042113	feature_id[0].value <= threshold=13.408552169799805
node_1: feature_name=GO:0007568	feature_id[534].value > threshold=5.0313897132873535
node_913: feature_name=GO:0032763	feature_id[629].value > threshold=0.31753237545490265
node_1195: feature_name=GO:0071301	feature_id[442].value <= threshold=0.11534593254327774
node_1196: feature_name=GO:0046500	feature_id[171].value <= threshold=0.6314916908740997
node_1197: feature_name=GO:0030852	feature_id[22].value <= threshold=2.9768868684768677
node_1198: feature_name=hsa04672	feature_id[276].value <= threshold=8.06681227684021
node_1199: feature_name=GO:0001836	feature_id[450].value > threshold=0.09961023926734924
node_1201: feature_name=GO:0070230	feature_id[64].value <= threshold=3.3956379890441895
node_1202: feature_name=GO:0070102	feature_id[49].value <= threshold=4.805386543273926
node_1203: feature_name=GO:0045553	feature_id[736].value <= threshold=1.4970332980155945

node_1204: feature_name=GO:0051311 feature_id[58].value <= threshold=3.301889181137085
node_1205: feature_name=GO:0030291 feature_id[147].value <= threshold=8.156315326690674
node_1206: feature_name=GO:0045637 feature_id[241].value > threshold=0.01834342861548066
node_1208: feature_name=GO:0002890 feature_id[98].value <= threshold=2.767483353614807
node_1209: feature_name=GO:0046632 feature_id[248].value <= threshold=11.663254261016846
node_1210: feature_name=GO:0008588 feature_id[26].value <= threshold=1.391375720500946
node_1211: feature_name=GO:0010639 feature_id[564].value <= threshold=18.975126266479492
node_1212: feature_name=GO:0046685 feature_id[782].value <= threshold=5.153738260269165
node_1213: feature_name=GO:0038116 feature_id[668].value <= threshold=1.7859655618667603
node_1214: feature_name=GO:0046449 feature_id[763].value <= threshold=5.603115558624268
node_1215: feature_name=GO:0048539 feature_id[319].value > threshold=0.5478232800960541
node_1269: feature_name=GO:0072341 feature_id[144].value <= threshold=1.3205850720405579
node_1270: feature_name=GO:0010835 feature_id[560].value > threshold=0.7877185940742493
node_1282: feature_name=GO:0010835 feature_id[560].value > threshold=0.9053532779216766
Class: negative genes

Rules_150 passed counts:6
node_0: feature_name=GO:0042113 feature_id[0].value <= threshold=13.408552169799805
node_1: feature_name=GO:0007568 feature_id[534].value > threshold=5.0313897132873535
node_913: feature_name=GO:0032763 feature_id[629].value > threshold=0.31753237545490265
node_1195: feature_name=GO:0071301 feature_id[442].value <= threshold=0.11534593254327774
node_1196: feature_name=GO:0046500 feature_id[171].value <= threshold=0.6314916908740997
node_1197: feature_name=GO:0030852 feature_id[22].value <= threshold=2.9768868684768677
node_1198: feature_name=hsa04672 feature_id[276].value <= threshold=8.06681227684021
node_1199: feature_name=GO:0001836 feature_id[450].value > threshold=0.09961023926734924
node_1201: feature_name=GO:0070230 feature_id[64].value <= threshold=3.3956379890441895
node_1202: feature_name=GO:0070102 feature_id[49].value <= threshold=4.805386543273926
node_1203: feature_name=GO:0045553 feature_id[736].value <= threshold=1.4970332980155945
node_1204: feature_name=GO:0051311 feature_id[58].value <= threshold=3.301889181137085
node_1205: feature_name=GO:0030291 feature_id[147].value <= threshold=8.156315326690674
node_1206: feature_name=GO:0045637 feature_id[241].value > threshold=0.01834342861548066
node_1208: feature_name=GO:0002890 feature_id[98].value <= threshold=2.767483353614807
node_1209: feature_name=GO:0046632 feature_id[248].value <= threshold=11.663254261016846
node_1210: feature_name=GO:0008588 feature_id[26].value <= threshold=1.391375720500946
node_1211: feature_name=GO:0010639 feature_id[564].value <= threshold=18.975126266479492

node_1212: feature_name=GO:0046685 feature_id[782].value <= threshold=5.153738260269165
node_1213: feature_name=GO:0038116 feature_id[668].value <= threshold=1.7859655618667603
node_1214: feature_name=GO:0046449 feature_id[763].value <= threshold=5.603115558624268
node_1215: feature_name=GO:0048539 feature_id[319].value > threshold=0.5478232800960541
node_1269: feature_name=GO:0072341 feature_id[144].value <= threshold=1.3205850720405579
node_1270: feature_name=GO:0010835 feature_id[560].value > threshold=0.7877185940742493
node_1282: feature_name=GO:0010835 feature_id[560].value <= threshold=0.9053532779216766
node_1283: feature_name=GO:0070741 feature_id[538].value <= threshold=4.904514789581299
Class: positive genes

Rules_151 passed counts:6
node_0: feature_name=GO:0042113 feature_id[0].value <= threshold=13.408552169799805
node_1: feature_name=GO:0007568 feature_id[534].value > threshold=5.0313897132873535
node_913: feature_name=GO:0032763 feature_id[629].value <= threshold=0.31753237545490265
node_914: feature_name=GO:0097193 feature_id[167].value <= threshold=28.171168327331543
node_915: feature_name=GO:0002903 feature_id[10].value > threshold=1.0534588098526
node_1159: feature_name=GO:0070198 feature_id[665].value > threshold=0.16219981759786606
node_1165: feature_name=GO:0042287 feature_id[20].value > threshold=0.915093183517456
node_1181: feature_name=GO:0060576 feature_id[204].value <= threshold=0.3862239122390747
node_1182: feature_name=GO:0042509 feature_id[694].value > threshold=5.3463897705078125
node_1184: feature_name=GO:0044424 feature_id[1].value > threshold=0.29986531287431717
Class: positive genes

Rules_152 passed counts:6
node_0: feature_name=GO:0042113 feature_id[0].value <= threshold=13.408552169799805
node_1: feature_name=GO:0007568 feature_id[534].value > threshold=5.0313897132873535
node_913: feature_name=GO:0032763 feature_id[629].value <= threshold=0.31753237545490265
node_914: feature_name=GO:0097193 feature_id[167].value <= threshold=28.171168327331543
node_915: feature_name=GO:0002903 feature_id[10].value <= threshold=1.0534588098526
node_916: feature_name=GO:1904029 feature_id[208].value <= threshold=23.852136611938477
node_917: feature_name=GO:0050897 feature_id[164].value > threshold=0.6870408356189728
node_1095: feature_name=GO:0072539 feature_id[68].value > threshold=2.5667877197265625
node_1153: feature_name=GO:0002708 feature_id[526].value <= threshold=6.108316421508789
Class: positive genes

Rules_153	passed counts:6
node_0: feature_name=GO:0042113	feature_id[0].value <= threshold=13.408552169799805
node_1: feature_name=GO:0007568	feature_id[534].value > threshold=5.0313897132873535
node_913: feature_name=GO:0032763	feature_id[629].value <= threshold=0.31753237545490265
node_914: feature_name=GO:0097193	feature_id[167].value <= threshold=28.171168327331543
node_915: feature_name=GO:0002903	feature_id[10].value <= threshold=1.0534588098526
node_916: feature_name=GO:1904029	feature_id[208].value <= threshold=23.852136611938477
node_917: feature_name=GO:0050897	feature_id[164].value > threshold=0.6870408356189728
node_1095: feature_name=GO:0072539	feature_id[68].value <= threshold=2.5667877197265625
node_1096: feature_name=GO:0046500	feature_id[171].value <= threshold=3.195197820663452
node_1097: feature_name=GO:0045861	feature_id[761].value > threshold=0.22614753991365433
node_1099: feature_name=GO:0043372	feature_id[242].value <= threshold=4.892506122589111
node_1100: feature_name=GO:0042162	feature_id[355].value <= threshold=11.339290618896484
node_1101: feature_name=hsa04660	feature_id[472].value <= threshold=24.27848720550537
node_1102: feature_name=GO:0007049	feature_id[259].value <= threshold=209.23831176757812
node_1103: feature_name=GO:0050897	feature_id[164].value > threshold=0.6919849812984467
node_1105: feature_name=GO:0045945	feature_id[267].value > threshold=3.307852625846863
node_1137: feature_name=GO:0072710	feature_id[251].value > threshold=0.4050363004207611
node_1139: feature_name=GO:0001666	feature_id[682].value > threshold=8.14579439163208
Class: positive genes	

Rules_154	passed counts:6
node_0: feature_name=GO:0042113	feature_id[0].value <= threshold=13.408552169799805
node_1: feature_name=GO:0007568	feature_id[534].value > threshold=5.0313897132873535
node_913: feature_name=GO:0032763	feature_id[629].value <= threshold=0.31753237545490265
node_914: feature_name=GO:0097193	feature_id[167].value <= threshold=28.171168327331543
node_915: feature_name=GO:0002903	feature_id[10].value <= threshold=1.0534588098526
node_916: feature_name=GO:1904029	feature_id[208].value <= threshold=23.852136611938477
node_917: feature_name=GO:0050897	feature_id[164].value <= threshold=0.6870408356189728
node_918: feature_name=GO:0006139	feature_id[474].value > threshold=1.3052097624921544e-07
node_920: feature_name=GO:0002821	feature_id[588].value <= threshold=13.81072187423706
node_921: feature_name=GO:0006298	feature_id[621].value <= threshold=24.078600883483887
node_922: feature_name=GO:0003908	feature_id[11].value <= threshold=1.7776933312416077
node_923: feature_name=GO:0030098	feature_id[273].value > threshold=0.0079949083738029
node_927: feature_name=GO:0006808	feature_id[513].value <= threshold=4.34592080116272

node_928: feature_name=GO:0071887 feature_id[283].value <= threshold=10.353787899017334
node_929: feature_name=GO:0038001 feature_id[28].value <= threshold=3.9469382762908936
node_930: feature_name=GO:0042287 feature_id[20].value <= threshold=3.6209195852279663
node_931: feature_name=GO:0003968 feature_id[47].value <= threshold=2.004227638244629
node_932: feature_name=GO:0002698 feature_id[395].value <= threshold=18.51447582244873
node_933: feature_name=GO:0044710 feature_id[719].value <= threshold=179.7340316772461
node_934: feature_name=GO:0007568 feature_id[534].value > threshold=5.035318374633789
node_936: feature_name=GO:0006216 feature_id[504].value <= threshold=2.2605666518211365
node_937: feature_name=GO:0048569 feature_id[793].value <= threshold=5.340231895446777
node_938: feature_name=GO:0001777 feature_id[380].value <= threshold=4.100832939147949
node_939: feature_name=GO:0007600 feature_id[122].value <= threshold=171.13383102416992
node_940: feature_name=GO:0001779 feature_id[378].value <= threshold=6.234851121902466
node_941: feature_name=GO:0030291 feature_id[147].value <= threshold=10.8051118850708
node_942: feature_name=GO:0048534 feature_id[790].value > threshold=0.017984486185014248
node_944: feature_name=GO:0070245 feature_id[326].value <= threshold=3.1282339096069336
node_945: feature_name=GO:0009086 feature_id[547].value <= threshold=0.2896959036588669
node_946: feature_name=GO:0001889 feature_id[387].value <= threshold=17.643128395080566
node_947: feature_name=GO:0048144 feature_id[192].value > threshold=0.20887330174446106
node_951: feature_name=GO:0023026 feature_id[69].value <= threshold=3.403424024581909
node_952: feature_name=GO:0048147 feature_id[788].value <= threshold=4.188283443450928
node_953: feature_name=GO:0090116 feature_id[97].value <= threshold=4.617154359817505
node_954: feature_name=GO:0002863 feature_id[490].value <= threshold=4.8968470096588135
node_955: feature_name=GO:0002524 feature_id[72].value <= threshold=3.035340189933777
node_956: feature_name=GO:0042130 feature_id[16].value <= threshold=7.285413980484009
node_957: feature_name=GO:0071456 feature_id[500].value <= threshold=21.212870597839355
node_958: feature_name=GO:2001238 feature_id[27].value <= threshold=7.849715709686279
node_959: feature_name=GO:0023030 feature_id[45].value <= threshold=1.7420591711997986
node_960: feature_name=GO:0072593 feature_id[381].value <= threshold=22.993029594421387
node_961: feature_name=GO:0034101 feature_id[646].value <= threshold=13.430044651031494
node_962: feature_name=GO:0097153 feature_id[226].value <= threshold=10.147814273834229
node_963: feature_name=GO:0001836 feature_id[450].value <= threshold=7.252067804336548
node_964: feature_name=GO:0032504 feature_id[244].value > threshold=0.15777301788330078
node_968: feature_name=GO:0043379 feature_id[714].value > threshold=1.5959861278533936
node_974: feature_name=GO:0002705 feature_id[541].value > threshold=3.148389160633087
Class: negative genes

Rules_155	passed counts:6
node_0: feature_name=GO:0042113	feature_id[0].value <= threshold=13.408552169799805
node_1: feature_name=GO:0007568	feature_id[534].value > threshold=5.0313897132873535
node_913: feature_name=GO:0032763	feature_id[629].value <= threshold=0.31753237545490265
node_914: feature_name=GO:0097193	feature_id[167].value <= threshold=28.171168327331543
node_915: feature_name=GO:0002903	feature_id[10].value <= threshold=1.0534588098526
node_916: feature_name=GO:1904029	feature_id[208].value <= threshold=23.852136611938477
node_917: feature_name=GO:0050897	feature_id[164].value <= threshold=0.6870408356189728
node_918: feature_name=GO:0006139	feature_id[474].value > threshold=1.3052097624921544e-07
node_920: feature_name=GO:0002821	feature_id[588].value <= threshold=13.81072187423706
node_921: feature_name=GO:0006298	feature_id[621].value <= threshold=24.078600883483887
node_922: feature_name=GO:0003908	feature_id[11].value <= threshold=1.7776933312416077
node_923: feature_name=GO:0030098	feature_id[273].value > threshold=0.0079949083738029
node_927: feature_name=GO:0006808	feature_id[513].value <= threshold=4.34592080116272
node_928: feature_name=GO:0071887	feature_id[283].value <= threshold=10.353787899017334
node_929: feature_name=GO:0038001	feature_id[28].value <= threshold=3.9469382762908936
node_930: feature_name=GO:0042287	feature_id[20].value <= threshold=3.6209195852279663
node_931: feature_name=GO:0003968	feature_id[47].value <= threshold=2.004227638244629
node_932: feature_name=GO:0002698	feature_id[395].value <= threshold=18.51447582244873
node_933: feature_name=GO:0044710	feature_id[719].value <= threshold=179.7340316772461
node_934: feature_name=GO:0007568	feature_id[534].value > threshold=5.035318374633789
node_936: feature_name=GO:0006216	feature_id[504].value <= threshold=2.2605666518211365
node_937: feature_name=GO:0048569	feature_id[793].value <= threshold=5.340231895446777
node_938: feature_name=GO:0001777	feature_id[380].value <= threshold=4.100832939147949
node_939: feature_name=GO:0007600	feature_id[122].value <= threshold=171.13383102416992
node_940: feature_name=GO:0001779	feature_id[378].value <= threshold=6.234851121902466
node_941: feature_name=GO:0030291	feature_id[147].value <= threshold=10.8051118850708
node_942: feature_name=GO:0048534	feature_id[790].value > threshold=0.017984486185014248
node_944: feature_name=GO:0070245	feature_id[326].value <= threshold=3.1282339096069336
node_945: feature_name=GO:0009086	feature_id[547].value <= threshold=0.2896959036588669
node_946: feature_name=GO:0001889	feature_id[387].value <= threshold=17.643128395080566
node_947: feature_name=GO:0048144	feature_id[192].value > threshold=0.20887330174446106
node_951: feature_name=GO:0023026	feature_id[69].value <= threshold=3.403424024581909
node_952: feature_name=GO:0048147	feature_id[788].value <= threshold=4.188283443450928

node_953: feature_name=GO:0090116 feature_id[97].value <= threshold=4.617154359817505
node_954: feature_name=GO:0002863 feature_id[490].value <= threshold=4.8968470096588135
node_955: feature_name=GO:0002524 feature_id[72].value <= threshold=3.035340189933777
node_956: feature_name=GO:0042130 feature_id[16].value <= threshold=7.285413980484009
node_957: feature_name=GO:0071456 feature_id[500].value <= threshold=21.212870597839355
node_958: feature_name=GO:2001238 feature_id[27].value <= threshold=7.849715709686279
node_959: feature_name=GO:0023030 feature_id[45].value <= threshold=1.7420591711997986
node_960: feature_name=GO:0072593 feature_id[381].value <= threshold=22.993029594421387
node_961: feature_name=GO:0034101 feature_id[646].value <= threshold=13.430044651031494
node_962: feature_name=GO:0097153 feature_id[226].value <= threshold=10.147814273834229
node_963: feature_name=GO:0001836 feature_id[450].value <= threshold=7.252067804336548
node_964: feature_name=GO:0032504 feature_id[244].value <= threshold=0.15777301788330078
node_965: feature_name=GO:0023026 feature_id[69].value <= threshold=1.5684430599212646
Class: negative genes

Rules_156 passed counts:6
node_0: feature_name=GO:0042113 feature_id[0].value <= threshold=13.408552169799805
node_1: feature_name=GO:0007568 feature_id[534].value <= threshold=5.0313897132873535
node_2: feature_name=GO:0002705 feature_id[541].value > threshold=3.200145721435547
node_870: feature_name=GO:0046634 feature_id[46].value <= threshold=8.9394211769104
node_871: feature_name=GO:0048523 feature_id[293].value <= threshold=19.963229179382324
node_872: feature_name=GO:2001237 feature_id[796].value > threshold=0.09761488810181618
node_880: feature_name=GO:0006808 feature_id[513].value <= threshold=1.7969900965690613
node_881: feature_name=GO:0032479 feature_id[441].value > threshold=0.13597124069929123
node_887: feature_name=GO:0043226 feature_id[138].value > threshold=12.855401992797852
node_903: feature_name=GO:0043231 feature_id[264].value > threshold=20.170435905456543
Class: negative genes

Rules_157 passed counts:6
node_0: feature_name=GO:0042113 feature_id[0].value <= threshold=13.408552169799805
node_1: feature_name=GO:0007568 feature_id[534].value <= threshold=5.0313897132873535
node_2: feature_name=GO:0002705 feature_id[541].value > threshold=3.200145721435547
node_870: feature_name=GO:0046634 feature_id[46].value <= threshold=8.9394211769104
node_871: feature_name=GO:0048523 feature_id[293].value <= threshold=19.963229179382324
node_872: feature_name=GO:2001237 feature_id[796].value > threshold=0.09761488810181618

node_880: feature_name=GO:0006808 feature_id[513].value <= threshold=1.7969900965690613
node_881: feature_name=GO:0032479 feature_id[441].value > threshold=0.13597124069929123
node_887: feature_name=GO:0043226 feature_id[138].value > threshold=12.855401992797852
node_903: feature_name=GO:0043231 feature_id[264].value <= threshold=20.170435905456543
node_904: feature_name=GO:0032480 feature_id[525].value > threshold=0.41479843854904175
Class: positive genes

Rules_158 passed counts:6
node_0: feature_name=GO:0042113 feature_id[0].value <= threshold=13.408552169799805
node_1: feature_name=GO:0007568 feature_id[534].value <= threshold=5.0313897132873535
node_2: feature_name=GO:0002705 feature_id[541].value <= threshold=3.200145721435547
node_3: feature_name=GO:1901525 feature_id[576].value > threshold=0.4466460347175598
node_617: feature_name=GO:0005622 feature_id[233].value > threshold=90.85730743408203
node_847: feature_name=GO:0002639 feature_id[466].value <= threshold=0.32853202521800995
node_848: feature_name=GO:0002636 feature_id[447].value > threshold=0.3303467631340027
node_864: feature_name=GO:0002200 feature_id[82].value > threshold=0.11118704453110695
Class: positive genes

Rules_159 passed counts:6
node_0: feature_name=GO:0042113 feature_id[0].value <= threshold=13.408552169799805
node_1: feature_name=GO:0007568 feature_id[534].value <= threshold=5.0313897132873535
node_2: feature_name=GO:0002705 feature_id[541].value <= threshold=3.200145721435547
node_3: feature_name=GO:1901525 feature_id[576].value > threshold=0.4466460347175598
node_617: feature_name=GO:0005622 feature_id[233].value <= threshold=90.85730743408203
node_618: feature_name=GO:1903147 feature_id[200].value <= threshold=7.326428413391113
node_619: feature_name=GO:0003964 feature_id[317].value <= threshold=5.575642108917236
node_620: feature_name=GO:0046498 feature_id[203].value <= threshold=6.620223522186279
node_621: feature_name=GO:0002562 feature_id[25].value <= threshold=10.287704467773438
node_622: feature_name=GO:0006919 feature_id[246].value > threshold=5.801334381103516
node_834: feature_name=GO:0045931 feature_id[266].value > threshold=0.973282665014267
node_836: feature_name=GO:0030889 feature_id[602].value > threshold=0.19142773747444153
Class: positive genes

Rules_160 passed counts:6
node_0: feature_name=GO:0042113 feature_id[0].value <= threshold=13.408552169799805

node_1: feature_name=GO:0007568 feature_id[534].value <= threshold=5.0313897132873535
node_2: feature_name=GO:0002705 feature_id[541].value <= threshold=3.200145721435547
node_3: feature_name=GO:1901525 feature_id[576].value <= threshold=0.4466460347175598
node_4: feature_name=GO:0048539 feature_id[319].value <= threshold=3.0399646759033203
node_5: feature_name=GO:0001910 feature_id[385].value <= threshold=3.7437864542007446
node_6: feature_name=GO:0043200 feature_id[706].value <= threshold=9.307284355163574
node_7: feature_name=GO:0001773 feature_id[308].value <= threshold=4.016931533813477
node_8: feature_name=GO:0090116 feature_id[97].value <= threshold=7.99645471572876
node_9: feature_name=GO:0019814 feature_id[189].value <= threshold=4.525782108306885
node_10: feature_name=GO:1902583 feature_id[215].value <= threshold=17.60310649871826
node_11: feature_name=GO:0045429 feature_id[737].value > threshold=1.6133361458778381
node_477: feature_name=GO:0036037 feature_id[445].value <= threshold=1.7181594371795654
node_478: feature_name=GO:0006244 feature_id[503].value > threshold=0.951388418674469
node_588: feature_name=GO:0010556 feature_id[784].value > threshold=0.00211568595841527
node_590: feature_name=GO:0016363 feature_id[90].value > threshold=1.3301835656166077
node_592: feature_name=GO:0033209 feature_id[281].value > threshold=0.47055448591709137
Class: negative genes

Rules_161 passed counts:6
node_0: feature_name=GO:0042113 feature_id[0].value <= threshold=13.408552169799805
node_1: feature_name=GO:0007568 feature_id[534].value <= threshold=5.0313897132873535
node_2: feature_name=GO:0002705 feature_id[541].value <= threshold=3.200145721435547
node_3: feature_name=GO:1901525 feature_id[576].value <= threshold=0.4466460347175598
node_4: feature_name=GO:0048539 feature_id[319].value <= threshold=3.0399646759033203
node_5: feature_name=GO:0001910 feature_id[385].value <= threshold=3.7437864542007446
node_6: feature_name=GO:0043200 feature_id[706].value <= threshold=9.307284355163574
node_7: feature_name=GO:0001773 feature_id[308].value <= threshold=4.016931533813477
node_8: feature_name=GO:0090116 feature_id[97].value <= threshold=7.99645471572876
node_9: feature_name=GO:0019814 feature_id[189].value <= threshold=4.525782108306885
node_10: feature_name=GO:1902583 feature_id[215].value <= threshold=17.60310649871826
node_11: feature_name=GO:0045429 feature_id[737].value > threshold=1.6133361458778381
node_477: feature_name=GO:0036037 feature_id[445].value <= threshold=1.7181594371795654
node_478: feature_name=GO:0006244 feature_id[503].value > threshold=0.951388418674469
node_588: feature_name=GO:0010556 feature_id[784].value > threshold=0.00211568595841527
node_590: feature_name=GO:0016363 feature_id[90].value > threshold=1.3301835656166077

node_592: feature_name=GO:0033209 feature_id[281].value <= threshold=0.47055448591709137
Class: positive genes

Rules_162 passed counts:6
node_0: feature_name=GO:0042113 feature_id[0].value <= threshold=13.408552169799805
node_1: feature_name=GO:0007568 feature_id[534].value <= threshold=5.0313897132873535
node_2: feature_name=GO:0002705 feature_id[541].value <= threshold=3.200145721435547
node_3: feature_name=GO:1901525 feature_id[576].value <= threshold=0.4466460347175598
node_4: feature_name=GO:0048539 feature_id[319].value <= threshold=3.0399646759033203
node_5: feature_name=GO:0001910 feature_id[385].value <= threshold=3.7437864542007446
node_6: feature_name=GO:0043200 feature_id[706].value <= threshold=9.307284355163574
node_7: feature_name=GO:0001773 feature_id[308].value <= threshold=4.016931533813477
node_8: feature_name=GO:0090116 feature_id[97].value <= threshold=7.99645471572876
node_9: feature_name=GO:0019814 feature_id[189].value <= threshold=4.525782108306885
node_10: feature_name=GO:1902583 feature_id[215].value <= threshold=17.60310649871826
node_11: feature_name=GO:0045429 feature_id[737].value <= threshold=1.6133361458778381
node_12: feature_name=GO:0003720 feature_id[228].value <= threshold=5.519326210021973
node_13: feature_name=GO:0046006 feature_id[364].value <= threshold=4.9985432624816895
node_14: feature_name=GO:0070424 feature_id[254].value <= threshold=6.233297109603882
node_15: feature_name=GO:0009892 feature_id[320].value <= threshold=59.99180793762207
node_16: feature_name=GO:0007064 feature_id[527].value <= threshold=11.57377815246582
node_17: feature_name=GO:0005575 feature_id[17].value <= threshold=15.417460918426514
node_18: feature_name=GO:0043368 feature_id[31].value <= threshold=4.059496641159058
node_19: feature_name=GO:0005164 feature_id[163].value <= threshold=5.793607711791992
node_20: feature_name=GO:0042130 feature_id[16].value <= threshold=5.073179721832275
node_21: feature_name=GO:0010216 feature_id[282].value <= threshold=4.834584474563599
node_22: feature_name=GO:0009628 feature_id[553].value <= threshold=29.314892768859863
node_23: feature_name=GO:0045628 feature_id[749].value <= threshold=3.1001367568969727
node_24: feature_name=GO:0042288 feature_id[100].value <= threshold=5.174932956695557
node_25: feature_name=GO:0002329 feature_id[406].value <= threshold=2.348930835723877
node_26: feature_name=GO:0051246 feature_id[642].value > threshold=0.6498909294605255
node_194: feature_name=GO:0009164 feature_id[546].value <= threshold=17.11970329284668
node_195: feature_name=GO:0001909 feature_id[386].value <= threshold=7.292566299438477
node_196: feature_name=GO:0003908 feature_id[11].value <= threshold=1.887226164340973
node_197: feature_name=GO:0009164 feature_id[546].value <= threshold=0.26642198860645294

node_198: feature_name=GO:2001242 feature_id[24].value <= threshold=9.207026481628418
node_199: feature_name=GO:0051246 feature_id[642].value > threshold=0.6504445374011993
node_203: feature_name=GO:0032461 feature_id[608].value <= threshold=2.607542634010315
node_204: feature_name=GO:0007584 feature_id[529].value <= threshold=16.099515914916992
node_205: feature_name=hsa05221 feature_id[349].value <= threshold=9.359850406646729
node_206: feature_name=GO:0002636 feature_id[447].value <= threshold=1.615447759628296
node_207: feature_name=GO:0033993 feature_id[421].value > threshold=2.96540611088858e-05
node_217: feature_name=GO:0002331 feature_id[404].value <= threshold=2.1883513927459717
node_218: feature_name=GO:0046498 feature_id[203].value <= threshold=4.085246205329895
node_219: feature_name=GO:0002832 feature_id[491].value <= threshold=3.0228612422943115
node_220: feature_name=GO:1904868 feature_id[306].value <= threshold=3.8674756288528442
node_221: feature_name=GO:0048545 feature_id[360].value <= threshold=27.38136100769043
node_222: feature_name=GO:0071310 feature_id[760].value <= threshold=2.3522024154663086
node_223: feature_name=GO:0001775 feature_id[505].value <= threshold=9.71301555633545
node_224: feature_name=GO:0071310 feature_id[760].value <= threshold=2.346743583679199
node_225: feature_name=GO:0001772 feature_id[91].value <= threshold=3.252573609352112
node_226: feature_name=GO:0036498 feature_id[359].value <= threshold=12.092026710510254
node_227: feature_name=hsa05340 feature_id[351].value <= threshold=0.7677814364433289
node_228: feature_name=GO:0032703 feature_id[617].value <= threshold=1.7450646758079529
node_229: feature_name=hsa04640 feature_id[79].value <= threshold=2.118402123451233
node_230: feature_name=GO:0042097 feature_id[675].value <= threshold=1.8110727667808533
node_231: feature_name=GO:0071391 feature_id[518].value <= threshold=2.691588521003723
node_232: feature_name=GO:0001666 feature_id[682].value <= threshold=5.225740432739258
node_233: feature_name=GO:0048539 feature_id[319].value <= threshold=1.8523842096328735
node_234: feature_name=GO:0038061 feature_id[671].value <= threshold=8.564527988433838
node_235: feature_name=GO:0002484 feature_id[600].value > threshold=3.1121134757995605
node_257: feature_name=GO:0045624 feature_id[750].value <= threshold=0.2939649187028408
Class: negative genes

Rules_163 passed counts:6
node_0: feature_name=GO:0042113 feature_id[0].value <= threshold=13.408552169799805
node_1: feature_name=GO:0007568 feature_id[534].value <= threshold=5.0313897132873535
node_2: feature_name=GO:0002705 feature_id[541].value <= threshold=3.200145721435547
node_3: feature_name=GO:1901525 feature_id[576].value <= threshold=0.4466460347175598
node_4: feature_name=GO:0048539 feature_id[319].value <= threshold=3.0399646759033203

node_5: feature_name=GO:0001910	feature_id[385].value <= threshold=3.7437864542007446
node_6: feature_name=GO:0043200	feature_id[706].value <= threshold=9.307284355163574
node_7: feature_name=GO:0001773	feature_id[308].value <= threshold=4.016931533813477
node_8: feature_name=GO:0090116	feature_id[97].value <= threshold=7.99645471572876
node_9: feature_name=GO:0019814	feature_id[189].value <= threshold=4.525782108306885
node_10: feature_name=GO:1902583	feature_id[215].value <= threshold=17.60310649871826
node_11: feature_name=GO:0045429	feature_id[737].value <= threshold=1.6133361458778381
node_12: feature_name=GO:0003720	feature_id[228].value <= threshold=5.519326210021973
node_13: feature_name=GO:0046006	feature_id[364].value <= threshold=4.9985432624816895
node_14: feature_name=GO:0070424	feature_id[254].value <= threshold=6.233297109603882
node_15: feature_name=GO:0009892	feature_id[320].value <= threshold=59.99180793762207
node_16: feature_name=GO:0007064	feature_id[527].value <= threshold=11.57377815246582
node_17: feature_name=GO:0005575	feature_id[17].value <= threshold=15.417460918426514
node_18: feature_name=GO:0043368	feature_id[31].value <= threshold=4.059496641159058
node_19: feature_name=GO:0005164	feature_id[163].value <= threshold=5.793607711791992
node_20: feature_name=GO:0042130	feature_id[16].value <= threshold=5.073179721832275
node_21: feature_name=GO:0010216	feature_id[282].value <= threshold=4.834584474563599
node_22: feature_name=GO:0009628	feature_id[553].value <= threshold=29.314892768859863
node_23: feature_name=GO:0045628	feature_id[749].value <= threshold=3.1001367568969727
node_24: feature_name=GO:0042288	feature_id[100].value <= threshold=5.174932956695557
node_25: feature_name=GO:0002329	feature_id[406].value <= threshold=2.348930835723877
node_26: feature_name=GO:0051246	feature_id[642].value <= threshold=0.6498909294605255
node_27: feature_name=GO:0042162	feature_id[355].value <= threshold=8.05103588104248
node_28: feature_name=hsa04668	feature_id[333].value <= threshold=5.614926338195801
node_29: feature_name=GO:0032200	feature_id[613].value <= threshold=28.735913276672363
node_30: feature_name=hsa04662	feature_id[334].value <= threshold=9.588344097137451
node_31: feature_name=GO:1901992	feature_id[105].value <= threshold=5.291427850723267
node_32: feature_name=GO:0072341	feature_id[144].value <= threshold=6.01465106010437
node_33: feature_name=GO:0047485	feature_id[121].value <= threshold=6.657836437225342
node_34: feature_name=GO:0090594	feature_id[738].value <= threshold=1.8673912286758423
node_35: feature_name=GO:0043525	feature_id[524].value <= threshold=2.881397008895874
node_36: feature_name=GO:0032504	feature_id[244].value <= threshold=17.22207546234131
node_37: feature_name=GO:0051348	feature_id[126].value <= threshold=5.933559417724609
node_38: feature_name=GO:0042522	feature_id[692].value <= threshold=1.78694087266922
node_39: feature_name=GO:0031100	feature_id[73].value <= threshold=3.2312101125717163

node_40: feature_name=GO:0042493 feature_id[149].value <= threshold=19.771170616149902
node_41: feature_name=GO:0001552 feature_id[366].value <= threshold=2.1711617708206177
node_42: feature_name=GO:0046483 feature_id[391].value <= threshold=320.9667053222656
node_43: feature_name=GO:0002439 feature_id[431].value <= threshold=1.832722783088684
node_44: feature_name=GO:0006555 feature_id[514].value <= threshold=5.321640968322754
node_45: feature_name=GO:0070424 feature_id[254].value > threshold=3.321003556251526
node_139: feature_name=GO:0042542 feature_id[689].value <= threshold=2.582065224647522
Class: negative genes

Rules_164 passed counts:5
node_0: feature_name=GO:0042113 feature_id[0].value > threshold=13.408552169799805
node_1454: feature_name=GO:0050851 feature_id[29].value > threshold=9.650307655334473
node_1534: feature_name=GO:1902166 feature_id[301].value > threshold=0.303210511803627
node_1548: feature_name=GO:0035872 feature_id[659].value <= threshold=35.09038162231445
node_1549: feature_name=GO:0032069 feature_id[614].value > threshold=3.441983938217163
node_1571: feature_name=GO:0050864 feature_id[288].value > threshold=7.516624450683594
node_1575: feature_name=GO:2000772 feature_id[95].value > threshold=0.9979664981365204
node_1579: feature_name=GO:0043525 feature_id[524].value <= threshold=22.19614315032959
node_1580: feature_name=GO:0032479 feature_id[441].value <= threshold=4.490016937255859
node_1581: feature_name=GO:0046677 feature_id[591].value <= threshold=5.264658212661743
Class: positive genes

Rules_165 passed counts:5
node_0: feature_name=GO:0042113 feature_id[0].value > threshold=13.408552169799805
node_1454: feature_name=GO:0050851 feature_id[29].value <= threshold=9.650307655334473
node_1455: feature_name=GO:0006304 feature_id[510].value <= threshold=5.960662126541138
node_1456: feature_name=GO:0032673 feature_id[623].value <= threshold=4.092082738876343
node_1457: feature_name=GO:0002429 feature_id[748].value > threshold=4.626799821853638
node_1463: feature_name=GO:0015672 feature_id[573].value > threshold=0.08226438239216805
node_1475: feature_name=GO:0030217 feature_id[589].value > threshold=19.77315902709961
node_1497: feature_name=GO:0030291 feature_id[147].value > threshold=4.140083312988281
node_1507: feature_name=GO:0006213 feature_id[507].value > threshold=0.31206823140382767
Class: negative genes

Rules_166 passed counts:5

node_0: feature_name=GO:0042113 feature_id[0].value > threshold=13.408552169799805
node_1454: feature_name=GO:0050851 feature_id[29].value <= threshold=9.650307655334473
node_1455: feature_name=GO:0006304 feature_id[510].value <= threshold=5.960662126541138
node_1456: feature_name=GO:0032673 feature_id[623].value <= threshold=4.092082738876343
node_1457: feature_name=GO:0002429 feature_id[748].value > threshold=4.626799821853638
node_1463: feature_name=GO:0015672 feature_id[573].value > threshold=0.08226438239216805
node_1475: feature_name=GO:0030217 feature_id[589].value > threshold=19.77315902709961
node_1497: feature_name=GO:0030291 feature_id[147].value <= threshold=4.140083312988281
node_1498: feature_name=GO:0050798 feature_id[170].value > threshold=12.055219173431396
node_1504: feature_name=GO:1902166 feature_id[301].value > threshold=0.7733870446681976
Class: positive genes

Rules_167 passed counts:5
node_0: feature_name=GO:0042113 feature_id[0].value > threshold=13.408552169799805
node_1454: feature_name=GO:0050851 feature_id[29].value <= threshold=9.650307655334473
node_1455: feature_name=GO:0006304 feature_id[510].value <= threshold=5.960662126541138
node_1456: feature_name=GO:0032673 feature_id[623].value <= threshold=4.092082738876343
node_1457: feature_name=GO:0002429 feature_id[748].value > threshold=4.626799821853638
node_1463: feature_name=GO:0015672 feature_id[573].value > threshold=0.08226438239216805
node_1475: feature_name=GO:0030217 feature_id[589].value > threshold=19.77315902709961
node_1497: feature_name=GO:0030291 feature_id[147].value <= threshold=4.140083312988281
node_1498: feature_name=GO:0050798 feature_id[170].value > threshold=12.055219173431396
node_1504: feature_name=GO:1902166 feature_id[301].value <= threshold=0.7733870446681976
Class: negative genes

Rules_168 passed counts:5
node_0: feature_name=GO:0042113 feature_id[0].value <= threshold=13.408552169799805
node_1: feature_name=GO:0007568 feature_id[534].value > threshold=5.0313897132873535
node_913: feature_name=GO:0032763 feature_id[629].value > threshold=0.31753237545490265
node_1195: feature_name=GO:0071301 feature_id[442].value > threshold=0.11534593254327774
node_1375: feature_name=GO:0046500 feature_id[171].value <= threshold=1.130434513092041
node_1376: feature_name=GO:0001782 feature_id[240].value <= threshold=6.639636993408203
node_1377: feature_name=GO:0043371 feature_id[707].value <= threshold=2.562113642692566
node_1378: feature_name=GO:0030291 feature_id[147].value <= threshold=6.834916830062866
node_1379: feature_name=GO:0045656 feature_id[756].value > threshold=0.6281269192695618

node_1393: feature_name=GO:0046007 feature_id[152].value <= threshold=1.020785927772522
node_1394: feature_name=GO:0000302 feature_id[375].value > threshold=20.847331047058105
node_1416: feature_name=GO:0030225 feature_id[587].value <= threshold=6.448119878768921
Class: positive genes

Rules_169 passed counts:5
node_0: feature_name=GO:0042113 feature_id[0].value <= threshold=13.408552169799805
node_1: feature_name=GO:0007568 feature_id[534].value > threshold=5.0313897132873535
node_913: feature_name=GO:0032763 feature_id[629].value > threshold=0.31753237545490265
node_1195: feature_name=GO:0071301 feature_id[442].value > threshold=0.11534593254327774
node_1375: feature_name=GO:0046500 feature_id[171].value <= threshold=1.130434513092041
node_1376: feature_name=GO:0001782 feature_id[240].value <= threshold=6.639636993408203
node_1377: feature_name=GO:0043371 feature_id[707].value <= threshold=2.562113642692566
node_1378: feature_name=GO:0030291 feature_id[147].value <= threshold=6.834916830062866
node_1379: feature_name=GO:0045656 feature_id[756].value > threshold=0.6281269192695618
node_1393: feature_name=GO:0046007 feature_id[152].value <= threshold=1.020785927772522
node_1394: feature_name=GO:0000302 feature_id[375].value <= threshold=20.847331047058105
node_1395: feature_name=hsa04110 feature_id[328].value <= threshold=43.297359466552734
node_1396: feature_name=GO:0050731 feature_id[804].value <= threshold=15.756641864776611
node_1397: feature_name=GO:0002440 feature_id[429].value <= threshold=0.4019751101732254
node_1398: feature_name=GO:0072610 feature_id[262].value <= threshold=0.5024027079343796
Class: negative genes

Rules_170 passed counts:5
node_0: feature_name=GO:0042113 feature_id[0].value <= threshold=13.408552169799805
node_1: feature_name=GO:0007568 feature_id[534].value > threshold=5.0313897132873535
node_913: feature_name=GO:0032763 feature_id[629].value > threshold=0.31753237545490265
node_1195: feature_name=GO:0071301 feature_id[442].value <= threshold=0.11534593254327774
node_1196: feature_name=GO:0046500 feature_id[171].value <= threshold=0.6314916908740997
node_1197: feature_name=GO:0030852 feature_id[22].value > threshold=2.9768868684768677
node_1329: feature_name=GO:0046668 feature_id[125].value > threshold=0.9442969560623169
node_1333: feature_name=GO:0006298 feature_id[621].value <= threshold=0.45032089948654175
node_1334: feature_name=GO:0046449 feature_id[763].value > threshold=0.2017408013343811
Class: negative genes

Rules_171 passed counts:5
node_0: feature_name=GO:0042113 feature_id[0].value <= threshold=13.408552169799805
node_1: feature_name=GO:0007568 feature_id[534].value > threshold=5.0313897132873535
node_913: feature_name=GO:0032763 feature_id[629].value > threshold=0.31753237545490265
node_1195: feature_name=GO:0071301 feature_id[442].value <= threshold=0.11534593254327774
node_1196: feature_name=GO:0046500 feature_id[171].value <= threshold=0.6314916908740997
node_1197: feature_name=GO:0030852 feature_id[22].value <= threshold=2.9768868684768677
node_1198: feature_name=hsa04672 feature_id[276].value <= threshold=8.06681227684021
node_1199: feature_name=GO:0001836 feature_id[450].value > threshold=0.09961023926734924
node_1201: feature_name=GO:0070230 feature_id[64].value > threshold=3.3956379890441895
node_1323: feature_name=GO:0032069 feature_id[614].value > threshold=1.899479329586029
Class: positive genes

Rules_172 passed counts:5
node_0: feature_name=GO:0042113 feature_id[0].value <= threshold=13.408552169799805
node_1: feature_name=GO:0007568 feature_id[534].value > threshold=5.0313897132873535
node_913: feature_name=GO:0032763 feature_id[629].value > threshold=0.31753237545490265
node_1195: feature_name=GO:0071301 feature_id[442].value <= threshold=0.11534593254327774
node_1196: feature_name=GO:0046500 feature_id[171].value <= threshold=0.6314916908740997
node_1197: feature_name=GO:0030852 feature_id[22].value <= threshold=2.9768868684768677
node_1198: feature_name=hsa04672 feature_id[276].value <= threshold=8.06681227684021
node_1199: feature_name=GO:0001836 feature_id[450].value > threshold=0.09961023926734924
node_1201: feature_name=GO:0070230 feature_id[64].value <= threshold=3.3956379890441895
node_1202: feature_name=GO:0070102 feature_id[49].value <= threshold=4.805386543273926
node_1203: feature_name=GO:0045553 feature_id[736].value <= threshold=1.4970332980155945
node_1204: feature_name=GO:0051311 feature_id[58].value <= threshold=3.301889181137085
node_1205: feature_name=GO:0030291 feature_id[147].value <= threshold=8.156315326690674
node_1206: feature_name=GO:0045637 feature_id[241].value > threshold=0.01834342861548066
node_1208: feature_name=GO:0002890 feature_id[98].value <= threshold=2.767483353614807
node_1209: feature_name=GO:0046632 feature_id[248].value <= threshold=11.663254261016846
node_1210: feature_name=GO:0008588 feature_id[26].value <= threshold=1.391375720500946
node_1211: feature_name=GO:0010639 feature_id[564].value <= threshold=18.975126266479492
node_1212: feature_name=GO:0046685 feature_id[782].value > threshold=5.153738260269165
node_1296: feature_name=GO:0019692 feature_id[584].value <= threshold=0.0685717724263668
node_1297: feature_name=GO:0034103 feature_id[318].value <= threshold=6.262972354888916

Class: positive genes

Rules_173 passed counts:5
node_0: feature_name=GO:0042113 feature_id[0].value <= threshold=13.408552169799805
node_1: feature_name=GO:0007568 feature_id[534].value > threshold=5.0313897132873535
node_913: feature_name=GO:0032763 feature_id[629].value > threshold=0.31753237545490265
node_1195: feature_name=GO:0071301 feature_id[442].value <= threshold=0.11534593254327774
node_1196: feature_name=GO:0046500 feature_id[171].value <= threshold=0.6314916908740997
node_1197: feature_name=GO:0030852 feature_id[22].value <= threshold=2.9768868684768677
node_1198: feature_name=hsa04672 feature_id[276].value <= threshold=8.06681227684021
node_1199: feature_name=GO:0001836 feature_id[450].value > threshold=0.09961023926734924
node_1201: feature_name=GO:0070230 feature_id[64].value <= threshold=3.3956379890441895
node_1202: feature_name=GO:0070102 feature_id[49].value <= threshold=4.805386543273926
node_1203: feature_name=GO:0045553 feature_id[736].value <= threshold=1.4970332980155945
node_1204: feature_name=GO:0051311 feature_id[58].value <= threshold=3.301889181137085
node_1205: feature_name=GO:0030291 feature_id[147].value <= threshold=8.156315326690674
node_1206: feature_name=GO:0045637 feature_id[241].value > threshold=0.01834342861548066
node_1208: feature_name=GO:0002890 feature_id[98].value <= threshold=2.767483353614807
node_1209: feature_name=GO:0046632 feature_id[248].value <= threshold=11.663254261016846
node_1210: feature_name=GO:0008588 feature_id[26].value <= threshold=1.391375720500946
node_1211: feature_name=GO:0010639 feature_id[564].value <= threshold=18.975126266479492
node_1212: feature_name=GO:0046685 feature_id[782].value <= threshold=5.153738260269165
node_1213: feature_name=GO:0038116 feature_id[668].value <= threshold=1.7859655618667603
node_1214: feature_name=GO:0046449 feature_id[763].value <= threshold=5.603115558624268
node_1215: feature_name=GO:0048539 feature_id[319].value <= threshold=0.5478232800960541
node_1216: feature_name=GO:0007089 feature_id[522].value <= threshold=0.9940084517002106
node_1217: feature_name=GO:0042287 feature_id[20].value <= threshold=2.693045496940613
node_1218: feature_name=GO:0044446 feature_id[184].value <= threshold=113.0758171081543
node_1219: feature_name=GO:0098602 feature_id[702].value <= threshold=68.95328903198242
node_1220: feature_name=GO:0035726 feature_id[270].value > threshold=1.7167096138000488
node_1258: feature_name=GO:0033689 feature_id[644].value > threshold=0.709069699048996
Class: negative genes

Rules_174 passed counts:5
node_0: feature_name=GO:0042113 feature_id[0].value <= threshold=13.408552169799805

node_1: feature_name=GO:0007568 feature_id[534].value > threshold=5.0313897132873535
node_913: feature_name=GO:0032763 feature_id[629].value > threshold=0.31753237545490265
node_1195: feature_name=GO:0071301 feature_id[442].value <= threshold=0.11534593254327774
node_1196: feature_name=GO:0046500 feature_id[171].value <= threshold=0.6314916908740997
node_1197: feature_name=GO:0030852 feature_id[22].value <= threshold=2.9768868684768677
node_1198: feature_name=hsa04672 feature_id[276].value <= threshold=8.06681227684021
node_1199: feature_name=GO:0001836 feature_id[450].value > threshold=0.09961023926734924
node_1201: feature_name=GO:0070230 feature_id[64].value <= threshold=3.3956379890441895
node_1202: feature_name=GO:0070102 feature_id[49].value <= threshold=4.805386543273926
node_1203: feature_name=GO:0045553 feature_id[736].value <= threshold=1.4970332980155945
node_1204: feature_name=GO:0051311 feature_id[58].value <= threshold=3.301889181137085
node_1205: feature_name=GO:0030291 feature_id[147].value <= threshold=8.156315326690674
node_1206: feature_name=GO:0045637 feature_id[241].value > threshold=0.01834342861548066
node_1208: feature_name=GO:0002890 feature_id[98].value <= threshold=2.767483353614807
node_1209: feature_name=GO:0046632 feature_id[248].value <= threshold=11.663254261016846
node_1210: feature_name=GO:0008588 feature_id[26].value <= threshold=1.391375720500946
node_1211: feature_name=GO:0010639 feature_id[564].value <= threshold=18.975126266479492
node_1212: feature_name=GO:0046685 feature_id[782].value <= threshold=5.153738260269165
node_1213: feature_name=GO:0038116 feature_id[668].value <= threshold=1.7859655618667603
node_1214: feature_name=GO:0046449 feature_id[763].value <= threshold=5.603115558624268
node_1215: feature_name=GO:0048539 feature_id[319].value <= threshold=0.5478232800960541
node_1216: feature_name=GO:0007089 feature_id[522].value <= threshold=0.9940084517002106
node_1217: feature_name=GO:0042287 feature_id[20].value <= threshold=2.693045496940613
node_1218: feature_name=GO:0044446 feature_id[184].value <= threshold=113.0758171081543
node_1219: feature_name=GO:0098602 feature_id[702].value <= threshold=68.95328903198242
node_1220: feature_name=GO:0035726 feature_id[270].value <= threshold=1.7167096138000488
node_1221: feature_name=GO:1902564 feature_id[85].value <= threshold=2.8364468812942505
node_1222: feature_name=GO:0006979 feature_id[44].value > threshold=0.6869173645973206
node_1224: feature_name=GO:0044464 feature_id[56].value <= threshold=67.13260269165039
node_1225: feature_name=GO:0045629 feature_id[746].value <= threshold=2.3414241075515747
node_1226: feature_name=GO:0002903 feature_id[10].value > threshold=2.715258240699768
node_1252: feature_name=GO:0051984 feature_id[459].value > threshold=0.44557371735572815
Class: negative genes

Rules_175 passed counts:5

node_0: feature_name=GO:0042113 feature_id[0].value <= threshold=13.408552169799805
node_1: feature_name=GO:0007568 feature_id[534].value > threshold=5.0313897132873535
node_913: feature_name=GO:0032763 feature_id[629].value <= threshold=0.31753237545490265
node_914: feature_name=GO:0097193 feature_id[167].value <= threshold=28.171168327331543
node_915: feature_name=GO:0002903 feature_id[10].value <= threshold=1.0534588098526
node_916: feature_name=GO:1904029 feature_id[208].value <= threshold=23.852136611938477
node_917: feature_name=GO:0050897 feature_id[164].value > threshold=0.6870408356189728
node_1095: feature_name=GO:0072539 feature_id[68].value <= threshold=2.5667877197265625
node_1096: feature_name=GO:0046500 feature_id[171].value <= threshold=3.195197820663452
node_1097: feature_name=GO:0045861 feature_id[761].value > threshold=0.22614753991365433
node_1099: feature_name=GO:0043372 feature_id[242].value <= threshold=4.892506122589111
node_1100: feature_name=GO:0042162 feature_id[355].value <= threshold=11.339290618896484
node_1101: feature_name=hsa04660 feature_id[472].value > threshold=24.27848720550537
node_1143: feature_name=GO:0019814 feature_id[189].value <= threshold=0.5058850646018982
Class: positive genes

Rules_176 passed counts:5
node_0: feature_name=GO:0042113 feature_id[0].value <= threshold=13.408552169799805
node_1: feature_name=GO:0007568 feature_id[534].value > threshold=5.0313897132873535
node_913: feature_name=GO:0032763 feature_id[629].value <= threshold=0.31753237545490265
node_914: feature_name=GO:0097193 feature_id[167].value <= threshold=28.171168327331543
node_915: feature_name=GO:0002903 feature_id[10].value <= threshold=1.0534588098526
node_916: feature_name=GO:1904029 feature_id[208].value <= threshold=23.852136611938477
node_917: feature_name=GO:0050897 feature_id[164].value <= threshold=0.6870408356189728
node_918: feature_name=GO:0006139 feature_id[474].value > threshold=1.3052097624921544e-07
node_920: feature_name=GO:0002821 feature_id[588].value <= threshold=13.81072187423706
node_921: feature_name=GO:0006298 feature_id[621].value <= threshold=24.078600883483887
node_922: feature_name=GO:0003908 feature_id[11].value <= threshold=1.7776933312416077
node_923: feature_name=GO:0030098 feature_id[273].value > threshold=0.0079949083738029
node_927: feature_name=GO:0006808 feature_id[513].value <= threshold=4.34592080116272
node_928: feature_name=GO:0071887 feature_id[283].value <= threshold=10.353787899017334
node_929: feature_name=GO:0038001 feature_id[28].value <= threshold=3.9469382762908936
node_930: feature_name=GO:0042287 feature_id[20].value <= threshold=3.6209195852279663
node_931: feature_name=GO:0003968 feature_id[47].value <= threshold=2.004227638244629
node_932: feature_name=GO:0002698 feature_id[395].value <= threshold=18.51447582244873

node_933: feature_name=GO:0044710 feature_id[719].value <= threshold=179.7340316772461
node_934: feature_name=GO:0007568 feature_id[534].value > threshold=5.035318374633789
node_936: feature_name=GO:0006216 feature_id[504].value <= threshold=2.2605666518211365
node_937: feature_name=GO:0048569 feature_id[793].value <= threshold=5.340231895446777
node_938: feature_name=GO:0001777 feature_id[380].value <= threshold=4.100832939147949
node_939: feature_name=GO:0007600 feature_id[122].value <= threshold=171.13383102416992
node_940: feature_name=GO:0001779 feature_id[378].value <= threshold=6.234851121902466
node_941: feature_name=GO:0030291 feature_id[147].value <= threshold=10.8051118850708
node_942: feature_name=GO:0048534 feature_id[790].value > threshold=0.017984486185014248
node_944: feature_name=GO:0070245 feature_id[326].value <= threshold=3.1282339096069336
node_945: feature_name=GO:0009086 feature_id[547].value > threshold=0.2896959036588669
node_1017: feature_name=GO:0000302 feature_id[375].value > threshold=0.305715873837471
node_1019: feature_name=GO:0070141 feature_id[37].value <= threshold=4.857294321060181
node_1020: feature_name=GO:0009086 feature_id[547].value > threshold=0.2945319563150406
node_1024: feature_name=GO:0010332 feature_id[557].value <= threshold=15.092710971832275
node_1025: feature_name=GO:0002524 feature_id[72].value <= threshold=2.531021237373352
node_1026: feature_name=GO:0016032 feature_id[571].value <= threshold=60.173330307006836
node_1027: feature_name=GO:0002832 feature_id[491].value <= threshold=2.0699684023857117
node_1028: feature_name=GO:0008588 feature_id[26].value <= threshold=2.150238513946533
node_1029: feature_name=GO:0009615 feature_id[265].value > threshold=0.02372477948665619
node_1031: feature_name=GO:0002763 feature_id[271].value <= threshold=6.462319850921631
node_1032: feature_name=GO:0045840 feature_id[697].value <= threshold=10.30175256729126
node_1033: feature_name=GO:0051402 feature_id[568].value <= threshold=1.2523645758628845
node_1034: feature_name=GO:0008625 feature_id[99].value > threshold=1.086933195590973
node_1038: feature_name=GO:0016064 feature_id[570].value <= threshold=1.333651602268219
Class: positive genes

Rules_177 passed counts:5
node_0: feature_name=GO:0042113 feature_id[0].value <= threshold=13.408552169799805
node_1: feature_name=GO:0007568 feature_id[534].value > threshold=5.0313897132873535
node_913: feature_name=GO:0032763 feature_id[629].value <= threshold=0.31753237545490265
node_914: feature_name=GO:0097193 feature_id[167].value <= threshold=28.171168327331543
node_915: feature_name=GO:0002903 feature_id[10].value <= threshold=1.0534588098526
node_916: feature_name=GO:1904029 feature_id[208].value <= threshold=23.852136611938477
node_917: feature_name=GO:0050897 feature_id[164].value <= threshold=0.6870408356189728

node_918: feature_name=GO:0006139 feature_id[474].value > threshold=1.3052097624921544e-07
node_920: feature_name=GO:0002821 feature_id[588].value <= threshold=13.81072187423706
node_921: feature_name=GO:0006298 feature_id[621].value <= threshold=24.078600883483887
node_922: feature_name=GO:0003908 feature_id[11].value <= threshold=1.7776933312416077
node_923: feature_name=GO:0030098 feature_id[273].value > threshold=0.0079949083738029
node_927: feature_name=GO:0006808 feature_id[513].value <= threshold=4.34592080116272
node_928: feature_name=GO:0071887 feature_id[283].value <= threshold=10.353787899017334
node_929: feature_name=GO:0038001 feature_id[28].value <= threshold=3.9469382762908936
node_930: feature_name=GO:0042287 feature_id[20].value <= threshold=3.6209195852279663
node_931: feature_name=GO:0003968 feature_id[47].value <= threshold=2.004227638244629
node_932: feature_name=GO:0002698 feature_id[395].value <= threshold=18.51447582244873
node_933: feature_name=GO:0044710 feature_id[719].value <= threshold=179.7340316772461
node_934: feature_name=GO:0007568 feature_id[534].value > threshold=5.035318374633789
node_936: feature_name=GO:0006216 feature_id[504].value <= threshold=2.2605666518211365
node_937: feature_name=GO:0048569 feature_id[793].value <= threshold=5.340231895446777
node_938: feature_name=GO:0001777 feature_id[380].value <= threshold=4.100832939147949
node_939: feature_name=GO:0007600 feature_id[122].value <= threshold=171.13383102416992
node_940: feature_name=GO:0001779 feature_id[378].value <= threshold=6.234851121902466
node_941: feature_name=GO:0030291 feature_id[147].value <= threshold=10.8051118850708
node_942: feature_name=GO:0048534 feature_id[790].value > threshold=0.017984486185014248
node_944: feature_name=GO:0070245 feature_id[326].value <= threshold=3.1282339096069336
node_945: feature_name=GO:0009086 feature_id[547].value <= threshold=0.2896959036588669
node_946: feature_name=GO:0001889 feature_id[387].value <= threshold=17.643128395080566
node_947: feature_name=GO:0048144 feature_id[192].value > threshold=0.20887330174446106
node_951: feature_name=GO:0023026 feature_id[69].value <= threshold=3.403424024581909
node_952: feature_name=GO:0048147 feature_id[788].value <= threshold=4.188283443450928
node_953: feature_name=GO:0090116 feature_id[97].value > threshold=4.617154359817505
node_1007: feature_name=hsa04650 feature_id[13].value <= threshold=4.294154644012451
Class: negative genes

Rules_178 passed counts:5
node_0: feature_name=GO:0042113 feature_id[0].value <= threshold=13.408552169799805
node_1: feature_name=GO:0007568 feature_id[534].value <= threshold=5.0313897132873535
node_2: feature_name=GO:0002705 feature_id[541].value > threshold=3.200145721435547
node_870: feature_name=GO:0046634 feature_id[46].value > threshold=8.9394211769104

Class: positive genes

Rules_179 passed counts:5
node_0: feature_name=GO:0042113 feature_id[0].value <= threshold=13.408552169799805
node_1: feature_name=GO:0007568 feature_id[534].value <= threshold=5.0313897132873535
node_2: feature_name=GO:0002705 feature_id[541].value > threshold=3.200145721435547
node_870: feature_name=GO:0046634 feature_id[46].value <= threshold=8.9394211769104
node_871: feature_name=GO:0048523 feature_id[293].value <= threshold=19.963229179382324
node_872: feature_name=GO:2001237 feature_id[796].value > threshold=0.09761488810181618
node_880: feature_name=GO:0006808 feature_id[513].value <= threshold=1.7969900965690613
node_881: feature_name=GO:0032479 feature_id[441].value <= threshold=0.13597124069929123
node_882: feature_name=GO:0000060 feature_id[223].value > threshold=2.2508251667022705
Class: positive genes

Rules_180 passed counts:5
node_0: feature_name=GO:0042113 feature_id[0].value <= threshold=13.408552169799805
node_1: feature_name=GO:0007568 feature_id[534].value <= threshold=5.0313897132873535
node_2: feature_name=GO:0002705 feature_id[541].value <= threshold=3.200145721435547
node_3: feature_name=GO:1901525 feature_id[576].value > threshold=0.4466460347175598
node_617: feature_name=GO:0005622 feature_id[233].value <= threshold=90.85730743408203
node_618: feature_name=GO:1903147 feature_id[200].value <= threshold=7.326428413391113
node_619: feature_name=GO:0003964 feature_id[317].value <= threshold=5.575642108917236
node_620: feature_name=GO:0046498 feature_id[203].value <= threshold=6.620223522186279
node_621: feature_name=GO:0002562 feature_id[25].value <= threshold=10.287704467773438
node_622: feature_name=GO:0006919 feature_id[246].value <= threshold=5.801334381103516
node_623: feature_name=GO:0001889 feature_id[387].value > threshold=2.679414987564087
node_785: feature_name=GO:0030887 feature_id[274].value <= threshold=1.1379476189613342
node_786: feature_name=GO:1902564 feature_id[85].value <= threshold=1.4637635350227356
node_787: feature_name=GO:0045402 feature_id[286].value <= threshold=1.5249269604682922
node_788: feature_name=GO:0034614 feature_id[655].value <= threshold=9.142115116119385
node_789: feature_name=GO:0000783 feature_id[191].value > threshold=1.5124736428260803
node_825: feature_name=GO:0033262 feature_id[643].value > threshold=0.7662047147750854
Class: negative genes

Rules_181 passed counts:5

node_0: feature_name=GO:0042113 feature_id[0].value <= threshold=13.408552169799805
node_1: feature_name=GO:0007568 feature_id[534].value <= threshold=5.0313897132873535
node_2: feature_name=GO:0002705 feature_id[541].value <= threshold=3.200145721435547
node_3: feature_name=GO:1901525 feature_id[576].value > threshold=0.4466460347175598
node_617: feature_name=GO:0005622 feature_id[233].value <= threshold=90.85730743408203
node_618: feature_name=GO:1903147 feature_id[200].value <= threshold=7.326428413391113
node_619: feature_name=GO:0003964 feature_id[317].value <= threshold=5.575642108917236
node_620: feature_name=GO:0046498 feature_id[203].value <= threshold=6.620223522186279
node_621: feature_name=GO:0002562 feature_id[25].value <= threshold=10.287704467773438
node_622: feature_name=GO:0006919 feature_id[246].value <= threshold=5.801334381103516
node_623: feature_name=GO:0001889 feature_id[387].value <= threshold=2.679414987564087
node_624: feature_name=GO:0006555 feature_id[514].value <= threshold=7.819535493850708
node_625: feature_name=GO:0080134 feature_id[650].value <= threshold=38.91967582702637
node_626: feature_name=GO:0042127 feature_id[480].value <= threshold=37.72536659240723
node_627: feature_name=GO:2001251 feature_id[220].value <= threshold=32.564422607421875
node_628: feature_name=GO:0002829 feature_id[493].value <= threshold=2.444612741470337
node_629: feature_name=GO:0008588 feature_id[26].value <= threshold=2.3601274490356445
node_630: feature_name=GO:0033151 feature_id[7].value <= threshold=5.134376287460327
node_631: feature_name=GO:0048145 feature_id[348].value <= threshold=8.836549282073975
node_632: feature_name=GO:0030887 feature_id[274].value <= threshold=1.7000296115875244
node_633: feature_name=GO:0097190 feature_id[117].value <= threshold=33.02078819274902
node_634: feature_name=GO:0046685 feature_id[782].value <= threshold=8.040813446044922
node_635: feature_name=GO:0006346 feature_id[236].value <= threshold=6.384642839431763
node_636: feature_name=GO:0048537 feature_id[66].value <= threshold=3.1739262342453003
node_637: feature_name=GO:0070245 feature_id[326].value <= threshold=3.0491198301315308
node_638: feature_name=GO:0038065 feature_id[670].value <= threshold=2.0590850114822388
node_639: feature_name=GO:0005488 feature_id[187].value <= threshold=84.80076217651367
node_640: feature_name=GO:0060249 feature_id[390].value <= threshold=0.07226398587226868
node_641: feature_name=GO:0002683 feature_id[456].value > threshold=0.7958214282989502
node_647: feature_name=GO:0045638 feature_id[94].value > threshold=0.7937153577804565
Class: positive genes

Rules_182 passed counts:5
node_0: feature_name=GO:0042113 feature_id[0].value <= threshold=13.408552169799805
node_1: feature_name=GO:0007568 feature_id[534].value <= threshold=5.0313897132873535

node_2: feature_name=GO:0002705 feature_id[541].value <= threshold=3.200145721435547
node_3: feature_name=GO:1901525 feature_id[576].value <= threshold=0.4466460347175598
node_4: feature_name=GO:0048539 feature_id[319].value <= threshold=3.0399646759033203
node_5: feature_name=GO:0001910 feature_id[385].value <= threshold=3.7437864542007446
node_6: feature_name=GO:0043200 feature_id[706].value <= threshold=9.307284355163574
node_7: feature_name=GO:0001773 feature_id[308].value <= threshold=4.016931533813477
node_8: feature_name=GO:0090116 feature_id[97].value <= threshold=7.99645471572876
node_9: feature_name=GO:0019814 feature_id[189].value <= threshold=4.525782108306885
node_10: feature_name=GO:1902583 feature_id[215].value <= threshold=17.60310649871826
node_11: feature_name=GO:0045429 feature_id[737].value > threshold=1.6133361458778381
node_477: feature_name=GO:0036037 feature_id[445].value <= threshold=1.7181594371795654
node_478: feature_name=GO:0006244 feature_id[503].value <= threshold=0.951388418674469
node_479: feature_name=GO:0032461 feature_id[608].value <= threshold=2.6103241443634033
node_480: feature_name=GO:0044710 feature_id[719].value > threshold=2.228096718681627e-06
node_494: feature_name=GO:0009314 feature_id[296].value <= threshold=34.93696117401123
node_495: feature_name=GO:0050778 feature_id[802].value <= threshold=39.29364013671875
node_496: feature_name=GO:1903038 feature_id[492].value <= threshold=5.849650859832764
node_497: feature_name=GO:0002699 feature_id[606].value <= threshold=5.5405943393707275
node_498: feature_name=hsa00983 feature_id[181].value <= threshold=25.851045608520508
node_499: feature_name=GO:0002309 feature_id[416].value <= threshold=1.6235689520835876
node_500: feature_name=GO:1901698 feature_id[792].value <= threshold=47.70275688171387
node_501: feature_name=GO:0010216 feature_id[282].value <= threshold=1.6849713921546936
node_502: feature_name=GO:0006266 feature_id[297].value <= threshold=2.7199249267578125
node_503: feature_name=GO:0005488 feature_id[187].value > threshold=3.0373169010999845e-06
node_507: feature_name=GO:0045429 feature_id[737].value > threshold=1.614579975605011
node_511: feature_name=GO:0034103 feature_id[318].value <= threshold=5.832815647125244
node_512: feature_name=GO:0010663 feature_id[561].value <= threshold=4.157005429267883
node_513: feature_name=GO:0030887 feature_id[274].value <= threshold=1.8964150547981262
node_514: feature_name=GO:0048294 feature_id[113].value <= threshold=1.866134524345398
node_515: feature_name=GO:0072539 feature_id[68].value <= threshold=0.8600535988807678
node_516: feature_name=GO:0045840 feature_id[697].value <= threshold=4.963376045227051
node_517: feature_name=GO:0019222 feature_id[129].value > threshold=4.0122095015249215e-06
node_523: feature_name=GO:0008150 feature_id[528].value <= threshold=40.503862380981445
node_524: feature_name=GO:0006359 feature_id[87].value <= threshold=2.954534411430359
node_525: feature_name=GO:0010225 feature_id[558].value > threshold=1.8247058987617493

node_535: feature_name=GO:0046427 feature_id[766].value > threshold=0.07467848435044289
Class: negative genes

Rules_183 passed counts:5
node_0: feature_name=GO:0042113 feature_id[0].value <= threshold=13.408552169799805
node_1: feature_name=GO:0007568 feature_id[534].value <= threshold=5.0313897132873535
node_2: feature_name=GO:0002705 feature_id[541].value <= threshold=3.200145721435547
node_3: feature_name=GO:1901525 feature_id[576].value <= threshold=0.4466460347175598
node_4: feature_name=GO:0048539 feature_id[319].value <= threshold=3.0399646759033203
node_5: feature_name=GO:0001910 feature_id[385].value <= threshold=3.7437864542007446
node_6: feature_name=GO:0043200 feature_id[706].value <= threshold=9.307284355163574
node_7: feature_name=GO:0001773 feature_id[308].value <= threshold=4.016931533813477
node_8: feature_name=GO:0090116 feature_id[97].value <= threshold=7.99645471572876
node_9: feature_name=GO:0019814 feature_id[189].value <= threshold=4.525782108306885
node_10: feature_name=GO:1902583 feature_id[215].value <= threshold=17.60310649871826
node_11: feature_name=GO:0045429 feature_id[737].value > threshold=1.6133361458778381
node_477: feature_name=GO:0036037 feature_id[445].value <= threshold=1.7181594371795654
node_478: feature_name=GO:0006244 feature_id[503].value <= threshold=0.951388418674469
node_479: feature_name=GO:0032461 feature_id[608].value <= threshold=2.6103241443634033
node_480: feature_name=GO:0044710 feature_id[719].value > threshold=2.228096718681627e-06
node_494: feature_name=GO:0009314 feature_id[296].value <= threshold=34.93696117401123
node_495: feature_name=GO:0050778 feature_id[802].value <= threshold=39.29364013671875
node_496: feature_name=GO:1903038 feature_id[492].value <= threshold=5.849650859832764
node_497: feature_name=GO:0002699 feature_id[606].value <= threshold=5.5405943393707275
node_498: feature_name=hsa00983 feature_id[181].value <= threshold=25.851045608520508
node_499: feature_name=GO:0002309 feature_id[416].value <= threshold=1.6235689520835876
node_500: feature_name=GO:1901698 feature_id[792].value <= threshold=47.70275688171387
node_501: feature_name=GO:0010216 feature_id[282].value <= threshold=1.6849713921546936
node_502: feature_name=GO:0006266 feature_id[297].value <= threshold=2.7199249267578125
node_503: feature_name=GO:0005488 feature_id[187].value > threshold=3.0373169010999845e-06
node_507: feature_name=GO:0045429 feature_id[737].value > threshold=1.614579975605011
node_511: feature_name=GO:0034103 feature_id[318].value <= threshold=5.832815647125244
node_512: feature_name=GO:0010663 feature_id[561].value <= threshold=4.157005429267883
node_513: feature_name=GO:0030887 feature_id[274].value <= threshold=1.8964150547981262
node_514: feature_name=GO:0048294 feature_id[113].value <= threshold=1.866134524345398

node_515: feature_name=GO:0072539 feature_id[68].value <= threshold=0.8600535988807678
node_516: feature_name=GO:0045840 feature_id[697].value <= threshold=4.963376045227051
node_517: feature_name=GO:0019222 feature_id[129].value > threshold=4.0122095015249215e-06
node_523: feature_name=GO:0008150 feature_id[528].value <= threshold=40.503862380981445
node_524: feature_name=GO:0006359 feature_id[87].value <= threshold=2.954534411430359
node_525: feature_name=GO:0010225 feature_id[558].value <= threshold=1.8247058987617493
node_526: feature_name=GO:0032461 feature_id[608].value > threshold=1.6246166825294495
node_532: feature_name=GO:0050672 feature_id[800].value > threshold=0.301830917596817
Class: negative genes

Rules_184 passed counts:5
node_0: feature_name=GO:0042113 feature_id[0].value <= threshold=13.408552169799805
node_1: feature_name=GO:0007568 feature_id[534].value <= threshold=5.0313897132873535
node_2: feature_name=GO:0002705 feature_id[541].value <= threshold=3.200145721435547
node_3: feature_name=GO:1901525 feature_id[576].value <= threshold=0.4466460347175598
node_4: feature_name=GO:0048539 feature_id[319].value <= threshold=3.0399646759033203
node_5: feature_name=GO:0001910 feature_id[385].value <= threshold=3.7437864542007446
node_6: feature_name=GO:0043200 feature_id[706].value <= threshold=9.307284355163574
node_7: feature_name=GO:0001773 feature_id[308].value <= threshold=4.016931533813477
node_8: feature_name=GO:0090116 feature_id[97].value <= threshold=7.99645471572876
node_9: feature_name=GO:0019814 feature_id[189].value <= threshold=4.525782108306885
node_10: feature_name=GO:1902583 feature_id[215].value <= threshold=17.60310649871826
node_11: feature_name=GO:0045429 feature_id[737].value <= threshold=1.6133361458778381
node_12: feature_name=GO:0003720 feature_id[228].value <= threshold=5.519326210021973
node_13: feature_name=GO:0046006 feature_id[364].value <= threshold=4.9985432624816895
node_14: feature_name=GO:0070424 feature_id[254].value <= threshold=6.233297109603882
node_15: feature_name=GO:0009892 feature_id[320].value <= threshold=59.99180793762207
node_16: feature_name=GO:0007064 feature_id[527].value <= threshold=11.57377815246582
node_17: feature_name=GO:0005575 feature_id[17].value <= threshold=15.417460918426514
node_18: feature_name=GO:0043368 feature_id[31].value <= threshold=4.059496641159058
node_19: feature_name=GO:0005164 feature_id[163].value <= threshold=5.793607711791992
node_20: feature_name=GO:0042130 feature_id[16].value <= threshold=5.073179721832275
node_21: feature_name=GO:0010216 feature_id[282].value <= threshold=4.834584474563599
node_22: feature_name=GO:0009628 feature_id[553].value <= threshold=29.314892768859863
node_23: feature_name=GO:0045628 feature_id[749].value <= threshold=3.1001367568969727

node_24: feature_name=GO:0042288	feature_id[100].value <= threshold=5.174932956695557
node_25: feature_name=GO:0002329	feature_id[406].value <= threshold=2.348930835723877
node_26: feature_name=GO:0051246	feature_id[642].value > threshold=0.6498909294605255
node_194: feature_name=GO:0009164	feature_id[546].value <= threshold=17.11970329284668
node_195: feature_name=GO:0001909	feature_id[386].value <= threshold=7.292566299438477
node_196: feature_name=GO:0003908	feature_id[11].value <= threshold=1.887226164340973
node_197: feature_name=GO:0009164	feature_id[546].value > threshold=0.26642198860645294
node_359: feature_name=GO:0009164	feature_id[546].value > threshold=0.2696942090988159
node_361: feature_name=hsa05202	feature_id[50].value <= threshold=14.989679336547852
node_362: feature_name=GO:0001775	feature_id[505].value <= threshold=15.726949691772461
node_363: feature_name=GO:0002634	feature_id[449].value <= threshold=2.825531005859375
node_364: feature_name=GO:0010038	feature_id[457].value <= threshold=9.901018619537354
node_365: feature_name=GO:0044238	feature_id[295].value <= threshold=139.80535888671875
node_366: feature_name=GO:0051246	feature_id[642].value > threshold=0.6527281999588013
node_368: feature_name=GO:0044092	feature_id[432].value > threshold=11.084641933441162
node_436: feature_name=GO:0071901	feature_id[407].value <= threshold=1.6024202108383179

Class: negative genes

Rules_185	passed counts:5
node_0: feature_name=GO:0042113	feature_id[0].value <= threshold=13.408552169799805
node_1: feature_name=GO:0007568	feature_id[534].value <= threshold=5.0313897132873535
node_2: feature_name=GO:0002705	feature_id[541].value <= threshold=3.200145721435547
node_3: feature_name=GO:1901525	feature_id[576].value <= threshold=0.4466460347175598
node_4: feature_name=GO:0048539	feature_id[319].value <= threshold=3.0399646759033203
node_5: feature_name=GO:0001910	feature_id[385].value <= threshold=3.7437864542007446
node_6: feature_name=GO:0043200	feature_id[706].value <= threshold=9.307284355163574
node_7: feature_name=GO:0001773	feature_id[308].value <= threshold=4.016931533813477
node_8: feature_name=GO:0090116	feature_id[97].value <= threshold=7.99645471572876
node_9: feature_name=GO:0019814	feature_id[189].value <= threshold=4.525782108306885
node_10: feature_name=GO:1902583	feature_id[215].value <= threshold=17.60310649871826
node_11: feature_name=GO:0045429	feature_id[737].value <= threshold=1.6133361458778381
node_12: feature_name=GO:0003720	feature_id[228].value <= threshold=5.519326210021973
node_13: feature_name=GO:0046006	feature_id[364].value <= threshold=4.9985432624816895
node_14: feature_name=GO:0070424	feature_id[254].value <= threshold=6.233297109603882
node_15: feature_name=GO:0009892	feature_id[320].value <= threshold=59.99180793762207

node_16: feature_name=GO:0007064	feature_id[527].value <= threshold=11.57377815246582
node_17: feature_name=GO:0005575	feature_id[17].value <= threshold=15.417460918426514
node_18: feature_name=GO:0043368	feature_id[31].value <= threshold=4.059496641159058
node_19: feature_name=GO:0005164	feature_id[163].value <= threshold=5.793607711791992
node_20: feature_name=GO:0042130	feature_id[16].value <= threshold=5.073179721832275
node_21: feature_name=GO:0010216	feature_id[282].value <= threshold=4.834584474563599
node_22: feature_name=GO:0009628	feature_id[553].value <= threshold=29.314892768859863
node_23: feature_name=GO:0045628	feature_id[749].value <= threshold=3.1001367568969727
node_24: feature_name=GO:0042288	feature_id[100].value <= threshold=5.174932956695557
node_25: feature_name=GO:0002329	feature_id[406].value <= threshold=2.348930835723877
node_26: feature_name=GO:0051246	feature_id[642].value > threshold=0.6498909294605255
node_194: feature_name=GO:0009164	feature_id[546].value <= threshold=17.11970329284668
node_195: feature_name=GO:0001909	feature_id[386].value <= threshold=7.292566299438477
node_196: feature_name=GO:0003908	feature_id[11].value <= threshold=1.887226164340973
node_197: feature_name=GO:0009164	feature_id[546].value <= threshold=0.26642198860645294
node_198: feature_name=GO:2001242	feature_id[24].value <= threshold=9.207026481628418
node_199: feature_name=GO:0051246	feature_id[642].value > threshold=0.6504445374011993
node_203: feature_name=GO:0032461	feature_id[608].value <= threshold=2.607542634010315
node_204: feature_name=GO:0007584	feature_id[529].value <= threshold=16.099515914916992
node_205: feature_name=hsa05221	feature_id[349].value <= threshold=9.359850406646729
node_206: feature_name=GO:0002636	feature_id[447].value <= threshold=1.615447759628296
node_207: feature_name=GO:0033993	feature_id[421].value > threshold=2.96540611088858e-05
node_217: feature_name=GO:0002331	feature_id[404].value <= threshold=2.1883513927459717
node_218: feature_name=GO:0046498	feature_id[203].value <= threshold=4.085246205329895
node_219: feature_name=GO:0002832	feature_id[491].value <= threshold=3.0228612422943115
node_220: feature_name=GO:1904868	feature_id[306].value <= threshold=3.8674756288528442
node_221: feature_name=GO:0048545	feature_id[360].value <= threshold=27.38136100769043
node_222: feature_name=GO:0071310	feature_id[760].value <= threshold=2.3522024154663086
node_223: feature_name=GO:0001775	feature_id[505].value <= threshold=9.71301555633545
node_224: feature_name=GO:0071310	feature_id[760].value <= threshold=2.346743583679199
node_225: feature_name=GO:0001772	feature_id[91].value <= threshold=3.252573609352112
node_226: feature_name=GO:0036498	feature_id[359].value <= threshold=12.092026710510254
node_227: feature_name=hsa05340	feature_id[351].value <= threshold=0.7677814364433289
node_228: feature_name=GO:0032703	feature_id[617].value <= threshold=1.7450646758079529
node_229: feature_name=hsa04640	feature_id[79].value <= threshold=2.118402123451233

node_230: feature_name=GO:0042097 feature_id[675].value <= threshold=1.8110727667808533
node_231: feature_name=GO:0071391 feature_id[518].value <= threshold=2.691588521003723
node_232: feature_name=GO:0001666 feature_id[682].value <= threshold=5.225740432739258
node_233: feature_name=GO:0048539 feature_id[319].value <= threshold=1.8523842096328735
node_234: feature_name=GO:0038061 feature_id[671].value > threshold=8.564527988433838
node_260: feature_name=hsa04115 feature_id[324].value <= threshold=0.9803315252065659
Class: negative genes

Rules_186 passed counts:5
node_0: feature_name=GO:0042113 feature_id[0].value <= threshold=13.408552169799805
node_1: feature_name=GO:0007568 feature_id[534].value <= threshold=5.0313897132873535
node_2: feature_name=GO:0002705 feature_id[541].value <= threshold=3.200145721435547
node_3: feature_name=GO:1901525 feature_id[576].value <= threshold=0.4466460347175598
node_4: feature_name=GO:0048539 feature_id[319].value <= threshold=3.0399646759033203
node_5: feature_name=GO:0001910 feature_id[385].value <= threshold=3.7437864542007446
node_6: feature_name=GO:0043200 feature_id[706].value <= threshold=9.307284355163574
node_7: feature_name=GO:0001773 feature_id[308].value <= threshold=4.016931533813477
node_8: feature_name=GO:0090116 feature_id[97].value <= threshold=7.99645471572876
node_9: feature_name=GO:0019814 feature_id[189].value <= threshold=4.525782108306885
node_10: feature_name=GO:1902583 feature_id[215].value <= threshold=17.60310649871826
node_11: feature_name=GO:0045429 feature_id[737].value <= threshold=1.6133361458778381
node_12: feature_name=GO:0003720 feature_id[228].value <= threshold=5.519326210021973
node_13: feature_name=GO:0046006 feature_id[364].value <= threshold=4.9985432624816895
node_14: feature_name=GO:0070424 feature_id[254].value <= threshold=6.233297109603882
node_15: feature_name=GO:0009892 feature_id[320].value <= threshold=59.99180793762207
node_16: feature_name=GO:0007064 feature_id[527].value <= threshold=11.57377815246582
node_17: feature_name=GO:0005575 feature_id[17].value <= threshold=15.417460918426514
node_18: feature_name=GO:0043368 feature_id[31].value <= threshold=4.059496641159058
node_19: feature_name=GO:0005164 feature_id[163].value <= threshold=5.793607711791992
node_20: feature_name=GO:0042130 feature_id[16].value <= threshold=5.073179721832275
node_21: feature_name=GO:0010216 feature_id[282].value <= threshold=4.834584474563599
node_22: feature_name=GO:0009628 feature_id[553].value <= threshold=29.314892768859863
node_23: feature_name=GO:0045628 feature_id[749].value <= threshold=3.1001367568969727
node_24: feature_name=GO:0042288 feature_id[100].value <= threshold=5.174932956695557
node_25: feature_name=GO:0002329 feature_id[406].value <= threshold=2.348930835723877

node_26: feature_name=GO:0051246 feature_id[642].value > threshold=0.6498909294605255
node_194: feature_name=GO:0009164 feature_id[546].value <= threshold=17.11970329284668
node_195: feature_name=GO:0001909 feature_id[386].value <= threshold=7.292566299438477
node_196: feature_name=GO:0003908 feature_id[11].value <= threshold=1.887226164340973
node_197: feature_name=GO:0009164 feature_id[546].value <= threshold=0.26642198860645294
node_198: feature_name=GO:2001242 feature_id[24].value <= threshold=9.207026481628418
node_199: feature_name=GO:0051246 feature_id[642].value > threshold=0.6504445374011993
node_203: feature_name=GO:0032461 feature_id[608].value <= threshold=2.607542634010315
node_204: feature_name=GO:0007584 feature_id[529].value <= threshold=16.099515914916992
node_205: feature_name=hsa05221 feature_id[349].value <= threshold=9.359850406646729
node_206: feature_name=GO:0002636 feature_id[447].value <= threshold=1.615447759628296
node_207: feature_name=GO:0033993 feature_id[421].value <= threshold=2.96540611088858e-05
node_208: feature_name=GO:0006968 feature_id[517].value <= threshold=1.0216356217861176
node_209: feature_name=GO:0048545 feature_id[360].value <= threshold=0.007158883148804307
node_210: feature_name=GO:0002250 feature_id[417].value > threshold=0.3659738004207611
node_212: feature_name=GO:0034644 feature_id[652].value <= threshold=0.06354941427707672
Class: negative genes

Rules_187 passed counts:5
node_0: feature_name=GO:0042113 feature_id[0].value <= threshold=13.408552169799805
node_1: feature_name=GO:0007568 feature_id[534].value <= threshold=5.0313897132873535
node_2: feature_name=GO:0002705 feature_id[541].value <= threshold=3.200145721435547
node_3: feature_name=GO:1901525 feature_id[576].value <= threshold=0.4466460347175598
node_4: feature_name=GO:0048539 feature_id[319].value <= threshold=3.0399646759033203
node_5: feature_name=GO:0001910 feature_id[385].value <= threshold=3.7437864542007446
node_6: feature_name=GO:0043200 feature_id[706].value <= threshold=9.307284355163574
node_7: feature_name=GO:0001773 feature_id[308].value <= threshold=4.016931533813477
node_8: feature_name=GO:0090116 feature_id[97].value <= threshold=7.99645471572876
node_9: feature_name=GO:0019814 feature_id[189].value <= threshold=4.525782108306885
node_10: feature_name=GO:1902583 feature_id[215].value <= threshold=17.60310649871826
node_11: feature_name=GO:0045429 feature_id[737].value <= threshold=1.6133361458778381
node_12: feature_name=GO:0003720 feature_id[228].value <= threshold=5.519326210021973
node_13: feature_name=GO:0046006 feature_id[364].value <= threshold=4.9985432624816895
node_14: feature_name=GO:0070424 feature_id[254].value <= threshold=6.233297109603882
node_15: feature_name=GO:0009892 feature_id[320].value <= threshold=59.99180793762207

node_16: feature_name=GO:0007064 feature_id[527].value <= threshold=11.57377815246582
node_17: feature_name=GO:0005575 feature_id[17].value <= threshold=15.417460918426514
node_18: feature_name=GO:0043368 feature_id[31].value <= threshold=4.059496641159058
node_19: feature_name=GO:0005164 feature_id[163].value <= threshold=5.793607711791992
node_20: feature_name=GO:0042130 feature_id[16].value <= threshold=5.073179721832275
node_21: feature_name=GO:0010216 feature_id[282].value <= threshold=4.834584474563599
node_22: feature_name=GO:0009628 feature_id[553].value <= threshold=29.314892768859863
node_23: feature_name=GO:0045628 feature_id[749].value <= threshold=3.1001367568969727
node_24: feature_name=GO:0042288 feature_id[100].value <= threshold=5.174932956695557
node_25: feature_name=GO:0002329 feature_id[406].value <= threshold=2.348930835723877
node_26: feature_name=GO:0051246 feature_id[642].value <= threshold=0.6498909294605255
node_27: feature_name=GO:0042162 feature_id[355].value <= threshold=8.05103588104248
node_28: feature_name=hsa04668 feature_id[333].value <= threshold=5.614926338195801
node_29: feature_name=GO:0032200 feature_id[613].value <= threshold=28.735913276672363
node_30: feature_name=hsa04662 feature_id[334].value <= threshold=9.588344097137451
node_31: feature_name=GO:1901992 feature_id[105].value <= threshold=5.291427850723267
node_32: feature_name=GO:0072341 feature_id[144].value <= threshold=6.01465106010437
node_33: feature_name=GO:0047485 feature_id[121].value <= threshold=6.657836437225342
node_34: feature_name=GO:0090594 feature_id[738].value <= threshold=1.8673912286758423
node_35: feature_name=GO:0043525 feature_id[524].value <= threshold=2.881397008895874
node_36: feature_name=GO:0032504 feature_id[244].value <= threshold=17.22207546234131
node_37: feature_name=GO:0051348 feature_id[126].value <= threshold=5.933559417724609
node_38: feature_name=GO:0042522 feature_id[692].value <= threshold=1.78694087266922
node_39: feature_name=GO:0031100 feature_id[73].value <= threshold=3.2312101125717163
node_40: feature_name=GO:0042493 feature_id[149].value <= threshold=19.771170616149902
node_41: feature_name=GO:0001552 feature_id[366].value <= threshold=2.1711617708206177
node_42: feature_name=GO:0046483 feature_id[391].value > threshold=320.9667053222656
node_148: feature_name=GO:0060576 feature_id[204].value > threshold=0.22821159660816193
Class: negative genes

Rules_188 passed counts:5
node_0: feature_name=GO:0042113 feature_id[0].value <= threshold=13.408552169799805
node_1: feature_name=GO:0007568 feature_id[534].value <= threshold=5.0313897132873535
node_2: feature_name=GO:0002705 feature_id[541].value <= threshold=3.200145721435547
node_3: feature_name=GO:1901525 feature_id[576].value <= threshold=0.4466460347175598

node_4: feature_name=GO:0048539 feature_id[319].value <= threshold=3.0399646759033203
node_5: feature_name=GO:0001910 feature_id[385].value <= threshold=3.7437864542007446
node_6: feature_name=GO:0043200 feature_id[706].value <= threshold=9.307284355163574
node_7: feature_name=GO:0001773 feature_id[308].value <= threshold=4.016931533813477
node_8: feature_name=GO:0090116 feature_id[97].value <= threshold=7.99645471572876
node_9: feature_name=GO:0019814 feature_id[189].value <= threshold=4.525782108306885
node_10: feature_name=GO:1902583 feature_id[215].value <= threshold=17.60310649871826
node_11: feature_name=GO:0045429 feature_id[737].value <= threshold=1.6133361458778381
node_12: feature_name=GO:0003720 feature_id[228].value <= threshold=5.519326210021973
node_13: feature_name=GO:0046006 feature_id[364].value <= threshold=4.9985432624816895
node_14: feature_name=GO:0070424 feature_id[254].value <= threshold=6.233297109603882
node_15: feature_name=GO:0009892 feature_id[320].value <= threshold=59.99180793762207
node_16: feature_name=GO:0007064 feature_id[527].value <= threshold=11.57377815246582
node_17: feature_name=GO:0005575 feature_id[17].value <= threshold=15.417460918426514
node_18: feature_name=GO:0043368 feature_id[31].value <= threshold=4.059496641159058
node_19: feature_name=GO:0005164 feature_id[163].value <= threshold=5.793607711791992
node_20: feature_name=GO:0042130 feature_id[16].value <= threshold=5.073179721832275
node_21: feature_name=GO:0010216 feature_id[282].value <= threshold=4.834584474563599
node_22: feature_name=GO:0009628 feature_id[553].value <= threshold=29.314892768859863
node_23: feature_name=GO:0045628 feature_id[749].value <= threshold=3.1001367568969727
node_24: feature_name=GO:0042288 feature_id[100].value <= threshold=5.174932956695557
node_25: feature_name=GO:0002329 feature_id[406].value <= threshold=2.348930835723877
node_26: feature_name=GO:0051246 feature_id[642].value <= threshold=0.6498909294605255
node_27: feature_name=GO:0042162 feature_id[355].value <= threshold=8.05103588104248
node_28: feature_name=hsa04668 feature_id[333].value <= threshold=5.614926338195801
node_29: feature_name=GO:0032200 feature_id[613].value <= threshold=28.735913276672363
node_30: feature_name=hsa04662 feature_id[334].value <= threshold=9.588344097137451
node_31: feature_name=GO:1901992 feature_id[105].value <= threshold=5.291427850723267
node_32: feature_name=GO:0072341 feature_id[144].value <= threshold=6.01465106010437
node_33: feature_name=GO:0047485 feature_id[121].value <= threshold=6.657836437225342
node_34: feature_name=GO:0090594 feature_id[738].value <= threshold=1.8673912286758423
node_35: feature_name=GO:0043525 feature_id[524].value <= threshold=2.881397008895874
node_36: feature_name=GO:0032504 feature_id[244].value <= threshold=17.22207546234131
node_37: feature_name=GO:0051348 feature_id[126].value <= threshold=5.933559417724609
node_38: feature_name=GO:0042522 feature_id[692].value <= threshold=1.78694087266922

node_39: feature_name=GO:0031100 feature_id[73].value <= threshold=3.2312101125717163
node_40: feature_name=GO:0042493 feature_id[149].value <= threshold=19.771170616149902
node_41: feature_name=GO:0001552 feature_id[366].value <= threshold=2.1711617708206177
node_42: feature_name=GO:0046483 feature_id[391].value <= threshold=320.9667053222656
node_43: feature_name=GO:0002439 feature_id[431].value > threshold=1.832722783088684
node_145: feature_name=GO:0000723 feature_id[372].value <= threshold=1.608974039554596
Class: negative genes

Rules_189 passed counts:5
node_0: feature_name=GO:0042113 feature_id[0].value <= threshold=13.408552169799805
node_1: feature_name=GO:0007568 feature_id[534].value <= threshold=5.0313897132873535
node_2: feature_name=GO:0002705 feature_id[541].value <= threshold=3.200145721435547
node_3: feature_name=GO:1901525 feature_id[576].value <= threshold=0.4466460347175598
node_4: feature_name=GO:0048539 feature_id[319].value <= threshold=3.0399646759033203
node_5: feature_name=GO:0001910 feature_id[385].value <= threshold=3.7437864542007446
node_6: feature_name=GO:0043200 feature_id[706].value <= threshold=9.307284355163574
node_7: feature_name=GO:0001773 feature_id[308].value <= threshold=4.016931533813477
node_8: feature_name=GO:0090116 feature_id[97].value <= threshold=7.99645471572876
node_9: feature_name=GO:0019814 feature_id[189].value <= threshold=4.525782108306885
node_10: feature_name=GO:1902583 feature_id[215].value <= threshold=17.60310649871826
node_11: feature_name=GO:0045429 feature_id[737].value <= threshold=1.6133361458778381
node_12: feature_name=GO:0003720 feature_id[228].value <= threshold=5.519326210021973
node_13: feature_name=GO:0046006 feature_id[364].value <= threshold=4.9985432624816895
node_14: feature_name=GO:0070424 feature_id[254].value <= threshold=6.233297109603882
node_15: feature_name=GO:0009892 feature_id[320].value <= threshold=59.99180793762207
node_16: feature_name=GO:0007064 feature_id[527].value <= threshold=11.57377815246582
node_17: feature_name=GO:0005575 feature_id[17].value <= threshold=15.417460918426514
node_18: feature_name=GO:0043368 feature_id[31].value <= threshold=4.059496641159058
node_19: feature_name=GO:0005164 feature_id[163].value <= threshold=5.793607711791992
node_20: feature_name=GO:0042130 feature_id[16].value <= threshold=5.073179721832275
node_21: feature_name=GO:0010216 feature_id[282].value <= threshold=4.834584474563599
node_22: feature_name=GO:0009628 feature_id[553].value <= threshold=29.314892768859863
node_23: feature_name=GO:0045628 feature_id[749].value <= threshold=3.1001367568969727
node_24: feature_name=GO:0042288 feature_id[100].value <= threshold=5.174932956695557
node_25: feature_name=GO:0002329 feature_id[406].value <= threshold=2.348930835723877

node_26: feature_name=GO:0051246	feature_id[642].value <= threshold=0.6498909294605255
node_27: feature_name=GO:0042162	feature_id[355].value <= threshold=8.05103588104248
node_28: feature_name=hsa04668	feature_id[333].value <= threshold=5.614926338195801
node_29: feature_name=GO:0032200	feature_id[613].value <= threshold=28.735913276672363
node_30: feature_name=hsa04662	feature_id[334].value <= threshold=9.588344097137451
node_31: feature_name=GO:1901992	feature_id[105].value <= threshold=5.291427850723267
node_32: feature_name=GO:0072341	feature_id[144].value <= threshold=6.01465106010437
node_33: feature_name=GO:0047485	feature_id[121].value <= threshold=6.657836437225342
node_34: feature_name=GO:0090594	feature_id[738].value <= threshold=1.8673912286758423
node_35: feature_name=GO:0043525	feature_id[524].value <= threshold=2.881397008895874
node_36: feature_name=GO:0032504	feature_id[244].value <= threshold=17.22207546234131
node_37: feature_name=GO:0051348	feature_id[126].value <= threshold=5.933559417724609
node_38: feature_name=GO:0042522	feature_id[692].value <= threshold=1.78694087266922
node_39: feature_name=GO:0031100	feature_id[73].value <= threshold=3.2312101125717163
node_40: feature_name=GO:0042493	feature_id[149].value <= threshold=19.771170616149902
node_41: feature_name=GO:0001552	feature_id[366].value <= threshold=2.1711617708206177
node_42: feature_name=GO:0046483	feature_id[391].value <= threshold=320.9667053222656
node_43: feature_name=GO:0002439	feature_id[431].value <= threshold=1.832722783088684
node_44: feature_name=GO:0006555	feature_id[514].value <= threshold=5.321640968322754
node_45: feature_name=GO:0070424	feature_id[254].value <= threshold=3.321003556251526
node_46: feature_name=hsa05144	feature_id[751].value <= threshold=2.635499954223633
node_47: feature_name=GO:0032633	feature_id[627].value <= threshold=1.2592533230781555
node_48: feature_name=GO:0019814	feature_id[189].value <= threshold=2.1583125591278076
node_49: feature_name=GO:1901989	feature_id[30].value <= threshold=3.3150794506073
node_50: feature_name=GO:1903318	feature_id[196].value <= threshold=1.62252539396286
node_51: feature_name=GO:0060576	feature_id[204].value <= threshold=2.1097792387008667
node_52: feature_name=hsa05221	feature_id[349].value > threshold=0.8723124265670776
node_96: feature_name=hsa05221	feature_id[349].value > threshold=0.8756309151649475
node_98: feature_name=GO:0046794	feature_id[794].value <= threshold=6.585465431213379
node_99: feature_name=GO:0005035	feature_id[133].value <= threshold=0.6986835598945618
node_100: feature_name=hsa04640	feature_id[79].value <= threshold=1.458605408668518
node_101: feature_name=GO:0045945	feature_id[267].value > threshold=1.6378425359725952
node_103: feature_name=GO:0048145	feature_id[348].value > threshold=0.11509107798337936

Class: negative genes

Rules_190 passed counts:4
node_0: feature_name=GO:0042113 feature_id[0].value > threshold=13.408552169799805
node_1454: feature_name=GO:0050851 feature_id[29].value > threshold=9.650307655334473
node_1534: feature_name=GO:1902166 feature_id[301].value > threshold=0.303210511803627
node_1548: feature_name=GO:0035872 feature_id[659].value > threshold=35.09038162231445
node_1594: feature_name=hsa05210 feature_id[358].value > threshold=27.461133003234863
Class: positive genes

Rules_191 passed counts:4
node_0: feature_name=GO:0042113 feature_id[0].value > threshold=13.408552169799805
node_1454: feature_name=GO:0050851 feature_id[29].value > threshold=9.650307655334473
node_1534: feature_name=GO:1902166 feature_id[301].value > threshold=0.303210511803627
node_1548: feature_name=GO:0035872 feature_id[659].value <= threshold=35.09038162231445
node_1549: feature_name=GO:0032069 feature_id[614].value > threshold=3.441983938217163
node_1571: feature_name=GO:0050864 feature_id[288].value <= threshold=7.516624450683594
node_1572: feature_name=GO:0060574 feature_id[369].value <= threshold=2.804360032081604
Class: negative genes

Rules_192 passed counts:4
node_0: feature_name=GO:0042113 feature_id[0].value > threshold=13.408552169799805
node_1454: feature_name=GO:0050851 feature_id[29].value <= threshold=9.650307655334473
node_1455: feature_name=GO:0006304 feature_id[510].value > threshold=5.960662126541138
node_1531: feature_name=GO:2001251 feature_id[220].value > threshold=24.98499584197998
Class: negative genes

Rules_193 passed counts:4
node_0: feature_name=GO:0042113 feature_id[0].value > threshold=13.408552169799805
node_1454: feature_name=GO:0050851 feature_id[29].value <= threshold=9.650307655334473
node_1455: feature_name=GO:0006304 feature_id[510].value <= threshold=5.960662126541138
node_1456: feature_name=GO:0032673 feature_id[623].value > threshold=4.092082738876343
node_1510: feature_name=GO:0046898 feature_id[161].value <= threshold=2.2367727756500244
node_1511: feature_name=GO:0030183 feature_id[590].value > threshold=12.193635940551758
node_1521: feature_name=GO:0032504 feature_id[244].value <= threshold=0.8662047982215881
Class: positive genes

Rules_194	passed counts:4
node_0: feature_name=GO:0042113	feature_id[0].value > threshold=13.408552169799805
node_1454: feature_name=GO:0050851	feature_id[29].value <= threshold=9.650307655334473
node_1455: feature_name=GO:0006304	feature_id[510].value <= threshold=5.960662126541138
node_1456: feature_name=GO:0032673	feature_id[623].value <= threshold=4.092082738876343
node_1457: feature_name=GO:0002429	feature_id[748].value > threshold=4.626799821853638
node_1463: feature_name=GO:0015672	feature_id[573].value <= threshold=0.08226438239216805
node_1464: feature_name=GO:0038065	feature_id[670].value <= threshold=0.3765842020511627
node_1465: feature_name=GO:0002253	feature_id[397].value <= threshold=11.476495742797852
Class: positive genes	

Rules_195	passed counts:4
node_0: feature_name=GO:0042113	feature_id[0].value <= threshold=13.408552169799805
node_1: feature_name=GO:0007568	feature_id[534].value > threshold=5.0313897132873535
node_913: feature_name=GO:0032763	feature_id[629].value > threshold=0.31753237545490265
node_1195: feature_name=GO:0071301	feature_id[442].value > threshold=0.11534593254327774
node_1375: feature_name=GO:0046500	feature_id[171].value <= threshold=1.130434513092041
node_1376: feature_name=GO:0001782	feature_id[240].value <= threshold=6.639636993408203
node_1377: feature_name=GO:0043371	feature_id[707].value <= threshold=2.562113642692566
node_1378: feature_name=GO:0030291	feature_id[147].value > threshold=6.834916830062866
node_1424: feature_name=GO:0002439	feature_id[431].value > threshold=0.6129993498325348
node_1428: feature_name=GO:0009314	feature_id[296].value <= threshold=13.65259075164795
node_1429: feature_name=GO:0043011	feature_id[150].value > threshold=0.23608403280377388
Class: positive genes	

Rules_196	passed counts:4
node_0: feature_name=GO:0042113	feature_id[0].value <= threshold=13.408552169799805
node_1: feature_name=GO:0007568	feature_id[534].value > threshold=5.0313897132873535
node_913: feature_name=GO:0032763	feature_id[629].value > threshold=0.31753237545490265
node_1195: feature_name=GO:0071301	feature_id[442].value <= threshold=0.11534593254327774
node_1196: feature_name=GO:0046500	feature_id[171].value > threshold=0.6314916908740997
node_1340: feature_name=GO:0043627	feature_id[562].value <= threshold=25.17277240753174
node_1341: feature_name=GO:0010835	feature_id[560].value <= threshold=1.0601619482040405
node_1342: feature_name=GO:0016363	feature_id[90].value > threshold=6.534365892410278
Class: positive genes	

Rules_197 passed counts:4
node_0: feature_name=GO:0042113 feature_id[0].value <= threshold=13.408552169799805
node_1: feature_name=GO:0007568 feature_id[534].value > threshold=5.0313897132873535
node_913: feature_name=GO:0032763 feature_id[629].value > threshold=0.31753237545490265
node_1195: feature_name=GO:0071301 feature_id[442].value <= threshold=0.11534593254327774
node_1196: feature_name=GO:0046500 feature_id[171].value <= threshold=0.6314916908740997
node_1197: feature_name=GO:0030852 feature_id[22].value <= threshold=2.9768868684768677
node_1198: feature_name=hsa04672 feature_id[276].value <= threshold=8.06681227684021
node_1199: feature_name=GO:0001836 feature_id[450].value > threshold=0.09961023926734924
node_1201: feature_name=GO:0070230 feature_id[64].value <= threshold=3.3956379890441895
node_1202: feature_name=GO:0070102 feature_id[49].value <= threshold=4.805386543273926
node_1203: feature_name=GO:0045553 feature_id[736].value <= threshold=1.4970332980155945
node_1204: feature_name=GO:0051311 feature_id[58].value <= threshold=3.301889181137085
node_1205: feature_name=GO:0030291 feature_id[147].value <= threshold=8.156315326690674
node_1206: feature_name=GO:0045637 feature_id[241].value > threshold=0.018343428615480066
node_1208: feature_name=GO:0002890 feature_id[98].value <= threshold=2.767483353614807
node_1209: feature_name=GO:0046632 feature_id[248].value <= threshold=11.663254261016846
node_1210: feature_name=GO:0008588 feature_id[26].value <= threshold=1.391375720500946
node_1211: feature_name=GO:0010639 feature_id[564].value <= threshold=18.975126266479492
node_1212: feature_name=GO:0046685 feature_id[782].value <= threshold=5.153738260269165
node_1213: feature_name=GO:0038116 feature_id[668].value <= threshold=1.7859655618667603
node_1214: feature_name=GO:0046449 feature_id[763].value <= threshold=5.603115558624268
node_1215: feature_name=GO:0048539 feature_id[319].value > threshold=0.5478232800960541
node_1269: feature_name=GO:0072341 feature_id[144].value > threshold=1.3205850720405579
node_1287: feature_name=GO:0045346 feature_id[426].value <= threshold=3.6343181133270264
Class: positive genes

Rules_198 passed counts:4
node_0: feature_name=GO:0042113 feature_id[0].value <= threshold=13.408552169799805
node_1: feature_name=GO:0007568 feature_id[534].value > threshold=5.0313897132873535
node_913: feature_name=GO:0032763 feature_id[629].value > threshold=0.31753237545490265
node_1195: feature_name=GO:0071301 feature_id[442].value <= threshold=0.11534593254327774
node_1196: feature_name=GO:0046500 feature_id[171].value <= threshold=0.6314916908740997
node_1197: feature_name=GO:0030852 feature_id[22].value <= threshold=2.9768868684768677

node_1198: feature_name=hsa04672 feature_id[276].value <= threshold=8.06681227684021
node_1199: feature_name=GO:0001836 feature_id[450].value > threshold=0.09961023926734924
node_1201: feature_name=GO:0070230 feature_id[64].value <= threshold=3.3956379890441895
node_1202: feature_name=GO:0070102 feature_id[49].value <= threshold=4.805386543273926
node_1203: feature_name=GO:0045553 feature_id[736].value <= threshold=1.4970332980155945
node_1204: feature_name=GO:0051311 feature_id[58].value <= threshold=3.301889181137085
node_1205: feature_name=GO:0030291 feature_id[147].value <= threshold=8.156315326690674
node_1206: feature_name=GO:0045637 feature_id[241].value > threshold=0.01834342861548066
node_1208: feature_name=GO:0002890 feature_id[98].value <= threshold=2.767483353614807
node_1209: feature_name=GO:0046632 feature_id[248].value <= threshold=11.663254261016846
node_1210: feature_name=GO:0008588 feature_id[26].value <= threshold=1.391375720500946
node_1211: feature_name=GO:0010639 feature_id[564].value <= threshold=18.975126266479492
node_1212: feature_name=GO:0046685 feature_id[782].value <= threshold=5.153738260269165
node_1213: feature_name=GO:0038116 feature_id[668].value <= threshold=1.7859655618667603
node_1214: feature_name=GO:0046449 feature_id[763].value <= threshold=5.603115558624268
node_1215: feature_name=GO:0048539 feature_id[319].value <= threshold=0.5478232800960541
node_1216: feature_name=GO:0007089 feature_id[522].value <= threshold=0.9940084517002106
node_1217: feature_name=GO:0042287 feature_id[20].value > threshold=2.693045496940613
node_1263: feature_name=GO:0001816 feature_id[685].value <= threshold=18.00125026702881
Class: negative genes

Rules_199 passed counts:4
node_0: feature_name=GO:0042113 feature_id[0].value <= threshold=13.408552169799805
node_1: feature_name=GO:0007568 feature_id[534].value > threshold=5.0313897132873535
node_913: feature_name=GO:0032763 feature_id[629].value > threshold=0.31753237545490265
node_1195: feature_name=GO:0071301 feature_id[442].value <= threshold=0.11534593254327774
node_1196: feature_name=GO:0046500 feature_id[171].value <= threshold=0.6314916908740997
node_1197: feature_name=GO:0030852 feature_id[22].value <= threshold=2.9768868684768677
node_1198: feature_name=hsa04672 feature_id[276].value <= threshold=8.06681227684021
node_1199: feature_name=GO:0001836 feature_id[450].value > threshold=0.09961023926734924
node_1201: feature_name=GO:0070230 feature_id[64].value <= threshold=3.3956379890441895
node_1202: feature_name=GO:0070102 feature_id[49].value <= threshold=4.805386543273926
node_1203: feature_name=GO:0045553 feature_id[736].value <= threshold=1.4970332980155945
node_1204: feature_name=GO:0051311 feature_id[58].value <= threshold=3.301889181137085
node_1205: feature_name=GO:0030291 feature_id[147].value <= threshold=8.156315326690674

node_1206: feature_name=GO:0045637 feature_id[241].value > threshold=0.01834342861548066
node_1208: feature_name=GO:0002890 feature_id[98].value <= threshold=2.767483353614807
node_1209: feature_name=GO:0046632 feature_id[248].value <= threshold=11.663254261016846
node_1210: feature_name=GO:0008588 feature_id[26].value <= threshold=1.391375720500946
node_1211: feature_name=GO:0010639 feature_id[564].value <= threshold=18.975126266479492
node_1212: feature_name=GO:0046685 feature_id[782].value <= threshold=5.153738260269165
node_1213: feature_name=GO:0038116 feature_id[668].value <= threshold=1.7859655618667603
node_1214: feature_name=GO:0046449 feature_id[763].value <= threshold=5.603115558624268
node_1215: feature_name=GO:0048539 feature_id[319].value <= threshold=0.5478232800960541
node_1216: feature_name=GO:0007089 feature_id[522].value <= threshold=0.9940084517002106
node_1217: feature_name=GO:0042287 feature_id[20].value <= threshold=2.693045496940613
node_1218: feature_name=GO:0044446 feature_id[184].value <= threshold=113.0758171081543
node_1219: feature_name=GO:0098602 feature_id[702].value <= threshold=68.95328903198242
node_1220: feature_name=GO:0035726 feature_id[270].value <= threshold=1.7167096138000488
node_1221: feature_name=GO:1902564 feature_id[85].value <= threshold=2.8364468812942505
node_1222: feature_name=GO:0006979 feature_id[44].value > threshold=0.6869173645973206
node_1224: feature_name=GO:0044464 feature_id[56].value <= threshold=67.13260269165039
node_1225: feature_name=GO:0045629 feature_id[746].value <= threshold=2.3414241075515747
node_1226: feature_name=GO:0002903 feature_id[10].value <= threshold=2.715258240699768
node_1227: feature_name=GO:0043375 feature_id[717].value <= threshold=1.9525017738342285
node_1228: feature_name=GO:0032762 feature_id[630].value > threshold=0.29527929425239563
node_1232: feature_name=GO:0048569 feature_id[793].value <= threshold=3.419509768486023
node_1233: feature_name=GO:0045577 feature_id[109].value <= threshold=4.633661270141602
node_1234: feature_name=GO:0070245 feature_id[326].value > threshold=2.441925883293152
node_1240: feature_name=GO:0008283 feature_id[415].value > threshold=8.742806434631348
Class: negative genes

Rules_200 passed counts:4
node_0: feature_name=GO:0042113 feature_id[0].value <= threshold=13.408552169799805
node_1: feature_name=GO:0007568 feature_id[534].value > threshold=5.0313897132873535
node_913: feature_name=GO:0032763 feature_id[629].value > threshold=0.31753237545490265
node_1195: feature_name=GO:0071301 feature_id[442].value <= threshold=0.11534593254327774
node_1196: feature_name=GO:0046500 feature_id[171].value <= threshold=0.6314916908740997
node_1197: feature_name=GO:0030852 feature_id[22].value <= threshold=2.9768868684768677
node_1198: feature_name=hsa04672 feature_id[276].value <= threshold=8.06681227684021

node_1199: feature_name=GO:0001836 feature_id[450].value > threshold=0.09961023926734924
node_1201: feature_name=GO:0070230 feature_id[64].value <= threshold=3.3956379890441895
node_1202: feature_name=GO:0070102 feature_id[49].value <= threshold=4.805386543273926
node_1203: feature_name=GO:0045553 feature_id[736].value <= threshold=1.4970332980155945
node_1204: feature_name=GO:0051311 feature_id[58].value <= threshold=3.301889181137085
node_1205: feature_name=GO:0030291 feature_id[147].value <= threshold=8.156315326690674
node_1206: feature_name=GO:0045637 feature_id[241].value > threshold=0.01834342861548066
node_1208: feature_name=GO:0002890 feature_id[98].value <= threshold=2.767483353614807
node_1209: feature_name=GO:0046632 feature_id[248].value <= threshold=11.663254261016846
node_1210: feature_name=GO:0008588 feature_id[26].value <= threshold=1.391375720500946
node_1211: feature_name=GO:0010639 feature_id[564].value <= threshold=18.975126266479492
node_1212: feature_name=GO:0046685 feature_id[782].value <= threshold=5.153738260269165
node_1213: feature_name=GO:0038116 feature_id[668].value <= threshold=1.7859655618667603
node_1214: feature_name=GO:0046449 feature_id[763].value <= threshold=5.603115558624268
node_1215: feature_name=GO:0048539 feature_id[319].value <= threshold=0.5478232800960541
node_1216: feature_name=GO:0007089 feature_id[522].value <= threshold=0.9940084517002106
node_1217: feature_name=GO:0042287 feature_id[20].value <= threshold=2.693045496940613
node_1218: feature_name=GO:0044446 feature_id[184].value <= threshold=113.0758171081543
node_1219: feature_name=GO:0098602 feature_id[702].value <= threshold=68.95328903198242
node_1220: feature_name=GO:0035726 feature_id[270].value <= threshold=1.7167096138000488
node_1221: feature_name=GO:1902564 feature_id[85].value <= threshold=2.8364468812942505
node_1222: feature_name=GO:0006979 feature_id[44].value > threshold=0.6869173645973206
node_1224: feature_name=GO:0044464 feature_id[56].value <= threshold=67.13260269165039
node_1225: feature_name=GO:0045629 feature_id[746].value <= threshold=2.3414241075515747
node_1226: feature_name=GO:0002903 feature_id[10].value <= threshold=2.715258240699768
node_1227: feature_name=GO:0043375 feature_id[717].value <= threshold=1.9525017738342285
node_1228: feature_name=GO:0032762 feature_id[630].value > threshold=0.29527929425239563
node_1232: feature_name=GO:0048569 feature_id[793].value <= threshold=3.419509768486023
node_1233: feature_name=GO:0045577 feature_id[109].value <= threshold=4.633661270141602
node_1234: feature_name=GO:0070245 feature_id[326].value <= threshold=2.441925883293152
node_1235: feature_name=GO:0002864 feature_id[488].value > threshold=3.03904390335083
node_1237: feature_name=GO:0002260 feature_id[396].value > threshold=0.4489758163690567
Class: negative genes

Rules_201 passed counts:4

node_0: feature_name=GO:0042113 feature_id[0].value <= threshold=13.408552169799805
node_1: feature_name=GO:0007568 feature_id[534].value > threshold=5.0313897132873535
node_913: feature_name=GO:0032763 feature_id[629].value > threshold=0.31753237545490265
node_1195: feature_name=GO:0071301 feature_id[442].value <= threshold=0.11534593254327774
node_1196: feature_name=GO:0046500 feature_id[171].value <= threshold=0.6314916908740997
node_1197: feature_name=GO:0030852 feature_id[22].value <= threshold=2.9768868684768677
node_1198: feature_name=hsa04672 feature_id[276].value <= threshold=8.06681227684021
node_1199: feature_name=GO:0001836 feature_id[450].value <= threshold=0.09961023926734924
Class: positive genes

Rules_202 passed counts:4
node_0: feature_name=GO:0042113 feature_id[0].value <= threshold=13.408552169799805
node_1: feature_name=GO:0007568 feature_id[534].value > threshold=5.0313897132873535
node_913: feature_name=GO:0032763 feature_id[629].value <= threshold=0.31753237545490265
node_914: feature_name=GO:0097193 feature_id[167].value <= threshold=28.171168327331543
node_915: feature_name=GO:0002903 feature_id[10].value > threshold=1.0534588098526
node_1159: feature_name=GO:0070198 feature_id[665].value > threshold=0.16219981759786606
node_1165: feature_name=GO:0042287 feature_id[20].value <= threshold=0.915093183517456
node_1166: feature_name=GO:0046006 feature_id[364].value > threshold=0.12353959679603577
node_1168: feature_name=GO:0046634 feature_id[46].value > threshold=4.166545033454895
node_1178: feature_name=GO:0002517 feature_id[88].value <= threshold=1.9423661828041077
Class: positive genes

Rules_203 passed counts:4
node_0: feature_name=GO:0042113 feature_id[0].value <= threshold=13.408552169799805
node_1: feature_name=GO:0007568 feature_id[534].value > threshold=5.0313897132873535
node_913: feature_name=GO:0032763 feature_id[629].value <= threshold=0.31753237545490265
node_914: feature_name=GO:0097193 feature_id[167].value <= threshold=28.171168327331543
node_915: feature_name=GO:0002903 feature_id[10].value > threshold=1.0534588098526
node_1159: feature_name=GO:0070198 feature_id[665].value > threshold=0.16219981759786606
node_1165: feature_name=GO:0042287 feature_id[20].value <= threshold=0.915093183517456
node_1166: feature_name=GO:0046006 feature_id[364].value > threshold=0.12353959679603577
node_1168: feature_name=GO:0046634 feature_id[46].value <= threshold=4.166545033454895
node_1169: feature_name=GO:0044346 feature_id[724].value <= threshold=0.9571778774261475
node_1170: feature_name=GO:0031060 feature_id[160].value <= threshold=1.1418312191963196

Class: positive genes

Rules_204 passed counts:4
node_0: feature_name=GO:0042113 feature_id[0].value <= threshold=13.408552169799805
node_1: feature_name=GO:0007568 feature_id[534].value > threshold=5.0313897132873535
node_913: feature_name=GO:0032763 feature_id[629].value <= threshold=0.31753237545490265
node_914: feature_name=GO:0097193 feature_id[167].value <= threshold=28.171168327331543
node_915: feature_name=GO:0002903 feature_id[10].value > threshold=1.0534588098526
node_1159: feature_name=GO:0070198 feature_id[665].value > threshold=0.16219981759786606
node_1165: feature_name=GO:0042287 feature_id[20].value <= threshold=0.915093183517456
node_1166: feature_name=GO:0046006 feature_id[364].value <= threshold=0.12353959679603577
Class: positive genes

Rules_205 passed counts:4
node_0: feature_name=GO:0042113 feature_id[0].value <= threshold=13.408552169799805
node_1: feature_name=GO:0007568 feature_id[534].value > threshold=5.0313897132873535
node_913: feature_name=GO:0032763 feature_id[629].value <= threshold=0.31753237545490265
node_914: feature_name=GO:0097193 feature_id[167].value <= threshold=28.171168327331543
node_915: feature_name=GO:0002903 feature_id[10].value <= threshold=1.0534588098526
node_916: feature_name=GO:1904029 feature_id[208].value > threshold=23.852136611938477
node_1156: feature_name=hsa05203 feature_id[578].value <= threshold=32.333744049072266
Class: positive genes

Rules_206 passed counts:4
node_0: feature_name=GO:0042113 feature_id[0].value <= threshold=13.408552169799805
node_1: feature_name=GO:0007568 feature_id[534].value > threshold=5.0313897132873535
node_913: feature_name=GO:0032763 feature_id[629].value <= threshold=0.31753237545490265
node_914: feature_name=GO:0097193 feature_id[167].value <= threshold=28.171168327331543
node_915: feature_name=GO:0002903 feature_id[10].value <= threshold=1.0534588098526
node_916: feature_name=GO:1904029 feature_id[208].value <= threshold=23.852136611938477
node_917: feature_name=GO:0050897 feature_id[164].value > threshold=0.6870408356189728
node_1095: feature_name=GO:0072539 feature_id[68].value <= threshold=2.5667877197265625
node_1096: feature_name=GO:0046500 feature_id[171].value > threshold=3.195197820663452
Class: positive genes

Rules_207 passed counts:4
node_0: feature_name=GO:0042113 feature_id[0].value <= threshold=13.408552169799805
node_1: feature_name=GO:0007568 feature_id[534].value > threshold=5.0313897132873535
node_913: feature_name=GO:0032763 feature_id[629].value <= threshold=0.31753237545490265
node_914: feature_name=GO:0097193 feature_id[167].value <= threshold=28.171168327331543
node_915: feature_name=GO:0002903 feature_id[10].value <= threshold=1.0534588098526
node_916: feature_name=GO:1904029 feature_id[208].value <= threshold=23.852136611938477
node_917: feature_name=GO:0050897 feature_id[164].value > threshold=0.6870408356189728
node_1095: feature_name=GO:0072539 feature_id[68].value <= threshold=2.5667877197265625
node_1096: feature_name=GO:0046500 feature_id[171].value <= threshold=3.195197820663452
node_1097: feature_name=GO:0045861 feature_id[761].value > threshold=0.22614753991365433
node_1099: feature_name=GO:0043372 feature_id[242].value <= threshold=4.892506122589111
node_1100: feature_name=GO:0042162 feature_id[355].value > threshold=11.339290618896484
node_1148: feature_name=GO:0002643 feature_id[4].value > threshold=0.3193020150065422
Class: positive genes

Rules_208 passed counts:4
node_0: feature_name=GO:0042113 feature_id[0].value <= threshold=13.408552169799805
node_1: feature_name=GO:0007568 feature_id[534].value > threshold=5.0313897132873535
node_913: feature_name=GO:0032763 feature_id[629].value <= threshold=0.31753237545490265
node_914: feature_name=GO:0097193 feature_id[167].value <= threshold=28.171168327331543
node_915: feature_name=GO:0002903 feature_id[10].value <= threshold=1.0534588098526
node_916: feature_name=GO:1904029 feature_id[208].value <= threshold=23.852136611938477
node_917: feature_name=GO:0050897 feature_id[164].value <= threshold=0.6870408356189728
node_918: feature_name=GO:0006139 feature_id[474].value > threshold=1.3052097624921544e-07
node_920: feature_name=GO:0002821 feature_id[588].value <= threshold=13.81072187423706
node_921: feature_name=GO:0006298 feature_id[621].value <= threshold=24.078600883483887
node_922: feature_name=GO:0003908 feature_id[11].value <= threshold=1.7776933312416077
node_923: feature_name=GO:0030098 feature_id[273].value > threshold=0.0079949083738029
node_927: feature_name=GO:0006808 feature_id[513].value <= threshold=4.34592080116272
node_928: feature_name=GO:0071887 feature_id[283].value <= threshold=10.353787899017334
node_929: feature_name=GO:0038001 feature_id[28].value <= threshold=3.9469382762908936
node_930: feature_name=GO:0042287 feature_id[20].value <= threshold=3.6209195852279663
node_931: feature_name=GO:0003968 feature_id[47].value <= threshold=2.004227638244629
node_932: feature_name=GO:0002698 feature_id[395].value <= threshold=18.51447582244873

node_933: feature_name=GO:0044710 feature_id[719].value <= threshold=179.7340316772461
node_934: feature_name=GO:0007568 feature_id[534].value > threshold=5.035318374633789
node_936: feature_name=GO:0006216 feature_id[504].value <= threshold=2.2605666518211365
node_937: feature_name=GO:0048569 feature_id[793].value <= threshold=5.340231895446777
node_938: feature_name=GO:0001777 feature_id[380].value <= threshold=4.100832939147949
node_939: feature_name=GO:0007600 feature_id[122].value <= threshold=171.13383102416992
node_940: feature_name=GO:0001779 feature_id[378].value <= threshold=6.234851121902466
node_941: feature_name=GO:0030291 feature_id[147].value <= threshold=10.8051118850708
node_942: feature_name=GO:0048534 feature_id[790].value > threshold=0.017984486185014248
node_944: feature_name=GO:0070245 feature_id[326].value <= threshold=3.1282339096069336
node_945: feature_name=GO:0009086 feature_id[547].value <= threshold=0.2896959036588669
node_946: feature_name=GO:0001889 feature_id[387].value <= threshold=17.643128395080566
node_947: feature_name=GO:0048144 feature_id[192].value > threshold=0.20887330174446106
node_951: feature_name=GO:0023026 feature_id[69].value > threshold=3.403424024581909
node_1013: feature_name=GO:0034109 feature_id[601].value <= threshold=10.167142868041992
Class: negative genes

Rules_209 passed counts:4
node_0: feature_name=GO:0042113 feature_id[0].value <= threshold=13.408552169799805
node_1: feature_name=GO:0007568 feature_id[534].value <= threshold=5.0313897132873535
node_2: feature_name=GO:0002705 feature_id[541].value <= threshold=3.200145721435547
node_3: feature_name=GO:1901525 feature_id[576].value > threshold=0.4466460347175598
node_617: feature_name=GO:0005622 feature_id[233].value <= threshold=90.85730743408203
node_618: feature_name=GO:1903147 feature_id[200].value <= threshold=7.326428413391113
node_619: feature_name=GO:0003964 feature_id[317].value <= threshold=5.575642108917236
node_620: feature_name=GO:0046498 feature_id[203].value <= threshold=6.620223522186279
node_621: feature_name=GO:0002562 feature_id[25].value <= threshold=10.287704467773438
node_622: feature_name=GO:0006919 feature_id[246].value <= threshold=5.801334381103516
node_623: feature_name=GO:0001889 feature_id[387].value > threshold=2.679414987564087
node_785: feature_name=GO:0030887 feature_id[274].value > threshold=1.1379476189613342
node_831: feature_name=GO:0002274 feature_id[130].value <= threshold=1.7421051859855652
Class: positive genes

Rules_210 passed counts:4
node_0: feature_name=GO:0042113 feature_id[0].value <= threshold=13.408552169799805

node_1: feature_name=GO:0007568 feature_id[534].value <= threshold=5.0313897132873535
node_2: feature_name=GO:0002705 feature_id[541].value <= threshold=3.200145721435547
node_3: feature_name=GO:1901525 feature_id[576].value > threshold=0.4466460347175598
node_617: feature_name=GO:0005622 feature_id[233].value <= threshold=90.85730743408203
node_618: feature_name=GO:1903147 feature_id[200].value <= threshold=7.326428413391113
node_619: feature_name=GO:0003964 feature_id[317].value <= threshold=5.575642108917236
node_620: feature_name=GO:0046498 feature_id[203].value <= threshold=6.620223522186279
node_621: feature_name=GO:0002562 feature_id[25].value <= threshold=10.287704467773438
node_622: feature_name=GO:0006919 feature_id[246].value <= threshold=5.801334381103516
node_623: feature_name=GO:0001889 feature_id[387].value > threshold=2.679414987564087
node_785: feature_name=GO:0030887 feature_id[274].value <= threshold=1.1379476189613342
node_786: feature_name=GO:1902564 feature_id[85].value <= threshold=1.4637635350227356
node_787: feature_name=GO:0045402 feature_id[286].value <= threshold=1.5249269604682922
node_788: feature_name=GO:0034614 feature_id[655].value <= threshold=9.142115116119385
node_789: feature_name=GO:0000783 feature_id[191].value > threshold=1.5124736428260803
node_825: feature_name=GO:0033262 feature_id[643].value <= threshold=0.7662047147750854
Class: positive genes

Rules_211 passed counts:4
node_0: feature_name=GO:0042113 feature_id[0].value <= threshold=13.408552169799805
node_1: feature_name=GO:0007568 feature_id[534].value <= threshold=5.0313897132873535
node_2: feature_name=GO:0002705 feature_id[541].value <= threshold=3.200145721435547
node_3: feature_name=GO:1901525 feature_id[576].value > threshold=0.4466460347175598
node_617: feature_name=GO:0005622 feature_id[233].value <= threshold=90.85730743408203
node_618: feature_name=GO:1903147 feature_id[200].value <= threshold=7.326428413391113
node_619: feature_name=GO:0003964 feature_id[317].value <= threshold=5.575642108917236
node_620: feature_name=GO:0046498 feature_id[203].value <= threshold=6.620223522186279
node_621: feature_name=GO:0002562 feature_id[25].value <= threshold=10.287704467773438
node_622: feature_name=GO:0006919 feature_id[246].value <= threshold=5.801334381103516
node_623: feature_name=GO:0001889 feature_id[387].value <= threshold=2.679414987564087
node_624: feature_name=GO:0006555 feature_id[514].value <= threshold=7.819535493850708
node_625: feature_name=GO:0080134 feature_id[650].value <= threshold=38.91967582702637
node_626: feature_name=GO:0042127 feature_id[480].value <= threshold=37.72536659240723
node_627: feature_name=GO:2001251 feature_id[220].value <= threshold=32.564422607421875
node_628: feature_name=GO:0002829 feature_id[493].value <= threshold=2.444612741470337

node_629: feature_name=GO:0008588 feature_id[26].value <= threshold=2.3601274490356445
node_630: feature_name=GO:0033151 feature_id[7].value <= threshold=5.134376287460327
node_631: feature_name=GO:0048145 feature_id[348].value <= threshold=8.836549282073975
node_632: feature_name=GO:0030887 feature_id[274].value <= threshold=1.7000296115875244
node_633: feature_name=GO:0097190 feature_id[117].value <= threshold=33.02078819274902
node_634: feature_name=GO:0046685 feature_id[782].value <= threshold=8.040813446044922
node_635: feature_name=GO:0006346 feature_id[236].value <= threshold=6.384642839431763
node_636: feature_name=GO:0048537 feature_id[66].value <= threshold=3.1739262342453003
node_637: feature_name=GO:0070245 feature_id[326].value <= threshold=3.0491198301315308
node_638: feature_name=GO:0038065 feature_id[670].value > threshold=2.0590850114822388
node_762: feature_name=GO:0033554 feature_id[418].value <= threshold=3.435133218765259
Class: negative genes

Rules_212 passed counts:4
node_0: feature_name=GO:0042113 feature_id[0].value <= threshold=13.408552169799805
node_1: feature_name=GO:0007568 feature_id[534].value <= threshold=5.0313897132873535
node_2: feature_name=GO:0002705 feature_id[541].value <= threshold=3.200145721435547
node_3: feature_name=GO:1901525 feature_id[576].value > threshold=0.4466460347175598
node_617: feature_name=GO:0005622 feature_id[233].value <= threshold=90.85730743408203
node_618: feature_name=GO:1903147 feature_id[200].value <= threshold=7.326428413391113
node_619: feature_name=GO:0003964 feature_id[317].value <= threshold=5.575642108917236
node_620: feature_name=GO:0046498 feature_id[203].value <= threshold=6.620223522186279
node_621: feature_name=GO:0002562 feature_id[25].value <= threshold=10.287704467773438
node_622: feature_name=GO:0006919 feature_id[246].value <= threshold=5.801334381103516
node_623: feature_name=GO:0001889 feature_id[387].value <= threshold=2.679414987564087
node_624: feature_name=GO:0006555 feature_id[514].value <= threshold=7.819535493850708
node_625: feature_name=GO:0080134 feature_id[650].value <= threshold=38.91967582702637
node_626: feature_name=GO:0042127 feature_id[480].value <= threshold=37.72536659240723
node_627: feature_name=GO:2001251 feature_id[220].value <= threshold=32.564422607421875
node_628: feature_name=GO:0002829 feature_id[493].value <= threshold=2.444612741470337
node_629: feature_name=GO:0008588 feature_id[26].value <= threshold=2.3601274490356445
node_630: feature_name=GO:0033151 feature_id[7].value <= threshold=5.134376287460327
node_631: feature_name=GO:0048145 feature_id[348].value <= threshold=8.836549282073975
node_632: feature_name=GO:0030887 feature_id[274].value <= threshold=1.7000296115875244
node_633: feature_name=GO:0097190 feature_id[117].value <= threshold=33.02078819274902

node_634: feature_name=GO:0046685 feature_id[782].value <= threshold=8.040813446044922
node_635: feature_name=GO:0006346 feature_id[236].value <= threshold=6.384642839431763
node_636: feature_name=GO:0048537 feature_id[66].value <= threshold=3.1739262342453003
node_637: feature_name=GO:0070245 feature_id[326].value <= threshold=3.0491198301315308
node_638: feature_name=GO:0038065 feature_id[670].value <= threshold=2.0590850114822388
node_639: feature_name=GO:0005488 feature_id[187].value <= threshold=84.80076217651367
node_640: feature_name=GO:0060249 feature_id[390].value > threshold=0.07226398587226868
node_652: feature_name=GO:0006974 feature_id[516].value > threshold=5.17782768838515e-06
node_654: feature_name=hsa05210 feature_id[358].value <= threshold=18.730005264282227
node_655: feature_name=GO:0071850 feature_id[40].value <= threshold=5.475317001342773
node_656: feature_name=GO:1904029 feature_id[208].value <= threshold=26.575013160705566
node_657: feature_name=GO:0090594 feature_id[738].value <= threshold=3.000791072845459
node_658: feature_name=GO:0010948 feature_id[569].value > threshold=0.04386143572628498
node_666: feature_name=GO:0032764 feature_id[3].value <= threshold=2.5201677083969116
node_667: feature_name=GO:0023026 feature_id[69].value <= threshold=3.2760006189346313
node_668: feature_name=GO:0032464 feature_id[83].value <= threshold=2.5927021503448486
node_669: feature_name=GO:0071901 feature_id[407].value <= threshold=18.238012313842773
node_670: feature_name=GO:0015671 feature_id[103].value <= threshold=1.3113192915916443
node_671: feature_name=GO:0008340 feature_id[539].value <= threshold=4.860779762268066
node_672: feature_name=GO:0046006 feature_id[364].value <= threshold=2.4317692518234253
node_673: feature_name=GO:0042991 feature_id[684].value <= threshold=5.553565502166748
node_674: feature_name=GO:0002327 feature_id[408].value <= threshold=2.404940366744995
node_675: feature_name=GO:0001836 feature_id[450].value > threshold=8.647934436798096
node_707: feature_name=GO:0097202 feature_id[292].value > threshold=1.5505802929401398
Class: negative genes

Rules_213 passed counts:4
node_0: feature_name=GO:0042113 feature_id[0].value <= threshold=13.408552169799805
node_1: feature_name=GO:0007568 feature_id[534].value <= threshold=5.0313897132873535
node_2: feature_name=GO:0002705 feature_id[541].value <= threshold=3.200145721435547
node_3: feature_name=GO:1901525 feature_id[576].value > threshold=0.4466460347175598
node_617: feature_name=GO:0005622 feature_id[233].value <= threshold=90.85730743408203
node_618: feature_name=GO:1903147 feature_id[200].value <= threshold=7.326428413391113
node_619: feature_name=GO:0003964 feature_id[317].value <= threshold=5.575642108917236
node_620: feature_name=GO:0046498 feature_id[203].value <= threshold=6.620223522186279

node_621: feature_name=GO:0002562	feature_id[25].value <= threshold=10.287704467773438
node_622: feature_name=GO:0006919	feature_id[246].value <= threshold=5.801334381103516
node_623: feature_name=GO:0001889	feature_id[387].value <= threshold=2.679414987564087
node_624: feature_name=GO:0006555	feature_id[514].value <= threshold=7.819535493850708
node_625: feature_name=GO:0080134	feature_id[650].value <= threshold=38.91967582702637
node_626: feature_name=GO:0042127	feature_id[480].value <= threshold=37.72536659240723
node_627: feature_name=GO:2001251	feature_id[220].value <= threshold=32.564422607421875
node_628: feature_name=GO:0002829	feature_id[493].value <= threshold=2.444612741470337
node_629: feature_name=GO:0008588	feature_id[26].value <= threshold=2.3601274490356445
node_630: feature_name=GO:0033151	feature_id[7].value <= threshold=5.134376287460327
node_631: feature_name=GO:0048145	feature_id[348].value <= threshold=8.836549282073975
node_632: feature_name=GO:0030887	feature_id[274].value <= threshold=1.7000296115875244
node_633: feature_name=GO:0097190	feature_id[117].value <= threshold=33.02078819274902
node_634: feature_name=GO:0046685	feature_id[782].value <= threshold=8.040813446044922
node_635: feature_name=GO:0006346	feature_id[236].value <= threshold=6.384642839431763
node_636: feature_name=GO:0048537	feature_id[66].value <= threshold=3.1739262342453003
node_637: feature_name=GO:0070245	feature_id[326].value <= threshold=3.0491198301315308
node_638: feature_name=GO:0038065	feature_id[670].value <= threshold=2.0590850114822388
node_639: feature_name=GO:0005488	feature_id[187].value <= threshold=84.80076217651367
node_640: feature_name=GO:0060249	feature_id[390].value > threshold=0.07226398587226868
node_652: feature_name=GO:0006974	feature_id[516].value > threshold=5.17782768838515e-06
node_654: feature_name=hsa05210	feature_id[358].value <= threshold=18.730005264282227
node_655: feature_name=GO:0071850	feature_id[40].value <= threshold=5.475317001342773
node_656: feature_name=GO:1904029	feature_id[208].value <= threshold=26.575013160705566
node_657: feature_name=GO:0090594	feature_id[738].value <= threshold=3.000791072845459
node_658: feature_name=GO:0010948	feature_id[569].value > threshold=0.04386143572628498
node_666: feature_name=GO:0032764	feature_id[3].value <= threshold=2.5201677083969116
node_667: feature_name=GO:0023026	feature_id[69].value <= threshold=3.2760006189346313
node_668: feature_name=GO:0032464	feature_id[83].value <= threshold=2.5927021503448486
node_669: feature_name=GO:0071901	feature_id[407].value <= threshold=18.238012313842773
node_670: feature_name=GO:0015671	feature_id[103].value <= threshold=1.3113192915916443
node_671: feature_name=GO:0008340	feature_id[539].value <= threshold=4.860779762268066
node_672: feature_name=GO:0046006	feature_id[364].value <= threshold=2.4317692518234253
node_673: feature_name=GO:0042991	feature_id[684].value <= threshold=5.553565502166748
node_674: feature_name=GO:0002327	feature_id[408].value <= threshold=2.404940366744995

node_675: feature_name=GO:0001836 feature_id[450].value <= threshold=8.647934436798096
node_676: feature_name=GO:0048539 feature_id[319].value > threshold=1.6308802962303162
node_704: feature_name=GO:0044446 feature_id[184].value <= threshold=19.456976175308228
Class: negative genes

Rules_214 passed counts:4
node_0: feature_name=GO:0042113 feature_id[0].value <= threshold=13.408552169799805
node_1: feature_name=GO:0007568 feature_id[534].value <= threshold=5.0313897132873535
node_2: feature_name=GO:0002705 feature_id[541].value <= threshold=3.200145721435547
node_3: feature_name=GO:1901525 feature_id[576].value > threshold=0.4466460347175598
node_617: feature_name=GO:0005622 feature_id[233].value <= threshold=90.85730743408203
node_618: feature_name=GO:1903147 feature_id[200].value <= threshold=7.326428413391113
node_619: feature_name=GO:0003964 feature_id[317].value <= threshold=5.575642108917236
node_620: feature_name=GO:0046498 feature_id[203].value <= threshold=6.620223522186279
node_621: feature_name=GO:0002562 feature_id[25].value <= threshold=10.287704467773438
node_622: feature_name=GO:0006919 feature_id[246].value <= threshold=5.801334381103516
node_623: feature_name=GO:0001889 feature_id[387].value <= threshold=2.679414987564087
node_624: feature_name=GO:0006555 feature_id[514].value <= threshold=7.819535493850708
node_625: feature_name=GO:0080134 feature_id[650].value <= threshold=38.91967582702637
node_626: feature_name=GO:0042127 feature_id[480].value <= threshold=37.72536659240723
node_627: feature_name=GO:2001251 feature_id[220].value <= threshold=32.564422607421875
node_628: feature_name=GO:0002829 feature_id[493].value <= threshold=2.444612741470337
node_629: feature_name=GO:0008588 feature_id[26].value <= threshold=2.3601274490356445
node_630: feature_name=GO:0033151 feature_id[7].value <= threshold=5.134376287460327
node_631: feature_name=GO:0048145 feature_id[348].value <= threshold=8.836549282073975
node_632: feature_name=GO:0030887 feature_id[274].value <= threshold=1.7000296115875244
node_633: feature_name=GO:0097190 feature_id[117].value <= threshold=33.02078819274902
node_634: feature_name=GO:0046685 feature_id[782].value <= threshold=8.040813446044922
node_635: feature_name=GO:0006346 feature_id[236].value <= threshold=6.384642839431763
node_636: feature_name=GO:0048537 feature_id[66].value <= threshold=3.1739262342453003
node_637: feature_name=GO:0070245 feature_id[326].value <= threshold=3.0491198301315308
node_638: feature_name=GO:0038065 feature_id[670].value <= threshold=2.0590850114822388
node_639: feature_name=GO:0005488 feature_id[187].value <= threshold=84.80076217651367
node_640: feature_name=GO:0060249 feature_id[390].value > threshold=0.07226398587226868
node_652: feature_name=GO:0006974 feature_id[516].value > threshold=5.17782768838515e-06

node_654: feature_name=hsa05210 feature_id[358].value <= threshold=18.730005264282227
node_655: feature_name=GO:0071850 feature_id[40].value <= threshold=5.475317001342773
node_656: feature_name=GO:1904029 feature_id[208].value <= threshold=26.575013160705566
node_657: feature_name=GO:0090594 feature_id[738].value <= threshold=3.000791072845459
node_658: feature_name=GO:0010948 feature_id[569].value > threshold=0.04386143572628498
node_666: feature_name=GO:0032764 feature_id[3].value <= threshold=2.5201677083969116
node_667: feature_name=GO:0023026 feature_id[69].value <= threshold=3.2760006189346313
node_668: feature_name=GO:0032464 feature_id[83].value <= threshold=2.5927021503448486
node_669: feature_name=GO:0071901 feature_id[407].value <= threshold=18.238012313842773
node_670: feature_name=GO:0015671 feature_id[103].value <= threshold=1.3113192915916443
node_671: feature_name=GO:0008340 feature_id[539].value <= threshold=4.860779762268066
node_672: feature_name=GO:0046006 feature_id[364].value <= threshold=2.4317692518234253
node_673: feature_name=GO:0042991 feature_id[684].value <= threshold=5.553565502166748
node_674: feature_name=GO:0002327 feature_id[408].value <= threshold=2.404940366744995
node_675: feature_name=GO:0001836 feature_id[450].value <= threshold=8.647934436798096
node_676: feature_name=GO:0048539 feature_id[319].value <= threshold=1.6308802962303162
node_677: feature_name=GO:0022408 feature_id[279].value > threshold=4.064727783203125
node_701: feature_name=GO:0033077 feature_id[134].value <= threshold=2.082643210887909
Class: negative genes

Rules_215 passed counts:4
node_0: feature_name=GO:0042113 feature_id[0].value <= threshold=13.408552169799805
node_1: feature_name=GO:0007568 feature_id[534].value <= threshold=5.0313897132873535
node_2: feature_name=GO:0002705 feature_id[541].value <= threshold=3.200145721435547
node_3: feature_name=GO:1901525 feature_id[576].value > threshold=0.4466460347175598
node_617: feature_name=GO:0005622 feature_id[233].value <= threshold=90.85730743408203
node_618: feature_name=GO:1903147 feature_id[200].value <= threshold=7.326428413391113
node_619: feature_name=GO:0003964 feature_id[317].value <= threshold=5.575642108917236
node_620: feature_name=GO:0046498 feature_id[203].value <= threshold=6.620223522186279
node_621: feature_name=GO:0002562 feature_id[25].value <= threshold=10.287704467773438
node_622: feature_name=GO:0006919 feature_id[246].value <= threshold=5.801334381103516
node_623: feature_name=GO:0001889 feature_id[387].value <= threshold=2.679414987564087
node_624: feature_name=GO:0006555 feature_id[514].value <= threshold=7.819535493850708
node_625: feature_name=GO:0080134 feature_id[650].value <= threshold=38.91967582702637
node_626: feature_name=GO:0042127 feature_id[480].value <= threshold=37.72536659240723

node_627: feature_name=GO:2001251 feature_id[220].value <= threshold=32.564422607421875
node_628: feature_name=GO:0002829 feature_id[493].value <= threshold=2.444612741470337
node_629: feature_name=GO:0008588 feature_id[26].value <= threshold=2.3601274490356445
node_630: feature_name=GO:0033151 feature_id[7].value <= threshold=5.134376287460327
node_631: feature_name=GO:0048145 feature_id[348].value <= threshold=8.836549282073975
node_632: feature_name=GO:0030887 feature_id[274].value <= threshold=1.7000296115875244
node_633: feature_name=GO:0097190 feature_id[117].value <= threshold=33.02078819274902
node_634: feature_name=GO:0046685 feature_id[782].value <= threshold=8.040813446044922
node_635: feature_name=GO:0006346 feature_id[236].value <= threshold=6.384642839431763
node_636: feature_name=GO:0048537 feature_id[66].value <= threshold=3.1739262342453003
node_637: feature_name=GO:0070245 feature_id[326].value <= threshold=3.0491198301315308
node_638: feature_name=GO:0038065 feature_id[670].value <= threshold=2.0590850114822388
node_639: feature_name=GO:0005488 feature_id[187].value <= threshold=84.80076217651367
node_640: feature_name=GO:0060249 feature_id[390].value <= threshold=0.07226398587226868
node_641: feature_name=GO:0002683 feature_id[456].value > threshold=0.7958214282989502
node_647: feature_name=GO:0045638 feature_id[94].value <= threshold=0.7937153577804565
node_648: feature_name=GO:0007600 feature_id[122].value > threshold=0.009634195128455758
Class: negative genes

Rules_216 passed counts:4
node_0: feature_name=GO:0042113 feature_id[0].value <= threshold=13.408552169799805
node_1: feature_name=GO:0007568 feature_id[534].value <= threshold=5.0313897132873535
node_2: feature_name=GO:0002705 feature_id[541].value <= threshold=3.200145721435547
node_3: feature_name=GO:1901525 feature_id[576].value > threshold=0.4466460347175598
node_617: feature_name=GO:0005622 feature_id[233].value <= threshold=90.85730743408203
node_618: feature_name=GO:1903147 feature_id[200].value <= threshold=7.326428413391113
node_619: feature_name=GO:0003964 feature_id[317].value <= threshold=5.575642108917236
node_620: feature_name=GO:0046498 feature_id[203].value <= threshold=6.620223522186279
node_621: feature_name=GO:0002562 feature_id[25].value <= threshold=10.287704467773438
node_622: feature_name=GO:0006919 feature_id[246].value <= threshold=5.801334381103516
node_623: feature_name=GO:0001889 feature_id[387].value <= threshold=2.679414987564087
node_624: feature_name=GO:0006555 feature_id[514].value <= threshold=7.819535493850708
node_625: feature_name=GO:0080134 feature_id[650].value <= threshold=38.91967582702637
node_626: feature_name=GO:0042127 feature_id[480].value <= threshold=37.72536659240723
node_627: feature_name=GO:2001251 feature_id[220].value <= threshold=32.564422607421875

node_628: feature_name=GO:0002829 feature_id[493].value <= threshold=2.444612741470337
node_629: feature_name=GO:0008588 feature_id[26].value <= threshold=2.3601274490356445
node_630: feature_name=GO:0033151 feature_id[7].value <= threshold=5.134376287460327
node_631: feature_name=GO:0048145 feature_id[348].value <= threshold=8.836549282073975
node_632: feature_name=GO:0030887 feature_id[274].value <= threshold=1.7000296115875244
node_633: feature_name=GO:0097190 feature_id[117].value <= threshold=33.02078819274902
node_634: feature_name=GO:0046685 feature_id[782].value <= threshold=8.040813446044922
node_635: feature_name=GO:0006346 feature_id[236].value <= threshold=6.384642839431763
node_636: feature_name=GO:0048537 feature_id[66].value <= threshold=3.1739262342453003
node_637: feature_name=GO:0070245 feature_id[326].value <= threshold=3.0491198301315308
node_638: feature_name=GO:0038065 feature_id[670].value <= threshold=2.0590850114822388
node_639: feature_name=GO:0005488 feature_id[187].value <= threshold=84.80076217651367
node_640: feature_name=GO:0060249 feature_id[390].value <= threshold=0.07226398587226868
node_641: feature_name=GO:0002683 feature_id[456].value <= threshold=0.7958214282989502
node_642: feature_name=GO:0006346 feature_id[236].value > threshold=0.4950565919280052
node_644: feature_name=GO:2001020 feature_id[275].value <= threshold=0.35050614178180695
Class: negative genes

Rules_217 passed counts:4
node_0: feature_name=GO:0042113 feature_id[0].value <= threshold=13.408552169799805
node_1: feature_name=GO:0007568 feature_id[534].value <= threshold=5.0313897132873535
node_2: feature_name=GO:0002705 feature_id[541].value <= threshold=3.200145721435547
node_3: feature_name=GO:1901525 feature_id[576].value <= threshold=0.4466460347175598
node_4: feature_name=GO:0048539 feature_id[319].value <= threshold=3.0399646759033203
node_5: feature_name=GO:0001910 feature_id[385].value <= threshold=3.7437864542007446
node_6: feature_name=GO:0043200 feature_id[706].value <= threshold=9.307284355163574
node_7: feature_name=GO:0001773 feature_id[308].value <= threshold=4.016931533813477
node_8: feature_name=GO:0090116 feature_id[97].value <= threshold=7.99645471572876
node_9: feature_name=GO:0019814 feature_id[189].value <= threshold=4.525782108306885
node_10: feature_name=GO:1902583 feature_id[215].value <= threshold=17.60310649871826
node_11: feature_name=GO:0045429 feature_id[737].value > threshold=1.6133361458778381
node_477: feature_name=GO:0036037 feature_id[445].value > threshold=1.7181594371795654
node_595: feature_name=GO:0015671 feature_id[103].value > threshold=0.0885394886136055
Class: positive genes

Rules_218	passed counts:4
node_0: feature_name=GO:0042113	feature_id[0].value <= threshold=13.408552169799805
node_1: feature_name=GO:0007568	feature_id[534].value <= threshold=5.0313897132873535
node_2: feature_name=GO:0002705	feature_id[541].value <= threshold=3.200145721435547
node_3: feature_name=GO:1901525	feature_id[576].value <= threshold=0.4466460347175598
node_4: feature_name=GO:0048539	feature_id[319].value <= threshold=3.0399646759033203
node_5: feature_name=GO:0001910	feature_id[385].value <= threshold=3.7437864542007446
node_6: feature_name=GO:0043200	feature_id[706].value <= threshold=9.307284355163574
node_7: feature_name=GO:0001773	feature_id[308].value <= threshold=4.016931533813477
node_8: feature_name=GO:0090116	feature_id[97].value <= threshold=7.99645471572876
node_9: feature_name=GO:0019814	feature_id[189].value <= threshold=4.525782108306885
node_10: feature_name=GO:1902583	feature_id[215].value <= threshold=17.60310649871826
node_11: feature_name=GO:0045429	feature_id[737].value > threshold=1.6133361458778381
node_477: feature_name=GO:0036037	feature_id[445].value <= threshold=1.7181594371795654
node_478: feature_name=GO:0006244	feature_id[503].value > threshold=0.951388418674469
node_588: feature_name=GO:0010556	feature_id[784].value <= threshold=0.002115685958415270
Class: positive genes	

Rules_219	passed counts:4
node_0: feature_name=GO:0042113	feature_id[0].value <= threshold=13.408552169799805
node_1: feature_name=GO:0007568	feature_id[534].value <= threshold=5.0313897132873535
node_2: feature_name=GO:0002705	feature_id[541].value <= threshold=3.200145721435547
node_3: feature_name=GO:1901525	feature_id[576].value <= threshold=0.4466460347175598
node_4: feature_name=GO:0048539	feature_id[319].value <= threshold=3.0399646759033203
node_5: feature_name=GO:0001910	feature_id[385].value <= threshold=3.7437864542007446
node_6: feature_name=GO:0043200	feature_id[706].value <= threshold=9.307284355163574
node_7: feature_name=GO:0001773	feature_id[308].value <= threshold=4.016931533813477
node_8: feature_name=GO:0090116	feature_id[97].value <= threshold=7.99645471572876
node_9: feature_name=GO:0019814	feature_id[189].value <= threshold=4.525782108306885
node_10: feature_name=GO:1902583	feature_id[215].value <= threshold=17.60310649871826
node_11: feature_name=GO:0045429	feature_id[737].value <= threshold=1.6133361458778381
node_12: feature_name=GO:0003720	feature_id[228].value <= threshold=5.519326210021973
node_13: feature_name=GO:0046006	feature_id[364].value <= threshold=4.9985432624816895
node_14: feature_name=GO:0070424	feature_id[254].value <= threshold=6.233297109603882
node_15: feature_name=GO:0009892	feature_id[320].value <= threshold=59.99180793762207

node_16: feature_name=GO:0007064 feature_id[527].value <= threshold=11.57377815246582
node_17: feature_name=GO:0005575 feature_id[17].value <= threshold=15.417460918426514
node_18: feature_name=GO:0043368 feature_id[31].value <= threshold=4.059496641159058
node_19: feature_name=GO:0005164 feature_id[163].value <= threshold=5.793607711791992
node_20: feature_name=GO:0042130 feature_id[16].value <= threshold=5.073179721832275
node_21: feature_name=GO:0010216 feature_id[282].value <= threshold=4.834584474563599
node_22: feature_name=GO:0009628 feature_id[553].value <= threshold=29.314892768859863
node_23: feature_name=GO:0045628 feature_id[749].value <= threshold=3.1001367568969727
node_24: feature_name=GO:0042288 feature_id[100].value <= threshold=5.174932956695557
node_25: feature_name=GO:0002329 feature_id[406].value <= threshold=2.348930835723877
node_26: feature_name=GO:0051246 feature_id[642].value > threshold=0.6498909294605255
node_194: feature_name=GO:0009164 feature_id[546].value <= threshold=17.11970329284668
node_195: feature_name=GO:0001909 feature_id[386].value <= threshold=7.292566299438477
node_196: feature_name=GO:0003908 feature_id[11].value <= threshold=1.887226164340973
node_197: feature_name=GO:0009164 feature_id[546].value > threshold=0.26642198860645294
node_359: feature_name=GO:0009164 feature_id[546].value > threshold=0.2696942090988159
node_361: feature_name=hsa05202 feature_id[50].value <= threshold=14.989679336547852
node_362: feature_name=GO:0001775 feature_id[505].value <= threshold=15.726949691772461
node_363: feature_name=GO:0002634 feature_id[449].value <= threshold=2.825531005859375
node_364: feature_name=GO:0010038 feature_id[457].value <= threshold=9.901018619537354
node_365: feature_name=GO:0044238 feature_id[295].value <= threshold=139.80535888671875
node_366: feature_name=GO:0051246 feature_id[642].value > threshold=0.6527281999588013
node_368: feature_name=GO:0044092 feature_id[432].value <= threshold=11.084641933441162
node_369: feature_name=GO:0042162 feature_id[355].value <= threshold=9.683155536651611
node_370: feature_name=GO:0045945 feature_id[267].value <= threshold=3.6181851625442505
node_371: feature_name=GO:0036296 feature_id[252].value <= threshold=3.214281916618347
node_372: feature_name=GO:0097506 feature_id[51].value > threshold=2.9644681215286255
node_430: feature_name=GO:0016444 feature_id[53].value > threshold=0.6698829531669617
Class: negative genes

Rules_220 passed counts:4
node_0: feature_name=GO:0042113 feature_id[0].value <= threshold=13.408552169799805
node_1: feature_name=GO:0007568 feature_id[534].value <= threshold=5.0313897132873535
node_2: feature_name=GO:0002705 feature_id[541].value <= threshold=3.200145721435547
node_3: feature_name=GO:1901525 feature_id[576].value <= threshold=0.4466460347175598

node_4: feature_name=GO:0048539	feature_id[319].value <= threshold=3.0399646759033203
node_5: feature_name=GO:0001910	feature_id[385].value <= threshold=3.7437864542007446
node_6: feature_name=GO:0043200	feature_id[706].value <= threshold=9.307284355163574
node_7: feature_name=GO:0001773	feature_id[308].value <= threshold=4.016931533813477
node_8: feature_name=GO:0090116	feature_id[97].value <= threshold=7.99645471572876
node_9: feature_name=GO:0019814	feature_id[189].value <= threshold=4.525782108306885
node_10: feature_name=GO:1902583	feature_id[215].value <= threshold=17.60310649871826
node_11: feature_name=GO:0045429	feature_id[737].value <= threshold=1.6133361458778381
node_12: feature_name=GO:0003720	feature_id[228].value <= threshold=5.519326210021973
node_13: feature_name=GO:0046006	feature_id[364].value <= threshold=4.9985432624816895
node_14: feature_name=GO:0070424	feature_id[254].value <= threshold=6.233297109603882
node_15: feature_name=GO:0009892	feature_id[320].value <= threshold=59.99180793762207
node_16: feature_name=GO:0007064	feature_id[527].value <= threshold=11.57377815246582
node_17: feature_name=GO:0005575	feature_id[17].value <= threshold=15.417460918426514
node_18: feature_name=GO:0043368	feature_id[31].value <= threshold=4.059496641159058
node_19: feature_name=GO:0005164	feature_id[163].value <= threshold=5.793607711791992
node_20: feature_name=GO:0042130	feature_id[16].value <= threshold=5.073179721832275
node_21: feature_name=GO:0010216	feature_id[282].value <= threshold=4.834584474563599
node_22: feature_name=GO:0009628	feature_id[553].value <= threshold=29.314892768859863
node_23: feature_name=GO:0045628	feature_id[749].value <= threshold=3.1001367568969727
node_24: feature_name=GO:0042288	feature_id[100].value <= threshold=5.174932956695557
node_25: feature_name=GO:0002329	feature_id[406].value <= threshold=2.348930835723877
node_26: feature_name=GO:0051246	feature_id[642].value > threshold=0.6498909294605255
node_194: feature_name=GO:0009164	feature_id[546].value <= threshold=17.11970329284668
node_195: feature_name=GO:0001909	feature_id[386].value <= threshold=7.292566299438477
node_196: feature_name=GO:0003908	feature_id[11].value <= threshold=1.887226164340973
node_197: feature_name=GO:0009164	feature_id[546].value > threshold=0.26642198860645294
node_359: feature_name=GO:0009164	feature_id[546].value > threshold=0.2696942090988159
node_361: feature_name=hsa05202	feature_id[50].value <= threshold=14.989679336547852
node_362: feature_name=GO:0001775	feature_id[505].value <= threshold=15.726949691772461
node_363: feature_name=GO:0002634	feature_id[449].value <= threshold=2.825531005859375
node_364: feature_name=GO:0010038	feature_id[457].value <= threshold=9.901018619537354
node_365: feature_name=GO:0044238	feature_id[295].value <= threshold=139.80535888671875
node_366: feature_name=GO:0051246	feature_id[642].value > threshold=0.6527281999588013
node_368: feature_name=GO:0044092	feature_id[432].value <= threshold=11.084641933441162

node_369: feature_name=GO:0042162 feature_id[355].value <= threshold=9.683155536651611
node_370: feature_name=GO:0045945 feature_id[267].value <= threshold=3.6181851625442505
node_371: feature_name=GO:0036296 feature_id[252].value <= threshold=3.214281916618347
node_372: feature_name=GO:0097506 feature_id[51].value <= threshold=2.9644681215286255
node_373: feature_name=GO:0019740 feature_id[581].value > threshold=2.2853575944900513
node_427: feature_name=hsa04933 feature_id[744].value > threshold=0.9997820556163788
Class: negative genes

Rules_221 passed counts:4
node_0: feature_name=GO:0042113 feature_id[0].value <= threshold=13.408552169799805
node_1: feature_name=GO:0007568 feature_id[534].value <= threshold=5.0313897132873535
node_2: feature_name=GO:0002705 feature_id[541].value <= threshold=3.200145721435547
node_3: feature_name=GO:1901525 feature_id[576].value <= threshold=0.4466460347175598
node_4: feature_name=GO:0048539 feature_id[319].value <= threshold=3.0399646759033203
node_5: feature_name=GO:0001910 feature_id[385].value <= threshold=3.7437864542007446
node_6: feature_name=GO:0043200 feature_id[706].value <= threshold=9.307284355163574
node_7: feature_name=GO:0001773 feature_id[308].value <= threshold=4.016931533813477
node_8: feature_name=GO:0090116 feature_id[97].value <= threshold=7.99645471572876
node_9: feature_name=GO:0019814 feature_id[189].value <= threshold=4.525782108306885
node_10: feature_name=GO:1902583 feature_id[215].value <= threshold=17.60310649871826
node_11: feature_name=GO:0045429 feature_id[737].value <= threshold=1.6133361458778381
node_12: feature_name=GO:0003720 feature_id[228].value <= threshold=5.519326210021973
node_13: feature_name=GO:0046006 feature_id[364].value <= threshold=4.9985432624816895
node_14: feature_name=GO:0070424 feature_id[254].value <= threshold=6.233297109603882
node_15: feature_name=GO:0009892 feature_id[320].value <= threshold=59.99180793762207
node_16: feature_name=GO:0007064 feature_id[527].value <= threshold=11.57377815246582
node_17: feature_name=GO:0005575 feature_id[17].value <= threshold=15.417460918426514
node_18: feature_name=GO:0043368 feature_id[31].value <= threshold=4.059496641159058
node_19: feature_name=GO:0005164 feature_id[163].value <= threshold=5.793607711791992
node_20: feature_name=GO:0042130 feature_id[16].value <= threshold=5.073179721832275
node_21: feature_name=GO:0010216 feature_id[282].value <= threshold=4.834584474563599
node_22: feature_name=GO:0009628 feature_id[553].value <= threshold=29.314892768859863
node_23: feature_name=GO:0045628 feature_id[749].value <= threshold=3.1001367568969727
node_24: feature_name=GO:0042288 feature_id[100].value <= threshold=5.174932956695557
node_25: feature_name=GO:0002329 feature_id[406].value <= threshold=2.348930835723877

node_26: feature_name=GO:0051246 feature_id[642].value > threshold=0.6498909294605255
node_194: feature_name=GO:0009164 feature_id[546].value <= threshold=17.11970329284668
node_195: feature_name=GO:0001909 feature_id[386].value <= threshold=7.292566299438477
node_196: feature_name=GO:0003908 feature_id[11].value <= threshold=1.887226164340973
node_197: feature_name=GO:0009164 feature_id[546].value <= threshold=0.26642198860645294
node_198: feature_name=GO:2001242 feature_id[24].value <= threshold=9.207026481628418
node_199: feature_name=GO:0051246 feature_id[642].value > threshold=0.6504445374011993
node_203: feature_name=GO:0032461 feature_id[608].value <= threshold=2.607542634010315
node_204: feature_name=GO:0007584 feature_id[529].value <= threshold=16.099515914916992
node_205: feature_name=hsa05221 feature_id[349].value <= threshold=9.359850406646729
node_206: feature_name=GO:0002636 feature_id[447].value <= threshold=1.615447759628296
node_207: feature_name=GO:0033993 feature_id[421].value > threshold=2.96540611088858e-05
node_217: feature_name=GO:0002331 feature_id[404].value <= threshold=2.1883513927459717
node_218: feature_name=GO:0046498 feature_id[203].value <= threshold=4.085246205329895
node_219: feature_name=GO:0002832 feature_id[491].value > threshold=3.0228612422943115
node_325: feature_name=GO:0002715 feature_id[484].value <= threshold=0.8928120732307434
Class: negative genes

Rules_222 passed counts:4
node_0: feature_name=GO:0042113 feature_id[0].value <= threshold=13.408552169799805
node_1: feature_name=GO:0007568 feature_id[534].value <= threshold=5.0313897132873535
node_2: feature_name=GO:0002705 feature_id[541].value <= threshold=3.200145721435547
node_3: feature_name=GO:1901525 feature_id[576].value <= threshold=0.4466460347175598
node_4: feature_name=GO:0048539 feature_id[319].value <= threshold=3.0399646759033203
node_5: feature_name=GO:0001910 feature_id[385].value <= threshold=3.7437864542007446
node_6: feature_name=GO:0043200 feature_id[706].value <= threshold=9.307284355163574
node_7: feature_name=GO:0001773 feature_id[308].value <= threshold=4.016931533813477
node_8: feature_name=GO:0090116 feature_id[97].value <= threshold=7.99645471572876
node_9: feature_name=GO:0019814 feature_id[189].value <= threshold=4.525782108306885
node_10: feature_name=GO:1902583 feature_id[215].value <= threshold=17.60310649871826
node_11: feature_name=GO:0045429 feature_id[737].value <= threshold=1.6133361458778381
node_12: feature_name=GO:0003720 feature_id[228].value <= threshold=5.519326210021973
node_13: feature_name=GO:0046006 feature_id[364].value <= threshold=4.9985432624816895
node_14: feature_name=GO:0070424 feature_id[254].value <= threshold=6.233297109603882
node_15: feature_name=GO:0009892 feature_id[320].value <= threshold=59.99180793762207

node_16: feature_name=GO:0007064 feature_id[527].value <= threshold=11.57377815246582
node_17: feature_name=GO:0005575 feature_id[17].value <= threshold=15.417460918426514
node_18: feature_name=GO:0043368 feature_id[31].value <= threshold=4.059496641159058
node_19: feature_name=GO:0005164 feature_id[163].value <= threshold=5.793607711791992
node_20: feature_name=GO:0042130 feature_id[16].value <= threshold=5.073179721832275
node_21: feature_name=GO:0010216 feature_id[282].value <= threshold=4.834584474563599
node_22: feature_name=GO:0009628 feature_id[553].value <= threshold=29.314892768859863
node_23: feature_name=GO:0045628 feature_id[749].value <= threshold=3.1001367568969727
node_24: feature_name=GO:0042288 feature_id[100].value <= threshold=5.174932956695557
node_25: feature_name=GO:0002329 feature_id[406].value <= threshold=2.348930835723877
node_26: feature_name=GO:0051246 feature_id[642].value > threshold=0.6498909294605255
node_194: feature_name=GO:0009164 feature_id[546].value <= threshold=17.11970329284668
node_195: feature_name=GO:0001909 feature_id[386].value <= threshold=7.292566299438477
node_196: feature_name=GO:0003908 feature_id[11].value <= threshold=1.887226164340973
node_197: feature_name=GO:0009164 feature_id[546].value <= threshold=0.26642198860645294
node_198: feature_name=GO:2001242 feature_id[24].value <= threshold=9.207026481628418
node_199: feature_name=GO:0051246 feature_id[642].value > threshold=0.6504445374011993
node_203: feature_name=GO:0032461 feature_id[608].value <= threshold=2.607542634010315
node_204: feature_name=GO:0007584 feature_id[529].value <= threshold=16.099515914916992
node_205: feature_name=hsa05221 feature_id[349].value <= threshold=9.359850406646729
node_206: feature_name=GO:0002636 feature_id[447].value <= threshold=1.615447759628296
node_207: feature_name=GO:0033993 feature_id[421].value > threshold=2.965406110888858e-05
node_217: feature_name=GO:0002331 feature_id[404].value <= threshold=2.1883513927459717
node_218: feature_name=GO:0046498 feature_id[203].value <= threshold=4.085246205329895
node_219: feature_name=GO:0002832 feature_id[491].value <= threshold=3.0228612422943115
node_220: feature_name=GO:1904868 feature_id[306].value > threshold=3.8674756288528442
node_322: feature_name=GO:0045945 feature_id[267].value <= threshold=1.61331245303154
Class: negative genes

Rules_223 passed counts:4
node_0: feature_name=GO:0042113 feature_id[0].value <= threshold=13.408552169799805
node_1: feature_name=GO:0007568 feature_id[534].value <= threshold=5.0313897132873535
node_2: feature_name=GO:0002705 feature_id[541].value <= threshold=3.200145721435547
node_3: feature_name=GO:1901525 feature_id[576].value <= threshold=0.4466460347175598
node_4: feature_name=GO:0048539 feature_id[319].value <= threshold=3.0399646759033203

node_5: feature_name=GO:0001910	feature_id[385].value <= threshold=3.7437864542007446
node_6: feature_name=GO:0043200	feature_id[706].value <= threshold=9.307284355163574
node_7: feature_name=GO:0001773	feature_id[308].value <= threshold=4.016931533813477
node_8: feature_name=GO:0090116	feature_id[97].value <= threshold=7.99645471572876
node_9: feature_name=GO:0019814	feature_id[189].value <= threshold=4.525782108306885
node_10: feature_name=GO:1902583	feature_id[215].value <= threshold=17.60310649871826
node_11: feature_name=GO:0045429	feature_id[737].value <= threshold=1.6133361458778381
node_12: feature_name=GO:0003720	feature_id[228].value <= threshold=5.519326210021973
node_13: feature_name=GO:0046006	feature_id[364].value <= threshold=4.9985432624816895
node_14: feature_name=GO:0070424	feature_id[254].value <= threshold=6.233297109603882
node_15: feature_name=GO:0009892	feature_id[320].value <= threshold=59.99180793762207
node_16: feature_name=GO:0007064	feature_id[527].value <= threshold=11.57377815246582
node_17: feature_name=GO:0005575	feature_id[17].value <= threshold=15.417460918426514
node_18: feature_name=GO:0043368	feature_id[31].value <= threshold=4.059496641159058
node_19: feature_name=GO:0005164	feature_id[163].value <= threshold=5.793607711791992
node_20: feature_name=GO:0042130	feature_id[16].value <= threshold=5.073179721832275
node_21: feature_name=GO:0010216	feature_id[282].value <= threshold=4.834584474563599
node_22: feature_name=GO:0009628	feature_id[553].value <= threshold=29.314892768859863
node_23: feature_name=GO:0045628	feature_id[749].value <= threshold=3.1001367568969727
node_24: feature_name=GO:0042288	feature_id[100].value <= threshold=5.174932956695557
node_25: feature_name=GO:0002329	feature_id[406].value <= threshold=2.348930835723877
node_26: feature_name=GO:0051246	feature_id[642].value > threshold=0.6498909294605255
node_194: feature_name=GO:0009164	feature_id[546].value <= threshold=17.11970329284668
node_195: feature_name=GO:0001909	feature_id[386].value <= threshold=7.292566299438477
node_196: feature_name=GO:0003908	feature_id[11].value <= threshold=1.887226164340973
node_197: feature_name=GO:0009164	feature_id[546].value <= threshold=0.26642198860645294
node_198: feature_name=GO:2001242	feature_id[24].value <= threshold=9.207026481628418
node_199: feature_name=GO:0051246	feature_id[642].value > threshold=0.6504445374011993
node_203: feature_name=GO:0032461	feature_id[608].value <= threshold=2.607542634010315
node_204: feature_name=GO:0007584	feature_id[529].value <= threshold=16.099515914916992
node_205: feature_name=hsa05221	feature_id[349].value <= threshold=9.359850406646729
node_206: feature_name=GO:0002636	feature_id[447].value <= threshold=1.615447759628296
node_207: feature_name=GO:0033993	feature_id[421].value > threshold=2.96540611088858e-05
node_217: feature_name=GO:0002331	feature_id[404].value <= threshold=2.1883513927459717
node_218: feature_name=GO:0046498	feature_id[203].value <= threshold=4.085246205329895

node_219: feature_name=GO:0002832 feature_id[491].value <= threshold=3.0228612422943115
node_220: feature_name=GO:1904868 feature_id[306].value <= threshold=3.8674756288528442
node_221: feature_name=GO:0048545 feature_id[360].value > threshold=27.38136100769043
node_319: feature_name=GO:0047485 feature_id[121].value <= threshold=1.0277213156223297
Class: negative genes

Rules_224 passed counts:4
node_0: feature_name=GO:0042113 feature_id[0].value <= threshold=13.408552169799805
node_1: feature_name=GO:0007568 feature_id[534].value <= threshold=5.0313897132873535
node_2: feature_name=GO:0002705 feature_id[541].value <= threshold=3.200145721435547
node_3: feature_name=GO:1901525 feature_id[576].value <= threshold=0.4466460347175598
node_4: feature_name=GO:0048539 feature_id[319].value <= threshold=3.0399646759033203
node_5: feature_name=GO:0001910 feature_id[385].value <= threshold=3.7437864542007446
node_6: feature_name=GO:0043200 feature_id[706].value <= threshold=9.307284355163574
node_7: feature_name=GO:0001773 feature_id[308].value <= threshold=4.016931533813477
node_8: feature_name=GO:0090116 feature_id[97].value <= threshold=7.99645471572876
node_9: feature_name=GO:0019814 feature_id[189].value <= threshold=4.525782108306885
node_10: feature_name=GO:1902583 feature_id[215].value <= threshold=17.60310649871826
node_11: feature_name=GO:0045429 feature_id[737].value <= threshold=1.6133361458778381
node_12: feature_name=GO:0003720 feature_id[228].value <= threshold=5.519326210021973
node_13: feature_name=GO:0046006 feature_id[364].value <= threshold=4.9985432624816895
node_14: feature_name=GO:0070424 feature_id[254].value <= threshold=6.233297109603882
node_15: feature_name=GO:0009892 feature_id[320].value <= threshold=59.99180793762207
node_16: feature_name=GO:0007064 feature_id[527].value <= threshold=11.57377815246582
node_17: feature_name=GO:0005575 feature_id[17].value <= threshold=15.417460918426514
node_18: feature_name=GO:0043368 feature_id[31].value <= threshold=4.059496641159058
node_19: feature_name=GO:0005164 feature_id[163].value <= threshold=5.793607711791992
node_20: feature_name=GO:0042130 feature_id[16].value <= threshold=5.073179721832275
node_21: feature_name=GO:0010216 feature_id[282].value <= threshold=4.834584474563599
node_22: feature_name=GO:0009628 feature_id[553].value <= threshold=29.314892768859863
node_23: feature_name=GO:0045628 feature_id[749].value <= threshold=3.1001367568969727
node_24: feature_name=GO:0042288 feature_id[100].value <= threshold=5.174932956695557
node_25: feature_name=GO:0002329 feature_id[406].value <= threshold=2.348930835723877
node_26: feature_name=GO:0051246 feature_id[642].value > threshold=0.6498909294605255
node_194: feature_name=GO:0009164 feature_id[546].value <= threshold=17.11970329284668

node_195: feature_name=GO:0001909 feature_id[386].value <= threshold=7.292566299438477
node_196: feature_name=GO:0003908 feature_id[11].value <= threshold=1.887226164340973
node_197: feature_name=GO:0009164 feature_id[546].value <= threshold=0.26642198860645294
node_198: feature_name=GO:2001242 feature_id[24].value <= threshold=9.207026481628418
node_199: feature_name=GO:0051246 feature_id[642].value > threshold=0.6504445374011993
node_203: feature_name=GO:0032461 feature_id[608].value <= threshold=2.607542634010315
node_204: feature_name=GO:0007584 feature_id[529].value <= threshold=16.099515914916992
node_205: feature_name=hsa05221 feature_id[349].value <= threshold=9.359850406646729
node_206: feature_name=GO:0002636 feature_id[447].value <= threshold=1.615447759628296
node_207: feature_name=GO:0033993 feature_id[421].value > threshold=2.96540611088858e-05
node_217: feature_name=GO:0002331 feature_id[404].value <= threshold=2.1883513927459717
node_218: feature_name=GO:0046498 feature_id[203].value <= threshold=4.085246205329895
node_219: feature_name=GO:0002832 feature_id[491].value <= threshold=3.0228612422943115
node_220: feature_name=GO:1904868 feature_id[306].value <= threshold=3.8674756288528442
node_221: feature_name=GO:0048545 feature_id[360].value <= threshold=27.38136100769043
node_222: feature_name=GO:0071310 feature_id[760].value <= threshold=2.3522024154663086
node_223: feature_name=GO:0001775 feature_id[505].value <= threshold=9.71301555633545
node_224: feature_name=GO:0071310 feature_id[760].value <= threshold=2.346743583679199
node_225: feature_name=GO:0001772 feature_id[91].value <= threshold=3.252573609352112
node_226: feature_name=GO:0036498 feature_id[359].value <= threshold=12.092026710510254
node_227: feature_name=hsa05340 feature_id[351].value > threshold=0.7677814364433289
node_281: feature_name=GO:0006359 feature_id[87].value > threshold=0.846381276845932
node_295: feature_name=GO:1902275 feature_id[435].value <= threshold=0.2884182333946228
Class: negative genes

Rules_225 passed counts:4
node_0: feature_name=GO:0042113 feature_id[0].value <= threshold=13.408552169799805
node_1: feature_name=GO:0007568 feature_id[534].value <= threshold=5.0313897132873535
node_2: feature_name=GO:0002705 feature_id[541].value <= threshold=3.200145721435547
node_3: feature_name=GO:1901525 feature_id[576].value <= threshold=0.4466460347175598
node_4: feature_name=GO:0048539 feature_id[319].value <= threshold=3.0399646759033203
node_5: feature_name=GO:0001910 feature_id[385].value <= threshold=3.7437864542007446
node_6: feature_name=GO:0043200 feature_id[706].value <= threshold=9.307284355163574
node_7: feature_name=GO:0001773 feature_id[308].value <= threshold=4.016931533813477
node_8: feature_name=GO:0090116 feature_id[97].value <= threshold=7.99645471572876

node_9: feature_name=GO:0019814	feature_id[189].value <= threshold=4.525782108306885
node_10: feature_name=GO:1902583	feature_id[215].value <= threshold=17.60310649871826
node_11: feature_name=GO:0045429	feature_id[737].value <= threshold=1.6133361458778381
node_12: feature_name=GO:0003720	feature_id[228].value <= threshold=5.519326210021973
node_13: feature_name=GO:0046006	feature_id[364].value <= threshold=4.9985432624816895
node_14: feature_name=GO:0070424	feature_id[254].value <= threshold=6.233297109603882
node_15: feature_name=GO:0009892	feature_id[320].value <= threshold=59.99180793762207
node_16: feature_name=GO:0007064	feature_id[527].value <= threshold=11.57377815246582
node_17: feature_name=GO:0005575	feature_id[17].value <= threshold=15.417460918426514
node_18: feature_name=GO:0043368	feature_id[31].value <= threshold=4.059496641159058
node_19: feature_name=GO:0005164	feature_id[163].value <= threshold=5.793607711791992
node_20: feature_name=GO:0042130	feature_id[16].value <= threshold=5.073179721832275
node_21: feature_name=GO:0010216	feature_id[282].value <= threshold=4.834584474563599
node_22: feature_name=GO:0009628	feature_id[553].value <= threshold=29.314892768859863
node_23: feature_name=GO:0045628	feature_id[749].value <= threshold=3.1001367568969727
node_24: feature_name=GO:0042288	feature_id[100].value <= threshold=5.174932956695557
node_25: feature_name=GO:0002329	feature_id[406].value <= threshold=2.348930835723877
node_26: feature_name=GO:0051246	feature_id[642].value > threshold=0.6498909294605255
node_194: feature_name=GO:0009164	feature_id[546].value <= threshold=17.11970329284668
node_195: feature_name=GO:0001909	feature_id[386].value <= threshold=7.292566299438477
node_196: feature_name=GO:0003908	feature_id[11].value <= threshold=1.887226164340973
node_197: feature_name=GO:0009164	feature_id[546].value <= threshold=0.26642198860645294
node_198: feature_name=GO:2001242	feature_id[24].value <= threshold=9.207026481628418
node_199: feature_name=GO:0051246	feature_id[642].value > threshold=0.6504445374011993
node_203: feature_name=GO:0032461	feature_id[608].value <= threshold=2.607542634010315
node_204: feature_name=GO:0007584	feature_id[529].value <= threshold=16.099515914916992
node_205: feature_name=hsa05221	feature_id[349].value <= threshold=9.359850406646729
node_206: feature_name=GO:0002636	feature_id[447].value <= threshold=1.615447759628296
node_207: feature_name=GO:0033993	feature_id[421].value > threshold=2.96540611088858e-05
node_217: feature_name=GO:0002331	feature_id[404].value <= threshold=2.1883513927459717
node_218: feature_name=GO:0046498	feature_id[203].value <= threshold=4.085246205329895
node_219: feature_name=GO:0002832	feature_id[491].value <= threshold=3.0228612422943115
node_220: feature_name=GO:1904868	feature_id[306].value <= threshold=3.8674756288528442
node_221: feature_name=GO:0048545	feature_id[360].value <= threshold=27.38136100769043
node_222: feature_name=GO:0071310	feature_id[760].value <= threshold=2.3522024154663086

node_223: feature_name=GO:0001775 feature_id[505].value <= threshold=9.71301555633545
node_224: feature_name=GO:0071310 feature_id[760].value <= threshold=2.346743583679199
node_225: feature_name=GO:0001772 feature_id[91].value <= threshold=3.252573609352112
node_226: feature_name=GO:0036498 feature_id[359].value <= threshold=12.092026710510254
node_227: feature_name=hsa05340 feature_id[351].value <= threshold=0.7677814364433289
node_228: feature_name=GO:0032703 feature_id[617].value <= threshold=1.7450646758079529
node_229: feature_name=hsa04640 feature_id[79].value <= threshold=2.118402123451233
node_230: feature_name=GO:0042097 feature_id[675].value <= threshold=1.8110727667808533
node_231: feature_name=GO:0071391 feature_id[518].value <= threshold=2.691588521003723
node_232: feature_name=GO:0001666 feature_id[682].value <= threshold=5.225740432739258
node_233: feature_name=GO:0048539 feature_id[319].value > threshold=1.8523842096328735
node_263: feature_name=GO:0002507 feature_id[34].value <= threshold=0.6999551057815552
Class: negative genes

Rules_226 passed counts:4
node_0: feature_name=GO:0042113 feature_id[0].value <= threshold=13.408552169799805
node_1: feature_name=GO:0007568 feature_id[534].value <= threshold=5.0313897132873535
node_2: feature_name=GO:0002705 feature_id[541].value <= threshold=3.200145721435547
node_3: feature_name=GO:1901525 feature_id[576].value <= threshold=0.4466460347175598
node_4: feature_name=GO:0048539 feature_id[319].value <= threshold=3.0399646759033203
node_5: feature_name=GO:0001910 feature_id[385].value <= threshold=3.7437864542007446
node_6: feature_name=GO:0043200 feature_id[706].value <= threshold=9.307284355163574
node_7: feature_name=GO:0001773 feature_id[308].value <= threshold=4.016931533813477
node_8: feature_name=GO:0090116 feature_id[97].value <= threshold=7.99645471572876
node_9: feature_name=GO:0019814 feature_id[189].value <= threshold=4.525782108306885
node_10: feature_name=GO:1902583 feature_id[215].value <= threshold=17.60310649871826
node_11: feature_name=GO:0045429 feature_id[737].value <= threshold=1.6133361458778381
node_12: feature_name=GO:0003720 feature_id[228].value <= threshold=5.519326210021973
node_13: feature_name=GO:0046006 feature_id[364].value <= threshold=4.9985432624816895
node_14: feature_name=GO:0070424 feature_id[254].value <= threshold=6.233297109603882
node_15: feature_name=GO:0009892 feature_id[320].value <= threshold=59.99180793762207
node_16: feature_name=GO:0007064 feature_id[527].value <= threshold=11.57377815246582
node_17: feature_name=GO:0005575 feature_id[17].value <= threshold=15.417460918426514
node_18: feature_name=GO:0043368 feature_id[31].value <= threshold=4.059496641159058
node_19: feature_name=GO:0005164 feature_id[163].value <= threshold=5.793607711791992

node_20: feature_name=GO:0042130 feature_id[16].value <= threshold=5.073179721832275
node_21: feature_name=GO:0010216 feature_id[282].value <= threshold=4.834584474563599
node_22: feature_name=GO:0009628 feature_id[553].value <= threshold=29.314892768859863
node_23: feature_name=GO:0045628 feature_id[749].value <= threshold=3.1001367568969727
node_24: feature_name=GO:0042288 feature_id[100].value <= threshold=5.174932956695557
node_25: feature_name=GO:0002329 feature_id[406].value <= threshold=2.348930835723877
node_26: feature_name=GO:0051246 feature_id[642].value <= threshold=0.6498909294605255
node_27: feature_name=GO:0042162 feature_id[355].value <= threshold=8.05103588104248
node_28: feature_name=hsa04668 feature_id[333].value <= threshold=5.614926338195801
node_29: feature_name=GO:0032200 feature_id[613].value <= threshold=28.735913276672363
node_30: feature_name=hsa04662 feature_id[334].value <= threshold=9.588344097137451
node_31: feature_name=GO:1901992 feature_id[105].value <= threshold=5.291427850723267
node_32: feature_name=GO:0072341 feature_id[144].value <= threshold=6.01465106010437
node_33: feature_name=GO:0047485 feature_id[121].value <= threshold=6.657836437225342
node_34: feature_name=GO:0090594 feature_id[738].value <= threshold=1.8673912286758423
node_35: feature_name=GO:0043525 feature_id[524].value <= threshold=2.881397008895874
node_36: feature_name=GO:0032504 feature_id[244].value <= threshold=17.22207546234131
node_37: feature_name=GO:0051348 feature_id[126].value <= threshold=5.933559417724609
node_38: feature_name=GO:0042522 feature_id[692].value <= threshold=1.78694087266922
node_39: feature_name=GO:0031100 feature_id[73].value <= threshold=3.2312101125717163
node_40: feature_name=GO:0042493 feature_id[149].value <= threshold=19.771170616149902
node_41: feature_name=GO:0001552 feature_id[366].value > threshold=2.1711617708206177
node_151: feature_name=GO:0002706 feature_id[565].value > threshold=0.03241767734289169
Class: negative genes

Rules_227 passed counts:4
node_0: feature_name=GO:0042113 feature_id[0].value <= threshold=13.408552169799805
node_1: feature_name=GO:0007568 feature_id[534].value <= threshold=5.0313897132873535
node_2: feature_name=GO:0002705 feature_id[541].value <= threshold=3.200145721435547
node_3: feature_name=GO:1901525 feature_id[576].value <= threshold=0.4466460347175598
node_4: feature_name=GO:0048539 feature_id[319].value <= threshold=3.0399646759033203
node_5: feature_name=GO:0001910 feature_id[385].value <= threshold=3.7437864542007446
node_6: feature_name=GO:0043200 feature_id[706].value <= threshold=9.307284355163574
node_7: feature_name=GO:0001773 feature_id[308].value <= threshold=4.016931533813477
node_8: feature_name=GO:0090116 feature_id[97].value <= threshold=7.99645471572876

node_9: feature_name=GO:0019814 feature_id[189].value <= threshold=4.525782108306885
node_10: feature_name=GO:1902583 feature_id[215].value <= threshold=17.60310649871826
node_11: feature_name=GO:0045429 feature_id[737].value <= threshold=1.6133361458778381
node_12: feature_name=GO:0003720 feature_id[228].value <= threshold=5.519326210021973
node_13: feature_name=GO:0046006 feature_id[364].value <= threshold=4.9985432624816895
node_14: feature_name=GO:0070424 feature_id[254].value <= threshold=6.233297109603882
node_15: feature_name=GO:0009892 feature_id[320].value <= threshold=59.99180793762207
node_16: feature_name=GO:0007064 feature_id[527].value <= threshold=11.57377815246582
node_17: feature_name=GO:0005575 feature_id[17].value <= threshold=15.417460918426514
node_18: feature_name=GO:0043368 feature_id[31].value <= threshold=4.059496641159058
node_19: feature_name=GO:0005164 feature_id[163].value <= threshold=5.793607711791992
node_20: feature_name=GO:0042130 feature_id[16].value <= threshold=5.073179721832275
node_21: feature_name=GO:0010216 feature_id[282].value <= threshold=4.834584474563599
node_22: feature_name=GO:0009628 feature_id[553].value <= threshold=29.314892768859863
node_23: feature_name=GO:0045628 feature_id[749].value <= threshold=3.1001367568969727
node_24: feature_name=GO:0042288 feature_id[100].value <= threshold=5.174932956695557
node_25: feature_name=GO:0002329 feature_id[406].value <= threshold=2.348930835723877
node_26: feature_name=GO:0051246 feature_id[642].value <= threshold=0.6498909294605255
node_27: feature_name=GO:0042162 feature_id[355].value <= threshold=8.05103588104248
node_28: feature_name=hsa04668 feature_id[333].value <= threshold=5.614926338195801
node_29: feature_name=GO:0032200 feature_id[613].value <= threshold=28.735913276672363
node_30: feature_name=hsa04662 feature_id[334].value <= threshold=9.588344097137451
node_31: feature_name=GO:1901992 feature_id[105].value <= threshold=5.291427850723267
node_32: feature_name=GO:0072341 feature_id[144].value <= threshold=6.01465106010437
node_33: feature_name=GO:0047485 feature_id[121].value <= threshold=6.657836437225342
node_34: feature_name=GO:0090594 feature_id[738].value <= threshold=1.8673912286758423
node_35: feature_name=GO:0043525 feature_id[524].value <= threshold=2.881397008895874
node_36: feature_name=GO:0032504 feature_id[244].value <= threshold=17.22207546234131
node_37: feature_name=GO:0051348 feature_id[126].value <= threshold=5.933559417724609
node_38: feature_name=GO:0042522 feature_id[692].value <= threshold=1.78694087266922
node_39: feature_name=GO:0031100 feature_id[73].value <= threshold=3.2312101125717163
node_40: feature_name=GO:0042493 feature_id[149].value <= threshold=19.771170616149902
node_41: feature_name=GO:0001552 feature_id[366].value <= threshold=2.1711617708206177
node_42: feature_name=GO:0046483 feature_id[391].value <= threshold=320.9667053222656
node_43: feature_name=GO:0002439 feature_id[431].value <= threshold=1.832722783088684

node_44: feature_name=GO:0006555 feature_id[514].value <= threshold=5.321640968322754
node_45: feature_name=GO:0070424 feature_id[254].value <= threshold=3.321003556251526
node_46: feature_name=hsa05144 feature_id[751].value <= threshold=2.635499954223633
node_47: feature_name=GO:0032633 feature_id[627].value <= threshold=1.2592533230781555
node_48: feature_name=GO:0019814 feature_id[189].value <= threshold=2.1583125591278076
node_49: feature_name=GO:1901989 feature_id[30].value <= threshold=3.3150794506073
node_50: feature_name=GO:1903318 feature_id[196].value <= threshold=1.62252539396286
node_51: feature_name=GO:0060576 feature_id[204].value <= threshold=2.1097792387008667
node_52: feature_name=hsa05221 feature_id[349].value > threshold=0.8723124265670776
node_96: feature_name=hsa05221 feature_id[349].value > threshold=0.8756309151649475
node_98: feature_name=GO:0046794 feature_id[794].value <= threshold=6.585465431213379
node_99: feature_name=GO:0005035 feature_id[133].value <= threshold=0.6986835598945618
node_100: feature_name=hsa04640 feature_id[79].value > threshold=1.458605408668518
node_106: feature_name=GO:0071901 feature_id[407].value <= threshold=0.31348803639411926
Class: negative genes

Rules_228 passed counts:3
node_0: feature_name=GO:0042113 feature_id[0].value > threshold=13.408552169799805
node_1454: feature_name=GO:0050851 feature_id[29].value > threshold=9.650307655334473
node_1534: feature_name=GO:1902166 feature_id[301].value > threshold=0.303210511803627
node_1548: feature_name=GO:0035872 feature_id[659].value <= threshold=35.09038162231445
node_1549: feature_name=GO:0032069 feature_id[614].value > threshold=3.441983938217163
node_1571: feature_name=GO:0050864 feature_id[288].value > threshold=7.516624450683594
node_1575: feature_name=GO:2000772 feature_id[95].value > threshold=0.9979664981365204
node_1579: feature_name=GO:0043525 feature_id[524].value <= threshold=22.19614315032959
node_1580: feature_name=GO:0032479 feature_id[441].value <= threshold=4.490016937255859
node_1581: feature_name=GO:0046677 feature_id[591].value > threshold=5.264658212661743
Class: negative genes

Rules_229 passed counts:3
node_0: feature_name=GO:0042113 feature_id[0].value > threshold=13.408552169799805
node_1454: feature_name=GO:0050851 feature_id[29].value > threshold=9.650307655334473
node_1534: feature_name=GO:1902166 feature_id[301].value > threshold=0.303210511803627
node_1548: feature_name=GO:0035872 feature_id[659].value <= threshold=35.09038162231445
node_1549: feature_name=GO:0032069 feature_id[614].value > threshold=3.441983938217163

node_1571: feature_name=GO:0050864 feature_id[288].value > threshold=7.516624450683594
node_1575: feature_name=GO:2000772 feature_id[95].value <= threshold=0.9979664981365204
node_1576: feature_name=GO:0042802 feature_id[795].value > threshold=18.696807861328125
Class: negative genes

Rules_230 passed counts:3
node_0: feature_name=GO:0042113 feature_id[0].value > threshold=13.408552169799805
node_1454: feature_name=GO:0050851 feature_id[29].value > threshold=9.650307655334473
node_1534: feature_name=GO:1902166 feature_id[301].value > threshold=0.303210511803627
node_1548: feature_name=GO:0035872 feature_id[659].value <= threshold=35.09038162231445
node_1549: feature_name=GO:0032069 feature_id[614].value > threshold=3.441983938217163
node_1571: feature_name=GO:0050864 feature_id[288].value > threshold=7.516624450683594
node_1575: feature_name=GO:2000772 feature_id[95].value <= threshold=0.9979664981365204
node_1576: feature_name=GO:0042802 feature_id[795].value <= threshold=18.696807861328125
Class: positive genes

Rules_231 passed counts:3
node_0: feature_name=GO:0042113 feature_id[0].value > threshold=13.408552169799805
node_1454: feature_name=GO:0050851 feature_id[29].value > threshold=9.650307655334473
node_1534: feature_name=GO:1902166 feature_id[301].value > threshold=0.303210511803627
node_1548: feature_name=GO:0035872 feature_id[659].value <= threshold=35.09038162231445
node_1549: feature_name=GO:0032069 feature_id[614].value <= threshold=3.441983938217163
node_1550: feature_name=GO:0050897 feature_id[164].value <= threshold=1.3939869403839111
node_1551: feature_name=GO:0051454 feature_id[307].value <= threshold=0.9144491851329803
node_1552: feature_name=GO:0035825 feature_id[662].value > threshold=0.08555268123745918
node_1556: feature_name=GO:0031265 feature_id[48].value <= threshold=0.7173363864421844
node_1557: feature_name=GO:0002200 feature_id[82].value <= threshold=5.254605531692505
Class: positive genes

Rules_232 passed counts:3
node_0: feature_name=GO:0042113 feature_id[0].value > threshold=13.408552169799805
node_1454: feature_name=GO:0050851 feature_id[29].value > threshold=9.650307655334473
node_1534: feature_name=GO:1902166 feature_id[301].value <= threshold=0.303210511803627
node_1535: feature_name=GO:0042287 feature_id[20].value <= threshold=1.4291933178901672
node_1536: feature_name=GO:0023026 feature_id[69].value <= threshold=1.7696257829666138

node_1537: feature_name=GO:0048661 feature_id[733].value <= threshold=3.2364230155944824
Class: positive genes

Rules_233 passed counts:3
node_0: feature_name=GO:0042113 feature_id[0].value > threshold=13.408552169799805
node_1454: feature_name=GO:0050851 feature_id[29].value <= threshold=9.650307655334473
node_1455: feature_name=GO:0006304 feature_id[510].value <= threshold=5.960662126541138
node_1456: feature_name=GO:0032673 feature_id[623].value > threshold=4.092082738876343
node_1510: feature_name=GO:0046898 feature_id[161].value <= threshold=2.2367727756500244
node_1511: feature_name=GO:0030183 feature_id[590].value > threshold=12.193635940551758
node_1521: feature_name=GO:0032504 feature_id[244].value > threshold=0.8662047982215881
node_1523: feature_name=GO:0045589 feature_id[347].value <= threshold=3.9446849822998047
Class: positive genes

Rules_234 passed counts:3
node_0: feature_name=GO:0042113 feature_id[0].value > threshold=13.408552169799805
node_1454: feature_name=GO:0050851 feature_id[29].value <= threshold=9.650307655334473
node_1455: feature_name=GO:0006304 feature_id[510].value <= threshold=5.960662126541138
node_1456: feature_name=GO:0032673 feature_id[623].value > threshold=4.092082738876343
node_1510: feature_name=GO:0046898 feature_id[161].value <= threshold=2.2367727756500244
node_1511: feature_name=GO:0030183 feature_id[590].value <= threshold=12.193635940551758
node_1512: feature_name=GO:0032480 feature_id[525].value > threshold=0.5560729652643204
node_1514: feature_name=GO:1902554 feature_id[660].value > threshold=1.3245088458061218
node_1518: feature_name=GO:0010952 feature_id[483].value <= threshold=9.767204761505127
Class: negative genes

Rules_235 passed counts:3
node_0: feature_name=GO:0042113 feature_id[0].value > threshold=13.408552169799805
node_1454: feature_name=GO:0050851 feature_id[29].value <= threshold=9.650307655334473
node_1455: feature_name=GO:0006304 feature_id[510].value <= threshold=5.960662126541138
node_1456: feature_name=GO:0032673 feature_id[623].value > threshold=4.092082738876343
node_1510: feature_name=GO:0046898 feature_id[161].value <= threshold=2.2367727756500244
node_1511: feature_name=GO:0030183 feature_id[590].value <= threshold=12.193635940551758
node_1512: feature_name=GO:0032480 feature_id[525].value <= threshold=0.5560729652643204
Class: negative genes

Rules_236 passed counts:3
node_0: feature_name=GO:0042113 feature_id[0].value > threshold=13.408552169799805
node_1454: feature_name=GO:0050851 feature_id[29].value <= threshold=9.650307655334473
node_1455: feature_name=GO:0006304 feature_id[510].value <= threshold=5.960662126541138
node_1456: feature_name=GO:0032673 feature_id[623].value <= threshold=4.092082738876343
node_1457: feature_name=GO:0002429 feature_id[748].value > threshold=4.626799821853638
node_1463: feature_name=GO:0015672 feature_id[573].value > threshold=0.08226438239216805
node_1475: feature_name=GO:0030217 feature_id[589].value <= threshold=19.77315902709961
node_1476: feature_name=GO:0002381 feature_id[96].value <= threshold=4.645626783370972
node_1477: feature_name=GO:0045058 feature_id[727].value <= threshold=5.092289447784424
node_1478: feature_name=GO:0032703 feature_id[617].value > threshold=2.6942304372787476
node_1482: feature_name=GO:1904894 feature_id[726].value > threshold=4.65221294760704
Class: positive genes

Rules_237 passed counts:3
node_0: feature_name=GO:0042113 feature_id[0].value > threshold=13.408552169799805
node_1454: feature_name=GO:0050851 feature_id[29].value <= threshold=9.650307655334473
node_1455: feature_name=GO:0006304 feature_id[510].value <= threshold=5.960662126541138
node_1456: feature_name=GO:0032673 feature_id[623].value <= threshold=4.092082738876343
node_1457: feature_name=GO:0002429 feature_id[748].value > threshold=4.626799821853638
node_1463: feature_name=GO:0015672 feature_id[573].value <= threshold=0.08226438239216805
node_1464: feature_name=GO:0038065 feature_id[670].value > threshold=0.3765842020511627
node_1468: feature_name=GO:0046685 feature_id[782].value > threshold=2.9275591373443604
Class: negative genes

Rules_238 passed counts:3
node_0: feature_name=GO:0042113 feature_id[0].value <= threshold=13.408552169799805
node_1: feature_name=GO:0007568 feature_id[534].value > threshold=5.0313897132873535
node_913: feature_name=GO:0032763 feature_id[629].value > threshold=0.31753237545490265
node_1195: feature_name=GO:0071301 feature_id[442].value > threshold=0.11534593254327774
node_1375: feature_name=GO:0046500 feature_id[171].value > threshold=1.130434513092041
node_1437: feature_name=GO:0033343 feature_id[639].value > threshold=0.98012974858284
node_1443: feature_name=GO:0007126 feature_id[520].value <= threshold=0.40390433371067047
node_1444: feature_name=GO:0042267 feature_id[148].value > threshold=0.3329026997089386

Class: negative genes

Rules_239 passed counts:3
node_0: feature_name=GO:0042113 feature_id[0].value <= threshold=13.408552169799805
node_1: feature_name=GO:0007568 feature_id[534].value > threshold=5.0313897132873535
node_913: feature_name=GO:0032763 feature_id[629].value > threshold=0.31753237545490265
node_1195: feature_name=GO:0071301 feature_id[442].value > threshold=0.11534593254327774
node_1375: feature_name=GO:0046500 feature_id[171].value <= threshold=1.130434513092041
node_1376: feature_name=GO:0001782 feature_id[240].value <= threshold=6.639636993408203
node_1377: feature_name=GO:0043371 feature_id[707].value <= threshold=2.562113642692566
node_1378: feature_name=GO:0030291 feature_id[147].value > threshold=6.834916830062866
node_1424: feature_name=GO:0002439 feature_id[431].value <= threshold=0.6129993498325348
node_1425: feature_name=GO:0042162 feature_id[355].value <= threshold=0.09355577081441879
Class: negative genes

Rules_240 passed counts:3
node_0: feature_name=GO:0042113 feature_id[0].value <= threshold=13.408552169799805
node_1: feature_name=GO:0007568 feature_id[534].value > threshold=5.0313897132873535
node_913: feature_name=GO:0032763 feature_id[629].value > threshold=0.31753237545490265
node_1195: feature_name=GO:0071301 feature_id[442].value > threshold=0.11534593254327774
node_1375: feature_name=GO:0046500 feature_id[171].value <= threshold=1.130434513092041
node_1376: feature_name=GO:0001782 feature_id[240].value <= threshold=6.639636993408203
node_1377: feature_name=GO:0043371 feature_id[707].value <= threshold=2.562113642692566
node_1378: feature_name=GO:0030291 feature_id[147].value <= threshold=6.834916830062866
node_1379: feature_name=GO:0045656 feature_id[756].value > threshold=0.6281269192695618
node_1393: feature_name=GO:0046007 feature_id[152].value <= threshold=1.020785927772522
node_1394: feature_name=GO:0000302 feature_id[375].value <= threshold=20.847331047058105
node_1395: feature_name=hsa04110 feature_id[328].value <= threshold=43.297359466552734
node_1396: feature_name=GO:0050731 feature_id[804].value > threshold=15.756641864776611
Class: positive genes

Rules_241 passed counts:3
node_0: feature_name=GO:0042113 feature_id[0].value <= threshold=13.408552169799805
node_1: feature_name=GO:0007568 feature_id[534].value > threshold=5.0313897132873535
node_913: feature_name=GO:0032763 feature_id[629].value > threshold=0.31753237545490265

node_1195: feature_name=GO:0071301 feature_id[442].value <= threshold=0.11534593254327774
node_1196: feature_name=GO:0046500 feature_id[171].value > threshold=0.6314916908740997
node_1340: feature_name=GO:0043627 feature_id[562].value <= threshold=25.17277240753174
node_1341: feature_name=GO:0010835 feature_id[560].value <= threshold=1.0601619482040405
node_1342: feature_name=GO:0016363 feature_id[90].value <= threshold=6.534365892410278
node_1343: feature_name=GO:0030983 feature_id[291].value > threshold=3.5806901454925537
node_1359: feature_name=GO:0046632 feature_id[248].value > threshold=0.5181337743997574
node_1361: feature_name=GO:0033554 feature_id[418].value > threshold=43.66302299499512
Class: negative genes

Rules_242 passed counts:3
node_0: feature_name=GO:0042113 feature_id[0].value <= threshold=13.408552169799805
node_1: feature_name=GO:0007568 feature_id[534].value > threshold=5.0313897132873535
node_913: feature_name=GO:0032763 feature_id[629].value > threshold=0.31753237545490265
node_1195: feature_name=GO:0071301 feature_id[442].value <= threshold=0.11534593254327774
node_1196: feature_name=GO:0046500 feature_id[171].value > threshold=0.6314916908740997
node_1340: feature_name=GO:0043627 feature_id[562].value <= threshold=25.17277240753174
node_1341: feature_name=GO:0010835 feature_id[560].value <= threshold=1.0601619482040405
node_1342: feature_name=GO:0016363 feature_id[90].value <= threshold=6.534365892410278
node_1343: feature_name=GO:0030983 feature_id[291].value <= threshold=3.5806901454925537
node_1344: feature_name=GO:0023030 feature_id[45].value > threshold=0.6541633009910583
Class: positive genes

Rules_243 passed counts:3
node_0: feature_name=GO:0042113 feature_id[0].value <= threshold=13.408552169799805
node_1: feature_name=GO:0007568 feature_id[534].value > threshold=5.0313897132873535
node_913: feature_name=GO:0032763 feature_id[629].value > threshold=0.31753237545490265
node_1195: feature_name=GO:0071301 feature_id[442].value <= threshold=0.11534593254327774
node_1196: feature_name=GO:0046500 feature_id[171].value > threshold=0.6314916908740997
node_1340: feature_name=GO:0043627 feature_id[562].value <= threshold=25.17277240753174
node_1341: feature_name=GO:0010835 feature_id[560].value <= threshold=1.0601619482040405
node_1342: feature_name=GO:0016363 feature_id[90].value <= threshold=6.534365892410278
node_1343: feature_name=GO:0030983 feature_id[291].value <= threshold=3.5806901454925537
node_1344: feature_name=GO:0023030 feature_id[45].value <= threshold=0.6541633009910583
node_1345: feature_name=GO:0070245 feature_id[326].value > threshold=1.9657342433929443

node_1355: feature_name=GO:0010952 feature_id[483].value <= threshold=2.8519983291625977
Class: positive genes

Rules_244 passed counts:3
node_0: feature_name=GO:0042113 feature_id[0].value <= threshold=13.408552169799805
node_1: feature_name=GO:0007568 feature_id[534].value > threshold=5.0313897132873535
node_913: feature_name=GO:0032763 feature_id[629].value > threshold=0.31753237545490265
node_1195: feature_name=GO:0071301 feature_id[442].value <= threshold=0.11534593254327774
node_1196: feature_name=GO:0046500 feature_id[171].value <= threshold=0.6314916908740997
node_1197: feature_name=GO:0030852 feature_id[22].value <= threshold=2.9768868684768677
node_1198: feature_name=hsa04672 feature_id[276].value > threshold=8.06681227684021
node_1326: feature_name=hsa04640 feature_id[79].value > threshold=26.757009506225586
Class: negative genes

Rules_245 passed counts:3
node_0: feature_name=GO:0042113 feature_id[0].value <= threshold=13.408552169799805
node_1: feature_name=GO:0007568 feature_id[534].value > threshold=5.0313897132873535
node_913: feature_name=GO:0032763 feature_id[629].value > threshold=0.31753237545490265
node_1195: feature_name=GO:0071301 feature_id[442].value <= threshold=0.11534593254327774
node_1196: feature_name=GO:0046500 feature_id[171].value <= threshold=0.6314916908740997
node_1197: feature_name=GO:0030852 feature_id[22].value <= threshold=2.9768868684768677
node_1198: feature_name=hsa04672 feature_id[276].value <= threshold=8.06681227684021
node_1199: feature_name=GO:0001836 feature_id[450].value > threshold=0.09961023926734924
node_1201: feature_name=GO:0070230 feature_id[64].value <= threshold=3.3956379890441895
node_1202: feature_name=GO:0070102 feature_id[49].value > threshold=4.805386543273926
Class: positive genes

Rules_246 passed counts:3
node_0: feature_name=GO:0042113 feature_id[0].value <= threshold=13.408552169799805
node_1: feature_name=GO:0007568 feature_id[534].value > threshold=5.0313897132873535
node_913: feature_name=GO:0032763 feature_id[629].value > threshold=0.31753237545490265
node_1195: feature_name=GO:0071301 feature_id[442].value <= threshold=0.11534593254327774
node_1196: feature_name=GO:0046500 feature_id[171].value <= threshold=0.6314916908740997
node_1197: feature_name=GO:0030852 feature_id[22].value <= threshold=2.9768868684768677
node_1198: feature_name=hsa04672 feature_id[276].value <= threshold=8.06681227684021

node_1199: feature_name=GO:0001836 feature_id[450].value > threshold=0.09961023926734924
node_1201: feature_name=GO:0070230 feature_id[64].value <= threshold=3.3956379890441895
node_1202: feature_name=GO:0070102 feature_id[49].value <= threshold=4.805386543273926
node_1203: feature_name=GO:0045553 feature_id[736].value > threshold=1.4970332980155945
node_1319: feature_name=GO:0031667 feature_id[648].value <= threshold=25.942169189453125
Class: positive genes

Rules_247 passed counts:3
node_0: feature_name=GO:0042113 feature_id[0].value <= threshold=13.408552169799805
node_1: feature_name=GO:0007568 feature_id[534].value > threshold=5.0313897132873535
node_913: feature_name=GO:0032763 feature_id[629].value > threshold=0.31753237545490265
node_1195: feature_name=GO:0071301 feature_id[442].value <= threshold=0.11534593254327774
node_1196: feature_name=GO:0046500 feature_id[171].value <= threshold=0.6314916908740997
node_1197: feature_name=GO:0030852 feature_id[22].value <= threshold=2.9768868684768677
node_1198: feature_name=hsa04672 feature_id[276].value <= threshold=8.06681227684021
node_1199: feature_name=GO:0001836 feature_id[450].value > threshold=0.09961023926734924
node_1201: feature_name=GO:0070230 feature_id[64].value <= threshold=3.3956379890441895
node_1202: feature_name=GO:0070102 feature_id[49].value <= threshold=4.805386543273926
node_1203: feature_name=GO:0045553 feature_id[736].value <= threshold=1.4970332980155945
node_1204: feature_name=GO:0051311 feature_id[58].value <= threshold=3.301889181137085
node_1205: feature_name=GO:0030291 feature_id[147].value <= threshold=8.156315326690674
node_1206: feature_name=GO:0045637 feature_id[241].value > threshold=0.01834342861548066
node_1208: feature_name=GO:0002890 feature_id[98].value <= threshold=2.767483353614807
node_1209: feature_name=GO:0046632 feature_id[248].value <= threshold=11.663254261016846
node_1210: feature_name=GO:0008588 feature_id[26].value <= threshold=1.391375720500946
node_1211: feature_name=GO:0010639 feature_id[564].value <= threshold=18.975126266479492
node_1212: feature_name=GO:0046685 feature_id[782].value > threshold=5.153738260269165
node_1296: feature_name=GO:0019692 feature_id[584].value > threshold=0.0685717724263668
node_1300: feature_name=GO:0032649 feature_id[626].value <= threshold=0.2627730891108513
node_1301: feature_name=hsa05144 feature_id[751].value <= threshold=2.427334427833557
Class: positive genes

Rules_248 passed counts:3
node_0: feature_name=GO:0042113 feature_id[0].value <= threshold=13.408552169799805
node_1: feature_name=GO:0007568 feature_id[534].value > threshold=5.0313897132873535

node_913: feature_name=GO:0032763 feature_id[629].value > threshold=0.31753237545490265
node_1195: feature_name=GO:0071301 feature_id[442].value <= threshold=0.11534593254327774
node_1196: feature_name=GO:0046500 feature_id[171].value <= threshold=0.6314916908740997
node_1197: feature_name=GO:0030852 feature_id[22].value <= threshold=2.9768868684768677
node_1198: feature_name=hsa04672 feature_id[276].value <= threshold=8.06681227684021
node_1199: feature_name=GO:0001836 feature_id[450].value > threshold=0.09961023926734924
node_1201: feature_name=GO:0070230 feature_id[64].value <= threshold=3.3956379890441895
node_1202: feature_name=GO:0070102 feature_id[49].value <= threshold=4.805386543273926
node_1203: feature_name=GO:0045553 feature_id[736].value <= threshold=1.4970332980155945
node_1204: feature_name=GO:0051311 feature_id[58].value <= threshold=3.301889181137085
node_1205: feature_name=GO:0030291 feature_id[147].value <= threshold=8.156315326690674
node_1206: feature_name=GO:0045637 feature_id[241].value > threshold=0.01834342861548066
node_1208: feature_name=GO:0002890 feature_id[98].value <= threshold=2.767483353614807
node_1209: feature_name=GO:0046632 feature_id[248].value <= threshold=11.663254261016846
node_1210: feature_name=GO:0008588 feature_id[26].value <= threshold=1.391375720500946
node_1211: feature_name=GO:0010639 feature_id[564].value <= threshold=18.975126266479492
node_1212: feature_name=GO:0046685 feature_id[782].value <= threshold=5.153738260269165
node_1213: feature_name=GO:0038116 feature_id[668].value <= threshold=1.7859655618667603
node_1214: feature_name=GO:0046449 feature_id[763].value <= threshold=5.603115558624268
node_1215: feature_name=GO:0048539 feature_id[319].value > threshold=0.5478232800960541
node_1269: feature_name=GO:0072341 feature_id[144].value <= threshold=1.3205850720405579
node_1270: feature_name=GO:0010835 feature_id[560].value <= threshold=0.7877185940742493
node_1271: feature_name=GO:0000097 feature_id[376].value > threshold=0.9960366785526276
node_1279: feature_name=hsa05142 feature_id[769].value > threshold=7.861773252487183
Class: positive genes

Rules_249 passed counts:3
node_0: feature_name=GO:0042113 feature_id[0].value <= threshold=13.408552169799805
node_1: feature_name=GO:0007568 feature_id[534].value > threshold=5.0313897132873535
node_913: feature_name=GO:0032763 feature_id[629].value > threshold=0.31753237545490265
node_1195: feature_name=GO:0071301 feature_id[442].value <= threshold=0.11534593254327774
node_1196: feature_name=GO:0046500 feature_id[171].value <= threshold=0.6314916908740997
node_1197: feature_name=GO:0030852 feature_id[22].value <= threshold=2.9768868684768677
node_1198: feature_name=hsa04672 feature_id[276].value <= threshold=8.06681227684021
node_1199: feature_name=GO:0001836 feature_id[450].value > threshold=0.09961023926734924

node_1201: feature_name=GO:0070230 feature_id[64].value <= threshold=3.3956379890441895
node_1202: feature_name=GO:0070102 feature_id[49].value <= threshold=4.805386543273926
node_1203: feature_name=GO:0045553 feature_id[736].value <= threshold=1.4970332980155945
node_1204: feature_name=GO:0051311 feature_id[58].value <= threshold=3.301889181137085
node_1205: feature_name=GO:0030291 feature_id[147].value <= threshold=8.156315326690674
node_1206: feature_name=GO:0045637 feature_id[241].value > threshold=0.01834342861548066
node_1208: feature_name=GO:0002890 feature_id[98].value <= threshold=2.767483353614807
node_1209: feature_name=GO:0046632 feature_id[248].value <= threshold=11.663254261016846
node_1210: feature_name=GO:0008588 feature_id[26].value <= threshold=1.391375720500946
node_1211: feature_name=GO:0010639 feature_id[564].value <= threshold=18.975126266479492
node_1212: feature_name=GO:0046685 feature_id[782].value <= threshold=5.153738260269165
node_1213: feature_name=GO:0038116 feature_id[668].value <= threshold=1.7859655618667603
node_1214: feature_name=GO:0046449 feature_id[763].value <= threshold=5.603115558624268
node_1215: feature_name=GO:0048539 feature_id[319].value > threshold=0.5478232800960541
node_1269: feature_name=GO:0072341 feature_id[144].value <= threshold=1.3205850720405579
node_1270: feature_name=GO:0010835 feature_id[560].value <= threshold=0.7877185940742493
node_1271: feature_name=GO:0000097 feature_id[376].value > threshold=0.9960366785526276
node_1279: feature_name=hsa05142 feature_id[769].value <= threshold=7.861773252487183
Class: negative genes

Rules_250 passed counts:3
node_0: feature_name=GO:0042113 feature_id[0].value <= threshold=13.408552169799805
node_1: feature_name=GO:0007568 feature_id[534].value > threshold=5.0313897132873535
node_913: feature_name=GO:0032763 feature_id[629].value > threshold=0.31753237545490265
node_1195: feature_name=GO:0071301 feature_id[442].value <= threshold=0.11534593254327774
node_1196: feature_name=GO:0046500 feature_id[171].value <= threshold=0.6314916908740997
node_1197: feature_name=GO:0030852 feature_id[22].value <= threshold=2.9768868684768677
node_1198: feature_name=hsa04672 feature_id[276].value <= threshold=8.06681227684021
node_1199: feature_name=GO:0001836 feature_id[450].value > threshold=0.09961023926734924
node_1201: feature_name=GO:0070230 feature_id[64].value <= threshold=3.3956379890441895
node_1202: feature_name=GO:0070102 feature_id[49].value <= threshold=4.805386543273926
node_1203: feature_name=GO:0045553 feature_id[736].value <= threshold=1.4970332980155945
node_1204: feature_name=GO:0051311 feature_id[58].value <= threshold=3.301889181137085
node_1205: feature_name=GO:0030291 feature_id[147].value <= threshold=8.156315326690674
node_1206: feature_name=GO:0045637 feature_id[241].value > threshold=0.01834342861548066

node_1208: feature_name=GO:0002890 feature_id[98].value <= threshold=2.767483353614807
node_1209: feature_name=GO:0046632 feature_id[248].value <= threshold=11.663254261016846
node_1210: feature_name=GO:0008588 feature_id[26].value <= threshold=1.391375720500946
node_1211: feature_name=GO:0010639 feature_id[564].value <= threshold=18.975126266479492
node_1212: feature_name=GO:0046685 feature_id[782].value <= threshold=5.153738260269165
node_1213: feature_name=GO:0038116 feature_id[668].value <= threshold=1.7859655618667603
node_1214: feature_name=GO:0046449 feature_id[763].value <= threshold=5.603115558624268
node_1215: feature_name=GO:0048539 feature_id[319].value <= threshold=0.5478232800960541
node_1216: feature_name=GO:0007089 feature_id[522].value <= threshold=0.9940084517002106
node_1217: feature_name=GO:0042287 feature_id[20].value > threshold=2.693045496940613
node_1263: feature_name=GO:0001816 feature_id[685].value > threshold=18.00125026702881
Class: positive genes

Rules_251 passed counts:3
node_0: feature_name=GO:0042113 feature_id[0].value <= threshold=13.408552169799805
node_1: feature_name=GO:0007568 feature_id[534].value > threshold=5.0313897132873535
node_913: feature_name=GO:0032763 feature_id[629].value > threshold=0.31753237545490265
node_1195: feature_name=GO:0071301 feature_id[442].value <= threshold=0.11534593254327774
node_1196: feature_name=GO:0046500 feature_id[171].value <= threshold=0.6314916908740997
node_1197: feature_name=GO:0030852 feature_id[22].value <= threshold=2.9768868684768677
node_1198: feature_name=hsa04672 feature_id[276].value <= threshold=8.06681227684021
node_1199: feature_name=GO:0001836 feature_id[450].value > threshold=0.09961023926734924
node_1201: feature_name=GO:0070230 feature_id[64].value <= threshold=3.3956379890441895
node_1202: feature_name=GO:0070102 feature_id[49].value <= threshold=4.805386543273926
node_1203: feature_name=GO:0045553 feature_id[736].value <= threshold=1.4970332980155945
node_1204: feature_name=GO:0051311 feature_id[58].value <= threshold=3.301889181137085
node_1205: feature_name=GO:0030291 feature_id[147].value <= threshold=8.156315326690674
node_1206: feature_name=GO:0045637 feature_id[241].value > threshold=0.01834342861548066
node_1208: feature_name=GO:0002890 feature_id[98].value <= threshold=2.767483353614807
node_1209: feature_name=GO:0046632 feature_id[248].value <= threshold=11.663254261016846
node_1210: feature_name=GO:0008588 feature_id[26].value <= threshold=1.391375720500946
node_1211: feature_name=GO:0010639 feature_id[564].value <= threshold=18.975126266479492
node_1212: feature_name=GO:0046685 feature_id[782].value <= threshold=5.153738260269165
node_1213: feature_name=GO:0038116 feature_id[668].value <= threshold=1.7859655618667603
node_1214: feature_name=GO:0046449 feature_id[763].value <= threshold=5.603115558624268

node_1215: feature_name=GO:0048539 feature_id[319].value <= threshold=0.5478232800960541
node_1216: feature_name=GO:0007089 feature_id[522].value <= threshold=0.9940084517002106
node_1217: feature_name=GO:0042287 feature_id[20].value <= threshold=2.693045496940613
node_1218: feature_name=GO:0044446 feature_id[184].value <= threshold=113.0758171081543
node_1219: feature_name=GO:0098602 feature_id[702].value <= threshold=68.95328903198242
node_1220: feature_name=GO:0035726 feature_id[270].value > threshold=1.7167096138000488
node_1258: feature_name=GO:0033689 feature_id[644].value <= threshold=0.709069699048996
Class: positive genes

Rules_252 passed counts:3
node_0: feature_name=GO:0042113 feature_id[0].value <= threshold=13.408552169799805
node_1: feature_name=GO:0007568 feature_id[534].value > threshold=5.0313897132873535
node_913: feature_name=GO:0032763 feature_id[629].value > threshold=0.31753237545490265
node_1195: feature_name=GO:0071301 feature_id[442].value <= threshold=0.11534593254327774
node_1196: feature_name=GO:0046500 feature_id[171].value <= threshold=0.6314916908740997
node_1197: feature_name=GO:0030852 feature_id[22].value <= threshold=2.9768868684768677
node_1198: feature_name=hsa04672 feature_id[276].value <= threshold=8.06681227684021
node_1199: feature_name=GO:0001836 feature_id[450].value > threshold=0.09961023926734924
node_1201: feature_name=GO:0070230 feature_id[64].value <= threshold=3.3956379890441895
node_1202: feature_name=GO:0070102 feature_id[49].value <= threshold=4.805386543273926
node_1203: feature_name=GO:0045553 feature_id[736].value <= threshold=1.4970332980155945
node_1204: feature_name=GO:0051311 feature_id[58].value <= threshold=3.301889181137085
node_1205: feature_name=GO:0030291 feature_id[147].value <= threshold=8.156315326690674
node_1206: feature_name=GO:0045637 feature_id[241].value > threshold=0.01834342861548066
node_1208: feature_name=GO:0002890 feature_id[98].value <= threshold=2.767483353614807
node_1209: feature_name=GO:0046632 feature_id[248].value <= threshold=11.663254261016846
node_1210: feature_name=GO:0008588 feature_id[26].value <= threshold=1.391375720500946
node_1211: feature_name=GO:0010639 feature_id[564].value <= threshold=18.975126266479492
node_1212: feature_name=GO:0046685 feature_id[782].value <= threshold=5.153738260269165
node_1213: feature_name=GO:0038116 feature_id[668].value <= threshold=1.7859655618667603
node_1214: feature_name=GO:0046449 feature_id[763].value <= threshold=5.603115558624268
node_1215: feature_name=GO:0048539 feature_id[319].value <= threshold=0.5478232800960541
node_1216: feature_name=GO:0007089 feature_id[522].value <= threshold=0.9940084517002106
node_1217: feature_name=GO:0042287 feature_id[20].value <= threshold=2.693045496940613
node_1218: feature_name=GO:0044446 feature_id[184].value <= threshold=113.0758171081543

node_1219: feature_name=GO:0098602 feature_id[702].value <= threshold=68.95328903198242
node_1220: feature_name=GO:0035726 feature_id[270].value <= threshold=1.7167096138000488
node_1221: feature_name=GO:1902564 feature_id[85].value <= threshold=2.8364468812942505
node_1222: feature_name=GO:0006979 feature_id[44].value > threshold=0.6869173645973206
node_1224: feature_name=GO:0044464 feature_id[56].value <= threshold=67.13260269165039
node_1225: feature_name=GO:0045629 feature_id[746].value <= threshold=2.3414241075515747
node_1226: feature_name=GO:0002903 feature_id[10].value <= threshold=2.715258240699768
node_1227: feature_name=GO:0043375 feature_id[717].value <= threshold=1.9525017738342285
node_1228: feature_name=GO:0032762 feature_id[630].value > threshold=0.29527929425239563
node_1232: feature_name=GO:0048569 feature_id[793].value <= threshold=3.419509768486023
node_1233: feature_name=GO:0045577 feature_id[109].value > threshold=4.633661270141602
node_1243: feature_name=GO:0006139 feature_id[474].value > threshold=0.37312396615743637
Class: negative genes

Rules_253 passed counts:3
node_0: feature_name=GO:0042113 feature_id[0].value <= threshold=13.408552169799805
node_1: feature_name=GO:0007568 feature_id[534].value > threshold=5.0313897132873535
node_913: feature_name=GO:0032763 feature_id[629].value <= threshold=0.31753237545490265
node_914: feature_name=GO:0097193 feature_id[167].value <= threshold=28.171168327331543
node_915: feature_name=GO:0002903 feature_id[10].value > threshold=1.0534588098526
node_1159: feature_name=GO:0070198 feature_id[665].value > threshold=0.16219981759786606
node_1165: feature_name=GO:0042287 feature_id[20].value <= threshold=0.915093183517456
node_1166: feature_name=GO:0046006 feature_id[364].value > threshold=0.12353959679603577
node_1168: feature_name=GO:0046634 feature_id[46].value <= threshold=4.166545033454895
node_1169: feature_name=GO:0044346 feature_id[724].value <= threshold=0.9571778774261475
node_1170: feature_name=GO:0031060 feature_id[160].value > threshold=1.1418312191963196
Class: negative genes

Rules_254 passed counts:3
node_0: feature_name=GO:0042113 feature_id[0].value <= threshold=13.408552169799805
node_1: feature_name=GO:0007568 feature_id[534].value > threshold=5.0313897132873535
node_913: feature_name=GO:0032763 feature_id[629].value <= threshold=0.31753237545490265
node_914: feature_name=GO:0097193 feature_id[167].value <= threshold=28.171168327331543
node_915: feature_name=GO:0002903 feature_id[10].value > threshold=1.0534588098526
node_1159: feature_name=GO:0070198 feature_id[665].value <= threshold=0.16219981759786606

node_1160: feature_name=GO:0019692 feature_id[584].value > threshold=1.4449326992034912
Class: positive genes

Rules_255 passed counts:3
node_0: feature_name=GO:0042113 feature_id[0].value <= threshold=13.408552169799805
node_1: feature_name=GO:0007568 feature_id[534].value > threshold=5.0313897132873535
node_913: feature_name=GO:0032763 feature_id[629].value <= threshold=0.31753237545490265
node_914: feature_name=GO:0097193 feature_id[167].value <= threshold=28.171168327331543
node_915: feature_name=GO:0002903 feature_id[10].value <= threshold=1.0534588098526
node_916: feature_name=GO:1904029 feature_id[208].value <= threshold=23.852136611938477
node_917: feature_name=GO:0050897 feature_id[164].value > threshold=0.6870408356189728
node_1095: feature_name=GO:0072539 feature_id[68].value <= threshold=2.5667877197265625
node_1096: feature_name=GO:0046500 feature_id[171].value <= threshold=3.195197820663452
node_1097: feature_name=GO:0045861 feature_id[761].value > threshold=0.22614753991365433
node_1099: feature_name=GO:0043372 feature_id[242].value > threshold=4.892506122589111
Class: positive genes

Rules_256 passed counts:3
node_0: feature_name=GO:0042113 feature_id[0].value <= threshold=13.408552169799805
node_1: feature_name=GO:0007568 feature_id[534].value > threshold=5.0313897132873535
node_913: feature_name=GO:0032763 feature_id[629].value <= threshold=0.31753237545490265
node_914: feature_name=GO:0097193 feature_id[167].value <= threshold=28.171168327331543
node_915: feature_name=GO:0002903 feature_id[10].value <= threshold=1.0534588098526
node_916: feature_name=GO:1904029 feature_id[208].value <= threshold=23.852136611938477
node_917: feature_name=GO:0050897 feature_id[164].value > threshold=0.6870408356189728
node_1095: feature_name=GO:0072539 feature_id[68].value <= threshold=2.5667877197265625
node_1096: feature_name=GO:0046500 feature_id[171].value <= threshold=3.195197820663452
node_1097: feature_name=GO:0045861 feature_id[761].value > threshold=0.22614753991365433
node_1099: feature_name=GO:0043372 feature_id[242].value <= threshold=4.892506122589111
node_1100: feature_name=GO:0042162 feature_id[355].value <= threshold=11.339290618896484
node_1101: feature_name=hsa04660 feature_id[472].value <= threshold=24.27848720550537
node_1102: feature_name=GO:0007049 feature_id[259].value <= threshold=209.23831176757812
node_1103: feature_name=GO:0050897 feature_id[164].value > threshold=0.6919849812984467
node_1105: feature_name=GO:0045945 feature_id[267].value <= threshold=3.307852625846863
node_1106: feature_name=GO:0046641 feature_id[773].value <= threshold=3.527579426765442

node_1107: feature_name=GO:0002262 feature_id[394].value > threshold=0.08302562311291695
node_1111: feature_name=GO:0043226 feature_id[138].value > threshold=2.4930146992119262e-05
node_1113: feature_name=GO:0045787 feature_id[446].value > threshold=0.07611752673983574
node_1115: feature_name=GO:0045840 feature_id[697].value > threshold=0.10580676794052124
node_1117: feature_name=GO:0006927 feature_id[225].value <= threshold=4.950021743774414
node_1118: feature_name=GO:0002643 feature_id[4].value <= threshold=3.364396333694458
node_1119: feature_name=GO:0016571 feature_id[725].value <= threshold=38.268903732299805
node_1120: feature_name=GO:0072593 feature_id[381].value > threshold=19.997477531433105
node_1128: feature_name=GO:0006304 feature_id[510].value > threshold=0.7586463987827301
Class: positive genes

Rules_257 passed counts:3
node_0: feature_name=GO:0042113 feature_id[0].value <= threshold=13.408552169799805
node_1: feature_name=GO:0007568 feature_id[534].value > threshold=5.0313897132873535
node_913: feature_name=GO:0032763 feature_id[629].value <= threshold=0.31753237545490265
node_914: feature_name=GO:0097193 feature_id[167].value <= threshold=28.171168327331543
node_915: feature_name=GO:0002903 feature_id[10].value <= threshold=1.0534588098526
node_916: feature_name=GO:1904029 feature_id[208].value <= threshold=23.852136611938477
node_917: feature_name=GO:0050897 feature_id[164].value > threshold=0.6870408356189728
node_1095: feature_name=GO:0072539 feature_id[68].value <= threshold=2.5667877197265625
node_1096: feature_name=GO:0046500 feature_id[171].value <= threshold=3.195197820663452
node_1097: feature_name=GO:0045861 feature_id[761].value <= threshold=0.22614753991365433
Class: positive genes

Rules_258 passed counts:3
node_0: feature_name=GO:0042113 feature_id[0].value <= threshold=13.408552169799805
node_1: feature_name=GO:0007568 feature_id[534].value > threshold=5.0313897132873535
node_913: feature_name=GO:0032763 feature_id[629].value <= threshold=0.31753237545490265
node_914: feature_name=GO:0097193 feature_id[167].value <= threshold=28.171168327331543
node_915: feature_name=GO:0002903 feature_id[10].value <= threshold=1.0534588098526
node_916: feature_name=GO:1904029 feature_id[208].value <= threshold=23.852136611938477
node_917: feature_name=GO:0050897 feature_id[164].value <= threshold=0.6870408356189728
node_918: feature_name=GO:0006139 feature_id[474].value > threshold=1.3052097624921544e-07
node_920: feature_name=GO:0002821 feature_id[588].value <= threshold=13.81072187423706
node_921: feature_name=GO:0006298 feature_id[621].value <= threshold=24.078600883483887

node_922: feature_name=GO:0003908 feature_id[11].value > threshold=1.7776933312416077
node_1090: feature_name=GO:0046685 feature_id[782].value > threshold=0.9642224758863449
Class: positive genes

Rules_259 passed counts:3
node_0: feature_name=GO:0042113 feature_id[0].value <= threshold=13.408552169799805
node_1: feature_name=GO:0007568 feature_id[534].value > threshold=5.0313897132873535
node_913: feature_name=GO:0032763 feature_id[629].value <= threshold=0.31753237545490265
node_914: feature_name=GO:0097193 feature_id[167].value <= threshold=28.171168327331543
node_915: feature_name=GO:0002903 feature_id[10].value <= threshold=1.0534588098526
node_916: feature_name=GO:1904029 feature_id[208].value <= threshold=23.852136611938477
node_917: feature_name=GO:0050897 feature_id[164].value <= threshold=0.6870408356189728
node_918: feature_name=GO:0006139 feature_id[474].value > threshold=1.3052097624921544e-07
node_920: feature_name=GO:0002821 feature_id[588].value <= threshold=13.81072187423706
node_921: feature_name=GO:0006298 feature_id[621].value <= threshold=24.078600883483887
node_922: feature_name=GO:0003908 feature_id[11].value <= threshold=1.7776933312416077
node_923: feature_name=GO:0030098 feature_id[273].value > threshold=0.0079949083738029
node_927: feature_name=GO:0006808 feature_id[513].value <= threshold=4.34592080116272
node_928: feature_name=GO:0071887 feature_id[283].value <= threshold=10.353787899017334
node_929: feature_name=GO:0038001 feature_id[28].value <= threshold=3.9469382762908936
node_930: feature_name=GO:0042287 feature_id[20].value <= threshold=3.6209195852279663
node_931: feature_name=GO:0003968 feature_id[47].value <= threshold=2.004227638244629
node_932: feature_name=GO:0002698 feature_id[395].value <= threshold=18.51447582244873
node_933: feature_name=GO:0044710 feature_id[719].value <= threshold=179.7340316772461
node_934: feature_name=GO:0007568 feature_id[534].value > threshold=5.035318374633789
node_936: feature_name=GO:0006216 feature_id[504].value <= threshold=2.2605666518211365
node_937: feature_name=GO:0048569 feature_id[793].value <= threshold=5.340231895446777
node_938: feature_name=GO:0001777 feature_id[380].value <= threshold=4.100832939147949
node_939: feature_name=GO:0007600 feature_id[122].value <= threshold=171.13383102416992
node_940: feature_name=GO:0001779 feature_id[378].value <= threshold=6.234851121902466
node_941: feature_name=GO:0030291 feature_id[147].value <= threshold=10.8051118850708
node_942: feature_name=GO:0048534 feature_id[790].value > threshold=0.017984486185014248
node_944: feature_name=GO:0070245 feature_id[326].value > threshold=3.1282339096069336
node_1066: feature_name=GO:0070227 feature_id[57].value > threshold=2.323003053665161
Class: negative genes

Rules_260	passed counts:3
node_0: feature_name=GO:0042113	feature_id[0].value <= threshold=13.408552169799805
node_1: feature_name=GO:0007568	feature_id[534].value > threshold=5.0313897132873535
node_913: feature_name=GO:0032763	feature_id[629].value <= threshold=0.31753237545490265
node_914: feature_name=GO:0097193	feature_id[167].value <= threshold=28.171168327331543
node_915: feature_name=GO:0002903	feature_id[10].value <= threshold=1.0534588098526
node_916: feature_name=GO:1904029	feature_id[208].value <= threshold=23.852136611938477
node_917: feature_name=GO:0050897	feature_id[164].value <= threshold=0.6870408356189728
node_918: feature_name=GO:0006139	feature_id[474].value > threshold=1.3052097624921544e-07
node_920: feature_name=GO:0002821	feature_id[588].value <= threshold=13.81072187423706
node_921: feature_name=GO:0006298	feature_id[621].value <= threshold=24.078600883483887
node_922: feature_name=GO:0003908	feature_id[11].value <= threshold=1.7776933312416077
node_923: feature_name=GO:0030098	feature_id[273].value > threshold=0.0079949083738029
node_927: feature_name=GO:0006808	feature_id[513].value <= threshold=4.34592080116272
node_928: feature_name=GO:0071887	feature_id[283].value <= threshold=10.353787899017334
node_929: feature_name=GO:0038001	feature_id[28].value <= threshold=3.9469382762908936
node_930: feature_name=GO:0042287	feature_id[20].value <= threshold=3.6209195852279663
node_931: feature_name=GO:0003968	feature_id[47].value <= threshold=2.004227638244629
node_932: feature_name=GO:0002698	feature_id[395].value <= threshold=18.51447582244873
node_933: feature_name=GO:0044710	feature_id[719].value <= threshold=179.7340316772461
node_934: feature_name=GO:0007568	feature_id[534].value > threshold=5.035318374633789
node_936: feature_name=GO:0006216	feature_id[504].value <= threshold=2.2605666518211365
node_937: feature_name=GO:0048569	feature_id[793].value <= threshold=5.340231895446777
node_938: feature_name=GO:0001777	feature_id[380].value <= threshold=4.100832939147949
node_939: feature_name=GO:0007600	feature_id[122].value <= threshold=171.13383102416992
node_940: feature_name=GO:0001779	feature_id[378].value <= threshold=6.234851121902466
node_941: feature_name=GO:0030291	feature_id[147].value <= threshold=10.8051118850708
node_942: feature_name=GO:0048534	feature_id[790].value > threshold=0.017984486185014248
node_944: feature_name=GO:0070245	feature_id[326].value <= threshold=3.1282339096069336
node_945: feature_name=GO:0009086	feature_id[547].value > threshold=0.2896959036588669
node_1017: feature_name=GO:0000302	feature_id[375].value > threshold=0.305715873837471
node_1019: feature_name=GO:0070141	feature_id[37].value > threshold=4.857294321060181
node_1063: feature_name=GO:0002292	feature_id[352].value <= threshold=0.9737851917743683
Class: positive genes	

Rules_261	passed counts:3
node_0: feature_name=GO:0042113	feature_id[0].value <= threshold=13.408552169799805
node_1: feature_name=GO:0007568	feature_id[534].value > threshold=5.0313897132873535
node_913: feature_name=GO:0032763	feature_id[629].value <= threshold=0.31753237545490265
node_914: feature_name=GO:0097193	feature_id[167].value <= threshold=28.171168327331543
node_915: feature_name=GO:0002903	feature_id[10].value <= threshold=1.0534588098526
node_916: feature_name=GO:1904029	feature_id[208].value <= threshold=23.852136611938477
node_917: feature_name=GO:0050897	feature_id[164].value <= threshold=0.6870408356189728
node_918: feature_name=GO:0006139	feature_id[474].value > threshold=1.3052097624921544e-07
node_920: feature_name=GO:0002821	feature_id[588].value <= threshold=13.81072187423706
node_921: feature_name=GO:0006298	feature_id[621].value <= threshold=24.078600883483887
node_922: feature_name=GO:0003908	feature_id[11].value <= threshold=1.7776933312416077
node_923: feature_name=GO:0030098	feature_id[273].value > threshold=0.0079949083738029
node_927: feature_name=GO:0006808	feature_id[513].value <= threshold=4.34592080116272
node_928: feature_name=GO:0071887	feature_id[283].value <= threshold=10.353787899017334
node_929: feature_name=GO:0038001	feature_id[28].value <= threshold=3.9469382762908936
node_930: feature_name=GO:0042287	feature_id[20].value <= threshold=3.6209195852279663
node_931: feature_name=GO:0003968	feature_id[47].value <= threshold=2.004227638244629
node_932: feature_name=GO:0002698	feature_id[395].value <= threshold=18.51447582244873
node_933: feature_name=GO:0044710	feature_id[719].value <= threshold=179.7340316772461
node_934: feature_name=GO:0007568	feature_id[534].value > threshold=5.035318374633789
node_936: feature_name=GO:0006216	feature_id[504].value <= threshold=2.2605666518211365
node_937: feature_name=GO:0048569	feature_id[793].value <= threshold=5.340231895446777
node_938: feature_name=GO:0001777	feature_id[380].value <= threshold=4.100832939147949
node_939: feature_name=GO:0007600	feature_id[122].value <= threshold=171.13383102416992
node_940: feature_name=GO:0001779	feature_id[378].value <= threshold=6.234851121902466
node_941: feature_name=GO:0030291	feature_id[147].value <= threshold=10.8051118850708
node_942: feature_name=GO:0048534	feature_id[790].value > threshold=0.017984486185014248
node_944: feature_name=GO:0070245	feature_id[326].value <= threshold=3.1282339096069336
node_945: feature_name=GO:0009086	feature_id[547].value <= threshold=0.2896959036588669
node_946: feature_name=GO:0001889	feature_id[387].value <= threshold=17.643128395080566
node_947: feature_name=GO:0048144	feature_id[192].value > threshold=0.20887330174446106
node_951: feature_name=GO:0023026	feature_id[69].value <= threshold=3.403424024581909
node_952: feature_name=GO:0048147	feature_id[788].value > threshold=4.188283443450928

node_1010: feature_name=GO:0016363 feature_id[90].value <= threshold=1.0286503434181213
Class: positive genes

Rules_262 passed counts:3
node_0: feature_name=GO:0042113 feature_id[0].value <= threshold=13.408552169799805
node_1: feature_name=GO:0007568 feature_id[534].value > threshold=5.0313897132873535
node_913: feature_name=GO:0032763 feature_id[629].value <= threshold=0.31753237545490265
node_914: feature_name=GO:0097193 feature_id[167].value <= threshold=28.171168327331543
node_915: feature_name=GO:0002903 feature_id[10].value <= threshold=1.0534588098526
node_916: feature_name=GO:1904029 feature_id[208].value <= threshold=23.852136611938477
node_917: feature_name=GO:0050897 feature_id[164].value <= threshold=0.6870408356189728
node_918: feature_name=GO:0006139 feature_id[474].value > threshold=1.3052097624921544e-07
node_920: feature_name=GO:0002821 feature_id[588].value <= threshold=13.81072187423706
node_921: feature_name=GO:0006298 feature_id[621].value <= threshold=24.078600883483887
node_922: feature_name=GO:0003908 feature_id[11].value <= threshold=1.7776933312416077
node_923: feature_name=GO:0030098 feature_id[273].value > threshold=0.0079949083738029
node_927: feature_name=GO:0006808 feature_id[513].value <= threshold=4.34592080116272
node_928: feature_name=GO:0071887 feature_id[283].value <= threshold=10.353787899017334
node_929: feature_name=GO:0038001 feature_id[28].value <= threshold=3.9469382762908936
node_930: feature_name=GO:0042287 feature_id[20].value <= threshold=3.6209195852279663
node_931: feature_name=GO:0003968 feature_id[47].value <= threshold=2.004227638244629
node_932: feature_name=GO:0002698 feature_id[395].value <= threshold=18.51447582244873
node_933: feature_name=GO:0044710 feature_id[719].value <= threshold=179.7340316772461
node_934: feature_name=GO:0007568 feature_id[534].value > threshold=5.035318374633789
node_936: feature_name=GO:0006216 feature_id[504].value <= threshold=2.2605666518211365
node_937: feature_name=GO:0048569 feature_id[793].value <= threshold=5.340231895446777
node_938: feature_name=GO:0001777 feature_id[380].value <= threshold=4.100832939147949
node_939: feature_name=GO:0007600 feature_id[122].value <= threshold=171.13383102416992
node_940: feature_name=GO:0001779 feature_id[378].value <= threshold=6.234851121902466
node_941: feature_name=GO:0030291 feature_id[147].value <= threshold=10.8051118850708
node_942: feature_name=GO:0048534 feature_id[790].value > threshold=0.017984486185014248
node_944: feature_name=GO:0070245 feature_id[326].value <= threshold=3.1282339096069336
node_945: feature_name=GO:0009086 feature_id[547].value <= threshold=0.2896959036588669
node_946: feature_name=GO:0001889 feature_id[387].value <= threshold=17.643128395080566
node_947: feature_name=GO:0048144 feature_id[192].value <= threshold=0.20887330174446106

node_948: feature_name=GO:0042110 feature_id[193].value > threshold=2.3200501203536987
Class: negative genes

Rules_263 passed counts:3
node_0: feature_name=GO:0042113 feature_id[0].value <= threshold=13.408552169799805
node_1: feature_name=GO:0007568 feature_id[534].value > threshold=5.0313897132873535
node_913: feature_name=GO:0032763 feature_id[629].value <= threshold=0.31753237545490265
node_914: feature_name=GO:0097193 feature_id[167].value <= threshold=28.171168327331543
node_915: feature_name=GO:0002903 feature_id[10].value <= threshold=1.0534588098526
node_916: feature_name=GO:1904029 feature_id[208].value <= threshold=23.852136611938477
node_917: feature_name=GO:0050897 feature_id[164].value <= threshold=0.6870408356189728
node_918: feature_name=GO:0006139 feature_id[474].value > threshold=1.3052097624921544e-07
node_920: feature_name=GO:0002821 feature_id[588].value <= threshold=13.81072187423706
node_921: feature_name=GO:0006298 feature_id[621].value <= threshold=24.078600883483887
node_922: feature_name=GO:0003908 feature_id[11].value <= threshold=1.7776933312416077
node_923: feature_name=GO:0030098 feature_id[273].value <= threshold=0.0079949083738029
node_924: feature_name=GO:0001773 feature_id[308].value > threshold=0.09269188344478607
Class: negative genes

Rules_264 passed counts:3
node_0: feature_name=GO:0042113 feature_id[0].value <= threshold=13.408552169799805
node_1: feature_name=GO:0007568 feature_id[534].value > threshold=5.0313897132873535
node_913: feature_name=GO:0032763 feature_id[629].value <= threshold=0.31753237545490265
node_914: feature_name=GO:0097193 feature_id[167].value <= threshold=28.171168327331543
node_915: feature_name=GO:0002903 feature_id[10].value <= threshold=1.0534588098526
node_916: feature_name=GO:1904029 feature_id[208].value <= threshold=23.852136611938477
node_917: feature_name=GO:0050897 feature_id[164].value <= threshold=0.6870408356189728
node_918: feature_name=GO:0006139 feature_id[474].value > threshold=1.3052097624921544e-07
node_920: feature_name=GO:0002821 feature_id[588].value <= threshold=13.81072187423706
node_921: feature_name=GO:0006298 feature_id[621].value <= threshold=24.078600883483887
node_922: feature_name=GO:0003908 feature_id[11].value <= threshold=1.7776933312416077
node_923: feature_name=GO:0030098 feature_id[273].value <= threshold=0.0079949083738029
node_924: feature_name=GO:0001773 feature_id[308].value <= threshold=0.09269188344478607
Class: positive genes

Rules_265 passed counts:3
node_0: feature_name=GO:0042113 feature_id[0].value <= threshold=13.408552169799805
node_1: feature_name=GO:0007568 feature_id[534].value <= threshold=5.0313897132873535
node_2: feature_name=GO:0002705 feature_id[541].value > threshold=3.200145721435547
node_870: feature_name=GO:0046634 feature_id[46].value <= threshold=8.9394211769104
node_871: feature_name=GO:0048523 feature_id[293].value <= threshold=19.963229179382324
node_872: feature_name=GO:2001237 feature_id[796].value > threshold=0.09761488810181618
node_880: feature_name=GO:0006808 feature_id[513].value > threshold=1.7969900965690613
Class: positive genes

Rules_266 passed counts:3
node_0: feature_name=GO:0042113 feature_id[0].value <= threshold=13.408552169799805
node_1: feature_name=GO:0007568 feature_id[534].value <= threshold=5.0313897132873535
node_2: feature_name=GO:0002705 feature_id[541].value <= threshold=3.200145721435547
node_3: feature_name=GO:1901525 feature_id[576].value > threshold=0.4466460347175598
node_617: feature_name=GO:0005622 feature_id[233].value > threshold=90.85730743408203
node_847: feature_name=GO:0002639 feature_id[466].value <= threshold=0.32853202521800995
node_848: feature_name=GO:0002636 feature_id[447].value <= threshold=0.3303467631340027
node_849: feature_name=GO:0001783 feature_id[33].value <= threshold=0.07426292821764946
Class: positive genes

Rules_267 passed counts:3
node_0: feature_name=GO:0042113 feature_id[0].value <= threshold=13.408552169799805
node_1: feature_name=GO:0007568 feature_id[534].value <= threshold=5.0313897132873535
node_2: feature_name=GO:0002705 feature_id[541].value <= threshold=3.200145721435547
node_3: feature_name=GO:1901525 feature_id[576].value > threshold=0.4466460347175598
node_617: feature_name=GO:0005622 feature_id[233].value <= threshold=90.85730743408203
node_618: feature_name=GO:1903147 feature_id[200].value > threshold=7.326428413391113
Class: positive genes

Rules_268 passed counts:3
node_0: feature_name=GO:0042113 feature_id[0].value <= threshold=13.408552169799805
node_1: feature_name=GO:0007568 feature_id[534].value <= threshold=5.0313897132873535
node_2: feature_name=GO:0002705 feature_id[541].value <= threshold=3.200145721435547
node_3: feature_name=GO:1901525 feature_id[576].value > threshold=0.4466460347175598

node_617: feature_name=GO:0005622 feature_id[233].value <= threshold=90.85730743408203
node_618: feature_name=GO:1903147 feature_id[200].value <= threshold=7.326428413391113
node_619: feature_name=GO:0003964 feature_id[317].value > threshold=5.575642108917236
node_843: feature_name=GO:0002293 feature_id[206].value <= threshold=0.668750561773777
Class: positive genes

Rules_269 passed counts:3
node_0: feature_name=GO:0042113 feature_id[0].value <= threshold=13.408552169799805
node_1: feature_name=GO:0007568 feature_id[534].value <= threshold=5.0313897132873535
node_2: feature_name=GO:0002705 feature_id[541].value <= threshold=3.200145721435547
node_3: feature_name=GO:1901525 feature_id[576].value > threshold=0.4466460347175598
node_617: feature_name=GO:0005622 feature_id[233].value <= threshold=90.85730743408203
node_618: feature_name=GO:1903147 feature_id[200].value <= threshold=7.326428413391113
node_619: feature_name=GO:0003964 feature_id[317].value <= threshold=5.575642108917236
node_620: feature_name=GO:0046498 feature_id[203].value > threshold=6.620223522186279
node_840: feature_name=GO:0015672 feature_id[573].value > threshold=0.3655537310987711
Class: positive genes

Rules_270 passed counts:3
node_0: feature_name=GO:0042113 feature_id[0].value <= threshold=13.408552169799805
node_1: feature_name=GO:0007568 feature_id[534].value <= threshold=5.0313897132873535
node_2: feature_name=GO:0002705 feature_id[541].value <= threshold=3.200145721435547
node_3: feature_name=GO:1901525 feature_id[576].value > threshold=0.4466460347175598
node_617: feature_name=GO:0005622 feature_id[233].value <= threshold=90.85730743408203
node_618: feature_name=GO:1903147 feature_id[200].value <= threshold=7.326428413391113
node_619: feature_name=GO:0003964 feature_id[317].value <= threshold=5.575642108917236
node_620: feature_name=GO:0046498 feature_id[203].value <= threshold=6.620223522186279
node_621: feature_name=GO:0002562 feature_id[25].value <= threshold=10.287704467773438
node_622: feature_name=GO:0006919 feature_id[246].value <= threshold=5.801334381103516
node_623: feature_name=GO:0001889 feature_id[387].value > threshold=2.679414987564087
node_785: feature_name=GO:0030887 feature_id[274].value <= threshold=1.1379476189613342
node_786: feature_name=GO:1902564 feature_id[85].value > threshold=1.4637635350227356
Class: positive genes

Rules_271 passed counts:3

node_0: feature_name=GO:0042113 feature_id[0].value <= threshold=13.408552169799805
node_1: feature_name=GO:0007568 feature_id[534].value <= threshold=5.0313897132873535
node_2: feature_name=GO:0002705 feature_id[541].value <= threshold=3.200145721435547
node_3: feature_name=GO:1901525 feature_id[576].value > threshold=0.4466460347175598
node_617: feature_name=GO:0005622 feature_id[233].value <= threshold=90.85730743408203
node_618: feature_name=GO:1903147 feature_id[200].value <= threshold=7.326428413391113
node_619: feature_name=GO:0003964 feature_id[317].value <= threshold=5.575642108917236
node_620: feature_name=GO:0046498 feature_id[203].value <= threshold=6.620223522186279
node_621: feature_name=GO:0002562 feature_id[25].value <= threshold=10.287704467773438
node_622: feature_name=GO:0006919 feature_id[246].value <= threshold=5.801334381103516
node_623: feature_name=GO:0001889 feature_id[387].value <= threshold=2.679414987564087
node_624: feature_name=GO:0006555 feature_id[514].value <= threshold=7.819535493850708
node_625: feature_name=GO:0080134 feature_id[650].value <= threshold=38.91967582702637
node_626: feature_name=GO:0042127 feature_id[480].value <= threshold=37.72536659240723
node_627: feature_name=GO:2001251 feature_id[220].value <= threshold=32.564422607421875
node_628: feature_name=GO:0002829 feature_id[493].value <= threshold=2.444612741470337
node_629: feature_name=GO:0008588 feature_id[26].value <= threshold=2.3601274490356445
node_630: feature_name=GO:0033151 feature_id[7].value <= threshold=5.134376287460327
node_631: feature_name=GO:0048145 feature_id[348].value <= threshold=8.836549282073975
node_632: feature_name=GO:0030887 feature_id[274].value <= threshold=1.7000296115875244
node_633: feature_name=GO:0097190 feature_id[117].value <= threshold=33.02078819274902
node_634: feature_name=GO:0046685 feature_id[782].value <= threshold=8.040813446044922
node_635: feature_name=GO:0006346 feature_id[236].value <= threshold=6.384642839431763
node_636: feature_name=GO:0048537 feature_id[66].value <= threshold=3.1739262342453003
node_637: feature_name=GO:0070245 feature_id[326].value > threshold=3.0491198301315308
node_765: feature_name=GO:0045429 feature_id[737].value > threshold=1.1315002739429474
Class: negative genes

Rules_272 passed counts:3
node_0: feature_name=GO:0042113 feature_id[0].value <= threshold=13.408552169799805
node_1: feature_name=GO:0007568 feature_id[534].value <= threshold=5.0313897132873535
node_2: feature_name=GO:0002705 feature_id[541].value <= threshold=3.200145721435547
node_3: feature_name=GO:1901525 feature_id[576].value > threshold=0.4466460347175598
node_617: feature_name=GO:0005622 feature_id[233].value <= threshold=90.85730743408203
node_618: feature_name=GO:1903147 feature_id[200].value <= threshold=7.326428413391113

node_619: feature_name=GO:0003964 feature_id[317].value <= threshold=5.575642108917236
node_620: feature_name=GO:0046498 feature_id[203].value <= threshold=6.620223522186279
node_621: feature_name=GO:0002562 feature_id[25].value <= threshold=10.287704467773438
node_622: feature_name=GO:0006919 feature_id[246].value <= threshold=5.801334381103516
node_623: feature_name=GO:0001889 feature_id[387].value <= threshold=2.679414987564087
node_624: feature_name=GO:0006555 feature_id[514].value <= threshold=7.819535493850708
node_625: feature_name=GO:0080134 feature_id[650].value <= threshold=38.91967582702637
node_626: feature_name=GO:0042127 feature_id[480].value <= threshold=37.72536659240723
node_627: feature_name=GO:2001251 feature_id[220].value <= threshold=32.564422607421875
node_628: feature_name=GO:0002829 feature_id[493].value <= threshold=2.444612741470337
node_629: feature_name=GO:0008588 feature_id[26].value <= threshold=2.3601274490356445
node_630: feature_name=GO:0033151 feature_id[7].value <= threshold=5.134376287460327
node_631: feature_name=GO:0048145 feature_id[348].value <= threshold=8.836549282073975
node_632: feature_name=GO:0030887 feature_id[274].value <= threshold=1.7000296115875244
node_633: feature_name=GO:0097190 feature_id[117].value <= threshold=33.02078819274902
node_634: feature_name=GO:0046685 feature_id[782].value <= threshold=8.040813446044922
node_635: feature_name=GO:0006346 feature_id[236].value <= threshold=6.384642839431763
node_636: feature_name=GO:0048537 feature_id[66].value <= threshold=3.1739262342453003
node_637: feature_name=GO:0070245 feature_id[326].value <= threshold=3.0491198301315308
node_638: feature_name=GO:0038065 feature_id[670].value <= threshold=2.0590850114822388
node_639: feature_name=GO:0005488 feature_id[187].value > threshold=84.80076217651367
node_755: feature_name=GO:0031049 feature_id[5].value > threshold=0.707693487405777
node_759: feature_name=GO:0002832 feature_id[491].value > threshold=0.07645885832607746
Class: positive genes

Rules_273 passed counts:3
node_0: feature_name=GO:0042113 feature_id[0].value <= threshold=13.408552169799805
node_1: feature_name=GO:0007568 feature_id[534].value <= threshold=5.0313897132873535
node_2: feature_name=GO:0002705 feature_id[541].value <= threshold=3.200145721435547
node_3: feature_name=GO:1901525 feature_id[576].value > threshold=0.4466460347175598
node_617: feature_name=GO:0005622 feature_id[233].value <= threshold=90.85730743408203
node_618: feature_name=GO:1903147 feature_id[200].value <= threshold=7.326428413391113
node_619: feature_name=GO:0003964 feature_id[317].value <= threshold=5.575642108917236
node_620: feature_name=GO:0046498 feature_id[203].value <= threshold=6.620223522186279
node_621: feature_name=GO:0002562 feature_id[25].value <= threshold=10.287704467773438

node_622: feature_name=GO:0006919	feature_id[246].value <= threshold=5.801334381103516
node_623: feature_name=GO:0001889	feature_id[387].value <= threshold=2.679414987564087
node_624: feature_name=GO:0006555	feature_id[514].value <= threshold=7.819535493850708
node_625: feature_name=GO:0080134	feature_id[650].value <= threshold=38.91967582702637
node_626: feature_name=GO:0042127	feature_id[480].value <= threshold=37.72536659240723
node_627: feature_name=GO:2001251	feature_id[220].value <= threshold=32.564422607421875
node_628: feature_name=GO:0002829	feature_id[493].value <= threshold=2.444612741470337
node_629: feature_name=GO:0008588	feature_id[26].value <= threshold=2.3601274490356445
node_630: feature_name=GO:0033151	feature_id[7].value <= threshold=5.134376287460327
node_631: feature_name=GO:0048145	feature_id[348].value <= threshold=8.836549282073975
node_632: feature_name=GO:0030887	feature_id[274].value <= threshold=1.7000296115875244
node_633: feature_name=GO:0097190	feature_id[117].value <= threshold=33.02078819274902
node_634: feature_name=GO:0046685	feature_id[782].value <= threshold=8.040813446044922
node_635: feature_name=GO:0006346	feature_id[236].value <= threshold=6.384642839431763
node_636: feature_name=GO:0048537	feature_id[66].value <= threshold=3.1739262342453003
node_637: feature_name=GO:0070245	feature_id[326].value <= threshold=3.0491198301315308
node_638: feature_name=GO:0038065	feature_id[670].value <= threshold=2.0590850114822388
node_639: feature_name=GO:0005488	feature_id[187].value <= threshold=84.80076217651367
node_640: feature_name=GO:0060249	feature_id[390].value > threshold=0.07226398587226868
node_652: feature_name=GO:0006974	feature_id[516].value > threshold=5.17782768838515e-06
node_654: feature_name=hsa05210	feature_id[358].value <= threshold=18.730005264282227
node_655: feature_name=GO:0071850	feature_id[40].value <= threshold=5.475317001342773
node_656: feature_name=GO:1904029	feature_id[208].value <= threshold=26.575013160705566
node_657: feature_name=GO:0090594	feature_id[738].value <= threshold=3.000791072845459
node_658: feature_name=GO:0010948	feature_id[569].value > threshold=0.04386143572628498
node_666: feature_name=GO:0032764	feature_id[3].value <= threshold=2.5201677083969116
node_667: feature_name=GO:0023026	feature_id[69].value <= threshold=3.2760006189346313
node_668: feature_name=GO:0032464	feature_id[83].value <= threshold=2.5927021503448486
node_669: feature_name=GO:0071901	feature_id[407].value <= threshold=18.238012313842773
node_670: feature_name=GO:0015671	feature_id[103].value <= threshold=1.3113192915916443
node_671: feature_name=GO:0008340	feature_id[539].value <= threshold=4.860779762268066
node_672: feature_name=GO:0046006	feature_id[364].value > threshold=2.4317692518234253
node_716: feature_name=GO:0030155	feature_id[594].value > threshold=0.13681869907304645

Class: negative genes

Rules_274	passed counts:3
node_0: feature_name=GO:0042113	feature_id[0].value <= threshold=13.408552169799805
node_1: feature_name=GO:0007568	feature_id[534].value <= threshold=5.0313897132873535
node_2: feature_name=GO:0002705	feature_id[541].value <= threshold=3.200145721435547
node_3: feature_name=GO:1901525	feature_id[576].value > threshold=0.4466460347175598
node_617: feature_name=GO:0005622	feature_id[233].value <= threshold=90.85730743408203
node_618: feature_name=GO:1903147	feature_id[200].value <= threshold=7.326428413391113
node_619: feature_name=GO:0003964	feature_id[317].value <= threshold=5.575642108917236
node_620: feature_name=GO:0046498	feature_id[203].value <= threshold=6.620223522186279
node_621: feature_name=GO:0002562	feature_id[25].value <= threshold=10.287704467773438
node_622: feature_name=GO:0006919	feature_id[246].value <= threshold=5.801334381103516
node_623: feature_name=GO:0001889	feature_id[387].value <= threshold=2.679414987564087
node_624: feature_name=GO:0006555	feature_id[514].value <= threshold=7.819535493850708
node_625: feature_name=GO:0080134	feature_id[650].value <= threshold=38.91967582702637
node_626: feature_name=GO:0042127	feature_id[480].value <= threshold=37.72536659240723
node_627: feature_name=GO:2001251	feature_id[220].value <= threshold=32.564422607421875
node_628: feature_name=GO:0002829	feature_id[493].value <= threshold=2.444612741470337
node_629: feature_name=GO:0008588	feature_id[26].value <= threshold=2.3601274490356445
node_630: feature_name=GO:0033151	feature_id[7].value <= threshold=5.134376287460327
node_631: feature_name=GO:0048145	feature_id[348].value <= threshold=8.836549282073975
node_632: feature_name=GO:0030887	feature_id[274].value <= threshold=1.7000296115875244
node_633: feature_name=GO:0097190	feature_id[117].value <= threshold=33.02078819274902
node_634: feature_name=GO:0046685	feature_id[782].value <= threshold=8.040813446044922
node_635: feature_name=GO:0006346	feature_id[236].value <= threshold=6.384642839431763
node_636: feature_name=GO:0048537	feature_id[66].value <= threshold=3.1739262342453003
node_637: feature_name=GO:0070245	feature_id[326].value <= threshold=3.0491198301315308
node_638: feature_name=GO:0038065	feature_id[670].value <= threshold=2.0590850114822388
node_639: feature_name=GO:0005488	feature_id[187].value <= threshold=84.80076217651367
node_640: feature_name=GO:0060249	feature_id[390].value > threshold=0.07226398587226868
node_652: feature_name=GO:0006974	feature_id[516].value > threshold=5.17782768838515e-06
node_654: feature_name=hsa05210	feature_id[358].value <= threshold=18.730005264282227
node_655: feature_name=GO:0071850	feature_id[40].value <= threshold=5.475317001342773
node_656: feature_name=GO:1904029	feature_id[208].value <= threshold=26.575013160705566
node_657: feature_name=GO:0090594	feature_id[738].value <= threshold=3.000791072845459
node_658: feature_name=GO:0010948	feature_id[569].value > threshold=0.04386143572628498

node_666: feature_name=GO:0032764 feature_id[3].value <= threshold=2.5201677083969116
node_667: feature_name=GO:0023026 feature_id[69].value <= threshold=3.2760006189346313
node_668: feature_name=GO:0032464 feature_id[83].value <= threshold=2.5927021503448486
node_669: feature_name=GO:0071901 feature_id[407].value <= threshold=18.238012313842773
node_670: feature_name=GO:0015671 feature_id[103].value <= threshold=1.3113192915916443
node_671: feature_name=GO:0008340 feature_id[539].value <= threshold=4.860779762268066
node_672: feature_name=GO:0046006 feature_id[364].value <= threshold=2.4317692518234253
node_673: feature_name=GO:0042991 feature_id[684].value > threshold=5.553565502166748
node_713: feature_name=GO:0046427 feature_id[766].value > threshold=0.28762511909008026
Class: negative genes

Rules_275 passed counts:3
node_0: feature_name=GO:0042113 feature_id[0].value <= threshold=13.408552169799805
node_1: feature_name=GO:0007568 feature_id[534].value <= threshold=5.0313897132873535
node_2: feature_name=GO:0002705 feature_id[541].value <= threshold=3.200145721435547
node_3: feature_name=GO:1901525 feature_id[576].value > threshold=0.4466460347175598
node_617: feature_name=GO:0005622 feature_id[233].value <= threshold=90.85730743408203
node_618: feature_name=GO:1903147 feature_id[200].value <= threshold=7.326428413391113
node_619: feature_name=GO:0003964 feature_id[317].value <= threshold=5.575642108917236
node_620: feature_name=GO:0046498 feature_id[203].value <= threshold=6.620223522186279
node_621: feature_name=GO:0002562 feature_id[25].value <= threshold=10.287704467773438
node_622: feature_name=GO:0006919 feature_id[246].value <= threshold=5.801334381103516
node_623: feature_name=GO:0001889 feature_id[387].value <= threshold=2.679414987564087
node_624: feature_name=GO:0006555 feature_id[514].value <= threshold=7.819535493850708
node_625: feature_name=GO:0080134 feature_id[650].value <= threshold=38.91967582702637
node_626: feature_name=GO:0042127 feature_id[480].value <= threshold=37.72536659240723
node_627: feature_name=GO:2001251 feature_id[220].value <= threshold=32.564422607421875
node_628: feature_name=GO:0002829 feature_id[493].value <= threshold=2.444612741470337
node_629: feature_name=GO:0008588 feature_id[26].value <= threshold=2.3601274490356445
node_630: feature_name=GO:0033151 feature_id[7].value <= threshold=5.134376287460327
node_631: feature_name=GO:0048145 feature_id[348].value <= threshold=8.836549282073975
node_632: feature_name=GO:0030887 feature_id[274].value <= threshold=1.7000296115875244
node_633: feature_name=GO:0097190 feature_id[117].value <= threshold=33.02078819274902
node_634: feature_name=GO:0046685 feature_id[782].value <= threshold=8.040813446044922
node_635: feature_name=GO:0006346 feature_id[236].value <= threshold=6.384642839431763

node_636: feature_name=GO:0048537 feature_id[66].value <= threshold=3.1739262342453003
node_637: feature_name=GO:0070245 feature_id[326].value <= threshold=3.0491198301315308
node_638: feature_name=GO:0038065 feature_id[670].value <= threshold=2.0590850114822388
node_639: feature_name=GO:0005488 feature_id[187].value <= threshold=84.80076217651367
node_640: feature_name=GO:0060249 feature_id[390].value > threshold=0.07226398587226868
node_652: feature_name=GO:0006974 feature_id[516].value > threshold=5.17782768838515e-06
node_654: feature_name=hsa05210 feature_id[358].value <= threshold=18.730005264282227
node_655: feature_name=GO:0071850 feature_id[40].value <= threshold=5.475317001342773
node_656: feature_name=GO:1904029 feature_id[208].value <= threshold=26.575013160705566
node_657: feature_name=GO:0090594 feature_id[738].value <= threshold=3.000791072845459
node_658: feature_name=GO:0010948 feature_id[569].value > threshold=0.04386143572628498
node_666: feature_name=GO:0032764 feature_id[3].value <= threshold=2.5201677083969116
node_667: feature_name=GO:0023026 feature_id[69].value <= threshold=3.2760006189346313
node_668: feature_name=GO:0032464 feature_id[83].value <= threshold=2.5927021503448486
node_669: feature_name=GO:0071901 feature_id[407].value <= threshold=18.238012313842773
node_670: feature_name=GO:0015671 feature_id[103].value <= threshold=1.3113192915916443
node_671: feature_name=GO:0008340 feature_id[539].value <= threshold=4.860779762268066
node_672: feature_name=GO:0046006 feature_id[364].value <= threshold=2.4317692518234253
node_673: feature_name=GO:0042991 feature_id[684].value <= threshold=5.553565502166748
node_674: feature_name=GO:0002327 feature_id[408].value <= threshold=2.404940366744995
node_675: feature_name=GO:0001836 feature_id[450].value <= threshold=8.647934436798096
node_676: feature_name=GO:0048539 feature_id[319].value <= threshold=1.6308802962303162
node_677: feature_name=GO:0022408 feature_id[279].value <= threshold=4.064727783203125
node_678: feature_name=GO:0030099 feature_id[598].value > threshold=0.0001809061213862151
node_684: feature_name=GO:0001816 feature_id[685].value <= threshold=1.3516043054551119e-05
node_685: feature_name=GO:0019104 feature_id[124].value <= threshold=0.5158462524414062
Class: negative genes

Rules_276 passed counts:3
node_0: feature_name=GO:0042113 feature_id[0].value <= threshold=13.408552169799805
node_1: feature_name=GO:0007568 feature_id[534].value <= threshold=5.0313897132873535
node_2: feature_name=GO:0002705 feature_id[541].value <= threshold=3.200145721435547
node_3: feature_name=GO:1901525 feature_id[576].value > threshold=0.4466460347175598
node_617: feature_name=GO:0005622 feature_id[233].value <= threshold=90.85730743408203
node_618: feature_name=GO:1903147 feature_id[200].value <= threshold=7.326428413391113

node_619: feature_name=GO:0003964 feature_id[317].value <= threshold=5.575642108917236
node_620: feature_name=GO:0046498 feature_id[203].value <= threshold=6.620223522186279
node_621: feature_name=GO:0002562 feature_id[25].value <= threshold=10.287704467773438
node_622: feature_name=GO:0006919 feature_id[246].value <= threshold=5.801334381103516
node_623: feature_name=GO:0001889 feature_id[387].value <= threshold=2.679414987564087
node_624: feature_name=GO:0006555 feature_id[514].value <= threshold=7.819535493850708
node_625: feature_name=GO:0080134 feature_id[650].value <= threshold=38.91967582702637
node_626: feature_name=GO:0042127 feature_id[480].value <= threshold=37.72536659240723
node_627: feature_name=GO:2001251 feature_id[220].value <= threshold=32.564422607421875
node_628: feature_name=GO:0002829 feature_id[493].value <= threshold=2.444612741470337
node_629: feature_name=GO:0008588 feature_id[26].value <= threshold=2.3601274490356445
node_630: feature_name=GO:0033151 feature_id[7].value <= threshold=5.134376287460327
node_631: feature_name=GO:0048145 feature_id[348].value <= threshold=8.836549282073975
node_632: feature_name=GO:0030887 feature_id[274].value <= threshold=1.7000296115875244
node_633: feature_name=GO:0097190 feature_id[117].value <= threshold=33.02078819274902
node_634: feature_name=GO:0046685 feature_id[782].value <= threshold=8.040813446044922
node_635: feature_name=GO:0006346 feature_id[236].value <= threshold=6.384642839431763
node_636: feature_name=GO:0048537 feature_id[66].value <= threshold=3.1739262342453003
node_637: feature_name=GO:0070245 feature_id[326].value <= threshold=3.0491198301315308
node_638: feature_name=GO:0038065 feature_id[670].value <= threshold=2.0590850114822388
node_639: feature_name=GO:0005488 feature_id[187].value <= threshold=84.80076217651367
node_640: feature_name=GO:0060249 feature_id[390].value <= threshold=0.07226398587226868
node_641: feature_name=GO:0002683 feature_id[456].value <= threshold=0.7958214282989502
node_642: feature_name=GO:0006346 feature_id[236].value > threshold=0.4950565919280052
node_644: feature_name=GO:2001020 feature_id[275].value > threshold=0.35050614178180695
Class: positive genes

Rules_277 passed counts:3
node_0: feature_name=GO:0042113 feature_id[0].value <= threshold=13.408552169799805
node_1: feature_name=GO:0007568 feature_id[534].value <= threshold=5.0313897132873535
node_2: feature_name=GO:0002705 feature_id[541].value <= threshold=3.200145721435547
node_3: feature_name=GO:1901525 feature_id[576].value <= threshold=0.4466460347175598
node_4: feature_name=GO:0048539 feature_id[319].value > threshold=3.0399646759033203
Class: positive genes

Rules_278 passed counts:3
node_0: feature_name=GO:0042113 feature_id[0].value <= threshold=13.408552169799805
node_1: feature_name=GO:0007568 feature_id[534].value <= threshold=5.0313897132873535
node_2: feature_name=GO:0002705 feature_id[541].value <= threshold=3.200145721435547
node_3: feature_name=GO:1901525 feature_id[576].value <= threshold=0.4466460347175598
node_4: feature_name=GO:0048539 feature_id[319].value <= threshold=3.0399646759033203
node_5: feature_name=GO:0001910 feature_id[385].value <= threshold=3.7437864542007446
node_6: feature_name=GO:0043200 feature_id[706].value > threshold=9.307284355163574
node_612: feature_name=GO:0002376 feature_id[277].value > threshold=1.4492701292037964
Class: positive genes

Rules_279 passed counts:3
node_0: feature_name=GO:0042113 feature_id[0].value <= threshold=13.408552169799805
node_1: feature_name=GO:0007568 feature_id[534].value <= threshold=5.0313897132873535
node_2: feature_name=GO:0002705 feature_id[541].value <= threshold=3.200145721435547
node_3: feature_name=GO:1901525 feature_id[576].value <= threshold=0.4466460347175598
node_4: feature_name=GO:0048539 feature_id[319].value <= threshold=3.0399646759033203
node_5: feature_name=GO:0001910 feature_id[385].value <= threshold=3.7437864542007446
node_6: feature_name=GO:0043200 feature_id[706].value <= threshold=9.307284355163574
node_7: feature_name=GO:0001773 feature_id[308].value > threshold=4.016931533813477
node_609: feature_name=GO:0010948 feature_id[569].value > threshold=0.6070263236761093
Class: negative genes

Rules_280 passed counts:3
node_0: feature_name=GO:0042113 feature_id[0].value <= threshold=13.408552169799805
node_1: feature_name=GO:0007568 feature_id[534].value <= threshold=5.0313897132873535
node_2: feature_name=GO:0002705 feature_id[541].value <= threshold=3.200145721435547
node_3: feature_name=GO:1901525 feature_id[576].value <= threshold=0.4466460347175598
node_4: feature_name=GO:0048539 feature_id[319].value <= threshold=3.0399646759033203
node_5: feature_name=GO:0001910 feature_id[385].value <= threshold=3.7437864542007446
node_6: feature_name=GO:0043200 feature_id[706].value <= threshold=9.307284355163574
node_7: feature_name=GO:0001773 feature_id[308].value > threshold=4.016931533813477
node_609: feature_name=GO:0010948 feature_id[569].value <= threshold=0.6070263236761093
Class: positive genes

Rules_281 passed counts:3
node_0: feature_name=GO:0042113 feature_id[0].value <= threshold=13.408552169799805
node_1: feature_name=GO:0007568 feature_id[534].value <= threshold=5.0313897132873535
node_2: feature_name=GO:0002705 feature_id[541].value <= threshold=3.200145721435547
node_3: feature_name=GO:1901525 feature_id[576].value <= threshold=0.4466460347175598
node_4: feature_name=GO:0048539 feature_id[319].value <= threshold=3.0399646759033203
node_5: feature_name=GO:0001910 feature_id[385].value <= threshold=3.7437864542007446
node_6: feature_name=GO:0043200 feature_id[706].value <= threshold=9.307284355163574
node_7: feature_name=GO:0001773 feature_id[308].value <= threshold=4.016931533813477
node_8: feature_name=GO:0090116 feature_id[97].value <= threshold=7.99645471572876
node_9: feature_name=GO:0019814 feature_id[189].value <= threshold=4.525782108306885
node_10: feature_name=GO:1902583 feature_id[215].value <= threshold=17.60310649871826
node_11: feature_name=GO:0045429 feature_id[737].value > threshold=1.6133361458778381
node_477: feature_name=GO:0036037 feature_id[445].value <= threshold=1.7181594371795654
node_478: feature_name=GO:0006244 feature_id[503].value <= threshold=0.951388418674469
node_479: feature_name=GO:0032461 feature_id[608].value <= threshold=2.6103241443634033
node_480: feature_name=GO:0044710 feature_id[719].value > threshold=2.228096718681627e-06
node_494: feature_name=GO:0009314 feature_id[296].value <= threshold=34.93696117401123
node_495: feature_name=GO:0050778 feature_id[802].value <= threshold=39.29364013671875
node_496: feature_name=GO:1903038 feature_id[492].value <= threshold=5.849650859832764
node_497: feature_name=GO:0002699 feature_id[606].value <= threshold=5.5405943393707275
node_498: feature_name=hsa00983 feature_id[181].value <= threshold=25.851045608520508
node_499: feature_name=GO:0002309 feature_id[416].value <= threshold=1.6235689520835876
node_500: feature_name=GO:1901698 feature_id[792].value <= threshold=47.70275688171387
node_501: feature_name=GO:0010216 feature_id[282].value <= threshold=1.6849713921546936
node_502: feature_name=GO:0006266 feature_id[297].value > threshold=2.7199249267578125
node_572: feature_name=GO:0060255 feature_id[216].value <= threshold=2.504572629928589
Class: negative genes

Rules_282 passed counts:3
node_0: feature_name=GO:0042113 feature_id[0].value <= threshold=13.408552169799805
node_1: feature_name=GO:0007568 feature_id[534].value <= threshold=5.0313897132873535
node_2: feature_name=GO:0002705 feature_id[541].value <= threshold=3.200145721435547
node_3: feature_name=GO:1901525 feature_id[576].value <= threshold=0.4466460347175598
node_4: feature_name=GO:0048539 feature_id[319].value <= threshold=3.0399646759033203

node_5: feature_name=GO:0001910 feature_id[385].value <= threshold=3.7437864542007446
node_6: feature_name=GO:0043200 feature_id[706].value <= threshold=9.307284355163574
node_7: feature_name=GO:0001773 feature_id[308].value <= threshold=4.016931533813477
node_8: feature_name=GO:0090116 feature_id[97].value <= threshold=7.99645471572876
node_9: feature_name=GO:0019814 feature_id[189].value <= threshold=4.525782108306885
node_10: feature_name=GO:1902583 feature_id[215].value <= threshold=17.60310649871826
node_11: feature_name=GO:0045429 feature_id[737].value > threshold=1.6133361458778381
node_477: feature_name=GO:0036037 feature_id[445].value <= threshold=1.7181594371795654
node_478: feature_name=GO:0006244 feature_id[503].value <= threshold=0.951388418674469
node_479: feature_name=GO:0032461 feature_id[608].value <= threshold=2.6103241443634033
node_480: feature_name=GO:0044710 feature_id[719].value > threshold=2.228096718681627e-06
node_494: feature_name=GO:0009314 feature_id[296].value <= threshold=34.93696117401123
node_495: feature_name=GO:0050778 feature_id[802].value <= threshold=39.29364013671875
node_496: feature_name=GO:1903038 feature_id[492].value <= threshold=5.849650859832764
node_497: feature_name=GO:0002699 feature_id[606].value <= threshold=5.5405943393707275
node_498: feature_name=hsa00983 feature_id[181].value <= threshold=25.851045608520508
node_499: feature_name=GO:0002309 feature_id[416].value <= threshold=1.6235689520835876
node_500: feature_name=GO:1901698 feature_id[792].value <= threshold=47.70275688171387
node_501: feature_name=GO:0010216 feature_id[282].value <= threshold=1.6849713921546936
node_502: feature_name=GO:0006266 feature_id[297].value <= threshold=2.7199249267578125
node_503: feature_name=GO:0005488 feature_id[187].value > threshold=3.0373169010999845e-06
node_507: feature_name=GO:0045429 feature_id[737].value > threshold=1.614579975605011
node_511: feature_name=GO:0034103 feature_id[318].value <= threshold=5.832815647125244
node_512: feature_name=GO:0010663 feature_id[561].value <= threshold=4.157005429267883
node_513: feature_name=GO:0030887 feature_id[274].value <= threshold=1.8964150547981262
node_514: feature_name=GO:0048294 feature_id[113].value <= threshold=1.866134524345398
node_515: feature_name=GO:0072539 feature_id[68].value <= threshold=0.8600535988807678
node_516: feature_name=GO:0045840 feature_id[697].value <= threshold=4.963376045227051
node_517: feature_name=GO:0019222 feature_id[129].value > threshold=4.0122095015249215e-06
node_523: feature_name=GO:0008150 feature_id[528].value <= threshold=40.503862380981445
node_524: feature_name=GO:0006359 feature_id[87].value > threshold=2.954534411430359
node_538: feature_name=GO:0009897 feature_id[38].value > threshold=0.00021361259859986603
Class: negative genes

Rules_283 passed counts:3

node_0: feature_name=GO:0042113	feature_id[0].value <= threshold=13.408552169799805
node_1: feature_name=GO:0007568	feature_id[534].value <= threshold=5.0313897132873535
node_2: feature_name=GO:0002705	feature_id[541].value <= threshold=3.200145721435547
node_3: feature_name=GO:1901525	feature_id[576].value <= threshold=0.4466460347175598
node_4: feature_name=GO:0048539	feature_id[319].value <= threshold=3.0399646759033203
node_5: feature_name=GO:0001910	feature_id[385].value <= threshold=3.7437864542007446
node_6: feature_name=GO:0043200	feature_id[706].value <= threshold=9.307284355163574
node_7: feature_name=GO:0001773	feature_id[308].value <= threshold=4.016931533813477
node_8: feature_name=GO:0090116	feature_id[97].value <= threshold=7.99645471572876
node_9: feature_name=GO:0019814	feature_id[189].value <= threshold=4.525782108306885
node_10: feature_name=GO:1902583	feature_id[215].value <= threshold=17.60310649871826
node_11: feature_name=GO:0045429	feature_id[737].value > threshold=1.6133361458778381
node_477: feature_name=GO:0036037	feature_id[445].value <= threshold=1.7181594371795654
node_478: feature_name=GO:0006244	feature_id[503].value <= threshold=0.951388418674469
node_479: feature_name=GO:0032461	feature_id[608].value <= threshold=2.6103241443634033
node_480: feature_name=GO:0044710	feature_id[719].value > threshold=2.228096718681627e-06
node_494: feature_name=GO:0009314	feature_id[296].value <= threshold=34.93696117401123
node_495: feature_name=GO:0050778	feature_id[802].value <= threshold=39.29364013671875
node_496: feature_name=GO:1903038	feature_id[492].value <= threshold=5.849650859832764
node_497: feature_name=GO:0002699	feature_id[606].value <= threshold=5.5405943393707275
node_498: feature_name=hsa00983	feature_id[181].value <= threshold=25.851045608520508
node_499: feature_name=GO:0002309	feature_id[416].value <= threshold=1.6235689520835876
node_500: feature_name=GO:1901698	feature_id[792].value <= threshold=47.70275688171387
node_501: feature_name=GO:0010216	feature_id[282].value <= threshold=1.6849713921546936
node_502: feature_name=GO:0006266	feature_id[297].value <= threshold=2.7199249267578125
node_503: feature_name=GO:0005488	feature_id[187].value > threshold=3.0373169010999845e-06
node_507: feature_name=GO:0045429	feature_id[737].value > threshold=1.614579975605011
node_511: feature_name=GO:0034103	feature_id[318].value <= threshold=5.832815647125244
node_512: feature_name=GO:0010663	feature_id[561].value <= threshold=4.157005429267883
node_513: feature_name=GO:0030887	feature_id[274].value <= threshold=1.8964150547981262
node_514: feature_name=GO:0048294	feature_id[113].value <= threshold=1.866134524345398
node_515: feature_name=GO:0072539	feature_id[68].value <= threshold=0.8600535988807678
node_516: feature_name=GO:0045840	feature_id[697].value <= threshold=4.963376045227051
node_517: feature_name=GO:0019222	feature_id[129].value <= threshold=4.0122095015249215e-06
node_518: feature_name=GO:0007568	feature_id[534].value > threshold=3.2433676719665527

node_520: feature_name=GO:0030888 feature_id[120].value > threshold=0.121795654296875
Class: positive genes

Rules_284 passed counts:3
node_0: feature_name=GO:0042113 feature_id[0].value <= threshold=13.408552169799805
node_1: feature_name=GO:0007568 feature_id[534].value <= threshold=5.0313897132873535
node_2: feature_name=GO:0002705 feature_id[541].value <= threshold=3.200145721435547
node_3: feature_name=GO:1901525 feature_id[576].value <= threshold=0.4466460347175598
node_4: feature_name=GO:0048539 feature_id[319].value <= threshold=3.0399646759033203
node_5: feature_name=GO:0001910 feature_id[385].value <= threshold=3.7437864542007446
node_6: feature_name=GO:0043200 feature_id[706].value <= threshold=9.307284355163574
node_7: feature_name=GO:0001773 feature_id[308].value <= threshold=4.016931533813477
node_8: feature_name=GO:0090116 feature_id[97].value <= threshold=7.99645471572876
node_9: feature_name=GO:0019814 feature_id[189].value <= threshold=4.525782108306885
node_10: feature_name=GO:1902583 feature_id[215].value <= threshold=17.60310649871826
node_11: feature_name=GO:0045429 feature_id[737].value > threshold=1.6133361458778381
node_477: feature_name=GO:0036037 feature_id[445].value <= threshold=1.7181594371795654
node_478: feature_name=GO:0006244 feature_id[503].value <= threshold=0.951388418674469
node_479: feature_name=GO:0032461 feature_id[608].value <= threshold=2.6103241443634033
node_480: feature_name=GO:0044710 feature_id[719].value > threshold=2.228096718681627e-06
node_494: feature_name=GO:0009314 feature_id[296].value <= threshold=34.93696117401123
node_495: feature_name=GO:0050778 feature_id[802].value <= threshold=39.29364013671875
node_496: feature_name=GO:1903038 feature_id[492].value <= threshold=5.849650859832764
node_497: feature_name=GO:0002699 feature_id[606].value <= threshold=5.5405943393707275
node_498: feature_name=hsa00983 feature_id[181].value <= threshold=25.851045608520508
node_499: feature_name=GO:0002309 feature_id[416].value <= threshold=1.6235689520835876
node_500: feature_name=GO:1901698 feature_id[792].value <= threshold=47.70275688171387
node_501: feature_name=GO:0010216 feature_id[282].value <= threshold=1.6849713921546936
node_502: feature_name=GO:0006266 feature_id[297].value <= threshold=2.7199249267578125
node_503: feature_name=GO:0005488 feature_id[187].value <= threshold=3.0373169010999845e-06
node_504: feature_name=GO:0002377 feature_id[322].value <= threshold=0.056253451853990555
Class: negative genes

Rules_285 passed counts:3
node_0: feature_name=GO:0042113 feature_id[0].value <= threshold=13.408552169799805

node_1: feature_name=GO:0007568 feature_id[534].value <= threshold=5.0313897132873535
node_2: feature_name=GO:0002705 feature_id[541].value <= threshold=3.200145721435547
node_3: feature_name=GO:1901525 feature_id[576].value <= threshold=0.4466460347175598
node_4: feature_name=GO:0048539 feature_id[319].value <= threshold=3.0399646759033203
node_5: feature_name=GO:0001910 feature_id[385].value <= threshold=3.7437864542007446
node_6: feature_name=GO:0043200 feature_id[706].value <= threshold=9.307284355163574
node_7: feature_name=GO:0001773 feature_id[308].value <= threshold=4.016931533813477
node_8: feature_name=GO:0090116 feature_id[97].value <= threshold=7.99645471572876
node_9: feature_name=GO:0019814 feature_id[189].value <= threshold=4.525782108306885
node_10: feature_name=GO:1902583 feature_id[215].value <= threshold=17.60310649871826
node_11: feature_name=GO:0045429 feature_id[737].value <= threshold=1.6133361458778381
node_12: feature_name=GO:0003720 feature_id[228].value <= threshold=5.519326210021973
node_13: feature_name=GO:0046006 feature_id[364].value <= threshold=4.9985432624816895
node_14: feature_name=GO:0070424 feature_id[254].value <= threshold=6.233297109603882
node_15: feature_name=GO:0009892 feature_id[320].value <= threshold=59.99180793762207
node_16: feature_name=GO:0007064 feature_id[527].value <= threshold=11.57377815246582
node_17: feature_name=GO:0005575 feature_id[17].value <= threshold=15.417460918426514
node_18: feature_name=GO:0043368 feature_id[31].value <= threshold=4.059496641159058
node_19: feature_name=GO:0005164 feature_id[163].value <= threshold=5.793607711791992
node_20: feature_name=GO:0042130 feature_id[16].value <= threshold=5.073179721832275
node_21: feature_name=GO:0010216 feature_id[282].value <= threshold=4.834584474563599
node_22: feature_name=GO:0009628 feature_id[553].value <= threshold=29.314892768859863
node_23: feature_name=GO:0045628 feature_id[749].value <= threshold=3.1001367568969727
node_24: feature_name=GO:0042288 feature_id[100].value <= threshold=5.174932956695557
node_25: feature_name=GO:0002329 feature_id[406].value > threshold=2.348930835723877
node_449: feature_name=GO:0006725 feature_id[523].value <= threshold=2.542858638321377e-06
Class: positive genes

Rules_286 passed counts:3
node_0: feature_name=GO:0042113 feature_id[0].value <= threshold=13.408552169799805
node_1: feature_name=GO:0007568 feature_id[534].value <= threshold=5.0313897132873535
node_2: feature_name=GO:0002705 feature_id[541].value <= threshold=3.200145721435547
node_3: feature_name=GO:1901525 feature_id[576].value <= threshold=0.4466460347175598
node_4: feature_name=GO:0048539 feature_id[319].value <= threshold=3.0399646759033203
node_5: feature_name=GO:0001910 feature_id[385].value <= threshold=3.7437864542007446

node_6: feature_name=GO:0043200 feature_id[706].value <= threshold=9.307284355163574
node_7: feature_name=GO:0001773 feature_id[308].value <= threshold=4.016931533813477
node_8: feature_name=GO:0090116 feature_id[97].value <= threshold=7.99645471572876
node_9: feature_name=GO:0019814 feature_id[189].value <= threshold=4.525782108306885
node_10: feature_name=GO:1902583 feature_id[215].value <= threshold=17.60310649871826
node_11: feature_name=GO:0045429 feature_id[737].value <= threshold=1.6133361458778381
node_12: feature_name=GO:0003720 feature_id[228].value <= threshold=5.519326210021973
node_13: feature_name=GO:0046006 feature_id[364].value <= threshold=4.9985432624816895
node_14: feature_name=GO:0070424 feature_id[254].value <= threshold=6.233297109603882
node_15: feature_name=GO:0009892 feature_id[320].value <= threshold=59.99180793762207
node_16: feature_name=GO:0007064 feature_id[527].value <= threshold=11.57377815246582
node_17: feature_name=GO:0005575 feature_id[17].value <= threshold=15.417460918426514
node_18: feature_name=GO:0043368 feature_id[31].value <= threshold=4.059496641159058
node_19: feature_name=GO:0005164 feature_id[163].value <= threshold=5.793607711791992
node_20: feature_name=GO:0042130 feature_id[16].value <= threshold=5.073179721832275
node_21: feature_name=GO:0010216 feature_id[282].value <= threshold=4.834584474563599
node_22: feature_name=GO:0009628 feature_id[553].value <= threshold=29.314892768859863
node_23: feature_name=GO:0045628 feature_id[749].value <= threshold=3.1001367568969727
node_24: feature_name=GO:0042288 feature_id[100].value <= threshold=5.174932956695557
node_25: feature_name=GO:0002329 feature_id[406].value <= threshold=2.348930835723877
node_26: feature_name=GO:0051246 feature_id[642].value > threshold=0.6498909294605255
node_194: feature_name=GO:0009164 feature_id[546].value <= threshold=17.11970329284668
node_195: feature_name=GO:0001909 feature_id[386].value <= threshold=7.292566299438477
node_196: feature_name=GO:0003908 feature_id[11].value > threshold=1.887226164340973
node_444: feature_name=GO:0034103 feature_id[318].value > threshold=0.34810671210289
Class: positive genes

Rules_287 passed counts:3
node_0: feature_name=GO:0042113 feature_id[0].value <= threshold=13.408552169799805
node_1: feature_name=GO:0007568 feature_id[534].value <= threshold=5.0313897132873535
node_2: feature_name=GO:0002705 feature_id[541].value <= threshold=3.200145721435547
node_3: feature_name=GO:1901525 feature_id[576].value <= threshold=0.4466460347175598
node_4: feature_name=GO:0048539 feature_id[319].value <= threshold=3.0399646759033203
node_5: feature_name=GO:0001910 feature_id[385].value <= threshold=3.7437864542007446
node_6: feature_name=GO:0043200 feature_id[706].value <= threshold=9.307284355163574

node_7: feature_name=GO:0001773	feature_id[308].value <= threshold=4.016931533813477
node_8: feature_name=GO:0090116	feature_id[97].value <= threshold=7.99645471572876
node_9: feature_name=GO:0019814	feature_id[189].value <= threshold=4.525782108306885
node_10: feature_name=GO:1902583	feature_id[215].value <= threshold=17.60310649871826
node_11: feature_name=GO:0045429	feature_id[737].value <= threshold=1.6133361458778381
node_12: feature_name=GO:0003720	feature_id[228].value <= threshold=5.519326210021973
node_13: feature_name=GO:0046006	feature_id[364].value <= threshold=4.9985432624816895
node_14: feature_name=GO:0070424	feature_id[254].value <= threshold=6.233297109603882
node_15: feature_name=GO:0009892	feature_id[320].value <= threshold=59.99180793762207
node_16: feature_name=GO:0007064	feature_id[527].value <= threshold=11.57377815246582
node_17: feature_name=GO:0005575	feature_id[17].value <= threshold=15.417460918426514
node_18: feature_name=GO:0043368	feature_id[31].value <= threshold=4.059496641159058
node_19: feature_name=GO:0005164	feature_id[163].value <= threshold=5.793607711791992
node_20: feature_name=GO:0042130	feature_id[16].value <= threshold=5.073179721832275
node_21: feature_name=GO:0010216	feature_id[282].value <= threshold=4.834584474563599
node_22: feature_name=GO:0009628	feature_id[553].value <= threshold=29.314892768859863
node_23: feature_name=GO:0045628	feature_id[749].value <= threshold=3.1001367568969727
node_24: feature_name=GO:0042288	feature_id[100].value <= threshold=5.174932956695557
node_25: feature_name=GO:0002329	feature_id[406].value <= threshold=2.348930835723877
node_26: feature_name=GO:0051246	feature_id[642].value > threshold=0.6498909294605255
node_194: feature_name=GO:0009164	feature_id[546].value <= threshold=17.11970329284668
node_195: feature_name=GO:0001909	feature_id[386].value <= threshold=7.292566299438477
node_196: feature_name=GO:0003908	feature_id[11].value <= threshold=1.887226164340973
node_197: feature_name=GO:0009164	feature_id[546].value > threshold=0.26642198860645294
node_359: feature_name=GO:0009164	feature_id[546].value > threshold=0.2696942090988159
node_361: feature_name=hsa05202	feature_id[50].value <= threshold=14.989679336547852
node_362: feature_name=GO:0001775	feature_id[505].value <= threshold=15.726949691772461
node_363: feature_name=GO:0002634	feature_id[449].value <= threshold=2.825531005859375
node_364: feature_name=GO:0010038	feature_id[457].value <= threshold=9.901018619537354
node_365: feature_name=GO:0044238	feature_id[295].value <= threshold=139.80535888671875
node_366: feature_name=GO:0051246	feature_id[642].value > threshold=0.6527281999588013
node_368: feature_name=GO:0044092	feature_id[432].value > threshold=11.084641933441162
node_436: feature_name=GO:0071901	feature_id[407].value > threshold=1.6024202108383179

Class: positive genes

Rules_288	passed counts:3
node_0: feature_name=GO:0042113	feature_id[0].value <= threshold=13.408552169799805
node_1: feature_name=GO:0007568	feature_id[534].value <= threshold=5.0313897132873535
node_2: feature_name=GO:0002705	feature_id[541].value <= threshold=3.200145721435547
node_3: feature_name=GO:1901525	feature_id[576].value <= threshold=0.4466460347175598
node_4: feature_name=GO:0048539	feature_id[319].value <= threshold=3.0399646759033203
node_5: feature_name=GO:0001910	feature_id[385].value <= threshold=3.7437864542007446
node_6: feature_name=GO:0043200	feature_id[706].value <= threshold=9.307284355163574
node_7: feature_name=GO:0001773	feature_id[308].value <= threshold=4.016931533813477
node_8: feature_name=GO:0090116	feature_id[97].value <= threshold=7.99645471572876
node_9: feature_name=GO:0019814	feature_id[189].value <= threshold=4.525782108306885
node_10: feature_name=GO:1902583	feature_id[215].value <= threshold=17.60310649871826
node_11: feature_name=GO:0045429	feature_id[737].value <= threshold=1.6133361458778381
node_12: feature_name=GO:0003720	feature_id[228].value <= threshold=5.519326210021973
node_13: feature_name=GO:0046006	feature_id[364].value <= threshold=4.9985432624816895
node_14: feature_name=GO:0070424	feature_id[254].value <= threshold=6.233297109603882
node_15: feature_name=GO:0009892	feature_id[320].value <= threshold=59.99180793762207
node_16: feature_name=GO:0007064	feature_id[527].value <= threshold=11.57377815246582
node_17: feature_name=GO:0005575	feature_id[17].value <= threshold=15.417460918426514
node_18: feature_name=GO:0043368	feature_id[31].value <= threshold=4.059496641159058
node_19: feature_name=GO:0005164	feature_id[163].value <= threshold=5.793607711791992
node_20: feature_name=GO:0042130	feature_id[16].value <= threshold=5.073179721832275
node_21: feature_name=GO:0010216	feature_id[282].value <= threshold=4.834584474563599
node_22: feature_name=GO:0009628	feature_id[553].value <= threshold=29.314892768859863
node_23: feature_name=GO:0045628	feature_id[749].value <= threshold=3.1001367568969727
node_24: feature_name=GO:0042288	feature_id[100].value <= threshold=5.174932956695557
node_25: feature_name=GO:0002329	feature_id[406].value <= threshold=2.348930835723877
node_26: feature_name=GO:0051246	feature_id[642].value > threshold=0.6498909294605255
node_194: feature_name=GO:0009164	feature_id[546].value <= threshold=17.11970329284668
node_195: feature_name=GO:0001909	feature_id[386].value <= threshold=7.292566299438477
node_196: feature_name=GO:0003908	feature_id[11].value <= threshold=1.887226164340973
node_197: feature_name=GO:0009164	feature_id[546].value > threshold=0.26642198860645294
node_359: feature_name=GO:0009164	feature_id[546].value > threshold=0.2696942090988159
node_361: feature_name=hsa05202	feature_id[50].value <= threshold=14.989679336547852
node_362: feature_name=GO:0001775	feature_id[505].value <= threshold=15.726949691772461

node_363: feature_name=GO:0002634 feature_id[449].value <= threshold=2.825531005859375
node_364: feature_name=GO:0010038 feature_id[457].value <= threshold=9.901018619537354
node_365: feature_name=GO:0044238 feature_id[295].value <= threshold=139.80535888671875
node_366: feature_name=GO:0051246 feature_id[642].value > threshold=0.6527281999588013
node_368: feature_name=GO:0044092 feature_id[432].value <= threshold=11.084641933441162
node_369: feature_name=GO:0042162 feature_id[355].value <= threshold=9.683155536651611
node_370: feature_name=GO:0045945 feature_id[267].value <= threshold=3.6181851625442505
node_371: feature_name=GO:0036296 feature_id[252].value <= threshold=3.214281916618347
node_372: feature_name=GO:0097506 feature_id[51].value <= threshold=2.9644681215286255
node_373: feature_name=GO:0019740 feature_id[581].value <= threshold=2.2853575944900513
node_374: feature_name=GO:0019660 feature_id[70].value <= threshold=1.2831979990005493
node_375: feature_name=GO:0071901 feature_id[407].value <= threshold=14.498775482177734
node_376: feature_name=GO:0043170 feature_id[467].value > threshold=4.473330818655086e-06
node_380: feature_name=GO:0046718 feature_id[781].value <= threshold=3.494875192642212
node_381: feature_name=GO:0030983 feature_id[291].value <= threshold=10.994179248809814
node_382: feature_name=GO:0010663 feature_id[561].value <= threshold=2.7056833505630493
node_383: feature_name=GO:0031667 feature_id[648].value > threshold=0.0020462179090827703
node_387: feature_name=GO:1990572 feature_id[243].value > threshold=1.9359037280082703
node_409: feature_name=GO:0050866 feature_id[338].value <= threshold=0.9498499482870102
Class: negative genes

Rules_289 passed counts:3
node_0: feature_name=GO:0042113 feature_id[0].value <= threshold=13.408552169799805
node_1: feature_name=GO:0007568 feature_id[534].value <= threshold=5.0313897132873535
node_2: feature_name=GO:0002705 feature_id[541].value <= threshold=3.200145721435547
node_3: feature_name=GO:1901525 feature_id[576].value <= threshold=0.4466460347175598
node_4: feature_name=GO:0048539 feature_id[319].value <= threshold=3.0399646759033203
node_5: feature_name=GO:0001910 feature_id[385].value <= threshold=3.7437864542007446
node_6: feature_name=GO:0043200 feature_id[706].value <= threshold=9.307284355163574
node_7: feature_name=GO:0001773 feature_id[308].value <= threshold=4.016931533813477
node_8: feature_name=GO:0090116 feature_id[97].value <= threshold=7.99645471572876
node_9: feature_name=GO:0019814 feature_id[189].value <= threshold=4.525782108306885
node_10: feature_name=GO:1902583 feature_id[215].value <= threshold=17.60310649871826
node_11: feature_name=GO:0045429 feature_id[737].value <= threshold=1.6133361458778381
node_12: feature_name=GO:0003720 feature_id[228].value <= threshold=5.519326210021973

node_13: feature_name=GO:0046006 feature_id[364].value <= threshold=4.9985432624816895
node_14: feature_name=GO:0070424 feature_id[254].value <= threshold=6.233297109603882
node_15: feature_name=GO:0009892 feature_id[320].value <= threshold=59.99180793762207
node_16: feature_name=GO:0007064 feature_id[527].value <= threshold=11.57377815246582
node_17: feature_name=GO:0005575 feature_id[17].value <= threshold=15.417460918426514
node_18: feature_name=GO:0043368 feature_id[31].value <= threshold=4.059496641159058
node_19: feature_name=GO:0005164 feature_id[163].value <= threshold=5.793607711791992
node_20: feature_name=GO:0042130 feature_id[16].value <= threshold=5.073179721832275
node_21: feature_name=GO:0010216 feature_id[282].value <= threshold=4.834584474563599
node_22: feature_name=GO:0009628 feature_id[553].value <= threshold=29.314892768859863
node_23: feature_name=GO:0045628 feature_id[749].value <= threshold=3.1001367568969727
node_24: feature_name=GO:0042288 feature_id[100].value <= threshold=5.174932956695557
node_25: feature_name=GO:0002329 feature_id[406].value <= threshold=2.348930835723877
node_26: feature_name=GO:0051246 feature_id[642].value > threshold=0.6498909294605255
node_194: feature_name=GO:0009164 feature_id[546].value <= threshold=17.11970329284668
node_195: feature_name=GO:0001909 feature_id[386].value <= threshold=7.292566299438477
node_196: feature_name=GO:0003908 feature_id[11].value <= threshold=1.887226164340973
node_197: feature_name=GO:0009164 feature_id[546].value <= threshold=0.26642198860645294
node_198: feature_name=GO:2001242 feature_id[24].value <= threshold=9.207026481628418
node_199: feature_name=GO:0051246 feature_id[642].value > threshold=0.6504445374011993
node_203: feature_name=GO:0032461 feature_id[608].value <= threshold=2.607542634010315
node_204: feature_name=GO:0007584 feature_id[529].value <= threshold=16.099515914916992
node_205: feature_name=hsa05221 feature_id[349].value <= threshold=9.359850406646729
node_206: feature_name=GO:0002636 feature_id[447].value <= threshold=1.615447759628296
node_207: feature_name=GO:0033993 feature_id[421].value > threshold=2.96540611088858e-05
node_217: feature_name=GO:0002331 feature_id[404].value > threshold=2.1883513927459717
node_331: feature_name=GO:0097192 feature_id[515].value > threshold=0.03834732994437218
Class: negative genes

Rules_290 passed counts:3
node_0: feature_name=GO:0042113 feature_id[0].value <= threshold=13.408552169799805
node_1: feature_name=GO:0007568 feature_id[534].value <= threshold=5.0313897132873535
node_2: feature_name=GO:0002705 feature_id[541].value <= threshold=3.200145721435547
node_3: feature_name=GO:1901525 feature_id[576].value <= threshold=0.4466460347175598
node_4: feature_name=GO:0048539 feature_id[319].value <= threshold=3.0399646759033203

node_5: feature_name=GO:0001910 feature_id[385].value <= threshold=3.7437864542007446
node_6: feature_name=GO:0043200 feature_id[706].value <= threshold=9.307284355163574
node_7: feature_name=GO:0001773 feature_id[308].value <= threshold=4.016931533813477
node_8: feature_name=GO:0090116 feature_id[97].value <= threshold=7.99645471572876
node_9: feature_name=GO:0019814 feature_id[189].value <= threshold=4.525782108306885
node_10: feature_name=GO:1902583 feature_id[215].value <= threshold=17.60310649871826
node_11: feature_name=GO:0045429 feature_id[737].value <= threshold=1.6133361458778381
node_12: feature_name=GO:0003720 feature_id[228].value <= threshold=5.519326210021973
node_13: feature_name=GO:0046006 feature_id[364].value <= threshold=4.9985432624816895
node_14: feature_name=GO:0070424 feature_id[254].value <= threshold=6.233297109603882
node_15: feature_name=GO:0009892 feature_id[320].value <= threshold=59.99180793762207
node_16: feature_name=GO:0007064 feature_id[527].value <= threshold=11.57377815246582
node_17: feature_name=GO:0005575 feature_id[17].value <= threshold=15.417460918426514
node_18: feature_name=GO:0043368 feature_id[31].value <= threshold=4.059496641159058
node_19: feature_name=GO:0005164 feature_id[163].value <= threshold=5.793607711791992
node_20: feature_name=GO:0042130 feature_id[16].value <= threshold=5.073179721832275
node_21: feature_name=GO:0010216 feature_id[282].value <= threshold=4.834584474563599
node_22: feature_name=GO:0009628 feature_id[553].value <= threshold=29.314892768859863
node_23: feature_name=GO:0045628 feature_id[749].value <= threshold=3.1001367568969727
node_24: feature_name=GO:0042288 feature_id[100].value <= threshold=5.174932956695557
node_25: feature_name=GO:0002329 feature_id[406].value <= threshold=2.348930835723877
node_26: feature_name=GO:0051246 feature_id[642].value > threshold=0.6498909294605255
node_194: feature_name=GO:0009164 feature_id[546].value <= threshold=17.11970329284668
node_195: feature_name=GO:0001909 feature_id[386].value <= threshold=7.292566299438477
node_196: feature_name=GO:0003908 feature_id[11].value <= threshold=1.887226164340973
node_197: feature_name=GO:0009164 feature_id[546].value <= threshold=0.26642198860645294
node_198: feature_name=GO:2001242 feature_id[24].value <= threshold=9.207026481628418
node_199: feature_name=GO:0051246 feature_id[642].value > threshold=0.6504445374011993
node_203: feature_name=GO:0032461 feature_id[608].value <= threshold=2.607542634010315
node_204: feature_name=GO:0007584 feature_id[529].value <= threshold=16.099515914916992
node_205: feature_name=hsa05221 feature_id[349].value <= threshold=9.359850406646729
node_206: feature_name=GO:0002636 feature_id[447].value <= threshold=1.615447759628296
node_207: feature_name=GO:0033993 feature_id[421].value > threshold=2.96540611088858e-05
node_217: feature_name=GO:0002331 feature_id[404].value <= threshold=2.1883513927459717
node_218: feature_name=GO:0046498 feature_id[203].value > threshold=4.085246205329895

node_328: feature_name=GO:0071706 feature_id[552].value > threshold=0.21486687660217285
Class: negative genes

Rules_291 passed counts:3
node_0: feature_name=GO:0042113 feature_id[0].value <= threshold=13.408552169799805
node_1: feature_name=GO:0007568 feature_id[534].value <= threshold=5.0313897132873535
node_2: feature_name=GO:0002705 feature_id[541].value <= threshold=3.200145721435547
node_3: feature_name=GO:1901525 feature_id[576].value <= threshold=0.4466460347175598
node_4: feature_name=GO:0048539 feature_id[319].value <= threshold=3.0399646759033203
node_5: feature_name=GO:0001910 feature_id[385].value <= threshold=3.7437864542007446
node_6: feature_name=GO:0043200 feature_id[706].value <= threshold=9.307284355163574
node_7: feature_name=GO:0001773 feature_id[308].value <= threshold=4.016931533813477
node_8: feature_name=GO:0090116 feature_id[97].value <= threshold=7.99645471572876
node_9: feature_name=GO:0019814 feature_id[189].value <= threshold=4.525782108306885
node_10: feature_name=GO:1902583 feature_id[215].value <= threshold=17.60310649871826
node_11: feature_name=GO:0045429 feature_id[737].value <= threshold=1.6133361458778381
node_12: feature_name=GO:0003720 feature_id[228].value <= threshold=5.519326210021973
node_13: feature_name=GO:0046006 feature_id[364].value <= threshold=4.9985432624816895
node_14: feature_name=GO:0070424 feature_id[254].value <= threshold=6.233297109603882
node_15: feature_name=GO:0009892 feature_id[320].value <= threshold=59.99180793762207
node_16: feature_name=GO:0007064 feature_id[527].value <= threshold=11.57377815246582
node_17: feature_name=GO:0005575 feature_id[17].value <= threshold=15.417460918426514
node_18: feature_name=GO:0043368 feature_id[31].value <= threshold=4.059496641159058
node_19: feature_name=GO:0005164 feature_id[163].value <= threshold=5.793607711791992
node_20: feature_name=GO:0042130 feature_id[16].value <= threshold=5.073179721832275
node_21: feature_name=GO:0010216 feature_id[282].value <= threshold=4.834584474563599
node_22: feature_name=GO:0009628 feature_id[553].value <= threshold=29.314892768859863
node_23: feature_name=GO:0045628 feature_id[749].value <= threshold=3.1001367568969727
node_24: feature_name=GO:0042288 feature_id[100].value <= threshold=5.174932956695557
node_25: feature_name=GO:0002329 feature_id[406].value <= threshold=2.348930835723877
node_26: feature_name=GO:0051246 feature_id[642].value > threshold=0.6498909294605255
node_194: feature_name=GO:0009164 feature_id[546].value <= threshold=17.11970329284668
node_195: feature_name=GO:0001909 feature_id[386].value <= threshold=7.292566299438477
node_196: feature_name=GO:0003908 feature_id[11].value <= threshold=1.887226164340973
node_197: feature_name=GO:0009164 feature_id[546].value <= threshold=0.26642198860645294

node_198: feature_name=GO:2001242 feature_id[24].value <= threshold=9.207026481628418
node_199: feature_name=GO:0051246 feature_id[642].value > threshold=0.6504445374011993
node_203: feature_name=GO:0032461 feature_id[608].value <= threshold=2.607542634010315
node_204: feature_name=GO:0007584 feature_id[529].value <= threshold=16.099515914916992
node_205: feature_name=hsa05221 feature_id[349].value <= threshold=9.359850406646729
node_206: feature_name=GO:0002636 feature_id[447].value <= threshold=1.615447759628296
node_207: feature_name=GO:0033993 feature_id[421].value > threshold=2.96540611088858e-05
node_217: feature_name=GO:0002331 feature_id[404].value <= threshold=2.1883513927459717
node_218: feature_name=GO:0046498 feature_id[203].value <= threshold=4.085246205329895
node_219: feature_name=GO:0002832 feature_id[491].value <= threshold=3.0228612422943115
node_220: feature_name=GO:1904868 feature_id[306].value <= threshold=3.8674756288528442
node_221: feature_name=GO:0048545 feature_id[360].value <= threshold=27.38136100769043
node_222: feature_name=GO:0071310 feature_id[760].value <= threshold=2.3522024154663086
node_223: feature_name=GO:0001775 feature_id[505].value <= threshold=9.71301555633545
node_224: feature_name=GO:0071310 feature_id[760].value <= threshold=2.346743583679199
node_225: feature_name=GO:0001772 feature_id[91].value <= threshold=3.252573609352112
node_226: feature_name=GO:0036498 feature_id[359].value <= threshold=12.092026710510254
node_227: feature_name=hsa05340 feature_id[351].value > threshold=0.7677814364433289
node_281: feature_name=GO:0006359 feature_id[87].value > threshold=0.846381276845932
node_295: feature_name=GO:1902275 feature_id[435].value > threshold=0.2884182333946228
Class: positive genes

Rules_292 passed counts:3
node_0: feature_name=GO:0042113 feature_id[0].value <= threshold=13.408552169799805
node_1: feature_name=GO:0007568 feature_id[534].value <= threshold=5.0313897132873535
node_2: feature_name=GO:0002705 feature_id[541].value <= threshold=3.200145721435547
node_3: feature_name=GO:1901525 feature_id[576].value <= threshold=0.4466460347175598
node_4: feature_name=GO:0048539 feature_id[319].value <= threshold=3.0399646759033203
node_5: feature_name=GO:0001910 feature_id[385].value <= threshold=3.7437864542007446
node_6: feature_name=GO:0043200 feature_id[706].value <= threshold=9.307284355163574
node_7: feature_name=GO:0001773 feature_id[308].value <= threshold=4.016931533813477
node_8: feature_name=GO:0090116 feature_id[97].value <= threshold=7.99645471572876
node_9: feature_name=GO:0019814 feature_id[189].value <= threshold=4.525782108306885
node_10: feature_name=GO:1902583 feature_id[215].value <= threshold=17.60310649871826
node_11: feature_name=GO:0045429 feature_id[737].value <= threshold=1.6133361458778381

node_12: feature_name=GO:0003720	feature_id[228].value <= threshold=5.519326210021973
node_13: feature_name=GO:0046006	feature_id[364].value <= threshold=4.9985432624816895
node_14: feature_name=GO:0070424	feature_id[254].value <= threshold=6.233297109603882
node_15: feature_name=GO:0009892	feature_id[320].value <= threshold=59.99180793762207
node_16: feature_name=GO:0007064	feature_id[527].value <= threshold=11.57377815246582
node_17: feature_name=GO:0005575	feature_id[17].value <= threshold=15.417460918426514
node_18: feature_name=GO:0043368	feature_id[31].value <= threshold=4.059496641159058
node_19: feature_name=GO:0005164	feature_id[163].value <= threshold=5.793607711791992
node_20: feature_name=GO:0042130	feature_id[16].value <= threshold=5.073179721832275
node_21: feature_name=GO:0010216	feature_id[282].value <= threshold=4.834584474563599
node_22: feature_name=GO:0009628	feature_id[553].value <= threshold=29.314892768859863
node_23: feature_name=GO:0045628	feature_id[749].value <= threshold=3.1001367568969727
node_24: feature_name=GO:0042288	feature_id[100].value <= threshold=5.174932956695557
node_25: feature_name=GO:0002329	feature_id[406].value <= threshold=2.348930835723877
node_26: feature_name=GO:0051246	feature_id[642].value > threshold=0.6498909294605255
node_194: feature_name=GO:0009164	feature_id[546].value <= threshold=17.11970329284668
node_195: feature_name=GO:0001909	feature_id[386].value <= threshold=7.292566299438477
node_196: feature_name=GO:0003908	feature_id[11].value <= threshold=1.887226164340973
node_197: feature_name=GO:0009164	feature_id[546].value <= threshold=0.26642198860645294
node_198: feature_name=GO:2001242	feature_id[24].value <= threshold=9.207026481628418
node_199: feature_name=GO:0051246	feature_id[642].value > threshold=0.6504445374011993
node_203: feature_name=GO:0032461	feature_id[608].value <= threshold=2.607542634010315
node_204: feature_name=GO:0007584	feature_id[529].value <= threshold=16.099515914916992
node_205: feature_name=hsa05221	feature_id[349].value <= threshold=9.359850406646729
node_206: feature_name=GO:0002636	feature_id[447].value <= threshold=1.615447759628296
node_207: feature_name=GO:0033993	feature_id[421].value > threshold=2.96540611088858e-05
node_217: feature_name=GO:0002331	feature_id[404].value <= threshold=2.1883513927459717
node_218: feature_name=GO:0046498	feature_id[203].value <= threshold=4.085246205329895
node_219: feature_name=GO:0002832	feature_id[491].value <= threshold=3.0228612422943115
node_220: feature_name=GO:1904868	feature_id[306].value <= threshold=3.8674756288528442
node_221: feature_name=GO:0048545	feature_id[360].value <= threshold=27.38136100769043
node_222: feature_name=GO:0071310	feature_id[760].value <= threshold=2.3522024154663086
node_223: feature_name=GO:0001775	feature_id[505].value <= threshold=9.71301555633545
node_224: feature_name=GO:0071310	feature_id[760].value <= threshold=2.346743583679199
node_225: feature_name=GO:0001772	feature_id[91].value <= threshold=3.252573609352112

node_226: feature_name=GO:0036498 feature_id[359].value <= threshold=12.092026710510254
node_227: feature_name=hsa05340 feature_id[351].value <= threshold=0.7677814364433289
node_228: feature_name=GO:0032703 feature_id[617].value <= threshold=1.7450646758079529
node_229: feature_name=hsa04640 feature_id[79].value <= threshold=2.118402123451233
node_230: feature_name=GO:0042097 feature_id[675].value <= threshold=1.8110727667808533
node_231: feature_name=GO:0071391 feature_id[518].value <= threshold=2.691588521003723
node_232: feature_name=GO:0001666 feature_id[682].value > threshold=5.225740432739258
node_266: feature_name=GO:0036294 feature_id[612].value > threshold=3.7958332896232605
Class: negative genes

Rules_293 passed counts:3
node_0: feature_name=GO:0042113 feature_id[0].value <= threshold=13.408552169799805
node_1: feature_name=GO:0007568 feature_id[534].value <= threshold=5.0313897132873535
node_2: feature_name=GO:0002705 feature_id[541].value <= threshold=3.200145721435547
node_3: feature_name=GO:1901525 feature_id[576].value <= threshold=0.4466460347175598
node_4: feature_name=GO:0048539 feature_id[319].value <= threshold=3.0399646759033203
node_5: feature_name=GO:0001910 feature_id[385].value <= threshold=3.7437864542007446
node_6: feature_name=GO:0043200 feature_id[706].value <= threshold=9.307284355163574
node_7: feature_name=GO:0001773 feature_id[308].value <= threshold=4.016931533813477
node_8: feature_name=GO:0090116 feature_id[97].value <= threshold=7.99645471572876
node_9: feature_name=GO:0019814 feature_id[189].value <= threshold=4.525782108306885
node_10: feature_name=GO:1902583 feature_id[215].value <= threshold=17.60310649871826
node_11: feature_name=GO:0045429 feature_id[737].value <= threshold=1.6133361458778381
node_12: feature_name=GO:0003720 feature_id[228].value <= threshold=5.519326210021973
node_13: feature_name=GO:0046006 feature_id[364].value <= threshold=4.9985432624816895
node_14: feature_name=GO:0070424 feature_id[254].value <= threshold=6.233297109603882
node_15: feature_name=GO:0009892 feature_id[320].value <= threshold=59.99180793762207
node_16: feature_name=GO:0007064 feature_id[527].value <= threshold=11.57377815246582
node_17: feature_name=GO:0005575 feature_id[17].value <= threshold=15.417460918426514
node_18: feature_name=GO:0043368 feature_id[31].value <= threshold=4.059496641159058
node_19: feature_name=GO:0005164 feature_id[163].value <= threshold=5.793607711791992
node_20: feature_name=GO:0042130 feature_id[16].value <= threshold=5.073179721832275
node_21: feature_name=GO:0010216 feature_id[282].value <= threshold=4.834584474563599
node_22: feature_name=GO:0009628 feature_id[553].value <= threshold=29.314892768859863
node_23: feature_name=GO:0045628 feature_id[749].value <= threshold=3.1001367568969727

node_24: feature_name=GO:0042288 feature_id[100].value <= threshold=5.174932956695557
node_25: feature_name=GO:0002329 feature_id[406].value <= threshold=2.348930835723877
node_26: feature_name=GO:0051246 feature_id[642].value > threshold=0.6498909294605255
node_194: feature_name=GO:0009164 feature_id[546].value <= threshold=17.11970329284668
node_195: feature_name=GO:0001909 feature_id[386].value <= threshold=7.292566299438477
node_196: feature_name=GO:0003908 feature_id[11].value <= threshold=1.887226164340973
node_197: feature_name=GO:0009164 feature_id[546].value <= threshold=0.26642198860645294
node_198: feature_name=GO:2001242 feature_id[24].value <= threshold=9.207026481628418
node_199: feature_name=GO:0051246 feature_id[642].value > threshold=0.6504445374011993
node_203: feature_name=GO:0032461 feature_id[608].value <= threshold=2.607542634010315
node_204: feature_name=GO:0007584 feature_id[529].value <= threshold=16.099515914916992
node_205: feature_name=hsa05221 feature_id[349].value <= threshold=9.359850406646729
node_206: feature_name=GO:0002636 feature_id[447].value <= threshold=1.615447759628296
node_207: feature_name=GO:0033993 feature_id[421].value <= threshold=2.96540611088858e-05
node_208: feature_name=GO:0006968 feature_id[517].value <= threshold=1.0216356217861176
node_209: feature_name=GO:0048545 feature_id[360].value <= threshold=0.007158883148804307
node_210: feature_name=GO:0002250 feature_id[417].value > threshold=0.3659738004207611
node_212: feature_name=GO:0034644 feature_id[652].value > threshold=0.06354941427707672
Class: positive genes

Rules_294 passed counts:3
node_0: feature_name=GO:0042113 feature_id[0].value <= threshold=13.408552169799805
node_1: feature_name=GO:0007568 feature_id[534].value <= threshold=5.0313897132873535
node_2: feature_name=GO:0002705 feature_id[541].value <= threshold=3.200145721435547
node_3: feature_name=GO:1901525 feature_id[576].value <= threshold=0.4466460347175598
node_4: feature_name=GO:0048539 feature_id[319].value <= threshold=3.0399646759033203
node_5: feature_name=GO:0001910 feature_id[385].value <= threshold=3.7437864542007446
node_6: feature_name=GO:0043200 feature_id[706].value <= threshold=9.307284355163574
node_7: feature_name=GO:0001773 feature_id[308].value <= threshold=4.016931533813477
node_8: feature_name=GO:0090116 feature_id[97].value <= threshold=7.99645471572876
node_9: feature_name=GO:0019814 feature_id[189].value <= threshold=4.525782108306885
node_10: feature_name=GO:1902583 feature_id[215].value <= threshold=17.60310649871826
node_11: feature_name=GO:0045429 feature_id[737].value <= threshold=1.6133361458778381
node_12: feature_name=GO:0003720 feature_id[228].value <= threshold=5.519326210021973
node_13: feature_name=GO:0046006 feature_id[364].value <= threshold=4.9985432624816895

node_14: feature_name=GO:0070424 feature_id[254].value <= threshold=6.233297109603882
node_15: feature_name=GO:0009892 feature_id[320].value <= threshold=59.99180793762207
node_16: feature_name=GO:0007064 feature_id[527].value <= threshold=11.57377815246582
node_17: feature_name=GO:0005575 feature_id[17].value <= threshold=15.417460918426514
node_18: feature_name=GO:0043368 feature_id[31].value <= threshold=4.059496641159058
node_19: feature_name=GO:0005164 feature_id[163].value <= threshold=5.793607711791992
node_20: feature_name=GO:0042130 feature_id[16].value <= threshold=5.073179721832275
node_21: feature_name=GO:0010216 feature_id[282].value <= threshold=4.834584474563599
node_22: feature_name=GO:0009628 feature_id[553].value <= threshold=29.314892768859863
node_23: feature_name=GO:0045628 feature_id[749].value <= threshold=3.1001367568969727
node_24: feature_name=GO:0042288 feature_id[100].value <= threshold=5.174932956695557
node_25: feature_name=GO:0002329 feature_id[406].value <= threshold=2.348930835723877
node_26: feature_name=GO:0051246 feature_id[642].value <= threshold=0.6498909294605255
node_27: feature_name=GO:0042162 feature_id[355].value <= threshold=8.05103588104248
node_28: feature_name=hsa04668 feature_id[333].value <= threshold=5.614926338195801
node_29: feature_name=GO:0032200 feature_id[613].value <= threshold=28.735913276672363
node_30: feature_name=hsa04662 feature_id[334].value <= threshold=9.588344097137451
node_31: feature_name=GO:1901992 feature_id[105].value <= threshold=5.291427850723267
node_32: feature_name=GO:0072341 feature_id[144].value <= threshold=6.01465106010437
node_33: feature_name=GO:0047485 feature_id[121].value <= threshold=6.657836437225342
node_34: feature_name=GO:0090594 feature_id[738].value <= threshold=1.8673912286758423
node_35: feature_name=GO:0043525 feature_id[524].value <= threshold=2.881397008895874
node_36: feature_name=GO:0032504 feature_id[244].value > threshold=17.22207546234131
node_168: feature_name=GO:0044446 feature_id[184].value <= threshold=2.6137850284576416
Class: negative genes

Rules_295 passed counts:3
node_0: feature_name=GO:0042113 feature_id[0].value <= threshold=13.408552169799805
node_1: feature_name=GO:0007568 feature_id[534].value <= threshold=5.0313897132873535
node_2: feature_name=GO:0002705 feature_id[541].value <= threshold=3.200145721435547
node_3: feature_name=GO:1901525 feature_id[576].value <= threshold=0.4466460347175598
node_4: feature_name=GO:0048539 feature_id[319].value <= threshold=3.0399646759033203
node_5: feature_name=GO:0001910 feature_id[385].value <= threshold=3.7437864542007446
node_6: feature_name=GO:0043200 feature_id[706].value <= threshold=9.307284355163574
node_7: feature_name=GO:0001773 feature_id[308].value <= threshold=4.016931533813477

node_8: feature_name=GO:0090116 feature_id[97].value <= threshold=7.99645471572876
node_9: feature_name=GO:0019814 feature_id[189].value <= threshold=4.525782108306885
node_10: feature_name=GO:1902583 feature_id[215].value <= threshold=17.60310649871826
node_11: feature_name=GO:0045429 feature_id[737].value <= threshold=1.6133361458778381
node_12: feature_name=GO:0003720 feature_id[228].value <= threshold=5.519326210021973
node_13: feature_name=GO:0046006 feature_id[364].value <= threshold=4.9985432624816895
node_14: feature_name=GO:0070424 feature_id[254].value <= threshold=6.233297109603882
node_15: feature_name=GO:0009892 feature_id[320].value <= threshold=59.99180793762207
node_16: feature_name=GO:0007064 feature_id[527].value <= threshold=11.57377815246582
node_17: feature_name=GO:0005575 feature_id[17].value <= threshold=15.417460918426514
node_18: feature_name=GO:0043368 feature_id[31].value <= threshold=4.059496641159058
node_19: feature_name=GO:0005164 feature_id[163].value <= threshold=5.793607711791992
node_20: feature_name=GO:0042130 feature_id[16].value <= threshold=5.073179721832275
node_21: feature_name=GO:0010216 feature_id[282].value <= threshold=4.834584474563599
node_22: feature_name=GO:0009628 feature_id[553].value <= threshold=29.314892768859863
node_23: feature_name=GO:0045628 feature_id[749].value <= threshold=3.1001367568969727
node_24: feature_name=GO:0042288 feature_id[100].value <= threshold=5.174932956695557
node_25: feature_name=GO:0002329 feature_id[406].value <= threshold=2.348930835723877
node_26: feature_name=GO:0051246 feature_id[642].value <= threshold=0.6498909294605255
node_27: feature_name=GO:0042162 feature_id[355].value <= threshold=8.05103588104248
node_28: feature_name=hsa04668 feature_id[333].value <= threshold=5.614926338195801
node_29: feature_name=GO:0032200 feature_id[613].value <= threshold=28.735913276672363
node_30: feature_name=hsa04662 feature_id[334].value <= threshold=9.588344097137451
node_31: feature_name=GO:1901992 feature_id[105].value <= threshold=5.291427850723267
node_32: feature_name=GO:0072341 feature_id[144].value <= threshold=6.01465106010437
node_33: feature_name=GO:0047485 feature_id[121].value <= threshold=6.657836437225342
node_34: feature_name=GO:0090594 feature_id[738].value <= threshold=1.8673912286758423
node_35: feature_name=GO:0043525 feature_id[524].value <= threshold=2.881397008895874
node_36: feature_name=GO:0032504 feature_id[244].value <= threshold=17.22207546234131
node_37: feature_name=GO:0051348 feature_id[126].value > threshold=5.933559417724609
node_165: feature_name=GO:0032204 feature_id[747].value <= threshold=2.1291196420788765
Class: negative genes

Rules_296 passed counts:3
node_0: feature_name=GO:0042113 feature_id[0].value <= threshold=13.408552169799805

node_1: feature_name=GO:0007568 feature_id[534].value <= threshold=5.0313897132873535
node_2: feature_name=GO:0002705 feature_id[541].value <= threshold=3.200145721435547
node_3: feature_name=GO:1901525 feature_id[576].value <= threshold=0.4466460347175598
node_4: feature_name=GO:0048539 feature_id[319].value <= threshold=3.0399646759033203
node_5: feature_name=GO:0001910 feature_id[385].value <= threshold=3.7437864542007446
node_6: feature_name=GO:0043200 feature_id[706].value <= threshold=9.307284355163574
node_7: feature_name=GO:0001773 feature_id[308].value <= threshold=4.016931533813477
node_8: feature_name=GO:0090116 feature_id[97].value <= threshold=7.99645471572876
node_9: feature_name=GO:0019814 feature_id[189].value <= threshold=4.525782108306885
node_10: feature_name=GO:1902583 feature_id[215].value <= threshold=17.60310649871826
node_11: feature_name=GO:0045429 feature_id[737].value <= threshold=1.6133361458778381
node_12: feature_name=GO:0003720 feature_id[228].value <= threshold=5.519326210021973
node_13: feature_name=GO:0046006 feature_id[364].value <= threshold=4.9985432624816895
node_14: feature_name=GO:0070424 feature_id[254].value <= threshold=6.233297109603882
node_15: feature_name=GO:0009892 feature_id[320].value <= threshold=59.99180793762207
node_16: feature_name=GO:0007064 feature_id[527].value <= threshold=11.57377815246582
node_17: feature_name=GO:0005575 feature_id[17].value <= threshold=15.417460918426514
node_18: feature_name=GO:0043368 feature_id[31].value <= threshold=4.059496641159058
node_19: feature_name=GO:0005164 feature_id[163].value <= threshold=5.793607711791992
node_20: feature_name=GO:0042130 feature_id[16].value <= threshold=5.073179721832275
node_21: feature_name=GO:0010216 feature_id[282].value <= threshold=4.834584474563599
node_22: feature_name=GO:0009628 feature_id[553].value <= threshold=29.314892768859863
node_23: feature_name=GO:0045628 feature_id[749].value <= threshold=3.1001367568969727
node_24: feature_name=GO:0042288 feature_id[100].value <= threshold=5.174932956695557
node_25: feature_name=GO:0002329 feature_id[406].value <= threshold=2.348930835723877
node_26: feature_name=GO:0051246 feature_id[642].value <= threshold=0.6498909294605255
node_27: feature_name=GO:0042162 feature_id[355].value <= threshold=8.05103588104248
node_28: feature_name=hsa04668 feature_id[333].value <= threshold=5.614926338195801
node_29: feature_name=GO:0032200 feature_id[613].value <= threshold=28.735913276672363
node_30: feature_name=hsa04662 feature_id[334].value <= threshold=9.588344097137451
node_31: feature_name=GO:1901992 feature_id[105].value <= threshold=5.291427850723267
node_32: feature_name=GO:0072341 feature_id[144].value <= threshold=6.01465106010437
node_33: feature_name=GO:0047485 feature_id[121].value <= threshold=6.657836437225342
node_34: feature_name=GO:0090594 feature_id[738].value <= threshold=1.8673912286758423
node_35: feature_name=GO:0043525 feature_id[524].value <= threshold=2.881397008895874

node_36: feature_name=GO:0032504 feature_id[244].value <= threshold=17.22207546234131
node_37: feature_name=GO:0051348 feature_id[126].value <= threshold=5.933559417724609
node_38: feature_name=GO:0042522 feature_id[692].value <= threshold=1.78694087266922
node_39: feature_name=GO:0031100 feature_id[73].value <= threshold=3.2312101125717163
node_40: feature_name=GO:0042493 feature_id[149].value <= threshold=19.771170616149902
node_41: feature_name=GO:0001552 feature_id[366].value <= threshold=2.1711617708206177
node_42: feature_name=GO:0046483 feature_id[391].value <= threshold=320.9667053222656
node_43: feature_name=GO:0002439 feature_id[431].value <= threshold=1.832722783088684
node_44: feature_name=GO:0006555 feature_id[514].value <= threshold=5.321640968322754
node_45: feature_name=GO:0070424 feature_id[254].value <= threshold=3.321003556251526
node_46: feature_name=hsa05144 feature_id[751].value <= threshold=2.635499954223633
node_47: feature_name=GO:0032633 feature_id[627].value <= threshold=1.2592533230781555
node_48: feature_name=GO:0019814 feature_id[189].value <= threshold=2.1583125591278076
node_49: feature_name=GO:1901989 feature_id[30].value <= threshold=3.3150794506073
node_50: feature_name=GO:1903318 feature_id[196].value <= threshold=1.62252539396286
node_51: feature_name=GO:0060576 feature_id[204].value <= threshold=2.1097792387008667
node_52: feature_name=hsa05221 feature_id[349].value > threshold=0.8723124265670776
node_96: feature_name=hsa05221 feature_id[349].value > threshold=0.8756309151649475
node_98: feature_name=GO:0046794 feature_id[794].value <= threshold=6.585465431213379
node_99: feature_name=GO:0005035 feature_id[133].value > threshold=0.6986835598945618
node_109: feature_name=GO:0045637 feature_id[241].value <= threshold=0.3161832243204117
Class: negative genes

Rules_297 passed counts:2
node_0: feature_name=GO:0042113 feature_id[0].value > threshold=13.408552169799805
node_1454: feature_name=GO:0050851 feature_id[29].value > threshold=9.650307655334473
node_1534: feature_name=GO:1902166 feature_id[301].value > threshold=0.303210511803627
node_1548: feature_name=GO:0035872 feature_id[659].value <= threshold=35.09038162231445
node_1549: feature_name=GO:0032069 feature_id[614].value > threshold=3.441983938217163
node_1571: feature_name=GO:0050864 feature_id[288].value > threshold=7.516624450683594
node_1575: feature_name=GO:2000772 feature_id[95].value > threshold=0.9979664981365204
node_1579: feature_name=GO:0043525 feature_id[524].value > threshold=22.19614315032959
node_1591: feature_name=GO:0044238 feature_id[295].value <= threshold=161.96461868286133
Class: negative genes

Rules_298 passed counts:2
node_0: feature_name=GO:0042113 feature_id[0].value > threshold=13.408552169799805
node_1454: feature_name=GO:0050851 feature_id[29].value > threshold=9.650307655334473
node_1534: feature_name=GO:1902166 feature_id[301].value > threshold=0.303210511803627
node_1548: feature_name=GO:0035872 feature_id[659].value <= threshold=35.09038162231445
node_1549: feature_name=GO:0032069 feature_id[614].value > threshold=3.441983938217163
node_1571: feature_name=GO:0050864 feature_id[288].value <= threshold=7.516624450683594
node_1572: feature_name=GO:0060574 feature_id[369].value > threshold=2.804360032081604
Class: positive genes

Rules_299 passed counts:2
node_0: feature_name=GO:0042113 feature_id[0].value > threshold=13.408552169799805
node_1454: feature_name=GO:0050851 feature_id[29].value > threshold=9.650307655334473
node_1534: feature_name=GO:1902166 feature_id[301].value > threshold=0.303210511803627
node_1548: feature_name=GO:0035872 feature_id[659].value <= threshold=35.09038162231445
node_1549: feature_name=GO:0032069 feature_id[614].value <= threshold=3.441983938217163
node_1550: feature_name=GO:0050897 feature_id[164].value <= threshold=1.3939869403839111
node_1551: feature_name=GO:0051454 feature_id[307].value > threshold=0.9144491851329803
node_1565: feature_name=GO:0071453 feature_id[740].value <= threshold=1.420312225818634
Class: negative genes

Rules_300 passed counts:2
node_0: feature_name=GO:0042113 feature_id[0].value > threshold=13.408552169799805
node_1454: feature_name=GO:0050851 feature_id[29].value > threshold=9.650307655334473
node_1534: feature_name=GO:1902166 feature_id[301].value > threshold=0.303210511803627
node_1548: feature_name=GO:0035872 feature_id[659].value <= threshold=35.09038162231445
node_1549: feature_name=GO:0032069 feature_id[614].value <= threshold=3.441983938217163
node_1550: feature_name=GO:0050897 feature_id[164].value <= threshold=1.3939869403839111
node_1551: feature_name=GO:0051454 feature_id[307].value <= threshold=0.9144491851329803
node_1552: feature_name=GO:0035825 feature_id[662].value <= threshold=0.08555268123745918
node_1553: feature_name=GO:0032761 feature_id[633].value > threshold=1.6124996542930603
Class: positive genes

Rules_301 passed counts:2
node_0: feature_name=GO:0042113 feature_id[0].value > threshold=13.408552169799805

node_1454: feature_name=GO:0050851 feature_id[29].value > threshold=9.650307655334473
node_1534: feature_name=GO:1902166 feature_id[301].value <= threshold=0.303210511803627
node_1535: feature_name=GO:0042287 feature_id[20].value <= threshold=1.4291933178901672
node_1536: feature_name=GO:0023026 feature_id[69].value > threshold=1.7696257829666138
node_1542: feature_name=GO:0042288 feature_id[100].value <= threshold=0.17694193124771118
Class: negative genes

Rules_302 passed counts:2
node_0: feature_name=GO:0042113 feature_id[0].value > threshold=13.408552169799805
node_1454: feature_name=GO:0050851 feature_id[29].value <= threshold=9.650307655334473
node_1455: feature_name=GO:0006304 feature_id[510].value <= threshold=5.960662126541138
node_1456: feature_name=GO:0032673 feature_id[623].value > threshold=4.092082738876343
node_1510: feature_name=GO:0046898 feature_id[161].value > threshold=2.2367727756500244
node_1528: feature_name=GO:0038001 feature_id[28].value > threshold=4.990844249725342
Class: negative genes

Rules_303 passed counts:2
node_0: feature_name=GO:0042113 feature_id[0].value > threshold=13.408552169799805
node_1454: feature_name=GO:0050851 feature_id[29].value <= threshold=9.650307655334473
node_1455: feature_name=GO:0006304 feature_id[510].value <= threshold=5.960662126541138
node_1456: feature_name=GO:0032673 feature_id[623].value <= threshold=4.092082738876343
node_1457: feature_name=GO:0002429 feature_id[748].value > threshold=4.626799821853638
node_1463: feature_name=GO:0015672 feature_id[573].value > threshold=0.08226438239216805
node_1475: feature_name=GO:0030217 feature_id[589].value > threshold=19.77315902709961
node_1497: feature_name=GO:0030291 feature_id[147].value <= threshold=4.140083312988281
node_1498: feature_name=GO:0050798 feature_id[170].value <= threshold=12.055219173431396
node_1499: feature_name=GO:2000773 feature_id[114].value > threshold=7.385613679885864
Class: positive genes

Rules_304 passed counts:2
node_0: feature_name=GO:0042113 feature_id[0].value > threshold=13.408552169799805
node_1454: feature_name=GO:0050851 feature_id[29].value <= threshold=9.650307655334473
node_1455: feature_name=GO:0006304 feature_id[510].value <= threshold=5.960662126541138
node_1456: feature_name=GO:0032673 feature_id[623].value <= threshold=4.092082738876343
node_1457: feature_name=GO:0002429 feature_id[748].value > threshold=4.626799821853638

node_1463: feature_name=GO:0015672 feature_id[573].value > threshold=0.08226438239216805
node_1475: feature_name=GO:0030217 feature_id[589].value <= threshold=19.77315902709961
node_1476: feature_name=GO:0002381 feature_id[96].value > threshold=4.645626783370972
node_1490: feature_name=GO:0042113 feature_id[0].value <= threshold=17.225549697875977
node_1491: feature_name=GO:0002902 feature_id[59].value > threshold=3.440378785133362
node_1493: feature_name=GO:0006979 feature_id[44].value > threshold=24.42934513092041
Class: negative genes

Rules_305 passed counts:2
node_0: feature_name=GO:0042113 feature_id[0].value > threshold=13.408552169799805
node_1454: feature_name=GO:0050851 feature_id[29].value <= threshold=9.650307655334473
node_1455: feature_name=GO:0006304 feature_id[510].value <= threshold=5.960662126541138
node_1456: feature_name=GO:0032673 feature_id[623].value <= threshold=4.092082738876343
node_1457: feature_name=GO:0002429 feature_id[748].value > threshold=4.626799821853638
node_1463: feature_name=GO:0015672 feature_id[573].value <= threshold=0.08226438239216805
node_1464: feature_name=GO:0038065 feature_id[670].value > threshold=0.3765842020511627
node_1468: feature_name=GO:0046685 feature_id[782].value <= threshold=2.9275591373443604
node_1469: feature_name=GO:0050897 feature_id[164].value > threshold=2.1307075023651123
node_1471: feature_name=GO:0023056 feature_id[632].value <= threshold=59.85031509399414
Class: negative genes

Rules_306 passed counts:2
node_0: feature_name=GO:0042113 feature_id[0].value > threshold=13.408552169799805
node_1454: feature_name=GO:0050851 feature_id[29].value <= threshold=9.650307655334473
node_1455: feature_name=GO:0006304 feature_id[510].value <= threshold=5.960662126541138
node_1456: feature_name=GO:0032673 feature_id[623].value <= threshold=4.092082738876343
node_1457: feature_name=GO:0002429 feature_id[748].value <= threshold=4.626799821853638
node_1458: feature_name=GO:0005575 feature_id[17].value <= threshold=0.7239105105400085
Class: positive genes

Rules_307 passed counts:2
node_0: feature_name=GO:0042113 feature_id[0].value <= threshold=13.408552169799805
node_1: feature_name=GO:0007568 feature_id[534].value > threshold=5.0313897132873535
node_913: feature_name=GO:0032763 feature_id[629].value > threshold=0.31753237545490265
node_1195: feature_name=GO:0071301 feature_id[442].value > threshold=0.11534593254327774

node_1375: feature_name=GO:0046500 feature_id[171].value > threshold=1.130434513092041
node_1437: feature_name=GO:0033343 feature_id[639].value > threshold=0.98012974858284
node_1443: feature_name=GO:0007126 feature_id[520].value > threshold=0.40390433371067047
node_1447: feature_name=GO:0019058 feature_id[245].value > threshold=9.540197849273682
node_1451: feature_name=hsa04650 feature_id[13].value <= threshold=1.7103192806243896
Class: negative genes

Rules_308 passed counts:2
node_0: feature_name=GO:0042113 feature_id[0].value <= threshold=13.408552169799805
node_1: feature_name=GO:0007568 feature_id[534].value > threshold=5.0313897132873535
node_913: feature_name=GO:0032763 feature_id[629].value > threshold=0.31753237545490265
node_1195: feature_name=GO:0071301 feature_id[442].value > threshold=0.11534593254327774
node_1375: feature_name=GO:0046500 feature_id[171].value <= threshold=1.130434513092041
node_1376: feature_name=GO:0001782 feature_id[240].value <= threshold=6.639636993408203
node_1377: feature_name=GO:0043371 feature_id[707].value <= threshold=2.562113642692566
node_1378: feature_name=GO:0030291 feature_id[147].value <= threshold=6.834916830062866
node_1379: feature_name=GO:0045656 feature_id[756].value > threshold=0.6281269192695618
node_1393: feature_name=GO:0046007 feature_id[152].value > threshold=1.020785927772522
node_1419: feature_name=GO:0009636 feature_id[551].value <= threshold=15.796520233154297
node_1420: feature_name=GO:0008588 feature_id[26].value > threshold=0.5794805586338043
Class: negative genes

Rules_309 passed counts:2
node_0: feature_name=GO:0042113 feature_id[0].value <= threshold=13.408552169799805
node_1: feature_name=GO:0007568 feature_id[534].value > threshold=5.0313897132873535
node_913: feature_name=GO:0032763 feature_id[629].value > threshold=0.31753237545490265
node_1195: feature_name=GO:0071301 feature_id[442].value > threshold=0.11534593254327774
node_1375: feature_name=GO:0046500 feature_id[171].value <= threshold=1.130434513092041
node_1376: feature_name=GO:0001782 feature_id[240].value <= threshold=6.639636993408203
node_1377: feature_name=GO:0043371 feature_id[707].value <= threshold=2.562113642692566
node_1378: feature_name=GO:0030291 feature_id[147].value <= threshold=6.834916830062866
node_1379: feature_name=GO:0045656 feature_id[756].value > threshold=0.6281269192695618
node_1393: feature_name=GO:0046007 feature_id[152].value <= threshold=1.020785927772522
node_1394: feature_name=GO:0000302 feature_id[375].value <= threshold=20.847331047058105
node_1395: feature_name=hsa04110 feature_id[328].value > threshold=43.297359466552734

node_1411: feature_name=GO:0002318 feature_id[413].value <= threshold=0.8484916985034943
node_1412: feature_name=GO:0045191 feature_id[730].value <= threshold=0.5140747725963593
Class: positive genes

Rules_310 passed counts:2
node_0: feature_name=GO:0042113 feature_id[0].value <= threshold=13.408552169799805
node_1: feature_name=GO:0007568 feature_id[534].value > threshold=5.0313897132873535
node_913: feature_name=GO:0032763 feature_id[629].value > threshold=0.31753237545490265
node_1195: feature_name=GO:0071301 feature_id[442].value > threshold=0.11534593254327774
node_1375: feature_name=GO:0046500 feature_id[171].value <= threshold=1.130434513092041
node_1376: feature_name=GO:0001782 feature_id[240].value <= threshold=6.639636993408203
node_1377: feature_name=GO:0043371 feature_id[707].value <= threshold=2.562113642692566
node_1378: feature_name=GO:0030291 feature_id[147].value <= threshold=6.834916830062866
node_1379: feature_name=GO:0045656 feature_id[756].value <= threshold=0.6281269192695618
node_1380: feature_name=GO:0034103 feature_id[318].value > threshold=0.0158024481497705
node_1382: feature_name=GO:0048872 feature_id[498].value > threshold=19.454792976379395
node_1390: feature_name=GO:0043011 feature_id[150].value <= threshold=3.861676812171936
Class: positive genes

Rules_311 passed counts:2
node_0: feature_name=GO:0042113 feature_id[0].value <= threshold=13.408552169799805
node_1: feature_name=GO:0007568 feature_id[534].value > threshold=5.0313897132873535
node_913: feature_name=GO:0032763 feature_id[629].value > threshold=0.31753237545490265
node_1195: feature_name=GO:0071301 feature_id[442].value > threshold=0.11534593254327774
node_1375: feature_name=GO:0046500 feature_id[171].value <= threshold=1.130434513092041
node_1376: feature_name=GO:0001782 feature_id[240].value <= threshold=6.639636993408203
node_1377: feature_name=GO:0043371 feature_id[707].value <= threshold=2.562113642692566
node_1378: feature_name=GO:0030291 feature_id[147].value <= threshold=6.834916830062866
node_1379: feature_name=GO:0045656 feature_id[756].value <= threshold=0.6281269192695618
node_1380: feature_name=GO:0034103 feature_id[318].value <= threshold=0.0158024481497705
Class: positive genes

Rules_312 passed counts:2
node_0: feature_name=GO:0042113 feature_id[0].value <= threshold=13.408552169799805
node_1: feature_name=GO:0007568 feature_id[534].value > threshold=5.0313897132873535

node_913: feature_name=GO:0032763 feature_id[629].value > threshold=0.31753237545490265
node_1195: feature_name=GO:0071301 feature_id[442].value <= threshold=0.11534593254327774
node_1196: feature_name=GO:0046500 feature_id[171].value > threshold=0.6314916908740997
node_1340: feature_name=GO:0043627 feature_id[562].value > threshold=25.17277240753174
node_1368: feature_name=GO:0071310 feature_id[760].value > threshold=80.00252914428711
node_1372: feature_name=GO:0044403 feature_id[723].value <= threshold=6.509235143661499
Class: positive genes

Rules_313 passed counts:2
node_0: feature_name=GO:0042113 feature_id[0].value <= threshold=13.408552169799805
node_1: feature_name=GO:0007568 feature_id[534].value > threshold=5.0313897132873535
node_913: feature_name=GO:0032763 feature_id[629].value > threshold=0.31753237545490265
node_1195: feature_name=GO:0071301 feature_id[442].value <= threshold=0.11534593254327774
node_1196: feature_name=GO:0046500 feature_id[171].value > threshold=0.6314916908740997
node_1340: feature_name=GO:0043627 feature_id[562].value <= threshold=25.17277240753174
node_1341: feature_name=GO:0010835 feature_id[560].value <= threshold=1.0601619482040405
node_1342: feature_name=GO:0016363 feature_id[90].value <= threshold=6.534365892410278
node_1343: feature_name=GO:0030983 feature_id[291].value > threshold=3.5806901454925537
node_1359: feature_name=GO:0046632 feature_id[248].value > threshold=0.5181337743997574
node_1361: feature_name=GO:0033554 feature_id[418].value <= threshold=43.66302299499512
node_1362: feature_name=GO:2000773 feature_id[114].value <= threshold=0.22911779582500458
Class: negative genes

Rules_314 passed counts:2
node_0: feature_name=GO:0042113 feature_id[0].value <= threshold=13.408552169799805
node_1: feature_name=GO:0007568 feature_id[534].value > threshold=5.0313897132873535
node_913: feature_name=GO:0032763 feature_id[629].value > threshold=0.31753237545490265
node_1195: feature_name=GO:0071301 feature_id[442].value <= threshold=0.11534593254327774
node_1196: feature_name=GO:0046500 feature_id[171].value > threshold=0.6314916908740997
node_1340: feature_name=GO:0043627 feature_id[562].value <= threshold=25.17277240753174
node_1341: feature_name=GO:0010835 feature_id[560].value <= threshold=1.0601619482040405
node_1342: feature_name=GO:0016363 feature_id[90].value <= threshold=6.534365892410278
node_1343: feature_name=GO:0030983 feature_id[291].value <= threshold=3.5806901454925537
node_1344: feature_name=GO:0023030 feature_id[45].value <= threshold=0.6541633009910583
node_1345: feature_name=GO:0070245 feature_id[326].value <= threshold=1.9657342433929443

node_1346: feature_name=GO:0050897 feature_id[164].value > threshold=1.6622408628463745
Class: positive genes

Rules_315 passed counts:2
node_0: feature_name=GO:0042113 feature_id[0].value <= threshold=13.408552169799805
node_1: feature_name=GO:0007568 feature_id[534].value > threshold=5.0313897132873535
node_913: feature_name=GO:0032763 feature_id[629].value > threshold=0.31753237545490265
node_1195: feature_name=GO:0071301 feature_id[442].value <= threshold=0.11534593254327774
node_1196: feature_name=GO:0046500 feature_id[171].value > threshold=0.6314916908740997
node_1340: feature_name=GO:0043627 feature_id[562].value <= threshold=25.17277240753174
node_1341: feature_name=GO:0010835 feature_id[560].value <= threshold=1.0601619482040405
node_1342: feature_name=GO:0016363 feature_id[90].value <= threshold=6.534365892410278
node_1343: feature_name=GO:0030983 feature_id[291].value <= threshold=3.5806901454925537
node_1344: feature_name=GO:0023030 feature_id[45].value <= threshold=0.6541633009910583
node_1345: feature_name=GO:0070245 feature_id[326].value <= threshold=1.9657342433929443
node_1346: feature_name=GO:0050897 feature_id[164].value <= threshold=1.6622408628463745
node_1347: feature_name=GO:0052548 feature_id[205].value <= threshold=0.1624109297990799
Class: positive genes

Rules_316 passed counts:2
node_0: feature_name=GO:0042113 feature_id[0].value <= threshold=13.408552169799805
node_1: feature_name=GO:0007568 feature_id[534].value > threshold=5.0313897132873535
node_913: feature_name=GO:0032763 feature_id[629].value > threshold=0.31753237545490265
node_1195: feature_name=GO:0071301 feature_id[442].value <= threshold=0.11534593254327774
node_1196: feature_name=GO:0046500 feature_id[171].value <= threshold=0.6314916908740997
node_1197: feature_name=GO:0030852 feature_id[22].value > threshold=2.9768868684768677
node_1329: feature_name=GO:0046668 feature_id[125].value > threshold=0.9442969560623169
node_1333: feature_name=GO:0006298 feature_id[621].value <= threshold=0.45032089948654175
node_1334: feature_name=GO:0046449 feature_id[763].value <= threshold=0.2017408013343811
Class: positive genes

Rules_317 passed counts:2
node_0: feature_name=GO:0042113 feature_id[0].value <= threshold=13.408552169799805
node_1: feature_name=GO:0007568 feature_id[534].value > threshold=5.0313897132873535
node_913: feature_name=GO:0032763 feature_id[629].value > threshold=0.31753237545490265

node_1195: feature_name=GO:0071301 feature_id[442].value <= threshold=0.11534593254327774
node_1196: feature_name=GO:0046500 feature_id[171].value <= threshold=0.6314916908740997
node_1197: feature_name=GO:0030852 feature_id[22].value > threshold=2.9768868684768677
node_1329: feature_name=GO:0046668 feature_id[125].value <= threshold=0.9442969560623169
node_1330: feature_name=GO:0002467 feature_id[39].value > threshold=2.990131974220276
Class: positive genes

Rules_318 passed counts:2
node_0: feature_name=GO:0042113 feature_id[0].value <= threshold=13.408552169799805
node_1: feature_name=GO:0007568 feature_id[534].value > threshold=5.0313897132873535
node_913: feature_name=GO:0032763 feature_id[629].value > threshold=0.31753237545490265
node_1195: feature_name=GO:0071301 feature_id[442].value <= threshold=0.11534593254327774
node_1196: feature_name=GO:0046500 feature_id[171].value <= threshold=0.6314916908740997
node_1197: feature_name=GO:0030852 feature_id[22].value <= threshold=2.9768868684768677
node_1198: feature_name=hsa04672 feature_id[276].value <= threshold=8.06681227684021
node_1199: feature_name=GO:0001836 feature_id[450].value > threshold=0.09961023926734924
node_1201: feature_name=GO:0070230 feature_id[64].value > threshold=3.3956379890441895
node_1323: feature_name=GO:0032069 feature_id[614].value <= threshold=1.899479329586029
Class: negative genes

Rules_319 passed counts:2
node_0: feature_name=GO:0042113 feature_id[0].value <= threshold=13.408552169799805
node_1: feature_name=GO:0007568 feature_id[534].value > threshold=5.0313897132873535
node_913: feature_name=GO:0032763 feature_id[629].value > threshold=0.31753237545490265
node_1195: feature_name=GO:0071301 feature_id[442].value <= threshold=0.11534593254327774
node_1196: feature_name=GO:0046500 feature_id[171].value <= threshold=0.6314916908740997
node_1197: feature_name=GO:0030852 feature_id[22].value <= threshold=2.9768868684768677
node_1198: feature_name=hsa04672 feature_id[276].value <= threshold=8.06681227684021
node_1199: feature_name=GO:0001836 feature_id[450].value > threshold=0.09961023926734924
node_1201: feature_name=GO:0070230 feature_id[64].value <= threshold=3.3956379890441895
node_1202: feature_name=GO:0070102 feature_id[49].value <= threshold=4.805386543273926
node_1203: feature_name=GO:0045553 feature_id[736].value <= threshold=1.4970332980155945
node_1204: feature_name=GO:0051311 feature_id[58].value > threshold=3.301889181137085
Class: positive genes

Rules_320 passed counts:2
node_0: feature_name=GO:0042113 feature_id[0].value <= threshold=13.408552169799805
node_1: feature_name=GO:0007568 feature_id[534].value > threshold=5.0313897132873535
node_913: feature_name=GO:0032763 feature_id[629].value > threshold=0.31753237545490265
node_1195: feature_name=GO:0071301 feature_id[442].value <= threshold=0.11534593254327774
node_1196: feature_name=GO:0046500 feature_id[171].value <= threshold=0.6314916908740997
node_1197: feature_name=GO:0030852 feature_id[22].value <= threshold=2.9768868684768677
node_1198: feature_name=hsa04672 feature_id[276].value <= threshold=8.06681227684021
node_1199: feature_name=GO:0001836 feature_id[450].value > threshold=0.09961023926734924
node_1201: feature_name=GO:0070230 feature_id[64].value <= threshold=3.3956379890441895
node_1202: feature_name=GO:0070102 feature_id[49].value <= threshold=4.805386543273926
node_1203: feature_name=GO:0045553 feature_id[736].value <= threshold=1.4970332980155945
node_1204: feature_name=GO:0051311 feature_id[58].value <= threshold=3.301889181137085
node_1205: feature_name=GO:0030291 feature_id[147].value > threshold=8.156315326690674
Class: positive genes

Rules_321 passed counts:2
node_0: feature_name=GO:0042113 feature_id[0].value <= threshold=13.408552169799805
node_1: feature_name=GO:0007568 feature_id[534].value > threshold=5.0313897132873535
node_913: feature_name=GO:0032763 feature_id[629].value > threshold=0.31753237545490265
node_1195: feature_name=GO:0071301 feature_id[442].value <= threshold=0.11534593254327774
node_1196: feature_name=GO:0046500 feature_id[171].value <= threshold=0.6314916908740997
node_1197: feature_name=GO:0030852 feature_id[22].value <= threshold=2.9768868684768677
node_1198: feature_name=hsa04672 feature_id[276].value <= threshold=8.06681227684021
node_1199: feature_name=GO:0001836 feature_id[450].value > threshold=0.09961023926734924
node_1201: feature_name=GO:0070230 feature_id[64].value <= threshold=3.3956379890441895
node_1202: feature_name=GO:0070102 feature_id[49].value <= threshold=4.805386543273926
node_1203: feature_name=GO:0045553 feature_id[736].value <= threshold=1.4970332980155945
node_1204: feature_name=GO:0051311 feature_id[58].value <= threshold=3.301889181137085
node_1205: feature_name=GO:0030291 feature_id[147].value <= threshold=8.156315326690674
node_1206: feature_name=GO:0045637 feature_id[241].value > threshold=0.01834342861548066
node_1208: feature_name=GO:0002890 feature_id[98].value > threshold=2.767483353614807
Class: positive genes

Rules_322 passed counts:2

node_0: feature_name=GO:0042113	feature_id[0].value <= threshold=13.408552169799805
node_1: feature_name=GO:0007568	feature_id[534].value > threshold=5.0313897132873535
node_913: feature_name=GO:0032763	feature_id[629].value > threshold=0.31753237545490265
node_1195: feature_name=GO:0071301	feature_id[442].value <= threshold=0.11534593254327774
node_1196: feature_name=GO:0046500	feature_id[171].value <= threshold=0.6314916908740997
node_1197: feature_name=GO:0030852	feature_id[22].value <= threshold=2.9768868684768677
node_1198: feature_name=hsa04672	feature_id[276].value <= threshold=8.06681227684021
node_1199: feature_name=GO:0001836	feature_id[450].value > threshold=0.09961023926734924
node_1201: feature_name=GO:0070230	feature_id[64].value <= threshold=3.3956379890441895
node_1202: feature_name=GO:0070102	feature_id[49].value <= threshold=4.805386543273926
node_1203: feature_name=GO:0045553	feature_id[736].value <= threshold=1.4970332980155945
node_1204: feature_name=GO:0051311	feature_id[58].value <= threshold=3.301889181137085
node_1205: feature_name=GO:0030291	feature_id[147].value <= threshold=8.156315326690674
node_1206: feature_name=GO:0045637	feature_id[241].value > threshold=0.01834342861548066
node_1208: feature_name=GO:0002890	feature_id[98].value <= threshold=2.767483353614807
node_1209: feature_name=GO:0046632	feature_id[248].value > threshold=11.663254261016846
Class: positive genes	

Rules_323	passed counts:2
node_0: feature_name=GO:0042113	feature_id[0].value <= threshold=13.408552169799805
node_1: feature_name=GO:0007568	feature_id[534].value > threshold=5.0313897132873535
node_913: feature_name=GO:0032763	feature_id[629].value > threshold=0.31753237545490265
node_1195: feature_name=GO:0071301	feature_id[442].value <= threshold=0.11534593254327774
node_1196: feature_name=GO:0046500	feature_id[171].value <= threshold=0.6314916908740997
node_1197: feature_name=GO:0030852	feature_id[22].value <= threshold=2.9768868684768677
node_1198: feature_name=hsa04672	feature_id[276].value <= threshold=8.06681227684021
node_1199: feature_name=GO:0001836	feature_id[450].value > threshold=0.09961023926734924
node_1201: feature_name=GO:0070230	feature_id[64].value <= threshold=3.3956379890441895
node_1202: feature_name=GO:0070102	feature_id[49].value <= threshold=4.805386543273926
node_1203: feature_name=GO:0045553	feature_id[736].value <= threshold=1.4970332980155945
node_1204: feature_name=GO:0051311	feature_id[58].value <= threshold=3.301889181137085
node_1205: feature_name=GO:0030291	feature_id[147].value <= threshold=8.156315326690674
node_1206: feature_name=GO:0045637	feature_id[241].value > threshold=0.01834342861548066
node_1208: feature_name=GO:0002890	feature_id[98].value <= threshold=2.767483353614807
node_1209: feature_name=GO:0046632	feature_id[248].value <= threshold=11.663254261016846

node_1210: feature_name=GO:0008588 feature_id[26].value <= threshold=1.391375720500946
node_1211: feature_name=GO:0010639 feature_id[564].value > threshold=18.975126266479492
node_1305: feature_name=GO:0045840 feature_id[697].value <= threshold=8.670292615890503
Class: positive genes

Rules_324 passed counts:2
node_0: feature_name=GO:0042113 feature_id[0].value <= threshold=13.408552169799805
node_1: feature_name=GO:0007568 feature_id[534].value > threshold=5.0313897132873535
node_913: feature_name=GO:0032763 feature_id[629].value > threshold=0.31753237545490265
node_1195: feature_name=GO:0071301 feature_id[442].value <= threshold=0.11534593254327774
node_1196: feature_name=GO:0046500 feature_id[171].value <= threshold=0.6314916908740997
node_1197: feature_name=GO:0030852 feature_id[22].value <= threshold=2.9768868684768677
node_1198: feature_name=hsa04672 feature_id[276].value <= threshold=8.06681227684021
node_1199: feature_name=GO:0001836 feature_id[450].value > threshold=0.09961023926734924
node_1201: feature_name=GO:0070230 feature_id[64].value <= threshold=3.3956379890441895
node_1202: feature_name=GO:0070102 feature_id[49].value <= threshold=4.805386543273926
node_1203: feature_name=GO:0045553 feature_id[736].value <= threshold=1.4970332980155945
node_1204: feature_name=GO:0051311 feature_id[58].value <= threshold=3.301889181137085
node_1205: feature_name=GO:0030291 feature_id[147].value <= threshold=8.156315326690674
node_1206: feature_name=GO:0045637 feature_id[241].value > threshold=0.01834342861548066
node_1208: feature_name=GO:0002890 feature_id[98].value <= threshold=2.767483353614807
node_1209: feature_name=GO:0046632 feature_id[248].value <= threshold=11.663254261016846
node_1210: feature_name=GO:0008588 feature_id[26].value <= threshold=1.391375720500946
node_1211: feature_name=GO:0010639 feature_id[564].value <= threshold=18.975126266479492
node_1212: feature_name=GO:0046685 feature_id[782].value <= threshold=5.153738260269165
node_1213: feature_name=GO:0038116 feature_id[668].value > threshold=1.7859655618667603
node_1293: feature_name=hsa05142 feature_id[769].value <= threshold=11.968927383422852
Class: positive genes

Rules_325 passed counts:2
node_0: feature_name=GO:0042113 feature_id[0].value <= threshold=13.408552169799805
node_1: feature_name=GO:0007568 feature_id[534].value > threshold=5.0313897132873535
node_913: feature_name=GO:0032763 feature_id[629].value > threshold=0.31753237545490265
node_1195: feature_name=GO:0071301 feature_id[442].value <= threshold=0.11534593254327774
node_1196: feature_name=GO:0046500 feature_id[171].value <= threshold=0.6314916908740997

node_1197: feature_name=GO:0030852 feature_id[22].value <= threshold=2.9768868684768677
node_1198: feature_name=hsa04672 feature_id[276].value <= threshold=8.06681227684021
node_1199: feature_name=GO:0001836 feature_id[450].value > threshold=0.09961023926734924
node_1201: feature_name=GO:0070230 feature_id[64].value <= threshold=3.3956379890441895
node_1202: feature_name=GO:0070102 feature_id[49].value <= threshold=4.805386543273926
node_1203: feature_name=GO:0045553 feature_id[736].value <= threshold=1.4970332980155945
node_1204: feature_name=GO:0051311 feature_id[58].value <= threshold=3.301889181137085
node_1205: feature_name=GO:0030291 feature_id[147].value <= threshold=8.156315326690674
node_1206: feature_name=GO:0045637 feature_id[241].value > threshold=0.01834342861548066
node_1208: feature_name=GO:0002890 feature_id[98].value <= threshold=2.767483353614807
node_1209: feature_name=GO:0046632 feature_id[248].value <= threshold=11.663254261016846
node_1210: feature_name=GO:0008588 feature_id[26].value <= threshold=1.391375720500946
node_1211: feature_name=GO:0010639 feature_id[564].value <= threshold=18.975126266479492
node_1212: feature_name=GO:0046685 feature_id[782].value <= threshold=5.153738260269165
node_1213: feature_name=GO:0038116 feature_id[668].value <= threshold=1.7859655618667603
node_1214: feature_name=GO:0046449 feature_id[763].value > threshold=5.603115558624268
node_1290: feature_name=GO:0042770 feature_id[688].value <= threshold=1.4568010568618774
Class: positive genes

Rules_326 passed counts:2
node_0: feature_name=GO:0042113 feature_id[0].value <= threshold=13.408552169799805
node_1: feature_name=GO:0007568 feature_id[534].value > threshold=5.0313897132873535
node_913: feature_name=GO:0032763 feature_id[629].value > threshold=0.31753237545490265
node_1195: feature_name=GO:0071301 feature_id[442].value <= threshold=0.11534593254327774
node_1196: feature_name=GO:0046500 feature_id[171].value <= threshold=0.6314916908740997
node_1197: feature_name=GO:0030852 feature_id[22].value <= threshold=2.9768868684768677
node_1198: feature_name=hsa04672 feature_id[276].value <= threshold=8.06681227684021
node_1199: feature_name=GO:0001836 feature_id[450].value > threshold=0.09961023926734924
node_1201: feature_name=GO:0070230 feature_id[64].value <= threshold=3.3956379890441895
node_1202: feature_name=GO:0070102 feature_id[49].value <= threshold=4.805386543273926
node_1203: feature_name=GO:0045553 feature_id[736].value <= threshold=1.4970332980155945
node_1204: feature_name=GO:0051311 feature_id[58].value <= threshold=3.301889181137085
node_1205: feature_name=GO:0030291 feature_id[147].value <= threshold=8.156315326690674
node_1206: feature_name=GO:0045637 feature_id[241].value > threshold=0.01834342861548066
node_1208: feature_name=GO:0002890 feature_id[98].value <= threshold=2.767483353614807

node_1209: feature_name=GO:0046632 feature_id[248].value <= threshold=11.663254261016846
node_1210: feature_name=GO:0008588 feature_id[26].value <= threshold=1.391375720500946
node_1211: feature_name=GO:0010639 feature_id[564].value <= threshold=18.975126266479492
node_1212: feature_name=GO:0046685 feature_id[782].value <= threshold=5.153738260269165
node_1213: feature_name=GO:0038116 feature_id[668].value <= threshold=1.7859655618667603
node_1214: feature_name=GO:0046449 feature_id[763].value <= threshold=5.603115558624268
node_1215: feature_name=GO:0048539 feature_id[319].value > threshold=0.5478232800960541
node_1269: feature_name=GO:0072341 feature_id[144].value > threshold=1.3205850720405579
node_1287: feature_name=GO:0045346 feature_id[426].value > threshold=3.6343181133270264
Class: negative genes

Rules_327 passed counts:2
node_0: feature_name=GO:0042113 feature_id[0].value <= threshold=13.408552169799805
node_1: feature_name=GO:0007568 feature_id[534].value > threshold=5.0313897132873535
node_913: feature_name=GO:0032763 feature_id[629].value > threshold=0.31753237545490265
node_1195: feature_name=GO:0071301 feature_id[442].value <= threshold=0.11534593254327774
node_1196: feature_name=GO:0046500 feature_id[171].value <= threshold=0.6314916908740997
node_1197: feature_name=GO:0030852 feature_id[22].value <= threshold=2.9768868684768677
node_1198: feature_name=hsa04672 feature_id[276].value <= threshold=8.06681227684021
node_1199: feature_name=GO:0001836 feature_id[450].value > threshold=0.09961023926734924
node_1201: feature_name=GO:0070230 feature_id[64].value <= threshold=3.3956379890441895
node_1202: feature_name=GO:0070102 feature_id[49].value <= threshold=4.805386543273926
node_1203: feature_name=GO:0045553 feature_id[736].value <= threshold=1.4970332980155945
node_1204: feature_name=GO:0051311 feature_id[58].value <= threshold=3.301889181137085
node_1205: feature_name=GO:0030291 feature_id[147].value <= threshold=8.156315326690674
node_1206: feature_name=GO:0045637 feature_id[241].value > threshold=0.01834342861548066
node_1208: feature_name=GO:0002890 feature_id[98].value <= threshold=2.767483353614807
node_1209: feature_name=GO:0046632 feature_id[248].value <= threshold=11.663254261016846
node_1210: feature_name=GO:0008588 feature_id[26].value <= threshold=1.391375720500946
node_1211: feature_name=GO:0010639 feature_id[564].value <= threshold=18.975126266479492
node_1212: feature_name=GO:0046685 feature_id[782].value <= threshold=5.153738260269165
node_1213: feature_name=GO:0038116 feature_id[668].value <= threshold=1.7859655618667603
node_1214: feature_name=GO:0046449 feature_id[763].value <= threshold=5.603115558624268
node_1215: feature_name=GO:0048539 feature_id[319].value <= threshold=0.5478232800960541
node_1216: feature_name=GO:0007089 feature_id[522].value > threshold=0.9940084517002106

node_1266: feature_name=GO:0042129 feature_id[593].value > threshold=0.9326772093772888
Class: positive genes

Rules_328 passed counts:2
node_0: feature_name=GO:0042113 feature_id[0].value <= threshold=13.408552169799805
node_1: feature_name=GO:0007568 feature_id[534].value > threshold=5.0313897132873535
node_913: feature_name=GO:0032763 feature_id[629].value > threshold=0.31753237545490265
node_1195: feature_name=GO:0071301 feature_id[442].value <= threshold=0.11534593254327774
node_1196: feature_name=GO:0046500 feature_id[171].value <= threshold=0.6314916908740997
node_1197: feature_name=GO:0030852 feature_id[22].value <= threshold=2.9768868684768677
node_1198: feature_name=hsa04672 feature_id[276].value <= threshold=8.06681227684021
node_1199: feature_name=GO:0001836 feature_id[450].value > threshold=0.09961023926734924
node_1201: feature_name=GO:0070230 feature_id[64].value <= threshold=3.3956379890441895
node_1202: feature_name=GO:0070102 feature_id[49].value <= threshold=4.805386543273926
node_1203: feature_name=GO:0045553 feature_id[736].value <= threshold=1.4970332980155945
node_1204: feature_name=GO:0051311 feature_id[58].value <= threshold=3.301889181137085
node_1205: feature_name=GO:0030291 feature_id[147].value <= threshold=8.156315326690674
node_1206: feature_name=GO:0045637 feature_id[241].value > threshold=0.01834342861548066
node_1208: feature_name=GO:0002890 feature_id[98].value <= threshold=2.767483353614807
node_1209: feature_name=GO:0046632 feature_id[248].value <= threshold=11.663254261016846
node_1210: feature_name=GO:0008588 feature_id[26].value <= threshold=1.391375720500946
node_1211: feature_name=GO:0010639 feature_id[564].value <= threshold=18.975126266479492
node_1212: feature_name=GO:0046685 feature_id[782].value <= threshold=5.153738260269165
node_1213: feature_name=GO:0038116 feature_id[668].value <= threshold=1.7859655618667603
node_1214: feature_name=GO:0046449 feature_id[763].value <= threshold=5.603115558624268
node_1215: feature_name=GO:0048539 feature_id[319].value <= threshold=0.5478232800960541
node_1216: feature_name=GO:0007089 feature_id[522].value <= threshold=0.9940084517002106
node_1217: feature_name=GO:0042287 feature_id[20].value <= threshold=2.693045496940613
node_1218: feature_name=GO:0044446 feature_id[184].value <= threshold=113.0758171081543
node_1219: feature_name=GO:0098602 feature_id[702].value <= threshold=68.95328903198242
node_1220: feature_name=GO:0035726 feature_id[270].value <= threshold=1.7167096138000488
node_1221: feature_name=GO:1902564 feature_id[85].value <= threshold=2.8364468812942505
node_1222: feature_name=GO:0006979 feature_id[44].value > threshold=0.6869173645973206
node_1224: feature_name=GO:0044464 feature_id[56].value <= threshold=67.13260269165039
node_1225: feature_name=GO:0045629 feature_id[746].value <= threshold=2.3414241075515747

node_1226: feature_name=GO:0002903 feature_id[10].value > threshold=2.715258240699768
node_1252: feature_name=GO:0051984 feature_id[459].value <= threshold=0.44557371735572815
Class: positive genes

Rules_329 passed counts:2
node_0: feature_name=GO:0042113 feature_id[0].value <= threshold=13.408552169799805
node_1: feature_name=GO:0007568 feature_id[534].value > threshold=5.0313897132873535
node_913: feature_name=GO:0032763 feature_id[629].value > threshold=0.31753237545490265
node_1195: feature_name=GO:0071301 feature_id[442].value <= threshold=0.11534593254327774
node_1196: feature_name=GO:0046500 feature_id[171].value <= threshold=0.6314916908740997
node_1197: feature_name=GO:0030852 feature_id[22].value <= threshold=2.9768868684768677
node_1198: feature_name=hsa04672 feature_id[276].value <= threshold=8.06681227684021
node_1199: feature_name=GO:0001836 feature_id[450].value > threshold=0.09961023926734924
node_1201: feature_name=GO:0070230 feature_id[64].value <= threshold=3.3956379890441895
node_1202: feature_name=GO:0070102 feature_id[49].value <= threshold=4.805386543273926
node_1203: feature_name=GO:0045553 feature_id[736].value <= threshold=1.4970332980155945
node_1204: feature_name=GO:0051311 feature_id[58].value <= threshold=3.301889181137085
node_1205: feature_name=GO:0030291 feature_id[147].value <= threshold=8.156315326690674
node_1206: feature_name=GO:0045637 feature_id[241].value > threshold=0.01834342861548066
node_1208: feature_name=GO:0002890 feature_id[98].value <= threshold=2.767483353614807
node_1209: feature_name=GO:0046632 feature_id[248].value <= threshold=11.663254261016846
node_1210: feature_name=GO:0008588 feature_id[26].value <= threshold=1.391375720500946
node_1211: feature_name=GO:0010639 feature_id[564].value <= threshold=18.975126266479492
node_1212: feature_name=GO:0046685 feature_id[782].value <= threshold=5.153738260269165
node_1213: feature_name=GO:0038116 feature_id[668].value <= threshold=1.7859655618667603
node_1214: feature_name=GO:0046449 feature_id[763].value <= threshold=5.603115558624268
node_1215: feature_name=GO:0048539 feature_id[319].value <= threshold=0.5478232800960541
node_1216: feature_name=GO:0007089 feature_id[522].value <= threshold=0.9940084517002106
node_1217: feature_name=GO:0042287 feature_id[20].value <= threshold=2.693045496940613
node_1218: feature_name=GO:0044446 feature_id[184].value <= threshold=113.0758171081543
node_1219: feature_name=GO:0098602 feature_id[702].value <= threshold=68.95328903198242
node_1220: feature_name=GO:0035726 feature_id[270].value <= threshold=1.7167096138000488
node_1221: feature_name=GO:1902564 feature_id[85].value <= threshold=2.8364468812942505
node_1222: feature_name=GO:0006979 feature_id[44].value > threshold=0.6869173645973206
node_1224: feature_name=GO:0044464 feature_id[56].value <= threshold=67.13260269165039

node_1225: feature_name=GO:0045629 feature_id[746].value <= threshold=2.3414241075515747
node_1226: feature_name=GO:0002903 feature_id[10].value <= threshold=2.715258240699768
node_1227: feature_name=GO:0043375 feature_id[717].value <= threshold=1.9525017738342285
node_1228: feature_name=GO:0032762 feature_id[630].value > threshold=0.29527929425239563
node_1232: feature_name=GO:0048569 feature_id[793].value > threshold=3.419509768486023
node_1246: feature_name=GO:0032649 feature_id[626].value <= threshold=0.7534940838813782
Class: positive genes

Rules_330 passed counts:2
node_0: feature_name=GO:0042113 feature_id[0].value <= threshold=13.408552169799805
node_1: feature_name=GO:0007568 feature_id[534].value > threshold=5.0313897132873535
node_913: feature_name=GO:0032763 feature_id[629].value > threshold=0.31753237545490265
node_1195: feature_name=GO:0071301 feature_id[442].value <= threshold=0.11534593254327774
node_1196: feature_name=GO:0046500 feature_id[171].value <= threshold=0.6314916908740997
node_1197: feature_name=GO:0030852 feature_id[22].value <= threshold=2.9768868684768677
node_1198: feature_name=hsa04672 feature_id[276].value <= threshold=8.06681227684021
node_1199: feature_name=GO:0001836 feature_id[450].value > threshold=0.09961023926734924
node_1201: feature_name=GO:0070230 feature_id[64].value <= threshold=3.3956379890441895
node_1202: feature_name=GO:0070102 feature_id[49].value <= threshold=4.805386543273926
node_1203: feature_name=GO:0045553 feature_id[736].value <= threshold=1.4970332980155945
node_1204: feature_name=GO:0051311 feature_id[58].value <= threshold=3.301889181137085
node_1205: feature_name=GO:0030291 feature_id[147].value <= threshold=8.156315326690674
node_1206: feature_name=GO:0045637 feature_id[241].value > threshold=0.01834342861548066
node_1208: feature_name=GO:0002890 feature_id[98].value <= threshold=2.767483353614807
node_1209: feature_name=GO:0046632 feature_id[248].value <= threshold=11.663254261016846
node_1210: feature_name=GO:0008588 feature_id[26].value <= threshold=1.391375720500946
node_1211: feature_name=GO:0010639 feature_id[564].value <= threshold=18.975126266479492
node_1212: feature_name=GO:0046685 feature_id[782].value <= threshold=5.153738260269165
node_1213: feature_name=GO:0038116 feature_id[668].value <= threshold=1.7859655618667603
node_1214: feature_name=GO:0046449 feature_id[763].value <= threshold=5.603115558624268
node_1215: feature_name=GO:0048539 feature_id[319].value <= threshold=0.5478232800960541
node_1216: feature_name=GO:0007089 feature_id[522].value <= threshold=0.9940084517002106
node_1217: feature_name=GO:0042287 feature_id[20].value <= threshold=2.693045496940613
node_1218: feature_name=GO:0044446 feature_id[184].value <= threshold=113.0758171081543
node_1219: feature_name=GO:0098602 feature_id[702].value <= threshold=68.95328903198242

node_1220: feature_name=GO:0035726 feature_id[270].value <= threshold=1.7167096138000488
node_1221: feature_name=GO:1902564 feature_id[85].value <= threshold=2.8364468812942505
node_1222: feature_name=GO:0006979 feature_id[44].value > threshold=0.6869173645973206
node_1224: feature_name=GO:0044464 feature_id[56].value <= threshold=67.13260269165039
node_1225: feature_name=GO:0045629 feature_id[746].value <= threshold=2.3414241075515747
node_1226: feature_name=GO:0002903 feature_id[10].value <= threshold=2.715258240699768
node_1227: feature_name=GO:0043375 feature_id[717].value <= threshold=1.9525017738342285
node_1228: feature_name=GO:0032762 feature_id[630].value <= threshold=0.29527929425239563
node_1229: feature_name=GO:0002562 feature_id[25].value > threshold=1.3898683190345764
Class: negative genes

Rules_331 passed counts:2
node_0: feature_name=GO:0042113 feature_id[0].value <= threshold=13.408552169799805
node_1: feature_name=GO:0007568 feature_id[534].value > threshold=5.0313897132873535
node_913: feature_name=GO:0032763 feature_id[629].value > threshold=0.31753237545490265
node_1195: feature_name=GO:0071301 feature_id[442].value <= threshold=0.11534593254327774
node_1196: feature_name=GO:0046500 feature_id[171].value <= threshold=0.6314916908740997
node_1197: feature_name=GO:0030852 feature_id[22].value <= threshold=2.9768868684768677
node_1198: feature_name=hsa04672 feature_id[276].value <= threshold=8.06681227684021
node_1199: feature_name=GO:0001836 feature_id[450].value > threshold=0.09961023926734924
node_1201: feature_name=GO:0070230 feature_id[64].value <= threshold=3.3956379890441895
node_1202: feature_name=GO:0070102 feature_id[49].value <= threshold=4.805386543273926
node_1203: feature_name=GO:0045553 feature_id[736].value <= threshold=1.4970332980155945
node_1204: feature_name=GO:0051311 feature_id[58].value <= threshold=3.301889181137085
node_1205: feature_name=GO:0030291 feature_id[147].value <= threshold=8.156315326690674
node_1206: feature_name=GO:0045637 feature_id[241].value <= threshold=0.018343428615480566
Class: positive genes

Rules_332 passed counts:2
node_0: feature_name=GO:0042113 feature_id[0].value <= threshold=13.408552169799805
node_1: feature_name=GO:0007568 feature_id[534].value > threshold=5.0313897132873535
node_913: feature_name=GO:0032763 feature_id[629].value <= threshold=0.31753237545490265
node_914: feature_name=GO:0097193 feature_id[167].value > threshold=28.171168327331543
node_1188: feature_name=GO:0002704 feature_id[470].value <= threshold=0.8429980874061584
node_1189: feature_name=GO:0009411 feature_id[67].value <= threshold=6.957151174545288

Class: negative genes

Rules_333 passed counts:2
node_0: feature_name=GO:0042113 feature_id[0].value <= threshold=13.408552169799805
node_1: feature_name=GO:0007568 feature_id[534].value > threshold=5.0313897132873535
node_913: feature_name=GO:0032763 feature_id[629].value <= threshold=0.31753237545490265
node_914: feature_name=GO:0097193 feature_id[167].value <= threshold=28.171168327331543
node_915: feature_name=GO:0002903 feature_id[10].value > threshold=1.0534588098526
node_1159: feature_name=GO:0070198 feature_id[665].value > threshold=0.16219981759786606
node_1165: feature_name=GO:0042287 feature_id[20].value <= threshold=0.915093183517456
node_1166: feature_name=GO:0046006 feature_id[364].value > threshold=0.12353959679603577
node_1168: feature_name=GO:0046634 feature_id[46].value <= threshold=4.166545033454895
node_1169: feature_name=GO:0044346 feature_id[724].value > threshold=0.9571778774261475
node_1173: feature_name=GO:0060374 feature_id[76].value > threshold=2.239573121070862
node_1175: feature_name=GO:0071887 feature_id[283].value > threshold=1.6318552792072296
Class: positive genes

Rules_334 passed counts:2
node_0: feature_name=GO:0042113 feature_id[0].value <= threshold=13.408552169799805
node_1: feature_name=GO:0007568 feature_id[534].value > threshold=5.0313897132873535
node_913: feature_name=GO:0032763 feature_id[629].value <= threshold=0.31753237545490265
node_914: feature_name=GO:0097193 feature_id[167].value <= threshold=28.171168327331543
node_915: feature_name=GO:0002903 feature_id[10].value > threshold=1.0534588098526
node_1159: feature_name=GO:0070198 feature_id[665].value <= threshold=0.16219981759786606
node_1160: feature_name=GO:0019692 feature_id[584].value <= threshold=1.4449326992034912
node_1161: feature_name=GO:0019814 feature_id[189].value > threshold=3.0963553190231323
Class: positive genes

Rules_335 passed counts:2
node_0: feature_name=GO:0042113 feature_id[0].value <= threshold=13.408552169799805
node_1: feature_name=GO:0007568 feature_id[534].value > threshold=5.0313897132873535
node_913: feature_name=GO:0032763 feature_id[629].value <= threshold=0.31753237545490265
node_914: feature_name=GO:0097193 feature_id[167].value <= threshold=28.171168327331543
node_915: feature_name=GO:0002903 feature_id[10].value <= threshold=1.0534588098526
node_916: feature_name=GO:1904029 feature_id[208].value <= threshold=23.852136611938477

node_917: feature_name=GO:0050897 feature_id[164].value > threshold=0.6870408356189728
node_1095: feature_name=GO:0072539 feature_id[68].value > threshold=2.5667877197265625
node_1153: feature_name=GO:0002708 feature_id[526].value > threshold=6.108316421508789
Class: negative genes

Rules_336 passed counts:2
node_0: feature_name=GO:0042113 feature_id[0].value <= threshold=13.408552169799805
node_1: feature_name=GO:0007568 feature_id[534].value > threshold=5.0313897132873535
node_913: feature_name=GO:0032763 feature_id[629].value <= threshold=0.31753237545490265
node_914: feature_name=GO:0097193 feature_id[167].value <= threshold=28.171168327331543
node_915: feature_name=GO:0002903 feature_id[10].value <= threshold=1.0534588098526
node_916: feature_name=GO:1904029 feature_id[208].value <= threshold=23.852136611938477
node_917: feature_name=GO:0050897 feature_id[164].value > threshold=0.6870408356189728
node_1095: feature_name=GO:0072539 feature_id[68].value <= threshold=2.5667877197265625
node_1096: feature_name=GO:0046500 feature_id[171].value <= threshold=3.195197820663452
node_1097: feature_name=GO:0045861 feature_id[761].value > threshold=0.22614753991365433
node_1099: feature_name=GO:0043372 feature_id[242].value <= threshold=4.892506122589111
node_1100: feature_name=GO:0042162 feature_id[355].value > threshold=11.339290618896484
node_1148: feature_name=GO:0002643 feature_id[4].value <= threshold=0.3193020150065422
Class: negative genes

Rules_337 passed counts:2
node_0: feature_name=GO:0042113 feature_id[0].value <= threshold=13.408552169799805
node_1: feature_name=GO:0007568 feature_id[534].value > threshold=5.0313897132873535
node_913: feature_name=GO:0032763 feature_id[629].value <= threshold=0.31753237545490265
node_914: feature_name=GO:0097193 feature_id[167].value <= threshold=28.171168327331543
node_915: feature_name=GO:0002903 feature_id[10].value <= threshold=1.0534588098526
node_916: feature_name=GO:1904029 feature_id[208].value <= threshold=23.852136611938477
node_917: feature_name=GO:0050897 feature_id[164].value > threshold=0.6870408356189728
node_1095: feature_name=GO:0072539 feature_id[68].value <= threshold=2.5667877197265625
node_1096: feature_name=GO:0046500 feature_id[171].value <= threshold=3.195197820663452
node_1097: feature_name=GO:0045861 feature_id[761].value > threshold=0.22614753991365433
node_1099: feature_name=GO:0043372 feature_id[242].value <= threshold=4.892506122589111
node_1100: feature_name=GO:0042162 feature_id[355].value <= threshold=11.339290618896484
node_1101: feature_name=hsa04660 feature_id[472].value <= threshold=24.27848720550537

node_1102: feature_name=GO:0007049 feature_id[259].value > threshold=209.23831176757812
Class: positive genes

Rules_338 passed counts:2
node_0: feature_name=GO:0042113 feature_id[0].value <= threshold=13.408552169799805
node_1: feature_name=GO:0007568 feature_id[534].value > threshold=5.0313897132873535
node_913: feature_name=GO:0032763 feature_id[629].value <= threshold=0.31753237545490265
node_914: feature_name=GO:0097193 feature_id[167].value <= threshold=28.171168327331543
node_915: feature_name=GO:0002903 feature_id[10].value <= threshold=1.0534588098526
node_916: feature_name=GO:1904029 feature_id[208].value <= threshold=23.852136611938477
node_917: feature_name=GO:0050897 feature_id[164].value > threshold=0.6870408356189728
node_1095: feature_name=GO:0072539 feature_id[68].value <= threshold=2.5667877197265625
node_1096: feature_name=GO:0046500 feature_id[171].value <= threshold=3.195197820663452
node_1097: feature_name=GO:0045861 feature_id[761].value > threshold=0.22614753991365433
node_1099: feature_name=GO:0043372 feature_id[242].value <= threshold=4.892506122589111
node_1100: feature_name=GO:0042162 feature_id[355].value <= threshold=11.339290618896484
node_1101: feature_name=hsa04660 feature_id[472].value <= threshold=24.27848720550537
node_1102: feature_name=GO:0007049 feature_id[259].value <= threshold=209.23831176757812
node_1103: feature_name=GO:0050897 feature_id[164].value > threshold=0.6919849812984467
node_1105: feature_name=GO:0045945 feature_id[267].value <= threshold=3.307852625846863
node_1106: feature_name=GO:0046641 feature_id[773].value > threshold=3.527579426765442
node_1134: feature_name=GO:0045736 feature_id[61].value <= threshold=0.6813804358243942
Class: positive genes

Rules_339 passed counts:2
node_0: feature_name=GO:0042113 feature_id[0].value <= threshold=13.408552169799805
node_1: feature_name=GO:0007568 feature_id[534].value > threshold=5.0313897132873535
node_913: feature_name=GO:0032763 feature_id[629].value <= threshold=0.31753237545490265
node_914: feature_name=GO:0097193 feature_id[167].value <= threshold=28.171168327331543
node_915: feature_name=GO:0002903 feature_id[10].value <= threshold=1.0534588098526
node_916: feature_name=GO:1904029 feature_id[208].value <= threshold=23.852136611938477
node_917: feature_name=GO:0050897 feature_id[164].value > threshold=0.6870408356189728
node_1095: feature_name=GO:0072539 feature_id[68].value <= threshold=2.5667877197265625
node_1096: feature_name=GO:0046500 feature_id[171].value <= threshold=3.195197820663452
node_1097: feature_name=GO:0045861 feature_id[761].value > threshold=0.22614753991365433

node_1099: feature_name=GO:0043372 feature_id[242].value <= threshold=4.892506122589111
node_1100: feature_name=GO:0042162 feature_id[355].value <= threshold=11.339290618896484
node_1101: feature_name=hsa04660 feature_id[472].value <= threshold=24.27848720550537
node_1102: feature_name=GO:0007049 feature_id[259].value <= threshold=209.23831176757812
node_1103: feature_name=GO:0050897 feature_id[164].value > threshold=0.6919849812984467
node_1105: feature_name=GO:0045945 feature_id[267].value <= threshold=3.307852625846863
node_1106: feature_name=GO:0046641 feature_id[773].value <= threshold=3.527579426765442
node_1107: feature_name=GO:0002262 feature_id[394].value > threshold=0.08302562311291695
node_1111: feature_name=GO:0043226 feature_id[138].value > threshold=2.4930146992119262e-05
node_1113: feature_name=GO:0045787 feature_id[446].value > threshold=0.07611752673983574
node_1115: feature_name=GO:0045840 feature_id[697].value > threshold=0.10580676794052124
node_1117: feature_name=GO:0006927 feature_id[225].value <= threshold=4.950021743774414
node_1118: feature_name=GO:0002643 feature_id[4].value <= threshold=3.364396333694458
node_1119: feature_name=GO:0016571 feature_id[725].value <= threshold=38.268903732299805
node_1120: feature_name=GO:0072593 feature_id[381].value <= threshold=19.997477531433105
node_1121: feature_name=GO:0070245 feature_id[326].value <= threshold=3.2926437854766846
node_1122: feature_name=GO:0008327 feature_id[77].value > threshold=1.4845957159996033
node_1124: feature_name=GO:0000783 feature_id[191].value > threshold=2.9206448197364807
Class: positive genes

Rules_340 passed counts:2
node_0: feature_name=GO:0042113 feature_id[0].value <= threshold=13.408552169799805
node_1: feature_name=GO:0007568 feature_id[534].value > threshold=5.0313897132873535
node_913: feature_name=GO:0032763 feature_id[629].value <= threshold=0.31753237545490265
node_914: feature_name=GO:0097193 feature_id[167].value <= threshold=28.171168327331543
node_915: feature_name=GO:0002903 feature_id[10].value <= threshold=1.0534588098526
node_916: feature_name=GO:1904029 feature_id[208].value <= threshold=23.852136611938477
node_917: feature_name=GO:0050897 feature_id[164].value > threshold=0.6870408356189728
node_1095: feature_name=GO:0072539 feature_id[68].value <= threshold=2.5667877197265625
node_1096: feature_name=GO:0046500 feature_id[171].value <= threshold=3.195197820663452
node_1097: feature_name=GO:0045861 feature_id[761].value > threshold=0.22614753991365433
node_1099: feature_name=GO:0043372 feature_id[242].value <= threshold=4.892506122589111
node_1100: feature_name=GO:0042162 feature_id[355].value <= threshold=11.339290618896484
node_1101: feature_name=hsa04660 feature_id[472].value <= threshold=24.27848720550537
node_1102: feature_name=GO:0007049 feature_id[259].value <= threshold=209.23831176757812

node_1103: feature_name=GO:0050897 feature_id[164].value > threshold=0.6919849812984467
node_1105: feature_name=GO:0045945 feature_id[267].value <= threshold=3.307852625846863
node_1106: feature_name=GO:0046641 feature_id[773].value <= threshold=3.527579426765442
node_1107: feature_name=GO:0002262 feature_id[394].value <= threshold=0.08302562311291695
node_1108: feature_name=GO:0097694 feature_id[176].value > threshold=0.491131991147995
Class: positive genes

Rules_341 passed counts:2
node_0: feature_name=GO:0042113 feature_id[0].value <= threshold=13.408552169799805
node_1: feature_name=GO:0007568 feature_id[534].value > threshold=5.0313897132873535
node_913: feature_name=GO:0032763 feature_id[629].value <= threshold=0.31753237545490265
node_914: feature_name=GO:0097193 feature_id[167].value <= threshold=28.171168327331543
node_915: feature_name=GO:0002903 feature_id[10].value <= threshold=1.0534588098526
node_916: feature_name=GO:1904029 feature_id[208].value <= threshold=23.852136611938477
node_917: feature_name=GO:0050897 feature_id[164].value > threshold=0.6870408356189728
node_1095: feature_name=GO:0072539 feature_id[68].value <= threshold=2.5667877197265625
node_1096: feature_name=GO:0046500 feature_id[171].value <= threshold=3.195197820663452
node_1097: feature_name=GO:0045861 feature_id[761].value > threshold=0.22614753991365433
node_1099: feature_name=GO:0043372 feature_id[242].value <= threshold=4.892506122589111
node_1100: feature_name=GO:0042162 feature_id[355].value <= threshold=11.339290618896484
node_1101: feature_name=hsa04660 feature_id[472].value <= threshold=24.27848720550537
node_1102: feature_name=GO:0007049 feature_id[259].value <= threshold=209.23831176757812
node_1103: feature_name=GO:0050897 feature_id[164].value <= threshold=0.6919849812984467
Class: positive genes

Rules_342 passed counts:2
node_0: feature_name=GO:0042113 feature_id[0].value <= threshold=13.408552169799805
node_1: feature_name=GO:0007568 feature_id[534].value > threshold=5.0313897132873535
node_913: feature_name=GO:0032763 feature_id[629].value <= threshold=0.31753237545490265
node_914: feature_name=GO:0097193 feature_id[167].value <= threshold=28.171168327331543
node_915: feature_name=GO:0002903 feature_id[10].value <= threshold=1.0534588098526
node_916: feature_name=GO:1904029 feature_id[208].value <= threshold=23.852136611938477
node_917: feature_name=GO:0050897 feature_id[164].value <= threshold=0.6870408356189728
node_918: feature_name=GO:0006139 feature_id[474].value > threshold=1.3052097624921544e-07
node_920: feature_name=GO:0002821 feature_id[588].value > threshold=13.81072187423706

Class: positive genes

Rules_343 passed counts:2
node_0: feature_name=GO:0042113 feature_id[0].value <= threshold=13.408552169799805
node_1: feature_name=GO:0007568 feature_id[534].value > threshold=5.0313897132873535
node_913: feature_name=GO:0032763 feature_id[629].value <= threshold=0.31753237545490265
node_914: feature_name=GO:0097193 feature_id[167].value <= threshold=28.171168327331543
node_915: feature_name=GO:0002903 feature_id[10].value <= threshold=1.0534588098526
node_916: feature_name=GO:1904029 feature_id[208].value <= threshold=23.852136611938477
node_917: feature_name=GO:0050897 feature_id[164].value <= threshold=0.6870408356189728
node_918: feature_name=GO:0006139 feature_id[474].value > threshold=1.3052097624921544e-07
node_920: feature_name=GO:0002821 feature_id[588].value <= threshold=13.81072187423706
node_921: feature_name=GO:0006298 feature_id[621].value > threshold=24.078600883483887
Class: positive genes

Rules_344 passed counts:2
node_0: feature_name=GO:0042113 feature_id[0].value <= threshold=13.408552169799805
node_1: feature_name=GO:0007568 feature_id[534].value > threshold=5.0313897132873535
node_913: feature_name=GO:0032763 feature_id[629].value <= threshold=0.31753237545490265
node_914: feature_name=GO:0097193 feature_id[167].value <= threshold=28.171168327331543
node_915: feature_name=GO:0002903 feature_id[10].value <= threshold=1.0534588098526
node_916: feature_name=GO:1904029 feature_id[208].value <= threshold=23.852136611938477
node_917: feature_name=GO:0050897 feature_id[164].value <= threshold=0.6870408356189728
node_918: feature_name=GO:0006139 feature_id[474].value > threshold=1.3052097624921544e-07
node_920: feature_name=GO:0002821 feature_id[588].value <= threshold=13.81072187423706
node_921: feature_name=GO:0006298 feature_id[621].value <= threshold=24.078600883483887
node_922: feature_name=GO:0003908 feature_id[11].value > threshold=1.7776933312416077
node_1090: feature_name=GO:0046685 feature_id[782].value <= threshold=0.9642224758863449
Class: negative genes

Rules_345 passed counts:2
node_0: feature_name=GO:0042113 feature_id[0].value <= threshold=13.408552169799805
node_1: feature_name=GO:0007568 feature_id[534].value > threshold=5.0313897132873535
node_913: feature_name=GO:0032763 feature_id[629].value <= threshold=0.31753237545490265
node_914: feature_name=GO:0097193 feature_id[167].value <= threshold=28.171168327331543

node_915: feature_name=GO:0002903 feature_id[10].value <= threshold=1.0534588098526
node_916: feature_name=GO:1904029 feature_id[208].value <= threshold=23.852136611938477
node_917: feature_name=GO:0050897 feature_id[164].value <= threshold=0.6870408356189728
node_918: feature_name=GO:0006139 feature_id[474].value > threshold=1.3052097624921544e-07
node_920: feature_name=GO:0002821 feature_id[588].value <= threshold=13.81072187423706
node_921: feature_name=GO:0006298 feature_id[621].value <= threshold=24.078600883483887
node_922: feature_name=GO:0003908 feature_id[11].value <= threshold=1.7776933312416077
node_923: feature_name=GO:0030098 feature_id[273].value > threshold=0.0079949083738029
node_927: feature_name=GO:0006808 feature_id[513].value > threshold=4.34592080116272
node_1087: feature_name=GO:0001777 feature_id[380].value <= threshold=0.9556370079517365
Class: positive genes

Rules_346 passed counts:2
node_0: feature_name=GO:0042113 feature_id[0].value <= threshold=13.408552169799805
node_1: feature_name=GO:0007568 feature_id[534].value > threshold=5.0313897132873535
node_913: feature_name=GO:0032763 feature_id[629].value <= threshold=0.31753237545490265
node_914: feature_name=GO:0097193 feature_id[167].value <= threshold=28.171168327331543
node_915: feature_name=GO:0002903 feature_id[10].value <= threshold=1.0534588098526
node_916: feature_name=GO:1904029 feature_id[208].value <= threshold=23.852136611938477
node_917: feature_name=GO:0050897 feature_id[164].value <= threshold=0.6870408356189728
node_918: feature_name=GO:0006139 feature_id[474].value > threshold=1.3052097624921544e-07
node_920: feature_name=GO:0002821 feature_id[588].value <= threshold=13.81072187423706
node_921: feature_name=GO:0006298 feature_id[621].value <= threshold=24.078600883483887
node_922: feature_name=GO:0003908 feature_id[11].value <= threshold=1.7776933312416077
node_923: feature_name=GO:0030098 feature_id[273].value > threshold=0.0079949083738029
node_927: feature_name=GO:0006808 feature_id[513].value <= threshold=4.34592080116272
node_928: feature_name=GO:0071887 feature_id[283].value > threshold=10.353787899017334
node_1084: feature_name=GO:0043011 feature_id[150].value > threshold=1.5169694423675537
Class: positive genes

Rules_347 passed counts:2
node_0: feature_name=GO:0042113 feature_id[0].value <= threshold=13.408552169799805
node_1: feature_name=GO:0007568 feature_id[534].value > threshold=5.0313897132873535
node_913: feature_name=GO:0032763 feature_id[629].value <= threshold=0.31753237545490265
node_914: feature_name=GO:0097193 feature_id[167].value <= threshold=28.171168327331543

node_915: feature_name=GO:0002903 feature_id[10].value <= threshold=1.0534588098526
node_916: feature_name=GO:1904029 feature_id[208].value <= threshold=23.852136611938477
node_917: feature_name=GO:0050897 feature_id[164].value <= threshold=0.6870408356189728
node_918: feature_name=GO:0006139 feature_id[474].value > threshold=1.3052097624921544e-07
node_920: feature_name=GO:0002821 feature_id[588].value <= threshold=13.81072187423706
node_921: feature_name=GO:0006298 feature_id[621].value <= threshold=24.078600883483887
node_922: feature_name=GO:0003908 feature_id[11].value <= threshold=1.7776933312416077
node_923: feature_name=GO:0030098 feature_id[273].value > threshold=0.0079949083738029
node_927: feature_name=GO:0006808 feature_id[513].value <= threshold=4.34592080116272
node_928: feature_name=GO:0071887 feature_id[283].value <= threshold=10.353787899017334
node_929: feature_name=GO:0038001 feature_id[28].value > threshold=3.9469382762908936
node_1081: feature_name=GO:0043465 feature_id[710].value > threshold=1.0033313930034637
Class: positive genes

Rules_348 passed counts:2
node_0: feature_name=GO:0042113 feature_id[0].value <= threshold=13.408552169799805
node_1: feature_name=GO:0007568 feature_id[534].value > threshold=5.0313897132873535
node_913: feature_name=GO:0032763 feature_id[629].value <= threshold=0.31753237545490265
node_914: feature_name=GO:0097193 feature_id[167].value <= threshold=28.171168327331543
node_915: feature_name=GO:0002903 feature_id[10].value <= threshold=1.0534588098526
node_916: feature_name=GO:1904029 feature_id[208].value <= threshold=23.852136611938477
node_917: feature_name=GO:0050897 feature_id[164].value <= threshold=0.6870408356189728
node_918: feature_name=GO:0006139 feature_id[474].value > threshold=1.3052097624921544e-07
node_920: feature_name=GO:0002821 feature_id[588].value <= threshold=13.81072187423706
node_921: feature_name=GO:0006298 feature_id[621].value <= threshold=24.078600883483887
node_922: feature_name=GO:0003908 feature_id[11].value <= threshold=1.7776933312416077
node_923: feature_name=GO:0030098 feature_id[273].value > threshold=0.0079949083738029
node_927: feature_name=GO:0006808 feature_id[513].value <= threshold=4.34592080116272
node_928: feature_name=GO:0071887 feature_id[283].value <= threshold=10.353787899017334
node_929: feature_name=GO:0038001 feature_id[28].value <= threshold=3.9469382762908936
node_930: feature_name=GO:0042287 feature_id[20].value > threshold=3.6209195852279663
node_1078: feature_name=hsa05202 feature_id[50].value > threshold=0.7917962670326233
Class: positive genes

Rules_349 passed counts:2

node_0: feature_name=GO:0042113 feature_id[0].value <= threshold=13.408552169799805
node_1: feature_name=GO:0007568 feature_id[534].value > threshold=5.0313897132873535
node_913: feature_name=GO:0032763 feature_id[629].value <= threshold=0.31753237545490265
node_914: feature_name=GO:0097193 feature_id[167].value <= threshold=28.171168327331543
node_915: feature_name=GO:0002903 feature_id[10].value <= threshold=1.0534588098526
node_916: feature_name=GO:1904029 feature_id[208].value <= threshold=23.852136611938477
node_917: feature_name=GO:0050897 feature_id[164].value <= threshold=0.6870408356189728
node_918: feature_name=GO:0006139 feature_id[474].value > threshold=1.3052097624921544e-07
node_920: feature_name=GO:0002821 feature_id[588].value <= threshold=13.81072187423706
node_921: feature_name=GO:0006298 feature_id[621].value <= threshold=24.078600883483887
node_922: feature_name=GO:0003908 feature_id[11].value <= threshold=1.7776933312416077
node_923: feature_name=GO:0030098 feature_id[273].value > threshold=0.0079949083738029
node_927: feature_name=GO:0006808 feature_id[513].value <= threshold=4.34592080116272
node_928: feature_name=GO:0071887 feature_id[283].value <= threshold=10.353787899017334
node_929: feature_name=GO:0038001 feature_id[28].value <= threshold=3.9469382762908936
node_930: feature_name=GO:0042287 feature_id[20].value <= threshold=3.6209195852279663
node_931: feature_name=GO:0003968 feature_id[47].value <= threshold=2.004227638244629
node_932: feature_name=GO:0002698 feature_id[395].value <= threshold=18.51447582244873
node_933: feature_name=GO:0044710 feature_id[719].value <= threshold=179.7340316772461
node_934: feature_name=GO:0007568 feature_id[534].value > threshold=5.035318374633789
node_936: feature_name=GO:0006216 feature_id[504].value <= threshold=2.2605666518211365
node_937: feature_name=GO:0048569 feature_id[793].value <= threshold=5.340231895446777
node_938: feature_name=GO:0001777 feature_id[380].value <= threshold=4.100832939147949
node_939: feature_name=GO:0007600 feature_id[122].value <= threshold=171.13383102416992
node_940: feature_name=GO:0001779 feature_id[378].value <= threshold=6.234851121902466
node_941: feature_name=GO:0030291 feature_id[147].value <= threshold=10.8051118850708
node_942: feature_name=GO:0048534 feature_id[790].value > threshold=0.017984486185014248
node_944: feature_name=GO:0070245 feature_id[326].value > threshold=3.1282339096069336
node_1066: feature_name=GO:0070227 feature_id[57].value <= threshold=2.323003053665161
Class: positive genes

Rules_350 passed counts:2
node_0: feature_name=GO:0042113 feature_id[0].value <= threshold=13.408552169799805
node_1: feature_name=GO:0007568 feature_id[534].value > threshold=5.0313897132873535
node_913: feature_name=GO:0032763 feature_id[629].value <= threshold=0.31753237545490265

node_914: feature_name=GO:0097193 feature_id[167].value <= threshold=28.171168327331543
node_915: feature_name=GO:0002903 feature_id[10].value <= threshold=1.0534588098526
node_916: feature_name=GO:1904029 feature_id[208].value <= threshold=23.852136611938477
node_917: feature_name=GO:0050897 feature_id[164].value <= threshold=0.6870408356189728
node_918: feature_name=GO:0006139 feature_id[474].value > threshold=1.3052097624921544e-07
node_920: feature_name=GO:0002821 feature_id[588].value <= threshold=13.81072187423706
node_921: feature_name=GO:0006298 feature_id[621].value <= threshold=24.078600883483887
node_922: feature_name=GO:0003908 feature_id[11].value <= threshold=1.7776933312416077
node_923: feature_name=GO:0030098 feature_id[273].value > threshold=0.0079949083738029
node_927: feature_name=GO:0006808 feature_id[513].value <= threshold=4.34592080116272
node_928: feature_name=GO:0071887 feature_id[283].value <= threshold=10.353787899017334
node_929: feature_name=GO:0038001 feature_id[28].value <= threshold=3.9469382762908936
node_930: feature_name=GO:0042287 feature_id[20].value <= threshold=3.6209195852279663
node_931: feature_name=GO:0003968 feature_id[47].value <= threshold=2.004227638244629
node_932: feature_name=GO:0002698 feature_id[395].value <= threshold=18.51447582244873
node_933: feature_name=GO:0044710 feature_id[719].value <= threshold=179.7340316772461
node_934: feature_name=GO:0007568 feature_id[534].value > threshold=5.035318374633789
node_936: feature_name=GO:0006216 feature_id[504].value <= threshold=2.2605666518211365
node_937: feature_name=GO:0048569 feature_id[793].value <= threshold=5.340231895446777
node_938: feature_name=GO:0001777 feature_id[380].value <= threshold=4.100832939147949
node_939: feature_name=GO:0007600 feature_id[122].value <= threshold=171.13383102416992
node_940: feature_name=GO:0001779 feature_id[378].value <= threshold=6.234851121902466
node_941: feature_name=GO:0030291 feature_id[147].value <= threshold=10.8051118850708
node_942: feature_name=GO:0048534 feature_id[790].value > threshold=0.017984486185014248
node_944: feature_name=GO:0070245 feature_id[326].value <= threshold=3.1282339096069336
node_945: feature_name=GO:0009086 feature_id[547].value > threshold=0.2896959036588669
node_1017: feature_name=GO:0000302 feature_id[375].value > threshold=0.305715873837471
node_1019: feature_name=GO:0070141 feature_id[37].value > threshold=4.857294321060181
node_1063: feature_name=GO:0002292 feature_id[352].value > threshold=0.9737851917743683
Class: negative genes

Rules_351 passed counts:2
node_0: feature_name=GO:0042113 feature_id[0].value <= threshold=13.408552169799805
node_1: feature_name=GO:0007568 feature_id[534].value > threshold=5.0313897132873535
node_913: feature_name=GO:0032763 feature_id[629].value <= threshold=0.31753237545490265

node_914: feature_name=GO:0097193 feature_id[167].value <= threshold=28.171168327331543
node_915: feature_name=GO:0002903 feature_id[10].value <= threshold=1.0534588098526
node_916: feature_name=GO:1904029 feature_id[208].value <= threshold=23.852136611938477
node_917: feature_name=GO:0050897 feature_id[164].value <= threshold=0.6870408356189728
node_918: feature_name=GO:0006139 feature_id[474].value > threshold=1.3052097624921544e-07
node_920: feature_name=GO:0002821 feature_id[588].value <= threshold=13.81072187423706
node_921: feature_name=GO:0006298 feature_id[621].value <= threshold=24.078600883483887
node_922: feature_name=GO:0003908 feature_id[11].value <= threshold=1.7776933312416077
node_923: feature_name=GO:0030098 feature_id[273].value > threshold=0.0079949083738029
node_927: feature_name=GO:0006808 feature_id[513].value <= threshold=4.34592080116272
node_928: feature_name=GO:0071887 feature_id[283].value <= threshold=10.353787899017334
node_929: feature_name=GO:0038001 feature_id[28].value <= threshold=3.9469382762908936
node_930: feature_name=GO:0042287 feature_id[20].value <= threshold=3.6209195852279663
node_931: feature_name=GO:0003968 feature_id[47].value <= threshold=2.004227638244629
node_932: feature_name=GO:0002698 feature_id[395].value <= threshold=18.51447582244873
node_933: feature_name=GO:0044710 feature_id[719].value <= threshold=179.7340316772461
node_934: feature_name=GO:0007568 feature_id[534].value > threshold=5.035318374633789
node_936: feature_name=GO:0006216 feature_id[504].value <= threshold=2.2605666518211365
node_937: feature_name=GO:0048569 feature_id[793].value <= threshold=5.340231895446777
node_938: feature_name=GO:0001777 feature_id[380].value <= threshold=4.100832939147949
node_939: feature_name=GO:0007600 feature_id[122].value <= threshold=171.13383102416992
node_940: feature_name=GO:0001779 feature_id[378].value <= threshold=6.234851121902466
node_941: feature_name=GO:0030291 feature_id[147].value <= threshold=10.8051118850708
node_942: feature_name=GO:0048534 feature_id[790].value > threshold=0.017984486185014248
node_944: feature_name=GO:0070245 feature_id[326].value <= threshold=3.1282339096069336
node_945: feature_name=GO:0009086 feature_id[547].value > threshold=0.2896959036588669
node_1017: feature_name=GO:0000302 feature_id[375].value > threshold=0.305715873837471
node_1019: feature_name=GO:0070141 feature_id[37].value <= threshold=4.857294321060181
node_1020: feature_name=GO:0009086 feature_id[547].value > threshold=0.2945319563150406
node_1024: feature_name=GO:0010332 feature_id[557].value <= threshold=15.092710971832275
node_1025: feature_name=GO:0002524 feature_id[72].value <= threshold=2.531021237373352
node_1026: feature_name=GO:0016032 feature_id[571].value <= threshold=60.173330307006836
node_1027: feature_name=GO:0002832 feature_id[491].value <= threshold=2.0699684023857117
node_1028: feature_name=GO:0008588 feature_id[26].value <= threshold=2.150238513946533
node_1029: feature_name=GO:0009615 feature_id[265].value > threshold=0.02372477948665619

node_1031: feature_name=GO:0002763 feature_id[271].value <= threshold=6.462319850921631
node_1032: feature_name=GO:0045840 feature_id[697].value <= threshold=10.30175256729126
node_1033: feature_name=GO:0051402 feature_id[568].value > threshold=1.2523645758628845
node_1041: feature_name=GO:0050852 feature_id[154].value > threshold=0.0008433434413745999
node_1043: feature_name=GO:0031667 feature_id[648].value > threshold=1.5418170094490051
node_1047: feature_name=GO:0009987 feature_id[554].value > threshold=1.8002276420593262
node_1051: feature_name=GO:0008588 feature_id[26].value > threshold=0.9623689949512482
node_1053: feature_name=GO:0005035 feature_id[133].value <= threshold=0.4232639968395233
Class: positive genes

Rules_352 passed counts:2
node_0: feature_name=GO:0042113 feature_id[0].value <= threshold=13.408552169799805
node_1: feature_name=GO:0007568 feature_id[534].value > threshold=5.0313897132873535
node_913: feature_name=GO:0032763 feature_id[629].value <= threshold=0.31753237545490265
node_914: feature_name=GO:0097193 feature_id[167].value <= threshold=28.171168327331543
node_915: feature_name=GO:0002903 feature_id[10].value <= threshold=1.0534588098526
node_916: feature_name=GO:1904029 feature_id[208].value <= threshold=23.852136611938477
node_917: feature_name=GO:0050897 feature_id[164].value <= threshold=0.6870408356189728
node_918: feature_name=GO:0006139 feature_id[474].value > threshold=1.3052097624921544e-07
node_920: feature_name=GO:0002821 feature_id[588].value <= threshold=13.81072187423706
node_921: feature_name=GO:0006298 feature_id[621].value <= threshold=24.078600883483887
node_922: feature_name=GO:0003908 feature_id[11].value <= threshold=1.7776933312416077
node_923: feature_name=GO:0030098 feature_id[273].value > threshold=0.0079949083738029
node_927: feature_name=GO:0006808 feature_id[513].value <= threshold=4.34592080116272
node_928: feature_name=GO:0071887 feature_id[283].value <= threshold=10.353787899017334
node_929: feature_name=GO:0038001 feature_id[28].value <= threshold=3.9469382762908936
node_930: feature_name=GO:0042287 feature_id[20].value <= threshold=3.6209195852279663
node_931: feature_name=GO:0003968 feature_id[47].value <= threshold=2.004227638244629
node_932: feature_name=GO:0002698 feature_id[395].value <= threshold=18.51447582244873
node_933: feature_name=GO:0044710 feature_id[719].value <= threshold=179.7340316772461
node_934: feature_name=GO:0007568 feature_id[534].value > threshold=5.035318374633789
node_936: feature_name=GO:0006216 feature_id[504].value <= threshold=2.2605666518211365
node_937: feature_name=GO:0048569 feature_id[793].value <= threshold=5.340231895446777
node_938: feature_name=GO:0001777 feature_id[380].value <= threshold=4.100832939147949
node_939: feature_name=GO:0007600 feature_id[122].value <= threshold=171.13383102416992

node_940: feature_name=GO:0001779 feature_id[378].value <= threshold=6.234851121902466
node_941: feature_name=GO:0030291 feature_id[147].value <= threshold=10.8051118850708
node_942: feature_name=GO:0048534 feature_id[790].value > threshold=0.017984486185014248
node_944: feature_name=GO:0070245 feature_id[326].value <= threshold=3.1282339096069336
node_945: feature_name=GO:0009086 feature_id[547].value > threshold=0.2896959036588669
node_1017: feature_name=GO:0000302 feature_id[375].value > threshold=0.305715873837471
node_1019: feature_name=GO:0070141 feature_id[37].value <= threshold=4.857294321060181
node_1020: feature_name=GO:0009086 feature_id[547].value > threshold=0.2945319563150406
node_1024: feature_name=GO:0010332 feature_id[557].value <= threshold=15.092710971832275
node_1025: feature_name=GO:0002524 feature_id[72].value <= threshold=2.531021237373352
node_1026: feature_name=GO:0016032 feature_id[571].value <= threshold=60.173330307006836
node_1027: feature_name=GO:0002832 feature_id[491].value <= threshold=2.0699684023857117
node_1028: feature_name=GO:0008588 feature_id[26].value <= threshold=2.150238513946533
node_1029: feature_name=GO:0009615 feature_id[265].value > threshold=0.02372477948665619
node_1031: feature_name=GO:0002763 feature_id[271].value <= threshold=6.462319850921631
node_1032: feature_name=GO:0045840 feature_id[697].value <= threshold=10.30175256729126
node_1033: feature_name=GO:0051402 feature_id[568].value > threshold=1.2523645758628845
node_1041: feature_name=GO:0050852 feature_id[154].value > threshold=0.0008433434413745999
node_1043: feature_name=GO:0031667 feature_id[648].value > threshold=1.5418170094490051
node_1047: feature_name=GO:0009987 feature_id[554].value <= threshold=1.8002276420593262
node_1048: feature_name=GO:0038061 feature_id[671].value <= threshold=1.3254979476332664
Class: negative genes

Rules_353 passed counts:2
node_0: feature_name=GO:0042113 feature_id[0].value <= threshold=13.408552169799805
node_1: feature_name=GO:0007568 feature_id[534].value > threshold=5.0313897132873535
node_913: feature_name=GO:0032763 feature_id[629].value <= threshold=0.31753237545490265
node_914: feature_name=GO:0097193 feature_id[167].value <= threshold=28.171168327331543
node_915: feature_name=GO:0002903 feature_id[10].value <= threshold=1.0534588098526
node_916: feature_name=GO:1904029 feature_id[208].value <= threshold=23.852136611938477
node_917: feature_name=GO:0050897 feature_id[164].value <= threshold=0.6870408356189728
node_918: feature_name=GO:0006139 feature_id[474].value > threshold=1.3052097624921544e-07
node_920: feature_name=GO:0002821 feature_id[588].value <= threshold=13.81072187423706
node_921: feature_name=GO:0006298 feature_id[621].value <= threshold=24.078600883483887
node_922: feature_name=GO:0003908 feature_id[11].value <= threshold=1.7776933312416077

node_923: feature_name=GO:0030098 feature_id[273].value > threshold=0.0079949083738029
node_927: feature_name=GO:0006808 feature_id[513].value <= threshold=4.34592080116272
node_928: feature_name=GO:0071887 feature_id[283].value <= threshold=10.353787899017334
node_929: feature_name=GO:0038001 feature_id[28].value <= threshold=3.9469382762908936
node_930: feature_name=GO:0042287 feature_id[20].value <= threshold=3.6209195852279663
node_931: feature_name=GO:0003968 feature_id[47].value <= threshold=2.004227638244629
node_932: feature_name=GO:0002698 feature_id[395].value <= threshold=18.51447582244873
node_933: feature_name=GO:0044710 feature_id[719].value <= threshold=179.7340316772461
node_934: feature_name=GO:0007568 feature_id[534].value > threshold=5.035318374633789
node_936: feature_name=GO:0006216 feature_id[504].value <= threshold=2.2605666518211365
node_937: feature_name=GO:0048569 feature_id[793].value <= threshold=5.340231895446777
node_938: feature_name=GO:0001777 feature_id[380].value <= threshold=4.100832939147949
node_939: feature_name=GO:0007600 feature_id[122].value <= threshold=171.13383102416992
node_940: feature_name=GO:0001779 feature_id[378].value <= threshold=6.234851121902466
node_941: feature_name=GO:0030291 feature_id[147].value <= threshold=10.8051118850708
node_942: feature_name=GO:0048534 feature_id[790].value > threshold=0.017984486185014248
node_944: feature_name=GO:0070245 feature_id[326].value <= threshold=3.1282339096069336
node_945: feature_name=GO:0009086 feature_id[547].value > threshold=0.2896959036588669
node_1017: feature_name=GO:0000302 feature_id[375].value > threshold=0.305715873837471
node_1019: feature_name=GO:0070141 feature_id[37].value <= threshold=4.857294321060181
node_1020: feature_name=GO:0009086 feature_id[547].value > threshold=0.2945319563150406
node_1024: feature_name=GO:0010332 feature_id[557].value <= threshold=15.092710971832275
node_1025: feature_name=GO:0002524 feature_id[72].value <= threshold=2.531021237373352
node_1026: feature_name=GO:0016032 feature_id[571].value <= threshold=60.173330307006836
node_1027: feature_name=GO:0002832 feature_id[491].value <= threshold=2.0699684023857117
node_1028: feature_name=GO:0008588 feature_id[26].value <= threshold=2.150238513946533
node_1029: feature_name=GO:0009615 feature_id[265].value > threshold=0.02372477948665619
node_1031: feature_name=GO:0002763 feature_id[271].value <= threshold=6.462319850921631
node_1032: feature_name=GO:0045840 feature_id[697].value <= threshold=10.30175256729126
node_1033: feature_name=GO:0051402 feature_id[568].value <= threshold=1.2523645758628845
node_1034: feature_name=GO:0008625 feature_id[99].value > threshold=1.086933195590973
node_1038: feature_name=GO:0016064 feature_id[570].value > threshold=1.333651602268219
Class: negative genes

Rules_354 passed counts:2

node_0: feature_name=GO:0042113	feature_id[0].value <= threshold=13.408552169799805
node_1: feature_name=GO:0007568	feature_id[534].value > threshold=5.0313897132873535
node_913: feature_name=GO:0032763	feature_id[629].value <= threshold=0.31753237545490265
node_914: feature_name=GO:0097193	feature_id[167].value <= threshold=28.171168327331543
node_915: feature_name=GO:0002903	feature_id[10].value <= threshold=1.0534588098526
node_916: feature_name=GO:1904029	feature_id[208].value <= threshold=23.852136611938477
node_917: feature_name=GO:0050897	feature_id[164].value <= threshold=0.6870408356189728
node_918: feature_name=GO:0006139	feature_id[474].value > threshold=1.3052097624921544e-07
node_920: feature_name=GO:0002821	feature_id[588].value <= threshold=13.81072187423706
node_921: feature_name=GO:0006298	feature_id[621].value <= threshold=24.078600883483887
node_922: feature_name=GO:0003908	feature_id[11].value <= threshold=1.7776933312416077
node_923: feature_name=GO:0030098	feature_id[273].value > threshold=0.0079949083738029
node_927: feature_name=GO:0006808	feature_id[513].value <= threshold=4.34592080116272
node_928: feature_name=GO:0071887	feature_id[283].value <= threshold=10.353787899017334
node_929: feature_name=GO:0038001	feature_id[28].value <= threshold=3.9469382762908936
node_930: feature_name=GO:0042287	feature_id[20].value <= threshold=3.6209195852279663
node_931: feature_name=GO:0003968	feature_id[47].value <= threshold=2.004227638244629
node_932: feature_name=GO:0002698	feature_id[395].value <= threshold=18.51447582244873
node_933: feature_name=GO:0044710	feature_id[719].value <= threshold=179.7340316772461
node_934: feature_name=GO:0007568	feature_id[534].value > threshold=5.035318374633789
node_936: feature_name=GO:0006216	feature_id[504].value <= threshold=2.2605666518211365
node_937: feature_name=GO:0048569	feature_id[793].value <= threshold=5.340231895446777
node_938: feature_name=GO:0001777	feature_id[380].value <= threshold=4.100832939147949
node_939: feature_name=GO:0007600	feature_id[122].value <= threshold=171.13383102416992
node_940: feature_name=GO:0001779	feature_id[378].value <= threshold=6.234851121902466
node_941: feature_name=GO:0030291	feature_id[147].value <= threshold=10.8051118850708
node_942: feature_name=GO:0048534	feature_id[790].value > threshold=0.017984486185014248
node_944: feature_name=GO:0070245	feature_id[326].value <= threshold=3.1282339096069336
node_945: feature_name=GO:0009086	feature_id[547].value > threshold=0.2896959036588669
node_1017: feature_name=GO:0000302	feature_id[375].value > threshold=0.305715873837471
node_1019: feature_name=GO:0070141	feature_id[37].value <= threshold=4.857294321060181
node_1020: feature_name=GO:0009086	feature_id[547].value <= threshold=0.2945319563150406
node_1021: feature_name=GO:0003684	feature_id[653].value > threshold=0.8139447569847107

Class: positive genes

Rules_355 passed counts:2
node_0: feature_name=GO:0042113 feature_id[0].value <= threshold=13.408552169799805
node_1: feature_name=GO:0007568 feature_id[534].value > threshold=5.0313897132873535
node_913: feature_name=GO:0032763 feature_id[629].value <= threshold=0.31753237545490265
node_914: feature_name=GO:0097193 feature_id[167].value <= threshold=28.171168327331543
node_915: feature_name=GO:0002903 feature_id[10].value <= threshold=1.0534588098526
node_916: feature_name=GO:1904029 feature_id[208].value <= threshold=23.852136611938477
node_917: feature_name=GO:0050897 feature_id[164].value <= threshold=0.6870408356189728
node_918: feature_name=GO:0006139 feature_id[474].value > threshold=1.3052097624921544e-07
node_920: feature_name=GO:0002821 feature_id[588].value <= threshold=13.81072187423706
node_921: feature_name=GO:0006298 feature_id[621].value <= threshold=24.078600883483887
node_922: feature_name=GO:0003908 feature_id[11].value <= threshold=1.7776933312416077
node_923: feature_name=GO:0030098 feature_id[273].value > threshold=0.0079949083738029
node_927: feature_name=GO:0006808 feature_id[513].value <= threshold=4.34592080116272
node_928: feature_name=GO:0071887 feature_id[283].value <= threshold=10.353787899017334
node_929: feature_name=GO:0038001 feature_id[28].value <= threshold=3.9469382762908936
node_930: feature_name=GO:0042287 feature_id[20].value <= threshold=3.6209195852279663
node_931: feature_name=GO:0003968 feature_id[47].value <= threshold=2.004227638244629
node_932: feature_name=GO:0002698 feature_id[395].value <= threshold=18.51447582244873
node_933: feature_name=GO:0044710 feature_id[719].value <= threshold=179.7340316772461
node_934: feature_name=GO:0007568 feature_id[534].value > threshold=5.035318374633789
node_936: feature_name=GO:0006216 feature_id[504].value <= threshold=2.2605666518211365
node_937: feature_name=GO:0048569 feature_id[793].value <= threshold=5.340231895446777
node_938: feature_name=GO:0001777 feature_id[380].value <= threshold=4.100832939147949
node_939: feature_name=GO:0007600 feature_id[122].value <= threshold=171.13383102416992
node_940: feature_name=GO:0001779 feature_id[378].value <= threshold=6.234851121902466
node_941: feature_name=GO:0030291 feature_id[147].value <= threshold=10.8051118850708
node_942: feature_name=GO:0048534 feature_id[790].value > threshold=0.017984486185014248
node_944: feature_name=GO:0070245 feature_id[326].value <= threshold=3.1282339096069336
node_945: feature_name=GO:0009086 feature_id[547].value > threshold=0.2896959036588669
node_1017: feature_name=GO:0000302 feature_id[375].value <= threshold=0.305715873837471
Class: positive genes

Rules_356 passed counts:2
node_0: feature_name=GO:0042113 feature_id[0].value <= threshold=13.408552169799805

node_1: feature_name=GO:0007568 feature_id[534].value > threshold=5.0313897132873535
node_913: feature_name=GO:0032763 feature_id[629].value <= threshold=0.31753237545490265
node_914: feature_name=GO:0097193 feature_id[167].value <= threshold=28.171168327331543
node_915: feature_name=GO:0002903 feature_id[10].value <= threshold=1.0534588098526
node_916: feature_name=GO:1904029 feature_id[208].value <= threshold=23.852136611938477
node_917: feature_name=GO:0050897 feature_id[164].value <= threshold=0.6870408356189728
node_918: feature_name=GO:0006139 feature_id[474].value > threshold=1.3052097624921544e-07
node_920: feature_name=GO:0002821 feature_id[588].value <= threshold=13.81072187423706
node_921: feature_name=GO:0006298 feature_id[621].value <= threshold=24.078600883483887
node_922: feature_name=GO:0003908 feature_id[11].value <= threshold=1.7776933312416077
node_923: feature_name=GO:0030098 feature_id[273].value > threshold=0.0079949083738029
node_927: feature_name=GO:0006808 feature_id[513].value <= threshold=4.34592080116272
node_928: feature_name=GO:0071887 feature_id[283].value <= threshold=10.353787899017334
node_929: feature_name=GO:0038001 feature_id[28].value <= threshold=3.9469382762908936
node_930: feature_name=GO:0042287 feature_id[20].value <= threshold=3.6209195852279663
node_931: feature_name=GO:0003968 feature_id[47].value <= threshold=2.004227638244629
node_932: feature_name=GO:0002698 feature_id[395].value <= threshold=18.51447582244873
node_933: feature_name=GO:0044710 feature_id[719].value <= threshold=179.7340316772461
node_934: feature_name=GO:0007568 feature_id[534].value > threshold=5.035318374633789
node_936: feature_name=GO:0006216 feature_id[504].value <= threshold=2.2605666518211365
node_937: feature_name=GO:0048569 feature_id[793].value <= threshold=5.340231895446777
node_938: feature_name=GO:0001777 feature_id[380].value <= threshold=4.100832939147949
node_939: feature_name=GO:0007600 feature_id[122].value <= threshold=171.13383102416992
node_940: feature_name=GO:0001779 feature_id[378].value <= threshold=6.234851121902466
node_941: feature_name=GO:0030291 feature_id[147].value <= threshold=10.8051118850708
node_942: feature_name=GO:0048534 feature_id[790].value > threshold=0.017984486185014248
node_944: feature_name=GO:0070245 feature_id[326].value <= threshold=3.1282339096069336
node_945: feature_name=GO:0009086 feature_id[547].value <= threshold=0.2896959036588669
node_946: feature_name=GO:0001889 feature_id[387].value <= threshold=17.643128395080566
node_947: feature_name=GO:0048144 feature_id[192].value > threshold=0.20887330174446106
node_951: feature_name=GO:0023026 feature_id[69].value > threshold=3.403424024581909
node_1013: feature_name=GO:0034109 feature_id[601].value > threshold=10.167142868041992
Class: positive genes

Rules_357 passed counts:2

node_0: feature_name=GO:0042113	feature_id[0].value <= threshold=13.408552169799805
node_1: feature_name=GO:0007568	feature_id[534].value > threshold=5.0313897132873535
node_913: feature_name=GO:0032763	feature_id[629].value <= threshold=0.31753237545490265
node_914: feature_name=GO:0097193	feature_id[167].value <= threshold=28.171168327331543
node_915: feature_name=GO:0002903	feature_id[10].value <= threshold=1.0534588098526
node_916: feature_name=GO:1904029	feature_id[208].value <= threshold=23.852136611938477
node_917: feature_name=GO:0050897	feature_id[164].value <= threshold=0.6870408356189728
node_918: feature_name=GO:0006139	feature_id[474].value > threshold=1.3052097624921544e-07
node_920: feature_name=GO:0002821	feature_id[588].value <= threshold=13.81072187423706
node_921: feature_name=GO:0006298	feature_id[621].value <= threshold=24.078600883483887
node_922: feature_name=GO:0003908	feature_id[11].value <= threshold=1.7776933312416077
node_923: feature_name=GO:0030098	feature_id[273].value > threshold=0.0079949083738029
node_927: feature_name=GO:0006808	feature_id[513].value <= threshold=4.34592080116272
node_928: feature_name=GO:0071887	feature_id[283].value <= threshold=10.353787899017334
node_929: feature_name=GO:0038001	feature_id[28].value <= threshold=3.9469382762908936
node_930: feature_name=GO:0042287	feature_id[20].value <= threshold=3.6209195852279663
node_931: feature_name=GO:0003968	feature_id[47].value <= threshold=2.004227638244629
node_932: feature_name=GO:0002698	feature_id[395].value <= threshold=18.51447582244873
node_933: feature_name=GO:0044710	feature_id[719].value <= threshold=179.7340316772461
node_934: feature_name=GO:0007568	feature_id[534].value > threshold=5.035318374633789
node_936: feature_name=GO:0006216	feature_id[504].value <= threshold=2.2605666518211365
node_937: feature_name=GO:0048569	feature_id[793].value <= threshold=5.340231895446777
node_938: feature_name=GO:0001777	feature_id[380].value <= threshold=4.100832939147949
node_939: feature_name=GO:0007600	feature_id[122].value <= threshold=171.13383102416992
node_940: feature_name=GO:0001779	feature_id[378].value <= threshold=6.234851121902466
node_941: feature_name=GO:0030291	feature_id[147].value <= threshold=10.8051118850708
node_942: feature_name=GO:0048534	feature_id[790].value > threshold=0.017984486185014248
node_944: feature_name=GO:0070245	feature_id[326].value <= threshold=3.1282339096069336
node_945: feature_name=GO:0009086	feature_id[547].value <= threshold=0.2896959036588669
node_946: feature_name=GO:0001889	feature_id[387].value <= threshold=17.643128395080566
node_947: feature_name=GO:0048144	feature_id[192].value > threshold=0.20887330174446106
node_951: feature_name=GO:0023026	feature_id[69].value <= threshold=3.403424024581909
node_952: feature_name=GO:0048147	feature_id[788].value <= threshold=4.188283443450928
node_953: feature_name=GO:0090116	feature_id[97].value > threshold=4.617154359817505
node_1007: feature_name=hsa04650	feature_id[13].value > threshold=4.294154644012451

Class: positive genes

Rules_358	passed counts:2
node_0: feature_name=GO:0042113	feature_id[0].value <= threshold=13.408552169799805
node_1: feature_name=GO:0007568	feature_id[534].value > threshold=5.0313897132873535
node_913: feature_name=GO:0032763	feature_id[629].value <= threshold=0.31753237545490265
node_914: feature_name=GO:0097193	feature_id[167].value <= threshold=28.171168327331543
node_915: feature_name=GO:0002903	feature_id[10].value <= threshold=1.0534588098526
node_916: feature_name=GO:1904029	feature_id[208].value <= threshold=23.852136611938477
node_917: feature_name=GO:0050897	feature_id[164].value <= threshold=0.6870408356189728
node_918: feature_name=GO:0006139	feature_id[474].value > threshold=1.3052097624921544e-07
node_920: feature_name=GO:0002821	feature_id[588].value <= threshold=13.81072187423706
node_921: feature_name=GO:0006298	feature_id[621].value <= threshold=24.078600883483887
node_922: feature_name=GO:0003908	feature_id[11].value <= threshold=1.7776933312416077
node_923: feature_name=GO:0030098	feature_id[273].value > threshold=0.0079949083738029
node_927: feature_name=GO:0006808	feature_id[513].value <= threshold=4.34592080116272
node_928: feature_name=GO:0071887	feature_id[283].value <= threshold=10.353787899017334
node_929: feature_name=GO:0038001	feature_id[28].value <= threshold=3.9469382762908936
node_930: feature_name=GO:0042287	feature_id[20].value <= threshold=3.6209195852279663
node_931: feature_name=GO:0003968	feature_id[47].value <= threshold=2.004227638244629
node_932: feature_name=GO:0002698	feature_id[395].value <= threshold=18.51447582244873
node_933: feature_name=GO:0044710	feature_id[719].value <= threshold=179.7340316772461
node_934: feature_name=GO:0007568	feature_id[534].value > threshold=5.035318374633789
node_936: feature_name=GO:0006216	feature_id[504].value <= threshold=2.2605666518211365
node_937: feature_name=GO:0048569	feature_id[793].value <= threshold=5.340231895446777
node_938: feature_name=GO:0001777	feature_id[380].value <= threshold=4.100832939147949
node_939: feature_name=GO:0007600	feature_id[122].value <= threshold=171.13383102416992
node_940: feature_name=GO:0001779	feature_id[378].value <= threshold=6.234851121902466
node_941: feature_name=GO:0030291	feature_id[147].value <= threshold=10.8051118850708
node_942: feature_name=GO:0048534	feature_id[790].value > threshold=0.017984486185014248
node_944: feature_name=GO:0070245	feature_id[326].value <= threshold=3.1282339096069336
node_945: feature_name=GO:0009086	feature_id[547].value <= threshold=0.2896959036588669
node_946: feature_name=GO:0001889	feature_id[387].value <= threshold=17.643128395080566
node_947: feature_name=GO:0048144	feature_id[192].value > threshold=0.20887330174446106
node_951: feature_name=GO:0023026	feature_id[69].value <= threshold=3.403424024581909

node_952: feature_name=GO:0048147 feature_id[788].value <= threshold=4.188283443450928
node_953: feature_name=GO:0090116 feature_id[97].value <= threshold=4.617154359817505
node_954: feature_name=GO:0002863 feature_id[490].value <= threshold=4.8968470096588135
node_955: feature_name=GO:0002524 feature_id[72].value <= threshold=3.035340189933777
node_956: feature_name=GO:0042130 feature_id[16].value <= threshold=7.285413980484009
node_957: feature_name=GO:0071456 feature_id[500].value <= threshold=21.212870597839355
node_958: feature_name=GO:2001238 feature_id[27].value <= threshold=7.849715709686279
node_959: feature_name=GO:0023030 feature_id[45].value <= threshold=1.7420591711997986
node_960: feature_name=GO:0072593 feature_id[381].value > threshold=22.993029594421387
node_986: feature_name=hsa04115 feature_id[324].value <= threshold=2.2171006202697754
Class: positive genes

Rules_359 passed counts:2
node_0: feature_name=GO:0042113 feature_id[0].value <= threshold=13.408552169799805
node_1: feature_name=GO:0007568 feature_id[534].value > threshold=5.0313897132873535
node_913: feature_name=GO:0032763 feature_id[629].value <= threshold=0.31753237545490265
node_914: feature_name=GO:0097193 feature_id[167].value <= threshold=28.171168327331543
node_915: feature_name=GO:0002903 feature_id[10].value <= threshold=1.0534588098526
node_916: feature_name=GO:1904029 feature_id[208].value <= threshold=23.852136611938477
node_917: feature_name=GO:0050897 feature_id[164].value <= threshold=0.6870408356189728
node_918: feature_name=GO:0006139 feature_id[474].value > threshold=1.3052097624921544e-07
node_920: feature_name=GO:0002821 feature_id[588].value <= threshold=13.81072187423706
node_921: feature_name=GO:0006298 feature_id[621].value <= threshold=24.078600883483887
node_922: feature_name=GO:0003908 feature_id[11].value <= threshold=1.7776933312416077
node_923: feature_name=GO:0030098 feature_id[273].value > threshold=0.0079949083738029
node_927: feature_name=GO:0006808 feature_id[513].value <= threshold=4.34592080116272
node_928: feature_name=GO:0071887 feature_id[283].value <= threshold=10.353787899017334
node_929: feature_name=GO:0038001 feature_id[28].value <= threshold=3.9469382762908936
node_930: feature_name=GO:0042287 feature_id[20].value <= threshold=3.6209195852279663
node_931: feature_name=GO:0003968 feature_id[47].value <= threshold=2.004227638244629
node_932: feature_name=GO:0002698 feature_id[395].value <= threshold=18.51447582244873
node_933: feature_name=GO:0044710 feature_id[719].value <= threshold=179.7340316772461
node_934: feature_name=GO:0007568 feature_id[534].value > threshold=5.035318374633789
node_936: feature_name=GO:0006216 feature_id[504].value <= threshold=2.2605666518211365
node_937: feature_name=GO:0048569 feature_id[793].value <= threshold=5.340231895446777

node_938: feature_name=GO:0001777 feature_id[380].value <= threshold=4.100832939147949
node_939: feature_name=GO:0007600 feature_id[122].value <= threshold=171.13383102416992
node_940: feature_name=GO:0001779 feature_id[378].value <= threshold=6.234851121902466
node_941: feature_name=GO:0030291 feature_id[147].value <= threshold=10.8051118850708
node_942: feature_name=GO:0048534 feature_id[790].value > threshold=0.017984486185014248
node_944: feature_name=GO:0070245 feature_id[326].value <= threshold=3.1282339096069336
node_945: feature_name=GO:0009086 feature_id[547].value <= threshold=0.2896959036588669
node_946: feature_name=GO:0001889 feature_id[387].value <= threshold=17.643128395080566
node_947: feature_name=GO:0048144 feature_id[192].value > threshold=0.20887330174446106
node_951: feature_name=GO:0023026 feature_id[69].value <= threshold=3.403424024581909
node_952: feature_name=GO:0048147 feature_id[788].value <= threshold=4.188283443450928
node_953: feature_name=GO:0090116 feature_id[97].value <= threshold=4.617154359817505
node_954: feature_name=GO:0002863 feature_id[490].value <= threshold=4.8968470096588135
node_955: feature_name=GO:0002524 feature_id[72].value <= threshold=3.035340189933777
node_956: feature_name=GO:0042130 feature_id[16].value <= threshold=7.285413980484009
node_957: feature_name=GO:0071456 feature_id[500].value <= threshold=21.212870597839355
node_958: feature_name=GO:2001238 feature_id[27].value <= threshold=7.849715709686279
node_959: feature_name=GO:0023030 feature_id[45].value <= threshold=1.7420591711997986
node_960: feature_name=GO:0072593 feature_id[381].value <= threshold=22.993029594421387
node_961: feature_name=GO:0034101 feature_id[646].value > threshold=13.430044651031494
node_983: feature_name=GO:0045143 feature_id[732].value <= threshold=0.021482162177562714
Class: negative genes

Rules_360 passed counts:2
node_0: feature_name=GO:0042113 feature_id[0].value <= threshold=13.408552169799805
node_1: feature_name=GO:0007568 feature_id[534].value > threshold=5.0313897132873535
node_913: feature_name=GO:0032763 feature_id[629].value <= threshold=0.31753237545490265
node_914: feature_name=GO:0097193 feature_id[167].value <= threshold=28.171168327331543
node_915: feature_name=GO:0002903 feature_id[10].value <= threshold=1.0534588098526
node_916: feature_name=GO:1904029 feature_id[208].value <= threshold=23.852136611938477
node_917: feature_name=GO:0050897 feature_id[164].value <= threshold=0.6870408356189728
node_918: feature_name=GO:0006139 feature_id[474].value > threshold=1.3052097624921544e-07
node_920: feature_name=GO:0002821 feature_id[588].value <= threshold=13.81072187423706
node_921: feature_name=GO:0006298 feature_id[621].value <= threshold=24.078600883483887
node_922: feature_name=GO:0003908 feature_id[11].value <= threshold=1.7776933312416077

node_923: feature_name=GO:0030098 feature_id[273].value > threshold=0.0079949083738029
node_927: feature_name=GO:0006808 feature_id[513].value <= threshold=4.34592080116272
node_928: feature_name=GO:0071887 feature_id[283].value <= threshold=10.353787899017334
node_929: feature_name=GO:0038001 feature_id[28].value <= threshold=3.9469382762908936
node_930: feature_name=GO:0042287 feature_id[20].value <= threshold=3.6209195852279663
node_931: feature_name=GO:0003968 feature_id[47].value <= threshold=2.004227638244629
node_932: feature_name=GO:0002698 feature_id[395].value <= threshold=18.51447582244873
node_933: feature_name=GO:0044710 feature_id[719].value <= threshold=179.7340316772461
node_934: feature_name=GO:0007568 feature_id[534].value > threshold=5.035318374633789
node_936: feature_name=GO:0006216 feature_id[504].value <= threshold=2.2605666518211365
node_937: feature_name=GO:0048569 feature_id[793].value <= threshold=5.340231895446777
node_938: feature_name=GO:0001777 feature_id[380].value <= threshold=4.100832939147949
node_939: feature_name=GO:0007600 feature_id[122].value <= threshold=171.13383102416992
node_940: feature_name=GO:0001779 feature_id[378].value <= threshold=6.234851121902466
node_941: feature_name=GO:0030291 feature_id[147].value <= threshold=10.8051118850708
node_942: feature_name=GO:0048534 feature_id[790].value > threshold=0.017984486185014248
node_944: feature_name=GO:0070245 feature_id[326].value <= threshold=3.1282339096069336
node_945: feature_name=GO:0009086 feature_id[547].value <= threshold=0.2896959036588669
node_946: feature_name=GO:0001889 feature_id[387].value <= threshold=17.643128395080566
node_947: feature_name=GO:0048144 feature_id[192].value > threshold=0.20887330174446106
node_951: feature_name=GO:0023026 feature_id[69].value <= threshold=3.403424024581909
node_952: feature_name=GO:0048147 feature_id[788].value <= threshold=4.188283443450928
node_953: feature_name=GO:0090116 feature_id[97].value <= threshold=4.617154359817505
node_954: feature_name=GO:0002863 feature_id[490].value <= threshold=4.8968470096588135
node_955: feature_name=GO:0002524 feature_id[72].value <= threshold=3.035340189933777
node_956: feature_name=GO:0042130 feature_id[16].value <= threshold=7.285413980484009
node_957: feature_name=GO:0071456 feature_id[500].value <= threshold=21.212870597839355
node_958: feature_name=GO:2001238 feature_id[27].value <= threshold=7.849715709686279
node_959: feature_name=GO:0023030 feature_id[45].value <= threshold=1.7420591711997986
node_960: feature_name=GO:0072593 feature_id[381].value <= threshold=22.993029594421387
node_961: feature_name=GO:0034101 feature_id[646].value <= threshold=13.430044651031494
node_962: feature_name=GO:0097153 feature_id[226].value > threshold=10.147814273834229
node_980: feature_name=GO:0071901 feature_id[407].value > threshold=1.0248211920261383
Class: negative genes

Rules_361	passed counts:2
node_0: feature_name=GO:0042113	feature_id[0].value <= threshold=13.408552169799805
node_1: feature_name=GO:0007568	feature_id[534].value > threshold=5.0313897132873535
node_913: feature_name=GO:0032763	feature_id[629].value <= threshold=0.31753237545490265
node_914: feature_name=GO:0097193	feature_id[167].value <= threshold=28.171168327331543
node_915: feature_name=GO:0002903	feature_id[10].value <= threshold=1.0534588098526
node_916: feature_name=GO:1904029	feature_id[208].value <= threshold=23.852136611938477
node_917: feature_name=GO:0050897	feature_id[164].value <= threshold=0.6870408356189728
node_918: feature_name=GO:0006139	feature_id[474].value > threshold=1.3052097624921544e-07
node_920: feature_name=GO:0002821	feature_id[588].value <= threshold=13.81072187423706
node_921: feature_name=GO:0006298	feature_id[621].value <= threshold=24.078600883483887
node_922: feature_name=GO:0003908	feature_id[11].value <= threshold=1.7776933312416077
node_923: feature_name=GO:0030098	feature_id[273].value > threshold=0.0079949083738029
node_927: feature_name=GO:0006808	feature_id[513].value <= threshold=4.34592080116272
node_928: feature_name=GO:0071887	feature_id[283].value <= threshold=10.353787899017334
node_929: feature_name=GO:0038001	feature_id[28].value <= threshold=3.9469382762908936
node_930: feature_name=GO:0042287	feature_id[20].value <= threshold=3.6209195852279663
node_931: feature_name=GO:0003968	feature_id[47].value <= threshold=2.004227638244629
node_932: feature_name=GO:0002698	feature_id[395].value <= threshold=18.51447582244873
node_933: feature_name=GO:0044710	feature_id[719].value <= threshold=179.7340316772461
node_934: feature_name=GO:0007568	feature_id[534].value > threshold=5.035318374633789
node_936: feature_name=GO:0006216	feature_id[504].value <= threshold=2.2605666518211365
node_937: feature_name=GO:0048569	feature_id[793].value <= threshold=5.340231895446777
node_938: feature_name=GO:0001777	feature_id[380].value <= threshold=4.100832939147949
node_939: feature_name=GO:0007600	feature_id[122].value <= threshold=171.13383102416992
node_940: feature_name=GO:0001779	feature_id[378].value <= threshold=6.234851121902466
node_941: feature_name=GO:0030291	feature_id[147].value <= threshold=10.8051118850708
node_942: feature_name=GO:0048534	feature_id[790].value > threshold=0.017984486185014248
node_944: feature_name=GO:0070245	feature_id[326].value <= threshold=3.1282339096069336
node_945: feature_name=GO:0009086	feature_id[547].value <= threshold=0.2896959036588669
node_946: feature_name=GO:0001889	feature_id[387].value <= threshold=17.643128395080566
node_947: feature_name=GO:0048144	feature_id[192].value > threshold=0.20887330174446106
node_951: feature_name=GO:0023026	feature_id[69].value <= threshold=3.403424024581909
node_952: feature_name=GO:0048147	feature_id[788].value <= threshold=4.188283443450928
node_953: feature_name=GO:0090116	feature_id[97].value <= threshold=4.617154359817505

node_954: feature_name=GO:0002863 feature_id[490].value <= threshold=4.896847009658135
node_955: feature_name=GO:0002524 feature_id[72].value <= threshold=3.035340189933777
node_956: feature_name=GO:0042130 feature_id[16].value <= threshold=7.285413980484009
node_957: feature_name=GO:0071456 feature_id[500].value <= threshold=21.212870597839355
node_958: feature_name=GO:2001238 feature_id[27].value <= threshold=7.849715709686279
node_959: feature_name=GO:0023030 feature_id[45].value <= threshold=1.7420591711997986
node_960: feature_name=GO:0072593 feature_id[381].value <= threshold=22.993029594421387
node_961: feature_name=GO:0034101 feature_id[646].value <= threshold=13.430044651031494
node_962: feature_name=GO:0097153 feature_id[226].value <= threshold=10.147814273834229
node_963: feature_name=GO:0001836 feature_id[450].value > threshold=7.252067804336548
node_977: feature_name=GO:1901030 feature_id[341].value <= threshold=2.0671581029891968
Class: positive genes

Rules_362 passed counts:2
node_0: feature_name=GO:0042113 feature_id[0].value <= threshold=13.408552169799805
node_1: feature_name=GO:0007568 feature_id[534].value > threshold=5.0313897132873535
node_913: feature_name=GO:0032763 feature_id[629].value <= threshold=0.31753237545490265
node_914: feature_name=GO:0097193 feature_id[167].value <= threshold=28.171168327331543
node_915: feature_name=GO:0002903 feature_id[10].value <= threshold=1.0534588098526
node_916: feature_name=GO:1904029 feature_id[208].value <= threshold=23.852136611938477
node_917: feature_name=GO:0050897 feature_id[164].value <= threshold=0.6870408356189728
node_918: feature_name=GO:0006139 feature_id[474].value > threshold=1.3052097624921544e-07
node_920: feature_name=GO:0002821 feature_id[588].value <= threshold=13.81072187423706
node_921: feature_name=GO:0006298 feature_id[621].value <= threshold=24.078600883483887
node_922: feature_name=GO:0003908 feature_id[11].value <= threshold=1.7776933312416077
node_923: feature_name=GO:0030098 feature_id[273].value > threshold=0.0079949083738029
node_927: feature_name=GO:0006808 feature_id[513].value <= threshold=4.34592080116272
node_928: feature_name=GO:0071887 feature_id[283].value <= threshold=10.353787899017334
node_929: feature_name=GO:0038001 feature_id[28].value <= threshold=3.9469382762908936
node_930: feature_name=GO:0042287 feature_id[20].value <= threshold=3.6209195852279663
node_931: feature_name=GO:0003968 feature_id[47].value <= threshold=2.004227638244629
node_932: feature_name=GO:0002698 feature_id[395].value <= threshold=18.51447582244873
node_933: feature_name=GO:0044710 feature_id[719].value <= threshold=179.7340316772461
node_934: feature_name=GO:0007568 feature_id[534].value > threshold=5.035318374633789
node_936: feature_name=GO:0006216 feature_id[504].value <= threshold=2.2605666518211365

node_937: feature_name=GO:0048569 feature_id[793].value <= threshold=5.340231895446777
node_938: feature_name=GO:0001777 feature_id[380].value <= threshold=4.100832939147949
node_939: feature_name=GO:0007600 feature_id[122].value <= threshold=171.13383102416992
node_940: feature_name=GO:0001779 feature_id[378].value <= threshold=6.234851121902466
node_941: feature_name=GO:0030291 feature_id[147].value <= threshold=10.8051118850708
node_942: feature_name=GO:0048534 feature_id[790].value > threshold=0.017984486185014248
node_944: feature_name=GO:0070245 feature_id[326].value <= threshold=3.1282339096069336
node_945: feature_name=GO:0009086 feature_id[547].value <= threshold=0.2896959036588669
node_946: feature_name=GO:0001889 feature_id[387].value <= threshold=17.643128395080566
node_947: feature_name=GO:0048144 feature_id[192].value > threshold=0.20887330174446106
node_951: feature_name=GO:0023026 feature_id[69].value <= threshold=3.403424024581909
node_952: feature_name=GO:0048147 feature_id[788].value <= threshold=4.188283443450928
node_953: feature_name=GO:0090116 feature_id[97].value <= threshold=4.617154359817505
node_954: feature_name=GO:0002863 feature_id[490].value <= threshold=4.8968470096588135
node_955: feature_name=GO:0002524 feature_id[72].value <= threshold=3.035340189933777
node_956: feature_name=GO:0042130 feature_id[16].value <= threshold=7.285413980484009
node_957: feature_name=GO:0071456 feature_id[500].value <= threshold=21.212870597839355
node_958: feature_name=GO:2001238 feature_id[27].value <= threshold=7.849715709686279
node_959: feature_name=GO:0023030 feature_id[45].value <= threshold=1.7420591711997986
node_960: feature_name=GO:0072593 feature_id[381].value <= threshold=22.993029594421387
node_961: feature_name=GO:0034101 feature_id[646].value <= threshold=13.430044651031494
node_962: feature_name=GO:0097153 feature_id[226].value <= threshold=10.147814273834229
node_963: feature_name=GO:0001836 feature_id[450].value <= threshold=7.252067804336548
node_964: feature_name=GO:0032504 feature_id[244].value > threshold=0.15777301788330078
node_968: feature_name=GO:0043379 feature_id[714].value <= threshold=1.5959861278533936
node_969: feature_name=GO:0001889 feature_id[387].value <= threshold=0.3281313180923462
node_970: feature_name=GO:0002467 feature_id[39].value > threshold=0.9327549636363983
Class: positive genes

Rules_363 passed counts:2
node_0: feature_name=GO:0042113 feature_id[0].value <= threshold=13.408552169799805
node_1: feature_name=GO:0007568 feature_id[534].value > threshold=5.0313897132873535
node_913: feature_name=GO:0032763 feature_id[629].value <= threshold=0.31753237545490265
node_914: feature_name=GO:0097193 feature_id[167].value <= threshold=28.171168327331543
node_915: feature_name=GO:0002903 feature_id[10].value <= threshold=1.0534588098526

node_916: feature_name=GO:1904029 feature_id[208].value <= threshold=23.852136611938477
node_917: feature_name=GO:0050897 feature_id[164].value <= threshold=0.6870408356189728
node_918: feature_name=GO:0006139 feature_id[474].value > threshold=1.3052097624921544e-07
node_920: feature_name=GO:0002821 feature_id[588].value <= threshold=13.81072187423706
node_921: feature_name=GO:0006298 feature_id[621].value <= threshold=24.078600883483887
node_922: feature_name=GO:0003908 feature_id[11].value <= threshold=1.7776933312416077
node_923: feature_name=GO:0030098 feature_id[273].value > threshold=0.0079949083738029
node_927: feature_name=GO:0006808 feature_id[513].value <= threshold=4.34592080116272
node_928: feature_name=GO:0071887 feature_id[283].value <= threshold=10.353787899017334
node_929: feature_name=GO:0038001 feature_id[28].value <= threshold=3.9469382762908936
node_930: feature_name=GO:0042287 feature_id[20].value <= threshold=3.6209195852279663
node_931: feature_name=GO:0003968 feature_id[47].value <= threshold=2.004227638244629
node_932: feature_name=GO:0002698 feature_id[395].value <= threshold=18.51447582244873
node_933: feature_name=GO:0044710 feature_id[719].value <= threshold=179.7340316772461
node_934: feature_name=GO:0007568 feature_id[534].value > threshold=5.035318374633789
node_936: feature_name=GO:0006216 feature_id[504].value <= threshold=2.2605666518211365
node_937: feature_name=GO:0048569 feature_id[793].value <= threshold=5.340231895446777
node_938: feature_name=GO:0001777 feature_id[380].value <= threshold=4.100832939147949
node_939: feature_name=GO:0007600 feature_id[122].value <= threshold=171.13383102416992
node_940: feature_name=GO:0001779 feature_id[378].value <= threshold=6.234851121902466
node_941: feature_name=GO:0030291 feature_id[147].value <= threshold=10.8051118850708
node_942: feature_name=GO:0048534 feature_id[790].value > threshold=0.017984486185014248
node_944: feature_name=GO:0070245 feature_id[326].value <= threshold=3.1282339096069336
node_945: feature_name=GO:0009086 feature_id[547].value <= threshold=0.2896959036588669
node_946: feature_name=GO:0001889 feature_id[387].value <= threshold=17.643128395080566
node_947: feature_name=GO:0048144 feature_id[192].value <= threshold=0.20887330174446106
node_948: feature_name=GO:0042110 feature_id[193].value <= threshold=2.3200501203536987
Class: positive genes

Rules_364 passed counts:2
node_0: feature_name=GO:0042113 feature_id[0].value <= threshold=13.408552169799805
node_1: feature_name=GO:0007568 feature_id[534].value > threshold=5.0313897132873535
node_913: feature_name=GO:0032763 feature_id[629].value <= threshold=0.31753237545490265
node_914: feature_name=GO:0097193 feature_id[167].value <= threshold=28.171168327331543
node_915: feature_name=GO:0002903 feature_id[10].value <= threshold=1.0534588098526

node_916: feature_name=GO:1904029 feature_id[208].value <= threshold=23.852136611938477
node_917: feature_name=GO:0050897 feature_id[164].value <= threshold=0.6870408356189728
node_918: feature_name=GO:0006139 feature_id[474].value <= threshold=1.3052097624921544e-07
Class: positive genes

Rules_365 passed counts:2
node_0: feature_name=GO:0042113 feature_id[0].value <= threshold=13.408552169799805
node_1: feature_name=GO:0007568 feature_id[534].value <= threshold=5.0313897132873535
node_2: feature_name=GO:0002705 feature_id[541].value > threshold=3.200145721435547
node_870: feature_name=GO:0046634 feature_id[46].value <= threshold=8.9394211769104
node_871: feature_name=GO:0048523 feature_id[293].value > threshold=19.963229179382324
node_909: feature_name=GO:0050731 feature_id[804].value <= threshold=1.0901886820793152
Class: negative genes

Rules_366 passed counts:2
node_0: feature_name=GO:0042113 feature_id[0].value <= threshold=13.408552169799805
node_1: feature_name=GO:0007568 feature_id[534].value <= threshold=5.0313897132873535
node_2: feature_name=GO:0002705 feature_id[541].value > threshold=3.200145721435547
node_870: feature_name=GO:0046634 feature_id[46].value <= threshold=8.9394211769104
node_871: feature_name=GO:0048523 feature_id[293].value <= threshold=19.963229179382324
node_872: feature_name=GO:2001237 feature_id[796].value > threshold=0.09761488810181618
node_880: feature_name=GO:0006808 feature_id[513].value <= threshold=1.7969900965690613
node_881: feature_name=GO:0032479 feature_id[441].value > threshold=0.13597124069929123
node_887: feature_name=GO:0043226 feature_id[138].value <= threshold=12.855401992797852
node_888: feature_name=GO:0060576 feature_id[204].value > threshold=2.0569241046905518
Class: positive genes

Rules_367 passed counts:2
node_0: feature_name=GO:0042113 feature_id[0].value <= threshold=13.408552169799805
node_1: feature_name=GO:0007568 feature_id[534].value <= threshold=5.0313897132873535
node_2: feature_name=GO:0002705 feature_id[541].value > threshold=3.200145721435547
node_870: feature_name=GO:0046634 feature_id[46].value <= threshold=8.9394211769104
node_871: feature_name=GO:0048523 feature_id[293].value <= threshold=19.963229179382324
node_872: feature_name=GO:2001237 feature_id[796].value > threshold=0.09761488810181618
node_880: feature_name=GO:0006808 feature_id[513].value <= threshold=1.7969900965690613

node_881: feature_name=GO:0032479 feature_id[441].value > threshold=0.13597124069929123
node_887: feature_name=GO:0043226 feature_id[138].value <= threshold=12.855401992797852
node_888: feature_name=GO:0060576 feature_id[204].value <= threshold=2.0569241046905518
node_889: feature_name=GO:0002719 feature_id[479].value > threshold=5.209295988082886
Class: positive genes

Rules_368 passed counts:2
node_0: feature_name=GO:0042113 feature_id[0].value <= threshold=13.408552169799805
node_1: feature_name=GO:0007568 feature_id[534].value <= threshold=5.0313897132873535
node_2: feature_name=GO:0002705 feature_id[541].value > threshold=3.200145721435547
node_870: feature_name=GO:0046634 feature_id[46].value <= threshold=8.9394211769104
node_871: feature_name=GO:0048523 feature_id[293].value <= threshold=19.963229179382324
node_872: feature_name=GO:2001237 feature_id[796].value > threshold=0.09761488810181618
node_880: feature_name=GO:0006808 feature_id[513].value <= threshold=1.7969900965690613
node_881: feature_name=GO:0032479 feature_id[441].value > threshold=0.13597124069929123
node_887: feature_name=GO:0043226 feature_id[138].value <= threshold=12.855401992797852
node_888: feature_name=GO:0060576 feature_id[204].value <= threshold=2.0569241046905518
node_889: feature_name=GO:0002719 feature_id[479].value <= threshold=5.209295988082886
node_890: feature_name=GO:0031104 feature_id[111].value > threshold=1.9710296988487244
Class: positive genes

Rules_369 passed counts:2
node_0: feature_name=GO:0042113 feature_id[0].value <= threshold=13.408552169799805
node_1: feature_name=GO:0007568 feature_id[534].value <= threshold=5.0313897132873535
node_2: feature_name=GO:0002705 feature_id[541].value > threshold=3.200145721435547
node_870: feature_name=GO:0046634 feature_id[46].value <= threshold=8.9394211769104
node_871: feature_name=GO:0048523 feature_id[293].value <= threshold=19.963229179382324
node_872: feature_name=GO:2001237 feature_id[796].value <= threshold=0.09761488810181618
node_873: feature_name=GO:0097028 feature_id[443].value > threshold=1.0470510721206665
node_877: feature_name=GO:0002718 feature_id[481].value > threshold=3.8375377655029297
Class: negative genes

Rules_370 passed counts:2
node_0: feature_name=GO:0042113 feature_id[0].value <= threshold=13.408552169799805
node_1: feature_name=GO:0007568 feature_id[534].value <= threshold=5.0313897132873535

node_2: feature_name=GO:0002705 feature_id[541].value <= threshold=3.200145721435547
node_3: feature_name=GO:1901525 feature_id[576].value > threshold=0.4466460347175598
node_617: feature_name=GO:0005622 feature_id[233].value > threshold=90.85730743408203
node_847: feature_name=GO:0002639 feature_id[466].value <= threshold=0.32853202521800995
node_848: feature_name=GO:0002636 feature_id[447].value <= threshold=0.3303467631340027
node_849: feature_name=GO:0001783 feature_id[33].value > threshold=0.07426292821764946
node_851: feature_name=GO:0032763 feature_id[629].value > threshold=0.21307963132858276
node_853: feature_name=GO:0045404 feature_id[741].value > threshold=0.4299612492322922
Class: positive genes

Rules_371 passed counts:2
node_0: feature_name=GO:0042113 feature_id[0].value <= threshold=13.408552169799805
node_1: feature_name=GO:0007568 feature_id[534].value <= threshold=5.0313897132873535
node_2: feature_name=GO:0002705 feature_id[541].value <= threshold=3.200145721435547
node_3: feature_name=GO:1901525 feature_id[576].value > threshold=0.4466460347175598
node_617: feature_name=GO:0005622 feature_id[233].value > threshold=90.85730743408203
node_847: feature_name=GO:0002639 feature_id[466].value <= threshold=0.32853202521800995
node_848: feature_name=GO:0002636 feature_id[447].value <= threshold=0.3303467631340027
node_849: feature_name=GO:0001783 feature_id[33].value > threshold=0.07426292821764946
node_851: feature_name=GO:0032763 feature_id[629].value > threshold=0.21307963132858276
node_853: feature_name=GO:0045404 feature_id[741].value <= threshold=0.4299612492322922
node_854: feature_name=GO:0033158 feature_id[379].value > threshold=0.09315907582640648
node_856: feature_name=GO:0008156 feature_id[239].value > threshold=0.07084834203124046
node_858: feature_name=GO:0038065 feature_id[670].value > threshold=0.5210016071796417
node_860: feature_name=GO:0050678 feature_id[798].value > threshold=1.3203300088644028
Class: positive genes

Rules_372 passed counts:2
node_0: feature_name=GO:0042113 feature_id[0].value <= threshold=13.408552169799805
node_1: feature_name=GO:0007568 feature_id[534].value <= threshold=5.0313897132873535
node_2: feature_name=GO:0002705 feature_id[541].value <= threshold=3.200145721435547
node_3: feature_name=GO:1901525 feature_id[576].value > threshold=0.4466460347175598
node_617: feature_name=GO:0005622 feature_id[233].value > threshold=90.85730743408203
node_847: feature_name=GO:0002639 feature_id[466].value <= threshold=0.32853202521800995
node_848: feature_name=GO:0002636 feature_id[447].value <= threshold=0.3303467631340027

node_849: feature_name=GO:0001783 feature_id[33].value > threshold=0.07426292821764946
node_851: feature_name=GO:0032763 feature_id[629].value > threshold=0.21307963132858276
node_853: feature_name=GO:0045404 feature_id[741].value <= threshold=0.4299612492322922
node_854: feature_name=GO:0033158 feature_id[379].value > threshold=0.09315907582640648
node_856: feature_name=GO:0008156 feature_id[239].value <= threshold=0.07084834203124046
Class: positive genes

Rules_373 passed counts:2
node_0: feature_name=GO:0042113 feature_id[0].value <= threshold=13.408552169799805
node_1: feature_name=GO:0007568 feature_id[534].value <= threshold=5.0313897132873535
node_2: feature_name=GO:0002705 feature_id[541].value <= threshold=3.200145721435547
node_3: feature_name=GO:1901525 feature_id[576].value > threshold=0.4466460347175598
node_617: feature_name=GO:0005622 feature_id[233].value > threshold=90.85730743408203
node_847: feature_name=GO:0002639 feature_id[466].value <= threshold=0.32853202521800995
node_848: feature_name=GO:0002636 feature_id[447].value <= threshold=0.3303467631340027
node_849: feature_name=GO:0001783 feature_id[33].value > threshold=0.07426292821764946
node_851: feature_name=GO:0032763 feature_id[629].value > threshold=0.21307963132858276
node_853: feature_name=GO:0045404 feature_id[741].value <= threshold=0.4299612492322922
node_854: feature_name=GO:0033158 feature_id[379].value <= threshold=0.09315907582640648
Class: positive genes

Rules_374 passed counts:2
node_0: feature_name=GO:0042113 feature_id[0].value <= threshold=13.408552169799805
node_1: feature_name=GO:0007568 feature_id[534].value <= threshold=5.0313897132873535
node_2: feature_name=GO:0002705 feature_id[541].value <= threshold=3.200145721435547
node_3: feature_name=GO:1901525 feature_id[576].value > threshold=0.4466460347175598
node_617: feature_name=GO:0005622 feature_id[233].value > threshold=90.85730743408203
node_847: feature_name=GO:0002639 feature_id[466].value <= threshold=0.32853202521800995
node_848: feature_name=GO:0002636 feature_id[447].value <= threshold=0.3303467631340027
node_849: feature_name=GO:0001783 feature_id[33].value > threshold=0.07426292821764946
node_851: feature_name=GO:0032763 feature_id[629].value <= threshold=0.21307963132858276
Class: positive genes

Rules_375 passed counts:2
node_0: feature_name=GO:0042113 feature_id[0].value <= threshold=13.408552169799805

node_1: feature_name=GO:0007568 feature_id[534].value <= threshold=5.0313897132873535
node_2: feature_name=GO:0002705 feature_id[541].value <= threshold=3.200145721435547
node_3: feature_name=GO:1901525 feature_id[576].value > threshold=0.4466460347175598
node_617: feature_name=GO:0005622 feature_id[233].value <= threshold=90.85730743408203
node_618: feature_name=GO:1903147 feature_id[200].value <= threshold=7.326428413391113
node_619: feature_name=GO:0003964 feature_id[317].value <= threshold=5.575642108917236
node_620: feature_name=GO:0046498 feature_id[203].value <= threshold=6.620223522186279
node_621: feature_name=GO:0002562 feature_id[25].value > threshold=10.287704467773438
Class: positive genes

Rules_376 passed counts:2
node_0: feature_name=GO:0042113 feature_id[0].value <= threshold=13.408552169799805
node_1: feature_name=GO:0007568 feature_id[534].value <= threshold=5.0313897132873535
node_2: feature_name=GO:0002705 feature_id[541].value <= threshold=3.200145721435547
node_3: feature_name=GO:1901525 feature_id[576].value > threshold=0.4466460347175598
node_617: feature_name=GO:0005622 feature_id[233].value <= threshold=90.85730743408203
node_618: feature_name=GO:1903147 feature_id[200].value <= threshold=7.326428413391113
node_619: feature_name=GO:0003964 feature_id[317].value <= threshold=5.575642108917236
node_620: feature_name=GO:0046498 feature_id[203].value <= threshold=6.620223522186279
node_621: feature_name=GO:0002562 feature_id[25].value <= threshold=10.287704467773438
node_622: feature_name=GO:0006919 feature_id[246].value <= threshold=5.801334381103516
node_623: feature_name=GO:0001889 feature_id[387].value > threshold=2.679414987564087
node_785: feature_name=GO:0030887 feature_id[274].value <= threshold=1.1379476189613342
node_786: feature_name=GO:1902564 feature_id[85].value <= threshold=1.4637635350227356
node_787: feature_name=GO:0045402 feature_id[286].value > threshold=1.5249269604682922
Class: positive genes

Rules_377 passed counts:2
node_0: feature_name=GO:0042113 feature_id[0].value <= threshold=13.408552169799805
node_1: feature_name=GO:0007568 feature_id[534].value <= threshold=5.0313897132873535
node_2: feature_name=GO:0002705 feature_id[541].value <= threshold=3.200145721435547
node_3: feature_name=GO:1901525 feature_id[576].value > threshold=0.4466460347175598
node_617: feature_name=GO:0005622 feature_id[233].value <= threshold=90.85730743408203
node_618: feature_name=GO:1903147 feature_id[200].value <= threshold=7.326428413391113
node_619: feature_name=GO:0003964 feature_id[317].value <= threshold=5.575642108917236

node_620: feature_name=GO:0046498 feature_id[203].value <= threshold=6.620223522186279
node_621: feature_name=GO:0002562 feature_id[25].value <= threshold=10.287704467773438
node_622: feature_name=GO:0006919 feature_id[246].value <= threshold=5.801334381103516
node_623: feature_name=GO:0001889 feature_id[387].value > threshold=2.679414987564087
node_785: feature_name=GO:0030887 feature_id[274].value <= threshold=1.1379476189613342
node_786: feature_name=GO:1902564 feature_id[85].value <= threshold=1.4637635350227356
node_787: feature_name=GO:0045402 feature_id[286].value <= threshold=1.5249269604682922
node_788: feature_name=GO:0034614 feature_id[655].value > threshold=9.142115116119385
Class: positive genes

Rules_378 passed counts:2
node_0: feature_name=GO:0042113 feature_id[0].value <= threshold=13.408552169799805
node_1: feature_name=GO:0007568 feature_id[534].value <= threshold=5.0313897132873535
node_2: feature_name=GO:0002705 feature_id[541].value <= threshold=3.200145721435547
node_3: feature_name=GO:1901525 feature_id[576].value > threshold=0.4466460347175598
node_617: feature_name=GO:0005622 feature_id[233].value <= threshold=90.85730743408203
node_618: feature_name=GO:1903147 feature_id[200].value <= threshold=7.326428413391113
node_619: feature_name=GO:0003964 feature_id[317].value <= threshold=5.575642108917236
node_620: feature_name=GO:0046498 feature_id[203].value <= threshold=6.620223522186279
node_621: feature_name=GO:0002562 feature_id[25].value <= threshold=10.287704467773438
node_622: feature_name=GO:0006919 feature_id[246].value <= threshold=5.801334381103516
node_623: feature_name=GO:0001889 feature_id[387].value > threshold=2.679414987564087
node_785: feature_name=GO:0030887 feature_id[274].value <= threshold=1.1379476189613342
node_786: feature_name=GO:1902564 feature_id[85].value <= threshold=1.4637635350227356
node_787: feature_name=GO:0045402 feature_id[286].value <= threshold=1.5249269604682922
node_788: feature_name=GO:0034614 feature_id[655].value <= threshold=9.142115116119385
node_789: feature_name=GO:0000783 feature_id[191].value <= threshold=1.5124736428260803
node_790: feature_name=GO:0048145 feature_id[348].value > threshold=0.026725558564066887
node_794: feature_name=GO:0044092 feature_id[432].value <= threshold=31.540730476379395
node_795: feature_name=GO:0042771 feature_id[686].value <= threshold=6.047890663146973
node_796: feature_name=GO:0070301 feature_id[81].value <= threshold=5.959718465805054
node_797: feature_name=GO:0006809 feature_id[511].value <= threshold=9.000734329223633
node_798: feature_name=GO:0008150 feature_id[528].value > threshold=0.40075723826885223
node_800: feature_name=GO:0010663 feature_id[561].value > threshold=0.20622049272060394
node_802: feature_name=GO:0009636 feature_id[551].value > threshold=0.12915228679776192

node_804: feature_name=GO:0002524 feature_id[72].value <= threshold=1.0410467386245728
node_805: feature_name=GO:0035872 feature_id[659].value <= threshold=6.628145217895508
node_806: feature_name=GO:0010225 feature_id[558].value <= threshold=4.188441753387451
node_807: feature_name=GO:0002262 feature_id[394].value <= threshold=8.843595027923584
node_808: feature_name=GO:0048569 feature_id[793].value <= threshold=4.370251417160034
node_809: feature_name=GO:0046982 feature_id[172].value <= threshold=0.010676630306988955
node_810: feature_name=GO:0005622 feature_id[233].value > threshold=17.8395357131958
Class: negative genes

Rules_379 passed counts:2
node_0: feature_name=GO:0042113 feature_id[0].value <= threshold=13.408552169799805
node_1: feature_name=GO:0007568 feature_id[534].value <= threshold=5.0313897132873535
node_2: feature_name=GO:0002705 feature_id[541].value <= threshold=3.200145721435547
node_3: feature_name=GO:1901525 feature_id[576].value > threshold=0.4466460347175598
node_617: feature_name=GO:0005622 feature_id[233].value <= threshold=90.85730743408203
node_618: feature_name=GO:1903147 feature_id[200].value <= threshold=7.326428413391113
node_619: feature_name=GO:0003964 feature_id[317].value <= threshold=5.575642108917236
node_620: feature_name=GO:0046498 feature_id[203].value <= threshold=6.620223522186279
node_621: feature_name=GO:0002562 feature_id[25].value <= threshold=10.287704467773438
node_622: feature_name=GO:0006919 feature_id[246].value <= threshold=5.801334381103516
node_623: feature_name=GO:0001889 feature_id[387].value > threshold=2.679414987564087
node_785: feature_name=GO:0030887 feature_id[274].value <= threshold=1.1379476189613342
node_786: feature_name=GO:1902564 feature_id[85].value <= threshold=1.4637635350227356
node_787: feature_name=GO:0045402 feature_id[286].value <= threshold=1.5249269604682922
node_788: feature_name=GO:0034614 feature_id[655].value <= threshold=9.142115116119385
node_789: feature_name=GO:0000783 feature_id[191].value <= threshold=1.5124736428260803
node_790: feature_name=GO:0048145 feature_id[348].value <= threshold=0.026725558564066887
node_791: feature_name=GO:0032496 feature_id[112].value > threshold=0.10570741072297096
Class: positive genes

Rules_380 passed counts:2
node_0: feature_name=GO:0042113 feature_id[0].value <= threshold=13.408552169799805
node_1: feature_name=GO:0007568 feature_id[534].value <= threshold=5.0313897132873535
node_2: feature_name=GO:0002705 feature_id[541].value <= threshold=3.200145721435547
node_3: feature_name=GO:1901525 feature_id[576].value > threshold=0.4466460347175598

node_617: feature_name=GO:0005622 feature_id[233].value <= threshold=90.85730743408203
node_618: feature_name=GO:1903147 feature_id[200].value <= threshold=7.326428413391113
node_619: feature_name=GO:0003964 feature_id[317].value <= threshold=5.575642108917236
node_620: feature_name=GO:0046498 feature_id[203].value <= threshold=6.620223522186279
node_621: feature_name=GO:0002562 feature_id[25].value <= threshold=10.287704467773438
node_622: feature_name=GO:0006919 feature_id[246].value <= threshold=5.801334381103516
node_623: feature_name=GO:0001889 feature_id[387].value <= threshold=2.679414987564087
node_624: feature_name=GO:0006555 feature_id[514].value > threshold=7.819535493850708
Class: positive genes

Rules_381 passed counts:2
node_0: feature_name=GO:0042113 feature_id[0].value <= threshold=13.408552169799805
node_1: feature_name=GO:0007568 feature_id[534].value <= threshold=5.0313897132873535
node_2: feature_name=GO:0002705 feature_id[541].value <= threshold=3.200145721435547
node_3: feature_name=GO:1901525 feature_id[576].value > threshold=0.4466460347175598
node_617: feature_name=GO:0005622 feature_id[233].value <= threshold=90.85730743408203
node_618: feature_name=GO:1903147 feature_id[200].value <= threshold=7.326428413391113
node_619: feature_name=GO:0003964 feature_id[317].value <= threshold=5.575642108917236
node_620: feature_name=GO:0046498 feature_id[203].value <= threshold=6.620223522186279
node_621: feature_name=GO:0002562 feature_id[25].value <= threshold=10.287704467773438
node_622: feature_name=GO:0006919 feature_id[246].value <= threshold=5.801334381103516
node_623: feature_name=GO:0001889 feature_id[387].value <= threshold=2.679414987564087
node_624: feature_name=GO:0006555 feature_id[514].value <= threshold=7.819535493850708
node_625: feature_name=GO:0080134 feature_id[650].value <= threshold=38.91967582702637
node_626: feature_name=GO:0042127 feature_id[480].value <= threshold=37.72536659240723
node_627: feature_name=GO:2001251 feature_id[220].value <= threshold=32.564422607421875
node_628: feature_name=GO:0002829 feature_id[493].value <= threshold=2.444612741470337
node_629: feature_name=GO:0008588 feature_id[26].value <= threshold=2.3601274490356445
node_630: feature_name=GO:0033151 feature_id[7].value <= threshold=5.134376287460327
node_631: feature_name=GO:0048145 feature_id[348].value <= threshold=8.836549282073975
node_632: feature_name=GO:0030887 feature_id[274].value <= threshold=1.7000296115875244
node_633: feature_name=GO:0097190 feature_id[117].value <= threshold=33.02078819274902
node_634: feature_name=GO:0046685 feature_id[782].value <= threshold=8.040813446044922
node_635: feature_name=GO:0006346 feature_id[236].value > threshold=6.384642839431763
node_771: feature_name=GO:0045190 feature_id[731].value > threshold=1.3931243419647217

Class: negative genes

Rules_382	passed counts:2
node_0: feature_name=GO:0042113	feature_id[0].value <= threshold=13.408552169799805
node_1: feature_name=GO:0007568	feature_id[534].value <= threshold=5.0313897132873535
node_2: feature_name=GO:0002705	feature_id[541].value <= threshold=3.200145721435547
node_3: feature_name=GO:1901525	feature_id[576].value > threshold=0.4466460347175598
node_617: feature_name=GO:0005622	feature_id[233].value <= threshold=90.85730743408203
node_618: feature_name=GO:1903147	feature_id[200].value <= threshold=7.326428413391113
node_619: feature_name=GO:0003964	feature_id[317].value <= threshold=5.575642108917236
node_620: feature_name=GO:0046498	feature_id[203].value <= threshold=6.620223522186279
node_621: feature_name=GO:0002562	feature_id[25].value <= threshold=10.287704467773438
node_622: feature_name=GO:0006919	feature_id[246].value <= threshold=5.801334381103516
node_623: feature_name=GO:0001889	feature_id[387].value <= threshold=2.679414987564087
node_624: feature_name=GO:0006555	feature_id[514].value <= threshold=7.819535493850708
node_625: feature_name=GO:0080134	feature_id[650].value <= threshold=38.91967582702637
node_626: feature_name=GO:0042127	feature_id[480].value <= threshold=37.72536659240723
node_627: feature_name=GO:2001251	feature_id[220].value <= threshold=32.564422607421875
node_628: feature_name=GO:0002829	feature_id[493].value <= threshold=2.444612741470337
node_629: feature_name=GO:0008588	feature_id[26].value <= threshold=2.3601274490356445
node_630: feature_name=GO:0033151	feature_id[7].value <= threshold=5.134376287460327
node_631: feature_name=GO:0048145	feature_id[348].value <= threshold=8.836549282073975
node_632: feature_name=GO:0030887	feature_id[274].value <= threshold=1.7000296115875244
node_633: feature_name=GO:0097190	feature_id[117].value <= threshold=33.02078819274902
node_634: feature_name=GO:0046685	feature_id[782].value <= threshold=8.040813446044922
node_635: feature_name=GO:0006346	feature_id[236].value > threshold=6.384642839431763
node_771: feature_name=GO:0045190	feature_id[731].value <= threshold=1.3931243419647217

Class: positive genes

Rules_383	passed counts:2
node_0: feature_name=GO:0042113	feature_id[0].value <= threshold=13.408552169799805
node_1: feature_name=GO:0007568	feature_id[534].value <= threshold=5.0313897132873535
node_2: feature_name=GO:0002705	feature_id[541].value <= threshold=3.200145721435547
node_3: feature_name=GO:1901525	feature_id[576].value > threshold=0.4466460347175598
node_617: feature_name=GO:0005622	feature_id[233].value <= threshold=90.85730743408203

node_618: feature_name=GO:1903147 feature_id[200].value <= threshold=7.326428413391113
node_619: feature_name=GO:0003964 feature_id[317].value <= threshold=5.575642108917236
node_620: feature_name=GO:0046498 feature_id[203].value <= threshold=6.620223522186279
node_621: feature_name=GO:0002562 feature_id[25].value <= threshold=10.287704467773438
node_622: feature_name=GO:0006919 feature_id[246].value <= threshold=5.801334381103516
node_623: feature_name=GO:0001889 feature_id[387].value <= threshold=2.679414987564087
node_624: feature_name=GO:0006555 feature_id[514].value <= threshold=7.819535493850708
node_625: feature_name=GO:0080134 feature_id[650].value <= threshold=38.91967582702637
node_626: feature_name=GO:0042127 feature_id[480].value <= threshold=37.72536659240723
node_627: feature_name=GO:2001251 feature_id[220].value <= threshold=32.564422607421875
node_628: feature_name=GO:0002829 feature_id[493].value <= threshold=2.444612741470337
node_629: feature_name=GO:0008588 feature_id[26].value <= threshold=2.3601274490356445
node_630: feature_name=GO:0033151 feature_id[7].value <= threshold=5.134376287460327
node_631: feature_name=GO:0048145 feature_id[348].value <= threshold=8.836549282073975
node_632: feature_name=GO:0030887 feature_id[274].value <= threshold=1.7000296115875244
node_633: feature_name=GO:0097190 feature_id[117].value <= threshold=33.02078819274902
node_634: feature_name=GO:0046685 feature_id[782].value <= threshold=8.040813446044922
node_635: feature_name=GO:0006346 feature_id[236].value <= threshold=6.384642839431763
node_636: feature_name=GO:0048537 feature_id[66].value > threshold=3.1739262342453003
node_768: feature_name=GO:0032845 feature_id[55].value > threshold=1.0962145626544952
Class: negative genes

Rules_384 passed counts:2
node_0: feature_name=GO:0042113 feature_id[0].value <= threshold=13.408552169799805
node_1: feature_name=GO:0007568 feature_id[534].value <= threshold=5.0313897132873535
node_2: feature_name=GO:0002705 feature_id[541].value <= threshold=3.200145721435547
node_3: feature_name=GO:1901525 feature_id[576].value > threshold=0.4466460347175598
node_617: feature_name=GO:0005622 feature_id[233].value <= threshold=90.85730743408203
node_618: feature_name=GO:1903147 feature_id[200].value <= threshold=7.326428413391113
node_619: feature_name=GO:0003964 feature_id[317].value <= threshold=5.575642108917236
node_620: feature_name=GO:0046498 feature_id[203].value <= threshold=6.620223522186279
node_621: feature_name=GO:0002562 feature_id[25].value <= threshold=10.287704467773438
node_622: feature_name=GO:0006919 feature_id[246].value <= threshold=5.801334381103516
node_623: feature_name=GO:0001889 feature_id[387].value <= threshold=2.679414987564087
node_624: feature_name=GO:0006555 feature_id[514].value <= threshold=7.819535493850708

node_625: feature_name=GO:0080134 feature_id[650].value <= threshold=38.91967582702637
node_626: feature_name=GO:0042127 feature_id[480].value <= threshold=37.72536659240723
node_627: feature_name=GO:2001251 feature_id[220].value <= threshold=32.564422607421875
node_628: feature_name=GO:0002829 feature_id[493].value <= threshold=2.444612741470337
node_629: feature_name=GO:0008588 feature_id[26].value <= threshold=2.3601274490356445
node_630: feature_name=GO:0033151 feature_id[7].value <= threshold=5.134376287460327
node_631: feature_name=GO:0048145 feature_id[348].value <= threshold=8.836549282073975
node_632: feature_name=GO:0030887 feature_id[274].value <= threshold=1.7000296115875244
node_633: feature_name=GO:0097190 feature_id[117].value <= threshold=33.02078819274902
node_634: feature_name=GO:0046685 feature_id[782].value <= threshold=8.040813446044922
node_635: feature_name=GO:0006346 feature_id[236].value <= threshold=6.384642839431763
node_636: feature_name=GO:0048537 feature_id[66].value > threshold=3.1739262342453003
node_768: feature_name=GO:0032845 feature_id[55].value <= threshold=1.0962145626544952
Class: positive genes

Rules_385 passed counts:2
node_0: feature_name=GO:0042113 feature_id[0].value <= threshold=13.408552169799805
node_1: feature_name=GO:0007568 feature_id[534].value <= threshold=5.0313897132873535
node_2: feature_name=GO:0002705 feature_id[541].value <= threshold=3.200145721435547
node_3: feature_name=GO:1901525 feature_id[576].value > threshold=0.4466460347175598
node_617: feature_name=GO:0005622 feature_id[233].value <= threshold=90.85730743408203
node_618: feature_name=GO:1903147 feature_id[200].value <= threshold=7.326428413391113
node_619: feature_name=GO:0003964 feature_id[317].value <= threshold=5.575642108917236
node_620: feature_name=GO:0046498 feature_id[203].value <= threshold=6.620223522186279
node_621: feature_name=GO:0002562 feature_id[25].value <= threshold=10.287704467773438
node_622: feature_name=GO:0006919 feature_id[246].value <= threshold=5.801334381103516
node_623: feature_name=GO:0001889 feature_id[387].value <= threshold=2.679414987564087
node_624: feature_name=GO:0006555 feature_id[514].value <= threshold=7.819535493850708
node_625: feature_name=GO:0080134 feature_id[650].value <= threshold=38.91967582702637
node_626: feature_name=GO:0042127 feature_id[480].value <= threshold=37.72536659240723
node_627: feature_name=GO:2001251 feature_id[220].value <= threshold=32.564422607421875
node_628: feature_name=GO:0002829 feature_id[493].value <= threshold=2.444612741470337
node_629: feature_name=GO:0008588 feature_id[26].value <= threshold=2.3601274490356445
node_630: feature_name=GO:0033151 feature_id[7].value <= threshold=5.134376287460327
node_631: feature_name=GO:0048145 feature_id[348].value <= threshold=8.836549282073975

node_632: feature_name=GO:0030887 feature_id[274].value <= threshold=1.7000296115875244
node_633: feature_name=GO:0097190 feature_id[117].value <= threshold=33.02078819274902
node_634: feature_name=GO:0046685 feature_id[782].value <= threshold=8.040813446044922
node_635: feature_name=GO:0006346 feature_id[236].value <= threshold=6.384642839431763
node_636: feature_name=GO:0048537 feature_id[66].value <= threshold=3.1739262342453003
node_637: feature_name=GO:0070245 feature_id[326].value > threshold=3.0491198301315308
node_765: feature_name=GO:0045429 feature_id[737].value <= threshold=1.1315002739429474
Class: positive genes

Rules_386 passed counts:2
node_0: feature_name=GO:0042113 feature_id[0].value <= threshold=13.408552169799805
node_1: feature_name=GO:0007568 feature_id[534].value <= threshold=5.0313897132873535
node_2: feature_name=GO:0002705 feature_id[541].value <= threshold=3.200145721435547
node_3: feature_name=GO:1901525 feature_id[576].value > threshold=0.4466460347175598
node_617: feature_name=GO:0005622 feature_id[233].value <= threshold=90.85730743408203
node_618: feature_name=GO:1903147 feature_id[200].value <= threshold=7.326428413391113
node_619: feature_name=GO:0003964 feature_id[317].value <= threshold=5.575642108917236
node_620: feature_name=GO:0046498 feature_id[203].value <= threshold=6.620223522186279
node_621: feature_name=GO:0002562 feature_id[25].value <= threshold=10.287704467773438
node_622: feature_name=GO:0006919 feature_id[246].value <= threshold=5.801334381103516
node_623: feature_name=GO:0001889 feature_id[387].value <= threshold=2.679414987564087
node_624: feature_name=GO:0006555 feature_id[514].value <= threshold=7.819535493850708
node_625: feature_name=GO:0080134 feature_id[650].value <= threshold=38.91967582702637
node_626: feature_name=GO:0042127 feature_id[480].value <= threshold=37.72536659240723
node_627: feature_name=GO:2001251 feature_id[220].value <= threshold=32.564422607421875
node_628: feature_name=GO:0002829 feature_id[493].value <= threshold=2.444612741470337
node_629: feature_name=GO:0008588 feature_id[26].value <= threshold=2.3601274490356445
node_630: feature_name=GO:0033151 feature_id[7].value <= threshold=5.134376287460327
node_631: feature_name=GO:0048145 feature_id[348].value <= threshold=8.836549282073975
node_632: feature_name=GO:0030887 feature_id[274].value <= threshold=1.7000296115875244
node_633: feature_name=GO:0097190 feature_id[117].value <= threshold=33.02078819274902
node_634: feature_name=GO:0046685 feature_id[782].value <= threshold=8.040813446044922
node_635: feature_name=GO:0006346 feature_id[236].value <= threshold=6.384642839431763
node_636: feature_name=GO:0048537 feature_id[66].value <= threshold=3.1739262342453003
node_637: feature_name=GO:0070245 feature_id[326].value <= threshold=3.0491198301315308

node_638: feature_name=GO:0038065 feature_id[670].value > threshold=2.0590850114822388
node_762: feature_name=GO:0033554 feature_id[418].value > threshold=3.435133218765259
Class: positive genes

Rules_387 passed counts:2
node_0: feature_name=GO:0042113 feature_id[0].value <= threshold=13.408552169799805
node_1: feature_name=GO:0007568 feature_id[534].value <= threshold=5.0313897132873535
node_2: feature_name=GO:0002705 feature_id[541].value <= threshold=3.200145721435547
node_3: feature_name=GO:1901525 feature_id[576].value > threshold=0.4466460347175598
node_617: feature_name=GO:0005622 feature_id[233].value <= threshold=90.85730743408203
node_618: feature_name=GO:1903147 feature_id[200].value <= threshold=7.326428413391113
node_619: feature_name=GO:0003964 feature_id[317].value <= threshold=5.575642108917236
node_620: feature_name=GO:0046498 feature_id[203].value <= threshold=6.620223522186279
node_621: feature_name=GO:0002562 feature_id[25].value <= threshold=10.287704467773438
node_622: feature_name=GO:0006919 feature_id[246].value <= threshold=5.801334381103516
node_623: feature_name=GO:0001889 feature_id[387].value <= threshold=2.679414987564087
node_624: feature_name=GO:0006555 feature_id[514].value <= threshold=7.819535493850708
node_625: feature_name=GO:0080134 feature_id[650].value <= threshold=38.91967582702637
node_626: feature_name=GO:0042127 feature_id[480].value <= threshold=37.72536659240723
node_627: feature_name=GO:2001251 feature_id[220].value <= threshold=32.564422607421875
node_628: feature_name=GO:0002829 feature_id[493].value <= threshold=2.444612741470337
node_629: feature_name=GO:0008588 feature_id[26].value <= threshold=2.3601274490356445
node_630: feature_name=GO:0033151 feature_id[7].value <= threshold=5.134376287460327
node_631: feature_name=GO:0048145 feature_id[348].value <= threshold=8.836549282073975
node_632: feature_name=GO:0030887 feature_id[274].value <= threshold=1.7000296115875244
node_633: feature_name=GO:0097190 feature_id[117].value <= threshold=33.02078819274902
node_634: feature_name=GO:0046685 feature_id[782].value <= threshold=8.040813446044922
node_635: feature_name=GO:0006346 feature_id[236].value <= threshold=6.384642839431763
node_636: feature_name=GO:0048537 feature_id[66].value <= threshold=3.1739262342453003
node_637: feature_name=GO:0070245 feature_id[326].value <= threshold=3.0491198301315308
node_638: feature_name=GO:0038065 feature_id[670].value <= threshold=2.0590850114822388
node_639: feature_name=GO:0005488 feature_id[187].value <= threshold=84.80076217651367
node_640: feature_name=GO:0060249 feature_id[390].value > threshold=0.07226398587226868
node_652: feature_name=GO:0006974 feature_id[516].value > threshold=5.17782768838515e-06
node_654: feature_name=hsa05210 feature_id[358].value <= threshold=18.730005264282227

node_655: feature_name=GO:0071850 feature_id[40].value <= threshold=5.475317001342773
node_656: feature_name=GO:1904029 feature_id[208].value <= threshold=26.575013160705566
node_657: feature_name=GO:0090594 feature_id[738].value <= threshold=3.000791072845459
node_658: feature_name=GO:0010948 feature_id[569].value > threshold=0.04386143572628498
node_666: feature_name=GO:0032764 feature_id[3].value > threshold=2.5201677083969116
node_740: feature_name=GO:0046500 feature_id[171].value > threshold=0.266752228140831
Class: negative genes

Rules_388 passed counts:2
node_0: feature_name=GO:0042113 feature_id[0].value <= threshold=13.408552169799805
node_1: feature_name=GO:0007568 feature_id[534].value <= threshold=5.0313897132873535
node_2: feature_name=GO:0002705 feature_id[541].value <= threshold=3.200145721435547
node_3: feature_name=GO:1901525 feature_id[576].value > threshold=0.4466460347175598
node_617: feature_name=GO:0005622 feature_id[233].value <= threshold=90.85730743408203
node_618: feature_name=GO:1903147 feature_id[200].value <= threshold=7.326428413391113
node_619: feature_name=GO:0003964 feature_id[317].value <= threshold=5.575642108917236
node_620: feature_name=GO:0046498 feature_id[203].value <= threshold=6.620223522186279
node_621: feature_name=GO:0002562 feature_id[25].value <= threshold=10.287704467773438
node_622: feature_name=GO:0006919 feature_id[246].value <= threshold=5.801334381103516
node_623: feature_name=GO:0001889 feature_id[387].value <= threshold=2.679414987564087
node_624: feature_name=GO:0006555 feature_id[514].value <= threshold=7.819535493850708
node_625: feature_name=GO:0080134 feature_id[650].value <= threshold=38.91967582702637
node_626: feature_name=GO:0042127 feature_id[480].value <= threshold=37.72536659240723
node_627: feature_name=GO:2001251 feature_id[220].value <= threshold=32.564422607421875
node_628: feature_name=GO:0002829 feature_id[493].value <= threshold=2.444612741470337
node_629: feature_name=GO:0008588 feature_id[26].value <= threshold=2.3601274490356445
node_630: feature_name=GO:0033151 feature_id[7].value <= threshold=5.134376287460327
node_631: feature_name=GO:0048145 feature_id[348].value <= threshold=8.836549282073975
node_632: feature_name=GO:0030887 feature_id[274].value <= threshold=1.7000296115875244
node_633: feature_name=GO:0097190 feature_id[117].value <= threshold=33.02078819274902
node_634: feature_name=GO:0046685 feature_id[782].value <= threshold=8.040813446044922
node_635: feature_name=GO:0006346 feature_id[236].value <= threshold=6.384642839431763
node_636: feature_name=GO:0048537 feature_id[66].value <= threshold=3.1739262342453003
node_637: feature_name=GO:0070245 feature_id[326].value <= threshold=3.0491198301315308
node_638: feature_name=GO:0038065 feature_id[670].value <= threshold=2.0590850114822388

node_639: feature_name=GO:0005488 feature_id[187].value <= threshold=84.80076217651367
node_640: feature_name=GO:0060249 feature_id[390].value > threshold=0.07226398587226868
node_652: feature_name=GO:0006974 feature_id[516].value > threshold=5.17782768838515e-06
node_654: feature_name=hsa05210 feature_id[358].value <= threshold=18.730005264282227
node_655: feature_name=GO:0071850 feature_id[40].value <= threshold=5.475317001342773
node_656: feature_name=GO:1904029 feature_id[208].value <= threshold=26.575013160705566
node_657: feature_name=GO:0090594 feature_id[738].value <= threshold=3.000791072845459
node_658: feature_name=GO:0010948 feature_id[569].value > threshold=0.04386143572628498
node_666: feature_name=GO:0032764 feature_id[3].value <= threshold=2.5201677083969116
node_667: feature_name=GO:0023026 feature_id[69].value > threshold=3.2760006189346313
node_737: feature_name=GO:0033158 feature_id[379].value <= threshold=0.46020837128162384
Class: positive genes

Rules_389 passed counts:2
node_0: feature_name=GO:0042113 feature_id[0].value <= threshold=13.408552169799805
node_1: feature_name=GO:0007568 feature_id[534].value <= threshold=5.0313897132873535
node_2: feature_name=GO:0002705 feature_id[541].value <= threshold=3.200145721435547
node_3: feature_name=GO:1901525 feature_id[576].value > threshold=0.4466460347175598
node_617: feature_name=GO:0005622 feature_id[233].value <= threshold=90.85730743408203
node_618: feature_name=GO:1903147 feature_id[200].value <= threshold=7.326428413391113
node_619: feature_name=GO:0003964 feature_id[317].value <= threshold=5.575642108917236
node_620: feature_name=GO:0046498 feature_id[203].value <= threshold=6.620223522186279
node_621: feature_name=GO:0002562 feature_id[25].value <= threshold=10.287704467773438
node_622: feature_name=GO:0006919 feature_id[246].value <= threshold=5.801334381103516
node_623: feature_name=GO:0001889 feature_id[387].value <= threshold=2.679414987564087
node_624: feature_name=GO:0006555 feature_id[514].value <= threshold=7.819535493850708
node_625: feature_name=GO:0080134 feature_id[650].value <= threshold=38.91967582702637
node_626: feature_name=GO:0042127 feature_id[480].value <= threshold=37.72536659240723
node_627: feature_name=GO:2001251 feature_id[220].value <= threshold=32.564422607421875
node_628: feature_name=GO:0002829 feature_id[493].value <= threshold=2.444612741470337
node_629: feature_name=GO:0008588 feature_id[26].value <= threshold=2.3601274490356445
node_630: feature_name=GO:0033151 feature_id[7].value <= threshold=5.134376287460327
node_631: feature_name=GO:0048145 feature_id[348].value <= threshold=8.836549282073975
node_632: feature_name=GO:0030887 feature_id[274].value <= threshold=1.7000296115875244
node_633: feature_name=GO:0097190 feature_id[117].value <= threshold=33.02078819274902

node_634: feature_name=GO:0046685 feature_id[782].value <= threshold=8.040813446044922
node_635: feature_name=GO:0006346 feature_id[236].value <= threshold=6.384642839431763
node_636: feature_name=GO:0048537 feature_id[66].value <= threshold=3.1739262342453003
node_637: feature_name=GO:0070245 feature_id[326].value <= threshold=3.0491198301315308
node_638: feature_name=GO:0038065 feature_id[670].value <= threshold=2.0590850114822388
node_639: feature_name=GO:0005488 feature_id[187].value <= threshold=84.80076217651367
node_640: feature_name=GO:0060249 feature_id[390].value > threshold=0.07226398587226868
node_652: feature_name=GO:0006974 feature_id[516].value > threshold=5.17782768838515e-06
node_654: feature_name=hsa05210 feature_id[358].value <= threshold=18.730005264282227
node_655: feature_name=GO:0071850 feature_id[40].value <= threshold=5.475317001342773
node_656: feature_name=GO:1904029 feature_id[208].value <= threshold=26.575013160705566
node_657: feature_name=GO:0090594 feature_id[738].value <= threshold=3.000791072845459
node_658: feature_name=GO:0010948 feature_id[569].value > threshold=0.04386143572628498
node_666: feature_name=GO:0032764 feature_id[3].value <= threshold=2.5201677083969116
node_667: feature_name=GO:0023026 feature_id[69].value <= threshold=3.2760006189346313
node_668: feature_name=GO:0032464 feature_id[83].value > threshold=2.5927021503448486
node_734: feature_name=GO:0032069 feature_id[614].value > threshold=0.8012068271636963
Class: negative genes

Rules_390 passed counts:2
node_0: feature_name=GO:0042113 feature_id[0].value <= threshold=13.408552169799805
node_1: feature_name=GO:0007568 feature_id[534].value <= threshold=5.0313897132873535
node_2: feature_name=GO:0002705 feature_id[541].value <= threshold=3.200145721435547
node_3: feature_name=GO:1901525 feature_id[576].value > threshold=0.4466460347175598
node_617: feature_name=GO:0005622 feature_id[233].value <= threshold=90.85730743408203
node_618: feature_name=GO:1903147 feature_id[200].value <= threshold=7.326428413391113
node_619: feature_name=GO:0003964 feature_id[317].value <= threshold=5.575642108917236
node_620: feature_name=GO:0046498 feature_id[203].value <= threshold=6.620223522186279
node_621: feature_name=GO:0002562 feature_id[25].value <= threshold=10.287704467773438
node_622: feature_name=GO:0006919 feature_id[246].value <= threshold=5.801334381103516
node_623: feature_name=GO:0001889 feature_id[387].value <= threshold=2.679414987564087
node_624: feature_name=GO:0006555 feature_id[514].value <= threshold=7.819535493850708
node_625: feature_name=GO:0080134 feature_id[650].value <= threshold=38.91967582702637
node_626: feature_name=GO:0042127 feature_id[480].value <= threshold=37.72536659240723
node_627: feature_name=GO:2001251 feature_id[220].value <= threshold=32.564422607421875

node_628: feature_name=GO:0002829 feature_id[493].value <= threshold=2.444612741470337
node_629: feature_name=GO:0008588 feature_id[26].value <= threshold=2.3601274490356445
node_630: feature_name=GO:0033151 feature_id[7].value <= threshold=5.134376287460327
node_631: feature_name=GO:0048145 feature_id[348].value <= threshold=8.836549282073975
node_632: feature_name=GO:0030887 feature_id[274].value <= threshold=1.7000296115875244
node_633: feature_name=GO:0097190 feature_id[117].value <= threshold=33.02078819274902
node_634: feature_name=GO:0046685 feature_id[782].value <= threshold=8.040813446044922
node_635: feature_name=GO:0006346 feature_id[236].value <= threshold=6.384642839431763
node_636: feature_name=GO:0048537 feature_id[66].value <= threshold=3.1739262342453003
node_637: feature_name=GO:0070245 feature_id[326].value <= threshold=3.0491198301315308
node_638: feature_name=GO:0038065 feature_id[670].value <= threshold=2.0590850114822388
node_639: feature_name=GO:0005488 feature_id[187].value <= threshold=84.80076217651367
node_640: feature_name=GO:0060249 feature_id[390].value > threshold=0.07226398587226868
node_652: feature_name=GO:0006974 feature_id[516].value > threshold=5.17782768838515e-06
node_654: feature_name=hsa05210 feature_id[358].value <= threshold=18.730005264282227
node_655: feature_name=GO:0071850 feature_id[40].value <= threshold=5.475317001342773
node_656: feature_name=GO:1904029 feature_id[208].value <= threshold=26.575013160705566
node_657: feature_name=GO:0090594 feature_id[738].value <= threshold=3.000791072845459
node_658: feature_name=GO:0010948 feature_id[569].value > threshold=0.04386143572628498
node_666: feature_name=GO:0032764 feature_id[3].value <= threshold=2.5201677083969116
node_667: feature_name=GO:0023026 feature_id[69].value <= threshold=3.2760006189346313
node_668: feature_name=GO:0032464 feature_id[83].value <= threshold=2.5927021503448486
node_669: feature_name=GO:0071901 feature_id[407].value > threshold=18.238012313842773
node_731: feature_name=GO:0002821 feature_id[588].value > threshold=0.05757370963692665
Class: negative genes

Rules_391 passed counts:2
node_0: feature_name=GO:0042113 feature_id[0].value <= threshold=13.408552169799805
node_1: feature_name=GO:0007568 feature_id[534].value <= threshold=5.0313897132873535
node_2: feature_name=GO:0002705 feature_id[541].value <= threshold=3.200145721435547
node_3: feature_name=GO:1901525 feature_id[576].value > threshold=0.4466460347175598
node_617: feature_name=GO:0005622 feature_id[233].value <= threshold=90.85730743408203
node_618: feature_name=GO:1903147 feature_id[200].value <= threshold=7.326428413391113
node_619: feature_name=GO:0003964 feature_id[317].value <= threshold=5.575642108917236
node_620: feature_name=GO:0046498 feature_id[203].value <= threshold=6.620223522186279

node_621: feature_name=GO:0002562 feature_id[25].value <= threshold=10.287704467773438
node_622: feature_name=GO:0006919 feature_id[246].value <= threshold=5.801334381103516
node_623: feature_name=GO:0001889 feature_id[387].value <= threshold=2.679414987564087
node_624: feature_name=GO:0006555 feature_id[514].value <= threshold=7.819535493850708
node_625: feature_name=GO:0080134 feature_id[650].value <= threshold=38.91967582702637
node_626: feature_name=GO:0042127 feature_id[480].value <= threshold=37.72536659240723
node_627: feature_name=GO:2001251 feature_id[220].value <= threshold=32.564422607421875
node_628: feature_name=GO:0002829 feature_id[493].value <= threshold=2.444612741470337
node_629: feature_name=GO:0008588 feature_id[26].value <= threshold=2.3601274490356445
node_630: feature_name=GO:0033151 feature_id[7].value <= threshold=5.134376287460327
node_631: feature_name=GO:0048145 feature_id[348].value <= threshold=8.836549282073975
node_632: feature_name=GO:0030887 feature_id[274].value <= threshold=1.7000296115875244
node_633: feature_name=GO:0097190 feature_id[117].value <= threshold=33.02078819274902
node_634: feature_name=GO:0046685 feature_id[782].value <= threshold=8.040813446044922
node_635: feature_name=GO:0006346 feature_id[236].value <= threshold=6.384642839431763
node_636: feature_name=GO:0048537 feature_id[66].value <= threshold=3.1739262342453003
node_637: feature_name=GO:0070245 feature_id[326].value <= threshold=3.0491198301315308
node_638: feature_name=GO:0038065 feature_id[670].value <= threshold=2.0590850114822388
node_639: feature_name=GO:0005488 feature_id[187].value <= threshold=84.80076217651367
node_640: feature_name=GO:0060249 feature_id[390].value > threshold=0.07226398587226868
node_652: feature_name=GO:0006974 feature_id[516].value > threshold=5.17782768838515e-06
node_654: feature_name=hsa05210 feature_id[358].value <= threshold=18.730005264282227
node_655: feature_name=GO:0071850 feature_id[40].value <= threshold=5.475317001342773
node_656: feature_name=GO:1904029 feature_id[208].value <= threshold=26.575013160705566
node_657: feature_name=GO:0090594 feature_id[738].value <= threshold=3.000791072845459
node_658: feature_name=GO:0010948 feature_id[569].value > threshold=0.04386143572628498
node_666: feature_name=GO:0032764 feature_id[3].value <= threshold=2.5201677083969116
node_667: feature_name=GO:0023026 feature_id[69].value <= threshold=3.2760006189346313
node_668: feature_name=GO:0032464 feature_id[83].value <= threshold=2.5927021503448486
node_669: feature_name=GO:0071901 feature_id[407].value <= threshold=18.238012313842773
node_670: feature_name=GO:0015671 feature_id[103].value > threshold=1.3113192915916443
node_722: feature_name=GO:0035067 feature_id[649].value > threshold=2.5819170475006104
Class: positive genes

Rules_392 passed counts:2

node_0: feature_name=GO:0042113	feature_id[0].value <= threshold=13.408552169799805
node_1: feature_name=GO:0007568	feature_id[534].value <= threshold=5.0313897132873535
node_2: feature_name=GO:0002705	feature_id[541].value <= threshold=3.200145721435547
node_3: feature_name=GO:1901525	feature_id[576].value > threshold=0.4466460347175598
node_617: feature_name=GO:0005622	feature_id[233].value <= threshold=90.85730743408203
node_618: feature_name=GO:1903147	feature_id[200].value <= threshold=7.326428413391113
node_619: feature_name=GO:0003964	feature_id[317].value <= threshold=5.575642108917236
node_620: feature_name=GO:0046498	feature_id[203].value <= threshold=6.620223522186279
node_621: feature_name=GO:0002562	feature_id[25].value <= threshold=10.287704467773438
node_622: feature_name=GO:0006919	feature_id[246].value <= threshold=5.801334381103516
node_623: feature_name=GO:0001889	feature_id[387].value <= threshold=2.679414987564087
node_624: feature_name=GO:0006555	feature_id[514].value <= threshold=7.819535493850708
node_625: feature_name=GO:0080134	feature_id[650].value <= threshold=38.91967582702637
node_626: feature_name=GO:0042127	feature_id[480].value <= threshold=37.72536659240723
node_627: feature_name=GO:2001251	feature_id[220].value <= threshold=32.564422607421875
node_628: feature_name=GO:0002829	feature_id[493].value <= threshold=2.444612741470337
node_629: feature_name=GO:0008588	feature_id[26].value <= threshold=2.3601274490356445
node_630: feature_name=GO:0033151	feature_id[7].value <= threshold=5.134376287460327
node_631: feature_name=GO:0048145	feature_id[348].value <= threshold=8.836549282073975
node_632: feature_name=GO:0030887	feature_id[274].value <= threshold=1.7000296115875244
node_633: feature_name=GO:0097190	feature_id[117].value <= threshold=33.02078819274902
node_634: feature_name=GO:0046685	feature_id[782].value <= threshold=8.040813446044922
node_635: feature_name=GO:0006346	feature_id[236].value <= threshold=6.384642839431763
node_636: feature_name=GO:0048537	feature_id[66].value <= threshold=3.1739262342453003
node_637: feature_name=GO:0070245	feature_id[326].value <= threshold=3.0491198301315308
node_638: feature_name=GO:0038065	feature_id[670].value <= threshold=2.0590850114822388
node_639: feature_name=GO:0005488	feature_id[187].value <= threshold=84.80076217651367
node_640: feature_name=GO:0060249	feature_id[390].value > threshold=0.07226398587226868
node_652: feature_name=GO:0006974	feature_id[516].value > threshold=5.17782768838515e-06
node_654: feature_name=hsa05210	feature_id[358].value <= threshold=18.730005264282227
node_655: feature_name=GO:0071850	feature_id[40].value <= threshold=5.475317001342773
node_656: feature_name=GO:1904029	feature_id[208].value <= threshold=26.575013160705566
node_657: feature_name=GO:0090594	feature_id[738].value <= threshold=3.000791072845459
node_658: feature_name=GO:0010948	feature_id[569].value > threshold=0.04386143572628498
node_666: feature_name=GO:0032764	feature_id[3].value <= threshold=2.5201677083969116

node_667: feature_name=GO:0023026 feature_id[69].value <= threshold=3.2760006189346313
node_668: feature_name=GO:0032464 feature_id[83].value <= threshold=2.5927021503448486
node_669: feature_name=GO:0071901 feature_id[407].value <= threshold=18.238012313842773
node_670: feature_name=GO:0015671 feature_id[103].value > threshold=1.3113192915916443
node_722: feature_name=GO:0035067 feature_id[649].value <= threshold=2.5819170475006104
node_723: feature_name=GO:0046677 feature_id[591].value > threshold=2.915908694267273
node_727: feature_name=hsa05223 feature_id[540].value <= threshold=3.5530080795288086
Class: positive genes

Rules_393 passed counts:2
node_0: feature_name=GO:0042113 feature_id[0].value <= threshold=13.408552169799805
node_1: feature_name=GO:0007568 feature_id[534].value <= threshold=5.0313897132873535
node_2: feature_name=GO:0002705 feature_id[541].value <= threshold=3.200145721435547
node_3: feature_name=GO:1901525 feature_id[576].value > threshold=0.4466460347175598
node_617: feature_name=GO:0005622 feature_id[233].value <= threshold=90.85730743408203
node_618: feature_name=GO:1903147 feature_id[200].value <= threshold=7.326428413391113
node_619: feature_name=GO:0003964 feature_id[317].value <= threshold=5.575642108917236
node_620: feature_name=GO:0046498 feature_id[203].value <= threshold=6.620223522186279
node_621: feature_name=GO:0002562 feature_id[25].value <= threshold=10.287704467773438
node_622: feature_name=GO:0006919 feature_id[246].value <= threshold=5.801334381103516
node_623: feature_name=GO:0001889 feature_id[387].value <= threshold=2.679414987564087
node_624: feature_name=GO:0006555 feature_id[514].value <= threshold=7.819535493850708
node_625: feature_name=GO:0080134 feature_id[650].value <= threshold=38.91967582702637
node_626: feature_name=GO:0042127 feature_id[480].value <= threshold=37.72536659240723
node_627: feature_name=GO:2001251 feature_id[220].value <= threshold=32.564422607421875
node_628: feature_name=GO:0002829 feature_id[493].value <= threshold=2.444612741470337
node_629: feature_name=GO:0008588 feature_id[26].value <= threshold=2.3601274490356445
node_630: feature_name=GO:0033151 feature_id[7].value <= threshold=5.134376287460327
node_631: feature_name=GO:0048145 feature_id[348].value <= threshold=8.836549282073975
node_632: feature_name=GO:0030887 feature_id[274].value <= threshold=1.7000296115875244
node_633: feature_name=GO:0097190 feature_id[117].value <= threshold=33.02078819274902
node_634: feature_name=GO:0046685 feature_id[782].value <= threshold=8.040813446044922
node_635: feature_name=GO:0006346 feature_id[236].value <= threshold=6.384642839431763
node_636: feature_name=GO:0048537 feature_id[66].value <= threshold=3.1739262342453003
node_637: feature_name=GO:0070245 feature_id[326].value <= threshold=3.0491198301315308

node_638: feature_name=GO:0038065 feature_id[670].value <= threshold=2.0590850114822388
node_639: feature_name=GO:0005488 feature_id[187].value <= threshold=84.80076217651367
node_640: feature_name=GO:0060249 feature_id[390].value > threshold=0.07226398587226868
node_652: feature_name=GO:0006974 feature_id[516].value > threshold=5.17782768838515e-06
node_654: feature_name=hsa05210 feature_id[358].value <= threshold=18.730005264282227
node_655: feature_name=GO:0071850 feature_id[40].value <= threshold=5.475317001342773
node_656: feature_name=GO:1904029 feature_id[208].value <= threshold=26.575013160705566
node_657: feature_name=GO:0090594 feature_id[738].value <= threshold=3.000791072845459
node_658: feature_name=GO:0010948 feature_id[569].value > threshold=0.04386143572628498
node_666: feature_name=GO:0032764 feature_id[3].value <= threshold=2.5201677083969116
node_667: feature_name=GO:0023026 feature_id[69].value <= threshold=3.2760006189346313
node_668: feature_name=GO:0032464 feature_id[83].value <= threshold=2.5927021503448486
node_669: feature_name=GO:0071901 feature_id[407].value <= threshold=18.238012313842773
node_670: feature_name=GO:0015671 feature_id[103].value <= threshold=1.3113192915916443
node_671: feature_name=GO:0008340 feature_id[539].value > threshold=4.860779762268066
node_719: feature_name=GO:1903037 feature_id[165].value <= threshold=0.4720069617033005
Class: negative genes

Rules_394 passed counts:2
node_0: feature_name=GO:0042113 feature_id[0].value <= threshold=13.408552169799805
node_1: feature_name=GO:0007568 feature_id[534].value <= threshold=5.0313897132873535
node_2: feature_name=GO:0002705 feature_id[541].value <= threshold=3.200145721435547
node_3: feature_name=GO:1901525 feature_id[576].value > threshold=0.4466460347175598
node_617: feature_name=GO:0005622 feature_id[233].value <= threshold=90.85730743408203
node_618: feature_name=GO:1903147 feature_id[200].value <= threshold=7.326428413391113
node_619: feature_name=GO:0003964 feature_id[317].value <= threshold=5.575642108917236
node_620: feature_name=GO:0046498 feature_id[203].value <= threshold=6.620223522186279
node_621: feature_name=GO:0002562 feature_id[25].value <= threshold=10.287704467773438
node_622: feature_name=GO:0006919 feature_id[246].value <= threshold=5.801334381103516
node_623: feature_name=GO:0001889 feature_id[387].value <= threshold=2.679414987564087
node_624: feature_name=GO:0006555 feature_id[514].value <= threshold=7.819535493850708
node_625: feature_name=GO:0080134 feature_id[650].value <= threshold=38.91967582702637
node_626: feature_name=GO:0042127 feature_id[480].value <= threshold=37.72536659240723
node_627: feature_name=GO:2001251 feature_id[220].value <= threshold=32.564422607421875
node_628: feature_name=GO:0002829 feature_id[493].value <= threshold=2.444612741470337

node_629: feature_name=GO:0008588 feature_id[26].value <= threshold=2.3601274490356445
node_630: feature_name=GO:0033151 feature_id[7].value <= threshold=5.134376287460327
node_631: feature_name=GO:0048145 feature_id[348].value <= threshold=8.836549282073975
node_632: feature_name=GO:0030887 feature_id[274].value <= threshold=1.7000296115875244
node_633: feature_name=GO:0097190 feature_id[117].value <= threshold=33.02078819274902
node_634: feature_name=GO:0046685 feature_id[782].value <= threshold=8.040813446044922
node_635: feature_name=GO:0006346 feature_id[236].value <= threshold=6.384642839431763
node_636: feature_name=GO:0048537 feature_id[66].value <= threshold=3.1739262342453003
node_637: feature_name=GO:0070245 feature_id[326].value <= threshold=3.0491198301315308
node_638: feature_name=GO:0038065 feature_id[670].value <= threshold=2.0590850114822388
node_639: feature_name=GO:0005488 feature_id[187].value <= threshold=84.80076217651367
node_640: feature_name=GO:0060249 feature_id[390].value > threshold=0.07226398587226868
node_652: feature_name=GO:0006974 feature_id[516].value > threshold=5.17782768838515e-06
node_654: feature_name=hsa05210 feature_id[358].value <= threshold=18.730005264282227
node_655: feature_name=GO:0071850 feature_id[40].value <= threshold=5.475317001342773
node_656: feature_name=GO:1904029 feature_id[208].value <= threshold=26.575013160705566
node_657: feature_name=GO:0090594 feature_id[738].value <= threshold=3.000791072845459
node_658: feature_name=GO:0010948 feature_id[569].value > threshold=0.04386143572628498
node_666: feature_name=GO:0032764 feature_id[3].value <= threshold=2.5201677083969116
node_667: feature_name=GO:0023026 feature_id[69].value <= threshold=3.2760006189346313
node_668: feature_name=GO:0032464 feature_id[83].value <= threshold=2.5927021503448486
node_669: feature_name=GO:0071901 feature_id[407].value <= threshold=18.238012313842773
node_670: feature_name=GO:0015671 feature_id[103].value <= threshold=1.3113192915916443
node_671: feature_name=GO:0008340 feature_id[539].value <= threshold=4.860779762268066
node_672: feature_name=GO:0046006 feature_id[364].value <= threshold=2.4317692518234253
node_673: feature_name=GO:0042991 feature_id[684].value <= threshold=5.553565502166748
node_674: feature_name=GO:0002327 feature_id[408].value > threshold=2.404940366744995
node_710: feature_name=GO:0010939 feature_id[142].value > threshold=3.3515632152557373
Class: positive genes

Rules_395 passed counts:2
node_0: feature_name=GO:0042113 feature_id[0].value <= threshold=13.408552169799805
node_1: feature_name=GO:0007568 feature_id[534].value <= threshold=5.0313897132873535
node_2: feature_name=GO:0002705 feature_id[541].value <= threshold=3.200145721435547
node_3: feature_name=GO:1901525 feature_id[576].value > threshold=0.4466460347175598

node_617: feature_name=GO:0005622	feature_id[233].value <= threshold=90.85730743408203
node_618: feature_name=GO:1903147	feature_id[200].value <= threshold=7.326428413391113
node_619: feature_name=GO:0003964	feature_id[317].value <= threshold=5.575642108917236
node_620: feature_name=GO:0046498	feature_id[203].value <= threshold=6.620223522186279
node_621: feature_name=GO:0002562	feature_id[25].value <= threshold=10.287704467773438
node_622: feature_name=GO:0006919	feature_id[246].value <= threshold=5.801334381103516
node_623: feature_name=GO:0001889	feature_id[387].value <= threshold=2.679414987564087
node_624: feature_name=GO:0006555	feature_id[514].value <= threshold=7.819535493850708
node_625: feature_name=GO:0080134	feature_id[650].value <= threshold=38.91967582702637
node_626: feature_name=GO:0042127	feature_id[480].value <= threshold=37.72536659240723
node_627: feature_name=GO:2001251	feature_id[220].value <= threshold=32.564422607421875
node_628: feature_name=GO:0002829	feature_id[493].value <= threshold=2.444612741470337
node_629: feature_name=GO:0008588	feature_id[26].value <= threshold=2.3601274490356445
node_630: feature_name=GO:0033151	feature_id[7].value <= threshold=5.134376287460327
node_631: feature_name=GO:0048145	feature_id[348].value <= threshold=8.836549282073975
node_632: feature_name=GO:0030887	feature_id[274].value <= threshold=1.7000296115875244
node_633: feature_name=GO:0097190	feature_id[117].value <= threshold=33.02078819274902
node_634: feature_name=GO:0046685	feature_id[782].value <= threshold=8.040813446044922
node_635: feature_name=GO:0006346	feature_id[236].value <= threshold=6.384642839431763
node_636: feature_name=GO:0048537	feature_id[66].value <= threshold=3.1739262342453003
node_637: feature_name=GO:0070245	feature_id[326].value <= threshold=3.0491198301315308
node_638: feature_name=GO:0038065	feature_id[670].value <= threshold=2.0590850114822388
node_639: feature_name=GO:0005488	feature_id[187].value <= threshold=84.80076217651367
node_640: feature_name=GO:0060249	feature_id[390].value > threshold=0.07226398587226868
node_652: feature_name=GO:0006974	feature_id[516].value > threshold=5.17782768838515e-06
node_654: feature_name=hsa05210	feature_id[358].value <= threshold=18.730005264282227
node_655: feature_name=GO:0071850	feature_id[40].value <= threshold=5.475317001342773
node_656: feature_name=GO:1904029	feature_id[208].value <= threshold=26.575013160705566
node_657: feature_name=GO:0090594	feature_id[738].value <= threshold=3.000791072845459
node_658: feature_name=GO:0010948	feature_id[569].value > threshold=0.04386143572628498
node_666: feature_name=GO:0032764	feature_id[3].value <= threshold=2.5201677083969116
node_667: feature_name=GO:0023026	feature_id[69].value <= threshold=3.2760006189346313
node_668: feature_name=GO:0032464	feature_id[83].value <= threshold=2.5927021503448486
node_669: feature_name=GO:0071901	feature_id[407].value <= threshold=18.238012313842773
node_670: feature_name=GO:0015671	feature_id[103].value <= threshold=1.3113192915916443

node_671: feature_name=GO:0008340	feature_id[539].value <= threshold=4.860779762268066
node_672: feature_name=GO:0046006	feature_id[364].value <= threshold=2.4317692518234253
node_673: feature_name=GO:0042991	feature_id[684].value <= threshold=5.553565502166748
node_674: feature_name=GO:0002327	feature_id[408].value <= threshold=2.404940366744995
node_675: feature_name=GO:0001836	feature_id[450].value <= threshold=8.647934436798096
node_676: feature_name=GO:0048539	feature_id[319].value <= threshold=1.6308802962303162
node_677: feature_name=GO:0022408	feature_id[279].value <= threshold=4.064727783203125
node_678: feature_name=GO:0030099	feature_id[598].value <= threshold=0.0001809061213862151
node_679: feature_name=hsa05219	feature_id[162].value > threshold=3.047200083732605
node_681: feature_name=GO:0050865	feature_id[234].value <= threshold=0.754447378218174
Class: positive genes	
Rules_396	passed counts:2
node_0: feature_name=GO:0042113	feature_id[0].value <= threshold=13.408552169799805
node_1: feature_name=GO:0007568	feature_id[534].value <= threshold=5.0313897132873535
node_2: feature_name=GO:0002705	feature_id[541].value <= threshold=3.200145721435547
node_3: feature_name=GO:1901525	feature_id[576].value > threshold=0.4466460347175598
node_617: feature_name=GO:0005622	feature_id[233].value <= threshold=90.85730743408203
node_618: feature_name=GO:1903147	feature_id[200].value <= threshold=7.326428413391113
node_619: feature_name=GO:0003964	feature_id[317].value <= threshold=5.575642108917236
node_620: feature_name=GO:0046498	feature_id[203].value <= threshold=6.620223522186279
node_621: feature_name=GO:0002562	feature_id[25].value <= threshold=10.287704467773438
node_622: feature_name=GO:0006919	feature_id[246].value <= threshold=5.801334381103516
node_623: feature_name=GO:0001889	feature_id[387].value <= threshold=2.679414987564087
node_624: feature_name=GO:0006555	feature_id[514].value <= threshold=7.819535493850708
node_625: feature_name=GO:0080134	feature_id[650].value <= threshold=38.91967582702637
node_626: feature_name=GO:0042127	feature_id[480].value <= threshold=37.72536659240723
node_627: feature_name=GO:2001251	feature_id[220].value <= threshold=32.564422607421875
node_628: feature_name=GO:0002829	feature_id[493].value <= threshold=2.444612741470337
node_629: feature_name=GO:0008588	feature_id[26].value <= threshold=2.3601274490356445
node_630: feature_name=GO:0033151	feature_id[7].value <= threshold=5.134376287460327
node_631: feature_name=GO:0048145	feature_id[348].value <= threshold=8.836549282073975
node_632: feature_name=GO:0030887	feature_id[274].value <= threshold=1.7000296115875244
node_633: feature_name=GO:0097190	feature_id[117].value <= threshold=33.02078819274902
node_634: feature_name=GO:0046685	feature_id[782].value <= threshold=8.040813446044922

node_635: feature_name=GO:0006346 feature_id[236].value <= threshold=6.384642839431763
node_636: feature_name=GO:0048537 feature_id[66].value <= threshold=3.1739262342453003
node_637: feature_name=GO:0070245 feature_id[326].value <= threshold=3.0491198301315308
node_638: feature_name=GO:0038065 feature_id[670].value <= threshold=2.0590850114822388
node_639: feature_name=GO:0005488 feature_id[187].value <= threshold=84.80076217651367
node_640: feature_name=GO:0060249 feature_id[390].value > threshold=0.07226398587226868
node_652: feature_name=GO:0006974 feature_id[516].value > threshold=5.17782768838515e-06
node_654: feature_name=hsa05210 feature_id[358].value <= threshold=18.730005264282227
node_655: feature_name=GO:0071850 feature_id[40].value <= threshold=5.475317001342773
node_656: feature_name=GO:1904029 feature_id[208].value <= threshold=26.575013160705566
node_657: feature_name=GO:0090594 feature_id[738].value <= threshold=3.000791072845459
node_658: feature_name=GO:0010948 feature_id[569].value <= threshold=0.043861435726284898
node_659: feature_name=GO:0002704 feature_id[470].value > threshold=0.7501857876777649
Class: positive genes

Rules_397 passed counts:2
node_0: feature_name=GO:0042113 feature_id[0].value <= threshold=13.408552169799805
node_1: feature_name=GO:0007568 feature_id[534].value <= threshold=5.0313897132873535
node_2: feature_name=GO:0002705 feature_id[541].value <= threshold=3.200145721435547
node_3: feature_name=GO:1901525 feature_id[576].value > threshold=0.4466460347175598
node_617: feature_name=GO:0005622 feature_id[233].value <= threshold=90.85730743408203
node_618: feature_name=GO:1903147 feature_id[200].value <= threshold=7.326428413391113
node_619: feature_name=GO:0003964 feature_id[317].value <= threshold=5.575642108917236
node_620: feature_name=GO:0046498 feature_id[203].value <= threshold=6.620223522186279
node_621: feature_name=GO:0002562 feature_id[25].value <= threshold=10.287704467773438
node_622: feature_name=GO:0006919 feature_id[246].value <= threshold=5.801334381103516
node_623: feature_name=GO:0001889 feature_id[387].value <= threshold=2.679414987564087
node_624: feature_name=GO:0006555 feature_id[514].value <= threshold=7.819535493850708
node_625: feature_name=GO:0080134 feature_id[650].value <= threshold=38.91967582702637
node_626: feature_name=GO:0042127 feature_id[480].value <= threshold=37.72536659240723
node_627: feature_name=GO:2001251 feature_id[220].value <= threshold=32.564422607421875
node_628: feature_name=GO:0002829 feature_id[493].value <= threshold=2.444612741470337
node_629: feature_name=GO:0008588 feature_id[26].value <= threshold=2.3601274490356445
node_630: feature_name=GO:0033151 feature_id[7].value <= threshold=5.134376287460327
node_631: feature_name=GO:0048145 feature_id[348].value <= threshold=8.836549282073975

node_632: feature_name=GO:0030887 feature_id[274].value <= threshold=1.7000296115875244
node_633: feature_name=GO:0097190 feature_id[117].value <= threshold=33.02078819274902
node_634: feature_name=GO:0046685 feature_id[782].value <= threshold=8.040813446044922
node_635: feature_name=GO:0006346 feature_id[236].value <= threshold=6.384642839431763
node_636: feature_name=GO:0048537 feature_id[66].value <= threshold=3.1739262342453003
node_637: feature_name=GO:0070245 feature_id[326].value <= threshold=3.0491198301315308
node_638: feature_name=GO:0038065 feature_id[670].value <= threshold=2.0590850114822388
node_639: feature_name=GO:0005488 feature_id[187].value <= threshold=84.80076217651367
node_640: feature_name=GO:0060249 feature_id[390].value > threshold=0.07226398587226868
node_652: feature_name=GO:0006974 feature_id[516].value > threshold=5.17782768838515e-06
node_654: feature_name=hsa05210 feature_id[358].value <= threshold=18.730005264282227
node_655: feature_name=GO:0071850 feature_id[40].value <= threshold=5.475317001342773
node_656: feature_name=GO:1904029 feature_id[208].value <= threshold=26.575013160705566
node_657: feature_name=GO:0090594 feature_id[738].value <= threshold=3.000791072845459
node_658: feature_name=GO:0010948 feature_id[569].value <= threshold=0.04386143572628498
node_659: feature_name=GO:0002704 feature_id[470].value <= threshold=0.7501857876777649
node_660: feature_name=GO:0006275 feature_id[80].value > threshold=3.2739195823669434
node_662: feature_name=GO:0042981 feature_id[177].value <= threshold=3.204610586166382
Class: positive genes

Rules_398 passed counts:2
node_0: feature_name=GO:0042113 feature_id[0].value <= threshold=13.408552169799805
node_1: feature_name=GO:0007568 feature_id[534].value <= threshold=5.0313897132873535
node_2: feature_name=GO:0002705 feature_id[541].value <= threshold=3.200145721435547
node_3: feature_name=GO:1901525 feature_id[576].value <= threshold=0.4466460347175598
node_4: feature_name=GO:0048539 feature_id[319].value <= threshold=3.0399646759033203
node_5: feature_name=GO:0001910 feature_id[385].value > threshold=3.7437864542007446
Class: positive genes

Rules_399 passed counts:2
node_0: feature_name=GO:0042113 feature_id[0].value <= threshold=13.408552169799805
node_1: feature_name=GO:0007568 feature_id[534].value <= threshold=5.0313897132873535
node_2: feature_name=GO:0002705 feature_id[541].value <= threshold=3.200145721435547
node_3: feature_name=GO:1901525 feature_id[576].value <= threshold=0.4466460347175598
node_4: feature_name=GO:0048539 feature_id[319].value <= threshold=3.0399646759033203

node_5: feature_name=GO:0001910 feature_id[385].value <= threshold=3.7437864542007446
node_6: feature_name=GO:0043200 feature_id[706].value > threshold=9.307284355163574
node_612: feature_name=GO:0002376 feature_id[277].value <= threshold=1.4492701292037964
Class: negative genes

Rules_400 passed counts:2
node_0: feature_name=GO:0042113 feature_id[0].value <= threshold=13.408552169799805
node_1: feature_name=GO:0007568 feature_id[534].value <= threshold=5.0313897132873535
node_2: feature_name=GO:0002705 feature_id[541].value <= threshold=3.200145721435547
node_3: feature_name=GO:1901525 feature_id[576].value <= threshold=0.4466460347175598
node_4: feature_name=GO:0048539 feature_id[319].value <= threshold=3.0399646759033203
node_5: feature_name=GO:0001910 feature_id[385].value <= threshold=3.7437864542007446
node_6: feature_name=GO:0043200 feature_id[706].value <= threshold=9.307284355163574
node_7: feature_name=GO:0001773 feature_id[308].value <= threshold=4.016931533813477
node_8: feature_name=GO:0090116 feature_id[97].value > threshold=7.99645471572876
node_606: feature_name=GO:0042493 feature_id[149].value > threshold=0.7003275454044342
Class: positive genes

Rules_401 passed counts:2
node_0: feature_name=GO:0042113 feature_id[0].value <= threshold=13.408552169799805
node_1: feature_name=GO:0007568 feature_id[534].value <= threshold=5.0313897132873535
node_2: feature_name=GO:0002705 feature_id[541].value <= threshold=3.200145721435547
node_3: feature_name=GO:1901525 feature_id[576].value <= threshold=0.4466460347175598
node_4: feature_name=GO:0048539 feature_id[319].value <= threshold=3.0399646759033203
node_5: feature_name=GO:0001910 feature_id[385].value <= threshold=3.7437864542007446
node_6: feature_name=GO:0043200 feature_id[706].value <= threshold=9.307284355163574
node_7: feature_name=GO:0001773 feature_id[308].value <= threshold=4.016931533813477
node_8: feature_name=GO:0090116 feature_id[97].value <= threshold=7.99645471572876
node_9: feature_name=GO:0019814 feature_id[189].value > threshold=4.525782108306885
node_603: feature_name=GO:0042802 feature_id[795].value > threshold=0.0685675137792714
Class: positive genes

Rules_402 passed counts:2
node_0: feature_name=GO:0042113 feature_id[0].value <= threshold=13.408552169799805
node_1: feature_name=GO:0007568 feature_id[534].value <= threshold=5.0313897132873535

node_2: feature_name=GO:0002705 feature_id[541].value <= threshold=3.200145721435547
node_3: feature_name=GO:1901525 feature_id[576].value <= threshold=0.4466460347175598
node_4: feature_name=GO:0048539 feature_id[319].value <= threshold=3.0399646759033203
node_5: feature_name=GO:0001910 feature_id[385].value <= threshold=3.7437864542007446
node_6: feature_name=GO:0043200 feature_id[706].value <= threshold=9.307284355163574
node_7: feature_name=GO:0001773 feature_id[308].value <= threshold=4.016931533813477
node_8: feature_name=GO:0090116 feature_id[97].value <= threshold=7.99645471572876
node_9: feature_name=GO:0019814 feature_id[189].value <= threshold=4.525782108306885
node_10: feature_name=GO:1902583 feature_id[215].value > threshold=17.60310649871826
node_600: feature_name=GO:0046634 feature_id[46].value <= threshold=0.2357492558658123
Class: positive genes

Rules_403 passed counts:2
node_0: feature_name=GO:0042113 feature_id[0].value <= threshold=13.408552169799805
node_1: feature_name=GO:0007568 feature_id[534].value <= threshold=5.0313897132873535
node_2: feature_name=GO:0002705 feature_id[541].value <= threshold=3.200145721435547
node_3: feature_name=GO:1901525 feature_id[576].value <= threshold=0.4466460347175598
node_4: feature_name=GO:0048539 feature_id[319].value <= threshold=3.0399646759033203
node_5: feature_name=GO:0001910 feature_id[385].value <= threshold=3.7437864542007446
node_6: feature_name=GO:0043200 feature_id[706].value <= threshold=9.307284355163574
node_7: feature_name=GO:0001773 feature_id[308].value <= threshold=4.016931533813477
node_8: feature_name=GO:0090116 feature_id[97].value <= threshold=7.99645471572876
node_9: feature_name=GO:0019814 feature_id[189].value <= threshold=4.525782108306885
node_10: feature_name=GO:1902583 feature_id[215].value <= threshold=17.60310649871826
node_11: feature_name=GO:0045429 feature_id[737].value > threshold=1.6133361458778381
node_477: feature_name=GO:0036037 feature_id[445].value > threshold=1.7181594371795654
node_595: feature_name=GO:0015671 feature_id[103].value <= threshold=0.0885394886136055
node_596: feature_name=GO:0097193 feature_id[167].value > threshold=2.440826654434204
Class: positive genes

Rules_404 passed counts:2
node_0: feature_name=GO:0042113 feature_id[0].value <= threshold=13.408552169799805
node_1: feature_name=GO:0007568 feature_id[534].value <= threshold=5.0313897132873535
node_2: feature_name=GO:0002705 feature_id[541].value <= threshold=3.200145721435547
node_3: feature_name=GO:1901525 feature_id[576].value <= threshold=0.4466460347175598

node_4: feature_name=GO:0048539 feature_id[319].value <= threshold=3.0399646759033203
node_5: feature_name=GO:0001910 feature_id[385].value <= threshold=3.7437864542007446
node_6: feature_name=GO:0043200 feature_id[706].value <= threshold=9.307284355163574
node_7: feature_name=GO:0001773 feature_id[308].value <= threshold=4.016931533813477
node_8: feature_name=GO:0090116 feature_id[97].value <= threshold=7.99645471572876
node_9: feature_name=GO:0019814 feature_id[189].value <= threshold=4.525782108306885
node_10: feature_name=GO:1902583 feature_id[215].value <= threshold=17.60310649871826
node_11: feature_name=GO:0045429 feature_id[737].value > threshold=1.6133361458778381
node_477: feature_name=GO:0036037 feature_id[445].value <= threshold=1.7181594371795654
node_478: feature_name=GO:0006244 feature_id[503].value <= threshold=0.951388418674469
node_479: feature_name=GO:0032461 feature_id[608].value > threshold=2.6103241443634033
node_585: feature_name=GO:1902532 feature_id[302].value > threshold=1.9927199482917786
Class: positive genes

Rules_405 passed counts:2
node_0: feature_name=GO:0042113 feature_id[0].value <= threshold=13.408552169799805
node_1: feature_name=GO:0007568 feature_id[534].value <= threshold=5.0313897132873535
node_2: feature_name=GO:0002705 feature_id[541].value <= threshold=3.200145721435547
node_3: feature_name=GO:1901525 feature_id[576].value <= threshold=0.4466460347175598
node_4: feature_name=GO:0048539 feature_id[319].value <= threshold=3.0399646759033203
node_5: feature_name=GO:0001910 feature_id[385].value <= threshold=3.7437864542007446
node_6: feature_name=GO:0043200 feature_id[706].value <= threshold=9.307284355163574
node_7: feature_name=GO:0001773 feature_id[308].value <= threshold=4.016931533813477
node_8: feature_name=GO:0090116 feature_id[97].value <= threshold=7.99645471572876
node_9: feature_name=GO:0019814 feature_id[189].value <= threshold=4.525782108306885
node_10: feature_name=GO:1902583 feature_id[215].value <= threshold=17.60310649871826
node_11: feature_name=GO:0045429 feature_id[737].value > threshold=1.6133361458778381
node_477: feature_name=GO:0036037 feature_id[445].value <= threshold=1.7181594371795654
node_478: feature_name=GO:0006244 feature_id[503].value <= threshold=0.951388418674469
node_479: feature_name=GO:0032461 feature_id[608].value <= threshold=2.6103241443634033
node_480: feature_name=GO:0044710 feature_id[719].value > threshold=2.228096718681627e-06
node_494: feature_name=GO:0009314 feature_id[296].value <= threshold=34.93696117401123
node_495: feature_name=GO:0050778 feature_id[802].value <= threshold=39.29364013671875
node_496: feature_name=GO:1903038 feature_id[492].value <= threshold=5.849650859832764
node_497: feature_name=GO:0002699 feature_id[606].value <= threshold=5.5405943393707275

node_498: feature_name=hsa00983 feature_id[181].value <= threshold=25.851045608520508
node_499: feature_name=GO:0002309 feature_id[416].value <= threshold=1.6235689520835876
node_500: feature_name=GO:1901698 feature_id[792].value <= threshold=47.70275688171387
node_501: feature_name=GO:0010216 feature_id[282].value > threshold=1.6849713921546936
node_575: feature_name=GO:0002250 feature_id[417].value > threshold=0.700900137424469
Class: negative genes

Rules_406 passed counts:2
node_0: feature_name=GO:0042113 feature_id[0].value <= threshold=13.408552169799805
node_1: feature_name=GO:0007568 feature_id[534].value <= threshold=5.0313897132873535
node_2: feature_name=GO:0002705 feature_id[541].value <= threshold=3.200145721435547
node_3: feature_name=GO:1901525 feature_id[576].value <= threshold=0.4466460347175598
node_4: feature_name=GO:0048539 feature_id[319].value <= threshold=3.0399646759033203
node_5: feature_name=GO:0001910 feature_id[385].value <= threshold=3.7437864542007446
node_6: feature_name=GO:0043200 feature_id[706].value <= threshold=9.307284355163574
node_7: feature_name=GO:0001773 feature_id[308].value <= threshold=4.016931533813477
node_8: feature_name=GO:0090116 feature_id[97].value <= threshold=7.99645471572876
node_9: feature_name=GO:0019814 feature_id[189].value <= threshold=4.525782108306885
node_10: feature_name=GO:1902583 feature_id[215].value <= threshold=17.60310649871826
node_11: feature_name=GO:0045429 feature_id[737].value > threshold=1.6133361458778381
node_477: feature_name=GO:0036037 feature_id[445].value <= threshold=1.7181594371795654
node_478: feature_name=GO:0006244 feature_id[503].value <= threshold=0.951388418674469
node_479: feature_name=GO:0032461 feature_id[608].value <= threshold=2.6103241443634033
node_480: feature_name=GO:0044710 feature_id[719].value > threshold=2.228096718681627e-06
node_494: feature_name=GO:0009314 feature_id[296].value <= threshold=34.93696117401123
node_495: feature_name=GO:0050778 feature_id[802].value <= threshold=39.29364013671875
node_496: feature_name=GO:1903038 feature_id[492].value <= threshold=5.849650859832764
node_497: feature_name=GO:0002699 feature_id[606].value <= threshold=5.5405943393707275
node_498: feature_name=hsa00983 feature_id[181].value <= threshold=25.851045608520508
node_499: feature_name=GO:0002309 feature_id[416].value <= threshold=1.6235689520835876
node_500: feature_name=GO:1901698 feature_id[792].value <= threshold=47.70275688171387
node_501: feature_name=GO:0010216 feature_id[282].value > threshold=1.6849713921546936
node_575: feature_name=GO:0002250 feature_id[417].value <= threshold=0.700900137424469
Class: positive genes

Rules_407	passed counts:2
node_0: feature_name=GO:0042113	feature_id[0].value <= threshold=13.408552169799805
node_1: feature_name=GO:0007568	feature_id[534].value <= threshold=5.0313897132873535
node_2: feature_name=GO:0002705	feature_id[541].value <= threshold=3.200145721435547
node_3: feature_name=GO:1901525	feature_id[576].value <= threshold=0.4466460347175598
node_4: feature_name=GO:0048539	feature_id[319].value <= threshold=3.0399646759033203
node_5: feature_name=GO:0001910	feature_id[385].value <= threshold=3.7437864542007446
node_6: feature_name=GO:0043200	feature_id[706].value <= threshold=9.307284355163574
node_7: feature_name=GO:0001773	feature_id[308].value <= threshold=4.016931533813477
node_8: feature_name=GO:0090116	feature_id[97].value <= threshold=7.99645471572876
node_9: feature_name=GO:0019814	feature_id[189].value <= threshold=4.525782108306885
node_10: feature_name=GO:1902583	feature_id[215].value <= threshold=17.60310649871826
node_11: feature_name=GO:0045429	feature_id[737].value > threshold=1.6133361458778381
node_477: feature_name=GO:0036037	feature_id[445].value <= threshold=1.7181594371795654
node_478: feature_name=GO:0006244	feature_id[503].value <= threshold=0.951388418674469
node_479: feature_name=GO:0032461	feature_id[608].value <= threshold=2.6103241443634033
node_480: feature_name=GO:0044710	feature_id[719].value > threshold=2.228096718681627e-06
node_494: feature_name=GO:0009314	feature_id[296].value <= threshold=34.93696117401123
node_495: feature_name=GO:0050778	feature_id[802].value <= threshold=39.29364013671875
node_496: feature_name=GO:1903038	feature_id[492].value <= threshold=5.849650859832764
node_497: feature_name=GO:0002699	feature_id[606].value <= threshold=5.5405943393707275
node_498: feature_name=hsa00983	feature_id[181].value <= threshold=25.851045608520508
node_499: feature_name=GO:0002309	feature_id[416].value <= threshold=1.6235689520835876
node_500: feature_name=GO:1901698	feature_id[792].value <= threshold=47.70275688171387
node_501: feature_name=GO:0010216	feature_id[282].value <= threshold=1.6849713921546936
node_502: feature_name=GO:0006266	feature_id[297].value > threshold=2.7199249267578125
node_572: feature_name=GO:0060255	feature_id[216].value > threshold=2.504572629928589
Class: positive genes	

Rules_408	passed counts:2
node_0: feature_name=GO:0042113	feature_id[0].value <= threshold=13.408552169799805
node_1: feature_name=GO:0007568	feature_id[534].value <= threshold=5.0313897132873535
node_2: feature_name=GO:0002705	feature_id[541].value <= threshold=3.200145721435547
node_3: feature_name=GO:1901525	feature_id[576].value <= threshold=0.4466460347175598
node_4: feature_name=GO:0048539	feature_id[319].value <= threshold=3.0399646759033203

node_5: feature_name=GO:0001910 feature_id[385].value <= threshold=3.7437864542007446
node_6: feature_name=GO:0043200 feature_id[706].value <= threshold=9.307284355163574
node_7: feature_name=GO:0001773 feature_id[308].value <= threshold=4.016931533813477
node_8: feature_name=GO:0090116 feature_id[97].value <= threshold=7.99645471572876
node_9: feature_name=GO:0019814 feature_id[189].value <= threshold=4.525782108306885
node_10: feature_name=GO:1902583 feature_id[215].value <= threshold=17.60310649871826
node_11: feature_name=GO:0045429 feature_id[737].value > threshold=1.6133361458778381
node_477: feature_name=GO:0036037 feature_id[445].value <= threshold=1.7181594371795654
node_478: feature_name=GO:0006244 feature_id[503].value <= threshold=0.951388418674469
node_479: feature_name=GO:0032461 feature_id[608].value <= threshold=2.6103241443634033
node_480: feature_name=GO:0044710 feature_id[719].value > threshold=2.228096718681627e-06
node_494: feature_name=GO:0009314 feature_id[296].value <= threshold=34.93696117401123
node_495: feature_name=GO:0050778 feature_id[802].value <= threshold=39.29364013671875
node_496: feature_name=GO:1903038 feature_id[492].value <= threshold=5.849650859832764
node_497: feature_name=GO:0002699 feature_id[606].value <= threshold=5.5405943393707275
node_498: feature_name=hsa00983 feature_id[181].value <= threshold=25.851045608520508
node_499: feature_name=GO:0002309 feature_id[416].value <= threshold=1.6235689520835876
node_500: feature_name=GO:1901698 feature_id[792].value <= threshold=47.70275688171387
node_501: feature_name=GO:0010216 feature_id[282].value <= threshold=1.6849713921546936
node_502: feature_name=GO:0006266 feature_id[297].value <= threshold=2.7199249267578125
node_503: feature_name=GO:0005488 feature_id[187].value > threshold=3.0373169010999845e-06
node_507: feature_name=GO:0045429 feature_id[737].value > threshold=1.614579975605011
node_511: feature_name=GO:0034103 feature_id[318].value <= threshold=5.832815647125244
node_512: feature_name=GO:0010663 feature_id[561].value <= threshold=4.157005429267883
node_513: feature_name=GO:0030887 feature_id[274].value <= threshold=1.8964150547981262
node_514: feature_name=GO:0048294 feature_id[113].value <= threshold=1.866134524345398
node_515: feature_name=GO:0072539 feature_id[68].value > threshold=0.8600535988807678
node_547: feature_name=GO:0002718 feature_id[481].value > threshold=4.013465642929077
node_557: feature_name=GO:0045471 feature_id[354].value <= threshold=1.8513423800468445
Class: positive genes

Rules_409 passed counts:2
node_0: feature_name=GO:0042113 feature_id[0].value <= threshold=13.408552169799805
node_1: feature_name=GO:0007568 feature_id[534].value <= threshold=5.0313897132873535
node_2: feature_name=GO:0002705 feature_id[541].value <= threshold=3.200145721435547

node_3: feature_name=GO:1901525	feature_id[576].value <= threshold=0.4466460347175598
node_4: feature_name=GO:0048539	feature_id[319].value <= threshold=3.0399646759033203
node_5: feature_name=GO:0001910	feature_id[385].value <= threshold=3.7437864542007446
node_6: feature_name=GO:0043200	feature_id[706].value <= threshold=9.307284355163574
node_7: feature_name=GO:0001773	feature_id[308].value <= threshold=4.016931533813477
node_8: feature_name=GO:0090116	feature_id[97].value <= threshold=7.99645471572876
node_9: feature_name=GO:0019814	feature_id[189].value <= threshold=4.525782108306885
node_10: feature_name=GO:1902583	feature_id[215].value <= threshold=17.60310649871826
node_11: feature_name=GO:0045429	feature_id[737].value > threshold=1.6133361458778381
node_477: feature_name=GO:0036037	feature_id[445].value <= threshold=1.7181594371795654
node_478: feature_name=GO:0006244	feature_id[503].value <= threshold=0.951388418674469
node_479: feature_name=GO:0032461	feature_id[608].value <= threshold=2.6103241443634033
node_480: feature_name=GO:0044710	feature_id[719].value > threshold=2.228096718681627e-06
node_494: feature_name=GO:0009314	feature_id[296].value <= threshold=34.93696117401123
node_495: feature_name=GO:0050778	feature_id[802].value <= threshold=39.29364013671875
node_496: feature_name=GO:1903038	feature_id[492].value <= threshold=5.849650859832764
node_497: feature_name=GO:0002699	feature_id[606].value <= threshold=5.5405943393707275
node_498: feature_name=hsa00983	feature_id[181].value <= threshold=25.851045608520508
node_499: feature_name=GO:0002309	feature_id[416].value <= threshold=1.6235689520835876
node_500: feature_name=GO:1901698	feature_id[792].value <= threshold=47.70275688171387
node_501: feature_name=GO:0010216	feature_id[282].value <= threshold=1.6849713921546936
node_502: feature_name=GO:0006266	feature_id[297].value <= threshold=2.7199249267578125
node_503: feature_name=GO:0005488	feature_id[187].value > threshold=3.0373169010999845e-06
node_507: feature_name=GO:0045429	feature_id[737].value > threshold=1.614579975605011
node_511: feature_name=GO:0034103	feature_id[318].value <= threshold=5.832815647125244
node_512: feature_name=GO:0010663	feature_id[561].value <= threshold=4.157005429267883
node_513: feature_name=GO:0030887	feature_id[274].value <= threshold=1.8964150547981262
node_514: feature_name=GO:0048294	feature_id[113].value <= threshold=1.866134524345398
node_515: feature_name=GO:0072539	feature_id[68].value <= threshold=0.8600535988807678
node_516: feature_name=GO:0045840	feature_id[697].value > threshold=4.963376045227051
node_544: feature_name=GO:0030888	feature_id[120].value > threshold=0.6045695245265961
Class: positive genes	

Rules_410	passed counts:2
node_0: feature_name=GO:0042113	feature_id[0].value <= threshold=13.408552169799805

node_1: feature_name=GO:0007568	feature_id[534].value <= threshold=5.0313897132873535
node_2: feature_name=GO:0002705	feature_id[541].value <= threshold=3.200145721435547
node_3: feature_name=GO:1901525	feature_id[576].value <= threshold=0.4466460347175598
node_4: feature_name=GO:0048539	feature_id[319].value <= threshold=3.0399646759033203
node_5: feature_name=GO:0001910	feature_id[385].value <= threshold=3.7437864542007446
node_6: feature_name=GO:0043200	feature_id[706].value <= threshold=9.307284355163574
node_7: feature_name=GO:0001773	feature_id[308].value <= threshold=4.016931533813477
node_8: feature_name=GO:0090116	feature_id[97].value <= threshold=7.99645471572876
node_9: feature_name=GO:0019814	feature_id[189].value <= threshold=4.525782108306885
node_10: feature_name=GO:1902583	feature_id[215].value <= threshold=17.60310649871826
node_11: feature_name=GO:0045429	feature_id[737].value > threshold=1.6133361458778381
node_477: feature_name=GO:0036037	feature_id[445].value <= threshold=1.7181594371795654
node_478: feature_name=GO:0006244	feature_id[503].value <= threshold=0.951388418674469
node_479: feature_name=GO:0032461	feature_id[608].value <= threshold=2.6103241443634033
node_480: feature_name=GO:0044710	feature_id[719].value > threshold=2.228096718681627e-06
node_494: feature_name=GO:0009314	feature_id[296].value <= threshold=34.93696117401123
node_495: feature_name=GO:0050778	feature_id[802].value <= threshold=39.29364013671875
node_496: feature_name=GO:1903038	feature_id[492].value <= threshold=5.849650859832764
node_497: feature_name=GO:0002699	feature_id[606].value <= threshold=5.5405943393707275
node_498: feature_name=hsa00983	feature_id[181].value <= threshold=25.851045608520508
node_499: feature_name=GO:0002309	feature_id[416].value <= threshold=1.6235689520835876
node_500: feature_name=GO:1901698	feature_id[792].value <= threshold=47.70275688171387
node_501: feature_name=GO:0010216	feature_id[282].value <= threshold=1.6849713921546936
node_502: feature_name=GO:0006266	feature_id[297].value <= threshold=2.7199249267578125
node_503: feature_name=GO:0005488	feature_id[187].value > threshold=3.0373169010999845e-06
node_507: feature_name=GO:0045429	feature_id[737].value > threshold=1.614579975605011
node_511: feature_name=GO:0034103	feature_id[318].value <= threshold=5.832815647125244
node_512: feature_name=GO:0010663	feature_id[561].value <= threshold=4.157005429267883
node_513: feature_name=GO:0030887	feature_id[274].value <= threshold=1.8964150547981262
node_514: feature_name=GO:0048294	feature_id[113].value <= threshold=1.866134524345398
node_515: feature_name=GO:0072539	feature_id[68].value <= threshold=0.8600535988807678
node_516: feature_name=GO:0045840	feature_id[697].value <= threshold=4.963376045227051
node_517: feature_name=GO:0019222	feature_id[129].value > threshold=4.0122095015249215e-06
node_523: feature_name=GO:0008150	feature_id[528].value > threshold=40.503862380981445
node_541: feature_name=GO:0042094	feature_id[677].value <= threshold=0.17812439054250717

Class: negative genes

Rules_411	passed counts:2
node_0: feature_name=GO:0042113	feature_id[0].value <= threshold=13.408552169799805
node_1: feature_name=GO:0007568	feature_id[534].value <= threshold=5.0313897132873535
node_2: feature_name=GO:0002705	feature_id[541].value <= threshold=3.200145721435547
node_3: feature_name=GO:1901525	feature_id[576].value <= threshold=0.4466460347175598
node_4: feature_name=GO:0048539	feature_id[319].value <= threshold=3.0399646759033203
node_5: feature_name=GO:0001910	feature_id[385].value <= threshold=3.7437864542007446
node_6: feature_name=GO:0043200	feature_id[706].value <= threshold=9.307284355163574
node_7: feature_name=GO:0001773	feature_id[308].value <= threshold=4.016931533813477
node_8: feature_name=GO:0090116	feature_id[97].value <= threshold=7.99645471572876
node_9: feature_name=GO:0019814	feature_id[189].value <= threshold=4.525782108306885
node_10: feature_name=GO:1902583	feature_id[215].value <= threshold=17.60310649871826
node_11: feature_name=GO:0045429	feature_id[737].value > threshold=1.6133361458778381
node_477: feature_name=GO:0036037	feature_id[445].value <= threshold=1.7181594371795654
node_478: feature_name=GO:0006244	feature_id[503].value <= threshold=0.951388418674469
node_479: feature_name=GO:0032461	feature_id[608].value <= threshold=2.6103241443634033
node_480: feature_name=GO:0044710	feature_id[719].value > threshold=2.228096718681627e-06
node_494: feature_name=GO:0009314	feature_id[296].value <= threshold=34.93696117401123
node_495: feature_name=GO:0050778	feature_id[802].value <= threshold=39.29364013671875
node_496: feature_name=GO:1903038	feature_id[492].value <= threshold=5.849650859832764
node_497: feature_name=GO:0002699	feature_id[606].value <= threshold=5.5405943393707275
node_498: feature_name=hsa00983	feature_id[181].value <= threshold=25.851045608520508
node_499: feature_name=GO:0002309	feature_id[416].value <= threshold=1.6235689520835876
node_500: feature_name=GO:1901698	feature_id[792].value <= threshold=47.70275688171387
node_501: feature_name=GO:0010216	feature_id[282].value <= threshold=1.6849713921546936
node_502: feature_name=GO:0006266	feature_id[297].value <= threshold=2.7199249267578125
node_503: feature_name=GO:0005488	feature_id[187].value <= threshold=3.0373169010999845e-06
node_504: feature_name=GO:0002377	feature_id[322].value > threshold=0.056253451853990555

Class: positive genes

Rules_412	passed counts:2
node_0: feature_name=GO:0042113	feature_id[0].value <= threshold=13.408552169799805
node_1: feature_name=GO:0007568	feature_id[534].value <= threshold=5.0313897132873535

node_2: feature_name=GO:0002705 feature_id[541].value <= threshold=3.200145721435547
node_3: feature_name=GO:1901525 feature_id[576].value <= threshold=0.4466460347175598
node_4: feature_name=GO:0048539 feature_id[319].value <= threshold=3.0399646759033203
node_5: feature_name=GO:0001910 feature_id[385].value <= threshold=3.7437864542007446
node_6: feature_name=GO:0043200 feature_id[706].value <= threshold=9.307284355163574
node_7: feature_name=GO:0001773 feature_id[308].value <= threshold=4.016931533813477
node_8: feature_name=GO:0090116 feature_id[97].value <= threshold=7.99645471572876
node_9: feature_name=GO:0019814 feature_id[189].value <= threshold=4.525782108306885
node_10: feature_name=GO:1902583 feature_id[215].value <= threshold=17.60310649871826
node_11: feature_name=GO:0045429 feature_id[737].value <= threshold=1.6133361458778381
node_12: feature_name=GO:0003720 feature_id[228].value > threshold=5.519326210021973
node_474: feature_name=GO:0043367 feature_id[253].value <= threshold=0.4537459146231413
Class: positive genes

Rules_413 passed counts:2
node_0: feature_name=GO:0042113 feature_id[0].value <= threshold=13.408552169799805
node_1: feature_name=GO:0007568 feature_id[534].value <= threshold=5.0313897132873535
node_2: feature_name=GO:0002705 feature_id[541].value <= threshold=3.200145721435547
node_3: feature_name=GO:1901525 feature_id[576].value <= threshold=0.4466460347175598
node_4: feature_name=GO:0048539 feature_id[319].value <= threshold=3.0399646759033203
node_5: feature_name=GO:0001910 feature_id[385].value <= threshold=3.7437864542007446
node_6: feature_name=GO:0043200 feature_id[706].value <= threshold=9.307284355163574
node_7: feature_name=GO:0001773 feature_id[308].value <= threshold=4.016931533813477
node_8: feature_name=GO:0090116 feature_id[97].value <= threshold=7.99645471572876
node_9: feature_name=GO:0019814 feature_id[189].value <= threshold=4.525782108306885
node_10: feature_name=GO:1902583 feature_id[215].value <= threshold=17.60310649871826
node_11: feature_name=GO:0045429 feature_id[737].value <= threshold=1.6133361458778381
node_12: feature_name=GO:0003720 feature_id[228].value <= threshold=5.519326210021973
node_13: feature_name=GO:0046006 feature_id[364].value <= threshold=4.9985432624816895
node_14: feature_name=GO:0070424 feature_id[254].value <= threshold=6.233297109603882
node_15: feature_name=GO:0009892 feature_id[320].value <= threshold=59.99180793762207
node_16: feature_name=GO:0007064 feature_id[527].value <= threshold=11.57377815246582
node_17: feature_name=GO:0005575 feature_id[17].value <= threshold=15.417460918426514
node_18: feature_name=GO:0043368 feature_id[31].value <= threshold=4.059496641159058
node_19: feature_name=GO:0005164 feature_id[163].value <= threshold=5.793607711791992

node_20: feature_name=GO:0042130 feature_id[16].value > threshold=5.073179721832275
node_464: feature_name=GO:0002562 feature_id[25].value > threshold=0.7275692522525787
Class: negative genes

Rules_414 passed counts:2
node_0: feature_name=GO:0042113 feature_id[0].value <= threshold=13.408552169799805
node_1: feature_name=GO:0007568 feature_id[534].value <= threshold=5.0313897132873535
node_2: feature_name=GO:0002705 feature_id[541].value <= threshold=3.200145721435547
node_3: feature_name=GO:1901525 feature_id[576].value <= threshold=0.4466460347175598
node_4: feature_name=GO:0048539 feature_id[319].value <= threshold=3.0399646759033203
node_5: feature_name=GO:0001910 feature_id[385].value <= threshold=3.7437864542007446
node_6: feature_name=GO:0043200 feature_id[706].value <= threshold=9.307284355163574
node_7: feature_name=GO:0001773 feature_id[308].value <= threshold=4.016931533813477
node_8: feature_name=GO:0090116 feature_id[97].value <= threshold=7.99645471572876
node_9: feature_name=GO:0019814 feature_id[189].value <= threshold=4.525782108306885
node_10: feature_name=GO:1902583 feature_id[215].value <= threshold=17.60310649871826
node_11: feature_name=GO:0045429 feature_id[737].value <= threshold=1.6133361458778381
node_12: feature_name=GO:0003720 feature_id[228].value <= threshold=5.519326210021973
node_13: feature_name=GO:0046006 feature_id[364].value <= threshold=4.9985432624816895
node_14: feature_name=GO:0070424 feature_id[254].value <= threshold=6.233297109603882
node_15: feature_name=GO:0009892 feature_id[320].value <= threshold=59.99180793762207
node_16: feature_name=GO:0007064 feature_id[527].value <= threshold=11.57377815246582
node_17: feature_name=GO:0005575 feature_id[17].value <= threshold=15.417460918426514
node_18: feature_name=GO:0043368 feature_id[31].value <= threshold=4.059496641159058
node_19: feature_name=GO:0005164 feature_id[163].value <= threshold=5.793607711791992
node_20: feature_name=GO:0042130 feature_id[16].value > threshold=5.073179721832275
node_464: feature_name=GO:0002562 feature_id[25].value <= threshold=0.7275692522525787
Class: positive genes

Rules_415 passed counts:2
node_0: feature_name=GO:0042113 feature_id[0].value <= threshold=13.408552169799805
node_1: feature_name=GO:0007568 feature_id[534].value <= threshold=5.0313897132873535
node_2: feature_name=GO:0002705 feature_id[541].value <= threshold=3.200145721435547
node_3: feature_name=GO:1901525 feature_id[576].value <= threshold=0.4466460347175598
node_4: feature_name=GO:0048539 feature_id[319].value <= threshold=3.0399646759033203

node_5: feature_name=GO:0001910	feature_id[385].value <= threshold=3.7437864542007446
node_6: feature_name=GO:0043200	feature_id[706].value <= threshold=9.307284355163574
node_7: feature_name=GO:0001773	feature_id[308].value <= threshold=4.016931533813477
node_8: feature_name=GO:0090116	feature_id[97].value <= threshold=7.99645471572876
node_9: feature_name=GO:0019814	feature_id[189].value <= threshold=4.525782108306885
node_10: feature_name=GO:1902583	feature_id[215].value <= threshold=17.60310649871826
node_11: feature_name=GO:0045429	feature_id[737].value <= threshold=1.6133361458778381
node_12: feature_name=GO:0003720	feature_id[228].value <= threshold=5.519326210021973
node_13: feature_name=GO:0046006	feature_id[364].value <= threshold=4.9985432624816895
node_14: feature_name=GO:0070424	feature_id[254].value <= threshold=6.233297109603882
node_15: feature_name=GO:0009892	feature_id[320].value <= threshold=59.99180793762207
node_16: feature_name=GO:0007064	feature_id[527].value <= threshold=11.57377815246582
node_17: feature_name=GO:0005575	feature_id[17].value <= threshold=15.417460918426514
node_18: feature_name=GO:0043368	feature_id[31].value <= threshold=4.059496641159058
node_19: feature_name=GO:0005164	feature_id[163].value <= threshold=5.793607711791992
node_20: feature_name=GO:0042130	feature_id[16].value <= threshold=5.073179721832275
node_21: feature_name=GO:0010216	feature_id[282].value <= threshold=4.834584474563599
node_22: feature_name=GO:0009628	feature_id[553].value <= threshold=29.314892768859863
node_23: feature_name=GO:0045628	feature_id[749].value <= threshold=3.1001367568969727
node_24: feature_name=GO:0042288	feature_id[100].value <= threshold=5.174932956695557
node_25: feature_name=GO:0002329	feature_id[406].value <= threshold=2.348930835723877
node_26: feature_name=GO:0051246	feature_id[642].value > threshold=0.6498909294605255
node_194: feature_name=GO:0009164	feature_id[546].value <= threshold=17.11970329284668
node_195: feature_name=GO:0001909	feature_id[386].value <= threshold=7.292566299438477
node_196: feature_name=GO:0003908	feature_id[11].value <= threshold=1.887226164340973
node_197: feature_name=GO:0009164	feature_id[546].value > threshold=0.26642198860645294
node_359: feature_name=GO:0009164	feature_id[546].value > threshold=0.2696942090988159
node_361: feature_name=hsa05202	feature_id[50].value <= threshold=14.989679336547852
node_362: feature_name=GO:0001775	feature_id[505].value <= threshold=15.726949691772461
node_363: feature_name=GO:0002634	feature_id[449].value <= threshold=2.825531005859375
node_364: feature_name=GO:0010038	feature_id[457].value <= threshold=9.901018619537354
node_365: feature_name=GO:0044238	feature_id[295].value <= threshold=139.80535888671875
node_366: feature_name=GO:0051246	feature_id[642].value > threshold=0.6527281999588013
node_368: feature_name=GO:0044092	feature_id[432].value <= threshold=11.084641933441162
node_369: feature_name=GO:0042162	feature_id[355].value <= threshold=9.683155536651611

node_370: feature_name=GO:0045945 feature_id[267].value <= threshold=3.6181851625442505
node_371: feature_name=GO:0036296 feature_id[252].value <= threshold=3.214281916618347
node_372: feature_name=GO:0097506 feature_id[51].value > threshold=2.9644681215286255
node_430: feature_name=GO:0016444 feature_id[53].value <= threshold=0.6698829531669617
Class: positive genes

Rules_416 passed counts:2
node_0: feature_name=GO:0042113 feature_id[0].value <= threshold=13.408552169799805
node_1: feature_name=GO:0007568 feature_id[534].value <= threshold=5.0313897132873535
node_2: feature_name=GO:0002705 feature_id[541].value <= threshold=3.200145721435547
node_3: feature_name=GO:1901525 feature_id[576].value <= threshold=0.4466460347175598
node_4: feature_name=GO:0048539 feature_id[319].value <= threshold=3.0399646759033203
node_5: feature_name=GO:0001910 feature_id[385].value <= threshold=3.7437864542007446
node_6: feature_name=GO:0043200 feature_id[706].value <= threshold=9.307284355163574
node_7: feature_name=GO:0001773 feature_id[308].value <= threshold=4.016931533813477
node_8: feature_name=GO:0090116 feature_id[97].value <= threshold=7.99645471572876
node_9: feature_name=GO:0019814 feature_id[189].value <= threshold=4.525782108306885
node_10: feature_name=GO:1902583 feature_id[215].value <= threshold=17.60310649871826
node_11: feature_name=GO:0045429 feature_id[737].value <= threshold=1.6133361458778381
node_12: feature_name=GO:0003720 feature_id[228].value <= threshold=5.519326210021973
node_13: feature_name=GO:0046006 feature_id[364].value <= threshold=4.9985432624816895
node_14: feature_name=GO:0070424 feature_id[254].value <= threshold=6.233297109603882
node_15: feature_name=GO:0009892 feature_id[320].value <= threshold=59.99180793762207
node_16: feature_name=GO:0007064 feature_id[527].value <= threshold=11.57377815246582
node_17: feature_name=GO:0005575 feature_id[17].value <= threshold=15.417460918426514
node_18: feature_name=GO:0043368 feature_id[31].value <= threshold=4.059496641159058
node_19: feature_name=GO:0005164 feature_id[163].value <= threshold=5.793607711791992
node_20: feature_name=GO:0042130 feature_id[16].value <= threshold=5.073179721832275
node_21: feature_name=GO:0010216 feature_id[282].value <= threshold=4.834584474563599
node_22: feature_name=GO:0009628 feature_id[553].value <= threshold=29.314892768859863
node_23: feature_name=GO:0045628 feature_id[749].value <= threshold=3.1001367568969727
node_24: feature_name=GO:0042288 feature_id[100].value <= threshold=5.174932956695557
node_25: feature_name=GO:0002329 feature_id[406].value <= threshold=2.348930835723877
node_26: feature_name=GO:0051246 feature_id[642].value > threshold=0.6498909294605255
node_194: feature_name=GO:0009164 feature_id[546].value <= threshold=17.11970329284668

node_195: feature_name=GO:0001909 feature_id[386].value <= threshold=7.292566299438477
node_196: feature_name=GO:0003908 feature_id[11].value <= threshold=1.887226164340973
node_197: feature_name=GO:0009164 feature_id[546].value > threshold=0.26642198860645294
node_359: feature_name=GO:0009164 feature_id[546].value > threshold=0.2696942090988159
node_361: feature_name=hsa05202 feature_id[50].value <= threshold=14.989679336547852
node_362: feature_name=GO:0001775 feature_id[505].value <= threshold=15.726949691772461
node_363: feature_name=GO:0002634 feature_id[449].value <= threshold=2.825531005859375
node_364: feature_name=GO:0010038 feature_id[457].value <= threshold=9.901018619537354
node_365: feature_name=GO:0044238 feature_id[295].value <= threshold=139.80535888671875
node_366: feature_name=GO:0051246 feature_id[642].value > threshold=0.6527281999588013
node_368: feature_name=GO:0044092 feature_id[432].value <= threshold=11.084641933441162
node_369: feature_name=GO:0042162 feature_id[355].value <= threshold=9.683155536651611
node_370: feature_name=GO:0045945 feature_id[267].value <= threshold=3.6181851625442505
node_371: feature_name=GO:0036296 feature_id[252].value <= threshold=3.214281916618347
node_372: feature_name=GO:0097506 feature_id[51].value <= threshold=2.9644681215286255
node_373: feature_name=GO:0019740 feature_id[581].value > threshold=2.2853575944900513
node_427: feature_name=hsa04933 feature_id[744].value <= threshold=0.9997820556163788
Class: positive genes

Rules_417 passed counts:2
node_0: feature_name=GO:0042113 feature_id[0].value <= threshold=13.408552169799805
node_1: feature_name=GO:0007568 feature_id[534].value <= threshold=5.0313897132873535
node_2: feature_name=GO:0002705 feature_id[541].value <= threshold=3.200145721435547
node_3: feature_name=GO:1901525 feature_id[576].value <= threshold=0.4466460347175598
node_4: feature_name=GO:0048539 feature_id[319].value <= threshold=3.0399646759033203
node_5: feature_name=GO:0001910 feature_id[385].value <= threshold=3.7437864542007446
node_6: feature_name=GO:0043200 feature_id[706].value <= threshold=9.307284355163574
node_7: feature_name=GO:0001773 feature_id[308].value <= threshold=4.016931533813477
node_8: feature_name=GO:0090116 feature_id[97].value <= threshold=7.99645471572876
node_9: feature_name=GO:0019814 feature_id[189].value <= threshold=4.525782108306885
node_10: feature_name=GO:1902583 feature_id[215].value <= threshold=17.60310649871826
node_11: feature_name=GO:0045429 feature_id[737].value <= threshold=1.6133361458778381
node_12: feature_name=GO:0003720 feature_id[228].value <= threshold=5.519326210021973
node_13: feature_name=GO:0046006 feature_id[364].value <= threshold=4.9985432624816895
node_14: feature_name=GO:0070424 feature_id[254].value <= threshold=6.233297109603882

node_15: feature_name=GO:0009892	feature_id[320].value <= threshold=59.99180793762207
node_16: feature_name=GO:0007064	feature_id[527].value <= threshold=11.57377815246582
node_17: feature_name=GO:0005575	feature_id[17].value <= threshold=15.417460918426514
node_18: feature_name=GO:0043368	feature_id[31].value <= threshold=4.059496641159058
node_19: feature_name=GO:0005164	feature_id[163].value <= threshold=5.793607711791992
node_20: feature_name=GO:0042130	feature_id[16].value <= threshold=5.073179721832275
node_21: feature_name=GO:0010216	feature_id[282].value <= threshold=4.834584474563599
node_22: feature_name=GO:0009628	feature_id[553].value <= threshold=29.314892768859863
node_23: feature_name=GO:0045628	feature_id[749].value <= threshold=3.1001367568969727
node_24: feature_name=GO:0042288	feature_id[100].value <= threshold=5.174932956695557
node_25: feature_name=GO:0002329	feature_id[406].value <= threshold=2.348930835723877
node_26: feature_name=GO:0051246	feature_id[642].value > threshold=0.6498909294605255
node_194: feature_name=GO:0009164	feature_id[546].value <= threshold=17.11970329284668
node_195: feature_name=GO:0001909	feature_id[386].value <= threshold=7.292566299438477
node_196: feature_name=GO:0003908	feature_id[11].value <= threshold=1.887226164340973
node_197: feature_name=GO:0009164	feature_id[546].value > threshold=0.26642198860645294
node_359: feature_name=GO:0009164	feature_id[546].value > threshold=0.2696942090988159
node_361: feature_name=hsa05202	feature_id[50].value <= threshold=14.989679336547852
node_362: feature_name=GO:0001775	feature_id[505].value <= threshold=15.726949691772461
node_363: feature_name=GO:0002634	feature_id[449].value <= threshold=2.825531005859375
node_364: feature_name=GO:0010038	feature_id[457].value <= threshold=9.901018619537354
node_365: feature_name=GO:0044238	feature_id[295].value <= threshold=139.80535888671875
node_366: feature_name=GO:0051246	feature_id[642].value > threshold=0.6527281999588013
node_368: feature_name=GO:0044092	feature_id[432].value <= threshold=11.084641933441162
node_369: feature_name=GO:0042162	feature_id[355].value <= threshold=9.683155536651611
node_370: feature_name=GO:0045945	feature_id[267].value <= threshold=3.6181851625442505
node_371: feature_name=GO:0036296	feature_id[252].value <= threshold=3.214281916618347
node_372: feature_name=GO:0097506	feature_id[51].value <= threshold=2.9644681215286255
node_373: feature_name=GO:0019740	feature_id[581].value <= threshold=2.2853575944900513
node_374: feature_name=GO:0019660	feature_id[70].value <= threshold=1.2831979990005493
node_375: feature_name=GO:0071901	feature_id[407].value <= threshold=14.498775482177734
node_376: feature_name=GO:0043170	feature_id[467].value > threshold=4.473330818655086e-06
node_380: feature_name=GO:0046718	feature_id[781].value > threshold=3.494875192642212
node_418: feature_name=GO:0002708	feature_id[526].value <= threshold=0.14747196435928345

Class: positive genes

Rules_418	passed counts:2
node_0: feature_name=GO:0042113	feature_id[0].value <= threshold=13.408552169799805
node_1: feature_name=GO:0007568	feature_id[534].value <= threshold=5.0313897132873535
node_2: feature_name=GO:0002705	feature_id[541].value <= threshold=3.200145721435547
node_3: feature_name=GO:1901525	feature_id[576].value <= threshold=0.4466460347175598
node_4: feature_name=GO:0048539	feature_id[319].value <= threshold=3.0399646759033203
node_5: feature_name=GO:0001910	feature_id[385].value <= threshold=3.7437864542007446
node_6: feature_name=GO:0043200	feature_id[706].value <= threshold=9.307284355163574
node_7: feature_name=GO:0001773	feature_id[308].value <= threshold=4.016931533813477
node_8: feature_name=GO:0090116	feature_id[97].value <= threshold=7.99645471572876
node_9: feature_name=GO:0019814	feature_id[189].value <= threshold=4.525782108306885
node_10: feature_name=GO:1902583	feature_id[215].value <= threshold=17.60310649871826
node_11: feature_name=GO:0045429	feature_id[737].value <= threshold=1.6133361458778381
node_12: feature_name=GO:0003720	feature_id[228].value <= threshold=5.519326210021973
node_13: feature_name=GO:0046006	feature_id[364].value <= threshold=4.9985432624816895
node_14: feature_name=GO:0070424	feature_id[254].value <= threshold=6.233297109603882
node_15: feature_name=GO:0009892	feature_id[320].value <= threshold=59.99180793762207
node_16: feature_name=GO:0007064	feature_id[527].value <= threshold=11.57377815246582
node_17: feature_name=GO:0005575	feature_id[17].value <= threshold=15.417460918426514
node_18: feature_name=GO:0043368	feature_id[31].value <= threshold=4.059496641159058
node_19: feature_name=GO:0005164	feature_id[163].value <= threshold=5.793607711791992
node_20: feature_name=GO:0042130	feature_id[16].value <= threshold=5.073179721832275
node_21: feature_name=GO:0010216	feature_id[282].value <= threshold=4.834584474563599
node_22: feature_name=GO:0009628	feature_id[553].value <= threshold=29.314892768859863
node_23: feature_name=GO:0045628	feature_id[749].value <= threshold=3.1001367568969727
node_24: feature_name=GO:0042288	feature_id[100].value <= threshold=5.174932956695557
node_25: feature_name=GO:0002329	feature_id[406].value <= threshold=2.348930835723877
node_26: feature_name=GO:0051246	feature_id[642].value > threshold=0.6498909294605255
node_194: feature_name=GO:0009164	feature_id[546].value <= threshold=17.11970329284668
node_195: feature_name=GO:0001909	feature_id[386].value <= threshold=7.292566299438477
node_196: feature_name=GO:0003908	feature_id[11].value <= threshold=1.887226164340973
node_197: feature_name=GO:0009164	feature_id[546].value > threshold=0.26642198860645294
node_359: feature_name=GO:0009164	feature_id[546].value > threshold=0.2696942090988159
node_361: feature_name=hsa05202	feature_id[50].value <= threshold=14.989679336547852

node_362: feature_name=GO:0001775 feature_id[505].value <= threshold=15.726949691772461
node_363: feature_name=GO:0002634 feature_id[449].value <= threshold=2.825531005859375
node_364: feature_name=GO:0010038 feature_id[457].value <= threshold=9.901018619537354
node_365: feature_name=GO:0044238 feature_id[295].value <= threshold=139.80535888671875
node_366: feature_name=GO:0051246 feature_id[642].value > threshold=0.6527281999588013
node_368: feature_name=GO:0044092 feature_id[432].value <= threshold=11.084641933441162
node_369: feature_name=GO:0042162 feature_id[355].value <= threshold=9.683155536651611
node_370: feature_name=GO:0045945 feature_id[267].value <= threshold=3.6181851625442505
node_371: feature_name=GO:0036296 feature_id[252].value <= threshold=3.214281916618347
node_372: feature_name=GO:0097506 feature_id[51].value <= threshold=2.9644681215286255
node_373: feature_name=GO:0019740 feature_id[581].value <= threshold=2.2853575944900513
node_374: feature_name=GO:0019660 feature_id[70].value <= threshold=1.2831979990005493
node_375: feature_name=GO:0071901 feature_id[407].value <= threshold=14.498775482177734
node_376: feature_name=GO:0043170 feature_id[467].value > threshold=4.473330818655086e-06
node_380: feature_name=GO:0046718 feature_id[781].value <= threshold=3.494875192642212
node_381: feature_name=GO:0030983 feature_id[291].value > threshold=10.994179248809814
node_415: feature_name=GO:0009615 feature_id[265].value > threshold=2.0143595933914185
Class: positive genes

Rules_419 passed counts:2
node_0: feature_name=GO:0042113 feature_id[0].value <= threshold=13.408552169799805
node_1: feature_name=GO:0007568 feature_id[534].value <= threshold=5.0313897132873535
node_2: feature_name=GO:0002705 feature_id[541].value <= threshold=3.200145721435547
node_3: feature_name=GO:1901525 feature_id[576].value <= threshold=0.4466460347175598
node_4: feature_name=GO:0048539 feature_id[319].value <= threshold=3.0399646759033203
node_5: feature_name=GO:0001910 feature_id[385].value <= threshold=3.7437864542007446
node_6: feature_name=GO:0043200 feature_id[706].value <= threshold=9.307284355163574
node_7: feature_name=GO:0001773 feature_id[308].value <= threshold=4.016931533813477
node_8: feature_name=GO:0090116 feature_id[97].value <= threshold=7.99645471572876
node_9: feature_name=GO:0019814 feature_id[189].value <= threshold=4.525782108306885
node_10: feature_name=GO:1902583 feature_id[215].value <= threshold=17.60310649871826
node_11: feature_name=GO:0045429 feature_id[737].value <= threshold=1.6133361458778381
node_12: feature_name=GO:0003720 feature_id[228].value <= threshold=5.519326210021973
node_13: feature_name=GO:0046006 feature_id[364].value <= threshold=4.9985432624816895
node_14: feature_name=GO:0070424 feature_id[254].value <= threshold=6.233297109603882

node_15: feature_name=GO:0009892	feature_id[320].value <= threshold=59.99180793762207
node_16: feature_name=GO:0007064	feature_id[527].value <= threshold=11.57377815246582
node_17: feature_name=GO:0005575	feature_id[17].value <= threshold=15.417460918426514
node_18: feature_name=GO:0043368	feature_id[31].value <= threshold=4.059496641159058
node_19: feature_name=GO:0005164	feature_id[163].value <= threshold=5.793607711791992
node_20: feature_name=GO:0042130	feature_id[16].value <= threshold=5.073179721832275
node_21: feature_name=GO:0010216	feature_id[282].value <= threshold=4.834584474563599
node_22: feature_name=GO:0009628	feature_id[553].value <= threshold=29.314892768859863
node_23: feature_name=GO:0045628	feature_id[749].value <= threshold=3.1001367568969727
node_24: feature_name=GO:0042288	feature_id[100].value <= threshold=5.174932956695557
node_25: feature_name=GO:0002329	feature_id[406].value <= threshold=2.348930835723877
node_26: feature_name=GO:0051246	feature_id[642].value > threshold=0.6498909294605255
node_194: feature_name=GO:0009164	feature_id[546].value <= threshold=17.11970329284668
node_195: feature_name=GO:0001909	feature_id[386].value <= threshold=7.292566299438477
node_196: feature_name=GO:0003908	feature_id[11].value <= threshold=1.887226164340973
node_197: feature_name=GO:0009164	feature_id[546].value > threshold=0.26642198860645294
node_359: feature_name=GO:0009164	feature_id[546].value > threshold=0.2696942090988159
node_361: feature_name=hsa05202	feature_id[50].value <= threshold=14.989679336547852
node_362: feature_name=GO:0001775	feature_id[505].value <= threshold=15.726949691772461
node_363: feature_name=GO:0002634	feature_id[449].value <= threshold=2.825531005859375
node_364: feature_name=GO:0010038	feature_id[457].value <= threshold=9.901018619537354
node_365: feature_name=GO:0044238	feature_id[295].value <= threshold=139.80535888671875
node_366: feature_name=GO:0051246	feature_id[642].value > threshold=0.6527281999588013
node_368: feature_name=GO:0044092	feature_id[432].value <= threshold=11.084641933441162
node_369: feature_name=GO:0042162	feature_id[355].value <= threshold=9.683155536651611
node_370: feature_name=GO:0045945	feature_id[267].value <= threshold=3.6181851625442505
node_371: feature_name=GO:0036296	feature_id[252].value <= threshold=3.214281916618347
node_372: feature_name=GO:0097506	feature_id[51].value <= threshold=2.9644681215286255
node_373: feature_name=GO:0019740	feature_id[581].value <= threshold=2.2853575944900513
node_374: feature_name=GO:0019660	feature_id[70].value <= threshold=1.2831979990005493
node_375: feature_name=GO:0071901	feature_id[407].value <= threshold=14.498775482177734
node_376: feature_name=GO:0043170	feature_id[467].value > threshold=4.473330818655086e-06
node_380: feature_name=GO:0046718	feature_id[781].value <= threshold=3.494875192642212
node_381: feature_name=GO:0030983	feature_id[291].value <= threshold=10.994179248809814
node_382: feature_name=GO:0010663	feature_id[561].value > threshold=2.7056833505630493

node_412: feature_name=GO:0034612 feature_id[656].value <= threshold=0.6953948438167572
Class: negative genes

Rules_420 passed counts:2
node_0: feature_name=GO:0042113 feature_id[0].value <= threshold=13.408552169799805
node_1: feature_name=GO:0007568 feature_id[534].value <= threshold=5.0313897132873535
node_2: feature_name=GO:0002705 feature_id[541].value <= threshold=3.200145721435547
node_3: feature_name=GO:1901525 feature_id[576].value <= threshold=0.4466460347175598
node_4: feature_name=GO:0048539 feature_id[319].value <= threshold=3.0399646759033203
node_5: feature_name=GO:0001910 feature_id[385].value <= threshold=3.7437864542007446
node_6: feature_name=GO:0043200 feature_id[706].value <= threshold=9.307284355163574
node_7: feature_name=GO:0001773 feature_id[308].value <= threshold=4.016931533813477
node_8: feature_name=GO:0090116 feature_id[97].value <= threshold=7.99645471572876
node_9: feature_name=GO:0019814 feature_id[189].value <= threshold=4.525782108306885
node_10: feature_name=GO:1902583 feature_id[215].value <= threshold=17.60310649871826
node_11: feature_name=GO:0045429 feature_id[737].value <= threshold=1.6133361458778381
node_12: feature_name=GO:0003720 feature_id[228].value <= threshold=5.519326210021973
node_13: feature_name=GO:0046006 feature_id[364].value <= threshold=4.9985432624816895
node_14: feature_name=GO:0070424 feature_id[254].value <= threshold=6.233297109603882
node_15: feature_name=GO:0009892 feature_id[320].value <= threshold=59.99180793762207
node_16: feature_name=GO:0007064 feature_id[527].value <= threshold=11.57377815246582
node_17: feature_name=GO:0005575 feature_id[17].value <= threshold=15.417460918426514
node_18: feature_name=GO:0043368 feature_id[31].value <= threshold=4.059496641159058
node_19: feature_name=GO:0005164 feature_id[163].value <= threshold=5.793607711791992
node_20: feature_name=GO:0042130 feature_id[16].value <= threshold=5.073179721832275
node_21: feature_name=GO:0010216 feature_id[282].value <= threshold=4.834584474563599
node_22: feature_name=GO:0009628 feature_id[553].value <= threshold=29.314892768859863
node_23: feature_name=GO:0045628 feature_id[749].value <= threshold=3.1001367568969727
node_24: feature_name=GO:0042288 feature_id[100].value <= threshold=5.174932956695557
node_25: feature_name=GO:0002329 feature_id[406].value <= threshold=2.348930835723877
node_26: feature_name=GO:0051246 feature_id[642].value > threshold=0.6498909294605255
node_194: feature_name=GO:0009164 feature_id[546].value <= threshold=17.11970329284668
node_195: feature_name=GO:0001909 feature_id[386].value <= threshold=7.292566299438477
node_196: feature_name=GO:0003908 feature_id[11].value <= threshold=1.887226164340973
node_197: feature_name=GO:0009164 feature_id[546].value > threshold=0.26642198860645294

node_359: feature_name=GO:0009164	feature_id[546].value > threshold=0.2696942090988159
node_361: feature_name=hsa05202	feature_id[50].value <= threshold=14.989679336547852
node_362: feature_name=GO:0001775	feature_id[505].value <= threshold=15.726949691772461
node_363: feature_name=GO:0002634	feature_id[449].value <= threshold=2.825531005859375
node_364: feature_name=GO:0010038	feature_id[457].value <= threshold=9.901018619537354
node_365: feature_name=GO:0044238	feature_id[295].value <= threshold=139.80535888671875
node_366: feature_name=GO:0051246	feature_id[642].value > threshold=0.6527281999588013
node_368: feature_name=GO:0044092	feature_id[432].value <= threshold=11.084641933441162
node_369: feature_name=GO:0042162	feature_id[355].value <= threshold=9.683155536651611
node_370: feature_name=GO:0045945	feature_id[267].value <= threshold=3.6181851625442505
node_371: feature_name=GO:0036296	feature_id[252].value <= threshold=3.214281916618347
node_372: feature_name=GO:0097506	feature_id[51].value <= threshold=2.9644681215286255
node_373: feature_name=GO:0019740	feature_id[581].value <= threshold=2.2853575944900513
node_374: feature_name=GO:0019660	feature_id[70].value <= threshold=1.2831979990005493
node_375: feature_name=GO:0071901	feature_id[407].value <= threshold=14.498775482177734
node_376: feature_name=GO:0043170	feature_id[467].value > threshold=4.473330818655086e-06
node_380: feature_name=GO:0046718	feature_id[781].value <= threshold=3.494875192642212
node_381: feature_name=GO:0030983	feature_id[291].value <= threshold=10.994179248809814
node_382: feature_name=GO:0010663	feature_id[561].value <= threshold=2.7056833505630493
node_383: feature_name=GO:0031667	feature_id[648].value > threshold=0.0020462179090827703
node_387: feature_name=GO:1990572	feature_id[243].value <= threshold=1.9359037280082703
node_388: feature_name=GO:0032753	feature_id[101].value > threshold=1.182796835899353
node_404: feature_name=GO:0002449	feature_id[427].value <= threshold=0.09284071624279022
Class: positive genes	
Rules_421	passed counts:2
node_0: feature_name=GO:0042113	feature_id[0].value <= threshold=13.408552169799805
node_1: feature_name=GO:0007568	feature_id[534].value <= threshold=5.0313897132873535
node_2: feature_name=GO:0002705	feature_id[541].value <= threshold=3.200145721435547
node_3: feature_name=GO:1901525	feature_id[576].value <= threshold=0.4466460347175598
node_4: feature_name=GO:0048539	feature_id[319].value <= threshold=3.0399646759033203
node_5: feature_name=GO:0001910	feature_id[385].value <= threshold=3.7437864542007446
node_6: feature_name=GO:0043200	feature_id[706].value <= threshold=9.307284355163574
node_7: feature_name=GO:0001773	feature_id[308].value <= threshold=4.016931533813477
node_8: feature_name=GO:0090116	feature_id[97].value <= threshold=7.99645471572876

node_9: feature_name=GO:0019814	feature_id[189].value <= threshold=4.525782108306885
node_10: feature_name=GO:1902583	feature_id[215].value <= threshold=17.60310649871826
node_11: feature_name=GO:0045429	feature_id[737].value <= threshold=1.6133361458778381
node_12: feature_name=GO:0003720	feature_id[228].value <= threshold=5.519326210021973
node_13: feature_name=GO:0046006	feature_id[364].value <= threshold=4.9985432624816895
node_14: feature_name=GO:0070424	feature_id[254].value <= threshold=6.233297109603882
node_15: feature_name=GO:0009892	feature_id[320].value <= threshold=59.99180793762207
node_16: feature_name=GO:0007064	feature_id[527].value <= threshold=11.57377815246582
node_17: feature_name=GO:0005575	feature_id[17].value <= threshold=15.417460918426514
node_18: feature_name=GO:0043368	feature_id[31].value <= threshold=4.059496641159058
node_19: feature_name=GO:0005164	feature_id[163].value <= threshold=5.793607711791992
node_20: feature_name=GO:0042130	feature_id[16].value <= threshold=5.073179721832275
node_21: feature_name=GO:0010216	feature_id[282].value <= threshold=4.834584474563599
node_22: feature_name=GO:0009628	feature_id[553].value <= threshold=29.314892768859863
node_23: feature_name=GO:0045628	feature_id[749].value <= threshold=3.1001367568969727
node_24: feature_name=GO:0042288	feature_id[100].value <= threshold=5.174932956695557
node_25: feature_name=GO:0002329	feature_id[406].value <= threshold=2.348930835723877
node_26: feature_name=GO:0051246	feature_id[642].value > threshold=0.6498909294605255
node_194: feature_name=GO:0009164	feature_id[546].value <= threshold=17.11970329284668
node_195: feature_name=GO:0001909	feature_id[386].value <= threshold=7.292566299438477
node_196: feature_name=GO:0003908	feature_id[11].value <= threshold=1.887226164340973
node_197: feature_name=GO:0009164	feature_id[546].value > threshold=0.26642198860645294
node_359: feature_name=GO:0009164	feature_id[546].value > threshold=0.2696942090988159
node_361: feature_name=hsa05202	feature_id[50].value <= threshold=14.989679336547852
node_362: feature_name=GO:0001775	feature_id[505].value <= threshold=15.726949691772461
node_363: feature_name=GO:0002634	feature_id[449].value <= threshold=2.825531005859375
node_364: feature_name=GO:0010038	feature_id[457].value <= threshold=9.901018619537354
node_365: feature_name=GO:0044238	feature_id[295].value <= threshold=139.80535888671875
node_366: feature_name=GO:0051246	feature_id[642].value > threshold=0.6527281999588013
node_368: feature_name=GO:0044092	feature_id[432].value <= threshold=11.084641933441162
node_369: feature_name=GO:0042162	feature_id[355].value <= threshold=9.683155536651611
node_370: feature_name=GO:0045945	feature_id[267].value <= threshold=3.6181851625442505
node_371: feature_name=GO:0036296	feature_id[252].value <= threshold=3.214281916618347
node_372: feature_name=GO:0097506	feature_id[51].value <= threshold=2.9644681215286255
node_373: feature_name=GO:0019740	feature_id[581].value <= threshold=2.2853575944900513

node_374: feature_name=GO:0019660 feature_id[70].value <= threshold=1.2831979990005493
node_375: feature_name=GO:0071901 feature_id[407].value <= threshold=14.498775482177734
node_376: feature_name=GO:0043170 feature_id[467].value > threshold=4.473330818655086e-06
node_380: feature_name=GO:0046718 feature_id[781].value <= threshold=3.494875192642212
node_381: feature_name=GO:0030983 feature_id[291].value <= threshold=10.994179248809814
node_382: feature_name=GO:0010663 feature_id[561].value <= threshold=2.7056833505630493
node_383: feature_name=GO:0031667 feature_id[648].value > threshold=0.0020462179090827703
node_387: feature_name=GO:1990572 feature_id[243].value <= threshold=1.9359037280082703
node_388: feature_name=GO:0032753 feature_id[101].value <= threshold=1.182796835899353
node_389: feature_name=GO:0097153 feature_id[226].value > threshold=0.34601132571697235
node_399: feature_name=GO:0036294 feature_id[612].value <= threshold=0.022128281882032752
Class: positive genes

Rules_422 passed counts:2
node_0: feature_name=GO:0042113 feature_id[0].value <= threshold=13.408552169799805
node_1: feature_name=GO:0007568 feature_id[534].value <= threshold=5.0313897132873535
node_2: feature_name=GO:0002705 feature_id[541].value <= threshold=3.200145721435547
node_3: feature_name=GO:1901525 feature_id[576].value <= threshold=0.4466460347175598
node_4: feature_name=GO:0048539 feature_id[319].value <= threshold=3.0399646759033203
node_5: feature_name=GO:0001910 feature_id[385].value <= threshold=3.7437864542007446
node_6: feature_name=GO:0043200 feature_id[706].value <= threshold=9.307284355163574
node_7: feature_name=GO:0001773 feature_id[308].value <= threshold=4.016931533813477
node_8: feature_name=GO:0090116 feature_id[97].value <= threshold=7.99645471572876
node_9: feature_name=GO:0019814 feature_id[189].value <= threshold=4.525782108306885
node_10: feature_name=GO:1902583 feature_id[215].value <= threshold=17.60310649871826
node_11: feature_name=GO:0045429 feature_id[737].value <= threshold=1.6133361458778381
node_12: feature_name=GO:0003720 feature_id[228].value <= threshold=5.519326210021973
node_13: feature_name=GO:0046006 feature_id[364].value <= threshold=4.9985432624816895
node_14: feature_name=GO:0070424 feature_id[254].value <= threshold=6.233297109603882
node_15: feature_name=GO:0009892 feature_id[320].value <= threshold=59.99180793762207
node_16: feature_name=GO:0007064 feature_id[527].value <= threshold=11.57377815246582
node_17: feature_name=GO:0005575 feature_id[17].value <= threshold=15.417460918426514
node_18: feature_name=GO:0043368 feature_id[31].value <= threshold=4.059496641159058
node_19: feature_name=GO:0005164 feature_id[163].value <= threshold=5.793607711791992
node_20: feature_name=GO:0042130 feature_id[16].value <= threshold=5.073179721832275

node_21: feature_name=GO:0010216 feature_id[282].value <= threshold=4.834584474563599
node_22: feature_name=GO:0009628 feature_id[553].value <= threshold=29.314892768859863
node_23: feature_name=GO:0045628 feature_id[749].value <= threshold=3.1001367568969727
node_24: feature_name=GO:0042288 feature_id[100].value <= threshold=5.174932956695557
node_25: feature_name=GO:0002329 feature_id[406].value <= threshold=2.348930835723877
node_26: feature_name=GO:0051246 feature_id[642].value > threshold=0.6498909294605255
node_194: feature_name=GO:0009164 feature_id[546].value <= threshold=17.11970329284668
node_195: feature_name=GO:0001909 feature_id[386].value <= threshold=7.292566299438477
node_196: feature_name=GO:0003908 feature_id[11].value <= threshold=1.887226164340973
node_197: feature_name=GO:0009164 feature_id[546].value > threshold=0.26642198860645294
node_359: feature_name=GO:0009164 feature_id[546].value > threshold=0.2696942090988159
node_361: feature_name=hsa05202 feature_id[50].value <= threshold=14.989679336547852
node_362: feature_name=GO:0001775 feature_id[505].value <= threshold=15.726949691772461
node_363: feature_name=GO:0002634 feature_id[449].value <= threshold=2.825531005859375
node_364: feature_name=GO:0010038 feature_id[457].value <= threshold=9.901018619537354
node_365: feature_name=GO:0044238 feature_id[295].value <= threshold=139.80535888671875
node_366: feature_name=GO:0051246 feature_id[642].value > threshold=0.6527281999588013
node_368: feature_name=GO:0044092 feature_id[432].value <= threshold=11.084641933441162
node_369: feature_name=GO:0042162 feature_id[355].value <= threshold=9.683155536651611
node_370: feature_name=GO:0045945 feature_id[267].value <= threshold=3.6181851625442505
node_371: feature_name=GO:0036296 feature_id[252].value <= threshold=3.214281916618347
node_372: feature_name=GO:0097506 feature_id[51].value <= threshold=2.9644681215286255
node_373: feature_name=GO:0019740 feature_id[581].value <= threshold=2.2853575944900513
node_374: feature_name=GO:0019660 feature_id[70].value <= threshold=1.2831979990005493
node_375: feature_name=GO:0071901 feature_id[407].value <= threshold=14.498775482177734
node_376: feature_name=GO:0043170 feature_id[467].value > threshold=4.473330818655086e-06
node_380: feature_name=GO:0046718 feature_id[781].value <= threshold=3.494875192642212
node_381: feature_name=GO:0030983 feature_id[291].value <= threshold=10.994179248809814
node_382: feature_name=GO:0010663 feature_id[561].value <= threshold=2.7056833505630493
node_383: feature_name=GO:0031667 feature_id[648].value <= threshold=0.0020462179090827703
node_384: feature_name=GO:0070102 feature_id[49].value > threshold=0.34830209612846375
Class: positive genes

Rules_423 passed counts:2
node_0: feature_name=GO:0042113 feature_id[0].value <= threshold=13.408552169799805

node_1: feature_name=GO:0007568 feature_id[534].value <= threshold=5.0313897132873535
node_2: feature_name=GO:0002705 feature_id[541].value <= threshold=3.200145721435547
node_3: feature_name=GO:1901525 feature_id[576].value <= threshold=0.4466460347175598
node_4: feature_name=GO:0048539 feature_id[319].value <= threshold=3.0399646759033203
node_5: feature_name=GO:0001910 feature_id[385].value <= threshold=3.7437864542007446
node_6: feature_name=GO:0043200 feature_id[706].value <= threshold=9.307284355163574
node_7: feature_name=GO:0001773 feature_id[308].value <= threshold=4.016931533813477
node_8: feature_name=GO:0090116 feature_id[97].value <= threshold=7.99645471572876
node_9: feature_name=GO:0019814 feature_id[189].value <= threshold=4.525782108306885
node_10: feature_name=GO:1902583 feature_id[215].value <= threshold=17.60310649871826
node_11: feature_name=GO:0045429 feature_id[737].value <= threshold=1.6133361458778381
node_12: feature_name=GO:0003720 feature_id[228].value <= threshold=5.519326210021973
node_13: feature_name=GO:0046006 feature_id[364].value <= threshold=4.9985432624816895
node_14: feature_name=GO:0070424 feature_id[254].value <= threshold=6.233297109603882
node_15: feature_name=GO:0009892 feature_id[320].value <= threshold=59.99180793762207
node_16: feature_name=GO:0007064 feature_id[527].value <= threshold=11.57377815246582
node_17: feature_name=GO:0005575 feature_id[17].value <= threshold=15.417460918426514
node_18: feature_name=GO:0043368 feature_id[31].value <= threshold=4.059496641159058
node_19: feature_name=GO:0005164 feature_id[163].value <= threshold=5.793607711791992
node_20: feature_name=GO:0042130 feature_id[16].value <= threshold=5.073179721832275
node_21: feature_name=GO:0010216 feature_id[282].value <= threshold=4.834584474563599
node_22: feature_name=GO:0009628 feature_id[553].value <= threshold=29.314892768859863
node_23: feature_name=GO:0045628 feature_id[749].value <= threshold=3.1001367568969727
node_24: feature_name=GO:0042288 feature_id[100].value <= threshold=5.174932956695557
node_25: feature_name=GO:0002329 feature_id[406].value <= threshold=2.348930835723877
node_26: feature_name=GO:0051246 feature_id[642].value > threshold=0.6498909294605255
node_194: feature_name=GO:0009164 feature_id[546].value <= threshold=17.11970329284668
node_195: feature_name=GO:0001909 feature_id[386].value <= threshold=7.292566299438477
node_196: feature_name=GO:0003908 feature_id[11].value <= threshold=1.887226164340973
node_197: feature_name=GO:0009164 feature_id[546].value > threshold=0.26642198860645294
node_359: feature_name=GO:0009164 feature_id[546].value <= threshold=0.2696942090988159
Class: positive genes

Rules_424 passed counts:2
node_0: feature_name=GO:0042113 feature_id[0].value <= threshold=13.408552169799805

node_1: feature_name=GO:0007568	feature_id[534].value <= threshold=5.0313897132873535
node_2: feature_name=GO:0002705	feature_id[541].value <= threshold=3.200145721435547
node_3: feature_name=GO:1901525	feature_id[576].value <= threshold=0.4466460347175598
node_4: feature_name=GO:0048539	feature_id[319].value <= threshold=3.0399646759033203
node_5: feature_name=GO:0001910	feature_id[385].value <= threshold=3.7437864542007446
node_6: feature_name=GO:0043200	feature_id[706].value <= threshold=9.307284355163574
node_7: feature_name=GO:0001773	feature_id[308].value <= threshold=4.016931533813477
node_8: feature_name=GO:0090116	feature_id[97].value <= threshold=7.99645471572876
node_9: feature_name=GO:0019814	feature_id[189].value <= threshold=4.525782108306885
node_10: feature_name=GO:1902583	feature_id[215].value <= threshold=17.60310649871826
node_11: feature_name=GO:0045429	feature_id[737].value <= threshold=1.6133361458778381
node_12: feature_name=GO:0003720	feature_id[228].value <= threshold=5.519326210021973
node_13: feature_name=GO:0046006	feature_id[364].value <= threshold=4.9985432624816895
node_14: feature_name=GO:0070424	feature_id[254].value <= threshold=6.233297109603882
node_15: feature_name=GO:0009892	feature_id[320].value <= threshold=59.99180793762207
node_16: feature_name=GO:0007064	feature_id[527].value <= threshold=11.57377815246582
node_17: feature_name=GO:0005575	feature_id[17].value <= threshold=15.417460918426514
node_18: feature_name=GO:0043368	feature_id[31].value <= threshold=4.059496641159058
node_19: feature_name=GO:0005164	feature_id[163].value <= threshold=5.793607711791992
node_20: feature_name=GO:0042130	feature_id[16].value <= threshold=5.073179721832275
node_21: feature_name=GO:0010216	feature_id[282].value <= threshold=4.834584474563599
node_22: feature_name=GO:0009628	feature_id[553].value <= threshold=29.314892768859863
node_23: feature_name=GO:0045628	feature_id[749].value <= threshold=3.1001367568969727
node_24: feature_name=GO:0042288	feature_id[100].value <= threshold=5.174932956695557
node_25: feature_name=GO:0002329	feature_id[406].value <= threshold=2.348930835723877
node_26: feature_name=GO:0051246	feature_id[642].value > threshold=0.6498909294605255
node_194: feature_name=GO:0009164	feature_id[546].value <= threshold=17.11970329284668
node_195: feature_name=GO:0001909	feature_id[386].value <= threshold=7.292566299438477
node_196: feature_name=GO:0003908	feature_id[11].value <= threshold=1.887226164340973
node_197: feature_name=GO:0009164	feature_id[546].value <= threshold=0.26642198860645294
node_198: feature_name=GO:2001242	feature_id[24].value <= threshold=9.207026481628418
node_199: feature_name=GO:0051246	feature_id[642].value > threshold=0.6504445374011993
node_203: feature_name=GO:0032461	feature_id[608].value <= threshold=2.607542634010315
node_204: feature_name=GO:0007584	feature_id[529].value <= threshold=16.099515914916992
node_205: feature_name=hsa05221	feature_id[349].value > threshold=9.359850406646729

node_337: feature_name=GO:0032743 feature_id[258].value > threshold=2.931445837020874
node_347: feature_name=GO:0000217 feature_id[765].value <= threshold=1.9003757238388062
Class: positive genes

Rules_425 passed counts:2
node_0: feature_name=GO:0042113 feature_id[0].value <= threshold=13.408552169799805
node_1: feature_name=GO:0007568 feature_id[534].value <= threshold=5.0313897132873535
node_2: feature_name=GO:0002705 feature_id[541].value <= threshold=3.200145721435547
node_3: feature_name=GO:1901525 feature_id[576].value <= threshold=0.4466460347175598
node_4: feature_name=GO:0048539 feature_id[319].value <= threshold=3.0399646759033203
node_5: feature_name=GO:0001910 feature_id[385].value <= threshold=3.7437864542007446
node_6: feature_name=GO:0043200 feature_id[706].value <= threshold=9.307284355163574
node_7: feature_name=GO:0001773 feature_id[308].value <= threshold=4.016931533813477
node_8: feature_name=GO:0090116 feature_id[97].value <= threshold=7.99645471572876
node_9: feature_name=GO:0019814 feature_id[189].value <= threshold=4.525782108306885
node_10: feature_name=GO:1902583 feature_id[215].value <= threshold=17.60310649871826
node_11: feature_name=GO:0045429 feature_id[737].value <= threshold=1.6133361458778381
node_12: feature_name=GO:0003720 feature_id[228].value <= threshold=5.519326210021973
node_13: feature_name=GO:0046006 feature_id[364].value <= threshold=4.9985432624816895
node_14: feature_name=GO:0070424 feature_id[254].value <= threshold=6.233297109603882
node_15: feature_name=GO:0009892 feature_id[320].value <= threshold=59.99180793762207
node_16: feature_name=GO:0007064 feature_id[527].value <= threshold=11.57377815246582
node_17: feature_name=GO:0005575 feature_id[17].value <= threshold=15.417460918426514
node_18: feature_name=GO:0043368 feature_id[31].value <= threshold=4.059496641159058
node_19: feature_name=GO:0005164 feature_id[163].value <= threshold=5.793607711791992
node_20: feature_name=GO:0042130 feature_id[16].value <= threshold=5.073179721832275
node_21: feature_name=GO:0010216 feature_id[282].value <= threshold=4.834584474563599
node_22: feature_name=GO:0009628 feature_id[553].value <= threshold=29.314892768859863
node_23: feature_name=GO:0045628 feature_id[749].value <= threshold=3.1001367568969727
node_24: feature_name=GO:0042288 feature_id[100].value <= threshold=5.174932956695557
node_25: feature_name=GO:0002329 feature_id[406].value <= threshold=2.348930835723877
node_26: feature_name=GO:0051246 feature_id[642].value > threshold=0.6498909294605255
node_194: feature_name=GO:0009164 feature_id[546].value <= threshold=17.11970329284668
node_195: feature_name=GO:0001909 feature_id[386].value <= threshold=7.292566299438477
node_196: feature_name=GO:0003908 feature_id[11].value <= threshold=1.887226164340973

node_197: feature_name=GO:0009164 feature_id[546].value <= threshold=0.26642198860645294
node_198: feature_name=GO:2001242 feature_id[24].value <= threshold=9.207026481628418
node_199: feature_name=GO:0051246 feature_id[642].value > threshold=0.6504445374011993
node_203: feature_name=GO:0032461 feature_id[608].value <= threshold=2.607542634010315
node_204: feature_name=GO:0007584 feature_id[529].value <= threshold=16.099515914916992
node_205: feature_name=hsa05221 feature_id[349].value <= threshold=9.359850406646729
node_206: feature_name=GO:0002636 feature_id[447].value > threshold=1.615447759628296
node_334: feature_name=GO:0002683 feature_id[456].value > threshold=0.5395609866827726
Class: negative genes

Rules_426 passed counts:2
node_0: feature_name=GO:0042113 feature_id[0].value <= threshold=13.408552169799805
node_1: feature_name=GO:0007568 feature_id[534].value <= threshold=5.0313897132873535
node_2: feature_name=GO:0002705 feature_id[541].value <= threshold=3.200145721435547
node_3: feature_name=GO:1901525 feature_id[576].value <= threshold=0.4466460347175598
node_4: feature_name=GO:0048539 feature_id[319].value <= threshold=3.0399646759033203
node_5: feature_name=GO:0001910 feature_id[385].value <= threshold=3.7437864542007446
node_6: feature_name=GO:0043200 feature_id[706].value <= threshold=9.307284355163574
node_7: feature_name=GO:0001773 feature_id[308].value <= threshold=4.016931533813477
node_8: feature_name=GO:0090116 feature_id[97].value <= threshold=7.99645471572876
node_9: feature_name=GO:0019814 feature_id[189].value <= threshold=4.525782108306885
node_10: feature_name=GO:1902583 feature_id[215].value <= threshold=17.60310649871826
node_11: feature_name=GO:0045429 feature_id[737].value <= threshold=1.6133361458778381
node_12: feature_name=GO:0003720 feature_id[228].value <= threshold=5.519326210021973
node_13: feature_name=GO:0046006 feature_id[364].value <= threshold=4.9985432624816895
node_14: feature_name=GO:0070424 feature_id[254].value <= threshold=6.233297109603882
node_15: feature_name=GO:0009892 feature_id[320].value <= threshold=59.99180793762207
node_16: feature_name=GO:0007064 feature_id[527].value <= threshold=11.57377815246582
node_17: feature_name=GO:0005575 feature_id[17].value <= threshold=15.417460918426514
node_18: feature_name=GO:0043368 feature_id[31].value <= threshold=4.059496641159058
node_19: feature_name=GO:0005164 feature_id[163].value <= threshold=5.793607711791992
node_20: feature_name=GO:0042130 feature_id[16].value <= threshold=5.073179721832275
node_21: feature_name=GO:0010216 feature_id[282].value <= threshold=4.834584474563599
node_22: feature_name=GO:0009628 feature_id[553].value <= threshold=29.314892768859863
node_23: feature_name=GO:0045628 feature_id[749].value <= threshold=3.1001367568969727

node_24: feature_name=GO:0042288 feature_id[100].value <= threshold=5.174932956695557
node_25: feature_name=GO:0002329 feature_id[406].value <= threshold=2.348930835723877
node_26: feature_name=GO:0051246 feature_id[642].value > threshold=0.6498909294605255
node_194: feature_name=GO:0009164 feature_id[546].value <= threshold=17.11970329284668
node_195: feature_name=GO:0001909 feature_id[386].value <= threshold=7.292566299438477
node_196: feature_name=GO:0003908 feature_id[11].value <= threshold=1.887226164340973
node_197: feature_name=GO:0009164 feature_id[546].value <= threshold=0.26642198860645294
node_198: feature_name=GO:2001242 feature_id[24].value <= threshold=9.207026481628418
node_199: feature_name=GO:0051246 feature_id[642].value > threshold=0.6504445374011993
node_203: feature_name=GO:0032461 feature_id[608].value <= threshold=2.607542634010315
node_204: feature_name=GO:0007584 feature_id[529].value <= threshold=16.099515914916992
node_205: feature_name=hsa05221 feature_id[349].value <= threshold=9.359850406646729
node_206: feature_name=GO:0002636 feature_id[447].value <= threshold=1.615447759628296
node_207: feature_name=GO:0033993 feature_id[421].value > threshold=2.96540611088858e-05
node_217: feature_name=GO:0002331 feature_id[404].value <= threshold=2.1883513927459717
node_218: feature_name=GO:0046498 feature_id[203].value <= threshold=4.085246205329895
node_219: feature_name=GO:0002832 feature_id[491].value <= threshold=3.0228612422943115
node_220: feature_name=GO:1904868 feature_id[306].value <= threshold=3.8674756288528442
node_221: feature_name=GO:0048545 feature_id[360].value <= threshold=27.38136100769043
node_222: feature_name=GO:0071310 feature_id[760].value > threshold=2.3522024154663086
node_306: feature_name=GO:0070233 feature_id[321].value > threshold=1.293377935886383
node_316: feature_name=GO:0009897 feature_id[38].value <= threshold=0.002804968156851828
Class: positive genes

Rules_427 passed counts:2
node_0: feature_name=GO:0042113 feature_id[0].value <= threshold=13.408552169799805
node_1: feature_name=GO:0007568 feature_id[534].value <= threshold=5.0313897132873535
node_2: feature_name=GO:0002705 feature_id[541].value <= threshold=3.200145721435547
node_3: feature_name=GO:1901525 feature_id[576].value <= threshold=0.4466460347175598
node_4: feature_name=GO:0048539 feature_id[319].value <= threshold=3.0399646759033203
node_5: feature_name=GO:0001910 feature_id[385].value <= threshold=3.7437864542007446
node_6: feature_name=GO:0043200 feature_id[706].value <= threshold=9.307284355163574
node_7: feature_name=GO:0001773 feature_id[308].value <= threshold=4.016931533813477
node_8: feature_name=GO:0090116 feature_id[97].value <= threshold=7.99645471572876
node_9: feature_name=GO:0019814 feature_id[189].value <= threshold=4.525782108306885

node_10: feature_name=GO:1902583	feature_id[215].value <= threshold=17.60310649871826
node_11: feature_name=GO:0045429	feature_id[737].value <= threshold=1.6133361458778381
node_12: feature_name=GO:0003720	feature_id[228].value <= threshold=5.519326210021973
node_13: feature_name=GO:0046006	feature_id[364].value <= threshold=4.9985432624816895
node_14: feature_name=GO:0070424	feature_id[254].value <= threshold=6.233297109603882
node_15: feature_name=GO:0009892	feature_id[320].value <= threshold=59.99180793762207
node_16: feature_name=GO:0007064	feature_id[527].value <= threshold=11.57377815246582
node_17: feature_name=GO:0005575	feature_id[17].value <= threshold=15.417460918426514
node_18: feature_name=GO:0043368	feature_id[31].value <= threshold=4.059496641159058
node_19: feature_name=GO:0005164	feature_id[163].value <= threshold=5.793607711791992
node_20: feature_name=GO:0042130	feature_id[16].value <= threshold=5.073179721832275
node_21: feature_name=GO:0010216	feature_id[282].value <= threshold=4.834584474563599
node_22: feature_name=GO:0009628	feature_id[553].value <= threshold=29.314892768859863
node_23: feature_name=GO:0045628	feature_id[749].value <= threshold=3.1001367568969727
node_24: feature_name=GO:0042288	feature_id[100].value <= threshold=5.174932956695557
node_25: feature_name=GO:0002329	feature_id[406].value <= threshold=2.348930835723877
node_26: feature_name=GO:0051246	feature_id[642].value > threshold=0.6498909294605255
node_194: feature_name=GO:0009164	feature_id[546].value <= threshold=17.11970329284668
node_195: feature_name=GO:0001909	feature_id[386].value <= threshold=7.292566299438477
node_196: feature_name=GO:0003908	feature_id[11].value <= threshold=1.887226164340973
node_197: feature_name=GO:0009164	feature_id[546].value <= threshold=0.26642198860645294
node_198: feature_name=GO:2001242	feature_id[24].value <= threshold=9.207026481628418
node_199: feature_name=GO:0051246	feature_id[642].value > threshold=0.6504445374011993
node_203: feature_name=GO:0032461	feature_id[608].value <= threshold=2.607542634010315
node_204: feature_name=GO:0007584	feature_id[529].value <= threshold=16.099515914916992
node_205: feature_name=hsa05221	feature_id[349].value <= threshold=9.359850406646729
node_206: feature_name=GO:0002636	feature_id[447].value <= threshold=1.615447759628296
node_207: feature_name=GO:0033993	feature_id[421].value > threshold=2.96540611088858e-05
node_217: feature_name=GO:0002331	feature_id[404].value <= threshold=2.1883513927459717
node_218: feature_name=GO:0046498	feature_id[203].value <= threshold=4.085246205329895
node_219: feature_name=GO:0002832	feature_id[491].value <= threshold=3.0228612422943115
node_220: feature_name=GO:1904868	feature_id[306].value <= threshold=3.8674756288528442
node_221: feature_name=GO:0048545	feature_id[360].value <= threshold=27.38136100769043
node_222: feature_name=GO:0071310	feature_id[760].value <= threshold=2.3522024154663086
node_223: feature_name=GO:0001775	feature_id[505].value <= threshold=9.71301555633545

node_224: feature_name=GO:0071310 feature_id[760].value <= threshold=2.346743583679199
node_225: feature_name=GO:0001772 feature_id[91].value <= threshold=3.252573609352112
node_226: feature_name=GO:0036498 feature_id[359].value <= threshold=12.092026710510254
node_227: feature_name=hsa05340 feature_id[351].value <= threshold=0.7677814364433289
node_228: feature_name=GO:0032703 feature_id[617].value > threshold=1.7450646758079529
node_278: feature_name=GO:0002683 feature_id[456].value <= threshold=0.05331694148480892
Class: positive genes

Rules_428 passed counts:2
node_0: feature_name=GO:0042113 feature_id[0].value <= threshold=13.408552169799805
node_1: feature_name=GO:0007568 feature_id[534].value <= threshold=5.0313897132873535
node_2: feature_name=GO:0002705 feature_id[541].value <= threshold=3.200145721435547
node_3: feature_name=GO:1901525 feature_id[576].value <= threshold=0.4466460347175598
node_4: feature_name=GO:0048539 feature_id[319].value <= threshold=3.0399646759033203
node_5: feature_name=GO:0001910 feature_id[385].value <= threshold=3.7437864542007446
node_6: feature_name=GO:0043200 feature_id[706].value <= threshold=9.307284355163574
node_7: feature_name=GO:0001773 feature_id[308].value <= threshold=4.016931533813477
node_8: feature_name=GO:0090116 feature_id[97].value <= threshold=7.99645471572876
node_9: feature_name=GO:0019814 feature_id[189].value <= threshold=4.525782108306885
node_10: feature_name=GO:1902583 feature_id[215].value <= threshold=17.60310649871826
node_11: feature_name=GO:0045429 feature_id[737].value <= threshold=1.6133361458778381
node_12: feature_name=GO:0003720 feature_id[228].value <= threshold=5.519326210021973
node_13: feature_name=GO:0046006 feature_id[364].value <= threshold=4.9985432624816895
node_14: feature_name=GO:0070424 feature_id[254].value <= threshold=6.233297109603882
node_15: feature_name=GO:0009892 feature_id[320].value <= threshold=59.99180793762207
node_16: feature_name=GO:0007064 feature_id[527].value <= threshold=11.57377815246582
node_17: feature_name=GO:0005575 feature_id[17].value <= threshold=15.417460918426514
node_18: feature_name=GO:0043368 feature_id[31].value <= threshold=4.059496641159058
node_19: feature_name=GO:0005164 feature_id[163].value <= threshold=5.793607711791992
node_20: feature_name=GO:0042130 feature_id[16].value <= threshold=5.073179721832275
node_21: feature_name=GO:0010216 feature_id[282].value <= threshold=4.834584474563599
node_22: feature_name=GO:0009628 feature_id[553].value <= threshold=29.314892768859863
node_23: feature_name=GO:0045628 feature_id[749].value <= threshold=3.1001367568969727
node_24: feature_name=GO:0042288 feature_id[100].value <= threshold=5.174932956695557
node_25: feature_name=GO:0002329 feature_id[406].value <= threshold=2.348930835723877

node_26: feature_name=GO:0051246 feature_id[642].value > threshold=0.6498909294605255
node_194: feature_name=GO:0009164 feature_id[546].value <= threshold=17.11970329284668
node_195: feature_name=GO:0001909 feature_id[386].value <= threshold=7.292566299438477
node_196: feature_name=GO:0003908 feature_id[11].value <= threshold=1.887226164340973
node_197: feature_name=GO:0009164 feature_id[546].value <= threshold=0.26642198860645294
node_198: feature_name=GO:2001242 feature_id[24].value <= threshold=9.207026481628418
node_199: feature_name=GO:0051246 feature_id[642].value > threshold=0.6504445374011993
node_203: feature_name=GO:0032461 feature_id[608].value <= threshold=2.607542634010315
node_204: feature_name=GO:0007584 feature_id[529].value <= threshold=16.099515914916992
node_205: feature_name=hsa05221 feature_id[349].value <= threshold=9.359850406646729
node_206: feature_name=GO:0002636 feature_id[447].value <= threshold=1.615447759628296
node_207: feature_name=GO:0033993 feature_id[421].value > threshold=2.96540611088858e-05
node_217: feature_name=GO:0002331 feature_id[404].value <= threshold=2.1883513927459717
node_218: feature_name=GO:0046498 feature_id[203].value <= threshold=4.085246205329895
node_219: feature_name=GO:0002832 feature_id[491].value <= threshold=3.0228612422943115
node_220: feature_name=GO:1904868 feature_id[306].value <= threshold=3.8674756288528442
node_221: feature_name=GO:0048545 feature_id[360].value <= threshold=27.38136100769043
node_222: feature_name=GO:0071310 feature_id[760].value <= threshold=2.3522024154663086
node_223: feature_name=GO:0001775 feature_id[505].value <= threshold=9.71301555633545
node_224: feature_name=GO:0071310 feature_id[760].value <= threshold=2.346743583679199
node_225: feature_name=GO:0001772 feature_id[91].value <= threshold=3.252573609352112
node_226: feature_name=GO:0036498 feature_id[359].value <= threshold=12.092026710510254
node_227: feature_name=hsa05340 feature_id[351].value <= threshold=0.7677814364433289
node_228: feature_name=GO:0032703 feature_id[617].value <= threshold=1.7450646758079529
node_229: feature_name=hsa04640 feature_id[79].value > threshold=2.118402123451233
node_275: feature_name=GO:0002250 feature_id[417].value <= threshold=0.040423303842544556
Class: positive genes

Rules_429 passed counts:2
node_0: feature_name=GO:0042113 feature_id[0].value <= threshold=13.408552169799805
node_1: feature_name=GO:0007568 feature_id[534].value <= threshold=5.0313897132873535
node_2: feature_name=GO:0002705 feature_id[541].value <= threshold=3.200145721435547
node_3: feature_name=GO:1901525 feature_id[576].value <= threshold=0.4466460347175598
node_4: feature_name=GO:0048539 feature_id[319].value <= threshold=3.0399646759033203
node_5: feature_name=GO:0001910 feature_id[385].value <= threshold=3.7437864542007446

node_6: feature_name=GO:0043200	feature_id[706].value <= threshold=9.307284355163574
node_7: feature_name=GO:0001773	feature_id[308].value <= threshold=4.016931533813477
node_8: feature_name=GO:0090116	feature_id[97].value <= threshold=7.99645471572876
node_9: feature_name=GO:0019814	feature_id[189].value <= threshold=4.525782108306885
node_10: feature_name=GO:1902583	feature_id[215].value <= threshold=17.60310649871826
node_11: feature_name=GO:0045429	feature_id[737].value <= threshold=1.6133361458778381
node_12: feature_name=GO:0003720	feature_id[228].value <= threshold=5.519326210021973
node_13: feature_name=GO:0046006	feature_id[364].value <= threshold=4.9985432624816895
node_14: feature_name=GO:0070424	feature_id[254].value <= threshold=6.233297109603882
node_15: feature_name=GO:0009892	feature_id[320].value <= threshold=59.99180793762207
node_16: feature_name=GO:0007064	feature_id[527].value <= threshold=11.57377815246582
node_17: feature_name=GO:0005575	feature_id[17].value <= threshold=15.417460918426514
node_18: feature_name=GO:0043368	feature_id[31].value <= threshold=4.059496641159058
node_19: feature_name=GO:0005164	feature_id[163].value <= threshold=5.793607711791992
node_20: feature_name=GO:0042130	feature_id[16].value <= threshold=5.073179721832275
node_21: feature_name=GO:0010216	feature_id[282].value <= threshold=4.834584474563599
node_22: feature_name=GO:0009628	feature_id[553].value <= threshold=29.314892768859863
node_23: feature_name=GO:0045628	feature_id[749].value <= threshold=3.1001367568969727
node_24: feature_name=GO:0042288	feature_id[100].value <= threshold=5.174932956695557
node_25: feature_name=GO:0002329	feature_id[406].value <= threshold=2.348930835723877
node_26: feature_name=GO:0051246	feature_id[642].value > threshold=0.6498909294605255
node_194: feature_name=GO:0009164	feature_id[546].value <= threshold=17.11970329284668
node_195: feature_name=GO:0001909	feature_id[386].value <= threshold=7.292566299438477
node_196: feature_name=GO:0003908	feature_id[11].value <= threshold=1.887226164340973
node_197: feature_name=GO:0009164	feature_id[546].value <= threshold=0.26642198860645294
node_198: feature_name=GO:2001242	feature_id[24].value <= threshold=9.207026481628418
node_199: feature_name=GO:0051246	feature_id[642].value > threshold=0.6504445374011993
node_203: feature_name=GO:0032461	feature_id[608].value <= threshold=2.607542634010315
node_204: feature_name=GO:0007584	feature_id[529].value <= threshold=16.099515914916992
node_205: feature_name=hsa05221	feature_id[349].value <= threshold=9.359850406646729
node_206: feature_name=GO:0002636	feature_id[447].value <= threshold=1.615447759628296
node_207: feature_name=GO:0033993	feature_id[421].value > threshold=2.96540611088858e-05
node_217: feature_name=GO:0002331	feature_id[404].value <= threshold=2.1883513927459717
node_218: feature_name=GO:0046498	feature_id[203].value <= threshold=4.085246205329895
node_219: feature_name=GO:0002832	feature_id[491].value <= threshold=3.0228612422943115

node_220: feature_name=GO:1904868 feature_id[306].value <= threshold=3.8674756288528442
node_221: feature_name=GO:0048545 feature_id[360].value <= threshold=27.38136100769043
node_222: feature_name=GO:0071310 feature_id[760].value <= threshold=2.3522024154663086
node_223: feature_name=GO:0001775 feature_id[505].value <= threshold=9.71301555633545
node_224: feature_name=GO:0071310 feature_id[760].value <= threshold=2.346743583679199
node_225: feature_name=GO:0001772 feature_id[91].value <= threshold=3.252573609352112
node_226: feature_name=GO:0036498 feature_id[359].value <= threshold=12.092026710510254
node_227: feature_name=hsa05340 feature_id[351].value <= threshold=0.7677814364433289
node_228: feature_name=GO:0032703 feature_id[617].value <= threshold=1.7450646758079529
node_229: feature_name=hsa04640 feature_id[79].value <= threshold=2.118402123451233
node_230: feature_name=GO:0042097 feature_id[675].value > threshold=1.8110727667808533
node_272: feature_name=GO:0031667 feature_id[648].value <= threshold=0.26628243178129196
Class: negative genes

Rules_430 passed counts:2
node_0: feature_name=GO:0042113 feature_id[0].value <= threshold=13.408552169799805
node_1: feature_name=GO:0007568 feature_id[534].value <= threshold=5.0313897132873535
node_2: feature_name=GO:0002705 feature_id[541].value <= threshold=3.200145721435547
node_3: feature_name=GO:1901525 feature_id[576].value <= threshold=0.4466460347175598
node_4: feature_name=GO:0048539 feature_id[319].value <= threshold=3.0399646759033203
node_5: feature_name=GO:0001910 feature_id[385].value <= threshold=3.7437864542007446
node_6: feature_name=GO:0043200 feature_id[706].value <= threshold=9.307284355163574
node_7: feature_name=GO:0001773 feature_id[308].value <= threshold=4.016931533813477
node_8: feature_name=GO:0090116 feature_id[97].value <= threshold=7.99645471572876
node_9: feature_name=GO:0019814 feature_id[189].value <= threshold=4.525782108306885
node_10: feature_name=GO:1902583 feature_id[215].value <= threshold=17.60310649871826
node_11: feature_name=GO:0045429 feature_id[737].value <= threshold=1.6133361458778381
node_12: feature_name=GO:0003720 feature_id[228].value <= threshold=5.519326210021973
node_13: feature_name=GO:0046006 feature_id[364].value <= threshold=4.9985432624816895
node_14: feature_name=GO:0070424 feature_id[254].value <= threshold=6.233297109603882
node_15: feature_name=GO:0009892 feature_id[320].value <= threshold=59.99180793762207
node_16: feature_name=GO:0007064 feature_id[527].value <= threshold=11.57377815246582
node_17: feature_name=GO:0005575 feature_id[17].value <= threshold=15.417460918426514
node_18: feature_name=GO:0043368 feature_id[31].value <= threshold=4.059496641159058
node_19: feature_name=GO:0005164 feature_id[163].value <= threshold=5.793607711791992

node_20: feature_name=GO:0042130 feature_id[16].value <= threshold=5.073179721832275
node_21: feature_name=GO:0010216 feature_id[282].value <= threshold=4.834584474563599
node_22: feature_name=GO:0009628 feature_id[553].value <= threshold=29.314892768859863
node_23: feature_name=GO:0045628 feature_id[749].value <= threshold=3.1001367568969727
node_24: feature_name=GO:0042288 feature_id[100].value <= threshold=5.174932956695557
node_25: feature_name=GO:0002329 feature_id[406].value <= threshold=2.348930835723877
node_26: feature_name=GO:0051246 feature_id[642].value > threshold=0.6498909294605255
node_194: feature_name=GO:0009164 feature_id[546].value <= threshold=17.11970329284668
node_195: feature_name=GO:0001909 feature_id[386].value <= threshold=7.292566299438477
node_196: feature_name=GO:0003908 feature_id[11].value <= threshold=1.887226164340973
node_197: feature_name=GO:0009164 feature_id[546].value <= threshold=0.26642198860645294
node_198: feature_name=GO:2001242 feature_id[24].value <= threshold=9.207026481628418
node_199: feature_name=GO:0051246 feature_id[642].value > threshold=0.6504445374011993
node_203: feature_name=GO:0032461 feature_id[608].value <= threshold=2.607542634010315
node_204: feature_name=GO:0007584 feature_id[529].value <= threshold=16.099515914916992
node_205: feature_name=hsa05221 feature_id[349].value <= threshold=9.359850406646729
node_206: feature_name=GO:0002636 feature_id[447].value <= threshold=1.615447759628296
node_207: feature_name=GO:0033993 feature_id[421].value > threshold=2.96540611088858e-05
node_217: feature_name=GO:0002331 feature_id[404].value <= threshold=2.1883513927459717
node_218: feature_name=GO:0046498 feature_id[203].value <= threshold=4.085246205329895
node_219: feature_name=GO:0002832 feature_id[491].value <= threshold=3.0228612422943115
node_220: feature_name=GO:1904868 feature_id[306].value <= threshold=3.8674756288528442
node_221: feature_name=GO:0048545 feature_id[360].value <= threshold=27.38136100769043
node_222: feature_name=GO:0071310 feature_id[760].value <= threshold=2.3522024154663086
node_223: feature_name=GO:0001775 feature_id[505].value <= threshold=9.71301555633545
node_224: feature_name=GO:0071310 feature_id[760].value <= threshold=2.346743583679199
node_225: feature_name=GO:0001772 feature_id[91].value <= threshold=3.252573609352112
node_226: feature_name=GO:0036498 feature_id[359].value <= threshold=12.092026710510254
node_227: feature_name=hsa05340 feature_id[351].value <= threshold=0.7677814364433289
node_228: feature_name=GO:0032703 feature_id[617].value <= threshold=1.7450646758079529
node_229: feature_name=hsa04640 feature_id[79].value <= threshold=2.118402123451233
node_230: feature_name=GO:0042097 feature_id[675].value <= threshold=1.8110727667808533
node_231: feature_name=GO:0071391 feature_id[518].value > threshold=2.691588521003723
node_269: feature_name=GO:0010952 feature_id[483].value <= threshold=1.3165376782417297
Class: negative genes

Rules_431	passed counts:2
node_0: feature_name=GO:0042113	feature_id[0].value <= threshold=13.408552169799805
node_1: feature_name=GO:0007568	feature_id[534].value <= threshold=5.0313897132873535
node_2: feature_name=GO:0002705	feature_id[541].value <= threshold=3.200145721435547
node_3: feature_name=GO:1901525	feature_id[576].value <= threshold=0.4466460347175598
node_4: feature_name=GO:0048539	feature_id[319].value <= threshold=3.0399646759033203
node_5: feature_name=GO:0001910	feature_id[385].value <= threshold=3.7437864542007446
node_6: feature_name=GO:0043200	feature_id[706].value <= threshold=9.307284355163574
node_7: feature_name=GO:0001773	feature_id[308].value <= threshold=4.016931533813477
node_8: feature_name=GO:0090116	feature_id[97].value <= threshold=7.99645471572876
node_9: feature_name=GO:0019814	feature_id[189].value <= threshold=4.525782108306885
node_10: feature_name=GO:1902583	feature_id[215].value <= threshold=17.60310649871826
node_11: feature_name=GO:0045429	feature_id[737].value <= threshold=1.6133361458778381
node_12: feature_name=GO:0003720	feature_id[228].value <= threshold=5.519326210021973
node_13: feature_name=GO:0046006	feature_id[364].value <= threshold=4.9985432624816895
node_14: feature_name=GO:0070424	feature_id[254].value <= threshold=6.233297109603882
node_15: feature_name=GO:0009892	feature_id[320].value <= threshold=59.99180793762207
node_16: feature_name=GO:0007064	feature_id[527].value <= threshold=11.57377815246582
node_17: feature_name=GO:0005575	feature_id[17].value <= threshold=15.417460918426514
node_18: feature_name=GO:0043368	feature_id[31].value <= threshold=4.059496641159058
node_19: feature_name=GO:0005164	feature_id[163].value <= threshold=5.793607711791992
node_20: feature_name=GO:0042130	feature_id[16].value <= threshold=5.073179721832275
node_21: feature_name=GO:0010216	feature_id[282].value <= threshold=4.834584474563599
node_22: feature_name=GO:0009628	feature_id[553].value <= threshold=29.314892768859863
node_23: feature_name=GO:0045628	feature_id[749].value <= threshold=3.1001367568969727
node_24: feature_name=GO:0042288	feature_id[100].value <= threshold=5.174932956695557
node_25: feature_name=GO:0002329	feature_id[406].value <= threshold=2.348930835723877
node_26: feature_name=GO:0051246	feature_id[642].value > threshold=0.6498909294605255
node_194: feature_name=GO:0009164	feature_id[546].value <= threshold=17.11970329284668
node_195: feature_name=GO:0001909	feature_id[386].value <= threshold=7.292566299438477
node_196: feature_name=GO:0003908	feature_id[11].value <= threshold=1.887226164340973
node_197: feature_name=GO:0009164	feature_id[546].value <= threshold=0.26642198860645294
node_198: feature_name=GO:2001242	feature_id[24].value <= threshold=9.207026481628418
node_199: feature_name=GO:0051246	feature_id[642].value > threshold=0.6504445374011993

node_203: feature_name=GO:0032461 feature_id[608].value <= threshold=2.607542634010315
node_204: feature_name=GO:0007584 feature_id[529].value <= threshold=16.099515914916992
node_205: feature_name=hsa05221 feature_id[349].value <= threshold=9.359850406646729
node_206: feature_name=GO:0002636 feature_id[447].value <= threshold=1.615447759628296
node_207: feature_name=GO:0033993 feature_id[421].value <= threshold=2.96540611088858e-05
node_208: feature_name=GO:0006968 feature_id[517].value > threshold=1.0216356217861176
Class: positive genes

Rules_432 passed counts:2
node_0: feature_name=GO:0042113 feature_id[0].value <= threshold=13.408552169799805
node_1: feature_name=GO:0007568 feature_id[534].value <= threshold=5.0313897132873535
node_2: feature_name=GO:0002705 feature_id[541].value <= threshold=3.200145721435547
node_3: feature_name=GO:1901525 feature_id[576].value <= threshold=0.4466460347175598
node_4: feature_name=GO:0048539 feature_id[319].value <= threshold=3.0399646759033203
node_5: feature_name=GO:0001910 feature_id[385].value <= threshold=3.7437864542007446
node_6: feature_name=GO:0043200 feature_id[706].value <= threshold=9.307284355163574
node_7: feature_name=GO:0001773 feature_id[308].value <= threshold=4.016931533813477
node_8: feature_name=GO:0090116 feature_id[97].value <= threshold=7.99645471572876
node_9: feature_name=GO:0019814 feature_id[189].value <= threshold=4.525782108306885
node_10: feature_name=GO:1902583 feature_id[215].value <= threshold=17.60310649871826
node_11: feature_name=GO:0045429 feature_id[737].value <= threshold=1.6133361458778381
node_12: feature_name=GO:0003720 feature_id[228].value <= threshold=5.519326210021973
node_13: feature_name=GO:0046006 feature_id[364].value <= threshold=4.9985432624816895
node_14: feature_name=GO:0070424 feature_id[254].value <= threshold=6.233297109603882
node_15: feature_name=GO:0009892 feature_id[320].value <= threshold=59.99180793762207
node_16: feature_name=GO:0007064 feature_id[527].value <= threshold=11.57377815246582
node_17: feature_name=GO:0005575 feature_id[17].value <= threshold=15.417460918426514
node_18: feature_name=GO:0043368 feature_id[31].value <= threshold=4.059496641159058
node_19: feature_name=GO:0005164 feature_id[163].value <= threshold=5.793607711791992
node_20: feature_name=GO:0042130 feature_id[16].value <= threshold=5.073179721832275
node_21: feature_name=GO:0010216 feature_id[282].value <= threshold=4.834584474563599
node_22: feature_name=GO:0009628 feature_id[553].value <= threshold=29.314892768859863
node_23: feature_name=GO:0045628 feature_id[749].value <= threshold=3.1001367568969727
node_24: feature_name=GO:0042288 feature_id[100].value <= threshold=5.174932956695557
node_25: feature_name=GO:0002329 feature_id[406].value <= threshold=2.348930835723877

node_26: feature_name=GO:0051246 feature_id[642].value > threshold=0.6498909294605255
node_194: feature_name=GO:0009164 feature_id[546].value <= threshold=17.11970329284668
node_195: feature_name=GO:0001909 feature_id[386].value <= threshold=7.292566299438477
node_196: feature_name=GO:0003908 feature_id[11].value <= threshold=1.887226164340973
node_197: feature_name=GO:0009164 feature_id[546].value <= threshold=0.26642198860645294
node_198: feature_name=GO:2001242 feature_id[24].value <= threshold=9.207026481628418
node_199: feature_name=GO:0051246 feature_id[642].value > threshold=0.6504445374011993
node_203: feature_name=GO:0032461 feature_id[608].value <= threshold=2.607542634010315
node_204: feature_name=GO:0007584 feature_id[529].value <= threshold=16.099515914916992
node_205: feature_name=hsa05221 feature_id[349].value <= threshold=9.359850406646729
node_206: feature_name=GO:0002636 feature_id[447].value <= threshold=1.615447759628296
node_207: feature_name=GO:0033993 feature_id[421].value <= threshold=2.96540611088858e-05
node_208: feature_name=GO:0006968 feature_id[517].value <= threshold=1.0216356217861176
node_209: feature_name=GO:0048545 feature_id[360].value > threshold=0.007158883148804307
Class: positive genes

Rules_433 passed counts:2
node_0: feature_name=GO:0042113 feature_id[0].value <= threshold=13.408552169799805
node_1: feature_name=GO:0007568 feature_id[534].value <= threshold=5.0313897132873535
node_2: feature_name=GO:0002705 feature_id[541].value <= threshold=3.200145721435547
node_3: feature_name=GO:1901525 feature_id[576].value <= threshold=0.4466460347175598
node_4: feature_name=GO:0048539 feature_id[319].value <= threshold=3.0399646759033203
node_5: feature_name=GO:0001910 feature_id[385].value <= threshold=3.7437864542007446
node_6: feature_name=GO:0043200 feature_id[706].value <= threshold=9.307284355163574
node_7: feature_name=GO:0001773 feature_id[308].value <= threshold=4.016931533813477
node_8: feature_name=GO:0090116 feature_id[97].value <= threshold=7.99645471572876
node_9: feature_name=GO:0019814 feature_id[189].value <= threshold=4.525782108306885
node_10: feature_name=GO:1902583 feature_id[215].value <= threshold=17.60310649871826
node_11: feature_name=GO:0045429 feature_id[737].value <= threshold=1.6133361458778381
node_12: feature_name=GO:0003720 feature_id[228].value <= threshold=5.519326210021973
node_13: feature_name=GO:0046006 feature_id[364].value <= threshold=4.9985432624816895
node_14: feature_name=GO:0070424 feature_id[254].value <= threshold=6.233297109603882
node_15: feature_name=GO:0009892 feature_id[320].value <= threshold=59.99180793762207
node_16: feature_name=GO:0007064 feature_id[527].value <= threshold=11.57377815246582
node_17: feature_name=GO:0005575 feature_id[17].value <= threshold=15.417460918426514

node_18: feature_name=GO:0043368 feature_id[31].value <= threshold=4.059496641159058
node_19: feature_name=GO:0005164 feature_id[163].value <= threshold=5.793607711791992
node_20: feature_name=GO:0042130 feature_id[16].value <= threshold=5.073179721832275
node_21: feature_name=GO:0010216 feature_id[282].value <= threshold=4.834584474563599
node_22: feature_name=GO:0009628 feature_id[553].value <= threshold=29.314892768859863
node_23: feature_name=GO:0045628 feature_id[749].value <= threshold=3.1001367568969727
node_24: feature_name=GO:0042288 feature_id[100].value <= threshold=5.174932956695557
node_25: feature_name=GO:0002329 feature_id[406].value <= threshold=2.348930835723877
node_26: feature_name=GO:0051246 feature_id[642].value <= threshold=0.6498909294605255
node_27: feature_name=GO:0042162 feature_id[355].value <= threshold=8.05103588104248
node_28: feature_name=hsa04668 feature_id[333].value <= threshold=5.614926338195801
node_29: feature_name=GO:0032200 feature_id[613].value <= threshold=28.735913276672363
node_30: feature_name=hsa04662 feature_id[334].value <= threshold=9.588344097137451
node_31: feature_name=GO:1901992 feature_id[105].value <= threshold=5.291427850723267
node_32: feature_name=GO:0072341 feature_id[144].value > threshold=6.01465106010437
node_180: feature_name=GO:0045621 feature_id[143].value > threshold=0.2295876294374466
Class: positive genes

Rules_434 passed counts:2
node_0: feature_name=GO:0042113 feature_id[0].value <= threshold=13.408552169799805
node_1: feature_name=GO:0007568 feature_id[534].value <= threshold=5.0313897132873535
node_2: feature_name=GO:0002705 feature_id[541].value <= threshold=3.200145721435547
node_3: feature_name=GO:1901525 feature_id[576].value <= threshold=0.4466460347175598
node_4: feature_name=GO:0048539 feature_id[319].value <= threshold=3.0399646759033203
node_5: feature_name=GO:0001910 feature_id[385].value <= threshold=3.7437864542007446
node_6: feature_name=GO:0043200 feature_id[706].value <= threshold=9.307284355163574
node_7: feature_name=GO:0001773 feature_id[308].value <= threshold=4.016931533813477
node_8: feature_name=GO:0090116 feature_id[97].value <= threshold=7.99645471572876
node_9: feature_name=GO:0019814 feature_id[189].value <= threshold=4.525782108306885
node_10: feature_name=GO:1902583 feature_id[215].value <= threshold=17.60310649871826
node_11: feature_name=GO:0045429 feature_id[737].value <= threshold=1.6133361458778381
node_12: feature_name=GO:0003720 feature_id[228].value <= threshold=5.519326210021973
node_13: feature_name=GO:0046006 feature_id[364].value <= threshold=4.9985432624816895
node_14: feature_name=GO:0070424 feature_id[254].value <= threshold=6.233297109603882
node_15: feature_name=GO:0009892 feature_id[320].value <= threshold=59.99180793762207

node_16: feature_name=GO:0007064 feature_id[527].value <= threshold=11.57377815246582
node_17: feature_name=GO:0005575 feature_id[17].value <= threshold=15.417460918426514
node_18: feature_name=GO:0043368 feature_id[31].value <= threshold=4.059496641159058
node_19: feature_name=GO:0005164 feature_id[163].value <= threshold=5.793607711791992
node_20: feature_name=GO:0042130 feature_id[16].value <= threshold=5.073179721832275
node_21: feature_name=GO:0010216 feature_id[282].value <= threshold=4.834584474563599
node_22: feature_name=GO:0009628 feature_id[553].value <= threshold=29.314892768859863
node_23: feature_name=GO:0045628 feature_id[749].value <= threshold=3.1001367568969727
node_24: feature_name=GO:0042288 feature_id[100].value <= threshold=5.174932956695557
node_25: feature_name=GO:0002329 feature_id[406].value <= threshold=2.348930835723877
node_26: feature_name=GO:0051246 feature_id[642].value <= threshold=0.6498909294605255
node_27: feature_name=GO:0042162 feature_id[355].value <= threshold=8.05103588104248
node_28: feature_name=hsa04668 feature_id[333].value <= threshold=5.614926338195801
node_29: feature_name=GO:0032200 feature_id[613].value <= threshold=28.735913276672363
node_30: feature_name=hsa04662 feature_id[334].value <= threshold=9.588344097137451
node_31: feature_name=GO:1901992 feature_id[105].value <= threshold=5.291427850723267
node_32: feature_name=GO:0072341 feature_id[144].value <= threshold=6.01465106010437
node_33: feature_name=GO:0047485 feature_id[121].value > threshold=6.657836437225342
node_177: feature_name=GO:0034097 feature_id[647].value > threshold=0.17321054253261536
Class: negative genes

Rules_435 passed counts:2
node_0: feature_name=GO:0042113 feature_id[0].value <= threshold=13.408552169799805
node_1: feature_name=GO:0007568 feature_id[534].value <= threshold=5.0313897132873535
node_2: feature_name=GO:0002705 feature_id[541].value <= threshold=3.200145721435547
node_3: feature_name=GO:1901525 feature_id[576].value <= threshold=0.4466460347175598
node_4: feature_name=GO:0048539 feature_id[319].value <= threshold=3.0399646759033203
node_5: feature_name=GO:0001910 feature_id[385].value <= threshold=3.7437864542007446
node_6: feature_name=GO:0043200 feature_id[706].value <= threshold=9.307284355163574
node_7: feature_name=GO:0001773 feature_id[308].value <= threshold=4.016931533813477
node_8: feature_name=GO:0090116 feature_id[97].value <= threshold=7.99645471572876
node_9: feature_name=GO:0019814 feature_id[189].value <= threshold=4.525782108306885
node_10: feature_name=GO:1902583 feature_id[215].value <= threshold=17.60310649871826
node_11: feature_name=GO:0045429 feature_id[737].value <= threshold=1.6133361458778381
node_12: feature_name=GO:0003720 feature_id[228].value <= threshold=5.519326210021973

node_13: feature_name=GO:0046006 feature_id[364].value <= threshold=4.9985432624816895
node_14: feature_name=GO:0070424 feature_id[254].value <= threshold=6.233297109603882
node_15: feature_name=GO:0009892 feature_id[320].value <= threshold=59.99180793762207
node_16: feature_name=GO:0007064 feature_id[527].value <= threshold=11.57377815246582
node_17: feature_name=GO:0005575 feature_id[17].value <= threshold=15.417460918426514
node_18: feature_name=GO:0043368 feature_id[31].value <= threshold=4.059496641159058
node_19: feature_name=GO:0005164 feature_id[163].value <= threshold=5.793607711791992
node_20: feature_name=GO:0042130 feature_id[16].value <= threshold=5.073179721832275
node_21: feature_name=GO:0010216 feature_id[282].value <= threshold=4.834584474563599
node_22: feature_name=GO:0009628 feature_id[553].value <= threshold=29.314892768859863
node_23: feature_name=GO:0045628 feature_id[749].value <= threshold=3.1001367568969727
node_24: feature_name=GO:0042288 feature_id[100].value <= threshold=5.174932956695557
node_25: feature_name=GO:0002329 feature_id[406].value <= threshold=2.348930835723877
node_26: feature_name=GO:0051246 feature_id[642].value <= threshold=0.6498909294605255
node_27: feature_name=GO:0042162 feature_id[355].value <= threshold=8.05103588104248
node_28: feature_name=hsa04668 feature_id[333].value <= threshold=5.614926338195801
node_29: feature_name=GO:0032200 feature_id[613].value <= threshold=28.735913276672363
node_30: feature_name=hsa04662 feature_id[334].value <= threshold=9.588344097137451
node_31: feature_name=GO:1901992 feature_id[105].value <= threshold=5.291427850723267
node_32: feature_name=GO:0072341 feature_id[144].value <= threshold=6.01465106010437
node_33: feature_name=GO:0047485 feature_id[121].value <= threshold=6.657836437225342
node_34: feature_name=GO:0090594 feature_id[738].value > threshold=1.8673912286758423
node_174: feature_name=GO:0010950 feature_id[567].value <= threshold=0.11882948130369186
Class: negative genes

Rules_436 passed counts:2
node_0: feature_name=GO:0042113 feature_id[0].value <= threshold=13.408552169799805
node_1: feature_name=GO:0007568 feature_id[534].value <= threshold=5.0313897132873535
node_2: feature_name=GO:0002705 feature_id[541].value <= threshold=3.200145721435547
node_3: feature_name=GO:1901525 feature_id[576].value <= threshold=0.4466460347175598
node_4: feature_name=GO:0048539 feature_id[319].value <= threshold=3.0399646759033203
node_5: feature_name=GO:0001910 feature_id[385].value <= threshold=3.7437864542007446
node_6: feature_name=GO:0043200 feature_id[706].value <= threshold=9.307284355163574
node_7: feature_name=GO:0001773 feature_id[308].value <= threshold=4.016931533813477
node_8: feature_name=GO:0090116 feature_id[97].value <= threshold=7.99645471572876

node_9: feature_name=GO:0019814 feature_id[189].value <= threshold=4.525782108306885
node_10: feature_name=GO:1902583 feature_id[215].value <= threshold=17.60310649871826
node_11: feature_name=GO:0045429 feature_id[737].value <= threshold=1.6133361458778381
node_12: feature_name=GO:0003720 feature_id[228].value <= threshold=5.519326210021973
node_13: feature_name=GO:0046006 feature_id[364].value <= threshold=4.9985432624816895
node_14: feature_name=GO:0070424 feature_id[254].value <= threshold=6.233297109603882
node_15: feature_name=GO:0009892 feature_id[320].value <= threshold=59.99180793762207
node_16: feature_name=GO:0007064 feature_id[527].value <= threshold=11.57377815246582
node_17: feature_name=GO:0005575 feature_id[17].value <= threshold=15.417460918426514
node_18: feature_name=GO:0043368 feature_id[31].value <= threshold=4.059496641159058
node_19: feature_name=GO:0005164 feature_id[163].value <= threshold=5.793607711791992
node_20: feature_name=GO:0042130 feature_id[16].value <= threshold=5.073179721832275
node_21: feature_name=GO:0010216 feature_id[282].value <= threshold=4.834584474563599
node_22: feature_name=GO:0009628 feature_id[553].value <= threshold=29.314892768859863
node_23: feature_name=GO:0045628 feature_id[749].value <= threshold=3.1001367568969727
node_24: feature_name=GO:0042288 feature_id[100].value <= threshold=5.174932956695557
node_25: feature_name=GO:0002329 feature_id[406].value <= threshold=2.348930835723877
node_26: feature_name=GO:0051246 feature_id[642].value <= threshold=0.6498909294605255
node_27: feature_name=GO:0042162 feature_id[355].value <= threshold=8.05103588104248
node_28: feature_name=hsa04668 feature_id[333].value <= threshold=5.614926338195801
node_29: feature_name=GO:0032200 feature_id[613].value <= threshold=28.735913276672363
node_30: feature_name=hsa04662 feature_id[334].value <= threshold=9.588344097137451
node_31: feature_name=GO:1901992 feature_id[105].value <= threshold=5.291427850723267
node_32: feature_name=GO:0072341 feature_id[144].value <= threshold=6.01465106010437
node_33: feature_name=GO:0047485 feature_id[121].value <= threshold=6.657836437225342
node_34: feature_name=GO:0090594 feature_id[738].value <= threshold=1.8673912286758423
node_35: feature_name=GO:0043525 feature_id[524].value > threshold=2.881397008895874
node_171: feature_name=GO:0007126 feature_id[520].value <= threshold=0.3579426407814026
Class: negative genes

Rules_437 passed counts:2
node_0: feature_name=GO:0042113 feature_id[0].value <= threshold=13.408552169799805
node_1: feature_name=GO:0007568 feature_id[534].value <= threshold=5.0313897132873535
node_2: feature_name=GO:0002705 feature_id[541].value <= threshold=3.200145721435547
node_3: feature_name=GO:1901525 feature_id[576].value <= threshold=0.4466460347175598

node_4: feature_name=GO:0048539 feature_id[319].value <= threshold=3.0399646759033203
node_5: feature_name=GO:0001910 feature_id[385].value <= threshold=3.7437864542007446
node_6: feature_name=GO:0043200 feature_id[706].value <= threshold=9.307284355163574
node_7: feature_name=GO:0001773 feature_id[308].value <= threshold=4.016931533813477
node_8: feature_name=GO:0090116 feature_id[97].value <= threshold=7.99645471572876
node_9: feature_name=GO:0019814 feature_id[189].value <= threshold=4.525782108306885
node_10: feature_name=GO:1902583 feature_id[215].value <= threshold=17.60310649871826
node_11: feature_name=GO:0045429 feature_id[737].value <= threshold=1.6133361458778381
node_12: feature_name=GO:0003720 feature_id[228].value <= threshold=5.519326210021973
node_13: feature_name=GO:0046006 feature_id[364].value <= threshold=4.9985432624816895
node_14: feature_name=GO:0070424 feature_id[254].value <= threshold=6.233297109603882
node_15: feature_name=GO:0009892 feature_id[320].value <= threshold=59.99180793762207
node_16: feature_name=GO:0007064 feature_id[527].value <= threshold=11.57377815246582
node_17: feature_name=GO:0005575 feature_id[17].value <= threshold=15.417460918426514
node_18: feature_name=GO:0043368 feature_id[31].value <= threshold=4.059496641159058
node_19: feature_name=GO:0005164 feature_id[163].value <= threshold=5.793607711791992
node_20: feature_name=GO:0042130 feature_id[16].value <= threshold=5.073179721832275
node_21: feature_name=GO:0010216 feature_id[282].value <= threshold=4.834584474563599
node_22: feature_name=GO:0009628 feature_id[553].value <= threshold=29.314892768859863
node_23: feature_name=GO:0045628 feature_id[749].value <= threshold=3.1001367568969727
node_24: feature_name=GO:0042288 feature_id[100].value <= threshold=5.174932956695557
node_25: feature_name=GO:0002329 feature_id[406].value <= threshold=2.348930835723877
node_26: feature_name=GO:0051246 feature_id[642].value <= threshold=0.6498909294605255
node_27: feature_name=GO:0042162 feature_id[355].value <= threshold=8.05103588104248
node_28: feature_name=hsa04668 feature_id[333].value <= threshold=5.614926338195801
node_29: feature_name=GO:0032200 feature_id[613].value <= threshold=28.735913276672363
node_30: feature_name=hsa04662 feature_id[334].value <= threshold=9.588344097137451
node_31: feature_name=GO:1901992 feature_id[105].value <= threshold=5.291427850723267
node_32: feature_name=GO:0072341 feature_id[144].value <= threshold=6.01465106010437
node_33: feature_name=GO:0047485 feature_id[121].value <= threshold=6.657836437225342
node_34: feature_name=GO:0090594 feature_id[738].value <= threshold=1.8673912286758423
node_35: feature_name=GO:0043525 feature_id[524].value <= threshold=2.881397008895874
node_36: feature_name=GO:0032504 feature_id[244].value <= threshold=17.22207546234131
node_37: feature_name=GO:0051348 feature_id[126].value <= threshold=5.933559417724609
node_38: feature_name=GO:0042522 feature_id[692].value > threshold=1.78694087266922

node_162: feature_name=GO:0006113 feature_id[497].value > threshold=1.119098722934723
Class: positive genes

Rules_438 passed counts:2
node_0: feature_name=GO:0042113 feature_id[0].value <= threshold=13.408552169799805
node_1: feature_name=GO:0007568 feature_id[534].value <= threshold=5.0313897132873535
node_2: feature_name=GO:0002705 feature_id[541].value <= threshold=3.200145721435547
node_3: feature_name=GO:1901525 feature_id[576].value <= threshold=0.4466460347175598
node_4: feature_name=GO:0048539 feature_id[319].value <= threshold=3.0399646759033203
node_5: feature_name=GO:0001910 feature_id[385].value <= threshold=3.7437864542007446
node_6: feature_name=GO:0043200 feature_id[706].value <= threshold=9.307284355163574
node_7: feature_name=GO:0001773 feature_id[308].value <= threshold=4.016931533813477
node_8: feature_name=GO:0090116 feature_id[97].value <= threshold=7.99645471572876
node_9: feature_name=GO:0019814 feature_id[189].value <= threshold=4.525782108306885
node_10: feature_name=GO:1902583 feature_id[215].value <= threshold=17.60310649871826
node_11: feature_name=GO:0045429 feature_id[737].value <= threshold=1.6133361458778381
node_12: feature_name=GO:0003720 feature_id[228].value <= threshold=5.519326210021973
node_13: feature_name=GO:0046006 feature_id[364].value <= threshold=4.9985432624816895
node_14: feature_name=GO:0070424 feature_id[254].value <= threshold=6.233297109603882
node_15: feature_name=GO:0009892 feature_id[320].value <= threshold=59.99180793762207
node_16: feature_name=GO:0007064 feature_id[527].value <= threshold=11.57377815246582
node_17: feature_name=GO:0005575 feature_id[17].value <= threshold=15.417460918426514
node_18: feature_name=GO:0043368 feature_id[31].value <= threshold=4.059496641159058
node_19: feature_name=GO:0005164 feature_id[163].value <= threshold=5.793607711791992
node_20: feature_name=GO:0042130 feature_id[16].value <= threshold=5.073179721832275
node_21: feature_name=GO:0010216 feature_id[282].value <= threshold=4.834584474563599
node_22: feature_name=GO:0009628 feature_id[553].value <= threshold=29.314892768859863
node_23: feature_name=GO:0045628 feature_id[749].value <= threshold=3.1001367568969727
node_24: feature_name=GO:0042288 feature_id[100].value <= threshold=5.174932956695557
node_25: feature_name=GO:0002329 feature_id[406].value <= threshold=2.348930835723877
node_26: feature_name=GO:0051246 feature_id[642].value <= threshold=0.6498909294605255
node_27: feature_name=GO:0042162 feature_id[355].value <= threshold=8.05103588104248
node_28: feature_name=hsa04668 feature_id[333].value <= threshold=5.614926338195801
node_29: feature_name=GO:0032200 feature_id[613].value <= threshold=28.735913276672363
node_30: feature_name=hsa04662 feature_id[334].value <= threshold=9.588344097137451

node_31: feature_name=GO:1901992 feature_id[105].value <= threshold=5.291427850723267
node_32: feature_name=GO:0072341 feature_id[144].value <= threshold=6.01465106010437
node_33: feature_name=GO:0047485 feature_id[121].value <= threshold=6.657836437225342
node_34: feature_name=GO:0090594 feature_id[738].value <= threshold=1.8673912286758423
node_35: feature_name=GO:0043525 feature_id[524].value <= threshold=2.881397008895874
node_36: feature_name=GO:0032504 feature_id[244].value <= threshold=17.22207546234131
node_37: feature_name=GO:0051348 feature_id[126].value <= threshold=5.933559417724609
node_38: feature_name=GO:0042522 feature_id[692].value <= threshold=1.78694087266922
node_39: feature_name=GO:0031100 feature_id[73].value > threshold=3.2312101125717163
node_157: feature_name=GO:0002376 feature_id[277].value > threshold=0.046988584101200104
node_159: feature_name=GO:0050794 feature_id[108].value <= threshold=0.21684371432638727
Class: positive genes

Rules_439 passed counts:2
node_0: feature_name=GO:0042113 feature_id[0].value <= threshold=13.408552169799805
node_1: feature_name=GO:0007568 feature_id[534].value <= threshold=5.0313897132873535
node_2: feature_name=GO:0002705 feature_id[541].value <= threshold=3.200145721435547
node_3: feature_name=GO:1901525 feature_id[576].value <= threshold=0.4466460347175598
node_4: feature_name=GO:0048539 feature_id[319].value <= threshold=3.0399646759033203
node_5: feature_name=GO:0001910 feature_id[385].value <= threshold=3.7437864542007446
node_6: feature_name=GO:0043200 feature_id[706].value <= threshold=9.307284355163574
node_7: feature_name=GO:0001773 feature_id[308].value <= threshold=4.016931533813477
node_8: feature_name=GO:0090116 feature_id[97].value <= threshold=7.99645471572876
node_9: feature_name=GO:0019814 feature_id[189].value <= threshold=4.525782108306885
node_10: feature_name=GO:1902583 feature_id[215].value <= threshold=17.60310649871826
node_11: feature_name=GO:0045429 feature_id[737].value <= threshold=1.6133361458778381
node_12: feature_name=GO:0003720 feature_id[228].value <= threshold=5.519326210021973
node_13: feature_name=GO:0046006 feature_id[364].value <= threshold=4.9985432624816895
node_14: feature_name=GO:0070424 feature_id[254].value <= threshold=6.233297109603882
node_15: feature_name=GO:0009892 feature_id[320].value <= threshold=59.99180793762207
node_16: feature_name=GO:0007064 feature_id[527].value <= threshold=11.57377815246582
node_17: feature_name=GO:0005575 feature_id[17].value <= threshold=15.417460918426514
node_18: feature_name=GO:0043368 feature_id[31].value <= threshold=4.059496641159058
node_19: feature_name=GO:0005164 feature_id[163].value <= threshold=5.793607711791992
node_20: feature_name=GO:0042130 feature_id[16].value <= threshold=5.073179721832275

node_21: feature_name=GO:0010216	feature_id[282].value <= threshold=4.834584474563599
node_22: feature_name=GO:0009628	feature_id[553].value <= threshold=29.314892768859863
node_23: feature_name=GO:0045628	feature_id[749].value <= threshold=3.1001367568969727
node_24: feature_name=GO:0042288	feature_id[100].value <= threshold=5.174932956695557
node_25: feature_name=GO:0002329	feature_id[406].value <= threshold=2.348930835723877
node_26: feature_name=GO:0051246	feature_id[642].value <= threshold=0.6498909294605255
node_27: feature_name=GO:0042162	feature_id[355].value <= threshold=8.05103588104248
node_28: feature_name=hsa04668	feature_id[333].value <= threshold=5.614926338195801
node_29: feature_name=GO:0032200	feature_id[613].value <= threshold=28.735913276672363
node_30: feature_name=hsa04662	feature_id[334].value <= threshold=9.588344097137451
node_31: feature_name=GO:1901992	feature_id[105].value <= threshold=5.291427850723267
node_32: feature_name=GO:0072341	feature_id[144].value <= threshold=6.01465106010437
node_33: feature_name=GO:0047485	feature_id[121].value <= threshold=6.657836437225342
node_34: feature_name=GO:0090594	feature_id[738].value <= threshold=1.8673912286758423
node_35: feature_name=GO:0043525	feature_id[524].value <= threshold=2.881397008895874
node_36: feature_name=GO:0032504	feature_id[244].value <= threshold=17.22207546234131
node_37: feature_name=GO:0051348	feature_id[126].value <= threshold=5.933559417724609
node_38: feature_name=GO:0042522	feature_id[692].value <= threshold=1.78694087266922
node_39: feature_name=GO:0031100	feature_id[73].value <= threshold=3.2312101125717163
node_40: feature_name=GO:0042493	feature_id[149].value > threshold=19.771170616149902
node_154: feature_name=GO:0002252	feature_id[460].value <= threshold=0.05236339755356312
Class: negative genes	

Rules_440	passed counts:2
node_0: feature_name=GO:0042113	feature_id[0].value <= threshold=13.408552169799805
node_1: feature_name=GO:0007568	feature_id[534].value <= threshold=5.0313897132873535
node_2: feature_name=GO:0002705	feature_id[541].value <= threshold=3.200145721435547
node_3: feature_name=GO:1901525	feature_id[576].value <= threshold=0.4466460347175598
node_4: feature_name=GO:0048539	feature_id[319].value <= threshold=3.0399646759033203
node_5: feature_name=GO:0001910	feature_id[385].value <= threshold=3.7437864542007446
node_6: feature_name=GO:0043200	feature_id[706].value <= threshold=9.307284355163574
node_7: feature_name=GO:0001773	feature_id[308].value <= threshold=4.016931533813477
node_8: feature_name=GO:0090116	feature_id[97].value <= threshold=7.99645471572876
node_9: feature_name=GO:0019814	feature_id[189].value <= threshold=4.525782108306885
node_10: feature_name=GO:1902583	feature_id[215].value <= threshold=17.60310649871826

node_11: feature_name=GO:0045429	feature_id[737].value <= threshold=1.6133361458778381
node_12: feature_name=GO:0003720	feature_id[228].value <= threshold=5.519326210021973
node_13: feature_name=GO:0046006	feature_id[364].value <= threshold=4.9985432624816895
node_14: feature_name=GO:0070424	feature_id[254].value <= threshold=6.233297109603882
node_15: feature_name=GO:0009892	feature_id[320].value <= threshold=59.99180793762207
node_16: feature_name=GO:0007064	feature_id[527].value <= threshold=11.57377815246582
node_17: feature_name=GO:0005575	feature_id[17].value <= threshold=15.417460918426514
node_18: feature_name=GO:0043368	feature_id[31].value <= threshold=4.059496641159058
node_19: feature_name=GO:0005164	feature_id[163].value <= threshold=5.793607711791992
node_20: feature_name=GO:0042130	feature_id[16].value <= threshold=5.073179721832275
node_21: feature_name=GO:0010216	feature_id[282].value <= threshold=4.834584474563599
node_22: feature_name=GO:0009628	feature_id[553].value <= threshold=29.314892768859863
node_23: feature_name=GO:0045628	feature_id[749].value <= threshold=3.1001367568969727
node_24: feature_name=GO:0042288	feature_id[100].value <= threshold=5.174932956695557
node_25: feature_name=GO:0002329	feature_id[406].value <= threshold=2.348930835723877
node_26: feature_name=GO:0051246	feature_id[642].value <= threshold=0.6498909294605255
node_27: feature_name=GO:0042162	feature_id[355].value <= threshold=8.05103588104248
node_28: feature_name=hsa04668	feature_id[333].value <= threshold=5.614926338195801
node_29: feature_name=GO:0032200	feature_id[613].value <= threshold=28.735913276672363
node_30: feature_name=hsa04662	feature_id[334].value <= threshold=9.588344097137451
node_31: feature_name=GO:1901992	feature_id[105].value <= threshold=5.291427850723267
node_32: feature_name=GO:0072341	feature_id[144].value <= threshold=6.01465106010437
node_33: feature_name=GO:0047485	feature_id[121].value <= threshold=6.657836437225342
node_34: feature_name=GO:0090594	feature_id[738].value <= threshold=1.8673912286758423
node_35: feature_name=GO:0043525	feature_id[524].value <= threshold=2.881397008895874
node_36: feature_name=GO:0032504	feature_id[244].value <= threshold=17.22207546234131
node_37: feature_name=GO:0051348	feature_id[126].value <= threshold=5.933559417724609
node_38: feature_name=GO:0042522	feature_id[692].value <= threshold=1.78694087266922
node_39: feature_name=GO:0031100	feature_id[73].value <= threshold=3.2312101125717163
node_40: feature_name=GO:0042493	feature_id[149].value <= threshold=19.771170616149902
node_41: feature_name=GO:0001552	feature_id[366].value <= threshold=2.1711617708206177
node_42: feature_name=GO:0046483	feature_id[391].value <= threshold=320.9667053222656
node_43: feature_name=GO:0002439	feature_id[431].value <= threshold=1.832722783088684
node_44: feature_name=GO:0006555	feature_id[514].value > threshold=5.321640968322754
node_142: feature_name=GO:0006555	feature_id[514].value <= threshold=6.031885147094727

Class: positive genes

Rules_441	passed counts:2
node_0: feature_name=GO:0042113	feature_id[0].value <= threshold=13.408552169799805
node_1: feature_name=GO:0007568	feature_id[534].value <= threshold=5.0313897132873535
node_2: feature_name=GO:0002705	feature_id[541].value <= threshold=3.200145721435547
node_3: feature_name=GO:1901525	feature_id[576].value <= threshold=0.4466460347175598
node_4: feature_name=GO:0048539	feature_id[319].value <= threshold=3.0399646759033203
node_5: feature_name=GO:0001910	feature_id[385].value <= threshold=3.7437864542007446
node_6: feature_name=GO:0043200	feature_id[706].value <= threshold=9.307284355163574
node_7: feature_name=GO:0001773	feature_id[308].value <= threshold=4.016931533813477
node_8: feature_name=GO:0090116	feature_id[97].value <= threshold=7.99645471572876
node_9: feature_name=GO:0019814	feature_id[189].value <= threshold=4.525782108306885
node_10: feature_name=GO:1902583	feature_id[215].value <= threshold=17.60310649871826
node_11: feature_name=GO:0045429	feature_id[737].value <= threshold=1.6133361458778381
node_12: feature_name=GO:0003720	feature_id[228].value <= threshold=5.519326210021973
node_13: feature_name=GO:0046006	feature_id[364].value <= threshold=4.9985432624816895
node_14: feature_name=GO:0070424	feature_id[254].value <= threshold=6.233297109603882
node_15: feature_name=GO:0009892	feature_id[320].value <= threshold=59.99180793762207
node_16: feature_name=GO:0007064	feature_id[527].value <= threshold=11.57377815246582
node_17: feature_name=GO:0005575	feature_id[17].value <= threshold=15.417460918426514
node_18: feature_name=GO:0043368	feature_id[31].value <= threshold=4.059496641159058
node_19: feature_name=GO:0005164	feature_id[163].value <= threshold=5.793607711791992
node_20: feature_name=GO:0042130	feature_id[16].value <= threshold=5.073179721832275
node_21: feature_name=GO:0010216	feature_id[282].value <= threshold=4.834584474563599
node_22: feature_name=GO:0009628	feature_id[553].value <= threshold=29.314892768859863
node_23: feature_name=GO:0045628	feature_id[749].value <= threshold=3.1001367568969727
node_24: feature_name=GO:0042288	feature_id[100].value <= threshold=5.174932956695557
node_25: feature_name=GO:0002329	feature_id[406].value <= threshold=2.348930835723877
node_26: feature_name=GO:0051246	feature_id[642].value <= threshold=0.6498909294605255
node_27: feature_name=GO:0042162	feature_id[355].value <= threshold=8.05103588104248
node_28: feature_name=hsa04668	feature_id[333].value <= threshold=5.614926338195801
node_29: feature_name=GO:0032200	feature_id[613].value <= threshold=28.735913276672363
node_30: feature_name=hsa04662	feature_id[334].value <= threshold=9.588344097137451
node_31: feature_name=GO:1901992	feature_id[105].value <= threshold=5.291427850723267

node_32: feature_name=GO:0072341 feature_id[144].value <= threshold=6.01465106010437
node_33: feature_name=GO:0047485 feature_id[121].value <= threshold=6.657836437225342
node_34: feature_name=GO:0090594 feature_id[738].value <= threshold=1.8673912286758423
node_35: feature_name=GO:0043525 feature_id[524].value <= threshold=2.881397008895874
node_36: feature_name=GO:0032504 feature_id[244].value <= threshold=17.22207546234131
node_37: feature_name=GO:0051348 feature_id[126].value <= threshold=5.933559417724609
node_38: feature_name=GO:0042522 feature_id[692].value <= threshold=1.78694087266922
node_39: feature_name=GO:0031100 feature_id[73].value <= threshold=3.2312101125717163
node_40: feature_name=GO:0042493 feature_id[149].value <= threshold=19.771170616149902
node_41: feature_name=GO:0001552 feature_id[366].value <= threshold=2.1711617708206177
node_42: feature_name=GO:0046483 feature_id[391].value <= threshold=320.9667053222656
node_43: feature_name=GO:0002439 feature_id[431].value <= threshold=1.832722783088684
node_44: feature_name=GO:0006555 feature_id[514].value <= threshold=5.321640968322754
node_45: feature_name=GO:0070424 feature_id[254].value <= threshold=3.321003556251526
node_46: feature_name=hsa05144 feature_id[751].value > threshold=2.635499954223633
node_134: feature_name=GO:0010165 feature_id[330].value > threshold=0.49422845244407654
node_136: feature_name=GO:0010955 feature_id[2].value > threshold=0.3874783217906952
Class: positive genes

Rules_442 passed counts:2
node_0: feature_name=GO:0042113 feature_id[0].value <= threshold=13.408552169799805
node_1: feature_name=GO:0007568 feature_id[534].value <= threshold=5.0313897132873535
node_2: feature_name=GO:0002705 feature_id[541].value <= threshold=3.200145721435547
node_3: feature_name=GO:1901525 feature_id[576].value <= threshold=0.4466460347175598
node_4: feature_name=GO:0048539 feature_id[319].value <= threshold=3.0399646759033203
node_5: feature_name=GO:0001910 feature_id[385].value <= threshold=3.7437864542007446
node_6: feature_name=GO:0043200 feature_id[706].value <= threshold=9.307284355163574
node_7: feature_name=GO:0001773 feature_id[308].value <= threshold=4.016931533813477
node_8: feature_name=GO:0090116 feature_id[97].value <= threshold=7.99645471572876
node_9: feature_name=GO:0019814 feature_id[189].value <= threshold=4.525782108306885
node_10: feature_name=GO:1902583 feature_id[215].value <= threshold=17.60310649871826
node_11: feature_name=GO:0045429 feature_id[737].value <= threshold=1.6133361458778381
node_12: feature_name=GO:0003720 feature_id[228].value <= threshold=5.519326210021973
node_13: feature_name=GO:0046006 feature_id[364].value <= threshold=4.9985432624816895
node_14: feature_name=GO:0070424 feature_id[254].value <= threshold=6.233297109603882

node_15: feature_name=GO:0009892	feature_id[320].value <= threshold=59.99180793762207
node_16: feature_name=GO:0007064	feature_id[527].value <= threshold=11.57377815246582
node_17: feature_name=GO:0005575	feature_id[17].value <= threshold=15.417460918426514
node_18: feature_name=GO:0043368	feature_id[31].value <= threshold=4.059496641159058
node_19: feature_name=GO:0005164	feature_id[163].value <= threshold=5.793607711791992
node_20: feature_name=GO:0042130	feature_id[16].value <= threshold=5.073179721832275
node_21: feature_name=GO:0010216	feature_id[282].value <= threshold=4.834584474563599
node_22: feature_name=GO:0009628	feature_id[553].value <= threshold=29.314892768859863
node_23: feature_name=GO:0045628	feature_id[749].value <= threshold=3.1001367568969727
node_24: feature_name=GO:0042288	feature_id[100].value <= threshold=5.174932956695557
node_25: feature_name=GO:0002329	feature_id[406].value <= threshold=2.348930835723877
node_26: feature_name=GO:0051246	feature_id[642].value <= threshold=0.6498909294605255
node_27: feature_name=GO:0042162	feature_id[355].value <= threshold=8.05103588104248
node_28: feature_name=hsa04668	feature_id[333].value <= threshold=5.614926338195801
node_29: feature_name=GO:0032200	feature_id[613].value <= threshold=28.735913276672363
node_30: feature_name=hsa04662	feature_id[334].value <= threshold=9.588344097137451
node_31: feature_name=GO:1901992	feature_id[105].value <= threshold=5.291427850723267
node_32: feature_name=GO:0072341	feature_id[144].value <= threshold=6.01465106010437
node_33: feature_name=GO:0047485	feature_id[121].value <= threshold=6.657836437225342
node_34: feature_name=GO:0090594	feature_id[738].value <= threshold=1.8673912286758423
node_35: feature_name=GO:0043525	feature_id[524].value <= threshold=2.881397008895874
node_36: feature_name=GO:0032504	feature_id[244].value <= threshold=17.22207546234131
node_37: feature_name=GO:0051348	feature_id[126].value <= threshold=5.933559417724609
node_38: feature_name=GO:0042522	feature_id[692].value <= threshold=1.78694087266922
node_39: feature_name=GO:0031100	feature_id[73].value <= threshold=3.2312101125717163
node_40: feature_name=GO:0042493	feature_id[149].value <= threshold=19.771170616149902
node_41: feature_name=GO:0001552	feature_id[366].value <= threshold=2.1711617708206177
node_42: feature_name=GO:0046483	feature_id[391].value <= threshold=320.9667053222656
node_43: feature_name=GO:0002439	feature_id[431].value <= threshold=1.832722783088684
node_44: feature_name=GO:0006555	feature_id[514].value <= threshold=5.321640968322754
node_45: feature_name=GO:0070424	feature_id[254].value <= threshold=3.321003556251526
node_46: feature_name=hsa05144	feature_id[751].value <= threshold=2.635499954223633
node_47: feature_name=GO:0032633	feature_id[627].value > threshold=1.2592533230781555
node_125: feature_name=GO:0033158	feature_id[379].value > threshold=0.9298970401287079
node_131: feature_name=GO:0005634	feature_id[247].value > threshold=0.20456184579416004

Class: positive genes

Rules_443	passed counts:2
node_0: feature_name=GO:0042113	feature_id[0].value <= threshold=13.408552169799805
node_1: feature_name=GO:0007568	feature_id[534].value <= threshold=5.0313897132873535
node_2: feature_name=GO:0002705	feature_id[541].value <= threshold=3.200145721435547
node_3: feature_name=GO:1901525	feature_id[576].value <= threshold=0.4466460347175598
node_4: feature_name=GO:0048539	feature_id[319].value <= threshold=3.0399646759033203
node_5: feature_name=GO:0001910	feature_id[385].value <= threshold=3.7437864542007446
node_6: feature_name=GO:0043200	feature_id[706].value <= threshold=9.307284355163574
node_7: feature_name=GO:0001773	feature_id[308].value <= threshold=4.016931533813477
node_8: feature_name=GO:0090116	feature_id[97].value <= threshold=7.99645471572876
node_9: feature_name=GO:0019814	feature_id[189].value <= threshold=4.525782108306885
node_10: feature_name=GO:1902583	feature_id[215].value <= threshold=17.60310649871826
node_11: feature_name=GO:0045429	feature_id[737].value <= threshold=1.6133361458778381
node_12: feature_name=GO:0003720	feature_id[228].value <= threshold=5.519326210021973
node_13: feature_name=GO:0046006	feature_id[364].value <= threshold=4.9985432624816895
node_14: feature_name=GO:0070424	feature_id[254].value <= threshold=6.233297109603882
node_15: feature_name=GO:0009892	feature_id[320].value <= threshold=59.99180793762207
node_16: feature_name=GO:0007064	feature_id[527].value <= threshold=11.57377815246582
node_17: feature_name=GO:0005575	feature_id[17].value <= threshold=15.417460918426514
node_18: feature_name=GO:0043368	feature_id[31].value <= threshold=4.059496641159058
node_19: feature_name=GO:0005164	feature_id[163].value <= threshold=5.793607711791992
node_20: feature_name=GO:0042130	feature_id[16].value <= threshold=5.073179721832275
node_21: feature_name=GO:0010216	feature_id[282].value <= threshold=4.834584474563599
node_22: feature_name=GO:0009628	feature_id[553].value <= threshold=29.314892768859863
node_23: feature_name=GO:0045628	feature_id[749].value <= threshold=3.1001367568969727
node_24: feature_name=GO:0042288	feature_id[100].value <= threshold=5.174932956695557
node_25: feature_name=GO:0002329	feature_id[406].value <= threshold=2.348930835723877
node_26: feature_name=GO:0051246	feature_id[642].value <= threshold=0.6498909294605255
node_27: feature_name=GO:0042162	feature_id[355].value <= threshold=8.05103588104248
node_28: feature_name=hsa04668	feature_id[333].value <= threshold=5.614926338195801
node_29: feature_name=GO:0032200	feature_id[613].value <= threshold=28.735913276672363
node_30: feature_name=hsa04662	feature_id[334].value <= threshold=9.588344097137451
node_31: feature_name=GO:1901992	feature_id[105].value <= threshold=5.291427850723267

node_32: feature_name=GO:0072341 feature_id[144].value <= threshold=6.01465106010437
node_33: feature_name=GO:0047485 feature_id[121].value <= threshold=6.657836437225342
node_34: feature_name=GO:0090594 feature_id[738].value <= threshold=1.8673912286758423
node_35: feature_name=GO:0043525 feature_id[524].value <= threshold=2.881397008895874
node_36: feature_name=GO:0032504 feature_id[244].value <= threshold=17.22207546234131
node_37: feature_name=GO:0051348 feature_id[126].value <= threshold=5.933559417724609
node_38: feature_name=GO:0042522 feature_id[692].value <= threshold=1.78694087266922
node_39: feature_name=GO:0031100 feature_id[73].value <= threshold=3.2312101125717163
node_40: feature_name=GO:0042493 feature_id[149].value <= threshold=19.771170616149902
node_41: feature_name=GO:0001552 feature_id[366].value <= threshold=2.1711617708206177
node_42: feature_name=GO:0046483 feature_id[391].value <= threshold=320.9667053222656
node_43: feature_name=GO:0002439 feature_id[431].value <= threshold=1.832722783088684
node_44: feature_name=GO:0006555 feature_id[514].value <= threshold=5.321640968322754
node_45: feature_name=GO:0070424 feature_id[254].value <= threshold=3.321003556251526
node_46: feature_name=hsa05144 feature_id[751].value <= threshold=2.635499954223633
node_47: feature_name=GO:0032633 feature_id[627].value <= threshold=1.2592533230781555
node_48: feature_name=GO:0019814 feature_id[189].value <= threshold=2.1583125591278076
node_49: feature_name=GO:1901989 feature_id[30].value <= threshold=3.3150794506073
node_50: feature_name=GO:1903318 feature_id[196].value <= threshold=1.62252539396286
node_51: feature_name=GO:0060576 feature_id[204].value <= threshold=2.1097792387008667
node_52: feature_name=hsa05221 feature_id[349].value > threshold=0.8723124265670776
node_96: feature_name=hsa05221 feature_id[349].value > threshold=0.8756309151649475
node_98: feature_name=GO:0046794 feature_id[794].value <= threshold=6.585465431213379
node_99: feature_name=GO:0005035 feature_id[133].value > threshold=0.6986835598945618
node_109: feature_name=GO:0045637 feature_id[241].value > threshold=0.3161832243204117
Class: positive genes

Rules_444 passed counts:2
node_0: feature_name=GO:0042113 feature_id[0].value <= threshold=13.408552169799805
node_1: feature_name=GO:0007568 feature_id[534].value <= threshold=5.0313897132873535
node_2: feature_name=GO:0002705 feature_id[541].value <= threshold=3.200145721435547
node_3: feature_name=GO:1901525 feature_id[576].value <= threshold=0.4466460347175598
node_4: feature_name=GO:0048539 feature_id[319].value <= threshold=3.0399646759033203
node_5: feature_name=GO:0001910 feature_id[385].value <= threshold=3.7437864542007446
node_6: feature_name=GO:0043200 feature_id[706].value <= threshold=9.307284355163574

node_7: feature_name=GO:0001773 feature_id[308].value <= threshold=4.016931533813477
node_8: feature_name=GO:0090116 feature_id[97].value <= threshold=7.99645471572876
node_9: feature_name=GO:0019814 feature_id[189].value <= threshold=4.525782108306885
node_10: feature_name=GO:1902583 feature_id[215].value <= threshold=17.60310649871826
node_11: feature_name=GO:0045429 feature_id[737].value <= threshold=1.6133361458778381
node_12: feature_name=GO:0003720 feature_id[228].value <= threshold=5.519326210021973
node_13: feature_name=GO:0046006 feature_id[364].value <= threshold=4.9985432624816895
node_14: feature_name=GO:0070424 feature_id[254].value <= threshold=6.233297109603882
node_15: feature_name=GO:0009892 feature_id[320].value <= threshold=59.99180793762207
node_16: feature_name=GO:0007064 feature_id[527].value <= threshold=11.57377815246582
node_17: feature_name=GO:0005575 feature_id[17].value <= threshold=15.417460918426514
node_18: feature_name=GO:0043368 feature_id[31].value <= threshold=4.059496641159058
node_19: feature_name=GO:0005164 feature_id[163].value <= threshold=5.793607711791992
node_20: feature_name=GO:0042130 feature_id[16].value <= threshold=5.073179721832275
node_21: feature_name=GO:0010216 feature_id[282].value <= threshold=4.834584474563599
node_22: feature_name=GO:0009628 feature_id[553].value <= threshold=29.314892768859863
node_23: feature_name=GO:0045628 feature_id[749].value <= threshold=3.1001367568969727
node_24: feature_name=GO:0042288 feature_id[100].value <= threshold=5.174932956695557
node_25: feature_name=GO:0002329 feature_id[406].value <= threshold=2.348930835723877
node_26: feature_name=GO:0051246 feature_id[642].value <= threshold=0.6498909294605255
node_27: feature_name=GO:0042162 feature_id[355].value <= threshold=8.05103588104248
node_28: feature_name=hsa04668 feature_id[333].value <= threshold=5.614926338195801
node_29: feature_name=GO:0032200 feature_id[613].value <= threshold=28.735913276672363
node_30: feature_name=hsa04662 feature_id[334].value <= threshold=9.588344097137451
node_31: feature_name=GO:1901992 feature_id[105].value <= threshold=5.291427850723267
node_32: feature_name=GO:0072341 feature_id[144].value <= threshold=6.01465106010437
node_33: feature_name=GO:0047485 feature_id[121].value <= threshold=6.657836437225342
node_34: feature_name=GO:0090594 feature_id[738].value <= threshold=1.8673912286758423
node_35: feature_name=GO:0043525 feature_id[524].value <= threshold=2.881397008895874
node_36: feature_name=GO:0032504 feature_id[244].value <= threshold=17.22207546234131
node_37: feature_name=GO:0051348 feature_id[126].value <= threshold=5.933559417724609
node_38: feature_name=GO:0042522 feature_id[692].value <= threshold=1.78694087266922
node_39: feature_name=GO:0031100 feature_id[73].value <= threshold=3.2312101125717163
node_40: feature_name=GO:0042493 feature_id[149].value <= threshold=19.771170616149902
node_41: feature_name=GO:0001552 feature_id[366].value <= threshold=2.1711617708206177

node_42: feature_name=GO:0046483 feature_id[391].value <= threshold=320.9667053222656
node_43: feature_name=GO:0002439 feature_id[431].value <= threshold=1.832722783088684
node_44: feature_name=GO:0006555 feature_id[514].value <= threshold=5.321640968322754
node_45: feature_name=GO:0070424 feature_id[254].value <= threshold=3.321003556251526
node_46: feature_name=hsa05144 feature_id[751].value <= threshold=2.635499954223633
node_47: feature_name=GO:0032633 feature_id[627].value <= threshold=1.2592533230781555
node_48: feature_name=GO:0019814 feature_id[189].value <= threshold=2.1583125591278076
node_49: feature_name=GO:1901989 feature_id[30].value <= threshold=3.3150794506073
node_50: feature_name=GO:1903318 feature_id[196].value <= threshold=1.62252539396286
node_51: feature_name=GO:0060576 feature_id[204].value <= threshold=2.1097792387008667
node_52: feature_name=hsa05221 feature_id[349].value <= threshold=0.8723124265670776
node_53: feature_name=GO:1903896 feature_id[135].value > threshold=1.397229254245758
node_93: feature_name=GO:0033554 feature_id[418].value <= threshold=0.0016432370175607502
Class: positive genes

Rules_445 passed counts:1
node_0: feature_name=GO:0042113 feature_id[0].value > threshold=13.408552169799805
node_1454: feature_name=GO:0050851 feature_id[29].value > threshold=9.650307655334473
node_1534: feature_name=GO:1902166 feature_id[301].value > threshold=0.303210511803627
node_1548: feature_name=GO:0035872 feature_id[659].value <= threshold=35.09038162231445
node_1549: feature_name=GO:0032069 feature_id[614].value > threshold=3.441983938217163
node_1571: feature_name=GO:0050864 feature_id[288].value > threshold=7.516624450683594
node_1575: feature_name=GO:2000772 feature_id[95].value > threshold=0.9979664981365204
node_1579: feature_name=GO:0043525 feature_id[524].value > threshold=22.19614315032959
node_1591: feature_name=GO:0044238 feature_id[295].value > threshold=161.96461868286133
Class: positive genes

Rules_446 passed counts:1
node_0: feature_name=GO:0042113 feature_id[0].value > threshold=13.408552169799805
node_1454: feature_name=GO:0050851 feature_id[29].value > threshold=9.650307655334473
node_1534: feature_name=GO:1902166 feature_id[301].value > threshold=0.303210511803627
node_1548: feature_name=GO:0035872 feature_id[659].value <= threshold=35.09038162231445
node_1549: feature_name=GO:0032069 feature_id[614].value > threshold=3.441983938217163
node_1571: feature_name=GO:0050864 feature_id[288].value > threshold=7.516624450683594
node_1575: feature_name=GO:2000772 feature_id[95].value > threshold=0.9979664981365204

node_1579: feature_name=GO:0043525 feature_id[524].value <= threshold=22.19614315032959
node_1580: feature_name=GO:0032479 feature_id[441].value > threshold=4.490016937255859
node_1584: feature_name=GO:0042130 feature_id[16].value > threshold=14.421725749969482
Class: negative genes

Rules_447 passed counts:1
node_0: feature_name=GO:0042113 feature_id[0].value > threshold=13.408552169799805
node_1454: feature_name=GO:0050851 feature_id[29].value > threshold=9.650307655334473
node_1534: feature_name=GO:1902166 feature_id[301].value > threshold=0.303210511803627
node_1548: feature_name=GO:0035872 feature_id[659].value <= threshold=35.09038162231445
node_1549: feature_name=GO:0032069 feature_id[614].value > threshold=3.441983938217163
node_1571: feature_name=GO:0050864 feature_id[288].value > threshold=7.516624450683594
node_1575: feature_name=GO:2000772 feature_id[95].value > threshold=0.9979664981365204
node_1579: feature_name=GO:0043525 feature_id[524].value <= threshold=22.19614315032959
node_1580: feature_name=GO:0032479 feature_id[441].value > threshold=4.490016937255859
node_1584: feature_name=GO:0042130 feature_id[16].value <= threshold=14.421725749969482
node_1585: feature_name=GO:0001782 feature_id[240].value > threshold=14.421442985534668
Class: negative genes

Rules_448 passed counts:1
node_0: feature_name=GO:0042113 feature_id[0].value > threshold=13.408552169799805
node_1454: feature_name=GO:0050851 feature_id[29].value > threshold=9.650307655334473
node_1534: feature_name=GO:1902166 feature_id[301].value > threshold=0.303210511803627
node_1548: feature_name=GO:0035872 feature_id[659].value <= threshold=35.09038162231445
node_1549: feature_name=GO:0032069 feature_id[614].value > threshold=3.441983938217163
node_1571: feature_name=GO:0050864 feature_id[288].value > threshold=7.516624450683594
node_1575: feature_name=GO:2000772 feature_id[95].value > threshold=0.9979664981365204
node_1579: feature_name=GO:0043525 feature_id[524].value <= threshold=22.19614315032959
node_1580: feature_name=GO:0032479 feature_id[441].value > threshold=4.490016937255859
node_1584: feature_name=GO:0042130 feature_id[16].value <= threshold=14.421725749969482
node_1585: feature_name=GO:0001782 feature_id[240].value <= threshold=14.421442985534668
node_1586: feature_name=GO:0038061 feature_id[671].value > threshold=36.05139923095703
Class: negative genes

Rules_449 passed counts:1

node_0: feature_name=GO:0042113 feature_id[0].value > threshold=13.408552169799805
node_1454: feature_name=GO:0050851 feature_id[29].value > threshold=9.650307655334473
node_1534: feature_name=GO:1902166 feature_id[301].value > threshold=0.303210511803627
node_1548: feature_name=GO:0035872 feature_id[659].value <= threshold=35.09038162231445
node_1549: feature_name=GO:0032069 feature_id[614].value <= threshold=3.441983938217163
node_1550: feature_name=GO:0050897 feature_id[164].value > threshold=1.3939869403839111
node_1568: feature_name=GO:0007406 feature_id[537].value > threshold=4.206701993942261
Class: positive genes

Rules_450 passed counts:1
node_0: feature_name=GO:0042113 feature_id[0].value > threshold=13.408552169799805
node_1454: feature_name=GO:0050851 feature_id[29].value > threshold=9.650307655334473
node_1534: feature_name=GO:1902166 feature_id[301].value > threshold=0.303210511803627
node_1548: feature_name=GO:0035872 feature_id[659].value <= threshold=35.09038162231445
node_1549: feature_name=GO:0032069 feature_id[614].value <= threshold=3.441983938217163
node_1550: feature_name=GO:0050897 feature_id[164].value <= threshold=1.3939869403839111
node_1551: feature_name=GO:0051454 feature_id[307].value <= threshold=0.9144491851329803
node_1552: feature_name=GO:0035825 feature_id[662].value > threshold=0.08555268123745918
node_1556: feature_name=GO:0031265 feature_id[48].value > threshold=0.7173363864421844
node_1560: feature_name=GO:0002562 feature_id[25].value > threshold=5.642775535583496
node_1562: feature_name=hsa04660 feature_id[472].value <= threshold=17.786678314208984
Class: negative genes

Rules_451 passed counts:1
node_0: feature_name=GO:0042113 feature_id[0].value > threshold=13.408552169799805
node_1454: feature_name=GO:0050851 feature_id[29].value > threshold=9.650307655334473
node_1534: feature_name=GO:1902166 feature_id[301].value > threshold=0.303210511803627
node_1548: feature_name=GO:0035872 feature_id[659].value <= threshold=35.09038162231445
node_1549: feature_name=GO:0032069 feature_id[614].value <= threshold=3.441983938217163
node_1550: feature_name=GO:0050897 feature_id[164].value <= threshold=1.3939869403839111
node_1551: feature_name=GO:0051454 feature_id[307].value <= threshold=0.9144491851329803
node_1552: feature_name=GO:0035825 feature_id[662].value > threshold=0.08555268123745918
node_1556: feature_name=GO:0031265 feature_id[48].value > threshold=0.7173363864421844
node_1560: feature_name=GO:0002562 feature_id[25].value <= threshold=5.642775535583496
Class: negative genes

Rules_452 passed counts:1
node_0: feature_name=GO:0042113 feature_id[0].value > threshold=13.408552169799805
node_1454: feature_name=GO:0050851 feature_id[29].value > threshold=9.650307655334473
node_1534: feature_name=GO:1902166 feature_id[301].value <= threshold=0.303210511803627
node_1535: feature_name=GO:0042287 feature_id[20].value > threshold=1.4291933178901672
node_1545: feature_name=GO:0006968 feature_id[517].value <= threshold=0.4102974385023117
Class: negative genes

Rules_453 passed counts:1
node_0: feature_name=GO:0042113 feature_id[0].value > threshold=13.408552169799805
node_1454: feature_name=GO:0050851 feature_id[29].value > threshold=9.650307655334473
node_1534: feature_name=GO:1902166 feature_id[301].value <= threshold=0.303210511803627
node_1535: feature_name=GO:0042287 feature_id[20].value <= threshold=1.4291933178901672
node_1536: feature_name=GO:0023026 feature_id[69].value <= threshold=1.7696257829666138
node_1537: feature_name=GO:0048661 feature_id[733].value > threshold=3.2364230155944824
node_1539: feature_name=GO:0002263 feature_id[392].value <= threshold=5.861323833465576
Class: positive genes

Rules_454 passed counts:1
node_0: feature_name=GO:0042113 feature_id[0].value > threshold=13.408552169799805
node_1454: feature_name=GO:0050851 feature_id[29].value <= threshold=9.650307655334473
node_1455: feature_name=GO:0006304 feature_id[510].value <= threshold=5.960662126541138
node_1456: feature_name=GO:0032673 feature_id[623].value > threshold=4.092082738876343
node_1510: feature_name=GO:0046898 feature_id[161].value <= threshold=2.2367727756500244
node_1511: feature_name=GO:0030183 feature_id[590].value > threshold=12.193635940551758
node_1521: feature_name=GO:0032504 feature_id[244].value > threshold=0.8662047982215881
node_1523: feature_name=GO:0045589 feature_id[347].value > threshold=3.9446849822998047
node_1525: feature_name=GO:2001235 feature_id[495].value > threshold=15.975362300872803
Class: positive genes

Rules_455 passed counts:1
node_0: feature_name=GO:0042113 feature_id[0].value > threshold=13.408552169799805
node_1454: feature_name=GO:0050851 feature_id[29].value <= threshold=9.650307655334473
node_1455: feature_name=GO:0006304 feature_id[510].value <= threshold=5.960662126541138

node_1456: feature_name=GO:0032673 feature_id[623].value > threshold=4.092082738876343
node_1510: feature_name=GO:0046898 feature_id[161].value <= threshold=2.2367727756500244
node_1511: feature_name=GO:0030183 feature_id[590].value <= threshold=12.193635940551758
node_1512: feature_name=GO:0032480 feature_id[525].value > threshold=0.5560729652643204
node_1514: feature_name=GO:1902554 feature_id[660].value > threshold=1.3245088458061218
node_1518: feature_name=GO:0010952 feature_id[483].value > threshold=9.767204761505127
Class: positive genes

Rules_456 passed counts:1
node_0: feature_name=GO:0042113 feature_id[0].value > threshold=13.408552169799805
node_1454: feature_name=GO:0050851 feature_id[29].value <= threshold=9.650307655334473
node_1455: feature_name=GO:0006304 feature_id[510].value <= threshold=5.960662126541138
node_1456: feature_name=GO:0032673 feature_id[623].value > threshold=4.092082738876343
node_1510: feature_name=GO:0046898 feature_id[161].value <= threshold=2.2367727756500244
node_1511: feature_name=GO:0030183 feature_id[590].value <= threshold=12.193635940551758
node_1512: feature_name=GO:0032480 feature_id[525].value > threshold=0.5560729652643204
node_1514: feature_name=GO:1902554 feature_id[660].value <= threshold=1.3245088458061218
node_1515: feature_name=GO:0042130 feature_id[16].value <= threshold=2.3747211694717407
Class: negative genes

Rules_457 passed counts:1
node_0: feature_name=GO:0042113 feature_id[0].value > threshold=13.408552169799805
node_1454: feature_name=GO:0050851 feature_id[29].value <= threshold=9.650307655334473
node_1455: feature_name=GO:0006304 feature_id[510].value <= threshold=5.960662126541138
node_1456: feature_name=GO:0032673 feature_id[623].value <= threshold=4.092082738876343
node_1457: feature_name=GO:0002429 feature_id[748].value > threshold=4.626799821853638
node_1463: feature_name=GO:0015672 feature_id[573].value > threshold=0.08226438239216805
node_1475: feature_name=GO:0030217 feature_id[589].value > threshold=19.77315902709961
node_1497: feature_name=GO:0030291 feature_id[147].value <= threshold=4.140083312988281
node_1498: feature_name=GO:0050798 feature_id[170].value <= threshold=12.055219173431396
node_1499: feature_name=GO:2000773 feature_id[114].value <= threshold=7.385613679885864
node_1500: feature_name=GO:0007050 feature_id[398].value > threshold=23.01229476928711
Class: positive genes

Rules_458 passed counts:1

node_0: feature_name=GO:0042113 feature_id[0].value > threshold=13.408552169799805
node_1454: feature_name=GO:0050851 feature_id[29].value <= threshold=9.650307655334473
node_1455: feature_name=GO:0006304 feature_id[510].value <= threshold=5.960662126541138
node_1456: feature_name=GO:0032673 feature_id[623].value <= threshold=4.092082738876343
node_1457: feature_name=GO:0002429 feature_id[748].value > threshold=4.626799821853638
node_1463: feature_name=GO:0015672 feature_id[573].value > threshold=0.08226438239216805
node_1475: feature_name=GO:0030217 feature_id[589].value <= threshold=19.77315902709961
node_1476: feature_name=GO:0002381 feature_id[96].value <= threshold=4.645626783370972
node_1477: feature_name=GO:0045058 feature_id[727].value > threshold=5.092289447784424
node_1485: feature_name=GO:0016447 feature_id[74].value > threshold=4.237667560577393
node_1487: feature_name=GO:0045628 feature_id[749].value > threshold=3.333345651626587
Class: positive genes

Rules_459 passed counts:1
node_0: feature_name=GO:0042113 feature_id[0].value > threshold=13.408552169799805
node_1454: feature_name=GO:0050851 feature_id[29].value <= threshold=9.650307655334473
node_1455: feature_name=GO:0006304 feature_id[510].value <= threshold=5.960662126541138
node_1456: feature_name=GO:0032673 feature_id[623].value <= threshold=4.092082738876343
node_1457: feature_name=GO:0002429 feature_id[748].value > threshold=4.626799821853638
node_1463: feature_name=GO:0015672 feature_id[573].value > threshold=0.08226438239216805
node_1475: feature_name=GO:0030217 feature_id[589].value <= threshold=19.77315902709961
node_1476: feature_name=GO:0002381 feature_id[96].value <= threshold=4.645626783370972
node_1477: feature_name=GO:0045058 feature_id[727].value <= threshold=5.092289447784424
node_1478: feature_name=GO:0032703 feature_id[617].value > threshold=2.6942304372787476
node_1482: feature_name=GO:1904894 feature_id[726].value <= threshold=4.65221294760704
Class: negative genes

Rules_460 passed counts:1
node_0: feature_name=GO:0042113 feature_id[0].value > threshold=13.408552169799805
node_1454: feature_name=GO:0050851 feature_id[29].value <= threshold=9.650307655334473
node_1455: feature_name=GO:0006304 feature_id[510].value <= threshold=5.960662126541138
node_1456: feature_name=GO:0032673 feature_id[623].value <= threshold=4.092082738876343
node_1457: feature_name=GO:0002429 feature_id[748].value > threshold=4.626799821853638
node_1463: feature_name=GO:0015672 feature_id[573].value > threshold=0.08226438239216805
node_1475: feature_name=GO:0030217 feature_id[589].value <= threshold=19.77315902709961

node_1476: feature_name=GO:0002381 feature_id[96].value <= threshold=4.645626783370972
node_1477: feature_name=GO:0045058 feature_id[727].value <= threshold=5.092289447784424
node_1478: feature_name=GO:0032703 feature_id[617].value <= threshold=2.6942304372787476
node_1479: feature_name=GO:0001909 feature_id[386].value <= threshold=0.47530554234981537
Class: positive genes

Rules_461 passed counts:1
node_0: feature_name=GO:0042113 feature_id[0].value > threshold=13.408552169799805
node_1454: feature_name=GO:0050851 feature_id[29].value <= threshold=9.650307655334473
node_1455: feature_name=GO:0006304 feature_id[510].value <= threshold=5.960662126541138
node_1456: feature_name=GO:0032673 feature_id[623].value <= threshold=4.092082738876343
node_1457: feature_name=GO:0002429 feature_id[748].value > threshold=4.626799821853638
node_1463: feature_name=GO:0015672 feature_id[573].value <= threshold=0.08226438239216805
node_1464: feature_name=GO:0038065 feature_id[670].value > threshold=0.3765842020511627
node_1468: feature_name=GO:0046685 feature_id[782].value <= threshold=2.9275591373443604
node_1469: feature_name=GO:0050897 feature_id[164].value > threshold=2.1307075023651123
node_1471: feature_name=GO:0023056 feature_id[632].value > threshold=59.85031509399414
Class: positive genes

Rules_462 passed counts:1
node_0: feature_name=GO:0042113 feature_id[0].value > threshold=13.408552169799805
node_1454: feature_name=GO:0050851 feature_id[29].value <= threshold=9.650307655334473
node_1455: feature_name=GO:0006304 feature_id[510].value <= threshold=5.960662126541138
node_1456: feature_name=GO:0032673 feature_id[623].value <= threshold=4.092082738876343
node_1457: feature_name=GO:0002429 feature_id[748].value <= threshold=4.626799821853638
node_1458: feature_name=GO:0005575 feature_id[17].value > threshold=0.7239105105400085
node_1460: feature_name=GO:0090116 feature_id[97].value > threshold=2.8600313663482666
Class: positive genes

Rules_463 passed counts:1
node_0: feature_name=GO:0042113 feature_id[0].value <= threshold=13.408552169799805
node_1: feature_name=GO:0007568 feature_id[534].value > threshold=5.0313897132873535
node_913: feature_name=GO:0032763 feature_id[629].value > threshold=0.31753237545490265
node_1195: feature_name=GO:0071301 feature_id[442].value > threshold=0.11534593254327774
node_1375: feature_name=GO:0046500 feature_id[171].value > threshold=1.130434513092041

node_1437: feature_name=GO:0033343 feature_id[639].value > threshold=0.98012974858284
node_1443: feature_name=GO:0007126 feature_id[520].value > threshold=0.40390433371067047
node_1447: feature_name=GO:0019058 feature_id[245].value > threshold=9.540197849273682
node_1451: feature_name=hsa04650 feature_id[13].value > threshold=1.7103192806243896
Class: positive genes

Rules_464 passed counts:1
node_0: feature_name=GO:0042113 feature_id[0].value <= threshold=13.408552169799805
node_1: feature_name=GO:0007568 feature_id[534].value > threshold=5.0313897132873535
node_913: feature_name=GO:0032763 feature_id[629].value > threshold=0.31753237545490265
node_1195: feature_name=GO:0071301 feature_id[442].value > threshold=0.11534593254327774
node_1375: feature_name=GO:0046500 feature_id[171].value > threshold=1.130434513092041
node_1437: feature_name=GO:0033343 feature_id[639].value > threshold=0.98012974858284
node_1443: feature_name=GO:0007126 feature_id[520].value > threshold=0.40390433371067047
node_1447: feature_name=GO:0019058 feature_id[245].value <= threshold=9.540197849273682
node_1448: feature_name=GO:0002718 feature_id[481].value <= threshold=0.052317868918180466
Class: negative genes

Rules_465 passed counts:1
node_0: feature_name=GO:0042113 feature_id[0].value <= threshold=13.408552169799805
node_1: feature_name=GO:0007568 feature_id[534].value > threshold=5.0313897132873535
node_913: feature_name=GO:0032763 feature_id[629].value > threshold=0.31753237545490265
node_1195: feature_name=GO:0071301 feature_id[442].value > threshold=0.11534593254327774
node_1375: feature_name=GO:0046500 feature_id[171].value > threshold=1.130434513092041
node_1437: feature_name=GO:0033343 feature_id[639].value > threshold=0.98012974858284
node_1443: feature_name=GO:0007126 feature_id[520].value <= threshold=0.40390433371067047
node_1444: feature_name=GO:0042267 feature_id[148].value <= threshold=0.3329026997089386
Class: positive genes

Rules_466 passed counts:1
node_0: feature_name=GO:0042113 feature_id[0].value <= threshold=13.408552169799805
node_1: feature_name=GO:0007568 feature_id[534].value > threshold=5.0313897132873535
node_913: feature_name=GO:0032763 feature_id[629].value > threshold=0.31753237545490265
node_1195: feature_name=GO:0071301 feature_id[442].value > threshold=0.11534593254327774
node_1375: feature_name=GO:0046500 feature_id[171].value > threshold=1.130434513092041

node_1437: feature_name=GO:0033343 feature_id[639].value <= threshold=0.98012974858284
node_1438: feature_name=GO:0019058 feature_id[245].value > threshold=11.779548168182373
node_1440: feature_name=GO:0032480 feature_id[525].value > threshold=6.815735578536987
Class: negative genes

Rules_467 passed counts:1
node_0: feature_name=GO:0042113 feature_id[0].value <= threshold=13.408552169799805
node_1: feature_name=GO:0007568 feature_id[534].value > threshold=5.0313897132873535
node_913: feature_name=GO:0032763 feature_id[629].value > threshold=0.31753237545490265
node_1195: feature_name=GO:0071301 feature_id[442].value > threshold=0.11534593254327774
node_1375: feature_name=GO:0046500 feature_id[171].value <= threshold=1.130434513092041
node_1376: feature_name=GO:0001782 feature_id[240].value <= threshold=6.639636993408203
node_1377: feature_name=GO:0043371 feature_id[707].value > threshold=2.562113642692566
node_1433: feature_name=GO:0044446 feature_id[184].value <= threshold=5.497895836830139
Class: negative genes

Rules_468 passed counts:1
node_0: feature_name=GO:0042113 feature_id[0].value <= threshold=13.408552169799805
node_1: feature_name=GO:0007568 feature_id[534].value > threshold=5.0313897132873535
node_913: feature_name=GO:0032763 feature_id[629].value > threshold=0.31753237545490265
node_1195: feature_name=GO:0071301 feature_id[442].value > threshold=0.11534593254327774
node_1375: feature_name=GO:0046500 feature_id[171].value <= threshold=1.130434513092041
node_1376: feature_name=GO:0001782 feature_id[240].value <= threshold=6.639636993408203
node_1377: feature_name=GO:0043371 feature_id[707].value <= threshold=2.562113642692566
node_1378: feature_name=GO:0030291 feature_id[147].value > threshold=6.834916830062866
node_1424: feature_name=GO:0002439 feature_id[431].value > threshold=0.6129993498325348
node_1428: feature_name=GO:0009314 feature_id[296].value <= threshold=13.65259075164795
node_1429: feature_name=GO:0043011 feature_id[150].value <= threshold=0.23608403280377388
Class: negative genes

Rules_469 passed counts:1
node_0: feature_name=GO:0042113 feature_id[0].value <= threshold=13.408552169799805
node_1: feature_name=GO:0007568 feature_id[534].value > threshold=5.0313897132873535
node_913: feature_name=GO:0032763 feature_id[629].value > threshold=0.31753237545490265
node_1195: feature_name=GO:0071301 feature_id[442].value > threshold=0.11534593254327774

node_1375: feature_name=GO:0046500 feature_id[171].value <= threshold=1.130434513092041
node_1376: feature_name=GO:0001782 feature_id[240].value <= threshold=6.639636993408203
node_1377: feature_name=GO:0043371 feature_id[707].value <= threshold=2.562113642692566
node_1378: feature_name=GO:0030291 feature_id[147].value <= threshold=6.834916830062866
node_1379: feature_name=GO:0045656 feature_id[756].value > threshold=0.6281269192695618
node_1393: feature_name=GO:0046007 feature_id[152].value <= threshold=1.020785927772522
node_1394: feature_name=GO:0000302 feature_id[375].value > threshold=20.847331047058105
node_1416: feature_name=GO:0030225 feature_id[587].value > threshold=6.448119878768921
Class: negative genes

Rules_470 passed counts:1
node_0: feature_name=GO:0042113 feature_id[0].value <= threshold=13.408552169799805
node_1: feature_name=GO:0007568 feature_id[534].value > threshold=5.0313897132873535
node_913: feature_name=GO:0032763 feature_id[629].value > threshold=0.31753237545490265
node_1195: feature_name=GO:0071301 feature_id[442].value > threshold=0.11534593254327774
node_1375: feature_name=GO:0046500 feature_id[171].value <= threshold=1.130434513092041
node_1376: feature_name=GO:0001782 feature_id[240].value <= threshold=6.639636993408203
node_1377: feature_name=GO:0043371 feature_id[707].value <= threshold=2.562113642692566
node_1378: feature_name=GO:0030291 feature_id[147].value <= threshold=6.834916830062866
node_1379: feature_name=GO:0045656 feature_id[756].value > threshold=0.6281269192695618
node_1393: feature_name=GO:0046007 feature_id[152].value <= threshold=1.020785927772522
node_1394: feature_name=GO:0000302 feature_id[375].value <= threshold=20.847331047058105
node_1395: feature_name=hsa04110 feature_id[328].value <= threshold=43.297359466552734
node_1396: feature_name=GO:0050731 feature_id[804].value <= threshold=15.756641864776611
node_1397: feature_name=GO:0002440 feature_id[429].value > threshold=0.4019751101732254
node_1403: feature_name=GO:0051348 feature_id[126].value > threshold=0.8975991010665894
node_1405: feature_name=GO:0009636 feature_id[551].value > threshold=1.019210398197174
node_1407: feature_name=GO:0045636 feature_id[743].value > threshold=4.099599123001099
Class: positive genes

Rules_471 passed counts:1
node_0: feature_name=GO:0042113 feature_id[0].value <= threshold=13.408552169799805
node_1: feature_name=GO:0007568 feature_id[534].value > threshold=5.0313897132873535
node_913: feature_name=GO:0032763 feature_id[629].value > threshold=0.31753237545490265
node_1195: feature_name=GO:0071301 feature_id[442].value > threshold=0.11534593254327774

node_1375: feature_name=GO:0046500 feature_id[171].value <= threshold=1.130434513092041
node_1376: feature_name=GO:0001782 feature_id[240].value <= threshold=6.639636993408203
node_1377: feature_name=GO:0043371 feature_id[707].value <= threshold=2.562113642692566
node_1378: feature_name=GO:0030291 feature_id[147].value <= threshold=6.834916830062866
node_1379: feature_name=GO:0045656 feature_id[756].value > threshold=0.6281269192695618
node_1393: feature_name=GO:0046007 feature_id[152].value <= threshold=1.020785927772522
node_1394: feature_name=GO:0000302 feature_id[375].value <= threshold=20.847331047058105
node_1395: feature_name=hsa04110 feature_id[328].value <= threshold=43.297359466552734
node_1396: feature_name=GO:0050731 feature_id[804].value <= threshold=15.756641864776611
node_1397: feature_name=GO:0002440 feature_id[429].value > threshold=0.4019751101732254
node_1403: feature_name=GO:0051348 feature_id[126].value > threshold=0.8975991010665894
node_1405: feature_name=GO:0009636 feature_id[551].value <= threshold=1.019210398197174
Class: positive genes

Rules_472 passed counts:1
node_0: feature_name=GO:0042113 feature_id[0].value <= threshold=13.408552169799805
node_1: feature_name=GO:0007568 feature_id[534].value > threshold=5.0313897132873535
node_913: feature_name=GO:0032763 feature_id[629].value > threshold=0.31753237545490265
node_1195: feature_name=GO:0071301 feature_id[442].value > threshold=0.11534593254327774
node_1375: feature_name=GO:0046500 feature_id[171].value <= threshold=1.130434513092041
node_1376: feature_name=GO:0001782 feature_id[240].value <= threshold=6.639636993408203
node_1377: feature_name=GO:0043371 feature_id[707].value <= threshold=2.562113642692566
node_1378: feature_name=GO:0030291 feature_id[147].value <= threshold=6.834916830062866
node_1379: feature_name=GO:0045656 feature_id[756].value > threshold=0.6281269192695618
node_1393: feature_name=GO:0046007 feature_id[152].value <= threshold=1.020785927772522
node_1394: feature_name=GO:0000302 feature_id[375].value <= threshold=20.847331047058105
node_1395: feature_name=hsa04110 feature_id[328].value <= threshold=43.297359466552734
node_1396: feature_name=GO:0050731 feature_id[804].value <= threshold=15.756641864776611
node_1397: feature_name=GO:0002440 feature_id[429].value > threshold=0.4019751101732254
node_1403: feature_name=GO:0051348 feature_id[126].value <= threshold=0.8975991010665894
Class: positive genes

Rules_473 passed counts:1
node_0: feature_name=GO:0042113 feature_id[0].value <= threshold=13.408552169799805
node_1: feature_name=GO:0007568 feature_id[534].value > threshold=5.0313897132873535

node_913: feature_name=GO:0032763 feature_id[629].value > threshold=0.31753237545490265
node_1195: feature_name=GO:0071301 feature_id[442].value > threshold=0.11534593254327774
node_1375: feature_name=GO:0046500 feature_id[171].value <= threshold=1.130434513092041
node_1376: feature_name=GO:0001782 feature_id[240].value <= threshold=6.639636993408203
node_1377: feature_name=GO:0043371 feature_id[707].value <= threshold=2.562113642692566
node_1378: feature_name=GO:0030291 feature_id[147].value <= threshold=6.834916830062866
node_1379: feature_name=GO:0045656 feature_id[756].value > threshold=0.6281269192695618
node_1393: feature_name=GO:0046007 feature_id[152].value <= threshold=1.020785927772522
node_1394: feature_name=GO:0000302 feature_id[375].value <= threshold=20.847331047058105
node_1395: feature_name=hsa04110 feature_id[328].value <= threshold=43.297359466552734
node_1396: feature_name=GO:0050731 feature_id[804].value <= threshold=15.756641864776611
node_1397: feature_name=GO:0002440 feature_id[429].value <= threshold=0.4019751101732254
node_1398: feature_name=GO:0072610 feature_id[262].value > threshold=0.5024027079343796
node_1400: feature_name=GO:0048523 feature_id[293].value <= threshold=20.737138748168945
Class: negative genes

Rules_474 passed counts:1
node_0: feature_name=GO:0042113 feature_id[0].value <= threshold=13.408552169799805
node_1: feature_name=GO:0007568 feature_id[534].value > threshold=5.0313897132873535
node_913: feature_name=GO:0032763 feature_id[629].value > threshold=0.31753237545490265
node_1195: feature_name=GO:0071301 feature_id[442].value > threshold=0.11534593254327774
node_1375: feature_name=GO:0046500 feature_id[171].value <= threshold=1.130434513092041
node_1376: feature_name=GO:0001782 feature_id[240].value <= threshold=6.639636993408203
node_1377: feature_name=GO:0043371 feature_id[707].value <= threshold=2.562113642692566
node_1378: feature_name=GO:0030291 feature_id[147].value <= threshold=6.834916830062866
node_1379: feature_name=GO:0045656 feature_id[756].value <= threshold=0.6281269192695618
node_1380: feature_name=GO:0034103 feature_id[318].value > threshold=0.0158024481497705
node_1382: feature_name=GO:0048872 feature_id[498].value > threshold=19.454792976379395
node_1390: feature_name=GO:0043011 feature_id[150].value > threshold=3.861676812171936
Class: negative genes

Rules_475 passed counts:1
node_0: feature_name=GO:0042113 feature_id[0].value <= threshold=13.408552169799805
node_1: feature_name=GO:0007568 feature_id[534].value > threshold=5.0313897132873535
node_913: feature_name=GO:0032763 feature_id[629].value > threshold=0.31753237545490265

node_1195: feature_name=GO:0071301 feature_id[442].value > threshold=0.11534593254327774
node_1375: feature_name=GO:0046500 feature_id[171].value <= threshold=1.130434513092041
node_1376: feature_name=GO:0001782 feature_id[240].value <= threshold=6.639636993408203
node_1377: feature_name=GO:0043371 feature_id[707].value <= threshold=2.562113642692566
node_1378: feature_name=GO:0030291 feature_id[147].value <= threshold=6.834916830062866
node_1379: feature_name=GO:0045656 feature_id[756].value <= threshold=0.6281269192695618
node_1380: feature_name=GO:0034103 feature_id[318].value > threshold=0.0158024481497705
node_1382: feature_name=GO:0048872 feature_id[498].value <= threshold=19.454792976379395
node_1383: feature_name=GO:0002665 feature_id[453].value > threshold=1.584219217300415
Class: positive genes

Rules_476 passed counts:1
node_0: feature_name=GO:0042113 feature_id[0].value <= threshold=13.408552169799805
node_1: feature_name=GO:0007568 feature_id[534].value > threshold=5.0313897132873535
node_913: feature_name=GO:0032763 feature_id[629].value > threshold=0.31753237545490265
node_1195: feature_name=GO:0071301 feature_id[442].value > threshold=0.11534593254327774
node_1375: feature_name=GO:0046500 feature_id[171].value <= threshold=1.130434513092041
node_1376: feature_name=GO:0001782 feature_id[240].value <= threshold=6.639636993408203
node_1377: feature_name=GO:0043371 feature_id[707].value <= threshold=2.562113642692566
node_1378: feature_name=GO:0030291 feature_id[147].value <= threshold=6.834916830062866
node_1379: feature_name=GO:0045656 feature_id[756].value <= threshold=0.6281269192695618
node_1380: feature_name=GO:0034103 feature_id[318].value > threshold=0.0158024481497705
node_1382: feature_name=GO:0048872 feature_id[498].value <= threshold=19.454792976379395
node_1383: feature_name=GO:0002665 feature_id[453].value <= threshold=1.584219217300415
node_1384: feature_name=GO:0045628 feature_id[749].value > threshold=3.1363080739974976
Class: positive genes

Rules_477 passed counts:1
node_0: feature_name=GO:0042113 feature_id[0].value <= threshold=13.408552169799805
node_1: feature_name=GO:0007568 feature_id[534].value > threshold=5.0313897132873535
node_913: feature_name=GO:0032763 feature_id[629].value > threshold=0.31753237545490265
node_1195: feature_name=GO:0071301 feature_id[442].value > threshold=0.11534593254327774
node_1375: feature_name=GO:0046500 feature_id[171].value <= threshold=1.130434513092041
node_1376: feature_name=GO:0001782 feature_id[240].value <= threshold=6.639636993408203
node_1377: feature_name=GO:0043371 feature_id[707].value <= threshold=2.562113642692566

node_1378: feature_name=GO:0030291 feature_id[147].value <= threshold=6.834916830062866
node_1379: feature_name=GO:0045656 feature_id[756].value <= threshold=0.6281269192695618
node_1380: feature_name=GO:0034103 feature_id[318].value > threshold=0.0158024481497705
node_1382: feature_name=GO:0048872 feature_id[498].value <= threshold=19.454792976379395
node_1383: feature_name=GO:0002665 feature_id[453].value <= threshold=1.584219217300415
node_1384: feature_name=GO:0045628 feature_id[749].value <= threshold=3.1363080739974976
node_1385: feature_name=GO:0010639 feature_id[564].value > threshold=26.597777366638184
Class: positive genes

Rules_478 passed counts:1
node_0: feature_name=GO:0042113 feature_id[0].value <= threshold=13.408552169799805
node_1: feature_name=GO:0007568 feature_id[534].value > threshold=5.0313897132873535
node_913: feature_name=GO:0032763 feature_id[629].value > threshold=0.31753237545490265
node_1195: feature_name=GO:0071301 feature_id[442].value <= threshold=0.11534593254327774
node_1196: feature_name=GO:0046500 feature_id[171].value > threshold=0.6314916908740997
node_1340: feature_name=GO:0043627 feature_id[562].value > threshold=25.17277240753174
node_1368: feature_name=GO:0071310 feature_id[760].value <= threshold=80.00252914428711
node_1369: feature_name=GO:1901989 feature_id[30].value <= threshold=1.159576177597046
Class: negative genes

Rules_479 passed counts:1
node_0: feature_name=GO:0042113 feature_id[0].value <= threshold=13.408552169799805
node_1: feature_name=GO:0007568 feature_id[534].value > threshold=5.0313897132873535
node_913: feature_name=GO:0032763 feature_id[629].value > threshold=0.31753237545490265
node_1195: feature_name=GO:0071301 feature_id[442].value <= threshold=0.11534593254327774
node_1196: feature_name=GO:0046500 feature_id[171].value > threshold=0.6314916908740997
node_1340: feature_name=GO:0043627 feature_id[562].value <= threshold=25.17277240753174
node_1341: feature_name=GO:0010835 feature_id[560].value <= threshold=1.0601619482040405
node_1342: feature_name=GO:0016363 feature_id[90].value <= threshold=6.534365892410278
node_1343: feature_name=GO:0030983 feature_id[291].value <= threshold=3.5806901454925537
node_1344: feature_name=GO:0023030 feature_id[45].value <= threshold=0.6541633009910583
node_1345: feature_name=GO:0070245 feature_id[326].value > threshold=1.9657342433929443
node_1355: feature_name=GO:0010952 feature_id[483].value > threshold=2.8519983291625977
Class: negative genes

Rules_480	passed counts:1
node_0: feature_name=GO:0042113	feature_id[0].value <= threshold=13.408552169799805
node_1: feature_name=GO:0007568	feature_id[534].value > threshold=5.0313897132873535
node_913: feature_name=GO:0032763	feature_id[629].value > threshold=0.31753237545490265
node_1195: feature_name=GO:0071301	feature_id[442].value <= threshold=0.11534593254327774
node_1196: feature_name=GO:0046500	feature_id[171].value > threshold=0.6314916908740997
node_1340: feature_name=GO:0043627	feature_id[562].value <= threshold=25.17277240753174
node_1341: feature_name=GO:0010835	feature_id[560].value <= threshold=1.0601619482040405
node_1342: feature_name=GO:0016363	feature_id[90].value <= threshold=6.534365892410278
node_1343: feature_name=GO:0030983	feature_id[291].value <= threshold=3.5806901454925537
node_1344: feature_name=GO:0023030	feature_id[45].value <= threshold=0.6541633009910583
node_1345: feature_name=GO:0070245	feature_id[326].value <= threshold=1.9657342433929443
node_1346: feature_name=GO:0050897	feature_id[164].value <= threshold=1.6622408628463745
node_1347: feature_name=GO:0052548	feature_id[205].value > threshold=0.1624109297990799
node_1349: feature_name=GO:0000082	feature_id[478].value > threshold=0.03540035802870989
node_1351: feature_name=GO:0045556	feature_id[742].value > threshold=1.6088207960128784
Class: positive genes	

Rules_481	passed counts:1
node_0: feature_name=GO:0042113	feature_id[0].value <= threshold=13.408552169799805
node_1: feature_name=GO:0007568	feature_id[534].value > threshold=5.0313897132873535
node_913: feature_name=GO:0032763	feature_id[629].value > threshold=0.31753237545490265
node_1195: feature_name=GO:0071301	feature_id[442].value <= threshold=0.11534593254327774
node_1196: feature_name=GO:0046500	feature_id[171].value > threshold=0.6314916908740997
node_1340: feature_name=GO:0043627	feature_id[562].value <= threshold=25.17277240753174
node_1341: feature_name=GO:0010835	feature_id[560].value <= threshold=1.0601619482040405
node_1342: feature_name=GO:0016363	feature_id[90].value <= threshold=6.534365892410278
node_1343: feature_name=GO:0030983	feature_id[291].value <= threshold=3.5806901454925537
node_1344: feature_name=GO:0023030	feature_id[45].value <= threshold=0.6541633009910583
node_1345: feature_name=GO:0070245	feature_id[326].value <= threshold=1.9657342433929443
node_1346: feature_name=GO:0050897	feature_id[164].value <= threshold=1.6622408628463745
node_1347: feature_name=GO:0052548	feature_id[205].value > threshold=0.1624109297990799
node_1349: feature_name=GO:0000082	feature_id[478].value <= threshold=0.03540035802870989
Class: positive genes	

Rules_482	passed counts:1
node_0: feature_name=GO:0042113	feature_id[0].value <= threshold=13.408552169799805
node_1: feature_name=GO:0007568	feature_id[534].value > threshold=5.0313897132873535
node_913: feature_name=GO:0032763	feature_id[629].value > threshold=0.31753237545490265
node_1195: feature_name=GO:0071301	feature_id[442].value <= threshold=0.11534593254327774
node_1196: feature_name=GO:0046500	feature_id[171].value <= threshold=0.6314916908740997
node_1197: feature_name=GO:0030852	feature_id[22].value > threshold=2.9768868684768677
node_1329: feature_name=GO:0046668	feature_id[125].value > threshold=0.9442969560623169
node_1333: feature_name=GO:0006298	feature_id[621].value > threshold=0.45032089948654175
node_1337: feature_name=GO:0050868	feature_id[377].value <= threshold=0.2427361086010933
Class: negative genes	

Rules_483	passed counts:1
node_0: feature_name=GO:0042113	feature_id[0].value <= threshold=13.408552169799805
node_1: feature_name=GO:0007568	feature_id[534].value > threshold=5.0313897132873535
node_913: feature_name=GO:0032763	feature_id[629].value > threshold=0.31753237545490265
node_1195: feature_name=GO:0071301	feature_id[442].value <= threshold=0.11534593254327774
node_1196: feature_name=GO:0046500	feature_id[171].value <= threshold=0.6314916908740997
node_1197: feature_name=GO:0030852	feature_id[22].value <= threshold=2.9768868684768677
node_1198: feature_name=hsa04672	feature_id[276].value <= threshold=8.06681227684021
node_1199: feature_name=GO:0001836	feature_id[450].value > threshold=0.09961023926734924
node_1201: feature_name=GO:0070230	feature_id[64].value <= threshold=3.3956379890441895
node_1202: feature_name=GO:0070102	feature_id[49].value <= threshold=4.805386543273926
node_1203: feature_name=GO:0045553	feature_id[736].value > threshold=1.4970332980155945
node_1319: feature_name=GO:0031667	feature_id[648].value > threshold=25.942169189453125
Class: negative genes	

Rules_484	passed counts:1
node_0: feature_name=GO:0042113	feature_id[0].value <= threshold=13.408552169799805
node_1: feature_name=GO:0007568	feature_id[534].value > threshold=5.0313897132873535
node_913: feature_name=GO:0032763	feature_id[629].value > threshold=0.31753237545490265
node_1195: feature_name=GO:0071301	feature_id[442].value <= threshold=0.11534593254327774
node_1196: feature_name=GO:0046500	feature_id[171].value <= threshold=0.6314916908740997
node_1197: feature_name=GO:0030852	feature_id[22].value <= threshold=2.9768868684768677
node_1198: feature_name=hsa04672	feature_id[276].value <= threshold=8.06681227684021

node_1199: feature_name=GO:0001836 feature_id[450].value > threshold=0.09961023926734924
node_1201: feature_name=GO:0070230 feature_id[64].value <= threshold=3.3956379890441895
node_1202: feature_name=GO:0070102 feature_id[49].value <= threshold=4.805386543273926
node_1203: feature_name=GO:0045553 feature_id[736].value <= threshold=1.4970332980155945
node_1204: feature_name=GO:0051311 feature_id[58].value <= threshold=3.301889181137085
node_1205: feature_name=GO:0030291 feature_id[147].value <= threshold=8.156315326690674
node_1206: feature_name=GO:0045637 feature_id[241].value > threshold=0.01834342861548066
node_1208: feature_name=GO:0002890 feature_id[98].value <= threshold=2.767483353614807
node_1209: feature_name=GO:0046632 feature_id[248].value <= threshold=11.663254261016846
node_1210: feature_name=GO:0008588 feature_id[26].value > threshold=1.391375720500946
node_1308: feature_name=GO:0002200 feature_id[82].value > threshold=1.0774684846401215
node_1310: feature_name=GO:0019814 feature_id[189].value <= threshold=0.7463377118110657
node_1311: feature_name=GO:0002517 feature_id[88].value > threshold=1.2976589798927307
Class: negative genes

Rules_485 passed counts:1
node_0: feature_name=GO:0042113 feature_id[0].value <= threshold=13.408552169799805
node_1: feature_name=GO:0007568 feature_id[534].value > threshold=5.0313897132873535
node_913: feature_name=GO:0032763 feature_id[629].value > threshold=0.31753237545490265
node_1195: feature_name=GO:0071301 feature_id[442].value <= threshold=0.11534593254327774
node_1196: feature_name=GO:0046500 feature_id[171].value <= threshold=0.6314916908740997
node_1197: feature_name=GO:0030852 feature_id[22].value <= threshold=2.9768868684768677
node_1198: feature_name=hsa04672 feature_id[276].value <= threshold=8.06681227684021
node_1199: feature_name=GO:0001836 feature_id[450].value > threshold=0.09961023926734924
node_1201: feature_name=GO:0070230 feature_id[64].value <= threshold=3.3956379890441895
node_1202: feature_name=GO:0070102 feature_id[49].value <= threshold=4.805386543273926
node_1203: feature_name=GO:0045553 feature_id[736].value <= threshold=1.4970332980155945
node_1204: feature_name=GO:0051311 feature_id[58].value <= threshold=3.301889181137085
node_1205: feature_name=GO:0030291 feature_id[147].value <= threshold=8.156315326690674
node_1206: feature_name=GO:0045637 feature_id[241].value > threshold=0.01834342861548066
node_1208: feature_name=GO:0002890 feature_id[98].value <= threshold=2.767483353614807
node_1209: feature_name=GO:0046632 feature_id[248].value <= threshold=11.663254261016846
node_1210: feature_name=GO:0008588 feature_id[26].value <= threshold=1.391375720500946
node_1211: feature_name=GO:0010639 feature_id[564].value > threshold=18.975126266479492
node_1305: feature_name=GO:0045840 feature_id[697].value > threshold=8.670292615890503

Class: negative genes

Rules_486 passed counts:1
node_0: feature_name=GO:0042113 feature_id[0].value <= threshold=13.408552169799805
node_1: feature_name=GO:0007568 feature_id[534].value > threshold=5.0313897132873535
node_913: feature_name=GO:0032763 feature_id[629].value > threshold=0.31753237545490265
node_1195: feature_name=GO:0071301 feature_id[442].value <= threshold=0.11534593254327774
node_1196: feature_name=GO:0046500 feature_id[171].value <= threshold=0.6314916908740997
node_1197: feature_name=GO:0030852 feature_id[22].value <= threshold=2.9768868684768677
node_1198: feature_name=hsa04672 feature_id[276].value <= threshold=8.06681227684021
node_1199: feature_name=GO:0001836 feature_id[450].value > threshold=0.09961023926734924
node_1201: feature_name=GO:0070230 feature_id[64].value <= threshold=3.3956379890441895
node_1202: feature_name=GO:0070102 feature_id[49].value <= threshold=4.805386543273926
node_1203: feature_name=GO:0045553 feature_id[736].value <= threshold=1.4970332980155945
node_1204: feature_name=GO:0051311 feature_id[58].value <= threshold=3.301889181137085
node_1205: feature_name=GO:0030291 feature_id[147].value <= threshold=8.156315326690674
node_1206: feature_name=GO:0045637 feature_id[241].value > threshold=0.01834342861548066
node_1208: feature_name=GO:0002890 feature_id[98].value <= threshold=2.767483353614807
node_1209: feature_name=GO:0046632 feature_id[248].value <= threshold=11.663254261016846
node_1210: feature_name=GO:0008588 feature_id[26].value <= threshold=1.391375720500946
node_1211: feature_name=GO:0010639 feature_id[564].value <= threshold=18.975126266479492
node_1212: feature_name=GO:0046685 feature_id[782].value > threshold=5.153738260269165
node_1296: feature_name=GO:0019692 feature_id[584].value > threshold=0.0685717724263668
node_1300: feature_name=GO:0032649 feature_id[626].value <= threshold=0.2627730891108513
node_1301: feature_name=hsa05144 feature_id[751].value > threshold=2.427334427833557
Class: negative genes

Rules_487 passed counts:1
node_0: feature_name=GO:0042113 feature_id[0].value <= threshold=13.408552169799805
node_1: feature_name=GO:0007568 feature_id[534].value > threshold=5.0313897132873535
node_913: feature_name=GO:0032763 feature_id[629].value > threshold=0.31753237545490265
node_1195: feature_name=GO:0071301 feature_id[442].value <= threshold=0.11534593254327774
node_1196: feature_name=GO:0046500 feature_id[171].value <= threshold=0.6314916908740997
node_1197: feature_name=GO:0030852 feature_id[22].value <= threshold=2.9768868684768677
node_1198: feature_name=hsa04672 feature_id[276].value <= threshold=8.06681227684021

node_1199: feature_name=GO:0001836 feature_id[450].value > threshold=0.09961023926734924
node_1201: feature_name=GO:0070230 feature_id[64].value <= threshold=3.3956379890441895
node_1202: feature_name=GO:0070102 feature_id[49].value <= threshold=4.805386543273926
node_1203: feature_name=GO:0045553 feature_id[736].value <= threshold=1.4970332980155945
node_1204: feature_name=GO:0051311 feature_id[58].value <= threshold=3.301889181137085
node_1205: feature_name=GO:0030291 feature_id[147].value <= threshold=8.156315326690674
node_1206: feature_name=GO:0045637 feature_id[241].value > threshold=0.01834342861548066
node_1208: feature_name=GO:0002890 feature_id[98].value <= threshold=2.767483353614807
node_1209: feature_name=GO:0046632 feature_id[248].value <= threshold=11.663254261016846
node_1210: feature_name=GO:0008588 feature_id[26].value <= threshold=1.391375720500946
node_1211: feature_name=GO:0010639 feature_id[564].value <= threshold=18.975126266479492
node_1212: feature_name=GO:0046685 feature_id[782].value > threshold=5.153738260269165
node_1296: feature_name=GO:0019692 feature_id[584].value <= threshold=0.0685717724263668
node_1297: feature_name=GO:0034103 feature_id[318].value > threshold=6.262972354888916
Class: negative genes

Rules_488 passed counts:1
node_0: feature_name=GO:0042113 feature_id[0].value <= threshold=13.408552169799805
node_1: feature_name=GO:0007568 feature_id[534].value > threshold=5.0313897132873535
node_913: feature_name=GO:0032763 feature_id[629].value > threshold=0.31753237545490265
node_1195: feature_name=GO:0071301 feature_id[442].value <= threshold=0.11534593254327774
node_1196: feature_name=GO:0046500 feature_id[171].value <= threshold=0.6314916908740997
node_1197: feature_name=GO:0030852 feature_id[22].value <= threshold=2.9768868684768677
node_1198: feature_name=hsa04672 feature_id[276].value <= threshold=8.06681227684021
node_1199: feature_name=GO:0001836 feature_id[450].value > threshold=0.09961023926734924
node_1201: feature_name=GO:0070230 feature_id[64].value <= threshold=3.3956379890441895
node_1202: feature_name=GO:0070102 feature_id[49].value <= threshold=4.805386543273926
node_1203: feature_name=GO:0045553 feature_id[736].value <= threshold=1.4970332980155945
node_1204: feature_name=GO:0051311 feature_id[58].value <= threshold=3.301889181137085
node_1205: feature_name=GO:0030291 feature_id[147].value <= threshold=8.156315326690674
node_1206: feature_name=GO:0045637 feature_id[241].value > threshold=0.01834342861548066
node_1208: feature_name=GO:0002890 feature_id[98].value <= threshold=2.767483353614807
node_1209: feature_name=GO:0046632 feature_id[248].value <= threshold=11.663254261016846
node_1210: feature_name=GO:0008588 feature_id[26].value <= threshold=1.391375720500946
node_1211: feature_name=GO:0010639 feature_id[564].value <= threshold=18.975126266479492

node_1212: feature_name=GO:0046685 feature_id[782].value <= threshold=5.153738260269165
node_1213: feature_name=GO:0038116 feature_id[668].value > threshold=1.7859655618667603
node_1293: feature_name=hsa05142 feature_id[769].value > threshold=11.968927383422852
Class: negative genes

Rules_489 passed counts:1
node_0: feature_name=GO:0042113 feature_id[0].value <= threshold=13.408552169799805
node_1: feature_name=GO:0007568 feature_id[534].value > threshold=5.0313897132873535
node_913: feature_name=GO:0032763 feature_id[629].value > threshold=0.31753237545490265
node_1195: feature_name=GO:0071301 feature_id[442].value <= threshold=0.11534593254327774
node_1196: feature_name=GO:0046500 feature_id[171].value <= threshold=0.6314916908740997
node_1197: feature_name=GO:0030852 feature_id[22].value <= threshold=2.9768868684768677
node_1198: feature_name=hsa04672 feature_id[276].value <= threshold=8.06681227684021
node_1199: feature_name=GO:0001836 feature_id[450].value > threshold=0.09961023926734924
node_1201: feature_name=GO:0070230 feature_id[64].value <= threshold=3.3956379890441895
node_1202: feature_name=GO:0070102 feature_id[49].value <= threshold=4.805386543273926
node_1203: feature_name=GO:0045553 feature_id[736].value <= threshold=1.4970332980155945
node_1204: feature_name=GO:0051311 feature_id[58].value <= threshold=3.301889181137085
node_1205: feature_name=GO:0030291 feature_id[147].value <= threshold=8.156315326690674
node_1206: feature_name=GO:0045637 feature_id[241].value > threshold=0.01834342861548066
node_1208: feature_name=GO:0002890 feature_id[98].value <= threshold=2.767483353614807
node_1209: feature_name=GO:0046632 feature_id[248].value <= threshold=11.663254261016846
node_1210: feature_name=GO:0008588 feature_id[26].value <= threshold=1.391375720500946
node_1211: feature_name=GO:0010639 feature_id[564].value <= threshold=18.975126266479492
node_1212: feature_name=GO:0046685 feature_id[782].value <= threshold=5.153738260269165
node_1213: feature_name=GO:0038116 feature_id[668].value <= threshold=1.7859655618667603
node_1214: feature_name=GO:0046449 feature_id[763].value > threshold=5.603115558624268
node_1290: feature_name=GO:0042770 feature_id[688].value > threshold=1.4568010568618774
Class: negative genes

Rules_490 passed counts:1
node_0: feature_name=GO:0042113 feature_id[0].value <= threshold=13.408552169799805
node_1: feature_name=GO:0007568 feature_id[534].value > threshold=5.0313897132873535
node_913: feature_name=GO:0032763 feature_id[629].value > threshold=0.31753237545490265
node_1195: feature_name=GO:0071301 feature_id[442].value <= threshold=0.11534593254327774

node_1196: feature_name=GO:0046500 feature_id[171].value <= threshold=0.6314916908740997
node_1197: feature_name=GO:0030852 feature_id[22].value <= threshold=2.9768868684768677
node_1198: feature_name=hsa04672 feature_id[276].value <= threshold=8.06681227684021
node_1199: feature_name=GO:0001836 feature_id[450].value > threshold=0.09961023926734924
node_1201: feature_name=GO:0070230 feature_id[64].value <= threshold=3.3956379890441895
node_1202: feature_name=GO:0070102 feature_id[49].value <= threshold=4.805386543273926
node_1203: feature_name=GO:0045553 feature_id[736].value <= threshold=1.4970332980155945
node_1204: feature_name=GO:0051311 feature_id[58].value <= threshold=3.301889181137085
node_1205: feature_name=GO:0030291 feature_id[147].value <= threshold=8.156315326690674
node_1206: feature_name=GO:0045637 feature_id[241].value > threshold=0.01834342861548066
node_1208: feature_name=GO:0002890 feature_id[98].value <= threshold=2.767483353614807
node_1209: feature_name=GO:0046632 feature_id[248].value <= threshold=11.663254261016846
node_1210: feature_name=GO:0008588 feature_id[26].value <= threshold=1.391375720500946
node_1211: feature_name=GO:0010639 feature_id[564].value <= threshold=18.975126266479492
node_1212: feature_name=GO:0046685 feature_id[782].value <= threshold=5.153738260269165
node_1213: feature_name=GO:0038116 feature_id[668].value <= threshold=1.7859655618667603
node_1214: feature_name=GO:0046449 feature_id[763].value <= threshold=5.603115558624268
node_1215: feature_name=GO:0048539 feature_id[319].value > threshold=0.5478232800960541
node_1269: feature_name=GO:0072341 feature_id[144].value <= threshold=1.3205850720405579
node_1270: feature_name=GO:0010835 feature_id[560].value > threshold=0.7877185940742493
node_1282: feature_name=GO:0010835 feature_id[560].value <= threshold=0.9053532779216766
node_1283: feature_name=GO:0070741 feature_id[538].value > threshold=4.904514789581299
Class: negative genes

Rules_491 passed counts:1
node_0: feature_name=GO:0042113 feature_id[0].value <= threshold=13.408552169799805
node_1: feature_name=GO:0007568 feature_id[534].value > threshold=5.0313897132873535
node_913: feature_name=GO:0032763 feature_id[629].value > threshold=0.31753237545490265
node_1195: feature_name=GO:0071301 feature_id[442].value <= threshold=0.11534593254327774
node_1196: feature_name=GO:0046500 feature_id[171].value <= threshold=0.6314916908740997
node_1197: feature_name=GO:0030852 feature_id[22].value <= threshold=2.9768868684768677
node_1198: feature_name=hsa04672 feature_id[276].value <= threshold=8.06681227684021
node_1199: feature_name=GO:0001836 feature_id[450].value > threshold=0.09961023926734924
node_1201: feature_name=GO:0070230 feature_id[64].value <= threshold=3.3956379890441895
node_1202: feature_name=GO:0070102 feature_id[49].value <= threshold=4.805386543273926

node_1203: feature_name=GO:0045553 feature_id[736].value <= threshold=1.4970332980155945
node_1204: feature_name=GO:0051311 feature_id[58].value <= threshold=3.301889181137085
node_1205: feature_name=GO:0030291 feature_id[147].value <= threshold=8.156315326690674
node_1206: feature_name=GO:0045637 feature_id[241].value > threshold=0.01834342861548066
node_1208: feature_name=GO:0002890 feature_id[98].value <= threshold=2.767483353614807
node_1209: feature_name=GO:0046632 feature_id[248].value <= threshold=11.663254261016846
node_1210: feature_name=GO:0008588 feature_id[26].value <= threshold=1.391375720500946
node_1211: feature_name=GO:0010639 feature_id[564].value <= threshold=18.975126266479492
node_1212: feature_name=GO:0046685 feature_id[782].value <= threshold=5.153738260269165
node_1213: feature_name=GO:0038116 feature_id[668].value <= threshold=1.7859655618667603
node_1214: feature_name=GO:0046449 feature_id[763].value <= threshold=5.603115558624268
node_1215: feature_name=GO:0048539 feature_id[319].value > threshold=0.5478232800960541
node_1269: feature_name=GO:0072341 feature_id[144].value <= threshold=1.3205850720405579
node_1270: feature_name=GO:0010835 feature_id[560].value <= threshold=0.7877185940742493
node_1271: feature_name=GO:0000097 feature_id[376].value <= threshold=0.9960366785526276
node_1272: feature_name=GO:0042509 feature_id[694].value > threshold=11.929262161254883
Class: positive genes

Rules_492 passed counts:1
node_0: feature_name=GO:0042113 feature_id[0].value <= threshold=13.408552169799805
node_1: feature_name=GO:0007568 feature_id[534].value > threshold=5.0313897132873535
node_913: feature_name=GO:0032763 feature_id[629].value > threshold=0.31753237545490265
node_1195: feature_name=GO:0071301 feature_id[442].value <= threshold=0.11534593254327774
node_1196: feature_name=GO:0046500 feature_id[171].value <= threshold=0.6314916908740997
node_1197: feature_name=GO:0030852 feature_id[22].value <= threshold=2.9768868684768677
node_1198: feature_name=hsa04672 feature_id[276].value <= threshold=8.06681227684021
node_1199: feature_name=GO:0001836 feature_id[450].value > threshold=0.09961023926734924
node_1201: feature_name=GO:0070230 feature_id[64].value <= threshold=3.3956379890441895
node_1202: feature_name=GO:0070102 feature_id[49].value <= threshold=4.805386543273926
node_1203: feature_name=GO:0045553 feature_id[736].value <= threshold=1.4970332980155945
node_1204: feature_name=GO:0051311 feature_id[58].value <= threshold=3.301889181137085
node_1205: feature_name=GO:0030291 feature_id[147].value <= threshold=8.156315326690674
node_1206: feature_name=GO:0045637 feature_id[241].value > threshold=0.01834342861548066
node_1208: feature_name=GO:0002890 feature_id[98].value <= threshold=2.767483353614807
node_1209: feature_name=GO:0046632 feature_id[248].value <= threshold=11.663254261016846

node_1210: feature_name=GO:0008588 feature_id[26].value <= threshold=1.391375720500946
node_1211: feature_name=GO:0010639 feature_id[564].value <= threshold=18.975126266479492
node_1212: feature_name=GO:0046685 feature_id[782].value <= threshold=5.153738260269165
node_1213: feature_name=GO:0038116 feature_id[668].value <= threshold=1.7859655618667603
node_1214: feature_name=GO:0046449 feature_id[763].value <= threshold=5.603115558624268
node_1215: feature_name=GO:0048539 feature_id[319].value > threshold=0.5478232800960541
node_1269: feature_name=GO:0072341 feature_id[144].value <= threshold=1.3205850720405579
node_1270: feature_name=GO:0010835 feature_id[560].value <= threshold=0.7877185940742493
node_1271: feature_name=GO:0000097 feature_id[376].value <= threshold=0.9960366785526276
node_1272: feature_name=GO:0042509 feature_id[694].value <= threshold=11.929262161254883
node_1273: feature_name=GO:0010216 feature_id[282].value > threshold=2.920253038406372
Class: positive genes

Rules_493 passed counts:1
node_0: feature_name=GO:0042113 feature_id[0].value <= threshold=13.408552169799805
node_1: feature_name=GO:0007568 feature_id[534].value > threshold=5.0313897132873535
node_913: feature_name=GO:0032763 feature_id[629].value > threshold=0.31753237545490265
node_1195: feature_name=GO:0071301 feature_id[442].value <= threshold=0.11534593254327774
node_1196: feature_name=GO:0046500 feature_id[171].value <= threshold=0.6314916908740997
node_1197: feature_name=GO:0030852 feature_id[22].value <= threshold=2.9768868684768677
node_1198: feature_name=hsa04672 feature_id[276].value <= threshold=8.06681227684021
node_1199: feature_name=GO:0001836 feature_id[450].value > threshold=0.09961023926734924
node_1201: feature_name=GO:0070230 feature_id[64].value <= threshold=3.3956379890441895
node_1202: feature_name=GO:0070102 feature_id[49].value <= threshold=4.805386543273926
node_1203: feature_name=GO:0045553 feature_id[736].value <= threshold=1.4970332980155945
node_1204: feature_name=GO:0051311 feature_id[58].value <= threshold=3.301889181137085
node_1205: feature_name=GO:0030291 feature_id[147].value <= threshold=8.156315326690674
node_1206: feature_name=GO:0045637 feature_id[241].value > threshold=0.01834342861548066
node_1208: feature_name=GO:0002890 feature_id[98].value <= threshold=2.767483353614807
node_1209: feature_name=GO:0046632 feature_id[248].value <= threshold=11.663254261016846
node_1210: feature_name=GO:0008588 feature_id[26].value <= threshold=1.391375720500946
node_1211: feature_name=GO:0010639 feature_id[564].value <= threshold=18.975126266479492
node_1212: feature_name=GO:0046685 feature_id[782].value <= threshold=5.153738260269165
node_1213: feature_name=GO:0038116 feature_id[668].value <= threshold=1.7859655618667603
node_1214: feature_name=GO:0046449 feature_id[763].value <= threshold=5.603115558624268

node_1215: feature_name=GO:0048539 feature_id[319].value > threshold=0.5478232800960541
node_1269: feature_name=GO:0072341 feature_id[144].value <= threshold=1.3205850720405579
node_1270: feature_name=GO:0010835 feature_id[560].value <= threshold=0.7877185940742493
node_1271: feature_name=GO:0000097 feature_id[376].value <= threshold=0.9960366785526276
node_1272: feature_name=GO:0042509 feature_id[694].value <= threshold=11.929262161254883
node_1273: feature_name=GO:0010216 feature_id[282].value <= threshold=2.920253038406372
node_1274: feature_name=hsa04650 feature_id[13].value <= threshold=0.01315402565523982
Class: positive genes

Rules_494 passed counts:1
node_0: feature_name=GO:0042113 feature_id[0].value <= threshold=13.408552169799805
node_1: feature_name=GO:0007568 feature_id[534].value > threshold=5.0313897132873535
node_913: feature_name=GO:0032763 feature_id[629].value > threshold=0.31753237545490265
node_1195: feature_name=GO:0071301 feature_id[442].value <= threshold=0.11534593254327774
node_1196: feature_name=GO:0046500 feature_id[171].value <= threshold=0.6314916908740997
node_1197: feature_name=GO:0030852 feature_id[22].value <= threshold=2.9768868684768677
node_1198: feature_name=hsa04672 feature_id[276].value <= threshold=8.06681227684021
node_1199: feature_name=GO:0001836 feature_id[450].value > threshold=0.09961023926734924
node_1201: feature_name=GO:0070230 feature_id[64].value <= threshold=3.3956379890441895
node_1202: feature_name=GO:0070102 feature_id[49].value <= threshold=4.805386543273926
node_1203: feature_name=GO:0045553 feature_id[736].value <= threshold=1.4970332980155945
node_1204: feature_name=GO:0051311 feature_id[58].value <= threshold=3.301889181137085
node_1205: feature_name=GO:0030291 feature_id[147].value <= threshold=8.156315326690674
node_1206: feature_name=GO:0045637 feature_id[241].value > threshold=0.01834342861548066
node_1208: feature_name=GO:0002890 feature_id[98].value <= threshold=2.767483353614807
node_1209: feature_name=GO:0046632 feature_id[248].value <= threshold=11.663254261016846
node_1210: feature_name=GO:0008588 feature_id[26].value <= threshold=1.391375720500946
node_1211: feature_name=GO:0010639 feature_id[564].value <= threshold=18.975126266479492
node_1212: feature_name=GO:0046685 feature_id[782].value <= threshold=5.153738260269165
node_1213: feature_name=GO:0038116 feature_id[668].value <= threshold=1.7859655618667603
node_1214: feature_name=GO:0046449 feature_id[763].value <= threshold=5.603115558624268
node_1215: feature_name=GO:0048539 feature_id[319].value <= threshold=0.5478232800960541
node_1216: feature_name=GO:0007089 feature_id[522].value > threshold=0.9940084517002106
node_1266: feature_name=GO:0042129 feature_id[593].value <= threshold=0.9326772093772888
Class: negative genes

Rules_495	passed counts:1
node_0: feature_name=GO:0042113	feature_id[0].value <= threshold=13.408552169799805
node_1: feature_name=GO:0007568	feature_id[534].value > threshold=5.0313897132873535
node_913: feature_name=GO:0032763	feature_id[629].value > threshold=0.31753237545490265
node_1195: feature_name=GO:0071301	feature_id[442].value <= threshold=0.11534593254327774
node_1196: feature_name=GO:0046500	feature_id[171].value <= threshold=0.6314916908740997
node_1197: feature_name=GO:0030852	feature_id[22].value <= threshold=2.9768868684768677
node_1198: feature_name=hsa04672	feature_id[276].value <= threshold=8.06681227684021
node_1199: feature_name=GO:0001836	feature_id[450].value > threshold=0.09961023926734924
node_1201: feature_name=GO:0070230	feature_id[64].value <= threshold=3.3956379890441895
node_1202: feature_name=GO:0070102	feature_id[49].value <= threshold=4.805386543273926
node_1203: feature_name=GO:0045553	feature_id[736].value <= threshold=1.4970332980155945
node_1204: feature_name=GO:0051311	feature_id[58].value <= threshold=3.301889181137085
node_1205: feature_name=GO:0030291	feature_id[147].value <= threshold=8.156315326690674
node_1206: feature_name=GO:0045637	feature_id[241].value > threshold=0.01834342861548066
node_1208: feature_name=GO:0002890	feature_id[98].value <= threshold=2.767483353614807
node_1209: feature_name=GO:0046632	feature_id[248].value <= threshold=11.663254261016846
node_1210: feature_name=GO:0008588	feature_id[26].value <= threshold=1.391375720500946
node_1211: feature_name=GO:0010639	feature_id[564].value <= threshold=18.975126266479492
node_1212: feature_name=GO:0046685	feature_id[782].value <= threshold=5.153738260269165
node_1213: feature_name=GO:0038116	feature_id[668].value <= threshold=1.7859655618667603
node_1214: feature_name=GO:0046449	feature_id[763].value <= threshold=5.603115558624268
node_1215: feature_name=GO:0048539	feature_id[319].value <= threshold=0.5478232800960541
node_1216: feature_name=GO:0007089	feature_id[522].value <= threshold=0.9940084517002106
node_1217: feature_name=GO:0042287	feature_id[20].value <= threshold=2.693045496940613
node_1218: feature_name=GO:0044446	feature_id[184].value > threshold=113.0758171081543
Class: positive genes	

Rules_496	passed counts:1
node_0: feature_name=GO:0042113	feature_id[0].value <= threshold=13.408552169799805
node_1: feature_name=GO:0007568	feature_id[534].value > threshold=5.0313897132873535
node_913: feature_name=GO:0032763	feature_id[629].value > threshold=0.31753237545490265
node_1195: feature_name=GO:0071301	feature_id[442].value <= threshold=0.11534593254327774
node_1196: feature_name=GO:0046500	feature_id[171].value <= threshold=0.6314916908740997

node_1197: feature_name=GO:0030852 feature_id[22].value <= threshold=2.9768868684768677
node_1198: feature_name=hsa04672 feature_id[276].value <= threshold=8.06681227684021
node_1199: feature_name=GO:0001836 feature_id[450].value > threshold=0.09961023926734924
node_1201: feature_name=GO:0070230 feature_id[64].value <= threshold=3.3956379890441895
node_1202: feature_name=GO:0070102 feature_id[49].value <= threshold=4.805386543273926
node_1203: feature_name=GO:0045553 feature_id[736].value <= threshold=1.4970332980155945
node_1204: feature_name=GO:0051311 feature_id[58].value <= threshold=3.301889181137085
node_1205: feature_name=GO:0030291 feature_id[147].value <= threshold=8.156315326690674
node_1206: feature_name=GO:0045637 feature_id[241].value > threshold=0.01834342861548066
node_1208: feature_name=GO:0002890 feature_id[98].value <= threshold=2.767483353614807
node_1209: feature_name=GO:0046632 feature_id[248].value <= threshold=11.663254261016846
node_1210: feature_name=GO:0008588 feature_id[26].value <= threshold=1.391375720500946
node_1211: feature_name=GO:0010639 feature_id[564].value <= threshold=18.975126266479492
node_1212: feature_name=GO:0046685 feature_id[782].value <= threshold=5.153738260269165
node_1213: feature_name=GO:0038116 feature_id[668].value <= threshold=1.7859655618667603
node_1214: feature_name=GO:0046449 feature_id[763].value <= threshold=5.603115558624268
node_1215: feature_name=GO:0048539 feature_id[319].value <= threshold=0.5478232800960541
node_1216: feature_name=GO:0007089 feature_id[522].value <= threshold=0.9940084517002106
node_1217: feature_name=GO:0042287 feature_id[20].value <= threshold=2.693045496940613
node_1218: feature_name=GO:0044446 feature_id[184].value <= threshold=113.0758171081543
node_1219: feature_name=GO:0098602 feature_id[702].value > threshold=68.95328903198242
Class: positive genes

Rules_497 passed counts:1
node_0: feature_name=GO:0042113 feature_id[0].value <= threshold=13.408552169799805
node_1: feature_name=GO:0007568 feature_id[534].value > threshold=5.0313897132873535
node_913: feature_name=GO:0032763 feature_id[629].value > threshold=0.31753237545490265
node_1195: feature_name=GO:0071301 feature_id[442].value <= threshold=0.11534593254327774
node_1196: feature_name=GO:0046500 feature_id[171].value <= threshold=0.6314916908740997
node_1197: feature_name=GO:0030852 feature_id[22].value <= threshold=2.9768868684768677
node_1198: feature_name=hsa04672 feature_id[276].value <= threshold=8.06681227684021
node_1199: feature_name=GO:0001836 feature_id[450].value > threshold=0.09961023926734924
node_1201: feature_name=GO:0070230 feature_id[64].value <= threshold=3.3956379890441895
node_1202: feature_name=GO:0070102 feature_id[49].value <= threshold=4.805386543273926
node_1203: feature_name=GO:0045553 feature_id[736].value <= threshold=1.4970332980155945

node_1204: feature_name=GO:0051311 feature_id[58].value <= threshold=3.301889181137085
node_1205: feature_name=GO:0030291 feature_id[147].value <= threshold=8.156315326690674
node_1206: feature_name=GO:0045637 feature_id[241].value > threshold=0.01834342861548066
node_1208: feature_name=GO:0002890 feature_id[98].value <= threshold=2.767483353614807
node_1209: feature_name=GO:0046632 feature_id[248].value <= threshold=11.663254261016846
node_1210: feature_name=GO:0008588 feature_id[26].value <= threshold=1.391375720500946
node_1211: feature_name=GO:0010639 feature_id[564].value <= threshold=18.975126266479492
node_1212: feature_name=GO:0046685 feature_id[782].value <= threshold=5.153738260269165
node_1213: feature_name=GO:0038116 feature_id[668].value <= threshold=1.7859655618667603
node_1214: feature_name=GO:0046449 feature_id[763].value <= threshold=5.603115558624268
node_1215: feature_name=GO:0048539 feature_id[319].value <= threshold=0.5478232800960541
node_1216: feature_name=GO:0007089 feature_id[522].value <= threshold=0.9940084517002106
node_1217: feature_name=GO:0042287 feature_id[20].value <= threshold=2.693045496940613
node_1218: feature_name=GO:0044446 feature_id[184].value <= threshold=113.0758171081543
node_1219: feature_name=GO:0098602 feature_id[702].value <= threshold=68.95328903198242
node_1220: feature_name=GO:0035726 feature_id[270].value <= threshold=1.7167096138000488
node_1221: feature_name=GO:1902564 feature_id[85].value > threshold=2.8364468812942505
Class: positive genes

Rules_498 passed counts:1
node_0: feature_name=GO:0042113 feature_id[0].value <= threshold=13.408552169799805
node_1: feature_name=GO:0007568 feature_id[534].value > threshold=5.0313897132873535
node_913: feature_name=GO:0032763 feature_id[629].value > threshold=0.31753237545490265
node_1195: feature_name=GO:0071301 feature_id[442].value <= threshold=0.11534593254327774
node_1196: feature_name=GO:0046500 feature_id[171].value <= threshold=0.6314916908740997
node_1197: feature_name=GO:0030852 feature_id[22].value <= threshold=2.9768868684768677
node_1198: feature_name=hsa04672 feature_id[276].value <= threshold=8.06681227684021
node_1199: feature_name=GO:0001836 feature_id[450].value > threshold=0.09961023926734924
node_1201: feature_name=GO:0070230 feature_id[64].value <= threshold=3.3956379890441895
node_1202: feature_name=GO:0070102 feature_id[49].value <= threshold=4.805386543273926
node_1203: feature_name=GO:0045553 feature_id[736].value <= threshold=1.4970332980155945
node_1204: feature_name=GO:0051311 feature_id[58].value <= threshold=3.301889181137085
node_1205: feature_name=GO:0030291 feature_id[147].value <= threshold=8.156315326690674
node_1206: feature_name=GO:0045637 feature_id[241].value > threshold=0.01834342861548066
node_1208: feature_name=GO:0002890 feature_id[98].value <= threshold=2.767483353614807

node_1209: feature_name=GO:0046632 feature_id[248].value <= threshold=11.663254261016846
node_1210: feature_name=GO:0008588 feature_id[26].value <= threshold=1.391375720500946
node_1211: feature_name=GO:0010639 feature_id[564].value <= threshold=18.975126266479492
node_1212: feature_name=GO:0046685 feature_id[782].value <= threshold=5.153738260269165
node_1213: feature_name=GO:0038116 feature_id[668].value <= threshold=1.7859655618667603
node_1214: feature_name=GO:0046449 feature_id[763].value <= threshold=5.603115558624268
node_1215: feature_name=GO:0048539 feature_id[319].value <= threshold=0.5478232800960541
node_1216: feature_name=GO:0007089 feature_id[522].value <= threshold=0.9940084517002106
node_1217: feature_name=GO:0042287 feature_id[20].value <= threshold=2.693045496940613
node_1218: feature_name=GO:0044446 feature_id[184].value <= threshold=113.0758171081543
node_1219: feature_name=GO:0098602 feature_id[702].value <= threshold=68.95328903198242
node_1220: feature_name=GO:0035726 feature_id[270].value <= threshold=1.7167096138000488
node_1221: feature_name=GO:1902564 feature_id[85].value <= threshold=2.8364468812942505
node_1222: feature_name=GO:0006979 feature_id[44].value > threshold=0.6869173645973206
node_1224: feature_name=GO:0044464 feature_id[56].value > threshold=67.13260269165039
Class: positive genes

Rules_499 passed counts:1
node_0: feature_name=GO:0042113 feature_id[0].value <= threshold=13.408552169799805
node_1: feature_name=GO:0007568 feature_id[534].value > threshold=5.0313897132873535
node_913: feature_name=GO:0032763 feature_id[629].value > threshold=0.31753237545490265
node_1195: feature_name=GO:0071301 feature_id[442].value <= threshold=0.11534593254327774
node_1196: feature_name=GO:0046500 feature_id[171].value <= threshold=0.6314916908740997
node_1197: feature_name=GO:0030852 feature_id[22].value <= threshold=2.9768868684768677
node_1198: feature_name=hsa04672 feature_id[276].value <= threshold=8.06681227684021
node_1199: feature_name=GO:0001836 feature_id[450].value > threshold=0.09961023926734924
node_1201: feature_name=GO:0070230 feature_id[64].value <= threshold=3.3956379890441895
node_1202: feature_name=GO:0070102 feature_id[49].value <= threshold=4.805386543273926
node_1203: feature_name=GO:0045553 feature_id[736].value <= threshold=1.4970332980155945
node_1204: feature_name=GO:0051311 feature_id[58].value <= threshold=3.301889181137085
node_1205: feature_name=GO:0030291 feature_id[147].value <= threshold=8.156315326690674
node_1206: feature_name=GO:0045637 feature_id[241].value > threshold=0.01834342861548066
node_1208: feature_name=GO:0002890 feature_id[98].value <= threshold=2.767483353614807
node_1209: feature_name=GO:0046632 feature_id[248].value <= threshold=11.663254261016846
node_1210: feature_name=GO:0008588 feature_id[26].value <= threshold=1.391375720500946

node_1211: feature_name=GO:0010639 feature_id[564].value <= threshold=18.975126266479492
node_1212: feature_name=GO:0046685 feature_id[782].value <= threshold=5.153738260269165
node_1213: feature_name=GO:0038116 feature_id[668].value <= threshold=1.7859655618667603
node_1214: feature_name=GO:0046449 feature_id[763].value <= threshold=5.603115558624268
node_1215: feature_name=GO:0048539 feature_id[319].value <= threshold=0.5478232800960541
node_1216: feature_name=GO:0007089 feature_id[522].value <= threshold=0.9940084517002106
node_1217: feature_name=GO:0042287 feature_id[20].value <= threshold=2.693045496940613
node_1218: feature_name=GO:0044446 feature_id[184].value <= threshold=113.0758171081543
node_1219: feature_name=GO:0098602 feature_id[702].value <= threshold=68.95328903198242
node_1220: feature_name=GO:0035726 feature_id[270].value <= threshold=1.7167096138000488
node_1221: feature_name=GO:1902564 feature_id[85].value <= threshold=2.8364468812942505
node_1222: feature_name=GO:0006979 feature_id[44].value > threshold=0.6869173645973206
node_1224: feature_name=GO:0044464 feature_id[56].value <= threshold=67.13260269165039
node_1225: feature_name=GO:0045629 feature_id[746].value > threshold=2.3414241075515747
Class: positive genes

Rules_500 passed counts:1
node_0: feature_name=GO:0042113 feature_id[0].value <= threshold=13.408552169799805
node_1: feature_name=GO:0007568 feature_id[534].value > threshold=5.0313897132873535
node_913: feature_name=GO:0032763 feature_id[629].value > threshold=0.31753237545490265
node_1195: feature_name=GO:0071301 feature_id[442].value <= threshold=0.11534593254327774
node_1196: feature_name=GO:0046500 feature_id[171].value <= threshold=0.6314916908740997
node_1197: feature_name=GO:0030852 feature_id[22].value <= threshold=2.9768868684768677
node_1198: feature_name=hsa04672 feature_id[276].value <= threshold=8.06681227684021
node_1199: feature_name=GO:0001836 feature_id[450].value > threshold=0.09961023926734924
node_1201: feature_name=GO:0070230 feature_id[64].value <= threshold=3.3956379890441895
node_1202: feature_name=GO:0070102 feature_id[49].value <= threshold=4.805386543273926
node_1203: feature_name=GO:0045553 feature_id[736].value <= threshold=1.4970332980155945
node_1204: feature_name=GO:0051311 feature_id[58].value <= threshold=3.301889181137085
node_1205: feature_name=GO:0030291 feature_id[147].value <= threshold=8.156315326690674
node_1206: feature_name=GO:0045637 feature_id[241].value > threshold=0.01834342861548066
node_1208: feature_name=GO:0002890 feature_id[98].value <= threshold=2.767483353614807
node_1209: feature_name=GO:0046632 feature_id[248].value <= threshold=11.663254261016846
node_1210: feature_name=GO:0008588 feature_id[26].value <= threshold=1.391375720500946
node_1211: feature_name=GO:0010639 feature_id[564].value <= threshold=18.975126266479492

node_1212: feature_name=GO:0046685 feature_id[782].value <= threshold=5.153738260269165
node_1213: feature_name=GO:0038116 feature_id[668].value <= threshold=1.7859655618667603
node_1214: feature_name=GO:0046449 feature_id[763].value <= threshold=5.603115558624268
node_1215: feature_name=GO:0048539 feature_id[319].value <= threshold=0.5478232800960541
node_1216: feature_name=GO:0007089 feature_id[522].value <= threshold=0.9940084517002106
node_1217: feature_name=GO:0042287 feature_id[20].value <= threshold=2.693045496940613
node_1218: feature_name=GO:0044446 feature_id[184].value <= threshold=113.0758171081543
node_1219: feature_name=GO:0098602 feature_id[702].value <= threshold=68.95328903198242
node_1220: feature_name=GO:0035726 feature_id[270].value <= threshold=1.7167096138000488
node_1221: feature_name=GO:1902564 feature_id[85].value <= threshold=2.8364468812942505
node_1222: feature_name=GO:0006979 feature_id[44].value > threshold=0.6869173645973206
node_1224: feature_name=GO:0044464 feature_id[56].value <= threshold=67.13260269165039
node_1225: feature_name=GO:0045629 feature_id[746].value <= threshold=2.3414241075515747
node_1226: feature_name=GO:0002903 feature_id[10].value <= threshold=2.715258240699768
node_1227: feature_name=GO:0043375 feature_id[717].value > threshold=1.9525017738342285
node_1249: feature_name=GO:0038061 feature_id[671].value > threshold=2.6873637437820435
Class: negative genes

Rules_501 passed counts:1
node_0: feature_name=GO:0042113 feature_id[0].value <= threshold=13.408552169799805
node_1: feature_name=GO:0007568 feature_id[534].value > threshold=5.0313897132873535
node_913: feature_name=GO:0032763 feature_id[629].value > threshold=0.31753237545490265
node_1195: feature_name=GO:0071301 feature_id[442].value <= threshold=0.11534593254327774
node_1196: feature_name=GO:0046500 feature_id[171].value <= threshold=0.6314916908740997
node_1197: feature_name=GO:0030852 feature_id[22].value <= threshold=2.9768868684768677
node_1198: feature_name=hsa04672 feature_id[276].value <= threshold=8.06681227684021
node_1199: feature_name=GO:0001836 feature_id[450].value > threshold=0.09961023926734924
node_1201: feature_name=GO:0070230 feature_id[64].value <= threshold=3.3956379890441895
node_1202: feature_name=GO:0070102 feature_id[49].value <= threshold=4.805386543273926
node_1203: feature_name=GO:0045553 feature_id[736].value <= threshold=1.4970332980155945
node_1204: feature_name=GO:0051311 feature_id[58].value <= threshold=3.301889181137085
node_1205: feature_name=GO:0030291 feature_id[147].value <= threshold=8.156315326690674
node_1206: feature_name=GO:0045637 feature_id[241].value > threshold=0.018343428615480
node_1208: feature_name=GO:0002890 feature_id[98].value <= threshold=2.767483353614807
node_1209: feature_name=GO:0046632 feature_id[248].value <= threshold=11.663254261016846

node_1210: feature_name=GO:0008588 feature_id[26].value <= threshold=1.391375720500946
node_1211: feature_name=GO:0010639 feature_id[564].value <= threshold=18.975126266479492
node_1212: feature_name=GO:0046685 feature_id[782].value <= threshold=5.153738260269165
node_1213: feature_name=GO:0038116 feature_id[668].value <= threshold=1.7859655618667603
node_1214: feature_name=GO:0046449 feature_id[763].value <= threshold=5.603115558624268
node_1215: feature_name=GO:0048539 feature_id[319].value <= threshold=0.5478232800960541
node_1216: feature_name=GO:0007089 feature_id[522].value <= threshold=0.9940084517002106
node_1217: feature_name=GO:0042287 feature_id[20].value <= threshold=2.693045496940613
node_1218: feature_name=GO:0044446 feature_id[184].value <= threshold=113.0758171081543
node_1219: feature_name=GO:0098602 feature_id[702].value <= threshold=68.95328903198242
node_1220: feature_name=GO:0035726 feature_id[270].value <= threshold=1.7167096138000488
node_1221: feature_name=GO:1902564 feature_id[85].value <= threshold=2.8364468812942505
node_1222: feature_name=GO:0006979 feature_id[44].value > threshold=0.6869173645973206
node_1224: feature_name=GO:0044464 feature_id[56].value <= threshold=67.13260269165039
node_1225: feature_name=GO:0045629 feature_id[746].value <= threshold=2.3414241075515747
node_1226: feature_name=GO:0002903 feature_id[10].value <= threshold=2.715258240699768
node_1227: feature_name=GO:0043375 feature_id[717].value > threshold=1.9525017738342285
node_1249: feature_name=GO:0038061 feature_id[671].value <= threshold=2.6873637437820435
Class: positive genes

Rules_502 passed counts:1
node_0: feature_name=GO:0042113 feature_id[0].value <= threshold=13.408552169799805
node_1: feature_name=GO:0007568 feature_id[534].value > threshold=5.0313897132873535
node_913: feature_name=GO:0032763 feature_id[629].value > threshold=0.31753237545490265
node_1195: feature_name=GO:0071301 feature_id[442].value <= threshold=0.11534593254327774
node_1196: feature_name=GO:0046500 feature_id[171].value <= threshold=0.6314916908740997
node_1197: feature_name=GO:0030852 feature_id[22].value <= threshold=2.9768868684768677
node_1198: feature_name=hsa04672 feature_id[276].value <= threshold=8.06681227684021
node_1199: feature_name=GO:0001836 feature_id[450].value > threshold=0.09961023926734924
node_1201: feature_name=GO:0070230 feature_id[64].value <= threshold=3.3956379890441895
node_1202: feature_name=GO:0070102 feature_id[49].value <= threshold=4.805386543273926
node_1203: feature_name=GO:0045553 feature_id[736].value <= threshold=1.4970332980155945
node_1204: feature_name=GO:0051311 feature_id[58].value <= threshold=3.301889181137085
node_1205: feature_name=GO:0030291 feature_id[147].value <= threshold=8.156315326690674
node_1206: feature_name=GO:0045637 feature_id[241].value > threshold=0.01834342861548066

node_1208: feature_name=GO:0002890 feature_id[98].value <= threshold=2.767483353614807
node_1209: feature_name=GO:0046632 feature_id[248].value <= threshold=11.663254261016846
node_1210: feature_name=GO:0008588 feature_id[26].value <= threshold=1.391375720500946
node_1211: feature_name=GO:0010639 feature_id[564].value <= threshold=18.975126266479492
node_1212: feature_name=GO:0046685 feature_id[782].value <= threshold=5.153738260269165
node_1213: feature_name=GO:0038116 feature_id[668].value <= threshold=1.7859655618667603
node_1214: feature_name=GO:0046449 feature_id[763].value <= threshold=5.603115558624268
node_1215: feature_name=GO:0048539 feature_id[319].value <= threshold=0.5478232800960541
node_1216: feature_name=GO:0007089 feature_id[522].value <= threshold=0.9940084517002106
node_1217: feature_name=GO:0042287 feature_id[20].value <= threshold=2.693045496940613
node_1218: feature_name=GO:0044446 feature_id[184].value <= threshold=113.0758171081543
node_1219: feature_name=GO:0098602 feature_id[702].value <= threshold=68.95328903198242
node_1220: feature_name=GO:0035726 feature_id[270].value <= threshold=1.7167096138000488
node_1221: feature_name=GO:1902564 feature_id[85].value <= threshold=2.8364468812942505
node_1222: feature_name=GO:0006979 feature_id[44].value > threshold=0.6869173645973206
node_1224: feature_name=GO:0044464 feature_id[56].value <= threshold=67.13260269165039
node_1225: feature_name=GO:0045629 feature_id[746].value <= threshold=2.3414241075515747
node_1226: feature_name=GO:0002903 feature_id[10].value <= threshold=2.715258240699768
node_1227: feature_name=GO:0043375 feature_id[717].value <= threshold=1.9525017738342285
node_1228: feature_name=GO:0032762 feature_id[630].value > threshold=0.29527929425239563
node_1232: feature_name=GO:0048569 feature_id[793].value <= threshold=3.419509768486023
node_1233: feature_name=GO:0045577 feature_id[109].value > threshold=4.633661270141602
node_1243: feature_name=GO:0006139 feature_id[474].value <= threshold=0.37312396615743637
Class: positive genes

Rules_503 passed counts:1
node_0: feature_name=GO:0042113 feature_id[0].value <= threshold=13.408552169799805
node_1: feature_name=GO:0007568 feature_id[534].value > threshold=5.0313897132873535
node_913: feature_name=GO:0032763 feature_id[629].value > threshold=0.31753237545490265
node_1195: feature_name=GO:0071301 feature_id[442].value <= threshold=0.11534593254327774
node_1196: feature_name=GO:0046500 feature_id[171].value <= threshold=0.6314916908740997
node_1197: feature_name=GO:0030852 feature_id[22].value <= threshold=2.9768868684768677
node_1198: feature_name=hsa04672 feature_id[276].value <= threshold=8.06681227684021
node_1199: feature_name=GO:0001836 feature_id[450].value > threshold=0.09961023926734924
node_1201: feature_name=GO:0070230 feature_id[64].value <= threshold=3.3956379890441895

node_1202: feature_name=GO:0070102	feature_id[49].value <= threshold=4.805386543273926
node_1203: feature_name=GO:0045553	feature_id[736].value <= threshold=1.4970332980155945
node_1204: feature_name=GO:0051311	feature_id[58].value <= threshold=3.301889181137085
node_1205: feature_name=GO:0030291	feature_id[147].value <= threshold=8.156315326690674
node_1206: feature_name=GO:0045637	feature_id[241].value > threshold=0.01834342861548066
node_1208: feature_name=GO:0002890	feature_id[98].value <= threshold=2.767483353614807
node_1209: feature_name=GO:0046632	feature_id[248].value <= threshold=11.663254261016846
node_1210: feature_name=GO:0008588	feature_id[26].value <= threshold=1.391375720500946
node_1211: feature_name=GO:0010639	feature_id[564].value <= threshold=18.975126266479492
node_1212: feature_name=GO:0046685	feature_id[782].value <= threshold=5.153738260269165
node_1213: feature_name=GO:0038116	feature_id[668].value <= threshold=1.7859655618667603
node_1214: feature_name=GO:0046449	feature_id[763].value <= threshold=5.603115558624268
node_1215: feature_name=GO:0048539	feature_id[319].value <= threshold=0.5478232800960541
node_1216: feature_name=GO:0007089	feature_id[522].value <= threshold=0.9940084517002106
node_1217: feature_name=GO:0042287	feature_id[20].value <= threshold=2.693045496940613
node_1218: feature_name=GO:0044446	feature_id[184].value <= threshold=113.0758171081543
node_1219: feature_name=GO:0098602	feature_id[702].value <= threshold=68.95328903198242
node_1220: feature_name=GO:0035726	feature_id[270].value <= threshold=1.7167096138000488
node_1221: feature_name=GO:1902564	feature_id[85].value <= threshold=2.8364468812942505
node_1222: feature_name=GO:0006979	feature_id[44].value > threshold=0.6869173645973206
node_1224: feature_name=GO:0044464	feature_id[56].value <= threshold=67.13260269165039
node_1225: feature_name=GO:0045629	feature_id[746].value <= threshold=2.3414241075515747
node_1226: feature_name=GO:0002903	feature_id[10].value <= threshold=2.715258240699768
node_1227: feature_name=GO:0043375	feature_id[717].value <= threshold=1.9525017738342285
node_1228: feature_name=GO:0032762	feature_id[630].value > threshold=0.29527929425239563
node_1232: feature_name=GO:0048569	feature_id[793].value <= threshold=3.419509768486023
node_1233: feature_name=GO:0045577	feature_id[109].value <= threshold=4.633661270141602
node_1234: feature_name=GO:0070245	feature_id[326].value > threshold=2.441925883293152
node_1240: feature_name=GO:0008283	feature_id[415].value <= threshold=8.742806434631348
Class: positive genes	

Rules_504	passed counts:1
node_0: feature_name=GO:0042113	feature_id[0].value <= threshold=13.408552169799805
node_1: feature_name=GO:0007568	feature_id[534].value > threshold=5.0313897132873535
node_913: feature_name=GO:0032763	feature_id[629].value > threshold=0.31753237545490265

node_1195: feature_name=GO:0071301 feature_id[442].value <= threshold=0.11534593254327774
node_1196: feature_name=GO:0046500 feature_id[171].value <= threshold=0.6314916908740997
node_1197: feature_name=GO:0030852 feature_id[22].value <= threshold=2.9768868684768677
node_1198: feature_name=hsa04672 feature_id[276].value <= threshold=8.06681227684021
node_1199: feature_name=GO:0001836 feature_id[450].value > threshold=0.09961023926734924
node_1201: feature_name=GO:0070230 feature_id[64].value <= threshold=3.3956379890441895
node_1202: feature_name=GO:0070102 feature_id[49].value <= threshold=4.805386543273926
node_1203: feature_name=GO:0045553 feature_id[736].value <= threshold=1.4970332980155945
node_1204: feature_name=GO:0051311 feature_id[58].value <= threshold=3.301889181137085
node_1205: feature_name=GO:0030291 feature_id[147].value <= threshold=8.156315326690674
node_1206: feature_name=GO:0045637 feature_id[241].value > threshold=0.01834342861548066
node_1208: feature_name=GO:0002890 feature_id[98].value <= threshold=2.767483353614807
node_1209: feature_name=GO:0046632 feature_id[248].value <= threshold=11.663254261016846
node_1210: feature_name=GO:0008588 feature_id[26].value <= threshold=1.391375720500946
node_1211: feature_name=GO:0010639 feature_id[564].value <= threshold=18.975126266479492
node_1212: feature_name=GO:0046685 feature_id[782].value <= threshold=5.153738260269165
node_1213: feature_name=GO:0038116 feature_id[668].value <= threshold=1.7859655618667603
node_1214: feature_name=GO:0046449 feature_id[763].value <= threshold=5.603115558624268
node_1215: feature_name=GO:0048539 feature_id[319].value <= threshold=0.5478232800960541
node_1216: feature_name=GO:0007089 feature_id[522].value <= threshold=0.9940084517002106
node_1217: feature_name=GO:0042287 feature_id[20].value <= threshold=2.693045496940613
node_1218: feature_name=GO:0044446 feature_id[184].value <= threshold=113.0758171081543
node_1219: feature_name=GO:0098602 feature_id[702].value <= threshold=68.95328903198242
node_1220: feature_name=GO:0035726 feature_id[270].value <= threshold=1.7167096138000488
node_1221: feature_name=GO:1902564 feature_id[85].value <= threshold=2.8364468812942505
node_1222: feature_name=GO:0006979 feature_id[44].value > threshold=0.6869173645973206
node_1224: feature_name=GO:0044464 feature_id[56].value <= threshold=67.13260269165039
node_1225: feature_name=GO:0045629 feature_id[746].value <= threshold=2.3414241075515747
node_1226: feature_name=GO:0002903 feature_id[10].value <= threshold=2.715258240699768
node_1227: feature_name=GO:0043375 feature_id[717].value <= threshold=1.9525017738342285
node_1228: feature_name=GO:0032762 feature_id[630].value > threshold=0.29527929425239563
node_1232: feature_name=GO:0048569 feature_id[793].value <= threshold=3.419509768486023
node_1233: feature_name=GO:0045577 feature_id[109].value <= threshold=4.633661270141602
node_1234: feature_name=GO:0070245 feature_id[326].value <= threshold=2.441925883293152
node_1235: feature_name=GO:0002864 feature_id[488].value > threshold=3.03904390335083

node_1237: feature_name=GO:0002260 feature_id[396].value <= threshold=0.4489758163690567
Class: positive genes

Rules_505 passed counts:1
node_0: feature_name=GO:0042113 feature_id[0].value <= threshold=13.408552169799805
node_1: feature_name=GO:0007568 feature_id[534].value > threshold=5.0313897132873535
node_913: feature_name=GO:0032763 feature_id[629].value > threshold=0.31753237545490265
node_1195: feature_name=GO:0071301 feature_id[442].value <= threshold=0.11534593254327774
node_1196: feature_name=GO:0046500 feature_id[171].value <= threshold=0.6314916908740997
node_1197: feature_name=GO:0030852 feature_id[22].value <= threshold=2.9768868684768677
node_1198: feature_name=hsa04672 feature_id[276].value <= threshold=8.06681227684021
node_1199: feature_name=GO:0001836 feature_id[450].value > threshold=0.09961023926734924
node_1201: feature_name=GO:0070230 feature_id[64].value <= threshold=3.3956379890441895
node_1202: feature_name=GO:0070102 feature_id[49].value <= threshold=4.805386543273926
node_1203: feature_name=GO:0045553 feature_id[736].value <= threshold=1.4970332980155945
node_1204: feature_name=GO:0051311 feature_id[58].value <= threshold=3.301889181137085
node_1205: feature_name=GO:0030291 feature_id[147].value <= threshold=8.156315326690674
node_1206: feature_name=GO:0045637 feature_id[241].value > threshold=0.01834342861548066
node_1208: feature_name=GO:0002890 feature_id[98].value <= threshold=2.767483353614807
node_1209: feature_name=GO:0046632 feature_id[248].value <= threshold=11.663254261016846
node_1210: feature_name=GO:0008588 feature_id[26].value <= threshold=1.391375720500946
node_1211: feature_name=GO:0010639 feature_id[564].value <= threshold=18.975126266479492
node_1212: feature_name=GO:0046685 feature_id[782].value <= threshold=5.153738260269165
node_1213: feature_name=GO:0038116 feature_id[668].value <= threshold=1.7859655618667603
node_1214: feature_name=GO:0046449 feature_id[763].value <= threshold=5.603115558624268
node_1215: feature_name=GO:0048539 feature_id[319].value <= threshold=0.5478232800960541
node_1216: feature_name=GO:0007089 feature_id[522].value <= threshold=0.9940084517002106
node_1217: feature_name=GO:0042287 feature_id[20].value <= threshold=2.693045496940613
node_1218: feature_name=GO:0044446 feature_id[184].value <= threshold=113.0758171081543
node_1219: feature_name=GO:0098602 feature_id[702].value <= threshold=68.95328903198242
node_1220: feature_name=GO:0035726 feature_id[270].value <= threshold=1.7167096138000488
node_1221: feature_name=GO:1902564 feature_id[85].value <= threshold=2.8364468812942505
node_1222: feature_name=GO:0006979 feature_id[44].value > threshold=0.6869173645973206
node_1224: feature_name=GO:0044464 feature_id[56].value <= threshold=67.13260269165039
node_1225: feature_name=GO:0045629 feature_id[746].value <= threshold=2.3414241075515747

node_1226: feature_name=GO:0002903 feature_id[10].value <= threshold=2.715258240699768
node_1227: feature_name=GO:0043375 feature_id[717].value <= threshold=1.9525017738342285
node_1228: feature_name=GO:0032762 feature_id[630].value <= threshold=0.29527929425239563
node_1229: feature_name=GO:0002562 feature_id[25].value <= threshold=1.3898683190345764
Class: positive genes

Rules_506 passed counts:1
node_0: feature_name=GO:0042113 feature_id[0].value <= threshold=13.408552169799805
node_1: feature_name=GO:0007568 feature_id[534].value > threshold=5.0313897132873535
node_913: feature_name=GO:0032763 feature_id[629].value > threshold=0.31753237545490265
node_1195: feature_name=GO:0071301 feature_id[442].value <= threshold=0.11534593254327774
node_1196: feature_name=GO:0046500 feature_id[171].value <= threshold=0.6314916908740997
node_1197: feature_name=GO:0030852 feature_id[22].value <= threshold=2.9768868684768677
node_1198: feature_name=hsa04672 feature_id[276].value <= threshold=8.06681227684021
node_1199: feature_name=GO:0001836 feature_id[450].value > threshold=0.09961023926734924
node_1201: feature_name=GO:0070230 feature_id[64].value <= threshold=3.3956379890441895
node_1202: feature_name=GO:0070102 feature_id[49].value <= threshold=4.805386543273926
node_1203: feature_name=GO:0045553 feature_id[736].value <= threshold=1.4970332980155945
node_1204: feature_name=GO:0051311 feature_id[58].value <= threshold=3.301889181137085
node_1205: feature_name=GO:0030291 feature_id[147].value <= threshold=8.156315326690674
node_1206: feature_name=GO:0045637 feature_id[241].value > threshold=0.01834342861548066
node_1208: feature_name=GO:0002890 feature_id[98].value <= threshold=2.767483353614807
node_1209: feature_name=GO:0046632 feature_id[248].value <= threshold=11.663254261016846
node_1210: feature_name=GO:0008588 feature_id[26].value <= threshold=1.391375720500946
node_1211: feature_name=GO:0010639 feature_id[564].value <= threshold=18.975126266479492
node_1212: feature_name=GO:0046685 feature_id[782].value <= threshold=5.153738260269165
node_1213: feature_name=GO:0038116 feature_id[668].value <= threshold=1.7859655618667603
node_1214: feature_name=GO:0046449 feature_id[763].value <= threshold=5.603115558624268
node_1215: feature_name=GO:0048539 feature_id[319].value <= threshold=0.5478232800960541
node_1216: feature_name=GO:0007089 feature_id[522].value <= threshold=0.9940084517002106
node_1217: feature_name=GO:0042287 feature_id[20].value <= threshold=2.693045496940613
node_1218: feature_name=GO:0044446 feature_id[184].value <= threshold=113.0758171081543
node_1219: feature_name=GO:0098602 feature_id[702].value <= threshold=68.95328903198242
node_1220: feature_name=GO:0035726 feature_id[270].value <= threshold=1.7167096138000488
node_1221: feature_name=GO:1902564 feature_id[85].value <= threshold=2.8364468812942505

node_1222: feature_name=GO:0006979 feature_id[44].value <= threshold=0.6869173645973206
Class: positive genes

Rules_507 passed counts:1
node_0: feature_name=GO:0042113 feature_id[0].value <= threshold=13.408552169799805
node_1: feature_name=GO:0007568 feature_id[534].value > threshold=5.0313897132873535
node_913: feature_name=GO:0032763 feature_id[629].value <= threshold=0.31753237545490265
node_914: feature_name=GO:0097193 feature_id[167].value > threshold=28.171168327331543
node_1188: feature_name=GO:0002704 feature_id[470].value > threshold=0.8429980874061584
node_1192: feature_name=GO:0019660 feature_id[70].value <= threshold=0.9662298560142517
Class: positive genes

Rules_508 passed counts:1
node_0: feature_name=GO:0042113 feature_id[0].value <= threshold=13.408552169799805
node_1: feature_name=GO:0007568 feature_id[534].value > threshold=5.0313897132873535
node_913: feature_name=GO:0032763 feature_id[629].value <= threshold=0.31753237545490265
node_914: feature_name=GO:0097193 feature_id[167].value <= threshold=28.171168327331543
node_915: feature_name=GO:0002903 feature_id[10].value > threshold=1.0534588098526
node_1159: feature_name=GO:0070198 feature_id[665].value > threshold=0.16219981759786606
node_1165: feature_name=GO:0042287 feature_id[20].value > threshold=0.915093183517456
node_1181: feature_name=GO:0060576 feature_id[204].value <= threshold=0.3862239122390747
node_1182: feature_name=GO:0042509 feature_id[694].value > threshold=5.3463897705078125
node_1184: feature_name=GO:0044424 feature_id[1].value <= threshold=0.29986531287431717
Class: negative genes

Rules_509 passed counts:1
node_0: feature_name=GO:0042113 feature_id[0].value <= threshold=13.408552169799805
node_1: feature_name=GO:0007568 feature_id[534].value > threshold=5.0313897132873535
node_913: feature_name=GO:0032763 feature_id[629].value <= threshold=0.31753237545490265
node_914: feature_name=GO:0097193 feature_id[167].value <= threshold=28.171168327331543
node_915: feature_name=GO:0002903 feature_id[10].value > threshold=1.0534588098526
node_1159: feature_name=GO:0070198 feature_id[665].value > threshold=0.16219981759786606
node_1165: feature_name=GO:0042287 feature_id[20].value <= threshold=0.915093183517456
node_1166: feature_name=GO:0046006 feature_id[364].value > threshold=0.12353959679603577
node_1168: feature_name=GO:0046634 feature_id[46].value > threshold=4.166545033454895

node_1178: feature_name=GO:0002517 feature_id[88].value > threshold=1.9423661828041077
Class: negative genes

Rules_510 passed counts:1
node_0: feature_name=GO:0042113 feature_id[0].value <= threshold=13.408552169799805
node_1: feature_name=GO:0007568 feature_id[534].value > threshold=5.0313897132873535
node_913: feature_name=GO:0032763 feature_id[629].value <= threshold=0.31753237545490265
node_914: feature_name=GO:0097193 feature_id[167].value <= threshold=28.171168327331543
node_915: feature_name=GO:0002903 feature_id[10].value > threshold=1.0534588098526
node_1159: feature_name=GO:0070198 feature_id[665].value > threshold=0.16219981759786606
node_1165: feature_name=GO:0042287 feature_id[20].value <= threshold=0.915093183517456
node_1166: feature_name=GO:0046006 feature_id[364].value > threshold=0.12353959679603577
node_1168: feature_name=GO:0046634 feature_id[46].value <= threshold=4.166545033454895
node_1169: feature_name=GO:0044346 feature_id[724].value > threshold=0.9571778774261475
node_1173: feature_name=GO:0060374 feature_id[76].value > threshold=2.239573121070862
node_1175: feature_name=GO:0071887 feature_id[283].value <= threshold=1.6318552792072296
Class: negative genes

Rules_511 passed counts:1
node_0: feature_name=GO:0042113 feature_id[0].value <= threshold=13.408552169799805
node_1: feature_name=GO:0007568 feature_id[534].value > threshold=5.0313897132873535
node_913: feature_name=GO:0032763 feature_id[629].value <= threshold=0.31753237545490265
node_914: feature_name=GO:0097193 feature_id[167].value <= threshold=28.171168327331543
node_915: feature_name=GO:0002903 feature_id[10].value <= threshold=1.0534588098526
node_916: feature_name=GO:1904029 feature_id[208].value > threshold=23.852136611938477
node_1156: feature_name=hsa05203 feature_id[578].value > threshold=32.333744049072266
Class: negative genes

Rules_512 passed counts:1
node_0: feature_name=GO:0042113 feature_id[0].value <= threshold=13.408552169799805
node_1: feature_name=GO:0007568 feature_id[534].value > threshold=5.0313897132873535
node_913: feature_name=GO:0032763 feature_id[629].value <= threshold=0.31753237545490265
node_914: feature_name=GO:0097193 feature_id[167].value <= threshold=28.171168327331543
node_915: feature_name=GO:0002903 feature_id[10].value <= threshold=1.0534588098526
node_916: feature_name=GO:1904029 feature_id[208].value <= threshold=23.852136611938477

node_917: feature_name=GO:0050897 feature_id[164].value > threshold=0.6870408356189728
node_1095: feature_name=GO:0072539 feature_id[68].value <= threshold=2.5667877197265625
node_1096: feature_name=GO:0046500 feature_id[171].value <= threshold=3.195197820663452
node_1097: feature_name=GO:0045861 feature_id[761].value > threshold=0.22614753991365433
node_1099: feature_name=GO:0043372 feature_id[242].value <= threshold=4.892506122589111
node_1100: feature_name=GO:0042162 feature_id[355].value <= threshold=11.339290618896484
node_1101: feature_name=hsa04660 feature_id[472].value > threshold=24.27848720550537
node_1143: feature_name=GO:0019814 feature_id[189].value > threshold=0.5058850646018982
node_1145: feature_name=GO:0032204 feature_id[747].value > threshold=3.3982995748519897
Class: positive genes

Rules_513 passed counts:1
node_0: feature_name=GO:0042113 feature_id[0].value <= threshold=13.408552169799805
node_1: feature_name=GO:0007568 feature_id[534].value > threshold=5.0313897132873535
node_913: feature_name=GO:0032763 feature_id[629].value <= threshold=0.31753237545490265
node_914: feature_name=GO:0097193 feature_id[167].value <= threshold=28.171168327331543
node_915: feature_name=GO:0002903 feature_id[10].value <= threshold=1.0534588098526
node_916: feature_name=GO:1904029 feature_id[208].value <= threshold=23.852136611938477
node_917: feature_name=GO:0050897 feature_id[164].value > threshold=0.6870408356189728
node_1095: feature_name=GO:0072539 feature_id[68].value <= threshold=2.5667877197265625
node_1096: feature_name=GO:0046500 feature_id[171].value <= threshold=3.195197820663452
node_1097: feature_name=GO:0045861 feature_id[761].value > threshold=0.22614753991365433
node_1099: feature_name=GO:0043372 feature_id[242].value <= threshold=4.892506122589111
node_1100: feature_name=GO:0042162 feature_id[355].value <= threshold=11.339290618896484
node_1101: feature_name=hsa04660 feature_id[472].value <= threshold=24.27848720550537
node_1102: feature_name=GO:0007049 feature_id[259].value <= threshold=209.23831176757812
node_1103: feature_name=GO:0050897 feature_id[164].value > threshold=0.6919849812984467
node_1105: feature_name=GO:0045945 feature_id[267].value > threshold=3.307852625846863
node_1137: feature_name=GO:0072710 feature_id[251].value > threshold=0.4050363004207611
node_1139: feature_name=GO:0001666 feature_id[682].value <= threshold=8.14579439163208
Class: negative genes

Rules_514 passed counts:1
node_0: feature_name=GO:0042113 feature_id[0].value <= threshold=13.408552169799805
node_1: feature_name=GO:0007568 feature_id[534].value > threshold=5.0313897132873535

node_913: feature_name=GO:0032763	feature_id[629].value <= threshold=0.31753237545490265
node_914: feature_name=GO:0097193	feature_id[167].value <= threshold=28.171168327331543
node_915: feature_name=GO:0002903	feature_id[10].value <= threshold=1.0534588098526
node_916: feature_name=GO:1904029	feature_id[208].value <= threshold=23.852136611938477
node_917: feature_name=GO:0050897	feature_id[164].value > threshold=0.6870408356189728
node_1095: feature_name=GO:0072539	feature_id[68].value <= threshold=2.5667877197265625
node_1096: feature_name=GO:0046500	feature_id[171].value <= threshold=3.195197820663452
node_1097: feature_name=GO:0045861	feature_id[761].value > threshold=0.22614753991365433
node_1099: feature_name=GO:0043372	feature_id[242].value <= threshold=4.892506122589111
node_1100: feature_name=GO:0042162	feature_id[355].value <= threshold=11.339290618896484
node_1101: feature_name=hsa04660	feature_id[472].value <= threshold=24.27848720550537
node_1102: feature_name=GO:0007049	feature_id[259].value <= threshold=209.23831176757812
node_1103: feature_name=GO:0050897	feature_id[164].value > threshold=0.6919849812984467
node_1105: feature_name=GO:0045945	feature_id[267].value <= threshold=3.307852625846863
node_1106: feature_name=GO:0046641	feature_id[773].value > threshold=3.527579426765442
node_1134: feature_name=GO:0045736	feature_id[61].value > threshold=0.6813804358243942

Class: negative genes

Rules_515	passed counts:1
node_0: feature_name=GO:0042113	feature_id[0].value <= threshold=13.408552169799805
node_1: feature_name=GO:0007568	feature_id[534].value > threshold=5.0313897132873535
node_913: feature_name=GO:0032763	feature_id[629].value <= threshold=0.31753237545490265
node_914: feature_name=GO:0097193	feature_id[167].value <= threshold=28.171168327331543
node_915: feature_name=GO:0002903	feature_id[10].value <= threshold=1.0534588098526
node_916: feature_name=GO:1904029	feature_id[208].value <= threshold=23.852136611938477
node_917: feature_name=GO:0050897	feature_id[164].value > threshold=0.6870408356189728
node_1095: feature_name=GO:0072539	feature_id[68].value <= threshold=2.5667877197265625
node_1096: feature_name=GO:0046500	feature_id[171].value <= threshold=3.195197820663452
node_1097: feature_name=GO:0045861	feature_id[761].value > threshold=0.22614753991365433
node_1099: feature_name=GO:0043372	feature_id[242].value <= threshold=4.892506122589111
node_1100: feature_name=GO:0042162	feature_id[355].value <= threshold=11.339290618896484
node_1101: feature_name=hsa04660	feature_id[472].value <= threshold=24.27848720550537
node_1102: feature_name=GO:0007049	feature_id[259].value <= threshold=209.23831176757812
node_1103: feature_name=GO:0050897	feature_id[164].value > threshold=0.6919849812984467
node_1105: feature_name=GO:0045945	feature_id[267].value <= threshold=3.307852625846863

node_1106: feature_name=GO:0046641 feature_id[773].value <= threshold=3.527579426765442
node_1107: feature_name=GO:0002262 feature_id[394].value > threshold=0.08302562311291695
node_1111: feature_name=GO:0043226 feature_id[138].value > threshold=2.4930146992119262e-05
node_1113: feature_name=GO:0045787 feature_id[446].value > threshold=0.07611752673983574
node_1115: feature_name=GO:0045840 feature_id[697].value > threshold=0.10580676794052124
node_1117: feature_name=GO:0006927 feature_id[225].value > threshold=4.950021743774414
Class: positive genes

Rules_516 passed counts:1
node_0: feature_name=GO:0042113 feature_id[0].value <= threshold=13.408552169799805
node_1: feature_name=GO:0007568 feature_id[534].value > threshold=5.0313897132873535
node_913: feature_name=GO:0032763 feature_id[629].value <= threshold=0.31753237545490265
node_914: feature_name=GO:0097193 feature_id[167].value <= threshold=28.171168327331543
node_915: feature_name=GO:0002903 feature_id[10].value <= threshold=1.0534588098526
node_916: feature_name=GO:1904029 feature_id[208].value <= threshold=23.852136611938477
node_917: feature_name=GO:0050897 feature_id[164].value > threshold=0.6870408356189728
node_1095: feature_name=GO:0072539 feature_id[68].value <= threshold=2.5667877197265625
node_1096: feature_name=GO:0046500 feature_id[171].value <= threshold=3.195197820663452
node_1097: feature_name=GO:0045861 feature_id[761].value > threshold=0.22614753991365433
node_1099: feature_name=GO:0043372 feature_id[242].value <= threshold=4.892506122589111
node_1100: feature_name=GO:0042162 feature_id[355].value <= threshold=11.339290618896484
node_1101: feature_name=hsa04660 feature_id[472].value <= threshold=24.27848720550537
node_1102: feature_name=GO:0007049 feature_id[259].value <= threshold=209.23831176757812
node_1103: feature_name=GO:0050897 feature_id[164].value > threshold=0.6919849812984467
node_1105: feature_name=GO:0045945 feature_id[267].value <= threshold=3.307852625846863
node_1106: feature_name=GO:0046641 feature_id[773].value <= threshold=3.527579426765442
node_1107: feature_name=GO:0002262 feature_id[394].value > threshold=0.08302562311291695
node_1111: feature_name=GO:0043226 feature_id[138].value > threshold=2.4930146992119262e-05
node_1113: feature_name=GO:0045787 feature_id[446].value > threshold=0.07611752673983574
node_1115: feature_name=GO:0045840 feature_id[697].value > threshold=0.10580676794052124
node_1117: feature_name=GO:0006927 feature_id[225].value <= threshold=4.950021743774414
node_1118: feature_name=GO:0002643 feature_id[4].value > threshold=3.364396333694458
Class: positive genes

Rules_517 passed counts:1

node_0: feature_name=GO:0042113 feature_id[0].value <= threshold=13.408552169799805
node_1: feature_name=GO:0007568 feature_id[534].value > threshold=5.0313897132873535
node_913: feature_name=GO:0032763 feature_id[629].value <= threshold=0.31753237545490265
node_914: feature_name=GO:0097193 feature_id[167].value <= threshold=28.171168327331543
node_915: feature_name=GO:0002903 feature_id[10].value <= threshold=1.0534588098526
node_916: feature_name=GO:1904029 feature_id[208].value <= threshold=23.852136611938477
node_917: feature_name=GO:0050897 feature_id[164].value > threshold=0.6870408356189728
node_1095: feature_name=GO:0072539 feature_id[68].value <= threshold=2.5667877197265625
node_1096: feature_name=GO:0046500 feature_id[171].value <= threshold=3.195197820663452
node_1097: feature_name=GO:0045861 feature_id[761].value > threshold=0.22614753991365433
node_1099: feature_name=GO:0043372 feature_id[242].value <= threshold=4.892506122589111
node_1100: feature_name=GO:0042162 feature_id[355].value <= threshold=11.339290618896484
node_1101: feature_name=hsa04660 feature_id[472].value <= threshold=24.27848720550537
node_1102: feature_name=GO:0007049 feature_id[259].value <= threshold=209.23831176757812
node_1103: feature_name=GO:0050897 feature_id[164].value > threshold=0.6919849812984467
node_1105: feature_name=GO:0045945 feature_id[267].value <= threshold=3.307852625846863
node_1106: feature_name=GO:0046641 feature_id[773].value <= threshold=3.527579426765442
node_1107: feature_name=GO:0002262 feature_id[394].value > threshold=0.08302562311291695
node_1111: feature_name=GO:0043226 feature_id[138].value > threshold=2.4930146992119262e-05
node_1113: feature_name=GO:0045787 feature_id[446].value > threshold=0.07611752673983574
node_1115: feature_name=GO:0045840 feature_id[697].value > threshold=0.10580676794052124
node_1117: feature_name=GO:0006927 feature_id[225].value <= threshold=4.950021743774414
node_1118: feature_name=GO:0002643 feature_id[4].value <= threshold=3.364396333694458
node_1119: feature_name=GO:0016571 feature_id[725].value > threshold=38.268903732299805
Class: positive genes

Rules_518 passed counts:1
node_0: feature_name=GO:0042113 feature_id[0].value <= threshold=13.408552169799805
node_1: feature_name=GO:0007568 feature_id[534].value > threshold=5.0313897132873535
node_913: feature_name=GO:0032763 feature_id[629].value <= threshold=0.31753237545490265
node_914: feature_name=GO:0097193 feature_id[167].value <= threshold=28.171168327331543
node_915: feature_name=GO:0002903 feature_id[10].value <= threshold=1.0534588098526
node_916: feature_name=GO:1904029 feature_id[208].value <= threshold=23.852136611938477
node_917: feature_name=GO:0050897 feature_id[164].value > threshold=0.6870408356189728
node_1095: feature_name=GO:0072539 feature_id[68].value <= threshold=2.5667877197265625

node_1096: feature_name=GO:0046500 feature_id[171].value <= threshold=3.195197820663452
node_1097: feature_name=GO:0045861 feature_id[761].value > threshold=0.22614753991365433
node_1099: feature_name=GO:0043372 feature_id[242].value <= threshold=4.892506122589111
node_1100: feature_name=GO:0042162 feature_id[355].value <= threshold=11.339290618896484
node_1101: feature_name=hsa04660 feature_id[472].value <= threshold=24.27848720550537
node_1102: feature_name=GO:0007049 feature_id[259].value <= threshold=209.23831176757812
node_1103: feature_name=GO:0050897 feature_id[164].value > threshold=0.6919849812984467
node_1105: feature_name=GO:0045945 feature_id[267].value <= threshold=3.307852625846863
node_1106: feature_name=GO:0046641 feature_id[773].value <= threshold=3.527579426765442
node_1107: feature_name=GO:0002262 feature_id[394].value > threshold=0.08302562311291695
node_1111: feature_name=GO:0043226 feature_id[138].value > threshold=2.4930146992119262e-05
node_1113: feature_name=GO:0045787 feature_id[446].value > threshold=0.07611752673983574
node_1115: feature_name=GO:0045840 feature_id[697].value > threshold=0.10580676794052124
node_1117: feature_name=GO:0006927 feature_id[225].value <= threshold=4.950021743774414
node_1118: feature_name=GO:0002643 feature_id[4].value <= threshold=3.364396333694458
node_1119: feature_name=GO:0016571 feature_id[725].value <= threshold=38.268903732299805
node_1120: feature_name=GO:0072593 feature_id[381].value <= threshold=19.997477531433105
node_1121: feature_name=GO:0070245 feature_id[326].value > threshold=3.2926437854766846
Class: positive genes

Rules_519 passed counts:1
node_0: feature_name=GO:0042113 feature_id[0].value <= threshold=13.408552169799805
node_1: feature_name=GO:0007568 feature_id[534].value > threshold=5.0313897132873535
node_913: feature_name=GO:0032763 feature_id[629].value <= threshold=0.31753237545490265
node_914: feature_name=GO:0097193 feature_id[167].value <= threshold=28.171168327331543
node_915: feature_name=GO:0002903 feature_id[10].value <= threshold=1.0534588098526
node_916: feature_name=GO:1904029 feature_id[208].value <= threshold=23.852136611938477
node_917: feature_name=GO:0050897 feature_id[164].value > threshold=0.6870408356189728
node_1095: feature_name=GO:0072539 feature_id[68].value <= threshold=2.5667877197265625
node_1096: feature_name=GO:0046500 feature_id[171].value <= threshold=3.195197820663452
node_1097: feature_name=GO:0045861 feature_id[761].value > threshold=0.22614753991365433
node_1099: feature_name=GO:0043372 feature_id[242].value <= threshold=4.892506122589111
node_1100: feature_name=GO:0042162 feature_id[355].value <= threshold=11.339290618896484
node_1101: feature_name=hsa04660 feature_id[472].value <= threshold=24.27848720550537
node_1102: feature_name=GO:0007049 feature_id[259].value <= threshold=209.23831176757812

node_1103: feature_name=GO:0050897 feature_id[164].value > threshold=0.6919849812984467
node_1105: feature_name=GO:0045945 feature_id[267].value <= threshold=3.307852625846863
node_1106: feature_name=GO:0046641 feature_id[773].value <= threshold=3.527579426765442
node_1107: feature_name=GO:0002262 feature_id[394].value > threshold=0.08302562311291695
node_1111: feature_name=GO:0043226 feature_id[138].value > threshold=2.4930146992119262e-05
node_1113: feature_name=GO:0045787 feature_id[446].value > threshold=0.07611752673983574
node_1115: feature_name=GO:0045840 feature_id[697].value <= threshold=0.10580676794052124
Class: positive genes

Rules_520 passed counts:1
node_0: feature_name=GO:0042113 feature_id[0].value <= threshold=13.408552169799805
node_1: feature_name=GO:0007568 feature_id[534].value > threshold=5.0313897132873535
node_913: feature_name=GO:0032763 feature_id[629].value <= threshold=0.31753237545490265
node_914: feature_name=GO:0097193 feature_id[167].value <= threshold=28.171168327331543
node_915: feature_name=GO:0002903 feature_id[10].value <= threshold=1.0534588098526
node_916: feature_name=GO:1904029 feature_id[208].value <= threshold=23.852136611938477
node_917: feature_name=GO:0050897 feature_id[164].value > threshold=0.6870408356189728
node_1095: feature_name=GO:0072539 feature_id[68].value <= threshold=2.5667877197265625
node_1096: feature_name=GO:0046500 feature_id[171].value <= threshold=3.195197820663452
node_1097: feature_name=GO:0045861 feature_id[761].value > threshold=0.22614753991365433
node_1099: feature_name=GO:0043372 feature_id[242].value <= threshold=4.892506122589111
node_1100: feature_name=GO:0042162 feature_id[355].value <= threshold=11.339290618896484
node_1101: feature_name=hsa04660 feature_id[472].value <= threshold=24.27848720550537
node_1102: feature_name=GO:0007049 feature_id[259].value <= threshold=209.23831176757812
node_1103: feature_name=GO:0050897 feature_id[164].value > threshold=0.6919849812984467
node_1105: feature_name=GO:0045945 feature_id[267].value <= threshold=3.307852625846863
node_1106: feature_name=GO:0046641 feature_id[773].value <= threshold=3.527579426765442
node_1107: feature_name=GO:0002262 feature_id[394].value > threshold=0.08302562311291695
node_1111: feature_name=GO:0043226 feature_id[138].value > threshold=2.4930146992119262e-05
node_1113: feature_name=GO:0045787 feature_id[446].value <= threshold=0.07611752673983574
Class: positive genes

Rules_521 passed counts:1
node_0: feature_name=GO:0042113 feature_id[0].value <= threshold=13.408552169799805
node_1: feature_name=GO:0007568 feature_id[534].value > threshold=5.0313897132873535

node_913: feature_name=GO:0032763 feature_id[629].value <= threshold=0.31753237545490265
node_914: feature_name=GO:0097193 feature_id[167].value <= threshold=28.171168327331543
node_915: feature_name=GO:0002903 feature_id[10].value <= threshold=1.0534588098526
node_916: feature_name=GO:1904029 feature_id[208].value <= threshold=23.852136611938477
node_917: feature_name=GO:0050897 feature_id[164].value > threshold=0.6870408356189728
node_1095: feature_name=GO:0072539 feature_id[68].value <= threshold=2.5667877197265625
node_1096: feature_name=GO:0046500 feature_id[171].value <= threshold=3.195197820663452
node_1097: feature_name=GO:0045861 feature_id[761].value > threshold=0.22614753991365433
node_1099: feature_name=GO:0043372 feature_id[242].value <= threshold=4.892506122589111
node_1100: feature_name=GO:0042162 feature_id[355].value <= threshold=11.339290618896484
node_1101: feature_name=hsa04660 feature_id[472].value <= threshold=24.27848720550537
node_1102: feature_name=GO:0007049 feature_id[259].value <= threshold=209.23831176757812
node_1103: feature_name=GO:0050897 feature_id[164].value > threshold=0.6919849812984467
node_1105: feature_name=GO:0045945 feature_id[267].value <= threshold=3.307852625846863
node_1106: feature_name=GO:0046641 feature_id[773].value <= threshold=3.527579426765442
node_1107: feature_name=GO:0002262 feature_id[394].value > threshold=0.08302562311291695
node_1111: feature_name=GO:0043226 feature_id[138].value <= threshold=2.4930146992119262e-05
Class: positive genes

Rules_522 passed counts:1
node_0: feature_name=GO:0042113 feature_id[0].value <= threshold=13.408552169799805
node_1: feature_name=GO:0007568 feature_id[534].value > threshold=5.0313897132873535
node_913: feature_name=GO:0032763 feature_id[629].value <= threshold=0.31753237545490265
node_914: feature_name=GO:0097193 feature_id[167].value <= threshold=28.171168327331543
node_915: feature_name=GO:0002903 feature_id[10].value <= threshold=1.0534588098526
node_916: feature_name=GO:1904029 feature_id[208].value <= threshold=23.852136611938477
node_917: feature_name=GO:0050897 feature_id[164].value > threshold=0.6870408356189728
node_1095: feature_name=GO:0072539 feature_id[68].value <= threshold=2.5667877197265625
node_1096: feature_name=GO:0046500 feature_id[171].value <= threshold=3.195197820663452
node_1097: feature_name=GO:0045861 feature_id[761].value > threshold=0.22614753991365433
node_1099: feature_name=GO:0043372 feature_id[242].value <= threshold=4.892506122589111
node_1100: feature_name=GO:0042162 feature_id[355].value <= threshold=11.339290618896484
node_1101: feature_name=hsa04660 feature_id[472].value <= threshold=24.27848720550537
node_1102: feature_name=GO:0007049 feature_id[259].value <= threshold=209.23831176757812
node_1103: feature_name=GO:0050897 feature_id[164].value > threshold=0.6919849812984467

node_1105: feature_name=GO:0045945 feature_id[267].value <= threshold=3.307852625846863
node_1106: feature_name=GO:0046641 feature_id[773].value <= threshold=3.527579426765442
node_1107: feature_name=GO:0002262 feature_id[394].value <= threshold=0.08302562311291695
node_1108: feature_name=GO:0097694 feature_id[176].value <= threshold=0.491131991147995
Class: negative genes

Rules_523 passed counts:1
node_0: feature_name=GO:0042113 feature_id[0].value <= threshold=13.408552169799805
node_1: feature_name=GO:0007568 feature_id[534].value > threshold=5.0313897132873535
node_913: feature_name=GO:0032763 feature_id[629].value <= threshold=0.31753237545490265
node_914: feature_name=GO:0097193 feature_id[167].value <= threshold=28.171168327331543
node_915: feature_name=GO:0002903 feature_id[10].value <= threshold=1.0534588098526
node_916: feature_name=GO:1904029 feature_id[208].value <= threshold=23.852136611938477
node_917: feature_name=GO:0050897 feature_id[164].value <= threshold=0.6870408356189728
node_918: feature_name=GO:0006139 feature_id[474].value > threshold=1.3052097624921544e-07
node_920: feature_name=GO:0002821 feature_id[588].value <= threshold=13.81072187423706
node_921: feature_name=GO:0006298 feature_id[621].value <= threshold=24.078600883483887
node_922: feature_name=GO:0003908 feature_id[11].value <= threshold=1.7776933312416077
node_923: feature_name=GO:0030098 feature_id[273].value > threshold=0.0079949083738029
node_927: feature_name=GO:0006808 feature_id[513].value > threshold=4.34592080116272
node_1087: feature_name=GO:0001777 feature_id[380].value > threshold=0.9556370079517365
Class: negative genes

Rules_524 passed counts:1
node_0: feature_name=GO:0042113 feature_id[0].value <= threshold=13.408552169799805
node_1: feature_name=GO:0007568 feature_id[534].value > threshold=5.0313897132873535
node_913: feature_name=GO:0032763 feature_id[629].value <= threshold=0.31753237545490265
node_914: feature_name=GO:0097193 feature_id[167].value <= threshold=28.171168327331543
node_915: feature_name=GO:0002903 feature_id[10].value <= threshold=1.0534588098526
node_916: feature_name=GO:1904029 feature_id[208].value <= threshold=23.852136611938477
node_917: feature_name=GO:0050897 feature_id[164].value <= threshold=0.6870408356189728
node_918: feature_name=GO:0006139 feature_id[474].value > threshold=1.3052097624921544e-07
node_920: feature_name=GO:0002821 feature_id[588].value <= threshold=13.81072187423706
node_921: feature_name=GO:0006298 feature_id[621].value <= threshold=24.078600883483887
node_922: feature_name=GO:0003908 feature_id[11].value <= threshold=1.7776933312416077

node_923: feature_name=GO:0030098 feature_id[273].value > threshold=0.0079949083738029
node_927: feature_name=GO:0006808 feature_id[513].value <= threshold=4.34592080116272
node_928: feature_name=GO:0071887 feature_id[283].value > threshold=10.353787899017334
node_1084: feature_name=GO:0043011 feature_id[150].value <= threshold=1.5169694423675537
Class: negative genes

Rules_525 passed counts:1
node_0: feature_name=GO:0042113 feature_id[0].value <= threshold=13.408552169799805
node_1: feature_name=GO:0007568 feature_id[534].value > threshold=5.0313897132873535
node_913: feature_name=GO:0032763 feature_id[629].value <= threshold=0.31753237545490265
node_914: feature_name=GO:0097193 feature_id[167].value <= threshold=28.171168327331543
node_915: feature_name=GO:0002903 feature_id[10].value <= threshold=1.0534588098526
node_916: feature_name=GO:1904029 feature_id[208].value <= threshold=23.852136611938477
node_917: feature_name=GO:0050897 feature_id[164].value <= threshold=0.6870408356189728
node_918: feature_name=GO:0006139 feature_id[474].value > threshold=1.3052097624921544e-07
node_920: feature_name=GO:0002821 feature_id[588].value <= threshold=13.81072187423706
node_921: feature_name=GO:0006298 feature_id[621].value <= threshold=24.078600883483887
node_922: feature_name=GO:0003908 feature_id[11].value <= threshold=1.7776933312416077
node_923: feature_name=GO:0030098 feature_id[273].value > threshold=0.0079949083738029
node_927: feature_name=GO:0006808 feature_id[513].value <= threshold=4.34592080116272
node_928: feature_name=GO:0071887 feature_id[283].value <= threshold=10.353787899017334
node_929: feature_name=GO:0038001 feature_id[28].value > threshold=3.9469382762908936
node_1081: feature_name=GO:0043465 feature_id[710].value <= threshold=1.0033313930034637
Class: negative genes

Rules_526 passed counts:1
node_0: feature_name=GO:0042113 feature_id[0].value <= threshold=13.408552169799805
node_1: feature_name=GO:0007568 feature_id[534].value > threshold=5.0313897132873535
node_913: feature_name=GO:0032763 feature_id[629].value <= threshold=0.31753237545490265
node_914: feature_name=GO:0097193 feature_id[167].value <= threshold=28.171168327331543
node_915: feature_name=GO:0002903 feature_id[10].value <= threshold=1.0534588098526
node_916: feature_name=GO:1904029 feature_id[208].value <= threshold=23.852136611938477
node_917: feature_name=GO:0050897 feature_id[164].value <= threshold=0.6870408356189728
node_918: feature_name=GO:0006139 feature_id[474].value > threshold=1.3052097624921544e-07
node_920: feature_name=GO:0002821 feature_id[588].value <= threshold=13.81072187423706

node_921: feature_name=GO:0006298 feature_id[621].value <= threshold=24.078600883483887
node_922: feature_name=GO:0003908 feature_id[11].value <= threshold=1.7776933312416077
node_923: feature_name=GO:0030098 feature_id[273].value > threshold=0.0079949083738029
node_927: feature_name=GO:0006808 feature_id[513].value <= threshold=4.34592080116272
node_928: feature_name=GO:0071887 feature_id[283].value <= threshold=10.353787899017334
node_929: feature_name=GO:0038001 feature_id[28].value <= threshold=3.9469382762908936
node_930: feature_name=GO:0042287 feature_id[20].value > threshold=3.6209195852279663
node_1078: feature_name=hsa05202 feature_id[50].value <= threshold=0.7917962670326233
Class: negative genes

Rules_527 passed counts:1
node_0: feature_name=GO:0042113 feature_id[0].value <= threshold=13.408552169799805
node_1: feature_name=GO:0007568 feature_id[534].value > threshold=5.0313897132873535
node_913: feature_name=GO:0032763 feature_id[629].value <= threshold=0.31753237545490265
node_914: feature_name=GO:0097193 feature_id[167].value <= threshold=28.171168327331543
node_915: feature_name=GO:0002903 feature_id[10].value <= threshold=1.0534588098526
node_916: feature_name=GO:1904029 feature_id[208].value <= threshold=23.852136611938477
node_917: feature_name=GO:0050897 feature_id[164].value <= threshold=0.6870408356189728
node_918: feature_name=GO:0006139 feature_id[474].value > threshold=1.3052097624921544e-07
node_920: feature_name=GO:0002821 feature_id[588].value <= threshold=13.81072187423706
node_921: feature_name=GO:0006298 feature_id[621].value <= threshold=24.078600883483887
node_922: feature_name=GO:0003908 feature_id[11].value <= threshold=1.7776933312416077
node_923: feature_name=GO:0030098 feature_id[273].value > threshold=0.0079949083738029
node_927: feature_name=GO:0006808 feature_id[513].value <= threshold=4.34592080116272
node_928: feature_name=GO:0071887 feature_id[283].value <= threshold=10.353787899017334
node_929: feature_name=GO:0038001 feature_id[28].value <= threshold=3.9469382762908936
node_930: feature_name=GO:0042287 feature_id[20].value <= threshold=3.6209195852279663
node_931: feature_name=GO:0003968 feature_id[47].value > threshold=2.004227638244629
Class: positive genes

Rules_528 passed counts:1
node_0: feature_name=GO:0042113 feature_id[0].value <= threshold=13.408552169799805
node_1: feature_name=GO:0007568 feature_id[534].value > threshold=5.0313897132873535
node_913: feature_name=GO:0032763 feature_id[629].value <= threshold=0.31753237545490265
node_914: feature_name=GO:0097193 feature_id[167].value <= threshold=28.171168327331543

node_915: feature_name=GO:0002903 feature_id[10].value <= threshold=1.0534588098526
node_916: feature_name=GO:1904029 feature_id[208].value <= threshold=23.852136611938477
node_917: feature_name=GO:0050897 feature_id[164].value <= threshold=0.6870408356189728
node_918: feature_name=GO:0006139 feature_id[474].value > threshold=1.3052097624921544e-07
node_920: feature_name=GO:0002821 feature_id[588].value <= threshold=13.81072187423706
node_921: feature_name=GO:0006298 feature_id[621].value <= threshold=24.078600883483887
node_922: feature_name=GO:0003908 feature_id[11].value <= threshold=1.7776933312416077
node_923: feature_name=GO:0030098 feature_id[273].value > threshold=0.0079949083738029
node_927: feature_name=GO:0006808 feature_id[513].value <= threshold=4.34592080116272
node_928: feature_name=GO:0071887 feature_id[283].value <= threshold=10.353787899017334
node_929: feature_name=GO:0038001 feature_id[28].value <= threshold=3.9469382762908936
node_930: feature_name=GO:0042287 feature_id[20].value <= threshold=3.6209195852279663
node_931: feature_name=GO:0003968 feature_id[47].value <= threshold=2.004227638244629
node_932: feature_name=GO:0002698 feature_id[395].value > threshold=18.51447582244873
Class: positive genes

Rules_529 passed counts:1
node_0: feature_name=GO:0042113 feature_id[0].value <= threshold=13.408552169799805
node_1: feature_name=GO:0007568 feature_id[534].value > threshold=5.0313897132873535
node_913: feature_name=GO:0032763 feature_id[629].value <= threshold=0.31753237545490265
node_914: feature_name=GO:0097193 feature_id[167].value <= threshold=28.171168327331543
node_915: feature_name=GO:0002903 feature_id[10].value <= threshold=1.0534588098526
node_916: feature_name=GO:1904029 feature_id[208].value <= threshold=23.852136611938477
node_917: feature_name=GO:0050897 feature_id[164].value <= threshold=0.6870408356189728
node_918: feature_name=GO:0006139 feature_id[474].value > threshold=1.3052097624921544e-07
node_920: feature_name=GO:0002821 feature_id[588].value <= threshold=13.81072187423706
node_921: feature_name=GO:0006298 feature_id[621].value <= threshold=24.078600883483887
node_922: feature_name=GO:0003908 feature_id[11].value <= threshold=1.7776933312416077
node_923: feature_name=GO:0030098 feature_id[273].value > threshold=0.0079949083738029
node_927: feature_name=GO:0006808 feature_id[513].value <= threshold=4.34592080116272
node_928: feature_name=GO:0071887 feature_id[283].value <= threshold=10.353787899017334
node_929: feature_name=GO:0038001 feature_id[28].value <= threshold=3.9469382762908936
node_930: feature_name=GO:0042287 feature_id[20].value <= threshold=3.6209195852279663
node_931: feature_name=GO:0003968 feature_id[47].value <= threshold=2.004227638244629
node_932: feature_name=GO:0002698 feature_id[395].value <= threshold=18.51447582244873

node_933: feature_name=GO:0044710 feature_id[719].value > threshold=179.7340316772461
Class: positive genes

Rules_530 passed counts:1
node_0: feature_name=GO:0042113 feature_id[0].value <= threshold=13.408552169799805
node_1: feature_name=GO:0007568 feature_id[534].value > threshold=5.0313897132873535
node_913: feature_name=GO:0032763 feature_id[629].value <= threshold=0.31753237545490265
node_914: feature_name=GO:0097193 feature_id[167].value <= threshold=28.171168327331543
node_915: feature_name=GO:0002903 feature_id[10].value <= threshold=1.0534588098526
node_916: feature_name=GO:1904029 feature_id[208].value <= threshold=23.852136611938477
node_917: feature_name=GO:0050897 feature_id[164].value <= threshold=0.6870408356189728
node_918: feature_name=GO:0006139 feature_id[474].value > threshold=1.3052097624921544e-07
node_920: feature_name=GO:0002821 feature_id[588].value <= threshold=13.81072187423706
node_921: feature_name=GO:0006298 feature_id[621].value <= threshold=24.078600883483887
node_922: feature_name=GO:0003908 feature_id[11].value <= threshold=1.7776933312416077
node_923: feature_name=GO:0030098 feature_id[273].value > threshold=0.0079949083738029
node_927: feature_name=GO:0006808 feature_id[513].value <= threshold=4.34592080116272
node_928: feature_name=GO:0071887 feature_id[283].value <= threshold=10.353787899017334
node_929: feature_name=GO:0038001 feature_id[28].value <= threshold=3.9469382762908936
node_930: feature_name=GO:0042287 feature_id[20].value <= threshold=3.6209195852279663
node_931: feature_name=GO:0003968 feature_id[47].value <= threshold=2.004227638244629
node_932: feature_name=GO:0002698 feature_id[395].value <= threshold=18.51447582244873
node_933: feature_name=GO:0044710 feature_id[719].value <= threshold=179.7340316772461
node_934: feature_name=GO:0007568 feature_id[534].value > threshold=5.035318374633789
node_936: feature_name=GO:0006216 feature_id[504].value > threshold=2.2605666518211365
Class: positive genes

Rules_531 passed counts:1
node_0: feature_name=GO:0042113 feature_id[0].value <= threshold=13.408552169799805
node_1: feature_name=GO:0007568 feature_id[534].value > threshold=5.0313897132873535
node_913: feature_name=GO:0032763 feature_id[629].value <= threshold=0.31753237545490265
node_914: feature_name=GO:0097193 feature_id[167].value <= threshold=28.171168327331543
node_915: feature_name=GO:0002903 feature_id[10].value <= threshold=1.0534588098526
node_916: feature_name=GO:1904029 feature_id[208].value <= threshold=23.852136611938477
node_917: feature_name=GO:0050897 feature_id[164].value <= threshold=0.6870408356189728

node_918: feature_name=GO:0006139 feature_id[474].value > threshold=1.3052097624921544e-07
node_920: feature_name=GO:0002821 feature_id[588].value <= threshold=13.81072187423706
node_921: feature_name=GO:0006298 feature_id[621].value <= threshold=24.078600883483887
node_922: feature_name=GO:0003908 feature_id[11].value <= threshold=1.7776933312416077
node_923: feature_name=GO:0030098 feature_id[273].value > threshold=0.0079949083738029
node_927: feature_name=GO:0006808 feature_id[513].value <= threshold=4.34592080116272
node_928: feature_name=GO:0071887 feature_id[283].value <= threshold=10.353787899017334
node_929: feature_name=GO:0038001 feature_id[28].value <= threshold=3.9469382762908936
node_930: feature_name=GO:0042287 feature_id[20].value <= threshold=3.6209195852279663
node_931: feature_name=GO:0003968 feature_id[47].value <= threshold=2.004227638244629
node_932: feature_name=GO:0002698 feature_id[395].value <= threshold=18.51447582244873
node_933: feature_name=GO:0044710 feature_id[719].value <= threshold=179.7340316772461
node_934: feature_name=GO:0007568 feature_id[534].value > threshold=5.035318374633789
node_936: feature_name=GO:0006216 feature_id[504].value <= threshold=2.2605666518211365
node_937: feature_name=GO:0048569 feature_id[793].value > threshold=5.340231895446777
Class: positive genes

Rules_532 passed counts:1
node_0: feature_name=GO:0042113 feature_id[0].value <= threshold=13.408552169799805
node_1: feature_name=GO:0007568 feature_id[534].value > threshold=5.0313897132873535
node_913: feature_name=GO:0032763 feature_id[629].value <= threshold=0.31753237545490265
node_914: feature_name=GO:0097193 feature_id[167].value <= threshold=28.171168327331543
node_915: feature_name=GO:0002903 feature_id[10].value <= threshold=1.0534588098526
node_916: feature_name=GO:1904029 feature_id[208].value <= threshold=23.852136611938477
node_917: feature_name=GO:0050897 feature_id[164].value <= threshold=0.6870408356189728
node_918: feature_name=GO:0006139 feature_id[474].value > threshold=1.3052097624921544e-07
node_920: feature_name=GO:0002821 feature_id[588].value <= threshold=13.81072187423706
node_921: feature_name=GO:0006298 feature_id[621].value <= threshold=24.078600883483887
node_922: feature_name=GO:0003908 feature_id[11].value <= threshold=1.7776933312416077
node_923: feature_name=GO:0030098 feature_id[273].value > threshold=0.0079949083738029
node_927: feature_name=GO:0006808 feature_id[513].value <= threshold=4.34592080116272
node_928: feature_name=GO:0071887 feature_id[283].value <= threshold=10.353787899017334
node_929: feature_name=GO:0038001 feature_id[28].value <= threshold=3.9469382762908936
node_930: feature_name=GO:0042287 feature_id[20].value <= threshold=3.6209195852279663
node_931: feature_name=GO:0003968 feature_id[47].value <= threshold=2.004227638244629

node_932: feature_name=GO:0002698 feature_id[395].value <= threshold=18.51447582244873
node_933: feature_name=GO:0044710 feature_id[719].value <= threshold=179.7340316772461
node_934: feature_name=GO:0007568 feature_id[534].value > threshold=5.035318374633789
node_936: feature_name=GO:0006216 feature_id[504].value <= threshold=2.2605666518211365
node_937: feature_name=GO:0048569 feature_id[793].value <= threshold=5.340231895446777
node_938: feature_name=GO:0001777 feature_id[380].value > threshold=4.100832939147949
Class: positive genes

Rules_533 passed counts:1
node_0: feature_name=GO:0042113 feature_id[0].value <= threshold=13.408552169799805
node_1: feature_name=GO:0007568 feature_id[534].value > threshold=5.0313897132873535
node_913: feature_name=GO:0032763 feature_id[629].value <= threshold=0.31753237545490265
node_914: feature_name=GO:0097193 feature_id[167].value <= threshold=28.171168327331543
node_915: feature_name=GO:0002903 feature_id[10].value <= threshold=1.0534588098526
node_916: feature_name=GO:1904029 feature_id[208].value <= threshold=23.852136611938477
node_917: feature_name=GO:0050897 feature_id[164].value <= threshold=0.6870408356189728
node_918: feature_name=GO:0006139 feature_id[474].value > threshold=1.3052097624921544e-07
node_920: feature_name=GO:0002821 feature_id[588].value <= threshold=13.81072187423706
node_921: feature_name=GO:0006298 feature_id[621].value <= threshold=24.078600883483887
node_922: feature_name=GO:0003908 feature_id[11].value <= threshold=1.7776933312416077
node_923: feature_name=GO:0030098 feature_id[273].value > threshold=0.0079949083738029
node_927: feature_name=GO:0006808 feature_id[513].value <= threshold=4.34592080116272
node_928: feature_name=GO:0071887 feature_id[283].value <= threshold=10.353787899017334
node_929: feature_name=GO:0038001 feature_id[28].value <= threshold=3.9469382762908936
node_930: feature_name=GO:0042287 feature_id[20].value <= threshold=3.6209195852279663
node_931: feature_name=GO:0003968 feature_id[47].value <= threshold=2.004227638244629
node_932: feature_name=GO:0002698 feature_id[395].value <= threshold=18.51447582244873
node_933: feature_name=GO:0044710 feature_id[719].value <= threshold=179.7340316772461
node_934: feature_name=GO:0007568 feature_id[534].value > threshold=5.035318374633789
node_936: feature_name=GO:0006216 feature_id[504].value <= threshold=2.2605666518211365
node_937: feature_name=GO:0048569 feature_id[793].value <= threshold=5.340231895446777
node_938: feature_name=GO:0001777 feature_id[380].value <= threshold=4.100832939147949
node_939: feature_name=GO:0007600 feature_id[122].value > threshold=171.13383102416992
Class: positive genes

Rules_534 passed counts:1
node_0: feature_name=GO:0042113 feature_id[0].value <= threshold=13.408552169799805
node_1: feature_name=GO:0007568 feature_id[534].value > threshold=5.0313897132873535
node_913: feature_name=GO:0032763 feature_id[629].value <= threshold=0.31753237545490265
node_914: feature_name=GO:0097193 feature_id[167].value <= threshold=28.171168327331543
node_915: feature_name=GO:0002903 feature_id[10].value <= threshold=1.0534588098526
node_916: feature_name=GO:1904029 feature_id[208].value <= threshold=23.852136611938477
node_917: feature_name=GO:0050897 feature_id[164].value <= threshold=0.6870408356189728
node_918: feature_name=GO:0006139 feature_id[474].value > threshold=1.3052097624921544e-07
node_920: feature_name=GO:0002821 feature_id[588].value <= threshold=13.81072187423706
node_921: feature_name=GO:0006298 feature_id[621].value <= threshold=24.078600883483887
node_922: feature_name=GO:0003908 feature_id[11].value <= threshold=1.7776933312416077
node_923: feature_name=GO:0030098 feature_id[273].value > threshold=0.0079949083738029
node_927: feature_name=GO:0006808 feature_id[513].value <= threshold=4.34592080116272
node_928: feature_name=GO:0071887 feature_id[283].value <= threshold=10.353787899017334
node_929: feature_name=GO:0038001 feature_id[28].value <= threshold=3.9469382762908936
node_930: feature_name=GO:0042287 feature_id[20].value <= threshold=3.6209195852279663
node_931: feature_name=GO:0003968 feature_id[47].value <= threshold=2.004227638244629
node_932: feature_name=GO:0002698 feature_id[395].value <= threshold=18.51447582244873
node_933: feature_name=GO:0044710 feature_id[719].value <= threshold=179.7340316772461
node_934: feature_name=GO:0007568 feature_id[534].value > threshold=5.035318374633789
node_936: feature_name=GO:0006216 feature_id[504].value <= threshold=2.2605666518211365
node_937: feature_name=GO:0048569 feature_id[793].value <= threshold=5.340231895446777
node_938: feature_name=GO:0001777 feature_id[380].value <= threshold=4.100832939147949
node_939: feature_name=GO:0007600 feature_id[122].value <= threshold=171.13383102416992
node_940: feature_name=GO:0001779 feature_id[378].value > threshold=6.234851121902466
Class: positive genes

Rules_535 passed counts:1
node_0: feature_name=GO:0042113 feature_id[0].value <= threshold=13.408552169799805
node_1: feature_name=GO:0007568 feature_id[534].value > threshold=5.0313897132873535
node_913: feature_name=GO:0032763 feature_id[629].value <= threshold=0.31753237545490265
node_914: feature_name=GO:0097193 feature_id[167].value <= threshold=28.171168327331543
node_915: feature_name=GO:0002903 feature_id[10].value <= threshold=1.0534588098526
node_916: feature_name=GO:1904029 feature_id[208].value <= threshold=23.852136611938477

node_917: feature_name=GO:0050897 feature_id[164].value <= threshold=0.6870408356189728
node_918: feature_name=GO:0006139 feature_id[474].value > threshold=1.3052097624921544e-07
node_920: feature_name=GO:0002821 feature_id[588].value <= threshold=13.81072187423706
node_921: feature_name=GO:0006298 feature_id[621].value <= threshold=24.078600883483887
node_922: feature_name=GO:0003908 feature_id[11].value <= threshold=1.7776933312416077
node_923: feature_name=GO:0030098 feature_id[273].value > threshold=0.0079949083738029
node_927: feature_name=GO:0006808 feature_id[513].value <= threshold=4.34592080116272
node_928: feature_name=GO:0071887 feature_id[283].value <= threshold=10.353787899017334
node_929: feature_name=GO:0038001 feature_id[28].value <= threshold=3.9469382762908936
node_930: feature_name=GO:0042287 feature_id[20].value <= threshold=3.6209195852279663
node_931: feature_name=GO:0003968 feature_id[47].value <= threshold=2.004227638244629
node_932: feature_name=GO:0002698 feature_id[395].value <= threshold=18.51447582244873
node_933: feature_name=GO:0044710 feature_id[719].value <= threshold=179.7340316772461
node_934: feature_name=GO:0007568 feature_id[534].value > threshold=5.035318374633789
node_936: feature_name=GO:0006216 feature_id[504].value <= threshold=2.2605666518211365
node_937: feature_name=GO:0048569 feature_id[793].value <= threshold=5.340231895446777
node_938: feature_name=GO:0001777 feature_id[380].value <= threshold=4.100832939147949
node_939: feature_name=GO:0007600 feature_id[122].value <= threshold=171.13383102416992
node_940: feature_name=GO:0001779 feature_id[378].value <= threshold=6.234851121902466
node_941: feature_name=GO:0030291 feature_id[147].value > threshold=10.8051118850708
Class: positive genes

Rules_536 passed counts:1
node_0: feature_name=GO:0042113 feature_id[0].value <= threshold=13.408552169799805
node_1: feature_name=GO:0007568 feature_id[534].value > threshold=5.0313897132873535
node_913: feature_name=GO:0032763 feature_id[629].value <= threshold=0.31753237545490265
node_914: feature_name=GO:0097193 feature_id[167].value <= threshold=28.171168327331543
node_915: feature_name=GO:0002903 feature_id[10].value <= threshold=1.0534588098526
node_916: feature_name=GO:1904029 feature_id[208].value <= threshold=23.852136611938477
node_917: feature_name=GO:0050897 feature_id[164].value <= threshold=0.6870408356189728
node_918: feature_name=GO:0006139 feature_id[474].value > threshold=1.3052097624921544e-07
node_920: feature_name=GO:0002821 feature_id[588].value <= threshold=13.81072187423706
node_921: feature_name=GO:0006298 feature_id[621].value <= threshold=24.078600883483887
node_922: feature_name=GO:0003908 feature_id[11].value <= threshold=1.7776933312416077
node_923: feature_name=GO:0030098 feature_id[273].value > threshold=0.0079949083738029

node_927: feature_name=GO:0006808 feature_id[513].value <= threshold=4.34592080116272
node_928: feature_name=GO:0071887 feature_id[283].value <= threshold=10.353787899017334
node_929: feature_name=GO:0038001 feature_id[28].value <= threshold=3.9469382762908936
node_930: feature_name=GO:0042287 feature_id[20].value <= threshold=3.6209195852279663
node_931: feature_name=GO:0003968 feature_id[47].value <= threshold=2.004227638244629
node_932: feature_name=GO:0002698 feature_id[395].value <= threshold=18.51447582244873
node_933: feature_name=GO:0044710 feature_id[719].value <= threshold=179.7340316772461
node_934: feature_name=GO:0007568 feature_id[534].value > threshold=5.035318374633789
node_936: feature_name=GO:0006216 feature_id[504].value <= threshold=2.2605666518211365
node_937: feature_name=GO:0048569 feature_id[793].value <= threshold=5.340231895446777
node_938: feature_name=GO:0001777 feature_id[380].value <= threshold=4.100832939147949
node_939: feature_name=GO:0007600 feature_id[122].value <= threshold=171.13383102416992
node_940: feature_name=GO:0001779 feature_id[378].value <= threshold=6.234851121902466
node_941: feature_name=GO:0030291 feature_id[147].value <= threshold=10.8051118850708
node_942: feature_name=GO:0048534 feature_id[790].value > threshold=0.017984486185014248
node_944: feature_name=GO:0070245 feature_id[326].value <= threshold=3.1282339096069336
node_945: feature_name=GO:0009086 feature_id[547].value > threshold=0.2896959036588669
node_1017: feature_name=GO:0000302 feature_id[375].value > threshold=0.305715873837471
node_1019: feature_name=GO:0070141 feature_id[37].value <= threshold=4.857294321060181
node_1020: feature_name=GO:0009086 feature_id[547].value > threshold=0.2945319563150406
node_1024: feature_name=GO:0010332 feature_id[557].value > threshold=15.092710971832275
Class: positive genes

Rules_537 passed counts:1
node_0: feature_name=GO:0042113 feature_id[0].value <= threshold=13.408552169799805
node_1: feature_name=GO:0007568 feature_id[534].value > threshold=5.0313897132873535
node_913: feature_name=GO:0032763 feature_id[629].value <= threshold=0.31753237545490265
node_914: feature_name=GO:0097193 feature_id[167].value <= threshold=28.171168327331543
node_915: feature_name=GO:0002903 feature_id[10].value <= threshold=1.0534588098526
node_916: feature_name=GO:1904029 feature_id[208].value <= threshold=23.852136611938477
node_917: feature_name=GO:0050897 feature_id[164].value <= threshold=0.6870408356189728
node_918: feature_name=GO:0006139 feature_id[474].value > threshold=1.3052097624921544e-07
node_920: feature_name=GO:0002821 feature_id[588].value <= threshold=13.81072187423706
node_921: feature_name=GO:0006298 feature_id[621].value <= threshold=24.078600883483887
node_922: feature_name=GO:0003908 feature_id[11].value <= threshold=1.7776933312416077

node_923: feature_name=GO:0030098 feature_id[273].value > threshold=0.0079949083738029
node_927: feature_name=GO:0006808 feature_id[513].value <= threshold=4.34592080116272
node_928: feature_name=GO:0071887 feature_id[283].value <= threshold=10.353787899017334
node_929: feature_name=GO:0038001 feature_id[28].value <= threshold=3.9469382762908936
node_930: feature_name=GO:0042287 feature_id[20].value <= threshold=3.6209195852279663
node_931: feature_name=GO:0003968 feature_id[47].value <= threshold=2.004227638244629
node_932: feature_name=GO:0002698 feature_id[395].value <= threshold=18.51447582244873
node_933: feature_name=GO:0044710 feature_id[719].value <= threshold=179.7340316772461
node_934: feature_name=GO:0007568 feature_id[534].value > threshold=5.035318374633789
node_936: feature_name=GO:0006216 feature_id[504].value <= threshold=2.2605666518211365
node_937: feature_name=GO:0048569 feature_id[793].value <= threshold=5.340231895446777
node_938: feature_name=GO:0001777 feature_id[380].value <= threshold=4.100832939147949
node_939: feature_name=GO:0007600 feature_id[122].value <= threshold=171.13383102416992
node_940: feature_name=GO:0001779 feature_id[378].value <= threshold=6.234851121902466
node_941: feature_name=GO:0030291 feature_id[147].value <= threshold=10.8051118850708
node_942: feature_name=GO:0048534 feature_id[790].value > threshold=0.017984486185014248
node_944: feature_name=GO:0070245 feature_id[326].value <= threshold=3.1282339096069336
node_945: feature_name=GO:0009086 feature_id[547].value > threshold=0.2896959036588669
node_1017: feature_name=GO:0000302 feature_id[375].value > threshold=0.305715873837471
node_1019: feature_name=GO:0070141 feature_id[37].value <= threshold=4.857294321060181
node_1020: feature_name=GO:0009086 feature_id[547].value > threshold=0.2945319563150406
node_1024: feature_name=GO:0010332 feature_id[557].value <= threshold=15.092710971832275
node_1025: feature_name=GO:0002524 feature_id[72].value > threshold=2.531021237373352
Class: positive genes

Rules_538 passed counts:1
node_0: feature_name=GO:0042113 feature_id[0].value <= threshold=13.408552169799805
node_1: feature_name=GO:0007568 feature_id[534].value > threshold=5.0313897132873535
node_913: feature_name=GO:0032763 feature_id[629].value <= threshold=0.31753237545490265
node_914: feature_name=GO:0097193 feature_id[167].value <= threshold=28.171168327331543
node_915: feature_name=GO:0002903 feature_id[10].value <= threshold=1.0534588098526
node_916: feature_name=GO:1904029 feature_id[208].value <= threshold=23.852136611938477
node_917: feature_name=GO:0050897 feature_id[164].value <= threshold=0.6870408356189728
node_918: feature_name=GO:0006139 feature_id[474].value > threshold=1.3052097624921544e-07
node_920: feature_name=GO:0002821 feature_id[588].value <= threshold=13.81072187423706

node_921: feature_name=GO:0006298 feature_id[621].value <= threshold=24.078600883483887
node_922: feature_name=GO:0003908 feature_id[11].value <= threshold=1.7776933312416077
node_923: feature_name=GO:0030098 feature_id[273].value > threshold=0.0079949083738029
node_927: feature_name=GO:0006808 feature_id[513].value <= threshold=4.34592080116272
node_928: feature_name=GO:0071887 feature_id[283].value <= threshold=10.353787899017334
node_929: feature_name=GO:0038001 feature_id[28].value <= threshold=3.9469382762908936
node_930: feature_name=GO:0042287 feature_id[20].value <= threshold=3.6209195852279663
node_931: feature_name=GO:0003968 feature_id[47].value <= threshold=2.004227638244629
node_932: feature_name=GO:0002698 feature_id[395].value <= threshold=18.51447582244873
node_933: feature_name=GO:0044710 feature_id[719].value <= threshold=179.7340316772461
node_934: feature_name=GO:0007568 feature_id[534].value > threshold=5.035318374633789
node_936: feature_name=GO:0006216 feature_id[504].value <= threshold=2.2605666518211365
node_937: feature_name=GO:0048569 feature_id[793].value <= threshold=5.340231895446777
node_938: feature_name=GO:0001777 feature_id[380].value <= threshold=4.100832939147949
node_939: feature_name=GO:0007600 feature_id[122].value <= threshold=171.13383102416992
node_940: feature_name=GO:0001779 feature_id[378].value <= threshold=6.234851121902466
node_941: feature_name=GO:0030291 feature_id[147].value <= threshold=10.8051118850708
node_942: feature_name=GO:0048534 feature_id[790].value > threshold=0.017984486185014248
node_944: feature_name=GO:0070245 feature_id[326].value <= threshold=3.1282339096069336
node_945: feature_name=GO:0009086 feature_id[547].value > threshold=0.2896959036588669
node_1017: feature_name=GO:0000302 feature_id[375].value > threshold=0.305715873837471
node_1019: feature_name=GO:0070141 feature_id[37].value <= threshold=4.857294321060181
node_1020: feature_name=GO:0009086 feature_id[547].value > threshold=0.2945319563150406
node_1024: feature_name=GO:0010332 feature_id[557].value <= threshold=15.092710971832275
node_1025: feature_name=GO:0002524 feature_id[72].value <= threshold=2.531021237373352
node_1026: feature_name=GO:0016032 feature_id[571].value > threshold=60.173330307006836
Class: positive genes

Rules_539 passed counts:1
node_0: feature_name=GO:0042113 feature_id[0].value <= threshold=13.408552169799805
node_1: feature_name=GO:0007568 feature_id[534].value > threshold=5.0313897132873535
node_913: feature_name=GO:0032763 feature_id[629].value <= threshold=0.31753237545490265
node_914: feature_name=GO:0097193 feature_id[167].value <= threshold=28.171168327331543
node_915: feature_name=GO:0002903 feature_id[10].value <= threshold=1.0534588098526
node_916: feature_name=GO:1904029 feature_id[208].value <= threshold=23.852136611938477

node_917: feature_name=GO:0050897 feature_id[164].value <= threshold=0.6870408356189728
node_918: feature_name=GO:0006139 feature_id[474].value > threshold=1.3052097624921544e-07
node_920: feature_name=GO:0002821 feature_id[588].value <= threshold=13.81072187423706
node_921: feature_name=GO:0006298 feature_id[621].value <= threshold=24.078600883483887
node_922: feature_name=GO:0003908 feature_id[11].value <= threshold=1.7776933312416077
node_923: feature_name=GO:0030098 feature_id[273].value > threshold=0.0079949083738029
node_927: feature_name=GO:0006808 feature_id[513].value <= threshold=4.34592080116272
node_928: feature_name=GO:0071887 feature_id[283].value <= threshold=10.353787899017334
node_929: feature_name=GO:0038001 feature_id[28].value <= threshold=3.9469382762908936
node_930: feature_name=GO:0042287 feature_id[20].value <= threshold=3.6209195852279663
node_931: feature_name=GO:0003968 feature_id[47].value <= threshold=2.004227638244629
node_932: feature_name=GO:0002698 feature_id[395].value <= threshold=18.51447582244873
node_933: feature_name=GO:0044710 feature_id[719].value <= threshold=179.7340316772461
node_934: feature_name=GO:0007568 feature_id[534].value > threshold=5.035318374633789
node_936: feature_name=GO:0006216 feature_id[504].value <= threshold=2.2605666518211365
node_937: feature_name=GO:0048569 feature_id[793].value <= threshold=5.340231895446777
node_938: feature_name=GO:0001777 feature_id[380].value <= threshold=4.100832939147949
node_939: feature_name=GO:0007600 feature_id[122].value <= threshold=171.13383102416992
node_940: feature_name=GO:0001779 feature_id[378].value <= threshold=6.234851121902466
node_941: feature_name=GO:0030291 feature_id[147].value <= threshold=10.8051118850708
node_942: feature_name=GO:0048534 feature_id[790].value > threshold=0.017984486185014248
node_944: feature_name=GO:0070245 feature_id[326].value <= threshold=3.1282339096069336
node_945: feature_name=GO:0009086 feature_id[547].value > threshold=0.2896959036588669
node_1017: feature_name=GO:0000302 feature_id[375].value > threshold=0.305715873837471
node_1019: feature_name=GO:0070141 feature_id[37].value <= threshold=4.857294321060181
node_1020: feature_name=GO:0009086 feature_id[547].value > threshold=0.2945319563150406
node_1024: feature_name=GO:0010332 feature_id[557].value <= threshold=15.092710971832275
node_1025: feature_name=GO:0002524 feature_id[72].value <= threshold=2.531021237373352
node_1026: feature_name=GO:0016032 feature_id[571].value <= threshold=60.173330307006836
node_1027: feature_name=GO:0002832 feature_id[491].value > threshold=2.0699684023857117
Class: positive genes

Rules_540 passed counts:1
node_0: feature_name=GO:0042113 feature_id[0].value <= threshold=13.408552169799805
node_1: feature_name=GO:0007568 feature_id[534].value > threshold=5.0313897132873535

node_913: feature_name=GO:0032763	feature_id[629].value <= threshold=0.31753237545490265
node_914: feature_name=GO:0097193	feature_id[167].value <= threshold=28.171168327331543
node_915: feature_name=GO:0002903	feature_id[10].value <= threshold=1.0534588098526
node_916: feature_name=GO:1904029	feature_id[208].value <= threshold=23.852136611938477
node_917: feature_name=GO:0050897	feature_id[164].value <= threshold=0.6870408356189728
node_918: feature_name=GO:0006139	feature_id[474].value > threshold=1.3052097624921544e-07
node_920: feature_name=GO:0002821	feature_id[588].value <= threshold=13.81072187423706
node_921: feature_name=GO:0006298	feature_id[621].value <= threshold=24.078600883483887
node_922: feature_name=GO:0003908	feature_id[11].value <= threshold=1.7776933312416077
node_923: feature_name=GO:0030098	feature_id[273].value > threshold=0.0079949083738029
node_927: feature_name=GO:0006808	feature_id[513].value <= threshold=4.34592080116272
node_928: feature_name=GO:0071887	feature_id[283].value <= threshold=10.353787899017334
node_929: feature_name=GO:0038001	feature_id[28].value <= threshold=3.9469382762908936
node_930: feature_name=GO:0042287	feature_id[20].value <= threshold=3.6209195852279663
node_931: feature_name=GO:0003968	feature_id[47].value <= threshold=2.004227638244629
node_932: feature_name=GO:0002698	feature_id[395].value <= threshold=18.51447582244873
node_933: feature_name=GO:0044710	feature_id[719].value <= threshold=179.7340316772461
node_934: feature_name=GO:0007568	feature_id[534].value > threshold=5.035318374633789
node_936: feature_name=GO:0006216	feature_id[504].value <= threshold=2.2605666518211365
node_937: feature_name=GO:0048569	feature_id[793].value <= threshold=5.340231895446777
node_938: feature_name=GO:0001777	feature_id[380].value <= threshold=4.100832939147949
node_939: feature_name=GO:0007600	feature_id[122].value <= threshold=171.13383102416992
node_940: feature_name=GO:0001779	feature_id[378].value <= threshold=6.234851121902466
node_941: feature_name=GO:0030291	feature_id[147].value <= threshold=10.8051118850708
node_942: feature_name=GO:0048534	feature_id[790].value > threshold=0.017984486185014248
node_944: feature_name=GO:0070245	feature_id[326].value <= threshold=3.1282339096069336
node_945: feature_name=GO:0009086	feature_id[547].value > threshold=0.2896959036588669
node_1017: feature_name=GO:0000302	feature_id[375].value > threshold=0.305715873837471
node_1019: feature_name=GO:0070141	feature_id[37].value <= threshold=4.857294321060181
node_1020: feature_name=GO:0009086	feature_id[547].value > threshold=0.2945319563150406
node_1024: feature_name=GO:0010332	feature_id[557].value <= threshold=15.092710971832275
node_1025: feature_name=GO:0002524	feature_id[72].value <= threshold=2.531021237373352
node_1026: feature_name=GO:0016032	feature_id[571].value <= threshold=60.173330307006836
node_1027: feature_name=GO:0002832	feature_id[491].value <= threshold=2.0699684023857117
node_1028: feature_name=GO:0008588	feature_id[26].value > threshold=2.150238513946533

Class: positive genes

Rules_541	passed counts:1
node_0: feature_name=GO:0042113	feature_id[0].value <= threshold=13.408552169799805
node_1: feature_name=GO:0007568	feature_id[534].value > threshold=5.0313897132873535
node_913: feature_name=GO:0032763	feature_id[629].value <= threshold=0.31753237545490265
node_914: feature_name=GO:0097193	feature_id[167].value <= threshold=28.171168327331543
node_915: feature_name=GO:0002903	feature_id[10].value <= threshold=1.0534588098526
node_916: feature_name=GO:1904029	feature_id[208].value <= threshold=23.852136611938477
node_917: feature_name=GO:0050897	feature_id[164].value <= threshold=0.6870408356189728
node_918: feature_name=GO:0006139	feature_id[474].value > threshold=1.3052097624921544e-07
node_920: feature_name=GO:0002821	feature_id[588].value <= threshold=13.81072187423706
node_921: feature_name=GO:0006298	feature_id[621].value <= threshold=24.078600883483887
node_922: feature_name=GO:0003908	feature_id[11].value <= threshold=1.7776933312416077
node_923: feature_name=GO:0030098	feature_id[273].value > threshold=0.0079949083738029
node_927: feature_name=GO:0006808	feature_id[513].value <= threshold=4.34592080116272
node_928: feature_name=GO:0071887	feature_id[283].value <= threshold=10.353787899017334
node_929: feature_name=GO:0038001	feature_id[28].value <= threshold=3.9469382762908936
node_930: feature_name=GO:0042287	feature_id[20].value <= threshold=3.6209195852279663
node_931: feature_name=GO:0003968	feature_id[47].value <= threshold=2.004227638244629
node_932: feature_name=GO:0002698	feature_id[395].value <= threshold=18.51447582244873
node_933: feature_name=GO:0044710	feature_id[719].value <= threshold=179.7340316772461
node_934: feature_name=GO:0007568	feature_id[534].value > threshold=5.035318374633789
node_936: feature_name=GO:0006216	feature_id[504].value <= threshold=2.2605666518211365
node_937: feature_name=GO:0048569	feature_id[793].value <= threshold=5.340231895446777
node_938: feature_name=GO:0001777	feature_id[380].value <= threshold=4.100832939147949
node_939: feature_name=GO:0007600	feature_id[122].value <= threshold=171.13383102416992
node_940: feature_name=GO:0001779	feature_id[378].value <= threshold=6.234851121902466
node_941: feature_name=GO:0030291	feature_id[147].value <= threshold=10.8051118850708
node_942: feature_name=GO:0048534	feature_id[790].value > threshold=0.017984486185014248
node_944: feature_name=GO:0070245	feature_id[326].value <= threshold=3.1282339096069336
node_945: feature_name=GO:0009086	feature_id[547].value > threshold=0.2896959036588669
node_1017: feature_name=GO:0000302	feature_id[375].value > threshold=0.305715873837471
node_1019: feature_name=GO:0070141	feature_id[37].value <= threshold=4.857294321060181
node_1020: feature_name=GO:0009086	feature_id[547].value > threshold=0.2945319563150406

node_1024: feature_name=GO:0010332 feature_id[557].value <= threshold=15.092710971832275
node_1025: feature_name=GO:0002524 feature_id[72].value <= threshold=2.531021237373352
node_1026: feature_name=GO:0016032 feature_id[571].value <= threshold=60.173330307006836
node_1027: feature_name=GO:0002832 feature_id[491].value <= threshold=2.0699684023857117
node_1028: feature_name=GO:0008588 feature_id[26].value <= threshold=2.150238513946533
node_1029: feature_name=GO:0009615 feature_id[265].value > threshold=0.02372477948665619
node_1031: feature_name=GO:0002763 feature_id[271].value > threshold=6.462319850921631
Class: positive genes

Rules_542 passed counts:1
node_0: feature_name=GO:0042113 feature_id[0].value <= threshold=13.408552169799805
node_1: feature_name=GO:0007568 feature_id[534].value > threshold=5.0313897132873535
node_913: feature_name=GO:0032763 feature_id[629].value <= threshold=0.31753237545490265
node_914: feature_name=GO:0097193 feature_id[167].value <= threshold=28.171168327331543
node_915: feature_name=GO:0002903 feature_id[10].value <= threshold=1.0534588098526
node_916: feature_name=GO:1904029 feature_id[208].value <= threshold=23.852136611938477
node_917: feature_name=GO:0050897 feature_id[164].value <= threshold=0.6870408356189728
node_918: feature_name=GO:0006139 feature_id[474].value > threshold=1.3052097624921544e-07
node_920: feature_name=GO:0002821 feature_id[588].value <= threshold=13.81072187423706
node_921: feature_name=GO:0006298 feature_id[621].value <= threshold=24.078600883483887
node_922: feature_name=GO:0003908 feature_id[11].value <= threshold=1.7776933312416077
node_923: feature_name=GO:0030098 feature_id[273].value > threshold=0.0079949083738029
node_927: feature_name=GO:0006808 feature_id[513].value <= threshold=4.34592080116272
node_928: feature_name=GO:0071887 feature_id[283].value <= threshold=10.353787899017334
node_929: feature_name=GO:0038001 feature_id[28].value <= threshold=3.9469382762908936
node_930: feature_name=GO:0042287 feature_id[20].value <= threshold=3.6209195852279663
node_931: feature_name=GO:0003968 feature_id[47].value <= threshold=2.004227638244629
node_932: feature_name=GO:0002698 feature_id[395].value <= threshold=18.51447582244873
node_933: feature_name=GO:0044710 feature_id[719].value <= threshold=179.7340316772461
node_934: feature_name=GO:0007568 feature_id[534].value > threshold=5.035318374633789
node_936: feature_name=GO:0006216 feature_id[504].value <= threshold=2.2605666518211365
node_937: feature_name=GO:0048569 feature_id[793].value <= threshold=5.340231895446777
node_938: feature_name=GO:0001777 feature_id[380].value <= threshold=4.100832939147949
node_939: feature_name=GO:0007600 feature_id[122].value <= threshold=171.13383102416992
node_940: feature_name=GO:0001779 feature_id[378].value <= threshold=6.234851121902466

node_941: feature_name=GO:0030291 feature_id[147].value <= threshold=10.8051118850708
node_942: feature_name=GO:0048534 feature_id[790].value > threshold=0.017984486185014248
node_944: feature_name=GO:0070245 feature_id[326].value <= threshold=3.1282339096069336
node_945: feature_name=GO:0009086 feature_id[547].value > threshold=0.2896959036588669
node_1017: feature_name=GO:0000302 feature_id[375].value > threshold=0.305715873837471
node_1019: feature_name=GO:0070141 feature_id[37].value <= threshold=4.857294321060181
node_1020: feature_name=GO:0009086 feature_id[547].value > threshold=0.2945319563150406
node_1024: feature_name=GO:0010332 feature_id[557].value <= threshold=15.092710971832275
node_1025: feature_name=GO:0002524 feature_id[72].value <= threshold=2.531021237373352
node_1026: feature_name=GO:0016032 feature_id[571].value <= threshold=60.173330307006836
node_1027: feature_name=GO:0002832 feature_id[491].value <= threshold=2.0699684023857117
node_1028: feature_name=GO:0008588 feature_id[26].value <= threshold=2.150238513946533
node_1029: feature_name=GO:0009615 feature_id[265].value > threshold=0.02372477948665619
node_1031: feature_name=GO:0002763 feature_id[271].value <= threshold=6.462319850921631
node_1032: feature_name=GO:0045840 feature_id[697].value > threshold=10.30175256729126
Class: positive genes

Rules_543 passed counts:1
node_0: feature_name=GO:0042113 feature_id[0].value <= threshold=13.408552169799805
node_1: feature_name=GO:0007568 feature_id[534].value > threshold=5.0313897132873535
node_913: feature_name=GO:0032763 feature_id[629].value <= threshold=0.31753237545490265
node_914: feature_name=GO:0097193 feature_id[167].value <= threshold=28.171168327331543
node_915: feature_name=GO:0002903 feature_id[10].value <= threshold=1.0534588098526
node_916: feature_name=GO:1904029 feature_id[208].value <= threshold=23.852136611938477
node_917: feature_name=GO:0050897 feature_id[164].value <= threshold=0.6870408356189728
node_918: feature_name=GO:0006139 feature_id[474].value > threshold=1.3052097624921544e-07
node_920: feature_name=GO:0002821 feature_id[588].value <= threshold=13.81072187423706
node_921: feature_name=GO:0006298 feature_id[621].value <= threshold=24.078600883483887
node_922: feature_name=GO:0003908 feature_id[11].value <= threshold=1.7776933312416077
node_923: feature_name=GO:0030098 feature_id[273].value > threshold=0.0079949083738029
node_927: feature_name=GO:0006808 feature_id[513].value <= threshold=4.34592080116272
node_928: feature_name=GO:0071887 feature_id[283].value <= threshold=10.353787899017334
node_929: feature_name=GO:0038001 feature_id[28].value <= threshold=3.9469382762908936
node_930: feature_name=GO:0042287 feature_id[20].value <= threshold=3.6209195852279663
node_931: feature_name=GO:0003968 feature_id[47].value <= threshold=2.004227638244629

node_932: feature_name=GO:0002698 feature_id[395].value <= threshold=18.51447582244873
node_933: feature_name=GO:0044710 feature_id[719].value <= threshold=179.7340316772461
node_934: feature_name=GO:0007568 feature_id[534].value > threshold=5.035318374633789
node_936: feature_name=GO:0006216 feature_id[504].value <= threshold=2.2605666518211365
node_937: feature_name=GO:0048569 feature_id[793].value <= threshold=5.340231895446777
node_938: feature_name=GO:0001777 feature_id[380].value <= threshold=4.100832939147949
node_939: feature_name=GO:0007600 feature_id[122].value <= threshold=171.13383102416992
node_940: feature_name=GO:0001779 feature_id[378].value <= threshold=6.234851121902466
node_941: feature_name=GO:0030291 feature_id[147].value <= threshold=10.8051118850708
node_942: feature_name=GO:0048534 feature_id[790].value > threshold=0.017984486185014248
node_944: feature_name=GO:0070245 feature_id[326].value <= threshold=3.1282339096069336
node_945: feature_name=GO:0009086 feature_id[547].value > threshold=0.2896959036588669
node_1017: feature_name=GO:0000302 feature_id[375].value > threshold=0.305715873837471
node_1019: feature_name=GO:0070141 feature_id[37].value <= threshold=4.857294321060181
node_1020: feature_name=GO:0009086 feature_id[547].value > threshold=0.2945319563150406
node_1024: feature_name=GO:0010332 feature_id[557].value <= threshold=15.092710971832275
node_1025: feature_name=GO:0002524 feature_id[72].value <= threshold=2.531021237373352
node_1026: feature_name=GO:0016032 feature_id[571].value <= threshold=60.173330307006836
node_1027: feature_name=GO:0002832 feature_id[491].value <= threshold=2.0699684023857117
node_1028: feature_name=GO:0008588 feature_id[26].value <= threshold=2.150238513946533
node_1029: feature_name=GO:0009615 feature_id[265].value > threshold=0.02372477948665619
node_1031: feature_name=GO:0002763 feature_id[271].value <= threshold=6.462319850921631
node_1032: feature_name=GO:0045840 feature_id[697].value <= threshold=10.30175256729126
node_1033: feature_name=GO:0051402 feature_id[568].value > threshold=1.2523645758628845
node_1041: feature_name=GO:0050852 feature_id[154].value > threshold=0.0008433434413745999
node_1043: feature_name=GO:0031667 feature_id[648].value > threshold=1.5418170094490051
node_1047: feature_name=GO:0009987 feature_id[554].value <= threshold=1.8002276420593262
node_1048: feature_name=GO:0038061 feature_id[671].value > threshold=1.3254979476332664
Class: positive genes

Rules_544 passed counts:1
node_0: feature_name=GO:0042113 feature_id[0].value <= threshold=13.408552169799805
node_1: feature_name=GO:0007568 feature_id[534].value > threshold=5.0313897132873535
node_913: feature_name=GO:0032763 feature_id[629].value <= threshold=0.31753237545490265
node_914: feature_name=GO:0097193 feature_id[167].value <= threshold=28.171168327331543

node_915: feature_name=GO:0002903	feature_id[10].value <= threshold=1.0534588098526
node_916: feature_name=GO:1904029	feature_id[208].value <= threshold=23.852136611938477
node_917: feature_name=GO:0050897	feature_id[164].value <= threshold=0.6870408356189728
node_918: feature_name=GO:0006139	feature_id[474].value > threshold=1.3052097624921544e-07
node_920: feature_name=GO:0002821	feature_id[588].value <= threshold=13.81072187423706
node_921: feature_name=GO:0006298	feature_id[621].value <= threshold=24.078600883483887
node_922: feature_name=GO:0003908	feature_id[11].value <= threshold=1.7776933312416077
node_923: feature_name=GO:0030098	feature_id[273].value > threshold=0.0079949083738029
node_927: feature_name=GO:0006808	feature_id[513].value <= threshold=4.34592080116272
node_928: feature_name=GO:0071887	feature_id[283].value <= threshold=10.353787899017334
node_929: feature_name=GO:0038001	feature_id[28].value <= threshold=3.9469382762908936
node_930: feature_name=GO:0042287	feature_id[20].value <= threshold=3.6209195852279663
node_931: feature_name=GO:0003968	feature_id[47].value <= threshold=2.004227638244629
node_932: feature_name=GO:0002698	feature_id[395].value <= threshold=18.51447582244873
node_933: feature_name=GO:0044710	feature_id[719].value <= threshold=179.7340316772461
node_934: feature_name=GO:0007568	feature_id[534].value > threshold=5.035318374633789
node_936: feature_name=GO:0006216	feature_id[504].value <= threshold=2.2605666518211365
node_937: feature_name=GO:0048569	feature_id[793].value <= threshold=5.340231895446777
node_938: feature_name=GO:0001777	feature_id[380].value <= threshold=4.100832939147949
node_939: feature_name=GO:0007600	feature_id[122].value <= threshold=171.13383102416992
node_940: feature_name=GO:0001779	feature_id[378].value <= threshold=6.234851121902466
node_941: feature_name=GO:0030291	feature_id[147].value <= threshold=10.8051118850708
node_942: feature_name=GO:0048534	feature_id[790].value > threshold=0.017984486185014248
node_944: feature_name=GO:0070245	feature_id[326].value <= threshold=3.1282339096069336
node_945: feature_name=GO:0009086	feature_id[547].value > threshold=0.2896959036588669
node_1017: feature_name=GO:0000302	feature_id[375].value > threshold=0.305715873837471
node_1019: feature_name=GO:0070141	feature_id[37].value <= threshold=4.857294321060181
node_1020: feature_name=GO:0009086	feature_id[547].value > threshold=0.2945319563150406
node_1024: feature_name=GO:0010332	feature_id[557].value <= threshold=15.092710971832275
node_1025: feature_name=GO:0002524	feature_id[72].value <= threshold=2.531021237373352
node_1026: feature_name=GO:0016032	feature_id[571].value <= threshold=60.173330307006836
node_1027: feature_name=GO:0002832	feature_id[491].value <= threshold=2.0699684023857117
node_1028: feature_name=GO:0008588	feature_id[26].value <= threshold=2.150238513946533
node_1029: feature_name=GO:0009615	feature_id[265].value > threshold=0.02372477948665619
node_1031: feature_name=GO:0002763	feature_id[271].value <= threshold=6.462319850921631

node_1032: feature_name=GO:0045840 feature_id[697].value <= threshold=10.30175256729126
node_1033: feature_name=GO:0051402 feature_id[568].value > threshold=1.2523645758628845
node_1041: feature_name=GO:0050852 feature_id[154].value > threshold=0.0008433434413745999
node_1043: feature_name=GO:0031667 feature_id[648].value <= threshold=1.5418170094490051
node_1044: feature_name=GO:0030101 feature_id[596].value > threshold=0.865232065320015
Class: negative genes

Rules_545 passed counts:1
node_0: feature_name=GO:0042113 feature_id[0].value <= threshold=13.408552169799805
node_1: feature_name=GO:0007568 feature_id[534].value > threshold=5.0313897132873535
node_913: feature_name=GO:0032763 feature_id[629].value <= threshold=0.31753237545490265
node_914: feature_name=GO:0097193 feature_id[167].value <= threshold=28.171168327331543
node_915: feature_name=GO:0002903 feature_id[10].value <= threshold=1.0534588098526
node_916: feature_name=GO:1904029 feature_id[208].value <= threshold=23.852136611938477
node_917: feature_name=GO:0050897 feature_id[164].value <= threshold=0.6870408356189728
node_918: feature_name=GO:0006139 feature_id[474].value > threshold=1.3052097624921544e-07
node_920: feature_name=GO:0002821 feature_id[588].value <= threshold=13.81072187423706
node_921: feature_name=GO:0006298 feature_id[621].value <= threshold=24.078600883483887
node_922: feature_name=GO:0003908 feature_id[11].value <= threshold=1.7776933312416077
node_923: feature_name=GO:0030098 feature_id[273].value > threshold=0.0079949083738029
node_927: feature_name=GO:0006808 feature_id[513].value <= threshold=4.34592080116272
node_928: feature_name=GO:0071887 feature_id[283].value <= threshold=10.353787899017334
node_929: feature_name=GO:0038001 feature_id[28].value <= threshold=3.9469382762908936
node_930: feature_name=GO:0042287 feature_id[20].value <= threshold=3.6209195852279663
node_931: feature_name=GO:0003968 feature_id[47].value <= threshold=2.004227638244629
node_932: feature_name=GO:0002698 feature_id[395].value <= threshold=18.51447582244873
node_933: feature_name=GO:0044710 feature_id[719].value <= threshold=179.7340316772461
node_934: feature_name=GO:0007568 feature_id[534].value > threshold=5.035318374633789
node_936: feature_name=GO:0006216 feature_id[504].value <= threshold=2.2605666518211365
node_937: feature_name=GO:0048569 feature_id[793].value <= threshold=5.340231895446777
node_938: feature_name=GO:0001777 feature_id[380].value <= threshold=4.100832939147949
node_939: feature_name=GO:0007600 feature_id[122].value <= threshold=171.13383102416992
node_940: feature_name=GO:0001779 feature_id[378].value <= threshold=6.234851121902466
node_941: feature_name=GO:0030291 feature_id[147].value <= threshold=10.8051118850708
node_942: feature_name=GO:0048534 feature_id[790].value > threshold=0.017984486185014248

node_944: feature_name=GO:0070245 feature_id[326].value <= threshold=3.1282339096069336
node_945: feature_name=GO:0009086 feature_id[547].value > threshold=0.2896959036588669
node_1017: feature_name=GO:0000302 feature_id[375].value > threshold=0.305715873837471
node_1019: feature_name=GO:0070141 feature_id[37].value <= threshold=4.857294321060181
node_1020: feature_name=GO:0009086 feature_id[547].value > threshold=0.2945319563150406
node_1024: feature_name=GO:0010332 feature_id[557].value <= threshold=15.092710971832275
node_1025: feature_name=GO:0002524 feature_id[72].value <= threshold=2.531021237373352
node_1026: feature_name=GO:0016032 feature_id[571].value <= threshold=60.173330307006836
node_1027: feature_name=GO:0002832 feature_id[491].value <= threshold=2.0699684023857117
node_1028: feature_name=GO:0008588 feature_id[26].value <= threshold=2.150238513946533
node_1029: feature_name=GO:0009615 feature_id[265].value > threshold=0.02372477948665619
node_1031: feature_name=GO:0002763 feature_id[271].value <= threshold=6.462319850921631
node_1032: feature_name=GO:0045840 feature_id[697].value <= threshold=10.30175256729126
node_1033: feature_name=GO:0051402 feature_id[568].value > threshold=1.2523645758628845
node_1041: feature_name=GO:0050852 feature_id[154].value > threshold=0.0008433434413745999
node_1043: feature_name=GO:0031667 feature_id[648].value <= threshold=1.5418170094490051
node_1044: feature_name=GO:0030101 feature_id[596].value <= threshold=0.865232065320015
Class: positive genes

Rules_546 passed counts:1
node_0: feature_name=GO:0042113 feature_id[0].value <= threshold=13.408552169799805
node_1: feature_name=GO:0007568 feature_id[534].value > threshold=5.0313897132873535
node_913: feature_name=GO:0032763 feature_id[629].value <= threshold=0.31753237545490265
node_914: feature_name=GO:0097193 feature_id[167].value <= threshold=28.171168327331543
node_915: feature_name=GO:0002903 feature_id[10].value <= threshold=1.0534588098526
node_916: feature_name=GO:1904029 feature_id[208].value <= threshold=23.852136611938477
node_917: feature_name=GO:0050897 feature_id[164].value <= threshold=0.6870408356189728
node_918: feature_name=GO:0006139 feature_id[474].value > threshold=1.3052097624921544e-07
node_920: feature_name=GO:0002821 feature_id[588].value <= threshold=13.81072187423706
node_921: feature_name=GO:0006298 feature_id[621].value <= threshold=24.078600883483887
node_922: feature_name=GO:0003908 feature_id[11].value <= threshold=1.7776933312416077
node_923: feature_name=GO:0030098 feature_id[273].value > threshold=0.0079949083738029
node_927: feature_name=GO:0006808 feature_id[513].value <= threshold=4.34592080116272
node_928: feature_name=GO:0071887 feature_id[283].value <= threshold=10.353787899017334
node_929: feature_name=GO:0038001 feature_id[28].value <= threshold=3.9469382762908936

node_930: feature_name=GO:0042287 feature_id[20].value <= threshold=3.6209195852279663
node_931: feature_name=GO:0003968 feature_id[47].value <= threshold=2.004227638244629
node_932: feature_name=GO:0002698 feature_id[395].value <= threshold=18.51447582244873
node_933: feature_name=GO:0044710 feature_id[719].value <= threshold=179.7340316772461
node_934: feature_name=GO:0007568 feature_id[534].value > threshold=5.035318374633789
node_936: feature_name=GO:0006216 feature_id[504].value <= threshold=2.2605666518211365
node_937: feature_name=GO:0048569 feature_id[793].value <= threshold=5.340231895446777
node_938: feature_name=GO:0001777 feature_id[380].value <= threshold=4.100832939147949
node_939: feature_name=GO:0007600 feature_id[122].value <= threshold=171.13383102416992
node_940: feature_name=GO:0001779 feature_id[378].value <= threshold=6.234851121902466
node_941: feature_name=GO:0030291 feature_id[147].value <= threshold=10.8051118850708
node_942: feature_name=GO:0048534 feature_id[790].value > threshold=0.017984486185014248
node_944: feature_name=GO:0070245 feature_id[326].value <= threshold=3.1282339096069336
node_945: feature_name=GO:0009086 feature_id[547].value > threshold=0.2896959036588669
node_1017: feature_name=GO:0000302 feature_id[375].value > threshold=0.305715873837471
node_1019: feature_name=GO:0070141 feature_id[37].value <= threshold=4.857294321060181
node_1020: feature_name=GO:0009086 feature_id[547].value > threshold=0.2945319563150406
node_1024: feature_name=GO:0010332 feature_id[557].value <= threshold=15.092710971832275
node_1025: feature_name=GO:0002524 feature_id[72].value <= threshold=2.531021237373352
node_1026: feature_name=GO:0016032 feature_id[571].value <= threshold=60.173330307006836
node_1027: feature_name=GO:0002832 feature_id[491].value <= threshold=2.0699684023857117
node_1028: feature_name=GO:0008588 feature_id[26].value <= threshold=2.150238513946533
node_1029: feature_name=GO:0009615 feature_id[265].value > threshold=0.02372477948665619
node_1031: feature_name=GO:0002763 feature_id[271].value <= threshold=6.462319850921631
node_1032: feature_name=GO:0045840 feature_id[697].value <= threshold=10.30175256729126
node_1033: feature_name=GO:0051402 feature_id[568].value > threshold=1.2523645758628845
node_1041: feature_name=GO:0050852 feature_id[154].value <= threshold=0.0008433434413745999
Class: positive genes

Rules_547 passed counts:1
node_0: feature_name=GO:0042113 feature_id[0].value <= threshold=13.408552169799805
node_1: feature_name=GO:0007568 feature_id[534].value > threshold=5.0313897132873535
node_913: feature_name=GO:0032763 feature_id[629].value <= threshold=0.31753237545490265
node_914: feature_name=GO:0097193 feature_id[167].value <= threshold=28.171168327331543
node_915: feature_name=GO:0002903 feature_id[10].value <= threshold=1.0534588098526

node_916: feature_name=GO:1904029 feature_id[208].value <= threshold=23.852136611938477
node_917: feature_name=GO:0050897 feature_id[164].value <= threshold=0.6870408356189728
node_918: feature_name=GO:0006139 feature_id[474].value > threshold=1.3052097624921544e-07
node_920: feature_name=GO:0002821 feature_id[588].value <= threshold=13.81072187423706
node_921: feature_name=GO:0006298 feature_id[621].value <= threshold=24.078600883483887
node_922: feature_name=GO:0003908 feature_id[11].value <= threshold=1.7776933312416077
node_923: feature_name=GO:0030098 feature_id[273].value > threshold=0.0079949083738029
node_927: feature_name=GO:0006808 feature_id[513].value <= threshold=4.34592080116272
node_928: feature_name=GO:0071887 feature_id[283].value <= threshold=10.353787899017334
node_929: feature_name=GO:0038001 feature_id[28].value <= threshold=3.9469382762908936
node_930: feature_name=GO:0042287 feature_id[20].value <= threshold=3.6209195852279663
node_931: feature_name=GO:0003968 feature_id[47].value <= threshold=2.004227638244629
node_932: feature_name=GO:0002698 feature_id[395].value <= threshold=18.51447582244873
node_933: feature_name=GO:0044710 feature_id[719].value <= threshold=179.7340316772461
node_934: feature_name=GO:0007568 feature_id[534].value > threshold=5.035318374633789
node_936: feature_name=GO:0006216 feature_id[504].value <= threshold=2.2605666518211365
node_937: feature_name=GO:0048569 feature_id[793].value <= threshold=5.340231895446777
node_938: feature_name=GO:0001777 feature_id[380].value <= threshold=4.100832939147949
node_939: feature_name=GO:0007600 feature_id[122].value <= threshold=171.13383102416992
node_940: feature_name=GO:0001779 feature_id[378].value <= threshold=6.234851121902466
node_941: feature_name=GO:0030291 feature_id[147].value <= threshold=10.8051118850708
node_942: feature_name=GO:0048534 feature_id[790].value > threshold=0.017984486185014248
node_944: feature_name=GO:0070245 feature_id[326].value <= threshold=3.1282339096069336
node_945: feature_name=GO:0009086 feature_id[547].value > threshold=0.2896959036588669
node_1017: feature_name=GO:0000302 feature_id[375].value > threshold=0.305715873837471
node_1019: feature_name=GO:0070141 feature_id[37].value <= threshold=4.857294321060181
node_1020: feature_name=GO:0009086 feature_id[547].value > threshold=0.2945319563150406
node_1024: feature_name=GO:0010332 feature_id[557].value <= threshold=15.092710971832275
node_1025: feature_name=GO:0002524 feature_id[72].value <= threshold=2.531021237373352
node_1026: feature_name=GO:0016032 feature_id[571].value <= threshold=60.173330307006836
node_1027: feature_name=GO:0002832 feature_id[491].value <= threshold=2.0699684023857117
node_1028: feature_name=GO:0008588 feature_id[26].value <= threshold=2.150238513946533
node_1029: feature_name=GO:0009615 feature_id[265].value > threshold=0.02372477948665619
node_1031: feature_name=GO:0002763 feature_id[271].value <= threshold=6.462319850921631
node_1032: feature_name=GO:0045840 feature_id[697].value <= threshold=10.30175256729126

node_1033: feature_name=GO:0051402 feature_id[568].value <= threshold=1.2523645758628845
node_1034: feature_name=GO:0008625 feature_id[99].value <= threshold=1.086933195590973
node_1035: feature_name=GO:0009636 feature_id[551].value > threshold=11.690044641494751
Class: positive genes

Rules_548 passed counts:1
node_0: feature_name=GO:0042113 feature_id[0].value <= threshold=13.408552169799805
node_1: feature_name=GO:0007568 feature_id[534].value > threshold=5.0313897132873535
node_913: feature_name=GO:0032763 feature_id[629].value <= threshold=0.31753237545490265
node_914: feature_name=GO:0097193 feature_id[167].value <= threshold=28.171168327331543
node_915: feature_name=GO:0002903 feature_id[10].value <= threshold=1.0534588098526
node_916: feature_name=GO:1904029 feature_id[208].value <= threshold=23.852136611938477
node_917: feature_name=GO:0050897 feature_id[164].value <= threshold=0.6870408356189728
node_918: feature_name=GO:0006139 feature_id[474].value > threshold=1.3052097624921544e-07
node_920: feature_name=GO:0002821 feature_id[588].value <= threshold=13.81072187423706
node_921: feature_name=GO:0006298 feature_id[621].value <= threshold=24.078600883483887
node_922: feature_name=GO:0003908 feature_id[11].value <= threshold=1.7776933312416077
node_923: feature_name=GO:0030098 feature_id[273].value > threshold=0.0079949083738029
node_927: feature_name=GO:0006808 feature_id[513].value <= threshold=4.34592080116272
node_928: feature_name=GO:0071887 feature_id[283].value <= threshold=10.353787899017334
node_929: feature_name=GO:0038001 feature_id[28].value <= threshold=3.9469382762908936
node_930: feature_name=GO:0042287 feature_id[20].value <= threshold=3.6209195852279663
node_931: feature_name=GO:0003968 feature_id[47].value <= threshold=2.004227638244629
node_932: feature_name=GO:0002698 feature_id[395].value <= threshold=18.51447582244873
node_933: feature_name=GO:0044710 feature_id[719].value <= threshold=179.7340316772461
node_934: feature_name=GO:0007568 feature_id[534].value > threshold=5.035318374633789
node_936: feature_name=GO:0006216 feature_id[504].value <= threshold=2.2605666518211365
node_937: feature_name=GO:0048569 feature_id[793].value <= threshold=5.340231895446777
node_938: feature_name=GO:0001777 feature_id[380].value <= threshold=4.100832939147949
node_939: feature_name=GO:0007600 feature_id[122].value <= threshold=171.13383102416992
node_940: feature_name=GO:0001779 feature_id[378].value <= threshold=6.234851121902466
node_941: feature_name=GO:0030291 feature_id[147].value <= threshold=10.8051118850708
node_942: feature_name=GO:0048534 feature_id[790].value > threshold=0.017984486185014248
node_944: feature_name=GO:0070245 feature_id[326].value <= threshold=3.1282339096069336
node_945: feature_name=GO:0009086 feature_id[547].value > threshold=0.2896959036588669

node_1017: feature_name=GO:0000302 feature_id[375].value > threshold=0.305715873837471
node_1019: feature_name=GO:0070141 feature_id[37].value <= threshold=4.857294321060181
node_1020: feature_name=GO:0009086 feature_id[547].value > threshold=0.2945319563150406
node_1024: feature_name=GO:0010332 feature_id[557].value <= threshold=15.092710971832275
node_1025: feature_name=GO:0002524 feature_id[72].value <= threshold=2.531021237373352
node_1026: feature_name=GO:0016032 feature_id[571].value <= threshold=60.173330307006836
node_1027: feature_name=GO:0002832 feature_id[491].value <= threshold=2.0699684023857117
node_1028: feature_name=GO:0008588 feature_id[26].value <= threshold=2.150238513946533
node_1029: feature_name=GO:0009615 feature_id[265].value <= threshold=0.02372477948665619
Class: positive genes

Rules_549 passed counts:1
node_0: feature_name=GO:0042113 feature_id[0].value <= threshold=13.408552169799805
node_1: feature_name=GO:0007568 feature_id[534].value > threshold=5.0313897132873535
node_913: feature_name=GO:0032763 feature_id[629].value <= threshold=0.31753237545490265
node_914: feature_name=GO:0097193 feature_id[167].value <= threshold=28.171168327331543
node_915: feature_name=GO:0002903 feature_id[10].value <= threshold=1.0534588098526
node_916: feature_name=GO:1904029 feature_id[208].value <= threshold=23.852136611938477
node_917: feature_name=GO:0050897 feature_id[164].value <= threshold=0.6870408356189728
node_918: feature_name=GO:0006139 feature_id[474].value > threshold=1.3052097624921544e-07
node_920: feature_name=GO:0002821 feature_id[588].value <= threshold=13.81072187423706
node_921: feature_name=GO:0006298 feature_id[621].value <= threshold=24.078600883483887
node_922: feature_name=GO:0003908 feature_id[11].value <= threshold=1.7776933312416077
node_923: feature_name=GO:0030098 feature_id[273].value > threshold=0.0079949083738029
node_927: feature_name=GO:0006808 feature_id[513].value <= threshold=4.34592080116272
node_928: feature_name=GO:0071887 feature_id[283].value <= threshold=10.353787899017334
node_929: feature_name=GO:0038001 feature_id[28].value <= threshold=3.9469382762908936
node_930: feature_name=GO:0042287 feature_id[20].value <= threshold=3.6209195852279663
node_931: feature_name=GO:0003968 feature_id[47].value <= threshold=2.004227638244629
node_932: feature_name=GO:0002698 feature_id[395].value <= threshold=18.51447582244873
node_933: feature_name=GO:0044710 feature_id[719].value <= threshold=179.7340316772461
node_934: feature_name=GO:0007568 feature_id[534].value > threshold=5.035318374633789
node_936: feature_name=GO:0006216 feature_id[504].value <= threshold=2.2605666518211365
node_937: feature_name=GO:0048569 feature_id[793].value <= threshold=5.340231895446777
node_938: feature_name=GO:0001777 feature_id[380].value <= threshold=4.100832939147949

node_939: feature_name=GO:0007600 feature_id[122].value <= threshold=171.13383102416992
node_940: feature_name=GO:0001779 feature_id[378].value <= threshold=6.234851121902466
node_941: feature_name=GO:0030291 feature_id[147].value <= threshold=10.8051118850708
node_942: feature_name=GO:0048534 feature_id[790].value > threshold=0.017984486185014248
node_944: feature_name=GO:0070245 feature_id[326].value <= threshold=3.1282339096069336
node_945: feature_name=GO:0009086 feature_id[547].value > threshold=0.2896959036588669
node_1017: feature_name=GO:0000302 feature_id[375].value > threshold=0.305715873837471
node_1019: feature_name=GO:0070141 feature_id[37].value <= threshold=4.857294321060181
node_1020: feature_name=GO:0009086 feature_id[547].value <= threshold=0.2945319563150406
node_1021: feature_name=GO:0003684 feature_id[653].value <= threshold=0.8139447569847107
Class: negative genes

Rules_550 passed counts:1
node_0: feature_name=GO:0042113 feature_id[0].value <= threshold=13.408552169799805
node_1: feature_name=GO:0007568 feature_id[534].value > threshold=5.0313897132873535
node_913: feature_name=GO:0032763 feature_id[629].value <= threshold=0.31753237545490265
node_914: feature_name=GO:0097193 feature_id[167].value <= threshold=28.171168327331543
node_915: feature_name=GO:0002903 feature_id[10].value <= threshold=1.0534588098526
node_916: feature_name=GO:1904029 feature_id[208].value <= threshold=23.852136611938477
node_917: feature_name=GO:0050897 feature_id[164].value <= threshold=0.6870408356189728
node_918: feature_name=GO:0006139 feature_id[474].value > threshold=1.3052097624921544e-07
node_920: feature_name=GO:0002821 feature_id[588].value <= threshold=13.81072187423706
node_921: feature_name=GO:0006298 feature_id[621].value <= threshold=24.078600883483887
node_922: feature_name=GO:0003908 feature_id[11].value <= threshold=1.7776933312416077
node_923: feature_name=GO:0030098 feature_id[273].value > threshold=0.0079949083738029
node_927: feature_name=GO:0006808 feature_id[513].value <= threshold=4.34592080116272
node_928: feature_name=GO:0071887 feature_id[283].value <= threshold=10.353787899017334
node_929: feature_name=GO:0038001 feature_id[28].value <= threshold=3.9469382762908936
node_930: feature_name=GO:0042287 feature_id[20].value <= threshold=3.6209195852279663
node_931: feature_name=GO:0003968 feature_id[47].value <= threshold=2.004227638244629
node_932: feature_name=GO:0002698 feature_id[395].value <= threshold=18.51447582244873
node_933: feature_name=GO:0044710 feature_id[719].value <= threshold=179.7340316772461
node_934: feature_name=GO:0007568 feature_id[534].value > threshold=5.035318374633789
node_936: feature_name=GO:0006216 feature_id[504].value <= threshold=2.2605666518211365
node_937: feature_name=GO:0048569 feature_id[793].value <= threshold=5.340231895446777

node_938: feature_name=GO:0001777 feature_id[380].value <= threshold=4.100832939147949
node_939: feature_name=GO:0007600 feature_id[122].value <= threshold=171.13383102416992
node_940: feature_name=GO:0001779 feature_id[378].value <= threshold=6.234851121902466
node_941: feature_name=GO:0030291 feature_id[147].value <= threshold=10.8051118850708
node_942: feature_name=GO:0048534 feature_id[790].value > threshold=0.017984486185014248
node_944: feature_name=GO:0070245 feature_id[326].value <= threshold=3.1282339096069336
node_945: feature_name=GO:0009086 feature_id[547].value <= threshold=0.2896959036588669
node_946: feature_name=GO:0001889 feature_id[387].value > threshold=17.643128395080566
Class: positive genes

Rules_551 passed counts:1
node_0: feature_name=GO:0042113 feature_id[0].value <= threshold=13.408552169799805
node_1: feature_name=GO:0007568 feature_id[534].value > threshold=5.0313897132873535
node_913: feature_name=GO:0032763 feature_id[629].value <= threshold=0.31753237545490265
node_914: feature_name=GO:0097193 feature_id[167].value <= threshold=28.171168327331543
node_915: feature_name=GO:0002903 feature_id[10].value <= threshold=1.0534588098526
node_916: feature_name=GO:1904029 feature_id[208].value <= threshold=23.852136611938477
node_917: feature_name=GO:0050897 feature_id[164].value <= threshold=0.6870408356189728
node_918: feature_name=GO:0006139 feature_id[474].value > threshold=1.3052097624921544e-07
node_920: feature_name=GO:0002821 feature_id[588].value <= threshold=13.81072187423706
node_921: feature_name=GO:0006298 feature_id[621].value <= threshold=24.078600883483887
node_922: feature_name=GO:0003908 feature_id[11].value <= threshold=1.7776933312416077
node_923: feature_name=GO:0030098 feature_id[273].value > threshold=0.0079949083738029
node_927: feature_name=GO:0006808 feature_id[513].value <= threshold=4.34592080116272
node_928: feature_name=GO:0071887 feature_id[283].value <= threshold=10.353787899017334
node_929: feature_name=GO:0038001 feature_id[28].value <= threshold=3.9469382762908936
node_930: feature_name=GO:0042287 feature_id[20].value <= threshold=3.6209195852279663
node_931: feature_name=GO:0003968 feature_id[47].value <= threshold=2.004227638244629
node_932: feature_name=GO:0002698 feature_id[395].value <= threshold=18.51447582244873
node_933: feature_name=GO:0044710 feature_id[719].value <= threshold=179.7340316772461
node_934: feature_name=GO:0007568 feature_id[534].value > threshold=5.035318374633789
node_936: feature_name=GO:0006216 feature_id[504].value <= threshold=2.2605666518211365
node_937: feature_name=GO:0048569 feature_id[793].value <= threshold=5.340231895446777
node_938: feature_name=GO:0001777 feature_id[380].value <= threshold=4.100832939147949
node_939: feature_name=GO:0007600 feature_id[122].value <= threshold=171.13383102416992

node_940: feature_name=GO:0001779 feature_id[378].value <= threshold=6.234851121902466
node_941: feature_name=GO:0030291 feature_id[147].value <= threshold=10.8051118850708
node_942: feature_name=GO:0048534 feature_id[790].value > threshold=0.017984486185014248
node_944: feature_name=GO:0070245 feature_id[326].value <= threshold=3.1282339096069336
node_945: feature_name=GO:0009086 feature_id[547].value <= threshold=0.2896959036588669
node_946: feature_name=GO:0001889 feature_id[387].value <= threshold=17.643128395080566
node_947: feature_name=GO:0048144 feature_id[192].value > threshold=0.20887330174446106
node_951: feature_name=GO:0023026 feature_id[69].value <= threshold=3.403424024581909
node_952: feature_name=GO:0048147 feature_id[788].value <= threshold=4.188283443450928
node_953: feature_name=GO:0090116 feature_id[97].value <= threshold=4.617154359817505
node_954: feature_name=GO:0002863 feature_id[490].value > threshold=4.8968470096588135
node_1004: feature_name=GO:0002822 feature_id[405].value > threshold=10.4080171585083
Class: negative genes

Rules_552 passed counts:1
node_0: feature_name=GO:0042113 feature_id[0].value <= threshold=13.408552169799805
node_1: feature_name=GO:0007568 feature_id[534].value > threshold=5.0313897132873535
node_913: feature_name=GO:0032763 feature_id[629].value <= threshold=0.31753237545490265
node_914: feature_name=GO:0097193 feature_id[167].value <= threshold=28.171168327331543
node_915: feature_name=GO:0002903 feature_id[10].value <= threshold=1.0534588098526
node_916: feature_name=GO:1904029 feature_id[208].value <= threshold=23.852136611938477
node_917: feature_name=GO:0050897 feature_id[164].value <= threshold=0.6870408356189728
node_918: feature_name=GO:0006139 feature_id[474].value > threshold=1.3052097624921544e-07
node_920: feature_name=GO:0002821 feature_id[588].value <= threshold=13.81072187423706
node_921: feature_name=GO:0006298 feature_id[621].value <= threshold=24.078600883483887
node_922: feature_name=GO:0003908 feature_id[11].value <= threshold=1.7776933312416077
node_923: feature_name=GO:0030098 feature_id[273].value > threshold=0.0079949083738029
node_927: feature_name=GO:0006808 feature_id[513].value <= threshold=4.34592080116272
node_928: feature_name=GO:0071887 feature_id[283].value <= threshold=10.353787899017334
node_929: feature_name=GO:0038001 feature_id[28].value <= threshold=3.9469382762908936
node_930: feature_name=GO:0042287 feature_id[20].value <= threshold=3.6209195852279663
node_931: feature_name=GO:0003968 feature_id[47].value <= threshold=2.004227638244629
node_932: feature_name=GO:0002698 feature_id[395].value <= threshold=18.51447582244873
node_933: feature_name=GO:0044710 feature_id[719].value <= threshold=179.7340316772461
node_934: feature_name=GO:0007568 feature_id[534].value > threshold=5.035318374633789

node_936: feature_name=GO:0006216 feature_id[504].value <= threshold=2.2605666518211365
node_937: feature_name=GO:0048569 feature_id[793].value <= threshold=5.340231895446777
node_938: feature_name=GO:0001777 feature_id[380].value <= threshold=4.100832939147949
node_939: feature_name=GO:0007600 feature_id[122].value <= threshold=171.13383102416992
node_940: feature_name=GO:0001779 feature_id[378].value <= threshold=6.234851121902466
node_941: feature_name=GO:0030291 feature_id[147].value <= threshold=10.8051118850708
node_942: feature_name=GO:0048534 feature_id[790].value > threshold=0.017984486185014248
node_944: feature_name=GO:0070245 feature_id[326].value <= threshold=3.1282339096069336
node_945: feature_name=GO:0009086 feature_id[547].value <= threshold=0.2896959036588669
node_946: feature_name=GO:0001889 feature_id[387].value <= threshold=17.643128395080566
node_947: feature_name=GO:0048144 feature_id[192].value > threshold=0.20887330174446106
node_951: feature_name=GO:0023026 feature_id[69].value <= threshold=3.403424024581909
node_952: feature_name=GO:0048147 feature_id[788].value <= threshold=4.188283443450928
node_953: feature_name=GO:0090116 feature_id[97].value <= threshold=4.617154359817505
node_954: feature_name=GO:0002863 feature_id[490].value > threshold=4.8968470096588135
node_1004: feature_name=GO:0002822 feature_id[405].value <= threshold=10.4080171585083
Class: positive genes

Rules_553 passed counts:1
node_0: feature_name=GO:0042113 feature_id[0].value <= threshold=13.408552169799805
node_1: feature_name=GO:0007568 feature_id[534].value > threshold=5.0313897132873535
node_913: feature_name=GO:0032763 feature_id[629].value <= threshold=0.31753237545490265
node_914: feature_name=GO:0097193 feature_id[167].value <= threshold=28.171168327331543
node_915: feature_name=GO:0002903 feature_id[10].value <= threshold=1.0534588098526
node_916: feature_name=GO:1904029 feature_id[208].value <= threshold=23.852136611938477
node_917: feature_name=GO:0050897 feature_id[164].value <= threshold=0.6870408356189728
node_918: feature_name=GO:0006139 feature_id[474].value > threshold=1.3052097624921544e-07
node_920: feature_name=GO:0002821 feature_id[588].value <= threshold=13.81072187423706
node_921: feature_name=GO:0006298 feature_id[621].value <= threshold=24.078600883483887
node_922: feature_name=GO:0003908 feature_id[11].value <= threshold=1.7776933312416077
node_923: feature_name=GO:0030098 feature_id[273].value > threshold=0.0079949083738029
node_927: feature_name=GO:0006808 feature_id[513].value <= threshold=4.34592080116272
node_928: feature_name=GO:0071887 feature_id[283].value <= threshold=10.353787899017334
node_929: feature_name=GO:0038001 feature_id[28].value <= threshold=3.9469382762908936
node_930: feature_name=GO:0042287 feature_id[20].value <= threshold=3.6209195852279663

node_931: feature_name=GO:0003968 feature_id[47].value <= threshold=2.004227638244629
node_932: feature_name=GO:0002698 feature_id[395].value <= threshold=18.51447582244873
node_933: feature_name=GO:0044710 feature_id[719].value <= threshold=179.7340316772461
node_934: feature_name=GO:0007568 feature_id[534].value > threshold=5.035318374633789
node_936: feature_name=GO:0006216 feature_id[504].value <= threshold=2.2605666518211365
node_937: feature_name=GO:0048569 feature_id[793].value <= threshold=5.340231895446777
node_938: feature_name=GO:0001777 feature_id[380].value <= threshold=4.100832939147949
node_939: feature_name=GO:0007600 feature_id[122].value <= threshold=171.13383102416992
node_940: feature_name=GO:0001779 feature_id[378].value <= threshold=6.234851121902466
node_941: feature_name=GO:0030291 feature_id[147].value <= threshold=10.8051118850708
node_942: feature_name=GO:0048534 feature_id[790].value > threshold=0.017984486185014248
node_944: feature_name=GO:0070245 feature_id[326].value <= threshold=3.1282339096069336
node_945: feature_name=GO:0009086 feature_id[547].value <= threshold=0.2896959036588669
node_946: feature_name=GO:0001889 feature_id[387].value <= threshold=17.643128395080566
node_947: feature_name=GO:0048144 feature_id[192].value > threshold=0.20887330174446106
node_951: feature_name=GO:0023026 feature_id[69].value <= threshold=3.403424024581909
node_952: feature_name=GO:0048147 feature_id[788].value <= threshold=4.188283443450928
node_953: feature_name=GO:0090116 feature_id[97].value <= threshold=4.617154359817505
node_954: feature_name=GO:0002863 feature_id[490].value <= threshold=4.8968470096588135
node_955: feature_name=GO:0002524 feature_id[72].value > threshold=3.035340189933777
node_1001: feature_name=hsa05144 feature_id[751].value > threshold=16.534432888031006
Class: negative genes

Rules_554 passed counts:1
node_0: feature_name=GO:0042113 feature_id[0].value <= threshold=13.408552169799805
node_1: feature_name=GO:0007568 feature_id[534].value > threshold=5.0313897132873535
node_913: feature_name=GO:0032763 feature_id[629].value <= threshold=0.31753237545490265
node_914: feature_name=GO:0097193 feature_id[167].value <= threshold=28.171168327331543
node_915: feature_name=GO:0002903 feature_id[10].value <= threshold=1.0534588098526
node_916: feature_name=GO:1904029 feature_id[208].value <= threshold=23.852136611938477
node_917: feature_name=GO:0050897 feature_id[164].value <= threshold=0.6870408356189728
node_918: feature_name=GO:0006139 feature_id[474].value > threshold=1.3052097624921544e-07
node_920: feature_name=GO:0002821 feature_id[588].value <= threshold=13.81072187423706
node_921: feature_name=GO:0006298 feature_id[621].value <= threshold=24.078600883483887
node_922: feature_name=GO:0003908 feature_id[11].value <= threshold=1.7776933312416077

node_923: feature_name=GO:0030098 feature_id[273].value > threshold=0.0079949083738029
node_927: feature_name=GO:0006808 feature_id[513].value <= threshold=4.34592080116272
node_928: feature_name=GO:0071887 feature_id[283].value <= threshold=10.353787899017334
node_929: feature_name=GO:0038001 feature_id[28].value <= threshold=3.9469382762908936
node_930: feature_name=GO:0042287 feature_id[20].value <= threshold=3.6209195852279663
node_931: feature_name=GO:0003968 feature_id[47].value <= threshold=2.004227638244629
node_932: feature_name=GO:0002698 feature_id[395].value <= threshold=18.51447582244873
node_933: feature_name=GO:0044710 feature_id[719].value <= threshold=179.7340316772461
node_934: feature_name=GO:0007568 feature_id[534].value > threshold=5.035318374633789
node_936: feature_name=GO:0006216 feature_id[504].value <= threshold=2.2605666518211365
node_937: feature_name=GO:0048569 feature_id[793].value <= threshold=5.340231895446777
node_938: feature_name=GO:0001777 feature_id[380].value <= threshold=4.100832939147949
node_939: feature_name=GO:0007600 feature_id[122].value <= threshold=171.13383102416992
node_940: feature_name=GO:0001779 feature_id[378].value <= threshold=6.234851121902466
node_941: feature_name=GO:0030291 feature_id[147].value <= threshold=10.8051118850708
node_942: feature_name=GO:0048534 feature_id[790].value > threshold=0.017984486185014248
node_944: feature_name=GO:0070245 feature_id[326].value <= threshold=3.1282339096069336
node_945: feature_name=GO:0009086 feature_id[547].value <= threshold=0.2896959036588669
node_946: feature_name=GO:0001889 feature_id[387].value <= threshold=17.643128395080566
node_947: feature_name=GO:0048144 feature_id[192].value > threshold=0.20887330174446106
node_951: feature_name=GO:0023026 feature_id[69].value <= threshold=3.403424024581909
node_952: feature_name=GO:0048147 feature_id[788].value <= threshold=4.188283443450928
node_953: feature_name=GO:0090116 feature_id[97].value <= threshold=4.617154359817505
node_954: feature_name=GO:0002863 feature_id[490].value <= threshold=4.8968470096588135
node_955: feature_name=GO:0002524 feature_id[72].value > threshold=3.035340189933777
node_1001: feature_name=hsa05144 feature_id[751].value <= threshold=16.534432888031006
Class: positive genes

Rules_555 passed counts:1
node_0: feature_name=GO:0042113 feature_id[0].value <= threshold=13.408552169799805
node_1: feature_name=GO:0007568 feature_id[534].value > threshold=5.0313897132873535
node_913: feature_name=GO:0032763 feature_id[629].value <= threshold=0.31753237545490265
node_914: feature_name=GO:0097193 feature_id[167].value <= threshold=28.171168327331543
node_915: feature_name=GO:0002903 feature_id[10].value <= threshold=1.0534588098526
node_916: feature_name=GO:1904029 feature_id[208].value <= threshold=23.852136611938477

node_917: feature_name=GO:0050897 feature_id[164].value <= threshold=0.6870408356189728
node_918: feature_name=GO:0006139 feature_id[474].value > threshold=1.3052097624921544e-07
node_920: feature_name=GO:0002821 feature_id[588].value <= threshold=13.81072187423706
node_921: feature_name=GO:0006298 feature_id[621].value <= threshold=24.078600883483887
node_922: feature_name=GO:0003908 feature_id[11].value <= threshold=1.7776933312416077
node_923: feature_name=GO:0030098 feature_id[273].value > threshold=0.0079949083738029
node_927: feature_name=GO:0006808 feature_id[513].value <= threshold=4.34592080116272
node_928: feature_name=GO:0071887 feature_id[283].value <= threshold=10.353787899017334
node_929: feature_name=GO:0038001 feature_id[28].value <= threshold=3.9469382762908936
node_930: feature_name=GO:0042287 feature_id[20].value <= threshold=3.6209195852279663
node_931: feature_name=GO:0003968 feature_id[47].value <= threshold=2.004227638244629
node_932: feature_name=GO:0002698 feature_id[395].value <= threshold=18.51447582244873
node_933: feature_name=GO:0044710 feature_id[719].value <= threshold=179.7340316772461
node_934: feature_name=GO:0007568 feature_id[534].value > threshold=5.035318374633789
node_936: feature_name=GO:0006216 feature_id[504].value <= threshold=2.2605666518211365
node_937: feature_name=GO:0048569 feature_id[793].value <= threshold=5.340231895446777
node_938: feature_name=GO:0001777 feature_id[380].value <= threshold=4.100832939147949
node_939: feature_name=GO:0007600 feature_id[122].value <= threshold=171.13383102416992
node_940: feature_name=GO:0001779 feature_id[378].value <= threshold=6.234851121902466
node_941: feature_name=GO:0030291 feature_id[147].value <= threshold=10.8051118850708
node_942: feature_name=GO:0048534 feature_id[790].value > threshold=0.017984486185014248
node_944: feature_name=GO:0070245 feature_id[326].value <= threshold=3.1282339096069336
node_945: feature_name=GO:0009086 feature_id[547].value <= threshold=0.2896959036588669
node_946: feature_name=GO:0001889 feature_id[387].value <= threshold=17.643128395080566
node_947: feature_name=GO:0048144 feature_id[192].value > threshold=0.20887330174446106
node_951: feature_name=GO:0023026 feature_id[69].value <= threshold=3.403424024581909
node_952: feature_name=GO:0048147 feature_id[788].value <= threshold=4.188283443450928
node_953: feature_name=GO:0090116 feature_id[97].value <= threshold=4.617154359817505
node_954: feature_name=GO:0002863 feature_id[490].value <= threshold=4.8968470096588135
node_955: feature_name=GO:0002524 feature_id[72].value <= threshold=3.035340189933777
node_956: feature_name=GO:0042130 feature_id[16].value > threshold=7.285413980484009
node_998: feature_name=GO:0002517 feature_id[88].value > threshold=2.6873340606689453
Class: negative genes

Rules_556 passed counts:1

node_0: feature_name=GO:0042113	feature_id[0].value <= threshold=13.408552169799805
node_1: feature_name=GO:0007568	feature_id[534].value > threshold=5.0313897132873535
node_913: feature_name=GO:0032763	feature_id[629].value <= threshold=0.31753237545490265
node_914: feature_name=GO:0097193	feature_id[167].value <= threshold=28.171168327331543
node_915: feature_name=GO:0002903	feature_id[10].value <= threshold=1.0534588098526
node_916: feature_name=GO:1904029	feature_id[208].value <= threshold=23.852136611938477
node_917: feature_name=GO:0050897	feature_id[164].value <= threshold=0.6870408356189728
node_918: feature_name=GO:0006139	feature_id[474].value > threshold=1.3052097624921544e-07
node_920: feature_name=GO:0002821	feature_id[588].value <= threshold=13.81072187423706
node_921: feature_name=GO:0006298	feature_id[621].value <= threshold=24.078600883483887
node_922: feature_name=GO:0003908	feature_id[11].value <= threshold=1.7776933312416077
node_923: feature_name=GO:0030098	feature_id[273].value > threshold=0.0079949083738029
node_927: feature_name=GO:0006808	feature_id[513].value <= threshold=4.34592080116272
node_928: feature_name=GO:0071887	feature_id[283].value <= threshold=10.353787899017334
node_929: feature_name=GO:0038001	feature_id[28].value <= threshold=3.9469382762908936
node_930: feature_name=GO:0042287	feature_id[20].value <= threshold=3.6209195852279663
node_931: feature_name=GO:0003968	feature_id[47].value <= threshold=2.004227638244629
node_932: feature_name=GO:0002698	feature_id[395].value <= threshold=18.51447582244873
node_933: feature_name=GO:0044710	feature_id[719].value <= threshold=179.7340316772461
node_934: feature_name=GO:0007568	feature_id[534].value > threshold=5.035318374633789
node_936: feature_name=GO:0006216	feature_id[504].value <= threshold=2.2605666518211365
node_937: feature_name=GO:0048569	feature_id[793].value <= threshold=5.340231895446777
node_938: feature_name=GO:0001777	feature_id[380].value <= threshold=4.100832939147949
node_939: feature_name=GO:0007600	feature_id[122].value <= threshold=171.13383102416992
node_940: feature_name=GO:0001779	feature_id[378].value <= threshold=6.234851121902466
node_941: feature_name=GO:0030291	feature_id[147].value <= threshold=10.8051118850708
node_942: feature_name=GO:0048534	feature_id[790].value > threshold=0.017984486185014248
node_944: feature_name=GO:0070245	feature_id[326].value <= threshold=3.1282339096069336
node_945: feature_name=GO:0009086	feature_id[547].value <= threshold=0.2896959036588669
node_946: feature_name=GO:0001889	feature_id[387].value <= threshold=17.643128395080566
node_947: feature_name=GO:0048144	feature_id[192].value > threshold=0.20887330174446106
node_951: feature_name=GO:0023026	feature_id[69].value <= threshold=3.403424024581909
node_952: feature_name=GO:0048147	feature_id[788].value <= threshold=4.188283443450928
node_953: feature_name=GO:0090116	feature_id[97].value <= threshold=4.617154359817505
node_954: feature_name=GO:0002863	feature_id[490].value <= threshold=4.8968470096588135

node_955: feature_name=GO:0002524 feature_id[72].value <= threshold=3.035340189933777
node_956: feature_name=GO:0042130 feature_id[16].value > threshold=7.285413980484009
node_998: feature_name=GO:0002517 feature_id[88].value <= threshold=2.6873340606689453
Class: positive genes

Rules_557 passed counts:1
node_0: feature_name=GO:0042113 feature_id[0].value <= threshold=13.408552169799805
node_1: feature_name=GO:0007568 feature_id[534].value > threshold=5.0313897132873535
node_913: feature_name=GO:0032763 feature_id[629].value <= threshold=0.31753237545490265
node_914: feature_name=GO:0097193 feature_id[167].value <= threshold=28.171168327331543
node_915: feature_name=GO:0002903 feature_id[10].value <= threshold=1.0534588098526
node_916: feature_name=GO:1904029 feature_id[208].value <= threshold=23.852136611938477
node_917: feature_name=GO:0050897 feature_id[164].value <= threshold=0.6870408356189728
node_918: feature_name=GO:0006139 feature_id[474].value > threshold=1.3052097624921544e-07
node_920: feature_name=GO:0002821 feature_id[588].value <= threshold=13.81072187423706
node_921: feature_name=GO:0006298 feature_id[621].value <= threshold=24.078600883483887
node_922: feature_name=GO:0003908 feature_id[11].value <= threshold=1.7776933312416077
node_923: feature_name=GO:0030098 feature_id[273].value > threshold=0.0079949083738029
node_927: feature_name=GO:0006808 feature_id[513].value <= threshold=4.34592080116272
node_928: feature_name=GO:0071887 feature_id[283].value <= threshold=10.353787899017334
node_929: feature_name=GO:0038001 feature_id[28].value <= threshold=3.9469382762908936
node_930: feature_name=GO:0042287 feature_id[20].value <= threshold=3.6209195852279663
node_931: feature_name=GO:0003968 feature_id[47].value <= threshold=2.004227638244629
node_932: feature_name=GO:0002698 feature_id[395].value <= threshold=18.51447582244873
node_933: feature_name=GO:0044710 feature_id[719].value <= threshold=179.7340316772461
node_934: feature_name=GO:0007568 feature_id[534].value > threshold=5.035318374633789
node_936: feature_name=GO:0006216 feature_id[504].value <= threshold=2.2605666518211365
node_937: feature_name=GO:0048569 feature_id[793].value <= threshold=5.340231895446777
node_938: feature_name=GO:0001777 feature_id[380].value <= threshold=4.100832939147949
node_939: feature_name=GO:0007600 feature_id[122].value <= threshold=171.13383102416992
node_940: feature_name=GO:0001779 feature_id[378].value <= threshold=6.234851121902466
node_941: feature_name=GO:0030291 feature_id[147].value <= threshold=10.8051118850708
node_942: feature_name=GO:0048534 feature_id[790].value > threshold=0.017984486185014248
node_944: feature_name=GO:0070245 feature_id[326].value <= threshold=3.1282339096069336
node_945: feature_name=GO:0009086 feature_id[547].value <= threshold=0.2896959036588669

node_946: feature_name=GO:0001889 feature_id[387].value <= threshold=17.643128395080566
node_947: feature_name=GO:0048144 feature_id[192].value > threshold=0.20887330174446106
node_951: feature_name=GO:0023026 feature_id[69].value <= threshold=3.403424024581909
node_952: feature_name=GO:0048147 feature_id[788].value <= threshold=4.188283443450928
node_953: feature_name=GO:0090116 feature_id[97].value <= threshold=4.617154359817505
node_954: feature_name=GO:0002863 feature_id[490].value <= threshold=4.8968470096588135
node_955: feature_name=GO:0002524 feature_id[72].value <= threshold=3.035340189933777
node_956: feature_name=GO:0042130 feature_id[16].value <= threshold=7.285413980484009
node_957: feature_name=GO:0071456 feature_id[500].value > threshold=21.212870597839355
node_995: feature_name=GO:0042130 feature_id[16].value > threshold=0.17710351943969727
Class: negative genes

Rules_558 passed counts:1
node_0: feature_name=GO:0042113 feature_id[0].value <= threshold=13.408552169799805
node_1: feature_name=GO:0007568 feature_id[534].value > threshold=5.0313897132873535
node_913: feature_name=GO:0032763 feature_id[629].value <= threshold=0.31753237545490265
node_914: feature_name=GO:0097193 feature_id[167].value <= threshold=28.171168327331543
node_915: feature_name=GO:0002903 feature_id[10].value <= threshold=1.0534588098526
node_916: feature_name=GO:1904029 feature_id[208].value <= threshold=23.852136611938477
node_917: feature_name=GO:0050897 feature_id[164].value <= threshold=0.6870408356189728
node_918: feature_name=GO:0006139 feature_id[474].value > threshold=1.3052097624921544e-07
node_920: feature_name=GO:0002821 feature_id[588].value <= threshold=13.81072187423706
node_921: feature_name=GO:0006298 feature_id[621].value <= threshold=24.078600883483887
node_922: feature_name=GO:0003908 feature_id[11].value <= threshold=1.7776933312416077
node_923: feature_name=GO:0030098 feature_id[273].value > threshold=0.0079949083738029
node_927: feature_name=GO:0006808 feature_id[513].value <= threshold=4.34592080116272
node_928: feature_name=GO:0071887 feature_id[283].value <= threshold=10.353787899017334
node_929: feature_name=GO:0038001 feature_id[28].value <= threshold=3.9469382762908936
node_930: feature_name=GO:0042287 feature_id[20].value <= threshold=3.6209195852279663
node_931: feature_name=GO:0003968 feature_id[47].value <= threshold=2.004227638244629
node_932: feature_name=GO:0002698 feature_id[395].value <= threshold=18.51447582244873
node_933: feature_name=GO:0044710 feature_id[719].value <= threshold=179.7340316772461
node_934: feature_name=GO:0007568 feature_id[534].value > threshold=5.035318374633789
node_936: feature_name=GO:0006216 feature_id[504].value <= threshold=2.2605666518211365
node_937: feature_name=GO:0048569 feature_id[793].value <= threshold=5.340231895446777

node_938: feature_name=GO:0001777 feature_id[380].value <= threshold=4.100832939147949
node_939: feature_name=GO:0007600 feature_id[122].value <= threshold=171.13383102416992
node_940: feature_name=GO:0001779 feature_id[378].value <= threshold=6.234851121902466
node_941: feature_name=GO:0030291 feature_id[147].value <= threshold=10.8051118850708
node_942: feature_name=GO:0048534 feature_id[790].value > threshold=0.017984486185014248
node_944: feature_name=GO:0070245 feature_id[326].value <= threshold=3.1282339096069336
node_945: feature_name=GO:0009086 feature_id[547].value <= threshold=0.2896959036588669
node_946: feature_name=GO:0001889 feature_id[387].value <= threshold=17.643128395080566
node_947: feature_name=GO:0048144 feature_id[192].value > threshold=0.20887330174446106
node_951: feature_name=GO:0023026 feature_id[69].value <= threshold=3.403424024581909
node_952: feature_name=GO:0048147 feature_id[788].value <= threshold=4.188283443450928
node_953: feature_name=GO:0090116 feature_id[97].value <= threshold=4.617154359817505
node_954: feature_name=GO:0002863 feature_id[490].value <= threshold=4.8968470096588135
node_955: feature_name=GO:0002524 feature_id[72].value <= threshold=3.035340189933777
node_956: feature_name=GO:0042130 feature_id[16].value <= threshold=7.285413980484009
node_957: feature_name=GO:0071456 feature_id[500].value > threshold=21.212870597839355
node_995: feature_name=GO:0042130 feature_id[16].value <= threshold=0.17710351943969727
Class: positive genes

Rules_559 passed counts:1
node_0: feature_name=GO:0042113 feature_id[0].value <= threshold=13.408552169799805
node_1: feature_name=GO:0007568 feature_id[534].value > threshold=5.0313897132873535
node_913: feature_name=GO:0032763 feature_id[629].value <= threshold=0.31753237545490265
node_914: feature_name=GO:0097193 feature_id[167].value <= threshold=28.171168327331543
node_915: feature_name=GO:0002903 feature_id[10].value <= threshold=1.0534588098526
node_916: feature_name=GO:1904029 feature_id[208].value <= threshold=23.852136611938477
node_917: feature_name=GO:0050897 feature_id[164].value <= threshold=0.6870408356189728
node_918: feature_name=GO:0006139 feature_id[474].value > threshold=1.3052097624921544e-07
node_920: feature_name=GO:0002821 feature_id[588].value <= threshold=13.81072187423706
node_921: feature_name=GO:0006298 feature_id[621].value <= threshold=24.078600883483887
node_922: feature_name=GO:0003908 feature_id[11].value <= threshold=1.7776933312416077
node_923: feature_name=GO:0030098 feature_id[273].value > threshold=0.0079949083738029
node_927: feature_name=GO:0006808 feature_id[513].value <= threshold=4.34592080116272
node_928: feature_name=GO:0071887 feature_id[283].value <= threshold=10.353787899017334
node_929: feature_name=GO:0038001 feature_id[28].value <= threshold=3.9469382762908936

node_930: feature_name=GO:0042287 feature_id[20].value <= threshold=3.6209195852279663
node_931: feature_name=GO:0003968 feature_id[47].value <= threshold=2.004227638244629
node_932: feature_name=GO:0002698 feature_id[395].value <= threshold=18.51447582244873
node_933: feature_name=GO:0044710 feature_id[719].value <= threshold=179.7340316772461
node_934: feature_name=GO:0007568 feature_id[534].value > threshold=5.035318374633789
node_936: feature_name=GO:0006216 feature_id[504].value <= threshold=2.2605666518211365
node_937: feature_name=GO:0048569 feature_id[793].value <= threshold=5.340231895446777
node_938: feature_name=GO:0001777 feature_id[380].value <= threshold=4.100832939147949
node_939: feature_name=GO:0007600 feature_id[122].value <= threshold=171.13383102416992
node_940: feature_name=GO:0001779 feature_id[378].value <= threshold=6.234851121902466
node_941: feature_name=GO:0030291 feature_id[147].value <= threshold=10.8051118850708
node_942: feature_name=GO:0048534 feature_id[790].value > threshold=0.017984486185014248
node_944: feature_name=GO:0070245 feature_id[326].value <= threshold=3.1282339096069336
node_945: feature_name=GO:0009086 feature_id[547].value <= threshold=0.2896959036588669
node_946: feature_name=GO:0001889 feature_id[387].value <= threshold=17.643128395080566
node_947: feature_name=GO:0048144 feature_id[192].value > threshold=0.20887330174446106
node_951: feature_name=GO:0023026 feature_id[69].value <= threshold=3.403424024581909
node_952: feature_name=GO:0048147 feature_id[788].value <= threshold=4.188283443450928
node_953: feature_name=GO:0090116 feature_id[97].value <= threshold=4.617154359817505
node_954: feature_name=GO:0002863 feature_id[490].value <= threshold=4.8968470096588135
node_955: feature_name=GO:0002524 feature_id[72].value <= threshold=3.035340189933777
node_956: feature_name=GO:0042130 feature_id[16].value <= threshold=7.285413980484009
node_957: feature_name=GO:0071456 feature_id[500].value <= threshold=21.212870597839355
node_958: feature_name=GO:2001238 feature_id[27].value > threshold=7.849715709686279
node_992: feature_name=GO:0016265 feature_id[235].value > threshold=58.356693267822266
Class: negative genes

Rules_560 passed counts:1
node_0: feature_name=GO:0042113 feature_id[0].value <= threshold=13.408552169799805
node_1: feature_name=GO:0007568 feature_id[534].value > threshold=5.0313897132873535
node_913: feature_name=GO:0032763 feature_id[629].value <= threshold=0.31753237545490265
node_914: feature_name=GO:0097193 feature_id[167].value <= threshold=28.171168327331543
node_915: feature_name=GO:0002903 feature_id[10].value <= threshold=1.0534588098526
node_916: feature_name=GO:1904029 feature_id[208].value <= threshold=23.852136611938477
node_917: feature_name=GO:0050897 feature_id[164].value <= threshold=0.6870408356189728

node_918: feature_name=GO:0006139 feature_id[474].value > threshold=1.3052097624921544e-07
node_920: feature_name=GO:0002821 feature_id[588].value <= threshold=13.81072187423706
node_921: feature_name=GO:0006298 feature_id[621].value <= threshold=24.078600883483887
node_922: feature_name=GO:0003908 feature_id[11].value <= threshold=1.7776933312416077
node_923: feature_name=GO:0030098 feature_id[273].value > threshold=0.0079949083738029
node_927: feature_name=GO:0006808 feature_id[513].value <= threshold=4.34592080116272
node_928: feature_name=GO:0071887 feature_id[283].value <= threshold=10.353787899017334
node_929: feature_name=GO:0038001 feature_id[28].value <= threshold=3.9469382762908936
node_930: feature_name=GO:0042287 feature_id[20].value <= threshold=3.6209195852279663
node_931: feature_name=GO:0003968 feature_id[47].value <= threshold=2.004227638244629
node_932: feature_name=GO:0002698 feature_id[395].value <= threshold=18.51447582244873
node_933: feature_name=GO:0044710 feature_id[719].value <= threshold=179.7340316772461
node_934: feature_name=GO:0007568 feature_id[534].value > threshold=5.035318374633789
node_936: feature_name=GO:0006216 feature_id[504].value <= threshold=2.2605666518211365
node_937: feature_name=GO:0048569 feature_id[793].value <= threshold=5.340231895446777
node_938: feature_name=GO:0001777 feature_id[380].value <= threshold=4.100832939147949
node_939: feature_name=GO:0007600 feature_id[122].value <= threshold=171.13383102416992
node_940: feature_name=GO:0001779 feature_id[378].value <= threshold=6.234851121902466
node_941: feature_name=GO:0030291 feature_id[147].value <= threshold=10.8051118850708
node_942: feature_name=GO:0048534 feature_id[790].value > threshold=0.017984486185014248
node_944: feature_name=GO:0070245 feature_id[326].value <= threshold=3.1282339096069336
node_945: feature_name=GO:0009086 feature_id[547].value <= threshold=0.2896959036588669
node_946: feature_name=GO:0001889 feature_id[387].value <= threshold=17.643128395080566
node_947: feature_name=GO:0048144 feature_id[192].value > threshold=0.20887330174446106
node_951: feature_name=GO:0023026 feature_id[69].value <= threshold=3.403424024581909
node_952: feature_name=GO:0048147 feature_id[788].value <= threshold=4.188283443450928
node_953: feature_name=GO:0090116 feature_id[97].value <= threshold=4.617154359817505
node_954: feature_name=GO:0002863 feature_id[490].value <= threshold=4.8968470096588135
node_955: feature_name=GO:0002524 feature_id[72].value <= threshold=3.035340189933777
node_956: feature_name=GO:0042130 feature_id[16].value <= threshold=7.285413980484009
node_957: feature_name=GO:0071456 feature_id[500].value <= threshold=21.212870597839355
node_958: feature_name=GO:2001238 feature_id[27].value > threshold=7.849715709686279
node_992: feature_name=GO:0016265 feature_id[235].value <= threshold=58.356693267822266
Class: positive genes

Rules_561	passed counts:1
node_0: feature_name=GO:0042113	feature_id[0].value <= threshold=13.408552169799805
node_1: feature_name=GO:0007568	feature_id[534].value > threshold=5.0313897132873535
node_913: feature_name=GO:0032763	feature_id[629].value <= threshold=0.31753237545490265
node_914: feature_name=GO:0097193	feature_id[167].value <= threshold=28.171168327331543
node_915: feature_name=GO:0002903	feature_id[10].value <= threshold=1.0534588098526
node_916: feature_name=GO:1904029	feature_id[208].value <= threshold=23.852136611938477
node_917: feature_name=GO:0050897	feature_id[164].value <= threshold=0.6870408356189728
node_918: feature_name=GO:0006139	feature_id[474].value > threshold=1.3052097624921544e-07
node_920: feature_name=GO:0002821	feature_id[588].value <= threshold=13.81072187423706
node_921: feature_name=GO:0006298	feature_id[621].value <= threshold=24.078600883483887
node_922: feature_name=GO:0003908	feature_id[11].value <= threshold=1.7776933312416077
node_923: feature_name=GO:0030098	feature_id[273].value > threshold=0.0079949083738029
node_927: feature_name=GO:0006808	feature_id[513].value <= threshold=4.34592080116272
node_928: feature_name=GO:0071887	feature_id[283].value <= threshold=10.353787899017334
node_929: feature_name=GO:0038001	feature_id[28].value <= threshold=3.9469382762908936
node_930: feature_name=GO:0042287	feature_id[20].value <= threshold=3.6209195852279663
node_931: feature_name=GO:0003968	feature_id[47].value <= threshold=2.004227638244629
node_932: feature_name=GO:0002698	feature_id[395].value <= threshold=18.51447582244873
node_933: feature_name=GO:0044710	feature_id[719].value <= threshold=179.7340316772461
node_934: feature_name=GO:0007568	feature_id[534].value > threshold=5.035318374633789
node_936: feature_name=GO:0006216	feature_id[504].value <= threshold=2.2605666518211365
node_937: feature_name=GO:0048569	feature_id[793].value <= threshold=5.340231895446777
node_938: feature_name=GO:0001777	feature_id[380].value <= threshold=4.100832939147949
node_939: feature_name=GO:0007600	feature_id[122].value <= threshold=171.13383102416992
node_940: feature_name=GO:0001779	feature_id[378].value <= threshold=6.234851121902466
node_941: feature_name=GO:0030291	feature_id[147].value <= threshold=10.8051118850708
node_942: feature_name=GO:0048534	feature_id[790].value > threshold=0.017984486185014248
node_944: feature_name=GO:0070245	feature_id[326].value <= threshold=3.1282339096069336
node_945: feature_name=GO:0009086	feature_id[547].value <= threshold=0.2896959036588669
node_946: feature_name=GO:0001889	feature_id[387].value <= threshold=17.643128395080566
node_947: feature_name=GO:0048144	feature_id[192].value > threshold=0.20887330174446106
node_951: feature_name=GO:0023026	feature_id[69].value <= threshold=3.403424024581909
node_952: feature_name=GO:0048147	feature_id[788].value <= threshold=4.188283443450928
node_953: feature_name=GO:0090116	feature_id[97].value <= threshold=4.617154359817505

node_954: feature_name=GO:0002863 feature_id[490].value <= threshold=4.8968470096588135
node_955: feature_name=GO:0002524 feature_id[72].value <= threshold=3.035340189933777
node_956: feature_name=GO:0042130 feature_id[16].value <= threshold=7.285413980484009
node_957: feature_name=GO:0071456 feature_id[500].value <= threshold=21.212870597839355
node_958: feature_name=GO:2001238 feature_id[27].value <= threshold=7.849715709686279
node_959: feature_name=GO:0023030 feature_id[45].value > threshold=1.7420591711997986
node_989: feature_name=GO:0030183 feature_id[590].value > threshold=1.2184761017560959
Class: positive genes

Rules_562 passed counts:1
node_0: feature_name=GO:0042113 feature_id[0].value <= threshold=13.408552169799805
node_1: feature_name=GO:0007568 feature_id[534].value > threshold=5.0313897132873535
node_913: feature_name=GO:0032763 feature_id[629].value <= threshold=0.31753237545490265
node_914: feature_name=GO:0097193 feature_id[167].value <= threshold=28.171168327331543
node_915: feature_name=GO:0002903 feature_id[10].value <= threshold=1.0534588098526
node_916: feature_name=GO:1904029 feature_id[208].value <= threshold=23.852136611938477
node_917: feature_name=GO:0050897 feature_id[164].value <= threshold=0.6870408356189728
node_918: feature_name=GO:0006139 feature_id[474].value > threshold=1.3052097624921544e-07
node_920: feature_name=GO:0002821 feature_id[588].value <= threshold=13.81072187423706
node_921: feature_name=GO:0006298 feature_id[621].value <= threshold=24.078600883483887
node_922: feature_name=GO:0003908 feature_id[11].value <= threshold=1.7776933312416077
node_923: feature_name=GO:0030098 feature_id[273].value > threshold=0.0079949083738029
node_927: feature_name=GO:0006808 feature_id[513].value <= threshold=4.34592080116272
node_928: feature_name=GO:0071887 feature_id[283].value <= threshold=10.353787899017334
node_929: feature_name=GO:0038001 feature_id[28].value <= threshold=3.9469382762908936
node_930: feature_name=GO:0042287 feature_id[20].value <= threshold=3.6209195852279663
node_931: feature_name=GO:0003968 feature_id[47].value <= threshold=2.004227638244629
node_932: feature_name=GO:0002698 feature_id[395].value <= threshold=18.51447582244873
node_933: feature_name=GO:0044710 feature_id[719].value <= threshold=179.7340316772461
node_934: feature_name=GO:0007568 feature_id[534].value > threshold=5.035318374633789
node_936: feature_name=GO:0006216 feature_id[504].value <= threshold=2.2605666518211365
node_937: feature_name=GO:0048569 feature_id[793].value <= threshold=5.340231895446777
node_938: feature_name=GO:0001777 feature_id[380].value <= threshold=4.100832939147949
node_939: feature_name=GO:0007600 feature_id[122].value <= threshold=171.13383102416992
node_940: feature_name=GO:0001779 feature_id[378].value <= threshold=6.234851121902466

node_941: feature_name=GO:0030291	feature_id[147].value <= threshold=10.8051118850708
node_942: feature_name=GO:0048534	feature_id[790].value > threshold=0.017984486185014248
node_944: feature_name=GO:0070245	feature_id[326].value <= threshold=3.1282339096069336
node_945: feature_name=GO:0009086	feature_id[547].value <= threshold=0.2896959036588669
node_946: feature_name=GO:0001889	feature_id[387].value <= threshold=17.643128395080566
node_947: feature_name=GO:0048144	feature_id[192].value > threshold=0.20887330174446106
node_951: feature_name=GO:0023026	feature_id[69].value <= threshold=3.403424024581909
node_952: feature_name=GO:0048147	feature_id[788].value <= threshold=4.188283443450928
node_953: feature_name=GO:0090116	feature_id[97].value <= threshold=4.617154359817505
node_954: feature_name=GO:0002863	feature_id[490].value <= threshold=4.8968470096588135
node_955: feature_name=GO:0002524	feature_id[72].value <= threshold=3.035340189933777
node_956: feature_name=GO:0042130	feature_id[16].value <= threshold=7.285413980484009
node_957: feature_name=GO:0071456	feature_id[500].value <= threshold=21.212870597839355
node_958: feature_name=GO:2001238	feature_id[27].value <= threshold=7.849715709686279
node_959: feature_name=GO:0023030	feature_id[45].value > threshold=1.7420591711997986
node_989: feature_name=GO:0030183	feature_id[590].value <= threshold=1.2184761017560959

Class: negative genes

Rules_563	passed counts:1
node_0: feature_name=GO:0042113	feature_id[0].value <= threshold=13.408552169799805
node_1: feature_name=GO:0007568	feature_id[534].value > threshold=5.0313897132873535
node_913: feature_name=GO:0032763	feature_id[629].value <= threshold=0.31753237545490265
node_914: feature_name=GO:0097193	feature_id[167].value <= threshold=28.171168327331543
node_915: feature_name=GO:0002903	feature_id[10].value <= threshold=1.0534588098526
node_916: feature_name=GO:1904029	feature_id[208].value <= threshold=23.852136611938477
node_917: feature_name=GO:0050897	feature_id[164].value <= threshold=0.6870408356189728
node_918: feature_name=GO:0006139	feature_id[474].value > threshold=1.3052097624921544e-07
node_920: feature_name=GO:0002821	feature_id[588].value <= threshold=13.81072187423706
node_921: feature_name=GO:0006298	feature_id[621].value <= threshold=24.078600883483887
node_922: feature_name=GO:0003908	feature_id[11].value <= threshold=1.7776933312416077
node_923: feature_name=GO:0030098	feature_id[273].value > threshold=0.0079949083738029
node_927: feature_name=GO:0006808	feature_id[513].value <= threshold=4.34592080116272
node_928: feature_name=GO:0071887	feature_id[283].value <= threshold=10.353787899017334
node_929: feature_name=GO:0038001	feature_id[28].value <= threshold=3.9469382762908936
node_930: feature_name=GO:0042287	feature_id[20].value <= threshold=3.6209195852279663

node_931: feature_name=GO:0003968 feature_id[47].value <= threshold=2.004227638244629
node_932: feature_name=GO:0002698 feature_id[395].value <= threshold=18.51447582244873
node_933: feature_name=GO:0044710 feature_id[719].value <= threshold=179.7340316772461
node_934: feature_name=GO:0007568 feature_id[534].value > threshold=5.035318374633789
node_936: feature_name=GO:0006216 feature_id[504].value <= threshold=2.2605666518211365
node_937: feature_name=GO:0048569 feature_id[793].value <= threshold=5.340231895446777
node_938: feature_name=GO:0001777 feature_id[380].value <= threshold=4.100832939147949
node_939: feature_name=GO:0007600 feature_id[122].value <= threshold=171.13383102416992
node_940: feature_name=GO:0001779 feature_id[378].value <= threshold=6.234851121902466
node_941: feature_name=GO:0030291 feature_id[147].value <= threshold=10.8051118850708
node_942: feature_name=GO:0048534 feature_id[790].value > threshold=0.017984486185014248
node_944: feature_name=GO:0070245 feature_id[326].value <= threshold=3.1282339096069336
node_945: feature_name=GO:0009086 feature_id[547].value <= threshold=0.2896959036588669
node_946: feature_name=GO:0001889 feature_id[387].value <= threshold=17.643128395080566
node_947: feature_name=GO:0048144 feature_id[192].value > threshold=0.20887330174446106
node_951: feature_name=GO:0023026 feature_id[69].value <= threshold=3.403424024581909
node_952: feature_name=GO:0048147 feature_id[788].value <= threshold=4.188283443450928
node_953: feature_name=GO:0090116 feature_id[97].value <= threshold=4.617154359817505
node_954: feature_name=GO:0002863 feature_id[490].value <= threshold=4.8968470096588135
node_955: feature_name=GO:0002524 feature_id[72].value <= threshold=3.035340189933777
node_956: feature_name=GO:0042130 feature_id[16].value <= threshold=7.285413980484009
node_957: feature_name=GO:0071456 feature_id[500].value <= threshold=21.212870597839355
node_958: feature_name=GO:2001238 feature_id[27].value <= threshold=7.849715709686279
node_959: feature_name=GO:0023030 feature_id[45].value <= threshold=1.7420591711997986
node_960: feature_name=GO:0072593 feature_id[381].value <= threshold=22.993029594421387
node_961: feature_name=GO:0034101 feature_id[646].value > threshold=13.430044651031494
node_983: feature_name=GO:0045143 feature_id[732].value > threshold=0.021482162177562714
Class: positive genes

Rules_564 passed counts:1
node_0: feature_name=GO:0042113 feature_id[0].value <= threshold=13.408552169799805
node_1: feature_name=GO:0007568 feature_id[534].value > threshold=5.0313897132873535
node_913: feature_name=GO:0032763 feature_id[629].value <= threshold=0.31753237545490265
node_914: feature_name=GO:0097193 feature_id[167].value <= threshold=28.171168327331543
node_915: feature_name=GO:0002903 feature_id[10].value <= threshold=1.0534588098526

node_916: feature_name=GO:1904029 feature_id[208].value <= threshold=23.852136611938477
node_917: feature_name=GO:0050897 feature_id[164].value <= threshold=0.6870408356189728
node_918: feature_name=GO:0006139 feature_id[474].value > threshold=1.3052097624921544e-07
node_920: feature_name=GO:0002821 feature_id[588].value <= threshold=13.81072187423706
node_921: feature_name=GO:0006298 feature_id[621].value <= threshold=24.078600883483887
node_922: feature_name=GO:0003908 feature_id[11].value <= threshold=1.7776933312416077
node_923: feature_name=GO:0030098 feature_id[273].value > threshold=0.0079949083738029
node_927: feature_name=GO:0006808 feature_id[513].value <= threshold=4.34592080116272
node_928: feature_name=GO:0071887 feature_id[283].value <= threshold=10.353787899017334
node_929: feature_name=GO:0038001 feature_id[28].value <= threshold=3.9469382762908936
node_930: feature_name=GO:0042287 feature_id[20].value <= threshold=3.6209195852279663
node_931: feature_name=GO:0003968 feature_id[47].value <= threshold=2.004227638244629
node_932: feature_name=GO:0002698 feature_id[395].value <= threshold=18.51447582244873
node_933: feature_name=GO:0044710 feature_id[719].value <= threshold=179.7340316772461
node_934: feature_name=GO:0007568 feature_id[534].value > threshold=5.035318374633789
node_936: feature_name=GO:0006216 feature_id[504].value <= threshold=2.2605666518211365
node_937: feature_name=GO:0048569 feature_id[793].value <= threshold=5.340231895446777
node_938: feature_name=GO:0001777 feature_id[380].value <= threshold=4.100832939147949
node_939: feature_name=GO:0007600 feature_id[122].value <= threshold=171.13383102416992
node_940: feature_name=GO:0001779 feature_id[378].value <= threshold=6.234851121902466
node_941: feature_name=GO:0030291 feature_id[147].value <= threshold=10.8051118850708
node_942: feature_name=GO:0048534 feature_id[790].value > threshold=0.017984486185014248
node_944: feature_name=GO:0070245 feature_id[326].value <= threshold=3.1282339096069336
node_945: feature_name=GO:0009086 feature_id[547].value <= threshold=0.2896959036588669
node_946: feature_name=GO:0001889 feature_id[387].value <= threshold=17.643128395080566
node_947: feature_name=GO:0048144 feature_id[192].value > threshold=0.20887330174446106
node_951: feature_name=GO:0023026 feature_id[69].value <= threshold=3.403424024581909
node_952: feature_name=GO:0048147 feature_id[788].value <= threshold=4.188283443450928
node_953: feature_name=GO:0090116 feature_id[97].value <= threshold=4.617154359817505
node_954: feature_name=GO:0002863 feature_id[490].value <= threshold=4.8968470096588135
node_955: feature_name=GO:0002524 feature_id[72].value <= threshold=3.035340189933777
node_956: feature_name=GO:0042130 feature_id[16].value <= threshold=7.285413980484009
node_957: feature_name=GO:0071456 feature_id[500].value <= threshold=21.212870597839355
node_958: feature_name=GO:2001238 feature_id[27].value <= threshold=7.849715709686279
node_959: feature_name=GO:0023030 feature_id[45].value <= threshold=1.7420591711997986

node_960: feature_name=GO:0072593 feature_id[381].value <= threshold=22.993029594421387
node_961: feature_name=GO:0034101 feature_id[646].value <= threshold=13.430044651031494
node_962: feature_name=GO:0097153 feature_id[226].value > threshold=10.147814273834229
node_980: feature_name=GO:0071901 feature_id[407].value <= threshold=1.0248211920261383
Class: positive genes

Rules_565 passed counts:1
node_0: feature_name=GO:0042113 feature_id[0].value <= threshold=13.408552169799805
node_1: feature_name=GO:0007568 feature_id[534].value > threshold=5.0313897132873535
node_913: feature_name=GO:0032763 feature_id[629].value <= threshold=0.31753237545490265
node_914: feature_name=GO:0097193 feature_id[167].value <= threshold=28.171168327331543
node_915: feature_name=GO:0002903 feature_id[10].value <= threshold=1.0534588098526
node_916: feature_name=GO:1904029 feature_id[208].value <= threshold=23.852136611938477
node_917: feature_name=GO:0050897 feature_id[164].value <= threshold=0.6870408356189728
node_918: feature_name=GO:0006139 feature_id[474].value > threshold=1.3052097624921544e-07
node_920: feature_name=GO:0002821 feature_id[588].value <= threshold=13.81072187423706
node_921: feature_name=GO:0006298 feature_id[621].value <= threshold=24.078600883483887
node_922: feature_name=GO:0003908 feature_id[11].value <= threshold=1.7776933312416077
node_923: feature_name=GO:0030098 feature_id[273].value > threshold=0.0079949083738029
node_927: feature_name=GO:0006808 feature_id[513].value <= threshold=4.34592080116272
node_928: feature_name=GO:0071887 feature_id[283].value <= threshold=10.353787899017334
node_929: feature_name=GO:0038001 feature_id[28].value <= threshold=3.9469382762908936
node_930: feature_name=GO:0042287 feature_id[20].value <= threshold=3.6209195852279663
node_931: feature_name=GO:0003968 feature_id[47].value <= threshold=2.004227638244629
node_932: feature_name=GO:0002698 feature_id[395].value <= threshold=18.51447582244873
node_933: feature_name=GO:0044710 feature_id[719].value <= threshold=179.7340316772461
node_934: feature_name=GO:0007568 feature_id[534].value > threshold=5.035318374633789
node_936: feature_name=GO:0006216 feature_id[504].value <= threshold=2.2605666518211365
node_937: feature_name=GO:0048569 feature_id[793].value <= threshold=5.340231895446777
node_938: feature_name=GO:0001777 feature_id[380].value <= threshold=4.100832939147949
node_939: feature_name=GO:0007600 feature_id[122].value <= threshold=171.13383102416992
node_940: feature_name=GO:0001779 feature_id[378].value <= threshold=6.234851121902466
node_941: feature_name=GO:0030291 feature_id[147].value <= threshold=10.8051118850708
node_942: feature_name=GO:0048534 feature_id[790].value > threshold=0.017984486185014248
node_944: feature_name=GO:0070245 feature_id[326].value <= threshold=3.1282339096069336

node_945: feature_name=GO:0009086 feature_id[547].value <= threshold=0.2896959036588669
node_946: feature_name=GO:0001889 feature_id[387].value <= threshold=17.643128395080566
node_947: feature_name=GO:0048144 feature_id[192].value > threshold=0.20887330174446106
node_951: feature_name=GO:0023026 feature_id[69].value <= threshold=3.403424024581909
node_952: feature_name=GO:0048147 feature_id[788].value <= threshold=4.188283443450928
node_953: feature_name=GO:0090116 feature_id[97].value <= threshold=4.617154359817505
node_954: feature_name=GO:0002863 feature_id[490].value <= threshold=4.8968470096588135
node_955: feature_name=GO:0002524 feature_id[72].value <= threshold=3.035340189933777
node_956: feature_name=GO:0042130 feature_id[16].value <= threshold=7.285413980484009
node_957: feature_name=GO:0071456 feature_id[500].value <= threshold=21.212870597839355
node_958: feature_name=GO:2001238 feature_id[27].value <= threshold=7.849715709686279
node_959: feature_name=GO:0023030 feature_id[45].value <= threshold=1.7420591711997986
node_960: feature_name=GO:0072593 feature_id[381].value <= threshold=22.993029594421387
node_961: feature_name=GO:0034101 feature_id[646].value <= threshold=13.430044651031494
node_962: feature_name=GO:0097153 feature_id[226].value <= threshold=10.147814273834229
node_963: feature_name=GO:0001836 feature_id[450].value <= threshold=7.252067804336548
node_964: feature_name=GO:0032504 feature_id[244].value > threshold=0.15777301788330078
node_968: feature_name=GO:0043379 feature_id[714].value > threshold=1.5959861278533936
node_974: feature_name=GO:0002705 feature_id[541].value <= threshold=3.148389160633087
Class: positive genes

Rules_566 passed counts:1
node_0: feature_name=GO:0042113 feature_id[0].value <= threshold=13.408552169799805
node_1: feature_name=GO:0007568 feature_id[534].value > threshold=5.0313897132873535
node_913: feature_name=GO:0032763 feature_id[629].value <= threshold=0.31753237545490265
node_914: feature_name=GO:0097193 feature_id[167].value <= threshold=28.171168327331543
node_915: feature_name=GO:0002903 feature_id[10].value <= threshold=1.0534588098526
node_916: feature_name=GO:1904029 feature_id[208].value <= threshold=23.852136611938477
node_917: feature_name=GO:0050897 feature_id[164].value <= threshold=0.6870408356189728
node_918: feature_name=GO:0006139 feature_id[474].value > threshold=1.3052097624921544e-07
node_920: feature_name=GO:0002821 feature_id[588].value <= threshold=13.81072187423706
node_921: feature_name=GO:0006298 feature_id[621].value <= threshold=24.078600883483887
node_922: feature_name=GO:0003908 feature_id[11].value <= threshold=1.7776933312416077
node_923: feature_name=GO:0030098 feature_id[273].value > threshold=0.0079949083738029
node_927: feature_name=GO:0006808 feature_id[513].value <= threshold=4.34592080116272

node_928: feature_name=GO:0071887 feature_id[283].value <= threshold=10.353787899017334
node_929: feature_name=GO:0038001 feature_id[28].value <= threshold=3.9469382762908936
node_930: feature_name=GO:0042287 feature_id[20].value <= threshold=3.6209195852279663
node_931: feature_name=GO:0003968 feature_id[47].value <= threshold=2.004227638244629
node_932: feature_name=GO:0002698 feature_id[395].value <= threshold=18.51447582244873
node_933: feature_name=GO:0044710 feature_id[719].value <= threshold=179.7340316772461
node_934: feature_name=GO:0007568 feature_id[534].value > threshold=5.035318374633789
node_936: feature_name=GO:0006216 feature_id[504].value <= threshold=2.2605666518211365
node_937: feature_name=GO:0048569 feature_id[793].value <= threshold=5.340231895446777
node_938: feature_name=GO:0001777 feature_id[380].value <= threshold=4.100832939147949
node_939: feature_name=GO:0007600 feature_id[122].value <= threshold=171.13383102416992
node_940: feature_name=GO:0001779 feature_id[378].value <= threshold=6.234851121902466
node_941: feature_name=GO:0030291 feature_id[147].value <= threshold=10.8051118850708
node_942: feature_name=GO:0048534 feature_id[790].value > threshold=0.017984486185014248
node_944: feature_name=GO:0070245 feature_id[326].value <= threshold=3.1282339096069336
node_945: feature_name=GO:0009086 feature_id[547].value <= threshold=0.2896959036588669
node_946: feature_name=GO:0001889 feature_id[387].value <= threshold=17.643128395080566
node_947: feature_name=GO:0048144 feature_id[192].value > threshold=0.20887330174446106
node_951: feature_name=GO:0023026 feature_id[69].value <= threshold=3.403424024581909
node_952: feature_name=GO:0048147 feature_id[788].value <= threshold=4.188283443450928
node_953: feature_name=GO:0090116 feature_id[97].value <= threshold=4.617154359817505
node_954: feature_name=GO:0002863 feature_id[490].value <= threshold=4.8968470096588135
node_955: feature_name=GO:0002524 feature_id[72].value <= threshold=3.035340189933777
node_956: feature_name=GO:0042130 feature_id[16].value <= threshold=7.285413980484009
node_957: feature_name=GO:0071456 feature_id[500].value <= threshold=21.212870597839355
node_958: feature_name=GO:2001238 feature_id[27].value <= threshold=7.849715709686279
node_959: feature_name=GO:0023030 feature_id[45].value <= threshold=1.7420591711997986
node_960: feature_name=GO:0072593 feature_id[381].value <= threshold=22.993029594421387
node_961: feature_name=GO:0034101 feature_id[646].value <= threshold=13.430044651031494
node_962: feature_name=GO:0097153 feature_id[226].value <= threshold=10.147814273834229
node_963: feature_name=GO:0001836 feature_id[450].value <= threshold=7.252067804336548
node_964: feature_name=GO:0032504 feature_id[244].value <= threshold=0.15777301788330078
node_965: feature_name=GO:0023026 feature_id[69].value > threshold=1.5684430599212646
Class: positive genes

Rules_567 passed counts:1
node_0: feature_name=GO:0042113 feature_id[0].value <= threshold=13.408552169799805
node_1: feature_name=GO:0007568 feature_id[534].value > threshold=5.0313897132873535
node_913: feature_name=GO:0032763 feature_id[629].value <= threshold=0.31753237545490265
node_914: feature_name=GO:0097193 feature_id[167].value <= threshold=28.171168327331543
node_915: feature_name=GO:0002903 feature_id[10].value <= threshold=1.0534588098526
node_916: feature_name=GO:1904029 feature_id[208].value <= threshold=23.852136611938477
node_917: feature_name=GO:0050897 feature_id[164].value <= threshold=0.6870408356189728
node_918: feature_name=GO:0006139 feature_id[474].value > threshold=1.3052097624921544e-07
node_920: feature_name=GO:0002821 feature_id[588].value <= threshold=13.81072187423706
node_921: feature_name=GO:0006298 feature_id[621].value <= threshold=24.078600883483887
node_922: feature_name=GO:0003908 feature_id[11].value <= threshold=1.7776933312416077
node_923: feature_name=GO:0030098 feature_id[273].value > threshold=0.0079949083738029
node_927: feature_name=GO:0006808 feature_id[513].value <= threshold=4.34592080116272
node_928: feature_name=GO:0071887 feature_id[283].value <= threshold=10.353787899017334
node_929: feature_name=GO:0038001 feature_id[28].value <= threshold=3.9469382762908936
node_930: feature_name=GO:0042287 feature_id[20].value <= threshold=3.6209195852279663
node_931: feature_name=GO:0003968 feature_id[47].value <= threshold=2.004227638244629
node_932: feature_name=GO:0002698 feature_id[395].value <= threshold=18.51447582244873
node_933: feature_name=GO:0044710 feature_id[719].value <= threshold=179.7340316772461
node_934: feature_name=GO:0007568 feature_id[534].value > threshold=5.035318374633789
node_936: feature_name=GO:0006216 feature_id[504].value <= threshold=2.2605666518211365
node_937: feature_name=GO:0048569 feature_id[793].value <= threshold=5.340231895446777
node_938: feature_name=GO:0001777 feature_id[380].value <= threshold=4.100832939147949
node_939: feature_name=GO:0007600 feature_id[122].value <= threshold=171.13383102416992
node_940: feature_name=GO:0001779 feature_id[378].value <= threshold=6.234851121902466
node_941: feature_name=GO:0030291 feature_id[147].value <= threshold=10.8051118850708
node_942: feature_name=GO:0048534 feature_id[790].value <= threshold=0.017984486185014248
Class: positive genes

Rules_568 passed counts:1
node_0: feature_name=GO:0042113 feature_id[0].value <= threshold=13.408552169799805
node_1: feature_name=GO:0007568 feature_id[534].value > threshold=5.0313897132873535
node_913: feature_name=GO:0032763 feature_id[629].value <= threshold=0.31753237545490265
node_914: feature_name=GO:0097193 feature_id[167].value <= threshold=28.171168327331543

node_915: feature_name=GO:0002903 feature_id[10].value <= threshold=1.0534588098526
node_916: feature_name=GO:1904029 feature_id[208].value <= threshold=23.852136611938477
node_917: feature_name=GO:0050897 feature_id[164].value <= threshold=0.6870408356189728
node_918: feature_name=GO:0006139 feature_id[474].value > threshold=1.3052097624921544e-07
node_920: feature_name=GO:0002821 feature_id[588].value <= threshold=13.81072187423706
node_921: feature_name=GO:0006298 feature_id[621].value <= threshold=24.078600883483887
node_922: feature_name=GO:0003908 feature_id[11].value <= threshold=1.7776933312416077
node_923: feature_name=GO:0030098 feature_id[273].value > threshold=0.0079949083738029
node_927: feature_name=GO:0006808 feature_id[513].value <= threshold=4.34592080116272
node_928: feature_name=GO:0071887 feature_id[283].value <= threshold=10.353787899017334
node_929: feature_name=GO:0038001 feature_id[28].value <= threshold=3.9469382762908936
node_930: feature_name=GO:0042287 feature_id[20].value <= threshold=3.6209195852279663
node_931: feature_name=GO:0003968 feature_id[47].value <= threshold=2.004227638244629
node_932: feature_name=GO:0002698 feature_id[395].value <= threshold=18.51447582244873
node_933: feature_name=GO:0044710 feature_id[719].value <= threshold=179.7340316772461
node_934: feature_name=GO:0007568 feature_id[534].value <= threshold=5.035318374633789
Class: positive genes

Rules_569 passed counts:1
node_0: feature_name=GO:0042113 feature_id[0].value <= threshold=13.408552169799805
node_1: feature_name=GO:0007568 feature_id[534].value <= threshold=5.0313897132873535
node_2: feature_name=GO:0002705 feature_id[541].value > threshold=3.200145721435547
node_870: feature_name=GO:0046634 feature_id[46].value <= threshold=8.9394211769104
node_871: feature_name=GO:0048523 feature_id[293].value <= threshold=19.963229179382324
node_872: feature_name=GO:2001237 feature_id[796].value > threshold=0.09761488810181618
node_880: feature_name=GO:0006808 feature_id[513].value <= threshold=1.7969900965690613
node_881: feature_name=GO:0032479 feature_id[441].value > threshold=0.13597124069929123
node_887: feature_name=GO:0043226 feature_id[138].value > threshold=12.855401992797852
node_903: feature_name=GO:0043231 feature_id[264].value <= threshold=20.170435905456543
node_904: feature_name=GO:0032480 feature_id[525].value <= threshold=0.41479843854904175
Class: negative genes

Rules_570 passed counts:1
node_0: feature_name=GO:0042113 feature_id[0].value <= threshold=13.408552169799805
node_1: feature_name=GO:0007568 feature_id[534].value <= threshold=5.0313897132873535

node_2: feature_name=GO:0002705 feature_id[541].value > threshold=3.200145721435547
node_870: feature_name=GO:0046634 feature_id[46].value <= threshold=8.9394211769104
node_871: feature_name=GO:0048523 feature_id[293].value <= threshold=19.963229179382324
node_872: feature_name=GO:2001237 feature_id[796].value > threshold=0.09761488810181618
node_880: feature_name=GO:0006808 feature_id[513].value <= threshold=1.7969900965690613
node_881: feature_name=GO:0032479 feature_id[441].value > threshold=0.13597124069929123
node_887: feature_name=GO:0043226 feature_id[138].value <= threshold=12.855401992797852
node_888: feature_name=GO:0060576 feature_id[204].value <= threshold=2.0569241046905518
node_889: feature_name=GO:0002719 feature_id[479].value <= threshold=5.209295988082886
node_890: feature_name=GO:0031104 feature_id[111].value <= threshold=1.9710296988487244
node_891: feature_name=GO:2001251 feature_id[220].value > threshold=2.3109558820724487
Class: positive genes

Rules_571 passed counts:1
node_0: feature_name=GO:0042113 feature_id[0].value <= threshold=13.408552169799805
node_1: feature_name=GO:0007568 feature_id[534].value <= threshold=5.0313897132873535
node_2: feature_name=GO:0002705 feature_id[541].value > threshold=3.200145721435547
node_870: feature_name=GO:0046634 feature_id[46].value <= threshold=8.9394211769104
node_871: feature_name=GO:0048523 feature_id[293].value <= threshold=19.963229179382324
node_872: feature_name=GO:2001237 feature_id[796].value > threshold=0.09761488810181618
node_880: feature_name=GO:0006808 feature_id[513].value <= threshold=1.7969900965690613
node_881: feature_name=GO:0032479 feature_id[441].value > threshold=0.13597124069929123
node_887: feature_name=GO:0043226 feature_id[138].value <= threshold=12.855401992797852
node_888: feature_name=GO:0060576 feature_id[204].value <= threshold=2.0569241046905518
node_889: feature_name=GO:0002719 feature_id[479].value <= threshold=5.209295988082886
node_890: feature_name=GO:0031104 feature_id[111].value <= threshold=1.9710296988487244
node_891: feature_name=GO:2001251 feature_id[220].value <= threshold=2.3109558820724487
node_892: feature_name=GO:0033077 feature_id[134].value > threshold=8.212203025817871
Class: positive genes

Rules_572 passed counts:1
node_0: feature_name=GO:0042113 feature_id[0].value <= threshold=13.408552169799805
node_1: feature_name=GO:0007568 feature_id[534].value <= threshold=5.0313897132873535
node_2: feature_name=GO:0002705 feature_id[541].value > threshold=3.200145721435547
node_870: feature_name=GO:0046634 feature_id[46].value <= threshold=8.9394211769104

node_871: feature_name=GO:0048523 feature_id[293].value <= threshold=19.963229179382324
node_872: feature_name=GO:2001237 feature_id[796].value > threshold=0.09761488810181618
node_880: feature_name=GO:0006808 feature_id[513].value <= threshold=1.7969900965690613
node_881: feature_name=GO:0032479 feature_id[441].value > threshold=0.13597124069929123
node_887: feature_name=GO:0043226 feature_id[138].value <= threshold=12.855401992797852
node_888: feature_name=GO:0060576 feature_id[204].value <= threshold=2.0569241046905518
node_889: feature_name=GO:0002719 feature_id[479].value <= threshold=5.209295988082886
node_890: feature_name=GO:0031104 feature_id[111].value <= threshold=1.9710296988487244
node_891: feature_name=GO:2001251 feature_id[220].value <= threshold=2.3109558820724487
node_892: feature_name=GO:0033077 feature_id[134].value <= threshold=8.212203025817871
node_893: feature_name=GO:0044026 feature_id[15].value > threshold=1.613231599330902
node_895: feature_name=GO:0046638 feature_id[776].value > threshold=2.7034546732902527
Class: negative genes

Rules_573 passed counts:1
node_0: feature_name=GO:0042113 feature_id[0].value <= threshold=13.408552169799805
node_1: feature_name=GO:0007568 feature_id[534].value <= threshold=5.0313897132873535
node_2: feature_name=GO:0002705 feature_id[541].value > threshold=3.200145721435547
node_870: feature_name=GO:0046634 feature_id[46].value <= threshold=8.9394211769104
node_871: feature_name=GO:0048523 feature_id[293].value <= threshold=19.963229179382324
node_872: feature_name=GO:2001237 feature_id[796].value > threshold=0.09761488810181618
node_880: feature_name=GO:0006808 feature_id[513].value <= threshold=1.7969900965690613
node_881: feature_name=GO:0032479 feature_id[441].value > threshold=0.13597124069929123
node_887: feature_name=GO:0043226 feature_id[138].value <= threshold=12.855401992797852
node_888: feature_name=GO:0060576 feature_id[204].value <= threshold=2.0569241046905518
node_889: feature_name=GO:0002719 feature_id[479].value <= threshold=5.209295988082886
node_890: feature_name=GO:0031104 feature_id[111].value <= threshold=1.9710296988487244
node_891: feature_name=GO:2001251 feature_id[220].value <= threshold=2.3109558820724487
node_892: feature_name=GO:0033077 feature_id[134].value <= threshold=8.212203025817871
node_893: feature_name=GO:0044026 feature_id[15].value > threshold=1.613231599330902
node_895: feature_name=GO:0046638 feature_id[776].value <= threshold=2.7034546732902527
Class: positive genes

Rules_574 passed counts:1
node_0: feature_name=GO:0042113 feature_id[0].value <= threshold=13.408552169799805

node_1: feature_name=GO:0007568 feature_id[534].value <= threshold=5.0313897132873535
node_2: feature_name=GO:0002705 feature_id[541].value > threshold=3.200145721435547
node_870: feature_name=GO:0046634 feature_id[46].value <= threshold=8.9394211769104
node_871: feature_name=GO:0048523 feature_id[293].value <= threshold=19.963229179382324
node_872: feature_name=GO:2001237 feature_id[796].value > threshold=0.09761488810181618
node_880: feature_name=GO:0006808 feature_id[513].value <= threshold=1.7969900965690613
node_881: feature_name=GO:0032479 feature_id[441].value <= threshold=0.13597124069929123
node_882: feature_name=GO:0000060 feature_id[223].value <= threshold=2.2508251667022705
node_883: feature_name=GO:0048144 feature_id[192].value <= threshold=0.0181948971003294
Class: positive genes

Rules_575 passed counts:1
node_0: feature_name=GO:0042113 feature_id[0].value <= threshold=13.408552169799805
node_1: feature_name=GO:0007568 feature_id[534].value <= threshold=5.0313897132873535
node_2: feature_name=GO:0002705 feature_id[541].value > threshold=3.200145721435547
node_870: feature_name=GO:0046634 feature_id[46].value <= threshold=8.9394211769104
node_871: feature_name=GO:0048523 feature_id[293].value <= threshold=19.963229179382324
node_872: feature_name=GO:2001237 feature_id[796].value <= threshold=0.09761488810181618
node_873: feature_name=GO:0097028 feature_id[443].value <= threshold=1.0470510721206665
node_874: feature_name=GO:0043066 feature_id[667].value > threshold=2.3243058919906616
Class: positive genes

Rules_576 passed counts:1
node_0: feature_name=GO:0042113 feature_id[0].value <= threshold=13.408552169799805
node_1: feature_name=GO:0007568 feature_id[534].value <= threshold=5.0313897132873535
node_2: feature_name=GO:0002705 feature_id[541].value <= threshold=3.200145721435547
node_3: feature_name=GO:1901525 feature_id[576].value > threshold=0.4466460347175598
node_617: feature_name=GO:0005622 feature_id[233].value > threshold=90.85730743408203
node_847: feature_name=GO:0002639 feature_id[466].value > threshold=0.32853202521800995
node_867: feature_name=GO:0046498 feature_id[203].value > threshold=0.5923898071050644
Class: negative genes

Rules_577 passed counts:1
node_0: feature_name=GO:0042113 feature_id[0].value <= threshold=13.408552169799805
node_1: feature_name=GO:0007568 feature_id[534].value <= threshold=5.0313897132873535

node_2: feature_name=GO:0002705 feature_id[541].value <= threshold=3.200145721435547
node_3: feature_name=GO:1901525 feature_id[576].value > threshold=0.4466460347175598
node_617: feature_name=GO:0005622 feature_id[233].value > threshold=90.85730743408203
node_847: feature_name=GO:0002639 feature_id[466].value <= threshold=0.32853202521800995
node_848: feature_name=GO:0002636 feature_id[447].value > threshold=0.3303467631340027
node_864: feature_name=GO:0002200 feature_id[82].value <= threshold=0.11118704453110695
Class: negative genes

Rules_578 passed counts:1
node_0: feature_name=GO:0042113 feature_id[0].value <= threshold=13.408552169799805
node_1: feature_name=GO:0007568 feature_id[534].value <= threshold=5.0313897132873535
node_2: feature_name=GO:0002705 feature_id[541].value <= threshold=3.200145721435547
node_3: feature_name=GO:1901525 feature_id[576].value > threshold=0.4466460347175598
node_617: feature_name=GO:0005622 feature_id[233].value > threshold=90.85730743408203
node_847: feature_name=GO:0002639 feature_id[466].value <= threshold=0.32853202521800995
node_848: feature_name=GO:0002636 feature_id[447].value <= threshold=0.3303467631340027
node_849: feature_name=GO:0001783 feature_id[33].value > threshold=0.07426292821764946
node_851: feature_name=GO:0032763 feature_id[629].value > threshold=0.21307963132858276
node_853: feature_name=GO:0045404 feature_id[741].value <= threshold=0.4299612492322922
node_854: feature_name=GO:0033158 feature_id[379].value > threshold=0.09315907582640648
node_856: feature_name=GO:0008156 feature_id[239].value > threshold=0.07084834203124046
node_858: feature_name=GO:0038065 feature_id[670].value > threshold=0.5210016071796417
node_860: feature_name=GO:0050678 feature_id[798].value <= threshold=1.3203300088644028
Class: negative genes

Rules_579 passed counts:1
node_0: feature_name=GO:0042113 feature_id[0].value <= threshold=13.408552169799805
node_1: feature_name=GO:0007568 feature_id[534].value <= threshold=5.0313897132873535
node_2: feature_name=GO:0002705 feature_id[541].value <= threshold=3.200145721435547
node_3: feature_name=GO:1901525 feature_id[576].value > threshold=0.4466460347175598
node_617: feature_name=GO:0005622 feature_id[233].value <= threshold=90.85730743408203
node_618: feature_name=GO:1903147 feature_id[200].value <= threshold=7.326428413391113
node_619: feature_name=GO:0003964 feature_id[317].value > threshold=5.575642108917236
node_843: feature_name=GO:0002293 feature_id[206].value > threshold=0.668750561773777
Class: negative genes

Rules_580 passed counts:1
node_0: feature_name=GO:0042113 feature_id[0].value <= threshold=13.408552169799805
node_1: feature_name=GO:0007568 feature_id[534].value <= threshold=5.0313897132873535
node_2: feature_name=GO:0002705 feature_id[541].value <= threshold=3.200145721435547
node_3: feature_name=GO:1901525 feature_id[576].value > threshold=0.4466460347175598
node_617: feature_name=GO:0005622 feature_id[233].value <= threshold=90.85730743408203
node_618: feature_name=GO:1903147 feature_id[200].value <= threshold=7.326428413391113
node_619: feature_name=GO:0003964 feature_id[317].value <= threshold=5.575642108917236
node_620: feature_name=GO:0046498 feature_id[203].value > threshold=6.620223522186279
node_840: feature_name=GO:0015672 feature_id[573].value <= threshold=0.3655537310987711
Class: negative genes

Rules_581 passed counts:1
node_0: feature_name=GO:0042113 feature_id[0].value <= threshold=13.408552169799805
node_1: feature_name=GO:0007568 feature_id[534].value <= threshold=5.0313897132873535
node_2: feature_name=GO:0002705 feature_id[541].value <= threshold=3.200145721435547
node_3: feature_name=GO:1901525 feature_id[576].value > threshold=0.4466460347175598
node_617: feature_name=GO:0005622 feature_id[233].value <= threshold=90.85730743408203
node_618: feature_name=GO:1903147 feature_id[200].value <= threshold=7.326428413391113
node_619: feature_name=GO:0003964 feature_id[317].value <= threshold=5.575642108917236
node_620: feature_name=GO:0046498 feature_id[203].value <= threshold=6.620223522186279
node_621: feature_name=GO:0002562 feature_id[25].value <= threshold=10.287704467773438
node_622: feature_name=GO:0006919 feature_id[246].value > threshold=5.801334381103516
node_834: feature_name=GO:0045931 feature_id[266].value > threshold=0.973282665014267
node_836: feature_name=GO:0030889 feature_id[602].value <= threshold=0.19142773747444153
Class: negative genes

Rules_582 passed counts:1
node_0: feature_name=GO:0042113 feature_id[0].value <= threshold=13.408552169799805
node_1: feature_name=GO:0007568 feature_id[534].value <= threshold=5.0313897132873535
node_2: feature_name=GO:0002705 feature_id[541].value <= threshold=3.200145721435547
node_3: feature_name=GO:1901525 feature_id[576].value > threshold=0.4466460347175598
node_617: feature_name=GO:0005622 feature_id[233].value <= threshold=90.85730743408203
node_618: feature_name=GO:1903147 feature_id[200].value <= threshold=7.326428413391113

node_619: feature_name=GO:0003964 feature_id[317].value <= threshold=5.575642108917236
node_620: feature_name=GO:0046498 feature_id[203].value <= threshold=6.620223522186279
node_621: feature_name=GO:0002562 feature_id[25].value <= threshold=10.287704467773438
node_622: feature_name=GO:0006919 feature_id[246].value <= threshold=5.801334381103516
node_623: feature_name=GO:0001889 feature_id[387].value > threshold=2.679414987564087
node_785: feature_name=GO:0030887 feature_id[274].value > threshold=1.1379476189613342
node_831: feature_name=GO:0002274 feature_id[130].value > threshold=1.7421051859855652
Class: negative genes

Rules_583 passed counts:1
node_0: feature_name=GO:0042113 feature_id[0].value <= threshold=13.408552169799805
node_1: feature_name=GO:0007568 feature_id[534].value <= threshold=5.0313897132873535
node_2: feature_name=GO:0002705 feature_id[541].value <= threshold=3.200145721435547
node_3: feature_name=GO:1901525 feature_id[576].value > threshold=0.4466460347175598
node_617: feature_name=GO:0005622 feature_id[233].value <= threshold=90.85730743408203
node_618: feature_name=GO:1903147 feature_id[200].value <= threshold=7.326428413391113
node_619: feature_name=GO:0003964 feature_id[317].value <= threshold=5.575642108917236
node_620: feature_name=GO:0046498 feature_id[203].value <= threshold=6.620223522186279
node_621: feature_name=GO:0002562 feature_id[25].value <= threshold=10.287704467773438
node_622: feature_name=GO:0006919 feature_id[246].value <= threshold=5.801334381103516
node_623: feature_name=GO:0001889 feature_id[387].value > threshold=2.679414987564087
node_785: feature_name=GO:0030887 feature_id[274].value <= threshold=1.1379476189613342
node_786: feature_name=GO:1902564 feature_id[85].value <= threshold=1.4637635350227356
node_787: feature_name=GO:0045402 feature_id[286].value <= threshold=1.5249269604682922
node_788: feature_name=GO:0034614 feature_id[655].value <= threshold=9.142115116119385
node_789: feature_name=GO:0000783 feature_id[191].value <= threshold=1.5124736428260803
node_790: feature_name=GO:0048145 feature_id[348].value > threshold=0.026725558564066887
node_794: feature_name=GO:0044092 feature_id[432].value > threshold=31.540730476379395
Class: positive genes

Rules_584 passed counts:1
node_0: feature_name=GO:0042113 feature_id[0].value <= threshold=13.408552169799805
node_1: feature_name=GO:0007568 feature_id[534].value <= threshold=5.0313897132873535
node_2: feature_name=GO:0002705 feature_id[541].value <= threshold=3.200145721435547
node_3: feature_name=GO:1901525 feature_id[576].value > threshold=0.4466460347175598

node_617: feature_name=GO:0005622 feature_id[233].value <= threshold=90.85730743408203
node_618: feature_name=GO:1903147 feature_id[200].value <= threshold=7.326428413391113
node_619: feature_name=GO:0003964 feature_id[317].value <= threshold=5.575642108917236
node_620: feature_name=GO:0046498 feature_id[203].value <= threshold=6.620223522186279
node_621: feature_name=GO:0002562 feature_id[25].value <= threshold=10.287704467773438
node_622: feature_name=GO:0006919 feature_id[246].value <= threshold=5.801334381103516
node_623: feature_name=GO:0001889 feature_id[387].value > threshold=2.679414987564087
node_785: feature_name=GO:0030887 feature_id[274].value <= threshold=1.1379476189613342
node_786: feature_name=GO:1902564 feature_id[85].value <= threshold=1.4637635350227356
node_787: feature_name=GO:0045402 feature_id[286].value <= threshold=1.5249269604682922
node_788: feature_name=GO:0034614 feature_id[655].value <= threshold=9.142115116119385
node_789: feature_name=GO:0000783 feature_id[191].value <= threshold=1.5124736428260803
node_790: feature_name=GO:0048145 feature_id[348].value > threshold=0.026725558564066887
node_794: feature_name=GO:0044092 feature_id[432].value <= threshold=31.540730476379395
node_795: feature_name=GO:0042771 feature_id[686].value > threshold=6.047890663146973
Class: positive genes

Rules_585 passed counts:1
node_0: feature_name=GO:0042113 feature_id[0].value <= threshold=13.408552169799805
node_1: feature_name=GO:0007568 feature_id[534].value <= threshold=5.0313897132873535
node_2: feature_name=GO:0002705 feature_id[541].value <= threshold=3.200145721435547
node_3: feature_name=GO:1901525 feature_id[576].value > threshold=0.4466460347175598
node_617: feature_name=GO:0005622 feature_id[233].value <= threshold=90.85730743408203
node_618: feature_name=GO:1903147 feature_id[200].value <= threshold=7.326428413391113
node_619: feature_name=GO:0003964 feature_id[317].value <= threshold=5.575642108917236
node_620: feature_name=GO:0046498 feature_id[203].value <= threshold=6.620223522186279
node_621: feature_name=GO:0002562 feature_id[25].value <= threshold=10.287704467773438
node_622: feature_name=GO:0006919 feature_id[246].value <= threshold=5.801334381103516
node_623: feature_name=GO:0001889 feature_id[387].value > threshold=2.679414987564087
node_785: feature_name=GO:0030887 feature_id[274].value <= threshold=1.1379476189613342
node_786: feature_name=GO:1902564 feature_id[85].value <= threshold=1.4637635350227356
node_787: feature_name=GO:0045402 feature_id[286].value <= threshold=1.5249269604682922
node_788: feature_name=GO:0034614 feature_id[655].value <= threshold=9.142115116119385
node_789: feature_name=GO:0000783 feature_id[191].value <= threshold=1.5124736428260803
node_790: feature_name=GO:0048145 feature_id[348].value > threshold=0.026725558564066887

node_794: feature_name=GO:0044092 feature_id[432].value <= threshold=31.540730476379395
node_795: feature_name=GO:0042771 feature_id[686].value <= threshold=6.047890663146973
node_796: feature_name=GO:0070301 feature_id[81].value > threshold=5.959718465805054
Class: positive genes

Rules_586 passed counts:1
node_0: feature_name=GO:0042113 feature_id[0].value <= threshold=13.408552169799805
node_1: feature_name=GO:0007568 feature_id[534].value <= threshold=5.0313897132873535
node_2: feature_name=GO:0002705 feature_id[541].value <= threshold=3.200145721435547
node_3: feature_name=GO:1901525 feature_id[576].value > threshold=0.4466460347175598
node_617: feature_name=GO:0005622 feature_id[233].value <= threshold=90.85730743408203
node_618: feature_name=GO:1903147 feature_id[200].value <= threshold=7.326428413391113
node_619: feature_name=GO:0003964 feature_id[317].value <= threshold=5.575642108917236
node_620: feature_name=GO:0046498 feature_id[203].value <= threshold=6.620223522186279
node_621: feature_name=GO:0002562 feature_id[25].value <= threshold=10.287704467773438
node_622: feature_name=GO:0006919 feature_id[246].value <= threshold=5.801334381103516
node_623: feature_name=GO:0001889 feature_id[387].value > threshold=2.679414987564087
node_785: feature_name=GO:0030887 feature_id[274].value <= threshold=1.1379476189613342
node_786: feature_name=GO:1902564 feature_id[85].value <= threshold=1.4637635350227356
node_787: feature_name=GO:0045402 feature_id[286].value <= threshold=1.5249269604682922
node_788: feature_name=GO:0034614 feature_id[655].value <= threshold=9.142115116119385
node_789: feature_name=GO:0000783 feature_id[191].value <= threshold=1.5124736428260803
node_790: feature_name=GO:0048145 feature_id[348].value > threshold=0.026725558564066887
node_794: feature_name=GO:0044092 feature_id[432].value <= threshold=31.540730476379395
node_795: feature_name=GO:0042771 feature_id[686].value <= threshold=6.047890663146973
node_796: feature_name=GO:0070301 feature_id[81].value <= threshold=5.959718465805054
node_797: feature_name=GO:0006809 feature_id[511].value > threshold=9.000734329223633
Class: positive genes

Rules_587 passed counts:1
node_0: feature_name=GO:0042113 feature_id[0].value <= threshold=13.408552169799805
node_1: feature_name=GO:0007568 feature_id[534].value <= threshold=5.0313897132873535
node_2: feature_name=GO:0002705 feature_id[541].value <= threshold=3.200145721435547
node_3: feature_name=GO:1901525 feature_id[576].value > threshold=0.4466460347175598
node_617: feature_name=GO:0005622 feature_id[233].value <= threshold=90.85730743408203

node_618: feature_name=GO:1903147 feature_id[200].value <= threshold=7.326428413391113
node_619: feature_name=GO:0003964 feature_id[317].value <= threshold=5.575642108917236
node_620: feature_name=GO:0046498 feature_id[203].value <= threshold=6.620223522186279
node_621: feature_name=GO:0002562 feature_id[25].value <= threshold=10.287704467773438
node_622: feature_name=GO:0006919 feature_id[246].value <= threshold=5.801334381103516
node_623: feature_name=GO:0001889 feature_id[387].value > threshold=2.679414987564087
node_785: feature_name=GO:0030887 feature_id[274].value <= threshold=1.1379476189613342
node_786: feature_name=GO:1902564 feature_id[85].value <= threshold=1.4637635350227356
node_787: feature_name=GO:0045402 feature_id[286].value <= threshold=1.5249269604682922
node_788: feature_name=GO:0034614 feature_id[655].value <= threshold=9.142115116119385
node_789: feature_name=GO:0000783 feature_id[191].value <= threshold=1.5124736428260803
node_790: feature_name=GO:0048145 feature_id[348].value > threshold=0.026725558564066887
node_794: feature_name=GO:0044092 feature_id[432].value <= threshold=31.540730476379395
node_795: feature_name=GO:0042771 feature_id[686].value <= threshold=6.047890663146973
node_796: feature_name=GO:0070301 feature_id[81].value <= threshold=5.959718465805054
node_797: feature_name=GO:0006809 feature_id[511].value <= threshold=9.000734329223633
node_798: feature_name=GO:0008150 feature_id[528].value > threshold=0.40075723826885223
node_800: feature_name=GO:0010663 feature_id[561].value > threshold=0.20622049272060394
node_802: feature_name=GO:0009636 feature_id[551].value > threshold=0.12915228679776192
node_804: feature_name=GO:0002524 feature_id[72].value > threshold=1.0410467386245728
Class: positive genes

Rules_588 passed counts:1
node_0: feature_name=GO:0042113 feature_id[0].value <= threshold=13.408552169799805
node_1: feature_name=GO:0007568 feature_id[534].value <= threshold=5.0313897132873535
node_2: feature_name=GO:0002705 feature_id[541].value <= threshold=3.200145721435547
node_3: feature_name=GO:1901525 feature_id[576].value > threshold=0.4466460347175598
node_617: feature_name=GO:0005622 feature_id[233].value <= threshold=90.85730743408203
node_618: feature_name=GO:1903147 feature_id[200].value <= threshold=7.326428413391113
node_619: feature_name=GO:0003964 feature_id[317].value <= threshold=5.575642108917236
node_620: feature_name=GO:0046498 feature_id[203].value <= threshold=6.620223522186279
node_621: feature_name=GO:0002562 feature_id[25].value <= threshold=10.287704467773438
node_622: feature_name=GO:0006919 feature_id[246].value <= threshold=5.801334381103516
node_623: feature_name=GO:0001889 feature_id[387].value > threshold=2.679414987564087
node_785: feature_name=GO:0030887 feature_id[274].value <= threshold=1.1379476189613342

node_786: feature_name=GO:1902564 feature_id[85].value <= threshold=1.4637635350227356
node_787: feature_name=GO:0045402 feature_id[286].value <= threshold=1.5249269604682922
node_788: feature_name=GO:0034614 feature_id[655].value <= threshold=9.142115116119385
node_789: feature_name=GO:0000783 feature_id[191].value <= threshold=1.5124736428260803
node_790: feature_name=GO:0048145 feature_id[348].value > threshold=0.026725558564066887
node_794: feature_name=GO:0044092 feature_id[432].value <= threshold=31.540730476379395
node_795: feature_name=GO:0042771 feature_id[686].value <= threshold=6.047890663146973
node_796: feature_name=GO:0070301 feature_id[81].value <= threshold=5.959718465805054
node_797: feature_name=GO:0006809 feature_id[511].value <= threshold=9.000734329223633
node_798: feature_name=GO:0008150 feature_id[528].value > threshold=0.40075723826885223
node_800: feature_name=GO:0010663 feature_id[561].value > threshold=0.20622049272060394
node_802: feature_name=GO:0009636 feature_id[551].value > threshold=0.12915228679776192
node_804: feature_name=GO:0002524 feature_id[72].value <= threshold=1.0410467386245728
node_805: feature_name=GO:0035872 feature_id[659].value > threshold=6.628145217895508
Class: positive genes

Rules_589 passed counts:1
node_0: feature_name=GO:0042113 feature_id[0].value <= threshold=13.408552169799805
node_1: feature_name=GO:0007568 feature_id[534].value <= threshold=5.0313897132873535
node_2: feature_name=GO:0002705 feature_id[541].value <= threshold=3.200145721435547
node_3: feature_name=GO:1901525 feature_id[576].value > threshold=0.4466460347175598
node_617: feature_name=GO:0005622 feature_id[233].value <= threshold=90.85730743408203
node_618: feature_name=GO:1903147 feature_id[200].value <= threshold=7.326428413391113
node_619: feature_name=GO:0003964 feature_id[317].value <= threshold=5.575642108917236
node_620: feature_name=GO:0046498 feature_id[203].value <= threshold=6.620223522186279
node_621: feature_name=GO:0002562 feature_id[25].value <= threshold=10.287704467773438
node_622: feature_name=GO:0006919 feature_id[246].value <= threshold=5.801334381103516
node_623: feature_name=GO:0001889 feature_id[387].value > threshold=2.679414987564087
node_785: feature_name=GO:0030887 feature_id[274].value <= threshold=1.1379476189613342
node_786: feature_name=GO:1902564 feature_id[85].value <= threshold=1.4637635350227356
node_787: feature_name=GO:0045402 feature_id[286].value <= threshold=1.5249269604682922
node_788: feature_name=GO:0034614 feature_id[655].value <= threshold=9.142115116119385
node_789: feature_name=GO:0000783 feature_id[191].value <= threshold=1.5124736428260803
node_790: feature_name=GO:0048145 feature_id[348].value > threshold=0.026725558564066887
node_794: feature_name=GO:0044092 feature_id[432].value <= threshold=31.540730476379395

node_795: feature_name=GO:0042771 feature_id[686].value <= threshold=6.047890663146973
node_796: feature_name=GO:0070301 feature_id[81].value <= threshold=5.959718465805054
node_797: feature_name=GO:0006809 feature_id[511].value <= threshold=9.000734329223633
node_798: feature_name=GO:0008150 feature_id[528].value > threshold=0.40075723826885223
node_800: feature_name=GO:0010663 feature_id[561].value > threshold=0.20622049272060394
node_802: feature_name=GO:0009636 feature_id[551].value > threshold=0.12915228679776192
node_804: feature_name=GO:0002524 feature_id[72].value <= threshold=1.0410467386245728
node_805: feature_name=GO:0035872 feature_id[659].value <= threshold=6.628145217895508
node_806: feature_name=GO:0010225 feature_id[558].value > threshold=4.188441753387451
Class: positive genes

Rules_590 passed counts:1
node_0: feature_name=GO:0042113 feature_id[0].value <= threshold=13.408552169799805
node_1: feature_name=GO:0007568 feature_id[534].value <= threshold=5.0313897132873535
node_2: feature_name=GO:0002705 feature_id[541].value <= threshold=3.200145721435547
node_3: feature_name=GO:1901525 feature_id[576].value > threshold=0.4466460347175598
node_617: feature_name=GO:0005622 feature_id[233].value <= threshold=90.85730743408203
node_618: feature_name=GO:1903147 feature_id[200].value <= threshold=7.326428413391113
node_619: feature_name=GO:0003964 feature_id[317].value <= threshold=5.575642108917236
node_620: feature_name=GO:0046498 feature_id[203].value <= threshold=6.620223522186279
node_621: feature_name=GO:0002562 feature_id[25].value <= threshold=10.287704467773438
node_622: feature_name=GO:0006919 feature_id[246].value <= threshold=5.801334381103516
node_623: feature_name=GO:0001889 feature_id[387].value > threshold=2.679414987564087
node_785: feature_name=GO:0030887 feature_id[274].value <= threshold=1.1379476189613342
node_786: feature_name=GO:1902564 feature_id[85].value <= threshold=1.4637635350227356
node_787: feature_name=GO:0045402 feature_id[286].value <= threshold=1.5249269604682922
node_788: feature_name=GO:0034614 feature_id[655].value <= threshold=9.142115116119385
node_789: feature_name=GO:0000783 feature_id[191].value <= threshold=1.5124736428260803
node_790: feature_name=GO:0048145 feature_id[348].value > threshold=0.026725558564066887
node_794: feature_name=GO:0044092 feature_id[432].value <= threshold=31.540730476379395
node_795: feature_name=GO:0042771 feature_id[686].value <= threshold=6.047890663146973
node_796: feature_name=GO:0070301 feature_id[81].value <= threshold=5.959718465805054
node_797: feature_name=GO:0006809 feature_id[511].value <= threshold=9.000734329223633
node_798: feature_name=GO:0008150 feature_id[528].value > threshold=0.40075723826885223
node_800: feature_name=GO:0010663 feature_id[561].value > threshold=0.20622049272060394

node_802: feature_name=GO:0009636 feature_id[551].value > threshold=0.12915228679776192
node_804: feature_name=GO:0002524 feature_id[72].value <= threshold=1.0410467386245728
node_805: feature_name=GO:0035872 feature_id[659].value <= threshold=6.628145217895508
node_806: feature_name=GO:0010225 feature_id[558].value <= threshold=4.188441753387451
node_807: feature_name=GO:0002262 feature_id[394].value > threshold=8.843595027923584
Class: positive genes

Rules_591 passed counts:1
node_0: feature_name=GO:0042113 feature_id[0].value <= threshold=13.408552169799805
node_1: feature_name=GO:0007568 feature_id[534].value <= threshold=5.0313897132873535
node_2: feature_name=GO:0002705 feature_id[541].value <= threshold=3.200145721435547
node_3: feature_name=GO:1901525 feature_id[576].value > threshold=0.4466460347175598
node_617: feature_name=GO:0005622 feature_id[233].value <= threshold=90.85730743408203
node_618: feature_name=GO:1903147 feature_id[200].value <= threshold=7.326428413391113
node_619: feature_name=GO:0003964 feature_id[317].value <= threshold=5.575642108917236
node_620: feature_name=GO:0046498 feature_id[203].value <= threshold=6.620223522186279
node_621: feature_name=GO:0002562 feature_id[25].value <= threshold=10.287704467773438
node_622: feature_name=GO:0006919 feature_id[246].value <= threshold=5.801334381103516
node_623: feature_name=GO:0001889 feature_id[387].value > threshold=2.679414987564087
node_785: feature_name=GO:0030887 feature_id[274].value <= threshold=1.1379476189613342
node_786: feature_name=GO:1902564 feature_id[85].value <= threshold=1.4637635350227356
node_787: feature_name=GO:0045402 feature_id[286].value <= threshold=1.5249269604682922
node_788: feature_name=GO:0034614 feature_id[655].value <= threshold=9.142115116119385
node_789: feature_name=GO:0000783 feature_id[191].value <= threshold=1.5124736428260803
node_790: feature_name=GO:0048145 feature_id[348].value > threshold=0.026725558564066887
node_794: feature_name=GO:0044092 feature_id[432].value <= threshold=31.540730476379395
node_795: feature_name=GO:0042771 feature_id[686].value <= threshold=6.047890663146973
node_796: feature_name=GO:0070301 feature_id[81].value <= threshold=5.959718465805054
node_797: feature_name=GO:0006809 feature_id[511].value <= threshold=9.000734329223633
node_798: feature_name=GO:0008150 feature_id[528].value > threshold=0.40075723826885223
node_800: feature_name=GO:0010663 feature_id[561].value > threshold=0.20622049272060394
node_802: feature_name=GO:0009636 feature_id[551].value > threshold=0.12915228679776192
node_804: feature_name=GO:0002524 feature_id[72].value <= threshold=1.0410467386245728
node_805: feature_name=GO:0035872 feature_id[659].value <= threshold=6.628145217895508
node_806: feature_name=GO:0010225 feature_id[558].value <= threshold=4.188441753387451

node_807: feature_name=GO:0002262 feature_id[394].value <= threshold=8.843595027923584
node_808: feature_name=GO:0048569 feature_id[793].value > threshold=4.370251417160034
node_814: feature_name=GO:0010033 feature_id[194].value > threshold=15.024241924285889
Class: negative genes

Rules_592 passed counts:1
node_0: feature_name=GO:0042113 feature_id[0].value <= threshold=13.408552169799805
node_1: feature_name=GO:0007568 feature_id[534].value <= threshold=5.0313897132873535
node_2: feature_name=GO:0002705 feature_id[541].value <= threshold=3.200145721435547
node_3: feature_name=GO:1901525 feature_id[576].value > threshold=0.4466460347175598
node_617: feature_name=GO:0005622 feature_id[233].value <= threshold=90.85730743408203
node_618: feature_name=GO:1903147 feature_id[200].value <= threshold=7.326428413391113
node_619: feature_name=GO:0003964 feature_id[317].value <= threshold=5.575642108917236
node_620: feature_name=GO:0046498 feature_id[203].value <= threshold=6.620223522186279
node_621: feature_name=GO:0002562 feature_id[25].value <= threshold=10.287704467773438
node_622: feature_name=GO:0006919 feature_id[246].value <= threshold=5.801334381103516
node_623: feature_name=GO:0001889 feature_id[387].value > threshold=2.679414987564087
node_785: feature_name=GO:0030887 feature_id[274].value <= threshold=1.1379476189613342
node_786: feature_name=GO:1902564 feature_id[85].value <= threshold=1.4637635350227356
node_787: feature_name=GO:0045402 feature_id[286].value <= threshold=1.5249269604682922
node_788: feature_name=GO:0034614 feature_id[655].value <= threshold=9.142115116119385
node_789: feature_name=GO:0000783 feature_id[191].value <= threshold=1.5124736428260803
node_790: feature_name=GO:0048145 feature_id[348].value > threshold=0.026725558564066887
node_794: feature_name=GO:0044092 feature_id[432].value <= threshold=31.540730476379395
node_795: feature_name=GO:0042771 feature_id[686].value <= threshold=6.047890663146973
node_796: feature_name=GO:0070301 feature_id[81].value <= threshold=5.959718465805054
node_797: feature_name=GO:0006809 feature_id[511].value <= threshold=9.000734329223633
node_798: feature_name=GO:0008150 feature_id[528].value > threshold=0.40075723826885223
node_800: feature_name=GO:0010663 feature_id[561].value > threshold=0.20622049272060394
node_802: feature_name=GO:0009636 feature_id[551].value > threshold=0.12915228679776192
node_804: feature_name=GO:0002524 feature_id[72].value <= threshold=1.0410467386245728
node_805: feature_name=GO:0035872 feature_id[659].value <= threshold=6.628145217895508
node_806: feature_name=GO:0010225 feature_id[558].value <= threshold=4.188441753387451
node_807: feature_name=GO:0002262 feature_id[394].value <= threshold=8.843595027923584
node_808: feature_name=GO:0048569 feature_id[793].value > threshold=4.370251417160034

node_814: feature_name=GO:0010033 feature_id[194].value <= threshold=15.024241924285889
Class: positive genes

Rules_593 passed counts:1
node_0: feature_name=GO:0042113 feature_id[0].value <= threshold=13.408552169799805
node_1: feature_name=GO:0007568 feature_id[534].value <= threshold=5.0313897132873535
node_2: feature_name=GO:0002705 feature_id[541].value <= threshold=3.200145721435547
node_3: feature_name=GO:1901525 feature_id[576].value > threshold=0.4466460347175598
node_617: feature_name=GO:0005622 feature_id[233].value <= threshold=90.85730743408203
node_618: feature_name=GO:1903147 feature_id[200].value <= threshold=7.326428413391113
node_619: feature_name=GO:0003964 feature_id[317].value <= threshold=5.575642108917236
node_620: feature_name=GO:0046498 feature_id[203].value <= threshold=6.620223522186279
node_621: feature_name=GO:0002562 feature_id[25].value <= threshold=10.287704467773438
node_622: feature_name=GO:0006919 feature_id[246].value <= threshold=5.801334381103516
node_623: feature_name=GO:0001889 feature_id[387].value > threshold=2.679414987564087
node_785: feature_name=GO:0030887 feature_id[274].value <= threshold=1.1379476189613342
node_786: feature_name=GO:1902564 feature_id[85].value <= threshold=1.4637635350227356
node_787: feature_name=GO:0045402 feature_id[286].value <= threshold=1.5249269604682922
node_788: feature_name=GO:0034614 feature_id[655].value <= threshold=9.142115116119385
node_789: feature_name=GO:0000783 feature_id[191].value <= threshold=1.5124736428260803
node_790: feature_name=GO:0048145 feature_id[348].value > threshold=0.026725558564066887
node_794: feature_name=GO:0044092 feature_id[432].value <= threshold=31.540730476379395
node_795: feature_name=GO:0042771 feature_id[686].value <= threshold=6.047890663146973
node_796: feature_name=GO:0070301 feature_id[81].value <= threshold=5.959718465805054
node_797: feature_name=GO:0006809 feature_id[511].value <= threshold=9.000734329223633
node_798: feature_name=GO:0008150 feature_id[528].value > threshold=0.40075723826885223
node_800: feature_name=GO:0010663 feature_id[561].value > threshold=0.20622049272060394
node_802: feature_name=GO:0009636 feature_id[551].value > threshold=0.12915228679776192
node_804: feature_name=GO:0002524 feature_id[72].value <= threshold=1.0410467386245728
node_805: feature_name=GO:0035872 feature_id[659].value <= threshold=6.628145217895508
node_806: feature_name=GO:0010225 feature_id[558].value <= threshold=4.188441753387451
node_807: feature_name=GO:0002262 feature_id[394].value <= threshold=8.843595027923584
node_808: feature_name=GO:0048569 feature_id[793].value <= threshold=4.370251417160034
node_809: feature_name=GO:0046982 feature_id[172].value <= threshold=0.010676630306988955
node_810: feature_name=GO:0005622 feature_id[233].value <= threshold=17.8395357131958

Class: positive genes

Rules_594 passed counts:1
node_0: feature_name=GO:0042113 feature_id[0].value <= threshold=13.408552169799805
node_1: feature_name=GO:0007568 feature_id[534].value <= threshold=5.0313897132873535
node_2: feature_name=GO:0002705 feature_id[541].value <= threshold=3.200145721435547
node_3: feature_name=GO:1901525 feature_id[576].value > threshold=0.4466460347175598
node_617: feature_name=GO:0005622 feature_id[233].value <= threshold=90.85730743408203
node_618: feature_name=GO:1903147 feature_id[200].value <= threshold=7.326428413391113
node_619: feature_name=GO:0003964 feature_id[317].value <= threshold=5.575642108917236
node_620: feature_name=GO:0046498 feature_id[203].value <= threshold=6.620223522186279
node_621: feature_name=GO:0002562 feature_id[25].value <= threshold=10.287704467773438
node_622: feature_name=GO:0006919 feature_id[246].value <= threshold=5.801334381103516
node_623: feature_name=GO:0001889 feature_id[387].value > threshold=2.679414987564087
node_785: feature_name=GO:0030887 feature_id[274].value <= threshold=1.1379476189613342
node_786: feature_name=GO:1902564 feature_id[85].value <= threshold=1.4637635350227356
node_787: feature_name=GO:0045402 feature_id[286].value <= threshold=1.5249269604682922
node_788: feature_name=GO:0034614 feature_id[655].value <= threshold=9.142115116119385
node_789: feature_name=GO:0000783 feature_id[191].value <= threshold=1.5124736428260803
node_790: feature_name=GO:0048145 feature_id[348].value > threshold=0.026725558564066887
node_794: feature_name=GO:0044092 feature_id[432].value <= threshold=31.540730476379395
node_795: feature_name=GO:0042771 feature_id[686].value <= threshold=6.047890663146973
node_796: feature_name=GO:0070301 feature_id[81].value <= threshold=5.959718465805054
node_797: feature_name=GO:0006809 feature_id[511].value <= threshold=9.000734329223633
node_798: feature_name=GO:0008150 feature_id[528].value > threshold=0.40075723826885223
node_800: feature_name=GO:0010663 feature_id[561].value > threshold=0.20622049272060394
node_802: feature_name=GO:0009636 feature_id[551].value <= threshold=0.12915228679776192
Class: positive genes

Rules_595 passed counts:1
node_0: feature_name=GO:0042113 feature_id[0].value <= threshold=13.408552169799805
node_1: feature_name=GO:0007568 feature_id[534].value <= threshold=5.0313897132873535
node_2: feature_name=GO:0002705 feature_id[541].value <= threshold=3.200145721435547
node_3: feature_name=GO:1901525 feature_id[576].value > threshold=0.4466460347175598
node_617: feature_name=GO:0005622 feature_id[233].value <= threshold=90.85730743408203

node_618: feature_name=GO:1903147 feature_id[200].value <= threshold=7.326428413391113
node_619: feature_name=GO:0003964 feature_id[317].value <= threshold=5.575642108917236
node_620: feature_name=GO:0046498 feature_id[203].value <= threshold=6.620223522186279
node_621: feature_name=GO:0002562 feature_id[25].value <= threshold=10.287704467773438
node_622: feature_name=GO:0006919 feature_id[246].value <= threshold=5.801334381103516
node_623: feature_name=GO:0001889 feature_id[387].value > threshold=2.679414987564087
node_785: feature_name=GO:0030887 feature_id[274].value <= threshold=1.1379476189613342
node_786: feature_name=GO:1902564 feature_id[85].value <= threshold=1.4637635350227356
node_787: feature_name=GO:0045402 feature_id[286].value <= threshold=1.5249269604682922
node_788: feature_name=GO:0034614 feature_id[655].value <= threshold=9.142115116119385
node_789: feature_name=GO:0000783 feature_id[191].value <= threshold=1.5124736428260803
node_790: feature_name=GO:0048145 feature_id[348].value > threshold=0.026725558564066887
node_794: feature_name=GO:0044092 feature_id[432].value <= threshold=31.540730476379395
node_795: feature_name=GO:0042771 feature_id[686].value <= threshold=6.047890663146973
node_796: feature_name=GO:0070301 feature_id[81].value <= threshold=5.959718465805054
node_797: feature_name=GO:0006809 feature_id[511].value <= threshold=9.000734329223633
node_798: feature_name=GO:0008150 feature_id[528].value > threshold=0.40075723826885223
node_800: feature_name=GO:0010663 feature_id[561].value <= threshold=0.20622049272060394
Class: positive genes

Rules_596 passed counts:1
node_0: feature_name=GO:0042113 feature_id[0].value <= threshold=13.408552169799805
node_1: feature_name=GO:0007568 feature_id[534].value <= threshold=5.0313897132873535
node_2: feature_name=GO:0002705 feature_id[541].value <= threshold=3.200145721435547
node_3: feature_name=GO:1901525 feature_id[576].value > threshold=0.4466460347175598
node_617: feature_name=GO:0005622 feature_id[233].value <= threshold=90.85730743408203
node_618: feature_name=GO:1903147 feature_id[200].value <= threshold=7.326428413391113
node_619: feature_name=GO:0003964 feature_id[317].value <= threshold=5.575642108917236
node_620: feature_name=GO:0046498 feature_id[203].value <= threshold=6.620223522186279
node_621: feature_name=GO:0002562 feature_id[25].value <= threshold=10.287704467773438
node_622: feature_name=GO:0006919 feature_id[246].value <= threshold=5.801334381103516
node_623: feature_name=GO:0001889 feature_id[387].value > threshold=2.679414987564087
node_785: feature_name=GO:0030887 feature_id[274].value <= threshold=1.1379476189613342
node_786: feature_name=GO:1902564 feature_id[85].value <= threshold=1.4637635350227356
node_787: feature_name=GO:0045402 feature_id[286].value <= threshold=1.5249269604682922

node_788: feature_name=GO:0034614 feature_id[655].value <= threshold=9.142115116119385
node_789: feature_name=GO:0000783 feature_id[191].value <= threshold=1.5124736428260803
node_790: feature_name=GO:0048145 feature_id[348].value > threshold=0.026725558564066887
node_794: feature_name=GO:0044092 feature_id[432].value <= threshold=31.540730476379395
node_795: feature_name=GO:0042771 feature_id[686].value <= threshold=6.047890663146973
node_796: feature_name=GO:0070301 feature_id[81].value <= threshold=5.959718465805054
node_797: feature_name=GO:0006809 feature_id[511].value <= threshold=9.000734329223633
node_798: feature_name=GO:0008150 feature_id[528].value <= threshold=0.40075723826885223
Class: positive genes

Rules_597 passed counts:1
node_0: feature_name=GO:0042113 feature_id[0].value <= threshold=13.408552169799805
node_1: feature_name=GO:0007568 feature_id[534].value <= threshold=5.0313897132873535
node_2: feature_name=GO:0002705 feature_id[541].value <= threshold=3.200145721435547
node_3: feature_name=GO:1901525 feature_id[576].value > threshold=0.4466460347175598
node_617: feature_name=GO:0005622 feature_id[233].value <= threshold=90.85730743408203
node_618: feature_name=GO:1903147 feature_id[200].value <= threshold=7.326428413391113
node_619: feature_name=GO:0003964 feature_id[317].value <= threshold=5.575642108917236
node_620: feature_name=GO:0046498 feature_id[203].value <= threshold=6.620223522186279
node_621: feature_name=GO:0002562 feature_id[25].value <= threshold=10.287704467773438
node_622: feature_name=GO:0006919 feature_id[246].value <= threshold=5.801334381103516
node_623: feature_name=GO:0001889 feature_id[387].value > threshold=2.679414987564087
node_785: feature_name=GO:0030887 feature_id[274].value <= threshold=1.1379476189613342
node_786: feature_name=GO:1902564 feature_id[85].value <= threshold=1.4637635350227356
node_787: feature_name=GO:0045402 feature_id[286].value <= threshold=1.5249269604682922
node_788: feature_name=GO:0034614 feature_id[655].value <= threshold=9.142115116119385
node_789: feature_name=GO:0000783 feature_id[191].value <= threshold=1.5124736428260803
node_790: feature_name=GO:0048145 feature_id[348].value <= threshold=0.026725558564066887
node_791: feature_name=GO:0032496 feature_id[112].value <= threshold=0.10570741072297096
Class: negative genes

Rules_598 passed counts:1
node_0: feature_name=GO:0042113 feature_id[0].value <= threshold=13.408552169799805
node_1: feature_name=GO:0007568 feature_id[534].value <= threshold=5.0313897132873535
node_2: feature_name=GO:0002705 feature_id[541].value <= threshold=3.200145721435547

node_3: feature_name=GO:1901525 feature_id[576].value > threshold=0.4466460347175598
node_617: feature_name=GO:0005622 feature_id[233].value <= threshold=90.85730743408203
node_618: feature_name=GO:1903147 feature_id[200].value <= threshold=7.326428413391113
node_619: feature_name=GO:0003964 feature_id[317].value <= threshold=5.575642108917236
node_620: feature_name=GO:0046498 feature_id[203].value <= threshold=6.620223522186279
node_621: feature_name=GO:0002562 feature_id[25].value <= threshold=10.287704467773438
node_622: feature_name=GO:0006919 feature_id[246].value <= threshold=5.801334381103516
node_623: feature_name=GO:0001889 feature_id[387].value <= threshold=2.679414987564087
node_624: feature_name=GO:0006555 feature_id[514].value <= threshold=7.819535493850708
node_625: feature_name=GO:0080134 feature_id[650].value > threshold=38.91967582702637
Class: positive genes

Rules_599 passed counts:1
node_0: feature_name=GO:0042113 feature_id[0].value <= threshold=13.408552169799805
node_1: feature_name=GO:0007568 feature_id[534].value <= threshold=5.0313897132873535
node_2: feature_name=GO:0002705 feature_id[541].value <= threshold=3.200145721435547
node_3: feature_name=GO:1901525 feature_id[576].value > threshold=0.4466460347175598
node_617: feature_name=GO:0005622 feature_id[233].value <= threshold=90.85730743408203
node_618: feature_name=GO:1903147 feature_id[200].value <= threshold=7.326428413391113
node_619: feature_name=GO:0003964 feature_id[317].value <= threshold=5.575642108917236
node_620: feature_name=GO:0046498 feature_id[203].value <= threshold=6.620223522186279
node_621: feature_name=GO:0002562 feature_id[25].value <= threshold=10.287704467773438
node_622: feature_name=GO:0006919 feature_id[246].value <= threshold=5.801334381103516
node_623: feature_name=GO:0001889 feature_id[387].value <= threshold=2.679414987564087
node_624: feature_name=GO:0006555 feature_id[514].value <= threshold=7.819535493850708
node_625: feature_name=GO:0080134 feature_id[650].value <= threshold=38.91967582702637
node_626: feature_name=GO:0042127 feature_id[480].value > threshold=37.72536659240723
Class: positive genes

Rules_600 passed counts:1
node_0: feature_name=GO:0042113 feature_id[0].value <= threshold=13.408552169799805
node_1: feature_name=GO:0007568 feature_id[534].value <= threshold=5.0313897132873535
node_2: feature_name=GO:0002705 feature_id[541].value <= threshold=3.200145721435547
node_3: feature_name=GO:1901525 feature_id[576].value > threshold=0.4466460347175598
node_617: feature_name=GO:0005622 feature_id[233].value <= threshold=90.85730743408203

node_618: feature_name=GO:1903147 feature_id[200].value <= threshold=7.326428413391113
node_619: feature_name=GO:0003964 feature_id[317].value <= threshold=5.575642108917236
node_620: feature_name=GO:0046498 feature_id[203].value <= threshold=6.620223522186279
node_621: feature_name=GO:0002562 feature_id[25].value <= threshold=10.287704467773438
node_622: feature_name=GO:0006919 feature_id[246].value <= threshold=5.801334381103516
node_623: feature_name=GO:0001889 feature_id[387].value <= threshold=2.679414987564087
node_624: feature_name=GO:0006555 feature_id[514].value <= threshold=7.819535493850708
node_625: feature_name=GO:0080134 feature_id[650].value <= threshold=38.91967582702637
node_626: feature_name=GO:0042127 feature_id[480].value <= threshold=37.72536659240723
node_627: feature_name=GO:2001251 feature_id[220].value > threshold=32.564422607421875
Class: positive genes

Rules_601 passed counts:1
node_0: feature_name=GO:0042113 feature_id[0].value <= threshold=13.408552169799805
node_1: feature_name=GO:0007568 feature_id[534].value <= threshold=5.0313897132873535
node_2: feature_name=GO:0002705 feature_id[541].value <= threshold=3.200145721435547
node_3: feature_name=GO:1901525 feature_id[576].value > threshold=0.4466460347175598
node_617: feature_name=GO:0005622 feature_id[233].value <= threshold=90.85730743408203
node_618: feature_name=GO:1903147 feature_id[200].value <= threshold=7.326428413391113
node_619: feature_name=GO:0003964 feature_id[317].value <= threshold=5.575642108917236
node_620: feature_name=GO:0046498 feature_id[203].value <= threshold=6.620223522186279
node_621: feature_name=GO:0002562 feature_id[25].value <= threshold=10.287704467773438
node_622: feature_name=GO:0006919 feature_id[246].value <= threshold=5.801334381103516
node_623: feature_name=GO:0001889 feature_id[387].value <= threshold=2.679414987564087
node_624: feature_name=GO:0006555 feature_id[514].value <= threshold=7.819535493850708
node_625: feature_name=GO:0080134 feature_id[650].value <= threshold=38.91967582702637
node_626: feature_name=GO:0042127 feature_id[480].value <= threshold=37.72536659240723
node_627: feature_name=GO:2001251 feature_id[220].value <= threshold=32.564422607421875
node_628: feature_name=GO:0002829 feature_id[493].value > threshold=2.444612741470337
Class: positive genes

Rules_602 passed counts:1
node_0: feature_name=GO:0042113 feature_id[0].value <= threshold=13.408552169799805
node_1: feature_name=GO:0007568 feature_id[534].value <= threshold=5.0313897132873535
node_2: feature_name=GO:0002705 feature_id[541].value <= threshold=3.200145721435547

node_3: feature_name=GO:1901525 feature_id[576].value > threshold=0.4466460347175598
node_617: feature_name=GO:0005622 feature_id[233].value <= threshold=90.85730743408203
node_618: feature_name=GO:1903147 feature_id[200].value <= threshold=7.326428413391113
node_619: feature_name=GO:0003964 feature_id[317].value <= threshold=5.575642108917236
node_620: feature_name=GO:0046498 feature_id[203].value <= threshold=6.620223522186279
node_621: feature_name=GO:0002562 feature_id[25].value <= threshold=10.287704467773438
node_622: feature_name=GO:0006919 feature_id[246].value <= threshold=5.801334381103516
node_623: feature_name=GO:0001889 feature_id[387].value <= threshold=2.679414987564087
node_624: feature_name=GO:0006555 feature_id[514].value <= threshold=7.819535493850708
node_625: feature_name=GO:0080134 feature_id[650].value <= threshold=38.91967582702637
node_626: feature_name=GO:0042127 feature_id[480].value <= threshold=37.72536659240723
node_627: feature_name=GO:2001251 feature_id[220].value <= threshold=32.564422607421875
node_628: feature_name=GO:0002829 feature_id[493].value <= threshold=2.444612741470337
node_629: feature_name=GO:0008588 feature_id[26].value > threshold=2.3601274490356445
Class: positive genes

Rules_603 passed counts:1
node_0: feature_name=GO:0042113 feature_id[0].value <= threshold=13.408552169799805
node_1: feature_name=GO:0007568 feature_id[534].value <= threshold=5.0313897132873535
node_2: feature_name=GO:0002705 feature_id[541].value <= threshold=3.200145721435547
node_3: feature_name=GO:1901525 feature_id[576].value > threshold=0.4466460347175598
node_617: feature_name=GO:0005622 feature_id[233].value <= threshold=90.85730743408203
node_618: feature_name=GO:1903147 feature_id[200].value <= threshold=7.326428413391113
node_619: feature_name=GO:0003964 feature_id[317].value <= threshold=5.575642108917236
node_620: feature_name=GO:0046498 feature_id[203].value <= threshold=6.620223522186279
node_621: feature_name=GO:0002562 feature_id[25].value <= threshold=10.287704467773438
node_622: feature_name=GO:0006919 feature_id[246].value <= threshold=5.801334381103516
node_623: feature_name=GO:0001889 feature_id[387].value <= threshold=2.679414987564087
node_624: feature_name=GO:0006555 feature_id[514].value <= threshold=7.819535493850708
node_625: feature_name=GO:0080134 feature_id[650].value <= threshold=38.91967582702637
node_626: feature_name=GO:0042127 feature_id[480].value <= threshold=37.72536659240723
node_627: feature_name=GO:2001251 feature_id[220].value <= threshold=32.564422607421875
node_628: feature_name=GO:0002829 feature_id[493].value <= threshold=2.444612741470337
node_629: feature_name=GO:0008588 feature_id[26].value <= threshold=2.3601274490356445
node_630: feature_name=GO:0033151 feature_id[7].value > threshold=5.134376287460327

Class: positive genes

Rules_604 passed counts:1
node_0: feature_name=GO:0042113 feature_id[0].value <= threshold=13.408552169799805
node_1: feature_name=GO:0007568 feature_id[534].value <= threshold=5.0313897132873535
node_2: feature_name=GO:0002705 feature_id[541].value <= threshold=3.200145721435547
node_3: feature_name=GO:1901525 feature_id[576].value > threshold=0.4466460347175598
node_617: feature_name=GO:0005622 feature_id[233].value <= threshold=90.85730743408203
node_618: feature_name=GO:1903147 feature_id[200].value <= threshold=7.326428413391113
node_619: feature_name=GO:0003964 feature_id[317].value <= threshold=5.575642108917236
node_620: feature_name=GO:0046498 feature_id[203].value <= threshold=6.620223522186279
node_621: feature_name=GO:0002562 feature_id[25].value <= threshold=10.287704467773438
node_622: feature_name=GO:0006919 feature_id[246].value <= threshold=5.801334381103516
node_623: feature_name=GO:0001889 feature_id[387].value <= threshold=2.679414987564087
node_624: feature_name=GO:0006555 feature_id[514].value <= threshold=7.819535493850708
node_625: feature_name=GO:0080134 feature_id[650].value <= threshold=38.91967582702637
node_626: feature_name=GO:0042127 feature_id[480].value <= threshold=37.72536659240723
node_627: feature_name=GO:2001251 feature_id[220].value <= threshold=32.564422607421875
node_628: feature_name=GO:0002829 feature_id[493].value <= threshold=2.444612741470337
node_629: feature_name=GO:0008588 feature_id[26].value <= threshold=2.3601274490356445
node_630: feature_name=GO:0033151 feature_id[7].value <= threshold=5.134376287460327
node_631: feature_name=GO:0048145 feature_id[348].value > threshold=8.836549282073975
Class: positive genes

Rules_605 passed counts:1
node_0: feature_name=GO:0042113 feature_id[0].value <= threshold=13.408552169799805
node_1: feature_name=GO:0007568 feature_id[534].value <= threshold=5.0313897132873535
node_2: feature_name=GO:0002705 feature_id[541].value <= threshold=3.200145721435547
node_3: feature_name=GO:1901525 feature_id[576].value > threshold=0.4466460347175598
node_617: feature_name=GO:0005622 feature_id[233].value <= threshold=90.85730743408203
node_618: feature_name=GO:1903147 feature_id[200].value <= threshold=7.326428413391113
node_619: feature_name=GO:0003964 feature_id[317].value <= threshold=5.575642108917236
node_620: feature_name=GO:0046498 feature_id[203].value <= threshold=6.620223522186279
node_621: feature_name=GO:0002562 feature_id[25].value <= threshold=10.287704467773438
node_622: feature_name=GO:0006919 feature_id[246].value <= threshold=5.801334381103516

node_623: feature_name=GO:0001889 feature_id[387].value <= threshold=2.679414987564087
node_624: feature_name=GO:0006555 feature_id[514].value <= threshold=7.819535493850708
node_625: feature_name=GO:0080134 feature_id[650].value <= threshold=38.91967582702637
node_626: feature_name=GO:0042127 feature_id[480].value <= threshold=37.72536659240723
node_627: feature_name=GO:2001251 feature_id[220].value <= threshold=32.564422607421875
node_628: feature_name=GO:0002829 feature_id[493].value <= threshold=2.444612741470337
node_629: feature_name=GO:0008588 feature_id[26].value <= threshold=2.3601274490356445
node_630: feature_name=GO:0033151 feature_id[7].value <= threshold=5.134376287460327
node_631: feature_name=GO:0048145 feature_id[348].value <= threshold=8.836549282073975
node_632: feature_name=GO:0030887 feature_id[274].value > threshold=1.7000296115875244
Class: positive genes

Rules_606 passed counts:1
node_0: feature_name=GO:0042113 feature_id[0].value <= threshold=13.408552169799805
node_1: feature_name=GO:0007568 feature_id[534].value <= threshold=5.0313897132873535
node_2: feature_name=GO:0002705 feature_id[541].value <= threshold=3.200145721435547
node_3: feature_name=GO:1901525 feature_id[576].value > threshold=0.4466460347175598
node_617: feature_name=GO:0005622 feature_id[233].value <= threshold=90.85730743408203
node_618: feature_name=GO:1903147 feature_id[200].value <= threshold=7.326428413391113
node_619: feature_name=GO:0003964 feature_id[317].value <= threshold=5.575642108917236
node_620: feature_name=GO:0046498 feature_id[203].value <= threshold=6.620223522186279
node_621: feature_name=GO:0002562 feature_id[25].value <= threshold=10.287704467773438
node_622: feature_name=GO:0006919 feature_id[246].value <= threshold=5.801334381103516
node_623: feature_name=GO:0001889 feature_id[387].value <= threshold=2.679414987564087
node_624: feature_name=GO:0006555 feature_id[514].value <= threshold=7.819535493850708
node_625: feature_name=GO:0080134 feature_id[650].value <= threshold=38.91967582702637
node_626: feature_name=GO:0042127 feature_id[480].value <= threshold=37.72536659240723
node_627: feature_name=GO:2001251 feature_id[220].value <= threshold=32.564422607421875
node_628: feature_name=GO:0002829 feature_id[493].value <= threshold=2.444612741470337
node_629: feature_name=GO:0008588 feature_id[26].value <= threshold=2.3601274490356445
node_630: feature_name=GO:0033151 feature_id[7].value <= threshold=5.134376287460327
node_631: feature_name=GO:0048145 feature_id[348].value <= threshold=8.836549282073975
node_632: feature_name=GO:0030887 feature_id[274].value <= threshold=1.7000296115875244
node_633: feature_name=GO:0097190 feature_id[117].value > threshold=33.02078819274902
Class: positive genes

Rules_607 passed counts:1
node_0: feature_name=GO:0042113 feature_id[0].value <= threshold=13.408552169799805
node_1: feature_name=GO:0007568 feature_id[534].value <= threshold=5.0313897132873535
node_2: feature_name=GO:0002705 feature_id[541].value <= threshold=3.200145721435547
node_3: feature_name=GO:1901525 feature_id[576].value > threshold=0.4466460347175598
node_617: feature_name=GO:0005622 feature_id[233].value <= threshold=90.85730743408203
node_618: feature_name=GO:1903147 feature_id[200].value <= threshold=7.326428413391113
node_619: feature_name=GO:0003964 feature_id[317].value <= threshold=5.575642108917236
node_620: feature_name=GO:0046498 feature_id[203].value <= threshold=6.620223522186279
node_621: feature_name=GO:0002562 feature_id[25].value <= threshold=10.287704467773438
node_622: feature_name=GO:0006919 feature_id[246].value <= threshold=5.801334381103516
node_623: feature_name=GO:0001889 feature_id[387].value <= threshold=2.679414987564087
node_624: feature_name=GO:0006555 feature_id[514].value <= threshold=7.819535493850708
node_625: feature_name=GO:0080134 feature_id[650].value <= threshold=38.91967582702637
node_626: feature_name=GO:0042127 feature_id[480].value <= threshold=37.72536659240723
node_627: feature_name=GO:2001251 feature_id[220].value <= threshold=32.564422607421875
node_628: feature_name=GO:0002829 feature_id[493].value <= threshold=2.444612741470337
node_629: feature_name=GO:0008588 feature_id[26].value <= threshold=2.3601274490356445
node_630: feature_name=GO:0033151 feature_id[7].value <= threshold=5.134376287460327
node_631: feature_name=GO:0048145 feature_id[348].value <= threshold=8.836549282073975
node_632: feature_name=GO:0030887 feature_id[274].value <= threshold=1.7000296115875244
node_633: feature_name=GO:0097190 feature_id[117].value <= threshold=33.02078819274902
node_634: feature_name=GO:0046685 feature_id[782].value > threshold=8.040813446044922
Class: positive genes

Rules_608 passed counts:1
node_0: feature_name=GO:0042113 feature_id[0].value <= threshold=13.408552169799805
node_1: feature_name=GO:0007568 feature_id[534].value <= threshold=5.0313897132873535
node_2: feature_name=GO:0002705 feature_id[541].value <= threshold=3.200145721435547
node_3: feature_name=GO:1901525 feature_id[576].value > threshold=0.4466460347175598
node_617: feature_name=GO:0005622 feature_id[233].value <= threshold=90.85730743408203
node_618: feature_name=GO:1903147 feature_id[200].value <= threshold=7.326428413391113
node_619: feature_name=GO:0003964 feature_id[317].value <= threshold=5.575642108917236
node_620: feature_name=GO:0046498 feature_id[203].value <= threshold=6.620223522186279

node_621: feature_name=GO:0002562 feature_id[25].value <= threshold=10.287704467773438
node_622: feature_name=GO:0006919 feature_id[246].value <= threshold=5.801334381103516
node_623: feature_name=GO:0001889 feature_id[387].value <= threshold=2.679414987564087
node_624: feature_name=GO:0006555 feature_id[514].value <= threshold=7.819535493850708
node_625: feature_name=GO:0080134 feature_id[650].value <= threshold=38.91967582702637
node_626: feature_name=GO:0042127 feature_id[480].value <= threshold=37.72536659240723
node_627: feature_name=GO:2001251 feature_id[220].value <= threshold=32.564422607421875
node_628: feature_name=GO:0002829 feature_id[493].value <= threshold=2.444612741470337
node_629: feature_name=GO:0008588 feature_id[26].value <= threshold=2.3601274490356445
node_630: feature_name=GO:0033151 feature_id[7].value <= threshold=5.134376287460327
node_631: feature_name=GO:0048145 feature_id[348].value <= threshold=8.836549282073975
node_632: feature_name=GO:0030887 feature_id[274].value <= threshold=1.7000296115875244
node_633: feature_name=GO:0097190 feature_id[117].value <= threshold=33.02078819274902
node_634: feature_name=GO:0046685 feature_id[782].value <= threshold=8.040813446044922
node_635: feature_name=GO:0006346 feature_id[236].value <= threshold=6.384642839431763
node_636: feature_name=GO:0048537 feature_id[66].value <= threshold=3.1739262342453003
node_637: feature_name=GO:0070245 feature_id[326].value <= threshold=3.0491198301315308
node_638: feature_name=GO:0038065 feature_id[670].value <= threshold=2.0590850114822388
node_639: feature_name=GO:0005488 feature_id[187].value > threshold=84.80076217651367
node_755: feature_name=GO:0031049 feature_id[5].value > threshold=0.707693487405777
node_759: feature_name=GO:0002832 feature_id[491].value <= threshold=0.07645885832607746
Class: negative genes

Rules_609 passed counts:1
node_0: feature_name=GO:0042113 feature_id[0].value <= threshold=13.408552169799805
node_1: feature_name=GO:0007568 feature_id[534].value <= threshold=5.0313897132873535
node_2: feature_name=GO:0002705 feature_id[541].value <= threshold=3.200145721435547
node_3: feature_name=GO:1901525 feature_id[576].value > threshold=0.4466460347175598
node_617: feature_name=GO:0005622 feature_id[233].value <= threshold=90.85730743408203
node_618: feature_name=GO:1903147 feature_id[200].value <= threshold=7.326428413391113
node_619: feature_name=GO:0003964 feature_id[317].value <= threshold=5.575642108917236
node_620: feature_name=GO:0046498 feature_id[203].value <= threshold=6.620223522186279
node_621: feature_name=GO:0002562 feature_id[25].value <= threshold=10.287704467773438
node_622: feature_name=GO:0006919 feature_id[246].value <= threshold=5.801334381103516
node_623: feature_name=GO:0001889 feature_id[387].value <= threshold=2.679414987564087

node_624: feature_name=GO:0006555 feature_id[514].value <= threshold=7.819535493850708
node_625: feature_name=GO:0080134 feature_id[650].value <= threshold=38.91967582702637
node_626: feature_name=GO:0042127 feature_id[480].value <= threshold=37.72536659240723
node_627: feature_name=GO:2001251 feature_id[220].value <= threshold=32.564422607421875
node_628: feature_name=GO:0002829 feature_id[493].value <= threshold=2.444612741470337
node_629: feature_name=GO:0008588 feature_id[26].value <= threshold=2.3601274490356445
node_630: feature_name=GO:0033151 feature_id[7].value <= threshold=5.134376287460327
node_631: feature_name=GO:0048145 feature_id[348].value <= threshold=8.836549282073975
node_632: feature_name=GO:0030887 feature_id[274].value <= threshold=1.7000296115875244
node_633: feature_name=GO:0097190 feature_id[117].value <= threshold=33.02078819274902
node_634: feature_name=GO:0046685 feature_id[782].value <= threshold=8.040813446044922
node_635: feature_name=GO:0006346 feature_id[236].value <= threshold=6.384642839431763
node_636: feature_name=GO:0048537 feature_id[66].value <= threshold=3.1739262342453003
node_637: feature_name=GO:0070245 feature_id[326].value <= threshold=3.0491198301315308
node_638: feature_name=GO:0038065 feature_id[670].value <= threshold=2.0590850114822388
node_639: feature_name=GO:0005488 feature_id[187].value > threshold=84.80076217651367
node_755: feature_name=GO:0031049 feature_id[5].value <= threshold=0.707693487405777
node_756: feature_name=GO:0006306 feature_id[345].value > threshold=15.554558753967285
Class: positive genes

Rules_610 passed counts:1
node_0: feature_name=GO:0042113 feature_id[0].value <= threshold=13.408552169799805
node_1: feature_name=GO:0007568 feature_id[534].value <= threshold=5.0313897132873535
node_2: feature_name=GO:0002705 feature_id[541].value <= threshold=3.200145721435547
node_3: feature_name=GO:1901525 feature_id[576].value > threshold=0.4466460347175598
node_617: feature_name=GO:0005622 feature_id[233].value <= threshold=90.85730743408203
node_618: feature_name=GO:1903147 feature_id[200].value <= threshold=7.326428413391113
node_619: feature_name=GO:0003964 feature_id[317].value <= threshold=5.575642108917236
node_620: feature_name=GO:0046498 feature_id[203].value <= threshold=6.620223522186279
node_621: feature_name=GO:0002562 feature_id[25].value <= threshold=10.287704467773438
node_622: feature_name=GO:0006919 feature_id[246].value <= threshold=5.801334381103516
node_623: feature_name=GO:0001889 feature_id[387].value <= threshold=2.679414987564087
node_624: feature_name=GO:0006555 feature_id[514].value <= threshold=7.819535493850708
node_625: feature_name=GO:0080134 feature_id[650].value <= threshold=38.91967582702637
node_626: feature_name=GO:0042127 feature_id[480].value <= threshold=37.72536659240723

node_627: feature_name=GO:2001251 feature_id[220].value <= threshold=32.564422607421875
node_628: feature_name=GO:0002829 feature_id[493].value <= threshold=2.444612741470337
node_629: feature_name=GO:0008588 feature_id[26].value <= threshold=2.3601274490356445
node_630: feature_name=GO:0033151 feature_id[7].value <= threshold=5.134376287460327
node_631: feature_name=GO:0048145 feature_id[348].value <= threshold=8.836549282073975
node_632: feature_name=GO:0030887 feature_id[274].value <= threshold=1.7000296115875244
node_633: feature_name=GO:0097190 feature_id[117].value <= threshold=33.02078819274902
node_634: feature_name=GO:0046685 feature_id[782].value <= threshold=8.040813446044922
node_635: feature_name=GO:0006346 feature_id[236].value <= threshold=6.384642839431763
node_636: feature_name=GO:0048537 feature_id[66].value <= threshold=3.1739262342453003
node_637: feature_name=GO:0070245 feature_id[326].value <= threshold=3.0491198301315308
node_638: feature_name=GO:0038065 feature_id[670].value <= threshold=2.0590850114822388
node_639: feature_name=GO:0005488 feature_id[187].value <= threshold=84.80076217651367
node_640: feature_name=GO:0060249 feature_id[390].value > threshold=0.07226398587226868
node_652: feature_name=GO:0006974 feature_id[516].value > threshold=5.17782768838515e-06
node_654: feature_name=hsa05210 feature_id[358].value > threshold=18.730005264282227
node_752: feature_name=GO:0050776 feature_id[803].value > threshold=12.918556213378906
Class: negative genes

Rules_611 passed counts:1
node_0: feature_name=GO:0042113 feature_id[0].value <= threshold=13.408552169799805
node_1: feature_name=GO:0007568 feature_id[534].value <= threshold=5.0313897132873535
node_2: feature_name=GO:0002705 feature_id[541].value <= threshold=3.200145721435547
node_3: feature_name=GO:1901525 feature_id[576].value > threshold=0.4466460347175598
node_617: feature_name=GO:0005622 feature_id[233].value <= threshold=90.85730743408203
node_618: feature_name=GO:1903147 feature_id[200].value <= threshold=7.326428413391113
node_619: feature_name=GO:0003964 feature_id[317].value <= threshold=5.575642108917236
node_620: feature_name=GO:0046498 feature_id[203].value <= threshold=6.620223522186279
node_621: feature_name=GO:0002562 feature_id[25].value <= threshold=10.287704467773438
node_622: feature_name=GO:0006919 feature_id[246].value <= threshold=5.801334381103516
node_623: feature_name=GO:0001889 feature_id[387].value <= threshold=2.679414987564087
node_624: feature_name=GO:0006555 feature_id[514].value <= threshold=7.819535493850708
node_625: feature_name=GO:0080134 feature_id[650].value <= threshold=38.91967582702637
node_626: feature_name=GO:0042127 feature_id[480].value <= threshold=37.72536659240723
node_627: feature_name=GO:2001251 feature_id[220].value <= threshold=32.564422607421875

node_628: feature_name=GO:0002829 feature_id[493].value <= threshold=2.444612741470337
node_629: feature_name=GO:0008588 feature_id[26].value <= threshold=2.3601274490356445
node_630: feature_name=GO:0033151 feature_id[7].value <= threshold=5.134376287460327
node_631: feature_name=GO:0048145 feature_id[348].value <= threshold=8.836549282073975
node_632: feature_name=GO:0030887 feature_id[274].value <= threshold=1.7000296115875244
node_633: feature_name=GO:0097190 feature_id[117].value <= threshold=33.02078819274902
node_634: feature_name=GO:0046685 feature_id[782].value <= threshold=8.040813446044922
node_635: feature_name=GO:0006346 feature_id[236].value <= threshold=6.384642839431763
node_636: feature_name=GO:0048537 feature_id[66].value <= threshold=3.1739262342453003
node_637: feature_name=GO:0070245 feature_id[326].value <= threshold=3.0491198301315308
node_638: feature_name=GO:0038065 feature_id[670].value <= threshold=2.0590850114822388
node_639: feature_name=GO:0005488 feature_id[187].value <= threshold=84.80076217651367
node_640: feature_name=GO:0060249 feature_id[390].value > threshold=0.07226398587226868
node_652: feature_name=GO:0006974 feature_id[516].value > threshold=5.17782768838515e-06
node_654: feature_name=hsa05210 feature_id[358].value > threshold=18.730005264282227
node_752: feature_name=GO:0050776 feature_id[803].value <= threshold=12.918556213378906
Class: positive genes

Rules_612 passed counts:1
node_0: feature_name=GO:0042113 feature_id[0].value <= threshold=13.408552169799805
node_1: feature_name=GO:0007568 feature_id[534].value <= threshold=5.0313897132873535
node_2: feature_name=GO:0002705 feature_id[541].value <= threshold=3.200145721435547
node_3: feature_name=GO:1901525 feature_id[576].value > threshold=0.4466460347175598
node_617: feature_name=GO:0005622 feature_id[233].value <= threshold=90.85730743408203
node_618: feature_name=GO:1903147 feature_id[200].value <= threshold=7.326428413391113
node_619: feature_name=GO:0003964 feature_id[317].value <= threshold=5.575642108917236
node_620: feature_name=GO:0046498 feature_id[203].value <= threshold=6.620223522186279
node_621: feature_name=GO:0002562 feature_id[25].value <= threshold=10.287704467773438
node_622: feature_name=GO:0006919 feature_id[246].value <= threshold=5.801334381103516
node_623: feature_name=GO:0001889 feature_id[387].value <= threshold=2.679414987564087
node_624: feature_name=GO:0006555 feature_id[514].value <= threshold=7.819535493850708
node_625: feature_name=GO:0080134 feature_id[650].value <= threshold=38.91967582702637
node_626: feature_name=GO:0042127 feature_id[480].value <= threshold=37.72536659240723
node_627: feature_name=GO:2001251 feature_id[220].value <= threshold=32.564422607421875
node_628: feature_name=GO:0002829 feature_id[493].value <= threshold=2.444612741470337

node_629: feature_name=GO:0008588 feature_id[26].value <= threshold=2.3601274490356445
node_630: feature_name=GO:0033151 feature_id[7].value <= threshold=5.134376287460327
node_631: feature_name=GO:0048145 feature_id[348].value <= threshold=8.836549282073975
node_632: feature_name=GO:0030887 feature_id[274].value <= threshold=1.7000296115875244
node_633: feature_name=GO:0097190 feature_id[117].value <= threshold=33.02078819274902
node_634: feature_name=GO:0046685 feature_id[782].value <= threshold=8.040813446044922
node_635: feature_name=GO:0006346 feature_id[236].value <= threshold=6.384642839431763
node_636: feature_name=GO:0048537 feature_id[66].value <= threshold=3.1739262342453003
node_637: feature_name=GO:0070245 feature_id[326].value <= threshold=3.0491198301315308
node_638: feature_name=GO:0038065 feature_id[670].value <= threshold=2.0590850114822388
node_639: feature_name=GO:0005488 feature_id[187].value <= threshold=84.80076217651367
node_640: feature_name=GO:0060249 feature_id[390].value > threshold=0.07226398587226868
node_652: feature_name=GO:0006974 feature_id[516].value > threshold=5.17782768838515e-06
node_654: feature_name=hsa05210 feature_id[358].value <= threshold=18.730005264282227
node_655: feature_name=GO:0071850 feature_id[40].value > threshold=5.475317001342773
node_749: feature_name=GO:0048146 feature_id[640].value > threshold=0.2769286334514618
Class: positive genes

Rules_613 passed counts:1
node_0: feature_name=GO:0042113 feature_id[0].value <= threshold=13.408552169799805
node_1: feature_name=GO:0007568 feature_id[534].value <= threshold=5.0313897132873535
node_2: feature_name=GO:0002705 feature_id[541].value <= threshold=3.200145721435547
node_3: feature_name=GO:1901525 feature_id[576].value > threshold=0.4466460347175598
node_617: feature_name=GO:0005622 feature_id[233].value <= threshold=90.85730743408203
node_618: feature_name=GO:1903147 feature_id[200].value <= threshold=7.326428413391113
node_619: feature_name=GO:0003964 feature_id[317].value <= threshold=5.575642108917236
node_620: feature_name=GO:0046498 feature_id[203].value <= threshold=6.620223522186279
node_621: feature_name=GO:0002562 feature_id[25].value <= threshold=10.287704467773438
node_622: feature_name=GO:0006919 feature_id[246].value <= threshold=5.801334381103516
node_623: feature_name=GO:0001889 feature_id[387].value <= threshold=2.679414987564087
node_624: feature_name=GO:0006555 feature_id[514].value <= threshold=7.819535493850708
node_625: feature_name=GO:0080134 feature_id[650].value <= threshold=38.91967582702637
node_626: feature_name=GO:0042127 feature_id[480].value <= threshold=37.72536659240723
node_627: feature_name=GO:2001251 feature_id[220].value <= threshold=32.564422607421875
node_628: feature_name=GO:0002829 feature_id[493].value <= threshold=2.444612741470337

node_629: feature_name=GO:0008588 feature_id[26].value <= threshold=2.3601274490356445
node_630: feature_name=GO:0033151 feature_id[7].value <= threshold=5.134376287460327
node_631: feature_name=GO:0048145 feature_id[348].value <= threshold=8.836549282073975
node_632: feature_name=GO:0030887 feature_id[274].value <= threshold=1.7000296115875244
node_633: feature_name=GO:0097190 feature_id[117].value <= threshold=33.02078819274902
node_634: feature_name=GO:0046685 feature_id[782].value <= threshold=8.040813446044922
node_635: feature_name=GO:0006346 feature_id[236].value <= threshold=6.384642839431763
node_636: feature_name=GO:0048537 feature_id[66].value <= threshold=3.1739262342453003
node_637: feature_name=GO:0070245 feature_id[326].value <= threshold=3.0491198301315308
node_638: feature_name=GO:0038065 feature_id[670].value <= threshold=2.0590850114822388
node_639: feature_name=GO:0005488 feature_id[187].value <= threshold=84.80076217651367
node_640: feature_name=GO:0060249 feature_id[390].value > threshold=0.07226398587226868
node_652: feature_name=GO:0006974 feature_id[516].value > threshold=5.17782768838515e-06
node_654: feature_name=hsa05210 feature_id[358].value <= threshold=18.730005264282227
node_655: feature_name=GO:0071850 feature_id[40].value > threshold=5.475317001342773
node_749: feature_name=GO:0048146 feature_id[640].value <= threshold=0.2769286334514618
Class: negative genes

Rules_614 passed counts:1
node_0: feature_name=GO:0042113 feature_id[0].value <= threshold=13.408552169799805
node_1: feature_name=GO:0007568 feature_id[534].value <= threshold=5.0313897132873535
node_2: feature_name=GO:0002705 feature_id[541].value <= threshold=3.200145721435547
node_3: feature_name=GO:1901525 feature_id[576].value > threshold=0.4466460347175598
node_617: feature_name=GO:0005622 feature_id[233].value <= threshold=90.85730743408203
node_618: feature_name=GO:1903147 feature_id[200].value <= threshold=7.326428413391113
node_619: feature_name=GO:0003964 feature_id[317].value <= threshold=5.575642108917236
node_620: feature_name=GO:0046498 feature_id[203].value <= threshold=6.620223522186279
node_621: feature_name=GO:0002562 feature_id[25].value <= threshold=10.287704467773438
node_622: feature_name=GO:0006919 feature_id[246].value <= threshold=5.801334381103516
node_623: feature_name=GO:0001889 feature_id[387].value <= threshold=2.679414987564087
node_624: feature_name=GO:0006555 feature_id[514].value <= threshold=7.819535493850708
node_625: feature_name=GO:0080134 feature_id[650].value <= threshold=38.91967582702637
node_626: feature_name=GO:0042127 feature_id[480].value <= threshold=37.72536659240723
node_627: feature_name=GO:2001251 feature_id[220].value <= threshold=32.564422607421875
node_628: feature_name=GO:0002829 feature_id[493].value <= threshold=2.444612741470337

node_629: feature_name=GO:0008588 feature_id[26].value <= threshold=2.3601274490356445
node_630: feature_name=GO:0033151 feature_id[7].value <= threshold=5.134376287460327
node_631: feature_name=GO:0048145 feature_id[348].value <= threshold=8.836549282073975
node_632: feature_name=GO:0030887 feature_id[274].value <= threshold=1.7000296115875244
node_633: feature_name=GO:0097190 feature_id[117].value <= threshold=33.02078819274902
node_634: feature_name=GO:0046685 feature_id[782].value <= threshold=8.040813446044922
node_635: feature_name=GO:0006346 feature_id[236].value <= threshold=6.384642839431763
node_636: feature_name=GO:0048537 feature_id[66].value <= threshold=3.1739262342453003
node_637: feature_name=GO:0070245 feature_id[326].value <= threshold=3.0491198301315308
node_638: feature_name=GO:0038065 feature_id[670].value <= threshold=2.0590850114822388
node_639: feature_name=GO:0005488 feature_id[187].value <= threshold=84.80076217651367
node_640: feature_name=GO:0060249 feature_id[390].value > threshold=0.07226398587226868
node_652: feature_name=GO:0006974 feature_id[516].value > threshold=5.17782768838515e-06
node_654: feature_name=hsa05210 feature_id[358].value <= threshold=18.730005264282227
node_655: feature_name=GO:0071850 feature_id[40].value <= threshold=5.475317001342773
node_656: feature_name=GO:1904029 feature_id[208].value > threshold=26.575013160705566
node_746: feature_name=GO:0046386 feature_id[767].value > threshold=0.10460815206170082
Class: positive genes

Rules_615 passed counts:1
node_0: feature_name=GO:0042113 feature_id[0].value <= threshold=13.408552169799805
node_1: feature_name=GO:0007568 feature_id[534].value <= threshold=5.0313897132873535
node_2: feature_name=GO:0002705 feature_id[541].value <= threshold=3.200145721435547
node_3: feature_name=GO:1901525 feature_id[576].value > threshold=0.4466460347175598
node_617: feature_name=GO:0005622 feature_id[233].value <= threshold=90.85730743408203
node_618: feature_name=GO:1903147 feature_id[200].value <= threshold=7.326428413391113
node_619: feature_name=GO:0003964 feature_id[317].value <= threshold=5.575642108917236
node_620: feature_name=GO:0046498 feature_id[203].value <= threshold=6.620223522186279
node_621: feature_name=GO:0002562 feature_id[25].value <= threshold=10.287704467773438
node_622: feature_name=GO:0006919 feature_id[246].value <= threshold=5.801334381103516
node_623: feature_name=GO:0001889 feature_id[387].value <= threshold=2.679414987564087
node_624: feature_name=GO:0006555 feature_id[514].value <= threshold=7.819535493850708
node_625: feature_name=GO:0080134 feature_id[650].value <= threshold=38.91967582702637
node_626: feature_name=GO:0042127 feature_id[480].value <= threshold=37.72536659240723
node_627: feature_name=GO:2001251 feature_id[220].value <= threshold=32.564422607421875

node_628: feature_name=GO:0002829	feature_id[493].value <= threshold=2.444612741470337
node_629: feature_name=GO:0008588	feature_id[26].value <= threshold=2.3601274490356445
node_630: feature_name=GO:0033151	feature_id[7].value <= threshold=5.134376287460327
node_631: feature_name=GO:0048145	feature_id[348].value <= threshold=8.836549282073975
node_632: feature_name=GO:0030887	feature_id[274].value <= threshold=1.7000296115875244
node_633: feature_name=GO:0097190	feature_id[117].value <= threshold=33.02078819274902
node_634: feature_name=GO:0046685	feature_id[782].value <= threshold=8.040813446044922
node_635: feature_name=GO:0006346	feature_id[236].value <= threshold=6.384642839431763
node_636: feature_name=GO:0048537	feature_id[66].value <= threshold=3.1739262342453003
node_637: feature_name=GO:0070245	feature_id[326].value <= threshold=3.0491198301315308
node_638: feature_name=GO:0038065	feature_id[670].value <= threshold=2.0590850114822388
node_639: feature_name=GO:0005488	feature_id[187].value <= threshold=84.80076217651367
node_640: feature_name=GO:0060249	feature_id[390].value > threshold=0.07226398587226868
node_652: feature_name=GO:0006974	feature_id[516].value > threshold=5.17782768838515e-06
node_654: feature_name=hsa05210	feature_id[358].value <= threshold=18.730005264282227
node_655: feature_name=GO:0071850	feature_id[40].value <= threshold=5.475317001342773
node_656: feature_name=GO:1904029	feature_id[208].value > threshold=26.575013160705566
node_746: feature_name=GO:0046386	feature_id[767].value <= threshold=0.10460815206170082
Class: negative genes	

Rules_616	passed counts:1
node_0: feature_name=GO:0042113	feature_id[0].value <= threshold=13.408552169799805
node_1: feature_name=GO:0007568	feature_id[534].value <= threshold=5.0313897132873535
node_2: feature_name=GO:0002705	feature_id[541].value <= threshold=3.200145721435547
node_3: feature_name=GO:1901525	feature_id[576].value > threshold=0.4466460347175598
node_617: feature_name=GO:0005622	feature_id[233].value <= threshold=90.85730743408203
node_618: feature_name=GO:1903147	feature_id[200].value <= threshold=7.326428413391113
node_619: feature_name=GO:0003964	feature_id[317].value <= threshold=5.575642108917236
node_620: feature_name=GO:0046498	feature_id[203].value <= threshold=6.620223522186279
node_621: feature_name=GO:0002562	feature_id[25].value <= threshold=10.287704467773438
node_622: feature_name=GO:0006919	feature_id[246].value <= threshold=5.801334381103516
node_623: feature_name=GO:0001889	feature_id[387].value <= threshold=2.679414987564087
node_624: feature_name=GO:0006555	feature_id[514].value <= threshold=7.819535493850708
node_625: feature_name=GO:0080134	feature_id[650].value <= threshold=38.91967582702637
node_626: feature_name=GO:0042127	feature_id[480].value <= threshold=37.72536659240723

node_627: feature_name=GO:2001251 feature_id[220].value <= threshold=32.564422607421875
node_628: feature_name=GO:0002829 feature_id[493].value <= threshold=2.444612741470337
node_629: feature_name=GO:0008588 feature_id[26].value <= threshold=2.3601274490356445
node_630: feature_name=GO:0033151 feature_id[7].value <= threshold=5.134376287460327
node_631: feature_name=GO:0048145 feature_id[348].value <= threshold=8.836549282073975
node_632: feature_name=GO:0030887 feature_id[274].value <= threshold=1.7000296115875244
node_633: feature_name=GO:0097190 feature_id[117].value <= threshold=33.02078819274902
node_634: feature_name=GO:0046685 feature_id[782].value <= threshold=8.040813446044922
node_635: feature_name=GO:0006346 feature_id[236].value <= threshold=6.384642839431763
node_636: feature_name=GO:0048537 feature_id[66].value <= threshold=3.1739262342453003
node_637: feature_name=GO:0070245 feature_id[326].value <= threshold=3.0491198301315308
node_638: feature_name=GO:0038065 feature_id[670].value <= threshold=2.0590850114822388
node_639: feature_name=GO:0005488 feature_id[187].value <= threshold=84.80076217651367
node_640: feature_name=GO:0060249 feature_id[390].value > threshold=0.07226398587226868
node_652: feature_name=GO:0006974 feature_id[516].value > threshold=5.17782768838515e-06
node_654: feature_name=hsa05210 feature_id[358].value <= threshold=18.730005264282227
node_655: feature_name=GO:0071850 feature_id[40].value <= threshold=5.475317001342773
node_656: feature_name=GO:1904029 feature_id[208].value <= threshold=26.575013160705566
node_657: feature_name=GO:0090594 feature_id[738].value > threshold=3.000791072845459
node_743: feature_name=GO:0051052 feature_id[310].value > threshold=3.3811163902282715
Class: negative genes

Rules_617 passed counts:1
node_0: feature_name=GO:0042113 feature_id[0].value <= threshold=13.408552169799805
node_1: feature_name=GO:0007568 feature_id[534].value <= threshold=5.0313897132873535
node_2: feature_name=GO:0002705 feature_id[541].value <= threshold=3.200145721435547
node_3: feature_name=GO:1901525 feature_id[576].value > threshold=0.4466460347175598
node_617: feature_name=GO:0005622 feature_id[233].value <= threshold=90.85730743408203
node_618: feature_name=GO:1903147 feature_id[200].value <= threshold=7.326428413391113
node_619: feature_name=GO:0003964 feature_id[317].value <= threshold=5.575642108917236
node_620: feature_name=GO:0046498 feature_id[203].value <= threshold=6.620223522186279
node_621: feature_name=GO:0002562 feature_id[25].value <= threshold=10.287704467773438
node_622: feature_name=GO:0006919 feature_id[246].value <= threshold=5.801334381103516
node_623: feature_name=GO:0001889 feature_id[387].value <= threshold=2.679414987564087
node_624: feature_name=GO:0006555 feature_id[514].value <= threshold=7.819535493850708

node_625: feature_name=GO:0080134 feature_id[650].value <= threshold=38.91967582702637
node_626: feature_name=GO:0042127 feature_id[480].value <= threshold=37.72536659240723
node_627: feature_name=GO:2001251 feature_id[220].value <= threshold=32.564422607421875
node_628: feature_name=GO:0002829 feature_id[493].value <= threshold=2.444612741470337
node_629: feature_name=GO:0008588 feature_id[26].value <= threshold=2.3601274490356445
node_630: feature_name=GO:0033151 feature_id[7].value <= threshold=5.134376287460327
node_631: feature_name=GO:0048145 feature_id[348].value <= threshold=8.836549282073975
node_632: feature_name=GO:0030887 feature_id[274].value <= threshold=1.7000296115875244
node_633: feature_name=GO:0097190 feature_id[117].value <= threshold=33.02078819274902
node_634: feature_name=GO:0046685 feature_id[782].value <= threshold=8.040813446044922
node_635: feature_name=GO:0006346 feature_id[236].value <= threshold=6.384642839431763
node_636: feature_name=GO:0048537 feature_id[66].value <= threshold=3.1739262342453003
node_637: feature_name=GO:0070245 feature_id[326].value <= threshold=3.0491198301315308
node_638: feature_name=GO:0038065 feature_id[670].value <= threshold=2.0590850114822388
node_639: feature_name=GO:0005488 feature_id[187].value <= threshold=84.80076217651367
node_640: feature_name=GO:0060249 feature_id[390].value > threshold=0.07226398587226868
node_652: feature_name=GO:0006974 feature_id[516].value > threshold=5.17782768838515e-06
node_654: feature_name=hsa05210 feature_id[358].value <= threshold=18.730005264282227
node_655: feature_name=GO:0071850 feature_id[40].value <= threshold=5.475317001342773
node_656: feature_name=GO:1904029 feature_id[208].value <= threshold=26.575013160705566
node_657: feature_name=GO:0090594 feature_id[738].value > threshold=3.000791072845459
node_743: feature_name=GO:0051052 feature_id[310].value <= threshold=3.3811163902282715
Class: positive genes

Rules_618 passed counts:1
node_0: feature_name=GO:0042113 feature_id[0].value <= threshold=13.408552169799805
node_1: feature_name=GO:0007568 feature_id[534].value <= threshold=5.0313897132873535
node_2: feature_name=GO:0002705 feature_id[541].value <= threshold=3.200145721435547
node_3: feature_name=GO:1901525 feature_id[576].value > threshold=0.4466460347175598
node_617: feature_name=GO:0005622 feature_id[233].value <= threshold=90.85730743408203
node_618: feature_name=GO:1903147 feature_id[200].value <= threshold=7.326428413391113
node_619: feature_name=GO:0003964 feature_id[317].value <= threshold=5.575642108917236
node_620: feature_name=GO:0046498 feature_id[203].value <= threshold=6.620223522186279
node_621: feature_name=GO:0002562 feature_id[25].value <= threshold=10.287704467773438
node_622: feature_name=GO:0006919 feature_id[246].value <= threshold=5.801334381103516

node_623: feature_name=GO:0001889 feature_id[387].value <= threshold=2.679414987564087
node_624: feature_name=GO:0006555 feature_id[514].value <= threshold=7.819535493850708
node_625: feature_name=GO:0080134 feature_id[650].value <= threshold=38.91967582702637
node_626: feature_name=GO:0042127 feature_id[480].value <= threshold=37.72536659240723
node_627: feature_name=GO:2001251 feature_id[220].value <= threshold=32.564422607421875
node_628: feature_name=GO:0002829 feature_id[493].value <= threshold=2.444612741470337
node_629: feature_name=GO:0008588 feature_id[26].value <= threshold=2.3601274490356445
node_630: feature_name=GO:0033151 feature_id[7].value <= threshold=5.134376287460327
node_631: feature_name=GO:0048145 feature_id[348].value <= threshold=8.836549282073975
node_632: feature_name=GO:0030887 feature_id[274].value <= threshold=1.7000296115875244
node_633: feature_name=GO:0097190 feature_id[117].value <= threshold=33.02078819274902
node_634: feature_name=GO:0046685 feature_id[782].value <= threshold=8.040813446044922
node_635: feature_name=GO:0006346 feature_id[236].value <= threshold=6.384642839431763
node_636: feature_name=GO:0048537 feature_id[66].value <= threshold=3.1739262342453003
node_637: feature_name=GO:0070245 feature_id[326].value <= threshold=3.0491198301315308
node_638: feature_name=GO:0038065 feature_id[670].value <= threshold=2.0590850114822388
node_639: feature_name=GO:0005488 feature_id[187].value <= threshold=84.80076217651367
node_640: feature_name=GO:0060249 feature_id[390].value > threshold=0.07226398587226868
node_652: feature_name=GO:0006974 feature_id[516].value > threshold=5.17782768838515e-06
node_654: feature_name=hsa05210 feature_id[358].value <= threshold=18.730005264282227
node_655: feature_name=GO:0071850 feature_id[40].value <= threshold=5.475317001342773
node_656: feature_name=GO:1904029 feature_id[208].value <= threshold=26.575013160705566
node_657: feature_name=GO:0090594 feature_id[738].value <= threshold=3.000791072845459
node_658: feature_name=GO:0010948 feature_id[569].value > threshold=0.04386143572628498
node_666: feature_name=GO:0032764 feature_id[3].value > threshold=2.5201677083969116
node_740: feature_name=GO:0046500 feature_id[171].value <= threshold=0.266752228140831
Class: positive genes

Rules_619 passed counts:1
node_0: feature_name=GO:0042113 feature_id[0].value <= threshold=13.408552169799805
node_1: feature_name=GO:0007568 feature_id[534].value <= threshold=5.0313897132873535
node_2: feature_name=GO:0002705 feature_id[541].value <= threshold=3.200145721435547
node_3: feature_name=GO:1901525 feature_id[576].value > threshold=0.4466460347175598
node_617: feature_name=GO:0005622 feature_id[233].value <= threshold=90.85730743408203
node_618: feature_name=GO:1903147 feature_id[200].value <= threshold=7.326428413391113

node_619: feature_name=GO:0003964 feature_id[317].value <= threshold=5.575642108917236
node_620: feature_name=GO:0046498 feature_id[203].value <= threshold=6.620223522186279
node_621: feature_name=GO:0002562 feature_id[25].value <= threshold=10.287704467773438
node_622: feature_name=GO:0006919 feature_id[246].value <= threshold=5.801334381103516
node_623: feature_name=GO:0001889 feature_id[387].value <= threshold=2.679414987564087
node_624: feature_name=GO:0006555 feature_id[514].value <= threshold=7.819535493850708
node_625: feature_name=GO:0080134 feature_id[650].value <= threshold=38.91967582702637
node_626: feature_name=GO:0042127 feature_id[480].value <= threshold=37.72536659240723
node_627: feature_name=GO:2001251 feature_id[220].value <= threshold=32.564422607421875
node_628: feature_name=GO:0002829 feature_id[493].value <= threshold=2.444612741470337
node_629: feature_name=GO:0008588 feature_id[26].value <= threshold=2.3601274490356445
node_630: feature_name=GO:0033151 feature_id[7].value <= threshold=5.134376287460327
node_631: feature_name=GO:0048145 feature_id[348].value <= threshold=8.836549282073975
node_632: feature_name=GO:0030887 feature_id[274].value <= threshold=1.7000296115875244
node_633: feature_name=GO:0097190 feature_id[117].value <= threshold=33.02078819274902
node_634: feature_name=GO:0046685 feature_id[782].value <= threshold=8.040813446044922
node_635: feature_name=GO:0006346 feature_id[236].value <= threshold=6.384642839431763
node_636: feature_name=GO:0048537 feature_id[66].value <= threshold=3.1739262342453003
node_637: feature_name=GO:0070245 feature_id[326].value <= threshold=3.0491198301315308
node_638: feature_name=GO:0038065 feature_id[670].value <= threshold=2.0590850114822388
node_639: feature_name=GO:0005488 feature_id[187].value <= threshold=84.80076217651367
node_640: feature_name=GO:0060249 feature_id[390].value > threshold=0.07226398587226868
node_652: feature_name=GO:0006974 feature_id[516].value > threshold=5.17782768838515e-06
node_654: feature_name=hsa05210 feature_id[358].value <= threshold=18.730005264282227
node_655: feature_name=GO:0071850 feature_id[40].value <= threshold=5.475317001342773
node_656: feature_name=GO:1904029 feature_id[208].value <= threshold=26.575013160705566
node_657: feature_name=GO:0090594 feature_id[738].value <= threshold=3.000791072845459
node_658: feature_name=GO:0010948 feature_id[569].value > threshold=0.04386143572628498
node_666: feature_name=GO:0032764 feature_id[3].value <= threshold=2.5201677083969116
node_667: feature_name=GO:0023026 feature_id[69].value <= threshold=3.2760006189346313
node_668: feature_name=GO:0032464 feature_id[83].value > threshold=2.5927021503448486
node_734: feature_name=GO:0032069 feature_id[614].value <= threshold=0.8012068271636963
Class: positive genes

Rules_620 passed counts:1

node_0: feature_name=GO:0042113	feature_id[0].value <= threshold=13.408552169799805
node_1: feature_name=GO:0007568	feature_id[534].value <= threshold=5.0313897132873535
node_2: feature_name=GO:0002705	feature_id[541].value <= threshold=3.200145721435547
node_3: feature_name=GO:1901525	feature_id[576].value > threshold=0.4466460347175598
node_617: feature_name=GO:0005622	feature_id[233].value <= threshold=90.85730743408203
node_618: feature_name=GO:1903147	feature_id[200].value <= threshold=7.326428413391113
node_619: feature_name=GO:0003964	feature_id[317].value <= threshold=5.575642108917236
node_620: feature_name=GO:0046498	feature_id[203].value <= threshold=6.620223522186279
node_621: feature_name=GO:0002562	feature_id[25].value <= threshold=10.287704467773438
node_622: feature_name=GO:0006919	feature_id[246].value <= threshold=5.801334381103516
node_623: feature_name=GO:0001889	feature_id[387].value <= threshold=2.679414987564087
node_624: feature_name=GO:0006555	feature_id[514].value <= threshold=7.819535493850708
node_625: feature_name=GO:0080134	feature_id[650].value <= threshold=38.91967582702637
node_626: feature_name=GO:0042127	feature_id[480].value <= threshold=37.72536659240723
node_627: feature_name=GO:2001251	feature_id[220].value <= threshold=32.564422607421875
node_628: feature_name=GO:0002829	feature_id[493].value <= threshold=2.444612741470337
node_629: feature_name=GO:0008588	feature_id[26].value <= threshold=2.3601274490356445
node_630: feature_name=GO:0033151	feature_id[7].value <= threshold=5.134376287460327
node_631: feature_name=GO:0048145	feature_id[348].value <= threshold=8.836549282073975
node_632: feature_name=GO:0030887	feature_id[274].value <= threshold=1.7000296115875244
node_633: feature_name=GO:0097190	feature_id[117].value <= threshold=33.02078819274902
node_634: feature_name=GO:0046685	feature_id[782].value <= threshold=8.040813446044922
node_635: feature_name=GO:0006346	feature_id[236].value <= threshold=6.384642839431763
node_636: feature_name=GO:0048537	feature_id[66].value <= threshold=3.1739262342453003
node_637: feature_name=GO:0070245	feature_id[326].value <= threshold=3.0491198301315308
node_638: feature_name=GO:0038065	feature_id[670].value <= threshold=2.0590850114822388
node_639: feature_name=GO:0005488	feature_id[187].value <= threshold=84.80076217651367
node_640: feature_name=GO:0060249	feature_id[390].value > threshold=0.07226398587226868
node_652: feature_name=GO:0006974	feature_id[516].value > threshold=5.17782768838515e-06
node_654: feature_name=hsa05210	feature_id[358].value <= threshold=18.730005264282227
node_655: feature_name=GO:0071850	feature_id[40].value <= threshold=5.475317001342773
node_656: feature_name=GO:1904029	feature_id[208].value <= threshold=26.575013160705566
node_657: feature_name=GO:0090594	feature_id[738].value <= threshold=3.000791072845459
node_658: feature_name=GO:0010948	feature_id[569].value > threshold=0.04386143572628498
node_666: feature_name=GO:0032764	feature_id[3].value <= threshold=2.5201677083969116

node_667: feature_name=GO:0023026 feature_id[69].value <= threshold=3.2760006189346313
node_668: feature_name=GO:0032464 feature_id[83].value <= threshold=2.5927021503448486
node_669: feature_name=GO:0071901 feature_id[407].value > threshold=18.238012313842773
node_731: feature_name=GO:0002821 feature_id[588].value <= threshold=0.05757370963692665
Class: positive genes

Rules_621 passed counts:1
node_0: feature_name=GO:0042113 feature_id[0].value <= threshold=13.408552169799805
node_1: feature_name=GO:0007568 feature_id[534].value <= threshold=5.0313897132873535
node_2: feature_name=GO:0002705 feature_id[541].value <= threshold=3.200145721435547
node_3: feature_name=GO:1901525 feature_id[576].value > threshold=0.4466460347175598
node_617: feature_name=GO:0005622 feature_id[233].value <= threshold=90.85730743408203
node_618: feature_name=GO:1903147 feature_id[200].value <= threshold=7.326428413391113
node_619: feature_name=GO:0003964 feature_id[317].value <= threshold=5.575642108917236
node_620: feature_name=GO:0046498 feature_id[203].value <= threshold=6.620223522186279
node_621: feature_name=GO:0002562 feature_id[25].value <= threshold=10.287704467773438
node_622: feature_name=GO:0006919 feature_id[246].value <= threshold=5.801334381103516
node_623: feature_name=GO:0001889 feature_id[387].value <= threshold=2.679414987564087
node_624: feature_name=GO:0006555 feature_id[514].value <= threshold=7.819535493850708
node_625: feature_name=GO:0080134 feature_id[650].value <= threshold=38.91967582702637
node_626: feature_name=GO:0042127 feature_id[480].value <= threshold=37.72536659240723
node_627: feature_name=GO:2001251 feature_id[220].value <= threshold=32.564422607421875
node_628: feature_name=GO:0002829 feature_id[493].value <= threshold=2.444612741470337
node_629: feature_name=GO:0008588 feature_id[26].value <= threshold=2.3601274490356445
node_630: feature_name=GO:0033151 feature_id[7].value <= threshold=5.134376287460327
node_631: feature_name=GO:0048145 feature_id[348].value <= threshold=8.836549282073975
node_632: feature_name=GO:0030887 feature_id[274].value <= threshold=1.7000296115875244
node_633: feature_name=GO:0097190 feature_id[117].value <= threshold=33.02078819274902
node_634: feature_name=GO:0046685 feature_id[782].value <= threshold=8.040813446044922
node_635: feature_name=GO:0006346 feature_id[236].value <= threshold=6.384642839431763
node_636: feature_name=GO:0048537 feature_id[66].value <= threshold=3.1739262342453003
node_637: feature_name=GO:0070245 feature_id[326].value <= threshold=3.0491198301315308
node_638: feature_name=GO:0038065 feature_id[670].value <= threshold=2.0590850114822388
node_639: feature_name=GO:0005488 feature_id[187].value <= threshold=84.80076217651367
node_640: feature_name=GO:0060249 feature_id[390].value > threshold=0.07226398587226868

node_652: feature_name=GO:0006974 feature_id[516].value > threshold=5.17782768838515e-06
node_654: feature_name=hsa05210 feature_id[358].value <= threshold=18.730005264282227
node_655: feature_name=GO:0071850 feature_id[40].value <= threshold=5.475317001342773
node_656: feature_name=GO:1904029 feature_id[208].value <= threshold=26.575013160705566
node_657: feature_name=GO:0090594 feature_id[738].value <= threshold=3.000791072845459
node_658: feature_name=GO:0010948 feature_id[569].value > threshold=0.04386143572628498
node_666: feature_name=GO:0032764 feature_id[3].value <= threshold=2.5201677083969116
node_667: feature_name=GO:0023026 feature_id[69].value <= threshold=3.2760006189346313
node_668: feature_name=GO:0032464 feature_id[83].value <= threshold=2.5927021503448486
node_669: feature_name=GO:0071901 feature_id[407].value <= threshold=18.238012313842773
node_670: feature_name=GO:0015671 feature_id[103].value > threshold=1.3113192915916443
node_722: feature_name=GO:0035067 feature_id[649].value <= threshold=2.5819170475006104
node_723: feature_name=GO:0046677 feature_id[591].value > threshold=2.915908694267273
node_727: feature_name=hsa05223 feature_id[540].value > threshold=3.5530080795288086
Class: negative genes

Rules_622 passed counts:1
node_0: feature_name=GO:0042113 feature_id[0].value <= threshold=13.408552169799805
node_1: feature_name=GO:0007568 feature_id[534].value <= threshold=5.0313897132873535
node_2: feature_name=GO:0002705 feature_id[541].value <= threshold=3.200145721435547
node_3: feature_name=GO:1901525 feature_id[576].value > threshold=0.4466460347175598
node_617: feature_name=GO:0005622 feature_id[233].value <= threshold=90.85730743408203
node_618: feature_name=GO:1903147 feature_id[200].value <= threshold=7.326428413391113
node_619: feature_name=GO:0003964 feature_id[317].value <= threshold=5.575642108917236
node_620: feature_name=GO:0046498 feature_id[203].value <= threshold=6.620223522186279
node_621: feature_name=GO:0002562 feature_id[25].value <= threshold=10.287704467773438
node_622: feature_name=GO:0006919 feature_id[246].value <= threshold=5.801334381103516
node_623: feature_name=GO:0001889 feature_id[387].value <= threshold=2.679414987564087
node_624: feature_name=GO:0006555 feature_id[514].value <= threshold=7.819535493850708
node_625: feature_name=GO:0080134 feature_id[650].value <= threshold=38.91967582702637
node_626: feature_name=GO:0042127 feature_id[480].value <= threshold=37.72536659240723
node_627: feature_name=GO:2001251 feature_id[220].value <= threshold=32.564422607421875
node_628: feature_name=GO:0002829 feature_id[493].value <= threshold=2.444612741470337
node_629: feature_name=GO:0008588 feature_id[26].value <= threshold=2.3601274490356445
node_630: feature_name=GO:0033151 feature_id[7].value <= threshold=5.134376287460327

node_631: feature_name=GO:0048145 feature_id[348].value <= threshold=8.836549282073975
node_632: feature_name=GO:0030887 feature_id[274].value <= threshold=1.7000296115875244
node_633: feature_name=GO:0097190 feature_id[117].value <= threshold=33.02078819274902
node_634: feature_name=GO:0046685 feature_id[782].value <= threshold=8.040813446044922
node_635: feature_name=GO:0006346 feature_id[236].value <= threshold=6.384642839431763
node_636: feature_name=GO:0048537 feature_id[66].value <= threshold=3.1739262342453003
node_637: feature_name=GO:0070245 feature_id[326].value <= threshold=3.0491198301315308
node_638: feature_name=GO:0038065 feature_id[670].value <= threshold=2.0590850114822388
node_639: feature_name=GO:0005488 feature_id[187].value <= threshold=84.80076217651367
node_640: feature_name=GO:0060249 feature_id[390].value > threshold=0.07226398587226868
node_652: feature_name=GO:0006974 feature_id[516].value > threshold=5.17782768838515e-06
node_654: feature_name=hsa05210 feature_id[358].value <= threshold=18.730005264282227
node_655: feature_name=GO:0071850 feature_id[40].value <= threshold=5.475317001342773
node_656: feature_name=GO:1904029 feature_id[208].value <= threshold=26.575013160705566
node_657: feature_name=GO:0090594 feature_id[738].value <= threshold=3.000791072845459
node_658: feature_name=GO:0010948 feature_id[569].value > threshold=0.04386143572628498
node_666: feature_name=GO:0032764 feature_id[3].value <= threshold=2.5201677083969116
node_667: feature_name=GO:0023026 feature_id[69].value <= threshold=3.2760006189346313
node_668: feature_name=GO:0032464 feature_id[83].value <= threshold=2.5927021503448486
node_669: feature_name=GO:0071901 feature_id[407].value <= threshold=18.238012313842773
node_670: feature_name=GO:0015671 feature_id[103].value > threshold=1.3113192915916443
node_722: feature_name=GO:0035067 feature_id[649].value <= threshold=2.5819170475006104
node_723: feature_name=GO:0046677 feature_id[591].value <= threshold=2.915908694267273
node_724: feature_name=GO:0045579 feature_id[41].value > threshold=1.265580177307129
Class: positive genes

Rules_623 passed counts:1
node_0: feature_name=GO:0042113 feature_id[0].value <= threshold=13.408552169799805
node_1: feature_name=GO:0007568 feature_id[534].value <= threshold=5.0313897132873535
node_2: feature_name=GO:0002705 feature_id[541].value <= threshold=3.200145721435547
node_3: feature_name=GO:1901525 feature_id[576].value > threshold=0.4466460347175598
node_617: feature_name=GO:0005622 feature_id[233].value <= threshold=90.85730743408203
node_618: feature_name=GO:1903147 feature_id[200].value <= threshold=7.326428413391113
node_619: feature_name=GO:0003964 feature_id[317].value <= threshold=5.575642108917236
node_620: feature_name=GO:0046498 feature_id[203].value <= threshold=6.620223522186279

node_621: feature_name=GO:0002562 feature_id[25].value <= threshold=10.287704467773438
node_622: feature_name=GO:0006919 feature_id[246].value <= threshold=5.801334381103516
node_623: feature_name=GO:0001889 feature_id[387].value <= threshold=2.679414987564087
node_624: feature_name=GO:0006555 feature_id[514].value <= threshold=7.819535493850708
node_625: feature_name=GO:0080134 feature_id[650].value <= threshold=38.91967582702637
node_626: feature_name=GO:0042127 feature_id[480].value <= threshold=37.72536659240723
node_627: feature_name=GO:2001251 feature_id[220].value <= threshold=32.564422607421875
node_628: feature_name=GO:0002829 feature_id[493].value <= threshold=2.444612741470337
node_629: feature_name=GO:0008588 feature_id[26].value <= threshold=2.3601274490356445
node_630: feature_name=GO:0033151 feature_id[7].value <= threshold=5.134376287460327
node_631: feature_name=GO:0048145 feature_id[348].value <= threshold=8.836549282073975
node_632: feature_name=GO:0030887 feature_id[274].value <= threshold=1.7000296115875244
node_633: feature_name=GO:0097190 feature_id[117].value <= threshold=33.02078819274902
node_634: feature_name=GO:0046685 feature_id[782].value <= threshold=8.040813446044922
node_635: feature_name=GO:0006346 feature_id[236].value <= threshold=6.384642839431763
node_636: feature_name=GO:0048537 feature_id[66].value <= threshold=3.1739262342453003
node_637: feature_name=GO:0070245 feature_id[326].value <= threshold=3.0491198301315308
node_638: feature_name=GO:0038065 feature_id[670].value <= threshold=2.0590850114822388
node_639: feature_name=GO:0005488 feature_id[187].value <= threshold=84.80076217651367
node_640: feature_name=GO:0060249 feature_id[390].value > threshold=0.07226398587226868
node_652: feature_name=GO:0006974 feature_id[516].value > threshold=5.17782768838515e-06
node_654: feature_name=hsa05210 feature_id[358].value <= threshold=18.730005264282227
node_655: feature_name=GO:0071850 feature_id[40].value <= threshold=5.475317001342773
node_656: feature_name=GO:1904029 feature_id[208].value <= threshold=26.575013160705566
node_657: feature_name=GO:0090594 feature_id[738].value <= threshold=3.000791072845459
node_658: feature_name=GO:0010948 feature_id[569].value > threshold=0.04386143572628498
node_666: feature_name=GO:0032764 feature_id[3].value <= threshold=2.5201677083969116
node_667: feature_name=GO:0023026 feature_id[69].value <= threshold=3.2760006189346313
node_668: feature_name=GO:0032464 feature_id[83].value <= threshold=2.5927021503448486
node_669: feature_name=GO:0071901 feature_id[407].value <= threshold=18.238012313842773
node_670: feature_name=GO:0015671 feature_id[103].value <= threshold=1.3113192915916443
node_671: feature_name=GO:0008340 feature_id[539].value > threshold=4.860779762268066
node_719: feature_name=GO:1903037 feature_id[165].value > threshold=0.4720069617033005
Class: positive genes

Rules_624	passed counts:1
node_0: feature_name=GO:0042113	feature_id[0].value <= threshold=13.408552169799805
node_1: feature_name=GO:0007568	feature_id[534].value <= threshold=5.0313897132873535
node_2: feature_name=GO:0002705	feature_id[541].value <= threshold=3.200145721435547
node_3: feature_name=GO:1901525	feature_id[576].value > threshold=0.4466460347175598
node_617: feature_name=GO:0005622	feature_id[233].value <= threshold=90.85730743408203
node_618: feature_name=GO:1903147	feature_id[200].value <= threshold=7.326428413391113
node_619: feature_name=GO:0003964	feature_id[317].value <= threshold=5.575642108917236
node_620: feature_name=GO:0046498	feature_id[203].value <= threshold=6.620223522186279
node_621: feature_name=GO:0002562	feature_id[25].value <= threshold=10.287704467773438
node_622: feature_name=GO:0006919	feature_id[246].value <= threshold=5.801334381103516
node_623: feature_name=GO:0001889	feature_id[387].value <= threshold=2.679414987564087
node_624: feature_name=GO:0006555	feature_id[514].value <= threshold=7.819535493850708
node_625: feature_name=GO:0080134	feature_id[650].value <= threshold=38.91967582702637
node_626: feature_name=GO:0042127	feature_id[480].value <= threshold=37.72536659240723
node_627: feature_name=GO:2001251	feature_id[220].value <= threshold=32.564422607421875
node_628: feature_name=GO:0002829	feature_id[493].value <= threshold=2.444612741470337
node_629: feature_name=GO:0008588	feature_id[26].value <= threshold=2.3601274490356445
node_630: feature_name=GO:0033151	feature_id[7].value <= threshold=5.134376287460327
node_631: feature_name=GO:0048145	feature_id[348].value <= threshold=8.836549282073975
node_632: feature_name=GO:0030887	feature_id[274].value <= threshold=1.7000296115875244
node_633: feature_name=GO:0097190	feature_id[117].value <= threshold=33.02078819274902
node_634: feature_name=GO:0046685	feature_id[782].value <= threshold=8.040813446044922
node_635: feature_name=GO:0006346	feature_id[236].value <= threshold=6.384642839431763
node_636: feature_name=GO:0048537	feature_id[66].value <= threshold=3.1739262342453003
node_637: feature_name=GO:0070245	feature_id[326].value <= threshold=3.0491198301315308
node_638: feature_name=GO:0038065	feature_id[670].value <= threshold=2.0590850114822388
node_639: feature_name=GO:0005488	feature_id[187].value <= threshold=84.80076217651367
node_640: feature_name=GO:0060249	feature_id[390].value > threshold=0.07226398587226868
node_652: feature_name=GO:0006974	feature_id[516].value > threshold=5.17782768838515e-06
node_654: feature_name=hsa05210	feature_id[358].value <= threshold=18.730005264282227
node_655: feature_name=GO:0071850	feature_id[40].value <= threshold=5.475317001342773
node_656: feature_name=GO:1904029	feature_id[208].value <= threshold=26.575013160705566
node_657: feature_name=GO:0090594	feature_id[738].value <= threshold=3.000791072845459
node_658: feature_name=GO:0010948	feature_id[569].value > threshold=0.04386143572628498

node_666: feature_name=GO:0032764 feature_id[3].value <= threshold=2.5201677083969116
node_667: feature_name=GO:0023026 feature_id[69].value <= threshold=3.2760006189346313
node_668: feature_name=GO:0032464 feature_id[83].value <= threshold=2.5927021503448486
node_669: feature_name=GO:0071901 feature_id[407].value <= threshold=18.238012313842773
node_670: feature_name=GO:0015671 feature_id[103].value <= threshold=1.3113192915916443
node_671: feature_name=GO:0008340 feature_id[539].value <= threshold=4.860779762268066
node_672: feature_name=GO:0046006 feature_id[364].value > threshold=2.4317692518234253
node_716: feature_name=GO:0030155 feature_id[594].value <= threshold=0.13681869907304645
Class: positive genes

Rules_625 passed counts:1
node_0: feature_name=GO:0042113 feature_id[0].value <= threshold=13.408552169799805
node_1: feature_name=GO:0007568 feature_id[534].value <= threshold=5.0313897132873535
node_2: feature_name=GO:0002705 feature_id[541].value <= threshold=3.200145721435547
node_3: feature_name=GO:1901525 feature_id[576].value > threshold=0.4466460347175598
node_617: feature_name=GO:0005622 feature_id[233].value <= threshold=90.85730743408203
node_618: feature_name=GO:1903147 feature_id[200].value <= threshold=7.326428413391113
node_619: feature_name=GO:0003964 feature_id[317].value <= threshold=5.575642108917236
node_620: feature_name=GO:0046498 feature_id[203].value <= threshold=6.620223522186279
node_621: feature_name=GO:0002562 feature_id[25].value <= threshold=10.287704467773438
node_622: feature_name=GO:0006919 feature_id[246].value <= threshold=5.801334381103516
node_623: feature_name=GO:0001889 feature_id[387].value <= threshold=2.679414987564087
node_624: feature_name=GO:0006555 feature_id[514].value <= threshold=7.819535493850708
node_625: feature_name=GO:0080134 feature_id[650].value <= threshold=38.91967582702637
node_626: feature_name=GO:0042127 feature_id[480].value <= threshold=37.72536659240723
node_627: feature_name=GO:2001251 feature_id[220].value <= threshold=32.564422607421875
node_628: feature_name=GO:0002829 feature_id[493].value <= threshold=2.444612741470337
node_629: feature_name=GO:0008588 feature_id[26].value <= threshold=2.3601274490356445
node_630: feature_name=GO:0033151 feature_id[7].value <= threshold=5.134376287460327
node_631: feature_name=GO:0048145 feature_id[348].value <= threshold=8.836549282073975
node_632: feature_name=GO:0030887 feature_id[274].value <= threshold=1.7000296115875244
node_633: feature_name=GO:0097190 feature_id[117].value <= threshold=33.02078819274902
node_634: feature_name=GO:0046685 feature_id[782].value <= threshold=8.040813446044922
node_635: feature_name=GO:0006346 feature_id[236].value <= threshold=6.384642839431763
node_636: feature_name=GO:0048537 feature_id[66].value <= threshold=3.1739262342453003

node_637: feature_name=GO:0070245 feature_id[326].value <= threshold=3.0491198301315308
node_638: feature_name=GO:0038065 feature_id[670].value <= threshold=2.0590850114822388
node_639: feature_name=GO:0005488 feature_id[187].value <= threshold=84.80076217651367
node_640: feature_name=GO:0060249 feature_id[390].value > threshold=0.07226398587226868
node_652: feature_name=GO:0006974 feature_id[516].value > threshold=5.17782768838515e-06
node_654: feature_name=hsa05210 feature_id[358].value <= threshold=18.730005264282227
node_655: feature_name=GO:0071850 feature_id[40].value <= threshold=5.475317001342773
node_656: feature_name=GO:1904029 feature_id[208].value <= threshold=26.575013160705566
node_657: feature_name=GO:0090594 feature_id[738].value <= threshold=3.000791072845459
node_658: feature_name=GO:0010948 feature_id[569].value > threshold=0.04386143572628498
node_666: feature_name=GO:0032764 feature_id[3].value <= threshold=2.5201677083969116
node_667: feature_name=GO:0023026 feature_id[69].value <= threshold=3.2760006189346313
node_668: feature_name=GO:0032464 feature_id[83].value <= threshold=2.5927021503448486
node_669: feature_name=GO:0071901 feature_id[407].value <= threshold=18.238012313842773
node_670: feature_name=GO:0015671 feature_id[103].value <= threshold=1.3113192915916443
node_671: feature_name=GO:0008340 feature_id[539].value <= threshold=4.860779762268066
node_672: feature_name=GO:0046006 feature_id[364].value <= threshold=2.4317692518234253
node_673: feature_name=GO:0042991 feature_id[684].value > threshold=5.553565502166748
node_713: feature_name=GO:0046427 feature_id[766].value <= threshold=0.28762511909008026
Class: positive genes

Rules_626 passed counts:1
node_0: feature_name=GO:0042113 feature_id[0].value <= threshold=13.408552169799805
node_1: feature_name=GO:0007568 feature_id[534].value <= threshold=5.0313897132873535
node_2: feature_name=GO:0002705 feature_id[541].value <= threshold=3.200145721435547
node_3: feature_name=GO:1901525 feature_id[576].value > threshold=0.4466460347175598
node_617: feature_name=GO:0005622 feature_id[233].value <= threshold=90.85730743408203
node_618: feature_name=GO:1903147 feature_id[200].value <= threshold=7.326428413391113
node_619: feature_name=GO:0003964 feature_id[317].value <= threshold=5.575642108917236
node_620: feature_name=GO:0046498 feature_id[203].value <= threshold=6.620223522186279
node_621: feature_name=GO:0002562 feature_id[25].value <= threshold=10.287704467773438
node_622: feature_name=GO:0006919 feature_id[246].value <= threshold=5.801334381103516
node_623: feature_name=GO:0001889 feature_id[387].value <= threshold=2.679414987564087
node_624: feature_name=GO:0006555 feature_id[514].value <= threshold=7.819535493850708
node_625: feature_name=GO:0080134 feature_id[650].value <= threshold=38.91967582702637

node_626: feature_name=GO:0042127 feature_id[480].value <= threshold=37.72536659240723
node_627: feature_name=GO:2001251 feature_id[220].value <= threshold=32.564422607421875
node_628: feature_name=GO:0002829 feature_id[493].value <= threshold=2.444612741470337
node_629: feature_name=GO:0008588 feature_id[26].value <= threshold=2.3601274490356445
node_630: feature_name=GO:0033151 feature_id[7].value <= threshold=5.134376287460327
node_631: feature_name=GO:0048145 feature_id[348].value <= threshold=8.836549282073975
node_632: feature_name=GO:0030887 feature_id[274].value <= threshold=1.7000296115875244
node_633: feature_name=GO:0097190 feature_id[117].value <= threshold=33.02078819274902
node_634: feature_name=GO:0046685 feature_id[782].value <= threshold=8.040813446044922
node_635: feature_name=GO:0006346 feature_id[236].value <= threshold=6.384642839431763
node_636: feature_name=GO:0048537 feature_id[66].value <= threshold=3.1739262342453003
node_637: feature_name=GO:0070245 feature_id[326].value <= threshold=3.0491198301315308
node_638: feature_name=GO:0038065 feature_id[670].value <= threshold=2.0590850114822388
node_639: feature_name=GO:0005488 feature_id[187].value <= threshold=84.80076217651367
node_640: feature_name=GO:0060249 feature_id[390].value > threshold=0.07226398587226868
node_652: feature_name=GO:0006974 feature_id[516].value > threshold=5.17782768838515e-06
node_654: feature_name=hsa05210 feature_id[358].value <= threshold=18.730005264282227
node_655: feature_name=GO:0071850 feature_id[40].value <= threshold=5.475317001342773
node_656: feature_name=GO:1904029 feature_id[208].value <= threshold=26.575013160705566
node_657: feature_name=GO:0090594 feature_id[738].value <= threshold=3.000791072845459
node_658: feature_name=GO:0010948 feature_id[569].value > threshold=0.04386143572628498
node_666: feature_name=GO:0032764 feature_id[3].value <= threshold=2.5201677083969116
node_667: feature_name=GO:0023026 feature_id[69].value <= threshold=3.2760006189346313
node_668: feature_name=GO:0032464 feature_id[83].value <= threshold=2.5927021503448486
node_669: feature_name=GO:0071901 feature_id[407].value <= threshold=18.238012313842773
node_670: feature_name=GO:0015671 feature_id[103].value <= threshold=1.3113192915916443
node_671: feature_name=GO:0008340 feature_id[539].value <= threshold=4.860779762268066
node_672: feature_name=GO:0046006 feature_id[364].value <= threshold=2.4317692518234253
node_673: feature_name=GO:0042991 feature_id[684].value <= threshold=5.553565502166748
node_674: feature_name=GO:0002327 feature_id[408].value <= threshold=2.404940366744995
node_675: feature_name=GO:0001836 feature_id[450].value > threshold=8.647934436798096
node_707: feature_name=GO:0097202 feature_id[292].value <= threshold=1.5505802929401398
Class: positive genes

Rules_627 passed counts:1

node_0: feature_name=GO:0042113	feature_id[0].value <= threshold=13.408552169799805
node_1: feature_name=GO:0007568	feature_id[534].value <= threshold=5.0313897132873535
node_2: feature_name=GO:0002705	feature_id[541].value <= threshold=3.200145721435547
node_3: feature_name=GO:1901525	feature_id[576].value > threshold=0.4466460347175598
node_617: feature_name=GO:0005622	feature_id[233].value <= threshold=90.85730743408203
node_618: feature_name=GO:1903147	feature_id[200].value <= threshold=7.326428413391113
node_619: feature_name=GO:0003964	feature_id[317].value <= threshold=5.575642108917236
node_620: feature_name=GO:0046498	feature_id[203].value <= threshold=6.620223522186279
node_621: feature_name=GO:0002562	feature_id[25].value <= threshold=10.287704467773438
node_622: feature_name=GO:0006919	feature_id[246].value <= threshold=5.801334381103516
node_623: feature_name=GO:0001889	feature_id[387].value <= threshold=2.679414987564087
node_624: feature_name=GO:0006555	feature_id[514].value <= threshold=7.819535493850708
node_625: feature_name=GO:0080134	feature_id[650].value <= threshold=38.91967582702637
node_626: feature_name=GO:0042127	feature_id[480].value <= threshold=37.72536659240723
node_627: feature_name=GO:2001251	feature_id[220].value <= threshold=32.564422607421875
node_628: feature_name=GO:0002829	feature_id[493].value <= threshold=2.444612741470337
node_629: feature_name=GO:0008588	feature_id[26].value <= threshold=2.3601274490356445
node_630: feature_name=GO:0033151	feature_id[7].value <= threshold=5.134376287460327
node_631: feature_name=GO:0048145	feature_id[348].value <= threshold=8.836549282073975
node_632: feature_name=GO:0030887	feature_id[274].value <= threshold=1.7000296115875244
node_633: feature_name=GO:0097190	feature_id[117].value <= threshold=33.02078819274902
node_634: feature_name=GO:0046685	feature_id[782].value <= threshold=8.040813446044922
node_635: feature_name=GO:0006346	feature_id[236].value <= threshold=6.384642839431763
node_636: feature_name=GO:0048537	feature_id[66].value <= threshold=3.1739262342453003
node_637: feature_name=GO:0070245	feature_id[326].value <= threshold=3.0491198301315308
node_638: feature_name=GO:0038065	feature_id[670].value <= threshold=2.0590850114822388
node_639: feature_name=GO:0005488	feature_id[187].value <= threshold=84.80076217651367
node_640: feature_name=GO:0060249	feature_id[390].value > threshold=0.07226398587226868
node_652: feature_name=GO:0006974	feature_id[516].value > threshold=5.17782768838515e-06
node_654: feature_name=hsa05210	feature_id[358].value <= threshold=18.730005264282227
node_655: feature_name=GO:0071850	feature_id[40].value <= threshold=5.475317001342773
node_656: feature_name=GO:1904029	feature_id[208].value <= threshold=26.575013160705566
node_657: feature_name=GO:0090594	feature_id[738].value <= threshold=3.000791072845459
node_658: feature_name=GO:0010948	feature_id[569].value > threshold=0.04386143572628498
node_666: feature_name=GO:0032764	feature_id[3].value <= threshold=2.5201677083969116

node_667: feature_name=GO:0023026 feature_id[69].value <= threshold=3.2760006189346313
node_668: feature_name=GO:0032464 feature_id[83].value <= threshold=2.5927021503448486
node_669: feature_name=GO:0071901 feature_id[407].value <= threshold=18.238012313842773
node_670: feature_name=GO:0015671 feature_id[103].value <= threshold=1.3113192915916443
node_671: feature_name=GO:0008340 feature_id[539].value <= threshold=4.860779762268066
node_672: feature_name=GO:0046006 feature_id[364].value <= threshold=2.4317692518234253
node_673: feature_name=GO:0042991 feature_id[684].value <= threshold=5.553565502166748
node_674: feature_name=GO:0002327 feature_id[408].value <= threshold=2.404940366744995
node_675: feature_name=GO:0001836 feature_id[450].value <= threshold=8.647934436798096
node_676: feature_name=GO:0048539 feature_id[319].value > threshold=1.6308802962303162
node_704: feature_name=GO:0044446 feature_id[184].value > threshold=19.456976175308228
Class: positive genes

Rules_628 passed counts:1
node_0: feature_name=GO:0042113 feature_id[0].value <= threshold=13.408552169799805
node_1: feature_name=GO:0007568 feature_id[534].value <= threshold=5.0313897132873535
node_2: feature_name=GO:0002705 feature_id[541].value <= threshold=3.200145721435547
node_3: feature_name=GO:1901525 feature_id[576].value > threshold=0.4466460347175598
node_617: feature_name=GO:0005622 feature_id[233].value <= threshold=90.85730743408203
node_618: feature_name=GO:1903147 feature_id[200].value <= threshold=7.326428413391113
node_619: feature_name=GO:0003964 feature_id[317].value <= threshold=5.575642108917236
node_620: feature_name=GO:0046498 feature_id[203].value <= threshold=6.620223522186279
node_621: feature_name=GO:0002562 feature_id[25].value <= threshold=10.287704467773438
node_622: feature_name=GO:0006919 feature_id[246].value <= threshold=5.801334381103516
node_623: feature_name=GO:0001889 feature_id[387].value <= threshold=2.679414987564087
node_624: feature_name=GO:0006555 feature_id[514].value <= threshold=7.819535493850708
node_625: feature_name=GO:0080134 feature_id[650].value <= threshold=38.91967582702637
node_626: feature_name=GO:0042127 feature_id[480].value <= threshold=37.72536659240723
node_627: feature_name=GO:2001251 feature_id[220].value <= threshold=32.564422607421875
node_628: feature_name=GO:0002829 feature_id[493].value <= threshold=2.444612741470337
node_629: feature_name=GO:0008588 feature_id[26].value <= threshold=2.3601274490356445
node_630: feature_name=GO:0033151 feature_id[7].value <= threshold=5.134376287460327
node_631: feature_name=GO:0048145 feature_id[348].value <= threshold=8.836549282073975
node_632: feature_name=GO:0030887 feature_id[274].value <= threshold=1.7000296115875244
node_633: feature_name=GO:0097190 feature_id[117].value <= threshold=33.02078819274902

node_634: feature_name=GO:0046685 feature_id[782].value <= threshold=8.040813446044922
node_635: feature_name=GO:0006346 feature_id[236].value <= threshold=6.384642839431763
node_636: feature_name=GO:0048537 feature_id[66].value <= threshold=3.1739262342453003
node_637: feature_name=GO:0070245 feature_id[326].value <= threshold=3.0491198301315308
node_638: feature_name=GO:0038065 feature_id[670].value <= threshold=2.0590850114822388
node_639: feature_name=GO:0005488 feature_id[187].value <= threshold=84.80076217651367
node_640: feature_name=GO:0060249 feature_id[390].value > threshold=0.07226398587226868
node_652: feature_name=GO:0006974 feature_id[516].value > threshold=5.17782768838515e-06
node_654: feature_name=hsa05210 feature_id[358].value <= threshold=18.730005264282227
node_655: feature_name=GO:0071850 feature_id[40].value <= threshold=5.475317001342773
node_656: feature_name=GO:1904029 feature_id[208].value <= threshold=26.575013160705566
node_657: feature_name=GO:0090594 feature_id[738].value <= threshold=3.000791072845459
node_658: feature_name=GO:0010948 feature_id[569].value > threshold=0.04386143572628498
node_666: feature_name=GO:0032764 feature_id[3].value <= threshold=2.5201677083969116
node_667: feature_name=GO:0023026 feature_id[69].value <= threshold=3.2760006189346313
node_668: feature_name=GO:0032464 feature_id[83].value <= threshold=2.5927021503448486
node_669: feature_name=GO:0071901 feature_id[407].value <= threshold=18.238012313842773
node_670: feature_name=GO:0015671 feature_id[103].value <= threshold=1.3113192915916443
node_671: feature_name=GO:0008340 feature_id[539].value <= threshold=4.860779762268066
node_672: feature_name=GO:0046006 feature_id[364].value <= threshold=2.4317692518234253
node_673: feature_name=GO:0042991 feature_id[684].value <= threshold=5.553565502166748
node_674: feature_name=GO:0002327 feature_id[408].value <= threshold=2.404940366744995
node_675: feature_name=GO:0001836 feature_id[450].value <= threshold=8.647934436798096
node_676: feature_name=GO:0048539 feature_id[319].value <= threshold=1.6308802962303162
node_677: feature_name=GO:0022408 feature_id[279].value > threshold=4.064727783203125
node_701: feature_name=GO:0033077 feature_id[134].value > threshold=2.082643210887909
Class: positive genes

Rules_629 passed counts:1
node_0: feature_name=GO:0042113 feature_id[0].value <= threshold=13.408552169799805
node_1: feature_name=GO:0007568 feature_id[534].value <= threshold=5.0313897132873535
node_2: feature_name=GO:0002705 feature_id[541].value <= threshold=3.200145721435547
node_3: feature_name=GO:1901525 feature_id[576].value > threshold=0.4466460347175598
node_617: feature_name=GO:0005622 feature_id[233].value <= threshold=90.85730743408203
node_618: feature_name=GO:1903147 feature_id[200].value <= threshold=7.326428413391113

node_619: feature_name=GO:0003964 feature_id[317].value <= threshold=5.575642108917236
node_620: feature_name=GO:0046498 feature_id[203].value <= threshold=6.620223522186279
node_621: feature_name=GO:0002562 feature_id[25].value <= threshold=10.287704467773438
node_622: feature_name=GO:0006919 feature_id[246].value <= threshold=5.801334381103516
node_623: feature_name=GO:0001889 feature_id[387].value <= threshold=2.679414987564087
node_624: feature_name=GO:0006555 feature_id[514].value <= threshold=7.819535493850708
node_625: feature_name=GO:0080134 feature_id[650].value <= threshold=38.91967582702637
node_626: feature_name=GO:0042127 feature_id[480].value <= threshold=37.72536659240723
node_627: feature_name=GO:2001251 feature_id[220].value <= threshold=32.564422607421875
node_628: feature_name=GO:0002829 feature_id[493].value <= threshold=2.444612741470337
node_629: feature_name=GO:0008588 feature_id[26].value <= threshold=2.3601274490356445
node_630: feature_name=GO:0033151 feature_id[7].value <= threshold=5.134376287460327
node_631: feature_name=GO:0048145 feature_id[348].value <= threshold=8.836549282073975
node_632: feature_name=GO:0030887 feature_id[274].value <= threshold=1.7000296115875244
node_633: feature_name=GO:0097190 feature_id[117].value <= threshold=33.02078819274902
node_634: feature_name=GO:0046685 feature_id[782].value <= threshold=8.040813446044922
node_635: feature_name=GO:0006346 feature_id[236].value <= threshold=6.384642839431763
node_636: feature_name=GO:0048537 feature_id[66].value <= threshold=3.1739262342453003
node_637: feature_name=GO:0070245 feature_id[326].value <= threshold=3.0491198301315308
node_638: feature_name=GO:0038065 feature_id[670].value <= threshold=2.0590850114822388
node_639: feature_name=GO:0005488 feature_id[187].value <= threshold=84.80076217651367
node_640: feature_name=GO:0060249 feature_id[390].value > threshold=0.07226398587226868
node_652: feature_name=GO:0006974 feature_id[516].value > threshold=5.17782768838515e-06
node_654: feature_name=hsa05210 feature_id[358].value <= threshold=18.730005264282227
node_655: feature_name=GO:0071850 feature_id[40].value <= threshold=5.475317001342773
node_656: feature_name=GO:1904029 feature_id[208].value <= threshold=26.575013160705566
node_657: feature_name=GO:0090594 feature_id[738].value <= threshold=3.000791072845459
node_658: feature_name=GO:0010948 feature_id[569].value > threshold=0.04386143572628498
node_666: feature_name=GO:0032764 feature_id[3].value <= threshold=2.5201677083969116
node_667: feature_name=GO:0023026 feature_id[69].value <= threshold=3.2760006189346313
node_668: feature_name=GO:0032464 feature_id[83].value <= threshold=2.5927021503448486
node_669: feature_name=GO:0071901 feature_id[407].value <= threshold=18.238012313842773
node_670: feature_name=GO:0015671 feature_id[103].value <= threshold=1.3113192915916443
node_671: feature_name=GO:0008340 feature_id[539].value <= threshold=4.860779762268066
node_672: feature_name=GO:0046006 feature_id[364].value <= threshold=2.4317692518234253

node_673: feature_name=GO:0042991 feature_id[684].value <= threshold=5.553565502166748
node_674: feature_name=GO:0002327 feature_id[408].value <= threshold=2.404940366744995
node_675: feature_name=GO:0001836 feature_id[450].value <= threshold=8.647934436798096
node_676: feature_name=GO:0048539 feature_id[319].value <= threshold=1.6308802962303162
node_677: feature_name=GO:0022408 feature_id[279].value <= threshold=4.064727783203125
node_678: feature_name=GO:0030099 feature_id[598].value > threshold=0.0001809061213862151
node_684: feature_name=GO:0001816 feature_id[685].value > threshold=1.3516043054551119e-05
node_688: feature_name=GO:0043200 feature_id[706].value > threshold=4.01608681678772
node_698: feature_name=GO:1901099 feature_id[572].value <= threshold=0.4925118386745453
Class: positive genes

Rules_630 passed counts:1
node_0: feature_name=GO:0042113 feature_id[0].value <= threshold=13.408552169799805
node_1: feature_name=GO:0007568 feature_id[534].value <= threshold=5.0313897132873535
node_2: feature_name=GO:0002705 feature_id[541].value <= threshold=3.200145721435547
node_3: feature_name=GO:1901525 feature_id[576].value > threshold=0.4466460347175598
node_617: feature_name=GO:0005622 feature_id[233].value <= threshold=90.85730743408203
node_618: feature_name=GO:1903147 feature_id[200].value <= threshold=7.326428413391113
node_619: feature_name=GO:0003964 feature_id[317].value <= threshold=5.575642108917236
node_620: feature_name=GO:0046498 feature_id[203].value <= threshold=6.620223522186279
node_621: feature_name=GO:0002562 feature_id[25].value <= threshold=10.287704467773438
node_622: feature_name=GO:0006919 feature_id[246].value <= threshold=5.801334381103516
node_623: feature_name=GO:0001889 feature_id[387].value <= threshold=2.679414987564087
node_624: feature_name=GO:0006555 feature_id[514].value <= threshold=7.819535493850708
node_625: feature_name=GO:0080134 feature_id[650].value <= threshold=38.91967582702637
node_626: feature_name=GO:0042127 feature_id[480].value <= threshold=37.72536659240723
node_627: feature_name=GO:2001251 feature_id[220].value <= threshold=32.564422607421875
node_628: feature_name=GO:0002829 feature_id[493].value <= threshold=2.444612741470337
node_629: feature_name=GO:0008588 feature_id[26].value <= threshold=2.3601274490356445
node_630: feature_name=GO:0033151 feature_id[7].value <= threshold=5.134376287460327
node_631: feature_name=GO:0048145 feature_id[348].value <= threshold=8.836549282073975
node_632: feature_name=GO:0030887 feature_id[274].value <= threshold=1.7000296115875244
node_633: feature_name=GO:0097190 feature_id[117].value <= threshold=33.02078819274902
node_634: feature_name=GO:0046685 feature_id[782].value <= threshold=8.040813446044922
node_635: feature_name=GO:0006346 feature_id[236].value <= threshold=6.384642839431763

node_636: feature_name=GO:0048537 feature_id[66].value <= threshold=3.1739262342453003
node_637: feature_name=GO:0070245 feature_id[326].value <= threshold=3.0491198301315308
node_638: feature_name=GO:0038065 feature_id[670].value <= threshold=2.0590850114822388
node_639: feature_name=GO:0005488 feature_id[187].value <= threshold=84.80076217651367
node_640: feature_name=GO:0060249 feature_id[390].value > threshold=0.07226398587226868
node_652: feature_name=GO:0006974 feature_id[516].value > threshold=5.17782768838515e-06
node_654: feature_name=hsa05210 feature_id[358].value <= threshold=18.730005264282227
node_655: feature_name=GO:0071850 feature_id[40].value <= threshold=5.475317001342773
node_656: feature_name=GO:1904029 feature_id[208].value <= threshold=26.575013160705566
node_657: feature_name=GO:0090594 feature_id[738].value <= threshold=3.000791072845459
node_658: feature_name=GO:0010948 feature_id[569].value > threshold=0.04386143572628498
node_666: feature_name=GO:0032764 feature_id[3].value <= threshold=2.5201677083969116
node_667: feature_name=GO:0023026 feature_id[69].value <= threshold=3.2760006189346313
node_668: feature_name=GO:0032464 feature_id[83].value <= threshold=2.5927021503448486
node_669: feature_name=GO:0071901 feature_id[407].value <= threshold=18.238012313842773
node_670: feature_name=GO:0015671 feature_id[103].value <= threshold=1.3113192915916443
node_671: feature_name=GO:0008340 feature_id[539].value <= threshold=4.860779762268066
node_672: feature_name=GO:0046006 feature_id[364].value <= threshold=2.4317692518234253
node_673: feature_name=GO:0042991 feature_id[684].value <= threshold=5.553565502166748
node_674: feature_name=GO:0002327 feature_id[408].value <= threshold=2.404940366744995
node_675: feature_name=GO:0001836 feature_id[450].value <= threshold=8.647934436798096
node_676: feature_name=GO:0048539 feature_id[319].value <= threshold=1.6308802962303162
node_677: feature_name=GO:0022408 feature_id[279].value <= threshold=4.064727783203125
node_678: feature_name=GO:0030099 feature_id[598].value > threshold=0.0001809061213862151
node_684: feature_name=GO:0001816 feature_id[685].value > threshold=1.3516043054551119e-05
node_688: feature_name=GO:0043200 feature_id[706].value <= threshold=4.01608681678772
node_689: feature_name=GO:1904029 feature_id[208].value > threshold=16.503823280334473
node_695: feature_name=GO:0007049 feature_id[259].value <= threshold=10.547314643859863
Class: positive genes

Rules_631 passed counts:1
node_0: feature_name=GO:0042113 feature_id[0].value <= threshold=13.408552169799805
node_1: feature_name=GO:0007568 feature_id[534].value <= threshold=5.0313897132873535
node_2: feature_name=GO:0002705 feature_id[541].value <= threshold=3.200145721435547
node_3: feature_name=GO:1901525 feature_id[576].value > threshold=0.4466460347175598

node_617: feature_name=GO:0005622 feature_id[233].value <= threshold=90.85730743408203
node_618: feature_name=GO:1903147 feature_id[200].value <= threshold=7.326428413391113
node_619: feature_name=GO:0003964 feature_id[317].value <= threshold=5.575642108917236
node_620: feature_name=GO:0046498 feature_id[203].value <= threshold=6.620223522186279
node_621: feature_name=GO:0002562 feature_id[25].value <= threshold=10.287704467773438
node_622: feature_name=GO:0006919 feature_id[246].value <= threshold=5.801334381103516
node_623: feature_name=GO:0001889 feature_id[387].value <= threshold=2.679414987564087
node_624: feature_name=GO:0006555 feature_id[514].value <= threshold=7.819535493850708
node_625: feature_name=GO:0080134 feature_id[650].value <= threshold=38.91967582702637
node_626: feature_name=GO:0042127 feature_id[480].value <= threshold=37.72536659240723
node_627: feature_name=GO:2001251 feature_id[220].value <= threshold=32.564422607421875
node_628: feature_name=GO:0002829 feature_id[493].value <= threshold=2.444612741470337
node_629: feature_name=GO:0008588 feature_id[26].value <= threshold=2.3601274490356445
node_630: feature_name=GO:0033151 feature_id[7].value <= threshold=5.134376287460327
node_631: feature_name=GO:0048145 feature_id[348].value <= threshold=8.836549282073975
node_632: feature_name=GO:0030887 feature_id[274].value <= threshold=1.7000296115875244
node_633: feature_name=GO:0097190 feature_id[117].value <= threshold=33.02078819274902
node_634: feature_name=GO:0046685 feature_id[782].value <= threshold=8.040813446044922
node_635: feature_name=GO:0006346 feature_id[236].value <= threshold=6.384642839431763
node_636: feature_name=GO:0048537 feature_id[66].value <= threshold=3.1739262342453003
node_637: feature_name=GO:0070245 feature_id[326].value <= threshold=3.0491198301315308
node_638: feature_name=GO:0038065 feature_id[670].value <= threshold=2.0590850114822388
node_639: feature_name=GO:0005488 feature_id[187].value <= threshold=84.80076217651367
node_640: feature_name=GO:0060249 feature_id[390].value > threshold=0.07226398587226868
node_652: feature_name=GO:0006974 feature_id[516].value > threshold=5.17782768838515e-06
node_654: feature_name=hsa05210 feature_id[358].value <= threshold=18.730005264282227
node_655: feature_name=GO:0071850 feature_id[40].value <= threshold=5.475317001342773
node_656: feature_name=GO:1904029 feature_id[208].value <= threshold=26.575013160705566
node_657: feature_name=GO:0090594 feature_id[738].value <= threshold=3.000791072845459
node_658: feature_name=GO:0010948 feature_id[569].value > threshold=0.04386143572628498
node_666: feature_name=GO:0032764 feature_id[3].value <= threshold=2.5201677083969116
node_667: feature_name=GO:0023026 feature_id[69].value <= threshold=3.2760006189346313
node_668: feature_name=GO:0032464 feature_id[83].value <= threshold=2.5927021503448486
node_669: feature_name=GO:0071901 feature_id[407].value <= threshold=18.238012313842773
node_670: feature_name=GO:0015671 feature_id[103].value <= threshold=1.3113192915916443

node_671: feature_name=GO:0008340 feature_id[539].value <= threshold=4.860779762268066
node_672: feature_name=GO:0046006 feature_id[364].value <= threshold=2.4317692518234253
node_673: feature_name=GO:0042991 feature_id[684].value <= threshold=5.553565502166748
node_674: feature_name=GO:0002327 feature_id[408].value <= threshold=2.404940366744995
node_675: feature_name=GO:0001836 feature_id[450].value <= threshold=8.647934436798096
node_676: feature_name=GO:0048539 feature_id[319].value <= threshold=1.6308802962303162
node_677: feature_name=GO:0022408 feature_id[279].value <= threshold=4.064727783203125
node_678: feature_name=GO:0030099 feature_id[598].value > threshold=0.0001809061213862151
node_684: feature_name=GO:0001816 feature_id[685].value > threshold=1.3516043054551119e-05
node_688: feature_name=GO:0043200 feature_id[706].value <= threshold=4.01608681678772
node_689: feature_name=GO:1904029 feature_id[208].value <= threshold=16.503823280334473
node_690: feature_name=GO:0045840 feature_id[697].value > threshold=6.23440408706665
node_692: feature_name=GO:0009615 feature_id[265].value > threshold=1.909807026386261
Class: positive genes

Rules_632 passed counts:1
node_0: feature_name=GO:0042113 feature_id[0].value <= threshold=13.408552169799805
node_1: feature_name=GO:0007568 feature_id[534].value <= threshold=5.0313897132873535
node_2: feature_name=GO:0002705 feature_id[541].value <= threshold=3.200145721435547
node_3: feature_name=GO:1901525 feature_id[576].value > threshold=0.4466460347175598
node_617: feature_name=GO:0005622 feature_id[233].value <= threshold=90.85730743408203
node_618: feature_name=GO:1903147 feature_id[200].value <= threshold=7.326428413391113
node_619: feature_name=GO:0003964 feature_id[317].value <= threshold=5.575642108917236
node_620: feature_name=GO:0046498 feature_id[203].value <= threshold=6.620223522186279
node_621: feature_name=GO:0002562 feature_id[25].value <= threshold=10.287704467773438
node_622: feature_name=GO:0006919 feature_id[246].value <= threshold=5.801334381103516
node_623: feature_name=GO:0001889 feature_id[387].value <= threshold=2.679414987564087
node_624: feature_name=GO:0006555 feature_id[514].value <= threshold=7.819535493850708
node_625: feature_name=GO:0080134 feature_id[650].value <= threshold=38.91967582702637
node_626: feature_name=GO:0042127 feature_id[480].value <= threshold=37.72536659240723
node_627: feature_name=GO:2001251 feature_id[220].value <= threshold=32.564422607421875
node_628: feature_name=GO:0002829 feature_id[493].value <= threshold=2.444612741470337
node_629: feature_name=GO:0008588 feature_id[26].value <= threshold=2.3601274490356445
node_630: feature_name=GO:0033151 feature_id[7].value <= threshold=5.134376287460327
node_631: feature_name=GO:0048145 feature_id[348].value <= threshold=8.836549282073975

node_632: feature_name=GO:0030887 feature_id[274].value <= threshold=1.7000296115875244
node_633: feature_name=GO:0097190 feature_id[117].value <= threshold=33.02078819274902
node_634: feature_name=GO:0046685 feature_id[782].value <= threshold=8.040813446044922
node_635: feature_name=GO:0006346 feature_id[236].value <= threshold=6.384642839431763
node_636: feature_name=GO:0048537 feature_id[66].value <= threshold=3.1739262342453003
node_637: feature_name=GO:0070245 feature_id[326].value <= threshold=3.0491198301315308
node_638: feature_name=GO:0038065 feature_id[670].value <= threshold=2.0590850114822388
node_639: feature_name=GO:0005488 feature_id[187].value <= threshold=84.80076217651367
node_640: feature_name=GO:0060249 feature_id[390].value > threshold=0.07226398587226868
node_652: feature_name=GO:0006974 feature_id[516].value > threshold=5.17782768838515e-06
node_654: feature_name=hsa05210 feature_id[358].value <= threshold=18.730005264282227
node_655: feature_name=GO:0071850 feature_id[40].value <= threshold=5.475317001342773
node_656: feature_name=GO:1904029 feature_id[208].value <= threshold=26.575013160705566
node_657: feature_name=GO:0090594 feature_id[738].value <= threshold=3.000791072845459
node_658: feature_name=GO:0010948 feature_id[569].value > threshold=0.04386143572628498
node_666: feature_name=GO:0032764 feature_id[3].value <= threshold=2.5201677083969116
node_667: feature_name=GO:0023026 feature_id[69].value <= threshold=3.2760006189346313
node_668: feature_name=GO:0032464 feature_id[83].value <= threshold=2.5927021503448486
node_669: feature_name=GO:0071901 feature_id[407].value <= threshold=18.238012313842773
node_670: feature_name=GO:0015671 feature_id[103].value <= threshold=1.3113192915916443
node_671: feature_name=GO:0008340 feature_id[539].value <= threshold=4.860779762268066
node_672: feature_name=GO:0046006 feature_id[364].value <= threshold=2.4317692518234253
node_673: feature_name=GO:0042991 feature_id[684].value <= threshold=5.553565502166748
node_674: feature_name=GO:0002327 feature_id[408].value <= threshold=2.404940366744995
node_675: feature_name=GO:0001836 feature_id[450].value <= threshold=8.647934436798096
node_676: feature_name=GO:0048539 feature_id[319].value <= threshold=1.6308802962303162
node_677: feature_name=GO:0022408 feature_id[279].value <= threshold=4.064727783203125
node_678: feature_name=GO:0030099 feature_id[598].value > threshold=0.0001809061213862151
node_684: feature_name=GO:0001816 feature_id[685].value <= threshold=1.3516043054551119e-05
node_685: feature_name=GO:0019104 feature_id[124].value > threshold=0.5158462524414062
Class: positive genes

Rules_633 passed counts:1
node_0: feature_name=GO:0042113 feature_id[0].value <= threshold=13.408552169799805
node_1: feature_name=GO:0007568 feature_id[534].value <= threshold=5.0313897132873535

node_2: feature_name=GO:0002705 feature_id[541].value <= threshold=3.200145721435547
node_3: feature_name=GO:1901525 feature_id[576].value > threshold=0.4466460347175598
node_617: feature_name=GO:0005622 feature_id[233].value <= threshold=90.85730743408203
node_618: feature_name=GO:1903147 feature_id[200].value <= threshold=7.326428413391113
node_619: feature_name=GO:0003964 feature_id[317].value <= threshold=5.575642108917236
node_620: feature_name=GO:0046498 feature_id[203].value <= threshold=6.620223522186279
node_621: feature_name=GO:0002562 feature_id[25].value <= threshold=10.287704467773438
node_622: feature_name=GO:0006919 feature_id[246].value <= threshold=5.801334381103516
node_623: feature_name=GO:0001889 feature_id[387].value <= threshold=2.679414987564087
node_624: feature_name=GO:0006555 feature_id[514].value <= threshold=7.819535493850708
node_625: feature_name=GO:0080134 feature_id[650].value <= threshold=38.91967582702637
node_626: feature_name=GO:0042127 feature_id[480].value <= threshold=37.72536659240723
node_627: feature_name=GO:2001251 feature_id[220].value <= threshold=32.564422607421875
node_628: feature_name=GO:0002829 feature_id[493].value <= threshold=2.444612741470337
node_629: feature_name=GO:0008588 feature_id[26].value <= threshold=2.3601274490356445
node_630: feature_name=GO:0033151 feature_id[7].value <= threshold=5.134376287460327
node_631: feature_name=GO:0048145 feature_id[348].value <= threshold=8.836549282073975
node_632: feature_name=GO:0030887 feature_id[274].value <= threshold=1.7000296115875244
node_633: feature_name=GO:0097190 feature_id[117].value <= threshold=33.02078819274902
node_634: feature_name=GO:0046685 feature_id[782].value <= threshold=8.040813446044922
node_635: feature_name=GO:0006346 feature_id[236].value <= threshold=6.384642839431763
node_636: feature_name=GO:0048537 feature_id[66].value <= threshold=3.1739262342453003
node_637: feature_name=GO:0070245 feature_id[326].value <= threshold=3.0491198301315308
node_638: feature_name=GO:0038065 feature_id[670].value <= threshold=2.0590850114822388
node_639: feature_name=GO:0005488 feature_id[187].value <= threshold=84.80076217651367
node_640: feature_name=GO:0060249 feature_id[390].value > threshold=0.07226398587226868
node_652: feature_name=GO:0006974 feature_id[516].value > threshold=5.17782768838515e-06
node_654: feature_name=hsa05210 feature_id[358].value <= threshold=18.730005264282227
node_655: feature_name=GO:0071850 feature_id[40].value <= threshold=5.475317001342773
node_656: feature_name=GO:1904029 feature_id[208].value <= threshold=26.575013160705566
node_657: feature_name=GO:0090594 feature_id[738].value <= threshold=3.000791072845459
node_658: feature_name=GO:0010948 feature_id[569].value > threshold=0.04386143572628498
node_666: feature_name=GO:0032764 feature_id[3].value <= threshold=2.5201677083969116
node_667: feature_name=GO:0023026 feature_id[69].value <= threshold=3.2760006189346313
node_668: feature_name=GO:0032464 feature_id[83].value <= threshold=2.5927021503448486

node_669: feature_name=GO:0071901 feature_id[407].value <= threshold=18.238012313842773
node_670: feature_name=GO:0015671 feature_id[103].value <= threshold=1.3113192915916443
node_671: feature_name=GO:0008340 feature_id[539].value <= threshold=4.860779762268066
node_672: feature_name=GO:0046006 feature_id[364].value <= threshold=2.4317692518234253
node_673: feature_name=GO:0042991 feature_id[684].value <= threshold=5.553565502166748
node_674: feature_name=GO:0002327 feature_id[408].value <= threshold=2.404940366744995
node_675: feature_name=GO:0001836 feature_id[450].value <= threshold=8.647934436798096
node_676: feature_name=GO:0048539 feature_id[319].value <= threshold=1.6308802962303162
node_677: feature_name=GO:0022408 feature_id[279].value <= threshold=4.064727783203125
node_678: feature_name=GO:0030099 feature_id[598].value <= threshold=0.0001809061213862151
node_679: feature_name=hsa05219 feature_id[162].value > threshold=3.047200083732605
node_681: feature_name=GO:0050865 feature_id[234].value > threshold=0.754447378218174
Class: negative genes

Rules_634 passed counts:1
node_0: feature_name=GO:0042113 feature_id[0].value <= threshold=13.408552169799805
node_1: feature_name=GO:0007568 feature_id[534].value <= threshold=5.0313897132873535
node_2: feature_name=GO:0002705 feature_id[541].value <= threshold=3.200145721435547
node_3: feature_name=GO:1901525 feature_id[576].value > threshold=0.4466460347175598
node_617: feature_name=GO:0005622 feature_id[233].value <= threshold=90.85730743408203
node_618: feature_name=GO:1903147 feature_id[200].value <= threshold=7.326428413391113
node_619: feature_name=GO:0003964 feature_id[317].value <= threshold=5.575642108917236
node_620: feature_name=GO:0046498 feature_id[203].value <= threshold=6.620223522186279
node_621: feature_name=GO:0002562 feature_id[25].value <= threshold=10.287704467773438
node_622: feature_name=GO:0006919 feature_id[246].value <= threshold=5.801334381103516
node_623: feature_name=GO:0001889 feature_id[387].value <= threshold=2.679414987564087
node_624: feature_name=GO:0006555 feature_id[514].value <= threshold=7.819535493850708
node_625: feature_name=GO:0080134 feature_id[650].value <= threshold=38.91967582702637
node_626: feature_name=GO:0042127 feature_id[480].value <= threshold=37.72536659240723
node_627: feature_name=GO:2001251 feature_id[220].value <= threshold=32.564422607421875
node_628: feature_name=GO:0002829 feature_id[493].value <= threshold=2.444612741470337
node_629: feature_name=GO:0008588 feature_id[26].value <= threshold=2.3601274490356445
node_630: feature_name=GO:0033151 feature_id[7].value <= threshold=5.134376287460327
node_631: feature_name=GO:0048145 feature_id[348].value <= threshold=8.836549282073975
node_632: feature_name=GO:0030887 feature_id[274].value <= threshold=1.7000296115875244

node_633: feature_name=GO:0097190 feature_id[117].value <= threshold=33.02078819274902
node_634: feature_name=GO:0046685 feature_id[782].value <= threshold=8.040813446044922
node_635: feature_name=GO:0006346 feature_id[236].value <= threshold=6.384642839431763
node_636: feature_name=GO:0048537 feature_id[66].value <= threshold=3.1739262342453003
node_637: feature_name=GO:0070245 feature_id[326].value <= threshold=3.0491198301315308
node_638: feature_name=GO:0038065 feature_id[670].value <= threshold=2.0590850114822388
node_639: feature_name=GO:0005488 feature_id[187].value <= threshold=84.80076217651367
node_640: feature_name=GO:0060249 feature_id[390].value > threshold=0.07226398587226868
node_652: feature_name=GO:0006974 feature_id[516].value > threshold=5.1778276883851 5e-06
node_654: feature_name=hsa05210 feature_id[358].value <= threshold=18.730005264282227
node_655: feature_name=GO:0071850 feature_id[40].value <= threshold=5.475317001342773
node_656: feature_name=GO:1904029 feature_id[208].value <= threshold=26.575013160705566
node_657: feature_name=GO:0090594 feature_id[738].value <= threshold=3.000791072845459
node_658: feature_name=GO:0010948 feature_id[569].value <= threshold=0.04386143572628498
node_659: feature_name=GO:0002704 feature_id[470].value <= threshold=0.7501857876777649
node_660: feature_name=GO:0006275 feature_id[80].value > threshold=3.2739195823669434
node_662: feature_name=GO:0042981 feature_id[177].value > threshold=3.204610586166382
Class: negative genes

Rules_635 passed counts:1
node_0: feature_name=GO:0042113 feature_id[0].value <= threshold=13.408552169799805
node_1: feature_name=GO:0007568 feature_id[534].value <= threshold=5.0313897132873535
node_2: feature_name=GO:0002705 feature_id[541].value <= threshold=3.200145721435547
node_3: feature_name=GO:1901525 feature_id[576].value > threshold=0.4466460347175598
node_617: feature_name=GO:0005622 feature_id[233].value <= threshold=90.85730743408203
node_618: feature_name=GO:1903147 feature_id[200].value <= threshold=7.326428413391113
node_619: feature_name=GO:0003964 feature_id[317].value <= threshold=5.575642108917236
node_620: feature_name=GO:0046498 feature_id[203].value <= threshold=6.620223522186279
node_621: feature_name=GO:0002562 feature_id[25].value <= threshold=10.287704467773438
node_622: feature_name=GO:0006919 feature_id[246].value <= threshold=5.801334381103516
node_623: feature_name=GO:0001889 feature_id[387].value <= threshold=2.679414987564087
node_624: feature_name=GO:0006555 feature_id[514].value <= threshold=7.819535493850708
node_625: feature_name=GO:0080134 feature_id[650].value <= threshold=38.91967582702637
node_626: feature_name=GO:0042127 feature_id[480].value <= threshold=37.72536659240723
node_627: feature_name=GO:2001251 feature_id[220].value <= threshold=32.564422607421875

node_628: feature_name=GO:0002829 feature_id[493].value <= threshold=2.444612741470337
node_629: feature_name=GO:0008588 feature_id[26].value <= threshold=2.3601274490356445
node_630: feature_name=GO:0033151 feature_id[7].value <= threshold=5.134376287460327
node_631: feature_name=GO:0048145 feature_id[348].value <= threshold=8.836549282073975
node_632: feature_name=GO:0030887 feature_id[274].value <= threshold=1.7000296115875244
node_633: feature_name=GO:0097190 feature_id[117].value <= threshold=33.02078819274902
node_634: feature_name=GO:0046685 feature_id[782].value <= threshold=8.040813446044922
node_635: feature_name=GO:0006346 feature_id[236].value <= threshold=6.384642839431763
node_636: feature_name=GO:0048537 feature_id[66].value <= threshold=3.1739262342453003
node_637: feature_name=GO:0070245 feature_id[326].value <= threshold=3.0491198301315308
node_638: feature_name=GO:0038065 feature_id[670].value <= threshold=2.0590850114822388
node_639: feature_name=GO:0005488 feature_id[187].value <= threshold=84.80076217651367
node_640: feature_name=GO:0060249 feature_id[390].value > threshold=0.07226398587226868
node_652: feature_name=GO:0006974 feature_id[516].value <= threshold=5.17782768838515e-06
Class: positive genes

Rules_636 passed counts:1
node_0: feature_name=GO:0042113 feature_id[0].value <= threshold=13.408552169799805
node_1: feature_name=GO:0007568 feature_id[534].value <= threshold=5.0313897132873535
node_2: feature_name=GO:0002705 feature_id[541].value <= threshold=3.200145721435547
node_3: feature_name=GO:1901525 feature_id[576].value > threshold=0.4466460347175598
node_617: feature_name=GO:0005622 feature_id[233].value <= threshold=90.85730743408203
node_618: feature_name=GO:1903147 feature_id[200].value <= threshold=7.326428413391113
node_619: feature_name=GO:0003964 feature_id[317].value <= threshold=5.575642108917236
node_620: feature_name=GO:0046498 feature_id[203].value <= threshold=6.620223522186279
node_621: feature_name=GO:0002562 feature_id[25].value <= threshold=10.287704467773438
node_622: feature_name=GO:0006919 feature_id[246].value <= threshold=5.801334381103516
node_623: feature_name=GO:0001889 feature_id[387].value <= threshold=2.679414987564087
node_624: feature_name=GO:0006555 feature_id[514].value <= threshold=7.819535493850708
node_625: feature_name=GO:0080134 feature_id[650].value <= threshold=38.91967582702637
node_626: feature_name=GO:0042127 feature_id[480].value <= threshold=37.72536659240723
node_627: feature_name=GO:2001251 feature_id[220].value <= threshold=32.564422607421875
node_628: feature_name=GO:0002829 feature_id[493].value <= threshold=2.444612741470337
node_629: feature_name=GO:0008588 feature_id[26].value <= threshold=2.3601274490356445
node_630: feature_name=GO:0033151 feature_id[7].value <= threshold=5.134376287460327

node_631: feature_name=GO:0048145 feature_id[348].value <= threshold=8.836549282073975
node_632: feature_name=GO:0030887 feature_id[274].value <= threshold=1.7000296115875244
node_633: feature_name=GO:0097190 feature_id[117].value <= threshold=33.02078819274902
node_634: feature_name=GO:0046685 feature_id[782].value <= threshold=8.040813446044922
node_635: feature_name=GO:0006346 feature_id[236].value <= threshold=6.384642839431763
node_636: feature_name=GO:0048537 feature_id[66].value <= threshold=3.1739262342453003
node_637: feature_name=GO:0070245 feature_id[326].value <= threshold=3.0491198301315308
node_638: feature_name=GO:0038065 feature_id[670].value <= threshold=2.0590850114822388
node_639: feature_name=GO:0005488 feature_id[187].value <= threshold=84.80076217651367
node_640: feature_name=GO:0060249 feature_id[390].value <= threshold=0.07226398587226868
node_641: feature_name=GO:0002683 feature_id[456].value > threshold=0.7958214282989502
node_647: feature_name=GO:0045638 feature_id[94].value <= threshold=0.7937153577804565
node_648: feature_name=GO:0007600 feature_id[122].value <= threshold=0.009634195128455758
Class: positive genes

Rules_637 passed counts:1
node_0: feature_name=GO:0042113 feature_id[0].value <= threshold=13.408552169799805
node_1: feature_name=GO:0007568 feature_id[534].value <= threshold=5.0313897132873535
node_2: feature_name=GO:0002705 feature_id[541].value <= threshold=3.200145721435547
node_3: feature_name=GO:1901525 feature_id[576].value <= threshold=0.4466460347175598
node_4: feature_name=GO:0048539 feature_id[319].value <= threshold=3.0399646759033203
node_5: feature_name=GO:0001910 feature_id[385].value <= threshold=3.7437864542007446
node_6: feature_name=GO:0043200 feature_id[706].value <= threshold=9.307284355163574
node_7: feature_name=GO:0001773 feature_id[308].value <= threshold=4.016931533813477
node_8: feature_name=GO:0090116 feature_id[97].value > threshold=7.99645471572876
node_606: feature_name=GO:0042493 feature_id[149].value <= threshold=0.7003275454044342
Class: negative genes

Rules_638 passed counts:1
node_0: feature_name=GO:0042113 feature_id[0].value <= threshold=13.408552169799805
node_1: feature_name=GO:0007568 feature_id[534].value <= threshold=5.0313897132873535
node_2: feature_name=GO:0002705 feature_id[541].value <= threshold=3.200145721435547
node_3: feature_name=GO:1901525 feature_id[576].value <= threshold=0.4466460347175598
node_4: feature_name=GO:0048539 feature_id[319].value <= threshold=3.0399646759033203
node_5: feature_name=GO:0001910 feature_id[385].value <= threshold=3.7437864542007446

node_6: feature_name=GO:0043200 feature_id[706].value <= threshold=9.307284355163574
node_7: feature_name=GO:0001773 feature_id[308].value <= threshold=4.016931533813477
node_8: feature_name=GO:0090116 feature_id[97].value <= threshold=7.99645471572876
node_9: feature_name=GO:0019814 feature_id[189].value > threshold=4.525782108306885
node_603: feature_name=GO:0042802 feature_id[795].value <= threshold=0.0685675137792714
Class: negative genes

Rules_639 passed counts:1
node_0: feature_name=GO:0042113 feature_id[0].value <= threshold=13.408552169799805
node_1: feature_name=GO:0007568 feature_id[534].value <= threshold=5.0313897132873535
node_2: feature_name=GO:0002705 feature_id[541].value <= threshold=3.200145721435547
node_3: feature_name=GO:1901525 feature_id[576].value <= threshold=0.4466460347175598
node_4: feature_name=GO:0048539 feature_id[319].value <= threshold=3.0399646759033203
node_5: feature_name=GO:0001910 feature_id[385].value <= threshold=3.7437864542007446
node_6: feature_name=GO:0043200 feature_id[706].value <= threshold=9.307284355163574
node_7: feature_name=GO:0001773 feature_id[308].value <= threshold=4.016931533813477
node_8: feature_name=GO:0090116 feature_id[97].value <= threshold=7.99645471572876
node_9: feature_name=GO:0019814 feature_id[189].value <= threshold=4.525782108306885
node_10: feature_name=GO:1902583 feature_id[215].value > threshold=17.60310649871826
node_600: feature_name=GO:0046634 feature_id[46].value > threshold=0.2357492558658123
Class: negative genes

Rules_640 passed counts:1
node_0: feature_name=GO:0042113 feature_id[0].value <= threshold=13.408552169799805
node_1: feature_name=GO:0007568 feature_id[534].value <= threshold=5.0313897132873535
node_2: feature_name=GO:0002705 feature_id[541].value <= threshold=3.200145721435547
node_3: feature_name=GO:1901525 feature_id[576].value <= threshold=0.4466460347175598
node_4: feature_name=GO:0048539 feature_id[319].value <= threshold=3.0399646759033203
node_5: feature_name=GO:0001910 feature_id[385].value <= threshold=3.7437864542007446
node_6: feature_name=GO:0043200 feature_id[706].value <= threshold=9.307284355163574
node_7: feature_name=GO:0001773 feature_id[308].value <= threshold=4.016931533813477
node_8: feature_name=GO:0090116 feature_id[97].value <= threshold=7.99645471572876
node_9: feature_name=GO:0019814 feature_id[189].value <= threshold=4.525782108306885
node_10: feature_name=GO:1902583 feature_id[215].value <= threshold=17.60310649871826
node_11: feature_name=GO:0045429 feature_id[737].value > threshold=1.6133361458778381

node_477: feature_name=GO:0036037 feature_id[445].value <= threshold=1.7181594371795654
node_478: feature_name=GO:0006244 feature_id[503].value <= threshold=0.951388418674469
node_479: feature_name=GO:0032461 feature_id[608].value > threshold=2.6103241443634033
node_585: feature_name=GO:1902532 feature_id[302].value <= threshold=1.9927199482917786
Class: negative genes

Rules_641 passed counts:1
node_0: feature_name=GO:0042113 feature_id[0].value <= threshold=13.408552169799805
node_1: feature_name=GO:0007568 feature_id[534].value <= threshold=5.0313897132873535
node_2: feature_name=GO:0002705 feature_id[541].value <= threshold=3.200145721435547
node_3: feature_name=GO:1901525 feature_id[576].value <= threshold=0.4466460347175598
node_4: feature_name=GO:0048539 feature_id[319].value <= threshold=3.0399646759033203
node_5: feature_name=GO:0001910 feature_id[385].value <= threshold=3.7437864542007446
node_6: feature_name=GO:0043200 feature_id[706].value <= threshold=9.307284355163574
node_7: feature_name=GO:0001773 feature_id[308].value <= threshold=4.016931533813477
node_8: feature_name=GO:0090116 feature_id[97].value <= threshold=7.99645471572876
node_9: feature_name=GO:0019814 feature_id[189].value <= threshold=4.525782108306885
node_10: feature_name=GO:1902583 feature_id[215].value <= threshold=17.60310649871826
node_11: feature_name=GO:0045429 feature_id[737].value > threshold=1.6133361458778381
node_477: feature_name=GO:0036037 feature_id[445].value <= threshold=1.7181594371795654
node_478: feature_name=GO:0006244 feature_id[503].value <= threshold=0.951388418674469
node_479: feature_name=GO:0032461 feature_id[608].value <= threshold=2.6103241443634033
node_480: feature_name=GO:0044710 feature_id[719].value > threshold=2.228096718681627e-06
node_494: feature_name=GO:0009314 feature_id[296].value > threshold=34.93696117401123
Class: positive genes

Rules_642 passed counts:1
node_0: feature_name=GO:0042113 feature_id[0].value <= threshold=13.408552169799805
node_1: feature_name=GO:0007568 feature_id[534].value <= threshold=5.0313897132873535
node_2: feature_name=GO:0002705 feature_id[541].value <= threshold=3.200145721435547
node_3: feature_name=GO:1901525 feature_id[576].value <= threshold=0.4466460347175598
node_4: feature_name=GO:0048539 feature_id[319].value <= threshold=3.0399646759033203
node_5: feature_name=GO:0001910 feature_id[385].value <= threshold=3.7437864542007446
node_6: feature_name=GO:0043200 feature_id[706].value <= threshold=9.307284355163574
node_7: feature_name=GO:0001773 feature_id[308].value <= threshold=4.016931533813477

node_8: feature_name=GO:0090116 feature_id[97].value <= threshold=7.99645471572876
node_9: feature_name=GO:0019814 feature_id[189].value <= threshold=4.525782108306885
node_10: feature_name=GO:1902583 feature_id[215].value <= threshold=17.60310649871826
node_11: feature_name=GO:0045429 feature_id[737].value > threshold=1.6133361458778381
node_477: feature_name=GO:0036037 feature_id[445].value <= threshold=1.7181594371795654
node_478: feature_name=GO:0006244 feature_id[503].value <= threshold=0.951388418674469
node_479: feature_name=GO:0032461 feature_id[608].value <= threshold=2.6103241443634033
node_480: feature_name=GO:0044710 feature_id[719].value > threshold=2.228096718681627e-06
node_494: feature_name=GO:0009314 feature_id[296].value <= threshold=34.93696117401123
node_495: feature_name=GO:0050778 feature_id[802].value > threshold=39.29364013671875
Class: positive genes

Rules_643 passed counts:1
node_0: feature_name=GO:0042113 feature_id[0].value <= threshold=13.408552169799805
node_1: feature_name=GO:0007568 feature_id[534].value <= threshold=5.0313897132873535
node_2: feature_name=GO:0002705 feature_id[541].value <= threshold=3.200145721435547
node_3: feature_name=GO:1901525 feature_id[576].value <= threshold=0.4466460347175598
node_4: feature_name=GO:0048539 feature_id[319].value <= threshold=3.0399646759033203
node_5: feature_name=GO:0001910 feature_id[385].value <= threshold=3.7437864542007446
node_6: feature_name=GO:0043200 feature_id[706].value <= threshold=9.307284355163574
node_7: feature_name=GO:0001773 feature_id[308].value <= threshold=4.016931533813477
node_8: feature_name=GO:0090116 feature_id[97].value <= threshold=7.99645471572876
node_9: feature_name=GO:0019814 feature_id[189].value <= threshold=4.525782108306885
node_10: feature_name=GO:1902583 feature_id[215].value <= threshold=17.60310649871826
node_11: feature_name=GO:0045429 feature_id[737].value > threshold=1.6133361458778381
node_477: feature_name=GO:0036037 feature_id[445].value <= threshold=1.7181594371795654
node_478: feature_name=GO:0006244 feature_id[503].value <= threshold=0.951388418674469
node_479: feature_name=GO:0032461 feature_id[608].value <= threshold=2.6103241443634033
node_480: feature_name=GO:0044710 feature_id[719].value > threshold=2.228096718681627e-06
node_494: feature_name=GO:0009314 feature_id[296].value <= threshold=34.93696117401123
node_495: feature_name=GO:0050778 feature_id[802].value <= threshold=39.29364013671875
node_496: feature_name=GO:1903038 feature_id[492].value > threshold=5.849650859832764
Class: positive genes

Rules_644 passed counts:1

node_0: feature_name=GO:0042113 feature_id[0].value <= threshold=13.408552169799805
node_1: feature_name=GO:0007568 feature_id[534].value <= threshold=5.0313897132873535
node_2: feature_name=GO:0002705 feature_id[541].value <= threshold=3.200145721435547
node_3: feature_name=GO:1901525 feature_id[576].value <= threshold=0.4466460347175598
node_4: feature_name=GO:0048539 feature_id[319].value <= threshold=3.0399646759033203
node_5: feature_name=GO:0001910 feature_id[385].value <= threshold=3.7437864542007446
node_6: feature_name=GO:0043200 feature_id[706].value <= threshold=9.307284355163574
node_7: feature_name=GO:0001773 feature_id[308].value <= threshold=4.016931533813477
node_8: feature_name=GO:0090116 feature_id[97].value <= threshold=7.99645471572876
node_9: feature_name=GO:0019814 feature_id[189].value <= threshold=4.525782108306885
node_10: feature_name=GO:1902583 feature_id[215].value <= threshold=17.60310649871826
node_11: feature_name=GO:0045429 feature_id[737].value > threshold=1.6133361458778381
node_477: feature_name=GO:0036037 feature_id[445].value <= threshold=1.7181594371795654
node_478: feature_name=GO:0006244 feature_id[503].value <= threshold=0.951388418674469
node_479: feature_name=GO:0032461 feature_id[608].value <= threshold=2.6103241443634033
node_480: feature_name=GO:0044710 feature_id[719].value > threshold=2.228096718681627e-06
node_494: feature_name=GO:0009314 feature_id[296].value <= threshold=34.93696117401123
node_495: feature_name=GO:0050778 feature_id[802].value <= threshold=39.29364013671875
node_496: feature_name=GO:1903038 feature_id[492].value <= threshold=5.849650859832764
node_497: feature_name=GO:0002699 feature_id[606].value > threshold=5.5405943393707275
Class: positive genes

Rules_645 passed counts:1
node_0: feature_name=GO:0042113 feature_id[0].value <= threshold=13.408552169799805
node_1: feature_name=GO:0007568 feature_id[534].value <= threshold=5.0313897132873535
node_2: feature_name=GO:0002705 feature_id[541].value <= threshold=3.200145721435547
node_3: feature_name=GO:1901525 feature_id[576].value <= threshold=0.4466460347175598
node_4: feature_name=GO:0048539 feature_id[319].value <= threshold=3.0399646759033203
node_5: feature_name=GO:0001910 feature_id[385].value <= threshold=3.7437864542007446
node_6: feature_name=GO:0043200 feature_id[706].value <= threshold=9.307284355163574
node_7: feature_name=GO:0001773 feature_id[308].value <= threshold=4.016931533813477
node_8: feature_name=GO:0090116 feature_id[97].value <= threshold=7.99645471572876
node_9: feature_name=GO:0019814 feature_id[189].value <= threshold=4.525782108306885
node_10: feature_name=GO:1902583 feature_id[215].value <= threshold=17.60310649871826
node_11: feature_name=GO:0045429 feature_id[737].value > threshold=1.6133361458778381

node_477: feature_name=GO:0036037 feature_id[445].value <= threshold=1.7181594371795654
node_478: feature_name=GO:0006244 feature_id[503].value <= threshold=0.951388418674469
node_479: feature_name=GO:0032461 feature_id[608].value <= threshold=2.6103241443634033
node_480: feature_name=GO:0044710 feature_id[719].value > threshold=2.228096718681627e-06
node_494: feature_name=GO:0009314 feature_id[296].value <= threshold=34.93696117401123
node_495: feature_name=GO:0050778 feature_id[802].value <= threshold=39.29364013671875
node_496: feature_name=GO:1903038 feature_id[492].value <= threshold=5.849650859832764
node_497: feature_name=GO:0002699 feature_id[606].value <= threshold=5.5405943393707275
node_498: feature_name=hsa00983 feature_id[181].value > threshold=25.851045608520508
Class: positive genes

Rules_646 passed counts:1
node_0: feature_name=GO:0042113 feature_id[0].value <= threshold=13.408552169799805
node_1: feature_name=GO:0007568 feature_id[534].value <= threshold=5.0313897132873535
node_2: feature_name=GO:0002705 feature_id[541].value <= threshold=3.200145721435547
node_3: feature_name=GO:1901525 feature_id[576].value <= threshold=0.4466460347175598
node_4: feature_name=GO:0048539 feature_id[319].value <= threshold=3.0399646759033203
node_5: feature_name=GO:0001910 feature_id[385].value <= threshold=3.7437864542007446
node_6: feature_name=GO:0043200 feature_id[706].value <= threshold=9.307284355163574
node_7: feature_name=GO:0001773 feature_id[308].value <= threshold=4.016931533813477
node_8: feature_name=GO:0090116 feature_id[97].value <= threshold=7.99645471572876
node_9: feature_name=GO:0019814 feature_id[189].value <= threshold=4.525782108306885
node_10: feature_name=GO:1902583 feature_id[215].value <= threshold=17.60310649871826
node_11: feature_name=GO:0045429 feature_id[737].value > threshold=1.6133361458778381
node_477: feature_name=GO:0036037 feature_id[445].value <= threshold=1.7181594371795654
node_478: feature_name=GO:0006244 feature_id[503].value <= threshold=0.951388418674469
node_479: feature_name=GO:0032461 feature_id[608].value <= threshold=2.6103241443634033
node_480: feature_name=GO:0044710 feature_id[719].value > threshold=2.228096718681627e-06
node_494: feature_name=GO:0009314 feature_id[296].value <= threshold=34.93696117401123
node_495: feature_name=GO:0050778 feature_id[802].value <= threshold=39.29364013671875
node_496: feature_name=GO:1903038 feature_id[492].value <= threshold=5.849650859832764
node_497: feature_name=GO:0002699 feature_id[606].value <= threshold=5.5405943393707275
node_498: feature_name=hsa00983 feature_id[181].value <= threshold=25.851045608520508
node_499: feature_name=GO:0002309 feature_id[416].value > threshold=1.6235689520835876
Class: positive genes

Rules_647 passed counts:1
node_0: feature_name=GO:0042113 feature_id[0].value <= threshold=13.408552169799805
node_1: feature_name=GO:0007568 feature_id[534].value <= threshold=5.0313897132873535
node_2: feature_name=GO:0002705 feature_id[541].value <= threshold=3.200145721435547
node_3: feature_name=GO:1901525 feature_id[576].value <= threshold=0.4466460347175598
node_4: feature_name=GO:0048539 feature_id[319].value <= threshold=3.0399646759033203
node_5: feature_name=GO:0001910 feature_id[385].value <= threshold=3.7437864542007446
node_6: feature_name=GO:0043200 feature_id[706].value <= threshold=9.307284355163574
node_7: feature_name=GO:0001773 feature_id[308].value <= threshold=4.016931533813477
node_8: feature_name=GO:0090116 feature_id[97].value <= threshold=7.99645471572876
node_9: feature_name=GO:0019814 feature_id[189].value <= threshold=4.525782108306885
node_10: feature_name=GO:1902583 feature_id[215].value <= threshold=17.60310649871826
node_11: feature_name=GO:0045429 feature_id[737].value > threshold=1.6133361458778381
node_477: feature_name=GO:0036037 feature_id[445].value <= threshold=1.7181594371795654
node_478: feature_name=GO:0006244 feature_id[503].value <= threshold=0.951388418674469
node_479: feature_name=GO:0032461 feature_id[608].value <= threshold=2.6103241443634033
node_480: feature_name=GO:0044710 feature_id[719].value > threshold=2.228096718681627e-06
node_494: feature_name=GO:0009314 feature_id[296].value <= threshold=34.93696117401123
node_495: feature_name=GO:0050778 feature_id[802].value <= threshold=39.29364013671875
node_496: feature_name=GO:1903038 feature_id[492].value <= threshold=5.849650859832764
node_497: feature_name=GO:0002699 feature_id[606].value <= threshold=5.5405943393707275
node_498: feature_name=hsa00983 feature_id[181].value <= threshold=25.851045608520508
node_499: feature_name=GO:0002309 feature_id[416].value <= threshold=1.6235689520835876
node_500: feature_name=GO:1901698 feature_id[792].value > threshold=47.70275688171387
Class: positive genes

Rules_648 passed counts:1
node_0: feature_name=GO:0042113 feature_id[0].value <= threshold=13.408552169799805
node_1: feature_name=GO:0007568 feature_id[534].value <= threshold=5.0313897132873535
node_2: feature_name=GO:0002705 feature_id[541].value <= threshold=3.200145721435547
node_3: feature_name=GO:1901525 feature_id[576].value <= threshold=0.4466460347175598
node_4: feature_name=GO:0048539 feature_id[319].value <= threshold=3.0399646759033203
node_5: feature_name=GO:0001910 feature_id[385].value <= threshold=3.7437864542007446
node_6: feature_name=GO:0043200 feature_id[706].value <= threshold=9.307284355163574

node_7: feature_name=GO:0001773 feature_id[308].value <= threshold=4.016931533813477
node_8: feature_name=GO:0090116 feature_id[97].value <= threshold=7.99645471572876
node_9: feature_name=GO:0019814 feature_id[189].value <= threshold=4.525782108306885
node_10: feature_name=GO:1902583 feature_id[215].value <= threshold=17.60310649871826
node_11: feature_name=GO:0045429 feature_id[737].value > threshold=1.6133361458778381
node_477: feature_name=GO:0036037 feature_id[445].value <= threshold=1.7181594371795654
node_478: feature_name=GO:0006244 feature_id[503].value <= threshold=0.951388418674469
node_479: feature_name=GO:0032461 feature_id[608].value <= threshold=2.6103241443634033
node_480: feature_name=GO:0044710 feature_id[719].value > threshold=2.228096718681627e-06
node_494: feature_name=GO:0009314 feature_id[296].value <= threshold=34.93696117401123
node_495: feature_name=GO:0050778 feature_id[802].value <= threshold=39.29364013671875
node_496: feature_name=GO:1903038 feature_id[492].value <= threshold=5.849650859832764
node_497: feature_name=GO:0002699 feature_id[606].value <= threshold=5.5405943393707275
node_498: feature_name=hsa00983 feature_id[181].value <= threshold=25.851045608520508
node_499: feature_name=GO:0002309 feature_id[416].value <= threshold=1.6235689520835876
node_500: feature_name=GO:1901698 feature_id[792].value <= threshold=47.70275688171387
node_501: feature_name=GO:0010216 feature_id[282].value <= threshold=1.6849713921546936
node_502: feature_name=GO:0006266 feature_id[297].value <= threshold=2.7199249267578125
node_503: feature_name=GO:0005488 feature_id[187].value > threshold=3.0373169010999845e-06
node_507: feature_name=GO:0045429 feature_id[737].value > threshold=1.614579975605011
node_511: feature_name=GO:0034103 feature_id[318].value > threshold=5.832815647125244
node_569: feature_name=GO:0006979 feature_id[44].value > threshold=2.6204053163528442
Class: negative genes

Rules_649 passed counts:1
node_0: feature_name=GO:0042113 feature_id[0].value <= threshold=13.408552169799805
node_1: feature_name=GO:0007568 feature_id[534].value <= threshold=5.0313897132873535
node_2: feature_name=GO:0002705 feature_id[541].value <= threshold=3.200145721435547
node_3: feature_name=GO:1901525 feature_id[576].value <= threshold=0.4466460347175598
node_4: feature_name=GO:0048539 feature_id[319].value <= threshold=3.0399646759033203
node_5: feature_name=GO:0001910 feature_id[385].value <= threshold=3.7437864542007446
node_6: feature_name=GO:0043200 feature_id[706].value <= threshold=9.307284355163574
node_7: feature_name=GO:0001773 feature_id[308].value <= threshold=4.016931533813477
node_8: feature_name=GO:0090116 feature_id[97].value <= threshold=7.99645471572876
node_9: feature_name=GO:0019814 feature_id[189].value <= threshold=4.525782108306885

node_10: feature_name=GO:1902583 feature_id[215].value <= threshold=17.60310649871826
node_11: feature_name=GO:0045429 feature_id[737].value > threshold=1.6133361458778381
node_477: feature_name=GO:0036037 feature_id[445].value <= threshold=1.7181594371795654
node_478: feature_name=GO:0006244 feature_id[503].value <= threshold=0.951388418674469
node_479: feature_name=GO:0032461 feature_id[608].value <= threshold=2.6103241443634033
node_480: feature_name=GO:0044710 feature_id[719].value > threshold=2.228096718681627e-06
node_494: feature_name=GO:0009314 feature_id[296].value <= threshold=34.93696117401123
node_495: feature_name=GO:0050778 feature_id[802].value <= threshold=39.29364013671875
node_496: feature_name=GO:1903038 feature_id[492].value <= threshold=5.849650859832764
node_497: feature_name=GO:0002699 feature_id[606].value <= threshold=5.5405943393707275
node_498: feature_name=hsa00983 feature_id[181].value <= threshold=25.851045608520508
node_499: feature_name=GO:0002309 feature_id[416].value <= threshold=1.6235689520835876
node_500: feature_name=GO:1901698 feature_id[792].value <= threshold=47.70275688171387
node_501: feature_name=GO:0010216 feature_id[282].value <= threshold=1.6849713921546936
node_502: feature_name=GO:0006266 feature_id[297].value <= threshold=2.7199249267578125
node_503: feature_name=GO:0005488 feature_id[187].value > threshold=3.0373169010999845e-06
node_507: feature_name=GO:0045429 feature_id[737].value > threshold=1.614579975605011
node_511: feature_name=GO:0034103 feature_id[318].value > threshold=5.832815647125244
node_569: feature_name=GO:0006979 feature_id[44].value <= threshold=2.6204053163528442
Class: positive genes

Rules_650 passed counts:1
node_0: feature_name=GO:0042113 feature_id[0].value <= threshold=13.408552169799805
node_1: feature_name=GO:0007568 feature_id[534].value <= threshold=5.0313897132873535
node_2: feature_name=GO:0002705 feature_id[541].value <= threshold=3.200145721435547
node_3: feature_name=GO:1901525 feature_id[576].value <= threshold=0.4466460347175598
node_4: feature_name=GO:0048539 feature_id[319].value <= threshold=3.0399646759033203
node_5: feature_name=GO:0001910 feature_id[385].value <= threshold=3.7437864542007446
node_6: feature_name=GO:0043200 feature_id[706].value <= threshold=9.307284355163574
node_7: feature_name=GO:0001773 feature_id[308].value <= threshold=4.016931533813477
node_8: feature_name=GO:0090116 feature_id[97].value <= threshold=7.99645471572876
node_9: feature_name=GO:0019814 feature_id[189].value <= threshold=4.525782108306885
node_10: feature_name=GO:1902583 feature_id[215].value <= threshold=17.60310649871826
node_11: feature_name=GO:0045429 feature_id[737].value > threshold=1.6133361458778381
node_477: feature_name=GO:0036037 feature_id[445].value <= threshold=1.7181594371795654

node_478: feature_name=GO:0006244 feature_id[503].value <= threshold=0.951388418674469
node_479: feature_name=GO:0032461 feature_id[608].value <= threshold=2.6103241443634033
node_480: feature_name=GO:0044710 feature_id[719].value > threshold=2.228096718681627e-06
node_494: feature_name=GO:0009314 feature_id[296].value <= threshold=34.93696117401123
node_495: feature_name=GO:0050778 feature_id[802].value <= threshold=39.29364013671875
node_496: feature_name=GO:1903038 feature_id[492].value <= threshold=5.849650859832764
node_497: feature_name=GO:0002699 feature_id[606].value <= threshold=5.5405943393707275
node_498: feature_name=hsa00983 feature_id[181].value <= threshold=25.851045608520508
node_499: feature_name=GO:0002309 feature_id[416].value <= threshold=1.6235689520835876
node_500: feature_name=GO:1901698 feature_id[792].value <= threshold=47.70275688171387
node_501: feature_name=GO:0010216 feature_id[282].value <= threshold=1.6849713921546936
node_502: feature_name=GO:0006266 feature_id[297].value <= threshold=2.7199249267578125
node_503: feature_name=GO:0005488 feature_id[187].value > threshold=3.0373169010999845e-06
node_507: feature_name=GO:0045429 feature_id[737].value > threshold=1.614579975605011
node_511: feature_name=GO:0034103 feature_id[318].value <= threshold=5.832815647125244
node_512: feature_name=GO:0010663 feature_id[561].value > threshold=4.157005429267883
node_566: feature_name=GO:2001243 feature_id[92].value > threshold=1.4889216423034668
Class: negative genes

Rules_651 passed counts:1
node_0: feature_name=GO:0042113 feature_id[0].value <= threshold=13.408552169799805
node_1: feature_name=GO:0007568 feature_id[534].value <= threshold=5.0313897132873535
node_2: feature_name=GO:0002705 feature_id[541].value <= threshold=3.200145721435547
node_3: feature_name=GO:1901525 feature_id[576].value <= threshold=0.4466460347175598
node_4: feature_name=GO:0048539 feature_id[319].value <= threshold=3.0399646759033203
node_5: feature_name=GO:0001910 feature_id[385].value <= threshold=3.7437864542007446
node_6: feature_name=GO:0043200 feature_id[706].value <= threshold=9.307284355163574
node_7: feature_name=GO:0001773 feature_id[308].value <= threshold=4.016931533813477
node_8: feature_name=GO:0090116 feature_id[97].value <= threshold=7.99645471572876
node_9: feature_name=GO:0019814 feature_id[189].value <= threshold=4.525782108306885
node_10: feature_name=GO:1902583 feature_id[215].value <= threshold=17.60310649871826
node_11: feature_name=GO:0045429 feature_id[737].value > threshold=1.6133361458778381
node_477: feature_name=GO:0036037 feature_id[445].value <= threshold=1.7181594371795654
node_478: feature_name=GO:0006244 feature_id[503].value <= threshold=0.951388418674469
node_479: feature_name=GO:0032461 feature_id[608].value <= threshold=2.6103241443634033

node_480: feature_name=GO:0044710 feature_id[719].value > threshold=2.228096718681627e-06
node_494: feature_name=GO:0009314 feature_id[296].value <= threshold=34.93696117401123
node_495: feature_name=GO:0050778 feature_id[802].value <= threshold=39.29364013671875
node_496: feature_name=GO:1903038 feature_id[492].value <= threshold=5.849650859832764
node_497: feature_name=GO:0002699 feature_id[606].value <= threshold=5.5405943393707275
node_498: feature_name=hsa00983 feature_id[181].value <= threshold=25.851045608520508
node_499: feature_name=GO:0002309 feature_id[416].value <= threshold=1.6235689520835876
node_500: feature_name=GO:1901698 feature_id[792].value <= threshold=47.70275688171387
node_501: feature_name=GO:0010216 feature_id[282].value <= threshold=1.6849713921546936
node_502: feature_name=GO:0006266 feature_id[297].value <= threshold=2.7199249267578125
node_503: feature_name=GO:0005488 feature_id[187].value > threshold=3.0373169010999845e-06
node_507: feature_name=GO:0045429 feature_id[737].value > threshold=1.614579975605011
node_511: feature_name=GO:0034103 feature_id[318].value <= threshold=5.832815647125244
node_512: feature_name=GO:0010663 feature_id[561].value > threshold=4.157005429267883
node_566: feature_name=GO:2001243 feature_id[92].value <= threshold=1.4889216423034668
Class: positive genes

Rules_652 passed counts:1
node_0: feature_name=GO:0042113 feature_id[0].value <= threshold=13.408552169799805
node_1: feature_name=GO:0007568 feature_id[534].value <= threshold=5.0313897132873535
node_2: feature_name=GO:0002705 feature_id[541].value <= threshold=3.200145721435547
node_3: feature_name=GO:1901525 feature_id[576].value <= threshold=0.4466460347175598
node_4: feature_name=GO:0048539 feature_id[319].value <= threshold=3.0399646759033203
node_5: feature_name=GO:0001910 feature_id[385].value <= threshold=3.7437864542007446
node_6: feature_name=GO:0043200 feature_id[706].value <= threshold=9.307284355163574
node_7: feature_name=GO:0001773 feature_id[308].value <= threshold=4.016931533813477
node_8: feature_name=GO:0090116 feature_id[97].value <= threshold=7.99645471572876
node_9: feature_name=GO:0019814 feature_id[189].value <= threshold=4.525782108306885
node_10: feature_name=GO:1902583 feature_id[215].value <= threshold=17.60310649871826
node_11: feature_name=GO:0045429 feature_id[737].value > threshold=1.6133361458778381
node_477: feature_name=GO:0036037 feature_id[445].value <= threshold=1.7181594371795654
node_478: feature_name=GO:0006244 feature_id[503].value <= threshold=0.951388418674469
node_479: feature_name=GO:0032461 feature_id[608].value <= threshold=2.6103241443634033
node_480: feature_name=GO:0044710 feature_id[719].value > threshold=2.228096718681627e-06
node_494: feature_name=GO:0009314 feature_id[296].value <= threshold=34.93696117401123

node_495: feature_name=GO:0050778	feature_id[802].value <= threshold=39.29364013671875
node_496: feature_name=GO:1903038	feature_id[492].value <= threshold=5.849650859832764
node_497: feature_name=GO:0002699	feature_id[606].value <= threshold=5.5405943393707275
node_498: feature_name=hsa00983	feature_id[181].value <= threshold=25.851045608520508
node_499: feature_name=GO:0002309	feature_id[416].value <= threshold=1.6235689520835876
node_500: feature_name=GO:1901698	feature_id[792].value <= threshold=47.70275688171387
node_501: feature_name=GO:0010216	feature_id[282].value <= threshold=1.6849713921546936
node_502: feature_name=GO:0006266	feature_id[297].value <= threshold=2.7199249267578125
node_503: feature_name=GO:0005488	feature_id[187].value > threshold=3.0373169010999845e-06
node_507: feature_name=GO:0045429	feature_id[737].value > threshold=1.614579975605011
node_511: feature_name=GO:0034103	feature_id[318].value <= threshold=5.832815647125244
node_512: feature_name=GO:0010663	feature_id[561].value <= threshold=4.157005429267883
node_513: feature_name=GO:0030887	feature_id[274].value > threshold=1.8964150547981262
node_563: feature_name=GO:0031667	feature_id[648].value > threshold=0.5378098785877228

Class: negative genes

Rules_653	passed counts:1
node_0: feature_name=GO:0042113	feature_id[0].value <= threshold=13.408552169799805
node_1: feature_name=GO:0007568	feature_id[534].value <= threshold=5.0313897132873535
node_2: feature_name=GO:0002705	feature_id[541].value <= threshold=3.200145721435547
node_3: feature_name=GO:1901525	feature_id[576].value <= threshold=0.4466460347175598
node_4: feature_name=GO:0048539	feature_id[319].value <= threshold=3.0399646759033203
node_5: feature_name=GO:0001910	feature_id[385].value <= threshold=3.7437864542007446
node_6: feature_name=GO:0043200	feature_id[706].value <= threshold=9.307284355163574
node_7: feature_name=GO:0001773	feature_id[308].value <= threshold=4.016931533813477
node_8: feature_name=GO:0090116	feature_id[97].value <= threshold=7.99645471572876
node_9: feature_name=GO:0019814	feature_id[189].value <= threshold=4.525782108306885
node_10: feature_name=GO:1902583	feature_id[215].value <= threshold=17.60310649871826
node_11: feature_name=GO:0045429	feature_id[737].value > threshold=1.6133361458778381
node_477: feature_name=GO:0036037	feature_id[445].value <= threshold=1.7181594371795654
node_478: feature_name=GO:0006244	feature_id[503].value <= threshold=0.951388418674469
node_479: feature_name=GO:0032461	feature_id[608].value <= threshold=2.6103241443634033
node_480: feature_name=GO:0044710	feature_id[719].value > threshold=2.228096718681627e-06
node_494: feature_name=GO:0009314	feature_id[296].value <= threshold=34.93696117401123
node_495: feature_name=GO:0050778	feature_id[802].value <= threshold=39.29364013671875

node_496: feature_name=GO:1903038 feature_id[492].value <= threshold=5.849650859832764
node_497: feature_name=GO:0002699 feature_id[606].value <= threshold=5.5405943393707275
node_498: feature_name=hsa00983 feature_id[181].value <= threshold=25.851045608520508
node_499: feature_name=GO:0002309 feature_id[416].value <= threshold=1.6235689520835876
node_500: feature_name=GO:1901698 feature_id[792].value <= threshold=47.70275688171387
node_501: feature_name=GO:0010216 feature_id[282].value <= threshold=1.6849713921546936
node_502: feature_name=GO:0006266 feature_id[297].value <= threshold=2.7199249267578125
node_503: feature_name=GO:0005488 feature_id[187].value > threshold=3.0373169010999845e-06
node_507: feature_name=GO:0045429 feature_id[737].value > threshold=1.614579975605011
node_511: feature_name=GO:0034103 feature_id[318].value <= threshold=5.832815647125244
node_512: feature_name=GO:0010663 feature_id[561].value <= threshold=4.157005429267883
node_513: feature_name=GO:0030887 feature_id[274].value > threshold=1.8964150547981262
node_563: feature_name=GO:0031667 feature_id[648].value <= threshold=0.5378098785877228
Class: positive genes

Rules_654 passed counts:1
node_0: feature_name=GO:0042113 feature_id[0].value <= threshold=13.408552169799805
node_1: feature_name=GO:0007568 feature_id[534].value <= threshold=5.0313897132873535
node_2: feature_name=GO:0002705 feature_id[541].value <= threshold=3.200145721435547
node_3: feature_name=GO:1901525 feature_id[576].value <= threshold=0.4466460347175598
node_4: feature_name=GO:0048539 feature_id[319].value <= threshold=3.0399646759033203
node_5: feature_name=GO:0001910 feature_id[385].value <= threshold=3.7437864542007446
node_6: feature_name=GO:0043200 feature_id[706].value <= threshold=9.307284355163574
node_7: feature_name=GO:0001773 feature_id[308].value <= threshold=4.016931533813477
node_8: feature_name=GO:0090116 feature_id[97].value <= threshold=7.99645471572876
node_9: feature_name=GO:0019814 feature_id[189].value <= threshold=4.525782108306885
node_10: feature_name=GO:1902583 feature_id[215].value <= threshold=17.60310649871826
node_11: feature_name=GO:0045429 feature_id[737].value > threshold=1.6133361458778381
node_477: feature_name=GO:0036037 feature_id[445].value <= threshold=1.7181594371795654
node_478: feature_name=GO:0006244 feature_id[503].value <= threshold=0.951388418674469
node_479: feature_name=GO:0032461 feature_id[608].value <= threshold=2.6103241443634033
node_480: feature_name=GO:0044710 feature_id[719].value > threshold=2.228096718681627e-06
node_494: feature_name=GO:0009314 feature_id[296].value <= threshold=34.93696117401123
node_495: feature_name=GO:0050778 feature_id[802].value <= threshold=39.29364013671875
node_496: feature_name=GO:1903038 feature_id[492].value <= threshold=5.849650859832764

node_497: feature_name=GO:0002699	feature_id[606].value <= threshold=5.5405943393707275
node_498: feature_name=hsa00983	feature_id[181].value <= threshold=25.851045608520508
node_499: feature_name=GO:0002309	feature_id[416].value <= threshold=1.6235689520835876
node_500: feature_name=GO:1901698	feature_id[792].value <= threshold=47.70275688171387
node_501: feature_name=GO:0010216	feature_id[282].value <= threshold=1.6849713921546936
node_502: feature_name=GO:0006266	feature_id[297].value <= threshold=2.7199249267578125
node_503: feature_name=GO:0005488	feature_id[187].value > threshold=3.0373169010999845e-06
node_507: feature_name=GO:0045429	feature_id[737].value > threshold=1.614579975605011
node_511: feature_name=GO:0034103	feature_id[318].value <= threshold=5.832815647125244
node_512: feature_name=GO:0010663	feature_id[561].value <= threshold=4.157005429267883
node_513: feature_name=GO:0030887	feature_id[274].value <= threshold=1.8964150547981262
node_514: feature_name=GO:0048294	feature_id[113].value > threshold=1.866134524345398
node_560: feature_name=GO:0001889	feature_id[387].value > threshold=0.44509559869766235
Class: negative genes	
Rules_655	passed counts:1
node_0: feature_name=GO:0042113	feature_id[0].value <= threshold=13.408552169799805
node_1: feature_name=GO:0007568	feature_id[534].value <= threshold=5.0313897132873535
node_2: feature_name=GO:0002705	feature_id[541].value <= threshold=3.200145721435547
node_3: feature_name=GO:1901525	feature_id[576].value <= threshold=0.4466460347175598
node_4: feature_name=GO:0048539	feature_id[319].value <= threshold=3.0399646759033203
node_5: feature_name=GO:0001910	feature_id[385].value <= threshold=3.7437864542007446
node_6: feature_name=GO:0043200	feature_id[706].value <= threshold=9.307284355163574
node_7: feature_name=GO:0001773	feature_id[308].value <= threshold=4.016931533813477
node_8: feature_name=GO:0090116	feature_id[97].value <= threshold=7.99645471572876
node_9: feature_name=GO:0019814	feature_id[189].value <= threshold=4.525782108306885
node_10: feature_name=GO:1902583	feature_id[215].value <= threshold=17.60310649871826
node_11: feature_name=GO:0045429	feature_id[737].value > threshold=1.6133361458778381
node_477: feature_name=GO:0036037	feature_id[445].value <= threshold=1.7181594371795654
node_478: feature_name=GO:0006244	feature_id[503].value <= threshold=0.951388418674469
node_479: feature_name=GO:0032461	feature_id[608].value <= threshold=2.6103241443634033
node_480: feature_name=GO:0044710	feature_id[719].value > threshold=2.228096718681627e-06
node_494: feature_name=GO:0009314	feature_id[296].value <= threshold=34.93696117401123
node_495: feature_name=GO:0050778	feature_id[802].value <= threshold=39.29364013671875
node_496: feature_name=GO:1903038	feature_id[492].value <= threshold=5.849650859832764

node_497: feature_name=GO:0002699 feature_id[606].value <= threshold=5.5405943393707275
node_498: feature_name=hsa00983 feature_id[181].value <= threshold=25.851045608520508
node_499: feature_name=GO:0002309 feature_id[416].value <= threshold=1.6235689520835876
node_500: feature_name=GO:1901698 feature_id[792].value <= threshold=47.70275688171387
node_501: feature_name=GO:0010216 feature_id[282].value <= threshold=1.6849713921546936
node_502: feature_name=GO:0006266 feature_id[297].value <= threshold=2.7199249267578125
node_503: feature_name=GO:0005488 feature_id[187].value > threshold=3.0373169010999845e-06
node_507: feature_name=GO:0045429 feature_id[737].value > threshold=1.614579975605011
node_511: feature_name=GO:0034103 feature_id[318].value <= threshold=5.832815647125244
node_512: feature_name=GO:0010663 feature_id[561].value <= threshold=4.157005429267883
node_513: feature_name=GO:0030887 feature_id[274].value <= threshold=1.8964150547981262
node_514: feature_name=GO:0048294 feature_id[113].value > threshold=1.866134524345398
node_560: feature_name=GO:0001889 feature_id[387].value <= threshold=0.44509559869766235
Class: positive genes

Rules_656 passed counts:1
node_0: feature_name=GO:0042113 feature_id[0].value <= threshold=13.408552169799805
node_1: feature_name=GO:0007568 feature_id[534].value <= threshold=5.0313897132873535
node_2: feature_name=GO:0002705 feature_id[541].value <= threshold=3.200145721435547
node_3: feature_name=GO:1901525 feature_id[576].value <= threshold=0.4466460347175598
node_4: feature_name=GO:0048539 feature_id[319].value <= threshold=3.0399646759033203
node_5: feature_name=GO:0001910 feature_id[385].value <= threshold=3.7437864542007446
node_6: feature_name=GO:0043200 feature_id[706].value <= threshold=9.307284355163574
node_7: feature_name=GO:0001773 feature_id[308].value <= threshold=4.016931533813477
node_8: feature_name=GO:0090116 feature_id[97].value <= threshold=7.99645471572876
node_9: feature_name=GO:0019814 feature_id[189].value <= threshold=4.525782108306885
node_10: feature_name=GO:1902583 feature_id[215].value <= threshold=17.60310649871826
node_11: feature_name=GO:0045429 feature_id[737].value > threshold=1.6133361458778381
node_477: feature_name=GO:0036037 feature_id[445].value <= threshold=1.7181594371795654
node_478: feature_name=GO:0006244 feature_id[503].value <= threshold=0.951388418674469
node_479: feature_name=GO:0032461 feature_id[608].value <= threshold=2.6103241443634033
node_480: feature_name=GO:0044710 feature_id[719].value > threshold=2.228096718681627e-06
node_494: feature_name=GO:0009314 feature_id[296].value <= threshold=34.93696117401123
node_495: feature_name=GO:0050778 feature_id[802].value <= threshold=39.29364013671875
node_496: feature_name=GO:1903038 feature_id[492].value <= threshold=5.849650859832764

node_497: feature_name=GO:0002699 feature_id[606].value <= threshold=5.5405943393707275
node_498: feature_name=hsa00983 feature_id[181].value <= threshold=25.851045608520508
node_499: feature_name=GO:0002309 feature_id[416].value <= threshold=1.6235689520835876
node_500: feature_name=GO:1901698 feature_id[792].value <= threshold=47.70275688171387
node_501: feature_name=GO:0010216 feature_id[282].value <= threshold=1.6849713921546936
node_502: feature_name=GO:0006266 feature_id[297].value <= threshold=2.7199249267578125
node_503: feature_name=GO:0005488 feature_id[187].value > threshold=3.0373169010999845e-06
node_507: feature_name=GO:0045429 feature_id[737].value > threshold=1.614579975605011
node_511: feature_name=GO:0034103 feature_id[318].value <= threshold=5.832815647125244
node_512: feature_name=GO:0010663 feature_id[561].value <= threshold=4.157005429267883
node_513: feature_name=GO:0030887 feature_id[274].value <= threshold=1.8964150547981262
node_514: feature_name=GO:0048294 feature_id[113].value <= threshold=1.866134524345398
node_515: feature_name=GO:0072539 feature_id[68].value > threshold=0.8600535988807678
node_547: feature_name=GO:0002718 feature_id[481].value > threshold=4.013465642929077
node_557: feature_name=GO:0045471 feature_id[354].value > threshold=1.8513423800468445
Class: negative genes

Rules_657 passed counts:1
node_0: feature_name=GO:0042113 feature_id[0].value <= threshold=13.408552169799805
node_1: feature_name=GO:0007568 feature_id[534].value <= threshold=5.0313897132873535
node_2: feature_name=GO:0002705 feature_id[541].value <= threshold=3.200145721435547
node_3: feature_name=GO:1901525 feature_id[576].value <= threshold=0.4466460347175598
node_4: feature_name=GO:0048539 feature_id[319].value <= threshold=3.0399646759033203
node_5: feature_name=GO:0001910 feature_id[385].value <= threshold=3.7437864542007446
node_6: feature_name=GO:0043200 feature_id[706].value <= threshold=9.307284355163574
node_7: feature_name=GO:0001773 feature_id[308].value <= threshold=4.016931533813477
node_8: feature_name=GO:0090116 feature_id[97].value <= threshold=7.99645471572876
node_9: feature_name=GO:0019814 feature_id[189].value <= threshold=4.525782108306885
node_10: feature_name=GO:1902583 feature_id[215].value <= threshold=17.60310649871826
node_11: feature_name=GO:0045429 feature_id[737].value > threshold=1.6133361458778381
node_477: feature_name=GO:0036037 feature_id[445].value <= threshold=1.7181594371795654
node_478: feature_name=GO:0006244 feature_id[503].value <= threshold=0.951388418674469
node_479: feature_name=GO:0032461 feature_id[608].value <= threshold=2.6103241443634033
node_480: feature_name=GO:0044710 feature_id[719].value > threshold=2.228096718681627e-06
node_494: feature_name=GO:0009314 feature_id[296].value <= threshold=34.93696117401123

node_495: feature_name=GO:0050778 feature_id[802].value <= threshold=39.29364013671875
node_496: feature_name=GO:1903038 feature_id[492].value <= threshold=5.849650859832764
node_497: feature_name=GO:0002699 feature_id[606].value <= threshold=5.5405943393707275
node_498: feature_name=hsa00983 feature_id[181].value <= threshold=25.851045608520508
node_499: feature_name=GO:0002309 feature_id[416].value <= threshold=1.6235689520835876
node_500: feature_name=GO:1901698 feature_id[792].value <= threshold=47.70275688171387
node_501: feature_name=GO:0010216 feature_id[282].value <= threshold=1.6849713921546936
node_502: feature_name=GO:0006266 feature_id[297].value <= threshold=2.7199249267578125
node_503: feature_name=GO:0005488 feature_id[187].value > threshold=3.0373169010999845e-06
node_507: feature_name=GO:0045429 feature_id[737].value > threshold=1.614579975605011
node_511: feature_name=GO:0034103 feature_id[318].value <= threshold=5.832815647125244
node_512: feature_name=GO:0010663 feature_id[561].value <= threshold=4.157005429267883
node_513: feature_name=GO:0030887 feature_id[274].value <= threshold=1.8964150547981262
node_514: feature_name=GO:0048294 feature_id[113].value <= threshold=1.866134524345398
node_515: feature_name=GO:0072539 feature_id[68].value > threshold=0.8600535988807678
node_547: feature_name=GO:0002718 feature_id[481].value <= threshold=4.013465642929077
node_548: feature_name=GO:0072539 feature_id[68].value > threshold=0.8829970061779022
node_550: feature_name=GO:0055064 feature_id[299].value > threshold=1.2614808082580566
Class: positive genes

Rules_658 passed counts:1
node_0: feature_name=GO:0042113 feature_id[0].value <= threshold=13.408552169799805
node_1: feature_name=GO:0007568 feature_id[534].value <= threshold=5.0313897132873535
node_2: feature_name=GO:0002705 feature_id[541].value <= threshold=3.200145721435547
node_3: feature_name=GO:1901525 feature_id[576].value <= threshold=0.4466460347175598
node_4: feature_name=GO:0048539 feature_id[319].value <= threshold=3.0399646759033203
node_5: feature_name=GO:0001910 feature_id[385].value <= threshold=3.7437864542007446
node_6: feature_name=GO:0043200 feature_id[706].value <= threshold=9.307284355163574
node_7: feature_name=GO:0001773 feature_id[308].value <= threshold=4.016931533813477
node_8: feature_name=GO:0090116 feature_id[97].value <= threshold=7.99645471572876
node_9: feature_name=GO:0019814 feature_id[189].value <= threshold=4.525782108306885
node_10: feature_name=GO:1902583 feature_id[215].value <= threshold=17.60310649871826
node_11: feature_name=GO:0045429 feature_id[737].value > threshold=1.6133361458778381
node_477: feature_name=GO:0036037 feature_id[445].value <= threshold=1.7181594371795654
node_478: feature_name=GO:0006244 feature_id[503].value <= threshold=0.951388418674469

node_479: feature_name=GO:0032461 feature_id[608].value <= threshold=2.6103241443634033
node_480: feature_name=GO:0044710 feature_id[719].value > threshold=2.228096718681627e-06
node_494: feature_name=GO:0009314 feature_id[296].value <= threshold=34.93696117401123
node_495: feature_name=GO:0050778 feature_id[802].value <= threshold=39.29364013671875
node_496: feature_name=GO:1903038 feature_id[492].value <= threshold=5.849650859832764
node_497: feature_name=GO:0002699 feature_id[606].value <= threshold=5.5405943393707275
node_498: feature_name=hsa00983 feature_id[181].value <= threshold=25.851045608520508
node_499: feature_name=GO:0002309 feature_id[416].value <= threshold=1.6235689520835876
node_500: feature_name=GO:1901698 feature_id[792].value <= threshold=47.70275688171387
node_501: feature_name=GO:0010216 feature_id[282].value <= threshold=1.6849713921546936
node_502: feature_name=GO:0006266 feature_id[297].value <= threshold=2.7199249267578125
node_503: feature_name=GO:0005488 feature_id[187].value > threshold=3.0373169010999845e-06
node_507: feature_name=GO:0045429 feature_id[737].value > threshold=1.614579975605011
node_511: feature_name=GO:0034103 feature_id[318].value <= threshold=5.832815647125244
node_512: feature_name=GO:0010663 feature_id[561].value <= threshold=4.157005429267883
node_513: feature_name=GO:0030887 feature_id[274].value <= threshold=1.8964150547981262
node_514: feature_name=GO:0048294 feature_id[113].value <= threshold=1.866134524345398
node_515: feature_name=GO:0072539 feature_id[68].value > threshold=0.8600535988807678
node_547: feature_name=GO:0002718 feature_id[481].value <= threshold=4.013465642929077
node_548: feature_name=GO:0072539 feature_id[68].value > threshold=0.8829970061779022
node_550: feature_name=GO:0055064 feature_id[299].value <= threshold=1.2614808082580566
node_551: feature_name=GO:0000307 feature_id[139].value > threshold=2.679197907447815
Class: positive genes

Rules_659 passed counts:1
node_0: feature_name=GO:0042113 feature_id[0].value <= threshold=13.408552169799805
node_1: feature_name=GO:0007568 feature_id[534].value <= threshold=5.0313897132873535
node_2: feature_name=GO:0002705 feature_id[541].value <= threshold=3.200145721435547
node_3: feature_name=GO:1901525 feature_id[576].value <= threshold=0.4466460347175598
node_4: feature_name=GO:0048539 feature_id[319].value <= threshold=3.0399646759033203
node_5: feature_name=GO:0001910 feature_id[385].value <= threshold=3.7437864542007446
node_6: feature_name=GO:0043200 feature_id[706].value <= threshold=9.307284355163574
node_7: feature_name=GO:0001773 feature_id[308].value <= threshold=4.016931533813477
node_8: feature_name=GO:0090116 feature_id[97].value <= threshold=7.99645471572876
node_9: feature_name=GO:0019814 feature_id[189].value <= threshold=4.525782108306885

node_10: feature_name=GO:1902583 feature_id[215].value <= threshold=17.60310649871826
node_11: feature_name=GO:0045429 feature_id[737].value > threshold=1.6133361458778381
node_477: feature_name=GO:0036037 feature_id[445].value <= threshold=1.7181594371795654
node_478: feature_name=GO:0006244 feature_id[503].value <= threshold=0.951388418674469
node_479: feature_name=GO:0032461 feature_id[608].value <= threshold=2.6103241443634033
node_480: feature_name=GO:0044710 feature_id[719].value > threshold=2.228096718681627e-06
node_494: feature_name=GO:0009314 feature_id[296].value <= threshold=34.93696117401123
node_495: feature_name=GO:0050778 feature_id[802].value <= threshold=39.29364013671875
node_496: feature_name=GO:1903038 feature_id[492].value <= threshold=5.849650859832764
node_497: feature_name=GO:0002699 feature_id[606].value <= threshold=5.5405943393707275
node_498: feature_name=hsa00983 feature_id[181].value <= threshold=25.851045608520508
node_499: feature_name=GO:0002309 feature_id[416].value <= threshold=1.6235689520835876
node_500: feature_name=GO:1901698 feature_id[792].value <= threshold=47.70275688171387
node_501: feature_name=GO:0010216 feature_id[282].value <= threshold=1.6849713921546936
node_502: feature_name=GO:0006266 feature_id[297].value <= threshold=2.7199249267578125
node_503: feature_name=GO:0005488 feature_id[187].value > threshold=3.0373169010999845e-06
node_507: feature_name=GO:0045429 feature_id[737].value > threshold=1.614579975605011
node_511: feature_name=GO:0034103 feature_id[318].value <= threshold=5.832815647125244
node_512: feature_name=GO:0010663 feature_id[561].value <= threshold=4.157005429267883
node_513: feature_name=GO:0030887 feature_id[274].value <= threshold=1.8964150547981262
node_514: feature_name=GO:0048294 feature_id[113].value <= threshold=1.866134524345398
node_515: feature_name=GO:0072539 feature_id[68].value > threshold=0.8600535988807678
node_547: feature_name=GO:0002718 feature_id[481].value <= threshold=4.013465642929077
node_548: feature_name=GO:0072539 feature_id[68].value > threshold=0.8829970061779022
node_550: feature_name=GO:0055064 feature_id[299].value <= threshold=1.2614808082580566
node_551: feature_name=GO:0000307 feature_id[139].value <= threshold=2.679197907447815
node_552: feature_name=GO:0046631 feature_id[86].value <= threshold=0.2878068685531616
Class: positive genes

Rules_660 passed counts:1
node_0: feature_name=GO:0042113 feature_id[0].value <= threshold=13.408552169799805
node_1: feature_name=GO:0007568 feature_id[534].value <= threshold=5.0313897132873535
node_2: feature_name=GO:0002705 feature_id[541].value <= threshold=3.200145721435547
node_3: feature_name=GO:1901525 feature_id[576].value <= threshold=0.4466460347175598
node_4: feature_name=GO:0048539 feature_id[319].value <= threshold=3.0399646759033203

node_5: feature_name=GO:0001910 feature_id[385].value <= threshold=3.7437864542007446
node_6: feature_name=GO:0043200 feature_id[706].value <= threshold=9.307284355163574
node_7: feature_name=GO:0001773 feature_id[308].value <= threshold=4.016931533813477
node_8: feature_name=GO:0090116 feature_id[97].value <= threshold=7.99645471572876
node_9: feature_name=GO:0019814 feature_id[189].value <= threshold=4.525782108306885
node_10: feature_name=GO:1902583 feature_id[215].value <= threshold=17.60310649871826
node_11: feature_name=GO:0045429 feature_id[737].value > threshold=1.6133361458778381
node_477: feature_name=GO:0036037 feature_id[445].value <= threshold=1.7181594371795654
node_478: feature_name=GO:0006244 feature_id[503].value <= threshold=0.951388418674469
node_479: feature_name=GO:0032461 feature_id[608].value <= threshold=2.6103241443634033
node_480: feature_name=GO:0044710 feature_id[719].value > threshold=2.228096718681627e-06
node_494: feature_name=GO:0009314 feature_id[296].value <= threshold=34.93696117401123
node_495: feature_name=GO:0050778 feature_id[802].value <= threshold=39.29364013671875
node_496: feature_name=GO:1903038 feature_id[492].value <= threshold=5.849650859832764
node_497: feature_name=GO:0002699 feature_id[606].value <= threshold=5.5405943393707275
node_498: feature_name=hsa00983 feature_id[181].value <= threshold=25.851045608520508
node_499: feature_name=GO:0002309 feature_id[416].value <= threshold=1.6235689520835876
node_500: feature_name=GO:1901698 feature_id[792].value <= threshold=47.70275688171387
node_501: feature_name=GO:0010216 feature_id[282].value <= threshold=1.6849713921546936
node_502: feature_name=GO:0006266 feature_id[297].value <= threshold=2.7199249267578125
node_503: feature_name=GO:0005488 feature_id[187].value > threshold=3.0373169010999845e-06
node_507: feature_name=GO:0045429 feature_id[737].value > threshold=1.614579975605011
node_511: feature_name=GO:0034103 feature_id[318].value <= threshold=5.832815647125244
node_512: feature_name=GO:0010663 feature_id[561].value <= threshold=4.157005429267883
node_513: feature_name=GO:0030887 feature_id[274].value <= threshold=1.8964150547981262
node_514: feature_name=GO:0048294 feature_id[113].value <= threshold=1.866134524345398
node_515: feature_name=GO:0072539 feature_id[68].value > threshold=0.8600535988807678
node_547: feature_name=GO:0002718 feature_id[481].value <= threshold=4.013465642929077
node_548: feature_name=GO:0072539 feature_id[68].value <= threshold=0.8829970061779022
Class: positive genes

Rules_661 passed counts:1
node_0: feature_name=GO:0042113 feature_id[0].value <= threshold=13.408552169799805
node_1: feature_name=GO:0007568 feature_id[534].value <= threshold=5.0313897132873535
node_2: feature_name=GO:0002705 feature_id[541].value <= threshold=3.200145721435547

node_3: feature_name=GO:1901525	feature_id[576].value <= threshold=0.4466460347175598
node_4: feature_name=GO:0048539	feature_id[319].value <= threshold=3.0399646759033203
node_5: feature_name=GO:0001910	feature_id[385].value <= threshold=3.7437864542007446
node_6: feature_name=GO:0043200	feature_id[706].value <= threshold=9.307284355163574
node_7: feature_name=GO:0001773	feature_id[308].value <= threshold=4.016931533813477
node_8: feature_name=GO:0090116	feature_id[97].value <= threshold=7.99645471572876
node_9: feature_name=GO:0019814	feature_id[189].value <= threshold=4.525782108306885
node_10: feature_name=GO:1902583	feature_id[215].value <= threshold=17.60310649871826
node_11: feature_name=GO:0045429	feature_id[737].value > threshold=1.6133361458778381
node_477: feature_name=GO:0036037	feature_id[445].value <= threshold=1.7181594371795654
node_478: feature_name=GO:0006244	feature_id[503].value <= threshold=0.951388418674469
node_479: feature_name=GO:0032461	feature_id[608].value <= threshold=2.6103241443634033
node_480: feature_name=GO:0044710	feature_id[719].value > threshold=2.228096718681627e-06
node_494: feature_name=GO:0009314	feature_id[296].value <= threshold=34.93696117401123
node_495: feature_name=GO:0050778	feature_id[802].value <= threshold=39.29364013671875
node_496: feature_name=GO:1903038	feature_id[492].value <= threshold=5.849650859832764
node_497: feature_name=GO:0002699	feature_id[606].value <= threshold=5.5405943393707275
node_498: feature_name=hsa00983	feature_id[181].value <= threshold=25.851045608520508
node_499: feature_name=GO:0002309	feature_id[416].value <= threshold=1.6235689520835876
node_500: feature_name=GO:1901698	feature_id[792].value <= threshold=47.70275688171387
node_501: feature_name=GO:0010216	feature_id[282].value <= threshold=1.6849713921546936
node_502: feature_name=GO:0006266	feature_id[297].value <= threshold=2.7199249267578125
node_503: feature_name=GO:0005488	feature_id[187].value > threshold=3.0373169010999845e-06
node_507: feature_name=GO:0045429	feature_id[737].value > threshold=1.614579975605011
node_511: feature_name=GO:0034103	feature_id[318].value <= threshold=5.832815647125244
node_512: feature_name=GO:0010663	feature_id[561].value <= threshold=4.157005429267883
node_513: feature_name=GO:0030887	feature_id[274].value <= threshold=1.8964150547981262
node_514: feature_name=GO:0048294	feature_id[113].value <= threshold=1.866134524345398
node_515: feature_name=GO:0072539	feature_id[68].value <= threshold=0.8600535988807678
node_516: feature_name=GO:0045840	feature_id[697].value <= threshold=4.963376045227051
node_517: feature_name=GO:0019222	feature_id[129].value > threshold=4.0122095015249215e-06
node_523: feature_name=GO:0008150	feature_id[528].value > threshold=40.503862380981445
node_541: feature_name=GO:0042094	feature_id[677].value > threshold=0.17812439054250717

Class: positive genes

Rules_662	passed counts:1
node_0: feature_name=GO:0042113	feature_id[0].value <= threshold=13.408552169799805
node_1: feature_name=GO:0007568	feature_id[534].value <= threshold=5.0313897132873535
node_2: feature_name=GO:0002705	feature_id[541].value <= threshold=3.200145721435547
node_3: feature_name=GO:1901525	feature_id[576].value <= threshold=0.4466460347175598
node_4: feature_name=GO:0048539	feature_id[319].value <= threshold=3.0399646759033203
node_5: feature_name=GO:0001910	feature_id[385].value <= threshold=3.7437864542007446
node_6: feature_name=GO:0043200	feature_id[706].value <= threshold=9.307284355163574
node_7: feature_name=GO:0001773	feature_id[308].value <= threshold=4.016931533813477
node_8: feature_name=GO:0090116	feature_id[97].value <= threshold=7.99645471572876
node_9: feature_name=GO:0019814	feature_id[189].value <= threshold=4.525782108306885
node_10: feature_name=GO:1902583	feature_id[215].value <= threshold=17.60310649871826
node_11: feature_name=GO:0045429	feature_id[737].value > threshold=1.6133361458778381
node_477: feature_name=GO:0036037	feature_id[445].value <= threshold=1.7181594371795654
node_478: feature_name=GO:0006244	feature_id[503].value <= threshold=0.951388418674469
node_479: feature_name=GO:0032461	feature_id[608].value <= threshold=2.6103241443634033
node_480: feature_name=GO:0044710	feature_id[719].value > threshold=2.228096718681627e-06
node_494: feature_name=GO:0009314	feature_id[296].value <= threshold=34.93696117401123
node_495: feature_name=GO:0050778	feature_id[802].value <= threshold=39.29364013671875
node_496: feature_name=GO:1903038	feature_id[492].value <= threshold=5.849650859832764
node_497: feature_name=GO:0002699	feature_id[606].value <= threshold=5.5405943393707275
node_498: feature_name=hsa00983	feature_id[181].value <= threshold=25.851045608520508
node_499: feature_name=GO:0002309	feature_id[416].value <= threshold=1.6235689520835876
node_500: feature_name=GO:1901698	feature_id[792].value <= threshold=47.70275688171387
node_501: feature_name=GO:0010216	feature_id[282].value <= threshold=1.6849713921546936
node_502: feature_name=GO:0006266	feature_id[297].value <= threshold=2.7199249267578125
node_503: feature_name=GO:0005488	feature_id[187].value > threshold=3.0373169010999845e-06
node_507: feature_name=GO:0045429	feature_id[737].value > threshold=1.614579975605011
node_511: feature_name=GO:0034103	feature_id[318].value <= threshold=5.832815647125244
node_512: feature_name=GO:0010663	feature_id[561].value <= threshold=4.157005429267883
node_513: feature_name=GO:0030887	feature_id[274].value <= threshold=1.8964150547981262
node_514: feature_name=GO:0048294	feature_id[113].value <= threshold=1.866134524345398
node_515: feature_name=GO:0072539	feature_id[68].value <= threshold=0.8600535988807678
node_516: feature_name=GO:0045840	feature_id[697].value <= threshold=4.963376045227051
node_517: feature_name=GO:0019222	feature_id[129].value > threshold=4.0122095015249215e-06

node_523: feature_name=GO:0008150 feature_id[528].value <= threshold=40.503862380981445
node_524: feature_name=GO:0006359 feature_id[87].value > threshold=2.954534411430359
node_538: feature_name=GO:0009897 feature_id[38].value <= threshold=0.00021361259859986603
Class: positive genes

Rules_663 passed counts:1
node_0: feature_name=GO:0042113 feature_id[0].value <= threshold=13.408552169799805
node_1: feature_name=GO:0007568 feature_id[534].value <= threshold=5.0313897132873535
node_2: feature_name=GO:0002705 feature_id[541].value <= threshold=3.200145721435547
node_3: feature_name=GO:1901525 feature_id[576].value <= threshold=0.4466460347175598
node_4: feature_name=GO:0048539 feature_id[319].value <= threshold=3.0399646759033203
node_5: feature_name=GO:0001910 feature_id[385].value <= threshold=3.7437864542007446
node_6: feature_name=GO:0043200 feature_id[706].value <= threshold=9.307284355163574
node_7: feature_name=GO:0001773 feature_id[308].value <= threshold=4.016931533813477
node_8: feature_name=GO:0090116 feature_id[97].value <= threshold=7.99645471572876
node_9: feature_name=GO:0019814 feature_id[189].value <= threshold=4.525782108306885
node_10: feature_name=GO:1902583 feature_id[215].value <= threshold=17.60310649871826
node_11: feature_name=GO:0045429 feature_id[737].value > threshold=1.6133361458778381
node_477: feature_name=GO:0036037 feature_id[445].value <= threshold=1.7181594371795654
node_478: feature_name=GO:0006244 feature_id[503].value <= threshold=0.951388418674469
node_479: feature_name=GO:0032461 feature_id[608].value <= threshold=2.6103241443634033
node_480: feature_name=GO:0044710 feature_id[719].value > threshold=2.228096718681627e-06
node_494: feature_name=GO:0009314 feature_id[296].value <= threshold=34.93696117401123
node_495: feature_name=GO:0050778 feature_id[802].value <= threshold=39.29364013671875
node_496: feature_name=GO:1903038 feature_id[492].value <= threshold=5.849650859832764
node_497: feature_name=GO:0002699 feature_id[606].value <= threshold=5.5405943393707275
node_498: feature_name=hsa00983 feature_id[181].value <= threshold=25.851045608520508
node_499: feature_name=GO:0002309 feature_id[416].value <= threshold=1.6235689520835876
node_500: feature_name=GO:1901698 feature_id[792].value <= threshold=47.70275688171387
node_501: feature_name=GO:0010216 feature_id[282].value <= threshold=1.6849713921546936
node_502: feature_name=GO:0006266 feature_id[297].value <= threshold=2.7199249267578125
node_503: feature_name=GO:0005488 feature_id[187].value > threshold=3.0373169010999845e-06
node_507: feature_name=GO:0045429 feature_id[737].value > threshold=1.614579975605011
node_511: feature_name=GO:0034103 feature_id[318].value <= threshold=5.832815647125244
node_512: feature_name=GO:0010663 feature_id[561].value <= threshold=4.157005429267883

node_513: feature_name=GO:0030887 feature_id[274].value <= threshold=1.8964150547981262
node_514: feature_name=GO:0048294 feature_id[113].value <= threshold=1.866134524345398
node_515: feature_name=GO:0072539 feature_id[68].value <= threshold=0.8600535988807678
node_516: feature_name=GO:0045840 feature_id[697].value <= threshold=4.963376045227051
node_517: feature_name=GO:0019222 feature_id[129].value > threshold=4.0122095015249215e-06
node_523: feature_name=GO:0008150 feature_id[528].value <= threshold=40.503862380981445
node_524: feature_name=GO:0006359 feature_id[87].value <= threshold=2.954534411430359
node_525: feature_name=GO:0010225 feature_id[558].value > threshold=1.8247058987617493
node_535: feature_name=GO:0046427 feature_id[766].value <= threshold=0.07467848435044289
Class: positive genes

Rules_664 passed counts:1
node_0: feature_name=GO:0042113 feature_id[0].value <= threshold=13.408552169799805
node_1: feature_name=GO:0007568 feature_id[534].value <= threshold=5.0313897132873535
node_2: feature_name=GO:0002705 feature_id[541].value <= threshold=3.200145721435547
node_3: feature_name=GO:1901525 feature_id[576].value <= threshold=0.4466460347175598
node_4: feature_name=GO:0048539 feature_id[319].value <= threshold=3.0399646759033203
node_5: feature_name=GO:0001910 feature_id[385].value <= threshold=3.7437864542007446
node_6: feature_name=GO:0043200 feature_id[706].value <= threshold=9.307284355163574
node_7: feature_name=GO:0001773 feature_id[308].value <= threshold=4.016931533813477
node_8: feature_name=GO:0090116 feature_id[97].value <= threshold=7.99645471572876
node_9: feature_name=GO:0019814 feature_id[189].value <= threshold=4.525782108306885
node_10: feature_name=GO:1902583 feature_id[215].value <= threshold=17.60310649871826
node_11: feature_name=GO:0045429 feature_id[737].value > threshold=1.6133361458778381
node_477: feature_name=GO:0036037 feature_id[445].value <= threshold=1.7181594371795654
node_478: feature_name=GO:0006244 feature_id[503].value <= threshold=0.951388418674469
node_479: feature_name=GO:0032461 feature_id[608].value <= threshold=2.6103241443634033
node_480: feature_name=GO:0044710 feature_id[719].value > threshold=2.228096718681627e-06
node_494: feature_name=GO:0009314 feature_id[296].value <= threshold=34.93696117401123
node_495: feature_name=GO:0050778 feature_id[802].value <= threshold=39.29364013671875
node_496: feature_name=GO:1903038 feature_id[492].value <= threshold=5.849650859832764
node_497: feature_name=GO:0002699 feature_id[606].value <= threshold=5.5405943393707275
node_498: feature_name=hsa00983 feature_id[181].value <= threshold=25.851045608520508
node_499: feature_name=GO:0002309 feature_id[416].value <= threshold=1.6235689520835876
node_500: feature_name=GO:1901698 feature_id[792].value <= threshold=47.70275688171387

node_501: feature_name=GO:0010216 feature_id[282].value <= threshold=1.6849713921546936
node_502: feature_name=GO:0006266 feature_id[297].value <= threshold=2.7199249267578125
node_503: feature_name=GO:0005488 feature_id[187].value > threshold=3.0373169010999845e-06
node_507: feature_name=GO:0045429 feature_id[737].value > threshold=1.614579975605011
node_511: feature_name=GO:0034103 feature_id[318].value <= threshold=5.832815647125244
node_512: feature_name=GO:0010663 feature_id[561].value <= threshold=4.157005429267883
node_513: feature_name=GO:0030887 feature_id[274].value <= threshold=1.8964150547981262
node_514: feature_name=GO:0048294 feature_id[113].value <= threshold=1.866134524345398
node_515: feature_name=GO:0072539 feature_id[68].value <= threshold=0.8600535988807678
node_516: feature_name=GO:0045840 feature_id[697].value <= threshold=4.963376045227051
node_517: feature_name=GO:0019222 feature_id[129].value > threshold=4.0122095015249215e-06
node_523: feature_name=GO:0008150 feature_id[528].value <= threshold=40.503862380981445
node_524: feature_name=GO:0006359 feature_id[87].value <= threshold=2.954534411430359
node_525: feature_name=GO:0010225 feature_id[558].value <= threshold=1.8247058987617493
node_526: feature_name=GO:0032461 feature_id[608].value > threshold=1.6246166825294495
node_532: feature_name=GO:0050672 feature_id[800].value <= threshold=0.301830917596817
Class: positive genes

Rules_665 passed counts:1
node_0: feature_name=GO:0042113 feature_id[0].value <= threshold=13.408552169799805
node_1: feature_name=GO:0007568 feature_id[534].value <= threshold=5.0313897132873535
node_2: feature_name=GO:0002705 feature_id[541].value <= threshold=3.200145721435547
node_3: feature_name=GO:1901525 feature_id[576].value <= threshold=0.4466460347175598
node_4: feature_name=GO:0048539 feature_id[319].value <= threshold=3.0399646759033203
node_5: feature_name=GO:0001910 feature_id[385].value <= threshold=3.7437864542007446
node_6: feature_name=GO:0043200 feature_id[706].value <= threshold=9.307284355163574
node_7: feature_name=GO:0001773 feature_id[308].value <= threshold=4.016931533813477
node_8: feature_name=GO:0090116 feature_id[97].value <= threshold=7.99645471572876
node_9: feature_name=GO:0019814 feature_id[189].value <= threshold=4.525782108306885
node_10: feature_name=GO:1902583 feature_id[215].value <= threshold=17.60310649871826
node_11: feature_name=GO:0045429 feature_id[737].value > threshold=1.6133361458778381
node_477: feature_name=GO:0036037 feature_id[445].value <= threshold=1.7181594371795654
node_478: feature_name=GO:0006244 feature_id[503].value <= threshold=0.951388418674469
node_479: feature_name=GO:0032461 feature_id[608].value <= threshold=2.6103241443634033
node_480: feature_name=GO:0044710 feature_id[719].value > threshold=2.228096718681627e-06

node_494: feature_name=GO:0009314	feature_id[296].value <= threshold=34.93696117401123
node_495: feature_name=GO:0050778	feature_id[802].value <= threshold=39.29364013671875
node_496: feature_name=GO:1903038	feature_id[492].value <= threshold=5.849650859832764
node_497: feature_name=GO:0002699	feature_id[606].value <= threshold=5.5405943393707275
node_498: feature_name=hsa00983	feature_id[181].value <= threshold=25.851045608520508
node_499: feature_name=GO:0002309	feature_id[416].value <= threshold=1.6235689520835876
node_500: feature_name=GO:1901698	feature_id[792].value <= threshold=47.70275688171387
node_501: feature_name=GO:0010216	feature_id[282].value <= threshold=1.6849713921546936
node_502: feature_name=GO:0006266	feature_id[297].value <= threshold=2.7199249267578125
node_503: feature_name=GO:0005488	feature_id[187].value > threshold=3.0373169010999845e-06
node_507: feature_name=GO:0045429	feature_id[737].value > threshold=1.614579975605011
node_511: feature_name=GO:0034103	feature_id[318].value <= threshold=5.832815647125244
node_512: feature_name=GO:0010663	feature_id[561].value <= threshold=4.157005429267883
node_513: feature_name=GO:0030887	feature_id[274].value <= threshold=1.8964150547981262
node_514: feature_name=GO:0048294	feature_id[113].value <= threshold=1.866134524345398
node_515: feature_name=GO:0072539	feature_id[68].value <= threshold=0.8600535988807678
node_516: feature_name=GO:0045840	feature_id[697].value <= threshold=4.963376045227051
node_517: feature_name=GO:0019222	feature_id[129].value > threshold=4.0122095015249215e-06
node_523: feature_name=GO:0008150	feature_id[528].value <= threshold=40.503862380981445
node_524: feature_name=GO:0006359	feature_id[87].value <= threshold=2.954534411430359
node_525: feature_name=GO:0010225	feature_id[558].value <= threshold=1.8247058987617493
node_526: feature_name=GO:0032461	feature_id[608].value <= threshold=1.6246166825294495
node_527: feature_name=GO:0007406	feature_id[537].value > threshold=2.375624656677246
node_529: feature_name=GO:0042368	feature_id[676].value > threshold=0.4141124486923218

Class: positive genes

Rules_666	passed counts:1
node_0: feature_name=GO:0042113	feature_id[0].value <= threshold=13.408552169799805
node_1: feature_name=GO:0007568	feature_id[534].value <= threshold=5.0313897132873535
node_2: feature_name=GO:0002705	feature_id[541].value <= threshold=3.200145721435547
node_3: feature_name=GO:1901525	feature_id[576].value <= threshold=0.4466460347175598
node_4: feature_name=GO:0048539	feature_id[319].value <= threshold=3.0399646759033203
node_5: feature_name=GO:0001910	feature_id[385].value <= threshold=3.7437864542007446
node_6: feature_name=GO:0043200	feature_id[706].value <= threshold=9.307284355163574
node_7: feature_name=GO:0001773	feature_id[308].value <= threshold=4.016931533813477

node_8: feature_name=GO:0090116 feature_id[97].value <= threshold=7.99645471572876
node_9: feature_name=GO:0019814 feature_id[189].value <= threshold=4.525782108306885
node_10: feature_name=GO:1902583 feature_id[215].value <= threshold=17.60310649871826
node_11: feature_name=GO:0045429 feature_id[737].value > threshold=1.6133361458778381
node_477: feature_name=GO:0036037 feature_id[445].value <= threshold=1.7181594371795654
node_478: feature_name=GO:0006244 feature_id[503].value <= threshold=0.951388418674469
node_479: feature_name=GO:0032461 feature_id[608].value <= threshold=2.6103241443634033
node_480: feature_name=GO:0044710 feature_id[719].value > threshold=2.228096718681627e-06
node_494: feature_name=GO:0009314 feature_id[296].value <= threshold=34.93696117401123
node_495: feature_name=GO:0050778 feature_id[802].value <= threshold=39.29364013671875
node_496: feature_name=GO:1903038 feature_id[492].value <= threshold=5.849650859832764
node_497: feature_name=GO:0002699 feature_id[606].value <= threshold=5.5405943393707275
node_498: feature_name=hsa00983 feature_id[181].value <= threshold=25.851045608520508
node_499: feature_name=GO:0002309 feature_id[416].value <= threshold=1.6235689520835876
node_500: feature_name=GO:1901698 feature_id[792].value <= threshold=47.70275688171387
node_501: feature_name=GO:0010216 feature_id[282].value <= threshold=1.6849713921546936
node_502: feature_name=GO:0006266 feature_id[297].value <= threshold=2.7199249267578125
node_503: feature_name=GO:0005488 feature_id[187].value > threshold=3.0373169010999845e-06
node_507: feature_name=GO:0045429 feature_id[737].value > threshold=1.614579975605011
node_511: feature_name=GO:0034103 feature_id[318].value <= threshold=5.832815647125244
node_512: feature_name=GO:0010663 feature_id[561].value <= threshold=4.157005429267883
node_513: feature_name=GO:0030887 feature_id[274].value <= threshold=1.8964150547981262
node_514: feature_name=GO:0048294 feature_id[113].value <= threshold=1.866134524345398
node_515: feature_name=GO:0072539 feature_id[68].value <= threshold=0.8600535988807678
node_516: feature_name=GO:0045840 feature_id[697].value <= threshold=4.963376045227051
node_517: feature_name=GO:0019222 feature_id[129].value <= threshold=4.0122095015249215e-06
node_518: feature_name=GO:0007568 feature_id[534].value > threshold=3.2433676719665527
node_520: feature_name=GO:0030888 feature_id[120].value <= threshold=0.121795654296875
Class: negative genes

Rules_667 passed counts:1
node_0: feature_name=GO:0042113 feature_id[0].value <= threshold=13.408552169799805
node_1: feature_name=GO:0007568 feature_id[534].value <= threshold=5.0313897132873535
node_2: feature_name=GO:0002705 feature_id[541].value <= threshold=3.200145721435547
node_3: feature_name=GO:1901525 feature_id[576].value <= threshold=0.4466460347175598

node_4: feature_name=GO:0048539 feature_id[319].value <= threshold=3.0399646759033203
node_5: feature_name=GO:0001910 feature_id[385].value <= threshold=3.7437864542007446
node_6: feature_name=GO:0043200 feature_id[706].value <= threshold=9.307284355163574
node_7: feature_name=GO:0001773 feature_id[308].value <= threshold=4.016931533813477
node_8: feature_name=GO:0090116 feature_id[97].value <= threshold=7.99645471572876
node_9: feature_name=GO:0019814 feature_id[189].value <= threshold=4.525782108306885
node_10: feature_name=GO:1902583 feature_id[215].value <= threshold=17.60310649871826
node_11: feature_name=GO:0045429 feature_id[737].value > threshold=1.6133361458778381
node_477: feature_name=GO:0036037 feature_id[445].value <= threshold=1.7181594371795654
node_478: feature_name=GO:0006244 feature_id[503].value <= threshold=0.951388418674469
node_479: feature_name=GO:0032461 feature_id[608].value <= threshold=2.6103241443634033
node_480: feature_name=GO:0044710 feature_id[719].value > threshold=2.228096718681627e-06
node_494: feature_name=GO:0009314 feature_id[296].value <= threshold=34.93696117401123
node_495: feature_name=GO:0050778 feature_id[802].value <= threshold=39.29364013671875
node_496: feature_name=GO:1903038 feature_id[492].value <= threshold=5.849650859832764
node_497: feature_name=GO:0002699 feature_id[606].value <= threshold=5.5405943393707275
node_498: feature_name=hsa00983 feature_id[181].value <= threshold=25.851045608520508
node_499: feature_name=GO:0002309 feature_id[416].value <= threshold=1.6235689520835876
node_500: feature_name=GO:1901698 feature_id[792].value <= threshold=47.70275688171387
node_501: feature_name=GO:0010216 feature_id[282].value <= threshold=1.6849713921546936
node_502: feature_name=GO:0006266 feature_id[297].value <= threshold=2.7199249267578125
node_503: feature_name=GO:0005488 feature_id[187].value > threshold=3.0373169010999845e-06
node_507: feature_name=GO:0045429 feature_id[737].value <= threshold=1.614579975605011
node_508: feature_name=GO:0003684 feature_id[653].value > threshold=0.9636852741241455
Class: positive genes

Rules_668 passed counts:1
node_0: feature_name=GO:0042113 feature_id[0].value <= threshold=13.408552169799805
node_1: feature_name=GO:0007568 feature_id[534].value <= threshold=5.0313897132873535
node_2: feature_name=GO:0002705 feature_id[541].value <= threshold=3.200145721435547
node_3: feature_name=GO:1901525 feature_id[576].value <= threshold=0.4466460347175598
node_4: feature_name=GO:0048539 feature_id[319].value <= threshold=3.0399646759033203
node_5: feature_name=GO:0001910 feature_id[385].value <= threshold=3.7437864542007446
node_6: feature_name=GO:0043200 feature_id[706].value <= threshold=9.307284355163574
node_7: feature_name=GO:0001773 feature_id[308].value <= threshold=4.016931533813477

node_8: feature_name=GO:0090116 feature_id[97].value <= threshold=7.99645471572876
node_9: feature_name=GO:0019814 feature_id[189].value <= threshold=4.525782108306885
node_10: feature_name=GO:1902583 feature_id[215].value <= threshold=17.60310649871826
node_11: feature_name=GO:0045429 feature_id[737].value > threshold=1.6133361458778381
node_477: feature_name=GO:0036037 feature_id[445].value <= threshold=1.7181594371795654
node_478: feature_name=GO:0006244 feature_id[503].value <= threshold=0.951388418674469
node_479: feature_name=GO:0032461 feature_id[608].value <= threshold=2.6103241443634033
node_480: feature_name=GO:0044710 feature_id[719].value > threshold=2.228096718681627e-06
node_494: feature_name=GO:0009314 feature_id[296].value <= threshold=34.93696117401123
node_495: feature_name=GO:0050778 feature_id[802].value <= threshold=39.29364013671875
node_496: feature_name=GO:1903038 feature_id[492].value <= threshold=5.849650859832764
node_497: feature_name=GO:0002699 feature_id[606].value <= threshold=5.5405943393707275
node_498: feature_name=hsa00983 feature_id[181].value <= threshold=25.851045608520508
node_499: feature_name=GO:0002309 feature_id[416].value <= threshold=1.6235689520835876
node_500: feature_name=GO:1901698 feature_id[792].value <= threshold=47.70275688171387
node_501: feature_name=GO:0010216 feature_id[282].value <= threshold=1.6849713921546936
node_502: feature_name=GO:0006266 feature_id[297].value <= threshold=2.7199249267578125
node_503: feature_name=GO:0005488 feature_id[187].value > threshold=3.0373169010999845e-06
node_507: feature_name=GO:0045429 feature_id[737].value <= threshold=1.614579975605011
node_508: feature_name=GO:0003684 feature_id[653].value <= threshold=0.9636852741241455
Class: negative genes

Rules_669 passed counts:1
node_0: feature_name=GO:0042113 feature_id[0].value <= threshold=13.408552169799805
node_1: feature_name=GO:0007568 feature_id[534].value <= threshold=5.0313897132873535
node_2: feature_name=GO:0002705 feature_id[541].value <= threshold=3.200145721435547
node_3: feature_name=GO:1901525 feature_id[576].value <= threshold=0.4466460347175598
node_4: feature_name=GO:0048539 feature_id[319].value <= threshold=3.0399646759033203
node_5: feature_name=GO:0001910 feature_id[385].value <= threshold=3.7437864542007446
node_6: feature_name=GO:0043200 feature_id[706].value <= threshold=9.307284355163574
node_7: feature_name=GO:0001773 feature_id[308].value <= threshold=4.016931533813477
node_8: feature_name=GO:0090116 feature_id[97].value <= threshold=7.99645471572876
node_9: feature_name=GO:0019814 feature_id[189].value <= threshold=4.525782108306885
node_10: feature_name=GO:1902583 feature_id[215].value <= threshold=17.60310649871826
node_11: feature_name=GO:0045429 feature_id[737].value > threshold=1.6133361458778381

node_477: feature_name=GO:0036037 feature_id[445].value <= threshold=1.7181594371795654
node_478: feature_name=GO:0006244 feature_id[503].value <= threshold=0.951388418674469
node_479: feature_name=GO:0032461 feature_id[608].value <= threshold=2.6103241443634033
node_480: feature_name=GO:0044710 feature_id[719].value <= threshold=2.228096718681627e-06
node_481: feature_name=GO:1904035 feature_id[654].value > threshold=0.05152595415711403
node_487: feature_name=GO:0046633 feature_id[115].value > threshold=0.7000146508216858
Class: positive genes

Rules_670 passed counts:1
node_0: feature_name=GO:0042113 feature_id[0].value <= threshold=13.408552169799805
node_1: feature_name=GO:0007568 feature_id[534].value <= threshold=5.0313897132873535
node_2: feature_name=GO:0002705 feature_id[541].value <= threshold=3.200145721435547
node_3: feature_name=GO:1901525 feature_id[576].value <= threshold=0.4466460347175598
node_4: feature_name=GO:0048539 feature_id[319].value <= threshold=3.0399646759033203
node_5: feature_name=GO:0001910 feature_id[385].value <= threshold=3.7437864542007446
node_6: feature_name=GO:0043200 feature_id[706].value <= threshold=9.307284355163574
node_7: feature_name=GO:0001773 feature_id[308].value <= threshold=4.016931533813477
node_8: feature_name=GO:0090116 feature_id[97].value <= threshold=7.99645471572876
node_9: feature_name=GO:0019814 feature_id[189].value <= threshold=4.525782108306885
node_10: feature_name=GO:1902583 feature_id[215].value <= threshold=17.60310649871826
node_11: feature_name=GO:0045429 feature_id[737].value > threshold=1.6133361458778381
node_477: feature_name=GO:0036037 feature_id[445].value <= threshold=1.7181594371795654
node_478: feature_name=GO:0006244 feature_id[503].value <= threshold=0.951388418674469
node_479: feature_name=GO:0032461 feature_id[608].value <= threshold=2.6103241443634033
node_480: feature_name=GO:0044710 feature_id[719].value <= threshold=2.228096718681627e-06
node_481: feature_name=GO:1904035 feature_id[654].value > threshold=0.05152595415711403
node_487: feature_name=GO:0046633 feature_id[115].value <= threshold=0.7000146508216858
node_488: feature_name=GO:0060576 feature_id[204].value > threshold=0.8788235783576965
Class: positive genes

Rules_671 passed counts:1
node_0: feature_name=GO:0042113 feature_id[0].value <= threshold=13.408552169799805
node_1: feature_name=GO:0007568 feature_id[534].value <= threshold=5.0313897132873535
node_2: feature_name=GO:0002705 feature_id[541].value <= threshold=3.200145721435547
node_3: feature_name=GO:1901525 feature_id[576].value <= threshold=0.4466460347175598

node_4: feature_name=GO:0048539 feature_id[319].value <= threshold=3.0399646759033203
node_5: feature_name=GO:0001910 feature_id[385].value <= threshold=3.7437864542007446
node_6: feature_name=GO:0043200 feature_id[706].value <= threshold=9.307284355163574
node_7: feature_name=GO:0001773 feature_id[308].value <= threshold=4.016931533813477
node_8: feature_name=GO:0090116 feature_id[97].value <= threshold=7.99645471572876
node_9: feature_name=GO:0019814 feature_id[189].value <= threshold=4.525782108306885
node_10: feature_name=GO:1902583 feature_id[215].value <= threshold=17.60310649871826
node_11: feature_name=GO:0045429 feature_id[737].value > threshold=1.6133361458778381
node_477: feature_name=GO:0036037 feature_id[445].value <= threshold=1.7181594371795654
node_478: feature_name=GO:0006244 feature_id[503].value <= threshold=0.951388418674469
node_479: feature_name=GO:0032461 feature_id[608].value <= threshold=2.6103241443634033
node_480: feature_name=GO:0044710 feature_id[719].value <= threshold=2.228096718681627e-06
node_481: feature_name=GO:1904035 feature_id[654].value > threshold=0.05152595415711403
node_487: feature_name=GO:0046633 feature_id[115].value <= threshold=0.7000146508216858
node_488: feature_name=GO:0060576 feature_id[204].value <= threshold=0.8788235783576965
node_489: feature_name=GO:0002694 feature_id[469].value > threshold=3.035288095474243
Class: positive genes

Rules_672 passed counts:1
node_0: feature_name=GO:0042113 feature_id[0].value <= threshold=13.408552169799805
node_1: feature_name=GO:0007568 feature_id[534].value <= threshold=5.0313897132873535
node_2: feature_name=GO:0002705 feature_id[541].value <= threshold=3.200145721435547
node_3: feature_name=GO:1901525 feature_id[576].value <= threshold=0.4466460347175598
node_4: feature_name=GO:0048539 feature_id[319].value <= threshold=3.0399646759033203
node_5: feature_name=GO:0001910 feature_id[385].value <= threshold=3.7437864542007446
node_6: feature_name=GO:0043200 feature_id[706].value <= threshold=9.307284355163574
node_7: feature_name=GO:0001773 feature_id[308].value <= threshold=4.016931533813477
node_8: feature_name=GO:0090116 feature_id[97].value <= threshold=7.99645471572876
node_9: feature_name=GO:0019814 feature_id[189].value <= threshold=4.525782108306885
node_10: feature_name=GO:1902583 feature_id[215].value <= threshold=17.60310649871826
node_11: feature_name=GO:0045429 feature_id[737].value > threshold=1.6133361458778381
node_477: feature_name=GO:0036037 feature_id[445].value <= threshold=1.7181594371795654
node_478: feature_name=GO:0006244 feature_id[503].value <= threshold=0.951388418674469
node_479: feature_name=GO:0032461 feature_id[608].value <= threshold=2.6103241443634033
node_480: feature_name=GO:0044710 feature_id[719].value <= threshold=2.228096718681627e-06

node_481: feature_name=GO:1904035 feature_id[654].value <= threshold=0.05152595415711403
node_482: feature_name=GO:2001235 feature_id[495].value <= threshold=1.1330987215042114
node_483: feature_name=hsa05169 feature_id[336].value > threshold=8.282931327819824
Class: positive genes

Rules_673 passed counts:1
node_0: feature_name=GO:0042113 feature_id[0].value <= threshold=13.408552169799805
node_1: feature_name=GO:0007568 feature_id[534].value <= threshold=5.0313897132873535
node_2: feature_name=GO:0002705 feature_id[541].value <= threshold=3.200145721435547
node_3: feature_name=GO:1901525 feature_id[576].value <= threshold=0.4466460347175598
node_4: feature_name=GO:0048539 feature_id[319].value <= threshold=3.0399646759033203
node_5: feature_name=GO:0001910 feature_id[385].value <= threshold=3.7437864542007446
node_6: feature_name=GO:0043200 feature_id[706].value <= threshold=9.307284355163574
node_7: feature_name=GO:0001773 feature_id[308].value <= threshold=4.016931533813477
node_8: feature_name=GO:0090116 feature_id[97].value <= threshold=7.99645471572876
node_9: feature_name=GO:0019814 feature_id[189].value <= threshold=4.525782108306885
node_10: feature_name=GO:1902583 feature_id[215].value <= threshold=17.60310649871826
node_11: feature_name=GO:0045429 feature_id[737].value <= threshold=1.6133361458778381
node_12: feature_name=GO:0003720 feature_id[228].value > threshold=5.519326210021973
node_474: feature_name=GO:0043367 feature_id[253].value > threshold=0.4537459146231413
Class: negative genes

Rules_674 passed counts:1
node_0: feature_name=GO:0042113 feature_id[0].value <= threshold=13.408552169799805
node_1: feature_name=GO:0007568 feature_id[534].value <= threshold=5.0313897132873535
node_2: feature_name=GO:0002705 feature_id[541].value <= threshold=3.200145721435547
node_3: feature_name=GO:1901525 feature_id[576].value <= threshold=0.4466460347175598
node_4: feature_name=GO:0048539 feature_id[319].value <= threshold=3.0399646759033203
node_5: feature_name=GO:0001910 feature_id[385].value <= threshold=3.7437864542007446
node_6: feature_name=GO:0043200 feature_id[706].value <= threshold=9.307284355163574
node_7: feature_name=GO:0001773 feature_id[308].value <= threshold=4.016931533813477
node_8: feature_name=GO:0090116 feature_id[97].value <= threshold=7.99645471572876
node_9: feature_name=GO:0019814 feature_id[189].value <= threshold=4.525782108306885
node_10: feature_name=GO:1902583 feature_id[215].value <= threshold=17.60310649871826
node_11: feature_name=GO:0045429 feature_id[737].value <= threshold=1.6133361458778381

node_12: feature_name=GO:0003720 feature_id[228].value <= threshold=5.519326210021973
node_13: feature_name=GO:0046006 feature_id[364].value > threshold=4.9985432624816895
Class: positive genes

Rules_675 passed counts:1
node_0: feature_name=GO:0042113 feature_id[0].value <= threshold=13.408552169799805
node_1: feature_name=GO:0007568 feature_id[534].value <= threshold=5.0313897132873535
node_2: feature_name=GO:0002705 feature_id[541].value <= threshold=3.200145721435547
node_3: feature_name=GO:1901525 feature_id[576].value <= threshold=0.4466460347175598
node_4: feature_name=GO:0048539 feature_id[319].value <= threshold=3.0399646759033203
node_5: feature_name=GO:0001910 feature_id[385].value <= threshold=3.7437864542007446
node_6: feature_name=GO:0043200 feature_id[706].value <= threshold=9.307284355163574
node_7: feature_name=GO:0001773 feature_id[308].value <= threshold=4.016931533813477
node_8: feature_name=GO:0090116 feature_id[97].value <= threshold=7.99645471572876
node_9: feature_name=GO:0019814 feature_id[189].value <= threshold=4.525782108306885
node_10: feature_name=GO:1902583 feature_id[215].value <= threshold=17.60310649871826
node_11: feature_name=GO:0045429 feature_id[737].value <= threshold=1.6133361458778381
node_12: feature_name=GO:0003720 feature_id[228].value <= threshold=5.519326210021973
node_13: feature_name=GO:0046006 feature_id[364].value <= threshold=4.9985432624816895
node_14: feature_name=GO:0070424 feature_id[254].value > threshold=6.233297109603882
Class: positive genes

Rules_676 passed counts:1
node_0: feature_name=GO:0042113 feature_id[0].value <= threshold=13.408552169799805
node_1: feature_name=GO:0007568 feature_id[534].value <= threshold=5.0313897132873535
node_2: feature_name=GO:0002705 feature_id[541].value <= threshold=3.200145721435547
node_3: feature_name=GO:1901525 feature_id[576].value <= threshold=0.4466460347175598
node_4: feature_name=GO:0048539 feature_id[319].value <= threshold=3.0399646759033203
node_5: feature_name=GO:0001910 feature_id[385].value <= threshold=3.7437864542007446
node_6: feature_name=GO:0043200 feature_id[706].value <= threshold=9.307284355163574
node_7: feature_name=GO:0001773 feature_id[308].value <= threshold=4.016931533813477
node_8: feature_name=GO:0090116 feature_id[97].value <= threshold=7.99645471572876
node_9: feature_name=GO:0019814 feature_id[189].value <= threshold=4.525782108306885
node_10: feature_name=GO:1902583 feature_id[215].value <= threshold=17.60310649871826
node_11: feature_name=GO:0045429 feature_id[737].value <= threshold=1.6133361458778381

node_12: feature_name=GO:0003720 feature_id[228].value <= threshold=5.519326210021973
node_13: feature_name=GO:0046006 feature_id[364].value <= threshold=4.9985432624816895
node_14: feature_name=GO:0070424 feature_id[254].value <= threshold=6.233297109603882
node_15: feature_name=GO:0009892 feature_id[320].value > threshold=59.99180793762207
Class: positive genes

Rules_677 passed counts:1
node_0: feature_name=GO:0042113 feature_id[0].value <= threshold=13.408552169799805
node_1: feature_name=GO:0007568 feature_id[534].value <= threshold=5.0313897132873535
node_2: feature_name=GO:0002705 feature_id[541].value <= threshold=3.200145721435547
node_3: feature_name=GO:1901525 feature_id[576].value <= threshold=0.4466460347175598
node_4: feature_name=GO:0048539 feature_id[319].value <= threshold=3.0399646759033203
node_5: feature_name=GO:0001910 feature_id[385].value <= threshold=3.7437864542007446
node_6: feature_name=GO:0043200 feature_id[706].value <= threshold=9.307284355163574
node_7: feature_name=GO:0001773 feature_id[308].value <= threshold=4.016931533813477
node_8: feature_name=GO:0090116 feature_id[97].value <= threshold=7.99645471572876
node_9: feature_name=GO:0019814 feature_id[189].value <= threshold=4.525782108306885
node_10: feature_name=GO:1902583 feature_id[215].value <= threshold=17.60310649871826
node_11: feature_name=GO:0045429 feature_id[737].value <= threshold=1.6133361458778381
node_12: feature_name=GO:0003720 feature_id[228].value <= threshold=5.519326210021973
node_13: feature_name=GO:0046006 feature_id[364].value <= threshold=4.9985432624816895
node_14: feature_name=GO:0070424 feature_id[254].value <= threshold=6.233297109603882
node_15: feature_name=GO:0009892 feature_id[320].value <= threshold=59.99180793762207
node_16: feature_name=GO:0007064 feature_id[527].value > threshold=11.57377815246582
Class: positive genes

Rules_678 passed counts:1
node_0: feature_name=GO:0042113 feature_id[0].value <= threshold=13.408552169799805
node_1: feature_name=GO:0007568 feature_id[534].value <= threshold=5.0313897132873535
node_2: feature_name=GO:0002705 feature_id[541].value <= threshold=3.200145721435547
node_3: feature_name=GO:1901525 feature_id[576].value <= threshold=0.4466460347175598
node_4: feature_name=GO:0048539 feature_id[319].value <= threshold=3.0399646759033203
node_5: feature_name=GO:0001910 feature_id[385].value <= threshold=3.7437864542007446
node_6: feature_name=GO:0043200 feature_id[706].value <= threshold=9.307284355163574
node_7: feature_name=GO:0001773 feature_id[308].value <= threshold=4.016931533813477

node_8: feature_name=GO:0090116 feature_id[97].value <= threshold=7.99645471572876
node_9: feature_name=GO:0019814 feature_id[189].value <= threshold=4.525782108306885
node_10: feature_name=GO:1902583 feature_id[215].value <= threshold=17.60310649871826
node_11: feature_name=GO:0045429 feature_id[737].value <= threshold=1.6133361458778381
node_12: feature_name=GO:0003720 feature_id[228].value <= threshold=5.519326210021973
node_13: feature_name=GO:0046006 feature_id[364].value <= threshold=4.9985432624816895
node_14: feature_name=GO:0070424 feature_id[254].value <= threshold=6.233297109603882
node_15: feature_name=GO:0009892 feature_id[320].value <= threshold=59.99180793762207
node_16: feature_name=GO:0007064 feature_id[527].value <= threshold=11.57377815246582
node_17: feature_name=GO:0005575 feature_id[17].value > threshold=15.417460918426514
Class: positive genes

Rules_679 passed counts:1
node_0: feature_name=GO:0042113 feature_id[0].value <= threshold=13.408552169799805
node_1: feature_name=GO:0007568 feature_id[534].value <= threshold=5.0313897132873535
node_2: feature_name=GO:0002705 feature_id[541].value <= threshold=3.200145721435547
node_3: feature_name=GO:1901525 feature_id[576].value <= threshold=0.4466460347175598
node_4: feature_name=GO:0048539 feature_id[319].value <= threshold=3.0399646759033203
node_5: feature_name=GO:0001910 feature_id[385].value <= threshold=3.7437864542007446
node_6: feature_name=GO:0043200 feature_id[706].value <= threshold=9.307284355163574
node_7: feature_name=GO:0001773 feature_id[308].value <= threshold=4.016931533813477
node_8: feature_name=GO:0090116 feature_id[97].value <= threshold=7.99645471572876
node_9: feature_name=GO:0019814 feature_id[189].value <= threshold=4.525782108306885
node_10: feature_name=GO:1902583 feature_id[215].value <= threshold=17.60310649871826
node_11: feature_name=GO:0045429 feature_id[737].value <= threshold=1.6133361458778381
node_12: feature_name=GO:0003720 feature_id[228].value <= threshold=5.519326210021973
node_13: feature_name=GO:0046006 feature_id[364].value <= threshold=4.9985432624816895
node_14: feature_name=GO:0070424 feature_id[254].value <= threshold=6.233297109603882
node_15: feature_name=GO:0009892 feature_id[320].value <= threshold=59.99180793762207
node_16: feature_name=GO:0007064 feature_id[527].value <= threshold=11.57377815246582
node_17: feature_name=GO:0005575 feature_id[17].value <= threshold=15.417460918426514
node_18: feature_name=GO:0043368 feature_id[31].value > threshold=4.059496641159058
Class: positive genes

Rules_680 passed counts:1

node_0: feature_name=GO:0042113 feature_id[0].value <= threshold=13.408552169799805
node_1: feature_name=GO:0007568 feature_id[534].value <= threshold=5.0313897132873535
node_2: feature_name=GO:0002705 feature_id[541].value <= threshold=3.200145721435547
node_3: feature_name=GO:1901525 feature_id[576].value <= threshold=0.4466460347175598
node_4: feature_name=GO:0048539 feature_id[319].value <= threshold=3.0399646759033203
node_5: feature_name=GO:0001910 feature_id[385].value <= threshold=3.7437864542007446
node_6: feature_name=GO:0043200 feature_id[706].value <= threshold=9.307284355163574
node_7: feature_name=GO:0001773 feature_id[308].value <= threshold=4.016931533813477
node_8: feature_name=GO:0090116 feature_id[97].value <= threshold=7.99645471572876
node_9: feature_name=GO:0019814 feature_id[189].value <= threshold=4.525782108306885
node_10: feature_name=GO:1902583 feature_id[215].value <= threshold=17.60310649871826
node_11: feature_name=GO:0045429 feature_id[737].value <= threshold=1.6133361458778381
node_12: feature_name=GO:0003720 feature_id[228].value <= threshold=5.519326210021973
node_13: feature_name=GO:0046006 feature_id[364].value <= threshold=4.9985432624816895
node_14: feature_name=GO:0070424 feature_id[254].value <= threshold=6.233297109603882
node_15: feature_name=GO:0009892 feature_id[320].value <= threshold=59.99180793762207
node_16: feature_name=GO:0007064 feature_id[527].value <= threshold=11.57377815246582
node_17: feature_name=GO:0005575 feature_id[17].value <= threshold=15.417460918426514
node_18: feature_name=GO:0043368 feature_id[31].value <= threshold=4.059496641159058
node_19: feature_name=GO:0005164 feature_id[163].value > threshold=5.793607711791992
Class: positive genes

Rules_681 passed counts:1
node_0: feature_name=GO:0042113 feature_id[0].value <= threshold=13.408552169799805
node_1: feature_name=GO:0007568 feature_id[534].value <= threshold=5.0313897132873535
node_2: feature_name=GO:0002705 feature_id[541].value <= threshold=3.200145721435547
node_3: feature_name=GO:1901525 feature_id[576].value <= threshold=0.4466460347175598
node_4: feature_name=GO:0048539 feature_id[319].value <= threshold=3.0399646759033203
node_5: feature_name=GO:0001910 feature_id[385].value <= threshold=3.7437864542007446
node_6: feature_name=GO:0043200 feature_id[706].value <= threshold=9.307284355163574
node_7: feature_name=GO:0001773 feature_id[308].value <= threshold=4.016931533813477
node_8: feature_name=GO:0090116 feature_id[97].value <= threshold=7.99645471572876
node_9: feature_name=GO:0019814 feature_id[189].value <= threshold=4.525782108306885
node_10: feature_name=GO:1902583 feature_id[215].value <= threshold=17.60310649871826
node_11: feature_name=GO:0045429 feature_id[737].value <= threshold=1.6133361458778381

node_12: feature_name=GO:0003720 feature_id[228].value <= threshold=5.519326210021973
node_13: feature_name=GO:0046006 feature_id[364].value <= threshold=4.9985432624816895
node_14: feature_name=GO:0070424 feature_id[254].value <= threshold=6.233297109603882
node_15: feature_name=GO:0009892 feature_id[320].value <= threshold=59.99180793762207
node_16: feature_name=GO:0007064 feature_id[527].value <= threshold=11.57377815246582
node_17: feature_name=GO:0005575 feature_id[17].value <= threshold=15.417460918426514
node_18: feature_name=GO:0043368 feature_id[31].value <= threshold=4.059496641159058
node_19: feature_name=GO:0005164 feature_id[163].value <= threshold=5.793607711791992
node_20: feature_name=GO:0042130 feature_id[16].value <= threshold=5.073179721832275
node_21: feature_name=GO:0010216 feature_id[282].value > threshold=4.834584474563599
node_461: feature_name=GO:2001020 feature_id[275].value > threshold=0.640952005982399
Class: positive genes

Rules_682 passed counts:1
node_0: feature_name=GO:0042113 feature_id[0].value <= threshold=13.408552169799805
node_1: feature_name=GO:0007568 feature_id[534].value <= threshold=5.0313897132873535
node_2: feature_name=GO:0002705 feature_id[541].value <= threshold=3.200145721435547
node_3: feature_name=GO:1901525 feature_id[576].value <= threshold=0.4466460347175598
node_4: feature_name=GO:0048539 feature_id[319].value <= threshold=3.0399646759033203
node_5: feature_name=GO:0001910 feature_id[385].value <= threshold=3.7437864542007446
node_6: feature_name=GO:0043200 feature_id[706].value <= threshold=9.307284355163574
node_7: feature_name=GO:0001773 feature_id[308].value <= threshold=4.016931533813477
node_8: feature_name=GO:0090116 feature_id[97].value <= threshold=7.99645471572876
node_9: feature_name=GO:0019814 feature_id[189].value <= threshold=4.525782108306885
node_10: feature_name=GO:1902583 feature_id[215].value <= threshold=17.60310649871826
node_11: feature_name=GO:0045429 feature_id[737].value <= threshold=1.6133361458778381
node_12: feature_name=GO:0003720 feature_id[228].value <= threshold=5.519326210021973
node_13: feature_name=GO:0046006 feature_id[364].value <= threshold=4.9985432624816895
node_14: feature_name=GO:0070424 feature_id[254].value <= threshold=6.233297109603882
node_15: feature_name=GO:0009892 feature_id[320].value <= threshold=59.99180793762207
node_16: feature_name=GO:0007064 feature_id[527].value <= threshold=11.57377815246582
node_17: feature_name=GO:0005575 feature_id[17].value <= threshold=15.417460918426514
node_18: feature_name=GO:0043368 feature_id[31].value <= threshold=4.059496641159058
node_19: feature_name=GO:0005164 feature_id[163].value <= threshold=5.793607711791992
node_20: feature_name=GO:0042130 feature_id[16].value <= threshold=5.073179721832275

node_21: feature_name=GO:0010216 feature_id[282].value > threshold=4.834584474563599
node_461: feature_name=GO:2001020 feature_id[275].value <= threshold=0.640952005982399
Class: negative genes

Rules_683 passed counts:1
node_0: feature_name=GO:0042113 feature_id[0].value <= threshold=13.408552169799805
node_1: feature_name=GO:0007568 feature_id[534].value <= threshold=5.0313897132873535
node_2: feature_name=GO:0002705 feature_id[541].value <= threshold=3.200145721435547
node_3: feature_name=GO:1901525 feature_id[576].value <= threshold=0.4466460347175598
node_4: feature_name=GO:0048539 feature_id[319].value <= threshold=3.0399646759033203
node_5: feature_name=GO:0001910 feature_id[385].value <= threshold=3.7437864542007446
node_6: feature_name=GO:0043200 feature_id[706].value <= threshold=9.307284355163574
node_7: feature_name=GO:0001773 feature_id[308].value <= threshold=4.016931533813477
node_8: feature_name=GO:0090116 feature_id[97].value <= threshold=7.99645471572876
node_9: feature_name=GO:0019814 feature_id[189].value <= threshold=4.525782108306885
node_10: feature_name=GO:1902583 feature_id[215].value <= threshold=17.60310649871826
node_11: feature_name=GO:0045429 feature_id[737].value <= threshold=1.6133361458778381
node_12: feature_name=GO:0003720 feature_id[228].value <= threshold=5.519326210021973
node_13: feature_name=GO:0046006 feature_id[364].value <= threshold=4.9985432624816895
node_14: feature_name=GO:0070424 feature_id[254].value <= threshold=6.233297109603882
node_15: feature_name=GO:0009892 feature_id[320].value <= threshold=59.99180793762207
node_16: feature_name=GO:0007064 feature_id[527].value <= threshold=11.57377815246582
node_17: feature_name=GO:0005575 feature_id[17].value <= threshold=15.417460918426514
node_18: feature_name=GO:0043368 feature_id[31].value <= threshold=4.059496641159058
node_19: feature_name=GO:0005164 feature_id[163].value <= threshold=5.793607711791992
node_20: feature_name=GO:0042130 feature_id[16].value <= threshold=5.073179721832275
node_21: feature_name=GO:0010216 feature_id[282].value <= threshold=4.834584474563599
node_22: feature_name=GO:0009628 feature_id[553].value > threshold=29.314892768859863
node_458: feature_name=GO:0070266 feature_id[84].value > threshold=0.37941037118434906
Class: negative genes

Rules_684 passed counts:1
node_0: feature_name=GO:0042113 feature_id[0].value <= threshold=13.408552169799805
node_1: feature_name=GO:0007568 feature_id[534].value <= threshold=5.0313897132873535
node_2: feature_name=GO:0002705 feature_id[541].value <= threshold=3.200145721435547

node_3: feature_name=GO:1901525 feature_id[576].value <= threshold=0.4466460347175598
node_4: feature_name=GO:0048539 feature_id[319].value <= threshold=3.0399646759033203
node_5: feature_name=GO:0001910 feature_id[385].value <= threshold=3.7437864542007446
node_6: feature_name=GO:0043200 feature_id[706].value <= threshold=9.307284355163574
node_7: feature_name=GO:0001773 feature_id[308].value <= threshold=4.016931533813477
node_8: feature_name=GO:0090116 feature_id[97].value <= threshold=7.99645471572876
node_9: feature_name=GO:0019814 feature_id[189].value <= threshold=4.525782108306885
node_10: feature_name=GO:1902583 feature_id[215].value <= threshold=17.60310649871826
node_11: feature_name=GO:0045429 feature_id[737].value <= threshold=1.6133361458778381
node_12: feature_name=GO:0003720 feature_id[228].value <= threshold=5.519326210021973
node_13: feature_name=GO:0046006 feature_id[364].value <= threshold=4.9985432624816895
node_14: feature_name=GO:0070424 feature_id[254].value <= threshold=6.233297109603882
node_15: feature_name=GO:0009892 feature_id[320].value <= threshold=59.99180793762207
node_16: feature_name=GO:0007064 feature_id[527].value <= threshold=11.57377815246582
node_17: feature_name=GO:0005575 feature_id[17].value <= threshold=15.417460918426514
node_18: feature_name=GO:0043368 feature_id[31].value <= threshold=4.059496641159058
node_19: feature_name=GO:0005164 feature_id[163].value <= threshold=5.793607711791992
node_20: feature_name=GO:0042130 feature_id[16].value <= threshold=5.073179721832275
node_21: feature_name=GO:0010216 feature_id[282].value <= threshold=4.834584474563599
node_22: feature_name=GO:0009628 feature_id[553].value > threshold=29.314892768859863
node_458: feature_name=GO:0070266 feature_id[84].value <= threshold=0.37941037118434906
Class: positive genes

Rules_685 passed counts:1
node_0: feature_name=GO:0042113 feature_id[0].value <= threshold=13.408552169799805
node_1: feature_name=GO:0007568 feature_id[534].value <= threshold=5.0313897132873535
node_2: feature_name=GO:0002705 feature_id[541].value <= threshold=3.200145721435547
node_3: feature_name=GO:1901525 feature_id[576].value <= threshold=0.4466460347175598
node_4: feature_name=GO:0048539 feature_id[319].value <= threshold=3.0399646759033203
node_5: feature_name=GO:0001910 feature_id[385].value <= threshold=3.7437864542007446
node_6: feature_name=GO:0043200 feature_id[706].value <= threshold=9.307284355163574
node_7: feature_name=GO:0001773 feature_id[308].value <= threshold=4.016931533813477
node_8: feature_name=GO:0090116 feature_id[97].value <= threshold=7.99645471572876
node_9: feature_name=GO:0019814 feature_id[189].value <= threshold=4.525782108306885
node_10: feature_name=GO:1902583 feature_id[215].value <= threshold=17.60310649871826

node_11: feature_name=GO:0045429 feature_id[737].value <= threshold=1.6133361458778381
node_12: feature_name=GO:0003720 feature_id[228].value <= threshold=5.519326210021973
node_13: feature_name=GO:0046006 feature_id[364].value <= threshold=4.9985432624816895
node_14: feature_name=GO:0070424 feature_id[254].value <= threshold=6.233297109603882
node_15: feature_name=GO:0009892 feature_id[320].value <= threshold=59.99180793762207
node_16: feature_name=GO:0007064 feature_id[527].value <= threshold=11.57377815246582
node_17: feature_name=GO:0005575 feature_id[17].value <= threshold=15.417460918426514
node_18: feature_name=GO:0043368 feature_id[31].value <= threshold=4.059496641159058
node_19: feature_name=GO:0005164 feature_id[163].value <= threshold=5.793607711791992
node_20: feature_name=GO:0042130 feature_id[16].value <= threshold=5.073179721832275
node_21: feature_name=GO:0010216 feature_id[282].value <= threshold=4.834584474563599
node_22: feature_name=GO:0009628 feature_id[553].value <= threshold=29.314892768859863
node_23: feature_name=GO:0045628 feature_id[749].value > threshold=3.1001367568969727
node_455: feature_name=GO:0001782 feature_id[240].value > threshold=0.23332832753658295
Class: negative genes

Rules_686 passed counts:1
node_0: feature_name=GO:0042113 feature_id[0].value <= threshold=13.408552169799805
node_1: feature_name=GO:0007568 feature_id[534].value <= threshold=5.0313897132873535
node_2: feature_name=GO:0002705 feature_id[541].value <= threshold=3.200145721435547
node_3: feature_name=GO:1901525 feature_id[576].value <= threshold=0.4466460347175598
node_4: feature_name=GO:0048539 feature_id[319].value <= threshold=3.0399646759033203
node_5: feature_name=GO:0001910 feature_id[385].value <= threshold=3.7437864542007446
node_6: feature_name=GO:0043200 feature_id[706].value <= threshold=9.307284355163574
node_7: feature_name=GO:0001773 feature_id[308].value <= threshold=4.016931533813477
node_8: feature_name=GO:0090116 feature_id[97].value <= threshold=7.99645471572876
node_9: feature_name=GO:0019814 feature_id[189].value <= threshold=4.525782108306885
node_10: feature_name=GO:1902583 feature_id[215].value <= threshold=17.60310649871826
node_11: feature_name=GO:0045429 feature_id[737].value <= threshold=1.6133361458778381
node_12: feature_name=GO:0003720 feature_id[228].value <= threshold=5.519326210021973
node_13: feature_name=GO:0046006 feature_id[364].value <= threshold=4.9985432624816895
node_14: feature_name=GO:0070424 feature_id[254].value <= threshold=6.233297109603882
node_15: feature_name=GO:0009892 feature_id[320].value <= threshold=59.99180793762207
node_16: feature_name=GO:0007064 feature_id[527].value <= threshold=11.57377815246582
node_17: feature_name=GO:0005575 feature_id[17].value <= threshold=15.417460918426514

node_18: feature_name=GO:0043368 feature_id[31].value <= threshold=4.059496641159058
node_19: feature_name=GO:0005164 feature_id[163].value <= threshold=5.793607711791992
node_20: feature_name=GO:0042130 feature_id[16].value <= threshold=5.073179721832275
node_21: feature_name=GO:0010216 feature_id[282].value <= threshold=4.834584474563599
node_22: feature_name=GO:0009628 feature_id[553].value <= threshold=29.314892768859863
node_23: feature_name=GO:0045628 feature_id[749].value > threshold=3.1001367568969727
node_455: feature_name=GO:0001782 feature_id[240].value <= threshold=0.23332832753658295
Class: positive genes

Rules_687 passed counts:1
node_0: feature_name=GO:0042113 feature_id[0].value <= threshold=13.408552169799805
node_1: feature_name=GO:0007568 feature_id[534].value <= threshold=5.0313897132873535
node_2: feature_name=GO:0002705 feature_id[541].value <= threshold=3.200145721435547
node_3: feature_name=GO:1901525 feature_id[576].value <= threshold=0.4466460347175598
node_4: feature_name=GO:0048539 feature_id[319].value <= threshold=3.0399646759033203
node_5: feature_name=GO:0001910 feature_id[385].value <= threshold=3.7437864542007446
node_6: feature_name=GO:0043200 feature_id[706].value <= threshold=9.307284355163574
node_7: feature_name=GO:0001773 feature_id[308].value <= threshold=4.016931533813477
node_8: feature_name=GO:0090116 feature_id[97].value <= threshold=7.99645471572876
node_9: feature_name=GO:0019814 feature_id[189].value <= threshold=4.525782108306885
node_10: feature_name=GO:1902583 feature_id[215].value <= threshold=17.60310649871826
node_11: feature_name=GO:0045429 feature_id[737].value <= threshold=1.6133361458778381
node_12: feature_name=GO:0003720 feature_id[228].value <= threshold=5.519326210021973
node_13: feature_name=GO:0046006 feature_id[364].value <= threshold=4.9985432624816895
node_14: feature_name=GO:0070424 feature_id[254].value <= threshold=6.233297109603882
node_15: feature_name=GO:0009892 feature_id[320].value <= threshold=59.99180793762207
node_16: feature_name=GO:0007064 feature_id[527].value <= threshold=11.57377815246582
node_17: feature_name=GO:0005575 feature_id[17].value <= threshold=15.417460918426514
node_18: feature_name=GO:0043368 feature_id[31].value <= threshold=4.059496641159058
node_19: feature_name=GO:0005164 feature_id[163].value <= threshold=5.793607711791992
node_20: feature_name=GO:0042130 feature_id[16].value <= threshold=5.073179721832275
node_21: feature_name=GO:0010216 feature_id[282].value <= threshold=4.834584474563599
node_22: feature_name=GO:0009628 feature_id[553].value <= threshold=29.314892768859863
node_23: feature_name=GO:0045628 feature_id[749].value <= threshold=3.1001367568969727
node_24: feature_name=GO:0042288 feature_id[100].value > threshold=5.174932956695557

node_452: feature_name=GO:0070233 feature_id[321].value > threshold=0.107149638235569
Class: negative genes

Rules_688 passed counts:1
node_0: feature_name=GO:0042113 feature_id[0].value <= threshold=13.408552169799805
node_1: feature_name=GO:0007568 feature_id[534].value <= threshold=5.0313897132873535
node_2: feature_name=GO:0002705 feature_id[541].value <= threshold=3.200145721435547
node_3: feature_name=GO:1901525 feature_id[576].value <= threshold=0.4466460347175598
node_4: feature_name=GO:0048539 feature_id[319].value <= threshold=3.0399646759033203
node_5: feature_name=GO:0001910 feature_id[385].value <= threshold=3.7437864542007446
node_6: feature_name=GO:0043200 feature_id[706].value <= threshold=9.307284355163574
node_7: feature_name=GO:0001773 feature_id[308].value <= threshold=4.016931533813477
node_8: feature_name=GO:0090116 feature_id[97].value <= threshold=7.99645471572876
node_9: feature_name=GO:0019814 feature_id[189].value <= threshold=4.525782108306885
node_10: feature_name=GO:1902583 feature_id[215].value <= threshold=17.60310649871826
node_11: feature_name=GO:0045429 feature_id[737].value <= threshold=1.6133361458778381
node_12: feature_name=GO:0003720 feature_id[228].value <= threshold=5.519326210021973
node_13: feature_name=GO:0046006 feature_id[364].value <= threshold=4.9985432624816895
node_14: feature_name=GO:0070424 feature_id[254].value <= threshold=6.233297109603882
node_15: feature_name=GO:0009892 feature_id[320].value <= threshold=59.99180793762207
node_16: feature_name=GO:0007064 feature_id[527].value <= threshold=11.57377815246582
node_17: feature_name=GO:0005575 feature_id[17].value <= threshold=15.417460918426514
node_18: feature_name=GO:0043368 feature_id[31].value <= threshold=4.059496641159058
node_19: feature_name=GO:0005164 feature_id[163].value <= threshold=5.793607711791992
node_20: feature_name=GO:0042130 feature_id[16].value <= threshold=5.073179721832275
node_21: feature_name=GO:0010216 feature_id[282].value <= threshold=4.834584474563599
node_22: feature_name=GO:0009628 feature_id[553].value <= threshold=29.314892768859863
node_23: feature_name=GO:0045628 feature_id[749].value <= threshold=3.1001367568969727
node_24: feature_name=GO:0042288 feature_id[100].value > threshold=5.174932956695557
node_452: feature_name=GO:0070233 feature_id[321].value <= threshold=0.107149638235569
Class: positive genes

Rules_689 passed counts:1
node_0: feature_name=GO:0042113 feature_id[0].value <= threshold=13.408552169799805
node_1: feature_name=GO:0007568 feature_id[534].value <= threshold=5.0313897132873535

node_2: feature_name=GO:0002705 feature_id[541].value <= threshold=3.200145721435547
node_3: feature_name=GO:1901525 feature_id[576].value <= threshold=0.4466460347175598
node_4: feature_name=GO:0048539 feature_id[319].value <= threshold=3.0399646759033203
node_5: feature_name=GO:0001910 feature_id[385].value <= threshold=3.7437864542007446
node_6: feature_name=GO:0043200 feature_id[706].value <= threshold=9.307284355163574
node_7: feature_name=GO:0001773 feature_id[308].value <= threshold=4.016931533813477
node_8: feature_name=GO:0090116 feature_id[97].value <= threshold=7.99645471572876
node_9: feature_name=GO:0019814 feature_id[189].value <= threshold=4.525782108306885
node_10: feature_name=GO:1902583 feature_id[215].value <= threshold=17.60310649871826
node_11: feature_name=GO:0045429 feature_id[737].value <= threshold=1.6133361458778381
node_12: feature_name=GO:0003720 feature_id[228].value <= threshold=5.519326210021973
node_13: feature_name=GO:0046006 feature_id[364].value <= threshold=4.9985432624816895
node_14: feature_name=GO:0070424 feature_id[254].value <= threshold=6.233297109603882
node_15: feature_name=GO:0009892 feature_id[320].value <= threshold=59.99180793762207
node_16: feature_name=GO:0007064 feature_id[527].value <= threshold=11.57377815246582
node_17: feature_name=GO:0005575 feature_id[17].value <= threshold=15.417460918426514
node_18: feature_name=GO:0043368 feature_id[31].value <= threshold=4.059496641159058
node_19: feature_name=GO:0005164 feature_id[163].value <= threshold=5.793607711791992
node_20: feature_name=GO:0042130 feature_id[16].value <= threshold=5.073179721832275
node_21: feature_name=GO:0010216 feature_id[282].value <= threshold=4.834584474563599
node_22: feature_name=GO:0009628 feature_id[553].value <= threshold=29.314892768859863
node_23: feature_name=GO:0045628 feature_id[749].value <= threshold=3.1001367568969727
node_24: feature_name=GO:0042288 feature_id[100].value <= threshold=5.174932956695557
node_25: feature_name=GO:0002329 feature_id[406].value <= threshold=2.348930835723877
node_26: feature_name=GO:0051246 feature_id[642].value > threshold=0.6498909294605255
node_194: feature_name=GO:0009164 feature_id[546].value > threshold=17.11970329284668
Class: positive genes

Rules_690 passed counts:1
node_0: feature_name=GO:0042113 feature_id[0].value <= threshold=13.408552169799805
node_1: feature_name=GO:0007568 feature_id[534].value <= threshold=5.0313897132873535
node_2: feature_name=GO:0002705 feature_id[541].value <= threshold=3.200145721435547
node_3: feature_name=GO:1901525 feature_id[576].value <= threshold=0.4466460347175598
node_4: feature_name=GO:0048539 feature_id[319].value <= threshold=3.0399646759033203
node_5: feature_name=GO:0001910 feature_id[385].value <= threshold=3.7437864542007446

node_6: feature_name=GO:0043200 feature_id[706].value <= threshold=9.307284355163574
node_7: feature_name=GO:0001773 feature_id[308].value <= threshold=4.016931533813477
node_8: feature_name=GO:0090116 feature_id[97].value <= threshold=7.99645471572876
node_9: feature_name=GO:0019814 feature_id[189].value <= threshold=4.525782108306885
node_10: feature_name=GO:1902583 feature_id[215].value <= threshold=17.60310649871826
node_11: feature_name=GO:0045429 feature_id[737].value <= threshold=1.6133361458778381
node_12: feature_name=GO:0003720 feature_id[228].value <= threshold=5.519326210021973
node_13: feature_name=GO:0046006 feature_id[364].value <= threshold=4.9985432624816895
node_14: feature_name=GO:0070424 feature_id[254].value <= threshold=6.233297109603882
node_15: feature_name=GO:0009892 feature_id[320].value <= threshold=59.99180793762207
node_16: feature_name=GO:0007064 feature_id[527].value <= threshold=11.57377815246582
node_17: feature_name=GO:0005575 feature_id[17].value <= threshold=15.417460918426514
node_18: feature_name=GO:0043368 feature_id[31].value <= threshold=4.059496641159058
node_19: feature_name=GO:0005164 feature_id[163].value <= threshold=5.793607711791992
node_20: feature_name=GO:0042130 feature_id[16].value <= threshold=5.073179721832275
node_21: feature_name=GO:0010216 feature_id[282].value <= threshold=4.834584474563599
node_22: feature_name=GO:0009628 feature_id[553].value <= threshold=29.314892768859863
node_23: feature_name=GO:0045628 feature_id[749].value <= threshold=3.1001367568969727
node_24: feature_name=GO:0042288 feature_id[100].value <= threshold=5.174932956695557
node_25: feature_name=GO:0002329 feature_id[406].value <= threshold=2.348930835723877
node_26: feature_name=GO:0051246 feature_id[642].value > threshold=0.6498909294605255
node_194: feature_name=GO:0009164 feature_id[546].value <= threshold=17.11970329284668
node_195: feature_name=GO:0001909 feature_id[386].value > threshold=7.292566299438477
Class: positive genes

Rules_691 passed counts:1
node_0: feature_name=GO:0042113 feature_id[0].value <= threshold=13.408552169799805
node_1: feature_name=GO:0007568 feature_id[534].value <= threshold=5.0313897132873535
node_2: feature_name=GO:0002705 feature_id[541].value <= threshold=3.200145721435547
node_3: feature_name=GO:1901525 feature_id[576].value <= threshold=0.4466460347175598
node_4: feature_name=GO:0048539 feature_id[319].value <= threshold=3.0399646759033203
node_5: feature_name=GO:0001910 feature_id[385].value <= threshold=3.7437864542007446
node_6: feature_name=GO:0043200 feature_id[706].value <= threshold=9.307284355163574
node_7: feature_name=GO:0001773 feature_id[308].value <= threshold=4.016931533813477
node_8: feature_name=GO:0090116 feature_id[97].value <= threshold=7.99645471572876

node_9: feature_name=GO:0019814 feature_id[189].value <= threshold=4.525782108306885
node_10: feature_name=GO:1902583 feature_id[215].value <= threshold=17.60310649871826
node_11: feature_name=GO:0045429 feature_id[737].value <= threshold=1.6133361458778381
node_12: feature_name=GO:0003720 feature_id[228].value <= threshold=5.519326210021973
node_13: feature_name=GO:0046006 feature_id[364].value <= threshold=4.9985432624816895
node_14: feature_name=GO:0070424 feature_id[254].value <= threshold=6.233297109603882
node_15: feature_name=GO:0009892 feature_id[320].value <= threshold=59.99180793762207
node_16: feature_name=GO:0007064 feature_id[527].value <= threshold=11.57377815246582
node_17: feature_name=GO:0005575 feature_id[17].value <= threshold=15.417460918426514
node_18: feature_name=GO:0043368 feature_id[31].value <= threshold=4.059496641159058
node_19: feature_name=GO:0005164 feature_id[163].value <= threshold=5.793607711791992
node_20: feature_name=GO:0042130 feature_id[16].value <= threshold=5.073179721832275
node_21: feature_name=GO:0010216 feature_id[282].value <= threshold=4.834584474563599
node_22: feature_name=GO:0009628 feature_id[553].value <= threshold=29.314892768859863
node_23: feature_name=GO:0045628 feature_id[749].value <= threshold=3.1001367568969727
node_24: feature_name=GO:0042288 feature_id[100].value <= threshold=5.174932956695557
node_25: feature_name=GO:0002329 feature_id[406].value <= threshold=2.348930835723877
node_26: feature_name=GO:0051246 feature_id[642].value > threshold=0.6498909294605255
node_194: feature_name=GO:0009164 feature_id[546].value <= threshold=17.11970329284668
node_195: feature_name=GO:0001909 feature_id[386].value <= threshold=7.292566299438477
node_196: feature_name=GO:0003908 feature_id[11].value <= threshold=1.887226164340973
node_197: feature_name=GO:0009164 feature_id[546].value > threshold=0.26642198860645294
node_359: feature_name=GO:0009164 feature_id[546].value > threshold=0.2696942090988159
node_361: feature_name=hsa05202 feature_id[50].value > threshold=14.989679336547852
Class: positive genes

Rules_692 passed counts:1
node_0: feature_name=GO:0042113 feature_id[0].value <= threshold=13.408552169799805
node_1: feature_name=GO:0007568 feature_id[534].value <= threshold=5.0313897132873535
node_2: feature_name=GO:0002705 feature_id[541].value <= threshold=3.200145721435547
node_3: feature_name=GO:1901525 feature_id[576].value <= threshold=0.4466460347175598
node_4: feature_name=GO:0048539 feature_id[319].value <= threshold=3.0399646759033203
node_5: feature_name=GO:0001910 feature_id[385].value <= threshold=3.7437864542007446
node_6: feature_name=GO:0043200 feature_id[706].value <= threshold=9.307284355163574
node_7: feature_name=GO:0001773 feature_id[308].value <= threshold=4.016931533813477

node_8: feature_name=GO:0090116 feature_id[97].value <= threshold=7.99645471572876
node_9: feature_name=GO:0019814 feature_id[189].value <= threshold=4.525782108306885
node_10: feature_name=GO:1902583 feature_id[215].value <= threshold=17.60310649871826
node_11: feature_name=GO:0045429 feature_id[737].value <= threshold=1.6133361458778381
node_12: feature_name=GO:0003720 feature_id[228].value <= threshold=5.519326210021973
node_13: feature_name=GO:0046006 feature_id[364].value <= threshold=4.9985432624816895
node_14: feature_name=GO:0070424 feature_id[254].value <= threshold=6.233297109603882
node_15: feature_name=GO:0009892 feature_id[320].value <= threshold=59.99180793762207
node_16: feature_name=GO:0007064 feature_id[527].value <= threshold=11.57377815246582
node_17: feature_name=GO:0005575 feature_id[17].value <= threshold=15.417460918426514
node_18: feature_name=GO:0043368 feature_id[31].value <= threshold=4.059496641159058
node_19: feature_name=GO:0005164 feature_id[163].value <= threshold=5.793607711791992
node_20: feature_name=GO:0042130 feature_id[16].value <= threshold=5.073179721832275
node_21: feature_name=GO:0010216 feature_id[282].value <= threshold=4.834584474563599
node_22: feature_name=GO:0009628 feature_id[553].value <= threshold=29.314892768859863
node_23: feature_name=GO:0045628 feature_id[749].value <= threshold=3.1001367568969727
node_24: feature_name=GO:0042288 feature_id[100].value <= threshold=5.174932956695557
node_25: feature_name=GO:0002329 feature_id[406].value <= threshold=2.348930835723877
node_26: feature_name=GO:0051246 feature_id[642].value > threshold=0.6498909294605255
node_194: feature_name=GO:0009164 feature_id[546].value <= threshold=17.11970329284668
node_195: feature_name=GO:0001909 feature_id[386].value <= threshold=7.292566299438477
node_196: feature_name=GO:0003908 feature_id[11].value <= threshold=1.887226164340973
node_197: feature_name=GO:0009164 feature_id[546].value > threshold=0.26642198860645294
node_359: feature_name=GO:0009164 feature_id[546].value > threshold=0.2696942090988159
node_361: feature_name=hsa05202 feature_id[50].value <= threshold=14.989679336547852
node_362: feature_name=GO:0001775 feature_id[505].value > threshold=15.726949691772461
Class: positive genes

Rules_693 passed counts:1
node_0: feature_name=GO:0042113 feature_id[0].value <= threshold=13.408552169799805
node_1: feature_name=GO:0007568 feature_id[534].value <= threshold=5.0313897132873535
node_2: feature_name=GO:0002705 feature_id[541].value <= threshold=3.200145721435547
node_3: feature_name=GO:1901525 feature_id[576].value <= threshold=0.4466460347175598
node_4: feature_name=GO:0048539 feature_id[319].value <= threshold=3.0399646759033203
node_5: feature_name=GO:0001910 feature_id[385].value <= threshold=3.7437864542007446

node_6: feature_name=GO:0043200 feature_id[706].value <= threshold=9.307284355163574
node_7: feature_name=GO:0001773 feature_id[308].value <= threshold=4.016931533813477
node_8: feature_name=GO:0090116 feature_id[97].value <= threshold=7.99645471572876
node_9: feature_name=GO:0019814 feature_id[189].value <= threshold=4.525782108306885
node_10: feature_name=GO:1902583 feature_id[215].value <= threshold=17.60310649871826
node_11: feature_name=GO:0045429 feature_id[737].value <= threshold=1.6133361458778381
node_12: feature_name=GO:0003720 feature_id[228].value <= threshold=5.519326210021973
node_13: feature_name=GO:0046006 feature_id[364].value <= threshold=4.9985432624816895
node_14: feature_name=GO:0070424 feature_id[254].value <= threshold=6.233297109603882
node_15: feature_name=GO:0009892 feature_id[320].value <= threshold=59.99180793762207
node_16: feature_name=GO:0007064 feature_id[527].value <= threshold=11.57377815246582
node_17: feature_name=GO:0005575 feature_id[17].value <= threshold=15.417460918426514
node_18: feature_name=GO:0043368 feature_id[31].value <= threshold=4.059496641159058
node_19: feature_name=GO:0005164 feature_id[163].value <= threshold=5.793607711791992
node_20: feature_name=GO:0042130 feature_id[16].value <= threshold=5.073179721832275
node_21: feature_name=GO:0010216 feature_id[282].value <= threshold=4.834584474563599
node_22: feature_name=GO:0009628 feature_id[553].value <= threshold=29.314892768859863
node_23: feature_name=GO:0045628 feature_id[749].value <= threshold=3.1001367568969727
node_24: feature_name=GO:0042288 feature_id[100].value <= threshold=5.174932956695557
node_25: feature_name=GO:0002329 feature_id[406].value <= threshold=2.348930835723877
node_26: feature_name=GO:0051246 feature_id[642].value > threshold=0.6498909294605255
node_194: feature_name=GO:0009164 feature_id[546].value <= threshold=17.11970329284668
node_195: feature_name=GO:0001909 feature_id[386].value <= threshold=7.292566299438477
node_196: feature_name=GO:0003908 feature_id[11].value <= threshold=1.887226164340973
node_197: feature_name=GO:0009164 feature_id[546].value > threshold=0.26642198860645294
node_359: feature_name=GO:0009164 feature_id[546].value > threshold=0.2696942090988159
node_361: feature_name=hsa05202 feature_id[50].value <= threshold=14.989679336547852
node_362: feature_name=GO:0001775 feature_id[505].value <= threshold=15.726949691772461
node_363: feature_name=GO:0002634 feature_id[449].value > threshold=2.825531005859375
Class: positive genes

Rules_694 passed counts:1
node_0: feature_name=GO:0042113 feature_id[0].value <= threshold=13.408552169799805
node_1: feature_name=GO:0007568 feature_id[534].value <= threshold=5.0313897132873535
node_2: feature_name=GO:0002705 feature_id[541].value <= threshold=3.200145721435547

node_3: feature_name=GO:1901525	feature_id[576].value <= threshold=0.4466460347175598
node_4: feature_name=GO:0048539	feature_id[319].value <= threshold=3.0399646759033203
node_5: feature_name=GO:0001910	feature_id[385].value <= threshold=3.7437864542007446
node_6: feature_name=GO:0043200	feature_id[706].value <= threshold=9.307284355163574
node_7: feature_name=GO:0001773	feature_id[308].value <= threshold=4.016931533813477
node_8: feature_name=GO:0090116	feature_id[97].value <= threshold=7.99645471572876
node_9: feature_name=GO:0019814	feature_id[189].value <= threshold=4.525782108306885
node_10: feature_name=GO:1902583	feature_id[215].value <= threshold=17.60310649871826
node_11: feature_name=GO:0045429	feature_id[737].value <= threshold=1.6133361458778381
node_12: feature_name=GO:0003720	feature_id[228].value <= threshold=5.519326210021973
node_13: feature_name=GO:0046006	feature_id[364].value <= threshold=4.9985432624816895
node_14: feature_name=GO:0070424	feature_id[254].value <= threshold=6.233297109603882
node_15: feature_name=GO:0009892	feature_id[320].value <= threshold=59.99180793762207
node_16: feature_name=GO:0007064	feature_id[527].value <= threshold=11.57377815246582
node_17: feature_name=GO:0005575	feature_id[17].value <= threshold=15.417460918426514
node_18: feature_name=GO:0043368	feature_id[31].value <= threshold=4.059496641159058
node_19: feature_name=GO:0005164	feature_id[163].value <= threshold=5.793607711791992
node_20: feature_name=GO:0042130	feature_id[16].value <= threshold=5.073179721832275
node_21: feature_name=GO:0010216	feature_id[282].value <= threshold=4.834584474563599
node_22: feature_name=GO:0009628	feature_id[553].value <= threshold=29.314892768859863
node_23: feature_name=GO:0045628	feature_id[749].value <= threshold=3.1001367568969727
node_24: feature_name=GO:0042288	feature_id[100].value <= threshold=5.174932956695557
node_25: feature_name=GO:0002329	feature_id[406].value <= threshold=2.348930835723877
node_26: feature_name=GO:0051246	feature_id[642].value > threshold=0.6498909294605255
node_194: feature_name=GO:0009164	feature_id[546].value <= threshold=17.11970329284668
node_195: feature_name=GO:0001909	feature_id[386].value <= threshold=7.292566299438477
node_196: feature_name=GO:0003908	feature_id[11].value <= threshold=1.887226164340973
node_197: feature_name=GO:0009164	feature_id[546].value > threshold=0.26642198860645294
node_359: feature_name=GO:0009164	feature_id[546].value > threshold=0.2696942090988159
node_361: feature_name=hsa05202	feature_id[50].value <= threshold=14.989679336547852
node_362: feature_name=GO:0001775	feature_id[505].value <= threshold=15.726949691772461
node_363: feature_name=GO:0002634	feature_id[449].value <= threshold=2.825531005859375
node_364: feature_name=GO:0010038	feature_id[457].value > threshold=9.901018619537354

Class: positive genes

Rules_695 passed counts:1
node_0: feature_name=GO:0042113 feature_id[0].value <= threshold=13.408552169799805
node_1: feature_name=GO:0007568 feature_id[534].value <= threshold=5.0313897132873535
node_2: feature_name=GO:0002705 feature_id[541].value <= threshold=3.200145721435547
node_3: feature_name=GO:1901525 feature_id[576].value <= threshold=0.4466460347175598
node_4: feature_name=GO:0048539 feature_id[319].value <= threshold=3.0399646759033203
node_5: feature_name=GO:0001910 feature_id[385].value <= threshold=3.7437864542007446
node_6: feature_name=GO:0043200 feature_id[706].value <= threshold=9.307284355163574
node_7: feature_name=GO:0001773 feature_id[308].value <= threshold=4.016931533813477
node_8: feature_name=GO:0090116 feature_id[97].value <= threshold=7.99645471572876
node_9: feature_name=GO:0019814 feature_id[189].value <= threshold=4.525782108306885
node_10: feature_name=GO:1902583 feature_id[215].value <= threshold=17.60310649871826
node_11: feature_name=GO:0045429 feature_id[737].value <= threshold=1.6133361458778381
node_12: feature_name=GO:0003720 feature_id[228].value <= threshold=5.519326210021973
node_13: feature_name=GO:0046006 feature_id[364].value <= threshold=4.9985432624816895
node_14: feature_name=GO:0070424 feature_id[254].value <= threshold=6.233297109603882
node_15: feature_name=GO:0009892 feature_id[320].value <= threshold=59.99180793762207
node_16: feature_name=GO:0007064 feature_id[527].value <= threshold=11.57377815246582
node_17: feature_name=GO:0005575 feature_id[17].value <= threshold=15.417460918426514
node_18: feature_name=GO:0043368 feature_id[31].value <= threshold=4.059496641159058
node_19: feature_name=GO:0005164 feature_id[163].value <= threshold=5.793607711791992
node_20: feature_name=GO:0042130 feature_id[16].value <= threshold=5.073179721832275
node_21: feature_name=GO:0010216 feature_id[282].value <= threshold=4.834584474563599
node_22: feature_name=GO:0009628 feature_id[553].value <= threshold=29.314892768859863
node_23: feature_name=GO:0045628 feature_id[749].value <= threshold=3.1001367568969727
node_24: feature_name=GO:0042288 feature_id[100].value <= threshold=5.174932956695557
node_25: feature_name=GO:0002329 feature_id[406].value <= threshold=2.348930835723877
node_26: feature_name=GO:0051246 feature_id[642].value > threshold=0.6498909294605255
node_194: feature_name=GO:0009164 feature_id[546].value <= threshold=17.11970329284668
node_195: feature_name=GO:0001909 feature_id[386].value <= threshold=7.292566299438477
node_196: feature_name=GO:0003908 feature_id[11].value <= threshold=1.887226164340973
node_197: feature_name=GO:0009164 feature_id[546].value > threshold=0.26642198860645294
node_359: feature_name=GO:0009164 feature_id[546].value > threshold=0.2696942090988159
node_361: feature_name=hsa05202 feature_id[50].value <= threshold=14.989679336547852
node_362: feature_name=GO:0001775 feature_id[505].value <= threshold=15.726949691772461

node_363: feature_name=GO:0002634 feature_id[449].value <= threshold=2.825531005859375
node_364: feature_name=GO:0010038 feature_id[457].value <= threshold=9.901018619537354
node_365: feature_name=GO:0044238 feature_id[295].value > threshold=139.80535888671875
Class: positive genes

Rules_696 passed counts:1
node_0: feature_name=GO:0042113 feature_id[0].value <= threshold=13.408552169799805
node_1: feature_name=GO:0007568 feature_id[534].value <= threshold=5.0313897132873535
node_2: feature_name=GO:0002705 feature_id[541].value <= threshold=3.200145721435547
node_3: feature_name=GO:1901525 feature_id[576].value <= threshold=0.4466460347175598
node_4: feature_name=GO:0048539 feature_id[319].value <= threshold=3.0399646759033203
node_5: feature_name=GO:0001910 feature_id[385].value <= threshold=3.7437864542007446
node_6: feature_name=GO:0043200 feature_id[706].value <= threshold=9.307284355163574
node_7: feature_name=GO:0001773 feature_id[308].value <= threshold=4.016931533813477
node_8: feature_name=GO:0090116 feature_id[97].value <= threshold=7.99645471572876
node_9: feature_name=GO:0019814 feature_id[189].value <= threshold=4.525782108306885
node_10: feature_name=GO:1902583 feature_id[215].value <= threshold=17.60310649871826
node_11: feature_name=GO:0045429 feature_id[737].value <= threshold=1.6133361458778381
node_12: feature_name=GO:0003720 feature_id[228].value <= threshold=5.519326210021973
node_13: feature_name=GO:0046006 feature_id[364].value <= threshold=4.9985432624816895
node_14: feature_name=GO:0070424 feature_id[254].value <= threshold=6.233297109603882
node_15: feature_name=GO:0009892 feature_id[320].value <= threshold=59.99180793762207
node_16: feature_name=GO:0007064 feature_id[527].value <= threshold=11.57377815246582
node_17: feature_name=GO:0005575 feature_id[17].value <= threshold=15.417460918426514
node_18: feature_name=GO:0043368 feature_id[31].value <= threshold=4.059496641159058
node_19: feature_name=GO:0005164 feature_id[163].value <= threshold=5.793607711791992
node_20: feature_name=GO:0042130 feature_id[16].value <= threshold=5.073179721832275
node_21: feature_name=GO:0010216 feature_id[282].value <= threshold=4.834584474563599
node_22: feature_name=GO:0009628 feature_id[553].value <= threshold=29.314892768859863
node_23: feature_name=GO:0045628 feature_id[749].value <= threshold=3.1001367568969727
node_24: feature_name=GO:0042288 feature_id[100].value <= threshold=5.174932956695557
node_25: feature_name=GO:0002329 feature_id[406].value <= threshold=2.348930835723877
node_26: feature_name=GO:0051246 feature_id[642].value > threshold=0.6498909294605255
node_194: feature_name=GO:0009164 feature_id[546].value <= threshold=17.11970329284668
node_195: feature_name=GO:0001909 feature_id[386].value <= threshold=7.292566299438477

node_196: feature_name=GO:0003908 feature_id[11].value <= threshold=1.887226164340973
node_197: feature_name=GO:0009164 feature_id[546].value > threshold=0.26642198860645294
node_359: feature_name=GO:0009164 feature_id[546].value > threshold=0.2696942090988159
node_361: feature_name=hsa05202 feature_id[50].value <= threshold=14.989679336547852
node_362: feature_name=GO:0001775 feature_id[505].value <= threshold=15.726949691772461
node_363: feature_name=GO:0002634 feature_id[449].value <= threshold=2.825531005859375
node_364: feature_name=GO:0010038 feature_id[457].value <= threshold=9.901018619537354
node_365: feature_name=GO:0044238 feature_id[295].value <= threshold=139.80535888671875
node_366: feature_name=GO:0051246 feature_id[642].value > threshold=0.6527281999588013
node_368: feature_name=GO:0044092 feature_id[432].value <= threshold=11.084641933441162
node_369: feature_name=GO:0042162 feature_id[355].value > threshold=9.683155536651611
Class: positive genes

Rules_697 passed counts:1
node_0: feature_name=GO:0042113 feature_id[0].value <= threshold=13.408552169799805
node_1: feature_name=GO:0007568 feature_id[534].value <= threshold=5.0313897132873535
node_2: feature_name=GO:0002705 feature_id[541].value <= threshold=3.200145721435547
node_3: feature_name=GO:1901525 feature_id[576].value <= threshold=0.4466460347175598
node_4: feature_name=GO:0048539 feature_id[319].value <= threshold=3.0399646759033203
node_5: feature_name=GO:0001910 feature_id[385].value <= threshold=3.7437864542007446
node_6: feature_name=GO:0043200 feature_id[706].value <= threshold=9.307284355163574
node_7: feature_name=GO:0001773 feature_id[308].value <= threshold=4.016931533813477
node_8: feature_name=GO:0090116 feature_id[97].value <= threshold=7.99645471572876
node_9: feature_name=GO:0019814 feature_id[189].value <= threshold=4.525782108306885
node_10: feature_name=GO:1902583 feature_id[215].value <= threshold=17.60310649871826
node_11: feature_name=GO:0045429 feature_id[737].value <= threshold=1.6133361458778381
node_12: feature_name=GO:0003720 feature_id[228].value <= threshold=5.519326210021973
node_13: feature_name=GO:0046006 feature_id[364].value <= threshold=4.9985432624816895
node_14: feature_name=GO:0070424 feature_id[254].value <= threshold=6.233297109603882
node_15: feature_name=GO:0009892 feature_id[320].value <= threshold=59.99180793762207
node_16: feature_name=GO:0007064 feature_id[527].value <= threshold=11.57377815246582
node_17: feature_name=GO:0005575 feature_id[17].value <= threshold=15.417460918426514
node_18: feature_name=GO:0043368 feature_id[31].value <= threshold=4.059496641159058
node_19: feature_name=GO:0005164 feature_id[163].value <= threshold=5.793607711791992
node_20: feature_name=GO:0042130 feature_id[16].value <= threshold=5.073179721832275

node_21: feature_name=GO:0010216 feature_id[282].value <= threshold=4.834584474563599
node_22: feature_name=GO:0009628 feature_id[553].value <= threshold=29.314892768859863
node_23: feature_name=GO:0045628 feature_id[749].value <= threshold=3.1001367568969727
node_24: feature_name=GO:0042288 feature_id[100].value <= threshold=5.174932956695557
node_25: feature_name=GO:0002329 feature_id[406].value <= threshold=2.348930835723877
node_26: feature_name=GO:0051246 feature_id[642].value > threshold=0.6498909294605255
node_194: feature_name=GO:0009164 feature_id[546].value <= threshold=17.11970329284668
node_195: feature_name=GO:0001909 feature_id[386].value <= threshold=7.292566299438477
node_196: feature_name=GO:0003908 feature_id[11].value <= threshold=1.887226164340973
node_197: feature_name=GO:0009164 feature_id[546].value > threshold=0.26642198860645294
node_359: feature_name=GO:0009164 feature_id[546].value > threshold=0.2696942090988159
node_361: feature_name=hsa05202 feature_id[50].value <= threshold=14.989679336547852
node_362: feature_name=GO:0001775 feature_id[505].value <= threshold=15.726949691772461
node_363: feature_name=GO:0002634 feature_id[449].value <= threshold=2.825531005859375
node_364: feature_name=GO:0010038 feature_id[457].value <= threshold=9.901018619537354
node_365: feature_name=GO:0044238 feature_id[295].value <= threshold=139.80535888671875
node_366: feature_name=GO:0051246 feature_id[642].value > threshold=0.6527281999588013
node_368: feature_name=GO:0044092 feature_id[432].value <= threshold=11.084641933441162
node_369: feature_name=GO:0042162 feature_id[355].value <= threshold=9.683155536651611
node_370: feature_name=GO:0045945 feature_id[267].value > threshold=3.6181851625442505
Class: positive genes

Rules_698 passed counts:1
node_0: feature_name=GO:0042113 feature_id[0].value <= threshold=13.408552169799805
node_1: feature_name=GO:0007568 feature_id[534].value <= threshold=5.0313897132873535
node_2: feature_name=GO:0002705 feature_id[541].value <= threshold=3.200145721435547
node_3: feature_name=GO:1901525 feature_id[576].value <= threshold=0.4466460347175598
node_4: feature_name=GO:0048539 feature_id[319].value <= threshold=3.0399646759033203
node_5: feature_name=GO:0001910 feature_id[385].value <= threshold=3.7437864542007446
node_6: feature_name=GO:0043200 feature_id[706].value <= threshold=9.307284355163574
node_7: feature_name=GO:0001773 feature_id[308].value <= threshold=4.016931533813477
node_8: feature_name=GO:0090116 feature_id[97].value <= threshold=7.99645471572876
node_9: feature_name=GO:0019814 feature_id[189].value <= threshold=4.525782108306885
node_10: feature_name=GO:1902583 feature_id[215].value <= threshold=17.60310649871826
node_11: feature_name=GO:0045429 feature_id[737].value <= threshold=1.6133361458778381

node_12: feature_name=GO:0003720 feature_id[228].value <= threshold=5.519326210021973
node_13: feature_name=GO:0046006 feature_id[364].value <= threshold=4.9985432624816895
node_14: feature_name=GO:0070424 feature_id[254].value <= threshold=6.233297109603882
node_15: feature_name=GO:0009892 feature_id[320].value <= threshold=59.99180793762207
node_16: feature_name=GO:0007064 feature_id[527].value <= threshold=11.57377815246582
node_17: feature_name=GO:0005575 feature_id[17].value <= threshold=15.417460918426514
node_18: feature_name=GO:0043368 feature_id[31].value <= threshold=4.059496641159058
node_19: feature_name=GO:0005164 feature_id[163].value <= threshold=5.793607711791992
node_20: feature_name=GO:0042130 feature_id[16].value <= threshold=5.073179721832275
node_21: feature_name=GO:0010216 feature_id[282].value <= threshold=4.834584474563599
node_22: feature_name=GO:0009628 feature_id[553].value <= threshold=29.314892768859863
node_23: feature_name=GO:0045628 feature_id[749].value <= threshold=3.1001367568969727
node_24: feature_name=GO:0042288 feature_id[100].value <= threshold=5.174932956695557
node_25: feature_name=GO:0002329 feature_id[406].value <= threshold=2.348930835723877
node_26: feature_name=GO:0051246 feature_id[642].value > threshold=0.6498909294605255
node_194: feature_name=GO:0009164 feature_id[546].value <= threshold=17.11970329284668
node_195: feature_name=GO:0001909 feature_id[386].value <= threshold=7.292566299438477
node_196: feature_name=GO:0003908 feature_id[11].value <= threshold=1.887226164340973
node_197: feature_name=GO:0009164 feature_id[546].value > threshold=0.26642198860645294
node_359: feature_name=GO:0009164 feature_id[546].value > threshold=0.2696942090988159
node_361: feature_name=hsa05202 feature_id[50].value <= threshold=14.989679336547852
node_362: feature_name=GO:0001775 feature_id[505].value <= threshold=15.726949691772461
node_363: feature_name=GO:0002634 feature_id[449].value <= threshold=2.825531005859375
node_364: feature_name=GO:0010038 feature_id[457].value <= threshold=9.901018619537354
node_365: feature_name=GO:0044238 feature_id[295].value <= threshold=139.80535888671875
node_366: feature_name=GO:0051246 feature_id[642].value > threshold=0.6527281999588013
node_368: feature_name=GO:0044092 feature_id[432].value <= threshold=11.084641933441162
node_369: feature_name=GO:0042162 feature_id[355].value <= threshold=9.683155536651611
node_370: feature_name=GO:0045945 feature_id[267].value <= threshold=3.6181851625442505
node_371: feature_name=GO:0036296 feature_id[252].value > threshold=3.214281916618347
Class: positive genes

Rules_699 passed counts:1
node_0: feature_name=GO:0042113 feature_id[0].value <= threshold=13.408552169799805
node_1: feature_name=GO:0007568 feature_id[534].value <= threshold=5.0313897132873535

node_2: feature_name=GO:0002705	feature_id[541].value <= threshold=3.200145721435547
node_3: feature_name=GO:1901525	feature_id[576].value <= threshold=0.4466460347175598
node_4: feature_name=GO:0048539	feature_id[319].value <= threshold=3.0399646759033203
node_5: feature_name=GO:0001910	feature_id[385].value <= threshold=3.7437864542007446
node_6: feature_name=GO:0043200	feature_id[706].value <= threshold=9.307284355163574
node_7: feature_name=GO:0001773	feature_id[308].value <= threshold=4.016931533813477
node_8: feature_name=GO:0090116	feature_id[97].value <= threshold=7.99645471572876
node_9: feature_name=GO:0019814	feature_id[189].value <= threshold=4.525782108306885
node_10: feature_name=GO:1902583	feature_id[215].value <= threshold=17.60310649871826
node_11: feature_name=GO:0045429	feature_id[737].value <= threshold=1.6133361458778381
node_12: feature_name=GO:0003720	feature_id[228].value <= threshold=5.519326210021973
node_13: feature_name=GO:0046006	feature_id[364].value <= threshold=4.9985432624816895
node_14: feature_name=GO:0070424	feature_id[254].value <= threshold=6.233297109603882
node_15: feature_name=GO:0009892	feature_id[320].value <= threshold=59.99180793762207
node_16: feature_name=GO:0007064	feature_id[527].value <= threshold=11.57377815246582
node_17: feature_name=GO:0005575	feature_id[17].value <= threshold=15.417460918426514
node_18: feature_name=GO:0043368	feature_id[31].value <= threshold=4.059496641159058
node_19: feature_name=GO:0005164	feature_id[163].value <= threshold=5.793607711791992
node_20: feature_name=GO:0042130	feature_id[16].value <= threshold=5.073179721832275
node_21: feature_name=GO:0010216	feature_id[282].value <= threshold=4.834584474563599
node_22: feature_name=GO:0009628	feature_id[553].value <= threshold=29.314892768859863
node_23: feature_name=GO:0045628	feature_id[749].value <= threshold=3.1001367568969727
node_24: feature_name=GO:0042288	feature_id[100].value <= threshold=5.174932956695557
node_25: feature_name=GO:0002329	feature_id[406].value <= threshold=2.348930835723877
node_26: feature_name=GO:0051246	feature_id[642].value > threshold=0.6498909294605255
node_194: feature_name=GO:0009164	feature_id[546].value <= threshold=17.11970329284668
node_195: feature_name=GO:0001909	feature_id[386].value <= threshold=7.292566299438477
node_196: feature_name=GO:0003908	feature_id[11].value <= threshold=1.887226164340973
node_197: feature_name=GO:0009164	feature_id[546].value > threshold=0.26642198860645294
node_359: feature_name=GO:0009164	feature_id[546].value > threshold=0.2696942090988159
node_361: feature_name=hsa05202	feature_id[50].value <= threshold=14.989679336547852
node_362: feature_name=GO:0001775	feature_id[505].value <= threshold=15.726949691772461
node_363: feature_name=GO:0002634	feature_id[449].value <= threshold=2.825531005859375
node_364: feature_name=GO:0010038	feature_id[457].value <= threshold=9.901018619537354
node_365: feature_name=GO:0044238	feature_id[295].value <= threshold=139.80535888671875

node_366: feature_name=GO:0051246 feature_id[642].value > threshold=0.6527281999588013
node_368: feature_name=GO:0044092 feature_id[432].value <= threshold=11.084641933441162
node_369: feature_name=GO:0042162 feature_id[355].value <= threshold=9.683155536651611
node_370: feature_name=GO:0045945 feature_id[267].value <= threshold=3.6181851625442505
node_371: feature_name=GO:0036296 feature_id[252].value <= threshold=3.214281916618347
node_372: feature_name=GO:0097506 feature_id[51].value <= threshold=2.9644681215286255
node_373: feature_name=GO:0019740 feature_id[581].value <= threshold=2.2853575944900513
node_374: feature_name=GO:0019660 feature_id[70].value > threshold=1.2831979990005493
node_424: feature_name=GO:0000079 feature_id[287].value > threshold=0.7065127044916153
Class: negative genes

Rules_700 passed counts:1
node_0: feature_name=GO:0042113 feature_id[0].value <= threshold=13.408552169799805
node_1: feature_name=GO:0007568 feature_id[534].value <= threshold=5.0313897132873535
node_2: feature_name=GO:0002705 feature_id[541].value <= threshold=3.200145721435547
node_3: feature_name=GO:1901525 feature_id[576].value <= threshold=0.4466460347175598
node_4: feature_name=GO:0048539 feature_id[319].value <= threshold=3.0399646759033203
node_5: feature_name=GO:0001910 feature_id[385].value <= threshold=3.7437864542007446
node_6: feature_name=GO:0043200 feature_id[706].value <= threshold=9.307284355163574
node_7: feature_name=GO:0001773 feature_id[308].value <= threshold=4.016931533813477
node_8: feature_name=GO:0090116 feature_id[97].value <= threshold=7.99645471572876
node_9: feature_name=GO:0019814 feature_id[189].value <= threshold=4.525782108306885
node_10: feature_name=GO:1902583 feature_id[215].value <= threshold=17.60310649871826
node_11: feature_name=GO:0045429 feature_id[737].value <= threshold=1.6133361458778381
node_12: feature_name=GO:0003720 feature_id[228].value <= threshold=5.519326210021973
node_13: feature_name=GO:0046006 feature_id[364].value <= threshold=4.9985432624816895
node_14: feature_name=GO:0070424 feature_id[254].value <= threshold=6.233297109603882
node_15: feature_name=GO:0009892 feature_id[320].value <= threshold=59.99180793762207
node_16: feature_name=GO:0007064 feature_id[527].value <= threshold=11.57377815246582
node_17: feature_name=GO:0005575 feature_id[17].value <= threshold=15.417460918426514
node_18: feature_name=GO:0043368 feature_id[31].value <= threshold=4.059496641159058
node_19: feature_name=GO:0005164 feature_id[163].value <= threshold=5.793607711791992
node_20: feature_name=GO:0042130 feature_id[16].value <= threshold=5.073179721832275
node_21: feature_name=GO:0010216 feature_id[282].value <= threshold=4.834584474563599
node_22: feature_name=GO:0009628 feature_id[553].value <= threshold=29.314892768859863

node_23: feature_name=GO:0045628 feature_id[749].value <= threshold=3.1001367568969727
node_24: feature_name=GO:0042288 feature_id[100].value <= threshold=5.174932956695557
node_25: feature_name=GO:0002329 feature_id[406].value <= threshold=2.348930835723877
node_26: feature_name=GO:0051246 feature_id[642].value > threshold=0.6498909294605255
node_194: feature_name=GO:0009164 feature_id[546].value <= threshold=17.11970329284668
node_195: feature_name=GO:0001909 feature_id[386].value <= threshold=7.292566299438477
node_196: feature_name=GO:0003908 feature_id[11].value <= threshold=1.887226164340973
node_197: feature_name=GO:0009164 feature_id[546].value > threshold=0.26642198860645294
node_359: feature_name=GO:0009164 feature_id[546].value > threshold=0.2696942090988159
node_361: feature_name=hsa05202 feature_id[50].value <= threshold=14.989679336547852
node_362: feature_name=GO:0001775 feature_id[505].value <= threshold=15.726949691772461
node_363: feature_name=GO:0002634 feature_id[449].value <= threshold=2.825531005859375
node_364: feature_name=GO:0010038 feature_id[457].value <= threshold=9.901018619537354
node_365: feature_name=GO:0044238 feature_id[295].value <= threshold=139.80535888671875
node_366: feature_name=GO:0051246 feature_id[642].value > threshold=0.6527281999588013
node_368: feature_name=GO:0044092 feature_id[432].value <= threshold=11.084641933441162
node_369: feature_name=GO:0042162 feature_id[355].value <= threshold=9.683155536651611
node_370: feature_name=GO:0045945 feature_id[267].value <= threshold=3.6181851625442505
node_371: feature_name=GO:0036296 feature_id[252].value <= threshold=3.214281916618347
node_372: feature_name=GO:0097506 feature_id[51].value <= threshold=2.9644681215286255
node_373: feature_name=GO:0019740 feature_id[581].value <= threshold=2.2853575944900513
node_374: feature_name=GO:0019660 feature_id[70].value > threshold=1.2831979990005493
node_424: feature_name=GO:0000079 feature_id[287].value <= threshold=0.7065127044916153
Class: positive genes

Rules_701 passed counts:1
node_0: feature_name=GO:0042113 feature_id[0].value <= threshold=13.408552169799805
node_1: feature_name=GO:0007568 feature_id[534].value <= threshold=5.0313897132873535
node_2: feature_name=GO:0002705 feature_id[541].value <= threshold=3.200145721435547
node_3: feature_name=GO:1901525 feature_id[576].value <= threshold=0.4466460347175598
node_4: feature_name=GO:0048539 feature_id[319].value <= threshold=3.0399646759033203
node_5: feature_name=GO:0001910 feature_id[385].value <= threshold=3.7437864542007446
node_6: feature_name=GO:0043200 feature_id[706].value <= threshold=9.307284355163574
node_7: feature_name=GO:0001773 feature_id[308].value <= threshold=4.016931533813477
node_8: feature_name=GO:0090116 feature_id[97].value <= threshold=7.99645471572876

node_9: feature_name=GO:0019814	feature_id[189].value <= threshold=4.525782108306885
node_10: feature_name=GO:1902583	feature_id[215].value <= threshold=17.60310649871826
node_11: feature_name=GO:0045429	feature_id[737].value <= threshold=1.6133361458778381
node_12: feature_name=GO:0003720	feature_id[228].value <= threshold=5.519326210021973
node_13: feature_name=GO:0046006	feature_id[364].value <= threshold=4.9985432624816895
node_14: feature_name=GO:0070424	feature_id[254].value <= threshold=6.233297109603882
node_15: feature_name=GO:0009892	feature_id[320].value <= threshold=59.99180793762207
node_16: feature_name=GO:0007064	feature_id[527].value <= threshold=11.57377815246582
node_17: feature_name=GO:0005575	feature_id[17].value <= threshold=15.417460918426514
node_18: feature_name=GO:0043368	feature_id[31].value <= threshold=4.059496641159058
node_19: feature_name=GO:0005164	feature_id[163].value <= threshold=5.793607711791992
node_20: feature_name=GO:0042130	feature_id[16].value <= threshold=5.073179721832275
node_21: feature_name=GO:0010216	feature_id[282].value <= threshold=4.834584474563599
node_22: feature_name=GO:0009628	feature_id[553].value <= threshold=29.314892768859863
node_23: feature_name=GO:0045628	feature_id[749].value <= threshold=3.1001367568969727
node_24: feature_name=GO:0042288	feature_id[100].value <= threshold=5.174932956695557
node_25: feature_name=GO:0002329	feature_id[406].value <= threshold=2.348930835723877
node_26: feature_name=GO:0051246	feature_id[642].value > threshold=0.6498909294605255
node_194: feature_name=GO:0009164	feature_id[546].value <= threshold=17.11970329284668
node_195: feature_name=GO:0001909	feature_id[386].value <= threshold=7.292566299438477
node_196: feature_name=GO:0003908	feature_id[11].value <= threshold=1.887226164340973
node_197: feature_name=GO:0009164	feature_id[546].value > threshold=0.26642198860645294
node_359: feature_name=GO:0009164	feature_id[546].value > threshold=0.2696942090988159
node_361: feature_name=hsa05202	feature_id[50].value <= threshold=14.989679336547852
node_362: feature_name=GO:0001775	feature_id[505].value <= threshold=15.726949691772461
node_363: feature_name=GO:0002634	feature_id[449].value <= threshold=2.825531005859375
node_364: feature_name=GO:0010038	feature_id[457].value <= threshold=9.901018619537354
node_365: feature_name=GO:0044238	feature_id[295].value <= threshold=139.80535888671875
node_366: feature_name=GO:0051246	feature_id[642].value > threshold=0.6527281999588013
node_368: feature_name=GO:0044092	feature_id[432].value <= threshold=11.084641933441162
node_369: feature_name=GO:0042162	feature_id[355].value <= threshold=9.683155536651611
node_370: feature_name=GO:0045945	feature_id[267].value <= threshold=3.6181851625442505
node_371: feature_name=GO:0036296	feature_id[252].value <= threshold=3.214281916618347
node_372: feature_name=GO:0097506	feature_id[51].value <= threshold=2.9644681215286255
node_373: feature_name=GO:0019740	feature_id[581].value <= threshold=2.2853575944900513

node_374: feature_name=GO:0019660 feature_id[70].value <= threshold=1.2831979990005493
node_375: feature_name=GO:0071901 feature_id[407].value > threshold=14.498775482177734
node_421: feature_name=GO:0042542 feature_id[689].value > threshold=0.3278133273124695
Class: positive genes

Rules_702 passed counts:1
node_0: feature_name=GO:0042113 feature_id[0].value <= threshold=13.408552169799805
node_1: feature_name=GO:0007568 feature_id[534].value <= threshold=5.0313897132873535
node_2: feature_name=GO:0002705 feature_id[541].value <= threshold=3.200145721435547
node_3: feature_name=GO:1901525 feature_id[576].value <= threshold=0.4466460347175598
node_4: feature_name=GO:0048539 feature_id[319].value <= threshold=3.0399646759033203
node_5: feature_name=GO:0001910 feature_id[385].value <= threshold=3.7437864542007446
node_6: feature_name=GO:0043200 feature_id[706].value <= threshold=9.307284355163574
node_7: feature_name=GO:0001773 feature_id[308].value <= threshold=4.016931533813477
node_8: feature_name=GO:0090116 feature_id[97].value <= threshold=7.99645471572876
node_9: feature_name=GO:0019814 feature_id[189].value <= threshold=4.525782108306885
node_10: feature_name=GO:1902583 feature_id[215].value <= threshold=17.60310649871826
node_11: feature_name=GO:0045429 feature_id[737].value <= threshold=1.6133361458778381
node_12: feature_name=GO:0003720 feature_id[228].value <= threshold=5.519326210021973
node_13: feature_name=GO:0046006 feature_id[364].value <= threshold=4.9985432624816895
node_14: feature_name=GO:0070424 feature_id[254].value <= threshold=6.233297109603882
node_15: feature_name=GO:0009892 feature_id[320].value <= threshold=59.99180793762207
node_16: feature_name=GO:0007064 feature_id[527].value <= threshold=11.57377815246582
node_17: feature_name=GO:0005575 feature_id[17].value <= threshold=15.417460918426514
node_18: feature_name=GO:0043368 feature_id[31].value <= threshold=4.059496641159058
node_19: feature_name=GO:0005164 feature_id[163].value <= threshold=5.793607711791992
node_20: feature_name=GO:0042130 feature_id[16].value <= threshold=5.073179721832275
node_21: feature_name=GO:0010216 feature_id[282].value <= threshold=4.834584474563599
node_22: feature_name=GO:0009628 feature_id[553].value <= threshold=29.314892768859863
node_23: feature_name=GO:0045628 feature_id[749].value <= threshold=3.1001367568969727
node_24: feature_name=GO:0042288 feature_id[100].value <= threshold=5.174932956695557
node_25: feature_name=GO:0002329 feature_id[406].value <= threshold=2.348930835723877
node_26: feature_name=GO:0051246 feature_id[642].value > threshold=0.6498909294605255
node_194: feature_name=GO:0009164 feature_id[546].value <= threshold=17.11970329284668
node_195: feature_name=GO:0001909 feature_id[386].value <= threshold=7.292566299438477

node_196: feature_name=GO:0003908 feature_id[11].value <= threshold=1.887226164340973
node_197: feature_name=GO:0009164 feature_id[546].value > threshold=0.26642198860645294
node_359: feature_name=GO:0009164 feature_id[546].value > threshold=0.2696942090988159
node_361: feature_name=hsa05202 feature_id[50].value <= threshold=14.989679336547852
node_362: feature_name=GO:0001775 feature_id[505].value <= threshold=15.726949691772461
node_363: feature_name=GO:0002634 feature_id[449].value <= threshold=2.825531005859375
node_364: feature_name=GO:0010038 feature_id[457].value <= threshold=9.901018619537354
node_365: feature_name=GO:0044238 feature_id[295].value <= threshold=139.80535888671875
node_366: feature_name=GO:0051246 feature_id[642].value > threshold=0.6527281999588013
node_368: feature_name=GO:0044092 feature_id[432].value <= threshold=11.084641933441162
node_369: feature_name=GO:0042162 feature_id[355].value <= threshold=9.683155536651611
node_370: feature_name=GO:0045945 feature_id[267].value <= threshold=3.6181851625442505
node_371: feature_name=GO:0036296 feature_id[252].value <= threshold=3.214281916618347
node_372: feature_name=GO:0097506 feature_id[51].value <= threshold=2.9644681215286255
node_373: feature_name=GO:0019740 feature_id[581].value <= threshold=2.2853575944900513
node_374: feature_name=GO:0019660 feature_id[70].value <= threshold=1.2831979990005493
node_375: feature_name=GO:0071901 feature_id[407].value > threshold=14.498775482177734
node_421: feature_name=GO:0042542 feature_id[689].value <= threshold=0.3278133273124695
Class: negative genes

Rules_703 passed counts:1
node_0: feature_name=GO:0042113 feature_id[0].value <= threshold=13.408552169799805
node_1: feature_name=GO:0007568 feature_id[534].value <= threshold=5.0313897132873535
node_2: feature_name=GO:0002705 feature_id[541].value <= threshold=3.200145721435547
node_3: feature_name=GO:1901525 feature_id[576].value <= threshold=0.4466460347175598
node_4: feature_name=GO:0048539 feature_id[319].value <= threshold=3.0399646759033203
node_5: feature_name=GO:0001910 feature_id[385].value <= threshold=3.7437864542007446
node_6: feature_name=GO:0043200 feature_id[706].value <= threshold=9.307284355163574
node_7: feature_name=GO:0001773 feature_id[308].value <= threshold=4.016931533813477
node_8: feature_name=GO:0090116 feature_id[97].value <= threshold=7.99645471572876
node_9: feature_name=GO:0019814 feature_id[189].value <= threshold=4.525782108306885
node_10: feature_name=GO:1902583 feature_id[215].value <= threshold=17.60310649871826
node_11: feature_name=GO:0045429 feature_id[737].value <= threshold=1.6133361458778381
node_12: feature_name=GO:0003720 feature_id[228].value <= threshold=5.519326210021973
node_13: feature_name=GO:0046006 feature_id[364].value <= threshold=4.9985432624816895

node_14: feature_name=GO:0070424	feature_id[254].value <= threshold=6.233297109603882
node_15: feature_name=GO:0009892	feature_id[320].value <= threshold=59.99180793762207
node_16: feature_name=GO:0007064	feature_id[527].value <= threshold=11.57377815246582
node_17: feature_name=GO:0005575	feature_id[17].value <= threshold=15.417460918426514
node_18: feature_name=GO:0043368	feature_id[31].value <= threshold=4.059496641159058
node_19: feature_name=GO:0005164	feature_id[163].value <= threshold=5.793607711791992
node_20: feature_name=GO:0042130	feature_id[16].value <= threshold=5.073179721832275
node_21: feature_name=GO:0010216	feature_id[282].value <= threshold=4.834584474563599
node_22: feature_name=GO:0009628	feature_id[553].value <= threshold=29.314892768859863
node_23: feature_name=GO:0045628	feature_id[749].value <= threshold=3.1001367568969727
node_24: feature_name=GO:0042288	feature_id[100].value <= threshold=5.174932956695557
node_25: feature_name=GO:0002329	feature_id[406].value <= threshold=2.348930835723877
node_26: feature_name=GO:0051246	feature_id[642].value > threshold=0.6498909294605255
node_194: feature_name=GO:0009164	feature_id[546].value <= threshold=17.11970329284668
node_195: feature_name=GO:0001909	feature_id[386].value <= threshold=7.292566299438477
node_196: feature_name=GO:0003908	feature_id[11].value <= threshold=1.887226164340973
node_197: feature_name=GO:0009164	feature_id[546].value > threshold=0.26642198860645294
node_359: feature_name=GO:0009164	feature_id[546].value > threshold=0.2696942090988159
node_361: feature_name=hsa05202	feature_id[50].value <= threshold=14.989679336547852
node_362: feature_name=GO:0001775	feature_id[505].value <= threshold=15.726949691772461
node_363: feature_name=GO:0002634	feature_id[449].value <= threshold=2.825531005859375
node_364: feature_name=GO:0010038	feature_id[457].value <= threshold=9.901018619537354
node_365: feature_name=GO:0044238	feature_id[295].value <= threshold=139.80535888671875
node_366: feature_name=GO:0051246	feature_id[642].value > threshold=0.6527281999588013
node_368: feature_name=GO:0044092	feature_id[432].value <= threshold=11.084641933441162
node_369: feature_name=GO:0042162	feature_id[355].value <= threshold=9.683155536651611
node_370: feature_name=GO:0045945	feature_id[267].value <= threshold=3.6181851625442505
node_371: feature_name=GO:0036296	feature_id[252].value <= threshold=3.214281916618347
node_372: feature_name=GO:0097506	feature_id[51].value <= threshold=2.9644681215286255
node_373: feature_name=GO:0019740	feature_id[581].value <= threshold=2.2853575944900513
node_374: feature_name=GO:0019660	feature_id[70].value <= threshold=1.2831979990005493
node_375: feature_name=GO:0071901	feature_id[407].value <= threshold=14.498775482177734
node_376: feature_name=GO:0043170	feature_id[467].value > threshold=4.473330818655086e-06
node_380: feature_name=GO:0046718	feature_id[781].value <= threshold=3.494875192642212
node_381: feature_name=GO:0030983	feature_id[291].value <= threshold=10.994179248809814

node_382: feature_name=GO:0010663 feature_id[561].value > threshold=2.7056833505630493
node_412: feature_name=GO:0034612 feature_id[656].value > threshold=0.6953948438167572
Class: positive genes

Rules_704 passed counts:1
node_0: feature_name=GO:0042113 feature_id[0].value <= threshold=13.408552169799805
node_1: feature_name=GO:0007568 feature_id[534].value <= threshold=5.0313897132873535
node_2: feature_name=GO:0002705 feature_id[541].value <= threshold=3.200145721435547
node_3: feature_name=GO:1901525 feature_id[576].value <= threshold=0.4466460347175598
node_4: feature_name=GO:0048539 feature_id[319].value <= threshold=3.0399646759033203
node_5: feature_name=GO:0001910 feature_id[385].value <= threshold=3.7437864542007446
node_6: feature_name=GO:0043200 feature_id[706].value <= threshold=9.307284355163574
node_7: feature_name=GO:0001773 feature_id[308].value <= threshold=4.016931533813477
node_8: feature_name=GO:0090116 feature_id[97].value <= threshold=7.99645471572876
node_9: feature_name=GO:0019814 feature_id[189].value <= threshold=4.525782108306885
node_10: feature_name=GO:1902583 feature_id[215].value <= threshold=17.60310649871826
node_11: feature_name=GO:0045429 feature_id[737].value <= threshold=1.6133361458778381
node_12: feature_name=GO:0003720 feature_id[228].value <= threshold=5.519326210021973
node_13: feature_name=GO:0046006 feature_id[364].value <= threshold=4.9985432624816895
node_14: feature_name=GO:0070424 feature_id[254].value <= threshold=6.233297109603882
node_15: feature_name=GO:0009892 feature_id[320].value <= threshold=59.99180793762207
node_16: feature_name=GO:0007064 feature_id[527].value <= threshold=11.57377815246582
node_17: feature_name=GO:0005575 feature_id[17].value <= threshold=15.417460918426514
node_18: feature_name=GO:0043368 feature_id[31].value <= threshold=4.059496641159058
node_19: feature_name=GO:0005164 feature_id[163].value <= threshold=5.793607711791992
node_20: feature_name=GO:0042130 feature_id[16].value <= threshold=5.073179721832275
node_21: feature_name=GO:0010216 feature_id[282].value <= threshold=4.834584474563599
node_22: feature_name=GO:0009628 feature_id[553].value <= threshold=29.314892768859863
node_23: feature_name=GO:0045628 feature_id[749].value <= threshold=3.1001367568969727
node_24: feature_name=GO:0042288 feature_id[100].value <= threshold=5.174932956695557
node_25: feature_name=GO:0002329 feature_id[406].value <= threshold=2.348930835723877
node_26: feature_name=GO:0051246 feature_id[642].value > threshold=0.6498909294605255
node_194: feature_name=GO:0009164 feature_id[546].value <= threshold=17.11970329284668
node_195: feature_name=GO:0001909 feature_id[386].value <= threshold=7.292566299438477
node_196: feature_name=GO:0003908 feature_id[11].value <= threshold=1.887226164340973

node_197: feature_name=GO:0009164	feature_id[546].value > threshold=0.26642198860645294
node_359: feature_name=GO:0009164	feature_id[546].value > threshold=0.2696942090988159
node_361: feature_name=hsa05202	feature_id[50].value <= threshold=14.989679336547852
node_362: feature_name=GO:0001775	feature_id[505].value <= threshold=15.726949691772461
node_363: feature_name=GO:0002634	feature_id[449].value <= threshold=2.825531005859375
node_364: feature_name=GO:0010038	feature_id[457].value <= threshold=9.901018619537354
node_365: feature_name=GO:0044238	feature_id[295].value <= threshold=139.80535888671875
node_366: feature_name=GO:0051246	feature_id[642].value > threshold=0.6527281999588013
node_368: feature_name=GO:0044092	feature_id[432].value <= threshold=11.084641933441162
node_369: feature_name=GO:0042162	feature_id[355].value <= threshold=9.683155536651611
node_370: feature_name=GO:0045945	feature_id[267].value <= threshold=3.6181851625442505
node_371: feature_name=GO:0036296	feature_id[252].value <= threshold=3.214281916618347
node_372: feature_name=GO:0097506	feature_id[51].value <= threshold=2.9644681215286255
node_373: feature_name=GO:0019740	feature_id[581].value <= threshold=2.2853575944900513
node_374: feature_name=GO:0019660	feature_id[70].value <= threshold=1.2831979990005493
node_375: feature_name=GO:0071901	feature_id[407].value <= threshold=14.498775482177734
node_376: feature_name=GO:0043170	feature_id[467].value > threshold=4.473330818655086e-06
node_380: feature_name=GO:0046718	feature_id[781].value <= threshold=3.494875192642212
node_381: feature_name=GO:0030983	feature_id[291].value <= threshold=10.994179248809814
node_382: feature_name=GO:0010663	feature_id[561].value <= threshold=2.7056833505630493
node_383: feature_name=GO:0031667	feature_id[648].value > threshold=0.0020462179090827703
node_387: feature_name=GO:1990572	feature_id[243].value > threshold=1.9359037280082703
node_409: feature_name=GO:0050866	feature_id[338].value > threshold=0.9498499482870102
Class: positive genes	

Rules_705	passed counts:1
node_0: feature_name=GO:0042113	feature_id[0].value <= threshold=13.408552169799805
node_1: feature_name=GO:0007568	feature_id[534].value <= threshold=5.0313897132873535
node_2: feature_name=GO:0002705	feature_id[541].value <= threshold=3.200145721435547
node_3: feature_name=GO:1901525	feature_id[576].value <= threshold=0.4466460347175598
node_4: feature_name=GO:0048539	feature_id[319].value <= threshold=3.0399646759033203
node_5: feature_name=GO:0001910	feature_id[385].value <= threshold=3.7437864542007446
node_6: feature_name=GO:0043200	feature_id[706].value <= threshold=9.307284355163574
node_7: feature_name=GO:0001773	feature_id[308].value <= threshold=4.016931533813477
node_8: feature_name=GO:0090116	feature_id[97].value <= threshold=7.99645471572876

node_9: feature_name=GO:0019814	feature_id[189].value <= threshold=4.525782108306885
node_10: feature_name=GO:1902583	feature_id[215].value <= threshold=17.60310649871826
node_11: feature_name=GO:0045429	feature_id[737].value <= threshold=1.6133361458778381
node_12: feature_name=GO:0003720	feature_id[228].value <= threshold=5.519326210021973
node_13: feature_name=GO:0046006	feature_id[364].value <= threshold=4.9985432624816895
node_14: feature_name=GO:0070424	feature_id[254].value <= threshold=6.233297109603882
node_15: feature_name=GO:0009892	feature_id[320].value <= threshold=59.99180793762207
node_16: feature_name=GO:0007064	feature_id[527].value <= threshold=11.57377815246582
node_17: feature_name=GO:0005575	feature_id[17].value <= threshold=15.417460918426514
node_18: feature_name=GO:0043368	feature_id[31].value <= threshold=4.059496641159058
node_19: feature_name=GO:0005164	feature_id[163].value <= threshold=5.793607711791992
node_20: feature_name=GO:0042130	feature_id[16].value <= threshold=5.073179721832275
node_21: feature_name=GO:0010216	feature_id[282].value <= threshold=4.834584474563599
node_22: feature_name=GO:0009628	feature_id[553].value <= threshold=29.314892768859863
node_23: feature_name=GO:0045628	feature_id[749].value <= threshold=3.1001367568969727
node_24: feature_name=GO:0042288	feature_id[100].value <= threshold=5.174932956695557
node_25: feature_name=GO:0002329	feature_id[406].value <= threshold=2.348930835723877
node_26: feature_name=GO:0051246	feature_id[642].value > threshold=0.6498909294605255
node_194: feature_name=GO:0009164	feature_id[546].value <= threshold=17.11970329284668
node_195: feature_name=GO:0001909	feature_id[386].value <= threshold=7.292566299438477
node_196: feature_name=GO:0003908	feature_id[11].value <= threshold=1.887226164340973
node_197: feature_name=GO:0009164	feature_id[546].value > threshold=0.26642198860645294
node_359: feature_name=GO:0009164	feature_id[546].value > threshold=0.2696942090988159
node_361: feature_name=hsa05202	feature_id[50].value <= threshold=14.989679336547852
node_362: feature_name=GO:0001775	feature_id[505].value <= threshold=15.726949691772461
node_363: feature_name=GO:0002634	feature_id[449].value <= threshold=2.825531005859375
node_364: feature_name=GO:0010038	feature_id[457].value <= threshold=9.901018619537354
node_365: feature_name=GO:0044238	feature_id[295].value <= threshold=139.80535888671875
node_366: feature_name=GO:0051246	feature_id[642].value > threshold=0.6527281999588013
node_368: feature_name=GO:0044092	feature_id[432].value <= threshold=11.084641933441162
node_369: feature_name=GO:0042162	feature_id[355].value <= threshold=9.683155536651611
node_370: feature_name=GO:0045945	feature_id[267].value <= threshold=3.6181851625442505
node_371: feature_name=GO:0036296	feature_id[252].value <= threshold=3.214281916618347
node_372: feature_name=GO:0097506	feature_id[51].value <= threshold=2.9644681215286255
node_373: feature_name=GO:0019740	feature_id[581].value <= threshold=2.2853575944900513

node_374: feature_name=GO:0019660 feature_id[70].value <= threshold=1.2831979990005493
node_375: feature_name=GO:0071901 feature_id[407].value <= threshold=14.498775482177734
node_376: feature_name=GO:0043170 feature_id[467].value > threshold=4.473330818655086e-06
node_380: feature_name=GO:0046718 feature_id[781].value <= threshold=3.494875192642212
node_381: feature_name=GO:0030983 feature_id[291].value <= threshold=10.994179248809814
node_382: feature_name=GO:0010663 feature_id[561].value <= threshold=2.7056833505630493
node_383: feature_name=GO:0031667 feature_id[648].value > threshold=0.0020462179090827703
node_387: feature_name=GO:1990572 feature_id[243].value <= threshold=1.9359037280082703
node_388: feature_name=GO:0032753 feature_id[101].value > threshold=1.182796835899353
node_404: feature_name=GO:0002449 feature_id[427].value > threshold=0.09284071624279022
node_406: feature_name=GO:0097506 feature_id[51].value > threshold=0.9462986290454865
Class: positive genes

Rules_706 passed counts:1
node_0: feature_name=GO:0042113 feature_id[0].value <= threshold=13.408552169799805
node_1: feature_name=GO:0007568 feature_id[534].value <= threshold=5.0313897132873535
node_2: feature_name=GO:0002705 feature_id[541].value <= threshold=3.200145721435547
node_3: feature_name=GO:1901525 feature_id[576].value <= threshold=0.4466460347175598
node_4: feature_name=GO:0048539 feature_id[319].value <= threshold=3.0399646759033203
node_5: feature_name=GO:0001910 feature_id[385].value <= threshold=3.7437864542007446
node_6: feature_name=GO:0043200 feature_id[706].value <= threshold=9.307284355163574
node_7: feature_name=GO:0001773 feature_id[308].value <= threshold=4.016931533813477
node_8: feature_name=GO:0090116 feature_id[97].value <= threshold=7.99645471572876
node_9: feature_name=GO:0019814 feature_id[189].value <= threshold=4.525782108306885
node_10: feature_name=GO:1902583 feature_id[215].value <= threshold=17.60310649871826
node_11: feature_name=GO:0045429 feature_id[737].value <= threshold=1.6133361458778381
node_12: feature_name=GO:0003720 feature_id[228].value <= threshold=5.519326210021973
node_13: feature_name=GO:0046006 feature_id[364].value <= threshold=4.9985432624816895
node_14: feature_name=GO:0070424 feature_id[254].value <= threshold=6.233297109603882
node_15: feature_name=GO:0009892 feature_id[320].value <= threshold=59.99180793762207
node_16: feature_name=GO:0007064 feature_id[527].value <= threshold=11.57377815246582
node_17: feature_name=GO:0005575 feature_id[17].value <= threshold=15.417460918426514
node_18: feature_name=GO:0043368 feature_id[31].value <= threshold=4.059496641159058
node_19: feature_name=GO:0005164 feature_id[163].value <= threshold=5.793607711791992
node_20: feature_name=GO:0042130 feature_id[16].value <= threshold=5.073179721832275

node_21: feature_name=GO:0010216 feature_id[282].value <= threshold=4.834584474563599
node_22: feature_name=GO:0009628 feature_id[553].value <= threshold=29.314892768859863
node_23: feature_name=GO:0045628 feature_id[749].value <= threshold=3.1001367568969727
node_24: feature_name=GO:0042288 feature_id[100].value <= threshold=5.174932956695557
node_25: feature_name=GO:0002329 feature_id[406].value <= threshold=2.348930835723877
node_26: feature_name=GO:0051246 feature_id[642].value > threshold=0.6498909294605255
node_194: feature_name=GO:0009164 feature_id[546].value <= threshold=17.11970329284668
node_195: feature_name=GO:0001909 feature_id[386].value <= threshold=7.292566299438477
node_196: feature_name=GO:0003908 feature_id[11].value <= threshold=1.887226164340973
node_197: feature_name=GO:0009164 feature_id[546].value > threshold=0.26642198860645294
node_359: feature_name=GO:0009164 feature_id[546].value > threshold=0.2696942090988159
node_361: feature_name=hsa05202 feature_id[50].value <= threshold=14.989679336547852
node_362: feature_name=GO:0001775 feature_id[505].value <= threshold=15.726949691772461
node_363: feature_name=GO:0002634 feature_id[449].value <= threshold=2.825531005859375
node_364: feature_name=GO:0010038 feature_id[457].value <= threshold=9.901018619537354
node_365: feature_name=GO:0044238 feature_id[295].value <= threshold=139.80535888671875
node_366: feature_name=GO:0051246 feature_id[642].value > threshold=0.6527281999588013
node_368: feature_name=GO:0044092 feature_id[432].value <= threshold=11.084641933441162
node_369: feature_name=GO:0042162 feature_id[355].value <= threshold=9.683155536651611
node_370: feature_name=GO:0045945 feature_id[267].value <= threshold=3.6181851625442505
node_371: feature_name=GO:0036296 feature_id[252].value <= threshold=3.214281916618347
node_372: feature_name=GO:0097506 feature_id[51].value <= threshold=2.9644681215286255
node_373: feature_name=GO:0019740 feature_id[581].value <= threshold=2.2853575944900513
node_374: feature_name=GO:0019660 feature_id[70].value <= threshold=1.2831979990005493
node_375: feature_name=GO:0071901 feature_id[407].value <= threshold=14.498775482177734
node_376: feature_name=GO:0043170 feature_id[467].value > threshold=4.473330818655086e-06
node_380: feature_name=GO:0046718 feature_id[781].value <= threshold=3.494875192642212
node_381: feature_name=GO:0030983 feature_id[291].value <= threshold=10.994179248809814
node_382: feature_name=GO:0010663 feature_id[561].value <= threshold=2.7056833505630493
node_383: feature_name=GO:0031667 feature_id[648].value > threshold=0.0020462179090827703
node_387: feature_name=GO:1990572 feature_id[243].value <= threshold=1.9359037280082703
node_388: feature_name=GO:0032753 feature_id[101].value <= threshold=1.182796835899353
node_389: feature_name=GO:0097153 feature_id[226].value > threshold=0.34601132571697235
node_399: feature_name=GO:0036294 feature_id[612].value > threshold=0.022128281882032752
node_401: feature_name=GO:0097153 feature_id[226].value <= threshold=0.40342725813388824

Class: positive genes

Rules_707	passed counts:1
node_0: feature_name=GO:0042113	feature_id[0].value <= threshold=13.408552169799805
node_1: feature_name=GO:0007568	feature_id[534].value <= threshold=5.0313897132873535
node_2: feature_name=GO:0002705	feature_id[541].value <= threshold=3.200145721435547
node_3: feature_name=GO:1901525	feature_id[576].value <= threshold=0.4466460347175598
node_4: feature_name=GO:0048539	feature_id[319].value <= threshold=3.0399646759033203
node_5: feature_name=GO:0001910	feature_id[385].value <= threshold=3.7437864542007446
node_6: feature_name=GO:0043200	feature_id[706].value <= threshold=9.307284355163574
node_7: feature_name=GO:0001773	feature_id[308].value <= threshold=4.016931533813477
node_8: feature_name=GO:0090116	feature_id[97].value <= threshold=7.99645471572876
node_9: feature_name=GO:0019814	feature_id[189].value <= threshold=4.525782108306885
node_10: feature_name=GO:1902583	feature_id[215].value <= threshold=17.60310649871826
node_11: feature_name=GO:0045429	feature_id[737].value <= threshold=1.6133361458778381
node_12: feature_name=GO:0003720	feature_id[228].value <= threshold=5.519326210021973
node_13: feature_name=GO:0046006	feature_id[364].value <= threshold=4.9985432624816895
node_14: feature_name=GO:0070424	feature_id[254].value <= threshold=6.233297109603882
node_15: feature_name=GO:0009892	feature_id[320].value <= threshold=59.99180793762207
node_16: feature_name=GO:0007064	feature_id[527].value <= threshold=11.57377815246582
node_17: feature_name=GO:0005575	feature_id[17].value <= threshold=15.417460918426514
node_18: feature_name=GO:0043368	feature_id[31].value <= threshold=4.059496641159058
node_19: feature_name=GO:0005164	feature_id[163].value <= threshold=5.793607711791992
node_20: feature_name=GO:0042130	feature_id[16].value <= threshold=5.073179721832275
node_21: feature_name=GO:0010216	feature_id[282].value <= threshold=4.834584474563599
node_22: feature_name=GO:0009628	feature_id[553].value <= threshold=29.314892768859863
node_23: feature_name=GO:0045628	feature_id[749].value <= threshold=3.1001367568969727
node_24: feature_name=GO:0042288	feature_id[100].value <= threshold=5.174932956695557
node_25: feature_name=GO:0002329	feature_id[406].value <= threshold=2.348930835723877
node_26: feature_name=GO:0051246	feature_id[642].value > threshold=0.6498909294605255
node_194: feature_name=GO:0009164	feature_id[546].value <= threshold=17.11970329284668
node_195: feature_name=GO:0001909	feature_id[386].value <= threshold=7.292566299438477
node_196: feature_name=GO:0003908	feature_id[11].value <= threshold=1.887226164340973
node_197: feature_name=GO:0009164	feature_id[546].value > threshold=0.26642198860645294
node_359: feature_name=GO:0009164	feature_id[546].value > threshold=0.2696942090988159

node_361: feature_name=hsa05202 feature_id[50].value <= threshold=14.989679336547852
node_362: feature_name=GO:0001775 feature_id[505].value <= threshold=15.726949691772461
node_363: feature_name=GO:0002634 feature_id[449].value <= threshold=2.825531005859375
node_364: feature_name=GO:0010038 feature_id[457].value <= threshold=9.901018619537354
node_365: feature_name=GO:0044238 feature_id[295].value <= threshold=139.80535888671875
node_366: feature_name=GO:0051246 feature_id[642].value > threshold=0.6527281999588013
node_368: feature_name=GO:0044092 feature_id[432].value <= threshold=11.084641933441162
node_369: feature_name=GO:0042162 feature_id[355].value <= threshold=9.683155536651611
node_370: feature_name=GO:0045945 feature_id[267].value <= threshold=3.6181851625442505
node_371: feature_name=GO:0036296 feature_id[252].value <= threshold=3.214281916618347
node_372: feature_name=GO:0097506 feature_id[51].value <= threshold=2.9644681215286255
node_373: feature_name=GO:0019740 feature_id[581].value <= threshold=2.2853575944900513
node_374: feature_name=GO:0019660 feature_id[70].value <= threshold=1.2831979990005493
node_375: feature_name=GO:0071901 feature_id[407].value <= threshold=14.498775482177734
node_376: feature_name=GO:0043170 feature_id[467].value > threshold=4.473330818655086e-06
node_380: feature_name=GO:0046718 feature_id[781].value <= threshold=3.494875192642212
node_381: feature_name=GO:0030983 feature_id[291].value <= threshold=10.994179248809814
node_382: feature_name=GO:0010663 feature_id[561].value <= threshold=2.7056833505630493
node_383: feature_name=GO:0031667 feature_id[648].value > threshold=0.0020462179090827703
node_387: feature_name=GO:1990572 feature_id[243].value <= threshold=1.9359037280082703
node_388: feature_name=GO:0032753 feature_id[101].value <= threshold=1.182796835899353
node_389: feature_name=GO:0097153 feature_id[226].value <= threshold=0.34601132571697235
node_390: feature_name=GO:0002829 feature_id[493].value > threshold=1.246164083480835
node_396: feature_name=GO:0032649 feature_id[626].value <= threshold=0.03106011636555195
Class: positive genes

Rules_708 passed counts:1
node_0: feature_name=GO:0042113 feature_id[0].value <= threshold=13.408552169799805
node_1: feature_name=GO:0007568 feature_id[534].value <= threshold=5.0313897132873535
node_2: feature_name=GO:0002705 feature_id[541].value <= threshold=3.200145721435547
node_3: feature_name=GO:1901525 feature_id[576].value <= threshold=0.4466460347175598
node_4: feature_name=GO:0048539 feature_id[319].value <= threshold=3.0399646759033203
node_5: feature_name=GO:0001910 feature_id[385].value <= threshold=3.7437864542007446
node_6: feature_name=GO:0043200 feature_id[706].value <= threshold=9.307284355163574
node_7: feature_name=GO:0001773 feature_id[308].value <= threshold=4.016931533813477

node_8: feature_name=GO:0090116	feature_id[97].value <= threshold=7.99645471572876
node_9: feature_name=GO:0019814	feature_id[189].value <= threshold=4.525782108306885
node_10: feature_name=GO:1902583	feature_id[215].value <= threshold=17.60310649871826
node_11: feature_name=GO:0045429	feature_id[737].value <= threshold=1.6133361458778381
node_12: feature_name=GO:0003720	feature_id[228].value <= threshold=5.519326210021973
node_13: feature_name=GO:0046006	feature_id[364].value <= threshold=4.9985432624816895
node_14: feature_name=GO:0070424	feature_id[254].value <= threshold=6.233297109603882
node_15: feature_name=GO:0009892	feature_id[320].value <= threshold=59.99180793762207
node_16: feature_name=GO:0007064	feature_id[527].value <= threshold=11.57377815246582
node_17: feature_name=GO:0005575	feature_id[17].value <= threshold=15.417460918426514
node_18: feature_name=GO:0043368	feature_id[31].value <= threshold=4.059496641159058
node_19: feature_name=GO:0005164	feature_id[163].value <= threshold=5.793607711791992
node_20: feature_name=GO:0042130	feature_id[16].value <= threshold=5.073179721832275
node_21: feature_name=GO:0010216	feature_id[282].value <= threshold=4.834584474563599
node_22: feature_name=GO:0009628	feature_id[553].value <= threshold=29.314892768859863
node_23: feature_name=GO:0045628	feature_id[749].value <= threshold=3.1001367568969727
node_24: feature_name=GO:0042288	feature_id[100].value <= threshold=5.174932956695557
node_25: feature_name=GO:0002329	feature_id[406].value <= threshold=2.348930835723877
node_26: feature_name=GO:0051246	feature_id[642].value > threshold=0.6498909294605255
node_194: feature_name=GO:0009164	feature_id[546].value <= threshold=17.11970329284668
node_195: feature_name=GO:0001909	feature_id[386].value <= threshold=7.292566299438477
node_196: feature_name=GO:0003908	feature_id[11].value <= threshold=1.887226164340973
node_197: feature_name=GO:0009164	feature_id[546].value > threshold=0.26642198860645294
node_359: feature_name=GO:0009164	feature_id[546].value > threshold=0.2696942090988159
node_361: feature_name=hsa05202	feature_id[50].value <= threshold=14.989679336547852
node_362: feature_name=GO:0001775	feature_id[505].value <= threshold=15.726949691772461
node_363: feature_name=GO:0002634	feature_id[449].value <= threshold=2.825531005859375
node_364: feature_name=GO:0010038	feature_id[457].value <= threshold=9.901018619537354
node_365: feature_name=GO:0044238	feature_id[295].value <= threshold=139.80535888671875
node_366: feature_name=GO:0051246	feature_id[642].value > threshold=0.6527281999588013
node_368: feature_name=GO:0044092	feature_id[432].value <= threshold=11.084641933441162
node_369: feature_name=GO:0042162	feature_id[355].value <= threshold=9.683155536651611
node_370: feature_name=GO:0045945	feature_id[267].value <= threshold=3.6181851625442505
node_371: feature_name=GO:0036296	feature_id[252].value <= threshold=3.214281916618347
node_372: feature_name=GO:0097506	feature_id[51].value <= threshold=2.9644681215286255

node_373: feature_name=GO:0019740 feature_id[581].value <= threshold=2.2853575944900513
node_374: feature_name=GO:0019660 feature_id[70].value <= threshold=1.2831979990005493
node_375: feature_name=GO:0071901 feature_id[407].value <= threshold=14.498775482177734
node_376: feature_name=GO:0043170 feature_id[467].value > threshold=4.473330818655086e-06
node_380: feature_name=GO:0046718 feature_id[781].value <= threshold=3.494875192642212
node_381: feature_name=GO:0030983 feature_id[291].value <= threshold=10.994179248809814
node_382: feature_name=GO:0010663 feature_id[561].value <= threshold=2.7056833505630493
node_383: feature_name=GO:0031667 feature_id[648].value > threshold=0.0020462179090827703
node_387: feature_name=GO:1990572 feature_id[243].value <= threshold=1.9359037280082703
node_388: feature_name=GO:0032753 feature_id[101].value <= threshold=1.182796835899353
node_389: feature_name=GO:0097153 feature_id[226].value <= threshold=0.34601132571697235
node_390: feature_name=GO:0002829 feature_id[493].value <= threshold=1.246164083480835
node_391: feature_name=GO:0006346 feature_id[236].value > threshold=2.040414571762085
node_393: feature_name=GO:0002821 feature_id[588].value > threshold=0.7741671800613403
Class: positive genes

Rules_709 passed counts:1
node_0: feature_name=GO:0042113 feature_id[0].value <= threshold=13.408552169799805
node_1: feature_name=GO:0007568 feature_id[534].value <= threshold=5.0313897132873535
node_2: feature_name=GO:0002705 feature_id[541].value <= threshold=3.200145721435547
node_3: feature_name=GO:1901525 feature_id[576].value <= threshold=0.4466460347175598
node_4: feature_name=GO:0048539 feature_id[319].value <= threshold=3.0399646759033203
node_5: feature_name=GO:0001910 feature_id[385].value <= threshold=3.7437864542007446
node_6: feature_name=GO:0043200 feature_id[706].value <= threshold=9.307284355163574
node_7: feature_name=GO:0001773 feature_id[308].value <= threshold=4.016931533813477
node_8: feature_name=GO:0090116 feature_id[97].value <= threshold=7.99645471572876
node_9: feature_name=GO:0019814 feature_id[189].value <= threshold=4.525782108306885
node_10: feature_name=GO:1902583 feature_id[215].value <= threshold=17.60310649871826
node_11: feature_name=GO:0045429 feature_id[737].value <= threshold=1.6133361458778381
node_12: feature_name=GO:0003720 feature_id[228].value <= threshold=5.519326210021973
node_13: feature_name=GO:0046006 feature_id[364].value <= threshold=4.9985432624816895
node_14: feature_name=GO:0070424 feature_id[254].value <= threshold=6.233297109603882
node_15: feature_name=GO:0009892 feature_id[320].value <= threshold=59.99180793762207
node_16: feature_name=GO:0007064 feature_id[527].value <= threshold=11.57377815246582
node_17: feature_name=GO:0005575 feature_id[17].value <= threshold=15.417460918426514

node_18: feature_name=GO:0043368 feature_id[31].value <= threshold=4.059496641159058
node_19: feature_name=GO:0005164 feature_id[163].value <= threshold=5.793607711791992
node_20: feature_name=GO:0042130 feature_id[16].value <= threshold=5.073179721832275
node_21: feature_name=GO:0010216 feature_id[282].value <= threshold=4.834584474563599
node_22: feature_name=GO:0009628 feature_id[553].value <= threshold=29.314892768859863
node_23: feature_name=GO:0045628 feature_id[749].value <= threshold=3.1001367568969727
node_24: feature_name=GO:0042288 feature_id[100].value <= threshold=5.174932956695557
node_25: feature_name=GO:0002329 feature_id[406].value <= threshold=2.348930835723877
node_26: feature_name=GO:0051246 feature_id[642].value > threshold=0.6498909294605255
node_194: feature_name=GO:0009164 feature_id[546].value <= threshold=17.11970329284668
node_195: feature_name=GO:0001909 feature_id[386].value <= threshold=7.292566299438477
node_196: feature_name=GO:0003908 feature_id[11].value <= threshold=1.887226164340973
node_197: feature_name=GO:0009164 feature_id[546].value > threshold=0.26642198860645294
node_359: feature_name=GO:0009164 feature_id[546].value > threshold=0.2696942090988159
node_361: feature_name=hsa05202 feature_id[50].value <= threshold=14.989679336547852
node_362: feature_name=GO:0001775 feature_id[505].value <= threshold=15.726949691772461
node_363: feature_name=GO:0002634 feature_id[449].value <= threshold=2.825531005859375
node_364: feature_name=GO:0010038 feature_id[457].value <= threshold=9.901018619537354
node_365: feature_name=GO:0044238 feature_id[295].value <= threshold=139.80535888671875
node_366: feature_name=GO:0051246 feature_id[642].value > threshold=0.6527281999588013
node_368: feature_name=GO:0044092 feature_id[432].value <= threshold=11.084641933441162
node_369: feature_name=GO:0042162 feature_id[355].value <= threshold=9.683155536651611
node_370: feature_name=GO:0045945 feature_id[267].value <= threshold=3.6181851625442505
node_371: feature_name=GO:0036296 feature_id[252].value <= threshold=3.214281916618347
node_372: feature_name=GO:0097506 feature_id[51].value <= threshold=2.9644681215286255
node_373: feature_name=GO:0019740 feature_id[581].value <= threshold=2.2853575944900513
node_374: feature_name=GO:0019660 feature_id[70].value <= threshold=1.2831979990005493
node_375: feature_name=GO:0071901 feature_id[407].value <= threshold=14.498775482177734
node_376: feature_name=GO:0043170 feature_id[467].value <= threshold=4.473330818655086e-06
node_377: feature_name=GO:0005634 feature_id[247].value > threshold=0.06159437051974237
Class: negative genes

Rules_710 passed counts:1
node_0: feature_name=GO:0042113 feature_id[0].value <= threshold=13.408552169799805
node_1: feature_name=GO:0007568 feature_id[534].value <= threshold=5.0313897132873535

node_2: feature_name=GO:0002705	feature_id[541].value <= threshold=3.200145721435547
node_3: feature_name=GO:1901525	feature_id[576].value <= threshold=0.4466460347175598
node_4: feature_name=GO:0048539	feature_id[319].value <= threshold=3.0399646759033203
node_5: feature_name=GO:0001910	feature_id[385].value <= threshold=3.7437864542007446
node_6: feature_name=GO:0043200	feature_id[706].value <= threshold=9.307284355163574
node_7: feature_name=GO:0001773	feature_id[308].value <= threshold=4.016931533813477
node_8: feature_name=GO:0090116	feature_id[97].value <= threshold=7.99645471572876
node_9: feature_name=GO:0019814	feature_id[189].value <= threshold=4.525782108306885
node_10: feature_name=GO:1902583	feature_id[215].value <= threshold=17.60310649871826
node_11: feature_name=GO:0045429	feature_id[737].value <= threshold=1.6133361458778381
node_12: feature_name=GO:0003720	feature_id[228].value <= threshold=5.519326210021973
node_13: feature_name=GO:0046006	feature_id[364].value <= threshold=4.9985432624816895
node_14: feature_name=GO:0070424	feature_id[254].value <= threshold=6.233297109603882
node_15: feature_name=GO:0009892	feature_id[320].value <= threshold=59.99180793762207
node_16: feature_name=GO:0007064	feature_id[527].value <= threshold=11.57377815246582
node_17: feature_name=GO:0005575	feature_id[17].value <= threshold=15.417460918426514
node_18: feature_name=GO:0043368	feature_id[31].value <= threshold=4.059496641159058
node_19: feature_name=GO:0005164	feature_id[163].value <= threshold=5.793607711791992
node_20: feature_name=GO:0042130	feature_id[16].value <= threshold=5.073179721832275
node_21: feature_name=GO:0010216	feature_id[282].value <= threshold=4.834584474563599
node_22: feature_name=GO:0009628	feature_id[553].value <= threshold=29.314892768859863
node_23: feature_name=GO:0045628	feature_id[749].value <= threshold=3.1001367568969727
node_24: feature_name=GO:0042288	feature_id[100].value <= threshold=5.174932956695557
node_25: feature_name=GO:0002329	feature_id[406].value <= threshold=2.348930835723877
node_26: feature_name=GO:0051246	feature_id[642].value > threshold=0.6498909294605255
node_194: feature_name=GO:0009164	feature_id[546].value <= threshold=17.11970329284668
node_195: feature_name=GO:0001909	feature_id[386].value <= threshold=7.292566299438477
node_196: feature_name=GO:0003908	feature_id[11].value <= threshold=1.887226164340973
node_197: feature_name=GO:0009164	feature_id[546].value > threshold=0.26642198860645294
node_359: feature_name=GO:0009164	feature_id[546].value > threshold=0.2696942090988159
node_361: feature_name=hsa05202	feature_id[50].value <= threshold=14.989679336547852
node_362: feature_name=GO:0001775	feature_id[505].value <= threshold=15.726949691772461
node_363: feature_name=GO:0002634	feature_id[449].value <= threshold=2.825531005859375
node_364: feature_name=GO:0010038	feature_id[457].value <= threshold=9.901018619537354
node_365: feature_name=GO:0044238	feature_id[295].value <= threshold=139.80535888671875

node_366: feature_name=GO:0051246 feature_id[642].value > threshold=0.6527281999588013
node_368: feature_name=GO:0044092 feature_id[432].value <= threshold=11.084641933441162
node_369: feature_name=GO:0042162 feature_id[355].value <= threshold=9.683155536651611
node_370: feature_name=GO:0045945 feature_id[267].value <= threshold=3.6181851625442505
node_371: feature_name=GO:0036296 feature_id[252].value <= threshold=3.214281916618347
node_372: feature_name=GO:0097506 feature_id[51].value <= threshold=2.9644681215286255
node_373: feature_name=GO:0019740 feature_id[581].value <= threshold=2.2853575944900513
node_374: feature_name=GO:0019660 feature_id[70].value <= threshold=1.2831979990005493
node_375: feature_name=GO:0071901 feature_id[407].value <= threshold=14.498775482177734
node_376: feature_name=GO:0043170 feature_id[467].value <= threshold=4.473330818655086e-06
node_377: feature_name=GO:0005634 feature_id[247].value <= threshold=0.06159437051974237
Class: positive genes

Rules_711 passed counts:1
node_0: feature_name=GO:0042113 feature_id[0].value <= threshold=13.408552169799805
node_1: feature_name=GO:0007568 feature_id[534].value <= threshold=5.0313897132873535
node_2: feature_name=GO:0002705 feature_id[541].value <= threshold=3.200145721435547
node_3: feature_name=GO:1901525 feature_id[576].value <= threshold=0.4466460347175598
node_4: feature_name=GO:0048539 feature_id[319].value <= threshold=3.0399646759033203
node_5: feature_name=GO:0001910 feature_id[385].value <= threshold=3.7437864542007446
node_6: feature_name=GO:0043200 feature_id[706].value <= threshold=9.307284355163574
node_7: feature_name=GO:0001773 feature_id[308].value <= threshold=4.016931533813477
node_8: feature_name=GO:0090116 feature_id[97].value <= threshold=7.99645471572876
node_9: feature_name=GO:0019814 feature_id[189].value <= threshold=4.525782108306885
node_10: feature_name=GO:1902583 feature_id[215].value <= threshold=17.60310649871826
node_11: feature_name=GO:0045429 feature_id[737].value <= threshold=1.6133361458778381
node_12: feature_name=GO:0003720 feature_id[228].value <= threshold=5.519326210021973
node_13: feature_name=GO:0046006 feature_id[364].value <= threshold=4.9985432624816895
node_14: feature_name=GO:0070424 feature_id[254].value <= threshold=6.233297109603882
node_15: feature_name=GO:0009892 feature_id[320].value <= threshold=59.99180793762207
node_16: feature_name=GO:0007064 feature_id[527].value <= threshold=11.57377815246582
node_17: feature_name=GO:0005575 feature_id[17].value <= threshold=15.417460918426514
node_18: feature_name=GO:0043368 feature_id[31].value <= threshold=4.059496641159058
node_19: feature_name=GO:0005164 feature_id[163].value <= threshold=5.793607711791992
node_20: feature_name=GO:0042130 feature_id[16].value <= threshold=5.073179721832275

node_21: feature_name=GO:0010216 feature_id[282].value <= threshold=4.834584474563599
node_22: feature_name=GO:0009628 feature_id[553].value <= threshold=29.314892768859863
node_23: feature_name=GO:0045628 feature_id[749].value <= threshold=3.1001367568969727
node_24: feature_name=GO:0042288 feature_id[100].value <= threshold=5.174932956695557
node_25: feature_name=GO:0002329 feature_id[406].value <= threshold=2.348930835723877
node_26: feature_name=GO:0051246 feature_id[642].value > threshold=0.6498909294605255
node_194: feature_name=GO:0009164 feature_id[546].value <= threshold=17.11970329284668
node_195: feature_name=GO:0001909 feature_id[386].value <= threshold=7.292566299438477
node_196: feature_name=GO:0003908 feature_id[11].value <= threshold=1.887226164340973
node_197: feature_name=GO:0009164 feature_id[546].value > threshold=0.26642198860645294
node_359: feature_name=GO:0009164 feature_id[546].value > threshold=0.2696942090988159
node_361: feature_name=hsa05202 feature_id[50].value <= threshold=14.989679336547852
node_362: feature_name=GO:0001775 feature_id[505].value <= threshold=15.726949691772461
node_363: feature_name=GO:0002634 feature_id[449].value <= threshold=2.825531005859375
node_364: feature_name=GO:0010038 feature_id[457].value <= threshold=9.901018619537354
node_365: feature_name=GO:0044238 feature_id[295].value <= threshold=139.80535888671875
node_366: feature_name=GO:0051246 feature_id[642].value <= threshold=0.6527281999588013
Class: positive genes

Rules_712 passed counts:1
node_0: feature_name=GO:0042113 feature_id[0].value <= threshold=13.408552169799805
node_1: feature_name=GO:0007568 feature_id[534].value <= threshold=5.0313897132873535
node_2: feature_name=GO:0002705 feature_id[541].value <= threshold=3.200145721435547
node_3: feature_name=GO:1901525 feature_id[576].value <= threshold=0.4466460347175598
node_4: feature_name=GO:0048539 feature_id[319].value <= threshold=3.0399646759033203
node_5: feature_name=GO:0001910 feature_id[385].value <= threshold=3.7437864542007446
node_6: feature_name=GO:0043200 feature_id[706].value <= threshold=9.307284355163574
node_7: feature_name=GO:0001773 feature_id[308].value <= threshold=4.016931533813477
node_8: feature_name=GO:0090116 feature_id[97].value <= threshold=7.99645471572876
node_9: feature_name=GO:0019814 feature_id[189].value <= threshold=4.525782108306885
node_10: feature_name=GO:1902583 feature_id[215].value <= threshold=17.60310649871826
node_11: feature_name=GO:0045429 feature_id[737].value <= threshold=1.6133361458778381
node_12: feature_name=GO:0003720 feature_id[228].value <= threshold=5.519326210021973
node_13: feature_name=GO:0046006 feature_id[364].value <= threshold=4.9985432624816895
node_14: feature_name=GO:0070424 feature_id[254].value <= threshold=6.233297109603882

node_15: feature_name=GO:0009892	feature_id[320].value <= threshold=59.99180793762207
node_16: feature_name=GO:0007064	feature_id[527].value <= threshold=11.57377815246582
node_17: feature_name=GO:0005575	feature_id[17].value <= threshold=15.417460918426514
node_18: feature_name=GO:0043368	feature_id[31].value <= threshold=4.059496641159058
node_19: feature_name=GO:0005164	feature_id[163].value <= threshold=5.793607711791992
node_20: feature_name=GO:0042130	feature_id[16].value <= threshold=5.073179721832275
node_21: feature_name=GO:0010216	feature_id[282].value <= threshold=4.834584474563599
node_22: feature_name=GO:0009628	feature_id[553].value <= threshold=29.314892768859863
node_23: feature_name=GO:0045628	feature_id[749].value <= threshold=3.1001367568969727
node_24: feature_name=GO:0042288	feature_id[100].value <= threshold=5.174932956695557
node_25: feature_name=GO:0002329	feature_id[406].value <= threshold=2.348930835723877
node_26: feature_name=GO:0051246	feature_id[642].value > threshold=0.6498909294605255
node_194: feature_name=GO:0009164	feature_id[546].value <= threshold=17.11970329284668
node_195: feature_name=GO:0001909	feature_id[386].value <= threshold=7.292566299438477
node_196: feature_name=GO:0003908	feature_id[11].value <= threshold=1.887226164340973
node_197: feature_name=GO:0009164	feature_id[546].value <= threshold=0.26642198860645294
node_198: feature_name=GO:2001242	feature_id[24].value > threshold=9.207026481628418
node_356: feature_name=GO:0009164	feature_id[546].value > threshold=0.11216866970062256
Class: negative genes	

Rules_713	passed counts:1
node_0: feature_name=GO:0042113	feature_id[0].value <= threshold=13.408552169799805
node_1: feature_name=GO:0007568	feature_id[534].value <= threshold=5.0313897132873535
node_2: feature_name=GO:0002705	feature_id[541].value <= threshold=3.200145721435547
node_3: feature_name=GO:1901525	feature_id[576].value <= threshold=0.4466460347175598
node_4: feature_name=GO:0048539	feature_id[319].value <= threshold=3.0399646759033203
node_5: feature_name=GO:0001910	feature_id[385].value <= threshold=3.7437864542007446
node_6: feature_name=GO:0043200	feature_id[706].value <= threshold=9.307284355163574
node_7: feature_name=GO:0001773	feature_id[308].value <= threshold=4.016931533813477
node_8: feature_name=GO:0090116	feature_id[97].value <= threshold=7.99645471572876
node_9: feature_name=GO:0019814	feature_id[189].value <= threshold=4.525782108306885
node_10: feature_name=GO:1902583	feature_id[215].value <= threshold=17.60310649871826
node_11: feature_name=GO:0045429	feature_id[737].value <= threshold=1.6133361458778381
node_12: feature_name=GO:0003720	feature_id[228].value <= threshold=5.519326210021973
node_13: feature_name=GO:0046006	feature_id[364].value <= threshold=4.9985432624816895

node_14: feature_name=GO:0070424 feature_id[254].value <= threshold=6.233297109603882
node_15: feature_name=GO:0009892 feature_id[320].value <= threshold=59.99180793762207
node_16: feature_name=GO:0007064 feature_id[527].value <= threshold=11.57377815246582
node_17: feature_name=GO:0005575 feature_id[17].value <= threshold=15.417460918426514
node_18: feature_name=GO:0043368 feature_id[31].value <= threshold=4.059496641159058
node_19: feature_name=GO:0005164 feature_id[163].value <= threshold=5.793607711791992
node_20: feature_name=GO:0042130 feature_id[16].value <= threshold=5.073179721832275
node_21: feature_name=GO:0010216 feature_id[282].value <= threshold=4.834584474563599
node_22: feature_name=GO:0009628 feature_id[553].value <= threshold=29.314892768859863
node_23: feature_name=GO:0045628 feature_id[749].value <= threshold=3.1001367568969727
node_24: feature_name=GO:0042288 feature_id[100].value <= threshold=5.174932956695557
node_25: feature_name=GO:0002329 feature_id[406].value <= threshold=2.348930835723877
node_26: feature_name=GO:0051246 feature_id[642].value > threshold=0.6498909294605255
node_194: feature_name=GO:0009164 feature_id[546].value <= threshold=17.11970329284668
node_195: feature_name=GO:0001909 feature_id[386].value <= threshold=7.292566299438477
node_196: feature_name=GO:0003908 feature_id[11].value <= threshold=1.887226164340973
node_197: feature_name=GO:0009164 feature_id[546].value <= threshold=0.26642198860645294
node_198: feature_name=GO:2001242 feature_id[24].value > threshold=9.207026481628418
node_356: feature_name=GO:0009164 feature_id[546].value <= threshold=0.11216866970062256
Class: positive genes

Rules_714 passed counts:1
node_0: feature_name=GO:0042113 feature_id[0].value <= threshold=13.408552169799805
node_1: feature_name=GO:0007568 feature_id[534].value <= threshold=5.0313897132873535
node_2: feature_name=GO:0002705 feature_id[541].value <= threshold=3.200145721435547
node_3: feature_name=GO:1901525 feature_id[576].value <= threshold=0.4466460347175598
node_4: feature_name=GO:0048539 feature_id[319].value <= threshold=3.0399646759033203
node_5: feature_name=GO:0001910 feature_id[385].value <= threshold=3.7437864542007446
node_6: feature_name=GO:0043200 feature_id[706].value <= threshold=9.307284355163574
node_7: feature_name=GO:0001773 feature_id[308].value <= threshold=4.016931533813477
node_8: feature_name=GO:0090116 feature_id[97].value <= threshold=7.99645471572876
node_9: feature_name=GO:0019814 feature_id[189].value <= threshold=4.525782108306885
node_10: feature_name=GO:1902583 feature_id[215].value <= threshold=17.60310649871826
node_11: feature_name=GO:0045429 feature_id[737].value <= threshold=1.6133361458778381
node_12: feature_name=GO:0003720 feature_id[228].value <= threshold=5.519326210021973

node_13: feature_name=GO:0046006 feature_id[364].value <= threshold=4.9985432624816895
node_14: feature_name=GO:0070424 feature_id[254].value <= threshold=6.233297109603882
node_15: feature_name=GO:0009892 feature_id[320].value <= threshold=59.99180793762207
node_16: feature_name=GO:0007064 feature_id[527].value <= threshold=11.57377815246582
node_17: feature_name=GO:0005575 feature_id[17].value <= threshold=15.417460918426514
node_18: feature_name=GO:0043368 feature_id[31].value <= threshold=4.059496641159058
node_19: feature_name=GO:0005164 feature_id[163].value <= threshold=5.793607711791992
node_20: feature_name=GO:0042130 feature_id[16].value <= threshold=5.073179721832275
node_21: feature_name=GO:0010216 feature_id[282].value <= threshold=4.834584474563599
node_22: feature_name=GO:0009628 feature_id[553].value <= threshold=29.314892768859863
node_23: feature_name=GO:0045628 feature_id[749].value <= threshold=3.1001367568969727
node_24: feature_name=GO:0042288 feature_id[100].value <= threshold=5.174932956695557
node_25: feature_name=GO:0002329 feature_id[406].value <= threshold=2.348930835723877
node_26: feature_name=GO:0051246 feature_id[642].value > threshold=0.6498909294605255
node_194: feature_name=GO:0009164 feature_id[546].value <= threshold=17.11970329284668
node_195: feature_name=GO:0001909 feature_id[386].value <= threshold=7.292566299438477
node_196: feature_name=GO:0003908 feature_id[11].value <= threshold=1.887226164340973
node_197: feature_name=GO:0009164 feature_id[546].value <= threshold=0.26642198860645294
node_198: feature_name=GO:2001242 feature_id[24].value <= threshold=9.207026481628418
node_199: feature_name=GO:0051246 feature_id[642].value > threshold=0.6504445374011993
node_203: feature_name=GO:0032461 feature_id[608].value > threshold=2.607542634010315
node_353: feature_name=hsa05164 feature_id[340].value > threshold=1.6018363796174526
Class: negative genes

Rules_715 passed counts:1
node_0: feature_name=GO:0042113 feature_id[0].value <= threshold=13.408552169799805
node_1: feature_name=GO:0007568 feature_id[534].value <= threshold=5.0313897132873535
node_2: feature_name=GO:0002705 feature_id[541].value <= threshold=3.200145721435547
node_3: feature_name=GO:1901525 feature_id[576].value <= threshold=0.4466460347175598
node_4: feature_name=GO:0048539 feature_id[319].value <= threshold=3.0399646759033203
node_5: feature_name=GO:0001910 feature_id[385].value <= threshold=3.7437864542007446
node_6: feature_name=GO:0043200 feature_id[706].value <= threshold=9.307284355163574
node_7: feature_name=GO:0001773 feature_id[308].value <= threshold=4.016931533813477
node_8: feature_name=GO:0090116 feature_id[97].value <= threshold=7.99645471572876
node_9: feature_name=GO:0019814 feature_id[189].value <= threshold=4.525782108306885

node_10: feature_name=GO:1902583 feature_id[215].value <= threshold=17.60310649871826
node_11: feature_name=GO:0045429 feature_id[737].value <= threshold=1.6133361458778381
node_12: feature_name=GO:0003720 feature_id[228].value <= threshold=5.519326210021973
node_13: feature_name=GO:0046006 feature_id[364].value <= threshold=4.9985432624816895
node_14: feature_name=GO:0070424 feature_id[254].value <= threshold=6.233297109603882
node_15: feature_name=GO:0009892 feature_id[320].value <= threshold=59.99180793762207
node_16: feature_name=GO:0007064 feature_id[527].value <= threshold=11.57377815246582
node_17: feature_name=GO:0005575 feature_id[17].value <= threshold=15.417460918426514
node_18: feature_name=GO:0043368 feature_id[31].value <= threshold=4.059496641159058
node_19: feature_name=GO:0005164 feature_id[163].value <= threshold=5.793607711791992
node_20: feature_name=GO:0042130 feature_id[16].value <= threshold=5.073179721832275
node_21: feature_name=GO:0010216 feature_id[282].value <= threshold=4.834584474563599
node_22: feature_name=GO:0009628 feature_id[553].value <= threshold=29.314892768859863
node_23: feature_name=GO:0045628 feature_id[749].value <= threshold=3.1001367568969727
node_24: feature_name=GO:0042288 feature_id[100].value <= threshold=5.174932956695557
node_25: feature_name=GO:0002329 feature_id[406].value <= threshold=2.348930835723877
node_26: feature_name=GO:0051246 feature_id[642].value > threshold=0.6498909294605255
node_194: feature_name=GO:0009164 feature_id[546].value <= threshold=17.11970329284668
node_195: feature_name=GO:0001909 feature_id[386].value <= threshold=7.292566299438477
node_196: feature_name=GO:0003908 feature_id[11].value <= threshold=1.887226164340973
node_197: feature_name=GO:0009164 feature_id[546].value <= threshold=0.26642198860645294
node_198: feature_name=GO:2001242 feature_id[24].value <= threshold=9.207026481628418
node_199: feature_name=GO:0051246 feature_id[642].value > threshold=0.6504445374011993
node_203: feature_name=GO:0032461 feature_id[608].value > threshold=2.607542634010315
node_353: feature_name=hsa05164 feature_id[340].value <= threshold=1.6018363796174526
Class: positive genes

Rules_716 passed counts:1
node_0: feature_name=GO:0042113 feature_id[0].value <= threshold=13.408552169799805
node_1: feature_name=GO:0007568 feature_id[534].value <= threshold=5.0313897132873535
node_2: feature_name=GO:0002705 feature_id[541].value <= threshold=3.200145721435547
node_3: feature_name=GO:1901525 feature_id[576].value <= threshold=0.4466460347175598
node_4: feature_name=GO:0048539 feature_id[319].value <= threshold=3.0399646759033203
node_5: feature_name=GO:0001910 feature_id[385].value <= threshold=3.7437864542007446
node_6: feature_name=GO:0043200 feature_id[706].value <= threshold=9.307284355163574

node_7: feature_name=GO:0001773 feature_id[308].value <= threshold=4.016931533813477
node_8: feature_name=GO:0090116 feature_id[97].value <= threshold=7.99645471572876
node_9: feature_name=GO:0019814 feature_id[189].value <= threshold=4.525782108306885
node_10: feature_name=GO:1902583 feature_id[215].value <= threshold=17.60310649871826
node_11: feature_name=GO:0045429 feature_id[737].value <= threshold=1.6133361458778381
node_12: feature_name=GO:0003720 feature_id[228].value <= threshold=5.519326210021973
node_13: feature_name=GO:0046006 feature_id[364].value <= threshold=4.9985432624816895
node_14: feature_name=GO:0070424 feature_id[254].value <= threshold=6.233297109603882
node_15: feature_name=GO:0009892 feature_id[320].value <= threshold=59.99180793762207
node_16: feature_name=GO:0007064 feature_id[527].value <= threshold=11.57377815246582
node_17: feature_name=GO:0005575 feature_id[17].value <= threshold=15.417460918426514
node_18: feature_name=GO:0043368 feature_id[31].value <= threshold=4.059496641159058
node_19: feature_name=GO:0005164 feature_id[163].value <= threshold=5.793607711791992
node_20: feature_name=GO:0042130 feature_id[16].value <= threshold=5.073179721832275
node_21: feature_name=GO:0010216 feature_id[282].value <= threshold=4.834584474563599
node_22: feature_name=GO:0009628 feature_id[553].value <= threshold=29.314892768859863
node_23: feature_name=GO:0045628 feature_id[749].value <= threshold=3.1001367568969727
node_24: feature_name=GO:0042288 feature_id[100].value <= threshold=5.174932956695557
node_25: feature_name=GO:0002329 feature_id[406].value <= threshold=2.348930835723877
node_26: feature_name=GO:0051246 feature_id[642].value > threshold=0.6498909294605255
node_194: feature_name=GO:0009164 feature_id[546].value <= threshold=17.11970329284668
node_195: feature_name=GO:0001909 feature_id[386].value <= threshold=7.292566299438477
node_196: feature_name=GO:0003908 feature_id[11].value <= threshold=1.887226164340973
node_197: feature_name=GO:0009164 feature_id[546].value <= threshold=0.26642198860645294
node_198: feature_name=GO:2001242 feature_id[24].value <= threshold=9.207026481628418
node_199: feature_name=GO:0051246 feature_id[642].value > threshold=0.6504445374011993
node_203: feature_name=GO:0032461 feature_id[608].value <= threshold=2.607542634010315
node_204: feature_name=GO:0007584 feature_id[529].value > threshold=16.099515914916992
node_350: feature_name=GO:0042542 feature_id[689].value > threshold=0.4581376388669014
Class: positive genes

Rules_717 passed counts:1
node_0: feature_name=GO:0042113 feature_id[0].value <= threshold=13.408552169799805
node_1: feature_name=GO:0007568 feature_id[534].value <= threshold=5.0313897132873535
node_2: feature_name=GO:0002705 feature_id[541].value <= threshold=3.200145721435547

node_3: feature_name=GO:1901525 feature_id[576].value <= threshold=0.4466460347175598
node_4: feature_name=GO:0048539 feature_id[319].value <= threshold=3.0399646759033203
node_5: feature_name=GO:0001910 feature_id[385].value <= threshold=3.7437864542007446
node_6: feature_name=GO:0043200 feature_id[706].value <= threshold=9.307284355163574
node_7: feature_name=GO:0001773 feature_id[308].value <= threshold=4.016931533813477
node_8: feature_name=GO:0090116 feature_id[97].value <= threshold=7.99645471572876
node_9: feature_name=GO:0019814 feature_id[189].value <= threshold=4.525782108306885
node_10: feature_name=GO:1902583 feature_id[215].value <= threshold=17.60310649871826
node_11: feature_name=GO:0045429 feature_id[737].value <= threshold=1.6133361458778381
node_12: feature_name=GO:0003720 feature_id[228].value <= threshold=5.519326210021973
node_13: feature_name=GO:0046006 feature_id[364].value <= threshold=4.9985432624816895
node_14: feature_name=GO:0070424 feature_id[254].value <= threshold=6.233297109603882
node_15: feature_name=GO:0009892 feature_id[320].value <= threshold=59.99180793762207
node_16: feature_name=GO:0007064 feature_id[527].value <= threshold=11.57377815246582
node_17: feature_name=GO:0005575 feature_id[17].value <= threshold=15.417460918426514
node_18: feature_name=GO:0043368 feature_id[31].value <= threshold=4.059496641159058
node_19: feature_name=GO:0005164 feature_id[163].value <= threshold=5.793607711791992
node_20: feature_name=GO:0042130 feature_id[16].value <= threshold=5.073179721832275
node_21: feature_name=GO:0010216 feature_id[282].value <= threshold=4.834584474563599
node_22: feature_name=GO:0009628 feature_id[553].value <= threshold=29.314892768859863
node_23: feature_name=GO:0045628 feature_id[749].value <= threshold=3.1001367568969727
node_24: feature_name=GO:0042288 feature_id[100].value <= threshold=5.174932956695557
node_25: feature_name=GO:0002329 feature_id[406].value <= threshold=2.348930835723877
node_26: feature_name=GO:0051246 feature_id[642].value > threshold=0.6498909294605255
node_194: feature_name=GO:0009164 feature_id[546].value <= threshold=17.11970329284668
node_195: feature_name=GO:0001909 feature_id[386].value <= threshold=7.292566299438477
node_196: feature_name=GO:0003908 feature_id[11].value <= threshold=1.887226164340973
node_197: feature_name=GO:0009164 feature_id[546].value <= threshold=0.26642198860645294
node_198: feature_name=GO:2001242 feature_id[24].value <= threshold=9.207026481628418
node_199: feature_name=GO:0051246 feature_id[642].value > threshold=0.6504445374011993
node_203: feature_name=GO:0032461 feature_id[608].value <= threshold=2.607542634010315
node_204: feature_name=GO:0007584 feature_id[529].value > threshold=16.099515914916992
node_350: feature_name=GO:0042542 feature_id[689].value <= threshold=0.4581376388669014
Class: negative genes

Rules_718	passed counts:1
node_0: feature_name=GO:0042113	feature_id[0].value <= threshold=13.408552169799805
node_1: feature_name=GO:0007568	feature_id[534].value <= threshold=5.0313897132873535
node_2: feature_name=GO:0002705	feature_id[541].value <= threshold=3.200145721435547
node_3: feature_name=GO:1901525	feature_id[576].value <= threshold=0.4466460347175598
node_4: feature_name=GO:0048539	feature_id[319].value <= threshold=3.0399646759033203
node_5: feature_name=GO:0001910	feature_id[385].value <= threshold=3.7437864542007446
node_6: feature_name=GO:0043200	feature_id[706].value <= threshold=9.307284355163574
node_7: feature_name=GO:0001773	feature_id[308].value <= threshold=4.016931533813477
node_8: feature_name=GO:0090116	feature_id[97].value <= threshold=7.99645471572876
node_9: feature_name=GO:0019814	feature_id[189].value <= threshold=4.525782108306885
node_10: feature_name=GO:1902583	feature_id[215].value <= threshold=17.60310649871826
node_11: feature_name=GO:0045429	feature_id[737].value <= threshold=1.6133361458778381
node_12: feature_name=GO:0003720	feature_id[228].value <= threshold=5.519326210021973
node_13: feature_name=GO:0046006	feature_id[364].value <= threshold=4.9985432624816895
node_14: feature_name=GO:0070424	feature_id[254].value <= threshold=6.233297109603882
node_15: feature_name=GO:0009892	feature_id[320].value <= threshold=59.99180793762207
node_16: feature_name=GO:0007064	feature_id[527].value <= threshold=11.57377815246582
node_17: feature_name=GO:0005575	feature_id[17].value <= threshold=15.417460918426514
node_18: feature_name=GO:0043368	feature_id[31].value <= threshold=4.059496641159058
node_19: feature_name=GO:0005164	feature_id[163].value <= threshold=5.793607711791992
node_20: feature_name=GO:0042130	feature_id[16].value <= threshold=5.073179721832275
node_21: feature_name=GO:0010216	feature_id[282].value <= threshold=4.834584474563599
node_22: feature_name=GO:0009628	feature_id[553].value <= threshold=29.314892768859863
node_23: feature_name=GO:0045628	feature_id[749].value <= threshold=3.1001367568969727
node_24: feature_name=GO:0042288	feature_id[100].value <= threshold=5.174932956695557
node_25: feature_name=GO:0002329	feature_id[406].value <= threshold=2.348930835723877
node_26: feature_name=GO:0051246	feature_id[642].value > threshold=0.6498909294605255
node_194: feature_name=GO:0009164	feature_id[546].value <= threshold=17.11970329284668
node_195: feature_name=GO:0001909	feature_id[386].value <= threshold=7.292566299438477
node_196: feature_name=GO:0003908	feature_id[11].value <= threshold=1.887226164340973
node_197: feature_name=GO:0009164	feature_id[546].value <= threshold=0.26642198860645294
node_198: feature_name=GO:2001242	feature_id[24].value <= threshold=9.207026481628418
node_199: feature_name=GO:0051246	feature_id[642].value > threshold=0.6504445374011993
node_203: feature_name=GO:0032461	feature_id[608].value <= threshold=2.607542634010315

node_204: feature_name=GO:0007584 feature_id[529].value <= threshold=16.099515914916992
node_205: feature_name=hsa05221 feature_id[349].value > threshold=9.359850406646729
node_337: feature_name=GO:0032743 feature_id[258].value > threshold=2.931445837020874
node_347: feature_name=GO:0000217 feature_id[765].value > threshold=1.9003757238388062
Class: negative genes

Rules_719 passed counts:1
node_0: feature_name=GO:0042113 feature_id[0].value <= threshold=13.408552169799805
node_1: feature_name=GO:0007568 feature_id[534].value <= threshold=5.0313897132873535
node_2: feature_name=GO:0002705 feature_id[541].value <= threshold=3.200145721435547
node_3: feature_name=GO:1901525 feature_id[576].value <= threshold=0.4466460347175598
node_4: feature_name=GO:0048539 feature_id[319].value <= threshold=3.0399646759033203
node_5: feature_name=GO:0001910 feature_id[385].value <= threshold=3.7437864542007446
node_6: feature_name=GO:0043200 feature_id[706].value <= threshold=9.307284355163574
node_7: feature_name=GO:0001773 feature_id[308].value <= threshold=4.016931533813477
node_8: feature_name=GO:0090116 feature_id[97].value <= threshold=7.99645471572876
node_9: feature_name=GO:0019814 feature_id[189].value <= threshold=4.525782108306885
node_10: feature_name=GO:1902583 feature_id[215].value <= threshold=17.60310649871826
node_11: feature_name=GO:0045429 feature_id[737].value <= threshold=1.6133361458778381
node_12: feature_name=GO:0003720 feature_id[228].value <= threshold=5.519326210021973
node_13: feature_name=GO:0046006 feature_id[364].value <= threshold=4.9985432624816895
node_14: feature_name=GO:0070424 feature_id[254].value <= threshold=6.233297109603882
node_15: feature_name=GO:0009892 feature_id[320].value <= threshold=59.99180793762207
node_16: feature_name=GO:0007064 feature_id[527].value <= threshold=11.57377815246582
node_17: feature_name=GO:0005575 feature_id[17].value <= threshold=15.417460918426514
node_18: feature_name=GO:0043368 feature_id[31].value <= threshold=4.059496641159058
node_19: feature_name=GO:0005164 feature_id[163].value <= threshold=5.793607711791992
node_20: feature_name=GO:0042130 feature_id[16].value <= threshold=5.073179721832275
node_21: feature_name=GO:0010216 feature_id[282].value <= threshold=4.834584474563599
node_22: feature_name=GO:0009628 feature_id[553].value <= threshold=29.314892768859863
node_23: feature_name=GO:0045628 feature_id[749].value <= threshold=3.1001367568969727
node_24: feature_name=GO:0042288 feature_id[100].value <= threshold=5.174932956695557
node_25: feature_name=GO:0002329 feature_id[406].value <= threshold=2.348930835723877
node_26: feature_name=GO:0051246 feature_id[642].value > threshold=0.6498909294605255
node_194: feature_name=GO:0009164 feature_id[546].value <= threshold=17.11970329284668

node_195: feature_name=GO:0001909 feature_id[386].value <= threshold=7.292566299438477
node_196: feature_name=GO:0003908 feature_id[11].value <= threshold=1.887226164340973
node_197: feature_name=GO:0009164 feature_id[546].value <= threshold=0.26642198860645294
node_198: feature_name=GO:2001242 feature_id[24].value <= threshold=9.207026481628418
node_199: feature_name=GO:0051246 feature_id[642].value > threshold=0.6504445374011993
node_203: feature_name=GO:0032461 feature_id[608].value <= threshold=2.607542634010315
node_204: feature_name=GO:0007584 feature_id[529].value <= threshold=16.099515914916992
node_205: feature_name=hsa05221 feature_id[349].value > threshold=9.359850406646729
node_337: feature_name=GO:0032743 feature_id[258].value <= threshold=2.931445837020874
node_338: feature_name=GO:0002460 feature_id[357].value > threshold=0.0003823822917183861
node_340: feature_name=GO:0019740 feature_id[581].value > threshold=1.0357294380664825
Class: positive genes

Rules_720 passed counts:1
node_0: feature_name=GO:0042113 feature_id[0].value <= threshold=13.408552169799805
node_1: feature_name=GO:0007568 feature_id[534].value <= threshold=5.0313897132873535
node_2: feature_name=GO:0002705 feature_id[541].value <= threshold=3.200145721435547
node_3: feature_name=GO:1901525 feature_id[576].value <= threshold=0.4466460347175598
node_4: feature_name=GO:0048539 feature_id[319].value <= threshold=3.0399646759033203
node_5: feature_name=GO:0001910 feature_id[385].value <= threshold=3.7437864542007446
node_6: feature_name=GO:0043200 feature_id[706].value <= threshold=9.307284355163574
node_7: feature_name=GO:0001773 feature_id[308].value <= threshold=4.016931533813477
node_8: feature_name=GO:0090116 feature_id[97].value <= threshold=7.99645471572876
node_9: feature_name=GO:0019814 feature_id[189].value <= threshold=4.525782108306885
node_10: feature_name=GO:1902583 feature_id[215].value <= threshold=17.60310649871826
node_11: feature_name=GO:0045429 feature_id[737].value <= threshold=1.6133361458778381
node_12: feature_name=GO:0003720 feature_id[228].value <= threshold=5.519326210021973
node_13: feature_name=GO:0046006 feature_id[364].value <= threshold=4.9985432624816895
node_14: feature_name=GO:0070424 feature_id[254].value <= threshold=6.233297109603882
node_15: feature_name=GO:0009892 feature_id[320].value <= threshold=59.99180793762207
node_16: feature_name=GO:0007064 feature_id[527].value <= threshold=11.57377815246582
node_17: feature_name=GO:0005575 feature_id[17].value <= threshold=15.417460918426514
node_18: feature_name=GO:0043368 feature_id[31].value <= threshold=4.059496641159058
node_19: feature_name=GO:0005164 feature_id[163].value <= threshold=5.793607711791992
node_20: feature_name=GO:0042130 feature_id[16].value <= threshold=5.073179721832275

node_21: feature_name=GO:0010216 feature_id[282].value <= threshold=4.834584474563599
node_22: feature_name=GO:0009628 feature_id[553].value <= threshold=29.314892768859863
node_23: feature_name=GO:0045628 feature_id[749].value <= threshold=3.1001367568969727
node_24: feature_name=GO:0042288 feature_id[100].value <= threshold=5.174932956695557
node_25: feature_name=GO:0002329 feature_id[406].value <= threshold=2.348930835723877
node_26: feature_name=GO:0051246 feature_id[642].value > threshold=0.6498909294605255
node_194: feature_name=GO:0009164 feature_id[546].value <= threshold=17.11970329284668
node_195: feature_name=GO:0001909 feature_id[386].value <= threshold=7.292566299438477
node_196: feature_name=GO:0003908 feature_id[11].value <= threshold=1.887226164340973
node_197: feature_name=GO:0009164 feature_id[546].value <= threshold=0.26642198860645294
node_198: feature_name=GO:2001242 feature_id[24].value <= threshold=9.207026481628418
node_199: feature_name=GO:0051246 feature_id[642].value > threshold=0.6504445374011993
node_203: feature_name=GO:0032461 feature_id[608].value <= threshold=2.607542634010315
node_204: feature_name=GO:0007584 feature_id[529].value <= threshold=16.099515914916992
node_205: feature_name=hsa05221 feature_id[349].value > threshold=9.359850406646729
node_337: feature_name=GO:0032743 feature_id[258].value <= threshold=2.931445837020874
node_338: feature_name=GO:0002460 feature_id[357].value > threshold=0.0003823822917183861
node_340: feature_name=GO:0019740 feature_id[581].value <= threshold=1.0357294380664825
node_341: feature_name=hsa05221 feature_id[349].value > threshold=9.390207767486572
node_343: feature_name=GO:0045629 feature_id[746].value > threshold=1.07101109623909
Class: positive genes

Rules_721 passed counts:1
node_0: feature_name=GO:0042113 feature_id[0].value <= threshold=13.408552169799805
node_1: feature_name=GO:0007568 feature_id[534].value <= threshold=5.0313897132873535
node_2: feature_name=GO:0002705 feature_id[541].value <= threshold=3.200145721435547
node_3: feature_name=GO:1901525 feature_id[576].value <= threshold=0.4466460347175598
node_4: feature_name=GO:0048539 feature_id[319].value <= threshold=3.0399646759033203
node_5: feature_name=GO:0001910 feature_id[385].value <= threshold=3.7437864542007446
node_6: feature_name=GO:0043200 feature_id[706].value <= threshold=9.307284355163574
node_7: feature_name=GO:0001773 feature_id[308].value <= threshold=4.016931533813477
node_8: feature_name=GO:0090116 feature_id[97].value <= threshold=7.99645471572876
node_9: feature_name=GO:0019814 feature_id[189].value <= threshold=4.525782108306885
node_10: feature_name=GO:1902583 feature_id[215].value <= threshold=17.60310649871826
node_11: feature_name=GO:0045429 feature_id[737].value <= threshold=1.6133361458778381

node_12: feature_name=GO:0003720 feature_id[228].value <= threshold=5.519326210021973
node_13: feature_name=GO:0046006 feature_id[364].value <= threshold=4.9985432624816895
node_14: feature_name=GO:0070424 feature_id[254].value <= threshold=6.233297109603882
node_15: feature_name=GO:0009892 feature_id[320].value <= threshold=59.99180793762207
node_16: feature_name=GO:0007064 feature_id[527].value <= threshold=11.57377815246582
node_17: feature_name=GO:0005575 feature_id[17].value <= threshold=15.417460918426514
node_18: feature_name=GO:0043368 feature_id[31].value <= threshold=4.059496641159058
node_19: feature_name=GO:0005164 feature_id[163].value <= threshold=5.793607711791992
node_20: feature_name=GO:0042130 feature_id[16].value <= threshold=5.073179721832275
node_21: feature_name=GO:0010216 feature_id[282].value <= threshold=4.834584474563599
node_22: feature_name=GO:0009628 feature_id[553].value <= threshold=29.314892768859863
node_23: feature_name=GO:0045628 feature_id[749].value <= threshold=3.1001367568969727
node_24: feature_name=GO:0042288 feature_id[100].value <= threshold=5.174932956695557
node_25: feature_name=GO:0002329 feature_id[406].value <= threshold=2.348930835723877
node_26: feature_name=GO:0051246 feature_id[642].value > threshold=0.6498909294605255
node_194: feature_name=GO:0009164 feature_id[546].value <= threshold=17.11970329284668
node_195: feature_name=GO:0001909 feature_id[386].value <= threshold=7.292566299438477
node_196: feature_name=GO:0003908 feature_id[11].value <= threshold=1.887226164340973
node_197: feature_name=GO:0009164 feature_id[546].value <= threshold=0.26642198860645294
node_198: feature_name=GO:2001242 feature_id[24].value <= threshold=9.207026481628418
node_199: feature_name=GO:0051246 feature_id[642].value > threshold=0.6504445374011993
node_203: feature_name=GO:0032461 feature_id[608].value <= threshold=2.607542634010315
node_204: feature_name=GO:0007584 feature_id[529].value <= threshold=16.099515914916992
node_205: feature_name=hsa05221 feature_id[349].value > threshold=9.359850406646729
node_337: feature_name=GO:0032743 feature_id[258].value <= threshold=2.931445837020874
node_338: feature_name=GO:0002460 feature_id[357].value > threshold=0.0003823822917183861
node_340: feature_name=GO:0019740 feature_id[581].value <= threshold=1.0357294380664825
node_341: feature_name=hsa05221 feature_id[349].value <= threshold=9.390207767486572
Class: positive genes

Rules_722 passed counts:1
node_0: feature_name=GO:0042113 feature_id[0].value <= threshold=13.408552169799805
node_1: feature_name=GO:0007568 feature_id[534].value <= threshold=5.0313897132873535
node_2: feature_name=GO:0002705 feature_id[541].value <= threshold=3.200145721435547
node_3: feature_name=GO:1901525 feature_id[576].value <= threshold=0.4466460347175598

node_4: feature_name=GO:0048539	feature_id[319].value <= threshold=3.0399646759033203
node_5: feature_name=GO:0001910	feature_id[385].value <= threshold=3.7437864542007446
node_6: feature_name=GO:0043200	feature_id[706].value <= threshold=9.307284355163574
node_7: feature_name=GO:0001773	feature_id[308].value <= threshold=4.016931533813477
node_8: feature_name=GO:0090116	feature_id[97].value <= threshold=7.99645471572876
node_9: feature_name=GO:0019814	feature_id[189].value <= threshold=4.525782108306885
node_10: feature_name=GO:1902583	feature_id[215].value <= threshold=17.60310649871826
node_11: feature_name=GO:0045429	feature_id[737].value <= threshold=1.6133361458778381
node_12: feature_name=GO:0003720	feature_id[228].value <= threshold=5.519326210021973
node_13: feature_name=GO:0046006	feature_id[364].value <= threshold=4.9985432624816895
node_14: feature_name=GO:0070424	feature_id[254].value <= threshold=6.233297109603882
node_15: feature_name=GO:0009892	feature_id[320].value <= threshold=59.99180793762207
node_16: feature_name=GO:0007064	feature_id[527].value <= threshold=11.57377815246582
node_17: feature_name=GO:0005575	feature_id[17].value <= threshold=15.417460918426514
node_18: feature_name=GO:0043368	feature_id[31].value <= threshold=4.059496641159058
node_19: feature_name=GO:0005164	feature_id[163].value <= threshold=5.793607711791992
node_20: feature_name=GO:0042130	feature_id[16].value <= threshold=5.073179721832275
node_21: feature_name=GO:0010216	feature_id[282].value <= threshold=4.834584474563599
node_22: feature_name=GO:0009628	feature_id[553].value <= threshold=29.314892768859863
node_23: feature_name=GO:0045628	feature_id[749].value <= threshold=3.1001367568969727
node_24: feature_name=GO:0042288	feature_id[100].value <= threshold=5.174932956695557
node_25: feature_name=GO:0002329	feature_id[406].value <= threshold=2.348930835723877
node_26: feature_name=GO:0051246	feature_id[642].value > threshold=0.6498909294605255
node_194: feature_name=GO:0009164	feature_id[546].value <= threshold=17.11970329284668
node_195: feature_name=GO:0001909	feature_id[386].value <= threshold=7.292566299438477
node_196: feature_name=GO:0003908	feature_id[11].value <= threshold=1.887226164340973
node_197: feature_name=GO:0009164	feature_id[546].value <= threshold=0.26642198860645294
node_198: feature_name=GO:2001242	feature_id[24].value <= threshold=9.207026481628418
node_199: feature_name=GO:0051246	feature_id[642].value > threshold=0.6504445374011993
node_203: feature_name=GO:0032461	feature_id[608].value <= threshold=2.607542634010315
node_204: feature_name=GO:0007584	feature_id[529].value <= threshold=16.099515914916992
node_205: feature_name=hsa05221	feature_id[349].value > threshold=9.359850406646729
node_337: feature_name=GO:0032743	feature_id[258].value <= threshold=2.931445837020874
node_338: feature_name=GO:0002460	feature_id[357].value <= threshold=0.0003823822917183861

Class: positive genes

Rules_723	passed counts:1
node_0: feature_name=GO:0042113	feature_id[0].value <= threshold=13.408552169799805
node_1: feature_name=GO:0007568	feature_id[534].value <= threshold=5.0313897132873535
node_2: feature_name=GO:0002705	feature_id[541].value <= threshold=3.200145721435547
node_3: feature_name=GO:1901525	feature_id[576].value <= threshold=0.4466460347175598
node_4: feature_name=GO:0048539	feature_id[319].value <= threshold=3.0399646759033203
node_5: feature_name=GO:0001910	feature_id[385].value <= threshold=3.7437864542007446
node_6: feature_name=GO:0043200	feature_id[706].value <= threshold=9.307284355163574
node_7: feature_name=GO:0001773	feature_id[308].value <= threshold=4.016931533813477
node_8: feature_name=GO:0090116	feature_id[97].value <= threshold=7.99645471572876
node_9: feature_name=GO:0019814	feature_id[189].value <= threshold=4.525782108306885
node_10: feature_name=GO:1902583	feature_id[215].value <= threshold=17.60310649871826
node_11: feature_name=GO:0045429	feature_id[737].value <= threshold=1.6133361458778381
node_12: feature_name=GO:0003720	feature_id[228].value <= threshold=5.519326210021973
node_13: feature_name=GO:0046006	feature_id[364].value <= threshold=4.9985432624816895
node_14: feature_name=GO:0070424	feature_id[254].value <= threshold=6.233297109603882
node_15: feature_name=GO:0009892	feature_id[320].value <= threshold=59.99180793762207
node_16: feature_name=GO:0007064	feature_id[527].value <= threshold=11.57377815246582
node_17: feature_name=GO:0005575	feature_id[17].value <= threshold=15.417460918426514
node_18: feature_name=GO:0043368	feature_id[31].value <= threshold=4.059496641159058
node_19: feature_name=GO:0005164	feature_id[163].value <= threshold=5.793607711791992
node_20: feature_name=GO:0042130	feature_id[16].value <= threshold=5.073179721832275
node_21: feature_name=GO:0010216	feature_id[282].value <= threshold=4.834584474563599
node_22: feature_name=GO:0009628	feature_id[553].value <= threshold=29.314892768859863
node_23: feature_name=GO:0045628	feature_id[749].value <= threshold=3.1001367568969727
node_24: feature_name=GO:0042288	feature_id[100].value <= threshold=5.174932956695557
node_25: feature_name=GO:0002329	feature_id[406].value <= threshold=2.348930835723877
node_26: feature_name=GO:0051246	feature_id[642].value > threshold=0.6498909294605255
node_194: feature_name=GO:0009164	feature_id[546].value <= threshold=17.11970329284668
node_195: feature_name=GO:0001909	feature_id[386].value <= threshold=7.292566299438477
node_196: feature_name=GO:0003908	feature_id[11].value <= threshold=1.887226164340973
node_197: feature_name=GO:0009164	feature_id[546].value <= threshold=0.26642198860645294
node_198: feature_name=GO:2001242	feature_id[24].value <= threshold=9.207026481628418
node_199: feature_name=GO:0051246	feature_id[642].value > threshold=0.6504445374011993

node_203: feature_name=GO:0032461 feature_id[608].value <= threshold=2.607542634010315
node_204: feature_name=GO:0007584 feature_id[529].value <= threshold=16.099515914916992
node_205: feature_name=hsa05221 feature_id[349].value <= threshold=9.359850406646729
node_206: feature_name=GO:0002636 feature_id[447].value > threshold=1.615447759628296
node_334: feature_name=GO:0002683 feature_id[456].value <= threshold=0.5395609866827726
Class: positive genes

Rules_724 passed counts:1
node_0: feature_name=GO:0042113 feature_id[0].value <= threshold=13.408552169799805
node_1: feature_name=GO:0007568 feature_id[534].value <= threshold=5.0313897132873535
node_2: feature_name=GO:0002705 feature_id[541].value <= threshold=3.200145721435547
node_3: feature_name=GO:1901525 feature_id[576].value <= threshold=0.4466460347175598
node_4: feature_name=GO:0048539 feature_id[319].value <= threshold=3.0399646759033203
node_5: feature_name=GO:0001910 feature_id[385].value <= threshold=3.7437864542007446
node_6: feature_name=GO:0043200 feature_id[706].value <= threshold=9.307284355163574
node_7: feature_name=GO:0001773 feature_id[308].value <= threshold=4.016931533813477
node_8: feature_name=GO:0090116 feature_id[97].value <= threshold=7.99645471572876
node_9: feature_name=GO:0019814 feature_id[189].value <= threshold=4.525782108306885
node_10: feature_name=GO:1902583 feature_id[215].value <= threshold=17.60310649871826
node_11: feature_name=GO:0045429 feature_id[737].value <= threshold=1.6133361458778381
node_12: feature_name=GO:0003720 feature_id[228].value <= threshold=5.519326210021973
node_13: feature_name=GO:0046006 feature_id[364].value <= threshold=4.9985432624816895
node_14: feature_name=GO:0070424 feature_id[254].value <= threshold=6.233297109603882
node_15: feature_name=GO:0009892 feature_id[320].value <= threshold=59.99180793762207
node_16: feature_name=GO:0007064 feature_id[527].value <= threshold=11.57377815246582
node_17: feature_name=GO:0005575 feature_id[17].value <= threshold=15.417460918426514
node_18: feature_name=GO:0043368 feature_id[31].value <= threshold=4.059496641159058
node_19: feature_name=GO:0005164 feature_id[163].value <= threshold=5.793607711791992
node_20: feature_name=GO:0042130 feature_id[16].value <= threshold=5.073179721832275
node_21: feature_name=GO:0010216 feature_id[282].value <= threshold=4.834584474563599
node_22: feature_name=GO:0009628 feature_id[553].value <= threshold=29.314892768859863
node_23: feature_name=GO:0045628 feature_id[749].value <= threshold=3.1001367568969727
node_24: feature_name=GO:0042288 feature_id[100].value <= threshold=5.174932956695557
node_25: feature_name=GO:0002329 feature_id[406].value <= threshold=2.348930835723877
node_26: feature_name=GO:0051246 feature_id[642].value > threshold=0.6498909294605255

node_194: feature_name=GO:0009164 feature_id[546].value <= threshold=17.11970329284668
node_195: feature_name=GO:0001909 feature_id[386].value <= threshold=7.292566299438477
node_196: feature_name=GO:0003908 feature_id[11].value <= threshold=1.887226164340973
node_197: feature_name=GO:0009164 feature_id[546].value <= threshold=0.26642198860645294
node_198: feature_name=GO:2001242 feature_id[24].value <= threshold=9.207026481628418
node_199: feature_name=GO:0051246 feature_id[642].value > threshold=0.6504445374011993
node_203: feature_name=GO:0032461 feature_id[608].value <= threshold=2.607542634010315
node_204: feature_name=GO:0007584 feature_id[529].value <= threshold=16.099515914916992
node_205: feature_name=hsa05221 feature_id[349].value <= threshold=9.359850406646729
node_206: feature_name=GO:0002636 feature_id[447].value <= threshold=1.615447759628296
node_207: feature_name=GO:0033993 feature_id[421].value > threshold=2.96540611088858e-05
node_217: feature_name=GO:0002331 feature_id[404].value > threshold=2.1883513927459717
node_331: feature_name=GO:0097192 feature_id[515].value <= threshold=0.03834732994437218
Class: positive genes

Rules_725 passed counts:1
node_0: feature_name=GO:0042113 feature_id[0].value <= threshold=13.408552169799805
node_1: feature_name=GO:0007568 feature_id[534].value <= threshold=5.0313897132873535
node_2: feature_name=GO:0002705 feature_id[541].value <= threshold=3.200145721435547
node_3: feature_name=GO:1901525 feature_id[576].value <= threshold=0.4466460347175598
node_4: feature_name=GO:0048539 feature_id[319].value <= threshold=3.0399646759033203
node_5: feature_name=GO:0001910 feature_id[385].value <= threshold=3.7437864542007446
node_6: feature_name=GO:0043200 feature_id[706].value <= threshold=9.307284355163574
node_7: feature_name=GO:0001773 feature_id[308].value <= threshold=4.016931533813477
node_8: feature_name=GO:0090116 feature_id[97].value <= threshold=7.99645471572876
node_9: feature_name=GO:0019814 feature_id[189].value <= threshold=4.525782108306885
node_10: feature_name=GO:1902583 feature_id[215].value <= threshold=17.60310649871826
node_11: feature_name=GO:0045429 feature_id[737].value <= threshold=1.6133361458778381
node_12: feature_name=GO:0003720 feature_id[228].value <= threshold=5.519326210021973
node_13: feature_name=GO:0046006 feature_id[364].value <= threshold=4.9985432624816895
node_14: feature_name=GO:0070424 feature_id[254].value <= threshold=6.233297109603882
node_15: feature_name=GO:0009892 feature_id[320].value <= threshold=59.99180793762207
node_16: feature_name=GO:0007064 feature_id[527].value <= threshold=11.57377815246582
node_17: feature_name=GO:0005575 feature_id[17].value <= threshold=15.417460918426514
node_18: feature_name=GO:0043368 feature_id[31].value <= threshold=4.059496641159058

node_19: feature_name=GO:0005164 feature_id[163].value <= threshold=5.793607711791992
node_20: feature_name=GO:0042130 feature_id[16].value <= threshold=5.073179721832275
node_21: feature_name=GO:0010216 feature_id[282].value <= threshold=4.834584474563599
node_22: feature_name=GO:0009628 feature_id[553].value <= threshold=29.314892768859863
node_23: feature_name=GO:0045628 feature_id[749].value <= threshold=3.1001367568969727
node_24: feature_name=GO:0042288 feature_id[100].value <= threshold=5.174932956695557
node_25: feature_name=GO:0002329 feature_id[406].value <= threshold=2.348930835723877
node_26: feature_name=GO:0051246 feature_id[642].value > threshold=0.6498909294605255
node_194: feature_name=GO:0009164 feature_id[546].value <= threshold=17.11970329284668
node_195: feature_name=GO:0001909 feature_id[386].value <= threshold=7.292566299438477
node_196: feature_name=GO:0003908 feature_id[11].value <= threshold=1.887226164340973
node_197: feature_name=GO:0009164 feature_id[546].value <= threshold=0.26642198860645294
node_198: feature_name=GO:2001242 feature_id[24].value <= threshold=9.207026481628418
node_199: feature_name=GO:0051246 feature_id[642].value > threshold=0.6504445374011993
node_203: feature_name=GO:0032461 feature_id[608].value <= threshold=2.607542634010315
node_204: feature_name=GO:0007584 feature_id[529].value <= threshold=16.099515914916992
node_205: feature_name=hsa05221 feature_id[349].value <= threshold=9.359850406646729
node_206: feature_name=GO:0002636 feature_id[447].value <= threshold=1.615447759628296
node_207: feature_name=GO:0033993 feature_id[421].value > threshold=2.96540611088858e-05
node_217: feature_name=GO:0002331 feature_id[404].value <= threshold=2.1883513927459717
node_218: feature_name=GO:0046498 feature_id[203].value > threshold=4.085246205329895
node_328: feature_name=GO:0071706 feature_id[552].value <= threshold=0.21486687660217285
Class: positive genes

Rules_726 passed counts:1
node_0: feature_name=GO:0042113 feature_id[0].value <= threshold=13.408552169799805
node_1: feature_name=GO:0007568 feature_id[534].value <= threshold=5.0313897132873535
node_2: feature_name=GO:0002705 feature_id[541].value <= threshold=3.200145721435547
node_3: feature_name=GO:1901525 feature_id[576].value <= threshold=0.4466460347175598
node_4: feature_name=GO:0048539 feature_id[319].value <= threshold=3.0399646759033203
node_5: feature_name=GO:0001910 feature_id[385].value <= threshold=3.7437864542007446
node_6: feature_name=GO:0043200 feature_id[706].value <= threshold=9.307284355163574
node_7: feature_name=GO:0001773 feature_id[308].value <= threshold=4.016931533813477
node_8: feature_name=GO:0090116 feature_id[97].value <= threshold=7.99645471572876
node_9: feature_name=GO:0019814 feature_id[189].value <= threshold=4.525782108306885

node_10: feature_name=GO:1902583 feature_id[215].value <= threshold=17.60310649871826
node_11: feature_name=GO:0045429 feature_id[737].value <= threshold=1.6133361458778381
node_12: feature_name=GO:0003720 feature_id[228].value <= threshold=5.519326210021973
node_13: feature_name=GO:0046006 feature_id[364].value <= threshold=4.9985432624816895
node_14: feature_name=GO:0070424 feature_id[254].value <= threshold=6.233297109603882
node_15: feature_name=GO:0009892 feature_id[320].value <= threshold=59.99180793762207
node_16: feature_name=GO:0007064 feature_id[527].value <= threshold=11.57377815246582
node_17: feature_name=GO:0005575 feature_id[17].value <= threshold=15.417460918426514
node_18: feature_name=GO:0043368 feature_id[31].value <= threshold=4.059496641159058
node_19: feature_name=GO:0005164 feature_id[163].value <= threshold=5.793607711791992
node_20: feature_name=GO:0042130 feature_id[16].value <= threshold=5.073179721832275
node_21: feature_name=GO:0010216 feature_id[282].value <= threshold=4.834584474563599
node_22: feature_name=GO:0009628 feature_id[553].value <= threshold=29.314892768859863
node_23: feature_name=GO:0045628 feature_id[749].value <= threshold=3.1001367568969727
node_24: feature_name=GO:0042288 feature_id[100].value <= threshold=5.174932956695557
node_25: feature_name=GO:0002329 feature_id[406].value <= threshold=2.348930835723877
node_26: feature_name=GO:0051246 feature_id[642].value > threshold=0.6498909294605255
node_194: feature_name=GO:0009164 feature_id[546].value <= threshold=17.11970329284668
node_195: feature_name=GO:0001909 feature_id[386].value <= threshold=7.292566299438477
node_196: feature_name=GO:0003908 feature_id[11].value <= threshold=1.887226164340973
node_197: feature_name=GO:0009164 feature_id[546].value <= threshold=0.26642198860645294
node_198: feature_name=GO:2001242 feature_id[24].value <= threshold=9.207026481628418
node_199: feature_name=GO:0051246 feature_id[642].value > threshold=0.6504445374011993
node_203: feature_name=GO:0032461 feature_id[608].value <= threshold=2.607542634010315
node_204: feature_name=GO:0007584 feature_id[529].value <= threshold=16.099515914916992
node_205: feature_name=hsa05221 feature_id[349].value <= threshold=9.359850406646729
node_206: feature_name=GO:0002636 feature_id[447].value <= threshold=1.615447759628296
node_207: feature_name=GO:0033993 feature_id[421].value > threshold=2.96540611088858e-05
node_217: feature_name=GO:0002331 feature_id[404].value <= threshold=2.1883513927459717
node_218: feature_name=GO:0046498 feature_id[203].value <= threshold=4.085246205329895
node_219: feature_name=GO:0002832 feature_id[491].value > threshold=3.0228612422943115
node_325: feature_name=GO:0002715 feature_id[484].value > threshold=0.8928120732307434
Class: positive genes

Rules_727 passed counts:1

node_0: feature_name=GO:0042113	feature_id[0].value <= threshold=13.408552169799805
node_1: feature_name=GO:0007568	feature_id[534].value <= threshold=5.0313897132873535
node_2: feature_name=GO:0002705	feature_id[541].value <= threshold=3.200145721435547
node_3: feature_name=GO:1901525	feature_id[576].value <= threshold=0.4466460347175598
node_4: feature_name=GO:0048539	feature_id[319].value <= threshold=3.0399646759033203
node_5: feature_name=GO:0001910	feature_id[385].value <= threshold=3.7437864542007446
node_6: feature_name=GO:0043200	feature_id[706].value <= threshold=9.307284355163574
node_7: feature_name=GO:0001773	feature_id[308].value <= threshold=4.016931533813477
node_8: feature_name=GO:0090116	feature_id[97].value <= threshold=7.99645471572876
node_9: feature_name=GO:0019814	feature_id[189].value <= threshold=4.525782108306885
node_10: feature_name=GO:1902583	feature_id[215].value <= threshold=17.60310649871826
node_11: feature_name=GO:0045429	feature_id[737].value <= threshold=1.6133361458778381
node_12: feature_name=GO:0003720	feature_id[228].value <= threshold=5.519326210021973
node_13: feature_name=GO:0046006	feature_id[364].value <= threshold=4.9985432624816895
node_14: feature_name=GO:0070424	feature_id[254].value <= threshold=6.233297109603882
node_15: feature_name=GO:0009892	feature_id[320].value <= threshold=59.99180793762207
node_16: feature_name=GO:0007064	feature_id[527].value <= threshold=11.57377815246582
node_17: feature_name=GO:0005575	feature_id[17].value <= threshold=15.417460918426514
node_18: feature_name=GO:0043368	feature_id[31].value <= threshold=4.059496641159058
node_19: feature_name=GO:0005164	feature_id[163].value <= threshold=5.793607711791992
node_20: feature_name=GO:0042130	feature_id[16].value <= threshold=5.073179721832275
node_21: feature_name=GO:0010216	feature_id[282].value <= threshold=4.834584474563599
node_22: feature_name=GO:0009628	feature_id[553].value <= threshold=29.314892768859863
node_23: feature_name=GO:0045628	feature_id[749].value <= threshold=3.1001367568969727
node_24: feature_name=GO:0042288	feature_id[100].value <= threshold=5.174932956695557
node_25: feature_name=GO:0002329	feature_id[406].value <= threshold=2.348930835723877
node_26: feature_name=GO:0051246	feature_id[642].value > threshold=0.6498909294605255
node_194: feature_name=GO:0009164	feature_id[546].value <= threshold=17.11970329284668
node_195: feature_name=GO:0001909	feature_id[386].value <= threshold=7.292566299438477
node_196: feature_name=GO:0003908	feature_id[11].value <= threshold=1.887226164340973
node_197: feature_name=GO:0009164	feature_id[546].value <= threshold=0.26642198860645294
node_198: feature_name=GO:2001242	feature_id[24].value <= threshold=9.207026481628418
node_199: feature_name=GO:0051246	feature_id[642].value > threshold=0.6504445374011993
node_203: feature_name=GO:0032461	feature_id[608].value <= threshold=2.607542634010315
node_204: feature_name=GO:0007584	feature_id[529].value <= threshold=16.099515914916992

node_205: feature_name=hsa05221	feature_id[349].value <= threshold=9.359850406646729
node_206: feature_name=GO:0002636	feature_id[447].value <= threshold=1.615447759628296
node_207: feature_name=GO:0033993	feature_id[421].value > threshold=2.96540611088858e-05
node_217: feature_name=GO:0002331	feature_id[404].value <= threshold=2.1883513927459717
node_218: feature_name=GO:0046498	feature_id[203].value <= threshold=4.085246205329895
node_219: feature_name=GO:0002832	feature_id[491].value <= threshold=3.0228612422943115
node_220: feature_name=GO:1904868	feature_id[306].value > threshold=3.8674756288528442
node_322: feature_name=GO:0045945	feature_id[267].value > threshold=1.61331245303154
Class: positive genes	

Rules_728	passed counts:1
node_0: feature_name=GO:0042113	feature_id[0].value <= threshold=13.408552169799805
node_1: feature_name=GO:0007568	feature_id[534].value <= threshold=5.0313897132873535
node_2: feature_name=GO:0002705	feature_id[541].value <= threshold=3.200145721435547
node_3: feature_name=GO:1901525	feature_id[576].value <= threshold=0.4466460347175598
node_4: feature_name=GO:0048539	feature_id[319].value <= threshold=3.0399646759033203
node_5: feature_name=GO:0001910	feature_id[385].value <= threshold=3.7437864542007446
node_6: feature_name=GO:0043200	feature_id[706].value <= threshold=9.307284355163574
node_7: feature_name=GO:0001773	feature_id[308].value <= threshold=4.016931533813477
node_8: feature_name=GO:0090116	feature_id[97].value <= threshold=7.99645471572876
node_9: feature_name=GO:0019814	feature_id[189].value <= threshold=4.525782108306885
node_10: feature_name=GO:1902583	feature_id[215].value <= threshold=17.60310649871826
node_11: feature_name=GO:0045429	feature_id[737].value <= threshold=1.6133361458778381
node_12: feature_name=GO:0003720	feature_id[228].value <= threshold=5.519326210021973
node_13: feature_name=GO:0046006	feature_id[364].value <= threshold=4.9985432624816895
node_14: feature_name=GO:0070424	feature_id[254].value <= threshold=6.233297109603882
node_15: feature_name=GO:0009892	feature_id[320].value <= threshold=59.99180793762207
node_16: feature_name=GO:0007064	feature_id[527].value <= threshold=11.57377815246582
node_17: feature_name=GO:0005575	feature_id[17].value <= threshold=15.417460918426514
node_18: feature_name=GO:0043368	feature_id[31].value <= threshold=4.059496641159058
node_19: feature_name=GO:0005164	feature_id[163].value <= threshold=5.793607711791992
node_20: feature_name=GO:0042130	feature_id[16].value <= threshold=5.073179721832275
node_21: feature_name=GO:0010216	feature_id[282].value <= threshold=4.834584474563599
node_22: feature_name=GO:0009628	feature_id[553].value <= threshold=29.314892768859863
node_23: feature_name=GO:0045628	feature_id[749].value <= threshold=3.1001367568969727

node_24: feature_name=GO:0042288	feature_id[100].value <= threshold=5.174932956695557
node_25: feature_name=GO:0002329	feature_id[406].value <= threshold=2.348930835723877
node_26: feature_name=GO:0051246	feature_id[642].value > threshold=0.6498909294605255
node_194: feature_name=GO:0009164	feature_id[546].value <= threshold=17.11970329284668
node_195: feature_name=GO:0001909	feature_id[386].value <= threshold=7.292566299438477
node_196: feature_name=GO:0003908	feature_id[11].value <= threshold=1.887226164340973
node_197: feature_name=GO:0009164	feature_id[546].value <= threshold=0.26642198860645294
node_198: feature_name=GO:2001242	feature_id[24].value <= threshold=9.207026481628418
node_199: feature_name=GO:0051246	feature_id[642].value > threshold=0.6504445374011993
node_203: feature_name=GO:0032461	feature_id[608].value <= threshold=2.607542634010315
node_204: feature_name=GO:0007584	feature_id[529].value <= threshold=16.099515914916992
node_205: feature_name=hsa05221	feature_id[349].value <= threshold=9.359850406646729
node_206: feature_name=GO:0002636	feature_id[447].value <= threshold=1.615447759628296
node_207: feature_name=GO:0033993	feature_id[421].value > threshold=2.96540611088858e-05
node_217: feature_name=GO:0002331	feature_id[404].value <= threshold=2.1883513927459717
node_218: feature_name=GO:0046498	feature_id[203].value <= threshold=4.085246205329895
node_219: feature_name=GO:0002832	feature_id[491].value <= threshold=3.0228612422943115
node_220: feature_name=GO:1904868	feature_id[306].value <= threshold=3.8674756288528442
node_221: feature_name=GO:0048545	feature_id[360].value > threshold=27.38136100769043
node_319: feature_name=GO:0047485	feature_id[121].value > threshold=1.0277213156223297
Class: positive genes	
Rules_729	passed counts:1
node_0: feature_name=GO:0042113	feature_id[0].value <= threshold=13.408552169799805
node_1: feature_name=GO:0007568	feature_id[534].value <= threshold=5.0313897132873535
node_2: feature_name=GO:0002705	feature_id[541].value <= threshold=3.200145721435547
node_3: feature_name=GO:1901525	feature_id[576].value <= threshold=0.4466460347175598
node_4: feature_name=GO:0048539	feature_id[319].value <= threshold=3.0399646759033203
node_5: feature_name=GO:0001910	feature_id[385].value <= threshold=3.7437864542007446
node_6: feature_name=GO:0043200	feature_id[706].value <= threshold=9.307284355163574
node_7: feature_name=GO:0001773	feature_id[308].value <= threshold=4.016931533813477
node_8: feature_name=GO:0090116	feature_id[97].value <= threshold=7.99645471572876
node_9: feature_name=GO:0019814	feature_id[189].value <= threshold=4.525782108306885
node_10: feature_name=GO:1902583	feature_id[215].value <= threshold=17.60310649871826
node_11: feature_name=GO:0045429	feature_id[737].value <= threshold=1.6133361458778381

node_12: feature_name=GO:0003720	feature_id[228].value <= threshold=5.519326210021973
node_13: feature_name=GO:0046006	feature_id[364].value <= threshold=4.9985432624816895
node_14: feature_name=GO:0070424	feature_id[254].value <= threshold=6.233297109603882
node_15: feature_name=GO:0009892	feature_id[320].value <= threshold=59.99180793762207
node_16: feature_name=GO:0007064	feature_id[527].value <= threshold=11.57377815246582
node_17: feature_name=GO:0005575	feature_id[17].value <= threshold=15.417460918426514
node_18: feature_name=GO:0043368	feature_id[31].value <= threshold=4.059496641159058
node_19: feature_name=GO:0005164	feature_id[163].value <= threshold=5.793607711791992
node_20: feature_name=GO:0042130	feature_id[16].value <= threshold=5.073179721832275
node_21: feature_name=GO:0010216	feature_id[282].value <= threshold=4.834584474563599
node_22: feature_name=GO:0009628	feature_id[553].value <= threshold=29.314892768859863
node_23: feature_name=GO:0045628	feature_id[749].value <= threshold=3.1001367568969727
node_24: feature_name=GO:0042288	feature_id[100].value <= threshold=5.174932956695557
node_25: feature_name=GO:0002329	feature_id[406].value <= threshold=2.348930835723877
node_26: feature_name=GO:0051246	feature_id[642].value > threshold=0.6498909294605255
node_194: feature_name=GO:0009164	feature_id[546].value <= threshold=17.11970329284668
node_195: feature_name=GO:0001909	feature_id[386].value <= threshold=7.292566299438477
node_196: feature_name=GO:0003908	feature_id[11].value <= threshold=1.887226164340973
node_197: feature_name=GO:0009164	feature_id[546].value <= threshold=0.26642198860645294
node_198: feature_name=GO:2001242	feature_id[24].value <= threshold=9.207026481628418
node_199: feature_name=GO:0051246	feature_id[642].value > threshold=0.6504445374011993
node_203: feature_name=GO:0032461	feature_id[608].value <= threshold=2.607542634010315
node_204: feature_name=GO:0007584	feature_id[529].value <= threshold=16.099515914916992
node_205: feature_name=hsa05221	feature_id[349].value <= threshold=9.359850406646729
node_206: feature_name=GO:0002636	feature_id[447].value <= threshold=1.615447759628296
node_207: feature_name=GO:0033993	feature_id[421].value > threshold=2.96540611088858e-05
node_217: feature_name=GO:0002331	feature_id[404].value <= threshold=2.1883513927459717
node_218: feature_name=GO:0046498	feature_id[203].value <= threshold=4.085246205329895
node_219: feature_name=GO:0002832	feature_id[491].value <= threshold=3.0228612422943115
node_220: feature_name=GO:1904868	feature_id[306].value <= threshold=3.8674756288528442
node_221: feature_name=GO:0048545	feature_id[360].value <= threshold=27.38136100769043
node_222: feature_name=GO:0071310	feature_id[760].value > threshold=2.3522024154663086
node_306: feature_name=GO:0070233	feature_id[321].value <= threshold=1.293377935886383
node_307: feature_name=hsa05221	feature_id[349].value > threshold=9.005411148071289
node_313: feature_name=GO:0008340	feature_id[539].value > threshold=1.3541167378425598

Class: positive genes

Rules_730	passed counts:1
node_0: feature_name=GO:0042113	feature_id[0].value <= threshold=13.408552169799805
node_1: feature_name=GO:0007568	feature_id[534].value <= threshold=5.0313897132873535
node_2: feature_name=GO:0002705	feature_id[541].value <= threshold=3.200145721435547
node_3: feature_name=GO:1901525	feature_id[576].value <= threshold=0.4466460347175598
node_4: feature_name=GO:0048539	feature_id[319].value <= threshold=3.0399646759033203
node_5: feature_name=GO:0001910	feature_id[385].value <= threshold=3.7437864542007446
node_6: feature_name=GO:0043200	feature_id[706].value <= threshold=9.307284355163574
node_7: feature_name=GO:0001773	feature_id[308].value <= threshold=4.016931533813477
node_8: feature_name=GO:0090116	feature_id[97].value <= threshold=7.99645471572876
node_9: feature_name=GO:0019814	feature_id[189].value <= threshold=4.525782108306885
node_10: feature_name=GO:1902583	feature_id[215].value <= threshold=17.60310649871826
node_11: feature_name=GO:0045429	feature_id[737].value <= threshold=1.6133361458778381
node_12: feature_name=GO:0003720	feature_id[228].value <= threshold=5.519326210021973
node_13: feature_name=GO:0046006	feature_id[364].value <= threshold=4.9985432624816895
node_14: feature_name=GO:0070424	feature_id[254].value <= threshold=6.233297109603882
node_15: feature_name=GO:0009892	feature_id[320].value <= threshold=59.99180793762207
node_16: feature_name=GO:0007064	feature_id[527].value <= threshold=11.57377815246582
node_17: feature_name=GO:0005575	feature_id[17].value <= threshold=15.417460918426514
node_18: feature_name=GO:0043368	feature_id[31].value <= threshold=4.059496641159058
node_19: feature_name=GO:0005164	feature_id[163].value <= threshold=5.793607711791992
node_20: feature_name=GO:0042130	feature_id[16].value <= threshold=5.073179721832275
node_21: feature_name=GO:0010216	feature_id[282].value <= threshold=4.834584474563599
node_22: feature_name=GO:0009628	feature_id[553].value <= threshold=29.314892768859863
node_23: feature_name=GO:0045628	feature_id[749].value <= threshold=3.1001367568969727
node_24: feature_name=GO:0042288	feature_id[100].value <= threshold=5.174932956695557
node_25: feature_name=GO:0002329	feature_id[406].value <= threshold=2.348930835723877
node_26: feature_name=GO:0051246	feature_id[642].value > threshold=0.6498909294605255
node_194: feature_name=GO:0009164	feature_id[546].value <= threshold=17.11970329284668
node_195: feature_name=GO:0001909	feature_id[386].value <= threshold=7.292566299438477
node_196: feature_name=GO:0003908	feature_id[11].value <= threshold=1.887226164340973
node_197: feature_name=GO:0009164	feature_id[546].value <= threshold=0.26642198860645294
node_198: feature_name=GO:2001242	feature_id[24].value <= threshold=9.207026481628418

node_199: feature_name=GO:0051246 feature_id[642].value > threshold=0.6504445374011993
node_203: feature_name=GO:0032461 feature_id[608].value <= threshold=2.607542634010315
node_204: feature_name=GO:0007584 feature_id[529].value <= threshold=16.099515914916992
node_205: feature_name=hsa05221 feature_id[349].value <= threshold=9.359850406646729
node_206: feature_name=GO:0002636 feature_id[447].value <= threshold=1.615447759628296
node_207: feature_name=GO:0033993 feature_id[421].value > threshold=2.96540611088858e-05
node_217: feature_name=GO:0002331 feature_id[404].value <= threshold=2.1883513927459717
node_218: feature_name=GO:0046498 feature_id[203].value <= threshold=4.085246205329895
node_219: feature_name=GO:0002832 feature_id[491].value <= threshold=3.0228612422943115
node_220: feature_name=GO:1904868 feature_id[306].value <= threshold=3.8674756288528442
node_221: feature_name=GO:0048545 feature_id[360].value <= threshold=27.38136100769043
node_222: feature_name=GO:0071310 feature_id[760].value > threshold=2.3522024154663086
node_306: feature_name=GO:0070233 feature_id[321].value <= threshold=1.293377935886383
node_307: feature_name=hsa05221 feature_id[349].value <= threshold=9.005411148071289
node_308: feature_name=GO:0032770 feature_id[604].value > threshold=3.2187339067459106
node_310: feature_name=GO:0002902 feature_id[59].value > threshold=1.6938291788101196
Class: positive genes

Rules_731 passed counts:1
node_0: feature_name=GO:0042113 feature_id[0].value <= threshold=13.408552169799805
node_1: feature_name=GO:0007568 feature_id[534].value <= threshold=5.0313897132873535
node_2: feature_name=GO:0002705 feature_id[541].value <= threshold=3.200145721435547
node_3: feature_name=GO:1901525 feature_id[576].value <= threshold=0.4466460347175598
node_4: feature_name=GO:0048539 feature_id[319].value <= threshold=3.0399646759033203
node_5: feature_name=GO:0001910 feature_id[385].value <= threshold=3.7437864542007446
node_6: feature_name=GO:0043200 feature_id[706].value <= threshold=9.307284355163574
node_7: feature_name=GO:0001773 feature_id[308].value <= threshold=4.016931533813477
node_8: feature_name=GO:0090116 feature_id[97].value <= threshold=7.99645471572876
node_9: feature_name=GO:0019814 feature_id[189].value <= threshold=4.525782108306885
node_10: feature_name=GO:1902583 feature_id[215].value <= threshold=17.60310649871826
node_11: feature_name=GO:0045429 feature_id[737].value <= threshold=1.6133361458778381
node_12: feature_name=GO:0003720 feature_id[228].value <= threshold=5.519326210021973
node_13: feature_name=GO:0046006 feature_id[364].value <= threshold=4.9985432624816895
node_14: feature_name=GO:0070424 feature_id[254].value <= threshold=6.233297109603882
node_15: feature_name=GO:0009892 feature_id[320].value <= threshold=59.99180793762207

node_16: feature_name=GO:0007064 feature_id[527].value <= threshold=11.57377815246582
node_17: feature_name=GO:0005575 feature_id[17].value <= threshold=15.417460918426514
node_18: feature_name=GO:0043368 feature_id[31].value <= threshold=4.059496641159058
node_19: feature_name=GO:0005164 feature_id[163].value <= threshold=5.793607711791992
node_20: feature_name=GO:0042130 feature_id[16].value <= threshold=5.073179721832275
node_21: feature_name=GO:0010216 feature_id[282].value <= threshold=4.834584474563599
node_22: feature_name=GO:0009628 feature_id[553].value <= threshold=29.314892768859863
node_23: feature_name=GO:0045628 feature_id[749].value <= threshold=3.1001367568969727
node_24: feature_name=GO:0042288 feature_id[100].value <= threshold=5.174932956695557
node_25: feature_name=GO:0002329 feature_id[406].value <= threshold=2.348930835723877
node_26: feature_name=GO:0051246 feature_id[642].value > threshold=0.6498909294605255
node_194: feature_name=GO:0009164 feature_id[546].value <= threshold=17.11970329284668
node_195: feature_name=GO:0001909 feature_id[386].value <= threshold=7.292566299438477
node_196: feature_name=GO:0003908 feature_id[11].value <= threshold=1.887226164340973
node_197: feature_name=GO:0009164 feature_id[546].value <= threshold=0.26642198860645294
node_198: feature_name=GO:2001242 feature_id[24].value <= threshold=9.207026481628418
node_199: feature_name=GO:0051246 feature_id[642].value > threshold=0.6504445374011993
node_203: feature_name=GO:0032461 feature_id[608].value <= threshold=2.607542634010315
node_204: feature_name=GO:0007584 feature_id[529].value <= threshold=16.099515914916992
node_205: feature_name=hsa05221 feature_id[349].value <= threshold=9.359850406646729
node_206: feature_name=GO:0002636 feature_id[447].value <= threshold=1.615447759628296
node_207: feature_name=GO:0033993 feature_id[421].value > threshold=2.96540611088858e-05
node_217: feature_name=GO:0002331 feature_id[404].value <= threshold=2.1883513927459717
node_218: feature_name=GO:0046498 feature_id[203].value <= threshold=4.085246205329895
node_219: feature_name=GO:0002832 feature_id[491].value <= threshold=3.0228612422943115
node_220: feature_name=GO:1904868 feature_id[306].value <= threshold=3.8674756288528442
node_221: feature_name=GO:0048545 feature_id[360].value <= threshold=27.38136100769043
node_222: feature_name=GO:0071310 feature_id[760].value <= threshold=2.3522024154663086
node_223: feature_name=GO:0001775 feature_id[505].value > threshold=9.71301555633545
Class: positive genes

Rules_732 passed counts:1
node_0: feature_name=GO:0042113 feature_id[0].value <= threshold=13.408552169799805
node_1: feature_name=GO:0007568 feature_id[534].value <= threshold=5.0313897132873535
node_2: feature_name=GO:0002705 feature_id[541].value <= threshold=3.200145721435547

node_3: feature_name=GO:1901525	feature_id[576].value <= threshold=0.4466460347175598
node_4: feature_name=GO:0048539	feature_id[319].value <= threshold=3.0399646759033203
node_5: feature_name=GO:0001910	feature_id[385].value <= threshold=3.7437864542007446
node_6: feature_name=GO:0043200	feature_id[706].value <= threshold=9.307284355163574
node_7: feature_name=GO:0001773	feature_id[308].value <= threshold=4.016931533813477
node_8: feature_name=GO:0090116	feature_id[97].value <= threshold=7.99645471572876
node_9: feature_name=GO:0019814	feature_id[189].value <= threshold=4.525782108306885
node_10: feature_name=GO:1902583	feature_id[215].value <= threshold=17.60310649871826
node_11: feature_name=GO:0045429	feature_id[737].value <= threshold=1.6133361458778381
node_12: feature_name=GO:0003720	feature_id[228].value <= threshold=5.519326210021973
node_13: feature_name=GO:0046006	feature_id[364].value <= threshold=4.9985432624816895
node_14: feature_name=GO:0070424	feature_id[254].value <= threshold=6.233297109603882
node_15: feature_name=GO:0009892	feature_id[320].value <= threshold=59.99180793762207
node_16: feature_name=GO:0007064	feature_id[527].value <= threshold=11.57377815246582
node_17: feature_name=GO:0005575	feature_id[17].value <= threshold=15.417460918426514
node_18: feature_name=GO:0043368	feature_id[31].value <= threshold=4.059496641159058
node_19: feature_name=GO:0005164	feature_id[163].value <= threshold=5.793607711791992
node_20: feature_name=GO:0042130	feature_id[16].value <= threshold=5.073179721832275
node_21: feature_name=GO:0010216	feature_id[282].value <= threshold=4.834584474563599
node_22: feature_name=GO:0009628	feature_id[553].value <= threshold=29.314892768859863
node_23: feature_name=GO:0045628	feature_id[749].value <= threshold=3.1001367568969727
node_24: feature_name=GO:0042288	feature_id[100].value <= threshold=5.174932956695557
node_25: feature_name=GO:0002329	feature_id[406].value <= threshold=2.348930835723877
node_26: feature_name=GO:0051246	feature_id[642].value > threshold=0.6498909294605255
node_194: feature_name=GO:0009164	feature_id[546].value <= threshold=17.11970329284668
node_195: feature_name=GO:0001909	feature_id[386].value <= threshold=7.292566299438477
node_196: feature_name=GO:0003908	feature_id[11].value <= threshold=1.887226164340973
node_197: feature_name=GO:0009164	feature_id[546].value <= threshold=0.26642198860645294
node_198: feature_name=GO:2001242	feature_id[24].value <= threshold=9.207026481628418
node_199: feature_name=GO:0051246	feature_id[642].value > threshold=0.6504445374011993
node_203: feature_name=GO:0032461	feature_id[608].value <= threshold=2.607542634010315
node_204: feature_name=GO:0007584	feature_id[529].value <= threshold=16.099515914916992
node_205: feature_name=hsa05221	feature_id[349].value <= threshold=9.359850406646729
node_206: feature_name=GO:0002636	feature_id[447].value <= threshold=1.615447759628296
node_207: feature_name=GO:0033993	feature_id[421].value > threshold=2.96540611088858e-05

node_217: feature_name=GO:0002331 feature_id[404].value <= threshold=2.1883513927459717
node_218: feature_name=GO:0046498 feature_id[203].value <= threshold=4.085246205329895
node_219: feature_name=GO:0002832 feature_id[491].value <= threshold=3.0228612422943115
node_220: feature_name=GO:1904868 feature_id[306].value <= threshold=3.8674756288528442
node_221: feature_name=GO:0048545 feature_id[360].value <= threshold=27.38136100769043
node_222: feature_name=GO:0071310 feature_id[760].value <= threshold=2.3522024154663086
node_223: feature_name=GO:0001775 feature_id[505].value <= threshold=9.71301555633545
node_224: feature_name=GO:0071310 feature_id[760].value > threshold=2.346743583679199
Class: positive genes

Rules_733 passed counts:1
node_0: feature_name=GO:0042113 feature_id[0].value <= threshold=13.408552169799805
node_1: feature_name=GO:0007568 feature_id[534].value <= threshold=5.0313897132873535
node_2: feature_name=GO:0002705 feature_id[541].value <= threshold=3.200145721435547
node_3: feature_name=GO:1901525 feature_id[576].value <= threshold=0.4466460347175598
node_4: feature_name=GO:0048539 feature_id[319].value <= threshold=3.0399646759033203
node_5: feature_name=GO:0001910 feature_id[385].value <= threshold=3.7437864542007446
node_6: feature_name=GO:0043200 feature_id[706].value <= threshold=9.307284355163574
node_7: feature_name=GO:0001773 feature_id[308].value <= threshold=4.016931533813477
node_8: feature_name=GO:0090116 feature_id[97].value <= threshold=7.99645471572876
node_9: feature_name=GO:0019814 feature_id[189].value <= threshold=4.525782108306885
node_10: feature_name=GO:1902583 feature_id[215].value <= threshold=17.60310649871826
node_11: feature_name=GO:0045429 feature_id[737].value <= threshold=1.6133361458778381
node_12: feature_name=GO:0003720 feature_id[228].value <= threshold=5.519326210021973
node_13: feature_name=GO:0046006 feature_id[364].value <= threshold=4.9985432624816895
node_14: feature_name=GO:0070424 feature_id[254].value <= threshold=6.233297109603882
node_15: feature_name=GO:0009892 feature_id[320].value <= threshold=59.99180793762207
node_16: feature_name=GO:0007064 feature_id[527].value <= threshold=11.57377815246582
node_17: feature_name=GO:0005575 feature_id[17].value <= threshold=15.417460918426514
node_18: feature_name=GO:0043368 feature_id[31].value <= threshold=4.059496641159058
node_19: feature_name=GO:0005164 feature_id[163].value <= threshold=5.793607711791992
node_20: feature_name=GO:0042130 feature_id[16].value <= threshold=5.073179721832275
node_21: feature_name=GO:0010216 feature_id[282].value <= threshold=4.834584474563599
node_22: feature_name=GO:0009628 feature_id[553].value <= threshold=29.314892768859863
node_23: feature_name=GO:0045628 feature_id[749].value <= threshold=3.1001367568969727

node_24: feature_name=GO:0042288 feature_id[100].value <= threshold=5.174932956695557
node_25: feature_name=GO:0002329 feature_id[406].value <= threshold=2.348930835723877
node_26: feature_name=GO:0051246 feature_id[642].value > threshold=0.6498909294605255
node_194: feature_name=GO:0009164 feature_id[546].value <= threshold=17.11970329284668
node_195: feature_name=GO:0001909 feature_id[386].value <= threshold=7.292566299438477
node_196: feature_name=GO:0003908 feature_id[11].value <= threshold=1.887226164340973
node_197: feature_name=GO:0009164 feature_id[546].value <= threshold=0.26642198860645294
node_198: feature_name=GO:2001242 feature_id[24].value <= threshold=9.207026481628418
node_199: feature_name=GO:0051246 feature_id[642].value > threshold=0.6504445374011993
node_203: feature_name=GO:0032461 feature_id[608].value <= threshold=2.607542634010315
node_204: feature_name=GO:0007584 feature_id[529].value <= threshold=16.099515914916992
node_205: feature_name=hsa05221 feature_id[349].value <= threshold=9.359850406646729
node_206: feature_name=GO:0002636 feature_id[447].value <= threshold=1.615447759628296
node_207: feature_name=GO:0033993 feature_id[421].value > threshold=2.96540611088858e-05
node_217: feature_name=GO:0002331 feature_id[404].value <= threshold=2.1883513927459717
node_218: feature_name=GO:0046498 feature_id[203].value <= threshold=4.085246205329895
node_219: feature_name=GO:0002832 feature_id[491].value <= threshold=3.0228612422943115
node_220: feature_name=GO:1904868 feature_id[306].value <= threshold=3.8674756288528442
node_221: feature_name=GO:0048545 feature_id[360].value <= threshold=27.38136100769043
node_222: feature_name=GO:0071310 feature_id[760].value <= threshold=2.3522024154663086
node_223: feature_name=GO:0001775 feature_id[505].value <= threshold=9.71301555633545
node_224: feature_name=GO:0071310 feature_id[760].value <= threshold=2.346743583679199
node_225: feature_name=GO:0001772 feature_id[91].value > threshold=3.252573609352112
node_301: feature_name=GO:0060249 feature_id[390].value > threshold=1.9239138662815094
Class: positive genes

Rules_734 passed counts:1
node_0: feature_name=GO:0042113 feature_id[0].value <= threshold=13.408552169799805
node_1: feature_name=GO:0007568 feature_id[534].value <= threshold=5.0313897132873535
node_2: feature_name=GO:0002705 feature_id[541].value <= threshold=3.200145721435547
node_3: feature_name=GO:1901525 feature_id[576].value <= threshold=0.4466460347175598
node_4: feature_name=GO:0048539 feature_id[319].value <= threshold=3.0399646759033203
node_5: feature_name=GO:0001910 feature_id[385].value <= threshold=3.7437864542007446
node_6: feature_name=GO:0043200 feature_id[706].value <= threshold=9.307284355163574
node_7: feature_name=GO:0001773 feature_id[308].value <= threshold=4.016931533813477

node_8: feature_name=GO:0090116	feature_id[97].value <= threshold=7.99645471572876
node_9: feature_name=GO:0019814	feature_id[189].value <= threshold=4.525782108306885
node_10: feature_name=GO:1902583	feature_id[215].value <= threshold=17.60310649871826
node_11: feature_name=GO:0045429	feature_id[737].value <= threshold=1.6133361458778381
node_12: feature_name=GO:0003720	feature_id[228].value <= threshold=5.519326210021973
node_13: feature_name=GO:0046006	feature_id[364].value <= threshold=4.9985432624816895
node_14: feature_name=GO:0070424	feature_id[254].value <= threshold=6.233297109603882
node_15: feature_name=GO:0009892	feature_id[320].value <= threshold=59.99180793762207
node_16: feature_name=GO:0007064	feature_id[527].value <= threshold=11.57377815246582
node_17: feature_name=GO:0005575	feature_id[17].value <= threshold=15.417460918426514
node_18: feature_name=GO:0043368	feature_id[31].value <= threshold=4.059496641159058
node_19: feature_name=GO:0005164	feature_id[163].value <= threshold=5.793607711791992
node_20: feature_name=GO:0042130	feature_id[16].value <= threshold=5.073179721832275
node_21: feature_name=GO:0010216	feature_id[282].value <= threshold=4.834584474563599
node_22: feature_name=GO:0009628	feature_id[553].value <= threshold=29.314892768859863
node_23: feature_name=GO:0045628	feature_id[749].value <= threshold=3.1001367568969727
node_24: feature_name=GO:0042288	feature_id[100].value <= threshold=5.174932956695557
node_25: feature_name=GO:0002329	feature_id[406].value <= threshold=2.348930835723877
node_26: feature_name=GO:0051246	feature_id[642].value > threshold=0.6498909294605255
node_194: feature_name=GO:0009164	feature_id[546].value <= threshold=17.11970329284668
node_195: feature_name=GO:0001909	feature_id[386].value <= threshold=7.292566299438477
node_196: feature_name=GO:0003908	feature_id[11].value <= threshold=1.887226164340973
node_197: feature_name=GO:0009164	feature_id[546].value <= threshold=0.26642198860645294
node_198: feature_name=GO:2001242	feature_id[24].value <= threshold=9.207026481628418
node_199: feature_name=GO:0051246	feature_id[642].value > threshold=0.6504445374011993
node_203: feature_name=GO:0032461	feature_id[608].value <= threshold=2.607542634010315
node_204: feature_name=GO:0007584	feature_id[529].value <= threshold=16.099515914916992
node_205: feature_name=hsa05221	feature_id[349].value <= threshold=9.359850406646729
node_206: feature_name=GO:0002636	feature_id[447].value <= threshold=1.615447759628296
node_207: feature_name=GO:0033993	feature_id[421].value > threshold=2.96540611088858e-05
node_217: feature_name=GO:0002331	feature_id[404].value <= threshold=2.1883513927459717
node_218: feature_name=GO:0046498	feature_id[203].value <= threshold=4.085246205329895
node_219: feature_name=GO:0002832	feature_id[491].value <= threshold=3.0228612422943115
node_220: feature_name=GO:1904868	feature_id[306].value <= threshold=3.8674756288528442
node_221: feature_name=GO:0048545	feature_id[360].value <= threshold=27.38136100769043

node_222: feature_name=GO:0071310 feature_id[760].value <= threshold=2.3522024154663086
node_223: feature_name=GO:0001775 feature_id[505].value <= threshold=9.71301555633545
node_224: feature_name=GO:0071310 feature_id[760].value <= threshold=2.346743583679199
node_225: feature_name=GO:0001772 feature_id[91].value > threshold=3.252573609352112
node_301: feature_name=GO:0060249 feature_id[390].value <= threshold=1.9239138662815094
Class: negative genes

Rules_735 passed counts:1
node_0: feature_name=GO:0042113 feature_id[0].value <= threshold=13.408552169799805
node_1: feature_name=GO:0007568 feature_id[534].value <= threshold=5.0313897132873535
node_2: feature_name=GO:0002705 feature_id[541].value <= threshold=3.200145721435547
node_3: feature_name=GO:1901525 feature_id[576].value <= threshold=0.4466460347175598
node_4: feature_name=GO:0048539 feature_id[319].value <= threshold=3.0399646759033203
node_5: feature_name=GO:0001910 feature_id[385].value <= threshold=3.7437864542007446
node_6: feature_name=GO:0043200 feature_id[706].value <= threshold=9.307284355163574
node_7: feature_name=GO:0001773 feature_id[308].value <= threshold=4.016931533813477
node_8: feature_name=GO:0090116 feature_id[97].value <= threshold=7.99645471572876
node_9: feature_name=GO:0019814 feature_id[189].value <= threshold=4.525782108306885
node_10: feature_name=GO:1902583 feature_id[215].value <= threshold=17.60310649871826
node_11: feature_name=GO:0045429 feature_id[737].value <= threshold=1.6133361458778381
node_12: feature_name=GO:0003720 feature_id[228].value <= threshold=5.519326210021973
node_13: feature_name=GO:0046006 feature_id[364].value <= threshold=4.9985432624816895
node_14: feature_name=GO:0070424 feature_id[254].value <= threshold=6.233297109603882
node_15: feature_name=GO:0009892 feature_id[320].value <= threshold=59.99180793762207
node_16: feature_name=GO:0007064 feature_id[527].value <= threshold=11.57377815246582
node_17: feature_name=GO:0005575 feature_id[17].value <= threshold=15.417460918426514
node_18: feature_name=GO:0043368 feature_id[31].value <= threshold=4.059496641159058
node_19: feature_name=GO:0005164 feature_id[163].value <= threshold=5.793607711791992
node_20: feature_name=GO:0042130 feature_id[16].value <= threshold=5.073179721832275
node_21: feature_name=GO:0010216 feature_id[282].value <= threshold=4.834584474563599
node_22: feature_name=GO:0009628 feature_id[553].value <= threshold=29.314892768859863
node_23: feature_name=GO:0045628 feature_id[749].value <= threshold=3.1001367568969727
node_24: feature_name=GO:0042288 feature_id[100].value <= threshold=5.174932956695557
node_25: feature_name=GO:0002329 feature_id[406].value <= threshold=2.348930835723877
node_26: feature_name=GO:0051246 feature_id[642].value > threshold=0.6498909294605255

node_194: feature_name=GO:0009164 feature_id[546].value <= threshold=17.11970329284668
node_195: feature_name=GO:0001909 feature_id[386].value <= threshold=7.292566299438477
node_196: feature_name=GO:0003908 feature_id[11].value <= threshold=1.887226164340973
node_197: feature_name=GO:0009164 feature_id[546].value <= threshold=0.26642198860645294
node_198: feature_name=GO:2001242 feature_id[24].value <= threshold=9.207026481628418
node_199: feature_name=GO:0051246 feature_id[642].value > threshold=0.6504445374011993
node_203: feature_name=GO:0032461 feature_id[608].value <= threshold=2.607542634010315
node_204: feature_name=GO:0007584 feature_id[529].value <= threshold=16.099515914916992
node_205: feature_name=hsa05221 feature_id[349].value <= threshold=9.359850406646729
node_206: feature_name=GO:0002636 feature_id[447].value <= threshold=1.615447759628296
node_207: feature_name=GO:0033993 feature_id[421].value > threshold=2.96540611088858e-05
node_217: feature_name=GO:0002331 feature_id[404].value <= threshold=2.1883513927459717
node_218: feature_name=GO:0046498 feature_id[203].value <= threshold=4.085246205329895
node_219: feature_name=GO:0002832 feature_id[491].value <= threshold=3.0228612422943115
node_220: feature_name=GO:1904868 feature_id[306].value <= threshold=3.8674756288528442
node_221: feature_name=GO:0048545 feature_id[360].value <= threshold=27.38136100769043
node_222: feature_name=GO:0071310 feature_id[760].value <= threshold=2.3522024154663086
node_223: feature_name=GO:0001775 feature_id[505].value <= threshold=9.71301555633545
node_224: feature_name=GO:0071310 feature_id[760].value <= threshold=2.346743583679199
node_225: feature_name=GO:0001772 feature_id[91].value <= threshold=3.252573609352112
node_226: feature_name=GO:0036498 feature_id[359].value > threshold=12.092026710510254
node_298: feature_name=GO:0045471 feature_id[354].value > threshold=0.153983018361032
Class: negative genes

Rules_736 passed counts:1
node_0: feature_name=GO:0042113 feature_id[0].value <= threshold=13.408552169799805
node_1: feature_name=GO:0007568 feature_id[534].value <= threshold=5.0313897132873535
node_2: feature_name=GO:0002705 feature_id[541].value <= threshold=3.200145721435547
node_3: feature_name=GO:1901525 feature_id[576].value <= threshold=0.4466460347175598
node_4: feature_name=GO:0048539 feature_id[319].value <= threshold=3.0399646759033203
node_5: feature_name=GO:0001910 feature_id[385].value <= threshold=3.7437864542007446
node_6: feature_name=GO:0043200 feature_id[706].value <= threshold=9.307284355163574
node_7: feature_name=GO:0001773 feature_id[308].value <= threshold=4.016931533813477
node_8: feature_name=GO:0090116 feature_id[97].value <= threshold=7.99645471572876
node_9: feature_name=GO:0019814 feature_id[189].value <= threshold=4.525782108306885

node_10: feature_name=GO:1902583	feature_id[215].value <= threshold=17.60310649871826
node_11: feature_name=GO:0045429	feature_id[737].value <= threshold=1.6133361458778381
node_12: feature_name=GO:0003720	feature_id[228].value <= threshold=5.519326210021973
node_13: feature_name=GO:0046006	feature_id[364].value <= threshold=4.9985432624816895
node_14: feature_name=GO:0070424	feature_id[254].value <= threshold=6.233297109603882
node_15: feature_name=GO:0009892	feature_id[320].value <= threshold=59.99180793762207
node_16: feature_name=GO:0007064	feature_id[527].value <= threshold=11.57377815246582
node_17: feature_name=GO:0005575	feature_id[17].value <= threshold=15.417460918426514
node_18: feature_name=GO:0043368	feature_id[31].value <= threshold=4.059496641159058
node_19: feature_name=GO:0005164	feature_id[163].value <= threshold=5.793607711791992
node_20: feature_name=GO:0042130	feature_id[16].value <= threshold=5.073179721832275
node_21: feature_name=GO:0010216	feature_id[282].value <= threshold=4.834584474563599
node_22: feature_name=GO:0009628	feature_id[553].value <= threshold=29.314892768859863
node_23: feature_name=GO:0045628	feature_id[749].value <= threshold=3.1001367568969727
node_24: feature_name=GO:0042288	feature_id[100].value <= threshold=5.174932956695557
node_25: feature_name=GO:0002329	feature_id[406].value <= threshold=2.348930835723877
node_26: feature_name=GO:0051246	feature_id[642].value > threshold=0.6498909294605255
node_194: feature_name=GO:0009164	feature_id[546].value <= threshold=17.11970329284668
node_195: feature_name=GO:0001909	feature_id[386].value <= threshold=7.292566299438477
node_196: feature_name=GO:0003908	feature_id[11].value <= threshold=1.887226164340973
node_197: feature_name=GO:0009164	feature_id[546].value <= threshold=0.26642198860645294
node_198: feature_name=GO:2001242	feature_id[24].value <= threshold=9.207026481628418
node_199: feature_name=GO:0051246	feature_id[642].value > threshold=0.6504445374011993
node_203: feature_name=GO:0032461	feature_id[608].value <= threshold=2.607542634010315
node_204: feature_name=GO:0007584	feature_id[529].value <= threshold=16.099515914916992
node_205: feature_name=hsa05221	feature_id[349].value <= threshold=9.359850406646729
node_206: feature_name=GO:0002636	feature_id[447].value <= threshold=1.615447759628296
node_207: feature_name=GO:0033993	feature_id[421].value > threshold=2.96540611088858e-05
node_217: feature_name=GO:0002331	feature_id[404].value <= threshold=2.1883513927459717
node_218: feature_name=GO:0046498	feature_id[203].value <= threshold=4.085246205329895
node_219: feature_name=GO:0002832	feature_id[491].value <= threshold=3.0228612422943115
node_220: feature_name=GO:1904868	feature_id[306].value <= threshold=3.8674756288528442
node_221: feature_name=GO:0048545	feature_id[360].value <= threshold=27.38136100769043
node_222: feature_name=GO:0071310	feature_id[760].value <= threshold=2.3522024154663086
node_223: feature_name=GO:0001775	feature_id[505].value <= threshold=9.71301555633545

node_224: feature_name=GO:0071310 feature_id[760].value <= threshold=2.346743583679199
node_225: feature_name=GO:0001772 feature_id[91].value <= threshold=3.252573609352112
node_226: feature_name=GO:0036498 feature_id[359].value > threshold=12.092026710510254
node_298: feature_name=GO:0045471 feature_id[354].value <= threshold=0.153983018361032
Class: positive genes

Rules_737 passed counts:1
node_0: feature_name=GO:0042113 feature_id[0].value <= threshold=13.408552169799805
node_1: feature_name=GO:0007568 feature_id[534].value <= threshold=5.0313897132873535
node_2: feature_name=GO:0002705 feature_id[541].value <= threshold=3.200145721435547
node_3: feature_name=GO:1901525 feature_id[576].value <= threshold=0.4466460347175598
node_4: feature_name=GO:0048539 feature_id[319].value <= threshold=3.0399646759033203
node_5: feature_name=GO:0001910 feature_id[385].value <= threshold=3.7437864542007446
node_6: feature_name=GO:0043200 feature_id[706].value <= threshold=9.307284355163574
node_7: feature_name=GO:0001773 feature_id[308].value <= threshold=4.016931533813477
node_8: feature_name=GO:0090116 feature_id[97].value <= threshold=7.99645471572876
node_9: feature_name=GO:0019814 feature_id[189].value <= threshold=4.525782108306885
node_10: feature_name=GO:1902583 feature_id[215].value <= threshold=17.60310649871826
node_11: feature_name=GO:0045429 feature_id[737].value <= threshold=1.6133361458778381
node_12: feature_name=GO:0003720 feature_id[228].value <= threshold=5.519326210021973
node_13: feature_name=GO:0046006 feature_id[364].value <= threshold=4.9985432624816895
node_14: feature_name=GO:0070424 feature_id[254].value <= threshold=6.233297109603882
node_15: feature_name=GO:0009892 feature_id[320].value <= threshold=59.99180793762207
node_16: feature_name=GO:0007064 feature_id[527].value <= threshold=11.57377815246582
node_17: feature_name=GO:0005575 feature_id[17].value <= threshold=15.417460918426514
node_18: feature_name=GO:0043368 feature_id[31].value <= threshold=4.059496641159058
node_19: feature_name=GO:0005164 feature_id[163].value <= threshold=5.793607711791992
node_20: feature_name=GO:0042130 feature_id[16].value <= threshold=5.073179721832275
node_21: feature_name=GO:0010216 feature_id[282].value <= threshold=4.834584474563599
node_22: feature_name=GO:0009628 feature_id[553].value <= threshold=29.314892768859863
node_23: feature_name=GO:0045628 feature_id[749].value <= threshold=3.1001367568969727
node_24: feature_name=GO:0042288 feature_id[100].value <= threshold=5.174932956695557
node_25: feature_name=GO:0002329 feature_id[406].value <= threshold=2.348930835723877
node_26: feature_name=GO:0051246 feature_id[642].value > threshold=0.6498909294605255
node_194: feature_name=GO:0009164 feature_id[546].value <= threshold=17.11970329284668

node_195: feature_name=GO:0001909 feature_id[386].value <= threshold=7.292566299438477
node_196: feature_name=GO:0003908 feature_id[11].value <= threshold=1.887226164340973
node_197: feature_name=GO:0009164 feature_id[546].value <= threshold=0.26642198860645294
node_198: feature_name=GO:2001242 feature_id[24].value <= threshold=9.207026481628418
node_199: feature_name=GO:0051246 feature_id[642].value > threshold=0.6504445374011993
node_203: feature_name=GO:0032461 feature_id[608].value <= threshold=2.607542634010315
node_204: feature_name=GO:0007584 feature_id[529].value <= threshold=16.099515914916992
node_205: feature_name=hsa05221 feature_id[349].value <= threshold=9.359850406646729
node_206: feature_name=GO:0002636 feature_id[447].value <= threshold=1.615447759628296
node_207: feature_name=GO:0033993 feature_id[421].value > threshold=2.965406110888858e-05
node_217: feature_name=GO:0002331 feature_id[404].value <= threshold=2.1883513927459717
node_218: feature_name=GO:0046498 feature_id[203].value <= threshold=4.085246205329895
node_219: feature_name=GO:0002832 feature_id[491].value <= threshold=3.0228612422943115
node_220: feature_name=GO:1904868 feature_id[306].value <= threshold=3.8674756288528442
node_221: feature_name=GO:0048545 feature_id[360].value <= threshold=27.38136100769043
node_222: feature_name=GO:0071310 feature_id[760].value <= threshold=2.3522024154663086
node_223: feature_name=GO:0001775 feature_id[505].value <= threshold=9.71301555633545
node_224: feature_name=GO:0071310 feature_id[760].value <= threshold=2.346743583679199
node_225: feature_name=GO:0001772 feature_id[91].value <= threshold=3.252573609352112
node_226: feature_name=GO:0036498 feature_id[359].value <= threshold=12.092026710510254
node_227: feature_name=hsa05340 feature_id[351].value > threshold=0.7677814364433289
node_281: feature_name=GO:0006359 feature_id[87].value <= threshold=0.846381276845932
node_282: feature_name=GO:0002326 feature_id[410].value > threshold=1.929194152355194
Class: positive genes

Rules_738 passed counts:1
node_0: feature_name=GO:0042113 feature_id[0].value <= threshold=13.408552169799805
node_1: feature_name=GO:0007568 feature_id[534].value <= threshold=5.0313897132873535
node_2: feature_name=GO:0002705 feature_id[541].value <= threshold=3.200145721435547
node_3: feature_name=GO:1901525 feature_id[576].value <= threshold=0.4466460347175598
node_4: feature_name=GO:0048539 feature_id[319].value <= threshold=3.0399646759033203
node_5: feature_name=GO:0001910 feature_id[385].value <= threshold=3.7437864542007446
node_6: feature_name=GO:0043200 feature_id[706].value <= threshold=9.307284355163574
node_7: feature_name=GO:0001773 feature_id[308].value <= threshold=4.016931533813477
node_8: feature_name=GO:0090116 feature_id[97].value <= threshold=7.99645471572876

node_9: feature_name=GO:0019814	feature_id[189].value <= threshold=4.525782108306885
node_10: feature_name=GO:1902583	feature_id[215].value <= threshold=17.60310649871826
node_11: feature_name=GO:0045429	feature_id[737].value <= threshold=1.6133361458778381
node_12: feature_name=GO:0003720	feature_id[228].value <= threshold=5.519326210021973
node_13: feature_name=GO:0046006	feature_id[364].value <= threshold=4.9985432624816895
node_14: feature_name=GO:0070424	feature_id[254].value <= threshold=6.233297109603882
node_15: feature_name=GO:0009892	feature_id[320].value <= threshold=59.99180793762207
node_16: feature_name=GO:0007064	feature_id[527].value <= threshold=11.57377815246582
node_17: feature_name=GO:0005575	feature_id[17].value <= threshold=15.417460918426514
node_18: feature_name=GO:0043368	feature_id[31].value <= threshold=4.059496641159058
node_19: feature_name=GO:0005164	feature_id[163].value <= threshold=5.793607711791992
node_20: feature_name=GO:0042130	feature_id[16].value <= threshold=5.073179721832275
node_21: feature_name=GO:0010216	feature_id[282].value <= threshold=4.834584474563599
node_22: feature_name=GO:0009628	feature_id[553].value <= threshold=29.314892768859863
node_23: feature_name=GO:0045628	feature_id[749].value <= threshold=3.1001367568969727
node_24: feature_name=GO:0042288	feature_id[100].value <= threshold=5.174932956695557
node_25: feature_name=GO:0002329	feature_id[406].value <= threshold=2.348930835723877
node_26: feature_name=GO:0051246	feature_id[642].value > threshold=0.6498909294605255
node_194: feature_name=GO:0009164	feature_id[546].value <= threshold=17.11970329284668
node_195: feature_name=GO:0001909	feature_id[386].value <= threshold=7.292566299438477
node_196: feature_name=GO:0003908	feature_id[11].value <= threshold=1.887226164340973
node_197: feature_name=GO:0009164	feature_id[546].value <= threshold=0.26642198860645294
node_198: feature_name=GO:2001242	feature_id[24].value <= threshold=9.207026481628418
node_199: feature_name=GO:0051246	feature_id[642].value > threshold=0.6504445374011993
node_203: feature_name=GO:0032461	feature_id[608].value <= threshold=2.607542634010315
node_204: feature_name=GO:0007584	feature_id[529].value <= threshold=16.099515914916992
node_205: feature_name=hsa05221	feature_id[349].value <= threshold=9.359850406646729
node_206: feature_name=GO:0002636	feature_id[447].value <= threshold=1.615447759628296
node_207: feature_name=GO:0033993	feature_id[421].value > threshold=2.96540611088858e-05
node_217: feature_name=GO:0002331	feature_id[404].value <= threshold=2.1883513927459717
node_218: feature_name=GO:0046498	feature_id[203].value <= threshold=4.085246205329895
node_219: feature_name=GO:0002832	feature_id[491].value <= threshold=3.0228612422943115
node_220: feature_name=GO:1904868	feature_id[306].value <= threshold=3.8674756288528442
node_221: feature_name=GO:0048545	feature_id[360].value <= threshold=27.38136100769043
node_222: feature_name=GO:0071310	feature_id[760].value <= threshold=2.3522024154663086

node_223: feature_name=GO:0001775 feature_id[505].value <= threshold=9.71301555633545
node_224: feature_name=GO:0071310 feature_id[760].value <= threshold=2.346743583679199
node_225: feature_name=GO:0001772 feature_id[91].value <= threshold=3.252573609352112
node_226: feature_name=GO:0036498 feature_id[359].value <= threshold=12.092026710510254
node_227: feature_name=hsa05340 feature_id[351].value > threshold=0.7677814364433289
node_281: feature_name=GO:0006359 feature_id[87].value <= threshold=0.846381276845932
node_282: feature_name=GO:0002326 feature_id[410].value <= threshold=1.929194152355194
node_283: feature_name=GO:0072610 feature_id[262].value > threshold=1.4640317559242249
Class: positive genes

Rules_739 passed counts:1
node_0: feature_name=GO:0042113 feature_id[0].value <= threshold=13.408552169799805
node_1: feature_name=GO:0007568 feature_id[534].value <= threshold=5.0313897132873535
node_2: feature_name=GO:0002705 feature_id[541].value <= threshold=3.200145721435547
node_3: feature_name=GO:1901525 feature_id[576].value <= threshold=0.4466460347175598
node_4: feature_name=GO:0048539 feature_id[319].value <= threshold=3.0399646759033203
node_5: feature_name=GO:0001910 feature_id[385].value <= threshold=3.7437864542007446
node_6: feature_name=GO:0043200 feature_id[706].value <= threshold=9.307284355163574
node_7: feature_name=GO:0001773 feature_id[308].value <= threshold=4.016931533813477
node_8: feature_name=GO:0090116 feature_id[97].value <= threshold=7.99645471572876
node_9: feature_name=GO:0019814 feature_id[189].value <= threshold=4.525782108306885
node_10: feature_name=GO:1902583 feature_id[215].value <= threshold=17.60310649871826
node_11: feature_name=GO:0045429 feature_id[737].value <= threshold=1.6133361458778381
node_12: feature_name=GO:0003720 feature_id[228].value <= threshold=5.519326210021973
node_13: feature_name=GO:0046006 feature_id[364].value <= threshold=4.9985432624816895
node_14: feature_name=GO:0070424 feature_id[254].value <= threshold=6.233297109603882
node_15: feature_name=GO:0009892 feature_id[320].value <= threshold=59.99180793762207
node_16: feature_name=GO:0007064 feature_id[527].value <= threshold=11.57377815246582
node_17: feature_name=GO:0005575 feature_id[17].value <= threshold=15.417460918426514
node_18: feature_name=GO:0043368 feature_id[31].value <= threshold=4.059496641159058
node_19: feature_name=GO:0005164 feature_id[163].value <= threshold=5.793607711791992
node_20: feature_name=GO:0042130 feature_id[16].value <= threshold=5.073179721832275
node_21: feature_name=GO:0010216 feature_id[282].value <= threshold=4.834584474563599
node_22: feature_name=GO:0009628 feature_id[553].value <= threshold=29.314892768859863
node_23: feature_name=GO:0045628 feature_id[749].value <= threshold=3.1001367568969727

node_24: feature_name=GO:0042288	feature_id[100].value <= threshold=5.174932956695557
node_25: feature_name=GO:0002329	feature_id[406].value <= threshold=2.348930835723877
node_26: feature_name=GO:0051246	feature_id[642].value > threshold=0.6498909294605255
node_194: feature_name=GO:0009164	feature_id[546].value <= threshold=17.11970329284668
node_195: feature_name=GO:0001909	feature_id[386].value <= threshold=7.292566299438477
node_196: feature_name=GO:0003908	feature_id[11].value <= threshold=1.887226164340973
node_197: feature_name=GO:0009164	feature_id[546].value <= threshold=0.26642198860645294
node_198: feature_name=GO:2001242	feature_id[24].value <= threshold=9.207026481628418
node_199: feature_name=GO:0051246	feature_id[642].value > threshold=0.6504445374011993
node_203: feature_name=GO:0032461	feature_id[608].value <= threshold=2.607542634010315
node_204: feature_name=GO:0007584	feature_id[529].value <= threshold=16.099515914916992
node_205: feature_name=hsa05221	feature_id[349].value <= threshold=9.359850406646729
node_206: feature_name=GO:0002636	feature_id[447].value <= threshold=1.615447759628296
node_207: feature_name=GO:0033993	feature_id[421].value > threshold=2.96540611088858e-05
node_217: feature_name=GO:0002331	feature_id[404].value <= threshold=2.1883513927459717
node_218: feature_name=GO:0046498	feature_id[203].value <= threshold=4.085246205329895
node_219: feature_name=GO:0002832	feature_id[491].value <= threshold=3.0228612422943115
node_220: feature_name=GO:1904868	feature_id[306].value <= threshold=3.8674756288528442
node_221: feature_name=GO:0048545	feature_id[360].value <= threshold=27.38136100769043
node_222: feature_name=GO:0071310	feature_id[760].value <= threshold=2.3522024154663086
node_223: feature_name=GO:0001775	feature_id[505].value <= threshold=9.71301555633545
node_224: feature_name=GO:0071310	feature_id[760].value <= threshold=2.346743583679199
node_225: feature_name=GO:0001772	feature_id[91].value <= threshold=3.252573609352112
node_226: feature_name=GO:0036498	feature_id[359].value <= threshold=12.092026710510254
node_227: feature_name=hsa05340	feature_id[351].value > threshold=0.7677814364433289
node_281: feature_name=GO:0006359	feature_id[87].value <= threshold=0.846381276845932
node_282: feature_name=GO:0002326	feature_id[410].value <= threshold=1.929194152355194
node_283: feature_name=GO:0072610	feature_id[262].value <= threshold=1.4640317559242249
node_284: feature_name=GO:0005575	feature_id[17].value > threshold=0.0011180754081578925
node_286: feature_name=GO:0070741	feature_id[538].value > threshold=1.5534479022026062
Class: positive genes	

Rules_740	passed counts:1
node_0: feature_name=GO:0042113	feature_id[0].value <= threshold=13.408552169799805
node_1: feature_name=GO:0007568	feature_id[534].value <= threshold=5.0313897132873535

node_2: feature_name=GO:0002705	feature_id[541].value <= threshold=3.200145721435547
node_3: feature_name=GO:1901525	feature_id[576].value <= threshold=0.4466460347175598
node_4: feature_name=GO:0048539	feature_id[319].value <= threshold=3.0399646759033203
node_5: feature_name=GO:0001910	feature_id[385].value <= threshold=3.7437864542007446
node_6: feature_name=GO:0043200	feature_id[706].value <= threshold=9.307284355163574
node_7: feature_name=GO:0001773	feature_id[308].value <= threshold=4.016931533813477
node_8: feature_name=GO:0090116	feature_id[97].value <= threshold=7.99645471572876
node_9: feature_name=GO:0019814	feature_id[189].value <= threshold=4.525782108306885
node_10: feature_name=GO:1902583	feature_id[215].value <= threshold=17.60310649871826
node_11: feature_name=GO:0045429	feature_id[737].value <= threshold=1.6133361458778381
node_12: feature_name=GO:0003720	feature_id[228].value <= threshold=5.519326210021973
node_13: feature_name=GO:0046006	feature_id[364].value <= threshold=4.9985432624816895
node_14: feature_name=GO:0070424	feature_id[254].value <= threshold=6.233297109603882
node_15: feature_name=GO:0009892	feature_id[320].value <= threshold=59.99180793762207
node_16: feature_name=GO:0007064	feature_id[527].value <= threshold=11.57377815246582
node_17: feature_name=GO:0005575	feature_id[17].value <= threshold=15.417460918426514
node_18: feature_name=GO:0043368	feature_id[31].value <= threshold=4.059496641159058
node_19: feature_name=GO:0005164	feature_id[163].value <= threshold=5.793607711791992
node_20: feature_name=GO:0042130	feature_id[16].value <= threshold=5.073179721832275
node_21: feature_name=GO:0010216	feature_id[282].value <= threshold=4.834584474563599
node_22: feature_name=GO:0009628	feature_id[553].value <= threshold=29.314892768859863
node_23: feature_name=GO:0045628	feature_id[749].value <= threshold=3.1001367568969727
node_24: feature_name=GO:0042288	feature_id[100].value <= threshold=5.174932956695557
node_25: feature_name=GO:0002329	feature_id[406].value <= threshold=2.348930835723877
node_26: feature_name=GO:0051246	feature_id[642].value > threshold=0.6498909294605255
node_194: feature_name=GO:0009164	feature_id[546].value <= threshold=17.11970329284668
node_195: feature_name=GO:0001909	feature_id[386].value <= threshold=7.292566299438477
node_196: feature_name=GO:0003908	feature_id[11].value <= threshold=1.887226164340973
node_197: feature_name=GO:0009164	feature_id[546].value <= threshold=0.26642198860645294
node_198: feature_name=GO:2001242	feature_id[24].value <= threshold=9.207026481628418
node_199: feature_name=GO:0051246	feature_id[642].value > threshold=0.6504445374011993
node_203: feature_name=GO:0032461	feature_id[608].value <= threshold=2.607542634010315
node_204: feature_name=GO:0007584	feature_id[529].value <= threshold=16.099515914916992
node_205: feature_name=hsa05221	feature_id[349].value <= threshold=9.359850406646729
node_206: feature_name=GO:0002636	feature_id[447].value <= threshold=1.615447759628296

node_207: feature_name=GO:0033993 feature_id[421].value > threshold=2.96540611088858e-05
node_217: feature_name=GO:0002331 feature_id[404].value <= threshold=2.1883513927459717
node_218: feature_name=GO:0046498 feature_id[203].value <= threshold=4.085246205329895
node_219: feature_name=GO:0002832 feature_id[491].value <= threshold=3.0228612422943115
node_220: feature_name=GO:1904868 feature_id[306].value <= threshold=3.8674756288528442
node_221: feature_name=GO:0048545 feature_id[360].value <= threshold=27.38136100769043
node_222: feature_name=GO:0071310 feature_id[760].value <= threshold=2.3522024154663086
node_223: feature_name=GO:0001775 feature_id[505].value <= threshold=9.71301555633545
node_224: feature_name=GO:0071310 feature_id[760].value <= threshold=2.346743583679199
node_225: feature_name=GO:0001772 feature_id[91].value <= threshold=3.252573609352112
node_226: feature_name=GO:0036498 feature_id[359].value <= threshold=12.092026710510254
node_227: feature_name=hsa05340 feature_id[351].value > threshold=0.7677814364433289
node_281: feature_name=GO:0006359 feature_id[87].value <= threshold=0.846381276845932
node_282: feature_name=GO:0002326 feature_id[410].value <= threshold=1.929194152355194
node_283: feature_name=GO:0072610 feature_id[262].value <= threshold=1.4640317559242249
node_284: feature_name=GO:0005575 feature_id[17].value > threshold=0.0011180754081578925
node_286: feature_name=GO:0070741 feature_id[538].value <= threshold=1.5534479022026062
node_287: feature_name=GO:0048583 feature_id[373].value <= threshold=0.018909001722931862
node_288: feature_name=GO:0032703 feature_id[617].value > threshold=0.034508541226387024
Class: positive genes

Rules_741 passed counts:1
node_0: feature_name=GO:0042113 feature_id[0].value <= threshold=13.408552169799805
node_1: feature_name=GO:0007568 feature_id[534].value <= threshold=5.0313897132873535
node_2: feature_name=GO:0002705 feature_id[541].value <= threshold=3.200145721435547
node_3: feature_name=GO:1901525 feature_id[576].value <= threshold=0.4466460347175598
node_4: feature_name=GO:0048539 feature_id[319].value <= threshold=3.0399646759033203
node_5: feature_name=GO:0001910 feature_id[385].value <= threshold=3.7437864542007446
node_6: feature_name=GO:0043200 feature_id[706].value <= threshold=9.307284355163574
node_7: feature_name=GO:0001773 feature_id[308].value <= threshold=4.016931533813477
node_8: feature_name=GO:0090116 feature_id[97].value <= threshold=7.99645471572876
node_9: feature_name=GO:0019814 feature_id[189].value <= threshold=4.525782108306885
node_10: feature_name=GO:1902583 feature_id[215].value <= threshold=17.60310649871826
node_11: feature_name=GO:0045429 feature_id[737].value <= threshold=1.6133361458778381
node_12: feature_name=GO:0003720 feature_id[228].value <= threshold=5.519326210021973

node_13: feature_name=GO:0046006	feature_id[364].value <= threshold=4.9985432624816895
node_14: feature_name=GO:0070424	feature_id[254].value <= threshold=6.233297109603882
node_15: feature_name=GO:0009892	feature_id[320].value <= threshold=59.99180793762207
node_16: feature_name=GO:0007064	feature_id[527].value <= threshold=11.57377815246582
node_17: feature_name=GO:0005575	feature_id[17].value <= threshold=15.417460918426514
node_18: feature_name=GO:0043368	feature_id[31].value <= threshold=4.059496641159058
node_19: feature_name=GO:0005164	feature_id[163].value <= threshold=5.793607711791992
node_20: feature_name=GO:0042130	feature_id[16].value <= threshold=5.073179721832275
node_21: feature_name=GO:0010216	feature_id[282].value <= threshold=4.834584474563599
node_22: feature_name=GO:0009628	feature_id[553].value <= threshold=29.314892768859863
node_23: feature_name=GO:0045628	feature_id[749].value <= threshold=3.1001367568969727
node_24: feature_name=GO:0042288	feature_id[100].value <= threshold=5.174932956695557
node_25: feature_name=GO:0002329	feature_id[406].value <= threshold=2.348930835723877
node_26: feature_name=GO:0051246	feature_id[642].value > threshold=0.6498909294605255
node_194: feature_name=GO:0009164	feature_id[546].value <= threshold=17.11970329284668
node_195: feature_name=GO:0001909	feature_id[386].value <= threshold=7.292566299438477
node_196: feature_name=GO:0003908	feature_id[11].value <= threshold=1.887226164340973
node_197: feature_name=GO:0009164	feature_id[546].value <= threshold=0.26642198860645294
node_198: feature_name=GO:2001242	feature_id[24].value <= threshold=9.207026481628418
node_199: feature_name=GO:0051246	feature_id[642].value > threshold=0.6504445374011993
node_203: feature_name=GO:0032461	feature_id[608].value <= threshold=2.607542634010315
node_204: feature_name=GO:0007584	feature_id[529].value <= threshold=16.099515914916992
node_205: feature_name=hsa05221	feature_id[349].value <= threshold=9.359850406646729
node_206: feature_name=GO:0002636	feature_id[447].value <= threshold=1.615447759628296
node_207: feature_name=GO:0033993	feature_id[421].value > threshold=2.96540611088858e-05
node_217: feature_name=GO:0002331	feature_id[404].value <= threshold=2.1883513927459717
node_218: feature_name=GO:0046498	feature_id[203].value <= threshold=4.085246205329895
node_219: feature_name=GO:0002832	feature_id[491].value <= threshold=3.0228612422943115
node_220: feature_name=GO:1904868	feature_id[306].value <= threshold=3.8674756288528442
node_221: feature_name=GO:0048545	feature_id[360].value <= threshold=27.38136100769043
node_222: feature_name=GO:0071310	feature_id[760].value <= threshold=2.3522024154663086
node_223: feature_name=GO:0001775	feature_id[505].value <= threshold=9.71301555633545
node_224: feature_name=GO:0071310	feature_id[760].value <= threshold=2.346743583679199
node_225: feature_name=GO:0001772	feature_id[91].value <= threshold=3.252573609352112
node_226: feature_name=GO:0036498	feature_id[359].value <= threshold=12.092026710510254

node_227: feature_name=hsa05340 feature_id[351].value > threshold=0.7677814364433289
node_281: feature_name=GO:0006359 feature_id[87].value <= threshold=0.846381276845932
node_282: feature_name=GO:0002326 feature_id[410].value <= threshold=1.929194152355194
node_283: feature_name=GO:0072610 feature_id[262].value <= threshold=1.4640317559242249
node_284: feature_name=GO:0005575 feature_id[17].value > threshold=0.0011180754081578925
node_286: feature_name=GO:0070741 feature_id[538].value <= threshold=1.5534479022026062
node_287: feature_name=GO:0048583 feature_id[373].value <= threshold=0.018909001722931862
node_288: feature_name=GO:0032703 feature_id[617].value <= threshold=0.034508541226387024
Class: negative genes

Rules_742 passed counts:1
node_0: feature_name=GO:0042113 feature_id[0].value <= threshold=13.408552169799805
node_1: feature_name=GO:0007568 feature_id[534].value <= threshold=5.0313897132873535
node_2: feature_name=GO:0002705 feature_id[541].value <= threshold=3.200145721435547
node_3: feature_name=GO:1901525 feature_id[576].value <= threshold=0.4466460347175598
node_4: feature_name=GO:0048539 feature_id[319].value <= threshold=3.0399646759033203
node_5: feature_name=GO:0001910 feature_id[385].value <= threshold=3.7437864542007446
node_6: feature_name=GO:0043200 feature_id[706].value <= threshold=9.307284355163574
node_7: feature_name=GO:0001773 feature_id[308].value <= threshold=4.016931533813477
node_8: feature_name=GO:0090116 feature_id[97].value <= threshold=7.99645471572876
node_9: feature_name=GO:0019814 feature_id[189].value <= threshold=4.525782108306885
node_10: feature_name=GO:1902583 feature_id[215].value <= threshold=17.60310649871826
node_11: feature_name=GO:0045429 feature_id[737].value <= threshold=1.6133361458778381
node_12: feature_name=GO:0003720 feature_id[228].value <= threshold=5.519326210021973
node_13: feature_name=GO:0046006 feature_id[364].value <= threshold=4.9985432624816895
node_14: feature_name=GO:0070424 feature_id[254].value <= threshold=6.233297109603882
node_15: feature_name=GO:0009892 feature_id[320].value <= threshold=59.99180793762207
node_16: feature_name=GO:0007064 feature_id[527].value <= threshold=11.57377815246582
node_17: feature_name=GO:0005575 feature_id[17].value <= threshold=15.417460918426514
node_18: feature_name=GO:0043368 feature_id[31].value <= threshold=4.059496641159058
node_19: feature_name=GO:0005164 feature_id[163].value <= threshold=5.793607711791992
node_20: feature_name=GO:0042130 feature_id[16].value <= threshold=5.073179721832275
node_21: feature_name=GO:0010216 feature_id[282].value <= threshold=4.834584474563599
node_22: feature_name=GO:0009628 feature_id[553].value <= threshold=29.314892768859863
node_23: feature_name=GO:0045628 feature_id[749].value <= threshold=3.1001367568969727

node_24: feature_name=GO:0042288 feature_id[100].value <= threshold=5.174932956695557
node_25: feature_name=GO:0002329 feature_id[406].value <= threshold=2.348930835723877
node_26: feature_name=GO:0051246 feature_id[642].value > threshold=0.6498909294605255
node_194: feature_name=GO:0009164 feature_id[546].value <= threshold=17.11970329284668
node_195: feature_name=GO:0001909 feature_id[386].value <= threshold=7.292566299438477
node_196: feature_name=GO:0003908 feature_id[11].value <= threshold=1.887226164340973
node_197: feature_name=GO:0009164 feature_id[546].value <= threshold=0.26642198860645294
node_198: feature_name=GO:2001242 feature_id[24].value <= threshold=9.207026481628418
node_199: feature_name=GO:0051246 feature_id[642].value > threshold=0.6504445374011993
node_203: feature_name=GO:0032461 feature_id[608].value <= threshold=2.607542634010315
node_204: feature_name=GO:0007584 feature_id[529].value <= threshold=16.099515914916992
node_205: feature_name=hsa05221 feature_id[349].value <= threshold=9.359850406646729
node_206: feature_name=GO:0002636 feature_id[447].value <= threshold=1.615447759628296
node_207: feature_name=GO:0033993 feature_id[421].value > threshold=2.96540611088858e-05
node_217: feature_name=GO:0002331 feature_id[404].value <= threshold=2.1883513927459717
node_218: feature_name=GO:0046498 feature_id[203].value <= threshold=4.085246205329895
node_219: feature_name=GO:0002832 feature_id[491].value <= threshold=3.0228612422943115
node_220: feature_name=GO:1904868 feature_id[306].value <= threshold=3.8674756288528442
node_221: feature_name=GO:0048545 feature_id[360].value <= threshold=27.38136100769043
node_222: feature_name=GO:0071310 feature_id[760].value <= threshold=2.3522024154663086
node_223: feature_name=GO:0001775 feature_id[505].value <= threshold=9.71301555633545
node_224: feature_name=GO:0071310 feature_id[760].value <= threshold=2.346743583679199
node_225: feature_name=GO:0001772 feature_id[91].value <= threshold=3.252573609352112
node_226: feature_name=GO:0036498 feature_id[359].value <= threshold=12.092026710510254
node_227: feature_name=hsa05340 feature_id[351].value > threshold=0.7677814364433289
node_281: feature_name=GO:0006359 feature_id[87].value <= threshold=0.846381276845932
node_282: feature_name=GO:0002326 feature_id[410].value <= threshold=1.929194152355194
node_283: feature_name=GO:0072610 feature_id[262].value <= threshold=1.4640317559242249
node_284: feature_name=GO:0005575 feature_id[17].value <= threshold=0.0011180754081578925
Class: positive genes

Rules_743 passed counts:1
node_0: feature_name=GO:0042113 feature_id[0].value <= threshold=13.408552169799805
node_1: feature_name=GO:0007568 feature_id[534].value <= threshold=5.0313897132873535
node_2: feature_name=GO:0002705 feature_id[541].value <= threshold=3.200145721435547

node_3: feature_name=GO:1901525	feature_id[576].value <= threshold=0.4466460347175598
node_4: feature_name=GO:0048539	feature_id[319].value <= threshold=3.0399646759033203
node_5: feature_name=GO:0001910	feature_id[385].value <= threshold=3.7437864542007446
node_6: feature_name=GO:0043200	feature_id[706].value <= threshold=9.307284355163574
node_7: feature_name=GO:0001773	feature_id[308].value <= threshold=4.016931533813477
node_8: feature_name=GO:0090116	feature_id[97].value <= threshold=7.99645471572876
node_9: feature_name=GO:0019814	feature_id[189].value <= threshold=4.525782108306885
node_10: feature_name=GO:1902583	feature_id[215].value <= threshold=17.60310649871826
node_11: feature_name=GO:0045429	feature_id[737].value <= threshold=1.6133361458778381
node_12: feature_name=GO:0003720	feature_id[228].value <= threshold=5.519326210021973
node_13: feature_name=GO:0046006	feature_id[364].value <= threshold=4.9985432624816895
node_14: feature_name=GO:0070424	feature_id[254].value <= threshold=6.233297109603882
node_15: feature_name=GO:0009892	feature_id[320].value <= threshold=59.99180793762207
node_16: feature_name=GO:0007064	feature_id[527].value <= threshold=11.57377815246582
node_17: feature_name=GO:0005575	feature_id[17].value <= threshold=15.417460918426514
node_18: feature_name=GO:0043368	feature_id[31].value <= threshold=4.059496641159058
node_19: feature_name=GO:0005164	feature_id[163].value <= threshold=5.793607711791992
node_20: feature_name=GO:0042130	feature_id[16].value <= threshold=5.073179721832275
node_21: feature_name=GO:0010216	feature_id[282].value <= threshold=4.834584474563599
node_22: feature_name=GO:0009628	feature_id[553].value <= threshold=29.314892768859863
node_23: feature_name=GO:0045628	feature_id[749].value <= threshold=3.1001367568969727
node_24: feature_name=GO:0042288	feature_id[100].value <= threshold=5.174932956695557
node_25: feature_name=GO:0002329	feature_id[406].value <= threshold=2.348930835723877
node_26: feature_name=GO:0051246	feature_id[642].value > threshold=0.6498909294605255
node_194: feature_name=GO:0009164	feature_id[546].value <= threshold=17.11970329284668
node_195: feature_name=GO:0001909	feature_id[386].value <= threshold=7.292566299438477
node_196: feature_name=GO:0003908	feature_id[11].value <= threshold=1.887226164340973
node_197: feature_name=GO:0009164	feature_id[546].value <= threshold=0.26642198860645294
node_198: feature_name=GO:2001242	feature_id[24].value <= threshold=9.207026481628418
node_199: feature_name=GO:0051246	feature_id[642].value > threshold=0.6504445374011993
node_203: feature_name=GO:0032461	feature_id[608].value <= threshold=2.607542634010315
node_204: feature_name=GO:0007584	feature_id[529].value <= threshold=16.099515914916992
node_205: feature_name=hsa05221	feature_id[349].value <= threshold=9.359850406646729
node_206: feature_name=GO:0002636	feature_id[447].value <= threshold=1.615447759628296
node_207: feature_name=GO:0033993	feature_id[421].value > threshold=2.96540611088858e-05

node_217: feature_name=GO:0002331 feature_id[404].value <= threshold=2.1883513927459717
node_218: feature_name=GO:0046498 feature_id[203].value <= threshold=4.085246205329895
node_219: feature_name=GO:0002832 feature_id[491].value <= threshold=3.0228612422943115
node_220: feature_name=GO:1904868 feature_id[306].value <= threshold=3.8674756288528442
node_221: feature_name=GO:0048545 feature_id[360].value <= threshold=27.38136100769043
node_222: feature_name=GO:0071310 feature_id[760].value <= threshold=2.3522024154663086
node_223: feature_name=GO:0001775 feature_id[505].value <= threshold=9.71301555633545
node_224: feature_name=GO:0071310 feature_id[760].value <= threshold=2.346743583679199
node_225: feature_name=GO:0001772 feature_id[91].value <= threshold=3.252573609352112
node_226: feature_name=GO:0036498 feature_id[359].value <= threshold=12.092026710510254
node_227: feature_name=hsa05340 feature_id[351].value <= threshold=0.7677814364433289
node_228: feature_name=GO:0032703 feature_id[617].value <= threshold=1.7450646758079529
node_229: feature_name=hsa04640 feature_id[79].value <= threshold=2.118402123451233
node_230: feature_name=GO:0042097 feature_id[675].value > threshold=1.8110727667808533
node_272: feature_name=GO:0031667 feature_id[648].value > threshold=0.26628243178129196
Class: positive genes

Rules_744 passed counts:1
node_0: feature_name=GO:0042113 feature_id[0].value <= threshold=13.408552169799805
node_1: feature_name=GO:0007568 feature_id[534].value <= threshold=5.0313897132873535
node_2: feature_name=GO:0002705 feature_id[541].value <= threshold=3.200145721435547
node_3: feature_name=GO:1901525 feature_id[576].value <= threshold=0.4466460347175598
node_4: feature_name=GO:0048539 feature_id[319].value <= threshold=3.0399646759033203
node_5: feature_name=GO:0001910 feature_id[385].value <= threshold=3.7437864542007446
node_6: feature_name=GO:0043200 feature_id[706].value <= threshold=9.307284355163574
node_7: feature_name=GO:0001773 feature_id[308].value <= threshold=4.016931533813477
node_8: feature_name=GO:0090116 feature_id[97].value <= threshold=7.99645471572876
node_9: feature_name=GO:0019814 feature_id[189].value <= threshold=4.525782108306885
node_10: feature_name=GO:1902583 feature_id[215].value <= threshold=17.60310649871826
node_11: feature_name=GO:0045429 feature_id[737].value <= threshold=1.6133361458778381
node_12: feature_name=GO:0003720 feature_id[228].value <= threshold=5.519326210021973
node_13: feature_name=GO:0046006 feature_id[364].value <= threshold=4.9985432624816895
node_14: feature_name=GO:0070424 feature_id[254].value <= threshold=6.233297109603882
node_15: feature_name=GO:0009892 feature_id[320].value <= threshold=59.99180793762207
node_16: feature_name=GO:0007064 feature_id[527].value <= threshold=11.57377815246582

node_17: feature_name=GO:0005575	feature_id[17].value <= threshold=15.417460918426514
node_18: feature_name=GO:0043368	feature_id[31].value <= threshold=4.059496641159058
node_19: feature_name=GO:0005164	feature_id[163].value <= threshold=5.793607711791992
node_20: feature_name=GO:0042130	feature_id[16].value <= threshold=5.073179721832275
node_21: feature_name=GO:0010216	feature_id[282].value <= threshold=4.834584474563599
node_22: feature_name=GO:0009628	feature_id[553].value <= threshold=29.314892768859863
node_23: feature_name=GO:0045628	feature_id[749].value <= threshold=3.1001367568969727
node_24: feature_name=GO:0042288	feature_id[100].value <= threshold=5.174932956695557
node_25: feature_name=GO:0002329	feature_id[406].value <= threshold=2.348930835723877
node_26: feature_name=GO:0051246	feature_id[642].value > threshold=0.6498909294605255
node_194: feature_name=GO:0009164	feature_id[546].value <= threshold=17.11970329284668
node_195: feature_name=GO:0001909	feature_id[386].value <= threshold=7.292566299438477
node_196: feature_name=GO:0003908	feature_id[11].value <= threshold=1.887226164340973
node_197: feature_name=GO:0009164	feature_id[546].value <= threshold=0.26642198860645294
node_198: feature_name=GO:2001242	feature_id[24].value <= threshold=9.207026481628418
node_199: feature_name=GO:0051246	feature_id[642].value > threshold=0.6504445374011993
node_203: feature_name=GO:0032461	feature_id[608].value <= threshold=2.607542634010315
node_204: feature_name=GO:0007584	feature_id[529].value <= threshold=16.099515914916992
node_205: feature_name=hsa05221	feature_id[349].value <= threshold=9.359850406646729
node_206: feature_name=GO:0002636	feature_id[447].value <= threshold=1.615447759628296
node_207: feature_name=GO:0033993	feature_id[421].value > threshold=2.96540611088858e-05
node_217: feature_name=GO:0002331	feature_id[404].value <= threshold=2.1883513927459717
node_218: feature_name=GO:0046498	feature_id[203].value <= threshold=4.085246205329895
node_219: feature_name=GO:0002832	feature_id[491].value <= threshold=3.0228612422943115
node_220: feature_name=GO:1904868	feature_id[306].value <= threshold=3.8674756288528442
node_221: feature_name=GO:0048545	feature_id[360].value <= threshold=27.38136100769043
node_222: feature_name=GO:0071310	feature_id[760].value <= threshold=2.3522024154663086
node_223: feature_name=GO:0001775	feature_id[505].value <= threshold=9.71301555633545
node_224: feature_name=GO:0071310	feature_id[760].value <= threshold=2.346743583679199
node_225: feature_name=GO:0001772	feature_id[91].value <= threshold=3.252573609352112
node_226: feature_name=GO:0036498	feature_id[359].value <= threshold=12.092026710510254
node_227: feature_name=hsa05340	feature_id[351].value <= threshold=0.7677814364433289
node_228: feature_name=GO:0032703	feature_id[617].value <= threshold=1.7450646758079529
node_229: feature_name=hsa04640	feature_id[79].value <= threshold=2.118402123451233
node_230: feature_name=GO:0042097	feature_id[675].value <= threshold=1.8110727667808533

node_231: feature_name=GO:0071391	feature_id[518].value > threshold=2.691588521003723
node_269: feature_name=GO:0010952	feature_id[483].value > threshold=1.3165376782417297
Class: positive genes	
Rules_745	passed counts:1
node_0: feature_name=GO:0042113	feature_id[0].value <= threshold=13.408552169799805
node_1: feature_name=GO:0007568	feature_id[534].value <= threshold=5.0313897132873535
node_2: feature_name=GO:0002705	feature_id[541].value <= threshold=3.200145721435547
node_3: feature_name=GO:1901525	feature_id[576].value <= threshold=0.4466460347175598
node_4: feature_name=GO:0048539	feature_id[319].value <= threshold=3.0399646759033203
node_5: feature_name=GO:0001910	feature_id[385].value <= threshold=3.7437864542007446
node_6: feature_name=GO:0043200	feature_id[706].value <= threshold=9.307284355163574
node_7: feature_name=GO:0001773	feature_id[308].value <= threshold=4.016931533813477
node_8: feature_name=GO:0090116	feature_id[97].value <= threshold=7.99645471572876
node_9: feature_name=GO:0019814	feature_id[189].value <= threshold=4.525782108306885
node_10: feature_name=GO:1902583	feature_id[215].value <= threshold=17.60310649871826
node_11: feature_name=GO:0045429	feature_id[737].value <= threshold=1.6133361458778381
node_12: feature_name=GO:0003720	feature_id[228].value <= threshold=5.519326210021973
node_13: feature_name=GO:0046006	feature_id[364].value <= threshold=4.9985432624816895
node_14: feature_name=GO:0070424	feature_id[254].value <= threshold=6.233297109603882
node_15: feature_name=GO:0009892	feature_id[320].value <= threshold=59.99180793762207
node_16: feature_name=GO:0007064	feature_id[527].value <= threshold=11.57377815246582
node_17: feature_name=GO:0005575	feature_id[17].value <= threshold=15.417460918426514
node_18: feature_name=GO:0043368	feature_id[31].value <= threshold=4.059496641159058
node_19: feature_name=GO:0005164	feature_id[163].value <= threshold=5.793607711791992
node_20: feature_name=GO:0042130	feature_id[16].value <= threshold=5.073179721832275
node_21: feature_name=GO:0010216	feature_id[282].value <= threshold=4.834584474563599
node_22: feature_name=GO:0009628	feature_id[553].value <= threshold=29.314892768859863
node_23: feature_name=GO:0045628	feature_id[749].value <= threshold=3.1001367568969727
node_24: feature_name=GO:0042288	feature_id[100].value <= threshold=5.174932956695557
node_25: feature_name=GO:0002329	feature_id[406].value <= threshold=2.348930835723877
node_26: feature_name=GO:0051246	feature_id[642].value > threshold=0.6498909294605255
node_194: feature_name=GO:0009164	feature_id[546].value <= threshold=17.11970329284668
node_195: feature_name=GO:0001909	feature_id[386].value <= threshold=7.292566299438477
node_196: feature_name=GO:0003908	feature_id[11].value <= threshold=1.887226164340973

node_197: feature_name=GO:0009164	feature_id[546].value <= threshold=0.26642198860645294
node_198: feature_name=GO:2001242	feature_id[24].value <= threshold=9.207026481628418
node_199: feature_name=GO:0051246	feature_id[642].value > threshold=0.6504445374011993
node_203: feature_name=GO:0032461	feature_id[608].value <= threshold=2.607542634010315
node_204: feature_name=GO:0007584	feature_id[529].value <= threshold=16.099515914916992
node_205: feature_name=hsa05221	feature_id[349].value <= threshold=9.359850406646729
node_206: feature_name=GO:0002636	feature_id[447].value <= threshold=1.615447759628296
node_207: feature_name=GO:0033993	feature_id[421].value > threshold=2.96540611088858e-05
node_217: feature_name=GO:0002331	feature_id[404].value <= threshold=2.1883513927459717
node_218: feature_name=GO:0046498	feature_id[203].value <= threshold=4.085246205329895
node_219: feature_name=GO:0002832	feature_id[491].value <= threshold=3.0228612422943115
node_220: feature_name=GO:1904868	feature_id[306].value <= threshold=3.8674756288528442
node_221: feature_name=GO:0048545	feature_id[360].value <= threshold=27.38136100769043
node_222: feature_name=GO:0071310	feature_id[760].value <= threshold=2.3522024154663086
node_223: feature_name=GO:0001775	feature_id[505].value <= threshold=9.71301555633545
node_224: feature_name=GO:0071310	feature_id[760].value <= threshold=2.346743583679199
node_225: feature_name=GO:0001772	feature_id[91].value <= threshold=3.252573609352112
node_226: feature_name=GO:0036498	feature_id[359].value <= threshold=12.092026710510254
node_227: feature_name=hsa05340	feature_id[351].value <= threshold=0.7677814364433289
node_228: feature_name=GO:0032703	feature_id[617].value <= threshold=1.7450646758079529
node_229: feature_name=hsa04640	feature_id[79].value <= threshold=2.118402123451233
node_230: feature_name=GO:0042097	feature_id[675].value <= threshold=1.8110727667808533
node_231: feature_name=GO:0071391	feature_id[518].value <= threshold=2.691588521003723
node_232: feature_name=GO:0001666	feature_id[682].value > threshold=5.225740432739258
node_266: feature_name=GO:0036294	feature_id[612].value <= threshold=3.7958332896232605
Class: positive genes	

Rules_746	passed counts:1
node_0: feature_name=GO:0042113	feature_id[0].value <= threshold=13.408552169799805
node_1: feature_name=GO:0007568	feature_id[534].value <= threshold=5.0313897132873535
node_2: feature_name=GO:0002705	feature_id[541].value <= threshold=3.200145721435547
node_3: feature_name=GO:1901525	feature_id[576].value <= threshold=0.4466460347175598
node_4: feature_name=GO:0048539	feature_id[319].value <= threshold=3.0399646759033203
node_5: feature_name=GO:0001910	feature_id[385].value <= threshold=3.7437864542007446
node_6: feature_name=GO:0043200	feature_id[706].value <= threshold=9.307284355163574

node_7: feature_name=GO:0001773	feature_id[308].value <= threshold=4.016931533813477
node_8: feature_name=GO:0090116	feature_id[97].value <= threshold=7.99645471572876
node_9: feature_name=GO:0019814	feature_id[189].value <= threshold=4.525782108306885
node_10: feature_name=GO:1902583	feature_id[215].value <= threshold=17.60310649871826
node_11: feature_name=GO:0045429	feature_id[737].value <= threshold=1.6133361458778381
node_12: feature_name=GO:0003720	feature_id[228].value <= threshold=5.519326210021973
node_13: feature_name=GO:0046006	feature_id[364].value <= threshold=4.9985432624816895
node_14: feature_name=GO:0070424	feature_id[254].value <= threshold=6.233297109603882
node_15: feature_name=GO:0009892	feature_id[320].value <= threshold=59.99180793762207
node_16: feature_name=GO:0007064	feature_id[527].value <= threshold=11.57377815246582
node_17: feature_name=GO:0005575	feature_id[17].value <= threshold=15.417460918426514
node_18: feature_name=GO:0043368	feature_id[31].value <= threshold=4.059496641159058
node_19: feature_name=GO:0005164	feature_id[163].value <= threshold=5.793607711791992
node_20: feature_name=GO:0042130	feature_id[16].value <= threshold=5.073179721832275
node_21: feature_name=GO:0010216	feature_id[282].value <= threshold=4.834584474563599
node_22: feature_name=GO:0009628	feature_id[553].value <= threshold=29.314892768859863
node_23: feature_name=GO:0045628	feature_id[749].value <= threshold=3.1001367568969727
node_24: feature_name=GO:0042288	feature_id[100].value <= threshold=5.174932956695557
node_25: feature_name=GO:0002329	feature_id[406].value <= threshold=2.348930835723877
node_26: feature_name=GO:0051246	feature_id[642].value > threshold=0.6498909294605255
node_194: feature_name=GO:0009164	feature_id[546].value <= threshold=17.11970329284668
node_195: feature_name=GO:0001909	feature_id[386].value <= threshold=7.292566299438477
node_196: feature_name=GO:0003908	feature_id[11].value <= threshold=1.887226164340973
node_197: feature_name=GO:0009164	feature_id[546].value <= threshold=0.26642198860645294
node_198: feature_name=GO:2001242	feature_id[24].value <= threshold=9.207026481628418
node_199: feature_name=GO:0051246	feature_id[642].value > threshold=0.6504445374011993
node_203: feature_name=GO:0032461	feature_id[608].value <= threshold=2.607542634010315
node_204: feature_name=GO:0007584	feature_id[529].value <= threshold=16.099515914916992
node_205: feature_name=hsa05221	feature_id[349].value <= threshold=9.359850406646729
node_206: feature_name=GO:0002636	feature_id[447].value <= threshold=1.615447759628296
node_207: feature_name=GO:0033993	feature_id[421].value > threshold=2.96540611088858e-05
node_217: feature_name=GO:0002331	feature_id[404].value <= threshold=2.1883513927459717
node_218: feature_name=GO:0046498	feature_id[203].value <= threshold=4.085246205329895
node_219: feature_name=GO:0002832	feature_id[491].value <= threshold=3.0228612422943115
node_220: feature_name=GO:1904868	feature_id[306].value <= threshold=3.8674756288528442

node_221: feature_name=GO:0048545 feature_id[360].value <= threshold=27.38136100769043
node_222: feature_name=GO:0071310 feature_id[760].value <= threshold=2.3522024154663086
node_223: feature_name=GO:0001775 feature_id[505].value <= threshold=9.71301555633545
node_224: feature_name=GO:0071310 feature_id[760].value <= threshold=2.346743583679199
node_225: feature_name=GO:0001772 feature_id[91].value <= threshold=3.252573609352112
node_226: feature_name=GO:0036498 feature_id[359].value <= threshold=12.092026710510254
node_227: feature_name=hsa05340 feature_id[351].value <= threshold=0.7677814364433289
node_228: feature_name=GO:0032703 feature_id[617].value <= threshold=1.7450646758079529
node_229: feature_name=hsa04640 feature_id[79].value <= threshold=2.118402123451233
node_230: feature_name=GO:0042097 feature_id[675].value <= threshold=1.8110727667808533
node_231: feature_name=GO:0071391 feature_id[518].value <= threshold=2.691588521003723
node_232: feature_name=GO:0001666 feature_id[682].value <= threshold=5.225740432739258
node_233: feature_name=GO:0048539 feature_id[319].value > threshold=1.8523842096328735
node_263: feature_name=GO:0002507 feature_id[34].value > threshold=0.6999551057815552
Class: positive genes

Rules_747 passed counts:1
node_0: feature_name=GO:0042113 feature_id[0].value <= threshold=13.408552169799805
node_1: feature_name=GO:0007568 feature_id[534].value <= threshold=5.0313897132873535
node_2: feature_name=GO:0002705 feature_id[541].value <= threshold=3.200145721435547
node_3: feature_name=GO:1901525 feature_id[576].value <= threshold=0.4466460347175598
node_4: feature_name=GO:0048539 feature_id[319].value <= threshold=3.0399646759033203
node_5: feature_name=GO:0001910 feature_id[385].value <= threshold=3.7437864542007446
node_6: feature_name=GO:0043200 feature_id[706].value <= threshold=9.307284355163574
node_7: feature_name=GO:0001773 feature_id[308].value <= threshold=4.016931533813477
node_8: feature_name=GO:0090116 feature_id[97].value <= threshold=7.99645471572876
node_9: feature_name=GO:0019814 feature_id[189].value <= threshold=4.525782108306885
node_10: feature_name=GO:1902583 feature_id[215].value <= threshold=17.60310649871826
node_11: feature_name=GO:0045429 feature_id[737].value <= threshold=1.6133361458778381
node_12: feature_name=GO:0003720 feature_id[228].value <= threshold=5.519326210021973
node_13: feature_name=GO:0046006 feature_id[364].value <= threshold=4.9985432624816895
node_14: feature_name=GO:0070424 feature_id[254].value <= threshold=6.233297109603882
node_15: feature_name=GO:0009892 feature_id[320].value <= threshold=59.99180793762207
node_16: feature_name=GO:0007064 feature_id[527].value <= threshold=11.57377815246582
node_17: feature_name=GO:0005575 feature_id[17].value <= threshold=15.417460918426514

node_18: feature_name=GO:0043368	feature_id[31].value <= threshold=4.059496641159058
node_19: feature_name=GO:0005164	feature_id[163].value <= threshold=5.793607711791992
node_20: feature_name=GO:0042130	feature_id[16].value <= threshold=5.073179721832275
node_21: feature_name=GO:0010216	feature_id[282].value <= threshold=4.834584474563599
node_22: feature_name=GO:0009628	feature_id[553].value <= threshold=29.314892768859863
node_23: feature_name=GO:0045628	feature_id[749].value <= threshold=3.1001367568969727
node_24: feature_name=GO:0042288	feature_id[100].value <= threshold=5.174932956695557
node_25: feature_name=GO:0002329	feature_id[406].value <= threshold=2.348930835723877
node_26: feature_name=GO:0051246	feature_id[642].value > threshold=0.6498909294605255
node_194: feature_name=GO:0009164	feature_id[546].value <= threshold=17.11970329284668
node_195: feature_name=GO:0001909	feature_id[386].value <= threshold=7.292566299438477
node_196: feature_name=GO:0003908	feature_id[11].value <= threshold=1.887226164340973
node_197: feature_name=GO:0009164	feature_id[546].value <= threshold=0.26642198860645294
node_198: feature_name=GO:2001242	feature_id[24].value <= threshold=9.207026481628418
node_199: feature_name=GO:0051246	feature_id[642].value > threshold=0.6504445374011993
node_203: feature_name=GO:0032461	feature_id[608].value <= threshold=2.607542634010315
node_204: feature_name=GO:0007584	feature_id[529].value <= threshold=16.099515914916992
node_205: feature_name=hsa05221	feature_id[349].value <= threshold=9.359850406646729
node_206: feature_name=GO:0002636	feature_id[447].value <= threshold=1.615447759628296
node_207: feature_name=GO:0033993	feature_id[421].value > threshold=2.96540611088858e-05
node_217: feature_name=GO:0002331	feature_id[404].value <= threshold=2.1883513927459717
node_218: feature_name=GO:0046498	feature_id[203].value <= threshold=4.085246205329895
node_219: feature_name=GO:0002832	feature_id[491].value <= threshold=3.0228612422943115
node_220: feature_name=GO:1904868	feature_id[306].value <= threshold=3.8674756288528442
node_221: feature_name=GO:0048545	feature_id[360].value <= threshold=27.38136100769043
node_222: feature_name=GO:0071310	feature_id[760].value <= threshold=2.3522024154663086
node_223: feature_name=GO:0001775	feature_id[505].value <= threshold=9.71301555633545
node_224: feature_name=GO:0071310	feature_id[760].value <= threshold=2.346743583679199
node_225: feature_name=GO:0001772	feature_id[91].value <= threshold=3.252573609352112
node_226: feature_name=GO:0036498	feature_id[359].value <= threshold=12.092026710510254
node_227: feature_name=hsa05340	feature_id[351].value <= threshold=0.7677814364433289
node_228: feature_name=GO:0032703	feature_id[617].value <= threshold=1.7450646758079529
node_229: feature_name=hsa04640	feature_id[79].value <= threshold=2.118402123451233
node_230: feature_name=GO:0042097	feature_id[675].value <= threshold=1.8110727667808533
node_231: feature_name=GO:0071391	feature_id[518].value <= threshold=2.691588521003723

node_232: feature_name=GO:0001666 feature_id[682].value <= threshold=5.225740432739258
node_233: feature_name=GO:0048539 feature_id[319].value <= threshold=1.8523842096328735
node_234: feature_name=GO:0038061 feature_id[671].value > threshold=8.564527988433838
node_260: feature_name=hsa04115 feature_id[324].value > threshold=0.9803315252065659
Class: positive genes

Rules_748 passed counts:1
node_0: feature_name=GO:0042113 feature_id[0].value <= threshold=13.408552169799805
node_1: feature_name=GO:0007568 feature_id[534].value <= threshold=5.0313897132873535
node_2: feature_name=GO:0002705 feature_id[541].value <= threshold=3.200145721435547
node_3: feature_name=GO:1901525 feature_id[576].value <= threshold=0.4466460347175598
node_4: feature_name=GO:0048539 feature_id[319].value <= threshold=3.0399646759033203
node_5: feature_name=GO:0001910 feature_id[385].value <= threshold=3.7437864542007446
node_6: feature_name=GO:0043200 feature_id[706].value <= threshold=9.307284355163574
node_7: feature_name=GO:0001773 feature_id[308].value <= threshold=4.016931533813477
node_8: feature_name=GO:0090116 feature_id[97].value <= threshold=7.99645471572876
node_9: feature_name=GO:0019814 feature_id[189].value <= threshold=4.525782108306885
node_10: feature_name=GO:1902583 feature_id[215].value <= threshold=17.60310649871826
node_11: feature_name=GO:0045429 feature_id[737].value <= threshold=1.6133361458778381
node_12: feature_name=GO:0003720 feature_id[228].value <= threshold=5.519326210021973
node_13: feature_name=GO:0046006 feature_id[364].value <= threshold=4.9985432624816895
node_14: feature_name=GO:0070424 feature_id[254].value <= threshold=6.233297109603882
node_15: feature_name=GO:0009892 feature_id[320].value <= threshold=59.99180793762207
node_16: feature_name=GO:0007064 feature_id[527].value <= threshold=11.57377815246582
node_17: feature_name=GO:0005575 feature_id[17].value <= threshold=15.417460918426514
node_18: feature_name=GO:0043368 feature_id[31].value <= threshold=4.059496641159058
node_19: feature_name=GO:0005164 feature_id[163].value <= threshold=5.793607711791992
node_20: feature_name=GO:0042130 feature_id[16].value <= threshold=5.073179721832275
node_21: feature_name=GO:0010216 feature_id[282].value <= threshold=4.834584474563599
node_22: feature_name=GO:0009628 feature_id[553].value <= threshold=29.314892768859863
node_23: feature_name=GO:0045628 feature_id[749].value <= threshold=3.1001367568969727
node_24: feature_name=GO:0042288 feature_id[100].value <= threshold=5.174932956695557
node_25: feature_name=GO:0002329 feature_id[406].value <= threshold=2.348930835723877
node_26: feature_name=GO:0051246 feature_id[642].value > threshold=0.6498909294605255
node_194: feature_name=GO:0009164 feature_id[546].value <= threshold=17.11970329284668

node_195: feature_name=GO:0001909 feature_id[386].value <= threshold=7.292566299438477
node_196: feature_name=GO:0003908 feature_id[11].value <= threshold=1.887226164340973
node_197: feature_name=GO:0009164 feature_id[546].value <= threshold=0.26642198860645294
node_198: feature_name=GO:2001242 feature_id[24].value <= threshold=9.207026481628418
node_199: feature_name=GO:0051246 feature_id[642].value > threshold=0.6504445374011993
node_203: feature_name=GO:0032461 feature_id[608].value <= threshold=2.607542634010315
node_204: feature_name=GO:0007584 feature_id[529].value <= threshold=16.099515914916992
node_205: feature_name=hsa05221 feature_id[349].value <= threshold=9.359850406646729
node_206: feature_name=GO:0002636 feature_id[447].value <= threshold=1.615447759628296
node_207: feature_name=GO:0033993 feature_id[421].value > threshold=2.96540611088858e-05
node_217: feature_name=GO:0002331 feature_id[404].value <= threshold=2.1883513927459717
node_218: feature_name=GO:0046498 feature_id[203].value <= threshold=4.085246205329895
node_219: feature_name=GO:0002832 feature_id[491].value <= threshold=3.0228612422943115
node_220: feature_name=GO:1904868 feature_id[306].value <= threshold=3.8674756288528442
node_221: feature_name=GO:0048545 feature_id[360].value <= threshold=27.38136100769043
node_222: feature_name=GO:0071310 feature_id[760].value <= threshold=2.3522024154663086
node_223: feature_name=GO:0001775 feature_id[505].value <= threshold=9.71301555633545
node_224: feature_name=GO:0071310 feature_id[760].value <= threshold=2.346743583679199
node_225: feature_name=GO:0001772 feature_id[91].value <= threshold=3.252573609352112
node_226: feature_name=GO:0036498 feature_id[359].value <= threshold=12.092026710510254
node_227: feature_name=hsa05340 feature_id[351].value <= threshold=0.7677814364433289
node_228: feature_name=GO:0032703 feature_id[617].value <= threshold=1.7450646758079529
node_229: feature_name=hsa04640 feature_id[79].value <= threshold=2.118402123451233
node_230: feature_name=GO:0042097 feature_id[675].value <= threshold=1.8110727667808533
node_231: feature_name=GO:0071391 feature_id[518].value <= threshold=2.691588521003723
node_232: feature_name=GO:0001666 feature_id[682].value <= threshold=5.225740432739258
node_233: feature_name=GO:0048539 feature_id[319].value <= threshold=1.8523842096328735
node_234: feature_name=GO:0038061 feature_id[671].value <= threshold=8.564527988433838
node_235: feature_name=GO:0002484 feature_id[600].value > threshold=3.1121134757995605
node_257: feature_name=GO:0045624 feature_id[750].value > threshold=0.2939649187028408
Class: positive genes

Rules_749 passed counts:1
node_0: feature_name=GO:0042113 feature_id[0].value <= threshold=13.408552169799805
node_1: feature_name=GO:0007568 feature_id[534].value <= threshold=5.0313897132873535

node_2: feature_name=GO:0002705	feature_id[541].value <= threshold=3.200145721435547
node_3: feature_name=GO:1901525	feature_id[576].value <= threshold=0.4466460347175598
node_4: feature_name=GO:0048539	feature_id[319].value <= threshold=3.0399646759033203
node_5: feature_name=GO:0001910	feature_id[385].value <= threshold=3.7437864542007446
node_6: feature_name=GO:0043200	feature_id[706].value <= threshold=9.307284355163574
node_7: feature_name=GO:0001773	feature_id[308].value <= threshold=4.016931533813477
node_8: feature_name=GO:0090116	feature_id[97].value <= threshold=7.99645471572876
node_9: feature_name=GO:0019814	feature_id[189].value <= threshold=4.525782108306885
node_10: feature_name=GO:1902583	feature_id[215].value <= threshold=17.60310649871826
node_11: feature_name=GO:0045429	feature_id[737].value <= threshold=1.6133361458778381
node_12: feature_name=GO:0003720	feature_id[228].value <= threshold=5.519326210021973
node_13: feature_name=GO:0046006	feature_id[364].value <= threshold=4.9985432624816895
node_14: feature_name=GO:0070424	feature_id[254].value <= threshold=6.233297109603882
node_15: feature_name=GO:0009892	feature_id[320].value <= threshold=59.99180793762207
node_16: feature_name=GO:0007064	feature_id[527].value <= threshold=11.57377815246582
node_17: feature_name=GO:0005575	feature_id[17].value <= threshold=15.417460918426514
node_18: feature_name=GO:0043368	feature_id[31].value <= threshold=4.059496641159058
node_19: feature_name=GO:0005164	feature_id[163].value <= threshold=5.793607711791992
node_20: feature_name=GO:0042130	feature_id[16].value <= threshold=5.073179721832275
node_21: feature_name=GO:0010216	feature_id[282].value <= threshold=4.834584474563599
node_22: feature_name=GO:0009628	feature_id[553].value <= threshold=29.314892768859863
node_23: feature_name=GO:0045628	feature_id[749].value <= threshold=3.1001367568969727
node_24: feature_name=GO:0042288	feature_id[100].value <= threshold=5.174932956695557
node_25: feature_name=GO:0002329	feature_id[406].value <= threshold=2.348930835723877
node_26: feature_name=GO:0051246	feature_id[642].value > threshold=0.6498909294605255
node_194: feature_name=GO:0009164	feature_id[546].value <= threshold=17.11970329284668
node_195: feature_name=GO:0001909	feature_id[386].value <= threshold=7.292566299438477
node_196: feature_name=GO:0003908	feature_id[11].value <= threshold=1.887226164340973
node_197: feature_name=GO:0009164	feature_id[546].value <= threshold=0.26642198860645294
node_198: feature_name=GO:2001242	feature_id[24].value <= threshold=9.207026481628418
node_199: feature_name=GO:0051246	feature_id[642].value > threshold=0.6504445374011993
node_203: feature_name=GO:0032461	feature_id[608].value <= threshold=2.607542634010315
node_204: feature_name=GO:0007584	feature_id[529].value <= threshold=16.099515914916992
node_205: feature_name=hsa05221	feature_id[349].value <= threshold=9.359850406646729
node_206: feature_name=GO:0002636	feature_id[447].value <= threshold=1.615447759628296

node_207: feature_name=GO:0033993 feature_id[421].value > threshold=2.96540611088858e-05
node_217: feature_name=GO:0002331 feature_id[404].value <= threshold=2.1883513927459717
node_218: feature_name=GO:0046498 feature_id[203].value <= threshold=4.085246205329895
node_219: feature_name=GO:0002832 feature_id[491].value <= threshold=3.0228612422943115
node_220: feature_name=GO:1904868 feature_id[306].value <= threshold=3.8674756288528442
node_221: feature_name=GO:0048545 feature_id[360].value <= threshold=27.38136100769043
node_222: feature_name=GO:0071310 feature_id[760].value <= threshold=2.3522024154663086
node_223: feature_name=GO:0001775 feature_id[505].value <= threshold=9.71301555633545
node_224: feature_name=GO:0071310 feature_id[760].value <= threshold=2.346743583679199
node_225: feature_name=GO:0001772 feature_id[91].value <= threshold=3.252573609352112
node_226: feature_name=GO:0036498 feature_id[359].value <= threshold=12.092026710510254
node_227: feature_name=hsa05340 feature_id[351].value <= threshold=0.7677814364433289
node_228: feature_name=GO:0032703 feature_id[617].value <= threshold=1.7450646758079529
node_229: feature_name=hsa04640 feature_id[79].value <= threshold=2.118402123451233
node_230: feature_name=GO:0042097 feature_id[675].value <= threshold=1.8110727667808533
node_231: feature_name=GO:0071391 feature_id[518].value <= threshold=2.691588521003723
node_232: feature_name=GO:0001666 feature_id[682].value <= threshold=5.225740432739258
node_233: feature_name=GO:0048539 feature_id[319].value <= threshold=1.8523842096328735
node_234: feature_name=GO:0038061 feature_id[671].value <= threshold=8.564527988433838
node_235: feature_name=GO:0002484 feature_id[600].value <= threshold=3.1121134757995605
node_236: feature_name=GO:0023023 feature_id[119].value > threshold=2.868617057800293
node_254: feature_name=GO:0019222 feature_id[129].value <= threshold=0.11867357417941093
Class: positive genes

Rules_750 passed counts:1
node_0: feature_name=GO:0042113 feature_id[0].value <= threshold=13.408552169799805
node_1: feature_name=GO:0007568 feature_id[534].value <= threshold=5.0313897132873535
node_2: feature_name=GO:0002705 feature_id[541].value <= threshold=3.200145721435547
node_3: feature_name=GO:1901525 feature_id[576].value <= threshold=0.4466460347175598
node_4: feature_name=GO:0048539 feature_id[319].value <= threshold=3.0399646759033203
node_5: feature_name=GO:0001910 feature_id[385].value <= threshold=3.7437864542007446
node_6: feature_name=GO:0043200 feature_id[706].value <= threshold=9.307284355163574
node_7: feature_name=GO:0001773 feature_id[308].value <= threshold=4.016931533813477
node_8: feature_name=GO:0090116 feature_id[97].value <= threshold=7.99645471572876
node_9: feature_name=GO:0019814 feature_id[189].value <= threshold=4.525782108306885

node_10: feature_name=GO:1902583	feature_id[215].value <= threshold=17.60310649871826
node_11: feature_name=GO:0045429	feature_id[737].value <= threshold=1.6133361458778381
node_12: feature_name=GO:0003720	feature_id[228].value <= threshold=5.519326210021973
node_13: feature_name=GO:0046006	feature_id[364].value <= threshold=4.9985432624816895
node_14: feature_name=GO:0070424	feature_id[254].value <= threshold=6.233297109603882
node_15: feature_name=GO:0009892	feature_id[320].value <= threshold=59.99180793762207
node_16: feature_name=GO:0007064	feature_id[527].value <= threshold=11.57377815246582
node_17: feature_name=GO:0005575	feature_id[17].value <= threshold=15.417460918426514
node_18: feature_name=GO:0043368	feature_id[31].value <= threshold=4.059496641159058
node_19: feature_name=GO:0005164	feature_id[163].value <= threshold=5.793607711791992
node_20: feature_name=GO:0042130	feature_id[16].value <= threshold=5.073179721832275
node_21: feature_name=GO:0010216	feature_id[282].value <= threshold=4.834584474563599
node_22: feature_name=GO:0009628	feature_id[553].value <= threshold=29.314892768859863
node_23: feature_name=GO:0045628	feature_id[749].value <= threshold=3.1001367568969727
node_24: feature_name=GO:0042288	feature_id[100].value <= threshold=5.174932956695557
node_25: feature_name=GO:0002329	feature_id[406].value <= threshold=2.348930835723877
node_26: feature_name=GO:0051246	feature_id[642].value > threshold=0.6498909294605255
node_194: feature_name=GO:0009164	feature_id[546].value <= threshold=17.11970329284668
node_195: feature_name=GO:0001909	feature_id[386].value <= threshold=7.292566299438477
node_196: feature_name=GO:0003908	feature_id[11].value <= threshold=1.887226164340973
node_197: feature_name=GO:0009164	feature_id[546].value <= threshold=0.26642198860645294
node_198: feature_name=GO:2001242	feature_id[24].value <= threshold=9.207026481628418
node_199: feature_name=GO:0051246	feature_id[642].value > threshold=0.6504445374011993
node_203: feature_name=GO:0032461	feature_id[608].value <= threshold=2.607542634010315
node_204: feature_name=GO:0007584	feature_id[529].value <= threshold=16.099515914916992
node_205: feature_name=hsa05221	feature_id[349].value <= threshold=9.359850406646729
node_206: feature_name=GO:0002636	feature_id[447].value <= threshold=1.615447759628296
node_207: feature_name=GO:0033993	feature_id[421].value > threshold=2.96540611088858e-05
node_217: feature_name=GO:0002331	feature_id[404].value <= threshold=2.1883513927459717
node_218: feature_name=GO:0046498	feature_id[203].value <= threshold=4.085246205329895
node_219: feature_name=GO:0002832	feature_id[491].value <= threshold=3.0228612422943115
node_220: feature_name=GO:1904868	feature_id[306].value <= threshold=3.8674756288528442
node_221: feature_name=GO:0048545	feature_id[360].value <= threshold=27.38136100769043
node_222: feature_name=GO:0071310	feature_id[760].value <= threshold=2.3522024154663086
node_223: feature_name=GO:0001775	feature_id[505].value <= threshold=9.71301555633545

node_224: feature_name=GO:0071310 feature_id[760].value <= threshold=2.346743583679199
node_225: feature_name=GO:0001772 feature_id[91].value <= threshold=3.252573609352112
node_226: feature_name=GO:0036498 feature_id[359].value <= threshold=12.092026710510254
node_227: feature_name=hsa05340 feature_id[351].value <= threshold=0.7677814364433289
node_228: feature_name=GO:0032703 feature_id[617].value <= threshold=1.7450646758079529
node_229: feature_name=hsa04640 feature_id[79].value <= threshold=2.118402123451233
node_230: feature_name=GO:0042097 feature_id[675].value <= threshold=1.8110727667808533
node_231: feature_name=GO:0071391 feature_id[518].value <= threshold=2.691588521003723
node_232: feature_name=GO:0001666 feature_id[682].value <= threshold=5.225740432739258
node_233: feature_name=GO:0048539 feature_id[319].value <= threshold=1.8523842096328735
node_234: feature_name=GO:0038061 feature_id[671].value <= threshold=8.564527988433838
node_235: feature_name=GO:0002484 feature_id[600].value <= threshold=3.1121134757995605
node_236: feature_name=GO:0023023 feature_id[119].value <= threshold=2.868617057800293
node_237: feature_name=GO:0010639 feature_id[564].value > threshold=13.410505771636963
node_251: feature_name=GO:0002696 feature_id[462].value > threshold=0.6140799224376678
Class: positive genes

Rules_751 passed counts:1
node_0: feature_name=GO:0042113 feature_id[0].value <= threshold=13.408552169799805
node_1: feature_name=GO:0007568 feature_id[534].value <= threshold=5.0313897132873535
node_2: feature_name=GO:0002705 feature_id[541].value <= threshold=3.200145721435547
node_3: feature_name=GO:1901525 feature_id[576].value <= threshold=0.4466460347175598
node_4: feature_name=GO:0048539 feature_id[319].value <= threshold=3.0399646759033203
node_5: feature_name=GO:0001910 feature_id[385].value <= threshold=3.7437864542007446
node_6: feature_name=GO:0043200 feature_id[706].value <= threshold=9.307284355163574
node_7: feature_name=GO:0001773 feature_id[308].value <= threshold=4.016931533813477
node_8: feature_name=GO:0090116 feature_id[97].value <= threshold=7.99645471572876
node_9: feature_name=GO:0019814 feature_id[189].value <= threshold=4.525782108306885
node_10: feature_name=GO:1902583 feature_id[215].value <= threshold=17.60310649871826
node_11: feature_name=GO:0045429 feature_id[737].value <= threshold=1.6133361458778381
node_12: feature_name=GO:0003720 feature_id[228].value <= threshold=5.519326210021973
node_13: feature_name=GO:0046006 feature_id[364].value <= threshold=4.9985432624816895
node_14: feature_name=GO:0070424 feature_id[254].value <= threshold=6.233297109603882
node_15: feature_name=GO:0009892 feature_id[320].value <= threshold=59.99180793762207
node_16: feature_name=GO:0007064 feature_id[527].value <= threshold=11.57377815246582

node_17: feature_name=GO:0005575	feature_id[17].value <= threshold=15.417460918426514
node_18: feature_name=GO:0043368	feature_id[31].value <= threshold=4.059496641159058
node_19: feature_name=GO:0005164	feature_id[163].value <= threshold=5.793607711791992
node_20: feature_name=GO:0042130	feature_id[16].value <= threshold=5.073179721832275
node_21: feature_name=GO:0010216	feature_id[282].value <= threshold=4.834584474563599
node_22: feature_name=GO:0009628	feature_id[553].value <= threshold=29.314892768859863
node_23: feature_name=GO:0045628	feature_id[749].value <= threshold=3.1001367568969727
node_24: feature_name=GO:0042288	feature_id[100].value <= threshold=5.174932956695557
node_25: feature_name=GO:0002329	feature_id[406].value <= threshold=2.348930835723877
node_26: feature_name=GO:0051246	feature_id[642].value > threshold=0.6498909294605255
node_194: feature_name=GO:0009164	feature_id[546].value <= threshold=17.11970329284668
node_195: feature_name=GO:0001909	feature_id[386].value <= threshold=7.292566299438477
node_196: feature_name=GO:0003908	feature_id[11].value <= threshold=1.887226164340973
node_197: feature_name=GO:0009164	feature_id[546].value <= threshold=0.26642198860645294
node_198: feature_name=GO:2001242	feature_id[24].value <= threshold=9.207026481628418
node_199: feature_name=GO:0051246	feature_id[642].value > threshold=0.6504445374011993
node_203: feature_name=GO:0032461	feature_id[608].value <= threshold=2.607542634010315
node_204: feature_name=GO:0007584	feature_id[529].value <= threshold=16.099515914916992
node_205: feature_name=hsa05221	feature_id[349].value <= threshold=9.359850406646729
node_206: feature_name=GO:0002636	feature_id[447].value <= threshold=1.615447759628296
node_207: feature_name=GO:0033993	feature_id[421].value > threshold=2.96540611088858e-05
node_217: feature_name=GO:0002331	feature_id[404].value <= threshold=2.1883513927459717
node_218: feature_name=GO:0046498	feature_id[203].value <= threshold=4.085246205329895
node_219: feature_name=GO:0002832	feature_id[491].value <= threshold=3.0228612422943115
node_220: feature_name=GO:1904868	feature_id[306].value <= threshold=3.8674756288528442
node_221: feature_name=GO:0048545	feature_id[360].value <= threshold=27.38136100769043
node_222: feature_name=GO:0071310	feature_id[760].value <= threshold=2.3522024154663086
node_223: feature_name=GO:0001775	feature_id[505].value <= threshold=9.71301555633545
node_224: feature_name=GO:0071310	feature_id[760].value <= threshold=2.346743583679199
node_225: feature_name=GO:0001772	feature_id[91].value <= threshold=3.252573609352112
node_226: feature_name=GO:0036498	feature_id[359].value <= threshold=12.092026710510254
node_227: feature_name=hsa05340	feature_id[351].value <= threshold=0.7677814364433289
node_228: feature_name=GO:0032703	feature_id[617].value <= threshold=1.7450646758079529
node_229: feature_name=hsa04640	feature_id[79].value <= threshold=2.118402123451233
node_230: feature_name=GO:0042097	feature_id[675].value <= threshold=1.8110727667808533

node_231: feature_name=GO:0071391 feature_id[518].value <= threshold=2.691588521003723
node_232: feature_name=GO:0001666 feature_id[682].value <= threshold=5.225740432739258
node_233: feature_name=GO:0048539 feature_id[319].value <= threshold=1.8523842096328735
node_234: feature_name=GO:0038061 feature_id[671].value <= threshold=8.564527988433838
node_235: feature_name=GO:0002484 feature_id[600].value <= threshold=3.1121134757995605
node_236: feature_name=GO:0023023 feature_id[119].value <= threshold=2.868617057800293
node_237: feature_name=GO:0010639 feature_id[564].value <= threshold=13.410505771636963
node_238: feature_name=GO:0080134 feature_id[650].value > threshold=0.013442101422697306
node_244: feature_name=hsa04060 feature_id[331].value > threshold=0.7175639569759369
node_246: feature_name=hsa04060 feature_id[331].value > threshold=0.7203521430492401
node_248: feature_name=hsa04110 feature_id[328].value > threshold=2.0177602767944336
Class: positive genes

Rules_752 passed counts:1
node_0: feature_name=GO:0042113 feature_id[0].value <= threshold=13.408552169799805
node_1: feature_name=GO:0007568 feature_id[534].value <= threshold=5.0313897132873535
node_2: feature_name=GO:0002705 feature_id[541].value <= threshold=3.200145721435547
node_3: feature_name=GO:1901525 feature_id[576].value <= threshold=0.4466460347175598
node_4: feature_name=GO:0048539 feature_id[319].value <= threshold=3.0399646759033203
node_5: feature_name=GO:0001910 feature_id[385].value <= threshold=3.7437864542007446
node_6: feature_name=GO:0043200 feature_id[706].value <= threshold=9.307284355163574
node_7: feature_name=GO:0001773 feature_id[308].value <= threshold=4.016931533813477
node_8: feature_name=GO:0090116 feature_id[97].value <= threshold=7.99645471572876
node_9: feature_name=GO:0019814 feature_id[189].value <= threshold=4.525782108306885
node_10: feature_name=GO:1902583 feature_id[215].value <= threshold=17.60310649871826
node_11: feature_name=GO:0045429 feature_id[737].value <= threshold=1.6133361458778381
node_12: feature_name=GO:0003720 feature_id[228].value <= threshold=5.519326210021973
node_13: feature_name=GO:0046006 feature_id[364].value <= threshold=4.9985432624816895
node_14: feature_name=GO:0070424 feature_id[254].value <= threshold=6.233297109603882
node_15: feature_name=GO:0009892 feature_id[320].value <= threshold=59.99180793762207
node_16: feature_name=GO:0007064 feature_id[527].value <= threshold=11.57377815246582
node_17: feature_name=GO:0005575 feature_id[17].value <= threshold=15.417460918426514
node_18: feature_name=GO:0043368 feature_id[31].value <= threshold=4.059496641159058
node_19: feature_name=GO:0005164 feature_id[163].value <= threshold=5.793607711791992
node_20: feature_name=GO:0042130 feature_id[16].value <= threshold=5.073179721832275

node_21: feature_name=GO:0010216 feature_id[282].value <= threshold=4.834584474563599
node_22: feature_name=GO:0009628 feature_id[553].value <= threshold=29.314892768859863
node_23: feature_name=GO:0045628 feature_id[749].value <= threshold=3.1001367568969727
node_24: feature_name=GO:0042288 feature_id[100].value <= threshold=5.174932956695557
node_25: feature_name=GO:0002329 feature_id[406].value <= threshold=2.348930835723877
node_26: feature_name=GO:0051246 feature_id[642].value > threshold=0.6498909294605255
node_194: feature_name=GO:0009164 feature_id[546].value <= threshold=17.11970329284668
node_195: feature_name=GO:0001909 feature_id[386].value <= threshold=7.292566299438477
node_196: feature_name=GO:0003908 feature_id[11].value <= threshold=1.887226164340973
node_197: feature_name=GO:0009164 feature_id[546].value <= threshold=0.26642198860645294
node_198: feature_name=GO:2001242 feature_id[24].value <= threshold=9.207026481628418
node_199: feature_name=GO:0051246 feature_id[642].value > threshold=0.6504445374011993
node_203: feature_name=GO:0032461 feature_id[608].value <= threshold=2.607542634010315
node_204: feature_name=GO:0007584 feature_id[529].value <= threshold=16.099515914916992
node_205: feature_name=hsa05221 feature_id[349].value <= threshold=9.359850406646729
node_206: feature_name=GO:0002636 feature_id[447].value <= threshold=1.615447759628296
node_207: feature_name=GO:0033993 feature_id[421].value > threshold=2.96540611088858e-05
node_217: feature_name=GO:0002331 feature_id[404].value <= threshold=2.1883513927459717
node_218: feature_name=GO:0046498 feature_id[203].value <= threshold=4.085246205329895
node_219: feature_name=GO:0002832 feature_id[491].value <= threshold=3.0228612422943115
node_220: feature_name=GO:1904868 feature_id[306].value <= threshold=3.8674756288528442
node_221: feature_name=GO:0048545 feature_id[360].value <= threshold=27.38136100769043
node_222: feature_name=GO:0071310 feature_id[760].value <= threshold=2.3522024154663086
node_223: feature_name=GO:0001775 feature_id[505].value <= threshold=9.71301555633545
node_224: feature_name=GO:0071310 feature_id[760].value <= threshold=2.346743583679199
node_225: feature_name=GO:0001772 feature_id[91].value <= threshold=3.252573609352112
node_226: feature_name=GO:0036498 feature_id[359].value <= threshold=12.092026710510254
node_227: feature_name=hsa05340 feature_id[351].value <= threshold=0.7677814364433289
node_228: feature_name=GO:0032703 feature_id[617].value <= threshold=1.7450646758079529
node_229: feature_name=hsa04640 feature_id[79].value <= threshold=2.118402123451233
node_230: feature_name=GO:0042097 feature_id[675].value <= threshold=1.8110727667808533
node_231: feature_name=GO:0071391 feature_id[518].value <= threshold=2.691588521003723
node_232: feature_name=GO:0001666 feature_id[682].value <= threshold=5.225740432739258
node_233: feature_name=GO:0048539 feature_id[319].value <= threshold=1.8523842096328735
node_234: feature_name=GO:0038061 feature_id[671].value <= threshold=8.564527988433838

node_235: feature_name=GO:0002484 feature_id[600].value <= threshold=3.1121134757995605
node_236: feature_name=GO:0023023 feature_id[119].value <= threshold=2.868617057800293
node_237: feature_name=GO:0010639 feature_id[564].value <= threshold=13.410505771636963
node_238: feature_name=GO:0080134 feature_id[650].value > threshold=0.013442101422697306
node_244: feature_name=hsa04060 feature_id[331].value > threshold=0.7175639569759369
node_246: feature_name=hsa04060 feature_id[331].value <= threshold=0.7203521430492401
Class: positive genes

Rules_753 passed counts:1
node_0: feature_name=GO:0042113 feature_id[0].value <= threshold=13.408552169799805
node_1: feature_name=GO:0007568 feature_id[534].value <= threshold=5.0313897132873535
node_2: feature_name=GO:0002705 feature_id[541].value <= threshold=3.200145721435547
node_3: feature_name=GO:1901525 feature_id[576].value <= threshold=0.4466460347175598
node_4: feature_name=GO:0048539 feature_id[319].value <= threshold=3.0399646759033203
node_5: feature_name=GO:0001910 feature_id[385].value <= threshold=3.7437864542007446
node_6: feature_name=GO:0043200 feature_id[706].value <= threshold=9.307284355163574
node_7: feature_name=GO:0001773 feature_id[308].value <= threshold=4.016931533813477
node_8: feature_name=GO:0090116 feature_id[97].value <= threshold=7.99645471572876
node_9: feature_name=GO:0019814 feature_id[189].value <= threshold=4.525782108306885
node_10: feature_name=GO:1902583 feature_id[215].value <= threshold=17.60310649871826
node_11: feature_name=GO:0045429 feature_id[737].value <= threshold=1.6133361458778381
node_12: feature_name=GO:0003720 feature_id[228].value <= threshold=5.519326210021973
node_13: feature_name=GO:0046006 feature_id[364].value <= threshold=4.9985432624816895
node_14: feature_name=GO:0070424 feature_id[254].value <= threshold=6.233297109603882
node_15: feature_name=GO:0009892 feature_id[320].value <= threshold=59.99180793762207
node_16: feature_name=GO:0007064 feature_id[527].value <= threshold=11.57377815246582
node_17: feature_name=GO:0005575 feature_id[17].value <= threshold=15.417460918426514
node_18: feature_name=GO:0043368 feature_id[31].value <= threshold=4.059496641159058
node_19: feature_name=GO:0005164 feature_id[163].value <= threshold=5.793607711791992
node_20: feature_name=GO:0042130 feature_id[16].value <= threshold=5.073179721832275
node_21: feature_name=GO:0010216 feature_id[282].value <= threshold=4.834584474563599
node_22: feature_name=GO:0009628 feature_id[553].value <= threshold=29.314892768859863
node_23: feature_name=GO:0045628 feature_id[749].value <= threshold=3.1001367568969727
node_24: feature_name=GO:0042288 feature_id[100].value <= threshold=5.174932956695557
node_25: feature_name=GO:0002329 feature_id[406].value <= threshold=2.348930835723877

node_26: feature_name=GO:0051246	feature_id[642].value > threshold=0.6498909294605255
node_194: feature_name=GO:0009164	feature_id[546].value <= threshold=17.11970329284668
node_195: feature_name=GO:0001909	feature_id[386].value <= threshold=7.292566299438477
node_196: feature_name=GO:0003908	feature_id[11].value <= threshold=1.887226164340973
node_197: feature_name=GO:0009164	feature_id[546].value <= threshold=0.26642198860645294
node_198: feature_name=GO:2001242	feature_id[24].value <= threshold=9.207026481628418
node_199: feature_name=GO:0051246	feature_id[642].value > threshold=0.6504445374011993
node_203: feature_name=GO:0032461	feature_id[608].value <= threshold=2.607542634010315
node_204: feature_name=GO:0007584	feature_id[529].value <= threshold=16.099515914916992
node_205: feature_name=hsa05221	feature_id[349].value <= threshold=9.359850406646729
node_206: feature_name=GO:0002636	feature_id[447].value <= threshold=1.615447759628296
node_207: feature_name=GO:0033993	feature_id[421].value > threshold=2.96540611088858e-05
node_217: feature_name=GO:0002331	feature_id[404].value <= threshold=2.1883513927459717
node_218: feature_name=GO:0046498	feature_id[203].value <= threshold=4.085246205329895
node_219: feature_name=GO:0002832	feature_id[491].value <= threshold=3.0228612422943115
node_220: feature_name=GO:1904868	feature_id[306].value <= threshold=3.8674756288528442
node_221: feature_name=GO:0048545	feature_id[360].value <= threshold=27.38136100769043
node_222: feature_name=GO:0071310	feature_id[760].value <= threshold=2.3522024154663086
node_223: feature_name=GO:0001775	feature_id[505].value <= threshold=9.71301555633545
node_224: feature_name=GO:0071310	feature_id[760].value <= threshold=2.346743583679199
node_225: feature_name=GO:0001772	feature_id[91].value <= threshold=3.252573609352112
node_226: feature_name=GO:0036498	feature_id[359].value <= threshold=12.092026710510254
node_227: feature_name=hsa05340	feature_id[351].value <= threshold=0.7677814364433289
node_228: feature_name=GO:0032703	feature_id[617].value <= threshold=1.7450646758079529
node_229: feature_name=hsa04640	feature_id[79].value <= threshold=2.118402123451233
node_230: feature_name=GO:0042097	feature_id[675].value <= threshold=1.8110727667808533
node_231: feature_name=GO:0071391	feature_id[518].value <= threshold=2.691588521003723
node_232: feature_name=GO:0001666	feature_id[682].value <= threshold=5.225740432739258
node_233: feature_name=GO:0048539	feature_id[319].value <= threshold=1.8523842096328735
node_234: feature_name=GO:0038061	feature_id[671].value <= threshold=8.564527988433838
node_235: feature_name=GO:0002484	feature_id[600].value <= threshold=3.1121134757995605
node_236: feature_name=GO:0023023	feature_id[119].value <= threshold=2.868617057800293
node_237: feature_name=GO:0010639	feature_id[564].value <= threshold=13.410505771636963
node_238: feature_name=GO:0080134	feature_id[650].value <= threshold=0.013442101422697306
node_239: feature_name=GO:0042991	feature_id[684].value > threshold=0.9682981073856354

Class: positive genes

Rules_754	passed counts:1
node_0: feature_name=GO:0042113	feature_id[0].value <= threshold=13.408552169799805
node_1: feature_name=GO:0007568	feature_id[534].value <= threshold=5.0313897132873535
node_2: feature_name=GO:0002705	feature_id[541].value <= threshold=3.200145721435547
node_3: feature_name=GO:1901525	feature_id[576].value <= threshold=0.4466460347175598
node_4: feature_name=GO:0048539	feature_id[319].value <= threshold=3.0399646759033203
node_5: feature_name=GO:0001910	feature_id[385].value <= threshold=3.7437864542007446
node_6: feature_name=GO:0043200	feature_id[706].value <= threshold=9.307284355163574
node_7: feature_name=GO:0001773	feature_id[308].value <= threshold=4.016931533813477
node_8: feature_name=GO:0090116	feature_id[97].value <= threshold=7.99645471572876
node_9: feature_name=GO:0019814	feature_id[189].value <= threshold=4.525782108306885
node_10: feature_name=GO:1902583	feature_id[215].value <= threshold=17.60310649871826
node_11: feature_name=GO:0045429	feature_id[737].value <= threshold=1.6133361458778381
node_12: feature_name=GO:0003720	feature_id[228].value <= threshold=5.519326210021973
node_13: feature_name=GO:0046006	feature_id[364].value <= threshold=4.9985432624816895
node_14: feature_name=GO:0070424	feature_id[254].value <= threshold=6.233297109603882
node_15: feature_name=GO:0009892	feature_id[320].value <= threshold=59.99180793762207
node_16: feature_name=GO:0007064	feature_id[527].value <= threshold=11.57377815246582
node_17: feature_name=GO:0005575	feature_id[17].value <= threshold=15.417460918426514
node_18: feature_name=GO:0043368	feature_id[31].value <= threshold=4.059496641159058
node_19: feature_name=GO:0005164	feature_id[163].value <= threshold=5.793607711791992
node_20: feature_name=GO:0042130	feature_id[16].value <= threshold=5.073179721832275
node_21: feature_name=GO:0010216	feature_id[282].value <= threshold=4.834584474563599
node_22: feature_name=GO:0009628	feature_id[553].value <= threshold=29.314892768859863
node_23: feature_name=GO:0045628	feature_id[749].value <= threshold=3.1001367568969727
node_24: feature_name=GO:0042288	feature_id[100].value <= threshold=5.174932956695557
node_25: feature_name=GO:0002329	feature_id[406].value <= threshold=2.348930835723877
node_26: feature_name=GO:0051246	feature_id[642].value > threshold=0.6498909294605255
node_194: feature_name=GO:0009164	feature_id[546].value <= threshold=17.11970329284668
node_195: feature_name=GO:0001909	feature_id[386].value <= threshold=7.292566299438477
node_196: feature_name=GO:0003908	feature_id[11].value <= threshold=1.887226164340973
node_197: feature_name=GO:0009164	feature_id[546].value <= threshold=0.26642198860645294
node_198: feature_name=GO:2001242	feature_id[24].value <= threshold=9.207026481628418

node_199: feature_name=GO:0051246 feature_id[642].value > threshold=0.6504445374011993
node_203: feature_name=GO:0032461 feature_id[608].value <= threshold=2.607542634010315
node_204: feature_name=GO:0007584 feature_id[529].value <= threshold=16.099515914916992
node_205: feature_name=hsa05221 feature_id[349].value <= threshold=9.359850406646729
node_206: feature_name=GO:0002636 feature_id[447].value <= threshold=1.615447759628296
node_207: feature_name=GO:0033993 feature_id[421].value > threshold=2.96540611088858e-05
node_217: feature_name=GO:0002331 feature_id[404].value <= threshold=2.1883513927459717
node_218: feature_name=GO:0046498 feature_id[203].value <= threshold=4.085246205329895
node_219: feature_name=GO:0002832 feature_id[491].value <= threshold=3.0228612422943115
node_220: feature_name=GO:1904868 feature_id[306].value <= threshold=3.8674756288528442
node_221: feature_name=GO:0048545 feature_id[360].value <= threshold=27.38136100769043
node_222: feature_name=GO:0071310 feature_id[760].value <= threshold=2.3522024154663086
node_223: feature_name=GO:0001775 feature_id[505].value <= threshold=9.71301555633545
node_224: feature_name=GO:0071310 feature_id[760].value <= threshold=2.346743583679199
node_225: feature_name=GO:0001772 feature_id[91].value <= threshold=3.252573609352112
node_226: feature_name=GO:0036498 feature_id[359].value <= threshold=12.092026710510254
node_227: feature_name=hsa05340 feature_id[351].value <= threshold=0.7677814364433289
node_228: feature_name=GO:0032703 feature_id[617].value <= threshold=1.7450646758079529
node_229: feature_name=hsa04640 feature_id[79].value <= threshold=2.118402123451233
node_230: feature_name=GO:0042097 feature_id[675].value <= threshold=1.8110727667808533
node_231: feature_name=GO:0071391 feature_id[518].value <= threshold=2.691588521003723
node_232: feature_name=GO:0001666 feature_id[682].value <= threshold=5.225740432739258
node_233: feature_name=GO:0048539 feature_id[319].value <= threshold=1.8523842096328735
node_234: feature_name=GO:0038061 feature_id[671].value <= threshold=8.564527988433838
node_235: feature_name=GO:0002484 feature_id[600].value <= threshold=3.1121134757995605
node_236: feature_name=GO:0023023 feature_id[119].value <= threshold=2.868617057800293
node_237: feature_name=GO:0010639 feature_id[564].value <= threshold=13.410505771636963
node_238: feature_name=GO:0080134 feature_id[650].value <= threshold=0.013442101422697306
node_239: feature_name=GO:0042991 feature_id[684].value <= threshold=0.9682981073856354
node_240: feature_name=GO:0050707 feature_id[797].value > threshold=1.0614736676216125
Class: positive genes

Rules_755 passed counts:1
node_0: feature_name=GO:0042113 feature_id[0].value <= threshold=13.408552169799805
node_1: feature_name=GO:0007568 feature_id[534].value <= threshold=5.0313897132873535

node_2: feature_name=GO:0002705 feature_id[541].value <= threshold=3.200145721435547
node_3: feature_name=GO:1901525 feature_id[576].value <= threshold=0.4466460347175598
node_4: feature_name=GO:0048539 feature_id[319].value <= threshold=3.0399646759033203
node_5: feature_name=GO:0001910 feature_id[385].value <= threshold=3.7437864542007446
node_6: feature_name=GO:0043200 feature_id[706].value <= threshold=9.307284355163574
node_7: feature_name=GO:0001773 feature_id[308].value <= threshold=4.016931533813477
node_8: feature_name=GO:0090116 feature_id[97].value <= threshold=7.99645471572876
node_9: feature_name=GO:0019814 feature_id[189].value <= threshold=4.525782108306885
node_10: feature_name=GO:1902583 feature_id[215].value <= threshold=17.60310649871826
node_11: feature_name=GO:0045429 feature_id[737].value <= threshold=1.6133361458778381
node_12: feature_name=GO:0003720 feature_id[228].value <= threshold=5.519326210021973
node_13: feature_name=GO:0046006 feature_id[364].value <= threshold=4.9985432624816895
node_14: feature_name=GO:0070424 feature_id[254].value <= threshold=6.233297109603882
node_15: feature_name=GO:0009892 feature_id[320].value <= threshold=59.99180793762207
node_16: feature_name=GO:0007064 feature_id[527].value <= threshold=11.57377815246582
node_17: feature_name=GO:0005575 feature_id[17].value <= threshold=15.417460918426514
node_18: feature_name=GO:0043368 feature_id[31].value <= threshold=4.059496641159058
node_19: feature_name=GO:0005164 feature_id[163].value <= threshold=5.793607711791992
node_20: feature_name=GO:0042130 feature_id[16].value <= threshold=5.073179721832275
node_21: feature_name=GO:0010216 feature_id[282].value <= threshold=4.834584474563599
node_22: feature_name=GO:0009628 feature_id[553].value <= threshold=29.314892768859863
node_23: feature_name=GO:0045628 feature_id[749].value <= threshold=3.1001367568969727
node_24: feature_name=GO:0042288 feature_id[100].value <= threshold=5.174932956695557
node_25: feature_name=GO:0002329 feature_id[406].value <= threshold=2.348930835723877
node_26: feature_name=GO:0051246 feature_id[642].value > threshold=0.6498909294605255
node_194: feature_name=GO:0009164 feature_id[546].value <= threshold=17.11970329284668
node_195: feature_name=GO:0001909 feature_id[386].value <= threshold=7.292566299438477
node_196: feature_name=GO:0003908 feature_id[11].value <= threshold=1.887226164340973
node_197: feature_name=GO:0009164 feature_id[546].value <= threshold=0.26642198860645294
node_198: feature_name=GO:2001242 feature_id[24].value <= threshold=9.207026481628418
node_199: feature_name=GO:0051246 feature_id[642].value <= threshold=0.6504445374011993
node_200: feature_name=GO:0071887 feature_id[283].value > threshold=0.13689306378364563
Class: negative genes

Rules_756 passed counts:1

node_0: feature_name=GO:0042113	feature_id[0].value <= threshold=13.408552169799805
node_1: feature_name=GO:0007568	feature_id[534].value <= threshold=5.0313897132873535
node_2: feature_name=GO:0002705	feature_id[541].value <= threshold=3.200145721435547
node_3: feature_name=GO:1901525	feature_id[576].value <= threshold=0.4466460347175598
node_4: feature_name=GO:0048539	feature_id[319].value <= threshold=3.0399646759033203
node_5: feature_name=GO:0001910	feature_id[385].value <= threshold=3.7437864542007446
node_6: feature_name=GO:0043200	feature_id[706].value <= threshold=9.307284355163574
node_7: feature_name=GO:0001773	feature_id[308].value <= threshold=4.016931533813477
node_8: feature_name=GO:0090116	feature_id[97].value <= threshold=7.99645471572876
node_9: feature_name=GO:0019814	feature_id[189].value <= threshold=4.525782108306885
node_10: feature_name=GO:1902583	feature_id[215].value <= threshold=17.60310649871826
node_11: feature_name=GO:0045429	feature_id[737].value <= threshold=1.6133361458778381
node_12: feature_name=GO:0003720	feature_id[228].value <= threshold=5.519326210021973
node_13: feature_name=GO:0046006	feature_id[364].value <= threshold=4.9985432624816895
node_14: feature_name=GO:0070424	feature_id[254].value <= threshold=6.233297109603882
node_15: feature_name=GO:0009892	feature_id[320].value <= threshold=59.99180793762207
node_16: feature_name=GO:0007064	feature_id[527].value <= threshold=11.57377815246582
node_17: feature_name=GO:0005575	feature_id[17].value <= threshold=15.417460918426514
node_18: feature_name=GO:0043368	feature_id[31].value <= threshold=4.059496641159058
node_19: feature_name=GO:0005164	feature_id[163].value <= threshold=5.793607711791992
node_20: feature_name=GO:0042130	feature_id[16].value <= threshold=5.073179721832275
node_21: feature_name=GO:0010216	feature_id[282].value <= threshold=4.834584474563599
node_22: feature_name=GO:0009628	feature_id[553].value <= threshold=29.314892768859863
node_23: feature_name=GO:0045628	feature_id[749].value <= threshold=3.1001367568969727
node_24: feature_name=GO:0042288	feature_id[100].value <= threshold=5.174932956695557
node_25: feature_name=GO:0002329	feature_id[406].value <= threshold=2.348930835723877
node_26: feature_name=GO:0051246	feature_id[642].value > threshold=0.6498909294605255
node_194: feature_name=GO:0009164	feature_id[546].value <= threshold=17.11970329284668
node_195: feature_name=GO:0001909	feature_id[386].value <= threshold=7.292566299438477
node_196: feature_name=GO:0003908	feature_id[11].value <= threshold=1.887226164340973
node_197: feature_name=GO:0009164	feature_id[546].value <= threshold=0.26642198860645294
node_198: feature_name=GO:2001242	feature_id[24].value <= threshold=9.207026481628418
node_199: feature_name=GO:0051246	feature_id[642].value <= threshold=0.6504445374011993
node_200: feature_name=GO:0071887	feature_id[283].value <= threshold=0.13689306378364563

Class: positive genes

Rules_757 passed counts:1
node_0: feature_name=GO:0042113 feature_id[0].value <= threshold=13.408552169799805
node_1: feature_name=GO:0007568 feature_id[534].value <= threshold=5.0313897132873535
node_2: feature_name=GO:0002705 feature_id[541].value <= threshold=3.200145721435547
node_3: feature_name=GO:1901525 feature_id[576].value <= threshold=0.4466460347175598
node_4: feature_name=GO:0048539 feature_id[319].value <= threshold=3.0399646759033203
node_5: feature_name=GO:0001910 feature_id[385].value <= threshold=3.7437864542007446
node_6: feature_name=GO:0043200 feature_id[706].value <= threshold=9.307284355163574
node_7: feature_name=GO:0001773 feature_id[308].value <= threshold=4.016931533813477
node_8: feature_name=GO:0090116 feature_id[97].value <= threshold=7.99645471572876
node_9: feature_name=GO:0019814 feature_id[189].value <= threshold=4.525782108306885
node_10: feature_name=GO:1902583 feature_id[215].value <= threshold=17.60310649871826
node_11: feature_name=GO:0045429 feature_id[737].value <= threshold=1.6133361458778381
node_12: feature_name=GO:0003720 feature_id[228].value <= threshold=5.519326210021973
node_13: feature_name=GO:0046006 feature_id[364].value <= threshold=4.9985432624816895
node_14: feature_name=GO:0070424 feature_id[254].value <= threshold=6.233297109603882
node_15: feature_name=GO:0009892 feature_id[320].value <= threshold=59.99180793762207
node_16: feature_name=GO:0007064 feature_id[527].value <= threshold=11.57377815246582
node_17: feature_name=GO:0005575 feature_id[17].value <= threshold=15.417460918426514
node_18: feature_name=GO:0043368 feature_id[31].value <= threshold=4.059496641159058
node_19: feature_name=GO:0005164 feature_id[163].value <= threshold=5.793607711791992
node_20: feature_name=GO:0042130 feature_id[16].value <= threshold=5.073179721832275
node_21: feature_name=GO:0010216 feature_id[282].value <= threshold=4.834584474563599
node_22: feature_name=GO:0009628 feature_id[553].value <= threshold=29.314892768859863
node_23: feature_name=GO:0045628 feature_id[749].value <= threshold=3.1001367568969727
node_24: feature_name=GO:0042288 feature_id[100].value <= threshold=5.174932956695557
node_25: feature_name=GO:0002329 feature_id[406].value <= threshold=2.348930835723877
node_26: feature_name=GO:0051246 feature_id[642].value <= threshold=0.6498909294605255
node_27: feature_name=GO:0042162 feature_id[355].value > threshold=8.05103588104248
Class: positive genes

Rules_758 passed counts:1
node_0: feature_name=GO:0042113 feature_id[0].value <= threshold=13.408552169799805
node_1: feature_name=GO:0007568 feature_id[534].value <= threshold=5.0313897132873535

node_2: feature_name=GO:0002705 feature_id[541].value <= threshold=3.200145721435547
node_3: feature_name=GO:1901525 feature_id[576].value <= threshold=0.4466460347175598
node_4: feature_name=GO:0048539 feature_id[319].value <= threshold=3.0399646759033203
node_5: feature_name=GO:0001910 feature_id[385].value <= threshold=3.7437864542007446
node_6: feature_name=GO:0043200 feature_id[706].value <= threshold=9.307284355163574
node_7: feature_name=GO:0001773 feature_id[308].value <= threshold=4.016931533813477
node_8: feature_name=GO:0090116 feature_id[97].value <= threshold=7.99645471572876
node_9: feature_name=GO:0019814 feature_id[189].value <= threshold=4.525782108306885
node_10: feature_name=GO:1902583 feature_id[215].value <= threshold=17.60310649871826
node_11: feature_name=GO:0045429 feature_id[737].value <= threshold=1.6133361458778381
node_12: feature_name=GO:0003720 feature_id[228].value <= threshold=5.519326210021973
node_13: feature_name=GO:0046006 feature_id[364].value <= threshold=4.9985432624816895
node_14: feature_name=GO:0070424 feature_id[254].value <= threshold=6.233297109603882
node_15: feature_name=GO:0009892 feature_id[320].value <= threshold=59.99180793762207
node_16: feature_name=GO:0007064 feature_id[527].value <= threshold=11.57377815246582
node_17: feature_name=GO:0005575 feature_id[17].value <= threshold=15.417460918426514
node_18: feature_name=GO:0043368 feature_id[31].value <= threshold=4.059496641159058
node_19: feature_name=GO:0005164 feature_id[163].value <= threshold=5.793607711791992
node_20: feature_name=GO:0042130 feature_id[16].value <= threshold=5.073179721832275
node_21: feature_name=GO:0010216 feature_id[282].value <= threshold=4.834584474563599
node_22: feature_name=GO:0009628 feature_id[553].value <= threshold=29.314892768859863
node_23: feature_name=GO:0045628 feature_id[749].value <= threshold=3.1001367568969727
node_24: feature_name=GO:0042288 feature_id[100].value <= threshold=5.174932956695557
node_25: feature_name=GO:0002329 feature_id[406].value <= threshold=2.348930835723877
node_26: feature_name=GO:0051246 feature_id[642].value <= threshold=0.6498909294605255
node_27: feature_name=GO:0042162 feature_id[355].value <= threshold=8.05103588104248
node_28: feature_name=hsa04668 feature_id[333].value > threshold=5.614926338195801
Class: positive genes

Rules_759 passed counts:1
node_0: feature_name=GO:0042113 feature_id[0].value <= threshold=13.408552169799805
node_1: feature_name=GO:0007568 feature_id[534].value <= threshold=5.0313897132873535
node_2: feature_name=GO:0002705 feature_id[541].value <= threshold=3.200145721435547
node_3: feature_name=GO:1901525 feature_id[576].value <= threshold=0.4466460347175598
node_4: feature_name=GO:0048539 feature_id[319].value <= threshold=3.0399646759033203

node_5: feature_name=GO:0001910 feature_id[385].value <= threshold=3.7437864542007446
node_6: feature_name=GO:0043200 feature_id[706].value <= threshold=9.307284355163574
node_7: feature_name=GO:0001773 feature_id[308].value <= threshold=4.016931533813477
node_8: feature_name=GO:0090116 feature_id[97].value <= threshold=7.99645471572876
node_9: feature_name=GO:0019814 feature_id[189].value <= threshold=4.525782108306885
node_10: feature_name=GO:1902583 feature_id[215].value <= threshold=17.60310649871826
node_11: feature_name=GO:0045429 feature_id[737].value <= threshold=1.6133361458778381
node_12: feature_name=GO:0003720 feature_id[228].value <= threshold=5.519326210021973
node_13: feature_name=GO:0046006 feature_id[364].value <= threshold=4.9985432624816895
node_14: feature_name=GO:0070424 feature_id[254].value <= threshold=6.233297109603882
node_15: feature_name=GO:0009892 feature_id[320].value <= threshold=59.99180793762207
node_16: feature_name=GO:0007064 feature_id[527].value <= threshold=11.57377815246582
node_17: feature_name=GO:0005575 feature_id[17].value <= threshold=15.417460918426514
node_18: feature_name=GO:0043368 feature_id[31].value <= threshold=4.059496641159058
node_19: feature_name=GO:0005164 feature_id[163].value <= threshold=5.793607711791992
node_20: feature_name=GO:0042130 feature_id[16].value <= threshold=5.073179721832275
node_21: feature_name=GO:0010216 feature_id[282].value <= threshold=4.834584474563599
node_22: feature_name=GO:0009628 feature_id[553].value <= threshold=29.314892768859863
node_23: feature_name=GO:0045628 feature_id[749].value <= threshold=3.1001367568969727
node_24: feature_name=GO:0042288 feature_id[100].value <= threshold=5.174932956695557
node_25: feature_name=GO:0002329 feature_id[406].value <= threshold=2.348930835723877
node_26: feature_name=GO:0051246 feature_id[642].value <= threshold=0.6498909294605255
node_27: feature_name=GO:0042162 feature_id[355].value <= threshold=8.05103588104248
node_28: feature_name=hsa04668 feature_id[333].value <= threshold=5.614926338195801
node_29: feature_name=GO:0032200 feature_id[613].value > threshold=28.735913276672363
Class: positive genes

Rules_760 passed counts:1
node_0: feature_name=GO:0042113 feature_id[0].value <= threshold=13.408552169799805
node_1: feature_name=GO:0007568 feature_id[534].value <= threshold=5.0313897132873535
node_2: feature_name=GO:0002705 feature_id[541].value <= threshold=3.200145721435547
node_3: feature_name=GO:1901525 feature_id[576].value <= threshold=0.4466460347175598
node_4: feature_name=GO:0048539 feature_id[319].value <= threshold=3.0399646759033203
node_5: feature_name=GO:0001910 feature_id[385].value <= threshold=3.7437864542007446
node_6: feature_name=GO:0043200 feature_id[706].value <= threshold=9.307284355163574

node_7: feature_name=GO:0001773 feature_id[308].value <= threshold=4.016931533813477
node_8: feature_name=GO:0090116 feature_id[97].value <= threshold=7.99645471572876
node_9: feature_name=GO:0019814 feature_id[189].value <= threshold=4.525782108306885
node_10: feature_name=GO:1902583 feature_id[215].value <= threshold=17.60310649871826
node_11: feature_name=GO:0045429 feature_id[737].value <= threshold=1.6133361458778381
node_12: feature_name=GO:0003720 feature_id[228].value <= threshold=5.519326210021973
node_13: feature_name=GO:0046006 feature_id[364].value <= threshold=4.9985432624816895
node_14: feature_name=GO:0070424 feature_id[254].value <= threshold=6.233297109603882
node_15: feature_name=GO:0009892 feature_id[320].value <= threshold=59.99180793762207
node_16: feature_name=GO:0007064 feature_id[527].value <= threshold=11.57377815246582
node_17: feature_name=GO:0005575 feature_id[17].value <= threshold=15.417460918426514
node_18: feature_name=GO:0043368 feature_id[31].value <= threshold=4.059496641159058
node_19: feature_name=GO:0005164 feature_id[163].value <= threshold=5.793607711791992
node_20: feature_name=GO:0042130 feature_id[16].value <= threshold=5.073179721832275
node_21: feature_name=GO:0010216 feature_id[282].value <= threshold=4.834584474563599
node_22: feature_name=GO:0009628 feature_id[553].value <= threshold=29.314892768859863
node_23: feature_name=GO:0045628 feature_id[749].value <= threshold=3.1001367568969727
node_24: feature_name=GO:0042288 feature_id[100].value <= threshold=5.174932956695557
node_25: feature_name=GO:0002329 feature_id[406].value <= threshold=2.348930835723877
node_26: feature_name=GO:0051246 feature_id[642].value <= threshold=0.6498909294605255
node_27: feature_name=GO:0042162 feature_id[355].value <= threshold=8.05103588104248
node_28: feature_name=hsa04668 feature_id[333].value <= threshold=5.614926338195801
node_29: feature_name=GO:0032200 feature_id[613].value <= threshold=28.735913276672363
node_30: feature_name=hsa04662 feature_id[334].value > threshold=9.588344097137451
node_188: feature_name=GO:0046427 feature_id[766].value > threshold=0.0169341042637825
Class: positive genes

Rules_761 passed counts:1
node_0: feature_name=GO:0042113 feature_id[0].value <= threshold=13.408552169799805
node_1: feature_name=GO:0007568 feature_id[534].value <= threshold=5.0313897132873535
node_2: feature_name=GO:0002705 feature_id[541].value <= threshold=3.200145721435547
node_3: feature_name=GO:1901525 feature_id[576].value <= threshold=0.4466460347175598
node_4: feature_name=GO:0048539 feature_id[319].value <= threshold=3.0399646759033203
node_5: feature_name=GO:0001910 feature_id[385].value <= threshold=3.7437864542007446
node_6: feature_name=GO:0043200 feature_id[706].value <= threshold=9.307284355163574

node_7: feature_name=GO:0001773 feature_id[308].value <= threshold=4.016931533813477
node_8: feature_name=GO:0090116 feature_id[97].value <= threshold=7.99645471572876
node_9: feature_name=GO:0019814 feature_id[189].value <= threshold=4.525782108306885
node_10: feature_name=GO:1902583 feature_id[215].value <= threshold=17.60310649871826
node_11: feature_name=GO:0045429 feature_id[737].value <= threshold=1.6133361458778381
node_12: feature_name=GO:0003720 feature_id[228].value <= threshold=5.519326210021973
node_13: feature_name=GO:0046006 feature_id[364].value <= threshold=4.9985432624816895
node_14: feature_name=GO:0070424 feature_id[254].value <= threshold=6.233297109603882
node_15: feature_name=GO:0009892 feature_id[320].value <= threshold=59.99180793762207
node_16: feature_name=GO:0007064 feature_id[527].value <= threshold=11.57377815246582
node_17: feature_name=GO:0005575 feature_id[17].value <= threshold=15.417460918426514
node_18: feature_name=GO:0043368 feature_id[31].value <= threshold=4.059496641159058
node_19: feature_name=GO:0005164 feature_id[163].value <= threshold=5.793607711791992
node_20: feature_name=GO:0042130 feature_id[16].value <= threshold=5.073179721832275
node_21: feature_name=GO:0010216 feature_id[282].value <= threshold=4.834584474563599
node_22: feature_name=GO:0009628 feature_id[553].value <= threshold=29.314892768859863
node_23: feature_name=GO:0045628 feature_id[749].value <= threshold=3.1001367568969727
node_24: feature_name=GO:0042288 feature_id[100].value <= threshold=5.174932956695557
node_25: feature_name=GO:0002329 feature_id[406].value <= threshold=2.348930835723877
node_26: feature_name=GO:0051246 feature_id[642].value <= threshold=0.6498909294605255
node_27: feature_name=GO:0042162 feature_id[355].value <= threshold=8.05103588104248
node_28: feature_name=hsa04668 feature_id[333].value <= threshold=5.614926338195801
node_29: feature_name=GO:0032200 feature_id[613].value <= threshold=28.735913276672363
node_30: feature_name=hsa04662 feature_id[334].value > threshold=9.588344097137451
node_188: feature_name=GO:0046427 feature_id[766].value <= threshold=0.0169341042637825
Class: negative genes

Rules_762 passed counts:1
node_0: feature_name=GO:0042113 feature_id[0].value <= threshold=13.408552169799805
node_1: feature_name=GO:0007568 feature_id[534].value <= threshold=5.0313897132873535
node_2: feature_name=GO:0002705 feature_id[541].value <= threshold=3.200145721435547
node_3: feature_name=GO:1901525 feature_id[576].value <= threshold=0.4466460347175598
node_4: feature_name=GO:0048539 feature_id[319].value <= threshold=3.0399646759033203
node_5: feature_name=GO:0001910 feature_id[385].value <= threshold=3.7437864542007446
node_6: feature_name=GO:0043200 feature_id[706].value <= threshold=9.307284355163574

node_7: feature_name=GO:0001773 feature_id[308].value <= threshold=4.016931533813477
node_8: feature_name=GO:0090116 feature_id[97].value <= threshold=7.99645471572876
node_9: feature_name=GO:0019814 feature_id[189].value <= threshold=4.525782108306885
node_10: feature_name=GO:1902583 feature_id[215].value <= threshold=17.60310649871826
node_11: feature_name=GO:0045429 feature_id[737].value <= threshold=1.6133361458778381
node_12: feature_name=GO:0003720 feature_id[228].value <= threshold=5.519326210021973
node_13: feature_name=GO:0046006 feature_id[364].value <= threshold=4.9985432624816895
node_14: feature_name=GO:0070424 feature_id[254].value <= threshold=6.233297109603882
node_15: feature_name=GO:0009892 feature_id[320].value <= threshold=59.99180793762207
node_16: feature_name=GO:0007064 feature_id[527].value <= threshold=11.57377815246582
node_17: feature_name=GO:0005575 feature_id[17].value <= threshold=15.417460918426514
node_18: feature_name=GO:0043368 feature_id[31].value <= threshold=4.059496641159058
node_19: feature_name=GO:0005164 feature_id[163].value <= threshold=5.793607711791992
node_20: feature_name=GO:0042130 feature_id[16].value <= threshold=5.073179721832275
node_21: feature_name=GO:0010216 feature_id[282].value <= threshold=4.834584474563599
node_22: feature_name=GO:0009628 feature_id[553].value <= threshold=29.314892768859863
node_23: feature_name=GO:0045628 feature_id[749].value <= threshold=3.1001367568969727
node_24: feature_name=GO:0042288 feature_id[100].value <= threshold=5.174932956695557
node_25: feature_name=GO:0002329 feature_id[406].value <= threshold=2.348930835723877
node_26: feature_name=GO:0051246 feature_id[642].value <= threshold=0.6498909294605255
node_27: feature_name=GO:0042162 feature_id[355].value <= threshold=8.05103588104248
node_28: feature_name=hsa04668 feature_id[333].value <= threshold=5.614926338195801
node_29: feature_name=GO:0032200 feature_id[613].value <= threshold=28.735913276672363
node_30: feature_name=hsa04662 feature_id[334].value <= threshold=9.588344097137451
node_31: feature_name=GO:1901992 feature_id[105].value > threshold=5.291427850723267
node_185: feature_name=GO:0033044 feature_id[383].value > threshold=8.054455757141113
Class: negative genes

Rules_763 passed counts:1
node_0: feature_name=GO:0042113 feature_id[0].value <= threshold=13.408552169799805
node_1: feature_name=GO:0007568 feature_id[534].value <= threshold=5.0313897132873535
node_2: feature_name=GO:0002705 feature_id[541].value <= threshold=3.200145721435547
node_3: feature_name=GO:1901525 feature_id[576].value <= threshold=0.4466460347175598
node_4: feature_name=GO:0048539 feature_id[319].value <= threshold=3.0399646759033203
node_5: feature_name=GO:0001910 feature_id[385].value <= threshold=3.7437864542007446

node_6: feature_name=GO:0043200 feature_id[706].value <= threshold=9.307284355163574
node_7: feature_name=GO:0001773 feature_id[308].value <= threshold=4.016931533813477
node_8: feature_name=GO:0090116 feature_id[97].value <= threshold=7.99645471572876
node_9: feature_name=GO:0019814 feature_id[189].value <= threshold=4.525782108306885
node_10: feature_name=GO:1902583 feature_id[215].value <= threshold=17.60310649871826
node_11: feature_name=GO:0045429 feature_id[737].value <= threshold=1.6133361458778381
node_12: feature_name=GO:0003720 feature_id[228].value <= threshold=5.519326210021973
node_13: feature_name=GO:0046006 feature_id[364].value <= threshold=4.9985432624816895
node_14: feature_name=GO:0070424 feature_id[254].value <= threshold=6.233297109603882
node_15: feature_name=GO:0009892 feature_id[320].value <= threshold=59.99180793762207
node_16: feature_name=GO:0007064 feature_id[527].value <= threshold=11.57377815246582
node_17: feature_name=GO:0005575 feature_id[17].value <= threshold=15.417460918426514
node_18: feature_name=GO:0043368 feature_id[31].value <= threshold=4.059496641159058
node_19: feature_name=GO:0005164 feature_id[163].value <= threshold=5.793607711791992
node_20: feature_name=GO:0042130 feature_id[16].value <= threshold=5.073179721832275
node_21: feature_name=GO:0010216 feature_id[282].value <= threshold=4.834584474563599
node_22: feature_name=GO:0009628 feature_id[553].value <= threshold=29.314892768859863
node_23: feature_name=GO:0045628 feature_id[749].value <= threshold=3.1001367568969727
node_24: feature_name=GO:0042288 feature_id[100].value <= threshold=5.174932956695557
node_25: feature_name=GO:0002329 feature_id[406].value <= threshold=2.348930835723877
node_26: feature_name=GO:0051246 feature_id[642].value <= threshold=0.6498909294605255
node_27: feature_name=GO:0042162 feature_id[355].value <= threshold=8.05103588104248
node_28: feature_name=hsa04668 feature_id[333].value <= threshold=5.614926338195801
node_29: feature_name=GO:0032200 feature_id[613].value <= threshold=28.735913276672363
node_30: feature_name=hsa04662 feature_id[334].value <= threshold=9.588344097137451
node_31: feature_name=GO:1901992 feature_id[105].value > threshold=5.291427850723267
node_185: feature_name=GO:0033044 feature_id[383].value <= threshold=8.054455757141113
Class: positive genes

Rules_764 passed counts:1
node_0: feature_name=GO:0042113 feature_id[0].value <= threshold=13.408552169799805
node_1: feature_name=GO:0007568 feature_id[534].value <= threshold=5.0313897132873535
node_2: feature_name=GO:0002705 feature_id[541].value <= threshold=3.200145721435547
node_3: feature_name=GO:1901525 feature_id[576].value <= threshold=0.4466460347175598
node_4: feature_name=GO:0048539 feature_id[319].value <= threshold=3.0399646759033203

node_5: feature_name=GO:0001910 feature_id[385].value <= threshold=3.7437864542007446
node_6: feature_name=GO:0043200 feature_id[706].value <= threshold=9.307284355163574
node_7: feature_name=GO:0001773 feature_id[308].value <= threshold=4.016931533813477
node_8: feature_name=GO:0090116 feature_id[97].value <= threshold=7.99645471572876
node_9: feature_name=GO:0019814 feature_id[189].value <= threshold=4.525782108306885
node_10: feature_name=GO:1902583 feature_id[215].value <= threshold=17.60310649871826
node_11: feature_name=GO:0045429 feature_id[737].value <= threshold=1.6133361458778381
node_12: feature_name=GO:0003720 feature_id[228].value <= threshold=5.519326210021973
node_13: feature_name=GO:0046006 feature_id[364].value <= threshold=4.9985432624816895
node_14: feature_name=GO:0070424 feature_id[254].value <= threshold=6.233297109603882
node_15: feature_name=GO:0009892 feature_id[320].value <= threshold=59.99180793762207
node_16: feature_name=GO:0007064 feature_id[527].value <= threshold=11.57377815246582
node_17: feature_name=GO:0005575 feature_id[17].value <= threshold=15.417460918426514
node_18: feature_name=GO:0043368 feature_id[31].value <= threshold=4.059496641159058
node_19: feature_name=GO:0005164 feature_id[163].value <= threshold=5.793607711791992
node_20: feature_name=GO:0042130 feature_id[16].value <= threshold=5.073179721832275
node_21: feature_name=GO:0010216 feature_id[282].value <= threshold=4.834584474563599
node_22: feature_name=GO:0009628 feature_id[553].value <= threshold=29.314892768859863
node_23: feature_name=GO:0045628 feature_id[749].value <= threshold=3.1001367568969727
node_24: feature_name=GO:0042288 feature_id[100].value <= threshold=5.174932956695557
node_25: feature_name=GO:0002329 feature_id[406].value <= threshold=2.348930835723877
node_26: feature_name=GO:0051246 feature_id[642].value <= threshold=0.6498909294605255
node_27: feature_name=GO:0042162 feature_id[355].value <= threshold=8.05103588104248
node_28: feature_name=hsa04668 feature_id[333].value <= threshold=5.614926338195801
node_29: feature_name=GO:0032200 feature_id[613].value <= threshold=28.735913276672363
node_30: feature_name=hsa04662 feature_id[334].value <= threshold=9.588344097137451
node_31: feature_name=GO:1901992 feature_id[105].value <= threshold=5.291427850723267
node_32: feature_name=GO:0072341 feature_id[144].value > threshold=6.01465106010437
node_180: feature_name=GO:0045621 feature_id[143].value <= threshold=0.2295876294374466
node_181: feature_name=GO:0002294 feature_id[419].value > threshold=0.146267332136631
Class: positive genes

Rules_765 passed counts:1
node_0: feature_name=GO:0042113 feature_id[0].value <= threshold=13.408552169799805
node_1: feature_name=GO:0007568 feature_id[534].value <= threshold=5.0313897132873535

node_2: feature_name=GO:0002705	feature_id[541].value <= threshold=3.200145721435547
node_3: feature_name=GO:1901525	feature_id[576].value <= threshold=0.4466460347175598
node_4: feature_name=GO:0048539	feature_id[319].value <= threshold=3.0399646759033203
node_5: feature_name=GO:0001910	feature_id[385].value <= threshold=3.7437864542007446
node_6: feature_name=GO:0043200	feature_id[706].value <= threshold=9.307284355163574
node_7: feature_name=GO:0001773	feature_id[308].value <= threshold=4.016931533813477
node_8: feature_name=GO:0090116	feature_id[97].value <= threshold=7.99645471572876
node_9: feature_name=GO:0019814	feature_id[189].value <= threshold=4.525782108306885
node_10: feature_name=GO:1902583	feature_id[215].value <= threshold=17.60310649871826
node_11: feature_name=GO:0045429	feature_id[737].value <= threshold=1.6133361458778381
node_12: feature_name=GO:0003720	feature_id[228].value <= threshold=5.519326210021973
node_13: feature_name=GO:0046006	feature_id[364].value <= threshold=4.9985432624816895
node_14: feature_name=GO:0070424	feature_id[254].value <= threshold=6.233297109603882
node_15: feature_name=GO:0009892	feature_id[320].value <= threshold=59.99180793762207
node_16: feature_name=GO:0007064	feature_id[527].value <= threshold=11.57377815246582
node_17: feature_name=GO:0005575	feature_id[17].value <= threshold=15.417460918426514
node_18: feature_name=GO:0043368	feature_id[31].value <= threshold=4.059496641159058
node_19: feature_name=GO:0005164	feature_id[163].value <= threshold=5.793607711791992
node_20: feature_name=GO:0042130	feature_id[16].value <= threshold=5.073179721832275
node_21: feature_name=GO:0010216	feature_id[282].value <= threshold=4.834584474563599
node_22: feature_name=GO:0009628	feature_id[553].value <= threshold=29.314892768859863
node_23: feature_name=GO:0045628	feature_id[749].value <= threshold=3.1001367568969727
node_24: feature_name=GO:0042288	feature_id[100].value <= threshold=5.174932956695557
node_25: feature_name=GO:0002329	feature_id[406].value <= threshold=2.348930835723877
node_26: feature_name=GO:0051246	feature_id[642].value <= threshold=0.6498909294605255
node_27: feature_name=GO:0042162	feature_id[355].value <= threshold=8.05103588104248
node_28: feature_name=hsa04668	feature_id[333].value <= threshold=5.614926338195801
node_29: feature_name=GO:0032200	feature_id[613].value <= threshold=28.735913276672363
node_30: feature_name=hsa04662	feature_id[334].value <= threshold=9.588344097137451
node_31: feature_name=GO:1901992	feature_id[105].value <= threshold=5.291427850723267
node_32: feature_name=GO:0072341	feature_id[144].value <= threshold=6.01465106010437
node_33: feature_name=GO:0047485	feature_id[121].value > threshold=6.657836437225342
node_177: feature_name=GO:0034097	feature_id[647].value <= threshold=0.17321054253261536

Class: positive genes

Rules_766	passed counts:1
node_0: feature_name=GO:0042113	feature_id[0].value <= threshold=13.408552169799805
node_1: feature_name=GO:0007568	feature_id[534].value <= threshold=5.0313897132873535
node_2: feature_name=GO:0002705	feature_id[541].value <= threshold=3.200145721435547
node_3: feature_name=GO:1901525	feature_id[576].value <= threshold=0.4466460347175598
node_4: feature_name=GO:0048539	feature_id[319].value <= threshold=3.0399646759033203
node_5: feature_name=GO:0001910	feature_id[385].value <= threshold=3.7437864542007446
node_6: feature_name=GO:0043200	feature_id[706].value <= threshold=9.307284355163574
node_7: feature_name=GO:0001773	feature_id[308].value <= threshold=4.016931533813477
node_8: feature_name=GO:0090116	feature_id[97].value <= threshold=7.99645471572876
node_9: feature_name=GO:0019814	feature_id[189].value <= threshold=4.525782108306885
node_10: feature_name=GO:1902583	feature_id[215].value <= threshold=17.60310649871826
node_11: feature_name=GO:0045429	feature_id[737].value <= threshold=1.6133361458778381
node_12: feature_name=GO:0003720	feature_id[228].value <= threshold=5.519326210021973
node_13: feature_name=GO:0046006	feature_id[364].value <= threshold=4.9985432624816895
node_14: feature_name=GO:0070424	feature_id[254].value <= threshold=6.233297109603882
node_15: feature_name=GO:0009892	feature_id[320].value <= threshold=59.99180793762207
node_16: feature_name=GO:0007064	feature_id[527].value <= threshold=11.57377815246582
node_17: feature_name=GO:0005575	feature_id[17].value <= threshold=15.417460918426514
node_18: feature_name=GO:0043368	feature_id[31].value <= threshold=4.059496641159058
node_19: feature_name=GO:0005164	feature_id[163].value <= threshold=5.793607711791992
node_20: feature_name=GO:0042130	feature_id[16].value <= threshold=5.073179721832275
node_21: feature_name=GO:0010216	feature_id[282].value <= threshold=4.834584474563599
node_22: feature_name=GO:0009628	feature_id[553].value <= threshold=29.314892768859863
node_23: feature_name=GO:0045628	feature_id[749].value <= threshold=3.1001367568969727
node_24: feature_name=GO:0042288	feature_id[100].value <= threshold=5.174932956695557
node_25: feature_name=GO:0002329	feature_id[406].value <= threshold=2.348930835723877
node_26: feature_name=GO:0051246	feature_id[642].value <= threshold=0.6498909294605255
node_27: feature_name=GO:0042162	feature_id[355].value <= threshold=8.05103588104248
node_28: feature_name=hsa04668	feature_id[333].value <= threshold=5.614926338195801
node_29: feature_name=GO:0032200	feature_id[613].value <= threshold=28.735913276672363
node_30: feature_name=hsa04662	feature_id[334].value <= threshold=9.588344097137451
node_31: feature_name=GO:1901992	feature_id[105].value <= threshold=5.291427850723267
node_32: feature_name=GO:0072341	feature_id[144].value <= threshold=6.01465106010437
node_33: feature_name=GO:0047485	feature_id[121].value <= threshold=6.657836437225342

node_34: feature_name=GO:0090594 feature_id[738].value > threshold=1.8673912286758423
node_174: feature_name=GO:0010950 feature_id[567].value > threshold=0.11882948130369186
Class: positive genes

Rules_767 passed counts:1
node_0: feature_name=GO:0042113 feature_id[0].value <= threshold=13.408552169799805
node_1: feature_name=GO:0007568 feature_id[534].value <= threshold=5.0313897132873535
node_2: feature_name=GO:0002705 feature_id[541].value <= threshold=3.200145721435547
node_3: feature_name=GO:1901525 feature_id[576].value <= threshold=0.4466460347175598
node_4: feature_name=GO:0048539 feature_id[319].value <= threshold=3.0399646759033203
node_5: feature_name=GO:0001910 feature_id[385].value <= threshold=3.7437864542007446
node_6: feature_name=GO:0043200 feature_id[706].value <= threshold=9.307284355163574
node_7: feature_name=GO:0001773 feature_id[308].value <= threshold=4.016931533813477
node_8: feature_name=GO:0090116 feature_id[97].value <= threshold=7.99645471572876
node_9: feature_name=GO:0019814 feature_id[189].value <= threshold=4.525782108306885
node_10: feature_name=GO:1902583 feature_id[215].value <= threshold=17.60310649871826
node_11: feature_name=GO:0045429 feature_id[737].value <= threshold=1.6133361458778381
node_12: feature_name=GO:0003720 feature_id[228].value <= threshold=5.519326210021973
node_13: feature_name=GO:0046006 feature_id[364].value <= threshold=4.9985432624816895
node_14: feature_name=GO:0070424 feature_id[254].value <= threshold=6.233297109603882
node_15: feature_name=GO:0009892 feature_id[320].value <= threshold=59.99180793762207
node_16: feature_name=GO:0007064 feature_id[527].value <= threshold=11.57377815246582
node_17: feature_name=GO:0005575 feature_id[17].value <= threshold=15.417460918426514
node_18: feature_name=GO:0043368 feature_id[31].value <= threshold=4.059496641159058
node_19: feature_name=GO:0005164 feature_id[163].value <= threshold=5.793607711791992
node_20: feature_name=GO:0042130 feature_id[16].value <= threshold=5.073179721832275
node_21: feature_name=GO:0010216 feature_id[282].value <= threshold=4.834584474563599
node_22: feature_name=GO:0009628 feature_id[553].value <= threshold=29.314892768859863
node_23: feature_name=GO:0045628 feature_id[749].value <= threshold=3.1001367568969727
node_24: feature_name=GO:0042288 feature_id[100].value <= threshold=5.174932956695557
node_25: feature_name=GO:0002329 feature_id[406].value <= threshold=2.348930835723877
node_26: feature_name=GO:0051246 feature_id[642].value <= threshold=0.6498909294605255
node_27: feature_name=GO:0042162 feature_id[355].value <= threshold=8.05103588104248
node_28: feature_name=hsa04668 feature_id[333].value <= threshold=5.614926338195801
node_29: feature_name=GO:0032200 feature_id[613].value <= threshold=28.735913276672363

node_30: feature_name=hsa04662	feature_id[334].value <= threshold=9.588344097137451
node_31: feature_name=GO:1901992	feature_id[105].value <= threshold=5.291427850723267
node_32: feature_name=GO:0072341	feature_id[144].value <= threshold=6.01465106010437
node_33: feature_name=GO:0047485	feature_id[121].value <= threshold=6.657836437225342
node_34: feature_name=GO:0090594	feature_id[738].value <= threshold=1.8673912286758423
node_35: feature_name=GO:0043525	feature_id[524].value > threshold=2.881397008895874
node_171: feature_name=GO:0007126	feature_id[520].value > threshold=0.3579426407814026
Class: positive genes	

Rules_768	passed counts:1
node_0: feature_name=GO:0042113	feature_id[0].value <= threshold=13.408552169799805
node_1: feature_name=GO:0007568	feature_id[534].value <= threshold=5.0313897132873535
node_2: feature_name=GO:0002705	feature_id[541].value <= threshold=3.200145721435547
node_3: feature_name=GO:1901525	feature_id[576].value <= threshold=0.4466460347175598
node_4: feature_name=GO:0048539	feature_id[319].value <= threshold=3.0399646759033203
node_5: feature_name=GO:0001910	feature_id[385].value <= threshold=3.7437864542007446
node_6: feature_name=GO:0043200	feature_id[706].value <= threshold=9.307284355163574
node_7: feature_name=GO:0001773	feature_id[308].value <= threshold=4.016931533813477
node_8: feature_name=GO:0090116	feature_id[97].value <= threshold=7.99645471572876
node_9: feature_name=GO:0019814	feature_id[189].value <= threshold=4.525782108306885
node_10: feature_name=GO:1902583	feature_id[215].value <= threshold=17.60310649871826
node_11: feature_name=GO:0045429	feature_id[737].value <= threshold=1.6133361458778381
node_12: feature_name=GO:0003720	feature_id[228].value <= threshold=5.519326210021973
node_13: feature_name=GO:0046006	feature_id[364].value <= threshold=4.9985432624816895
node_14: feature_name=GO:0070424	feature_id[254].value <= threshold=6.233297109603882
node_15: feature_name=GO:0009892	feature_id[320].value <= threshold=59.99180793762207
node_16: feature_name=GO:0007064	feature_id[527].value <= threshold=11.57377815246582
node_17: feature_name=GO:0005575	feature_id[17].value <= threshold=15.417460918426514
node_18: feature_name=GO:0043368	feature_id[31].value <= threshold=4.059496641159058
node_19: feature_name=GO:0005164	feature_id[163].value <= threshold=5.793607711791992
node_20: feature_name=GO:0042130	feature_id[16].value <= threshold=5.073179721832275
node_21: feature_name=GO:0010216	feature_id[282].value <= threshold=4.834584474563599
node_22: feature_name=GO:0009628	feature_id[553].value <= threshold=29.314892768859863
node_23: feature_name=GO:0045628	feature_id[749].value <= threshold=3.1001367568969727
node_24: feature_name=GO:0042288	feature_id[100].value <= threshold=5.174932956695557

node_25: feature_name=GO:0002329	feature_id[406].value <= threshold=2.348930835723877
node_26: feature_name=GO:0051246	feature_id[642].value <= threshold=0.6498909294605255
node_27: feature_name=GO:0042162	feature_id[355].value <= threshold=8.05103588104248
node_28: feature_name=hsa04668	feature_id[333].value <= threshold=5.614926338195801
node_29: feature_name=GO:0032200	feature_id[613].value <= threshold=28.735913276672363
node_30: feature_name=hsa04662	feature_id[334].value <= threshold=9.588344097137451
node_31: feature_name=GO:1901992	feature_id[105].value <= threshold=5.291427850723267
node_32: feature_name=GO:0072341	feature_id[144].value <= threshold=6.01465106010437
node_33: feature_name=GO:0047485	feature_id[121].value <= threshold=6.657836437225342
node_34: feature_name=GO:0090594	feature_id[738].value <= threshold=1.8673912286758423
node_35: feature_name=GO:0043525	feature_id[524].value <= threshold=2.881397008895874
node_36: feature_name=GO:0032504	feature_id[244].value > threshold=17.22207546234131
node_168: feature_name=GO:0044446	feature_id[184].value > threshold=2.6137850284576416
Class: positive genes	

Rules_769	passed counts:1
node_0: feature_name=GO:0042113	feature_id[0].value <= threshold=13.408552169799805
node_1: feature_name=GO:0007568	feature_id[534].value <= threshold=5.0313897132873535
node_2: feature_name=GO:0002705	feature_id[541].value <= threshold=3.200145721435547
node_3: feature_name=GO:1901525	feature_id[576].value <= threshold=0.4466460347175598
node_4: feature_name=GO:0048539	feature_id[319].value <= threshold=3.0399646759033203
node_5: feature_name=GO:0001910	feature_id[385].value <= threshold=3.7437864542007446
node_6: feature_name=GO:0043200	feature_id[706].value <= threshold=9.307284355163574
node_7: feature_name=GO:0001773	feature_id[308].value <= threshold=4.016931533813477
node_8: feature_name=GO:0090116	feature_id[97].value <= threshold=7.99645471572876
node_9: feature_name=GO:0019814	feature_id[189].value <= threshold=4.525782108306885
node_10: feature_name=GO:1902583	feature_id[215].value <= threshold=17.60310649871826
node_11: feature_name=GO:0045429	feature_id[737].value <= threshold=1.6133361458778381
node_12: feature_name=GO:0003720	feature_id[228].value <= threshold=5.519326210021973
node_13: feature_name=GO:0046006	feature_id[364].value <= threshold=4.9985432624816895
node_14: feature_name=GO:0070424	feature_id[254].value <= threshold=6.233297109603882
node_15: feature_name=GO:0009892	feature_id[320].value <= threshold=59.99180793762207
node_16: feature_name=GO:0007064	feature_id[527].value <= threshold=11.57377815246582
node_17: feature_name=GO:0005575	feature_id[17].value <= threshold=15.417460918426514
node_18: feature_name=GO:0043368	feature_id[31].value <= threshold=4.059496641159058

node_19: feature_name=GO:0005164 feature_id[163].value <= threshold=5.793607711791992
node_20: feature_name=GO:0042130 feature_id[16].value <= threshold=5.073179721832275
node_21: feature_name=GO:0010216 feature_id[282].value <= threshold=4.834584474563599
node_22: feature_name=GO:0009628 feature_id[553].value <= threshold=29.314892768859863
node_23: feature_name=GO:0045628 feature_id[749].value <= threshold=3.1001367568969727
node_24: feature_name=GO:0042288 feature_id[100].value <= threshold=5.174932956695557
node_25: feature_name=GO:0002329 feature_id[406].value <= threshold=2.348930835723877
node_26: feature_name=GO:0051246 feature_id[642].value <= threshold=0.6498909294605255
node_27: feature_name=GO:0042162 feature_id[355].value <= threshold=8.05103588104248
node_28: feature_name=hsa04668 feature_id[333].value <= threshold=5.614926338195801
node_29: feature_name=GO:0032200 feature_id[613].value <= threshold=28.735913276672363
node_30: feature_name=hsa04662 feature_id[334].value <= threshold=9.588344097137451
node_31: feature_name=GO:1901992 feature_id[105].value <= threshold=5.291427850723267
node_32: feature_name=GO:0072341 feature_id[144].value <= threshold=6.01465106010437
node_33: feature_name=GO:0047485 feature_id[121].value <= threshold=6.657836437225342
node_34: feature_name=GO:0090594 feature_id[738].value <= threshold=1.8673912286758423
node_35: feature_name=GO:0043525 feature_id[524].value <= threshold=2.881397008895874
node_36: feature_name=GO:0032504 feature_id[244].value <= threshold=17.22207546234131
node_37: feature_name=GO:0051348 feature_id[126].value > threshold=5.933559417724609
node_165: feature_name=GO:0032204 feature_id[747].value > threshold=2.1291196420788765
Class: positive genes

Rules_770 passed counts:1
node_0: feature_name=GO:0042113 feature_id[0].value <= threshold=13.408552169799805
node_1: feature_name=GO:0007568 feature_id[534].value <= threshold=5.0313897132873535
node_2: feature_name=GO:0002705 feature_id[541].value <= threshold=3.200145721435547
node_3: feature_name=GO:1901525 feature_id[576].value <= threshold=0.4466460347175598
node_4: feature_name=GO:0048539 feature_id[319].value <= threshold=3.0399646759033203
node_5: feature_name=GO:0001910 feature_id[385].value <= threshold=3.7437864542007446
node_6: feature_name=GO:0043200 feature_id[706].value <= threshold=9.307284355163574
node_7: feature_name=GO:0001773 feature_id[308].value <= threshold=4.016931533813477
node_8: feature_name=GO:0090116 feature_id[97].value <= threshold=7.99645471572876
node_9: feature_name=GO:0019814 feature_id[189].value <= threshold=4.525782108306885
node_10: feature_name=GO:1902583 feature_id[215].value <= threshold=17.60310649871826
node_11: feature_name=GO:0045429 feature_id[737].value <= threshold=1.6133361458778381

node_12: feature_name=GO:0003720 feature_id[228].value <= threshold=5.519326210021973
node_13: feature_name=GO:0046006 feature_id[364].value <= threshold=4.9985432624816895
node_14: feature_name=GO:0070424 feature_id[254].value <= threshold=6.233297109603882
node_15: feature_name=GO:0009892 feature_id[320].value <= threshold=59.99180793762207
node_16: feature_name=GO:0007064 feature_id[527].value <= threshold=11.57377815246582
node_17: feature_name=GO:0005575 feature_id[17].value <= threshold=15.417460918426514
node_18: feature_name=GO:0043368 feature_id[31].value <= threshold=4.059496641159058
node_19: feature_name=GO:0005164 feature_id[163].value <= threshold=5.793607711791992
node_20: feature_name=GO:0042130 feature_id[16].value <= threshold=5.073179721832275
node_21: feature_name=GO:0010216 feature_id[282].value <= threshold=4.834584474563599
node_22: feature_name=GO:0009628 feature_id[553].value <= threshold=29.314892768859863
node_23: feature_name=GO:0045628 feature_id[749].value <= threshold=3.1001367568969727
node_24: feature_name=GO:0042288 feature_id[100].value <= threshold=5.174932956695557
node_25: feature_name=GO:0002329 feature_id[406].value <= threshold=2.348930835723877
node_26: feature_name=GO:0051246 feature_id[642].value <= threshold=0.6498909294605255
node_27: feature_name=GO:0042162 feature_id[355].value <= threshold=8.05103588104248
node_28: feature_name=hsa04668 feature_id[333].value <= threshold=5.614926338195801
node_29: feature_name=GO:0032200 feature_id[613].value <= threshold=28.735913276672363
node_30: feature_name=hsa04662 feature_id[334].value <= threshold=9.588344097137451
node_31: feature_name=GO:1901992 feature_id[105].value <= threshold=5.291427850723267
node_32: feature_name=GO:0072341 feature_id[144].value <= threshold=6.01465106010437
node_33: feature_name=GO:0047485 feature_id[121].value <= threshold=6.657836437225342
node_34: feature_name=GO:0090594 feature_id[738].value <= threshold=1.8673912286758423
node_35: feature_name=GO:0043525 feature_id[524].value <= threshold=2.881397008895874
node_36: feature_name=GO:0032504 feature_id[244].value <= threshold=17.22207546234131
node_37: feature_name=GO:0051348 feature_id[126].value <= threshold=5.933559417724609
node_38: feature_name=GO:0042522 feature_id[692].value <= threshold=1.78694087266922
node_39: feature_name=GO:0031100 feature_id[73].value > threshold=3.2312101125717163
node_157: feature_name=GO:0002376 feature_id[277].value > threshold=0.046988584101200104
node_159: feature_name=GO:0050794 feature_id[108].value > threshold=0.21684371432638727
Class: negative genes

Rules_771 passed counts:1
node_0: feature_name=GO:0042113 feature_id[0].value <= threshold=13.408552169799805
node_1: feature_name=GO:0007568 feature_id[534].value <= threshold=5.0313897132873535

node_2: feature_name=GO:0002705 feature_id[541].value <= threshold=3.200145721435547
node_3: feature_name=GO:1901525 feature_id[576].value <= threshold=0.4466460347175598
node_4: feature_name=GO:0048539 feature_id[319].value <= threshold=3.0399646759033203
node_5: feature_name=GO:0001910 feature_id[385].value <= threshold=3.7437864542007446
node_6: feature_name=GO:0043200 feature_id[706].value <= threshold=9.307284355163574
node_7: feature_name=GO:0001773 feature_id[308].value <= threshold=4.016931533813477
node_8: feature_name=GO:0090116 feature_id[97].value <= threshold=7.99645471572876
node_9: feature_name=GO:0019814 feature_id[189].value <= threshold=4.525782108306885
node_10: feature_name=GO:1902583 feature_id[215].value <= threshold=17.60310649871826
node_11: feature_name=GO:0045429 feature_id[737].value <= threshold=1.6133361458778381
node_12: feature_name=GO:0003720 feature_id[228].value <= threshold=5.519326210021973
node_13: feature_name=GO:0046006 feature_id[364].value <= threshold=4.9985432624816895
node_14: feature_name=GO:0070424 feature_id[254].value <= threshold=6.233297109603882
node_15: feature_name=GO:0009892 feature_id[320].value <= threshold=59.99180793762207
node_16: feature_name=GO:0007064 feature_id[527].value <= threshold=11.57377815246582
node_17: feature_name=GO:0005575 feature_id[17].value <= threshold=15.417460918426514
node_18: feature_name=GO:0043368 feature_id[31].value <= threshold=4.059496641159058
node_19: feature_name=GO:0005164 feature_id[163].value <= threshold=5.793607711791992
node_20: feature_name=GO:0042130 feature_id[16].value <= threshold=5.073179721832275
node_21: feature_name=GO:0010216 feature_id[282].value <= threshold=4.834584474563599
node_22: feature_name=GO:0009628 feature_id[553].value <= threshold=29.314892768859863
node_23: feature_name=GO:0045628 feature_id[749].value <= threshold=3.1001367568969727
node_24: feature_name=GO:0042288 feature_id[100].value <= threshold=5.174932956695557
node_25: feature_name=GO:0002329 feature_id[406].value <= threshold=2.348930835723877
node_26: feature_name=GO:0051246 feature_id[642].value <= threshold=0.6498909294605255
node_27: feature_name=GO:0042162 feature_id[355].value <= threshold=8.05103588104248
node_28: feature_name=hsa04668 feature_id[333].value <= threshold=5.614926338195801
node_29: feature_name=GO:0032200 feature_id[613].value <= threshold=28.735913276672363
node_30: feature_name=hsa04662 feature_id[334].value <= threshold=9.588344097137451
node_31: feature_name=GO:1901992 feature_id[105].value <= threshold=5.291427850723267
node_32: feature_name=GO:0072341 feature_id[144].value <= threshold=6.01465106010437
node_33: feature_name=GO:0047485 feature_id[121].value <= threshold=6.657836437225342
node_34: feature_name=GO:0090594 feature_id[738].value <= threshold=1.8673912286758423
node_35: feature_name=GO:0043525 feature_id[524].value <= threshold=2.881397008895874
node_36: feature_name=GO:0032504 feature_id[244].value <= threshold=17.22207546234131

node_37: feature_name=GO:0051348 feature_id[126].value <= threshold=5.933559417724609
node_38: feature_name=GO:0042522 feature_id[692].value <= threshold=1.78694087266922
node_39: feature_name=GO:0031100 feature_id[73].value <= threshold=3.2312101125717163
node_40: feature_name=GO:0042493 feature_id[149].value > threshold=19.771170616149902
node_154: feature_name=GO:0002252 feature_id[460].value > threshold=0.05236339755356312
Class: positive genes

Rules_772 passed counts:1
node_0: feature_name=GO:0042113 feature_id[0].value <= threshold=13.408552169799805
node_1: feature_name=GO:0007568 feature_id[534].value <= threshold=5.0313897132873535
node_2: feature_name=GO:0002705 feature_id[541].value <= threshold=3.200145721435547
node_3: feature_name=GO:1901525 feature_id[576].value <= threshold=0.4466460347175598
node_4: feature_name=GO:0048539 feature_id[319].value <= threshold=3.0399646759033203
node_5: feature_name=GO:0001910 feature_id[385].value <= threshold=3.7437864542007446
node_6: feature_name=GO:0043200 feature_id[706].value <= threshold=9.307284355163574
node_7: feature_name=GO:0001773 feature_id[308].value <= threshold=4.016931533813477
node_8: feature_name=GO:0090116 feature_id[97].value <= threshold=7.99645471572876
node_9: feature_name=GO:0019814 feature_id[189].value <= threshold=4.525782108306885
node_10: feature_name=GO:1902583 feature_id[215].value <= threshold=17.60310649871826
node_11: feature_name=GO:0045429 feature_id[737].value <= threshold=1.6133361458778381
node_12: feature_name=GO:0003720 feature_id[228].value <= threshold=5.519326210021973
node_13: feature_name=GO:0046006 feature_id[364].value <= threshold=4.9985432624816895
node_14: feature_name=GO:0070424 feature_id[254].value <= threshold=6.233297109603882
node_15: feature_name=GO:0009892 feature_id[320].value <= threshold=59.99180793762207
node_16: feature_name=GO:0007064 feature_id[527].value <= threshold=11.57377815246582
node_17: feature_name=GO:0005575 feature_id[17].value <= threshold=15.417460918426514
node_18: feature_name=GO:0043368 feature_id[31].value <= threshold=4.059496641159058
node_19: feature_name=GO:0005164 feature_id[163].value <= threshold=5.793607711791992
node_20: feature_name=GO:0042130 feature_id[16].value <= threshold=5.073179721832275
node_21: feature_name=GO:0010216 feature_id[282].value <= threshold=4.834584474563599
node_22: feature_name=GO:0009628 feature_id[553].value <= threshold=29.314892768859863
node_23: feature_name=GO:0045628 feature_id[749].value <= threshold=3.1001367568969727
node_24: feature_name=GO:0042288 feature_id[100].value <= threshold=5.174932956695557
node_25: feature_name=GO:0002329 feature_id[406].value <= threshold=2.348930835723877
node_26: feature_name=GO:0051246 feature_id[642].value <= threshold=0.6498909294605255

node_27: feature_name=GO:0042162 feature_id[355].value <= threshold=8.05103588104248
node_28: feature_name=hsa04668 feature_id[333].value <= threshold=5.614926338195801
node_29: feature_name=GO:0032200 feature_id[613].value <= threshold=28.735913276672363
node_30: feature_name=hsa04662 feature_id[334].value <= threshold=9.588344097137451
node_31: feature_name=GO:1901992 feature_id[105].value <= threshold=5.291427850723267
node_32: feature_name=GO:0072341 feature_id[144].value <= threshold=6.01465106010437
node_33: feature_name=GO:0047485 feature_id[121].value <= threshold=6.657836437225342
node_34: feature_name=GO:0090594 feature_id[738].value <= threshold=1.8673912286758423
node_35: feature_name=GO:0043525 feature_id[524].value <= threshold=2.881397008895874
node_36: feature_name=GO:0032504 feature_id[244].value <= threshold=17.22207546234131
node_37: feature_name=GO:0051348 feature_id[126].value <= threshold=5.933559417724609
node_38: feature_name=GO:0042522 feature_id[692].value <= threshold=1.78694087266922
node_39: feature_name=GO:0031100 feature_id[73].value <= threshold=3.2312101125717163
node_40: feature_name=GO:0042493 feature_id[149].value <= threshold=19.771170616149902
node_41: feature_name=GO:0001552 feature_id[366].value > threshold=2.1711617708206177
node_151: feature_name=GO:0002706 feature_id[565].value <= threshold=0.03241767734289169
Class: positive genes

Rules_773 passed counts:1
node_0: feature_name=GO:0042113 feature_id[0].value <= threshold=13.408552169799805
node_1: feature_name=GO:0007568 feature_id[534].value <= threshold=5.0313897132873535
node_2: feature_name=GO:0002705 feature_id[541].value <= threshold=3.200145721435547
node_3: feature_name=GO:1901525 feature_id[576].value <= threshold=0.4466460347175598
node_4: feature_name=GO:0048539 feature_id[319].value <= threshold=3.0399646759033203
node_5: feature_name=GO:0001910 feature_id[385].value <= threshold=3.7437864542007446
node_6: feature_name=GO:0043200 feature_id[706].value <= threshold=9.307284355163574
node_7: feature_name=GO:0001773 feature_id[308].value <= threshold=4.016931533813477
node_8: feature_name=GO:0090116 feature_id[97].value <= threshold=7.99645471572876
node_9: feature_name=GO:0019814 feature_id[189].value <= threshold=4.525782108306885
node_10: feature_name=GO:1902583 feature_id[215].value <= threshold=17.60310649871826
node_11: feature_name=GO:0045429 feature_id[737].value <= threshold=1.6133361458778381
node_12: feature_name=GO:0003720 feature_id[228].value <= threshold=5.519326210021973
node_13: feature_name=GO:0046006 feature_id[364].value <= threshold=4.9985432624816895
node_14: feature_name=GO:0070424 feature_id[254].value <= threshold=6.233297109603882
node_15: feature_name=GO:0009892 feature_id[320].value <= threshold=59.99180793762207

node_16: feature_name=GO:0007064 feature_id[527].value <= threshold=11.57377815246582
node_17: feature_name=GO:0005575 feature_id[17].value <= threshold=15.417460918426514
node_18: feature_name=GO:0043368 feature_id[31].value <= threshold=4.059496641159058
node_19: feature_name=GO:0005164 feature_id[163].value <= threshold=5.793607711791992
node_20: feature_name=GO:0042130 feature_id[16].value <= threshold=5.073179721832275
node_21: feature_name=GO:0010216 feature_id[282].value <= threshold=4.834584474563599
node_22: feature_name=GO:0009628 feature_id[553].value <= threshold=29.314892768859863
node_23: feature_name=GO:0045628 feature_id[749].value <= threshold=3.1001367568969727
node_24: feature_name=GO:0042288 feature_id[100].value <= threshold=5.174932956695557
node_25: feature_name=GO:0002329 feature_id[406].value <= threshold=2.348930835723877
node_26: feature_name=GO:0051246 feature_id[642].value <= threshold=0.6498909294605255
node_27: feature_name=GO:0042162 feature_id[355].value <= threshold=8.05103588104248
node_28: feature_name=hsa04668 feature_id[333].value <= threshold=5.614926338195801
node_29: feature_name=GO:0032200 feature_id[613].value <= threshold=28.735913276672363
node_30: feature_name=hsa04662 feature_id[334].value <= threshold=9.588344097137451
node_31: feature_name=GO:1901992 feature_id[105].value <= threshold=5.291427850723267
node_32: feature_name=GO:0072341 feature_id[144].value <= threshold=6.01465106010437
node_33: feature_name=GO:0047485 feature_id[121].value <= threshold=6.657836437225342
node_34: feature_name=GO:0090594 feature_id[738].value <= threshold=1.8673912286758423
node_35: feature_name=GO:0043525 feature_id[524].value <= threshold=2.881397008895874
node_36: feature_name=GO:0032504 feature_id[244].value <= threshold=17.22207546234131
node_37: feature_name=GO:0051348 feature_id[126].value <= threshold=5.933559417724609
node_38: feature_name=GO:0042522 feature_id[692].value <= threshold=1.78694087266922
node_39: feature_name=GO:0031100 feature_id[73].value <= threshold=3.2312101125717163
node_40: feature_name=GO:0042493 feature_id[149].value <= threshold=19.771170616149902
node_41: feature_name=GO:0001552 feature_id[366].value <= threshold=2.1711617708206177
node_42: feature_name=GO:0046483 feature_id[391].value > threshold=320.9667053222656
node_148: feature_name=GO:0060576 feature_id[204].value <= threshold=0.22821159660816193
Class: positive genes

Rules_774 passed counts:1
node_0: feature_name=GO:0042113 feature_id[0].value <= threshold=13.408552169799805
node_1: feature_name=GO:0007568 feature_id[534].value <= threshold=5.0313897132873535
node_2: feature_name=GO:0002705 feature_id[541].value <= threshold=3.200145721435547
node_3: feature_name=GO:1901525 feature_id[576].value <= threshold=0.4466460347175598

node_4: feature_name=GO:0048539	feature_id[319].value <= threshold=3.0399646759033203
node_5: feature_name=GO:0001910	feature_id[385].value <= threshold=3.7437864542007446
node_6: feature_name=GO:0043200	feature_id[706].value <= threshold=9.307284355163574
node_7: feature_name=GO:0001773	feature_id[308].value <= threshold=4.016931533813477
node_8: feature_name=GO:0090116	feature_id[97].value <= threshold=7.99645471572876
node_9: feature_name=GO:0019814	feature_id[189].value <= threshold=4.525782108306885
node_10: feature_name=GO:1902583	feature_id[215].value <= threshold=17.60310649871826
node_11: feature_name=GO:0045429	feature_id[737].value <= threshold=1.6133361458778381
node_12: feature_name=GO:0003720	feature_id[228].value <= threshold=5.519326210021973
node_13: feature_name=GO:0046006	feature_id[364].value <= threshold=4.9985432624816895
node_14: feature_name=GO:0070424	feature_id[254].value <= threshold=6.233297109603882
node_15: feature_name=GO:0009892	feature_id[320].value <= threshold=59.99180793762207
node_16: feature_name=GO:0007064	feature_id[527].value <= threshold=11.57377815246582
node_17: feature_name=GO:0005575	feature_id[17].value <= threshold=15.417460918426514
node_18: feature_name=GO:0043368	feature_id[31].value <= threshold=4.059496641159058
node_19: feature_name=GO:0005164	feature_id[163].value <= threshold=5.793607711791992
node_20: feature_name=GO:0042130	feature_id[16].value <= threshold=5.073179721832275
node_21: feature_name=GO:0010216	feature_id[282].value <= threshold=4.834584474563599
node_22: feature_name=GO:0009628	feature_id[553].value <= threshold=29.314892768859863
node_23: feature_name=GO:0045628	feature_id[749].value <= threshold=3.1001367568969727
node_24: feature_name=GO:0042288	feature_id[100].value <= threshold=5.174932956695557
node_25: feature_name=GO:0002329	feature_id[406].value <= threshold=2.348930835723877
node_26: feature_name=GO:0051246	feature_id[642].value <= threshold=0.6498909294605255
node_27: feature_name=GO:0042162	feature_id[355].value <= threshold=8.05103588104248
node_28: feature_name=hsa04668	feature_id[333].value <= threshold=5.614926338195801
node_29: feature_name=GO:0032200	feature_id[613].value <= threshold=28.735913276672363
node_30: feature_name=hsa04662	feature_id[334].value <= threshold=9.588344097137451
node_31: feature_name=GO:1901992	feature_id[105].value <= threshold=5.291427850723267
node_32: feature_name=GO:0072341	feature_id[144].value <= threshold=6.01465106010437
node_33: feature_name=GO:0047485	feature_id[121].value <= threshold=6.657836437225342
node_34: feature_name=GO:0090594	feature_id[738].value <= threshold=1.8673912286758423
node_35: feature_name=GO:0043525	feature_id[524].value <= threshold=2.881397008895874
node_36: feature_name=GO:0032504	feature_id[244].value <= threshold=17.22207546234131
node_37: feature_name=GO:0051348	feature_id[126].value <= threshold=5.933559417724609
node_38: feature_name=GO:0042522	feature_id[692].value <= threshold=1.78694087266922

node_39: feature_name=GO:0031100 feature_id[73].value <= threshold=3.2312101125717163
node_40: feature_name=GO:0042493 feature_id[149].value <= threshold=19.771170616149902
node_41: feature_name=GO:0001552 feature_id[366].value <= threshold=2.1711617708206177
node_42: feature_name=GO:0046483 feature_id[391].value <= threshold=320.9667053222656
node_43: feature_name=GO:0002439 feature_id[431].value > threshold=1.832722783088684
node_145: feature_name=GO:0000723 feature_id[372].value > threshold=1.608974039554596
Class: positive genes

Rules_775 passed counts:1
node_0: feature_name=GO:0042113 feature_id[0].value <= threshold=13.408552169799805
node_1: feature_name=GO:0007568 feature_id[534].value <= threshold=5.0313897132873535
node_2: feature_name=GO:0002705 feature_id[541].value <= threshold=3.200145721435547
node_3: feature_name=GO:1901525 feature_id[576].value <= threshold=0.4466460347175598
node_4: feature_name=GO:0048539 feature_id[319].value <= threshold=3.0399646759033203
node_5: feature_name=GO:0001910 feature_id[385].value <= threshold=3.7437864542007446
node_6: feature_name=GO:0043200 feature_id[706].value <= threshold=9.307284355163574
node_7: feature_name=GO:0001773 feature_id[308].value <= threshold=4.016931533813477
node_8: feature_name=GO:0090116 feature_id[97].value <= threshold=7.99645471572876
node_9: feature_name=GO:0019814 feature_id[189].value <= threshold=4.525782108306885
node_10: feature_name=GO:1902583 feature_id[215].value <= threshold=17.60310649871826
node_11: feature_name=GO:0045429 feature_id[737].value <= threshold=1.6133361458778381
node_12: feature_name=GO:0003720 feature_id[228].value <= threshold=5.519326210021973
node_13: feature_name=GO:0046006 feature_id[364].value <= threshold=4.9985432624816895
node_14: feature_name=GO:0070424 feature_id[254].value <= threshold=6.233297109603882
node_15: feature_name=GO:0009892 feature_id[320].value <= threshold=59.99180793762207
node_16: feature_name=GO:0007064 feature_id[527].value <= threshold=11.57377815246582
node_17: feature_name=GO:0005575 feature_id[17].value <= threshold=15.417460918426514
node_18: feature_name=GO:0043368 feature_id[31].value <= threshold=4.059496641159058
node_19: feature_name=GO:0005164 feature_id[163].value <= threshold=5.793607711791992
node_20: feature_name=GO:0042130 feature_id[16].value <= threshold=5.073179721832275
node_21: feature_name=GO:0010216 feature_id[282].value <= threshold=4.834584474563599
node_22: feature_name=GO:0009628 feature_id[553].value <= threshold=29.314892768859863
node_23: feature_name=GO:0045628 feature_id[749].value <= threshold=3.1001367568969727
node_24: feature_name=GO:0042288 feature_id[100].value <= threshold=5.174932956695557
node_25: feature_name=GO:0002329 feature_id[406].value <= threshold=2.348930835723877

node_26: feature_name=GO:0051246	feature_id[642].value <= threshold=0.6498909294605255
node_27: feature_name=GO:0042162	feature_id[355].value <= threshold=8.05103588104248
node_28: feature_name=hsa04668	feature_id[333].value <= threshold=5.614926338195801
node_29: feature_name=GO:0032200	feature_id[613].value <= threshold=28.735913276672363
node_30: feature_name=hsa04662	feature_id[334].value <= threshold=9.588344097137451
node_31: feature_name=GO:1901992	feature_id[105].value <= threshold=5.291427850723267
node_32: feature_name=GO:0072341	feature_id[144].value <= threshold=6.01465106010437
node_33: feature_name=GO:0047485	feature_id[121].value <= threshold=6.657836437225342
node_34: feature_name=GO:0090594	feature_id[738].value <= threshold=1.8673912286758423
node_35: feature_name=GO:0043525	feature_id[524].value <= threshold=2.881397008895874
node_36: feature_name=GO:0032504	feature_id[244].value <= threshold=17.22207546234131
node_37: feature_name=GO:0051348	feature_id[126].value <= threshold=5.933559417724609
node_38: feature_name=GO:0042522	feature_id[692].value <= threshold=1.78694087266922
node_39: feature_name=GO:0031100	feature_id[73].value <= threshold=3.2312101125717163
node_40: feature_name=GO:0042493	feature_id[149].value <= threshold=19.771170616149902
node_41: feature_name=GO:0001552	feature_id[366].value <= threshold=2.1711617708206177
node_42: feature_name=GO:0046483	feature_id[391].value <= threshold=320.9667053222656
node_43: feature_name=GO:0002439	feature_id[431].value <= threshold=1.832722783088684
node_44: feature_name=GO:0006555	feature_id[514].value <= threshold=5.321640968322754
node_45: feature_name=GO:0070424	feature_id[254].value > threshold=3.321003556251526
node_139: feature_name=GO:0042542	feature_id[689].value > threshold=2.582065224647522
Class: positive genes	

Rules_776	passed counts:1
node_0: feature_name=GO:0042113	feature_id[0].value <= threshold=13.408552169799805
node_1: feature_name=GO:0007568	feature_id[534].value <= threshold=5.0313897132873535
node_2: feature_name=GO:0002705	feature_id[541].value <= threshold=3.200145721435547
node_3: feature_name=GO:1901525	feature_id[576].value <= threshold=0.4466460347175598
node_4: feature_name=GO:0048539	feature_id[319].value <= threshold=3.0399646759033203
node_5: feature_name=GO:0001910	feature_id[385].value <= threshold=3.7437864542007446
node_6: feature_name=GO:0043200	feature_id[706].value <= threshold=9.307284355163574
node_7: feature_name=GO:0001773	feature_id[308].value <= threshold=4.016931533813477
node_8: feature_name=GO:0090116	feature_id[97].value <= threshold=7.99645471572876
node_9: feature_name=GO:0019814	feature_id[189].value <= threshold=4.525782108306885
node_10: feature_name=GO:1902583	feature_id[215].value <= threshold=17.60310649871826

node_11: feature_name=GO:0045429 feature_id[737].value <= threshold=1.6133361458778381
node_12: feature_name=GO:0003720 feature_id[228].value <= threshold=5.519326210021973
node_13: feature_name=GO:0046006 feature_id[364].value <= threshold=4.9985432624816895
node_14: feature_name=GO:0070424 feature_id[254].value <= threshold=6.233297109603882
node_15: feature_name=GO:0009892 feature_id[320].value <= threshold=59.99180793762207
node_16: feature_name=GO:0007064 feature_id[527].value <= threshold=11.57377815246582
node_17: feature_name=GO:0005575 feature_id[17].value <= threshold=15.417460918426514
node_18: feature_name=GO:0043368 feature_id[31].value <= threshold=4.059496641159058
node_19: feature_name=GO:0005164 feature_id[163].value <= threshold=5.793607711791992
node_20: feature_name=GO:0042130 feature_id[16].value <= threshold=5.073179721832275
node_21: feature_name=GO:0010216 feature_id[282].value <= threshold=4.834584474563599
node_22: feature_name=GO:0009628 feature_id[553].value <= threshold=29.314892768859863
node_23: feature_name=GO:0045628 feature_id[749].value <= threshold=3.1001367568969727
node_24: feature_name=GO:0042288 feature_id[100].value <= threshold=5.174932956695557
node_25: feature_name=GO:0002329 feature_id[406].value <= threshold=2.348930835723877
node_26: feature_name=GO:0051246 feature_id[642].value <= threshold=0.6498909294605255
node_27: feature_name=GO:0042162 feature_id[355].value <= threshold=8.05103588104248
node_28: feature_name=hsa04668 feature_id[333].value <= threshold=5.614926338195801
node_29: feature_name=GO:0032200 feature_id[613].value <= threshold=28.735913276672363
node_30: feature_name=hsa04662 feature_id[334].value <= threshold=9.588344097137451
node_31: feature_name=GO:1901992 feature_id[105].value <= threshold=5.291427850723267
node_32: feature_name=GO:0072341 feature_id[144].value <= threshold=6.01465106010437
node_33: feature_name=GO:0047485 feature_id[121].value <= threshold=6.657836437225342
node_34: feature_name=GO:0090594 feature_id[738].value <= threshold=1.8673912286758423
node_35: feature_name=GO:0043525 feature_id[524].value <= threshold=2.881397008895874
node_36: feature_name=GO:0032504 feature_id[244].value <= threshold=17.22207546234131
node_37: feature_name=GO:0051348 feature_id[126].value <= threshold=5.933559417724609
node_38: feature_name=GO:0042522 feature_id[692].value <= threshold=1.78694087266922
node_39: feature_name=GO:0031100 feature_id[73].value <= threshold=3.2312101125717163
node_40: feature_name=GO:0042493 feature_id[149].value <= threshold=19.771170616149902
node_41: feature_name=GO:0001552 feature_id[366].value <= threshold=2.1711617708206177
node_42: feature_name=GO:0046483 feature_id[391].value <= threshold=320.9667053222656
node_43: feature_name=GO:0002439 feature_id[431].value <= threshold=1.832722783088684
node_44: feature_name=GO:0006555 feature_id[514].value <= threshold=5.321640968322754
node_45: feature_name=GO:0070424 feature_id[254].value <= threshold=3.321003556251526

node_46: feature_name=hsa05144 feature_id[751].value > threshold=2.635499954223633
node_134: feature_name=GO:0010165 feature_id[330].value > threshold=0.49422845244407654
node_136: feature_name=GO:0010955 feature_id[2].value <= threshold=0.3874783217906952
Class: negative genes

Rules_777 passed counts:1
node_0: feature_name=GO:0042113 feature_id[0].value <= threshold=13.408552169799805
node_1: feature_name=GO:0007568 feature_id[534].value <= threshold=5.0313897132873535
node_2: feature_name=GO:0002705 feature_id[541].value <= threshold=3.200145721435547
node_3: feature_name=GO:1901525 feature_id[576].value <= threshold=0.4466460347175598
node_4: feature_name=GO:0048539 feature_id[319].value <= threshold=3.0399646759033203
node_5: feature_name=GO:0001910 feature_id[385].value <= threshold=3.7437864542007446
node_6: feature_name=GO:0043200 feature_id[706].value <= threshold=9.307284355163574
node_7: feature_name=GO:0001773 feature_id[308].value <= threshold=4.016931533813477
node_8: feature_name=GO:0090116 feature_id[97].value <= threshold=7.99645471572876
node_9: feature_name=GO:0019814 feature_id[189].value <= threshold=4.525782108306885
node_10: feature_name=GO:1902583 feature_id[215].value <= threshold=17.60310649871826
node_11: feature_name=GO:0045429 feature_id[737].value <= threshold=1.6133361458778381
node_12: feature_name=GO:0003720 feature_id[228].value <= threshold=5.519326210021973
node_13: feature_name=GO:0046006 feature_id[364].value <= threshold=4.9985432624816895
node_14: feature_name=GO:0070424 feature_id[254].value <= threshold=6.233297109603882
node_15: feature_name=GO:0009892 feature_id[320].value <= threshold=59.99180793762207
node_16: feature_name=GO:0007064 feature_id[527].value <= threshold=11.57377815246582
node_17: feature_name=GO:0005575 feature_id[17].value <= threshold=15.417460918426514
node_18: feature_name=GO:0043368 feature_id[31].value <= threshold=4.059496641159058
node_19: feature_name=GO:0005164 feature_id[163].value <= threshold=5.793607711791992
node_20: feature_name=GO:0042130 feature_id[16].value <= threshold=5.073179721832275
node_21: feature_name=GO:0010216 feature_id[282].value <= threshold=4.834584474563599
node_22: feature_name=GO:0009628 feature_id[553].value <= threshold=29.314892768859863
node_23: feature_name=GO:0045628 feature_id[749].value <= threshold=3.1001367568969727
node_24: feature_name=GO:0042288 feature_id[100].value <= threshold=5.174932956695557
node_25: feature_name=GO:0002329 feature_id[406].value <= threshold=2.348930835723877
node_26: feature_name=GO:0051246 feature_id[642].value <= threshold=0.6498909294605255
node_27: feature_name=GO:0042162 feature_id[355].value <= threshold=8.05103588104248
node_28: feature_name=hsa04668 feature_id[333].value <= threshold=5.614926338195801

node_29: feature_name=GO:0032200 feature_id[613].value <= threshold=28.735913276672363
node_30: feature_name=hsa04662 feature_id[334].value <= threshold=9.588344097137451
node_31: feature_name=GO:1901992 feature_id[105].value <= threshold=5.291427850723267
node_32: feature_name=GO:0072341 feature_id[144].value <= threshold=6.01465106010437
node_33: feature_name=GO:0047485 feature_id[121].value <= threshold=6.657836437225342
node_34: feature_name=GO:0090594 feature_id[738].value <= threshold=1.8673912286758423
node_35: feature_name=GO:0043525 feature_id[524].value <= threshold=2.881397008895874
node_36: feature_name=GO:0032504 feature_id[244].value <= threshold=17.22207546234131
node_37: feature_name=GO:0051348 feature_id[126].value <= threshold=5.933559417724609
node_38: feature_name=GO:0042522 feature_id[692].value <= threshold=1.78694087266922
node_39: feature_name=GO:0031100 feature_id[73].value <= threshold=3.2312101125717163
node_40: feature_name=GO:0042493 feature_id[149].value <= threshold=19.771170616149902
node_41: feature_name=GO:0001552 feature_id[366].value <= threshold=2.1711617708206177
node_42: feature_name=GO:0046483 feature_id[391].value <= threshold=320.9667053222656
node_43: feature_name=GO:0002439 feature_id[431].value <= threshold=1.832722783088684
node_44: feature_name=GO:0006555 feature_id[514].value <= threshold=5.321640968322754
node_45: feature_name=GO:0070424 feature_id[254].value <= threshold=3.321003556251526
node_46: feature_name=hsa05144 feature_id[751].value <= threshold=2.635499954223633
node_47: feature_name=GO:0032633 feature_id[627].value > threshold=1.2592533230781555
node_125: feature_name=GO:0033158 feature_id[379].value > threshold=0.9298970401287079
node_131: feature_name=GO:0005634 feature_id[247].value <= threshold=0.20456184579416004
Class: negative genes

Rules_778 passed counts:1
node_0: feature_name=GO:0042113 feature_id[0].value <= threshold=13.408552169799805
node_1: feature_name=GO:0007568 feature_id[534].value <= threshold=5.0313897132873535
node_2: feature_name=GO:0002705 feature_id[541].value <= threshold=3.200145721435547
node_3: feature_name=GO:1901525 feature_id[576].value <= threshold=0.4466460347175598
node_4: feature_name=GO:0048539 feature_id[319].value <= threshold=3.0399646759033203
node_5: feature_name=GO:0001910 feature_id[385].value <= threshold=3.7437864542007446
node_6: feature_name=GO:0043200 feature_id[706].value <= threshold=9.307284355163574
node_7: feature_name=GO:0001773 feature_id[308].value <= threshold=4.016931533813477
node_8: feature_name=GO:0090116 feature_id[97].value <= threshold=7.99645471572876
node_9: feature_name=GO:0019814 feature_id[189].value <= threshold=4.525782108306885
node_10: feature_name=GO:1902583 feature_id[215].value <= threshold=17.60310649871826

node_11: feature_name=GO:0045429 feature_id[737].value <= threshold=1.6133361458778381
node_12: feature_name=GO:0003720 feature_id[228].value <= threshold=5.519326210021973
node_13: feature_name=GO:0046006 feature_id[364].value <= threshold=4.9985432624816895
node_14: feature_name=GO:0070424 feature_id[254].value <= threshold=6.233297109603882
node_15: feature_name=GO:0009892 feature_id[320].value <= threshold=59.99180793762207
node_16: feature_name=GO:0007064 feature_id[527].value <= threshold=11.57377815246582
node_17: feature_name=GO:0005575 feature_id[17].value <= threshold=15.417460918426514
node_18: feature_name=GO:0043368 feature_id[31].value <= threshold=4.059496641159058
node_19: feature_name=GO:0005164 feature_id[163].value <= threshold=5.793607711791992
node_20: feature_name=GO:0042130 feature_id[16].value <= threshold=5.073179721832275
node_21: feature_name=GO:0010216 feature_id[282].value <= threshold=4.834584474563599
node_22: feature_name=GO:0009628 feature_id[553].value <= threshold=29.314892768859863
node_23: feature_name=GO:0045628 feature_id[749].value <= threshold=3.1001367568969727
node_24: feature_name=GO:0042288 feature_id[100].value <= threshold=5.174932956695557
node_25: feature_name=GO:0002329 feature_id[406].value <= threshold=2.348930835723877
node_26: feature_name=GO:0051246 feature_id[642].value <= threshold=0.6498909294605255
node_27: feature_name=GO:0042162 feature_id[355].value <= threshold=8.05103588104248
node_28: feature_name=hsa04668 feature_id[333].value <= threshold=5.614926338195801
node_29: feature_name=GO:0032200 feature_id[613].value <= threshold=28.735913276672363
node_30: feature_name=hsa04662 feature_id[334].value <= threshold=9.588344097137451
node_31: feature_name=GO:1901992 feature_id[105].value <= threshold=5.291427850723267
node_32: feature_name=GO:0072341 feature_id[144].value <= threshold=6.01465106010437
node_33: feature_name=GO:0047485 feature_id[121].value <= threshold=6.657836437225342
node_34: feature_name=GO:0090594 feature_id[738].value <= threshold=1.8673912286758423
node_35: feature_name=GO:0043525 feature_id[524].value <= threshold=2.881397008895874
node_36: feature_name=GO:0032504 feature_id[244].value <= threshold=17.22207546234131
node_37: feature_name=GO:0051348 feature_id[126].value <= threshold=5.933559417724609
node_38: feature_name=GO:0042522 feature_id[692].value <= threshold=1.78694087266922
node_39: feature_name=GO:0031100 feature_id[73].value <= threshold=3.2312101125717163
node_40: feature_name=GO:0042493 feature_id[149].value <= threshold=19.771170616149902
node_41: feature_name=GO:0001552 feature_id[366].value <= threshold=2.1711617708206177
node_42: feature_name=GO:0046483 feature_id[391].value <= threshold=320.9667053222656
node_43: feature_name=GO:0002439 feature_id[431].value <= threshold=1.832722783088684
node_44: feature_name=GO:0006555 feature_id[514].value <= threshold=5.321640968322754
node_45: feature_name=GO:0070424 feature_id[254].value <= threshold=3.321003556251526

node_46: feature_name=hsa05144 feature_id[751].value <= threshold=2.635499954223633
node_47: feature_name=GO:0032633 feature_id[627].value > threshold=1.2592533230781555
node_125: feature_name=GO:0033158 feature_id[379].value <= threshold=0.9298970401287079
node_126: feature_name=GO:0051246 feature_id[642].value <= threshold=5.402702754508937e-06
node_127: feature_name=GO:0001817 feature_id[715].value > threshold=0.718599796295166
Class: negative genes

Rules_779 passed counts:1
node_0: feature_name=GO:0042113 feature_id[0].value <= threshold=13.408552169799805
node_1: feature_name=GO:0007568 feature_id[534].value <= threshold=5.0313897132873535
node_2: feature_name=GO:0002705 feature_id[541].value <= threshold=3.200145721435547
node_3: feature_name=GO:1901525 feature_id[576].value <= threshold=0.4466460347175598
node_4: feature_name=GO:0048539 feature_id[319].value <= threshold=3.0399646759033203
node_5: feature_name=GO:0001910 feature_id[385].value <= threshold=3.7437864542007446
node_6: feature_name=GO:0043200 feature_id[706].value <= threshold=9.307284355163574
node_7: feature_name=GO:0001773 feature_id[308].value <= threshold=4.016931533813477
node_8: feature_name=GO:0090116 feature_id[97].value <= threshold=7.99645471572876
node_9: feature_name=GO:0019814 feature_id[189].value <= threshold=4.525782108306885
node_10: feature_name=GO:1902583 feature_id[215].value <= threshold=17.60310649871826
node_11: feature_name=GO:0045429 feature_id[737].value <= threshold=1.6133361458778381
node_12: feature_name=GO:0003720 feature_id[228].value <= threshold=5.519326210021973
node_13: feature_name=GO:0046006 feature_id[364].value <= threshold=4.9985432624816895
node_14: feature_name=GO:0070424 feature_id[254].value <= threshold=6.233297109603882
node_15: feature_name=GO:0009892 feature_id[320].value <= threshold=59.99180793762207
node_16: feature_name=GO:0007064 feature_id[527].value <= threshold=11.57377815246582
node_17: feature_name=GO:0005575 feature_id[17].value <= threshold=15.417460918426514
node_18: feature_name=GO:0043368 feature_id[31].value <= threshold=4.059496641159058
node_19: feature_name=GO:0005164 feature_id[163].value <= threshold=5.793607711791992
node_20: feature_name=GO:0042130 feature_id[16].value <= threshold=5.073179721832275
node_21: feature_name=GO:0010216 feature_id[282].value <= threshold=4.834584474563599
node_22: feature_name=GO:0009628 feature_id[553].value <= threshold=29.314892768859863
node_23: feature_name=GO:0045628 feature_id[749].value <= threshold=3.1001367568969727
node_24: feature_name=GO:0042288 feature_id[100].value <= threshold=5.174932956695557
node_25: feature_name=GO:0002329 feature_id[406].value <= threshold=2.348930835723877
node_26: feature_name=GO:0051246 feature_id[642].value <= threshold=0.6498909294605255

node_27: feature_name=GO:0042162 feature_id[355].value <= threshold=8.05103588104248
node_28: feature_name=hsa04668 feature_id[333].value <= threshold=5.614926338195801
node_29: feature_name=GO:0032200 feature_id[613].value <= threshold=28.735913276672363
node_30: feature_name=hsa04662 feature_id[334].value <= threshold=9.588344097137451
node_31: feature_name=GO:1901992 feature_id[105].value <= threshold=5.291427850723267
node_32: feature_name=GO:0072341 feature_id[144].value <= threshold=6.01465106010437
node_33: feature_name=GO:0047485 feature_id[121].value <= threshold=6.657836437225342
node_34: feature_name=GO:0090594 feature_id[738].value <= threshold=1.8673912286758423
node_35: feature_name=GO:0043525 feature_id[524].value <= threshold=2.881397008895874
node_36: feature_name=GO:0032504 feature_id[244].value <= threshold=17.22207546234131
node_37: feature_name=GO:0051348 feature_id[126].value <= threshold=5.933559417724609
node_38: feature_name=GO:0042522 feature_id[692].value <= threshold=1.78694087266922
node_39: feature_name=GO:0031100 feature_id[73].value <= threshold=3.2312101125717163
node_40: feature_name=GO:0042493 feature_id[149].value <= threshold=19.771170616149902
node_41: feature_name=GO:0001552 feature_id[366].value <= threshold=2.1711617708206177
node_42: feature_name=GO:0046483 feature_id[391].value <= threshold=320.9667053222656
node_43: feature_name=GO:0002439 feature_id[431].value <= threshold=1.832722783088684
node_44: feature_name=GO:0006555 feature_id[514].value <= threshold=5.321640968322754
node_45: feature_name=GO:0070424 feature_id[254].value <= threshold=3.321003556251526
node_46: feature_name=hsa05144 feature_id[751].value <= threshold=2.635499954223633
node_47: feature_name=GO:0032633 feature_id[627].value > threshold=1.2592533230781555
node_125: feature_name=GO:0033158 feature_id[379].value <= threshold=0.9298970401287079
node_126: feature_name=GO:0051246 feature_id[642].value <= threshold=5.402702754508937e-06
node_127: feature_name=GO:0001817 feature_id[715].value <= threshold=0.718599796295166
Class: positive genes

Rules_780 passed counts:1
node_0: feature_name=GO:0042113 feature_id[0].value <= threshold=13.408552169799805
node_1: feature_name=GO:0007568 feature_id[534].value <= threshold=5.0313897132873535
node_2: feature_name=GO:0002705 feature_id[541].value <= threshold=3.200145721435547
node_3: feature_name=GO:1901525 feature_id[576].value <= threshold=0.4466460347175598
node_4: feature_name=GO:0048539 feature_id[319].value <= threshold=3.0399646759033203
node_5: feature_name=GO:0001910 feature_id[385].value <= threshold=3.7437864542007446
node_6: feature_name=GO:0043200 feature_id[706].value <= threshold=9.307284355163574
node_7: feature_name=GO:0001773 feature_id[308].value <= threshold=4.016931533813477

node_8: feature_name=GO:0090116	feature_id[97].value <= threshold=7.99645471572876
node_9: feature_name=GO:0019814	feature_id[189].value <= threshold=4.525782108306885
node_10: feature_name=GO:1902583	feature_id[215].value <= threshold=17.60310649871826
node_11: feature_name=GO:0045429	feature_id[737].value <= threshold=1.6133361458778381
node_12: feature_name=GO:0003720	feature_id[228].value <= threshold=5.519326210021973
node_13: feature_name=GO:0046006	feature_id[364].value <= threshold=4.9985432624816895
node_14: feature_name=GO:0070424	feature_id[254].value <= threshold=6.233297109603882
node_15: feature_name=GO:0009892	feature_id[320].value <= threshold=59.99180793762207
node_16: feature_name=GO:0007064	feature_id[527].value <= threshold=11.57377815246582
node_17: feature_name=GO:0005575	feature_id[17].value <= threshold=15.417460918426514
node_18: feature_name=GO:0043368	feature_id[31].value <= threshold=4.059496641159058
node_19: feature_name=GO:0005164	feature_id[163].value <= threshold=5.793607711791992
node_20: feature_name=GO:0042130	feature_id[16].value <= threshold=5.073179721832275
node_21: feature_name=GO:0010216	feature_id[282].value <= threshold=4.834584474563599
node_22: feature_name=GO:0009628	feature_id[553].value <= threshold=29.314892768859863
node_23: feature_name=GO:0045628	feature_id[749].value <= threshold=3.1001367568969727
node_24: feature_name=GO:0042288	feature_id[100].value <= threshold=5.174932956695557
node_25: feature_name=GO:0002329	feature_id[406].value <= threshold=2.348930835723877
node_26: feature_name=GO:0051246	feature_id[642].value <= threshold=0.6498909294605255
node_27: feature_name=GO:0042162	feature_id[355].value <= threshold=8.05103588104248
node_28: feature_name=hsa04668	feature_id[333].value <= threshold=5.614926338195801
node_29: feature_name=GO:0032200	feature_id[613].value <= threshold=28.735913276672363
node_30: feature_name=hsa04662	feature_id[334].value <= threshold=9.588344097137451
node_31: feature_name=GO:1901992	feature_id[105].value <= threshold=5.291427850723267
node_32: feature_name=GO:0072341	feature_id[144].value <= threshold=6.01465106010437
node_33: feature_name=GO:0047485	feature_id[121].value <= threshold=6.657836437225342
node_34: feature_name=GO:0090594	feature_id[738].value <= threshold=1.8673912286758423
node_35: feature_name=GO:0043525	feature_id[524].value <= threshold=2.881397008895874
node_36: feature_name=GO:0032504	feature_id[244].value <= threshold=17.22207546234131
node_37: feature_name=GO:0051348	feature_id[126].value <= threshold=5.933559417724609
node_38: feature_name=GO:0042522	feature_id[692].value <= threshold=1.78694087266922
node_39: feature_name=GO:0031100	feature_id[73].value <= threshold=3.2312101125717163
node_40: feature_name=GO:0042493	feature_id[149].value <= threshold=19.771170616149902
node_41: feature_name=GO:0001552	feature_id[366].value <= threshold=2.1711617708206177
node_42: feature_name=GO:0046483	feature_id[391].value <= threshold=320.9667053222656

node_43: feature_name=GO:0002439 feature_id[431].value <= threshold=1.832722783088684
node_44: feature_name=GO:0006555 feature_id[514].value <= threshold=5.321640968322754
node_45: feature_name=GO:0070424 feature_id[254].value <= threshold=3.321003556251526
node_46: feature_name=hsa05144 feature_id[751].value <= threshold=2.635499954223633
node_47: feature_name=GO:0032633 feature_id[627].value <= threshold=1.2592533230781555
node_48: feature_name=GO:0019814 feature_id[189].value > threshold=2.1583125591278076
node_122: feature_name=GO:0008156 feature_id[239].value > threshold=1.1327534914016724
Class: positive genes

Rules_781 passed counts:1
node_0: feature_name=GO:0042113 feature_id[0].value <= threshold=13.408552169799805
node_1: feature_name=GO:0007568 feature_id[534].value <= threshold=5.0313897132873535
node_2: feature_name=GO:0002705 feature_id[541].value <= threshold=3.200145721435547
node_3: feature_name=GO:1901525 feature_id[576].value <= threshold=0.4466460347175598
node_4: feature_name=GO:0048539 feature_id[319].value <= threshold=3.0399646759033203
node_5: feature_name=GO:0001910 feature_id[385].value <= threshold=3.7437864542007446
node_6: feature_name=GO:0043200 feature_id[706].value <= threshold=9.307284355163574
node_7: feature_name=GO:0001773 feature_id[308].value <= threshold=4.016931533813477
node_8: feature_name=GO:0090116 feature_id[97].value <= threshold=7.99645471572876
node_9: feature_name=GO:0019814 feature_id[189].value <= threshold=4.525782108306885
node_10: feature_name=GO:1902583 feature_id[215].value <= threshold=17.60310649871826
node_11: feature_name=GO:0045429 feature_id[737].value <= threshold=1.6133361458778381
node_12: feature_name=GO:0003720 feature_id[228].value <= threshold=5.519326210021973
node_13: feature_name=GO:0046006 feature_id[364].value <= threshold=4.9985432624816895
node_14: feature_name=GO:0070424 feature_id[254].value <= threshold=6.233297109603882
node_15: feature_name=GO:0009892 feature_id[320].value <= threshold=59.99180793762207
node_16: feature_name=GO:0007064 feature_id[527].value <= threshold=11.57377815246582
node_17: feature_name=GO:0005575 feature_id[17].value <= threshold=15.417460918426514
node_18: feature_name=GO:0043368 feature_id[31].value <= threshold=4.059496641159058
node_19: feature_name=GO:0005164 feature_id[163].value <= threshold=5.793607711791992
node_20: feature_name=GO:0042130 feature_id[16].value <= threshold=5.073179721832275
node_21: feature_name=GO:0010216 feature_id[282].value <= threshold=4.834584474563599
node_22: feature_name=GO:0009628 feature_id[553].value <= threshold=29.314892768859863
node_23: feature_name=GO:0045628 feature_id[749].value <= threshold=3.1001367568969727
node_24: feature_name=GO:0042288 feature_id[100].value <= threshold=5.174932956695557

node_25: feature_name=GO:0002329 feature_id[406].value <= threshold=2.348930835723877
node_26: feature_name=GO:0051246 feature_id[642].value <= threshold=0.6498909294605255
node_27: feature_name=GO:0042162 feature_id[355].value <= threshold=8.05103588104248
node_28: feature_name=hsa04668 feature_id[333].value <= threshold=5.614926338195801
node_29: feature_name=GO:0032200 feature_id[613].value <= threshold=28.735913276672363
node_30: feature_name=hsa04662 feature_id[334].value <= threshold=9.588344097137451
node_31: feature_name=GO:1901992 feature_id[105].value <= threshold=5.291427850723267
node_32: feature_name=GO:0072341 feature_id[144].value <= threshold=6.01465106010437
node_33: feature_name=GO:0047485 feature_id[121].value <= threshold=6.657836437225342
node_34: feature_name=GO:0090594 feature_id[738].value <= threshold=1.8673912286758423
node_35: feature_name=GO:0043525 feature_id[524].value <= threshold=2.881397008895874
node_36: feature_name=GO:0032504 feature_id[244].value <= threshold=17.22207546234131
node_37: feature_name=GO:0051348 feature_id[126].value <= threshold=5.933559417724609
node_38: feature_name=GO:0042522 feature_id[692].value <= threshold=1.78694087266922
node_39: feature_name=GO:0031100 feature_id[73].value <= threshold=3.2312101125717163
node_40: feature_name=GO:0042493 feature_id[149].value <= threshold=19.771170616149902
node_41: feature_name=GO:0001552 feature_id[366].value <= threshold=2.1711617708206177
node_42: feature_name=GO:0046483 feature_id[391].value <= threshold=320.9667053222656
node_43: feature_name=GO:0002439 feature_id[431].value <= threshold=1.832722783088684
node_44: feature_name=GO:0006555 feature_id[514].value <= threshold=5.321640968322754
node_45: feature_name=GO:0070424 feature_id[254].value <= threshold=3.321003556251526
node_46: feature_name=hsa05144 feature_id[751].value <= threshold=2.635499954223633
node_47: feature_name=GO:0032633 feature_id[627].value <= threshold=1.2592533230781555
node_48: feature_name=GO:0019814 feature_id[189].value <= threshold=2.1583125591278076
node_49: feature_name=GO:1901989 feature_id[30].value > threshold=3.3150794506073
node_119: feature_name=GO:0032268 feature_id[249].value <= threshold=0.04715394601225853
Class: positive genes

Rules_782 passed counts:1
node_0: feature_name=GO:0042113 feature_id[0].value <= threshold=13.408552169799805
node_1: feature_name=GO:0007568 feature_id[534].value <= threshold=5.0313897132873535
node_2: feature_name=GO:0002705 feature_id[541].value <= threshold=3.200145721435547
node_3: feature_name=GO:1901525 feature_id[576].value <= threshold=0.4466460347175598
node_4: feature_name=GO:0048539 feature_id[319].value <= threshold=3.0399646759033203
node_5: feature_name=GO:0001910 feature_id[385].value <= threshold=3.7437864542007446

node_6: feature_name=GO:0043200	feature_id[706].value <= threshold=9.307284355163574
node_7: feature_name=GO:0001773	feature_id[308].value <= threshold=4.016931533813477
node_8: feature_name=GO:0090116	feature_id[97].value <= threshold=7.99645471572876
node_9: feature_name=GO:0019814	feature_id[189].value <= threshold=4.525782108306885
node_10: feature_name=GO:1902583	feature_id[215].value <= threshold=17.60310649871826
node_11: feature_name=GO:0045429	feature_id[737].value <= threshold=1.6133361458778381
node_12: feature_name=GO:0003720	feature_id[228].value <= threshold=5.519326210021973
node_13: feature_name=GO:0046006	feature_id[364].value <= threshold=4.9985432624816895
node_14: feature_name=GO:0070424	feature_id[254].value <= threshold=6.233297109603882
node_15: feature_name=GO:0009892	feature_id[320].value <= threshold=59.99180793762207
node_16: feature_name=GO:0007064	feature_id[527].value <= threshold=11.57377815246582
node_17: feature_name=GO:0005575	feature_id[17].value <= threshold=15.417460918426514
node_18: feature_name=GO:0043368	feature_id[31].value <= threshold=4.059496641159058
node_19: feature_name=GO:0005164	feature_id[163].value <= threshold=5.793607711791992
node_20: feature_name=GO:0042130	feature_id[16].value <= threshold=5.073179721832275
node_21: feature_name=GO:0010216	feature_id[282].value <= threshold=4.834584474563599
node_22: feature_name=GO:0009628	feature_id[553].value <= threshold=29.314892768859863
node_23: feature_name=GO:0045628	feature_id[749].value <= threshold=3.1001367568969727
node_24: feature_name=GO:0042288	feature_id[100].value <= threshold=5.174932956695557
node_25: feature_name=GO:0002329	feature_id[406].value <= threshold=2.348930835723877
node_26: feature_name=GO:0051246	feature_id[642].value <= threshold=0.6498909294605255
node_27: feature_name=GO:0042162	feature_id[355].value <= threshold=8.05103588104248
node_28: feature_name=hsa04668	feature_id[333].value <= threshold=5.614926338195801
node_29: feature_name=GO:0032200	feature_id[613].value <= threshold=28.735913276672363
node_30: feature_name=hsa04662	feature_id[334].value <= threshold=9.588344097137451
node_31: feature_name=GO:1901992	feature_id[105].value <= threshold=5.291427850723267
node_32: feature_name=GO:0072341	feature_id[144].value <= threshold=6.01465106010437
node_33: feature_name=GO:0047485	feature_id[121].value <= threshold=6.657836437225342
node_34: feature_name=GO:0090594	feature_id[738].value <= threshold=1.8673912286758423
node_35: feature_name=GO:0043525	feature_id[524].value <= threshold=2.881397008895874
node_36: feature_name=GO:0032504	feature_id[244].value <= threshold=17.22207546234131
node_37: feature_name=GO:0051348	feature_id[126].value <= threshold=5.933559417724609
node_38: feature_name=GO:0042522	feature_id[692].value <= threshold=1.78694087266922
node_39: feature_name=GO:0031100	feature_id[73].value <= threshold=3.2312101125717163
node_40: feature_name=GO:0042493	feature_id[149].value <= threshold=19.771170616149902

node_41: feature_name=GO:0001552 feature_id[366].value <= threshold=2.1711617708206177
node_42: feature_name=GO:0046483 feature_id[391].value <= threshold=320.9667053222656
node_43: feature_name=GO:0002439 feature_id[431].value <= threshold=1.832722783088684
node_44: feature_name=GO:0006555 feature_id[514].value <= threshold=5.321640968322754
node_45: feature_name=GO:0070424 feature_id[254].value <= threshold=3.321003556251526
node_46: feature_name=hsa05144 feature_id[751].value <= threshold=2.635499954223633
node_47: feature_name=GO:0032633 feature_id[627].value <= threshold=1.2592533230781555
node_48: feature_name=GO:0019814 feature_id[189].value <= threshold=2.1583125591278076
node_49: feature_name=GO:1901989 feature_id[30].value <= threshold=3.3150794506073
node_50: feature_name=GO:1903318 feature_id[196].value > threshold=1.62252539396286
node_116: feature_name=GO:0005634 feature_id[247].value <= threshold=4.554542215373658e-06
Class: positive genes

Rules_783 passed counts:1
node_0: feature_name=GO:0042113 feature_id[0].value <= threshold=13.408552169799805
node_1: feature_name=GO:0007568 feature_id[534].value <= threshold=5.0313897132873535
node_2: feature_name=GO:0002705 feature_id[541].value <= threshold=3.200145721435547
node_3: feature_name=GO:1901525 feature_id[576].value <= threshold=0.4466460347175598
node_4: feature_name=GO:0048539 feature_id[319].value <= threshold=3.0399646759033203
node_5: feature_name=GO:0001910 feature_id[385].value <= threshold=3.7437864542007446
node_6: feature_name=GO:0043200 feature_id[706].value <= threshold=9.307284355163574
node_7: feature_name=GO:0001773 feature_id[308].value <= threshold=4.016931533813477
node_8: feature_name=GO:0090116 feature_id[97].value <= threshold=7.99645471572876
node_9: feature_name=GO:0019814 feature_id[189].value <= threshold=4.525782108306885
node_10: feature_name=GO:1902583 feature_id[215].value <= threshold=17.60310649871826
node_11: feature_name=GO:0045429 feature_id[737].value <= threshold=1.6133361458778381
node_12: feature_name=GO:0003720 feature_id[228].value <= threshold=5.519326210021973
node_13: feature_name=GO:0046006 feature_id[364].value <= threshold=4.9985432624816895
node_14: feature_name=GO:0070424 feature_id[254].value <= threshold=6.233297109603882
node_15: feature_name=GO:0009892 feature_id[320].value <= threshold=59.99180793762207
node_16: feature_name=GO:0007064 feature_id[527].value <= threshold=11.57377815246582
node_17: feature_name=GO:0005575 feature_id[17].value <= threshold=15.417460918426514
node_18: feature_name=GO:0043368 feature_id[31].value <= threshold=4.059496641159058
node_19: feature_name=GO:0005164 feature_id[163].value <= threshold=5.793607711791992
node_20: feature_name=GO:0042130 feature_id[16].value <= threshold=5.073179721832275

node_21: feature_name=GO:0010216	feature_id[282].value <= threshold=4.834584474563599
node_22: feature_name=GO:0009628	feature_id[553].value <= threshold=29.314892768859863
node_23: feature_name=GO:0045628	feature_id[749].value <= threshold=3.1001367568969727
node_24: feature_name=GO:0042288	feature_id[100].value <= threshold=5.174932956695557
node_25: feature_name=GO:0002329	feature_id[406].value <= threshold=2.348930835723877
node_26: feature_name=GO:0051246	feature_id[642].value <= threshold=0.6498909294605255
node_27: feature_name=GO:0042162	feature_id[355].value <= threshold=8.05103588104248
node_28: feature_name=hsa04668	feature_id[333].value <= threshold=5.614926338195801
node_29: feature_name=GO:0032200	feature_id[613].value <= threshold=28.735913276672363
node_30: feature_name=hsa04662	feature_id[334].value <= threshold=9.588344097137451
node_31: feature_name=GO:1901992	feature_id[105].value <= threshold=5.291427850723267
node_32: feature_name=GO:0072341	feature_id[144].value <= threshold=6.01465106010437
node_33: feature_name=GO:0047485	feature_id[121].value <= threshold=6.657836437225342
node_34: feature_name=GO:0090594	feature_id[738].value <= threshold=1.8673912286758423
node_35: feature_name=GO:0043525	feature_id[524].value <= threshold=2.881397008895874
node_36: feature_name=GO:0032504	feature_id[244].value <= threshold=17.22207546234131
node_37: feature_name=GO:0051348	feature_id[126].value <= threshold=5.933559417724609
node_38: feature_name=GO:0042522	feature_id[692].value <= threshold=1.78694087266922
node_39: feature_name=GO:0031100	feature_id[73].value <= threshold=3.2312101125717163
node_40: feature_name=GO:0042493	feature_id[149].value <= threshold=19.771170616149902
node_41: feature_name=GO:0001552	feature_id[366].value <= threshold=2.1711617708206177
node_42: feature_name=GO:0046483	feature_id[391].value <= threshold=320.9667053222656
node_43: feature_name=GO:0002439	feature_id[431].value <= threshold=1.832722783088684
node_44: feature_name=GO:0006555	feature_id[514].value <= threshold=5.321640968322754
node_45: feature_name=GO:0070424	feature_id[254].value <= threshold=3.321003556251526
node_46: feature_name=hsa05144	feature_id[751].value <= threshold=2.635499954223633
node_47: feature_name=GO:0032633	feature_id[627].value <= threshold=1.2592533230781555
node_48: feature_name=GO:0019814	feature_id[189].value <= threshold=2.1583125591278076
node_49: feature_name=GO:1901989	feature_id[30].value <= threshold=3.3150794506073
node_50: feature_name=GO:1903318	feature_id[196].value <= threshold=1.62252539396286
node_51: feature_name=GO:0060576	feature_id[204].value > threshold=2.1097792387008667
node_113: feature_name=GO:0060374	feature_id[76].value > threshold=1.0223779380321503

Class: positive genes

Rules_784 passed counts:1

node_0: feature_name=GO:0042113 feature_id[0].value <= threshold=13.408552169799805
node_1: feature_name=GO:0007568 feature_id[534].value <= threshold=5.0313897132873535
node_2: feature_name=GO:0002705 feature_id[541].value <= threshold=3.200145721435547
node_3: feature_name=GO:1901525 feature_id[576].value <= threshold=0.4466460347175598
node_4: feature_name=GO:0048539 feature_id[319].value <= threshold=3.0399646759033203
node_5: feature_name=GO:0001910 feature_id[385].value <= threshold=3.7437864542007446
node_6: feature_name=GO:0043200 feature_id[706].value <= threshold=9.307284355163574
node_7: feature_name=GO:0001773 feature_id[308].value <= threshold=4.016931533813477
node_8: feature_name=GO:0090116 feature_id[97].value <= threshold=7.99645471572876
node_9: feature_name=GO:0019814 feature_id[189].value <= threshold=4.525782108306885
node_10: feature_name=GO:1902583 feature_id[215].value <= threshold=17.60310649871826
node_11: feature_name=GO:0045429 feature_id[737].value <= threshold=1.6133361458778381
node_12: feature_name=GO:0003720 feature_id[228].value <= threshold=5.519326210021973
node_13: feature_name=GO:0046006 feature_id[364].value <= threshold=4.9985432624816895
node_14: feature_name=GO:0070424 feature_id[254].value <= threshold=6.233297109603882
node_15: feature_name=GO:0009892 feature_id[320].value <= threshold=59.99180793762207
node_16: feature_name=GO:0007064 feature_id[527].value <= threshold=11.57377815246582
node_17: feature_name=GO:0005575 feature_id[17].value <= threshold=15.417460918426514
node_18: feature_name=GO:0043368 feature_id[31].value <= threshold=4.059496641159058
node_19: feature_name=GO:0005164 feature_id[163].value <= threshold=5.793607711791992
node_20: feature_name=GO:0042130 feature_id[16].value <= threshold=5.073179721832275
node_21: feature_name=GO:0010216 feature_id[282].value <= threshold=4.834584474563599
node_22: feature_name=GO:0009628 feature_id[553].value <= threshold=29.314892768859863
node_23: feature_name=GO:0045628 feature_id[749].value <= threshold=3.1001367568969727
node_24: feature_name=GO:0042288 feature_id[100].value <= threshold=5.174932956695557
node_25: feature_name=GO:0002329 feature_id[406].value <= threshold=2.348930835723877
node_26: feature_name=GO:0051246 feature_id[642].value <= threshold=0.6498909294605255
node_27: feature_name=GO:0042162 feature_id[355].value <= threshold=8.05103588104248
node_28: feature_name=hsa04668 feature_id[333].value <= threshold=5.614926338195801
node_29: feature_name=GO:0032200 feature_id[613].value <= threshold=28.735913276672363
node_30: feature_name=hsa04662 feature_id[334].value <= threshold=9.588344097137451
node_31: feature_name=GO:1901992 feature_id[105].value <= threshold=5.291427850723267
node_32: feature_name=GO:0072341 feature_id[144].value <= threshold=6.01465106010437
node_33: feature_name=GO:0047485 feature_id[121].value <= threshold=6.657836437225342
node_34: feature_name=GO:0090594 feature_id[738].value <= threshold=1.8673912286758423

node_35: feature_name=GO:0043525 feature_id[524].value <= threshold=2.881397008895874
node_36: feature_name=GO:0032504 feature_id[244].value <= threshold=17.22207546234131
node_37: feature_name=GO:0051348 feature_id[126].value <= threshold=5.933559417724609
node_38: feature_name=GO:0042522 feature_id[692].value <= threshold=1.78694087266922
node_39: feature_name=GO:0031100 feature_id[73].value <= threshold=3.2312101125717163
node_40: feature_name=GO:0042493 feature_id[149].value <= threshold=19.771170616149902
node_41: feature_name=GO:0001552 feature_id[366].value <= threshold=2.1711617708206177
node_42: feature_name=GO:0046483 feature_id[391].value <= threshold=320.9667053222656
node_43: feature_name=GO:0002439 feature_id[431].value <= threshold=1.832722783088684
node_44: feature_name=GO:0006555 feature_id[514].value <= threshold=5.321640968322754
node_45: feature_name=GO:0070424 feature_id[254].value <= threshold=3.321003556251526
node_46: feature_name=hsa05144 feature_id[751].value <= threshold=2.635499954223633
node_47: feature_name=GO:0032633 feature_id[627].value <= threshold=1.2592533230781555
node_48: feature_name=GO:0019814 feature_id[189].value <= threshold=2.1583125591278076
node_49: feature_name=GO:1901989 feature_id[30].value <= threshold=3.3150794506073
node_50: feature_name=GO:1903318 feature_id[196].value <= threshold=1.62252539396286
node_51: feature_name=GO:0060576 feature_id[204].value <= threshold=2.1097792387008667
node_52: feature_name=hsa05221 feature_id[349].value > threshold=0.8723124265670776
node_96: feature_name=hsa05221 feature_id[349].value > threshold=0.8756309151649475
node_98: feature_name=GO:0046794 feature_id[794].value > threshold=6.585465431213379
Class: positive genes

Rules_785 passed counts:1
node_0: feature_name=GO:0042113 feature_id[0].value <= threshold=13.408552169799805
node_1: feature_name=GO:0007568 feature_id[534].value <= threshold=5.0313897132873535
node_2: feature_name=GO:0002705 feature_id[541].value <= threshold=3.200145721435547
node_3: feature_name=GO:1901525 feature_id[576].value <= threshold=0.4466460347175598
node_4: feature_name=GO:0048539 feature_id[319].value <= threshold=3.0399646759033203
node_5: feature_name=GO:0001910 feature_id[385].value <= threshold=3.7437864542007446
node_6: feature_name=GO:0043200 feature_id[706].value <= threshold=9.307284355163574
node_7: feature_name=GO:0001773 feature_id[308].value <= threshold=4.016931533813477
node_8: feature_name=GO:0090116 feature_id[97].value <= threshold=7.99645471572876
node_9: feature_name=GO:0019814 feature_id[189].value <= threshold=4.525782108306885
node_10: feature_name=GO:1902583 feature_id[215].value <= threshold=17.60310649871826
node_11: feature_name=GO:0045429 feature_id[737].value <= threshold=1.6133361458778381

node_12: feature_name=GO:0003720	feature_id[228].value <= threshold=5.519326210021973
node_13: feature_name=GO:0046006	feature_id[364].value <= threshold=4.9985432624816895
node_14: feature_name=GO:0070424	feature_id[254].value <= threshold=6.233297109603882
node_15: feature_name=GO:0009892	feature_id[320].value <= threshold=59.99180793762207
node_16: feature_name=GO:0007064	feature_id[527].value <= threshold=11.57377815246582
node_17: feature_name=GO:0005575	feature_id[17].value <= threshold=15.417460918426514
node_18: feature_name=GO:0043368	feature_id[31].value <= threshold=4.059496641159058
node_19: feature_name=GO:0005164	feature_id[163].value <= threshold=5.793607711791992
node_20: feature_name=GO:0042130	feature_id[16].value <= threshold=5.073179721832275
node_21: feature_name=GO:0010216	feature_id[282].value <= threshold=4.834584474563599
node_22: feature_name=GO:0009628	feature_id[553].value <= threshold=29.314892768859863
node_23: feature_name=GO:0045628	feature_id[749].value <= threshold=3.1001367568969727
node_24: feature_name=GO:0042288	feature_id[100].value <= threshold=5.174932956695557
node_25: feature_name=GO:0002329	feature_id[406].value <= threshold=2.348930835723877
node_26: feature_name=GO:0051246	feature_id[642].value <= threshold=0.6498909294605255
node_27: feature_name=GO:0042162	feature_id[355].value <= threshold=8.05103588104248
node_28: feature_name=hsa04668	feature_id[333].value <= threshold=5.614926338195801
node_29: feature_name=GO:0032200	feature_id[613].value <= threshold=28.735913276672363
node_30: feature_name=hsa04662	feature_id[334].value <= threshold=9.588344097137451
node_31: feature_name=GO:1901992	feature_id[105].value <= threshold=5.291427850723267
node_32: feature_name=GO:0072341	feature_id[144].value <= threshold=6.01465106010437
node_33: feature_name=GO:0047485	feature_id[121].value <= threshold=6.657836437225342
node_34: feature_name=GO:0090594	feature_id[738].value <= threshold=1.8673912286758423
node_35: feature_name=GO:0043525	feature_id[524].value <= threshold=2.881397008895874
node_36: feature_name=GO:0032504	feature_id[244].value <= threshold=17.22207546234131
node_37: feature_name=GO:0051348	feature_id[126].value <= threshold=5.933559417724609
node_38: feature_name=GO:0042522	feature_id[692].value <= threshold=1.78694087266922
node_39: feature_name=GO:0031100	feature_id[73].value <= threshold=3.2312101125717163
node_40: feature_name=GO:0042493	feature_id[149].value <= threshold=19.771170616149902
node_41: feature_name=GO:0001552	feature_id[366].value <= threshold=2.1711617708206177
node_42: feature_name=GO:0046483	feature_id[391].value <= threshold=320.9667053222656
node_43: feature_name=GO:0002439	feature_id[431].value <= threshold=1.832722783088684
node_44: feature_name=GO:0006555	feature_id[514].value <= threshold=5.321640968322754
node_45: feature_name=GO:0070424	feature_id[254].value <= threshold=3.321003556251526
node_46: feature_name=hsa05144	feature_id[751].value <= threshold=2.635499954223633

node_47: feature_name=GO:0032633	feature_id[627].value <= threshold=1.2592533230781555
node_48: feature_name=GO:0019814	feature_id[189].value <= threshold=2.1583125591278076
node_49: feature_name=GO:1901989	feature_id[30].value <= threshold=3.3150794506073
node_50: feature_name=GO:1903318	feature_id[196].value <= threshold=1.62252539396286
node_51: feature_name=GO:0060576	feature_id[204].value <= threshold=2.1097792387008667
node_52: feature_name=hsa05221	feature_id[349].value > threshold=0.8723124265670776
node_96: feature_name=hsa05221	feature_id[349].value > threshold=0.8756309151649475
node_98: feature_name=GO:0046794	feature_id[794].value <= threshold=6.585465431213379
node_99: feature_name=GO:0005035	feature_id[133].value <= threshold=0.6986835598945618
node_100: feature_name=hsa04640	feature_id[79].value > threshold=1.458605408668518
node_106: feature_name=GO:0071901	feature_id[407].value > threshold=0.31348803639411926
Class: positive genes	

Rules_786	passed counts:1
node_0: feature_name=GO:0042113	feature_id[0].value <= threshold=13.408552169799805
node_1: feature_name=GO:0007568	feature_id[534].value <= threshold=5.0313897132873535
node_2: feature_name=GO:0002705	feature_id[541].value <= threshold=3.200145721435547
node_3: feature_name=GO:1901525	feature_id[576].value <= threshold=0.4466460347175598
node_4: feature_name=GO:0048539	feature_id[319].value <= threshold=3.0399646759033203
node_5: feature_name=GO:0001910	feature_id[385].value <= threshold=3.7437864542007446
node_6: feature_name=GO:0043200	feature_id[706].value <= threshold=9.307284355163574
node_7: feature_name=GO:0001773	feature_id[308].value <= threshold=4.016931533813477
node_8: feature_name=GO:0090116	feature_id[97].value <= threshold=7.99645471572876
node_9: feature_name=GO:0019814	feature_id[189].value <= threshold=4.525782108306885
node_10: feature_name=GO:1902583	feature_id[215].value <= threshold=17.60310649871826
node_11: feature_name=GO:0045429	feature_id[737].value <= threshold=1.6133361458778381
node_12: feature_name=GO:0003720	feature_id[228].value <= threshold=5.519326210021973
node_13: feature_name=GO:0046006	feature_id[364].value <= threshold=4.9985432624816895
node_14: feature_name=GO:0070424	feature_id[254].value <= threshold=6.233297109603882
node_15: feature_name=GO:0009892	feature_id[320].value <= threshold=59.99180793762207
node_16: feature_name=GO:0007064	feature_id[527].value <= threshold=11.57377815246582
node_17: feature_name=GO:0005575	feature_id[17].value <= threshold=15.417460918426514
node_18: feature_name=GO:0043368	feature_id[31].value <= threshold=4.059496641159058
node_19: feature_name=GO:0005164	feature_id[163].value <= threshold=5.793607711791992
node_20: feature_name=GO:0042130	feature_id[16].value <= threshold=5.073179721832275

node_21: feature_name=GO:0010216	feature_id[282].value <= threshold=4.834584474563599
node_22: feature_name=GO:0009628	feature_id[553].value <= threshold=29.314892768859863
node_23: feature_name=GO:0045628	feature_id[749].value <= threshold=3.1001367568969727
node_24: feature_name=GO:0042288	feature_id[100].value <= threshold=5.174932956695557
node_25: feature_name=GO:0002329	feature_id[406].value <= threshold=2.348930835723877
node_26: feature_name=GO:0051246	feature_id[642].value <= threshold=0.6498909294605255
node_27: feature_name=GO:0042162	feature_id[355].value <= threshold=8.05103588104248
node_28: feature_name=hsa04668	feature_id[333].value <= threshold=5.614926338195801
node_29: feature_name=GO:0032200	feature_id[613].value <= threshold=28.735913276672363
node_30: feature_name=hsa04662	feature_id[334].value <= threshold=9.588344097137451
node_31: feature_name=GO:1901992	feature_id[105].value <= threshold=5.291427850723267
node_32: feature_name=GO:0072341	feature_id[144].value <= threshold=6.01465106010437
node_33: feature_name=GO:0047485	feature_id[121].value <= threshold=6.657836437225342
node_34: feature_name=GO:0090594	feature_id[738].value <= threshold=1.8673912286758423
node_35: feature_name=GO:0043525	feature_id[524].value <= threshold=2.881397008895874
node_36: feature_name=GO:0032504	feature_id[244].value <= threshold=17.22207546234131
node_37: feature_name=GO:0051348	feature_id[126].value <= threshold=5.933559417724609
node_38: feature_name=GO:0042522	feature_id[692].value <= threshold=1.78694087266922
node_39: feature_name=GO:0031100	feature_id[73].value <= threshold=3.2312101125717163
node_40: feature_name=GO:0042493	feature_id[149].value <= threshold=19.771170616149902
node_41: feature_name=GO:0001552	feature_id[366].value <= threshold=2.1711617708206177
node_42: feature_name=GO:0046483	feature_id[391].value <= threshold=320.9667053222656
node_43: feature_name=GO:0002439	feature_id[431].value <= threshold=1.832722783088684
node_44: feature_name=GO:0006555	feature_id[514].value <= threshold=5.321640968322754
node_45: feature_name=GO:0070424	feature_id[254].value <= threshold=3.321003556251526
node_46: feature_name=hsa05144	feature_id[751].value <= threshold=2.635499954223633
node_47: feature_name=GO:0032633	feature_id[627].value <= threshold=1.2592533230781555
node_48: feature_name=GO:0019814	feature_id[189].value <= threshold=2.1583125591278076
node_49: feature_name=GO:1901989	feature_id[30].value <= threshold=3.3150794506073
node_50: feature_name=GO:1903318	feature_id[196].value <= threshold=1.62252539396286
node_51: feature_name=GO:0060576	feature_id[204].value <= threshold=2.1097792387008667
node_52: feature_name=hsa05221	feature_id[349].value > threshold=0.8723124265670776
node_96: feature_name=hsa05221	feature_id[349].value > threshold=0.8756309151649475
node_98: feature_name=GO:0046794	feature_id[794].value <= threshold=6.585465431213379
node_99: feature_name=GO:0005035	feature_id[133].value <= threshold=0.6986835598945618

node_100: feature_name=hsa04640 feature_id[79].value <= threshold=1.458605408668518
node_101: feature_name=GO:0045945 feature_id[267].value > threshold=1.6378425359725952
node_103: feature_name=GO:0048145 feature_id[348].value <= threshold=0.11509107798337936
Class: positive genes

Rules_787 passed counts:1
node_0: feature_name=GO:0042113 feature_id[0].value <= threshold=13.408552169799805
node_1: feature_name=GO:0007568 feature_id[534].value <= threshold=5.0313897132873535
node_2: feature_name=GO:0002705 feature_id[541].value <= threshold=3.200145721435547
node_3: feature_name=GO:1901525 feature_id[576].value <= threshold=0.4466460347175598
node_4: feature_name=GO:0048539 feature_id[319].value <= threshold=3.0399646759033203
node_5: feature_name=GO:0001910 feature_id[385].value <= threshold=3.7437864542007446
node_6: feature_name=GO:0043200 feature_id[706].value <= threshold=9.307284355163574
node_7: feature_name=GO:0001773 feature_id[308].value <= threshold=4.016931533813477
node_8: feature_name=GO:0090116 feature_id[97].value <= threshold=7.99645471572876
node_9: feature_name=GO:0019814 feature_id[189].value <= threshold=4.525782108306885
node_10: feature_name=GO:1902583 feature_id[215].value <= threshold=17.60310649871826
node_11: feature_name=GO:0045429 feature_id[737].value <= threshold=1.6133361458778381
node_12: feature_name=GO:0003720 feature_id[228].value <= threshold=5.519326210021973
node_13: feature_name=GO:0046006 feature_id[364].value <= threshold=4.9985432624816895
node_14: feature_name=GO:0070424 feature_id[254].value <= threshold=6.233297109603882
node_15: feature_name=GO:0009892 feature_id[320].value <= threshold=59.99180793762207
node_16: feature_name=GO:0007064 feature_id[527].value <= threshold=11.57377815246582
node_17: feature_name=GO:0005575 feature_id[17].value <= threshold=15.417460918426514
node_18: feature_name=GO:0043368 feature_id[31].value <= threshold=4.059496641159058
node_19: feature_name=GO:0005164 feature_id[163].value <= threshold=5.793607711791992
node_20: feature_name=GO:0042130 feature_id[16].value <= threshold=5.073179721832275
node_21: feature_name=GO:0010216 feature_id[282].value <= threshold=4.834584474563599
node_22: feature_name=GO:0009628 feature_id[553].value <= threshold=29.314892768859863
node_23: feature_name=GO:0045628 feature_id[749].value <= threshold=3.1001367568969727
node_24: feature_name=GO:0042288 feature_id[100].value <= threshold=5.174932956695557
node_25: feature_name=GO:0002329 feature_id[406].value <= threshold=2.348930835723877
node_26: feature_name=GO:0051246 feature_id[642].value <= threshold=0.6498909294605255
node_27: feature_name=GO:0042162 feature_id[355].value <= threshold=8.05103588104248
node_28: feature_name=hsa04668 feature_id[333].value <= threshold=5.614926338195801

node_29: feature_name=GO:0032200 feature_id[613].value <= threshold=28.735913276672363
node_30: feature_name=hsa04662 feature_id[334].value <= threshold=9.588344097137451
node_31: feature_name=GO:1901992 feature_id[105].value <= threshold=5.291427850723267
node_32: feature_name=GO:0072341 feature_id[144].value <= threshold=6.01465106010437
node_33: feature_name=GO:0047485 feature_id[121].value <= threshold=6.657836437225342
node_34: feature_name=GO:0090594 feature_id[738].value <= threshold=1.8673912286758423
node_35: feature_name=GO:0043525 feature_id[524].value <= threshold=2.881397008895874
node_36: feature_name=GO:0032504 feature_id[244].value <= threshold=17.22207546234131
node_37: feature_name=GO:0051348 feature_id[126].value <= threshold=5.933559417724609
node_38: feature_name=GO:0042522 feature_id[692].value <= threshold=1.78694087266922
node_39: feature_name=GO:0031100 feature_id[73].value <= threshold=3.2312101125717163
node_40: feature_name=GO:0042493 feature_id[149].value <= threshold=19.771170616149902
node_41: feature_name=GO:0001552 feature_id[366].value <= threshold=2.1711617708206177
node_42: feature_name=GO:0046483 feature_id[391].value <= threshold=320.9667053222656
node_43: feature_name=GO:0002439 feature_id[431].value <= threshold=1.832722783088684
node_44: feature_name=GO:0006555 feature_id[514].value <= threshold=5.321640968322754
node_45: feature_name=GO:0070424 feature_id[254].value <= threshold=3.321003556251526
node_46: feature_name=hsa05144 feature_id[751].value <= threshold=2.635499954223633
node_47: feature_name=GO:0032633 feature_id[627].value <= threshold=1.2592533230781555
node_48: feature_name=GO:0019814 feature_id[189].value <= threshold=2.1583125591278076
node_49: feature_name=GO:1901989 feature_id[30].value <= threshold=3.3150794506073
node_50: feature_name=GO:1903318 feature_id[196].value <= threshold=1.62252539396286
node_51: feature_name=GO:0060576 feature_id[204].value <= threshold=2.1097792387008667
node_52: feature_name=hsa05221 feature_id[349].value > threshold=0.8723124265670776
node_96: feature_name=hsa05221 feature_id[349].value <= threshold=0.8756309151649475
Class: positive genes

Rules_788 passed counts:1
node_0: feature_name=GO:0042113 feature_id[0].value <= threshold=13.408552169799805
node_1: feature_name=GO:0007568 feature_id[534].value <= threshold=5.0313897132873535
node_2: feature_name=GO:0002705 feature_id[541].value <= threshold=3.200145721435547
node_3: feature_name=GO:1901525 feature_id[576].value <= threshold=0.4466460347175598
node_4: feature_name=GO:0048539 feature_id[319].value <= threshold=3.0399646759033203
node_5: feature_name=GO:0001910 feature_id[385].value <= threshold=3.7437864542007446
node_6: feature_name=GO:0043200 feature_id[706].value <= threshold=9.307284355163574

node_7: feature_name=GO:0001773 feature_id[308].value <= threshold=4.016931533813477
node_8: feature_name=GO:0090116 feature_id[97].value <= threshold=7.99645471572876
node_9: feature_name=GO:0019814 feature_id[189].value <= threshold=4.525782108306885
node_10: feature_name=GO:1902583 feature_id[215].value <= threshold=17.60310649871826
node_11: feature_name=GO:0045429 feature_id[737].value <= threshold=1.6133361458778381
node_12: feature_name=GO:0003720 feature_id[228].value <= threshold=5.519326210021973
node_13: feature_name=GO:0046006 feature_id[364].value <= threshold=4.9985432624816895
node_14: feature_name=GO:0070424 feature_id[254].value <= threshold=6.233297109603882
node_15: feature_name=GO:0009892 feature_id[320].value <= threshold=59.99180793762207
node_16: feature_name=GO:0007064 feature_id[527].value <= threshold=11.57377815246582
node_17: feature_name=GO:0005575 feature_id[17].value <= threshold=15.417460918426514
node_18: feature_name=GO:0043368 feature_id[31].value <= threshold=4.059496641159058
node_19: feature_name=GO:0005164 feature_id[163].value <= threshold=5.793607711791992
node_20: feature_name=GO:0042130 feature_id[16].value <= threshold=5.073179721832275
node_21: feature_name=GO:0010216 feature_id[282].value <= threshold=4.834584474563599
node_22: feature_name=GO:0009628 feature_id[553].value <= threshold=29.314892768859863
node_23: feature_name=GO:0045628 feature_id[749].value <= threshold=3.1001367568969727
node_24: feature_name=GO:0042288 feature_id[100].value <= threshold=5.174932956695557
node_25: feature_name=GO:0002329 feature_id[406].value <= threshold=2.348930835723877
node_26: feature_name=GO:0051246 feature_id[642].value <= threshold=0.6498909294605255
node_27: feature_name=GO:0042162 feature_id[355].value <= threshold=8.05103588104248
node_28: feature_name=hsa04668 feature_id[333].value <= threshold=5.614926338195801
node_29: feature_name=GO:0032200 feature_id[613].value <= threshold=28.735913276672363
node_30: feature_name=hsa04662 feature_id[334].value <= threshold=9.588344097137451
node_31: feature_name=GO:1901992 feature_id[105].value <= threshold=5.291427850723267
node_32: feature_name=GO:0072341 feature_id[144].value <= threshold=6.01465106010437
node_33: feature_name=GO:0047485 feature_id[121].value <= threshold=6.657836437225342
node_34: feature_name=GO:0090594 feature_id[738].value <= threshold=1.8673912286758423
node_35: feature_name=GO:0043525 feature_id[524].value <= threshold=2.881397008895874
node_36: feature_name=GO:0032504 feature_id[244].value <= threshold=17.22207546234131
node_37: feature_name=GO:0051348 feature_id[126].value <= threshold=5.933559417724609
node_38: feature_name=GO:0042522 feature_id[692].value <= threshold=1.78694087266922
node_39: feature_name=GO:0031100 feature_id[73].value <= threshold=3.2312101125717163
node_40: feature_name=GO:0042493 feature_id[149].value <= threshold=19.771170616149902
node_41: feature_name=GO:0001552 feature_id[366].value <= threshold=2.1711617708206177

node_42: feature_name=GO:0046483 feature_id[391].value <= threshold=320.9667053222656
node_43: feature_name=GO:0002439 feature_id[431].value <= threshold=1.832722783088684
node_44: feature_name=GO:0006555 feature_id[514].value <= threshold=5.321640968322754
node_45: feature_name=GO:0070424 feature_id[254].value <= threshold=3.321003556251526
node_46: feature_name=hsa05144 feature_id[751].value <= threshold=2.635499954223633
node_47: feature_name=GO:0032633 feature_id[627].value <= threshold=1.2592533230781555
node_48: feature_name=GO:0019814 feature_id[189].value <= threshold=2.1583125591278076
node_49: feature_name=GO:1901989 feature_id[30].value <= threshold=3.3150794506073
node_50: feature_name=GO:1903318 feature_id[196].value <= threshold=1.62252539396286
node_51: feature_name=GO:0060576 feature_id[204].value <= threshold=2.1097792387008667
node_52: feature_name=hsa05221 feature_id[349].value <= threshold=0.8723124265670776
node_53: feature_name=GO:1903896 feature_id[135].value <= threshold=1.397229254245758
node_54: feature_name=GO:0097028 feature_id[443].value > threshold=1.2365349531173706
node_88: feature_name=GO:0005488 feature_id[187].value > threshold=0.0009100764873437583
node_90: feature_name=GO:0042306 feature_id[705].value > threshold=1.845421314239502
Class: positive genes

Rules_789 passed counts:1
node_0: feature_name=GO:0042113 feature_id[0].value <= threshold=13.408552169799805
node_1: feature_name=GO:0007568 feature_id[534].value <= threshold=5.0313897132873535
node_2: feature_name=GO:0002705 feature_id[541].value <= threshold=3.200145721435547
node_3: feature_name=GO:1901525 feature_id[576].value <= threshold=0.4466460347175598
node_4: feature_name=GO:0048539 feature_id[319].value <= threshold=3.0399646759033203
node_5: feature_name=GO:0001910 feature_id[385].value <= threshold=3.7437864542007446
node_6: feature_name=GO:0043200 feature_id[706].value <= threshold=9.307284355163574
node_7: feature_name=GO:0001773 feature_id[308].value <= threshold=4.016931533813477
node_8: feature_name=GO:0090116 feature_id[97].value <= threshold=7.99645471572876
node_9: feature_name=GO:0019814 feature_id[189].value <= threshold=4.525782108306885
node_10: feature_name=GO:1902583 feature_id[215].value <= threshold=17.60310649871826
node_11: feature_name=GO:0045429 feature_id[737].value <= threshold=1.6133361458778381
node_12: feature_name=GO:0003720 feature_id[228].value <= threshold=5.519326210021973
node_13: feature_name=GO:0046006 feature_id[364].value <= threshold=4.9985432624816895
node_14: feature_name=GO:0070424 feature_id[254].value <= threshold=6.233297109603882
node_15: feature_name=GO:0009892 feature_id[320].value <= threshold=59.99180793762207
node_16: feature_name=GO:0007064 feature_id[527].value <= threshold=11.57377815246582
[truncated: 52,391 more chars]
